# Diverse LEF/TCF Expression in Human Colorectal Cancer Correlates with Altered Wnt-Regulated Transcriptome in a Meta-Analysis of Patient Biopsies

**DOI:** 10.3390/genes11050538

**Published:** 2020-05-11

**Authors:** Claus-Dieter Mayer, Soizick Magon de La Giclais, Fozan Alsehly, Stefan Hoppler

**Affiliations:** 1 Biomathematics and Statistics Scotland (BioSS) and Rowett Institute, University of Aberdeen, Aberdeen AB25 2ZD, Scotland, UK; c.mayer@abdn.ac.uk (C.-D.M.); soizickmagon@gmail.com (S.M.d.L.G.); 2 Biomathematics and Statistics Scotland (BioSS) and School of Mathematics & Statistics, University of Glasgow, Glasgow G12 8QQ, Scotland, UK; 3 Institut National des Sciences Appliquées de Toulouse, 135, Avenue de Rangueil, CEDEX 4, 31077 Toulouse, France; 4 Institute of Medical Sciences, University of Aberdeen, Aberdeen AB25 2ZD, Scotland, UK; f.alsehly.18@abdn.ac.uk

**Keywords:** Wnt, colorectal cancer, TCF, LEF, transcriptome

## Abstract

Aberrantly activated Wnt signaling causes cellular transformation that can lead to human colorectal cancer. Wnt signaling is mediated by Lymphoid Enhancer Factor/T-Cell Factor (LEF/TCF) DNA-binding factors. Here we investigate whether altered *LEF*/*TCF* expression is conserved in human colorectal tumor sample and may potentially be correlated with indicators of cancer progression. We carried out a meta-analysis of carefully selected publicly available gene expression data sets with paired tumor biopsy and adjacent matched normal tissues from colorectal cancer patients. Our meta-analysis confirms that among the four human *LEF*/*TCF* genes, *LEF1* and *TCF7* are preferentially expressed in tumor biopsies, while *TCF7L2* and *TCF7L1* in normal control tissue. We also confirm positive correlation of *LEF1* and *TCF7* expression with hallmarks of active Wnt signaling (i.e., *AXIN2* and *LGR5*). We are able to correlate differential *LEF*/*TCF* gene expression with distinct transcriptomes associated with cell adhesion, extracellular matrix organization, and Wnt receptor feedback regulation. We demonstrate here in human colorectal tumor sample correlation of altered *LEF*/*TCF* gene expression with quantitatively and qualitatively different transcriptomes, suggesting *LEF*/*TCF-*specific transcriptional regulation of Wnt target genes relevant for cancer progression and survival. This bioinformatics analysis provides a foundation for future more detailed, functional, and molecular analyses aimed at dissecting such functional differences.

## 1. Introduction

Wnt signaling functions in normal development and stem-cell-mediated homeostasis; and also, in the etiology of disease, such as cancer [1,2]. The best understood Wnt signaling mechanism is a nuclear β-catenin-mediated signal transduction pathway regulating transcriptional gene expression [3]. Wnt pathway-promoted nuclear β-catenin proteins regulate transcription indirectly by binding to and altering multi-protein complexes associated with sequence-specific DNA binding factors, predominantly of the Lymphoid Enhancer Factor/T-Cell Factor (LEF/TCF) protein family [4,5]. Humans have four genes encoding LEF/TCF proteins, similar to other mammals and most vertebrates [6,7]. With little or without nuclear β-catenin protein, LEF/TCF proteins mostly mediate transcriptional repression of Wnt-target genes. Moreover, with increased nuclear β-catenin levels, LEF/TCF proteins generally mediate transcriptional activation [8]. 

Different LEF/TCF genes are expressed in different tissues and at different stages, and together with alternative splicing and alternative promoter use this results in a rich variety of differentially expressed LEF/TCF protein isoforms [7,9,10]. There is considerable functional redundancy between different LEF/TCF proteins, yet also emerging evidence for quantitative and even qualitative differences. Quantitative difference refers here to different LEF/TCF protein isoforms being more or less effective at mediating transcriptional repression with little or without nuclear β-catenin protein; versus transcriptional activation with increased nuclear β-catenin levels [4,6,11,12]. Qualitative difference refers to the possibility of different LEF/TCF protein isoforms binding to different cis-regulatory target DNA sequences and thereby regulating different Wnt target genes [13,14].

Wnt signaling is particularly relevant in colorectal cancer, since the vast majority of colorectal cancers harbor Wnt/β-catenin pathway-activating mutations [15], which contrasts with a normal benevolent role in regulating stem-cell-mediated maintenance of intestinal and colorectal epithelial tissue [16,17]. Roles for LEF/TCF proteins has been studied in tissue-culture and animal models of intestinal stem cells and colorectal cancer, which suggested roles for *TCF7L2* and *TCF7* in normal colorectal tissue, where *LEF1* and *TCF7L1* are silent, but strong expression in colorectal tumor of *LEF1* [9,18,19,20,21] and *TCF7* [22,23]. 

Here, we aimed to assess whether predictions about Wnt signaling in colorectal cancer from tissue-culture and animal model systems could be confirmed by a meta-analysis of compatible transcriptomics studies of paired normal and tumor biopsy samples from human patient, and whether specific LEF/TCF gene-correlated transcriptomes imply LEF/TCF gene-specific function in normal or tumor tissue. 

Our meta-analysis of transcriptomics studies provides a clear picture of altered LEF/TCF gene expression, confirming *TCF7L2* in normal and *LEF1* expression in tumor tissue, but also higher than expected *TCF7L1* in normal and relatively higher *TCF7* expression in tumor tissue. Specific LEF/TCF gene expression is indeed correlated with differences in the overall transcriptome, with some of these differences suggestive of relevance for tumor progression.

## 2. Materials and Methods

Publically available data were searched within the databases Gene Expression Omnibus (GEO) and ArrayExpress using ‘colon cancer’ or ‘colorectal cancer’ as search words. From this list only studies that satisfied the following conditions were selected: a) samples from human biopsies (i.e. no rodent models or cell line data), b) paired samples from tumor with nearby normal tissue, c) a sample size of at least four patients in order to conduct meaningful statistical analyses. The selected studies with Genomic Spatial Event (GSE) database accession numbers are listed in Table 1. As a first exploratory analysis step a principal component analysis (PCA) based on scaled and centered variables was conducted using the prcomp function within R. The score plots of the first two principal components were studied to check for the separation of tumor and normal samples

**Figure 1:**
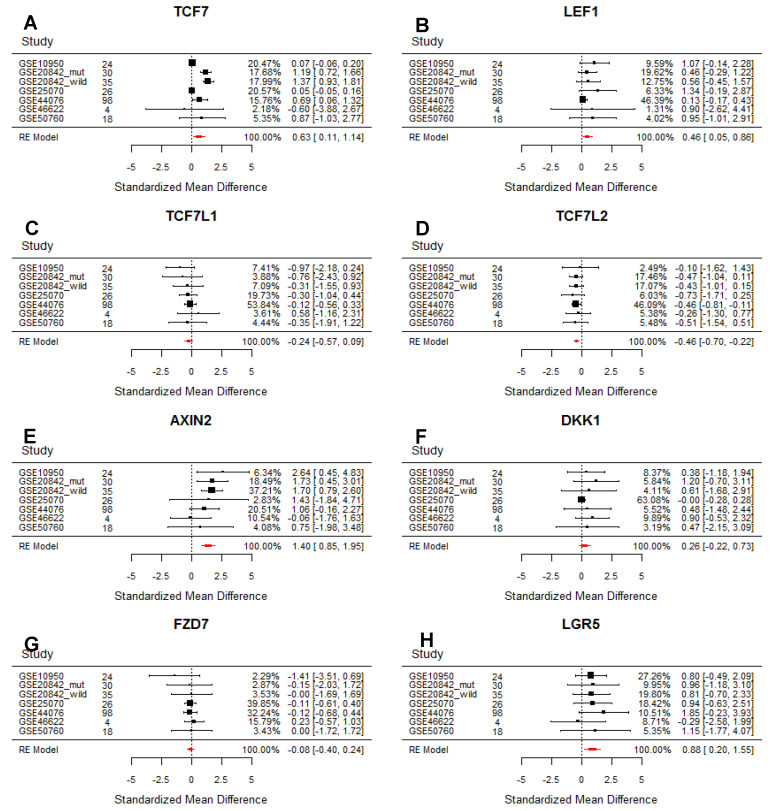
Forest plots of gene expression of all four LEF/TCF genes (**A**): *TCF7*, (**B**): *LEF1*, (**C**): *TCF7L1*, (**D**): *TCF7L2*, and (**E**): *AXIN2*, (**F**): *DICKKOPF-1* (*DKK1*), (**G**): *FZD7*, and (**H**): *LGR5*, from six selected studies. Columns from left to right indicate: accession no. of study (with GSE20842 [27] separated between kras-positive and kras-mutant samples); number of patients in individual studies; horizontal segments indicate the standardized mean difference between tumor and normal, and their confidence interval, with the size of the square dot being proportional with the weight of the study in the meta-analysis using a ‘random effects’ model. The corresponding values are written in the column on the right: weight of the individual study in percent as part of the meta-analysis; standardized mean difference; and in square brackets confidence interval. The red polygon in the bottom of each plot shows the summary estimate based on the random-effect model. Values to the left of the midline indicated higher expression in the control relative to the tumor sample, e.g., see *AXIN2* and *LGR5*. Individual studies with small sample size (i.e., few patients) as expected often have larger confidence intervals (therefore less reliability, e.g., see *TCF7* and *LEF1* data for GSE46622 study), but in the meta-analysis (in red) much tighter confidence intervals (therefore higher reliability). Note that, among the four LEF/TCF genes, *TCF7*, *LEF1*, are expressed higher, while *TCF7L1*, *TCF7L2* lower in tumor tissue.

Meta-Analysis of differential expression between tumor and normal for the eight selected genes of interest (Figure 1) was conducted using the R-package metafor [24]. The standardised mean difference (SMD) was chosen as a measure of differential gene expression here and a ‘random effects’ model was used for weighting of the studies. The random effects model is used to address the heterogeneity between studies caused by differences in study designs, study population and also by the different gene expression platforms used.

**Figure 2:**
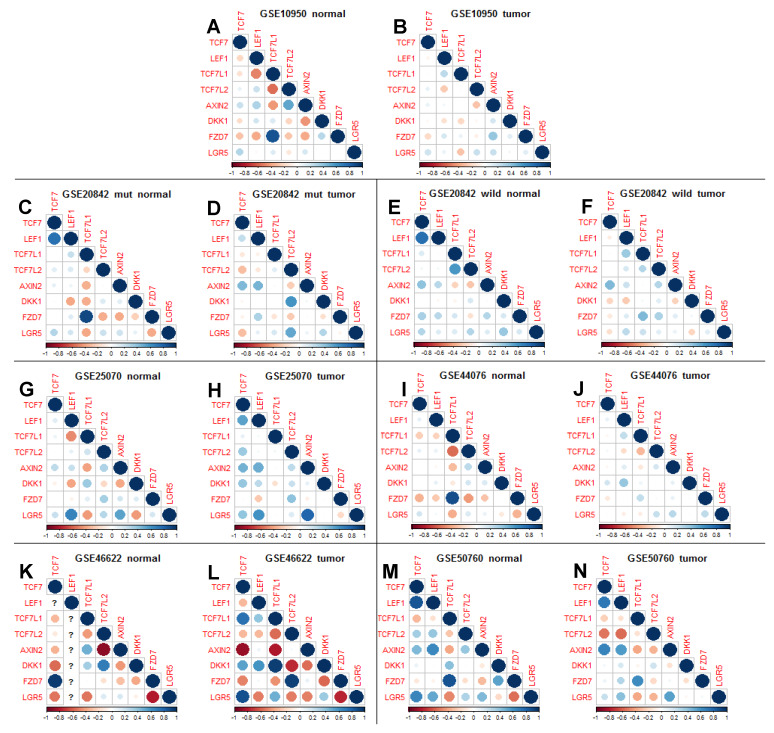
Correlation plot matrix of relative gene expression between eight selected genes in six selected studies (**A**–**N**), with normal control (**A**, **C**, **E**, **G**, **I**, **K**, **M**) separated from tumor sample (**B**, **D**, **F**, **H**, **J**, **L**, **N**), and additionally for the GSE20842 [27] between kras-mutant (“mut”) samples (**C**, **D**) and kras-positive (“wild” as in wildtype) samples (**E**, **F**). Blue dots indicate positive and red dots negative correlation. The size of the circle and the intensity of the color is proportional to the correlation coefficient; therefore, as an internal control, expected diagonal series of large blue dots where expression of genes is compared to the expression of the same gene). Missing values in GSE46622 [30] is due to low value data for *LEF1* in this study. Note positive correlation between *AXIN2* expression and *LGR5*, *TCF7* and *LEF1* expression, yet negative correlation with *TCF7L1* expression, while *TCF7L1* and *FZD7* expression are positively correlated, though clearly much more so in normal control tissue than in tumor. In contrast, the correlation between *AXIN2* and *TCF7* expression is clearly more robust in tumor compared to normal tissue. Interestingly, the unearthed correlation between *TCF7L1* and *FZD7* expression appears to be dependent on wild-type *kRAS* in the tumor (compare **D** with **F**, yet not in normal control **C**).

The correlation plots (Figure 2) were produced within the R-package corrplot [25]. For the meta-analysis of correlation differences between normal and tumor for each of the eight selected genes of interest we first selected 18,150 genes that were included in at least two of the studies. For each of the eight genes, we then calculated correlations within tumor and normal samples with each of the 18,150 other genes. The differences between tumor and normal correlation for each of the 8 × 18,150 = 145,200 combinations where averaged across the studies and tested against the null hypothesis of no correlation by a one-sample t-test, which corresponds to a random effect meta-analysis, where within study variation is assumed to be negligible compared to between study variation.

The organized expression data (Supplementary Table S1B–I) were filtered for standard deviation less than 0.2 where correlation coefficients association with a specific gene were ranked in all normal or all tumor samples (e.g., Supplementary Table S2), and where mean difference between them was independently calculated for p-value less than 0.05 (e.g., Table 2). The top 10 genes were presented in a ranked list (Table 2, Table 3 and Table 4; Supplementary Tables S2–4), and the top 100 list was used for gene ontology analysis, excluding the target gene itself (i.e *AXIN2* in the *AXIN2* list, the specific LEF/TCF gene in the specific LEF/TCF gene list etc.). The individual LEF/TCF correlation values were compared with each other (Supplementary Figure S1J), and with *AXIN2* (Supplementary Figure S1K). Gene ontology analysis was carried out on GOrilla (http://cbl-gorilla.cs.technion.ac.il) selecting the ‘Two unranked lists of genes’ setting comparing the first 100 genes of the ranked gene lists (see above) with the full 18,150 background list of all genes in the analyzed data. The top 5 gene ontology terms were listed together with order of magnitude or their p-value if below 0.001.

## 3. Results

### 3.1. Selection of Transcriptomics Data Sets and Quality Control

We carefully reviewed publicly available transcriptomics datasets of human colorectal cancer samples. We focused on the traditional and longest existing gene expression databases GEO and ArrayExpress. We selected published studies with a) sufficient numbers of samples for the intended statistical meta-analysis and b) paired samples from tumor with nearby normal tissue (Table 1). A similar review of available human RNA-seq datasets at the time did not identify studies with sufficient numbers of samples to justify a meaningful meta-analysis. Our initial selection of studies therefore contained mainly microarray studies as these form the majority of entries in those databases and also because the few RNAseq studies we obtained had either too small a sample size or other quality issues. We also note that a joint meta-analysis combining microarray and RNAseq data would be quite challenging as the nature of the data (continuous measurements versus normalized counts) and the corresponding analysis techniques (linear models versus generalized linear models) are fundamentally different.

Our hypothesis predicts that the transcriptome in tumor tissue would be sufficiently distinct from the one in control normal tissue as a prerequisite for our intended meta-analysis. A principal component analysis of the selected datasets (Supplementary Figure S1) therefore served as an additional quality control confirming separation of the transcriptome between tumor sample and normal control in all selected individual studies. The data from Kim et al. [31] showed a somewhat less distinct separation of tumor versus normal transcriptome, but a clear enough difference to retain this study in our meta-analysis. Another study, GSE46905 [32], which we had originally considered, was not taken forward because we could not find sufficiently clear separation between tumor and normal transcriptome (Supplementary Figure S2). 

### 3.2. Transcriptomics Expression of Eight Selected Genes

We first tested our hypothesis about differential expression of LEF/TCF genes between tumor and normal tissue with forest plots (Figure 1) of all four LEF/TCF genes (*TCF7*, *LEF1*, *TCF7L1*, *TCF7L2*). We chose to monitor additionally the expression of *AXIN2*, *DKK1*, *FZD7*, and *LGR5*. *AXIN2* is a direct Wnt target gene [33,34] used here as a reliable indicator of intracellular Wnt/β-catenin pathway activity, and because increased expression has been reported in colorectal tumor tissue [35]. *DKK1* is a direct Wnt target gene [36] with increased expression in many cancers [37], however, here *DKK1* was chosen particularly because its expression had previously been reported to be reduced in colorectal tumor [38]. *FZD7* is also a Wnt target gene [39], relevant in colorectal cancer [40] and normal intestinal epithelium [41]. *LGR5* is a Wnt target gene, which is the marker gene for normal adult intestinal stem cells [17], and for particularly aggressive colorectal cancer stem cells [16,22,23,42]. 

Our meta-analysis reveals that *AXIN2* (Figure 1E) is consistently expressed at a higher level in tumor relative to normal tissue, and so is *LGR5* (Figure 1H), indicating as expected increased Wnt/β-catenin signaling activity [35] and increased stem cell identity of tumor tissue [42]. Importantly, among the LEF/TCF genes, our meta-analysis also corroborates a switch from relatively higher *TCF7L2* (Figure 1D) and *TCF7L1* (Figure 1C) expression in normal control to relatively higher *TCF7* (Figure 1A) and *LEF1* (Figure 1B) expression in tumor tissue. Our meta-analysis did not highlight any dramatic changes in gene expression for the *FZD7* and *DKK1* genes between normal and tumor tissue.

### 3.3. Correlation of Expression between Eight Selected Genes

We next analyzed correlations in gene expression between those eight genes, positive or negative, initially in individual matrix plots (Figure 2). As expected, there is in general a positive correlation between *AXIN2* expression and *LGR5* expression (stem cell identity marker). There appears also generally a positive correlation between *TCF7* and *LEF1* expression and between those and *LGR5* expression, consistent with previous findings in mouse models [22,23]. If these correlations were linked to increased Wnt/β-catenin signaling activity, then we would also expect a positive correlation between *TCF7* and *LEF1* expression and *AXIN2* expression, which indeed is consistent with the data. Interestingly, *AXIN2* expression, which indicates Wnt/β-catenin pathway activity, is negatively correlated with *TCF7L1* expression. Comparing individual blots also suggests generally less strong correlation in tumor tissue. 

**Figure 3:**
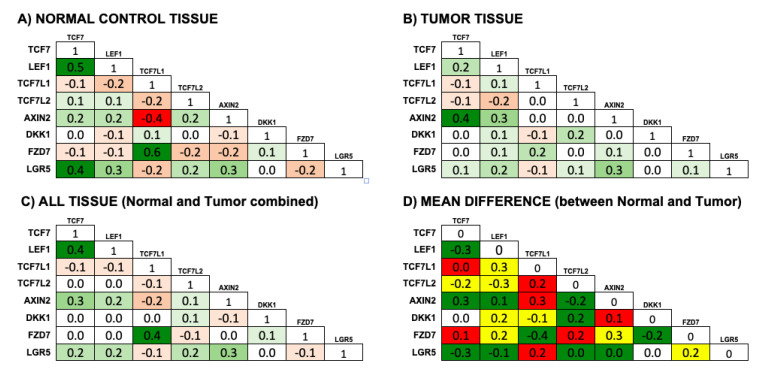
Correlation of transcript expression between eight selected genes (*TCF7*, *LEF1*, *TCF7L1*, *TCF7L2*, *AXIN2*, *DKK1*, *FZD7* and *LGR5*) in normal control tissue (**A**), in tumor tissue (**B**), and when analyzed combined in normal and tumor tissue (**C**) (negative numbers and graded red highlighting indicates negative correlation; positive numbers and graded green highlighting positive correlation). (**D**) Mean difference between normal and tumor tissue of correlation of transcript expression between eight selected genes (red highlighting with positive numbers indicates reduced negative correlation in tumor tissue; green highlighting with negative numbers indicates reduced, with positive numbers increased, positive correlation in tumor tissue; yellow highlighting with positive numbers indicates a switch from negative to positive, and with negative numbers to negative, correlation in tumor tissue. Note generally reduced correlations in tumor tissue; particularly note, reduced positive correlation between *TCF7* and *LEF1*, between *TCF7* and *LGR5*, and between *TCF7L1* and *FZD7* expression; and reduced negative correlation between *AXIN2* and *TCF7L1* expression. However, also note exceptional increased positive correlation between *AXIN2* and *TCF7* in tumor tissue.

We combined the transcriptomics data from these different studies in a meta-analysis, which provided an even clearer picture (Figure 3). However, we only carried out this meta-analysis with data sets from five of the six selected studies (having removed the data set with only a few patients and missing *LEF1* values, GSE46622 [30]). This meta-analysis allowed us to tease out more clearly differences between tumor and normal tissue; with correlations between transcripts among these eight genes being mostly much stronger and clearer in normal control tissue (Figure 3A) and generally much weaker in tumor samples (Figure 3B,D). Positive correlations linking *TCF7* with *LEF1* and with *LGR5* in normal tissue are reduced in tumor tissue, and the negative correlation between *AXIN2* and *TCF7L1* in normal tissue is also reduced in tumor tissue. The exception to this rule is a strengthened correlation between *AXIN2* and *TCF7* expression in tumor tissue. Remarkably, our analysis also reveals that *FZD7* expression is strongly positively correlated with *TCF7L1* expression. Even more remarkably, this correlation is absent or much reduced in tumor tissue, and in the one study that has relevant information on kRAS mutant status in samples [27], it suggests that this correlation between *TCF7L1* and *FZD7* is particularly strong in KRAS-mutant normal tissue (Figure 2C), and is strongly reduced in KRAS mutant tumor (Figure 2D).

### 3.4. Correlation between Eight Selected Genes and the Rest of the Transcriptome

We extended the analysis for correlations in gene expression between each of those eight genes with the whole rest of the transcriptome. We ranked the *TCF7* gene expression-correlated whole transcriptome expression (positively and negatively correlated) separately in normal tissue and in tumor tissue (Supplementary Table S2A), and then also independently ranked and analyzed the greatest transcript correlation differences between tumor and normal tissue (Table 2). In a gene ontology analysis, we searched for the suggested association of correlated gene expression with biomedical processes. We then repeated this independently for the other LEF/TCF gene expression-correlated transcriptomes, and the *AXIN2*-, *DKK1*-, *FZD7*-, *LGR5*-correlated transcriptomes (Table 2, Supplementary Table S1B-I, Supplementary Table S2).

In normal tissue, both *TCF7* and *LEF1* expression is positively correlated with gene expression associated with the immune system. This correlation with immune system-associated transcripts is more generally lost in tumor tissue. In normal tissue *TCF7L1* expression is positively, and *AXIN2* expression negatively correlated with gene expression associated with cell adhesion. While in tumor tissue *TCF7L1*, and even more so *LEF1* gene expression is correlated with transcripts associated with the extracellular matrix (ECM); and expression of *AXIN2* with *TCF7* is correlated with regulation of Wnt signaling (particularly Wnt receptor catabolic processes), with this correlation being stronger in tumor than normal tissue. However, we do not find any specific correlation with cyclin D1 (*CCND1*) gene expression [43,44], and *BMP4* expression [45] is only specifically correlated with *DKK1*.

### 3.5. Comparison of Lef/Tcf-Correlated Transcriptomes

Subsequently, we explicitly focused on comparing the different LEF/TCF gene-correlated transcriptomes with each other (Table 3, Supplementary Tables S1J and S3). A compound analysis of differences between all LEF/TCF gene-correlated transcriptomes (Supplementary Table S3) reveals that, overall, differences between *TCF7L1*- and *TCF7L2*-correlation dominate in normal tissue, while in tumor tissue, though they are still prominent, differences in particular with *TCF7*-correlation and also with *LEF1*-correlation become more noticeable. These overall differences can be associated with extracellular matrix, angiogenesis, and cell adhesion. However, clearly this compound analysis by itself does not reflect any meaningful biological or clinical situation and this gene ontology association here only serves to guide more specific analyses (Table 3). 

Detailed pairwise analysis of these prominent differences between *TCF7L1*- and *TCF7L2*-correlation in normal tissue suggests a stronger association of *TCF7L1*-correlated transcripts with the extracellular matrix, and a stronger association of *TCF7L2*-correlated transcripts with cell junctions and cell adhesion; but interestingly, in tumor tissue *TCF7L1* expression is more strongly correlated with transcripts associated with cell adhesion; and also with angiogenesis. Consistent with the single LEF/TCF gene correlation analysis above, any comparison between either *TCF7* or *LEF1*, on the one hand and with either *TCF7L1* or *TCF7L2*, on the other, highlights the higher correlation of expression of *TCF7* and *LEF1* with a transcriptome associated with the immune system. Remarkably, these pairwise comparisons also reveal that any link to extracellular matrix (ECM) generally excludes *TCF7* and *TCF7L2*, but generally includes both *TCF7L1* and *LEF1*; *TCF7L1* more in normal tissue, and *LEF1* more in tumor. Comparison between these two, i.e. between *TCF7L1*- and *LEF1*-correlated transcription, also reveals a potential correlation of *LEF1* expression with double strand break DNA repair in tumor. Pairwise comparison with *TCF7*, suggests *LEF1* in tumor may also be correlated with regulation of cell migration. The same comparison suggests a stronger correlation for *TCF7* in normal tissue to transcripts associated with sensory perception, which seems difficult to explain.

### 3.6. Comparison between AXIN2- and LEF/TCF-Correlated Transcriptomes

Since LEF/TCF proteins are known to function generally as nuclear effectors of WNT/β-catenin signal transduction [3,4,6], we compared individual LEF/TCF gene expression-correlated transcriptomes with the *AXIN2* expression-correlated transcriptome (Table 4; Supplementary Table S1K and S4), employing *AXIN2* expression again as an indicator of WNT/β-catenin pathway activity. A compound analysis of overall differences between the *AXIN2*- and all LEF/TCF gene expression-correlated transcriptomes (Supplementary Table S4) reveals that most differences are with the TCFL1-correlated transcriptome, particularly in normal tissue, suggesting a link with cell adhesion.

The direct comparison between the *AXIN2*- and all *TCF7L1* gene expression-correlated transcriptomes confirms the higher correlation of *TCF7L1* expression with cell adhesion-associated transcripts, not just in normal tissue, but also in tumor tissue. Remarkably, *LEF1* shares with *TCF7L1* this stronger link to cell adhesion in tumor tissue, and also an association in normal tissue with muscle, which seems difficult to explain, but could somehow be linked to shared molecular machinery functioning in cell migration. The *AXIN2*-correlated transcriptome in tumor tissue trumps however, in its association with transcripts indicating regulation of Wnt signaling, particularly Wnt receptor catabolic processes, and contrasting with all LEF/TCF-correlated transcriptomes, apart from, interestingly, that of *TCF7*.

## 4. Discussion

### 4.1. Meta-Analysis of Human Colorectal Cancer Biopsy Transcripome Studies

After careful selection of individual studies, our meta-analysis reveals a much clearer picture than any individual study, thereby strongly validating our approach. Particularly notable is the dramatic tightening of the confidence intervals of the gene expression of the four LEF/TCF genes and another four selected genes in the meta-analysis compared to those of individual studies (Figure 1). The meta-analysis of correlation of gene expression between eight selected genes (Figure 3) is also much clearer than just comparing individual studies with each other (Figure 2). Generally, our meta-analysis substantiates that correlations are stronger in normal control compared to tumor samples, consistent with the idea of a certain breakdown of controlled gene expression in tumor tissue. An interesting exception to this rule is the stronger association between *TCF7* and *AXIN2* expression in tumor tissue, which could suggest some specificity in TCF7-mediated WNT/β-catenin signaling in human tumor tissue, supporting earlier such suggestions in the mouse model [23]. Our analysis proved particularly informative when dissecting differences in transcriptome correlation between the four LEF/TCF genes (Table 3) and with *AXIN2* (Table 4)*,* comparing tumor with normal tissue samples. 

### 4.2. Differences in LEF/TCF Gene Expression Correlate with Transcriptomes Indicative of Tumor Progression

Our meta-analysis reveals associations that are potentially relevant for tumor progression and possibly metastasis. Cell adhesion-associated gene expression is correlated with *TCF7L2* expression specifically in normal tissue, and with *LEF1* and *TCF7L1* in tumor tissue. Extracellular matrix-associated gene expression is also correlated with *TCF7L1* specifically in normal tissue and with *LEF1* expression exclusively in tumor tissue. Furthermore, transcripts indicative of angiogenesis are correlated with *TCF7L1* in tumor; and transcripts indicative of DNA double-strand break repair and of cell cycle progress with *LEF1* in tumor. The expression of *NFE2L2*, a regulator of p53 and indicator of poor prognosis, is directly transcriptionally regulated by WNT/β-catenin/TCF7L2 in cell culture models of colorectal cancer [46]. In our meta-analysis, *NFE2L2* is noticeable for the large discrepancy between positive correlation with *LEF1* and negative correlation with *TCF7L1* (Supplementary Table S1J), which clearly substantiates the importance of *NFE2L2* as an important WNT/β-catenin target gene in human colorectal cancer.

### 4.3. Indicators of Cell Migration Are Correlated with LEF1, and Likely also Other LEF/TCF Genes

There is clear correlation of transcripts indicative of regulation of cell migration with *LEF1* in tumor tissue, consistent with the previously suggested prognostic value of increased *LEF1* expression in colorectal cancer for both increased metastasis and for shorter survival prospects of patients [19,20]. Increased *LEF1* expression is associated with several types of cancer and has been associated in many tissues with regulation of epithelial-to-mesenchymal transition (EMT) including transcriptional activation of EMT effectors, such as N-Cadherin, Vimentin, and Snail [47]. 

However, a more complex additional involvement in cell migration of *TCF7, TCF7L1* and *TCF7L2* remains likely. *TCF7L1* expression is correlated, but only in normal tissue, with muscle-associated transcripts, and specifically with expression of the key EMT inducer *ZEB1* [48] (Table 2 and Table 3). Interestingly, in the mouse, *Zeb1* had been described in a mutual feedback regulatory loop with *Tcf4/Tcf7l2*, rather than *Tcf3/Tcf7l1* [49]. The marker for cell migration and known Wnt target *HEF1/NEDD9* [50] is correlated in our analysis specifically with *TCF7L2* expression (Supplementary Table S1J). EphrinB2 (*EPHB2*) expression in a mouse model of colorectal cancer is subject to competing positive regulation by *Tcf7l2* and negative regulation by *Lef1* [51]. Moreover, the related ephrinB3 (*EPHB3*), a Paneth cell marker and tumor suppressor, has recently been linked in cell line models of human colorectal cancer to transcriptional suppression specifically by TCF7L1 [52]. Our analysis shows *EPHB2* and *EPHB3* expression, while positively correlated with *AXIN2*, both negatively correlated with *TCF7L1* expression. Among the ephrins, our analysis highlights the disparity for *EPHA1* expression between such negative correlation with *TCF7L1* in contrast to positive correlation with *TCF7*. Increased *TCF7* expression in colorectal cancer had previously been shown [22,53] and was recently correlated with cell migration and in extension possibly metastasis [54]. However, the expression of *TCF7* in normal and tumor tissue is more complicated than off and on (see below). 

### 4.4. TCF7/LEF1 Gene Expression Correlates with Transcriptome Associated with the Immune System

There is clear correlation throughout our meta-analysis between *TCF7*/*LEF1* expression in normal tissue and transcripts associated with the immune system. However, since *TCF7*/*LEF1* expression is generally low in the control samples from normal tissue (Figure 1), it is likely that the few cells expressing any *TCF7* and *LEF1* in these isolated normal tissue samples belong to the immune system, and any differences in amount of *TCF7*/*LEF1* transcripts in these samples may reflect varying amounts of immune tissue included in these samples, which would be correlated to immune system-typical transcripts, rather than suggesting a switch in target genes regulated by *TCF7*/*LEF1* in normal and tumor tissue. 

### 4.5. Feedback Regulation of Wnt Signaling Components at the Cell Membrane 

Possibly the most surprising insight from this meta-analysis concerns the suggested feedback regulation on Wnt signaling components at the cell membrane. Firstly, there is strong correlation of *TCF7L1* with *FZD7* expression, particularly in normal but also in tumor tissue (Figure 2 and Figure 3). *FZD7* encodes a cell-surface Wnt receptor in intestinal stem cells [41], which has been linked to colorectal cancer [55]. However, there appears to be a pattern; gene expression of *FZD8*, *FZD4*, and *FZD3* has a similar positive and specific correlation with *TCF7L1*, while *FZD5* expression is negatively correlated with *TCF7L1,* yet positively correlated with *TCF7L2* expression (e.g., Supplementary Table S1J). These correlations are weaker in tumor tissues, particularly with *FZD3.*

Secondly, *AXIN2* expression specifically in tumor tissue is positively correlated with transcripts associated with feedback regulation of the Wnt pathway (Table 2), particularly the Wnt receptor catabolic process (Table 4). Furthermore, there is a correlation with the expression of genes such as *RNF43* and *ZNRF3*, which function as Membrane E3 ligases to promote ubiquitination and degradation of Wnt receptor proteins, including FZD receptor proteins [56], and suggestively, *RNF43* and *ZNRF3* are frequently found mutated in colorectal cancer [57,58]. *LGR5* functions with R-spondin proteins (RSPO) to counteract RNF43 and ZNRF3 and prolong Wnt receptor function at the membrane [56,59]. Our analysis indicates that *LGR5* expression is positively correlated with *AXIN2*, and particularly with *TCF7* in normal tissue (Figure 3); also, the related *LGR4* is even more strongly correlated with *TCF7L2* expression (Table 3, Supplementary Table S2D). NEDD4 and NEDD4L were recently identified as regulators of LGR5 protein degradation [60]. In our analysis, *NEDD4* expression (but not *NEDD4L*) is conspicuous, initially for being differentially correlated with *AXIN2* expression, negatively in normal tissue and positively in cancer tissue; and additionally, for being positively correlated with *TCF7L1* in normal tissue, which is opposite to *AXIN2*, but being positively correlated with *TCF7* expression in tumor tissue, as *AXIN2*. These findings are consistent with the suggested role of NEDD4 as a tumor suppressor in colorectal cancer. Among the R-spondin genes, *RSPO2* expression is clearly correlated with *TCF7L1*, but only in normal tissue, while *RSPO3* expression is less strongly correlated with *TCF7L1*, both in normal and tumor tissue. 

However, generally, any correlation between transcripts associated with this regulation of Wnt signaling and particularly Wnt receptor catabolic processes is strongest with *AXIN2* and *TCF7* expression, suggesting specifically for WNT/β-catenin/TCF7 signaling to mediate this feedback regulatory mechanism in tumor tissue. This may relate to mouse organoid culture growth becoming R-spondin-independent with experimental Tcf7 overexpression [23].

### 4.6. Implications for Molecular Functions of LEF/TCF Proteins and Isoforms

It is clearly difficult to de-convolute the likely molecular functions of LEF/TCF proteins from our transcriptomics meta-analysis; to assess whether there are quantitative differences in the way they function as transcriptional repressors or activators; or whether there are qualitative differences in the direct target genes they regulate, possibly due to additional or altered DNA-binding ability [13,14,61]. However, the strong difference in transcriptome correlation between *AXIN2* and *TCF7L1* is striking, while *TCF7* generally appeared to show the least differences (Table 4). If we accept *AXIN2* expression as a proxy for WNT/β-catenin signaling activity, then our analysis is at least consistent with *TCF7L1* predominantly functioning as a transcriptional repressor and TCF7 generally predominantly as a transcriptional activator, supporting the concept of a quantitative difference between different LEF/TCF factors [6]. It is also striking that *LEF1* and *TCF7L1* expression share a correlation with transcripts associated with cell adhesion and ECM organization. These LEF/TCF genes both lack sequences encoding a C-clamp suggesting at least the possibility that the C-clamp-missing LEF1 and TCF7L1 proteins are specifically capable of regulating genes involved in cell adhesion and ECM organization in a qualitatively different way to C-clamp containing LEF/TCF proteins like those encoded by *TCF7* and *TCF7L2*, [14].

Our strict selection procedure resulted in six microarray experiments being considered, since for any meta-analysis, rigorous quality control is of most importance and we think that our unbiased filtering approach provided us with a small but compatible and informative set of studies. We had explored whether the available data could be mined for any information indicating expression of different LEF/TCF isoforms, but that proved impossible. Our analysis therefore only correlates the transcriptome with transcript expression from a LEF/TCF gene, potentially involving several potential gene product isoforms. Furthermore, since our analysis focuses on transcriptional responses, any post-translational functional modification of LEF/TCF proteins [62,63] remains beyond our ability to evaluate.

However, differences in TCF7 isoform expression between normal and tumor tissue have been described [23,63], and our analysis is at least consistent with a different mix of *TCF7* isoforms being expressed in normal and tumor tissue. More generally, it is well established that alternative isoforms are expressed from the same LEF/TCF gene [7,64]. Thus, future RNA-seq studies with deep enough sequencing are expected to distinguish different isoforms, as well as reveal regulation by Wnt signaling of alternative isoform expression in potential downstream target genes [65]. It would therefore be very interesting in a future meta-analysis to include RNAseq experiments from an even wider range of databases like cBioPortal [66] to extend the results obtained here. Additionally, future detailed functional investigation is needed and promises to be both important and informative. 

## 5. Conclusions

Our meta-analysis confirms differences in LEF/TCF gene expression in human colorectal cancer tissue and uncovers a correlation of this differential LEF/TCF gene expression with an altered transcriptome, which suggests differences in target gene regulation with likely relevance for tumor progression and metastasis. The analysis also reveals a likely feedback loop in tumor tissue from WNT/β-catenin/TCF7 signaling mediated transcriptional regulation in the nucleus to resulting changes in WNT receptor protein abundance at the membrane. Given the importance of WNT/β-catenin signaling for colorectal cancer and the diversity of known LEF/TCF expression and protein function, our analysis provides an important foundation for future studies to investigate LEF/TCF function and differential isoform expression in more detail.

## Figures and Tables

**Table 1:** Data Sets Mined in this Investigation.

Name	Description	Reference
GSE10950	24 colon normal and tumor pairs (48 arrays)	[26]
GSE20842	Paired samples of tumor and mucosa from a total of 65 patients (130 arrays). 30 of the patients carried mutated KRAS1. this data set was divided into two different studies: one with mutated KRAS (named «mutated») and one without (named «wild type»).	[27]
GSE25070	26 pairs of fresh frozen colorectal tumor and matched adjacent non-tumor tissue samples (52 arrays).	[28]
GSE44076	98 colorectal cancer patients and their pairs (196 arrays).	[29]
GSE46622	4 colorectal cancer patients and their pairs (8 arrays).	[30]
GSE50760	18 patients, colon and tumor paired (36 arrays).	[31]

**Table 2:** Correlated Transcriptome.

		More in Tumor	Less in Tumor
A: Largest Difference in the TCF7-Correlated Transcriptome between Tumor and Normal	Ranked Gene List Top 10	NECAB3, TCFL5, ZNRF3, IFT52, SLC2A12, SKP1, DYNLRB1, MBNL2, SSX2IP, CAMLG	WDFY4, TBC1D10C, TRAF3IP3, IKZF1, RASAL3, SP140, LTB, ARHGAP9, CD37, SASH3
	Associated Gene Ontology Top 5:	intraciliary transport involved in cilium assembly (10^−6^) intraciliary transport (10^−5^) protein-containing complex localization (10^−4^) Wnt signaling pathway (10^−4^) cell surface receptor signaling pathway involved in cell-cell signaling (10^−4^)	**lymphocyte activation (10^−27^)** **immune system process (10^−25^)** **regulation of immune system process (10^−20^)** **leukocyte activation (10^−20^)** **positive regulation of immune system process (10^−19^)**
B: Largest Difference in the *LEF1*-correlated transcriptome between Tumor and Normal	Ranked Gene List Top 10	SPRR2F, PDGFC, CAB39L, GRP, SOX11, COL12A1, ISM1, GLRB, DKK3, OLFML3	FAIM3, PVRIG, UGT1A10, CD79B, BANK1, PTPRCAP, BLK, NAPSB, FCRLA, CD19
	Associated Gene Ontology Top 5:	**extracellular matrix organization (10^−13^)** **cell adhesion (10^−12^)** **biological adhesion (10^−12^)** **extracellular structure organization (10^−12^)** **anatomical structure development (10^−8^)**	**immune system process (10^−21^)** **immune response (10^−18^)** **regulation of immune system process (10^−18^)** **regulation of lymphocyte activation (10^−15^)** **regulation of leukocyte activation (10^−14^)**
C: Largest Difference in the *TCF7L1*-correlated transcriptome between Tumor and Normal	Ranked Gene List Top 10	DCBLD1, HMHA1, ARHGAP17, SH3KBP1, LXN, TMIGD1, SLC24A6, JAG1, LRCH4, VAMP8	SSX2IP, SAE1, RHEB, CKMT2, CCNI, NAP1L1, BAG2, ZFAND1, PHGDH, RPS5
	Associated Gene Ontology Top 5:	angiogenesis (10^−4^) anatomical structure formation involved in morphogenesis (10^−4^) positive regulation of neuron death (10^−4^) blood vessel endothelial cell proliferation involved in sprouting angiogenesis (10^−4^) regulation of mast cell activation involved in immune response (10^−4^)	organic substance metabolic process (10^−5^) cellular metabolic process (10^−5^) metabolic process (10^−5^) primary metabolic process (10^−4^) nucleotide-excision repair, DNA damage recognition (10^−4^)
D: Largest Difference in the *TCF7L2*-correlated transcriptome between Tumor and Normal	Ranked Gene List Top 10	C3orf70, CDC42EP2, DNAJC24, FLJ25758, CDRT15, HIG2, CIT, GNG3, C1orf77, TSPAN5	ACP5, AOAH, SCAND3, PPP1R14C, KIAA0247, DNAH14, NECAP2, CHMP1B, C5orf20, CTSD
	Associated Gene Ontology Top 5:	optic nerve development (10^−4^)	immune system process (10^−6^) negative regulation of myeloid leukocyte mediated immunity (10^−5^) negative regulation of mast cell activation (10^−5^) negative regulation of eosinophil activation (10^−5^) negative regulation of MyD88-dependent toll-like receptor signaling pathway (10^−5^)
E: Largest Difference in the AXIN2-correlated transcriptome between Tumor and Normal	Ranked Gene List Top 10	NKD1, CAB39L, APCDD1, NRXN3, PRSS23, LY6G6D, CCDC46, PPP2R2C, HABP4, CKMT2	GFI1, ADAM28, MFSD1, TOR1B, CD8A, FAS, SPIB, MBP, SLAMF1, RIOK3
	Associated Gene Ontology Top 5:	regulation of cellular localization (10^−4^) cellular response to gamma radiation (10^−4^) Wnt signaling pathway (10^−4^) regulation of cell communication (10^−4^) regulation of vascular endothelial growth factor receptor signaling pathway (10^−4^)	adaptive immune response based on somatic recombination of immune receptors built from immunoglobulin superfamily domains (10^−^^5^) lymphocyte mediated immunity (10^−^^5^) immune response (10^−^^5^) immune system process (10^−^^5^) pancreatic A cell differentiation (10^−^^5^)
F: Largest Difference in the *DKK1*-correlated transcriptome between Tumor and Normal	Ranked Gene List Top 10	MIZF, BMP4, SLITRK6, OR7E91P, MGC34774, PANK3, PAQR8, ATAD4, GDA, GPR110	UCHL5IP, DPH1, LOC399744, PDGFA, MEIS3P1, PER2, NDNL2, MAFK, CCND2, STARD13
	Associated Gene Ontology Top 5:	lipid catabolic process (10^−5^) cellular lipid catabolic process (10^−4^) molting cycle process (10^−4^) hair cycle process (10^−4^)	positive regulation of ATPase activity (10^−^^4^)
G: Largest Difference in the *FZD7*-correlated transcriptome between Tumor and Normal	Ranked Gene List Top 10	RNFT1, DERL1, FAM49B, FAM91A1, CA13, SLC7A8, ARFGEF1, HTATIP2, B3GNT2, NUP62CL	FGFBP2, KANK1, SORBS2, GPR155, TSHZ1, PCSK2, CCDC92, SEMA3A, FAM149A, HOXA10
	Associated Gene Ontology Top 5:	Golgi reassembly (10^−4^)	anatomical structure development (10^−^^4^) developmental process (10^−^^4^) regulation of heart rate by chemical signal (10^−^^4^) proximal/distal pattern formation (10^−^^4^) negative regulation of protein polymerization (10^−^^4^)
H: Largest Difference in the *LGR5*-correlated transcriptome between Tumor and Normal	Ranked Gene List Top 10	ZAK, ISM2, SRPK3, SATB1, GRP, ACOT9, ZCCHC12, KLHL23, HEY2, FAHD2B	DHDDS, MGC29506, FAIM3, CHP2, FAM46C, SLC46A3, TMBIM6, POU2AF1, MEI1, BCL2L15
	Associated Gene Ontology Top 5:	regulation of systemic arterial blood pressure by atrial natriuretic peptide (10^−5^) regulation of developmental process (10^−5^) extracellular matrix organization (10^−5^) extracellular structure organization (10^−4^) regulation of systemic arterial blood pressure by hormone (10^−4^)	**B cell receptor signaling pathway (10^−10^)** adaptive immune response (10^−^^9^) immune system process (10^−^^7^) antigen receptor-mediated signaling pathway (10^−^^6^) immune response (10^−^^6^)

Transcriptome correlated to TCF7 (**A**), LEF1 (**B**), TCF7L1 (**C**), TCF7L2 (**D**), AXIN2 (**E**), DKK1 (**F**), FZD7 (**G**), and LGR5 (**H**), largest differences between normal and tumor tissue. (ranked lists of top 10 genes of mean differences (*p* ≤ 0.05) with top 5 Gene Ontology terms listed if *p*-value <10^−3^, shaded if 10^−4^ < 10^−5^, in normal font if 10^−6^ < 10^−9^, and in bold if <10^−10^, see also Supplementary Table S1B–I and Supplementary Table S2).

**Table 3:** Comparison of LEF/TCF-Correlated Transcriptomes.

A: Differences between TCF7 and LEF1-correlated Transcriptomes			**Higher *TCF7* Correlation**	**Higher *LEF1* Correlation**
	In Normal Tissue	Ranked Gene List Top 10	FLJ46257, AADACL4, OR4D9, OR8U9, OR1S1, KRTAP6-1, TRIM6-TRIM34, OR5H15, OR4M2, OSTN	IFNA1, AADACL1, LEF1, SCYL1BP1, EIF4EBP3, POLR2J3, FAM18B, WDR40A, NME1-NME2, RTCD1, OR51A2
		Associated Gene Ontology Top 5	**detection of chemical stimulus involved in sensory perception of smell (10^−30^)** **detection of chemical stimulus involved in sensory perception (10^−29^)** **detection of stimulus involved in sensory perception (10^−27^)** **detection of stimulus (10^−23^)** **G protein-coupled receptor signaling pathway (10^−16^)**	flavone metabolic process (10^−4^) flavonoid glucuronidation (10^−4^)
	In Tumor Tissue	Ranked Gene List Top 10	GBL, C2orf24, NECAB3, EIF6, MGAT4B, ACOT8, ACSF3, TMUB1, SLC35C2, OR2J3	C5orf13, C14orf139, ST3GAL6, ECM2, DOCK10, PDGFC, SLFN11, MDFIC, SGIP1, RASGRP3
		Associated Gene Ontology Top 5	positive regulation of mitochondrial translation (10^−4^) sulfur compound metabolic process (10^−4^) glycerol metabolic process (10^−4^) positive regulation of cellular amide metabolic process (10^−4^) alditol metabolic process (10^−4^)	**extracellular structure organization** **(10** **^−^** **^11^** **)** **extracellular matrix organization** **(10** **^−^** **^10^** **)** anatomical structure morphogenesis (10^−^^8^) regulation of cell migration (10^−^^8^) regulation of cell motility (10^−^^7^)
B: Differences between TCF7- and TCF7L1-correlated transcriptomes			**Higher *TCF7* Correlation**	**Higher *TCF7L1* Correlation**
	In Normal Tissue	Ranked Gene List Top 10	HMHA1, PTPN7, SP140, PTPRCAP, CD6, FAIM3, DENND2D, SYK, LCK, DENND1C	FAM127C, JAZF1, TSPAN2, ZEB1, CAP2, FBXL2, MEIS1, CYS1, AGTR1, EHBP1
		Associated Gene Ontology Top 5	**regulation of immune system process (10** **^−15^)** **regulation of lymphocyte activation (10** **^−15^)** **regulation of cell activation (10** **^−14^)** **regulation of leukocyte activation (10** **^−14^)** **immune system process (10** **^−13^)**	**muscle system process (10^−10^)** muscle contraction (10^−9^) actin-mediated cell contraction (10^−5^) system process (10^−5^) regulation of muscle contraction (10^−5^)
	In Tumor Tissue	Ranked Gene List Top 10	C20orf118, VDAC1, EIF6, ETV4, MST4, GPR89A, TRAP1, UBAC2, EPHA1, ARPC1B	TMEM91, PLAC9, FAM127C, SDPR, CAND2, TANC2, SYN2, C6orf204, RHOJ, ADH1B
		Associated Gene Ontology Top 5	chromosome segregation (10^−^^5^) pteridine-containing compound metabolic process (10^−^^5^) pteridine-containing compound biosynthetic process (10^−^^5^) viral process (10^−^^5^) symbiont process (10^−^^4^)	cell adhesion (10^−^^5^) biological adhesion (10^−^^5^) cyclic nucleotide metabolic process (10^−^^5^) cGMP metabolic process (10 **^−^** ^4^ ) melanocyte differentiation (10 **^−^** ^4^ )
C: Differences between TCF7- and TCF7L2-correlated transcriptomes			**Higher *TCF7* Correlation**	**Higher *TCF7L2* Correlation**
	In Normal Tissue	Ranked Gene List Top 10	FAM113B, GRAP, LYL1, HVCN1, CXCR5, FAM65B, FAM129C, LIMD2, KRI1, CCL21	CCDC68, ASAP2, B3GNT2, TMEM56, SH3RF1, FAM177A1, FAM126B, UGP2, MICALCL, MOBKL2B
		Associated Gene Ontology Top 5	lymphocyte activation (10^−^^6^) regulation of T cell activation (10^−^^6^) B cell activation (10^−^^5^) leukocyte activation (10^−^^5^) positive regulation of double-strand break repair via homologous recombination (10^−^^5^)	actin filament-based process (10^−^^5^) actin cytoskeleton organization (10^−^^5^) regulation of cytoskeleton organization (10^−^^5^) forebrain astrocyte development (10^−^^5^) midbody abscission (10^−^^4^)
	In Tumor Tissue	Ranked Gene List Top 10	TMEM198, PRPF6, GTF3C5, EIF3G, DSN1, SLC35C2, NECAB3, EIF6, RELL2, SNHG11	C18orf32, HEATR5A, ASAP2, SGMS2, DDX60L, FGD4, LGR4, SMCHD1, TBC1D12, ZG16
		Associated Gene Ontology Top 5	heterocycle metabolic process (10^−^^4^) cellular nitrogen compound metabolic process (10^−^^4^) nucleobase-containing compound metabolic process (10^−^^4^) snRNA modification (10^−^^4^) viral translational termination-reinitiation (10^−^^4^)	regulation of viral-induced cytoplasmic pattern recognition receptor signaling pathway (10^−^^4^) sphingomyelin biosynthetic process (10^−^^4^) regulation of MDA-5 signaling pathway (10^−^^4^)
D: Differences between LEF1- and TCF7L1-correlated transcriptomes			**Higher *LEF1* Correlation**	**Higher *TCF7L1* Correlation**
	In Normal Tissue	Ranked Gene List Top 10	HMHA1, DENND2D, UGT1A10, SSH2, BCL11B, SP140, CD6, STX19, ZNF101, EZH2	FAM127C, LOC401431, CAP2, WDR86, DDR2, MAP6, FILIP1, ILK, GNAO1, ZEB1
		Associated Gene Ontology Top 5	**regulation of immune system process (10^−11^)** **immune system process (10^−11^)** **regulation of cell activation (10^−10^)** **regulation of lymphocyte activation (10^−10^)** regulation of cytokine production (10^−9^)	muscle contraction (10^−^^7^) muscle system process (10^−^^6^) system process (10^−^^6^) supramolecular fiber organization (10^−^^5^) cytoskeleton organization (10^−^^5^)
	In Tumor Tissue	Ranked Gene List Top 10	VDAC1, NCAPG2, NOMO3, EZH2, GPR89A, FAM72A, NOP16, DNAJC2, USP6NL, C20orf118	TMEM91, SYPL2, PLAC9, DEFB124, ADH1B, ZCWPW2, NBPF10, JPH4, RNF165, LOC401431
		Associated Gene Ontology Top 5	nucleic acid metabolic process (10^−^^9^) DNA metabolic process (10^−^^8^) double-strand break repair via homologous recombination (10^−^^8^) nucleobase-containing compound metabolic process (10^−^^7^) cell cycle process (10^−^^7^)	synaptic signaling (10^−^^5^) trans-synaptic signaling (10^−^^5^) modulation of chemical synaptic transmission (10^−^^4^) regulation of trans-synaptic signaling (10^−^^4^) cell communication (10^−^^4^)
E: Differences between LEF1- and TCF7L2-correlated transcriptomes			**Higher *LEF1* Correlation**	**Higher *TCF7L2* Correlation**
	In Normal Tissue	Ranked Gene List Top 10	LYL1, GRAP, HHEX, STMN3, FAM113B, MFNG, EIF3G, FXYD5, FSCN1, DNMT1	ASAP2, XIAP, MED13, TBC1D12, FAM120AOS, ACAP2, BCL2L15, MPZL3, FNIP2, SH3RF1
		Associated Gene Ontology Top 5	immune system process (10^−8^) regulation of dendritic cell dendrite assembly (10^−7^) B cell activation (10^−6^) dendritic cell chemotaxis (10^−6^) positive regulation of biological process (10^−6^)	positive regulation of protein localization to endosome (10^−^^5^) negative regulation of cytoplasmic translational elongation (10^−^^5^) forebrain astrocyte development (10^−^^5^) regulation of protein localization to endosome (10^−^^5^) 5-methylcytosine catabolic process (10^−^^5^)
	In Tumor Tissue	Ranked Gene List Top 10	STRA6, CDH11, PDGFC, DIO2, ADAMTSL2, HEYL, TMEM204, NUAK1, SGIP1, VCAN	UGT1A10, ZNF774, VPS37B, ASAP2, HK2, LGR4, ZG16, EZR, FGD4, TSPAN15
		Associated Gene Ontology Top 5	**extracellular matrix organization (10^−20^)** **extracellular structure organization (10^−19^)** **animal organ morphogenesis (10^−11^)** **anatomical structure morphogenesis (10^−10^)** **developmental process (10^−10^)**	flavone metabolic process (10^−^^4^) negative regulation of cytokine secretion (10^−^^4^) flavonoid glucuronidation (10^−^^4^) regulation of cellular response to insulin stimulus (10^−^^4^) negative regulation of cytokine production (10^−^^4^)
F: Differences between TCF7L1- and TCF7L2-correlated transcriptomes			**Higher *TCF7L1* Correlation**	**Higher *TCF7L2* Correlation**
	In Normal Tissue	Ranked Gene List Top 10	TSPAN18, FAM127A, FAM127C, CLIP3, EFEMP2, ZBTB47, DACT3, EHD2, CFL2, DBN1	TMIGD1, NDFIP2, RAB11FIP1, TMEM45B, CDCP1, CLIC5, TMEM87B, BCL10, ABHD3, PTPN3
		Associated Gene Ontology Top 5	extracellular matrix organization (10^−^^8^) extracellular structure organization (10^−^^7^) muscle structure development (10^−^^7^) developmental process (10^−^^7^) biological adhesion (10^−^^7^)	cell-cell junction organization (10^−^^9^) cell junction organization (10^−^^8^) cardiac muscle cell-cardiac muscle cell adhesion (10^−^^8^) bundle of His cell-Purkinje myocyte adhesion involved in cell communication (10^−^^6^) regulation of action potential (10^−^^6^)
	In Tumor Tissue	Ranked Gene List Top 10	FAM127C, TMEM91, WTIP, DEFB124, RASL12, RAMP3, NRIP2, PALM, MAP6, TSPAN18	C9orf152, PKP2, F2RL1, C11orf53, MRPS35, TWF1, GNPNAT1, PLEK2, BCL10, TMPO
		Associated Gene Ontology Top 5	**cell adhesion (10^−10^)** **biological adhesion (10^−10^)** angiogenesis (10^−^^8^) extracellular matrix organization (10^−^^8^) anatomical structure formation involved in morphogenesis (10^−^^8^)	nucleobase-containing small molecule metabolic process (10^−^^6^) nucleotide-sugar metabolic process (10^−^^5^) carbohydrate derivative metabolic process (10^−^^5^) cell-cell junction organization (10^−^^4^) cell junction organization (10^−^^4^)

Comparison of transcriptome differentially correlated with LEF/TCF gene expression. Pairwise differences between two LEF/TCF genes in transcriptome correlation, comparing *TCF7* with *LEF1* (**A**), *TCF7* with *TCF7L1* (**B**), *TCF7* with *TCF7L2* (**C**), *LEF1* with *TCF7L1* (**D**), *LEF1* with *TCF7L2* (**E**) and *TCF7L1* with *TCF7L2* (**F**). (GO terms listed if *p*-value <10^−3^, shaded if 10^−4^ < 10^−5^, in normal font if 10^−6^ < 10^−9^, and in bold if <10^−10^). Also see Supplementary Table S1J and Supplementary Table S3.

**Table 4:** Comparison between *AXIN2*- and LEF/TCF-Correlated Transcriptomes.

A: Differences between AXIN2- and TCF7-correlated transcriptomes			**Less *TCF7* Correlation**	**Higher *TCF7* Correlation**
	In Normal Tissue	Ranked Gene List Top 10	AADACL1, FLJ21511, SCYL1BP1, RICS, WDR40A, IQCK, MAK10, DKFZP564O0823, SPTLC1, FAM18B	FAM65B, WDFY4, CXCR5, GAPT, SASH3, HVCN1, OR5H15, LIMD2, PRKCB, FAM129C
		Associated Gene Ontology Top 5	**no GO enrichment found (<10^−3^)**	**lymphocyte activation (10^−21^)** **leukocyte activation (10^−18^)** **immune system process (10^−18^)** **cell activation (10^−16^)** **regulation of immune system process (10^−16^)**
	In Tumor Tissue	Ranked Gene List Top 10	ZNF776, UGT1A4, OR1L6, C10orf99, SIPA1L2, POLG2, CCDC46, EXPH5, ZNF283, PSMAL	OR4F5, OR4B1, C19orf35, OR5H15, OR2W5, NECAB3, OR2G3, ARPC1B, TMEM198, PLOD3
		Associated Gene Ontology Top 5	**mitochondrion morphogenesis (10^−4^) ** **regulation of inner ear receptor cell differentiation (10^−4^)** **regulation of inner ear auditory receptor cell differentiation (10^−4^)** **regulation of mechanoreceptor differentiation (10^−4^)**	detection of chemical stimulus involved in sensory perception of smell (10^−^^7^) detection of chemical stimulus (10^−^^7^) detection of stimulus involved in sensory perception (10^−^^6^) detection of stimulus (10^−^^6^) G protein-coupled receptor signaling pathway (10^−4^)
B: Differences between *AXIN2*- and *LEF1*-correlated transcriptomes			**Less *LEF1* Correlation**	**Higher *LEF1* Correlation**
	In Normal Tissue	Ranked Gene List Top 10	RNF43, FAM84A, PWP2, AIFM3, ATP7B, HIST2H3C, EHF, LPAR2, RICS, ENTPD6	SPP1, C7, AP1S2, SLC16A4, COPZ2, DFNA5, KCNMB1, ITGA1, TRPS1, PDGFRL
		Associated Gene Ontology Top 5	epithelial cell differentiation (10^−5^) negative regulation of stem cell proliferation (10^−5^) epithelial cell morphogenesis involved in placental branching (10^−5^) developmental process involved in reproduction (10^−4^) negative regulation of epidermis development (10^−4^)	muscle system process (10^−8^) muscle contraction (10^−6^) relaxation of muscle (10^−6^) relaxation of vascular smooth muscle **regulation of immune system process (10^−16^)**
	In Tumor Tissue	Ranked Gene List Top 10	LLGL2, C10orf99, SLC25A10, FAM83F, MPST, PARS2, EPHB2, RNF43, ZNF576, INPP5J	RFTN1, VCAN, FAP, ZEB2, TSHZ3, MEIS1, SULF1, COL12A1, PDGFC, PDGFRB
		Associated Gene Ontology Top 5	Wnt receptor catabolic process (10^−5^) glucuronate catabolic process (10^−4^) glucuronate catabolic process to xylulose 5-phosphate (10^−4^) xylulose 5-phosphate biosynthetic process (10^−4^)	1. **cell adhesion (10^−17^)** 2. **biological adhesion (10^−17^)** 3. **anatomical structure morphogenesis (10^−12^)** 5. **extracellular matrix organization (10^−12^)** 6. **extracellular structure organization (10^−11^)**
C: Differences between *AXIN2* and *TCF7L1*-correlated transcriptomes			**Less *TCF7L1* Correlation**	**Higher *TCF7L1* Correlation**
	In Normal Tissue	Ranked Gene List Top 10	EHF, C9orf152, GRHL2, LOC57228, MYO6, TOX3, FAM84A, MAP7, ACSM3, IHH	FAM127C, PCDH7, ZEB1, GLI3, COPZ2, FAM129A, NEXN, RBPMS2, FERMT2, BHMT2
		Associated Gene Ontology Top 5	epithelial cell differentiation (10^−8^) columnar/cuboidal epithelial cell differentiation (10^−6^) regulation of microvillus organization (10^−5^) actin filament bundle organization (10^−5^) epidermal cell differentiation (10^−5^)	muscle system process (10^−9^) muscle contraction (10^−9^) cell adhesion (10^−7^) biological adhesion (10^−7^) anatomical structure morphogenesis (10^−6^)
	In Tumor Tissue	Ranked Gene List Top 10	C20orf118, C9orf152, GGH, VWA2, VDAC1, C19orf48, LLGL2, MCM4, PDCD11, RNF43	TMEM91, FAM127C, PLAC9, SDPR, RHOJ, LATS2, TNFSF12, C16orf45, FERMT2, TENC1
		Associated Gene Ontology Top 5	nitrogen compound metabolic process (10^−6^) organic substance metabolic process (10^−5^) macromolecule metabolic process (10^−5^) cellular metabolic process (10^−5^) Wnt receptor catabolic process (10^−5^)	**cell adhesion (10^−11^)** **biological adhesion (10^−11^)** organ growth (10^−5^) bone growth (10^−5^) cyclic nucleotide metabolic process (10^−5^)
D: **Differences between *AXIN2*- and *TCF7L2*-correlated transcriptomes**			**Less *TCF7L2* Correlation**	**Higher *TCF7L2* Correlation**
	In Normal Tissue	Ranked Gene List Top 10	MMS19, KRI1, AOF2, MFF, FRAG1, C9orf163, RPL29P2, EIF3B, RBM35B, MFRP	SMCHD1, RNF19A, RGP1, PALM2-AKAP2, CWC22, FLJ20184, DENND5B, MPZL3, FAM108B1, C18orf32
		Associated Gene Ontology Top 5	**translational initiation (10^−10^)** nucleic acid metabolic process (10^−9^) RNA metabolic process (10^−8^) RNA processing (10^−7^) viral transcription (10^−7^)	regulation of protein polyubiquitination (10^−4^) negative regulation of cellular protein localization (10^−4^) regulation of cellular protein catabolic process (10^−4^)
	In Tumor Tissue	Ranked Gene List Top 10	LY6G6D, CAB39L, APCDD1, NKD1, DPEP1, KRT23, NOTUM, STRA6, ADAMTSL2, SESN1	C18orf32, SMCHD1, ASAP2, EIF4E3, SLC45A3, VAPA, MIA3, KITLG, DDX60L, LGR4
		Associated Gene Ontology Top 5	negative regulation of Wnt signaling pathway (10^−5^) Wnt receptor catabolic process (10^−5^) pulmonary valve morphogenesis (10^−5^) regulation of Wnt signaling pathway (10^−4^) receptor catabolic process (10^−4^)	positive regulation of multicellular organismal process (10^−5^) multivesicular body sorting pathway (10^−5^) negative regulation of cell-cell adhesion (10^−5^) positive regulation of viral release from host cell (10^−5^) regulation of locomotion (10^−5^)

Comparison of the *AXIN2*-correlated transcriptome with the *TCF7*- (**A**), *LEF1*- (**B**), *TCF7L1*- (**C**), *TCF7L2*- (**D**) -correlated transcriptomes in normal and tumor tissue. (gene list top 10 genes and top 5 GO terms listed if *p*-value <10^−3^, shaded if 10^−4^ < 10^−5^, in normal font if 10^−6^ < 10^−9^, and in bold if <10^−10^). Also see Supplementary Table S1K.

## Supplementary Materials

The following are available online at https://www.mdpi.com/2073-4425/11/5/538/s1: Figure S1: Principal Component Analysis of selected studies, Figure S2: Principal Component Analysis of de-selected study, Table S1: Transcriptomics Data (Correlation Coefficients) Table S1A: Transcript correlation between eight selected genes (*TCF7*, *LEF1*, *TCF7L1*, *TCF7L2*, *AXIN2*, *DKK1*, *FZD7*, *LGR5*); Table S1B: The *TCF7*-correlated transcriptome; Table S1C: The *LEF1*correlated transcriptome; Table S1D: The *TCF7L1*-correlated transcriptome; Table S1E: The *TCF7L2*-correlated transcriptome; Table S1F: The *AXIN2*-correlated transcriptome; Table S1G: The *DKK1*-correlated transcriptome; Table S1H: The *FZD7*-correlated transcriptome; Table S1I: The *LGR5*-correlated transcriptome; Table S1J: Differences in LEF/TCF-correlated transcriptomes; Table S1K: Differences between *AXIN2*- and LEF/TCF-correlated transcriptomes, Table S2: Correlated Transcriptome in normal and tumor tissue, Table S3: Comparison of LEF/TCF-correlated transcriptomes, Table S4: Differences between AXIN1- and LEF/TCF-correlated transcriptomes.
