# Supplementary material for: Diverse LEF/TCF Expression in Human Colorectal Cancer Correlates with Altered Wnt-Regulated Transcriptome in a Meta-Analysis of Patient Biopsies
Source: Genes (Basel). 2020 May 11;11(5):538. doi: 10.3390/genes11050538 (PMC7288467; doi:10.3390/genes11050538)
Supplement: Supplementary file 1 [file genes-11-00538-s001.zip › Supplementary/Supplementary Figures.docx]

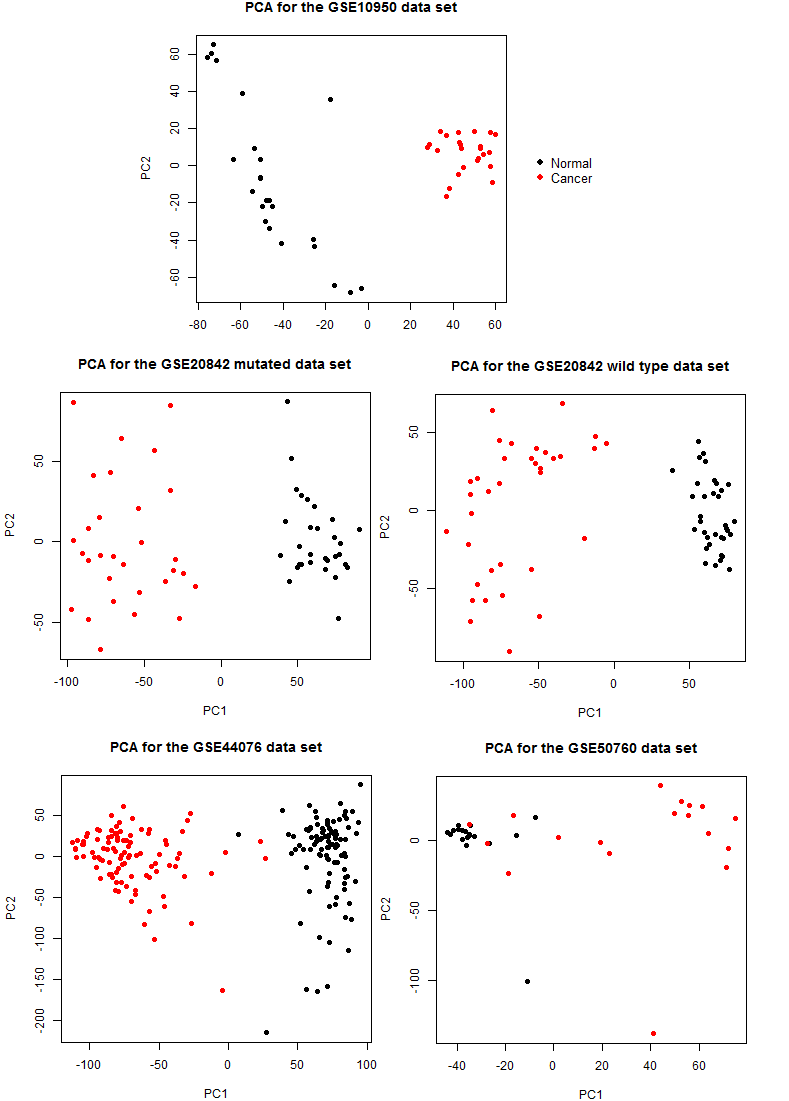


**A**

**C**

**B**

**E**

**D**

**Figure S1.** Principal Component Analysis (PCA) of studies as indicated (see Table 1 for more detail). As an additional quality control a PCA for each individual study was carried out to confirm separation of the transcriptome between tumor sample (red dots) and normal control (black dots) in all selected individual studies as indicated. Note that for these studies we can easily identify two clusters corresponding to the tumor samples and for normal samples. The Principal Component 1 (PC1, parallel to the X-axis in the graphs) clearly separates them in most studies (**A**–**D**) and separates them to a satisfactory degree (**E**).


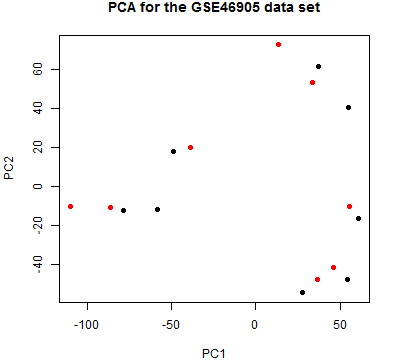


**Figure S2.** Principal Component Analysis (PCA) for the GSE46905 data set [32]. In the data from this study separation of the transcriptome between tumor sample (red dots) and normal control (black dots) in the PCA analysis is not possible. Moreover, since the number of patients in this study was relatively low, we removed this data set from the study.
