# Supplementary material for: Diverse LEF/TCF Expression in Human Colorectal Cancer Correlates with Altered Wnt-Regulated Transcriptome in a Meta-Analysis of Patient Biopsies
Source: Genes (Basel). 2020 May 11;11(5):538. doi: 10.3390/genes11050538 (PMC7288467; doi:10.3390/genes11050538)
Supplement: Supplementary file 1 [file genes-11-00538-s001.zip › Supplementary/Table S2.docx]

**Table S2: CORRELATED TRANSCRIPTOME**

**A: The TCF7-correlated transcriptome:**

|  | **Highest correlation in Normal Tissue:** | | **Highest correlation in Tumor Tissue:** | |
| --- | --- | --- | --- | --- |
|  | **Positive correlation:** | **Negative correlation:** | **Positive correlation:** | **Negative correlation:** |
| **Ranked Gene List Top 10:** | CXCR5, SASH3, RASAL3, WDFY4, FAM129C, FAM65B, ITGAL, FCRLA, NLRC3, IKZF1 | ENTPD3, SYNC, VPS37A, TTC33, AZI2, UQCRB, RPRD1A, SLAIN2, FBXO8, SOCS2 | ETV4, SLC5A6, TMEM198, NKD1, RALY, NECAB3, UCKL1, AHCY, DPEP1, ERGIC3 | BIRC2, FABP5, KIR2DL5B, XAGE2, NUMB, ZNF776, SGMS2, EIF4E3, DNAJC28, GLOD4 |
| **Associated Gene Ontology Top 5:** | 1. **adaptive immune response (10^‑12^)** 2. **regulation of immune system process (10^‑12^)** 3. **immune response (10^‑12^)** 4. **antigen receptor‑mediated signaling pathway (10^‑11^)** 5. **regulation of immune response (10^‑11^)** | no GO enrichment found (< 10^‑3^) | 1. regulation of canonical Wnt signaling pathway (10^‑5^) 2. positive regulation of Wnt signaling pathway (10^‑4^) 3. ncRNA metabolic process (10^‑4^) 4. regulation of Wnt signaling pathway (10^‑4^) 5. positive regulation of canonical Wnt signaling pathway (10^‑4^) | 1. regulation of autophagy (10^‑4^) 2. vesicle docking (10^‑4^) |

**B: The LEF1-correlated transcriptome:**

|  | **Highest correlation in Normal Tissue:** | | **Highest correlation in Tumor Tissue:** | |
| --- | --- | --- | --- | --- |
|  | **Positive correlation:** | **Negative correlation:** | **Positive correlation:** | **Negative correlation:** |
| **Ranked Gene List Top 10:** | FAIM3, RASAL3, PVRIG, CCR7, GRAP, BANK1, CD19, CXCR5, LTB, SUSD3 | NT5DC3, SPRR2F, OR2T35, SLAIN2, SYNC, ADH1B, BTC, ZCWPW2, WDR86, ATL3 | ADAMTSL2, COL12A1, TNFRSF19, DIO2, ITGA11, CCDC3, KIF26B, PDGFRB, COL5A2, APCDD1 | KIR2DL5B, SULT1A1, C6orf136, TSPAN15, KRTAP10-11, PWWP2B, ASPG, SLC25A23, CLMN, TMEM171 |
| **Associated Gene Ontology Top 5:** | 1. **immune system process (10^‑30^)** 2. **positive regulation of immune system process (10^‑24^)** 3. **regulation of immune system process (10^‑22^)** 4. **immune response (10^‑21^)** 5. **regulation of lymphocyte activation (10^‑20^)** | 1. Mullerian duct regression (10^‑4^) | 1. **extracellular matrix organization (10^‑20^)** 2. **extracellular structure organization (10^‑19^)** 3. **anatomical structure morphogenesis (10^‑14^)** 4. **skeletal system development (10^‑13^)** 5. **animal organ morphogenesis (10^‑13^)** | 1. phosphatidic acid biosynthetic process (10^‑5^) 2. phosphatidic acid metabolic process (10^‑5^) 3. glycerolipid biosynthetic process (10^‑4^) 4. diacylglycerol biosynthetic process (10^‑4^) 5. reticulophagy (10^‑4^) |

**C: The TCF7L1-correlated transcriptome:**

|  | **Highest correlation in Normal Tissue:** | | **Highest correlation in Tumor Tissue:** | |
| --- | --- | --- | --- | --- |
|  | **Positive correlation:** | **Negative correlation:** | **Positive correlation:** | **Negative correlation:** |
| **Ranked Gene List Top 10:** | FAM127C, GLI3, ZEB1, ARHGAP23, COL6A1, RAI2, PCDH7, JAZF1, EHBP1, FILIP1 | CALML4, C9orf152, STX19, CCDC109A, DENND2D, ZDHHC23, BAZ1A, HTATIP2, EHF, MAL2 | PLAC9, TSPAN18, NOTCH4, DCHS1, COL18A1, SH3PXD2A, NRIP2, LZTS2, BMPR2, NAV1 | C9orf152, VDAC1, DCTPP1, YEATS4, NUP37, THOC4, MAPKAPK5, XPNPEP1, MYCBP, LRRC8D |
| **Associated Gene Ontology Top 5:** | 1. animal organ morphogenesis (10^‑9^) 2. anatomical structure morphogenesis (10^‑9^) 3. regulation of developmental process (10^‑8^) 4. regulation of multicellular organismal process (10^‑8^) 5. cell adhesion (10^‑8^) | 1. regulation of translational initiation by eIF2 alpha phosphorylation (10^‑4^) 2. sulfide oxidation (10^‑4^) 3. sulfide oxidation, using sulfide:quinone oxidoreductase (10^‑4^) 4. negative regulation of nitric-oxide synthase biosynthetic process (10^‑4^) 5. hydrogen sulfide metabolic process (10^‑4^) | 1. **extracellular matrix organization (10^‑11^)** 2. **extracellular structure organization (10^‑10^)** 3. negative regulation of developmental process (10^‑6^) 4. collagen fibril organization (10^‑6^) 5. anatomical structure morphogenesis (10^‑6^) | 1. cellular nitrogen compound metabolic process (10^‑7^) 2. protein-containing complex subunit organization (10^‑7^) 3. cellular metabolic process (10^‑7^) 4. cellular protein-containing complex assembly (10^‑6^) 5. heterocycle metabolic process (10^‑6^) |

**D: The TCF7L2-correlated transcriptome:**

|  | **Highest correlation in Normal Tissue:** | | **Highest correlation in Tumor Tissue:** | |
| --- | --- | --- | --- | --- |
|  | **Positive correlation:** | **Negative correlation:** | **Positive correlation:** | **Negative correlation:** |
| **Ranked Gene List Top 10:** | TMIGD1, SH3RF1, MXD1, MIER3, MOBKL2B, CHMP1B, EFNB1, GOLIM4, MPZL3, KIF16B | CDK2AP1, NME7, NAT14, PDCL3, S1PR3, FAM127A, MFF, GLIPR2, ZBTB47, VPS37D | AOF1, BAT2D1, LGR4, CTAG1B, UGT1A10, ASAP2, MYSM1, FGD4, ATP1B1, C17orf74 | PLA2G4B, TTTY7, TNFRSF4, OR1D4, C17orf79, S1PR3, ZNF771, C20orf103, OLFM1, DNAH14 |
| **Associated Gene Ontology Top 5:** | 1. positive regulation of protein localization to endosome (10^‑5^) 2. positive regulation of calcium:sodium antiporter activity (10^‑5^) 3. regulation of protein localization to endosome (10^‑5^) 4. regulation of calcium:sodium antiporter activity (10^‑5^) 5. filopodium assembly (10^‑4^) | 1. regulation of epithelial to mesenchymal transition (10^‑5^) | 1. regulation of plasma membrane bounded cell projection organization (10^‑4^) 2. regulation of cell projection organization (10^‑4^) 3. actin filament organization (10^‑4^) 4. negative regulation of cholesterol storage (10^‑4^) 5. positive regulation of cell projection organization (10^‑4^) | no GO enrichment found (<10^‑3^) |

**E: The AXIN2-correlated transcriptome:**

|  | **Highest correlation in Normal Tissue:** | | **Highest correlation in Tumor Tissue:** | |
| --- | --- | --- | --- | --- |
|  | **Positive correlation:** | **Negative correlation:** | **Positive correlation:** | **Negative correlation:** |
| **Ranked Gene List Top 10:** | RNF43, FAM84A, AIFM3, ATP7B, EHF, LPAR2, RICS, ENTPD6, CYP2S1, OSBP2 | SPP1, SLC16A4, COPZ2, DFNA5, KCNMB1, ITGA1, TRPS1, PDGFRL, MYLK, TMEM45A | NKD1, APCDD1, RNF43, NOTUM, ZNRF3, LY6G6D, DPEP1, IRF2BP2, VWA2, CXXC5 | HCST, PRDM8, STX12, SSBP2, CD33, LHFPL2, NT5E, CD52, LPAR1, C14orf45 |
| **Associated Gene Ontology Top 5:** | 1. epithelial cell differentiation (10^‑5^) 2. establishment or maintenance of cell polarity (10^‑5^) 3. negative regulation of stem cell proliferation (10^‑5^) 4. establishment of cell polarity (10^‑5^) 5. epithelial cell morphogenesis involved in placental branching (10^‑5^) | 1. muscle system process (10^‑7^) 2. muscle contraction (10^‑5^) 3. cell adhesion (10^‑5^) 4. biological adhesion (10^‑5^) 5. relaxation of muscle (10^‑4^) | 1. regulation of Wnt signaling pathway (10^‑7^) 2. negative regulation of Wnt signaling pathway (10^‑6^) 3. molting cycle process (10^‑6^) 4. hair cycle process (10^‑6^) 5. regulation of canonical Wnt signaling pathway (10^‑6^) | 1. immune system process (10^‑7^) 2. immune response (10^‑7^) 3. regulation of cell shape (10^‑6^) 4. defense response (10^‑6^) 5. regulation of immune (10^‑6^) |

**F: The DKK1-correlated transcriptome:**

|  | **Highest correlation in Normal Tissue:** | | **Highest correlation in Tumor Tissue:** | |
| --- | --- | --- | --- | --- |
|  | **Positive correlation:** | **Negative correlation:** | **Positive correlation:** | **Negative correlation:** |
| **Ranked Gene List Top 10:** | HBG2, HGSNAT, PMEPA1, DNAJB1, UCHL5IP, CLCF1, EIF1, SERTAD1, OR4Q3, MST150 | MIZF, CDC2L6, FLJ41603, SLC6A10P, OR6N2, DCLRE1A, LOC63920, FLJ20674, ADAMTS19, ARVP6125 | BAMBI, MGC45800, KLK10, IGFL1, SERPINB5, NAT12, FLJ46257, GRHL3, RASSF9, AIM2 | OR8H2, OR5M3, PPP1R7, LRIG1, ATL1, N4BP2L1, FAM48A, CCND2, ZNF470, OR2T35 |
| **Associated Gene Ontology Top 5:** | 1. regulation of nucleic acid‑templated transcription (10^‑4^) 2. regulation of RNA biosynthetic process (10^‑4^) | no GO enrichment found (< 10^‑3^) | 1. cornification (10^‑4^) 2. anatomical structure morphogenesis (10^‑4^) 3. epidermis development (10^‑4^) 4. viral entry into host cell (10^‑4^) | 1. regulation of cellular macromolecule biosynthetic process (10^‑4^) 2. endosomal vesicle fusion (10^‑4^) 3. regulation of transcription, DNA-templated (10^‑4^) |

**G: The FZD7-correlated transcriptome:**

|  | **Highest correlation in Normal Tissue:** | | **Highest correlation in Tumor Tissue:** | |
| --- | --- | --- | --- | --- |
|  | **Positive correlation:** | **Negative correlation:** | **Positive correlation:** | **Negative correlation:** |
| **Ranked Gene List Top 10:** | AHNAK2, DENND5A, ZCCHC24, NACAD, NKX3-2, PRDM6, ZNF853, RAI2, HLX, KANK2 | FAM135A, OXNAD1, TMEM144, PAWR, TTC39B, FAM60A, FAM83B, CCDC125, BCL2L15, C5orf43 | LCA5, ITGA9, CCDC102A, ARHGAP23, FAM110B, HEG1, CERCAM, TPPP3, CHSY3, S1PR1 | UGT1A9, ATP2C2, TOX3, MNX1, OR8H2, METT11D1, IFNA13, A1CF, USP43, OR11H12 |
| **Associated Gene Ontology Top 5:** | 1. cardiac muscle tissue growth (10^‑5^) 2. protein transport from ciliary membrane to plasma membrane (10^‑5^) 3. glomerular visceral epithelial cell migration (10^‑5^) 4. dermatan sulfate proteoglycan metabolic process (10^‑4^) 5. system development (10^‑4^) | 1. establishment of protein localization (10^‑4^) 2. protein transport (10^‑4^) 3. peptide transport (10^‑4^) | 1. cardiac muscle tissue growth (10^‑5^) 2. leukotriene D4 biosynthetic process (10^‑4^) 3. leukotriene D4 metabolic process (10^‑4^) 4. lymph vessel development (10^‑4^) 5. signal complex assembly (10^‑4^) | 1. negative regulation of immature T cell proliferation (10^‑4^) 2. detection of chemical stimulus (10^‑4^) 3. detection of chemical stimulus involved in sensory perception of smell (10^‑4^) |

**H: The LGR5-correlated transcriptome:**

|  | **Highest correlation in Normal Tissue:** | | **Highest correlation in Tumor Tissue:** | |
| --- | --- | --- | --- | --- |
|  | **Positive correlation:** | **Negative correlation:** | **Positive correlation:** | **Negative correlation:** |
| **Ranked Gene List Top 10:** | KIAA1147, C9orf152, TET3, C20orf118, CYTH1, SPIN4, MNX1, HMGN1, C12orf66, TCF7 | FAM127C, ZCWPW2, WTIP, DAND5, C22orf39, KGFLP2, VPS37D, C1QTNF4, KANK2, CALY | FHDC1, ZNF260, TAB3, SOX4, PPM1H, CTPS2, ZXDB, C16orf88, TRAF5, ZNF195 | DHDDS, STX12, ST6GALNAC4, RHOC, C1orf144, CCND3, PEF1, AGPAT9, ATP6V0B, MUL1 |
| **Associated Gene Ontology Top 5:** | no GO enrichment found (< 10^‑3^) | 1. cell morphogenesis involved in differentiation (10^‑4^) | 1. regulation of gene expression (10^‑6^) 2. regulation of RNA metabolic process (10^‑5^) 3. regulation of nucleobase-containing compound metabolic process (10^‑5^) 4. regulation of nitrogen compound metabolic process (10^‑5^) 5. regulation of macromolecule metabolic process (10^‑5^) | 1. negative regulation of growth (10^‑4^) 2. activation of cysteine-type endopeptidase activity involved in apoptotic process by cytochrome c (10^‑4^) 3. cellular response to mechanical stimulus (10^‑4^) |

SUPPL.TABLE LEGEND: Transcriptome correlated to *TCF7* (A), *LEF1* (B), *TCF7L1* (C), *TCF7L2* (D), *AXIN2* (E), *DKK1* (F), *FZD7* (G), and *LGR5* (H), in paired normal and tumor tissue. (ranked lists of top 10 genes of averaged correlations (sd ≤ 0.2) with top 5 Gene Ontology (GO) terms listed if p-value <10^‑3^, shaded if 10^‑4^<10^-5^, in normal font if 10^‑6^<10^‑9^, and in bold if <10^‑10^) (see also Table 2 and Suppl. Table 1B-I).
