# Supplementary material for: Diverse LEF/TCF Expression in Human Colorectal Cancer Correlates with Altered Wnt-Regulated Transcriptome in a Meta-Analysis of Patient Biopsies
Source: Genes (Basel). 2020 May 11;11(5):538. doi: 10.3390/genes11050538 (PMC7288467; doi:10.3390/genes11050538)
Supplement: Supplementary file 1 [file genes-11-00538-s001.zip › Supplementary/Table S3.docx]

**Table S3: COMPARISON OF LEF/TCF-CORRELATED TRANSCRIPTOMES**

**Overall differences between LEF/TCF-correlated transcriptomes:**

|  | **Highest differential correlation in Normal Tissue:** | | **Highest differential correlation in Tumor Tissue:** | |
| --- | --- | --- | --- | --- |
| **Ranked Gene List:** | Gene Name | LEF/TCF correlation difference (pairwise) | Gene Name | LEF/TCF correlation difference (pairwise) |
| 1 | TMIGD1 | TCF7L2 high, TCF7L1 low | FAM127C | TCF7L1 high, TCF7L2 low |
| 2 | FAM127C | TCF7L1 high, TCF7L2 low | TMEM91 | TCF7L1 high, TCF7L2 low |
| 3 | TSPAN18 | TCF7L1 high, TCF7L2 low | WTIP | TCF7L1 high, TCF7L2 low |
| 4 | FAM127A | TCF7L1 high, TCF7L2 low | C9orf152 | TCF7L2 high, TCF7L1 low |
| 5 | CLIP3 | TCF7L1 high, TCF7L2 low | PLAC9 | TCF7L1 high, TCF7 low |
| 6 | EFEMP2 | TCF7L1 high, TCF7L2 low | C20orf118 | TCF7 high, TCF7L1 low |
| 7 | ZBTB47 | TCF7L1 high, TCF7L2 low | VDAC1 | TCF7 high, TCF7L1 low |
| 8 | DACT3 | TCF7L1 high, TCF7L2 low | C18orf32 | TCF7L2 high, TCF7 low |
| 9 | EHD2 | TCF7L1 high, TCF7L2 low | DEFB124 | TCF7L1 high, TCF7L2 low |
| 10 | CFL2 | TCF7L1 high, TCF7L2 low | HEATR5A | TCF7L2 high, TCF7 low |
| **Associated Gene Ontology Top 5:** | 1. extracellular matrix organization (10^‑7^) 2. extracellular structure organization (10^‑6^) 3. cell morphogenesis involved in differentiation (10^‑6^) 4. angiogenesis (10^‑5^) 5. developmental process (10^‑5^) | | 1. angiogenesis (10^‑4^) 2. cell adhesion (10^‑4^) 3. biological adhesion (10^‑4^) 4. negative regulation of membrane permeability (10^‑4^) 5. regulation of anatomical structure morphogenesis (10^‑4^) | |

TABLE LEGEND: Comparison of transcriptome differentially correlated with LEF/TCF gene expression. Highest differences between transcript correlation with different LEF/TCF gene expression (with gene list top 10, each of these genes explanation of difference in association with expression of which two LEF/TCF genes, and top 5 GO terms associated with those lists of top 100). (GO terms listed if p-value <10^‑3^, shaded if 10^‑4^<10^-5^, in normal font if 10^‑6^<10^‑9^, and in bold if <10^‑10^). Also see Suppl. Table 1J. and Table 3
