# Supplementary material for: Diverse LEF/TCF Expression in Human Colorectal Cancer Correlates with Altered Wnt-Regulated Transcriptome in a Meta-Analysis of Patient Biopsies
Source: Genes (Basel). 2020 May 11;11(5):538. doi: 10.3390/genes11050538 (PMC7288467; doi:10.3390/genes11050538)
Supplement: Supplementary file 1 [file genes-11-00538-s001.zip › Supplementary/Table S4.docx]

**Table S4: COMPARISON BETWEEN AXIN2- AND LEF/TCF-CORRELATED TRANSCRIPTOMES**

**Overall differences between AXIN2- and LEF/TCF-correlated transcriptomes:**

|  | **Highest differential correlation in Normal Tissue:** | | **Highest differential correlation in Tumor Tissue:** | |
| --- | --- | --- | --- | --- |
| **Ranked Gene List:** | Gene Name | LEF/TCF correlation difference (relative to AXIN2) | Gene Name | LEF/TCF correlation difference (relative to AXIN2) |
| 1 | FAM127C | More correlation with TCF7L1 | TMEM91 | More correlation with TCF7L1 |
| 2 | PCDH7 | More correlation with TCF7L1 | FAM127C | More correlation with TCF7L1 |
| 3 | ZEB1 | More correlation with TCF7L1 | PLAC9 | More correlation with TCF7L1 |
| 4 | GLI3 | More correlation with TCF7L1 | C20orf118 | Less correlation with TCF7L1 |
| 5 | COPZ2 | More correlation with TCF7L1 | SDPR | More correlation with TCF7L1 |
| 6 | FAM129A | More correlation with TCF7L1 | C9orf152 | Less correlation with TCF7L1 |
| 7 | NEXN | More correlation with TCF7L1 | LY6G6D | Less correlation with TCF7L2 |
| 8 | RBPMS2 | More correlation with TCF7L1 | RHOJ | More correlation with TCF7L1 |
| 9 | FERMT2 | More correlation with TCF7L1 | LATS2 | More correlation with TCF7L1 |
| 10 | BHMT2 | More correlation with TCF7L1 | TNFSF12 | More correlation with TCF7L1 |
| **Associated Gene Ontology Top 5:** | 1. muscle system process (10^‑9^) 2. muscle contraction (10^‑9^) 3. cell adhesion (10^‑7^) 4. biological adhesion (10^‑7^) 5. anatomical structure morphogenesis (10^‑6^) | | 1. **cell adhesion (10^‑10^)** 2. **biological adhesion (10^‑10^)** 3. regulation of developmental process (10^‑5^) 4. cell-substrate adhesion (10^‑4^) 5. cell-matrix adhesion (10^‑4^) | |

LEGEND: Comparison of the AXIN2-correlated transcriptome and the LEF/TCF-correlated transcriptomes. Overall differences in Normal Tissue and in Tumor Tissue with gene list top 10, each of these genes explanation of difference in association with expression of which LEF/TCF genes, and top 5 GO terms associated with those lists of top 100). Also see Suppl. Table 1K and Table 4.
