# Supplementary material for: Diverse LEF/TCF Expression in Human Colorectal Cancer Correlates with Altered Wnt-Regulated Transcriptome in a Meta-Analysis of Patient Biopsies
Source: Genes (Basel). 2020 May 11;11(5):538. doi: 10.3390/genes11050538 (PMC7288467; doi:10.3390/genes11050538)
Supplement: Supplementary file 1 [file Table S1.pdf › Supplementary/Table S1.pdf]

**ALL (R)**	TCF7	LEF1	TCF7L1	TCF7L2	AXIN2	DKK1
TCF7	1.000	0.367	0.110	0.009	0.301	0.004
LEF1	0.367	1.000	0.053	0.020	0.248	0.005
TCF7L1	0.110	0.053	1.000	0.062	0.199	0.004
TCF7L2	0.009	0.020	0.062	1.000	0.098	0.110
AXIN2	0.301	0.248	0.199	0.098	1.000	0.051
DKK1	0.004	0.005	0.004	0.110	0.051	1.000
FZD7	0.046	0.004	0.406	0.120	0.044	0.060
LGR5	0.244	0.249	0.145	0.150	0.309	0.024

**DIFF NvT (T)**	TCF7	LEF1	TCF7L1	TCF7L2	AXIN2	DKK1
TCF7	0.000	0.291	0.027	0.193	0.264	0.031
LEF1	0.291	0.000	0.313	0.278	0.065	0.162
TCF7L1	0.027	0.313	0.000	0.206	0.305	0.121
TCF7L2	0.193	0.278	0.206	0.000	0.209	0.165
AXIN2	0.264	0.065	0.305	0.209	0.000	0.138
DKK1	0.031	0.162	0.121	0.165	0.138	0.000
FZD7	0.127	0.170	0.430	0.236	0.257	0.160
LGR5	0.336	0.054	0.168	0.020	0.014	0.049

**Tumor (P)**	TCF7	LEF1	TCF7L1	TCF7L2	AXIN2	DKK1
TCF7	1.000	0.222	0.097	0.087	0.433	0.019
LEF1	0.222	1.000	0.104	0.158	0.280	0.076
TCF7L1	0.097	0.104	1.000	0.041	0.047	0.056
TCF7L2	0.087	0.158	0.041	1.000	0.006	0.192
AXIN2	0.433	0.280	0.047	0.006	1.000	0.018
DKK1	0.019	0.076	0.056	0.192	0.018	1.000
FZD7	0.018	0.089	0.191	0.001	0.084	0.020
LGR5	0.076	0.222	0.061	0.140	0.317	0.001

**Normal (N)**	TCF7	LEF1	TCF7L1	TCF7L2	AXIN2	DKK1
TCF7	1.000	0.513	0.123	0.105	0.169	0.012
LEF1	0.513	1.000	0.210	0.119	0.215	0.086
TCF7L1	0.123	0.210	1.000	0.123	0.351	0.064
TCF7L2	0.105	0.119	0.166	1.000	0.203	0.027
AXIN2	0.169	0.215	0.351	0.203	1.000	0.120
DKK1	0.012	0.086	0.064	0.027	0.120	1.000
FZD7	0.110	0.081	0.621	0.238	0.173	0.140
LGR5	0.413	0.276	0.229	0.160	0.302	0.049

FZD7	LGR5
0.046	0.244
0.004	0.249
0.406	0.145
0.120	0.150
0.044	0.309
0.060	0.024
1.000	0.055
0.055	1.000

FZD7	LGR5
0.127	0.336
0.170	0.054
0.430	0.168
0.236	0.020
0.257	0.014
0.160	0.049
0.000	0.216
0.216	0.000

FZD7	LGR5
0.018	0.076
0.089	0.222
0.191	0.061
0.001	0.140
0.084	0.317
0.020	0.001
1.000	0.053
0.053	1.000

FZD7	LGR5
0.110	0.413
0.081	0.276
0.621	0.229
0.238	0.160
0.173	0.302
0.140	0.049
1.000	0.162
0.162	1.000

Gene.Name	GSE10950_	GSE10950_	GSE20842..	GSE20842..	GSE20842..	GSE20842..	GSE25070_	GSE25070_
A1BG	-0.14932	0.250583	0.234743	0.173312	-0.16164	0.002191	0.282744	0.278863
A1CF	NA	NA	0.218938	-0.23903	0.096231	-0.19825	NA	NA
A2BP1	0.310729	-0.0295	-0.33226	0.170721	-0.08736	0.102512	0.220236	-0.08901
A2LD1	NA	NA	-0.07138	0.20986	-0.0392	-0.47055	NA	NA
A2M	-0.20437	-0.33864	0.062031	0.095979	-0.05253	0.464629	-0.64952	-0.56524
A2ML1	0.077359	0.347218	-0.24804	-0.13158	-0.2168	-0.16301	-0.10686	-0.24917
A3GALT2	NA	NA	0.215945	-0.34505	0.165289	-0.27964	0.109621	-0.06867
A4GALT	-0.33688	0.032823	0.232918	0.107915	0.219727	0.200429	-0.26195	-0.04974
A4GNT	0.300945	0.159748	-0.26774	-0.01191	0.048969	0.197536	0.18095	0.071181
AAA1	0.268989	0.465329	NA	NA	NA	NA	0.39785	0.399399
AAAS	0.246384	0.592832	0.23035	0.21333	0.103942	-0.04597	0.320229	0.33565
AACS	-0.31544	0.469728	0.044843	0.326282	0.255106	0.180588	0.392569	0.192546
AADAC	-0.12113	-0.03437	-0.21295	0.078064	-0.0083	-0.22424	0.12672	0.557404
AADACL1	-0.43348	-0.27055	-0.23619	-0.31927	-0.05453	-0.50724	-0.26379	-0.50935
AADACL2	-0.18413	-0.56773	0.166669	-0.3337	-0.08057	-0.07955	0.122842	0.253173
AADACL4	0.171565	0.711839	NA	NA	NA	NA	NA	NA
AADAT	-0.08095	0.486637	-0.43417	0.071328	-0.20546	-0.03545	0.359272	0.125509
AAK1	-0.01015	-0.2358	0.022337	-0.34292	-0.22265	-0.05354	0.310783	0.412136
AAMP	0.196529	0.046965	0.14961	0.141671	0.329447	-0.36479	-0.0421	-0.16969
AANAT	0.103708	-0.11364	0.165639	0.112754	0.145111	-0.0194	0.047214	0.34999
AARS	0.193522	0.175202	0.322482	0.279771	0.430575	0.089401	-0.06646	0.035313
AARS2	0.367787	0.393808	-0.28377	0.148696	0.024166	0.341932	NA	NA
AARSD1	-0.11376	-0.04007	0.067681	-0.12486	-0.00762	0.016684	0.207199	-0.0357
AASDH	-0.04845	-0.4597	-0.24887	-0.23392	-0.34226	-0.31961	0.142059	0.250242
AASDHPPT	-0.03425	-0.59212	-0.14367	-0.08338	-0.16467	-0.01741	0.137026	-0.05004
AASS	0.283613	-0.38785	0.086552	0.087476	-0.10993	0.137379	-0.23355	-0.06126
AATF	0.484814	0.557938	0.288269	0.315975	0.236291	0.222898	0.513636	0.341957
AATK	NA	NA	0.274891	0.167868	-0.08435	-0.19051	0.362616	0.307266
ABAT	0.367629	0.028969	0.163549	-0.27857	0.343186	-0.45657	-0.05441	-0.4455
ABCA1	-0.46468	-0.20602	0.104518	-0.32916	-0.1586	0.29167	-0.40923	-0.15154
ABCA10	0.044715	0.448162	-0.43206	0.08636	-0.34946	-0.19573	-0.00909	0.184471
ABCA11P	NA	NA	-0.20311	-0.08589	-0.21223	0.339654	NA	NA
ABCA12	0.221943	0.346667	-0.17711	0.299216	-0.09421	-0.12465	-0.29228	-0.47563
ABCA13	0.297124	0.162812	-0.17122	0.090652	-0.16422	-0.08473	-0.08157	0.105396
ABCA2	-0.34789	0.496005	0.114	0.007765	0.395655	0.075811	-0.07478	0.102958
ABCA3	-0.41245	-0.03886	0.01543	0.071649	0.016726	0.132083	-0.32277	-0.59097
ABCA4	-0.57226	0.005788	-0.06443	-0.00361	-0.05791	0.056988	0.359218	0.550346
ABCA5	-0.27363	-0.07142	0.202694	-0.24023	0.077482	-0.00096	0.133393	0.232283
ABCA6	-0.10503	-0.45333	-0.1351	-0.15731	-0.25953	0.09621	0.030879	0.208109
ABCA7	-0.23904	0.249507	0.210266	0.369176	0.212259	0.379958	-0.09004	0.360409
ABCA8	0.13668	-0.47829	0.055573	-0.04486	-0.1326	0.189701	-0.39292	-0.376
ABCA9	-0.21063	-0.59725	-0.22255	-0.00072	-0.0269	-0.07188	-0.09438	0.215021
ABCB1	0.305854	-0.02742	0.409259	-0.32574	-0.04493	-0.08741	0.249869	0.240427
ABCB10	-0.15531	-0.53859	-0.34899	-0.14959	-0.05749	-0.25848	0.11281	-0.13561
ABCB11	0.052759	0.059639	-0.06429	-0.24928	-0.21266	-0.0446	0.688621	0.06249
ABCB4	0.206684	-0.11857	0.082284	0.411078	0.107324	0.307071	-0.25499	0.113995
ABCB5	-0.01669	-0.25132	0.127632	-0.0489	0.052699	0.188872	-0.28967	-0.4297
ABCB6	-0.1861	0.142344	-0.03946	0.253971	0.337689	-0.36468	0.134511	0.227588
ABCB7	0.285815	0.529992	-0.08066	-0.13119	-0.20896	-0.2776	-0.50913	-0.58372
ABCB8	0.302832	0.67068	0.194588	0.074231	0.107423	-0.24292	0.236374	0.158482
ABCB9	0.183456	-0.32541	0.180137	-0.16107	0.301474	-0.10658	0.31459	0.085915

ABCC1	0.140077	-0.33726	0.12456	0.250195	0.225101	0.214822	0.201217	0.131323
ABCC10	0.257614	0.595153	0.315809	0.168883	0.384569	0.199226	0.335307	0.324098
ABCC11	0.222734	0.735953	-0.15457	0.053642	0.093806	0.072495	0.139666	-0.00359
ABCC12	0.24316	0.129377	-0.03631	-0.0382	0.094223	0.114525	-0.40339	-0.34379
ABCC13	0.154387	0.00142	-0.0257	-0.29464	-0.26595	-0.44871	0.112059	0.350651
ABCC2	-0.02544	0.034179	0.32697	-0.0716	0.040044	0.165834	0.401103	0.580765
ABCC3	-0.1183	0.184644	0.063478	0.268537	0.217922	-0.03677	0.462225	0.253093
ABCC4	0.375659	-0.54469	-0.24906	-0.06025	0.072202	0.064374	-0.26611	-0.53756
ABCC5	0.306241	-0.06442	-0.04325	-0.27785	0.364834	0.112371	-0.22252	-0.02298
ABCC6	0.208216	0.296512	0.215604	-0.21038	0.232848	-0.32065	0.312652	0.287764
ABCC8	-0.31096	0.052004	-0.1451	-0.07116	-0.17991	-0.33333	0.35686	0.607354
ABCC9	-0.44048	-0.5656	0.096931	-0.06517	-0.19493	0.045688	-0.34438	-0.05729
ABCD1	0.111985	0.206307	0.326557	-0.21231	0.211751	0.234549	-0.06136	0.30398
ABCD2	0.146484	0.138466	0.145937	0.201403	-0.07457	0.187874	0.126507	0.198787
ABCD3	-0.13915	-0.46364	-0.24021	-0.12621	-0.03523	-0.43381	NA	NA
ABCD4	0.10531	0.487147	-0.47797	-0.02096	-0.25566	-0.41522	0.604193	0.505091
ABCE1	-0.02866	-0.60946	-0.35844	0.131587	-0.16751	0.115636	0.462439	0.155379
ABCF1	0.328104	0.433069	-0.01991	0.172176	0.167425	0.508328	0.215954	0.458861
ABCF2	0.521308	-0.36464	0.018585	0.26816	0.158706	-0.05911	0.15181	0.084487
ABCF3	-0.05495	0.312401	0.300773	0.361322	0.171331	0.293288	0.431286	0.295004
ABCG1	0.041424	0.384199	0.364689	-0.29222	0.285658	-0.01881	-0.3166	-0.04808
ABCG2	0.399597	-0.29843	0.194075	-0.56493	-0.13573	-0.4416	0.035871	0.213127
ABCG4	-0.43392	-0.16644	-0.12311	-0.2167	-0.12789	0.165896	-0.45545	-0.60494
ABCG5	-0.04848	-0.15752	-0.00956	0.222345	-0.03007	-0.15324	-0.17336	0.326305
ABCG8	0.031183	0.56287	0.31344	0.274805	-0.36141	-0.24133	0.198235	0.519319
ABHD1	0.063666	-0.33782	0.114112	0.054763	-0.02199	0.228681	0.084372	-0.21926
ABHD10	-0.2746	0.174598	-0.35106	0.030252	-0.12982	-0.38149	0.640194	0.009097
ABHD11	-0.20634	-0.24736	0.099024	0.018874	0.186516	0.023004	0.253385	0.293153
ABHD12	0.181575	0.293799	-0.35732	-0.31351	-0.04606	-0.1677	0.440012	0.195353
ABHD12B	NA	NA	0.18201	-0.30023	-0.23874	0.241578	NA	NA
ABHD13	0.422827	0.574127	-0.04016	-0.33225	-0.03965	-0.54538	-0.13881	-0.30777
ABHD14A	0.059973	0.541461	-0.05351	0.201761	-0.18613	-0.1093	0.154996	-0.14949
ABHD14B	0.054125	0.432059	0.326378	0.056046	0.287936	0.039369	0.483352	0.382935
ABHD2	0.156281	0.450623	0.300733	0.212315	0.280922	-0.07906	-0.07155	-0.0499
ABHD3	-0.49322	-0.25257	-0.20739	-0.11451	-0.15011	-0.19426	0.115479	0.122922
ABHD4	-0.08444	0.487919	0.488728	-0.3645	-0.0874	0.023933	0.182884	0.325783
ABHD5	-0.21138	0.024976	-0.24037	-0.28156	-0.12801	-0.30472	-0.52738	-0.09621
ABHD6	0.184643	-0.05217	0.09031	-0.20139	0.137288	0.008961	-0.10273	0.238256
ABHD7	0.263508	-0.09042	0.478342	-0.04646	0.153133	-0.09216	-0.10734	-0.38428
ABHD8	-0.01947	0.174183	0.228662	-0.06796	0.049804	-0.04349	-0.38328	-0.43874
ABHD9	-0.0417	0.067701	0.182313	-0.03806	0.016218	0.158199	-0.45495	-0.55073
ABI1	-0.15803	0.046528	-0.01386	0.15823	-0.15058	-0.14558	-0.32221	-0.31554
ABI2	0.219324	-0.16236	0.179476	-0.03187	0.12484	0.096869	0.332586	-0.13683
ABI3	-0.0129	-0.39316	0.312393	0.428355	-0.09146	0.471093	-0.73578	-0.08658
ABI3BP	0.007878	-0.15344	-0.09556	0.127743	-0.07114	0.476267	-0.33395	-0.32539
ABL1	-0.03437	0.003905	-0.00136	-0.26333	0.256733	0.250403	-0.10257	0.133001
ABL2	-0.33093	-0.15457	-0.12394	-0.43303	-0.12461	-0.0414	-0.31846	0.086323
ABLIM1	-0.26453	-0.07069	-0.01105	0.05409	0.094838	0.392475	0.245479	0.218074
ABLIM2	0.380791	0.012971	0.318857	-0.35002	0.382949	-0.35455	0.162924	0.107079
ABLIM3	-0.54146	-0.1991	0.35754	0.010991	0.071019	0.218329	-0.46795	-0.17935
ABO	0.105645	0.176355	-0.01954	-0.15695	-0.12102	-0.26489	-0.11174	-0.03934
ABP1	0.203916	0.307195	0.244835	-0.13363	0.178457	-0.03803	0.227026	-0.01975

ABR	0.204129	-0.03848	-0.07064	0.039813	0.193709	0.252336	0.35524	0.155583
ABRA	-0.05299	-0.15443	0.015487	-0.19319	-0.05073	0.215088	0.283892	0.300785
ABT1	-0.27629	-0.15176	-0.34437	-0.02233	0.04083	-0.1096	0.027061	0.091256
ABTB1	-0.39594	0.472514	0.29203	0.009713	0.131867	0.066136	-0.45565	-0.21837
ABTB2	-0.08026	0.112981	-0.28597	-0.44181	-0.01655	-0.04149	0.485809	0.319401
ACAA1	-0.11315	-0.38599	0.03933	-0.12272	-0.06175	-0.14008	0.204345	0.151009
ACAA2	0.189243	-0.06882	-0.26546	-0.0261	-0.30185	-0.18016	-0.23068	-0.29291
ACACA	0.39971	0.09325	0.066245	0.174774	-0.20744	-0.1987	-0.17902	-0.23415
ACACB	0.267016	0.046553	0.188146	-0.14538	0.276708	-0.17471	-0.30193	-0.14825
ACAD10	-0.14392	0.084323	0.29732	-0.08537	0.400012	0.093796	-0.24311	-0.10289
ACAD11	-0.10216	-0.26041	-0.09456	0.150995	-0.12909	0.220411	-0.48167	0.221734
ACAD8	-0.20042	-0.47701	-0.04658	0.118831	0.132107	-0.16163	0.469755	0.544971
ACAD9	0.139742	0.28808	0.02116	0.275232	0.291907	0.203461	-0.37872	-0.2935
ACADL	-0.14213	-0.05994	0.126623	0.01365	-0.33283	0.011954	-0.04419	0.188089
ACADM	-0.33742	-0.59456	-0.1075	-0.14521	-0.21214	-0.19264	-0.01506	0.116121
ACADS	-0.34441	0.072551	0.162459	-0.29068	0.076901	-0.16099	0.031296	0.190738
ACADSB	0.00776	0.114181	-0.24313	-0.20369	0.265778	0.011125	0.204605	0.122271
ACADVL	-0.0843	0.307292	0.203866	0.235947	0.08191	0.172456	0.326342	0.529697
ACAN	0.204123	0.443949	-0.04104	-0.06717	-0.10878	0.00034	NA	NA
ACAP1	NA	NA	0.298233	0.603212	-0.07128	0.603818	NA	NA
ACAP2	NA	NA	-0.08026	-0.34751	-0.05367	-0.36915	NA	NA
ACAP3	NA	NA	0.059402	0.046123	0.226289	0.070822	NA	NA
ACAT1	-0.13671	-0.32267	-0.05436	-0.25406	-0.12173	-0.11782	-0.15122	-0.33727
ACAT2	0.018581	-0.32976	-0.2005	0.05193	-0.08555	-0.20791	0.040665	-0.12381
ACBD3	-0.34036	-0.41736	-0.23047	-0.06823	-0.14401	-0.10649	0.231888	0.117608
ACBD4	0.343211	0.175253	0.141699	-0.12096	-0.11401	0.082622	-0.33086	-0.11989
ACBD5	-0.28018	-0.41904	-0.25751	-0.17899	-0.37371	-0.18424	0.127407	0.317072
ACBD6	0.31793	0.116304	-0.25454	0.097091	-0.14236	0.149822	0.385364	0.054938
ACBD7	NA	NA	-0.05238	0.093087	-0.06481	-0.24645	0.315614	0.110378
ACCN1	0.288017	0.805687	0.463973	0.042517	0.124849	-0.21368	-0.10592	0.171197
ACCN2	0.321725	0.125187	0.126216	-0.09794	0.05932	-0.24155	-0.23952	-0.60365
ACCN3	0.051931	0.351491	-0.06111	0.11534	0.068146	-0.04059	0.185699	0.089698
ACCN4	-0.01659	0.267712	-0.05843	-0.16197	-0.1029	0.120805	-0.11362	0.095804
ACCN5	0.398056	0.69278	0.032754	0.084137	0.129068	-0.10399	0.158567	-0.39565
ACCS	NA	NA	0.218162	0.078846	0.305883	0.318052	NA	NA
ACD	0.509702	0.333001	-0.06688	0.343532	0.198875	0.066507	0.549299	0.146339
ACE	0.461518	0.614302	0.118606	0.034345	0.082227	0.055745	-0.33452	0.506538
ACE2	0.45691	0.191166	-0.06956	-0.1348	0.015845	-0.20733	0.162222	0.413517
ACER1	NA	NA	-0.09238	-0.24897	0.096366	-0.1426	NA	NA
ACER3	NA	NA	-0.34056	-0.31459	-0.17414	-0.25071	NA	NA
ACHE	0.104582	0.624039	0.240098	0.21862	-0.05175	-0.12146	0.039819	0.141
ACIN1	0.375201	0.307003	0.17588	0.187208	-0.01838	0.132303	0.25377	0.533458
ACLY	-0.15582	-0.75057	0.063779	0.324317	-0.06182	0.073858	0.193858	0.321013
ACMSD	0.235941	0.414402	0.401945	0.062681	0.122401	-0.42611	-0.12631	-0.18021
ACN9	-0.11623	-0.50459	-0.1664	-0.18589	-0.18741	-0.36639	-0.37626	-0.33179
ACO1	-0.46719	0.335548	-0.03697	-0.03292	0.08104	0.147137	-0.23067	0.338526
ACO2	-0.17415	-0.35356	0.199731	0.268558	0.158239	0.122344	0.022278	-0.1165
ACOT1	NA	NA	-0.34137	-0.14005	-0.10964	-0.05528	-0.31423	-0.13646
ACOT11	0.369911	0.232742	0.387805	-0.0167	0.405099	0.123734	0.317992	0.311797
ACOT12	-0.43211	0.443788	-0.03171	0.108067	0.208667	-0.33728	-0.05622	0.314827
ACOT2	-0.19213	-0.49196	-0.23236	-0.14275	-0.12928	-0.13398	-0.53329	-0.45989
ACOT4	0.332779	0.190858	0.117722	-0.02174	-0.15048	-0.17872	0.310044	0.280813

ACOT6	NA	NA	NA	NA	NA	NA	-0.14296	0.055252
ACOT7	-0.0676	0.367547	0.03377	-0.04253	0.248245	-0.07927	-0.03153	0.134117
ACOT8	0.56635	-0.31918	0.320015	-0.39651	0.280567	-0.41671	0.343916	-0.24392
ACOT9	-0.07105	-0.17213	0.040234	0.03373	-0.07048	-0.18253	-0.34837	-0.17491
ACOX1	-0.07235	-0.42493	0.185463	-0.35842	0.062169	-0.1286	0.229693	0.066569
ACOX2	0.41612	-0.3563	0.012339	-0.14546	0.087278	-0.31248	-0.16861	0.207187
ACOX3	-0.07027	0.33085	0.330973	0.011371	0.242774	-0.24015	-0.15776	-0.12922
ACOXL	0.167818	0.499132	-0.08309	-0.04376	-0.14507	0.122712	-0.03618	0.27991
ACP1	-0.04459	-0.6784	-0.05356	-0.09001	-0.09138	-0.38684	0.332035	0.001014
ACP2	-0.02852	0.343073	0.443926	0.225592	0.083093	-0.17058	0.078008	0.0869
ACP5	-0.32384	0.146627	0.282838	-0.14239	-0.18804	0.256966	-0.04016	0.183402
ACP6	0.260078	0.054327	-0.04274	0.172848	0.029249	-0.3275	0.259891	0.035503
ACPL2	-0.15607	-0.51291	-0.31587	0.046596	-0.07492	-0.17953	0.418691	0.255964
ACPP	0.034252	-0.28468	0.109654	0.070228	-0.24073	-0.58017	-0.12861	0.098508
ACPT	-0.07051	0.108694	0.034032	0.291448	0.170812	0.009893	0.329154	0.104946
ACR	0.273484	0.698419	0.006039	0.029008	-0.1363	-0.50698	-0.39011	-0.06059
ACRBP	-0.26975	-0.09833	0.079943	-0.25502	0.092091	-0.25159	0.057116	0.667021
ACRC	0.112753	-0.31832	-0.11761	0.396596	-0.19662	-0.2157	-0.53427	-0.37649
ACRV1	0.034021	0.46162	0.009647	0.241302	-0.33129	0.022319	-0.07874	-0.23066
ACSBG1	-0.09644	-0.20433	-0.03761	-0.15024	-0.00022	0.150427	-0.43909	-0.23331
ACSBG2	0.150032	-0.14478	-0.15924	-0.34887	0.265191	-0.19066	-0.16132	-0.01319
ACSF2	NA	NA	0.313459	-0.21893	0.262399	-0.0068	NA	NA
ACSF3	NA	NA	0.255572	0.275117	0.444681	0.079318	NA	NA
ACSL1	-0.49508	-0.47456	-0.05913	-0.13696	-0.0534	-0.09068	-0.54259	-0.11261
ACSL3	0.020228	-0.54267	-0.41134	-0.04182	-0.117	-0.24012	0.31006	0.215849
ACSL4	0.036258	-0.43787	0.124592	-0.0289	0.246226	0.335656	-0.47826	-0.12985
ACSL5	0.3206	0.157205	0.1691	-0.20037	0.330532	-0.26785	0.35704	0.399548
ACSL6	0.413161	0.320239	0.376674	0.415636	0.082834	0.136106	0.304983	0.206011
ACSM1	0.048029	0.146667	-0.08684	0.011823	0.010858	-0.1654	0.187453	-0.11849
ACSM2A	NA	NA	0.221453	-0.12794	0.12493	0.117255	NA	NA
ACSM3	-0.26542	-0.03512	-0.1683	-0.08082	-0.20288	-0.01645	0.113852	-0.05696
ACSM5	NA	NA	0.123722	-0.24872	-0.30699	-0.18997	NA	NA
ACSS1	-0.2103	-0.31571	0.08755	0.122546	0.359837	0.251242	0.471245	0.425438
ACSS2	0.125021	0.191959	0.100177	-0.04513	0.140033	-0.11419	0.12367	0.109639
ACSS3	NA	NA	-0.25049	-0.06184	-0.37968	0.329437	NA	NA
ACTA1	NA	NA	-0.23998	-0.14934	-0.11208	0.143702	-0.33528	0.001799
ACTA2	-0.15421	0.004701	0.26903	0.088285	0.0916	0.177351	0.026577	-0.18254
ACTB	-0.23777	-0.55014	0.133523	0.165322	0.130182	-0.01524	-0.27552	-0.10767
ACTBL2	NA	NA	0.422056	-0.03997	0.257742	0.015846	NA	NA
ACTC1	-0.38826	-0.10144	0.28425	0.014424	0.046164	-0.03796	NA	NA
ACTG1	-0.45497	-0.51138	0.279654	0.18992	0.028793	0.337199	0.749926	0.523947
ACTG2	-0.08495	0.041278	0.153736	0.085944	0.108293	-0.03019	-0.02779	-0.27362
ACTL6A	0.037427	-0.65836	-0.34347	-0.16292	0.176153	0.112394	0.477037	0.320313
ACTL6B	0.164981	0.559115	0.234705	-0.0611	-0.00818	-0.3706	-0.37757	0.086083
ACTL7A	0.335508	0.534851	0.336952	-0.0636	0.257463	0.166469	0.077887	0.133601
ACTL7B	0.322816	0.517098	0.125949	-0.16327	0.277682	0.166064	-0.20171	-0.3193
ACTL8	-0.04021	0.214391	-0.43272	-0.15533	-0.18416	-0.36891	-0.24127	0.061376
ACTN1	-0.32481	-0.14757	0.26308	0.050681	0.26761	0.537095	-0.11905	-0.21289
ACTN2	-0.21344	0.457296	-0.23119	0.304254	0.048928	0.10075	-0.69176	-0.55709
ACTN3	-0.12896	-0.33026	0.177514	-0.1447	0.310792	0.014873	0.178685	0.29301
ACTN4	0.107476	0.584835	0.108982	0.190214	0.184484	0.136366	0.565782	0.340139
ACTR10	-0.27189	-0.48035	-0.2524	-0.10176	-0.36743	-0.1834	-0.44466	-0.3532

ACTR1A	-0.27533	0.63967	0.221554	-0.20401	0.087813	-0.33734	-0.6742	-0.56744
ACTR1B	0.046427	0.239982	0.336397	0.300315	0.35433	-0.07747	0.219383	0.061904
ACTR2	-0.17812	-0.61844	-0.26818	0.120552	-0.09873	-0.24715	0.559527	0.361666
ACTR3	-0.08794	-0.54944	-0.07749	0.179914	-0.03405	-0.01674	-0.10845	-0.16873
ACTR3B	0.362611	0.581869	-0.13804	0.247997	0.09919	-0.24308	0.35806	-0.22516
ACTR5	0.422579	0.29795	0.231043	0.268235	0.259334	0.070487	0.120055	0.100244
ACTR6	-0.01505	-0.17312	-0.48503	-0.31677	-0.10402	-0.15258	-0.39191	-0.3761
ACTR8	0.359998	-0.56216	-0.28759	0.160811	0.045774	-0.33853	0.358249	0.067088
ACTRT1	0.299615	0.666934	-0.03347	-0.34116	0.102945	0.284143	-0.0409	-0.51591
ACTRT2	-0.34344	0.091879	0.092757	-0.1639	0.207864	-0.07436	NA	NA
ACVR1	0.036962	-0.4219	0.089869	-0.15084	0.005098	-0.13602	-0.33224	-0.23827
ACVR1B	0.049883	0.33743	0.012064	-0.04093	0.280702	0.044131	0.340604	0.039414
ACVR1C	0.172091	-0.15487	-0.2399	-0.17598	0.051319	-0.15219	-0.18285	-0.20987
ACVR2A	0.158199	-0.56225	0.005051	-0.28237	-0.06808	-0.45274	0.352484	0.205653
ACVR2B	0.360683	0.644048	0.048227	-0.16036	0.239411	-0.03864	-0.13425	-0.43669
ACVRL1	0.158403	0.551206	0.044075	-0.14105	-0.08887	0.164858	0.113932	0.205745
ACY1	0.188454	0.005877	0.055374	-0.12596	-0.00361	-0.29771	0.433013	0.477672
ACY3	0.201945	0.644737	0.369075	0.171535	0.165493	-0.32209	0.148647	0.458732
ACYP1	-0.04882	-0.41751	-0.25946	0.095348	-0.0356	0.048701	0.109546	0.054581
ACYP2	0.359785	0.740677	-0.26916	0.00889	-0.13955	-0.51978	0.112812	0.02581
ADA	0.341741	0.076555	0.409611	0.653376	0.066619	0.239726	0.078019	0.349159
ADAD1	NA	NA	-0.13874	0.259494	-0.10565	-0.28651	NA	NA
ADAD2	NA	NA	-0.12209	-0.28406	-0.06779	-0.03821	NA	NA
ADAL	0.005837	-0.50634	0.169242	-0.41491	-0.04811	-0.26189	-0.31861	-0.27649
ADAM10	-0.33033	0.094676	-0.33449	-0.22066	-0.48468	-0.48756	-0.00873	-0.14904
ADAM11	-0.03535	0.349819	-0.07435	-0.12773	-0.06476	-0.0941	0.044204	-0.21666
ADAM12	0.016671	0.171116	0.094508	0.628292	-0.07974	0.227583	-0.1499	-0.50822
ADAM15	-0.10149	0.646357	0.356118	0.273665	0.243489	-0.24771	-0.03478	-0.08468
ADAM17	-0.31471	0.062983	0.018438	-0.08977	0.158882	0.185292	-0.57809	-0.61115
ADAM18	0.113792	0.530744	0.077379	0.124616	0.199841	-0.04854	0.549079	-0.00273
ADAM19	-0.09285	0.096269	0.216426	0.478691	0.017181	0.697178	-0.00805	-0.16048
ADAM2	-0.26334	0.174742	0.31273	-0.07991	-0.06301	-0.14712	0.319896	0.045443
ADAM20	0.394517	0.432167	0.006976	-0.03844	0.156583	-0.31135	-0.1259	-0.0009
ADAM21	0.128623	0.012112	-0.07268	0.122887	-0.11161	-0.08142	0.08799	-0.15941
ADAM22	0.306247	0.549948	-0.25617	-0.04959	-0.21466	0.192129	-0.02146	0.124461
ADAM23	-0.17423	0.334781	0.222694	0.428772	-0.1714	0.277523	-0.08341	0.276082
ADAM28	-0.24712	-0.07163	0.175411	0.196673	-0.27409	0.517278	-0.53693	-0.00357
ADAM29	NA	NA	-0.07564	0.019574	-0.03523	0.082746	0.16129	0.257452
ADAM30	0.022012	0.153265	0.029943	0.337228	0.03198	-0.18267	0.104144	-0.04958
ADAM32	0.157428	0.103001	-0.12578	0.068658	-0.12551	-0.06593	0.000113	-0.24295
ADAM33	0.185964	0.258734	0.303166	-0.02299	0.027053	0.092845	-0.14397	0.04064
ADAM3A	NA	NA	NA	NA	NA	NA	0.593428	0.228043
ADAM6	NA	NA	-0.09706	-0.40984	0.202053	0.135045	0.638971	0.332579
ADAM7	-0.01677	0.298282	0.047168	-0.23647	-0.19153	0.038521	-0.34583	-0.31939
ADAM8	-0.56888	0.057468	0.019306	0.636839	-0.00865	0.64842	-0.11441	0.545363
ADAM9	-0.32091	0.404161	-0.2272	-0.06597	-0.11064	-0.16197	0.141494	0.047192
ADAMDEC1	-0.13379	-0.14246	-0.02785	0.003012	-0.24833	0.251602	-0.15209	0.18745
ADAMTS1	-0.32816	-0.45708	-0.13897	0.180028	-0.05386	0.036076	-0.48814	-0.33706
ADAMTS10	0.267499	-0.5565	0.160854	0.094932	0.136739	0.037735	0.144857	0.136599
ADAMTS12	0.052897	0.359765	0.29068	-0.33098	-0.07037	0.063791	0.070001	0.133389
ADAMTS13	0.156987	0.629201	-0.08458	-0.25074	0.125294	0.047766	0.184754	0.423396
ADAMTS14	0.040537	0.656757	-0.04205	-0.09115	-0.06756	0.203229	-0.27922	0.248235

ADAMTS15	-0.02627	0.40288	0.031152	-0.25158	0.185302	0.0779	0.292648	0.242139
ADAMTS16	0.065797	0.777978	0.254917	-0.2261	0.255075	0.188902	0.448856	0.44084
ADAMTS17	0.337932	0.676613	0.315156	-0.23298	0.008322	-0.00809	-0.24058	-0.13915
ADAMTS18	-0.15474	0.171766	0.068807	-0.28281	-0.03965	0.132926	-0.31803	0.053148
ADAMTS19	0.212423	0.31584	0.108719	-0.14607	-0.40653	0.232485	0.045238	0.185239
ADAMTS2	-0.06277	-0.26313	0.270875	-0.19412	-0.18208	0.081878	-0.18917	0.237559
ADAMTS20	0.106635	0.58373	-0.16839	-0.08166	0.142557	-0.22419	-0.01779	-0.25088
ADAMTS3	-0.06737	-0.07405	-0.04975	-0.23351	0.190431	0.283471	-0.48972	-0.22844
ADAMTS4	-0.37832	-0.53807	0.001063	0.112059	0.072766	0.207589	0.401251	0.331442
ADAMTS5	-0.35576	-0.42743	0.089821	-0.2167	0.040898	-0.10101	-0.1292	0.204914
ADAMTS6	-0.16409	-0.03736	-0.02337	-0.04497	-0.09413	0.207697	-0.11797	-0.06307
ADAMTS7	NA	NA	0.077706	0.005228	-0.28865	0.324872	-0.03909	0.271333
ADAMTS8	0.328597	0.053069	0.015633	-0.00146	0.295251	0.201403	-0.40559	-0.2625
ADAMTS9	-0.20031	-0.19127	-0.10564	0.23738	0.039609	0.230529	-0.28125	0.10416
ADAMTSL1	-0.23848	0.242234	-0.04173	-0.39211	-0.30631	0.206961	-0.30759	-0.17664
ADAMTSL2	0.094509	0.139465	0.273907	0.179202	0.042637	0.348113	0.201325	-0.043
ADAMTSL3	0.088394	-0.06536	0.20429	-0.11163	-0.00962	0.201098	-0.42286	-0.38814
ADAMTSL4	-0.197	0.185942	0.024997	-0.04638	-0.09687	0.40198	NA	NA
ADAMTSL5	0.003957	0.0451	0.381254	-0.1718	0.113712	-0.15367	0.430344	0.602321
ADAP1	NA	NA	0.348472	-0.09709	0.247622	-0.18598	NA	NA
ADAP2	NA	NA	0.235183	-0.03268	-0.39975	0.261293	NA	NA
ADAR	0.0277	0.346449	-0.02816	0.090752	0.080316	0.366865	-0.19242	0.100828
ADARB1	-0.22174	-0.24975	0.069365	0.222812	0.084063	0.524598	-0.0085	-0.17846
ADARB2	-0.06131	-0.13499	0.009656	0.20479	-0.1112	-0.09429	0.175599	-0.0011
ADAT1	-0.1912	-0.07459	0.114134	0.087162	0.319428	0.081681	0.208545	0.525722
ADAT2	NA	NA	-0.2439	-0.02196	0.075593	0.201271	NA	NA
ADAT3	NA	NA	0.259504	-0.06483	0.192517	-0.19165	NA	NA
ADC	0.043287	0.37542	0.032578	-0.30491	-0.16897	-0.01747	-0.16796	0.010415
ADCK1	0.515091	0.428864	0.005425	0.293088	0.335656	-0.02752	0.261558	0.424224
ADCK2	0.135589	0.2286	0.26248	0.112287	0.276532	0.003723	0.250897	-0.03502
ADCK4	-0.10737	-0.04034	0.1269	-0.31469	0.003426	-0.03177	0.289515	0.24383
ADCK5	0.205065	0.263374	0.4155	0.013305	0.264634	0.017422	0.175184	0.258728
ADCY1	0.058079	0.705941	-0.23093	0.21376	-0.04193	0.162633	0.052939	0.414074
ADCY10	NA	NA	-0.15382	0.242815	-0.01096	-0.3022	NA	NA
ADCY2	0.233276	0.205998	0.142602	-0.17978	-0.21177	-0.10622	-0.33636	-0.55015
ADCY3	0.253689	-0.11652	0.399413	-0.10585	0.402224	0.340081	-0.02246	0.470793
ADCY4	-0.33622	-0.02074	-0.09944	0.004544	-0.11175	-0.0632	-0.31473	0.272222
ADCY5	0.153182	-0.21106	0.045802	-0.12628	0.024855	0.201204	-0.16954	0.269524
ADCY6	-0.26536	-0.03018	-0.09837	0.034893	0.37003	0.189928	0.255559	0.3135
ADCY7	-0.31021	-0.3887	-0.36138	0.384595	-0.31164	0.350932	-0.40554	0.077868
ADCY8	0.223874	0.457008	0.068244	-0.03643	0.041139	0.073	0.230282	0.24091
ADCY9	-0.15977	-0.38296	-0.44197	-0.32669	0.356187	-0.12846	-0.34051	0.112926
ADCYAP1	0.208296	-0.15561	0.082652	0.138908	-0.07699	0.004495	-0.2598	-0.11271
ADCYAP1R1	0.513626	0.368796	-0.24205	-0.33438	0.120416	0.039968	-0.25956	0.018509
ADD1	0.040844	-0.47558	0.161268	-0.10123	0.043865	0.413051	-0.6179	-0.66401
ADD2	-0.3123	-0.08632	0.101837	0.187903	0.208701	-0.18934	-0.08032	0.168152
ADD3	0.096004	-0.16421	0.074298	-0.18537	0.095707	-0.22895	0.313867	0.064498
ADFP	-0.04095	-0.19117	-0.1464	-0.17476	0.054971	-0.04364	0.15032	0.551559
ADH1A	0.062107	0.029782	-0.11235	-0.07764	-0.14548	-0.04881	-0.02543	-0.12805
ADH1B	-0.34048	-0.01913	NA	NA	NA	NA	-0.05084	0.028566
ADH1C	0.073185	-0.02617	-0.13192	-0.22576	-0.15701	0.013234	0.023352	0.030407
ADH4	0.379784	0.085215	0.318586	-0.0954	0.007934	0.054044	-0.27964	-0.44522

ADH5	0.168018	-0.09585	0.155937	-0.45188	-0.05565	0.061335	-0.19952	-0.63418
ADH6	0.124323	0.086674	-0.089	0.097654	-0.11565	-0.09757	0.216788	0.356234
ADH7	NA	NA	-0.04284	-0.1655	0.224306	-0.24506	0.261808	0.425371
ADHFE1	-0.45114	0.019334	-0.0845	-0.06089	-0.26825	0.249983	-0.29973	0.026107
ADI1	-0.01519	0.484889	-0.44886	0.045595	-0.17235	-0.44478	-0.38724	-0.51611
ADIG	NA	NA	0.271025	-0.30816	0.028485	-0.17758	NA	NA
ADIPOQ	-0.01492	-0.11876	0.055534	-0.0962	0.043984	0.120388	0.313408	0.367606
ADIPOR1	-0.35313	-0.57513	0.164401	0.002428	-0.1857	-0.14894	0.544082	0.534146
ADIPOR2	0.262633	0.461597	-0.10836	-0.20015	-0.16388	-0.15895	0.34018	0.58245
ADK	0.00137	0.015267	-0.05699	-0.13493	0.206641	-0.49583	-0.00068	-0.2754
ADM	-0.59141	-0.19195	-0.20293	-0.08807	-0.02464	-0.30637	-0.26964	0.099823
ADM2	-0.2048	0.683172	0.148609	0.064016	0.218327	-0.13229	-0.24293	-0.04199
ADNP	0.322056	-0.66278	-0.13242	-0.42181	0.139521	0.138555	0.205908	-0.25701
ADNP2	NA	NA	-0.42929	-0.12722	-0.17057	0.040282	NA	NA
ADO	NA	NA	-0.24393	-0.24322	0.146091	-0.2543	NA	NA
ADORA1	0.161147	0.301454	0.368951	0.126953	0.10067	-0.08437	-0.41027	-0.40652
ADORA2A	-0.45788	0.549679	0.298622	0.64142	0.129746	0.266994	-0.13474	0.362691
ADORA2B	-0.05727	0.184072	-0.19922	-0.12018	-0.19638	0.242777	0.160578	-0.00344
ADORA3	-0.03902	0.10637	0.103715	-0.42734	-0.29109	-0.18886	-0.24704	-0.40677
ADPGK	-0.35018	-0.30878	-0.18284	0.160704	0.144164	0.283599	0.281243	0.617134
ADPRH	-0.0705	-0.33307	0.085485	0.046366	-0.07212	0.099025	-0.40195	-0.25753
ADPRHL1	0.237206	0.399144	-0.07116	-0.15154	0.008899	-0.53639	0.245407	0.281539
ADPRHL2	-0.53978	-0.04102	0.256918	0.190825	0.081932	0.122787	-0.07489	-0.20916
ADRA1A	0.165697	0.591486	0.014387	-0.2133	0.483374	0.046414	0.246005	-0.03749
ADRA1B	-0.21342	-0.25388	0.279956	0.444135	-0.23933	0.327468	0.505044	0.477967
ADRA1D	0.248369	0.575795	-0.01254	-0.36449	0.21387	-0.24958	-0.43756	-0.09264
ADRA2A	0.258863	0.477915	0.210867	0.267197	0.00699	0.040794	-0.16278	0.066358
ADRA2B	0.257159	0.734144	-0.27099	0.023825	-0.17981	0.012125	-0.39237	-0.52464
ADRA2C	0.424296	0.670947	-0.055	0.071486	0.013627	-0.12691	0.592597	0.421463
ADRB1	0.130236	0.726787	-0.18654	0.187633	0.081613	-0.12716	-0.35348	-0.17582
ADRB2	-0.05251	0.31457	-0.03907	0.154821	-0.31537	0.125246	-0.47297	0.387735
ADRB3	-0.08526	0.051749	-0.11754	-0.28314	0.240722	-0.05745	-0.58915	-0.31454
ADRBK1	0.163178	0.408823	0.030191	0.279469	0.308435	-0.06858	0.300209	-0.20578
ADRBK2	0.103119	0.068382	0.422194	0.419463	0.042839	0.46242	-0.33404	-0.03671
ADRM1	0.356219	0.133549	0.182498	0.00879	0.190017	-0.03264	0.171587	-0.14699
ADSL	0.217265	0.010265	0.191267	0.302896	0.102904	-0.19958	0.616289	0.031528
ADSS	-0.25946	-0.43822	-0.31732	0.185571	-0.16355	0.027808	0.483118	0.140097
ADSSL1	-0.33117	-0.18446	-0.09873	0.000579	-0.29208	-0.27251	0.182977	0.56072
AEBP1	-0.18549	-0.07712	0.185171	0.191702	0.013412	0.412236	-0.13906	-0.28911
AEBP2	0.548405	-0.08958	-0.32231	-0.42738	-0.0069	0.518413	-0.12804	0.02026
AEN	NA	NA	-0.09907	0.178652	0.292028	0.049293	NA	NA
AES	0.328499	0.235551	0.101538	0.354197	-0.00544	-0.06835	0.187497	-0.32391
AFAP1	-0.32134	0.318393	-0.0198	0.058633	-0.03474	-0.06861	NA	NA
AFAP1L1	-0.37568	-0.30916	-0.04768	0.022174	-0.04932	0.248483	NA	NA
AFAP1L2	-0.26598	0.072986	-0.22867	0.140332	-0.09493	0.151281	0.084737	-0.18903
AFF1	0.413599	0.692933	-0.15595	-0.24021	-0.23172	-0.25836	-0.2481	-0.35055
AFF2	-0.04914	0.126419	0.157036	0.362418	0.232212	-0.06931	0.077371	0.044444
AFF3	-0.10456	-0.08332	-0.10681	0.15455	-0.24624	0.242191	-0.49621	-0.48395
AFF4	-0.17713	-0.56272	0.039406	-0.31751	-0.08104	0.202384	-0.28864	-0.22628
AFG3L1	-0.13238	-0.42519	-0.00034	0.18427	0.217927	0.472504	0.450059	0.662734
AFG3L2	-0.0126	0.296554	0.001061	0.173148	0.101121	0.066408	0.058581	0.208152
AFM	0.230003	-0.0191	0.09079	-0.23415	-0.12353	0.056036	0.34052	-0.12682

AFMID	-0.06943	0.129526	0.194134	0.14506	-0.03303	-0.06603	0.319321	-0.03974
AFP	0.343589	0.041818	-0.03326	0.134736	0.068477	-0.23307	-0.06978	0.026413
AFTPH	-0.07681	-0.20063	-0.19496	-0.28333	-0.08528	0.030087	0.136287	-0.34821
AGA	0.226755	0.459146	-0.12388	-0.25469	-0.11587	-0.42786	-0.25201	-0.30162
AGAP1	NA	NA	-0.08228	0.041056	0.383238	0.161956	NA	NA
AGAP2	NA	NA	-0.15322	0.562624	-0.31995	0.168218	NA	NA
AGAP3	NA	NA	-0.00467	0.133508	0.09864	0.260583	NA	NA
AGBL1	NA	NA	-0.04896	-0.14341	-0.12104	-0.20228	-0.30465	-0.12289
AGBL2	0.520924	0.150023	-0.08008	0.024515	0.112745	-0.10111	0.125911	0.405806
AGBL3	0.282944	0.096927	0.020019	-0.03554	-0.11295	0.010327	NA	NA
AGBL4	0.21155	0.734211	-0.29885	-0.04083	0.089277	-0.05338	-0.26532	-0.07301
AGBL5	NA	NA	0.270697	-0.04555	0.652471	-0.13022	NA	NA
AGER	0.449227	0.341667	0.097817	0.413709	-0.04881	0.349036	0.092576	0.353563
AGFG1	NA	NA	-0.01392	-0.02629	0.076147	-0.43361	NA	NA
AGFG2	NA	NA	0.049614	0.039547	0.346274	-0.36505	NA	NA
AGGF1	0.344184	-0.59676	0.280538	-0.16357	-0.26869	-0.13018	0.35045	0.196451
AGK	0.483955	-0.23072	-0.1961	-0.01938	0.162184	0.125886	NA	NA
AGL	-0.20447	-0.43547	0.122594	-0.36047	0.0841	-0.04375	-0.37143	-0.33927
AGMAT	0.458363	0.501075	0.157156	0.178773	0.197192	-0.2126	0.417276	0.26244
AGPAT1	-0.22475	0.310647	-0.00272	0.164426	-0.02806	-0.07728	0.222001	0.34024
AGPAT2	0.093482	-0.09647	0.258412	0.028365	0.375613	-0.01894	0.1725	0.318759
AGPAT3	0.346492	-0.11968	-0.00057	0.068866	-0.06929	-0.47912	0.318459	0.462971
AGPAT4	-0.35809	-0.18635	-0.14935	0.137847	-0.37205	0.032036	-0.25291	-0.04467
AGPAT5	0.157567	0.167252	-0.15288	0.055458	-0.21064	-0.04457	0.369082	0.203509
AGPAT6	-0.34275	-0.14197	0.166644	0.282597	0.272858	0.195373	-0.39722	-0.46239
AGPAT9	NA	NA	-0.0882	-0.0491	-0.03668	0.032553	NA	NA
AGPS	0.482615	0.821852	-0.18067	-0.38095	0.022565	-0.42088	0.479136	0.243207
AGR2	-0.39933	0.1243	-0.33116	0.129544	-0.23808	-0.23423	-0.0495	-0.14983
AGR3	-0.35751	-0.10277	-0.25564	-0.12153	-0.30643	-0.47621	-0.1442	-0.34116
AGRN	NA	NA	0.210809	0.258238	0.06838	0.217272	-0.0211	0.08748
AGRP	0.00532	-0.12014	0.021781	0.041774	-0.18427	-0.03429	-0.28425	0.256056
AGT	0.227143	0.381511	0.001845	-0.03423	0.114877	-0.06228	0.306521	0.35399
AGTPBP1	-0.1902	-0.42694	-0.34274	-0.39139	0.051846	0.049822	-0.43398	-0.15415
AGTR1	0.10699	-0.34803	-0.03445	-0.33408	-0.00471	-0.22734	-0.18145	-0.21621
AGTR2	0.163347	0.336799	0.007908	-0.32512	-0.08777	-0.04631	0.547985	0.260072
AGTRAP	-0.03718	0.268584	0.155287	0.152073	0.028661	-0.21438	-0.14165	-0.30125
AGXT	0.114843	0.339552	-0.24401	-0.14788	0.124149	-0.34515	0.222686	-0.22481
AGXT2	-0.29791	0.13965	-0.06067	-0.02755	0.137854	-0.21977	0.186398	0.410335
AGXT2L1	-0.02778	-0.5451	0.023908	0.072251	0.083916	-0.12472	-0.06504	-0.57357
AGXT2L2	-0.29934	-0.02744	0.288021	0.085165	-0.00876	0.156984	NA	NA
AHCTF1	-0.12853	-0.27963	-0.2146	-0.32957	-0.03667	0.366932	0.261708	0.189154
AHCY	0.52849	-0.66683	0.276586	0.3564	0.476831	0.063793	0.636902	0.435625
AHCYL1	-0.45096	-0.48071	0.092601	0.188117	0.307368	0.100539	0.136803	0.242793
AHCYL2	NA	NA	-0.14061	-0.21114	-0.09752	-0.29471	NA	NA
AHDC1	-0.16064	0.051148	-0.18934	-0.13981	-0.08798	0.116869	-0.22334	-0.13389
AHI1	0.109312	0.141329	-0.29398	-0.20325	0.167256	0.091891	-0.05956	0.177677
AHNAK	-0.5676	-0.25684	-0.11123	-0.41428	-0.24005	0.043203	-0.40686	-0.31484
AHNAK2	NA	NA	0.054114	-0.15795	-0.03841	0.1713	NA	NA
AHR	-0.18186	0.481859	-0.15593	-0.14636	-0.2649	0.325218	0.145366	0.295422
AHRR	0.00618	0.029337	-0.21643	-0.24438	0.186107	0.091686	-0.03784	-0.06753
AHSA1	0.059679	-0.46578	-0.12293	0.317228	-0.09215	0.056299	-0.048	-0.17044
AHSA2	0.070903	-0.40164	0.030812	0.282044	0.056905	0.229854	-0.49908	-0.06296

AHSG	0.206203	0.408317	-0.22418	-0.06049	-0.37112	-0.33148	0.100091	-0.26411
AICDA	0.010091	0.643276	-0.09367	0.723494	-0.05029	0.316529	-0.1045	-0.24677
AIDA	NA	NA	-0.24286	0.019478	-0.24755	-0.42542	NA	NA
AIF1	-0.6215	-0.25447	0.250902	-0.10585	-0.2824	-0.00114	-0.59153	0.159794
AIF1L	NA	NA	-0.3493	-0.0291	-0.29346	-0.09632	NA	NA
AIFM1	0.255956	0.081638	-0.31532	0.071919	0.053813	-0.28434	0.10556	0.157434
AIFM2	0.090119	0.012909	-0.08241	-0.21619	0.414188	-0.10196	NA	NA
AIFM3	0.131228	0.199057	-0.03405	0.071428	0.378375	0.277506	NA	NA
AIG1	0.431912	0.38856	-0.0295	-0.32687	0.184634	-0.57573	0.330909	0.241771
AIM1	0.230022	-0.08151	0.290457	-0.093	0.326607	-0.14081	-0.18307	-0.27955
AIM1L	0.142287	0.499933	0.264671	-0.27485	0.299073	0.013951	0.239996	-0.26076
AIM2	0.084672	0.118387	-0.1361	0.595693	-0.62114	0.494397	-0.03036	0.088735
AIP	0.135252	-0.22113	0.326764	0.325777	0.154633	0.186008	0.39715	0.25927
AIPL1	0.016807	0.770286	-0.26654	-0.32281	-0.0044	-0.22656	0.633627	0.423655
AIRE	0.104102	0.377208	0.379378	0.643423	0.096091	0.335408	-0.53005	-0.47579
AJAP1	-0.05418	-0.16656	0.109242	-0.49386	-0.07808	0.079564	-0.16913	-0.084
AK1	-0.37187	-0.13686	0.127358	0.007867	-0.03281	-0.07073	-0.21028	-0.32028
AK2	0.144401	0.141505	0.088941	-0.03862	-0.22068	-0.42857	-0.2233	-0.02337
AK3	0.354271	0.642649	-0.32526	-0.14808	-0.22815	-0.42842	-0.06841	-0.03928
AK3L1	0.403836	0.186873	0.152142	-0.23401	0.458641	-0.19572	0.044797	-0.27155
AK5	0.247153	-0.04792	-0.18622	-0.28881	-0.3006	-0.31919	-0.06808	-0.15916
AK7	0.099274	0.202456	-0.23965	-0.33602	-0.03488	-0.22311	-0.53605	-0.29212
AKAP1	0.089144	-0.42676	-0.09091	0.076422	0.27871	0.002074	0.096605	-0.27891
AKAP10	-0.08979	-0.48717	-0.07091	0.053014	-0.17347	-0.23082	0.128425	0.159255
AKAP11	0.094067	-0.64179	0.134309	-0.1755	0.415118	0.405929	0.434225	0.099512
AKAP12	-0.02685	-0.35811	-0.12479	0.028547	-0.09362	0.302922	-0.4741	-0.29998
AKAP13	-0.19374	-0.18679	0.045683	0.002731	0.029146	0.413116	-0.41853	-0.09402
AKAP14	0.221842	0.466491	-0.28668	-0.27182	0.18511	-0.27401	-0.00717	0.492262
AKAP2	0.121626	0.461311	0.118351	0.173474	0.173723	0.085007	0.37697	0.137576
AKAP3	-0.33843	0.503508	-0.00119	0.21768	-0.41059	-0.23702	0.499156	0.290351
AKAP4	0.027919	0.623408	-0.10909	-0.26697	0.025624	-0.20033	0.138773	0.153338
AKAP5	0.150522	0.36413	-0.20218	-0.07064	-0.22903	-0.29317	-0.39766	-0.05689
AKAP6	-0.02442	-0.11689	0.129925	-0.0976	0.192933	-0.09269	-0.36564	-0.09776
AKAP7	0.414211	-0.22527	-0.15025	0.051543	-0.15797	0.127028	-0.04821	0.085884
AKAP8	0.377558	-0.16284	-0.32181	0.199316	0.19756	0.300536	0.475957	0.611605
AKAP8L	0.057448	-0.22321	0.072091	0.061485	0.342058	0.384259	-0.30462	0.21501
AKAP9	-0.03367	0.097436	-0.25926	0.050497	0.022389	-0.17714	-0.20655	-0.47763
AKIRIN1	NA	NA	0.185885	-0.19742	0.07117	-0.15154	NA	NA
AKIRIN2	NA	NA	-0.01968	0.014723	0.0376	0.110791	NA	NA
AKNA	-0.00668	0.209465	0.211895	0.382692	0.185091	0.482798	-0.52696	0.031758
AKR1A1	-0.22245	-0.11827	0.114061	0.082595	0.001039	-0.17673	-0.17996	0.122895
AKR1B1	-0.06928	-0.28554	0.051052	-0.25574	-0.11034	-0.18574	-0.35413	0.075847
AKR1B10	-0.05813	0.036105	-0.14496	-0.26355	-0.0618	-0.26339	0.119303	0.238129
AKR1C1	-0.04124	0.177978	-0.02007	-0.40241	0.087616	-0.48961	0.043858	0.235782
AKR1C2	NA	NA	NA	NA	NA	NA	0.012661	0.116318
AKR1C3	0.178776	0.226362	0.345259	-0.40057	0.037223	-0.28787	0.01457	0.144635
AKR1C4	0.288602	0.231835	0.200328	-0.21064	0.176922	-0.16183	0.341627	0.572807
AKR1CL1	0.346148	0.053041	NA	NA	NA	NA	0.436555	0.544463
AKR1CL2	0.178036	0.473326	-0.08531	-0.0211	-0.07493	-0.10996	0.300129	0.016338
AKR1D1	-0.22033	0.079883	-0.08839	-0.28698	0.009254	0.124861	-0.26413	-0.32323
AKR7A2	-0.30343	0.609807	-0.07532	-0.07873	0.052004	-0.22247	-0.04146	-0.22034
AKR7A3	-0.32127	9.7E-05	0.023237	-0.15086	0.190436	-0.23994	0.244541	0.219584

AKR7L	NA	NA	0.065144	-0.25145	0.068422	-0.15016	NA	NA
AKT1	-0.0799	0.280323	0.238622	0.174193	0.271131	-0.12178	0.104632	0.088566
AKT1S1	-0.08491	0.55199	0.044814	0.240779	0.085307	0.250203	-0.07494	0.134231
AKT2	0.263563	0.008264	0.20673	0.181986	0.020346	0.145176	0.382784	0.056027
AKT3	-0.03475	-0.10012	-0.07143	-0.07935	-0.21887	0.289433	-0.57121	-0.21362
AKTIP	-0.07524	-0.53165	0.01986	-0.22365	0.1707	-0.4833	0.172844	0.077449
ALAD	-0.02083	-0.04591	-0.06234	-0.02613	-0.06419	-0.30131	0.07607	-0.14958
ALAS1	-0.03287	0.187925	0.039298	0.046455	0.151278	0.092189	0.165411	0.076178
ALAS2	0.265299	0.64143	-0.04617	0.12397	0.038976	-0.09357	0.416067	-0.0753
ALB	0.283968	0.161772	0.003908	-0.15614	0.147151	0.387586	0.431051	0.123373
ALCAM	-0.07393	-0.27394	0.001392	-0.15939	-0.163	-0.12732	-0.1013	-0.40135
ALDH16A1	-0.04822	0.526658	0.017753	0.30477	0.051859	0.247052	0.205583	0.50119
ALDH18A1	NA	NA	0.025054	0.035679	0.229339	0.020104	0.091145	0.1158
ALDH1A1	-0.35562	-0.5318	-0.02112	-0.04155	-0.35385	-0.19701	-0.01552	-0.06682
ALDH1A2	-0.64511	-0.17948	-0.21268	0.320776	-0.08802	-0.17669	-0.11	-0.09518
ALDH1A3	0.020245	-0.49421	0.140585	0.149073	0.057014	0.002061	-0.36629	0.097885
ALDH1B1	0.123335	-0.26694	-0.0276	0.147804	0.045899	0.129223	-0.44135	-0.26616
ALDH1L1	-0.15772	0.218682	0.022668	0.031556	-0.42871	-0.21806	-0.08081	0.043096
ALDH1L2	NA	NA	-0.20241	-0.13142	-0.11066	0.039907	-0.38292	-0.38401
ALDH2	-0.2946	-0.13593	0.232659	0.128589	0.345745	-0.11137	0.441407	0.144823
ALDH3A1	-0.05618	0.640969	-0.17887	0.180618	0.01464	-0.05963	0.073322	-0.08857
ALDH3A2	-0.08193	-0.36466	-0.07125	-0.25695	-0.04397	-0.05125	-0.02413	-0.10275
ALDH3B1	0.073082	0.573277	0.3295	0.165341	0.089792	-0.27595	-0.12269	0.355495
ALDH3B2	0.009811	0.329227	0.266734	-0.06174	-0.0065	0.172462	0.081377	0.31963
ALDH4A1	0.301132	-0.02532	0.404315	0.011278	0.497336	-0.07639	0.201613	-0.28994
ALDH5A1	0.026922	-0.46339	-0.01662	0.289097	0.073961	0.560723	0.55812	0.251843
ALDH6A1	0.031257	-0.20193	-0.10272	0.110452	0.008655	-0.00363	-0.22343	-0.47529
ALDH7A1	0.193691	0.656127	0.286813	-0.20221	-0.04226	-0.04434	-0.03397	-0.2624
ALDH8A1	0.204108	0.458433	0.183577	0.066147	0.256396	-0.07789	-0.55856	-0.52907
ALDH9A1	-0.37844	0.065943	-0.2485	0.196667	-0.1199	-0.2758	-0.06589	0.033902
ALDOA	-0.39035	-0.62534	0.043633	0.003906	-0.0856	0.264459	0.364388	0.171485
ALDOB	-0.02356	0.335152	0.217819	0.146616	0.337488	-0.04698	0.044069	0.474931
ALDOC	-0.03519	-0.37172	0.121141	-0.47217	0.05622	-0.42874	0.268504	-0.01943
ALG1	0.04883	-0.1428	-0.43379	0.186592	0.073204	-0.17682	0.432453	0.044777
ALG10	0.130556	-0.18758	-0.20998	0.085724	-0.19404	0.009318	0.009218	0.304005
ALG10B	-0.00156	-0.00273	-0.23791	-0.12591	-0.14567	0.06508	NA	NA
ALG11	0.003247	-0.36003	-0.14805	-0.14454	-0.12128	-0.13131	-0.47039	-0.3156
ALG12	0.274259	0.750544	0.163349	0.115455	0.06847	-0.06844	0.531845	0.181578
ALG13	0.191341	-0.00959	-0.24315	-0.17952	-0.31624	-0.2063	-0.14471	-0.13731
ALG14	-0.10586	-0.41931	-0.00531	-0.15915	-0.06605	-0.52365	0.118838	0.001694
ALG2	-0.08952	-0.25871	-0.0787	0.165694	-0.17302	-0.19615	0.40549	0.324822
ALG3	0.373306	0.621293	0.113462	0.205685	0.096509	-0.18385	0.028894	-0.17039
ALG5	0.266233	0.062065	0.026161	0.136322	0.217959	-0.24559	-0.1507	-0.3144
ALG6	-0.27481	-0.66462	0.121872	-0.18729	0.130922	-0.05506	0.336814	0.389495
ALG8	0.193702	0.005774	-0.15905	-0.07074	-0.1537	-0.21566	0.425887	0.068461
ALG9	-0.16788	0.053502	-0.0438	-0.17316	-0.13873	-0.41942	-0.09909	0.17939
ALK	0.213825	0.311026	0.04671	-0.06308	-0.19981	0.12062	-0.12218	-0.12701
ALKBH1	-0.08827	0.015227	-0.33436	-0.06229	-0.16536	-0.35927	0.297308	0.465752
ALKBH2	0.061653	0.251995	0.01372	0.230795	0.159663	0.29712	0.407941	0.174047
ALKBH3	0.230749	0.147003	0.14602	-0.16887	-0.0433	-0.22746	0.459833	0.568198
ALKBH4	0.26322	0.603744	0.26228	0.047319	0.316363	0.239308	0.361801	0.091333
ALKBH5	-0.41337	-0.13799	0.147396	0.280916	-0.04358	-0.06173	-0.07925	0.089827

ALKBH6	0.284536	0.39363	-0.00455	0.179636	-0.0649	-0.01757	-0.14407	-0.15668
ALKBH7	0.327601	0.704814	0.368408	0.000491	0.0842	-0.23215	0.335381	0.011211
ALKBH8	0.049941	0.066705	-0.23223	-0.21913	-0.25735	0.069987	-0.1912	-0.20172
ALLC	0.087995	0.621029	-0.16874	0.111478	-0.11793	0.197418	0.164088	0.2893
ALMS1	0.056018	0.129894	-0.17579	-0.17257	0.158902	0.224568	0.406621	0.293943
ALOX12	0.08487	0.39963	-0.0878	-0.16512	-0.17554	0.035927	0.118068	0.228834
ALOX12B	-0.06843	0.421026	0.064564	-0.02914	0.009152	0.119263	0.239519	0.320435
ALOX12P2	NA	NA	-0.07568	0.182093	-0.36754	0.014573	0.038374	0.048773
ALOX15	-0.16562	-0.52133	-1.6E-05	-0.26036	-0.00251	0.041951	0.100352	-0.16154
ALOX15B	0.292552	0.43764	0.096758	-0.13205	-0.23092	-0.08659	-0.14995	-0.18992
ALOX5	-0.4019	0.095674	0.008633	0.655981	-0.25039	0.536396	-0.17976	0.534405
ALOX5AP	-0.55951	0.007546	0.371851	0.062876	-0.04358	0.345044	-0.41338	0.08099
ALOXE3	-0.41324	-0.0873	0.180712	-0.04798	-0.12956	-0.28979	0.169415	0.266419
ALPI	-0.28382	-0.36576	0.203579	-0.35682	-0.18874	-0.22076	-0.22348	0.433732
ALPK1	NA	NA	-0.00903	-0.05969	0.040201	0.073825	-0.29149	0.204326
ALPK2	NA	NA	0.035033	0.691643	-0.18119	0.368165	NA	NA
ALPK3	-0.00178	-0.06659	-0.22743	-0.13825	-0.01019	0.01207	0.011611	0.029677
ALPL	-0.51211	-0.01396	0.301517	0.057523	-0.00112	-0.01092	-0.23368	0.122481
ALPP	-0.10985	0.172803	0.095191	-0.28011	-0.27301	-0.30079	-0.54515	-0.49185
ALPPL2	-0.03625	0.670329	-0.34442	-0.2211	-0.08084	-0.1649	-0.23016	-0.35454
ALS2	-0.30533	-0.56781	-0.14699	-0.33176	-0.15012	-0.12387	-0.00337	0.202253
ALS2CL	0.08609	0.613064	0.028585	0.163208	0.326734	0.196112	0.399731	0.256639
ALS2CR11	0.236942	0.194479	-0.0694	-0.41349	-0.20697	0.161318	0.50871	0.182489
ALS2CR12	0.106565	-0.31963	0.312753	0.107942	-0.00102	-0.34966	-0.2522	-0.27771
ALS2CR4	-0.17795	-0.38808	-0.23557	-0.14476	-0.01039	-0.30766	0.564424	0.221512
ALS2CR8	0.401606	-0.10152	0.258922	-0.18288	-0.10209	-0.06793	0.002488	0.190829
ALX1	NA	NA	-0.2539	-0.09226	-0.16842	-0.00158	NA	NA
ALX3	0.230774	0.734854	-0.0188	-0.20089	0.178547	-0.07494	0.013762	-0.08531
ALX4	0.470931	0.089127	0.304125	-0.1375	0.142587	-0.03573	-0.26186	-0.19065
AMAC1	-0.02112	-0.20955	0.069187	-0.19131	-0.05542	0.281801	-0.42063	-0.29612
AMAC1L2	-0.29429	-0.32782	0.078979	0.173327	-0.22681	0.266445	-0.10555	-0.26608
AMACR	0.288647	-0.29159	0.135747	-0.07622	0.168748	-0.2148	0.536433	-0.05316
AMBN	0.305485	0.172242	0.095259	-0.12864	-0.10477	-0.23101	0.36759	-0.33438
AMBP	0.164377	-0.10459	-0.26884	-0.34298	-0.13707	-0.52696	0.036947	-0.42607
AMBRA1	NA	NA	0.125186	0.235569	0.29867	0.456591	NA	NA
AMD1	0.041153	-0.39916	-0.40038	-0.08562	-0.00239	0.083493	0.372644	0.259735
AMDHD1	0.258215	-0.24921	0.159847	-0.22426	-0.13108	0.246205	0.085973	0.208877
AMDHD2	0.053305	-0.2283	0.295326	-0.23307	0.100452	-0.04216	0.243496	0.403184
AMELX	0.272646	0.287913	0.038526	0.251612	0.010543	0.075824	0.49282	0.200275
AMELY	-0.13373	0.259299	-0.09052	-0.22484	0.006607	0.033397	0.505747	0.168082
AMFR	-0.51485	-0.49807	0.300078	0.094461	0.396777	-0.23488	0.397424	0.349276
AMH	-0.35994	0.701983	-0.18004	-0.05483	-0.22895	-0.45095	0.072922	0.010379
AMHR2	-0.15668	0.763348	0.154956	0.028413	0.127731	-0.01305	-0.29978	0.122236
AMICA1	-0.44562	0.177618	0.137509	-0.07246	-0.20243	0.04829	-0.41276	0.589085
AMIGO1	-0.04474	0.114134	0.307818	-0.19253	0.436401	-0.04141	-0.31567	-0.3346
AMIGO2	-0.07246	0.464663	0.130123	-0.09544	-0.33426	0.016829	-0.2519	-0.42301
AMIGO3	0.005645	0.201682	-0.10977	0.220903	0.227081	-0.01057	0.133235	-0.57453
AMMECR1	0.356028	0.028652	-0.346	-0.01057	-0.05663	-0.18675	0.159432	0.102601
AMMECR1l	0.331641	0.070631	0.090265	-0.15513	0.426266	0.123192	NA	NA
AMN	-0.23745	-0.22526	0.013319	0.078072	-0.00312	-0.08781	0.082238	0.115482
AMN1	0.243629	0.710067	-0.23835	-0.15408	-0.48711	-0.37922	NA	NA
AMOT	0.399771	-0.22822	0.077626	-0.1507	-0.04072	0.02636	0.373237	0.083073

AMOTL1	-0.25962	0.022124	0.131877	-0.16247	-0.13877	0.113032	NA	NA
AMOTL2	0.28046	0.617441	-0.11035	-0.0402	0.292002	0.026969	-0.5357	0.015025
AMPD1	0.333368	0.496832	0.05901	-0.3339	-0.15073	-0.25749	-0.13659	-0.05929
AMPD2	0.421848	0.372342	0.529756	0.199069	0.458035	0.60741	-0.21803	-0.33282
AMPD3	0.106723	0.640667	-0.28757	0.519798	-0.07582	0.304176	-0.46769	-0.01221
AMPH	-0.03623	-0.17114	-0.0525	0.116485	-0.29069	-0.15084	-0.06305	0.107857
AMT	0.029613	0.402786	-0.0099	0.149886	0.09676	0.049546	-0.19382	-0.18518
AMTN	-0.11629	0.148015	NA	NA	NA	NA	NA	NA
AMY1A	-0.02721	0.554897	NA	NA	NA	NA	-0.53532	-0.48345
AMY1C	-0.19011	-0.40823	0.035566	-0.08188	0.197318	0.173803	NA	NA
AMY2A	0.024948	-0.17214	NA	NA	NA	NA	0.625953	-0.15879
AMY2B	0.00867	-0.27579	-0.12058	0.123476	0.262223	-0.06657	-0.27512	0.038221
AMZ1	-0.36771	0.592228	0.064257	0.238028	-0.06486	-0.0929	-0.25891	-0.18907
AMZ2	0.15875	-0.44441	0.237994	0.310928	-0.01424	-0.22014	0.307562	-0.02755
ANAPC1	0.524069	-0.40553	-0.06162	0.138681	0.141753	0.261106	0.532039	0.178592
ANAPC10	-0.09309	-0.62501	-0.24352	-0.2238	-0.27074	-0.27659	0.18997	-0.00321
ANAPC11	0.193121	-0.05989	0.070684	0.071706	0.17706	-0.25979	0.12018	-0.26426
ANAPC13	-0.05825	-0.26139	-0.18451	0.023982	-0.28445	-0.20257	-0.16834	-0.11181
ANAPC2	0.144198	0.751036	0.197186	0.187146	0.217525	0.009558	0.332173	0.373351
ANAPC4	-0.10404	-0.10427	-0.27968	-0.0567	0.082457	0.120494	0.022013	0.167947
ANAPC5	-0.11258	-0.48441	-0.21807	0.344766	0.250875	0.047527	0.205685	0.514759
ANAPC7	-0.06845	-0.39169	-0.25455	0.108046	0.206695	0.026264	0.299652	0.222611
ANG	-0.27908	0.103007	-0.23799	0.062302	-0.27015	-0.29294	0.034756	0.095044
ANGEL1	0.421555	0.474913	-0.05275	-0.04825	0.065952	0.061051	0.442592	0.233144
ANGEL2	0.151692	-0.00242	-0.1575	-0.09557	-0.0418	0.046397	0.245719	0.25548
ANGPT1	-0.18529	-0.42248	0.099449	-0.19118	-0.35286	0.255698	0.111092	-0.08911
ANGPT2	-0.02062	-0.24133	-0.15475	0.186768	-0.04661	0.152223	-0.08211	0.183987
ANGPT4	0.103992	0.193986	0.01802	0.051154	-0.02701	-0.28222	-0.01712	0.197612
ANGPTL1	0.425037	-0.27115	0.084755	-0.04977	-0.28579	0.016536	-0.39946	-0.16761
ANGPTL2	-0.3289	-0.16392	0.139643	-0.09463	-0.01094	0.162595	-0.39532	-0.22145
ANGPTL3	0.171463	0.211987	0.285994	-0.20045	-0.02715	-0.07912	0.210705	-0.04145
ANGPTL4	-0.46646	-0.05	0.030658	-0.03904	0.074589	0.183432	-0.42316	0.208246
ANGPTL5	-0.2864	-0.33146	0.021862	-0.09129	-0.13229	-0.04139	0.301812	0.14095
ANGPTL6	0.377983	0.636291	0.037691	0.51792	0.203669	0.512737	0.100145	0.1403
ANGPTL7	0.131456	-0.06984	0.144719	-0.02121	-0.10496	0.260853	-0.2833	-0.1291
ANK1	-0.03665	0.64832	-0.0373	0.370353	-0.16024	0.180714	-0.47152	-0.21122
ANK2	-0.2158	-0.33715	-0.16613	0.074583	-0.04804	-0.06729	-0.46391	-0.30905
ANK3	0.01799	-0.20413	0.167224	-0.28343	0.324979	-0.20345	0.040346	-0.219
ANKAR	NA	NA	0.066751	-0.13766	-0.09976	0.164295	-0.18461	-0.2369
ANKDD1A	-0.35297	-0.18153	0.151642	0.059453	-0.23705	0.031577	-0.29344	0.081777
ANKFN1	0.16477	0.402055	-0.14213	-0.17217	-0.08383	-0.46862	-0.17984	-0.01913
ANKFY1	-0.14395	-0.4735	-0.26938	-0.07607	-0.02491	0.292087	0.075429	-0.27218
ANKH	0.179874	0.507512	0.094994	-0.12829	0.241092	0.094413	0.23389	-0.26698
ANKHD1	0.312543	-0.52239	0.317991	-0.09491	0.283698	0.426184	0.236863	0.278623
ANKIB1	NA	NA	0.132457	-0.1968	0.144328	-0.07108	NA	NA
ANKK1	0.105967	0.267826	0.030849	-0.14309	0.202335	0.392884	0.475611	0.562854
ANKLE1	NA	NA	-0.0107	0.114843	0.14534	0.066617	NA	NA
ANKLE2	NA	NA	0.115544	0.128021	0.338129	0.108559	NA	NA
ANKMY1	0.209976	0.532044	0.137998	-0.14564	0.300183	0.491873	0.152829	0.208506
ANKMY2	-0.13103	-0.48025	0.128021	-0.45956	-0.15886	-0.3019	-0.21256	-0.20944
ANKRA2	0.112235	-0.55494	0.061245	-0.07906	-0.0557	-0.17371	0.380976	0.178091
ANKRD1	-0.18119	-0.23031	0.176875	-0.18773	0.102265	-0.05323	-0.40458	-0.06007

ANKRD10	0.197896	-0.69169	-0.11221	0.084408	0.314761	0.354821	-0.04819	-0.07374
ANKRD11	0.474871	0.358896	0.058854	0.086784	0.228371	0.052315	-0.31489	-0.1545
ANKRD12	-0.19132	-0.51961	-0.20017	-0.04201	-0.15759	-0.02626	-0.45436	0.063035
ANKRD13A	-0.35834	-0.17004	-0.06879	-0.12228	-0.0923	-0.23425	NA	NA
ANKRD13B	0.306125	-0.15596	0.048881	0.048494	0.29256	-0.0115	0.133703	-0.03222
ANKRD13C	-0.27862	-0.65817	-0.20032	-0.30062	0.060098	-0.40973	0.283431	0.312668
ANKRD13D	0.169699	0.2614	0.297876	0.231948	0.25309	0.432029	0.064143	-0.02432
ANKRD16	0.15942	0.301639	0.40007	0.048073	0.139429	0.241658	0.231214	-0.2019
ANKRD17	-0.06703	-0.48922	0.160117	-0.05432	-0.02318	0.282271	0.134685	0.042284
ANKRD18A	NA	NA	0.086647	0.000697	0.217912	0.218158	NA	NA
ANKRD19	-0.05141	0.134572	0.268247	0.054232	0.044373	0.257889	0.066054	0.247844
ANKRD2	0.120585	-0.1976	0.011972	0.007163	-0.0035	-0.15768	0.061129	0.0135
ANKRD20A	0.130474	-0.1881	-0.2417	-0.1652	-0.06588	-0.18227	NA	NA
ANKRD22	-0.3172	-0.01744	0.210197	-0.1087	-0.0107	0.274769	0.019102	-0.11144
ANKRD23	0.058937	0.289583	-0.18643	0.149662	0.104279	0.089361	0.105619	0.260266
ANKRD24	NA	NA	-0.29308	-0.12786	-0.10878	-0.10084	-0.38042	-0.3755
ANKRD26	0.063	-0.57337	-0.15461	0.087647	-0.06922	-0.20407	0.15956	0.283945
ANKRD27	0.305108	-0.43682	0.257506	-0.12352	0.274354	-0.13955	0.032502	-0.56424
ANKRD28	0.097771	-0.30069	-0.22532	-0.22529	0.062908	-0.00105	-0.1608	-0.33425
ANKRD29	0.022137	-0.35102	0.178129	0.363545	-0.12689	0.561516	-0.34617	0.186317
ANKRD30A	0.276341	0.333982	-0.02398	-0.07999	-0.13735	0.117907	0.179744	0.145196
ANKRD30B	-0.20157	0.348284	NA	NA	NA	NA	-0.06563	0.188436
ANKRD32	0.034961	-0.58679	-0.17976	-0.10228	-0.0711	-0.13201	-0.27782	-0.34509
ANKRD33	-0.40679	0.399013	0.093297	-0.24304	0.402536	-0.10781	-0.29627	-0.12009
ANKRD34A	NA	NA	0.104616	0.173752	-0.06954	-0.00437	NA	NA
ANKRD34B	NA	NA	0.251582	0.365795	-0.15438	-0.20377	NA	NA
ANKRD35	-0.09447	0.099544	0.105251	0.086083	-0.08762	-0.04355	-0.26139	-0.10667
ANKRD36	-0.27467	-0.53198	NA	NA	NA	NA	0.067583	-0.073
ANKRD36B	NA	NA	-0.13361	0.132928	-0.00414	0.169296	NA	NA
ANKRD37	0.426457	0.209957	-0.29544	0.095884	-0.24806	-0.32056	0.056278	0.16801
ANKRD39	0.248137	0.519643	0.315225	-0.06304	0.04544	-0.2391	0.64382	0.079451
ANKRD40	-0.27313	0.072182	0.081924	0.03022	0.077356	-0.10858	-0.50546	-0.68674
ANKRD42	0.020596	-0.34789	-0.30445	-0.03835	0.08494	-0.34839	0.285751	0.332119
ANKRD43	0.087248	-0.12004	0.164197	-0.00325	0.474237	-0.25732	0.515294	0.31267
ANKRD44	-0.01365	-0.63525	0.32647	0.566121	-0.10438	0.743134	-0.62187	-0.58964
ANKRD45	-0.06695	0.325591	-0.01576	-0.36793	-0.15783	0.217964	-0.1347	0.109364
ANKRD46	-0.00704	-0.19133	-0.0209	-0.36127	-0.27056	-0.09739	0.507253	0.209158
ANKRD49	-0.10228	-0.56982	-0.17868	-0.11699	0.110079	0.013526	0.614193	0.44795
ANKRD5	0.340964	0.089898	-0.15961	-0.16704	-0.04622	0.207194	0.190308	0.450602
ANKRD50	-0.08917	-0.47419	-0.07092	-0.49049	-0.32908	-0.13268	-0.15722	-0.04729
ANKRD52	NA	NA	0.041815	-0.0852	-0.06917	-0.13221	NA	NA
ANKRD53	0.374417	-0.18223	-0.20339	-0.19797	0.075702	0.037725	NA	NA
ANKRD54	-0.16955	0.224276	0.21652	-0.17125	0.357318	-0.04765	NA	NA
ANKRD55	0.203687	-0.05635	0.012412	0.761795	-0.21378	0.220909	0.028095	0.1535
ANKRD57	-0.00985	0.365428	0.034074	-0.31458	0.098209	-0.09941	NA	NA
ANKRD6	0.198006	0.575738	0.055888	-0.07796	-0.33668	0.247207	-0.25868	-0.04787
ANKRD7	-0.09068	0.199079	-0.16421	0.092093	-0.19779	0.432367	0.099998	-0.12885
ANKRD9	0.144146	-0.0863	0.178249	-0.33088	0.227375	-0.11873	-0.40027	0.038939
ANKS1A	0.354443	-0.33186	-0.14255	-0.2818	0.048732	0.13101	-0.07251	-0.28475
ANKS1B	-0.24694	-0.32826	-0.02271	-0.09022	-0.33353	0.056341	-0.36497	-0.2473
ANKS3	0.114008	0.199378	0.015747	-0.00319	0.264018	0.204516	0.422496	0.408406
ANKS4B	0.526438	0.335031	-0.10478	-0.11398	0.281984	-0.30602	0.323432	0.065241

ANKS6	0.061781	0.143755	-0.10374	0.04347	0.343665	0.291058	-0.45918	-0.51123
ANKZF1	0.044604	-0.05831	0.314308	0.343674	0.37621	0.285121	0.15938	0.232729
ANLN	-0.17412	-0.1693	-0.42847	0.082889	0.00299	-0.13928	0.133743	0.037441
ANO1	NA	NA	-0.27588	-0.03	-0.17042	0.125186	NA	NA
ANO10	NA	NA	0.054947	-0.00116	0.228579	-0.0534	NA	NA
ANO2	NA	NA	-0.05932	-0.00166	-0.11227	-0.16073	NA	NA
ANO3	NA	NA	-0.25937	-0.05501	0.013519	0.004558	NA	NA
ANO4	NA	NA	-0.02943	-0.20653	-0.19086	-0.2811	NA	NA
ANO5	NA	NA	-0.11956	-0.48655	-0.33129	-0.21022	NA	NA
ANO6	NA	NA	-0.06325	-0.11084	0.283265	-0.24249	NA	NA
ANO7	NA	NA	-0.21272	0.265529	-0.05417	-0.07893	NA	NA
ANO8	NA	NA	-0.45273	0.360581	-0.00415	0.001822	NA	NA
ANO9	NA	NA	0.243966	0.267931	0.521252	0.057731	NA	NA
ANP32A	-0.02358	0.429177	-0.39042	0.02169	-0.03403	-0.35498	-0.37626	-0.30731
ANP32B	0.01014	-0.02298	-0.16832	0.392796	0.095909	0.07047	-0.3982	-0.36435
ANP32C	0.171213	-0.29551	-0.13353	0.277015	-0.08387	-0.03627	-0.57986	-0.24277
ANP32D	0.217969	0.266965	-0.22253	0.253206	0.037964	-0.30767	-0.33274	-0.09162
ANP32E	-0.03159	-0.20514	-0.27065	0.206436	-0.3237	-0.00942	0.11768	-0.18828
ANPEP	-0.315	0.203488	0.111082	-0.23423	0.088172	-0.14072	0.081446	0.211721
ANTXR1	-0.15085	-0.19524	0.012106	-0.05885	-0.05333	0.454196	-0.28661	-0.40781
ANTXR2	-0.50243	-0.40647	-0.14266	-0.26666	-0.20159	-0.22336	-0.50048	-0.30351
ANUBL1	0.103198	-0.23755	0.065091	-0.06073	0.138853	-0.13976	-0.07262	-0.08352
ANXA1	-0.31085	-0.38002	0.03039	-0.13305	-0.44967	0.07623	-0.0362	0.113859
ANXA10	-0.30984	0.157616	0.001017	-0.30574	-0.11421	-0.56411	0.092443	0.065243
ANXA11	-0.37036	0.114545	-0.2056	-0.03794	0.312324	0.183336	-0.4681	-0.17352
ANXA13	-0.02212	0.218673	0.034919	-0.10988	-0.00136	-0.19882	0.235449	0.383004
ANXA2	-0.35608	-0.16497	-0.07046	0.115061	-0.34159	-0.17257	0.265334	0.507926
ANXA2P1	NA	NA	0.301354	0.188089	-0.19163	0.157657	-0.51973	-0.13611
ANXA2P3	NA	NA	0.187083	0.039453	-0.18591	-0.11155	NA	NA
ANXA3	-0.21176	-0.32464	-0.1375	0.052458	-0.08844	-0.19065	-0.17264	-0.57776
ANXA4	0.043114	-0.49414	0.029696	-0.07452	0.279338	-0.11617	0.169377	0.027573
ANXA5	-0.43765	-0.31094	0.13199	0.144594	-0.10082	0.121168	0.180044	0.161364
ANXA6	-0.10639	0.117548	0.56824	0.205219	0.031034	0.262707	-0.05059	-0.06431
ANXA7	-0.03828	-0.25609	-0.16052	-0.18715	0.185205	-0.33388	-0.25204	-0.13619
ANXA8	-0.08674	0.148083	NA	NA	NA	NA	0.331729	0.567534
ANXA8L1	NA	NA	NA	NA	NA	NA	0.253493	-0.014
ANXA9	NA	NA	0.488037	-0.15437	0.004133	-0.16172	0.53212	-0.09515
AOAH	0.289893	0.25017	0.449427	0.045505	0.298748	0.250921	-0.32042	0.03666
AOC2	0.179446	0.337234	-0.07073	-0.24486	0.050706	-0.1394	0.284879	0.13447
AOC3	-0.29947	-0.00938	-0.02479	-0.03497	-0.11418	0.154409	-0.41803	-0.52494
AOF1	0.191658	-0.51368	-0.03594	0.074251	0.004239	-0.04116	0.515604	0.273018
AOF2	0.105814	0.211367	0.070464	0.199224	0.111747	0.096862	0.472434	0.150261
AOX1	0.114793	-0.349	0.161517	0.06122	-0.08931	-0.14466	-0.20128	0.084044
AP1B1	-0.18555	0.230346	0.224814	0.119355	0.320206	-0.07988	-0.30571	-0.00662
AP1G1	-0.42092	-0.61971	-0.38843	-0.02745	0.113258	-0.11493	0.187099	0.237188
AP1G2	-0.13983	-0.35215	-0.04473	0.397667	0.205451	0.371623	0.426863	0.44865
AP1GBP1	0.015685	0.32797	0.087018	0.099741	-0.09233	0.16616	-0.16251	0.25397
AP1M1	-0.38061	-0.34143	0.454592	0.190667	0.253935	0.288755	-0.48658	-0.19624
AP1M2	0.617964	0.315648	0.139531	0.242034	0.27278	-0.12485	0.184099	-0.08522
AP1S1	0.216071	-0.04534	0.216185	0.157257	0.140705	-0.27923	0.427464	0.098678
AP1S2	-0.24306	-0.25093	-0.02664	-0.02424	-0.06515	0.171099	-0.3237	-0.29806
AP1S3	-0.16389	0.11297	-0.03331	0.124268	0.033594	-0.05107	-0.20305	-0.35073

AP2A1	0.358475	0.659225	0.214771	-0.05998	0.194388	-0.0635	0.257173	-0.07633
AP2A2	-0.1377	-0.21274	0.161858	0.01981	0.308022	0.334161	0.214209	0.249508
AP2B1	-0.07784	0.333855	0.292638	0.208528	-0.00092	-0.00603	0.0911	-0.03443
AP2M1	-0.19847	0.279616	0.226526	0.244472	0.120269	-0.20619	0.127267	0.157625
AP2S1	-0.07319	-0.31208	-0.08844	0.091908	-0.19278	-0.10875	0.244376	0.067507
AP3B1	-0.26291	-0.57916	0.339265	-0.02535	0.093258	-0.03653	0.31699	0.175671
AP3B2	0.215776	0.207853	0.034105	0.293117	0.03012	-0.06857	-0.42059	-0.26245
AP3D1	0.008452	-0.09589	0.235052	0.336893	0.407289	0.19149	0.424023	0.335402
AP3M1	-0.02732	-0.18104	-0.0211	-0.15519	0.136592	0.022588	0.400253	0.432107
AP3M2	-0.22386	0.083887	-0.05516	0.389874	0.16812	0.23761	0.038284	0.052856
AP3S1	-0.34628	-0.68815	0.268793	-0.27833	-0.14121	-0.37935	0.168523	0.074398
AP3S2	0.165116	0.47651	-0.28799	-0.12966	-0.03436	-0.43971	-0.15498	0.01565
AP4B1	-0.24036	-0.08722	0.153692	-0.08358	0.183541	-0.02509	-0.04685	0.220919
AP4E1	-0.24005	-0.27287	-0.13043	-0.39511	0.099904	-0.06943	0.261242	0.461768
AP4M1	0.207691	-0.48128	0.431464	-0.11088	0.403222	0.004051	0.172931	0.304016
AP4S1	-0.1587	-0.20036	-0.34007	-0.24737	-0.41028	-0.18349	-0.28943	-0.10293
APAF1	-0.20547	-0.2144	-0.32878	0.114027	0.223307	-0.18572	-0.35219	-0.0814
APBA1	-0.3157	0.590615	-0.01847	-0.12545	-0.13044	-0.00122	0.270605	-0.31527
APBA2	-0.21154	-0.32758	0.126962	0.436291	0.160755	0.545719	0.111571	0.030684
APBA3	0.257671	0.762259	-0.2753	0.40978	-0.10107	-0.3763	0.152761	0.430643
APBB1	0.244931	0.050454	0.444961	0.073761	0.154325	-0.01071	-0.16728	0.112783
APBB1IP	-0.59424	-0.17812	0.402145	0.737443	-0.15993	0.725967	-0.48282	0.419305
APBB2	-0.37795	-0.40085	0.007371	-0.31365	-0.12725	-0.5459	0.224956	-0.06783
APBB3	-0.02873	0.221753	0.290456	0.078393	0.175187	0.124657	-0.11276	0.327264
APC	-0.03343	-0.21484	-0.0525	-0.3604	-0.04029	-0.01355	-0.11614	-0.03794
APC2	0.194838	0.333445	-0.27817	-0.06284	-0.07648	-0.04807	0.108645	-0.23268
APCDD1	0.202932	-0.49065	0.643107	0.226581	0.282919	-0.0602	0.474269	0.227352
APCDD1L	-0.25098	-0.0648	0.110738	0.127104	-0.10878	0.020484	NA	NA
APCS	0.485561	0.260509	0.184361	0.03442	0.002001	-0.3579	0.438556	0.403374
APEH	0.432341	0.129125	0.090087	0.090673	0.285449	0.130883	0.063257	-0.18711
APEX1	0.252993	-0.04741	-0.2326	0.408583	0.124622	0.116729	0.419132	0.43831
APEX2	0.401232	0.347003	-0.0684	0.047053	0.181077	0.040658	0.411369	0.275038
APH1A	-0.18757	-0.20661	0.22076	0.167829	0.152438	-0.02526	0.449438	-0.07023
APH1B	0.143737	-0.04027	0.32434	-0.32574	-0.24871	0.178105	-0.07198	0.263606
API5	0.341153	-0.33221	-0.40975	-0.1602	-0.08246	0.019645	0.360796	0.235485
APIP	-0.26147	-0.63179	-0.28253	-0.18873	-0.19013	-0.3122	-0.12587	-0.16609
APITD1	-0.28081	-0.38262	0.008652	0.200424	0.052058	-0.44752	0.259646	0.32123
APLF	NA	NA	-0.18795	-0.11938	0.20581	0.343116	NA	NA
APLN	-0.31468	-0.10375	-0.21423	-0.24771	0.114852	-0.21968	-0.34515	-0.2358
APLNR	NA	NA	0.28589	0.052115	0.00396	-0.07213	NA	NA
APLP1	-0.31905	-0.24319	0.121563	-0.36368	-0.12357	-0.18461	-0.22621	-0.27063
APLP2	-0.09439	-0.43652	0.176422	-0.00904	0.252094	0.195579	-0.45192	-0.5668
APOA1	0.246968	-0.00018	-0.28134	-0.21138	-0.0795	-0.24613	0.254372	0.476746
APOA1BP	0.045192	0.528419	0.211162	0.197868	-0.04451	-0.18428	0.604687	0.23037
APOA2	0.120235	0.137903	-0.0202	-0.25629	0.042905	0.120447	0.280198	0.361177
APOA4	0.153344	0.083742	-0.01736	-0.1327	-0.223	0.099349	0.431877	0.504221
APOA5	0.382858	0.559913	0.060401	-0.15403	0.29919	-0.26101	0.070358	0.408016
APOB	0.273249	-0.14057	0.03002	-0.31626	0.002537	-0.26222	0.304513	0.504414
APOB48R	0.098519	0.160141	-0.05885	0.148692	0.204354	0.381145	0.052223	0.007642
APOBEC1	0.248531	0.204976	-0.07526	-0.31554	0.029681	-0.1346	0.019051	0.318284
APOBEC2	-0.0079	-0.08773	0.002703	-0.08283	-0.04813	0.144861	0.161484	0.148278
APOBEC3A	-0.46454	0.098168	-0.09981	-0.16591	-0.05852	-0.13687	-0.06033	0.240222

APOBEC3B	-0.14962	0.071272	0.123117	-0.51773	0.071045	0.049809	0.413653	0.070547
APOBEC3C	0.154666	0.366737	0.191419	0.299122	-0.14519	0.067603	-0.05519	-0.0696
APOBEC3D	-0.02736	0.675643	0.319717	0.071539	0.245591	-0.01915	0.362146	0.067582
APOBEC3F	0.064986	-0.2492	0.01499	-0.2345	-0.09164	-0.00717	-0.08034	-0.22312
APOBEC3G	0.005599	0.462163	0.239569	0.431979	-0.286	0.52859	-0.36021	-0.0745
APOBEC3H	0.005813	0.240164	0.097668	0.190951	-0.2187	0.209851	NA	NA
APOBEC4	-0.16769	-0.00637	NA	NA	NA	NA	-0.17651	-0.03963
APOC1	0.031598	0.321387	0.062088	0.164926	-0.37543	-0.08582	-0.08719	-0.16096
APOC2	0.451333	0.532217	0.155322	-0.23306	-0.40571	-0.16973	-0.03377	0.182401
APOC3	-0.30317	-0.14102	-0.15389	0.2111	-0.05644	-0.0027	0.356727	0.481791
APOC4	-0.031	0.311035	-0.12535	-0.16762	-0.01715	0.146088	0.052638	0.51421
APOD	0.10755	-0.33127	-0.0779	0.102887	-0.21569	-0.06854	-0.0171	-0.02494
APOE	-0.19571	-0.00455	0.279477	0.214314	-0.33656	0.215489	-0.0874	0.155589
APOF	0.269009	0.58413	0.080176	-0.14958	0.048961	0.111548	-0.64109	-0.49751
APOH	-0.02931	0.123168	-0.13235	-0.13968	-0.12178	-0.3729	0.257244	-0.43419
APOL1	-0.02259	0.006718	0.131788	-0.07663	-0.13413	0.331399	-0.46643	-0.14736
APOL2	0.122466	0.599914	0.329896	-0.02125	-0.01219	0.015227	-0.48358	-0.50673
APOL3	-0.36959	0.286949	0.162108	0.227283	-0.22716	0.288152	-0.44754	0.077085
APOL4	0.267433	0.176961	0.270576	-0.06591	-0.11727	0.384108	-0.25528	-0.0396
APOL5	0.323162	0.3707	-0.25133	-0.28271	0.159467	-0.41803	-0.32318	-0.48234
APOL6	0.064718	0.656162	0.302316	-0.10771	0.117872	0.051386	0.035106	0.328955
APOLD1	0.246134	-0.34468	-0.07146	-0.05058	0.237572	-0.01726	-0.02073	-0.10505
APOM	0.124848	0.198616	0.151126	-0.48893	-0.03824	-0.49545	0.376418	0.505907
APOO	0.087109	0.28113	-0.03115	-0.19261	0.168806	-0.23657	NA	NA
APOOL	0.186998	-0.46523	-0.20208	-0.43545	-0.2564	-0.19898	NA	NA
APP	-0.41346	-0.01509	0.055171	0.254335	0.176978	0.25518	0.293983	0.002406
APPBP2	-0.05502	-0.1641	0.15711	-0.30554	-0.03238	-0.0307	-0.36587	-0.37786
APPL1	-0.02509	0.1159	-0.10912	-0.0694	0.151452	0.254	NA	NA
APPL2	0.092671	0.066588	-0.13041	-0.21505	-0.12067	-0.36849	NA	NA
APRT	0.373436	-0.01988	0.149709	0.159859	0.204594	-0.00952	0.347464	0.004466
APTX	0.068233	0.33249	-0.12686	-0.08155	0.050964	-0.5013	-0.06788	0.064274
AQP1	-0.0092	-0.19969	-0.03324	-0.10709	-0.06583	0.529754	-0.22183	0.012081
AQP10	0.201883	0.636631	0.055939	-0.11331	-0.16083	-0.23893	0.505224	0.406205
AQP11	0.146665	0.18665	0.056465	-0.33819	-0.09056	-0.47103	0.414881	0.239799
AQP12A	0.066365	-0.03365	-0.17041	-0.05379	-0.14233	-0.18182	0.247676	0.207617
AQP2	-0.08343	0.172137	-0.00248	-0.03329	0.064285	-0.07066	0.559856	0.59015
AQP3	-0.24868	-0.51345	0.12813	0.512609	0.182665	-0.06139	0.029059	0.026531
AQP4	-0.01211	0.017494	-0.05042	-0.19132	-0.17514	-0.16679	0.217967	0.026942
AQP5	-0.19652	-0.11307	-0.26358	0.106457	-0.23597	-0.17449	0.166569	-0.44941
AQP6	-0.14961	0.279134	-0.28969	0.07309	0.038786	-0.13242	-0.05134	-0.3583
AQP7	0.413202	0.277229	0.261124	-0.05499	0.430287	0.081979	-0.16117	0.464203
AQP8	0.055184	0.164565	-0.25426	-0.20441	-0.09087	-0.11894	-0.06111	-0.37797
AQP9	-0.47621	-0.10325	0.059617	0.145438	0.077126	-0.03842	-0.35565	-0.0184
AQR	0.469305	0.083199	-0.26082	-0.1634	-0.05945	0.186013	-0.259	-0.19077
AR	0.058089	0.126949	-0.00842	0.018272	-0.1362	0.348098	-0.16738	-0.02154
ARAF	0.482441	0.385698	0.191472	0.220048	0.256993	-0.12253	-0.39312	-0.30476
ARAP1	NA	NA	0.303126	0.257175	0.209218	0.389644	NA	NA
ARAP2	NA	NA	0.276919	-0.15151	-0.18031	0.254699	NA	NA
ARAP3	NA	NA	-0.00539	0.275594	0.100568	0.3195	NA	NA
ARC	-0.05033	0.193662	-0.11868	-0.20371	-0.10439	-0.03295	-0.38998	0.068532
ARCN1	-0.47409	-0.71655	-0.08624	0.178792	-0.02758	0.075192	0.456168	0.484535
ARD1A	0.393087	0.418231	-0.01543	0.253124	0.261439	0.128554	-0.04803	-0.35307

AREG	0.288775	0.384404	0.242044	0.049934	0.27787	-0.13837	0.204161	0.425598
ARF1	-0.40425	-0.24643	0.141067	0.194545	0.132855	0.071646	0.224391	-0.14415
ARF3	-0.08457	-0.34726	0.154086	0.052259	0.005589	-0.09487	-0.21639	-0.32014
ARF4	-0.39471	-0.54021	-0.15187	-0.10938	-0.18014	-0.03455	0.024695	0.038879
ARF5	0.076996	0.238473	0.151542	0.134072	0.158286	-0.00309	0.532843	0.158084
ARF6	0.317378	0.110165	-0.24496	0.125817	0.042808	0.213289	-0.64375	-0.45786
ARFGAP1	0.212041	0.162056	0.389991	0.163979	0.429321	0.080881	0.27714	0.291474
ARFGAP2	NA	NA	0.233017	0.262166	0.117191	0.042399	NA	NA
ARFGAP3	-0.63924	-0.5425	0.245456	0.052334	-0.25144	-0.0474	-0.49377	-0.48806
ARFGEF1	-0.09369	-0.43599	0.149145	-0.22414	-0.00966	0.07494	-0.16718	-0.2
ARFGEF2	0.251858	0.018727	-0.03127	-0.06214	0.25986	0.043054	0.33331	0.247204
ARFIP1	-0.04372	-0.45676	-0.45155	-0.11538	-0.12001	-0.21121	0.326073	0.271259
ARFIP2	-0.25652	-0.53498	0.299641	0.019252	0.007295	-0.06145	-0.11004	-0.1366
ARFRP1	0.105608	0.092022	0.256965	0.035769	0.365446	-0.25569	0.263194	0.360448
ARG1	-0.09259	0.504023	0.020473	-0.26681	0.004257	-0.50456	0.010102	0.315525
ARG2	0.414489	0.729763	-0.00318	0.225051	0.076152	0.13454	0.050765	0.36455
ARGFX	-0.09558	0.120473	NA	NA	NA	NA	0.314843	0.532743
ARGLU1	NA	NA	-0.28679	0.258211	-0.01266	-0.42889	NA	NA
ARHGAP1	-0.16269	0.060582	-0.0219	0.057565	0.2076	0.300586	-0.08377	-0.13542
ARHGAP10	-0.06979	-0.03813	0.274303	-0.01636	-0.28145	0.247101	-0.27488	-0.31396
ARHGAP11.	-0.15288	0.11702	-0.4248	0.041497	-0.26032	-0.0548	-0.09044	-0.03361
ARHGAP12	-0.23799	-0.36925	-0.25333	-0.24196	-0.11158	-0.40994	0.08291	0.045591
ARHGAP15	-0.56501	-0.38225	0.150192	0.569142	-0.27062	0.564011	-0.6651	0.033292
ARHGAP17	0.209677	0.127075	0.018979	-0.11298	0.097048	0.179646	-0.04258	0.194167
ARHGAP18	-0.12009	-0.46198	-0.05012	-0.13622	0.034415	-0.13603	-0.37262	-0.18119
ARHGAP19	-0.39042	-0.48253	0.088262	-0.14314	-0.06271	-0.18119	0.16147	0.528606
ARHGAP20	-0.15824	-0.54853	-0.15949	-0.03342	-0.22721	-0.0533	-0.16194	0.413013
ARHGAP21	-0.24938	0.383798	-0.36551	-0.33515	-0.05832	-0.23815	-0.5422	-0.14083
ARHGAP22	-0.54904	-0.54083	0.077463	0.392275	-0.2976	0.560562	-0.45443	-0.43494
ARHGAP23	NA	NA	-0.21104	0.087041	-0.0215	0.065667	NA	NA
ARHGAP24	-0.52035	-0.35132	-0.2029	0.39338	-0.25737	0.534672	-0.07522	0.060131
ARHGAP25	-0.45502	-0.10493	0.411014	0.742452	-0.11121	0.767215	-0.4078	0.232403
ARHGAP26	-0.05698	-0.24741	0.167708	0.019899	0.050921	0.266999	0.047196	0.349718
ARHGAP27	0.070716	0.269618	0.292875	-0.02488	0.222151	-0.00412	-0.21066	-0.1648
ARHGAP28	0.017514	-0.4161	-0.32526	-0.21609	-0.15172	-0.19412	-0.33798	0.044158
ARHGAP29	-0.50169	-0.36363	-0.07704	-0.09433	-0.14283	0.19977	-0.28903	0.077098
ARHGAP30	0.041498	0.055197	0.331846	0.680307	-0.07482	0.674227	-0.58357	0.29895
ARHGAP4	-0.05886	0.247111	0.024366	0.631496	0.10283	0.687988	0.157125	0.389184
ARHGAP5	0.110544	0.639335	-0.39851	-0.23228	-0.13776	-0.32161	0.027202	-0.0549
ARHGAP6	0.343617	0.447157	-0.12936	-0.16058	-0.12499	-0.32649	0.284464	0.016795
ARHGAP8	0.080251	0.713824	-0.12148	-0.07693	0.248328	0.207603	0.527862	0.281484
ARHGAP9	-0.47102	0.222938	0.264975	0.795443	-0.03548	0.674561	-0.66477	0.314005
ARHGDIA	0.025714	0.314775	0.252094	0.244354	0.115522	0.092985	0.314769	0.159745
ARHGDIB	-0.23492	0.14623	-0.19382	0.776045	-0.18127	0.534515	-0.65835	0.093309
ARHGDIG	0.28604	0.731608	-0.05144	-0.15216	-0.05337	-0.00669	0.301572	0.476202
ARHGEF1	0.09019	-0.34226	0.122103	0.537382	0.148735	0.494331	0.030918	-0.00342
ARHGEF10	-0.43748	-0.25401	-0.14699	0.068083	0.098464	0.175241	-0.09394	0.020288
ARHGEF10l	0.007003	0.43608	0.007483	0.204179	0.273447	-0.09676	0.048992	0.010911
ARHGEF11	0.170008	0.223021	0.095075	0.036429	0.173919	-0.04204	-0.11845	-0.09697
ARHGEF12	0.22484	0.488562	0.038978	-0.52689	-0.16684	-0.08375	-0.39082	-0.04085
ARHGEF15	-0.06398	0.200718	-0.03473	0.071493	-0.1586	0.020106	-0.44571	0.020382
ARHGEF16	-0.44702	0.238025	0.240645	-0.14811	0.306753	0.179644	0.103459	-0.13989

ARHGEF17	-0.37878	0.140112	0.003799	-0.02077	-0.11786	0.129295	-0.25503	-0.10088
ARHGEF18	0.091616	0.158883	-0.08664	-0.11785	0.162926	0.16254	0.045522	0.052872
ARHGEF19	-0.00611	0.374913	0.133658	0.516858	0.235425	-0.0002	0.427932	0.476877
ARHGEF2	-0.06206	0.52102	0.056606	0.243087	0.297078	0.164186	-0.07424	-0.04618
ARHGEF3	-0.09926	-0.43864	0.066819	0.395622	0.096295	0.53475	-0.17284	0.023006
ARHGEF4	0.337628	-0.02701	-0.39406	-0.1042	-0.01637	-0.16561	-0.4083	-0.18827
ARHGEF5	0.260028	0.195918	0.235221	-0.01252	0.299468	0.144393	0.496531	0.110785
ARHGEF6	-0.44359	0.004218	0.194037	0.414278	-0.20358	0.360326	-0.52215	0.214719
ARHGEF7	0.022345	-0.42374	0.036384	-0.0534	0.417878	0.242261	0.165815	-0.02303
ARHGEF9	0.27546	0.726157	-0.23206	0.084356	-0.09653	-0.10183	-0.22587	-0.02452
ARID1A	-0.14822	-0.52424	-0.10135	0.311985	0.316453	0.342424	-0.12593	0.239118
ARID1B	0.098998	0.610093	0.059084	0.079192	0.228232	0.12318	0.649205	0.402118
ARID2	-0.03843	-0.64659	-0.19493	-0.42644	0.206261	0.079823	-0.34136	-0.12475
ARID3A	0.325659	0.378866	0.055601	0.268599	0.232127	0.26438	0.339131	0.338683
ARID3B	0.023492	0.414069	-0.23476	-0.02887	-0.15936	-0.11201	NA	NA
ARID3C	-0.19789	0.460202	NA	NA	NA	NA	-0.11408	0.273525
ARID4A	-0.31498	-0.58345	-0.43386	-0.14245	-0.17454	0.018329	0.093603	0.170672
ARID4B	-0.30884	-0.47836	-0.24382	-0.12546	-0.18036	0.050667	-0.3507	-0.24919
ARID5A	0.182374	0.557866	0.258101	0.116275	0.197514	0.405424	-0.04687	0.009831
ARID5B	0.113943	-0.3192	0.04696	0.197866	-0.14998	0.439488	0.087463	0.131915
ARIH1	-0.37047	-0.50115	0.122209	-0.34479	0.323611	0.034081	-0.18703	-0.29081
ARIH2	-0.00343	-0.12546	-0.10426	-0.07847	0.336338	0.301461	0.161382	0.278914
ARL1	-0.24664	-0.42334	-0.2155	-0.03607	-0.36228	-0.3854	0.145053	0.171003
ARL10	0.03169	0.481347	0.031657	0.002404	0.070912	0.188129	0.32044	-0.12562
ARL11	0.087171	-0.15911	0.039504	0.12747	0.157538	0.420585	0.147102	0.274674
ARL13A	0.31291	0.544894	NA	NA	NA	NA	0.093003	-0.0679
ARL13B	-0.20977	-0.42363	-0.31623	0.118817	-0.13917	0.237352	-0.51775	-0.28975
ARL14	-0.08654	-0.04754	-0.23543	-0.33915	-0.06925	0.096209	-0.13605	0.137523
ARL15	-0.18777	-0.28044	0.011981	-0.16173	-0.06655	-0.2595	-0.3232	-0.20316
ARL16	NA	NA	0.209003	0.026088	0.402455	0.182045	-0.50583	-0.58013
ARL2	0.122413	0.500164	0.124656	-0.05841	0.0793	-0.11651	-0.27354	-0.18077
ARL2BP	0.39536	0.05593	-0.22034	0.00678	0.040167	-0.02757	0.297022	0.057688
ARL3	-0.42048	-0.03233	0.093399	-0.08077	0.231417	-0.17289	0.207075	0.16912
ARL4A	-0.15339	-0.36139	-0.14965	-0.34944	-0.17669	-0.51418	-0.23432	-0.26566
ARL4C	-0.26729	0.333286	0.604315	0.393647	-0.17762	0.43552	-0.3703	-0.09756
ARL4D	-0.2687	-0.20086	0.188816	-0.04356	0.0368	-0.06093	-0.17384	-0.04402
ARL5A	0.322155	0.369635	-0.15408	-0.22319	-0.27081	-0.11934	0.106364	-0.08878
ARL5B	-0.26557	-0.18182	-0.24713	-0.26705	-0.13275	0.064498	0.054335	0.154485
ARL6	0.282443	-0.06617	-0.23383	-0.03911	-0.19049	-0.10866	0.047244	0.034503
ARL6IP1	-0.45503	-0.58698	-0.37789	-0.13396	-0.02673	0.055298	-0.36564	-0.52122
ARL6IP4	-0.05446	-0.20537	0.204292	0.301363	0.316892	0.059862	-0.30414	-0.2349
ARL6IP5	-0.35224	0.034134	-0.13466	-0.37463	-0.18476	-0.28079	-0.56718	-0.55327
ARL6IP6	-0.19788	-0.56756	-0.22998	-0.09822	-0.06789	-0.22767	-0.10355	0.102739
ARL8A	0.286494	0.617942	0.204591	0.220971	0.004881	-0.05264	-0.12908	-0.18152
ARL8B	0.144335	-0.03174	-0.48273	-0.34244	-0.19337	-0.20504	-0.49931	-0.42476
ARL9	-0.08313	-0.14925	NA	NA	NA	NA	-0.46694	-0.16636
ARMC1	0.050861	0.228027	0.096174	-0.00376	-0.17771	-0.2932	-0.42561	-0.62489
ARMC10	0.071697	-0.45561	-0.12927	0.003519	0.01221	-0.07956	0.500133	0.285868
ARMC2	0.135874	-0.5417	0.40102	-0.1848	0.18361	-0.06648	-0.68885	-0.45203
ARMC3	NA	NA	-0.01962	-0.08326	-0.2303	-0.04177	NA	NA
ARMC4	0.183116	0.017271	-0.11765	-0.10499	-0.22973	0.023694	-0.05159	-0.12507
ARMC5	0.230792	0.242445	-0.2213	0.374377	0.341444	0.266116	-0.31183	-0.00743

ARMC6	0.476143	0.708533	0.083623	0.271714	0.172186	0.24095	0.123671	-0.269
ARMC7	-0.35307	0.34892	0.337831	0.103083	-0.08064	0.163572	0.24918	0.285669
ARMC8	0.020421	-0.2707	-0.55505	-0.51256	-0.18589	-0.2938	0.215602	0.343298
ARMC9	-0.28784	0.170204	0.079887	-0.0848	0.027265	-0.08282	-0.50272	-0.36654
ARMCX1	-0.10082	-0.58071	-0.19864	-0.0985	-0.21501	-0.16495	-0.40948	-0.28496
ARMCX2	0.001389	-0.3051	0.078605	0.05009	-0.20305	0.055439	-0.29434	-0.28984
ARMCX3	-0.11996	-0.55726	-0.076	0.028217	0.079167	-0.1097	-0.07025	-0.14507
ARMCX4	0.214405	-0.09774	0.08839	-0.00101	0.011917	0.280193	NA	NA
ARMCX5	0.336392	-0.56104	-0.24278	-0.29731	0.119386	0.207635	0.110242	0.142538
ARMCX6	-0.02667	-0.31188	-0.03705	0.055004	-0.11826	0.025307	-0.27078	-0.38719
ARMET	-0.17392	0.370299	0.070313	0.263862	0.066871	-0.01254	0.508152	0.113956
ARMETL1	0.081795	0.599125	-0.03969	-0.284	-0.06297	-0.25258	-0.07025	0.389766
ARNT	-0.57881	0.118209	-0.20977	0.059559	-0.04262	-0.09381	-0.38057	-0.3312
ARNT2	-0.13052	-0.31503	-0.13945	0.302088	-0.23119	0.042131	-0.40478	-0.04673
ARNTL	-0.14622	-0.29983	0.119067	0.396493	-0.29269	0.04427	0.221112	0.327363
ARNTL2	0.219618	0.747263	-0.06672	0.133427	-0.04948	0.044265	-0.33768	-0.12327
ARPC1A	0.087752	-0.46728	0.093961	0.13222	0.235756	-0.37195	0.334184	0.504934
ARPC1B	NA	NA	0.378182	0.439808	0.428614	0.140223	0.233585	0.334487
ARPC2	0.110669	-0.24395	0.347825	0.095145	-0.12776	0.208021	-0.0888	0.092255
ARPC3	-0.33744	-0.10594	-0.34736	0.229175	0.009644	-0.46649	0.027079	0.004423
ARPC4	0.041598	0.3672	-0.23814	0.316971	-0.14103	-0.34245	0.449502	0.195012
ARPC5	-0.07079	-0.12862	-0.02257	0.066075	-0.1996	-0.11784	-0.34077	-0.4107
ARPC5L	-0.18072	0.12415	-0.31123	0.269477	0.00492	-0.10305	0.294343	0.372289
ARPM1	0.314179	0.522059	0.114401	-0.11675	0.087851	-0.39091	-0.14361	-0.32047
ARPP-19	0.015652	-0.26348	-0.10745	-0.33917	-0.11763	0.081114	-0.71844	-0.62204
ARPP-21	-0.06122	-0.07744	-0.22233	-0.18673	0.087451	-0.14293	-0.20384	-0.17001
ARR3	0.133869	0.457952	-0.02466	-0.08091	-0.35282	-0.46674	0.072571	0.383263
ARRB1	0.042567	0.592057	0.296613	0.24136	0.224327	0.205363	-0.15819	0.035049
ARRB2	-0.18438	0.111391	0.343114	0.185204	0.03312	0.126338	-0.32303	0.395603
ARRDC1	0.092272	0.203218	0.384856	0.013857	0.157187	-0.04243	0.576842	0.354309
ARRDC2	0.082078	0.636493	0.071007	0.161294	0.108025	0.316883	-0.16223	0.23116
ARRDC3	-0.12696	-0.44747	-0.08952	-0.11898	-0.14473	-0.20912	0.278771	0.364368
ARRDC4	-0.30274	-0.31594	-0.27475	-0.35726	-0.19904	-0.25277	-0.31001	0.073606
ARRDC5	NA	NA	0.110909	0.372661	-0.21304	0.299886	-0.60392	0.121669
ARS2	0.489885	-0.15001	-0.12523	0.401658	0.351394	0.332684	0.047958	0.138399
ARSA	-0.24982	-0.13518	0.315919	-0.2049	0.18567	-0.16373	-0.15789	-0.0978
ARSB	-0.34279	-0.28846	0.266361	-0.02896	-0.30724	-0.26578	-0.29072	-0.28303
ARSD	-0.05575	-0.01698	0.411517	0.004448	0.285572	0.086873	0.21997	0.265916
ARSE	0.388993	0.365913	0.255371	0.223429	0.227362	0.108872	0.250977	-0.28409
ARSF	0.308426	0.583787	0.218479	-0.166	0.186125	-0.26724	0.051627	-0.16326
ARSG	0.28171	0.110081	-0.06529	0.199979	0.256646	0.381398	-0.25274	-0.25648
ARSH	0.095617	-0.19724	NA	NA	NA	NA	0.052126	0.366698
ARSI	0.000419	0.201171	0.107055	0.229705	-0.15164	-0.05249	-0.01966	-0.29933
ARSJ	-0.22027	-0.32351	0.116388	-0.15973	-0.5374	0.006679	-0.04431	-0.08366
ARSK	-0.07567	-0.31484	-0.11049	0.049686	-0.11654	-0.21395	0.423362	0.102619
ART1	0.032157	0.041639	0.051516	-0.00172	0.103885	0.217956	0.067325	-0.11381
ART3	0.29309	-0.28263	-0.17733	0.029843	-0.04678	0.10042	0.091878	-0.13897
ART4	0.162601	0.446999	-0.32686	0.528858	-0.10228	0.06857	0.074657	-0.50494
ART5	0.12664	-0.13571	-0.01503	-0.40258	0.076324	-0.16787	-0.00515	-0.28415
ARTN	0.087422	0.180219	0.366519	-0.05961	0.007852	-0.00566	0.131458	-0.18581
ARV1	-0.12189	-0.24619	-0.01418	-0.39009	-0.17532	-0.15286	0.384946	-0.19451
ARVCF	0.024095	0.392643	0.007562	-0.02964	0.331826	-0.18265	-0.28591	-0.18559

ARVP6125	0.440835	-0.16108	NA	NA	NA	NA	0.633215	0.542777
ARX	-0.19638	0.318136	-0.0681	0.061698	-0.20333	-0.17398	-0.34751	-0.47941
AS3MT	0.337162	-0.33013	0.17235	-0.31018	0.137321	0.076886	-0.1031	-0.17179
ASAH1	-0.36978	-0.43726	0.169082	-0.21259	-0.30059	-0.08413	-0.36369	-0.32322
ASAH2	0.160673	0.461245	-0.15845	-0.06576	-0.18012	0.013158	-0.57283	0.360422
ASAM	-0.00571	0.203728	0.093778	0.116574	-0.07918	-0.01671	-0.20253	-0.15152
ASAP1	NA	NA	-0.01952	0.028736	0.069264	0.323394	NA	NA
ASAP2	NA	NA	-0.36241	-0.31428	-0.02752	-0.20885	NA	NA
ASAP3	NA	NA	0.007201	-0.08091	-0.13017	-0.01183	NA	NA
ASB1	-0.22829	-0.08309	0.073786	0.107393	0.263404	0.331728	-0.55659	-0.41985
ASB10	-0.31492	-0.06324	0.249664	-0.02842	-0.02807	0.0103	-0.02611	-0.25286
ASB11	0.017067	0.657045	-0.03173	-0.16522	-0.23528	0.078727	0.462296	0.225882
ASB12	0.101271	0.157493	-0.27441	-0.30025	0.100512	-0.26597	0.269528	0.376356
ASB13	0.255451	0.04305	0.25769	0.110346	0.340006	-0.15608	0.618179	0.403834
ASB14	0.120122	0.308103	-0.15038	-0.20842	0.178286	0.223844	-0.15939	0.363369
ASB15	0.284536	0.207445	NA	NA	NA	NA	0.190291	0.063546
ASB16	0.426552	0.746589	0.068258	-0.013	-0.07444	-0.3119	-0.43891	-0.39218
ASB17	0.200098	0.72602	-0.05065	-0.40045	-0.27003	0.272757	0.18333	0.556794
ASB18	-0.02294	-0.21455	NA	NA	NA	NA	NA	NA
ASB2	0.25498	0.508351	0.180539	0.076708	0.116656	0.575408	-0.35335	-0.30418
ASB3	0.03784	-0.68877	-0.07566	0.059822	-0.29455	0.357435	-0.24393	-0.32926
ASB4	-0.19376	0.305531	-0.03899	-0.14829	-0.22845	-0.33406	-0.06497	-0.2169
ASB5	-0.01358	-0.14634	0.080541	-0.20228	0.333936	-0.17025	-0.40342	-0.29823
ASB6	0.02196	0.628771	0.216839	-0.04434	0.286694	0.054369	0.278521	0.229244
ASB7	-0.20715	0.311115	-0.27431	-0.31727	-0.13402	0.01974	-0.40127	-0.14676
ASB8	-0.36935	-0.30145	-0.15003	-0.18301	-0.08194	-0.43295	-0.55782	-0.44619
ASB9	0.474425	-0.34695	-0.35889	-0.18754	0.089355	-0.39175	0.072281	0.085683
ASCC1	-0.00294	0.201303	-0.17416	0.030892	0.053645	-0.22001	0.382777	0.327736
ASCC2	-0.1066	0.589935	0.247866	0.171892	0.26016	-0.03782	-0.22462	-0.16159
ASCC3	0.28288	-0.2807	-0.06311	-0.16454	0.062145	0.106385	-0.51222	-0.39396
ASCL1	-0.21869	0.524769	0.074363	-0.00913	-0.2124	-0.15027	-0.05188	0.389492
ASCL2	0.559955	0.117176	0.266271	0.223945	0.339711	0.053444	0.270888	0.006157
ASCL3	0.124631	0.678253	-0.12898	0.008998	0.148173	0.075302	0.451221	0.095731
ASCL4	0.158898	0.705158	NA	NA	NA	NA	0.000412	-0.16738
ASF1A	0.51562	-0.6058	-0.50579	-0.05332	-0.20888	-0.32154	0.600496	0.35165
ASF1B	0.036321	0.430531	-0.0069	0.353899	0.283267	-0.00619	0.502981	0.347759
ASGR1	0.237494	0.115789	0.367939	-0.18892	0.213581	0.013721	-0.16266	-0.15178
ASGR2	-0.04881	0.114518	-0.14574	-0.33326	-0.11514	0.015731	-0.20416	0.299693
ASH1L	0.297793	0.365602	-0.09928	-0.22295	-0.15593	0.041767	-0.33253	-0.03687
ASH2L	-0.13126	-0.40761	0.101828	-0.01013	0.258311	0.06334	0.398225	0.017642
ASIP	-0.16418	0.554019	0.081849	-0.4154	0.327829	0.331748	-0.35436	0.049807
ASL	0.348269	0.624057	0.231346	0.219822	0.172615	0.079148	-0.01892	0.150618
ASMT	0.287648	0.107925	-0.2976	0.450531	-0.19089	-0.12869	0.174684	-0.02499
ASMTL	0.009568	-0.03932	0.302282	0.210034	0.331816	0.145633	-0.03096	-0.09003
ASNA1	0.356537	0.759228	0.013665	0.176362	0.02294	-0.18673	-0.45882	0.09299
ASNS	0.056394	0.390936	-0.17636	-0.06801	0.309545	-0.46063	-0.2825	-0.22068
ASNSD1	-0.12716	-0.18116	0.289278	-0.37938	0.073128	-0.08078	0.566205	0.427953
ASPA	0.205572	-0.35604	-0.09616	-0.38815	-0.21682	0.013719	-0.28456	0.375541
ASPG	NA	NA	-0.25868	-0.04066	-0.04116	-0.05001	NA	NA
ASPH	-0.46372	-0.00057	0.061398	0.213126	-0.1985	-0.12192	-0.12012	0.149788
ASPHD1	0.356243	0.428859	0.217993	0.169079	0.439189	-0.14908	0.461978	0.408912
ASPHD2	0.168496	0.084799	0.390477	0.279234	-0.09429	-0.07503	0.139756	0.17947

ASPM	-0.32878	-0.09557	-0.39402	0.140005	-0.25228	-0.09054	0.160046	-0.07687
ASPN	-0.16066	-0.17599	0.142768	-0.10971	-0.04025	0.230286	0.016007	0.239753
ASPRV1	NA	NA	-0.38378	-0.17667	0.114164	-0.35387	NA	NA
ASPSCR1	0.229115	0.630133	0.4583	0.086099	0.450536	-0.01388	0.429285	-0.19706
ASRGL1	-0.39397	0.111678	-0.19857	0.426036	-0.13701	-0.07332	-0.0637	-0.24878
ASS1	-0.03678	0.118592	0.019936	0.203334	0.007087	0.017698	-0.32212	-0.22305
ASTE1	0.230001	-0.0744	-0.22619	-0.11024	0.135524	-0.00458	-0.21402	-0.14084
ASTL	-0.18589	0.504589	0.084251	-0.20581	0.083616	-0.20458	0.386754	0.525126
ASTN1	0.00816	0.472271	-0.25079	-0.14856	0.202781	0.207689	NA	NA
ASTN2	-0.05639	0.255315	0.13217	0.157432	0.008665	-0.08759	-0.21589	-0.13931
ASXL1	0.437637	-0.03292	0.098473	0.231687	0.276625	0.395185	0.468546	0.262276
ASXL2	0.359391	-0.56204	0.157034	-0.1716	0.280149	0.356049	-0.10794	-0.27929
ASXL3	NA	NA	0.251684	-0.18056	-0.15221	0.123621	NA	NA
ASZ1	0.496475	0.006312	0.265155	-0.23485	-0.02999	-0.1775	0.063033	-0.08481
ATAD1	-0.182	-0.50241	-0.15232	-0.31099	-0.12137	-0.2953	-0.54235	-0.5405
ATAD2	-0.27183	-0.23143	-0.25028	0.155342	-0.19234	-0.09276	0.51636	0.438526
ATAD2B	NA	NA	-0.21146	-0.10318	0.226682	0.170953	NA	NA
ATAD3A	-0.03689	0.643337	0.432038	0.24808	0.375601	0.22494	0.254357	-0.05438
ATAD3B	-0.28815	0.188254	0.205723	0.165253	0.249165	0.263964	0.507825	0.515568
ATAD3C	NA	NA	0.293072	-0.15106	0.097454	0.012092	0.475332	0.232746
ATAD4	-0.33092	0.015076	0.037362	0.167741	-0.08525	-0.28895	0.201472	0.044218
ATAD5	-0.07705	0.251174	-0.07542	0.120397	0.053621	-0.09903	NA	NA
ATCAY	-0.31516	-0.22818	0.11141	-0.15195	-0.00796	0.105133	-0.15095	-0.24839
ATE1	-0.0912	-0.02302	-0.42802	-0.01696	-0.07038	-0.39527	-0.22111	0.16677
ATF1	0.048307	-0.50781	-0.44935	0.016158	-0.13597	-0.29628	-0.29421	-0.4236
ATF2	-0.2133	-0.27669	0.00806	-0.38156	-0.23026	0.019775	0.586965	0.412909
ATF3	0.163444	-0.08445	-0.09694	-0.07011	-0.13827	-0.17594	-0.05856	-0.13152
ATF4	-0.3295	-0.27661	0.337226	0.089854	0.218512	0.02419	-0.31026	-0.05468
ATF5	0.014956	0.571093	-0.10053	0.455668	0.045837	0.238118	-0.52254	-0.30861
ATF6	-0.0911	0.605449	-0.26502	-0.12748	-0.20432	-0.01573	-0.4276	-0.48249
ATF6B	NA	NA	0.281695	0.022362	0.361213	0.294602	NA	NA
ATF7	-0.23674	0.054707	-0.16695	-0.1654	-0.41189	-0.57058	-0.24914	-0.21977
ATF7IP	0.084393	-0.00513	-0.17984	0.128188	-0.1599	0.139076	-0.21967	-0.024
ATF7IP2	-0.00311	0.205598	0.218206	0.014937	-0.00337	0.511078	0.069818	0.287618
ATG10	-0.05648	0.334052	0.159972	0.006839	-0.12968	-0.30492	0.156564	0.012097
ATG12	0.016477	0.620983	-0.11243	-0.0477	-0.20139	-0.42626	-0.30515	0.1193
ATG16L1	0.166769	-0.08019	0.129742	-0.44308	0.293656	0.442721	0.381536	0.479063
ATG16L2	-0.54557	0.044522	-0.17008	0.431207	0.012299	0.198584	-0.38511	0.230027
ATG2A	NA	NA	0.160331	-0.21606	0.200368	0.176341	NA	NA
ATG2B	NA	NA	-0.30283	0.041142	0.03847	-0.13226	NA	NA
ATG3	-0.01048	-0.39897	-0.33259	0.061966	-0.13344	-0.45431	-0.51084	-0.35358
ATG4A	-0.16804	0.212352	-0.26455	-0.16601	-0.27581	-0.49189	-0.20714	0.37121
ATG4B	-0.18826	0.654077	-0.2065	0.069734	0.031982	0.041246	0.534163	0.496625
ATG4C	-0.23535	-0.56371	-0.12381	0.022628	-0.22396	-0.41834	-0.57407	-0.28747
ATG4D	-0.24802	0.028038	0.077587	-0.06933	0.292484	-0.12592	-0.02561	0.101494
ATG5	0.570969	0.092641	-0.28463	-0.38728	0.096546	-0.48737	-0.09258	-0.22083
ATG7	-0.0181	0.565784	0.063628	-0.42198	0.107746	0.242748	-0.29474	-0.04601
ATG9A	0.256956	0.599711	0.586868	-0.06655	0.489499	0.067728	0.376725	0.272524
ATG9B	0.136513	0.583364	-0.01769	0.186337	0.077604	0.223871	0.479719	0.263636
ATHL1	0.069608	0.162527	-0.02486	0.49756	0.264546	0.410559	0.33482	0.667092
ATIC	0.500965	0.06145	0.181087	0.255149	0.303715	-0.21949	0.612787	0.113181
ATL1	NA	NA	0.099427	-0.10322	-0.11289	-0.45459	NA	NA

ATL2	NA	NA	-0.2184	-0.12844	-0.03745	-0.45539	NA	NA
ATL3	NA	NA	-0.01514	-0.19008	-0.23899	-0.55865	NA	NA
ATM	-0.03701	-0.59854	0.175857	0.027738	0.093555	0.197056	-0.41672	-0.20333
ATMIN	NA	NA	-0.32718	-0.10192	-0.02635	-0.3398	NA	NA
ATN1	0.392763	0.267521	-0.03632	-0.01908	-0.02873	0.17367	0.130846	0.174701
ATOH1	-0.29541	-0.01361	-0.15394	0.157249	-0.0873	-0.18575	0.172476	0.16046
ATOH7	0.298281	0.150346	-0.1334	-0.19226	0.162157	-0.08605	-0.10435	0.461476
ATOH8	0.116557	0.237915	0.104277	-0.0624	-0.04285	0.079112	-0.24212	-0.22497
ATOX1	0.287099	0.411156	0.43003	-0.10495	-0.11383	-0.33317	-0.14432	0.162331
ATP10A	0.348839	0.705214	0.079169	0.030305	-0.08892	0.212018	-0.13732	0.135544
ATP10B	NA	NA	0.238222	0.101345	0.119136	-0.18548	NA	NA
ATP10D	-0.22217	0.088478	0.312981	-0.2419	-0.24603	0.178972	0.146374	-0.0181
ATP11A	0.295022	0.270963	0.064949	0.107396	0.315876	0.35263	-0.35704	-0.2157
ATP11B	0.070145	-0.27954	-0.21221	-0.08586	-0.3148	-0.16423	-0.40089	-0.23825
ATP11C	0.236372	-0.53359	-0.40547	-0.28993	-0.05707	0.010916	-0.3019	0.01824
ATP12A	0.13467	0.129076	0.160878	0.269845	-0.2676	-0.10659	0.302218	-0.13971
ATP13A1	0.336597	0.428996	0.131461	0.320391	0.354605	0.331013	-0.18337	-0.10348
ATP13A2	-0.34839	0.457289	0.329096	0.179716	0.207362	-0.07861	0.329227	0.323794
ATP13A3	NA	NA	-0.2293	-0.12874	-0.00635	0.262274	NA	NA
ATP13A4	-0.02091	-0.22626	-0.02882	-0.13589	-0.20959	-0.23246	0.108275	-0.0891
ATP13A5	-0.0967	0.511483	0.059832	0.033864	-0.11398	-0.33692	-0.41294	0.270535
ATP1A1	-0.03985	-0.26654	0.354057	0.060337	0.513488	0.09822	0.168619	-0.01738
ATP1A2	0.105286	-0.22159	-0.09843	-0.14199	-0.05847	0.085691	-0.27361	-0.37607
ATP1A3	-0.24474	0.663566	0.338721	0.163148	0.436052	-0.17007	0.546274	0.227926
ATP1A4	0.106379	0.518017	0.315195	0.188313	0.454595	-0.20533	-0.33929	-0.14531
ATP1B1	-0.46915	-0.13397	-0.08976	-0.19566	-0.15814	0.102088	0.280086	0.291987
ATP1B2	-0.01796	-0.0462	0.354146	-0.00115	-0.06865	0.335973	0.054896	0.025964
ATP1B3	-0.17483	-0.20336	-0.18271	-0.19972	0.18428	-0.3892	-0.6413	-0.41222
ATP1B4	-0.2825	0.055223	-0.01153	-0.21164	0.020106	-0.26152	-0.08602	0.243885
ATP2A1	-0.06933	0.041892	-0.02041	-0.07952	-0.11359	-0.05228	0.024345	-0.14662
ATP2A2	-0.38828	-0.57402	-0.21181	0.08219	0.054756	0.108114	0.127561	0.326553
ATP2A3	-0.57203	0.516802	0.051181	0.510753	-0.01837	0.225627	-0.14828	-0.33291
ATP2B1	-0.08293	-0.448	-0.19681	-0.11251	0.051467	0.028594	-0.51975	-0.32008
ATP2B2	0.203444	0.61198	0.109244	-0.09759	0.005226	0.012747	0.235064	-0.00962
ATP2B3	0.009725	0.516088	0.132481	-0.37029	0.103564	-0.21339	-0.42328	-0.41779
ATP2B4	-0.19674	-0.22827	-0.00828	-0.05691	-0.09298	0.373103	-0.26343	-0.20404
ATP2C1	0.202115	-0.22603	-0.31856	-0.06452	-0.00283	0.138339	0.115848	0.139771
ATP2C2	-0.10131	0.060333	0.10911	0.047579	0.298967	0.309285	NA	NA
ATP4A	-0.32096	0.097889	-0.15991	0.497543	-0.15586	0.427218	0.092658	0.147376
ATP4B	0.13467	0.3333	-0.25792	-0.30367	0.432628	0.047173	0.507877	0.125868
ATP5A1	-0.15762	-0.17743	0.240767	0.077318	-0.11724	0.026273	0.178564	0.378457
ATP5B	-0.13161	0.312972	-0.12585	0.231689	-0.04754	0.051991	0.025108	0.293615
ATP5C1	-0.2127	-0.52611	-0.31761	-0.2131	-0.24033	-0.50495	0.116333	-0.16981
ATP5D	0.165622	0.498153	0.248915	0.007052	0.059757	-0.01866	0.123463	0.080371
ATP5E	0.183598	0.273175	-0.11784	-0.07482	-0.28825	-0.14921	NA	NA
ATP5F1	-0.18564	-0.61295	0.331907	-0.24489	0.041032	-0.33336	0.080411	-0.35367
ATP5G1	0.114504	0.317726	0.421647	-0.13383	-0.04677	-0.22057	0.225554	-0.17517
ATP5G2	0.130794	0.231695	0.257609	-0.2276	0.087037	0.166993	-0.08436	-0.12154
ATP5G3	0.003077	-0.02792	0.033212	-0.13673	0.000354	-0.34032	0.093437	-0.00127
ATP5H	-0.16315	-0.62875	0.283191	-0.40192	-0.09229	-0.32971	0.038673	-0.06835
ATP5I	0.044402	0.154571	-0.02972	-0.35239	-0.3652	-0.26229	-0.17916	0.032313
ATP5J	0.036125	-0.56397	0.15982	-0.30644	-0.29873	-0.15149	0.018621	-0.03552

ATP5J2	0.054645	-0.73666	-0.14375	0.196895	-0.13192	-0.37799	0.141223	0.110373
ATP5L	-0.02081	-0.20247	-0.06873	-0.12156	-0.32139	-0.17471	-0.46756	-0.6193
ATP5O	0.320929	0.484325	-0.03358	-0.26741	-0.16133	-0.46185	-0.02658	-0.16678
ATP5S	0.282447	-0.46196	-0.13084	-0.14103	-0.07673	-0.20017	-0.36716	-0.33006
ATP5SL	NA	NA	0.076829	0.11134	-0.1021	-0.48487	NA	NA
ATP6AP1	-0.20639	0.546693	0.343964	0.343684	0.502974	0.190796	-0.21138	-0.37478
ATP6AP2	-0.01659	-0.67038	0.064986	-0.07246	-0.16174	-0.18454	-0.29761	-0.43512
ATP6V0A1	-0.26311	0.147792	0.083442	-0.10377	0.000961	0.18498	-0.49102	-0.20797
ATP6V0A2	0.133745	0.130109	-0.16058	-0.11831	0.105174	-0.24231	0.122046	0.33283
ATP6V0A4	0.093412	0.610427	0.352437	-0.18039	0.110053	-0.48226	-0.13354	-0.2062
ATP6V0B	-0.37475	0.29789	0.360673	0.025826	0.165888	-0.13118	-0.15759	-0.2191
ATP6V0C	-0.10126	-0.2028	0.11119	0.005399	0.06389	0.093721	0.381849	-0.00579
ATP6V0D1	-0.06874	0.196249	0.252091	0.194286	0.026659	-0.08349	0.190139	0.139041
ATP6V0D2	0.081209	0.586659	-0.10954	-0.10617	-0.24944	0.02514	-0.17765	-0.2115
ATP6V0E1	-0.11918	-0.64437	-0.10702	-0.26816	-0.26806	-0.26857	-0.45049	-0.14976
ATP6V0E2	0.063593	-0.01756	0.581068	0.016684	0.135682	-0.14966	NA	NA
ATP6V1A	-0.34698	-0.31505	0.136525	-0.13728	-0.09918	0.041055	0.04697	-0.01924
ATP6V1B1	-0.41024	-0.5375	-0.23233	-0.1864	0.203535	0.027066	-0.16579	0.08269
ATP6V1B2	-0.35602	-0.5352	0.199927	0.064111	-0.26975	0.064067	0.077986	0.518753
ATP6V1C1	-0.12709	-0.16822	0.044389	-0.09041	-0.09672	-0.2093	-0.25383	0.30847
ATP6V1C2	0.203482	-0.05746	0.107703	-0.01669	0.256883	-0.23328	0.019673	0.30864
ATP6V1D	-0.34597	-0.09163	-0.3469	-0.30361	-0.61164	-0.34239	-0.07515	0.013047
ATP6V1E1	-0.31011	0.109761	0.151193	-0.12537	0.044032	-0.1616	0.275022	0.056256
ATP6V1E2	0.233892	-0.47806	0.062058	0.338741	0.10716	-0.31084	-0.01038	-0.05472
ATP6V1F	0.328113	0.281746	0.269669	-0.01281	-0.03704	-0.0637	-0.07377	-0.14969
ATP6V1G1	-0.07722	-0.45899	-0.0345	-0.01977	-0.03198	-0.22055	-0.41048	-0.4122
ATP6V1G2	-0.03325	-0.19609	-0.11709	0.047039	-0.41259	0.038539	-0.25128	-0.23092
ATP6V1G3	0.134521	0.073031	0.263504	0.409158	0.179112	0.208168	0.18271	0.219119
ATP6V1H	-0.06328	-0.44952	0.241755	-0.26663	0.018247	-0.53629	0.139032	-0.41737
ATP7A	0.067574	-0.39957	0.056592	-0.38773	0.074914	0.086057	0.243531	0.197063
ATP7B	0.121584	0.316812	0.385547	-0.32815	0.478782	0.153225	0.485799	0.373172
ATP8A1	-0.20015	-0.13613	-0.37353	-0.41089	-0.39567	-0.46447	0.303231	0.387096
ATP8A2	0.094021	0.424582	0.041151	0.043451	-0.24708	0.128938	0.383658	0.141066
ATP8B1	0.106141	-0.10202	0.01548	-0.12346	-0.12775	-0.19339	-0.02106	-0.18145
ATP8B2	-0.45814	-0.2213	0.062096	0.278058	-0.20656	0.534509	-0.54436	-0.40338
ATP8B3	-0.40158	0.116317	-0.27851	0.198342	-0.15403	0.239518	0.087575	0.016885
ATP8B4	-0.33354	-0.05371	-0.1576	-0.06503	-0.21452	-0.13935	-0.51459	0.323949
ATP9A	0.354074	-0.26383	0.070853	-0.36172	0.545641	-0.05422	0.295466	0.0477
ATP9B	-0.11746	0.734946	-0.05884	-0.02544	-0.13134	-0.09435	-0.44847	-0.06479
ATPAF1	-0.02999	-0.02138	0.095437	-0.07176	-0.02152	-0.03994	0.172019	-0.34282
ATPAF2	-0.10007	0.573679	0.165736	0.111472	-0.2634	0.034635	0.253396	0.120984
ATPBD3	0.300524	0.760353	0.148729	-0.11073	0.142434	-0.2832	0.002922	-0.32738
ATPBD4	0.38559	0.522742	0.147654	-0.29716	-0.03662	-0.05891	-0.57189	-0.37041
ATPIF1	-0.22863	-0.33616	-0.07859	-0.09083	0.123438	-0.41732	0.163072	-0.08839
ATR	-0.06754	0.52036	-0.2503	0.096253	0.128103	0.317115	-0.41266	-0.53668
ATRIP	NA	NA	0.12122	0.356525	0.31544	0.412082	NA	NA
ATRN	0.29304	-0.63647	-0.18224	-0.11034	-0.02065	-0.04724	-0.16295	0.247966
ATRNL1	0.181464	-0.02066	-0.01504	0.04408	-0.21415	0.178147	0.013878	-0.29555
ATRX	-0.00959	-0.19884	-0.23476	-0.06442	-0.1399	0.143067	-0.41804	-0.30707
ATXN1	-0.26831	-0.55521	0.253103	-0.23372	-0.14981	-0.23152	-0.37099	-0.30007
ATXN10	0.118138	-0.03902	-0.25338	0.287458	-0.4353	-0.04886	-0.46883	-0.42629
ATXN1L	NA	NA	0.213198	-0.0424	-0.11058	-0.21252	NA	NA

ATXN2	-0.04788	0.327475	-0.16596	-0.34275	0.390169	0.447239	-0.24742	-0.25417
ATXN2L	NA	NA	0.059338	0.225729	0.423154	0.376359	0.342964	0.280222
ATXN3	0.081438	0.538328	-0.30556	-0.08812	-0.16373	-0.20616	-0.14595	0.112632
ATXN3L	NA	NA	0.120403	0.061705	-0.14228	0.173546	NA	NA
ATXN7	0.353871	0.725009	0.201185	-0.2975	0.432529	0.471918	-0.40266	-0.11258
ATXN7L1	NA	NA	-0.14134	-0.05058	-0.04882	-0.17657	NA	NA
ATXN7L2	0.497367	0.592524	0.1935	0.201584	0.250779	-0.04777	0.358584	0.262938
ATXN7L3	NA	NA	0.280971	0.018433	0.212543	-0.13543	NA	NA
ATXN8OS	NA	NA	NA	NA	NA	NA	0.097185	-0.25365
AUH	0.136857	-0.29003	-0.03169	-0.22159	-0.02305	-0.46376	0.371706	0.308749
AUP1	0.030981	-0.47233	0.318165	0.310847	0.137715	-0.01901	-0.1914	-0.24346
AURKA	0.24044	0.099316	-0.04085	0.223565	0.148731	-0.15416	0.4621	0.169733
AURKAIP1	NA	NA	0.279536	0.179008	-0.01288	-0.30485	0.266723	0.110962
AURKB	-0.05651	0.173812	-0.22732	0.371791	0.006392	0.042613	0.611701	0.360825
AURKC	-0.1774	0.153711	-0.01649	-0.21962	0.128243	0.058836	0.102421	0.607472
AUTS2	0.241883	0.219043	-0.12175	0.23313	0.140168	0.409492	-0.28146	-0.21925
AVEN	0.162078	-0.41918	0.234361	0.183313	-0.04943	-0.00186	-0.47379	-0.38365
AVIL	0.100095	-0.26722	-0.1858	-0.18165	-0.01681	-0.04295	-0.13565	0.107189
AVL9	NA	NA	-0.08802	-0.0851	-0.08794	-0.14451	NA	NA
AVP	0.223595	-0.28292	0.041355	-0.12798	0.081863	-0.13712	0.005818	-0.05388
AVPI1	-0.17528	0.597384	-0.10713	-0.0674	0.139737	-0.00837	-0.10255	-0.14129
AVPR1A	0.011094	0.136207	0.166434	0.054953	0.022494	-0.03991	0.154529	-0.01996
AVPR1B	0.281399	0.063501	-0.03023	-0.00148	0.121193	-0.13242	-0.28663	-0.0688
AVPR2	-0.05951	0.265196	0.0106	0.014687	-0.07586	0.193252	-0.20333	-0.24395
AXIN1	0.216355	0.079776	0.275453	0.086683	0.51036	0.349376	0.587669	0.349805
AXIN2	0.303197	0.083623	0.44556	0.035322	0.36676	0.378388	0.483014	0.004939
AXL	-0.0947	0.214918	-0.0204	-0.14072	-0.05666	0.288284	-0.31073	-0.13017
AXUD1	-0.47319	0.232652	0.156546	0.113773	0.118746	-0.00673	-0.38149	-0.08296
AZGP1	0.530156	0.502492	0.44664	0.141841	0.453703	0.032765	0.19502	0.341184
AZI1	-0.08087	0.363725	0.221531	0.362674	0.415286	0.344854	0.07928	-0.13605
AZI2	0.021494	-0.60841	-0.32479	-0.33588	-0.32094	-0.53894	-0.60786	-0.50547
AZIN1	-0.07779	-0.51529	0.049396	-0.16451	-0.24879	-0.17109	0.04407	0.04651
AZU1	-0.06147	-0.11269	-0.10886	-0.10831	-0.13359	-0.11752	0.245888	0.247404
B2M	-0.19605	-0.54814	0.242276	-0.11713	-0.27453	-0.02187	-0.70365	-0.59597
B3GALNT1	-0.23364	-0.37829	0.057952	0.053606	-0.36618	0.067306	-0.0668	-0.04421
B3GALNT2	-0.10477	-0.22446	-0.05376	-0.39842	-0.05637	0.206485	-0.11613	-0.18684
B3GALT1	0.191054	0.290239	0.051396	-0.05811	-0.1334	-0.02453	0.580976	0.400657
B3GALT2	-0.03474	-0.0896	0.202123	-0.19445	-0.04231	-0.09204	-0.14837	-0.06779
B3GALT4	-0.0604	0.325069	0.008812	-0.05369	-0.25303	-0.35683	0.059428	0.066487
B3GALT5	0.061325	0.08703	-0.08074	-0.29354	-0.13183	-0.02021	-0.0191	0.086227
B3GALT6	-0.21563	0.007735	0.151681	0.110419	0.132875	0.18024	0.216617	0.209385
B3GALTL	-0.04592	0.187425	0.242996	0.092163	0.276329	0.065374	NA	NA
B3GAT1	-0.10019	0.738801	-0.10634	0.433557	-0.12546	0.097891	-0.2706	-0.26542
B3GAT2	-0.1573	0.311374	-0.12016	-0.2471	-0.08553	-0.25702	0.35473	0.258882
B3GAT3	-0.03058	0.699164	0.360747	0.192485	0.243112	-0.08549	0.430459	0.457843
B3GNT1	-0.07784	0.246375	-0.10202	0.023407	0.001986	-0.41783	0.412474	0.254661
B3GNT2	-0.41284	-0.15911	-0.07168	-0.12816	-0.09274	-0.47634	NA	NA
B3GNT3	0.552558	0.110295	-0.18408	0.18902	0.315637	-0.24585	-0.27567	-0.21065
B3GNT4	-0.13334	0.610942	-0.34027	0.209807	0.023425	-0.22847	0.084544	-0.09769
B3GNT5	-0.18973	-0.23747	-0.20787	-0.18249	0.124849	-0.22595	0.044596	0.13228
B3GNT6	-0.5555	0.529703	-0.02711	0.219626	0.100805	0.058499	0.348494	0.10953
B3GNT7	0.313514	-0.02676	0.230395	-0.10369	-0.05137	0.093091	0.159715	-0.03437

B3GNT8	0.419386	0.410947	0.036418	-0.29685	0.193237	-0.18701	NA	NA
B3GNT9	NA	NA	0.168153	0.167066	0.09461	-0.07481	NA	NA
B3GNTL1	0.016311	-0.30236	0.314173	0.193513	0.135541	-0.05806	0.322517	0.388047
B4GALNT1	-0.06039	-0.22356	0.310297	-0.05052	-0.02293	0.29155	NA	NA
B4GALNT2	-0.26879	0.188514	-0.13	-0.3927	0.06618	-0.08229	0.357581	0.23637
B4GALNT3	0.183783	-0.10471	0.033114	0.055736	0.255837	0.173082	-0.37962	-0.19823
B4GALNT4	-0.06464	0.134664	-0.01447	0.177156	0.132288	0.291217	0.114587	-0.16242
B4GALT1	-0.25032	0.086755	0.121923	0.167987	0.297222	0.181971	-0.17444	-0.02342
B4GALT2	-0.12473	0.658902	0.240498	0.096892	0.233817	-0.15986	0.073865	0.193529
B4GALT3	-0.00539	0.501651	0.117878	0.321145	-0.10981	0.130339	0.302119	0.006421
B4GALT4	-0.66551	-0.07606	-0.16732	0.178192	0.039428	-0.44446	-0.00688	0.217637
B4GALT5	0.26883	-0.49932	-0.00168	-0.31914	0.073463	0.104003	-0.06933	-0.13902
B4GALT6	0.067063	-0.4901	-0.05964	-0.11258	0.050783	-0.1392	-0.02106	0.036678
B4GALT7	-0.05395	0.464535	0.314398	0.091892	0.20549	0.156038	-0.21194	-0.21107
B9D1	NA	NA	0.067776	0.080792	0.061388	-0.36673	NA	NA
B9D2	NA	NA	-0.45196	0.161471	-0.1579	-0.38361	NA	NA
BAALC	-0.33563	-0.42722	0.253718	-0.04638	0.017329	0.144363	-0.23378	0.304306
BAAT	-0.15908	0.693993	-0.05936	0.100447	-0.03674	-0.21358	0.35993	0.155453
BACE1	0.269955	0.748339	-0.0855	0.0174	-0.17889	0.023067	-0.45404	-0.16836
BACE2	-0.35343	0.474639	0.116139	0.211261	0.211145	0.137725	-0.24746	-0.64573
BACH1	-0.25896	0.193441	-0.25238	-0.46206	-0.22105	0.000324	-0.53714	-0.21492
BACH2	-0.32775	-0.27757	0.14964	0.730153	-0.24793	0.554224	-0.07662	0.073448
BAD	-0.10519	-0.21216	0.118149	-0.25398	-0.1026	-0.29561	-0.06722	-0.15209
BAG1	-0.13336	0.25568	-0.20525	0.278806	0.036941	0.182375	0.05329	-0.04708
BAG2	0.166465	-0.15151	0.177054	-0.0869	-0.16915	-0.41647	NA	NA
BAG3	-0.38711	-0.13468	0.016705	-0.04016	0.315161	0.187365	-0.03981	-0.19007
BAG4	0.065712	0.505167	-0.37298	-0.25988	0.018918	-0.54371	-0.17146	0.027151
BAG5	-0.02953	-0.01267	-0.42036	0.009339	-0.11463	0.075485	0.028843	-0.16226
BAGE	0.253238	0.104702	-0.1223	0.095395	0.183182	-0.01098	-0.36675	-0.0263
BAGE4	0.102015	-0.00344	0.191662	-0.0503	0.003056	-0.22346	-0.2977	-0.17072
BAHCC1	NA	NA	-0.11395	0.046915	-0.08323	-0.06816	NA	NA
BAHD1	0.196055	0.247306	0.049818	-0.03106	0.071703	0.285803	-0.08074	-0.11542
BAI1	0.162934	0.577081	0.172073	-0.02989	0.063833	0.015358	0.318754	0.299186
BAI2	0.130021	0.683521	0.391478	-0.17661	0.184423	0.335187	-0.45702	-0.06733
BAI3	0.175627	-0.18094	0.098878	-0.2033	-0.20264	0.05942	-0.37295	-0.10484
BAIAP2	-0.21283	-0.12615	0.266985	0.018134	0.087718	0.199189	0.208221	-0.29083
BAIAP2L1	0.287412	0.213888	0.196851	0.022052	0.296105	0.117738	0.445672	0.270232
BAIAP2L2	-0.07178	0.225795	-0.05452	-0.02888	0.23898	-0.15108	0.390256	0.27147
BAIAP3	-0.05336	0.045289	0.156178	-0.02085	0.045014	0.279168	0.435808	0.203155
BAK1	-0.20458	0.170149	0.486927	-0.30252	-0.05052	0.061904	-0.44951	-0.34033
BAMBI	-0.08698	-0.04965	0.306936	-0.18791	-0.11039	-0.15077	0.548413	-0.26678
BANF1	-0.16646	-0.48676	-0.15058	0.549968	0.021324	0.049015	0.569626	0.360553
BANF2	NA	NA	-0.16757	-0.17195	0.06772	0.062233	NA	NA
BANK1	0.104499	0.145205	-0.12622	0.788881	-0.13228	0.697163	-0.2657	0.045308
BANP	NA	NA	0.061633	0.186215	0.270485	-0.12974	0.276217	0.451463
BAP1	0.204005	0.71624	0.249471	0.143296	0.463759	0.153862	0.420949	0.227495
BARD1	0.25631	0.738743	-0.09304	-0.02461	0.28062	0.189066	0.378141	0.102171
BARHL1	0.039952	0.199156	0.027888	-0.08399	0.166435	0.235272	-0.02709	-0.27605
BARHL2	-0.35779	0.570479	-0.02848	-0.18354	0.063224	0.013462	-0.37474	-0.19019
BARX1	-0.22886	-0.36115	0.144075	-0.00702	0.083771	0.139437	-0.057	-0.06553
BARX2	-0.38366	0.08313	-0.0488	-0.01801	-0.05763	-0.03763	0.243579	0.41074
BASP1	-0.43131	-0.08781	0.177932	0.783707	0.011087	0.530141	-0.28522	0.204833

BAT1	0.504997	0.283011	-0.3167	0.236912	-0.08413	-0.06564	0.138166	0.192853
BAT2	0.502922	0.157647	0.233174	0.346912	0.355348	0.330466	0.496087	0.271971
BAT2D1	-0.27002	-0.69433	-0.13294	0.121401	-0.18657	0.351118	0.585601	0.450437
BAT3	0.390802	0.734711	0.174441	0.185205	0.147948	0.436886	0.534325	0.222821
BAT4	0.146784	-0.48982	0.083325	-0.17736	-0.02379	-0.34797	-0.32883	0.004601
BAT5	0.49774	-0.03825	0.236748	-0.12717	0.172498	-0.07742	0.658475	0.392588
BATF	-0.0029	0.04853	0.041054	0.551104	-0.00731	0.4861	-0.27502	0.33692
BATF2	0.249041	-0.07054	0.436674	-0.28827	-0.12798	0.045692	-0.0894	0.240636
BATF3	NA	NA	0.236591	0.09707	-0.06897	0.357708	NA	NA
BAX	-0.10812	-0.16867	0.100334	0.349058	-0.0611	0.146797	0.194541	0.126396
BAZ1A	-0.06277	-0.159	-0.31646	-0.16956	0.092906	0.092721	-0.05982	0.220704
BAZ1B	0.369055	0.431933	0.15933	-0.06301	0.146837	0.276192	0.58919	0.45862
BAZ2A	0.142794	0.496309	-0.134	0.173545	0.123295	0.141188	0.206463	0.500357
BAZ2B	0.284122	-0.17371	0.128336	-0.36976	-0.03472	-0.10927	-0.33584	-0.06126
BBC3	0.146913	0.687732	0.118833	0.02208	0.376819	0.048375	-0.42512	-0.12872
BBOX1	-0.17791	-0.0822	-0.08612	-0.18916	-0.30764	-0.11072	-0.17146	-0.45723
BBS1	0.096512	-0.03174	-0.19086	-0.1525	0.07676	0.099502	0.5434	0.229854
BBS10	0.354685	-0.49829	0.100209	-0.18244	0.072175	-0.19474	NA	NA
BBS12	0.000236	-0.43228	-0.1973	-0.41723	-0.53692	-0.46037	0.148684	0.162596
BBS2	0.018384	-0.69125	0.516123	-0.06914	0.03159	0.000267	0.220178	-0.08237
BBS4	-0.12749	0.393228	0.145991	-0.12615	0.010505	0.05313	-0.14624	0.067362
BBS5	0.061706	-0.37462	0.05486	-0.07101	0.379379	0.228632	0.188722	-0.07821
BBS7	-0.12388	-0.49981	-0.3333	-0.20501	-0.11018	-0.43797	-0.12613	-0.23674
BBS9	0.267385	-0.08366	0.041767	-0.1036	-0.13998	-0.03665	-0.27863	-0.39937
BBX	0.121781	-0.41628	0.100301	-0.00811	-0.06035	0.403828	-0.34177	-0.44578
BCAM	0.36437	0.551628	0.09359	-0.01708	-0.02458	0.216837	0.425926	0.02931
BCAN	0.224958	0.784573	0.172555	0.026389	-0.11794	-0.21279	-0.2242	-0.36421
BCAP29	-0.33598	-0.36018	-0.16075	0.124233	-0.16517	-0.22037	-0.2833	-0.13649
BCAP31	0.188768	0.270094	0.053542	0.279359	0.320707	-0.0859	0.257255	0.201275
BCAR1	-0.27199	-0.02199	0.10552	-0.15724	0.165691	0.133488	0.344942	0.197257
BCAR3	-0.31002	-0.4219	0.107055	0.056741	-0.01937	-0.41551	0.182021	0.039053
BCAS1	-0.29402	-0.09358	-0.02556	0.14848	-0.0513	-0.16639	-0.03799	-0.02357
BCAS2	0.261872	0.394884	-0.17985	-0.03642	-0.24255	-0.17752	-0.58733	-0.26155
BCAS3	0.110836	-0.05346	0.427977	-0.21971	0.03608	0.051283	0.623218	0.182231
BCAS4	0.240647	0.412984	0.065499	0.428483	0.17387	0.079487	0.318425	-0.04607
BCAT1	-0.48959	-0.38974	0.039341	-0.00696	-0.10639	0.014933	-0.13393	0.012509
BCAT2	0.135429	0.479381	-0.49464	-0.14276	-0.12598	-0.2278	0.681019	-0.07661
BCCIP	-0.09657	-0.57356	-0.30355	-0.09748	0.13144	-0.08718	0.092261	0.009656
BCDIN3D	NA	NA	-0.08185	-0.04808	-0.2819	-0.29183	NA	NA
BCHE	0.048743	-0.2493	-0.2948	-0.17467	-0.03493	0.046175	-0.28603	-0.34688
BCKDHA	0.019894	0.115367	-0.07603	0.043249	-0.09014	0.194861	0.271994	-0.09868
BCKDHB	0.025957	-0.29826	-0.34478	-0.12708	0.051141	-0.41999	0.373727	0.087208
BCKDK	-0.08409	0.528006	0.290406	-0.02973	0.21865	0.077546	-0.1122	-0.03956
BCL10	-0.28482	-0.13549	-0.23286	-0.19072	0.135841	-0.20209	0.030083	0.176268
BCL11A	0.480983	0.210739	0.1292	0.473176	0.187685	0.40711	0.084867	-0.32376
BCL11B	0.200954	-0.11041	-0.16428	0.343156	0.194335	0.370587	0.174888	0.340933
BCL2	0.105368	-0.3759	0.045084	0.207839	-0.11374	0.415343	-0.26268	0.024523
BCL2A1	-0.59773	-0.50774	-0.14048	0.391557	-0.01531	0.157273	-0.38589	-0.38602
BCL2L1	0.351315	-0.36112	0.293118	0.102138	0.116383	-0.26375	0.547182	0.381058
BCL2L10	0.21477	-0.14233	-0.3151	-0.02961	0.148476	-0.56973	0.095903	0.179121
BCL2L11	-0.06478	-0.05939	-0.12067	-0.0922	-0.08825	-0.20059	0.243791	0.027202
BCL2L12	0.046386	0.178314	-0.27804	0.286973	0.123033	-0.03009	0.277687	-0.13117

BCL2L13	-0.11356	0.096846	-0.03837	-0.31517	-0.15558	-0.28022	0.510518	0.083025
BCL2L14	0.406451	0.288144	-0.1407	-0.13742	-0.25022	-0.37654	-0.16371	0.016669
BCL2L15	NA	NA	0.282522	-0.26244	0.074038	-0.03576	NA	NA
BCL2L2	-0.32506	-0.27739	-0.26396	-0.14616	0.264609	-0.33415	-0.5612	-0.2849
BCL3	0.305861	0.42479	-0.12513	0.028889	0.283972	0.32315	-0.28169	0.235778
BCL6	-0.57111	-0.10761	0.026813	0.416761	0.054151	0.531606	-0.50032	-0.22772
BCL6B	-0.48488	-0.45399	-0.07259	0.175709	0.014274	0.173191	-0.50347	-0.42037
BCL7A	0.100771	0.522664	0.068732	0.546985	0.287896	0.198651	-0.23827	-0.28571
BCL7B	0.278927	0.632279	0.013729	-0.17339	0.006031	-0.0814	-0.35746	-0.09976
BCL7C	NA	NA	0.303087	-0.08783	0.097334	-0.26142	0.448536	0.036656
BCL9	0.46367	0.26303	-0.13338	0.153223	-0.04521	0.166847	0.05407	-0.47894
BCL9L	0.151431	0.243292	0.16567	0.031447	0.143358	-0.22308	-0.05686	0.169418
BCLAF1	0.220629	-0.56636	-0.42719	-0.07485	0.121293	0.088597	0.210236	0.073452
BCMO1	0.246551	0.173723	0.01697	-0.25709	0.02656	-0.45932	-0.33848	0.093194
BCO2	NA	NA	-0.14257	-0.11003	0.006628	-0.0315	NA	NA
BCOR	-0.1615	-0.41422	0.194715	0.329566	0.280672	0.270333	0.273887	-0.27083
BCORL1	0.347513	0.613606	-0.10598	-0.03797	0.026661	0.093809	0.202372	-0.20327
BCORL2	0.177539	0.566363	0.168167	-0.19877	0.169219	0.190566	0.137069	-0.21814
BCR	0.037664	0.652746	0.252423	0.213178	0.476345	0.251687	0.449647	0.288808
BCS1L	0.550996	0.378177	0.002777	0.3813	0.170965	0.186238	0.63616	0.445944
BDH1	0.38097	0.228164	0.014116	0.211424	0.270146	-0.04656	NA	NA
BDH2	0.070564	-0.02124	0.197376	-0.40483	-0.14158	-0.14106	-0.31843	-0.02365
BDKRB1	-0.12082	0.009153	-0.30082	-0.2514	0.13086	-0.11006	-0.1145	0.271734
BDKRB2	0.03343	-0.17449	-0.02738	-0.2249	0.14856	-0.00633	-0.2855	0.127753
BDNF	-0.10235	0.691039	0.085517	0.074896	-0.03136	-0.16916	0.166149	0.133653
BDP1	0.411212	0.581888	-0.06028	-0.01452	-0.08754	0.126588	0.023356	-0.17268
BECN1	0.280656	-0.48402	0.314768	-0.06997	0.060239	-0.48982	-0.29895	-0.54084
BEGAIN	NA	NA	-0.3906	0.315386	-0.08418	-0.07728	NA	NA
BEND2	NA	NA	0.269061	0.261701	0.213327	-0.1152	NA	NA
BEND3	NA	NA	-0.46727	0.162005	0.105958	-0.12029	NA	NA
BEND4	NA	NA	0.075909	0.199949	-0.08257	-0.07404	NA	NA
BEND5	NA	NA	-0.00984	0.220246	-0.32049	0.064223	NA	NA
BEND6	NA	NA	0.0432	0.018915	-0.33892	0.206333	NA	NA
BEND7	NA	NA	0.328762	-0.21444	0.379226	-0.09102	NA	NA
BEST1	-0.51432	0.412875	-0.0446	-0.1012	-0.035	0.323925	NA	NA
BEST2	-0.09946	-0.53354	-0.11914	0.187411	-0.13121	-0.25066	NA	NA
BEST3	0.187919	0.611332	0.279872	-0.21238	0.081898	0.02442	NA	NA
BEST4	0.238044	0.049182	0.18572	-0.207	0.053656	-0.32428	NA	NA
BET1	0.222586	-0.60021	-0.03878	0.018169	-0.1272	-0.52391	0.06606	0.10217
BET1L	-0.09401	-0.03844	-0.01462	-0.16201	-0.11022	-0.27996	0.312799	0.186923
BET3L	NA	NA	-0.16749	-0.38251	0.263428	0.27386	NA	NA
BEX1	-0.13041	-0.4319	-0.2568	0.169171	-0.1193	-0.31302	0.082068	-0.22383
BEX2	NA	NA	-0.15961	0.46204	-0.23005	0.287018	-0.01233	0.226754
BEX4	NA	NA	-0.10702	0.137369	-0.33455	0.127868	NA	NA
BEX5	NA	NA	-0.0453	0.087649	-0.15912	0.182857	NA	NA
BFAR	0.140989	-0.16461	-0.00291	-0.27923	0.017031	-0.18093	0.08536	-0.19555
BFSP1	-0.01014	-0.35788	-0.30753	-0.10918	0.136507	-0.43521	0.08107	-0.22995
BFSP2	0.037746	0.237602	-0.06472	0.76926	-0.31302	-0.2079	-0.0475	-0.21887
BGLAP	-0.05423	0.102389	0.013618	0.217404	-0.18262	-0.01019	0.260047	0.008749
BGN	-0.1548	-0.06956	0.192289	0.088761	-0.07692	-0.1571	-0.09848	-0.25591
BHLHA15	NA	NA	0.012798	-0.0468	0.191129	-0.09353	NA	NA
BHLHB9	0.31725	-0.1074	-0.15355	-0.1363	-0.1061	-0.16155	-0.13435	-0.2172

BHLHE22	NA	NA	0.04216	0.093838	-0.11159	0.299864	NA	NA
BHLHE23	NA	NA	-0.13989	0.044407	-0.02974	0.148016	NA	NA
BHLHE40	NA	NA	0.149755	-0.01188	0.097399	0.387787	NA	NA
BHLHE41	NA	NA	0.092446	-0.07616	-0.20394	0.157622	NA	NA
BHMT	-0.19825	-0.35459	0.139317	-0.06022	0.107267	0.052506	0.185753	-0.11547
BHMT2	-0.08805	-0.31069	-0.04494	-0.10688	0.108026	-0.14425	-0.24444	-0.0554
BICC1	NA	NA	-0.05653	-0.3221	-0.23221	0.188061	-0.08976	0.135094
BICD1	0.125675	-0.09322	0.122137	0.482933	0.208059	0.192389	0.007339	0.100365
BICD2	-0.0997	0.106424	-0.00731	-0.04276	0.005156	0.281165	-0.13104	-0.02705
BID	-0.18875	-0.16985	-0.20917	0.192307	0.063995	-0.12576	0.178129	-0.18769
BIK	-0.08357	0.009363	-0.22901	0.025194	-0.15899	-0.13394	0.271331	0.072204
BIN1	0.469716	-0.001	0.282179	0.334354	0.413421	0.251037	0.237741	0.336741
BIN2	-0.4898	-0.16145	0.059147	0.2632	-0.11138	0.095834	-0.40761	-0.07945
BIN3	-0.12754	0.343163	0.248686	-0.03347	0.054139	0.093597	-0.33864	-0.07965
BIRC2	-0.54326	-0.18341	-0.21169	-0.09567	-0.47255	-0.15921	-0.4374	-0.1948
BIRC3	-0.14966	0.000281	-0.05926	0.38926	-0.16714	0.190006	-0.52949	0.074808
BIRC5	0.079928	0.142361	-0.1776	0.317235	0.063108	-0.09119	0.308488	-0.12348
BIRC6	0.473935	0.135899	-0.21441	0.067935	0.036442	0.153384	-0.08424	-0.16069
BIRC7	0.154146	0.390486	-0.13171	-0.06472	0.265724	-0.15905	0.313828	-0.06039
BIRC8	0.328249	0.642542	0.152926	0.299731	0.153764	-0.11837	0.706722	0.378426
BIVM	0.234523	-0.63574	0.434016	-0.06892	0.380268	-0.21321	0.55771	0.504873
BLCAP	0.11686	-0.01195	0.356836	0.205647	0.433549	0.015685	0.190075	0.045771
BLID	NA	NA	0.102562	-0.52939	0.160074	0.168176	NA	NA
BLK	-0.15844	0.451418	0.155808	0.805327	-0.10393	0.623888	-0.30308	0.056644
BLM	0.333428	0.034903	-0.32895	0.289773	0.232016	0.05411	0.320938	0.344584
BLMH	0.235776	0.191225	0.161824	0.487555	0.06556	0.253079	-0.51858	-0.42972
BLNK	-0.26288	-0.16776	0.009695	0.236188	-0.14594	0.191377	-0.40349	-0.53011
BLOC1S1	0.03221	0.293834	0.014121	-0.14196	-0.12583	-0.53332	-0.44444	-0.39457
BLOC1S2	-0.13574	0.061515	-0.00342	0.00687	-0.1532	0.044222	0.009115	0.10227
BLOC1S3	0.159624	0.325126	NA	NA	NA	NA	0.278488	0.215942
BLVRA	-0.43238	-0.20417	0.292693	0.066085	-0.18508	0.27887	-0.69982	-0.50227
BLVRB	-0.16417	0.28448	0.095892	-0.32878	-0.16785	-0.26973	-0.55476	-0.43145
BLZF1	-0.20198	-0.19458	-0.31836	-0.41675	-0.30485	-0.51353	-0.54004	-0.5412
BMF	0.051556	0.419865	0.311591	0.06524	0.053217	0.255431	-0.12921	0.312198
BMI1	0.022707	-0.25982	-0.32433	0.052492	0.053178	-0.06091	NA	NA
BMP1	-0.27533	-0.67358	0.28194	-0.19489	0.071096	-0.01027	-0.21442	-0.09771
BMP10	0.142561	0.211616	0.131519	-0.1664	-0.35927	-0.10059	0.211373	-0.1614
BMP15	0.315421	0.735934	0.341586	0.140196	-0.12426	0.087919	-0.14155	-0.00533
BMP2	-0.10009	0.070123	-0.27909	0.168388	-0.11966	0.118522	-0.12676	-0.06121
BMP2K	-0.44469	-0.31324	-0.01089	-0.20302	-0.43942	0.105421	0.027184	0.227795
BMP3	0.251722	-0.24761	-0.00313	-0.4953	-0.37742	-0.40543	-0.33356	-0.32251
BMP4	-0.10424	0.207557	0.473612	0.043465	0.117602	0.114641	0.463253	0.04184
BMP5	0.168218	-0.13894	-0.15597	-0.31556	0.047573	0.042898	-0.08367	0.04813
BMP6	-0.41255	-0.21481	-0.20655	0.016103	-0.16161	0.107437	-0.36681	-0.09277
BMP7	0.066524	0.447802	0.416469	0.587996	0.083771	-0.01198	-0.04433	-0.19876
BMP8A	0.281152	0.255478	-0.0422	-0.3048	0.001788	-0.19111	0.09366	0.294916
BMP8B	-0.34034	-0.2482	0.165624	-0.15028	0.121024	-0.04162	-0.71719	-0.5889
BMPER	0.098062	0.683638	0.348421	0.100162	0.035115	-0.19944	-0.20256	0.078122
BMPR1A	NA	NA	-0.08569	-0.46397	0.068703	-0.31296	-0.07369	-0.11703
BMPR1B	-0.01222	0.470635	0.110677	-0.23273	-0.16364	-0.09727	-0.12328	-0.12275
BMPR2	-0.08083	-0.73931	0.034841	-0.53397	-0.14873	-0.32693	-0.08623	-0.21163
BMS1	NA	NA	-0.06091	0.225715	0.236273	0.242775	NA	NA

BMX	-0.20852	0.114304	-0.20959	-0.09911	-0.45657	0.139042	-0.2101	-0.07618
BNC1	0.242319	0.727434	-0.09342	0.264687	-0.09076	0.119431	-0.2071	-0.04825
BNC2	-0.39037	-0.40337	0.090386	-0.0614	-0.01215	0.024523	-0.26937	-0.09796
BNIP1	0.152713	-0.24581	0.083451	-0.1218	-0.12386	-0.14759	0.0479	0.341273
BNIP2	-0.00862	-0.48991	-0.05245	-0.28646	-0.2984	0.200389	0.144096	0.247327
BNIP3	-0.04174	-0.16183	-0.15138	-0.10645	0.112268	0.072174	-0.40163	0.048747
BNIP3L	-0.48177	-0.61763	-0.02018	-0.30211	-0.36525	-0.07774	-0.46566	-0.30047
BNIPL	-0.13639	0.352122	-0.04509	-0.02656	-0.25753	0.108549	-0.22512	-0.04691
BOC	-0.10039	-0.12546	0.327588	-0.02698	-0.05837	0.086489	-0.25164	-0.00022
BOK	-0.1321	0.036751	0.293941	0.004688	0.253887	0.203282	-0.37288	-0.19865
BOLA1	-0.09662	0.309051	0.145796	-0.08918	-0.09919	-0.62357	-0.39802	-0.44666
BOLA2	-0.2122	-0.37699	NA	NA	NA	NA	0.152973	0.006003
BOLA3	0.352557	0.404872	-0.13397	-0.10956	-0.2902	-0.45	-0.08347	-0.24405
BOLL	0.217505	0.628064	-0.39273	0.120533	0.288946	-0.41784	0.492402	0.421056
BOP1	0.1491	0.506348	0.366894	0.24787	0.239595	0.245024	0.137962	-0.25706
BPESC1	0.316089	0.699539	-0.06908	0.136379	-0.11436	0.248572	NA	NA
BPGM	-0.33528	-0.64306	-0.12895	-0.161	-0.4994	-0.47033	-0.05251	0.130888
BPHL	-0.04059	0.021063	0.150775	-0.2953	-0.03884	-0.05409	0.475024	0.459665
BPI	-0.07673	0.492246	0.245692	-0.09971	-0.17023	-0.0352	0.47396	0.431431
BPIL1	0.21063	0.323833	0.073088	0.082245	0.116131	-0.21586	-0.03549	-0.05493
BPIL2	0.342063	0.390531	-0.29918	-0.03572	-0.16825	-0.08868	-0.02143	-0.27839
BPIL3	0.002532	0.508888	0.079979	-0.45259	0.35938	-0.29527	0.048177	-0.16402
BPNT1	0.036	0.147159	0.199276	-0.10283	-0.00855	-0.01039	-0.08578	0.333495
BPTF	0.300172	-0.44215	-0.18664	0.322899	-0.20311	0.217964	NA	NA
BRAF	0.007298	0.018166	-0.02611	-0.48551	-0.03076	-0.23406	0.313652	0.021205
BRAP	0.443114	0.36355	0.102061	-0.23816	0.084531	-0.19697	-0.08578	-0.04819
BRCA1	0.232231	0.663606	-0.05945	0.024259	0.241662	-0.05857	-0.0878	-0.21436
BRCA2	0.068312	-0.2103	0.124876	0.095026	0.211786	-0.08617	0.068607	-0.03642
BRCC3	-0.2528	-0.11536	-0.30841	-0.0069	0.198341	-0.19198	NA	NA
BRD1	-0.42112	-0.67573	-0.26713	0.098545	0.337253	0.043693	0.478867	0.307452
BRD2	0.420339	0.461394	-0.39964	0.395007	0.015264	0.044497	-0.36901	-0.00576
BRD3	0.051653	-0.23933	0.169557	0.253879	0.258566	0.378879	-0.0233	-0.16462
BRD4	0.32219	-0.03024	0.262653	0.025297	0.372333	0.316643	-0.37563	0.153008
BRD7	-0.05544	-0.72917	-0.06829	0.100655	0.172729	-0.23559	-0.02012	-0.13047
BRD7P3	NA	NA	0.068548	0.141253	0.338855	0.089742	NA	NA
BRD8	-0.11599	-0.66492	-0.06552	0.210212	0.109903	-0.05206	-0.11817	-0.19853
BRD9	-0.06805	0.484449	-0.00969	0.240934	0.305811	0.442411	0.408774	0.196579
BRDT	0.249324	0.703939	-0.21047	0.16516	-0.07586	-0.14297	0.075101	0.15764
BRE	0.198238	-0.10861	0.383915	0.171936	0.08767	-0.3366	0.557126	0.297803
BRF1	-0.03089	0.009142	-0.03539	0.030583	0.147821	0.108308	0.323265	0.464861
BRF2	-0.01881	-0.14183	0.366883	0.099397	0.26951	-0.14304	0.589917	0.345165
BRI3	0.112208	-0.3346	0.288903	-0.26682	0.103122	-0.21627	-0.0691	-0.10353
BRI3BP	-0.14574	0.071771	0.196857	0.176178	0.065523	-0.12829	-0.10089	0.06747
BRIP1	0.010618	-0.21514	-0.1579	0.127892	-0.03253	0.167871	0.255767	-0.35523
BRMS1	0.177168	-0.03395	0.151809	0.242273	0.162315	-0.01339	0.285543	0.252
BRMS1L	0.224535	-0.53041	-0.36603	-0.07079	-0.38156	-0.13449	-0.20552	-0.37029
BRP44	0.080794	-0.07401	-0.29139	-0.22816	-0.06618	-0.52093	0.261277	-0.08553
BRP44L	-0.08184	-0.13811	-0.16484	-0.16503	-0.0243	-0.33687	-0.33895	-0.5811
BRPF1	0.287149	0.738629	0.005496	-0.04223	0.391192	0.364431	-0.13636	-0.14561
BRPF3	0.039298	-0.14499	-0.44596	0.167464	0.210584	0.005297	-0.22013	-0.31538
BRS3	0.052943	0.473585	-0.2725	-0.07106	0.127293	-0.06785	-0.36234	-0.30844
BRSK1	0.245341	0.758224	0.141665	0.120273	-0.14201	-0.14534	-0.06109	0.316326

BRSK2	0.301397	-0.22475	-0.14896	-0.32706	0.050197	-0.09012	-0.05586	-0.11836
BRUNOL5	0.13906	0.393748	-0.10205	-0.10603	0.060119	0.113859	0.444105	0.349404
BRUNOL6	0.236574	0.356522	0.079791	0.032896	-0.2349	0.273438	-0.38631	-0.0098
BRWD1	-0.08244	-0.3353	-0.12331	-0.21233	-0.09524	0.063826	-0.08655	-0.3527
BRWD2	-0.04764	-0.69128	-0.25147	-0.10474	-0.07422	-0.03508	-0.42915	-0.11104
BRWD3	-0.18965	0.154692	-0.05301	-0.16007	0.133463	0.403399	-0.2309	-0.04257
BSCL2	0.025858	0.732883	0.288699	-0.14493	-0.04305	-0.35219	-0.14291	-0.36525
BSDC1	-0.53233	-0.12082	0.334268	0.04101	-0.00087	-0.15986	-0.04263	0.068032
BSG	0.121362	0.514478	0.074656	-0.0725	0.312143	-0.11342	0.324526	0.063156
BSN	0.259366	0.177076	-0.18089	0.311889	0.396338	-0.04732	0.124524	-0.14651
BSND	-0.28059	0.302819	0.047335	-0.06244	-0.17946	0.089864	0.114032	0.311002
BSPRY	-0.11901	0.353928	0.171949	-0.04355	0.05456	-0.41016	NA	NA
BST1	-0.41459	-0.15805	0.366453	-0.08906	-0.12719	-0.2137	-0.42824	0.402588
BST2	-0.34465	0.135355	-0.00274	0.321543	-0.28158	0.290525	-0.45264	-0.25806
BTAF1	-0.2358	-0.58008	-0.19747	0.060968	0.106427	0.327808	-0.2518	0.291543
BTBD1	-0.16499	-0.39449	0.121762	-0.11908	0.066345	-0.32128	0.026842	0.046999
BTBD10	-0.29872	-0.72084	-0.23149	-0.36501	-0.10613	-0.36948	-0.12354	-0.40042
BTBD11	0.253196	0.587868	0.226024	0.620159	0.180359	0.434836	0.069739	0.495472
BTBD12	-0.06419	0.60973	0.050596	0.143704	0.22906	0.433006	-0.45013	-0.30855
BTBD16	0.226911	-0.45595	-0.21207	-0.21994	0.315471	-0.37398	NA	NA
BTBD2	0.062094	0.505632	0.190941	0.163238	0.064718	-0.00591	NA	NA
BTBD3	-0.12741	-0.30419	-0.52595	-0.51499	-0.27401	-0.44409	-0.14366	-0.35997
BTBD6	-0.15401	-0.3072	-0.20587	-0.04538	0.242169	-0.15186	-0.27247	-0.36997
BTBD7	-0.13627	-0.00739	-0.33183	-0.35358	-0.25824	-0.18183	-0.03073	0.29854
BTBD8	0.212972	0.001636	0.254405	-0.1481	-0.33269	-0.05209	-0.435	0.027274
BTBD9	0.174255	0.563648	0.462205	0.099252	0.248157	0.124973	-0.29844	-0.33738
BTC	0.068867	-0.29919	-0.05248	-0.33129	-0.16022	-0.36906	-0.05347	-0.15639
BTD	0.340346	0.335218	0.054101	-0.20056	0.094206	-0.18263	0.3597	0.436674
BTF3	0.157344	-0.70778	-0.02348	-0.24314	-0.08063	-0.27388	0.313695	-0.04785
BTF3L4	0.258485	-0.20919	NA	NA	NA	NA	0.112798	-0.08759
BTG1	-0.14107	-0.32157	-0.27262	0.139258	-0.0978	0.058743	-0.32842	-0.38448
BTG2	0.003266	0.038973	0.234163	0.337297	0.014086	0.076247	0.262546	-0.29709
BTG3	-0.16904	-0.62983	-0.22339	-0.19266	-0.26769	-0.5205	-0.48152	-0.27648
BTG4	-0.00447	0.497626	-0.15376	-0.01961	0.164591	0.155313	0.185322	-0.06908
BTK	-0.63068	-0.0152	0.133649	0.631438	-0.14002	0.535265	-0.67377	-0.21704
BTLA	0.129754	0.736912	0.113916	0.396978	-0.38768	0.08295	-0.37001	-0.09018
BTN1A1	0.271459	0.383958	0.154693	0.238369	-0.0475	0.10526	0.477116	0.354019
BTN2A1	-0.01121	-0.61259	-0.32138	0.085308	-0.21446	-0.10669	-0.26016	0.08503
BTN2A2	-0.05526	0.1076	0.319113	0.37898	-0.12635	0.200546	-0.05575	0.238716
BTN2A3	0.404101	0.670667	0.299228	-0.37514	0.122481	-0.13534	0.076786	0.396241
BTN3A1	0.108077	0.371217	0.38031	0.187132	-0.1413	-0.08298	-0.15235	0.570086
BTN3A2	0.14306	-0.23365	0.127316	-0.14248	-0.03735	0.079728	-0.18729	0.000647
BTN3A3	-0.17943	-0.48246	0.127089	0.060429	-0.27701	-0.1262	-0.5085	-0.18344
BTNL2	0.204122	0.50513	-0.06216	-0.46421	0.109521	0.064368	-0.13522	-0.22966
BTNL3	0.097731	0.127661	0.065011	-0.05773	-0.01365	-0.46347	0.000327	0.117928
BTNL8	0.094042	-0.05291	0.111464	0.083772	-0.07023	-0.09888	-0.11186	-0.36474
BTNL9	-0.25009	-0.26718	0.034566	0.336384	0.048768	0.328831	0.297677	0.319148
BTRC	0.21888	-0.05562	0.116726	-0.13902	0.333961	-0.01005	0.167307	-0.11954
BUB1	-0.04552	-0.04561	-0.4436	0.265706	-0.17312	-0.1608	0.423371	0.030924
BUB1B	-0.20664	-0.25217	-0.16594	0.176121	-0.21782	-0.06976	-0.0369	-0.32764
BUB3	-0.35371	-0.67069	-0.30276	0.050754	-0.07177	0.034433	0.005588	-0.23778
BUD13	-0.06413	-0.00436	-0.10539	0.137151	0.031442	-0.16473	NA	NA

BUD31	0.328411	-0.1957	-0.27598	0.010367	-0.02764	-0.42779	NA	NA
BVES	0.096244	-0.07201	0.130273	-0.13093	-0.06419	0.267967	-0.17684	-0.20441
BXDC1	0.455415	-0.26214	-0.16808	-0.13434	0.261489	0.276435	0.1565	-0.16068
BXDC2	-0.09023	-0.47856	-0.21714	-0.0366	0.206504	0.274195	0.257585	-0.27165
BXDC5	0.098202	-0.4147	-0.09542	-0.11665	-0.04926	-0.44638	-0.03044	-0.2763
BYSL	0.483256	0.148746	-0.27558	0.181271	0.183468	0.203923	0.620372	0.193014
BZRAP1	0.065339	0.143747	0.037907	0.234745	0.056884	0.266317	0.143965	0.41177
BZW1	0.501294	0.644436	-0.33329	0.041493	-0.14645	-0.0808	-0.58837	-0.51804
BZW2	0.400017	-0.36457	-0.345	-0.07785	-0.09878	-0.19947	0.383577	-0.16204
C10orf10	NA	NA	0.077154	-0.0148	0.035616	-0.12151	-0.09462	0.101029
C10orf107	NA	NA	-0.04805	-0.101	-0.3429	-0.18738	-0.11686	-0.50845
C10orf11	NA	NA	-0.0844	0.170924	0.093264	-0.06736	-0.09746	-0.41457
C10orf111	NA	NA	-0.20898	0.037243	-0.25241	-0.06077	0.392078	0.491199
C10orf113	NA	NA	NA	NA	NA	NA	0.439933	0.04573
C10orf114	NA	NA	0.004037	0.064251	-0.05098	-0.24601	-0.19781	-0.10752
C10orf116	NA	NA	0.088727	-0.25724	0.019934	-0.05889	-0.01433	0.355711
C10orf118	NA	NA	-0.16262	-0.0356	0.124365	-0.28126	-0.26251	0.084186
C10orf119	NA	NA	-0.25445	-0.06831	0.151931	-0.01757	-0.08542	0.143147
C10orf12	NA	NA	-0.43698	-0.19256	0.204781	-0.06057	-0.25703	0.066564
C10orf120	NA	NA	NA	NA	NA	NA	-0.11496	-0.24022
C10orf122	NA	NA	0.14826	-0.2188	0.13714	-0.02852	NA	NA
C10orf125	NA	NA	0.23553	-0.14425	-0.08403	-0.41974	-0.23571	0.504815
C10orf128	NA	NA	0.183101	0.239046	-0.3317	0.203848	NA	NA
C10orf129	NA	NA	NA	NA	NA	NA	-0.42969	-0.39101
C10orf137	NA	NA	-0.2567	-0.08305	0.169429	0.264764	-0.46671	-0.14657
C10orf2	NA	NA	-0.19671	0.16908	0.327798	0.316912	NA	NA
C10orf25	NA	NA	-0.37841	0.233948	0.000416	-0.24865	-0.14896	0.064104
C10orf26	NA	NA	0.011013	0.09024	0.314464	-0.05873	-0.12634	0.379393
C10orf27	NA	NA	0.246325	-0.01807	0.064182	-0.14185	0.590948	0.179042
C10orf28	NA	NA	-0.05126	-0.36213	0.251309	-0.04109	-0.25466	0.026444
C10orf32	NA	NA	-0.17739	-0.48537	0.026711	0.016573	0.174881	0.330056
C10orf35	NA	NA	0.18385	0.173566	0.211662	-0.18596	0.168229	-0.05255
C10orf4	NA	NA	-0.2109	-0.15033	0.014749	-0.36527	0.334276	0.152305
C10orf46	NA	NA	-0.26893	-0.21087	0.015268	-0.15118	0.330416	0.347936
C10orf47	NA	NA	-0.01792	0.003519	0.205121	-0.08148	0.315834	-0.09549
C10orf53	NA	NA	-0.0229	-0.08685	0.104564	-0.38041	0.205971	-0.22919
C10orf54	NA	NA	0.495339	0.031404	-0.02203	-0.0485	-0.64523	0.148079
C10orf55	NA	NA	NA	NA	NA	NA	0.021413	-0.57563
C10orf57	NA	NA	-0.18457	-0.25561	-0.17535	-0.44393	-0.0467	-0.18074
C10orf58	NA	NA	0.281743	-0.24281	0.275598	-0.2162	-0.48821	-0.51829
C10orf62	NA	NA	-0.22536	-0.02397	-0.04887	0.306924	0.018391	0.462868
C10orf67	NA	NA	0.376373	0.050427	0.131639	0.257569	-0.15797	-0.00442
C10orf68	NA	NA	-0.06639	-0.01746	-0.50092	-0.05255	-0.03054	0.404184
C10orf71	NA	NA	NA	NA	NA	NA	0.517262	0.277822
C10orf72	NA	NA	0.035348	0.035917	-0.01889	0.242623	-0.08444	0.251063
C10orf76	NA	NA	0.007615	-0.34002	0.188056	-0.01697	0.362063	0.387701
C10orf78	NA	NA	-0.0254	0.02975	0.322239	-0.05396	-0.21772	-0.60144
C10orf79	NA	NA	-0.03256	0.103181	-0.18782	0.095387	0.103746	0.118392
C10orf81	NA	NA	0.106823	0.127318	0.299531	-0.08592	-0.03375	0.190957
C10orf82	NA	NA	0.052603	-0.11004	-0.08452	-0.11532	-0.29469	0.005029
C10orf84	NA	NA	-0.22372	-0.15211	-0.01412	-0.37423	0.145915	-0.24303
C10orf88	NA	NA	-0.36021	0.205179	0.056564	-0.42806	-0.36473	-0.28818

C10orf90	NA	NA	0.239953	-0.22665	0.086203	0.298148	0.133074	0.004926
C10orf91	NA	NA	0.039515	-0.20579	-0.00412	-0.00087	0.027944	0.083607
C10orf92	NA	NA	-0.09909	0.138223	-0.02463	0.08625	0.520378	0.546541
C10orf93	NA	NA	0.085567	-0.21781	0.266891	0.387095	-0.13789	0.197911
C10orf95	NA	NA	-0.31692	0.023117	0.113803	-0.35299	0.038348	0.13118
C10orf96	NA	NA	0.030222	0.05044	-0.1573	-0.19992	-0.05353	-0.18732
C10orf99	NA	NA	NA	NA	NA	NA	-0.06578	-0.09848
C11orf1	NA	NA	0.136668	-0.33933	-0.0155	-0.29805	0.409355	0.042764
C11orf10	NA	NA	0.263585	-0.27521	-0.14084	-0.13903	0.257134	-0.0092
C11orf16	NA	NA	-0.14081	-0.18207	0.220362	-0.24725	-0.22456	0.045392
C11orf17	NA	NA	0.060645	-0.03939	0.174118	-0.29574	-0.41348	-0.04289
C11orf2	NA	NA	0.297099	0.069222	0.287729	0.184837	0.088912	-0.06051
C11orf20	NA	NA	-0.32914	0.114411	-0.30149	-0.222	NA	NA
C11orf21	NA	NA	0.095684	0.408067	-0.0246	0.448793	NA	NA
C11orf24	NA	NA	0.000147	0.200904	-0.01771	-0.07034	-0.52291	-0.058
C11orf30	NA	NA	0.254909	-0.11704	0.02074	0.057055	0.517794	0.365088
C11orf31	NA	NA	-0.34076	0.366442	-0.01963	-0.12879	0.218403	0.605164
C11orf35	NA	NA	0.156445	-0.25394	0.285993	0.040084	0.361409	0.392328
C11orf36	NA	NA	0.190306	0.042836	-0.31068	0.253417	0.455556	0.272787
C11orf40	NA	NA	0.032473	-0.03354	0.020003	0.1024	-0.61477	0.134205
C11orf41	NA	NA	-0.24489	0.17811	-0.13327	-0.0116	NA	NA
C11orf42	NA	NA	0.061274	-0.10806	0.130731	-0.07182	0.160055	-0.04975
C11orf45	NA	NA	0.004306	0.145472	-0.3197	0.21334	0.107922	0.132385
C11orf46	NA	NA	-0.25068	-0.1922	-0.18252	-0.02927	-0.15111	-0.09893
C11orf48	NA	NA	-0.1695	0.331768	-0.18177	-0.31206	0.020538	-0.03417
C11orf49	NA	NA	-0.04622	0.065411	0.028641	0.050838	0.536682	-0.0053
C11orf51	NA	NA	-0.17092	0.060497	-0.11996	-0.41703	-0.22305	-0.33181
C11orf52	NA	NA	0.214511	-0.12329	-0.00899	-0.45199	0.247742	0.405615
C11orf53	NA	NA	NA	NA	NA	NA	0.061501	0.159561
C11orf54	NA	NA	0.037392	-0.30255	0.062474	-0.12529	0.429024	0.215705
C11orf57	NA	NA	-0.1631	-0.06232	-0.22141	-0.11446	NA	NA
C11orf58	NA	NA	-0.18304	-0.29611	0.136754	0.077586	NA	NA
C11orf61	NA	NA	-0.3171	-0.06798	-0.1591	-0.11989	-0.35392	-0.14746
C11orf63	NA	NA	-0.00858	-0.15181	-0.32677	0.089279	-0.62418	-0.57473
C11orf65	NA	NA	-0.07247	-0.27747	-0.16493	-0.38115	-0.11509	-0.28145
C11orf67	NA	NA	0.00944	-0.44768	-0.14136	-0.05631	NA	NA
C11orf68	NA	NA	0.430342	0.095635	0.19836	-0.08096	NA	NA
C11orf70	NA	NA	-0.10277	-0.26596	-0.2492	-0.21797	0.103249	-0.16913
C11orf71	NA	NA	0.154369	-0.23837	-0.15588	-0.39082	0.272469	0.631044
C11orf73	NA	NA	0.038554	-0.1739	-0.06051	-0.11313	NA	NA
C11orf74	NA	NA	0.011146	-0.36849	-0.21692	-0.44079	NA	NA
C11orf75	NA	NA	-0.16006	0.164955	-0.03307	-0.0582	NA	NA
C11orf80	NA	NA	-0.00026	-0.27356	0.047361	-0.23376	NA	NA
C11orf82	NA	NA	-0.37271	0.136525	-0.06153	0.049139	NA	NA
C11orf83	NA	NA	-0.34591	0.068758	-0.32462	-0.30352	NA	NA
C11orf84	NA	NA	0.126692	0.398278	0.152482	-0.04078	NA	NA
C11orf85	NA	NA	0.214038	0.300674	-0.05943	0.189912	NA	NA
C11orf86	NA	NA	-0.08388	-0.3386	0.048084	-0.05671	NA	NA
C11orf87	NA	NA	0.215072	-0.01851	-0.27384	0.114944	NA	NA
C11orf88	NA	NA	-0.14007	-0.00375	0.403008	-0.19089	NA	NA
C11orf9	NA	NA	0.211154	0.365546	0.006356	0.237019	0.405556	0.604186
C12orf10	NA	NA	0.228676	0.165074	0.198518	0.158452	-0.01787	-0.35627

C12orf11	NA	NA	-0.26457	0.035644	-0.05204	-0.36813	-0.32074	-0.07513
C12orf12	NA	NA	-0.12804	-0.35071	-0.26715	0.185372	-0.12337	0.066861
C12orf23	NA	NA	-0.10408	0.138713	-0.18064	-0.19809	-0.48399	-0.35364
C12orf24	NA	NA	-0.41578	0.323399	0.012463	-0.03779	0.162965	-0.16619
C12orf26	NA	NA	-0.45186	-0.02263	-0.34897	-0.34129	-0.17316	0.124968
C12orf28	NA	NA	-0.16093	-0.20717	0.239268	0.043401	0.276648	0.531322
C12orf29	NA	NA	-0.18309	-0.32229	0.032465	0.060228	-0.04028	-0.14261
C12orf32	NA	NA	0.07199	0.22756	-0.00856	-0.16845	0.234749	0.458796
C12orf34	NA	NA	0.016382	0.27369	0.276681	-0.25709	0.387124	0.69504
C12orf35	NA	NA	-0.1582	0.252407	-0.07084	0.054849	-0.63277	-0.22396
C12orf36	NA	NA	-0.07685	-0.18834	-0.06539	-0.10008	0.167446	-0.00369
C12orf39	NA	NA	0.032463	-0.13512	-0.04347	-0.15541	0.10255	0.51423
C12orf4	NA	NA	-0.46416	-0.25789	-0.26876	-0.25991	0.034651	0.307919
C12orf40	NA	NA	0.083117	-0.03459	-0.08806	0.03513	-0.17944	0.245905
C12orf41	NA	NA	-0.22538	0.149147	0.027337	-0.18219	0.054813	0.165663
C12orf42	NA	NA	-0.03345	0.721331	-0.13453	0.374092	0.369226	0.235513
C12orf43	NA	NA	-0.37144	0.310383	-0.05166	0.018615	-0.16551	0.011832
C12orf44	NA	NA	0.072144	0.120644	0.023073	0.174243	0.102073	0.25871
C12orf45	NA	NA	0.108158	0.095054	0.029755	-0.04125	-0.13364	-0.27092
C12orf48	NA	NA	-0.43418	-0.01938	-0.10521	-0.19722	0.044642	-0.04553
C12orf49	NA	NA	-0.01827	-0.10356	-0.09853	-0.29718	0.361565	0.233894
C12orf5	NA	NA	-0.25689	-0.07751	-0.38477	-0.17081	-0.23283	0.121649
C12orf50	NA	NA	-0.63712	-0.04613	0.26397	0.022377	0.282103	0.37458
C12orf51	NA	NA	0.036793	-0.20668	0.407775	0.230952	NA	NA
C12orf52	NA	NA	0.060751	0.200019	0.182804	-0.22231	-0.21954	-0.23084
C12orf53	NA	NA	0.026827	0.075913	-0.00771	0.22397	0.114545	0.029462
C12orf54	NA	NA	-0.35023	-0.32579	-0.1194	-0.26659	-0.559	-0.43034
C12orf56	NA	NA	0.219478	-0.22722	-0.10746	-0.0417	NA	NA
C12orf57	NA	NA	0.082679	0.030988	-0.357	-0.24996	-0.36068	-0.41847
C12orf59	NA	NA	0.112592	-0.23553	-0.08137	-0.41016	-0.0689	-0.31615
C12orf60	NA	NA	-0.06697	0.045911	-0.28676	-0.2453	0.278965	0.028115
C12orf61	NA	NA	-0.16359	0.006614	0.123137	-0.22384	-0.01512	0.390327
C12orf62	NA	NA	-0.02269	-0.43071	-0.19538	-0.34138	-0.6146	-0.38387
C12orf65	NA	NA	0.052552	-0.03923	-0.06666	0.014208	NA	NA
C12orf66	NA	NA	-0.09693	0.192419	0.105196	0.022579	NA	NA
C12orf68	NA	NA	-0.0363	0.18566	-0.0523	-0.13037	NA	NA
C12orf69	NA	NA	-0.15445	0.274221	0.061931	0.071051	NA	NA
C12orf73	NA	NA	0.041542	-0.0769	0.013921	-0.17383	NA	NA
C13orf1	NA	NA	-0.18458	-0.28984	0.110933	-0.61479	0.201234	-0.45518
C13orf16	NA	NA	0.110097	0.066687	0.009345	0.058694	-0.03752	0.221264
C13orf18	NA	NA	0.220497	-0.04014	0.267537	0.375009	0.019437	-0.06685
C13orf23	NA	NA	-0.01087	0.171334	0.294098	-0.36204	0.454347	0.101047
C13orf26	NA	NA	0.164905	-0.02744	-0.30292	-0.01708	-0.05942	-0.09911
C13orf33	NA	NA	0.060388	0.036357	0.07042	-0.2069	NA	NA
C14orf1	NA	NA	0.106762	0.033485	-0.12298	-0.35495	-0.46987	-0.36784
C14orf101	NA	NA	-0.07541	0.020848	-0.03868	0.103682	0.351812	0.324524
C14orf102	NA	NA	0.000479	-0.19735	0.181651	0.183951	-0.24231	0.205625
C14orf104	NA	NA	-0.4476	-0.07888	-0.19257	-0.31231	-0.06396	-0.3774
C14orf105	NA	NA	-0.19296	-0.21352	0.047813	0.122309	0.258526	0.398142
C14orf106	NA	NA	-0.26943	0.211681	-0.19559	0.061708	-0.20327	-0.06294
C14orf109	NA	NA	-0.31232	-0.29819	-0.29223	-0.4537	NA	NA
C14orf115	NA	NA	-0.092	0.407491	-0.15428	0.443492	0.251218	0.265492

C14orf118	NA	NA	-0.2745	-0.43758	0.006655	0.371562	0.413842	0.352202
C14orf119	NA	NA	-0.35445	-0.19019	-0.28623	-0.20626	-0.35432	-0.1536
C14orf126	NA	NA	-0.13812	0.043032	-0.06729	0.013269	0.344473	0.559542
C14orf129	NA	NA	-0.23255	-0.25843	-0.26454	-0.32116	-0.08888	0.019307
C14orf133	NA	NA	-0.03704	0.009611	-0.10752	-0.1604	0.635091	0.494786
C14orf135	NA	NA	-0.4415	0.048096	-0.2394	0.30867	-0.02531	0.140715
C14orf138	NA	NA	-0.60078	-0.03584	-0.36297	0.158116	0.143222	0.464241
C14orf139	NA	NA	-0.06091	0.268832	-0.32154	0.098861	-0.52182	0.187189
C14orf142	NA	NA	-0.32327	-0.02467	-0.26799	-0.51347	-0.53225	-0.41168
C14orf143	NA	NA	-0.00035	-0.17416	-0.10644	-0.39301	-0.07696	-0.27387
C14orf145	NA	NA	-0.39733	-0.05083	-0.23655	-0.04134	-0.22574	-0.05583
C14orf147	NA	NA	-0.14517	-0.34958	-0.04975	-0.2822	-0.05401	-0.15533
C14orf148	NA	NA	0.105748	0.220893	-0.24134	-0.2591	0.230902	0.17118
C14orf149	NA	NA	-0.16506	0.11262	-0.3787	-0.08497	-0.30736	-0.03847
C14orf153	NA	NA	-0.13926	-0.15558	-0.22452	0.088448	-0.34425	-0.09773
C14orf156	NA	NA	-0.16837	-0.09865	-0.37004	-0.21443	-0.25465	-0.10162
C14orf159	NA	NA	-0.04821	0.162287	-0.18658	0.362913	-0.04025	0.216151
C14orf162	NA	NA	0.188948	-0.31046	-0.04286	0.000346	-0.21599	-0.11531
C14orf166	NA	NA	-0.4999	0.017607	-0.28704	-0.49253	-0.66983	-0.5764
C14orf166E	NA	NA	NA	NA	NA	NA	-0.04665	0.16162
C14orf169	NA	NA	-0.32295	0.155133	0.12297	-0.03154	-0.36779	-0.36661
C14orf177	NA	NA	0.234025	0.046014	-0.1468	-0.26257	NA	NA
C14orf178	NA	NA	0.050618	0.038838	-0.12011	0.235037	NA	NA
C14orf2	NA	NA	-0.01536	-0.37062	-0.26425	-0.26493	0.027842	-0.12856
C14orf21	NA	NA	-0.17175	0.065776	0.20655	0.036399	-0.23077	0.087876
C14orf28	NA	NA	-0.15411	-0.07343	-0.41259	-0.39729	-0.17624	-0.10777
C14orf37	NA	NA	-0.06305	0.010042	-0.18009	0.058373	-0.29706	-0.20421
C14orf39	NA	NA	0.040571	-0.23009	0.025145	-0.13189	0.424705	0.200306
C14orf4	NA	NA	0.292846	-0.07726	0.278734	0.005019	-0.46751	-0.66147
C14orf43	NA	NA	-0.00843	-0.1823	-0.13508	0.18982	-0.05455	0.010823
C14orf45	NA	NA	-0.10183	-0.41223	-0.31028	-0.42161	-0.44019	-0.02034
C14orf49	NA	NA	-0.01965	0.356373	-0.15487	0.314788	-0.12596	0.318442
C14orf50	NA	NA	0.057967	-0.40297	-0.25054	-0.49761	0.014442	-0.25386
C14orf68	NA	NA	0.259301	-0.17946	0.162783	-0.25047	-0.15611	-0.19812
C14orf73	NA	NA	0.104912	0.068355	0.141105	0.276144	-0.01712	0.526115
C14orf79	NA	NA	0.079681	0.089284	0.094989	0.271805	0.135887	0.082525
C14orf80	NA	NA	-0.1726	0.157382	0.143768	-0.12772	-0.22417	-0.19391
C14orf93	NA	NA	0.08496	-0.04701	0.069039	-0.47605	0.115287	-0.17666
C15orf17	NA	NA	0.228651	0.019637	0.036711	0.049038	-0.12581	0.114155
C15orf2	NA	NA	-0.19935	0.150306	-0.17438	-0.06509	-0.22753	-0.06646
C15orf21	NA	NA	-0.38395	0.315477	0.229952	0.001731	-0.11804	0.3712
C15orf23	NA	NA	-0.30017	-0.05323	-0.26206	-0.46253	0.115459	-0.1483
C15orf24	NA	NA	0.060517	0.081442	-0.00591	-0.08846	0.324434	0.321736
C15orf26	NA	NA	-0.60101	-0.02484	-0.09615	-0.01459	-0.04689	-0.33217
C15orf27	NA	NA	-0.12364	0.620045	-0.27329	0.40399	0.061073	-0.01739
C15orf29	NA	NA	-0.02569	-0.1588	-0.36738	-0.31851	-0.44447	-0.27607
C15orf32	NA	NA	-0.01732	-0.18874	0.118419	0.22862	0.240258	-0.12133
C15orf33	NA	NA	-0.15943	-0.27492	0.161987	-0.05576	0.334703	0.036164
C15orf37	NA	NA	-0.16031	-0.18426	0.130029	-0.33757	0.145087	-0.18501
C15orf38	NA	NA	0.112682	-0.33175	0.022556	-0.27957	0.309698	0.176779
C15orf39	NA	NA	-0.04528	-0.03153	0.037479	0.059806	-0.13645	-0.10224
C15orf40	NA	NA	-0.06388	-0.06571	-0.09147	-0.49632	0.264406	0.350957

C15orf41	NA	NA	-0.23997	0.010712	-0.06258	0.145671	0.421317	0.185381
C15orf42	NA	NA	-0.37694	0.241998	0.198933	-0.17157	0.356783	0.350005
C15orf43	NA	NA	0.237913	-0.24071	-0.15103	-0.16005	0.31448	0.471473
C15orf44	NA	NA	-0.08116	-0.07407	0.030073	-0.06058	0.417454	0.151884
C15orf48	NA	NA	-0.05633	-0.33536	-0.37547	-0.30545	-0.31965	-0.18877
C15orf5	NA	NA	-0.3038	-0.26424	-0.08722	-0.44155	-0.48873	-0.34624
C15orf52	NA	NA	-0.07349	-0.04431	0.015489	-0.04801	NA	NA
C15orf55	NA	NA	-0.14621	0.162676	-0.36255	0.080704	NA	NA
C15orf56	NA	NA	0.188354	0.131476	-0.13199	0.283005	NA	NA
C15orf57	NA	NA	0.13198	-0.04315	-0.40472	-0.10448	NA	NA
C15orf59	NA	NA	0.064636	-0.00425	-0.18339	-0.07692	NA	NA
C15orf60	NA	NA	-0.29623	-0.07912	0.095554	0.231148	NA	NA
C15orf61	NA	NA	-0.11101	-0.01222	-0.03511	-0.55571	NA	NA
C15orf62	NA	NA	0.175706	0.440391	-0.05083	-0.15613	NA	NA
C16orf11	NA	NA	-0.02787	-0.03898	-0.12818	0.262738	NA	NA
C16orf13	NA	NA	-0.05583	0.188561	0.127216	-0.07196	NA	NA
C16orf3	NA	NA	-0.12519	-0.02116	-0.00107	0.2014	0.204837	-0.0512
C16orf45	NA	NA	0.040314	-0.11452	-0.19116	0.22501	-0.32507	-0.28015
C16orf46	NA	NA	0.031516	0.096294	-0.13476	-0.07889	0.247369	0.050675
C16orf48	NA	NA	-0.03155	-0.04844	0.204538	-0.09368	-0.35617	-0.57493
C16orf5	NA	NA	0.007207	0.021	0.260187	-0.24389	0.094856	0.098396
C16orf53	NA	NA	-0.06355	0.448748	0.434148	0.055933	-0.36965	-0.4594
C16orf54	NA	NA	0.254685	0.526382	-0.09309	-0.0385	-0.01623	0.148872
C16orf55	NA	NA	0.136256	0.135383	0.466671	-0.04289	0.100385	-0.35367
C16orf57	NA	NA	-0.09056	0.012837	0.134585	-0.32303	-0.49244	-0.54048
C16orf58	NA	NA	0.326041	0.389428	0.434139	0.319997	-0.39808	-0.38749
C16orf59	NA	NA	-0.36906	0.181087	0.096744	-0.17864	NA	NA
C16orf61	NA	NA	0.07908	-0.19458	-0.08669	-0.16341	-0.02734	0.106324
C16orf62	NA	NA	-0.01046	-0.00383	-0.01314	0.041291	NA	NA
C16orf63	NA	NA	-0.33108	-0.1985	0.084896	-0.31433	0.293068	-0.13209
C16orf7	NA	NA	0.364974	-0.22471	0.051083	0.132093	-0.24039	0.51221
C16orf70	NA	NA	0.012064	-0.19974	0.004129	-0.30346	NA	NA
C16orf71	NA	NA	0.018637	-0.04743	-0.22557	0.106757	NA	NA
C16orf72	NA	NA	-0.3664	-0.40856	0.196272	-0.08886	NA	NA
C16orf73	NA	NA	-0.09377	-0.59031	-0.17042	0.266404	NA	NA
C16orf74	NA	NA	0.088108	0.00978	-0.06372	-0.19399	NA	NA
C16orf78	NA	NA	0.393043	-0.24399	-0.01883	-0.001	0.256129	0.1779
C16orf79	NA	NA	-0.03872	0.134719	0.260735	0.056721	NA	NA
C16orf80	NA	NA	0.100659	0.091899	-0.12393	-0.3146	0.177327	-0.12924
C16orf82	NA	NA	0.228387	0.016419	0.010361	-0.06786	NA	NA
C16orf86	NA	NA	0.233709	0.009596	0.153687	0.01588	NA	NA
C16orf87	NA	NA	-0.20527	-0.09771	0.253693	-0.15742	NA	NA
C16orf88	NA	NA	-0.52699	0.068914	-0.01097	-0.02187	NA	NA
C16orf89	NA	NA	-0.00447	0.251366	-0.28853	0.232311	NA	NA
C17orf100	NA	NA	-0.24464	0.129092	-0.13563	-0.37034	NA	NA
C17orf101	NA	NA	0.026635	0.435095	0.153593	0.036354	NA	NA
C17orf28	NA	NA	0.343778	0.376677	0.091201	-0.15307	0.216967	0.25747
C17orf37	NA	NA	0.445436	-0.08446	-0.04427	-0.14804	-0.2203	-0.21084
C17orf39	NA	NA	0.004739	0.244731	-0.06293	-0.34933	-0.62205	-0.66957
C17orf42	NA	NA	0.035272	-0.34209	-0.17129	-0.21047	0.382853	0.264952
C17orf46	NA	NA	-0.0116	-0.18629	-0.1111	0.071177	0.379299	0.430073
C17orf47	NA	NA	-0.05508	-0.21343	0.174142	-0.0709	-0.12329	-0.32912

C17orf48	NA	NA	-0.0631	-0.26428	-0.38098	-0.22186	-0.0323	0.038892
C17orf49	NA	NA	0.100434	0.508091	-0.26585	0.215843	0.320988	0.236798
C17orf50	NA	NA	0.061563	-0.05001	-0.28603	-0.09289	0.203994	0.214375
C17orf51	NA	NA	-0.06954	0.114096	0.097831	-0.20254	NA	NA
C17orf53	NA	NA	-0.00738	0.202267	0.175453	-0.02663	0.285963	0.139898
C17orf55	NA	NA	-0.21856	-0.14592	-0.03002	-0.12438	0.399366	0.557592
C17orf56	NA	NA	0.032783	0.17417	0.306342	0.163278	0.213966	0.413005
C17orf57	NA	NA	-0.0347	0.132145	0.099199	-0.01109	-0.21087	-0.04234
C17orf58	NA	NA	0.140299	0.040689	-0.03577	-0.55551	0.318818	0.274355
C17orf59	NA	NA	0.163207	0.280015	-0.00312	0.051798	-0.02448	0.165029
C17orf61	NA	NA	0.151652	-0.11349	-0.29175	-0.07605	0.133276	0.301212
C17orf62	NA	NA	0.399655	0.28047	0.251515	0.095927	0.035408	0.45056
C17orf63	NA	NA	0.03193	-0.21541	0.173965	-0.04059	0.602991	0.485649
C17orf64	NA	NA	-0.13039	0.381162	0.066685	-0.57749	0.040298	0.254069
C17orf65	NA	NA	0.309336	-0.18371	0.12725	0.204677	0.331651	0.61156
C17orf66	NA	NA	0.212589	-0.22375	0.124417	0.131411	0.172081	0.454933
C17orf67	NA	NA	-0.1964	0.355652	-0.24578	0.353452	NA	NA
C17orf68	NA	NA	0.092532	0.332832	-0.16405	0.439522	-0.12274	0.098167
C17orf70	NA	NA	0.512008	0.099449	0.264341	0.320792	-0.23265	0.135463
C17orf71	NA	NA	0.268244	-0.08794	0.058971	-0.19343	-0.27584	-0.41284
C17orf72	NA	NA	-0.02264	0.319253	-0.35641	-0.02783	NA	NA
C17orf74	NA	NA	-0.2257	0.011172	-0.10848	0.270907	0.436804	0.075395
C17orf75	NA	NA	-0.05708	-0.21798	0.002633	-0.30591	0.112808	0.050178
C17orf76	NA	NA	-0.03793	-0.16667	-0.12849	-0.11345	-0.0207	-0.23346
C17orf77	NA	NA	0.325445	-0.07615	-0.13233	0.040477	0.123256	-0.15577
C17orf78	NA	NA	0.169038	-0.40934	0.038511	0.02948	0.350049	0.472061
C17orf79	NA	NA	0.189634	0.126566	0.153599	-0.18153	-0.60068	-0.62824
C17orf80	NA	NA	0.232514	-0.09255	0.077991	0.058256	0.420228	0.150956
C17orf81	NA	NA	0.151317	-0.0381	-0.14199	-0.23667	0.182574	-0.05774
C17orf85	NA	NA	-0.44836	-0.08751	-0.2484	0.043895	0.085253	0.442318
C17orf89	NA	NA	0.136187	-0.08272	-0.03815	-0.32847	NA	NA
C17orf90	NA	NA	0.341725	0.073315	0.097773	-0.10124	NA	NA
C17orf96	NA	NA	-0.04479	-0.03038	-0.16922	0.219871	NA	NA
C17orf97	NA	NA	0.022367	-0.07132	-0.1148	0.014317	NA	NA
C17orf98	NA	NA	-0.31512	-0.09091	0.098612	0.060365	NA	NA
C17orf99	NA	NA	-0.30202	-0.0042	-0.17363	-0.03148	NA	NA
C18orf1	NA	NA	0.173025	-0.12122	0.139501	0.169176	-0.13521	0.05796
C18orf10	NA	NA	0.110096	0.305904	-0.05407	-0.03277	0.153001	-0.37282
C18orf19	NA	NA	-0.25173	-0.13453	-0.21981	-0.01294	-0.0577	-0.15166
C18orf21	NA	NA	-0.0106	-0.22105	-0.29482	-0.18944	-0.5278	-0.37979
C18orf22	NA	NA	0.06375	0.246099	-0.05807	0.263905	0.023548	-0.08567
C18orf25	NA	NA	-0.15792	-0.17979	0.028629	-0.30668	-0.03774	0.219543
C18orf26	NA	NA	-0.16445	-0.03877	0.068094	0.135088	-0.1211	-0.10139
C18orf32	NA	NA	-0.06325	-0.07912	-0.39649	-0.37812	NA	NA
C18orf34	NA	NA	-0.19811	-0.03057	-0.44441	-0.10571	0.003429	0.131512
C18orf45	NA	NA	0.241834	0.181002	0.036077	0.06472	-0.20112	-0.31909
C18orf54	NA	NA	-0.03459	0.146858	0.009818	-0.00625	-0.04637	-0.19148
C18orf55	NA	NA	-0.15248	-0.06824	-0.35818	-0.38578	-0.15673	-0.30021
C18orf56	NA	NA	-0.3992	0.11111	-0.10758	-0.50293	-0.22715	-0.07361
C18orf8	NA	NA	0.347964	-0.21333	-0.01269	-0.141	-0.15333	0.632411
C19orf10	NA	NA	NA	NA	NA	NA	0.147503	0.028188
C19orf12	NA	NA	-0.01735	-0.17941	-0.06791	-0.28454	0.038015	0.211795

C19orf18	NA	NA	-0.25076	-0.19851	-0.16611	0.082615	-0.0639	0.188221
C19orf2	NA	NA	-0.28106	0.176213	0.116863	0.018627	0.277173	-0.05282
C19orf20	NA	NA	0.112455	-0.16581	-0.03514	-0.16993	0.117903	0.064747
C19orf21	NA	NA	0.084886	0.202683	0.36999	0.160582	0.077761	-0.08894
C19orf22	NA	NA	0.301177	0.123953	0.28197	-0.04635	0.201952	0.22898
C19orf24	NA	NA	0.246978	-0.03275	0.055728	-0.07128	0.458881	0.291575
C19orf25	NA	NA	-0.08344	0.013056	0.179907	-0.26236	0.331792	0.593259
C19orf26	NA	NA	0.058831	-0.03078	0.065466	-0.10309	-0.07984	0.120306
C19orf28	NA	NA	0.149048	0.34681	0.279886	0.263708	0.043428	0.050286
C19orf29	NA	NA	-0.31318	0.21997	0.0361	0.122116	0.003884	0.277842
C19orf30	NA	NA	0.236989	-0.12461	-0.19948	-0.00692	-0.59928	-0.48338
C19orf33	NA	NA	0.010017	-0.21847	-0.32077	-0.28335	0.313902	0.032604
C19orf34	NA	NA	0.310673	0.235166	-0.08726	-0.11464	-0.1811	-0.52909
C19orf35	NA	NA	NA	NA	NA	NA	0.433615	0.288157
C19orf36	NA	NA	-0.07793	0.217581	-0.01116	0.055893	-0.10495	-0.05104
C19orf38	NA	NA	0.141559	0.090028	-0.15668	-0.29001	NA	NA
C19orf40	NA	NA	-0.47358	-0.02869	0.033727	-0.17786	-0.03567	-0.06233
C19orf42	NA	NA	-0.34718	-0.10209	-0.15761	-0.24035	NA	NA
C19orf43	NA	NA	0.039133	0.372706	0.154093	-0.05487	NA	NA
C19orf44	NA	NA	0.279177	-0.06927	0.138229	0.170025	NA	NA
C19orf47	NA	NA	0.011507	0.048507	0.135147	-0.37776	NA	NA
C19orf48	NA	NA	0.30648	0.186063	0.217188	0.161248	0.47115	0.125586
C19orf52	NA	NA	0.059997	0.147877	0.167442	-0.01045	NA	NA
C19orf53	NA	NA	0.073027	0.268227	-0.18968	-0.22167	NA	NA
C19orf55	NA	NA	-0.02736	-0.10028	-0.03414	0.260824	0.653053	0.209655
C19orf57	NA	NA	-0.22776	-0.23414	0.039492	-0.1705	NA	NA
C19orf59	NA	NA	0.09126	0.141774	-0.05124	-0.16351	NA	NA
C19orf6	NA	NA	-0.15994	0.285815	0.199609	-0.02805	-0.03208	0.278262
C19orf60	NA	NA	0.438613	0.309195	0.259223	0.148843	NA	NA
C19orf66	NA	NA	0.478988	0.115197	0.149459	0.01895	NA	NA
C19orf69	NA	NA	0.311612	-0.39407	0.008565	-0.1497	NA	NA
C19orf70	NA	NA	0.257022	-0.22101	-0.0388	-0.05957	NA	NA
C19orf71	NA	NA	-0.04376	-0.11847	-0.07271	-0.19924	NA	NA
C1D	-0.01793	-0.3347	-0.23178	-0.03033	-0.31605	-0.32801	-0.67089	-0.41474
C1GALT1	-0.07488	-0.34136	-0.22499	-0.06342	-0.03429	-0.47275	0.005923	0.176674
C1GALT1C1	0.100113	0.314792	-0.03601	-0.0985	0.216565	-0.37693	0.073533	-0.0498
C1orf100	NA	NA	-0.26298	-0.24467	0.246789	0.122296	0.231537	0.086905
C1orf101	NA	NA	-0.17962	-0.1528	-0.49532	0.139412	0.107426	0.438494
C1orf103	NA	NA	-0.24831	0.016386	0.004595	-0.2636	0.038035	-0.26929
C1orf104	NA	NA	0.063925	-0.01598	-0.20061	-0.00764	0.549289	0.311159
C1orf105	NA	NA	-0.18325	-0.15092	-0.40061	-0.00508	0.152463	0.113042
C1orf106	NA	NA	-0.12112	0.056175	0.095484	-0.15193	0.483144	0.23195
C1orf107	NA	NA	-0.37724	-0.14715	-0.05439	0.136767	0.492386	0.268215
C1orf109	NA	NA	-0.09639	0.083939	0.223209	0.006994	0.583577	0.472491
C1orf110	NA	NA	0.113423	-0.25664	-0.21724	0.008535	-0.17403	0.011295
C1orf111	NA	NA	0.041194	-0.11339	0.032613	0.070304	0.206432	-0.03254
C1orf112	NA	NA	-0.32264	-0.06155	-0.01774	-0.13164	0.469614	0.241732
C1orf113	NA	NA	-0.06163	-0.09571	-0.16892	0.079208	-0.45681	-0.48666
C1orf114	NA	NA	0.131323	-0.1823	-0.31156	-0.15286	-0.20682	-0.23903
C1orf115	NA	NA	0.194472	-0.0998	0.038343	-0.26366	0.376221	0.393714
C1orf116	NA	NA	-0.14171	-0.11548	-0.03115	-0.06041	0.630433	0.448847
C1orf122	NA	NA	0.221656	0.030103	0.08518	-0.33269	0.090349	0.066551

C1orf123	NA	NA	0.181386	-0.00211	-0.14451	-0.3503	0.087563	0.101202
C1orf124	NA	NA	-0.24035	-0.19096	-0.4708	-0.37677	0.439453	0.400984
C1orf125	NA	NA	-0.41597	0.031173	-0.32055	-0.28847	-0.0304	-0.09878
C1orf127	NA	NA	0.008706	0.151212	-0.04973	-0.4216	0.011332	0.157685
C1orf128	NA	NA	-0.0027	0.265228	0.111285	-0.23988	0.353055	0.582159
C1orf129	NA	NA	0.328144	0.285346	-0.02806	0.063079	-0.34894	-0.31017
C1orf131	NA	NA	-0.41875	-0.21306	-0.04831	-0.61339	0.475782	0.258356
C1orf135	NA	NA	-0.26498	0.124801	0.235724	-0.27463	0.238102	0.251228
C1orf14	NA	NA	-0.03379	-0.18997	-0.06397	0.30459	-0.01073	0.087527
C1orf141	NA	NA	NA	NA	NA	NA	0.344603	0.330654
C1orf144	NA	NA	-0.00686	0.228802	0.017958	0.013403	-0.5157	-0.42634
C1orf146	NA	NA	NA	NA	NA	NA	0.179171	0.199805
C1orf150	NA	NA	0.018289	0.184475	-0.29373	-0.23471	-0.3193	-0.22993
C1orf151	NA	NA	-0.02295	-0.30856	0.088424	-0.15465	0.022182	-0.15938
C1orf152	NA	NA	-0.41349	0.209163	-0.44075	-0.28991	-0.24062	-0.15809
C1orf156	NA	NA	-0.33447	-0.06666	-0.20488	-0.40746	-0.16623	-0.37308
C1orf157	NA	NA	-0.44351	-0.00612	0.166034	-0.07808	0.487992	0.163044
C1orf158	NA	NA	-0.07474	-0.08332	-0.2204	-0.05857	0.01031	-0.09604
C1orf159	NA	NA	0.204055	-0.10997	0.23147	0.014542	0.366037	0.480749
C1orf162	NA	NA	0.227944	0.039156	-0.45326	0.277657	-0.32732	0.125871
C1orf163	NA	NA	0.153569	-0.12023	0.203346	-0.14529	0.287791	-0.07558
C1orf168	NA	NA	0.197382	-0.04175	-0.24421	-0.54036	0.061909	0.303759
C1orf170	NA	NA	-0.22155	0.020112	-0.0179	-0.14427	NA	NA
C1orf172	NA	NA	-0.18771	0.016711	0.062062	-0.42613	0.52648	0.128953
C1orf173	NA	NA	0.144829	0.382688	-0.22043	0.131512	-0.11752	0.083451
C1orf174	NA	NA	-0.02723	0.169384	0.189564	-0.13055	NA	NA
C1orf175	NA	NA	0.197338	-0.21087	0.202055	0.045607	-0.14185	0.015824
C1orf177	NA	NA	0.037403	-0.18752	0.10481	-0.13734	0.183825	0.236142
C1orf182	NA	NA	-0.07717	-0.1615	-0.2689	-0.48369	0.155363	-0.00213
C1orf183	NA	NA	0.081804	0.063752	0.174437	-0.31591	-0.13685	-0.01647
C1orf186	NA	NA	0.089656	0.139659	0.008108	-0.06333	0.09214	0.308363
C1orf187	NA	NA	-0.02455	-0.19148	0.029089	-0.11488	0.061334	0.200568
C1orf189	NA	NA	NA	NA	NA	NA	-0.03305	0.372871
C1orf190	NA	NA	-0.03197	-0.13687	-0.09077	0.1105	-0.30702	-0.2996
C1orf192	NA	NA	0.138339	-0.3314	-0.05001	0.234573	-0.32664	-0.33325
C1orf194	NA	NA	-0.17019	-0.16418	0.219024	-0.22093	NA	NA
C1orf198	NA	NA	-0.18332	0.067482	0.112075	0.238337	0.307588	0.083263
C1orf201	NA	NA	0.160999	-0.24356	0.192171	-0.10259	-0.19084	-0.20366
C1orf204	NA	NA	-0.22084	0.256769	-0.05184	0.250724	NA	NA
C1orf21	NA	NA	-0.22318	-0.28016	-0.26314	-0.5215	-0.20343	0.083551
C1orf210	NA	NA	0.157846	-0.18251	0.003611	-0.34969	0.062015	0.171126
C1orf212	NA	NA	0.194604	0.249259	-0.34037	0.0343	NA	NA
C1orf213	NA	NA	0.009328	0.033566	0.01248	-0.00361	NA	NA
C1orf216	NA	NA	0.280578	0.195301	0.137983	0.095954	NA	NA
C1orf226	NA	NA	-0.46745	-0.02676	0.014599	-0.29513	NA	NA
C1orf25	NA	NA	-0.10103	-0.13738	-0.14964	-0.34569	-0.32895	-0.51781
C1orf26	NA	NA	0.095255	-0.27543	-0.33457	-0.25888	0.01821	-0.21022
C1orf27	NA	NA	-0.4405	0.025666	-0.24541	0.000527	-0.2532	-0.02187
C1orf31	NA	NA	-0.31324	-0.15473	-0.36683	-0.46112	0.037286	-0.07103
C1orf35	NA	NA	0.060486	0.127892	0.191727	0.178646	0.594867	0.392464
C1orf38	NA	NA	0.314739	0.278199	-0.00037	0.233931	-0.54427	0.244557
C1orf43	NA	NA	0.185977	-0.00476	0.053465	-0.06135	-0.08613	-0.48048

C1orf49	NA	NA	0.034516	-0.27872	0.196155	-0.59547	-0.33198	-0.02025
C1orf50	NA	NA	0.112626	-0.15077	-0.06892	-0.39875	0.150784	-0.04386
C1orf51	NA	NA	-0.24504	-0.25079	-0.02269	0.033942	-0.23288	0.04335
C1orf52	NA	NA	-0.03403	-0.03623	-0.05771	-0.36891	0.075055	0.220232
C1orf53	NA	NA	-0.3056	-0.07195	-0.33347	-0.31279	0.2127	-0.11545
C1orf54	NA	NA	-0.15421	-0.20326	-0.31786	0.063751	-0.5144	-0.19765
C1orf55	NA	NA	-0.14133	-0.19819	-0.24491	-0.20156	0.375044	0.291515
C1orf56	NA	NA	-0.18376	0.433551	-0.09674	-0.02545	0.466346	0.285781
C1orf57	NA	NA	0.051288	-0.00445	-0.19137	-0.09893	-0.0245	-0.12038
C1orf58	NA	NA	-0.27727	-0.14415	-0.25039	-0.38786	-0.13585	0.207399
C1orf59	NA	NA	0.029814	-0.05928	0.350748	-0.25475	0.35517	0.459699
C1orf61	NA	NA	-0.03367	-0.14998	0.206842	-0.11328	-0.34284	-0.15166
C1orf63	NA	NA	-0.21474	0.146153	0.116127	-0.21643	-0.56468	-0.27145
C1orf64	NA	NA	-0.20016	-0.1839	-0.08305	0.015947	0.152873	0.069616
C1orf65	NA	NA	-0.27803	-0.11431	-0.11846	0.122929	0.097706	-0.14248
C1orf66	NA	NA	0.209851	0.09849	0.236755	-0.01591	0.639915	0.590332
C1orf69	NA	NA	0.047439	0.089735	0.104456	-0.16894	0.544005	0.458763
C1orf74	NA	NA	0.081468	0.086009	-0.03443	-0.13091	0.114233	-0.12767
C1orf77	NA	NA	-0.03662	-0.00606	-0.01376	-0.05881	0.00291	-0.31565
C1orf83	NA	NA	0.338398	-0.00361	0.359265	0.081301	-0.0861	-0.1029
C1orf84	NA	NA	0.278345	-0.08921	0.216996	0.278592	0.291037	0.351837
C1orf85	NA	NA	0.217668	-0.24647	-0.20865	-0.21428	-0.04643	0.006123
C1orf86	NA	NA	0.124129	-0.12322	0.112306	0.208165	0.133942	-0.112
C1orf87	NA	NA	0.034651	-0.0498	0.13998	-0.07423	-0.09289	-0.38623
C1orf88	NA	NA	0.468612	-0.21082	-0.19102	-0.2181	-0.17452	0.044329
C1orf89	NA	NA	0.274143	0.132626	0.355849	-0.16226	-0.01685	-0.07931
C1orf9	NA	NA	0.211385	-0.10332	0.014123	0.19077	-0.3602	-0.10201
C1orf91	NA	NA	0.406255	-0.04562	0.14188	-0.09466	0.078036	-0.1612
C1orf92	NA	NA	0.368635	-0.53524	-0.03643	-0.01704	-0.33804	-0.36328
C1orf94	NA	NA	-0.19509	-0.01955	-0.07703	0.0955	0.049474	-0.59081
C1orf95	NA	NA	-0.24169	-0.28156	-0.04288	-0.12359	-0.13392	-0.39027
C1orf96	NA	NA	-0.20284	0.128945	0.034919	0.047848	-0.04094	-0.18578
C1QA	-0.27029	0.141767	0.303862	-0.07202	-0.42316	0.073699	-0.34247	0.147707
C1QB	-0.19257	0.025584	0.361317	-0.23036	-0.31889	0.096753	-0.46979	0.014453
C1QBP	0.203207	-0.0891	-0.25552	0.442649	-0.22046	-0.05552	0.195467	-0.23368
C1QC	-0.29552	-0.04167	0.327305	0.055158	-0.28887	0.108342	-0.39276	0.156057
C1QL1	-0.32002	0.201484	-0.09183	-0.24728	-0.0608	0.149208	-0.03047	0.148411
C1QL2	0.116212	0.213132	0.018362	-0.07399	0.082925	0.201998	-0.18844	-0.0289
C1QL3	0.291067	0.598244	0.151782	0.093726	-0.44894	-0.00681	0.00282	-0.02621
C1QL4	0.049012	-0.21347	NA	NA	NA	NA	-0.11265	-0.29048
C1QTNF1	0.311255	0.236723	0.282008	0.231092	0.007143	0.033591	-0.07705	0.071061
C1QTNF2	0.391527	0.525353	-0.09311	-0.08759	-0.14399	0.058974	-0.31404	-0.16461
C1QTNF3	0.160825	-0.0075	-0.05508	-0.14687	-0.09102	0.062583	-0.39949	-0.15402
C1QTNF4	NA	NA	0.077024	-0.26866	-0.10585	0.064563	-0.09755	-0.14594
C1QTNF5	-0.13578	-0.19121	0.236508	-0.02747	-0.01306	-0.08171	-0.0172	0.107424
C1QTNF6	-0.07545	0.050075	0.071204	-0.11888	-0.27719	-0.26553	-0.14846	-0.04399
C1QTNF7	0.05783	-0.32294	-0.02222	-0.08949	-0.29799	0.071064	-0.18773	-0.25388
C1QTNF8	0.136768	0.422724	NA	NA	NA	NA	-0.27675	-0.18369
C1QTNF9	0.154328	0.017199	0.33189	-0.11223	-0.0474	0.015553	NA	NA
C1R	-0.31258	-0.41777	0.046923	0.292976	0.006841	0.471795	-0.24238	0.22693
C1RL	0.181117	-0.39247	-0.04599	0.057239	-0.18817	-0.31359	-0.3519	0.011319
C1S	-0.19615	-0.36405	0.098206	0.262821	-0.02917	0.542172	-0.19836	0.186526

C2	0.416514	0.036324	0.367548	0.237242	0.235034	0.417508	0.140549	0.308601
C20orf103	NA	NA	0.100463	-0.05639	-0.07863	0.029416	-0.1777	-0.28409
C20orf106	NA	NA	-0.40142	-0.1448	-0.04751	-0.10951	0.037788	0.07684
C20orf108	NA	NA	0.218515	-0.21216	0.187966	-0.52507	-0.04844	-0.14748
C20orf11	NA	NA	-0.04166	0.176565	0.377673	-0.22747	0.450526	0.198123
C20orf111	NA	NA	0.205544	-0.18104	0.2026	-0.19501	0.313337	0.198193
C20orf112	NA	NA	0.122572	0.117529	0.33212	-0.10589	0.000647	0.429354
C20orf114	NA	NA	0.079771	-0.19944	0.180804	-0.23987	-0.08786	0.126014
C20orf117	NA	NA	0.237242	-0.20317	0.342297	0.088953	0.100079	-0.03041
C20orf118	NA	NA	NA	NA	NA	NA	0.466113	0.291821
C20orf12	NA	NA	-0.33625	-0.03007	0.034852	-0.17157	0.492615	-0.01958
C20orf132	NA	NA	-0.09369	-0.21384	-0.18203	-0.07769	-0.28311	-0.2919
C20orf135	NA	NA	0.165025	-0.21013	0.061831	-0.36821	-0.17917	-0.01105
C20orf141	NA	NA	0.201612	-0.17153	0.01694	-0.34358	0.404891	0.458814
C20orf144	NA	NA	-0.07059	-0.07966	0.014733	0.118965	-0.00029	0.098912
C20orf151	NA	NA	0.194325	-0.07963	0.208161	-0.4106	-0.13458	-0.21765
C20orf152	NA	NA	-0.06132	-0.11303	0.267358	0.319615	-0.07833	-0.26426
C20orf160	NA	NA	0.058499	0.047931	-0.12287	0.28007	-0.17292	0.274729
C20orf165	NA	NA	0.053919	-0.11154	0.219177	-0.22122	0.353119	0.309351
C20orf166	NA	NA	0.233937	-0.16251	0.182204	-0.15341	-0.23244	-0.62624
C20orf177	NA	NA	0.203903	0.012222	0.317261	-0.05274	0.304219	0.239658
C20orf185	NA	NA	-0.28663	0.100758	0.339427	0.085861	-0.51283	-0.2297
C20orf186	NA	NA	-0.16485	-0.17802	0.047069	0.316233	-0.07183	0.229736
C20orf194	NA	NA	0.114762	0.004208	-0.07679	0.345605	NA	NA
C20orf195	NA	NA	0.063861	0.049314	0.092022	0.029268	0.290394	0.609858
C20orf196	NA	NA	-0.14559	0.136354	0.252677	-0.28885	NA	NA
C20orf197	NA	NA	-0.0591	-0.45391	0.250957	0.064907	NA	NA
C20orf20	NA	NA	0.179339	0.418642	0.286689	-0.26051	0.33885	0.211777
C20orf201	NA	NA	-0.2591	0.112567	-0.13245	0.09011	NA	NA
C20orf24	NA	NA	0.166381	-0.05718	0.334135	-0.10777	0.394496	0.275364
C20orf26	NA	NA	0.078928	-0.11127	-0.43093	-0.13147	-0.23315	0.101877
C20orf27	NA	NA	-0.13844	0.227039	-0.052	-0.07783	-0.07193	-0.43187
C20orf29	NA	NA	-0.21257	-0.16491	-0.02109	-0.25508	0.594311	0.364444
C20orf3	NA	NA	0.021929	0.358834	0.271323	0.2209	0.397331	0.184612
C20orf30	NA	NA	-0.19655	0.124239	-0.03734	-0.0947	0.609719	0.147433
C20orf4	NA	NA	0.007251	-0.09746	0.188719	0.187174	0.635122	0.430158
C20orf43	NA	NA	0.112774	0.157168	0.217384	-0.27807	0.346508	0.165775
C20orf46	NA	NA	0.407813	0.104003	0.122255	-0.00174	0.291065	0.395438
C20orf54	NA	NA	0.182182	-0.21524	0.128795	0.109389	0.441685	0.324599
C20orf7	NA	NA	-0.31676	-0.1117	-0.30161	-0.34283	-0.14987	-0.43637
C20orf71	NA	NA	0.060853	-0.11846	0.098067	-0.06971	0.299505	0.14255
C20orf72	NA	NA	-0.26833	-0.05802	-0.00254	-0.26126	0.700095	0.309283
C20orf79	NA	NA	0.177518	-0.09738	-0.23819	0.06859	0.434377	0.307741
C20orf85	NA	NA	-0.0447	-0.09714	-0.07438	0.259037	0.276287	0.019771
C20orf94	NA	NA	-0.11823	-0.09517	-0.02669	-0.00637	-0.30808	-0.43283
C20orf96	NA	NA	-0.10046	-0.14229	-0.1029	-0.3403	-0.12816	0.301127
C21orf119	NA	NA	-0.09869	-0.18878	-0.17358	-0.24544	0.214913	0.242267
C21orf2	NA	NA	0.078218	0.365621	-0.052	0.166045	-0.27193	-0.00863
C21orf29	NA	NA	0.155706	0.528658	0.012686	0.661183	0.106181	0.053767
C21orf33	NA	NA	-0.0086	0.064277	-0.10863	0.13182	0.315423	0.149648
C21orf34	NA	NA	0.184108	-0.26512	-0.05766	-0.2695	-0.38219	-0.2461
C21orf41	NA	NA	-0.01324	0.200417	-0.08247	0.037273	-0.20348	-0.06485

C21orf45	NA	NA	-0.1425	0.224572	-0.09024	-0.14399	-0.33148	-0.50303
C21orf49	NA	NA	0.115977	-0.06328	0.032527	-0.31127	-0.11156	-0.0952
C21orf56	NA	NA	0.452896	0.114007	-0.02734	0.048586	0.208121	0.022334
C21orf57	NA	NA	0.022413	-0.05062	-0.05084	0.014282	-0.21883	0.248461
C21orf58	NA	NA	0.141044	-0.01593	-0.09071	-0.0396	0.27713	-0.10153
C21orf59	NA	NA	-0.03734	0.366741	0.098366	-0.13132	0.477601	-0.11658
C21orf62	NA	NA	0.425451	0.379747	-0.20861	0.145053	0.076788	-0.12874
C21orf63	NA	NA	0.04671	0.041024	0.060233	0.023423	-0.05108	-0.03055
C21orf67	NA	NA	0.127558	0.089285	-0.23743	-0.28087	0.003824	-0.04725
C21orf7	NA	NA	-0.29583	0.406535	-0.10736	0.156456	-0.277	-0.18634
C21orf87	NA	NA	-0.31662	-0.23202	0.086123	-0.18901	-0.61874	0.165965
C21orf88	NA	NA	0.000196	-0.02602	-0.00215	0.015193	0.109184	-0.41397
C21orf90	NA	NA	-0.23057	-0.05865	-0.23708	0.111994	-0.07909	0.28075
C21orf91	NA	NA	-0.09453	-0.087	-0.39559	-0.16888	0.342238	0.492539
C21orf99	NA	NA	-0.23792	0.050312	-0.03081	0.265243	0.223834	-0.05569
C22orf13	NA	NA	0.36339	-0.18815	0.46348	-0.45986	0.166749	0.20608
C22orf15	NA	NA	0.134123	-0.04522	-0.10013	0.013638	0.426149	0.334509
C22orf23	NA	NA	-0.1538	-0.48889	0.155459	-0.03681	-0.55082	-0.57643
C22orf24	NA	NA	-0.17632	-0.14651	0.100614	0.062748	NA	NA
C22orf25	NA	NA	0.24169	-0.10884	0.407965	-0.26514	0.310639	0.510352
C22orf26	NA	NA	0.408985	0.206559	-0.14902	0.131366	NA	NA
C22orf28	NA	NA	-0.19317	-0.0243	-0.0109	-0.2061	NA	NA
C22orf29	NA	NA	0.524305	0.252136	0.528463	0.377913	NA	NA
C22orf31	NA	NA	-0.29205	-0.18525	0.066167	0.000732	NA	NA
C22orf32	NA	NA	0.115973	0.041056	0.077526	0.028635	NA	NA
C22orf39	NA	NA	0.096199	-0.20029	-0.07552	-0.42241	NA	NA
C22orf40	NA	NA	-0.12943	0.033626	-0.31444	-0.18136	NA	NA
C22orf9	NA	NA	0.196838	0.147919	0.174086	0.290048	0.353988	0.175587
C2CD2	NA	NA	0.200799	0.350884	0.136778	0.31409	NA	NA
C2CD2L	NA	NA	-0.00506	0.040233	0.10898	-0.30942	NA	NA
C2CD3	NA	NA	-0.15396	-0.07892	0.151303	0.283861	NA	NA
C2orf15	NA	NA	-0.08796	-0.01033	0.072382	-0.11406	NA	NA
C2orf16	NA	NA	0.050015	0.03807	-0.35907	0.026445	0.292562	-0.20634
C2orf18	NA	NA	0.444581	0.142721	0.366239	0.072706	0.611222	0.411777
C2orf24	NA	NA	0.492634	0.115175	0.222827	0.004462	0.339983	-0.04457
C2orf28	NA	NA	0.241638	-0.16907	-0.01607	-0.18451	0.262008	-0.25351
C2orf29	NA	NA	-0.00069	0.315996	0.105645	-0.05529	0.46757	0.203337
C2orf3	NA	NA	-0.12315	-0.47617	-0.02134	-0.13219	-0.13705	0.233386
C2orf34	NA	NA	-0.25476	0.026387	-0.16922	-0.23369	0.681922	0.180023
C2orf40	NA	NA	-0.08509	-0.07822	-0.17629	0.209634	NA	NA
C2orf42	NA	NA	-0.43907	0.197465	-0.3032	-0.34936	NA	NA
C2orf43	NA	NA	0.079496	-0.16852	-0.10615	-0.10955	NA	NA
C2orf44	NA	NA	0.179482	-0.0391	-0.02489	-0.0038	NA	NA
C2orf47	NA	NA	-0.08004	-0.01136	-0.23166	-0.41886	NA	NA
C2orf48	NA	NA	0.086575	0.126625	0.387742	-0.11604	NA	NA
C2orf49	NA	NA	-0.08977	-0.25817	-0.00411	-0.25607	NA	NA
C2orf50	NA	NA	0.135929	-0.11168	0.011861	0.291942	NA	NA
C2orf51	NA	NA	0.098052	-0.16674	-0.14528	-0.1227	NA	NA
C2orf53	NA	NA	-0.05031	-0.05254	-0.12498	0.052664	NA	NA
C2orf54	NA	NA	-0.0014	-0.001	0.041574	-0.55024	NA	NA
C2orf55	NA	NA	-0.0002	0.076004	0.181628	-0.15464	NA	NA
C2orf56	NA	NA	0.034169	-0.10797	0.059095	-0.0277	NA	NA

C2orf57	NA	NA	0.113992	-0.15265	-0.15974	0.045487	NA	NA
C2orf61	NA	NA	-0.12473	-0.05636	0.096673	0.017845	NA	NA
C2orf63	NA	NA	0.107798	-0.05765	-0.00878	0.292309	NA	NA
C2orf65	NA	NA	0.131611	-0.27091	0.127612	-0.12	NA	NA
C2orf66	NA	NA	-0.04574	-0.30909	-0.24203	-0.2006	NA	NA
C2orf68	NA	NA	-0.1146	-0.03103	0.24979	-0.23817	NA	NA
C2orf69	NA	NA	-0.23479	-0.33172	0.135086	-0.52071	NA	NA
C2orf7	NA	NA	0.190979	-0.16169	0.017971	-0.21163	-0.30316	-0.36964
C2orf70	NA	NA	-0.15271	0.149502	-0.13803	-0.24091	NA	NA
C2orf72	NA	NA	-0.12167	0.133023	-0.23321	-0.25458	NA	NA
C2orf73	NA	NA	0.146704	-0.27611	0.048661	-0.39384	NA	NA
C2orf74	NA	NA	-0.02529	-0.1329	-0.16829	0.041215	NA	NA
C2orf76	NA	NA	-0.0132	-0.25816	-0.33022	-0.14664	NA	NA
C2orf77	NA	NA	-0.2816	-0.00767	-0.16283	-0.14454	NA	NA
C2orf78	NA	NA	0.135688	-0.45656	0.155875	-0.12009	NA	NA
C2orf80	NA	NA	-0.07603	-0.20251	-0.2459	-0.00559	NA	NA
C2orf83	NA	NA	0.105004	-0.22526	-0.06075	0.049535	NA	NA
C3	0.01538	-0.00679	0.385716	0.644908	0.05956	0.76426	-0.27588	0.071348
C3AR1	-0.29546	-0.05858	0.200313	-0.27182	-0.33985	0.091387	-0.51566	0.104204
C3orf1	NA	NA	0.072839	-0.32202	-0.10479	-0.43318	-0.40353	0.038534
C3orf10	NA	NA	0.1124	0.035988	-0.16781	0.020709	-0.50529	-0.3125
C3orf14	NA	NA	0.292714	-0.07256	-0.06489	-0.3208	-0.26265	0.034704
C3orf15	NA	NA	-0.07053	0.145441	-0.2458	-0.22147	-0.21594	0.127589
C3orf17	NA	NA	-0.11731	-0.18938	-0.17336	0.479221	-0.41045	-0.41804
C3orf18	NA	NA	0.061634	-0.06223	-0.14659	-0.28432	-0.4191	-0.40222
C3orf19	NA	NA	-0.19784	-0.08583	-0.08659	-0.26563	0.249678	0.337287
C3orf20	NA	NA	-0.10715	-0.05413	-0.21159	-0.11812	-0.05397	-0.1723
C3orf21	NA	NA	-0.04803	0.021371	0.281598	-0.15917	-0.48926	-0.30438
C3orf22	NA	NA	0.039598	-0.03776	-0.04999	-0.39669	-0.62542	-0.24017
C3orf23	NA	NA	-0.31992	-0.27896	-0.28213	-0.42956	0.294522	0.170316
C3orf24	NA	NA	0.380976	0.065584	0.243394	0.008809	0.303039	0.184158
C3orf25	NA	NA	0.039896	-0.12174	-0.14985	-0.29111	0.585372	0.485102
C3orf26	NA	NA	-0.35197	0.27798	0.012883	-0.19509	0.163386	-0.0291
C3orf27	NA	NA	0.191855	-0.20502	0.200379	0.026099	-0.2102	-0.2366
C3orf30	NA	NA	-0.04228	0.121115	0.018533	0.147093	-0.2313	-0.17398
C3orf31	NA	NA	-0.36564	0.286706	0.265706	0.309702	-0.21714	-0.25517
C3orf32	NA	NA	-0.0637	-0.30348	0.288553	-0.17913	0.35988	0.457696
C3orf33	NA	NA	-0.26939	-0.2138	-0.08073	-0.12552	0.078448	-0.30494
C3orf34	NA	NA	0.115886	0.308507	-0.00881	-0.2527	-0.45528	-0.20583
C3orf35	NA	NA	-0.1039	-0.18151	0.179356	-0.25971	-0.37045	-0.23032
C3orf36	NA	NA	0.199576	-0.01658	-0.34835	0.435589	-0.4071	-0.37928
C3orf37	NA	NA	0.071849	0.423417	0.477645	0.300128	0.354599	0.059974
C3orf38	NA	NA	-0.49787	-0.33506	-0.31583	-0.57829	0.527457	0.527016
C3orf39	NA	NA	0.044313	0.017117	0.304358	-0.04758	0.187137	0.019495
C3orf42	NA	NA	-0.23639	0.06928	0.287579	0.366499	0.59258	0.637525
C3orf45	NA	NA	0.131364	0.043123	0.190288	-0.18037	-0.37392	-0.11326
C3orf52	NA	NA	-0.56474	0.015817	-0.11303	-0.31378	0.35483	0.341536
C3orf54	NA	NA	0.117654	0.268487	-0.1332	-0.1609	-0.32215	-0.09477
C3orf55	NA	NA	-0.08475	0.035244	-0.27114	0.092424	NA	NA
C3orf57	NA	NA	0.027586	0.285293	0.081146	-0.30844	0.271417	-0.39708
C3orf58	NA	NA	-0.30445	-0.06284	-0.18127	-0.12405	-0.14471	-0.37731
C3orf59	NA	NA	-0.02562	0.016882	-0.15427	-0.09079	-0.15768	0.385662

C3orf62	NA	NA	0.064832	-0.02527	0.105741	0.218634	0.072159	0.423677
C3orf63	NA	NA	-0.17208	-0.15765	0.251624	0.145787	0.047015	0.014004
C3orf64	NA	NA	-0.08458	-0.08742	-0.02793	0.011535	-0.54704	-0.26
C3orf70	NA	NA	-0.38892	-0.23498	-0.02965	-0.18049	NA	NA
C3orf71	NA	NA	0.147268	-0.3383	0.091457	-0.08669	NA	NA
C3orf75	NA	NA	0.03439	-0.02744	-0.12756	-0.39582	NA	NA
C4B	-0.27807	-0.23884	0.232164	0.42627	0.242405	0.588634	NA	NA
C4BPA	0.032093	0.058426	-0.24605	-0.38229	-0.0277	-0.44693	-0.19127	-0.07612
C4BPB	-0.25111	-0.02132	-0.20961	0.032955	-0.15292	-0.33653	-0.12941	0.14258
C4orf14	NA	NA	-0.01339	0.013247	-0.16638	-0.01323	0.403261	0.116933
C4orf17	NA	NA	0.463768	-0.25299	0.062955	-0.06708	-0.29941	-0.2439
C4orf19	NA	NA	-0.22978	-0.07415	-0.09842	-0.4295	NA	NA
C4orf22	NA	NA	0.246707	-0.01096	0.128939	0.02516	NA	NA
C4orf26	NA	NA	0.047962	-0.31934	-0.09132	0.268503	NA	NA
C4orf27	NA	NA	-0.09398	-0.01919	-0.184	-0.08974	NA	NA
C4orf29	NA	NA	-0.17045	-0.10831	-0.02083	-0.25968	NA	NA
C4orf3	NA	NA	-0.10639	-0.25221	-0.31246	0.164158	NA	NA
C4orf32	NA	NA	-0.18339	-0.11033	-0.07136	-0.13473	-0.40263	-0.12209
C4orf33	NA	NA	0.033336	-0.09623	-0.25202	-0.43316	NA	NA
C4orf34	NA	NA	-0.08726	-0.00423	-0.20728	-0.18161	-0.54181	-0.37969
C4orf36	NA	NA	-0.05776	-0.29768	-0.27831	-0.34389	0.228219	0.188788
C4orf37	NA	NA	-0.22204	-0.01494	0.033926	-0.14296	NA	NA
C4orf45	NA	NA	0.000782	-0.52977	0.292513	0.148937	NA	NA
C4orf46	NA	NA	-0.38343	0.145072	0.234803	-0.2646	NA	NA
C4orf47	NA	NA	-0.22644	-0.03462	-0.40259	-0.04769	NA	NA
C4orf48	NA	NA	0.227728	-0.0752	0.021296	-0.13599	NA	NA
C4orf6	NA	NA	0.059611	-0.03224	-0.12305	0.049427	0.372692	0.242725
C4orf7	NA	NA	0.000261	0.655606	-0.08166	0.404957	-0.1715	-0.01772
C5	-0.13611	0.031798	-0.18345	0.072255	0.015419	0.075994	-0.12824	0.046317
C5AR1	-0.49027	-0.01879	0.178495	-0.03138	-0.09559	-0.06067	-0.37726	0.125402
C5orf13	NA	NA	-0.28698	-0.15306	-0.15487	-0.05161	0.141502	-0.14055
C5orf15	NA	NA	0.179418	-0.13683	0.032469	-0.19695	-0.08091	0.402
C5orf20	NA	NA	0.100155	0.324773	-0.30915	0.421756	-0.73572	0.053837
C5orf22	NA	NA	-0.17538	-0.16395	-0.1719	-0.03102	NA	NA
C5orf24	NA	NA	0.20554	-0.23058	-0.19003	-0.04588	NA	NA
C5orf25	NA	NA	-0.27422	-0.27129	0.247185	-0.01367	NA	NA
C5orf27	NA	NA	0.272803	0.021004	0.225023	-0.15202	NA	NA
C5orf28	NA	NA	-0.04074	-0.28826	0.025042	0.10082	-0.59711	-0.57784
C5orf30	NA	NA	-0.19118	-0.12664	-0.01371	-0.4454	NA	NA
C5orf34	NA	NA	-0.41909	0.049116	0.172217	-0.3444	0.215698	0.200129
C5orf36	NA	NA	-0.12969	0.148337	-0.01665	-0.07723	-0.07139	-0.484
C5orf38	NA	NA	0.175031	0.056413	-0.16093	-0.26616	NA	NA
C5orf4	NA	NA	0.098194	-0.03868	0.177248	-0.33993	-0.07449	-0.1184
C5orf42	NA	NA	0.079472	-0.33857	-0.21385	0.338457	NA	NA
C5orf43	NA	NA	-0.34119	-0.09847	-0.24868	-0.38722	NA	NA
C5orf44	NA	NA	-0.08448	-0.39407	-0.07672	0.028876	NA	NA
C5orf45	NA	NA	0.1005	0.162026	0.185296	0.372317	NA	NA
C5orf46	NA	NA	0.163283	-0.27823	-0.0055	-0.05387	NA	NA
C5orf47	NA	NA	0.104126	-0.16069	-0.45424	0.387569	NA	NA
C5orf51	NA	NA	-0.07241	-0.47299	0.103565	0.1949	NA	NA
C6	0.194332	-0.29261	-0.4274	-0.12752	-0.28761	-0.35937	0.126373	0.489372
C6orf1	NA	NA	0.282004	0.20964	0.064993	-0.18026	0.081577	-0.16624

C6orf10	NA	NA	0.206972	-0.07616	-0.14795	0.067114	-0.40715	-0.73951
C6orf105	NA	NA	-0.28925	0.14349	-0.16683	-0.37144	0.056145	0.24186
C6orf106	NA	NA	0.028586	0.067562	0.125912	0.019375	0.259922	0.334475
C6orf108	NA	NA	0.193712	0.006815	0.071205	-0.10352	0.27296	0.355966
C6orf114	NA	NA	-0.25776	-0.1349	0.152377	-0.26125	-0.21496	0.169033
C6orf118	NA	NA	-0.03996	0.062268	-0.03289	0.16712	0.072485	0.475777
C6orf120	NA	NA	-0.24735	-0.04155	-0.0132	-0.31496	-0.19796	-0.13875
C6orf125	NA	NA	0.024748	0.100814	-0.14266	-0.22257	0.423856	-0.07802
C6orf129	NA	NA	-0.0715	0.146134	0.088838	-0.36854	0.497459	0.046995
C6orf130	NA	NA	0.025529	-0.00047	-0.02401	-0.36027	0.484508	0.348435
C6orf134	NA	NA	-0.12137	-0.00552	0.109861	0.350122	0.722303	0.640204
C6orf136	NA	NA	0.064073	-0.01557	0.063465	0.134407	-0.31659	-0.20221
C6orf141	NA	NA	-0.33983	-0.2584	-0.10548	0.10493	NA	NA
C6orf142	NA	NA	-0.06625	-0.15111	0.116365	-0.54685	-0.09602	0.378402
C6orf145	NA	NA	0.180196	0.024985	0.20232	0.126568	-0.65976	-0.18142
C6orf146	NA	NA	0.208758	-0.35091	-0.09092	-0.28264	0.25523	0.399269
C6orf15	NA	NA	0.121208	-0.2516	-0.07007	-0.00647	0.37141	-0.29682
C6orf150	NA	NA	-0.14148	0.365472	0.002725	0.199387	-0.09135	0.339408
C6orf153	NA	NA	0.023985	-0.27489	-0.13841	-0.0668	-0.45019	-0.45811
C6orf154	NA	NA	-0.18861	0.157068	-0.21706	0.014347	0.113989	-0.22937
C6orf162	NA	NA	-0.12144	-0.27293	0.021741	-0.05044	0.294435	-0.0697
C6orf165	NA	NA	-0.2743	-0.04311	-0.24975	-0.15431	0.042082	0.186917
C6orf167	NA	NA	-0.53614	0.019365	0.175806	-0.15855	-0.05825	-0.08847
C6orf168	NA	NA	-0.22188	-0.1278	0.109103	0.050428	-0.2771	-0.29971
C6orf170	NA	NA	0.216733	-0.125	0.429988	0.288124	-0.5779	-0.53983
C6orf182	NA	NA	-0.08928	-0.24612	-0.20073	-0.41626	-0.15454	0.089638
C6orf192	NA	NA	-0.09651	-0.33453	-0.00477	-0.05696	0.130335	0.360614
C6orf195	NA	NA	0.048355	-0.16633	0.163815	0.406441	0.436549	0.04977
C6orf201	NA	NA	0.126123	0.069312	-0.38712	-0.15358	-0.24437	0.13357
C6orf203	NA	NA	-0.18641	-0.17159	-0.00787	-0.16396	-0.39814	-0.58617
C6orf204	NA	NA	-0.19637	0.449003	-0.40525	0.451084	-0.71155	-0.43062
C6orf211	NA	NA	-0.0542	-0.4451	0.040476	0.015987	-0.19741	-0.25385
C6orf223	NA	NA	0.039859	-0.00262	0.066336	-0.10781	NA	NA
C6orf225	NA	NA	0.144991	-0.25028	-0.22149	-0.13913	NA	NA
C6orf25	NA	NA	-0.18604	-0.13413	0.072397	-0.03869	0.581991	0.59446
C6orf26	NA	NA	-0.20314	0.262458	0.238388	-0.03151	0.054863	0.210248
C6orf27	NA	NA	0.171781	-0.06916	0.046909	-0.02916	-0.25834	0.000381
C6orf35	NA	NA	-0.50586	-0.18544	-0.19831	-0.59171	-0.56727	-0.45115
C6orf47	NA	NA	0.193355	0.11047	0.343339	0.098933	-0.48904	-0.38359
C6orf48	NA	NA	0.118083	0.230837	0.040315	0.198897	0.156525	-0.04803
C6orf52	NA	NA	-0.26091	-0.22754	-0.16333	-0.40349	NA	NA
C6orf57	NA	NA	-0.10088	-0.27938	-0.11653	-0.35863	0.196331	-0.3108
C6orf58	NA	NA	-0.15487	0.015686	-0.40074	-0.3505	NA	NA
C6orf62	NA	NA	-0.30554	0.244125	-0.02602	-0.03126	-0.629	-0.34055
C6orf64	NA	NA	-0.16079	-0.02299	0.122725	-0.38684	0.309853	0.154674
C6orf70	NA	NA	-0.30379	-0.06488	0.051526	-0.16464	0.230655	0.565499
C6orf72	NA	NA	0.15195	-0.43368	0.061874	-0.26826	-0.56954	-0.61386
C6orf81	NA	NA	-0.1628	0.454018	-0.25753	-0.11628	-0.04798	-0.24176
C6orf89	NA	NA	-0.07443	0.125507	0.24771	0.056705	0.855459	0.327355
C7	0.062404	-0.39643	-0.17558	0.022384	-0.32113	0.142371	-0.26215	-0.44819
C7orf10	NA	NA	-0.05276	-0.33264	-0.24971	-0.11587	-0.15747	-0.01795
C7orf11	NA	NA	-0.24691	-0.1083	-0.02061	-0.45822	-0.34415	-0.46243

C7orf13	NA	NA	-0.20153	0.273175	0.173416	-0.01285	NA	NA
C7orf16	NA	NA	-0.00881	0.23582	-0.30783	0.343801	0.468194	0.489283
C7orf23	NA	NA	0.182991	0.477593	0.262566	0.368821	NA	NA
C7orf25	NA	NA	-0.11793	-0.12689	-0.06129	-0.45805	-0.2959	-0.2486
C7orf26	NA	NA	0.095927	0.039841	0.223046	0.08668	0.452988	0.451995
C7orf27	NA	NA	0.363506	0.212618	0.380337	0.26686	-0.32788	-0.41611
C7orf29	NA	NA	-0.18797	0.152639	0.219089	0.150683	0.327439	0.241373
C7orf30	NA	NA	-0.14413	-0.02735	-0.14758	-0.41124	0.027302	-0.07675
C7orf31	NA	NA	-0.30693	-0.05895	-0.22003	-0.13288	0.268064	0.208701
C7orf33	NA	NA	-0.04603	-0.26102	-0.33445	-0.1464	-0.23078	0.180858
C7orf34	NA	NA	-0.05438	-0.27754	0.178423	0.246116	-0.24708	-0.16325
C7orf36	NA	NA	-0.01365	-0.32282	0.089367	-0.01802	-0.25597	-0.38668
C7orf41	NA	NA	0.367495	-0.25811	0.159818	0.261188	NA	NA
C7orf43	NA	NA	0.130443	0.052198	0.079089	-0.22263	NA	NA
C7orf44	NA	NA	0.095146	0.220536	0.052713	0.113057	0.08146	0.018706
C7orf45	NA	NA	-0.03017	-0.0651	-0.44457	0.031716	NA	NA
C7orf49	NA	NA	-0.0698	0.097714	-0.08743	-0.14139	NA	NA
C7orf50	NA	NA	0.190035	0.167202	0.256028	-0.13713	NA	NA
C7orf53	NA	NA	-0.25607	0.059612	-0.07148	-0.20279	NA	NA
C7orf57	NA	NA	0.247158	0.093671	-0.19108	-0.00175	NA	NA
C7orf60	NA	NA	-0.19784	-0.02649	-0.23597	-0.08678	NA	NA
C7orf61	NA	NA	0.042951	0.111434	0.112009	0.166063	NA	NA
C7orf62	NA	NA	0.096407	-0.08391	0.190126	-0.1944	NA	NA
C7orf63	NA	NA	-0.18422	-0.17096	0.105847	-0.27771	NA	NA
C8A	0.218009	0.23851	-0.01085	0.074553	-0.03346	0.32761	-0.16785	-0.2127
C8B	0.095981	0.592332	0.064075	-0.17895	0.086395	-0.14464	0.029207	-0.00863
C8G	-0.47429	-0.04033	-0.13762	-0.41416	0.038035	-0.38994	-0.26252	0.279213
C8orf22	NA	NA	0.252152	-0.21639	0.53791	-0.22961	0.255343	-0.02781
C8orf31	NA	NA	0.01369	0.039486	0.11816	0.273319	-0.30746	0.286778
C8orf33	NA	NA	0.107298	0.005949	0.066691	0.080076	0.281442	0.062418
C8orf34	NA	NA	0.041788	-0.12418	-0.35788	-0.33165	0.460122	0.517931
C8orf37	NA	NA	-0.01553	0.0262	-0.24646	0.097368	-0.57222	-0.62536
C8orf38	NA	NA	0.071751	-0.1475	0.002316	-0.26029	0.088446	-0.38373
C8orf4	NA	NA	-0.33485	0.057573	-0.11579	-0.1879	-0.04842	0.017316
C8orf40	NA	NA	0.101247	-0.27684	0.00253	-0.34268	-0.00011	-0.26778
C8orf41	NA	NA	0.010732	0.391059	-0.08048	-0.46178	-0.12132	-0.36082
C8orf42	NA	NA	-0.14856	0.109252	-0.33994	-0.29601	-0.10297	0.363706
C8orf44	NA	NA	0.18287	-0.1725	0.059903	0.00573	0.266211	0.220658
C8orf45	NA	NA	0.09337	-0.03967	0.186678	0.004989	-0.54411	-0.61762
C8orf46	NA	NA	0.114317	-0.15932	-0.08012	0.028307	-0.20999	0.0606
C8orf47	NA	NA	0.118462	0.275094	-0.09434	-0.39469	0.072462	0.162809
C8orf48	NA	NA	0.009125	-0.2201	-0.20803	-0.34557	-0.30821	-0.19144
C8orf55	NA	NA	0.311925	0.128833	0.078568	-0.14202	0.011284	-0.19549
C8orf58	NA	NA	0.173822	0.161988	-0.33411	-0.06765	0.291336	0.415394
C8orf59	NA	NA	0.137841	-0.14586	0.11722	0.238015	NA	NA
C8orf73	NA	NA	0.079906	-0.00808	0.047169	-0.25476	NA	NA
C8orf74	NA	NA	NA	NA	NA	NA	0.456794	0.161428
C8orf76	NA	NA	0.070102	-0.23102	-0.17136	-0.23025	0.279283	0.282296
C8ORFK29	NA	NA	0.106447	-0.13392	0.137366	-0.12298	NA	NA
C9	0.069412	-0.11754	-0.04114	0.007642	-0.43541	-0.07621	-0.18591	-0.45442
C9orf100	NA	NA	-0.12118	0.236869	0.036444	-0.02532	0.336355	0.15513
C9orf102	NA	NA	-0.02584	-0.62512	-0.11354	-0.34779	-0.26655	0.202156

C9orf103	NA	NA	0.175785	-0.38438	-0.03596	-0.43707	0.016904	0.301745
C9orf106	NA	NA	NA	NA	NA	NA	-0.20396	0.226382
C9orf11	NA	NA	-0.11707	-0.17218	-0.12151	-0.43408	-0.02556	0.077479
C9orf114	NA	NA	0.223948	0.208695	0.291451	0.133941	0.463975	0.290877
C9orf116	NA	NA	-0.24972	-0.30946	-0.262	-0.39435	-0.0969	-0.11836
C9orf117	NA	NA	0.024352	0.071139	0.365354	-0.0163	0.088615	0.26434
C9orf123	NA	NA	-0.20309	-0.08954	-0.07015	-0.1375	0.112652	0.154836
C9orf125	NA	NA	-0.46033	0.147564	-0.07328	-0.42165	0.132495	0.01734
C9orf128	NA	NA	0.105323	0.073069	-0.09098	-0.34217	-0.34871	-0.08058
C9orf129	NA	NA	-0.14136	0.041096	0.099567	-0.08388	NA	NA
C9orf131	NA	NA	0.166342	0.190482	0.057304	0.304444	NA	NA
C9orf135	NA	NA	-0.0759	-0.37032	0.153867	-0.14637	NA	NA
C9orf140	NA	NA	0.16471	0.333991	0.430651	0.023432	0.517699	0.273729
C9orf142	NA	NA	0.255592	0.245639	0.242138	0.025841	0.407074	0.33928
C9orf150	NA	NA	0.002608	-0.33122	0.054306	-0.08144	-0.24859	-0.20628
C9orf152	NA	NA	NA	NA	NA	NA	0.196922	-0.05422
C9orf153	NA	NA	-0.03322	-0.03662	0.044439	0.275959	-0.17571	-0.17343
C9orf156	NA	NA	-0.24103	-0.19858	-0.24501	-0.30949	-0.26694	0.317204
C9orf16	NA	NA	0.358436	0.158821	0.164719	-0.33758	0.407465	-0.01141
C9orf163	NA	NA	0.153332	-0.14741	0.047103	-0.05255	NA	NA
C9orf21	NA	NA	0.071853	0.004783	-0.15494	0.155863	-0.23017	-0.02017
C9orf23	NA	NA	-0.00306	-0.04765	-0.01637	-0.23197	0.330313	0.232377
C9orf24	NA	NA	-0.02263	-0.11727	-0.00139	-0.26377	-0.20543	0.265787
C9orf25	NA	NA	0.263114	-0.13162	0.070051	-0.10619	-0.18496	0.074965
C9orf3	NA	NA	-9.4E-05	-0.01494	-0.27622	0.072369	0.019631	-0.05935
C9orf30	NA	NA	-0.09264	0.171758	-0.01064	-0.24823	-0.47916	-0.24237
C9orf37	NA	NA	-0.02384	0.387805	0.208399	-0.26274	-0.33938	-0.07243
C9orf4	NA	NA	-0.21419	-0.19108	-0.02568	-0.27297	-0.18763	0.084294
C9orf40	NA	NA	-0.33202	0.096646	-0.22284	-0.28897	0.033729	-0.04153
C9orf41	NA	NA	-0.32001	0.063955	-0.07624	-0.29262	0.170693	0.38078
C9orf43	NA	NA	-0.04375	0.10616	0.031336	-0.00341	-0.06287	0.147828
C9orf45	NA	NA	-0.13879	0.006488	-0.01594	0.306145	-0.17145	0.181386
C9orf46	NA	NA	-0.15	-0.3253	0.032492	-0.44855	0.1125	0.156188
C9orf47	NA	NA	0.282004	-0.01499	0.164575	0.265246	-0.12566	-0.01865
C9orf5	NA	NA	0.156112	-0.4244	0.16096	0.201539	-0.36515	-0.20378
C9orf50	NA	NA	0.379538	-0.30697	0.011253	0.132202	-0.38021	-0.34761
C9orf53	NA	NA	-0.00731	-0.06251	-0.30001	-0.13668	NA	NA
C9orf57	NA	NA	-0.13615	0.42079	0.303465	0.353098	NA	NA
C9orf6	NA	NA	-0.2403	-0.25276	-0.03987	-0.49258	0.470762	0.556175
C9orf62	NA	NA	0.08505	-0.12341	-0.18951	0.208079	NA	NA
C9orf64	NA	NA	0.094258	0.272495	0.100067	-0.00618	-0.46409	0.067338
C9orf66	NA	NA	-0.14117	-0.27813	-0.14845	-0.06654	-0.27645	-0.13591
C9orf68	NA	NA	-0.04708	-0.02891	0.042409	-0.24512	0.381319	0.192306
C9orf69	NA	NA	0.283917	0.204388	0.248441	0.176427	NA	NA
C9orf7	NA	NA	0.223208	0.056438	0.229898	-0.259	0.111674	0.025234
C9orf71	NA	NA	0.212645	-0.02209	0.157345	-0.21972	0.040578	0.332515
C9orf72	NA	NA	-0.29039	-0.10875	-0.19323	-0.38266	-0.42524	0.171536
C9orf78	NA	NA	0.018548	0.287034	0.068004	0.142024	-0.53945	-0.3029
C9orf79	NA	NA	-0.18386	-0.07599	0.108117	0.042188	0.46102	0.449221
C9orf80	NA	NA	-0.23345	-0.1778	-0.06033	-0.33094	-0.57771	-0.592
C9orf82	NA	NA	-0.34593	-0.07776	-0.13218	-0.16241	-0.15103	0.0836
C9orf84	NA	NA	0.00281	0.052466	-0.10476	-0.30787	0.668823	0.186468

C9orf85	NA	NA	-0.12676	-0.19202	-0.19531	-0.25611	-0.55244	-0.46997
C9orf86	NA	NA	0.313902	0.17436	0.387738	0.116218	0.072057	-0.00912
C9orf89	NA	NA	0.119406	-0.28775	-0.12158	0.06099	-0.03795	0.040435
C9orf9	NA	NA	0.167894	-0.19414	0.007005	-0.1542	-0.08237	-0.06437
C9orf91	NA	NA	-0.06406	0.42779	0.168978	-0.10485	-0.02312	0.288157
C9orf93	NA	NA	-0.17919	-0.04646	-0.28177	-0.19662	-0.00585	0.165628
C9orf95	NA	NA	0.089916	-0.00606	-0.20138	-0.22534	-0.35714	-0.13
C9orf96	NA	NA	NA	NA	NA	NA	0.371755	-0.11245
C9orf98	NA	NA	-0.05358	0.550379	-0.30899	0.297736	-0.17591	-0.15981
CA1	-0.02	-0.04446	0.005907	-0.39162	-0.06467	-0.14964	0.050636	-0.39255
CA10	0.378859	0.619433	0.163914	-0.1271	-0.04653	0.035984	-0.13796	0.093079
CA11	0.38599	0.198792	0.100394	-0.11534	0.0311	0.294097	0.056293	0.094543
CA12	-0.18029	0.131989	-0.00142	-0.30807	-0.10278	0.056433	-0.14093	-0.19454
CA13	0.141774	0.349052	0.299946	-0.01784	-0.07492	-0.30945	0.0506	0.114524
CA14	-0.33445	-0.29392	-0.30068	-0.20684	-0.3297	-0.13096	0.236269	-0.03076
CA2	-0.40976	-0.07291	-0.17696	-0.04897	-0.23498	-0.24348	-0.10995	-0.43104
CA3	0.116945	0.354194	0.023922	0.06269	-0.04081	-0.62243	0.308095	0.431203
CA4	-0.04362	0.192728	0.015013	-0.23152	0.053814	-0.19445	0.023435	-0.35568
CA5A	0.145811	0.496022	-0.15016	-0.07144	-0.0649	0.214969	-0.35497	0.042408
CA5B	0.115209	-0.09074	-0.17643	-0.2409	0.083528	0.396893	-0.45292	0.039232
CA6	-0.17548	-0.16396	-0.02242	-0.22106	-0.225	-0.18785	0.00298	-0.07147
CA7	0.043563	0.164037	-0.11344	-0.29554	-0.08697	-0.14373	-0.46249	-0.31421
CA8	-0.25812	-0.1879	-0.43414	0.257809	-0.17592	-0.41557	0.172718	-0.37331
CA9	-0.3205	0.160638	0.057634	-0.18372	0.176287	-0.22724	0.17268	0.47558
CAB39	-0.1378	-0.48139	-0.11784	-0.33427	-0.21734	-0.01576	-0.16118	0.030007
CAB39L	0.091361	-0.05893	0.543845	-0.19794	-0.03103	-0.20834	0.277012	-0.33737
CABC1	0.011666	-0.33783	0.053395	0.162227	0.232749	0.393915	0.217175	-0.09997
CABIN1	0.021477	0.634642	0.244096	0.233142	0.209328	-0.05188	0.433291	0.219683
CABLES1	0.039079	0.052533	0.118687	-0.40577	0.105559	-0.40249	-0.24631	-0.25433
CABLES2	0.173971	0.164862	-0.28154	0.090093	0.360425	0.020165	0.326117	0.144993
CABP1	-0.04043	0.420091	-0.17309	-0.16325	0.337381	-0.34972	0.097491	0.004278
CABP2	-0.07969	0.128948	0.389021	-0.07988	-0.02327	0.215164	-0.02338	0.35141
CABP4	0.146748	0.399208	0.140489	-0.01514	0.044291	-0.11476	-0.07067	-0.02744
CABP5	-0.11113	0.17368	0.129	-0.24478	-0.03861	-0.24049	-0.28537	-0.21762
CABP7	0.147628	-0.02381	-0.02689	0.189841	-0.25921	-0.04992	-0.0657	0.354484
CABYR	0.094094	0.192871	0.031142	-0.01066	-0.13438	-0.25308	-0.21101	0.042529
CACHD1	-0.08545	-0.64463	0.196307	0.086094	-0.14811	-0.25485	0.578245	0.204514
CACNA1A	0.013246	0.385285	0.434395	-0.19361	0.048389	-0.08189	-0.09039	0.263926
CACNA1B	-0.24431	-0.22823	0.021401	-0.09075	-0.08495	-0.10411	-0.61954	-0.58513
CACNA1C	-0.02193	-0.20259	0.070477	-0.00897	0.161304	0.253749	-0.27656	-0.31212
CACNA1D	0.065816	0.768833	-0.06758	0.116186	0.407682	-0.04127	0.520787	0.475384
CACNA1E	-0.0595	-0.24967	-0.10688	-0.01922	-0.20409	0.147328	-0.38601	0.065014
CACNA1F	-0.08065	0.189117	0.162342	0.220827	-0.24561	-0.29137	-0.04581	0.414989
CACNA1G	-0.21798	-0.05604	-0.24226	-0.05595	-0.08989	0.133009	0.198988	-0.05804
CACNA1H	0.012272	-0.13688	-0.12726	-0.18585	0.205213	0.183374	-0.21039	-0.29648
CACNA1I	0.416515	0.765819	0.059439	0.100819	-0.07573	0.349944	-0.38152	0.146125
CACNA1S	-0.06009	0.317179	0.168824	-0.01063	0.016025	-0.01904	-0.06043	-0.21488
CACNA2D1	-0.00725	0.216376	0.085574	-0.1681	0.114427	0.245967	0.130038	0.080788
CACNA2D2	0.09622	0.455682	-0.10382	0.256208	-0.01544	0.262878	0.010437	-0.34494
CACNA2D3	0.003414	0.071719	-0.11701	0.083643	-0.30796	-0.19228	-0.59686	-0.0808
CACNA2D4	0.055188	-0.22185	0.245948	-0.11976	-0.08948	-0.41776	-0.0026	0.118062
CACNB1	0.016478	-0.34164	-0.01034	-0.1997	0.010433	-0.27208	-0.35977	-0.25044

CACNB2	-0.10643	0.083713	0.011707	-0.40413	-0.0269	0.218184	0.010399	-0.24884
CACNB3	-0.54569	-0.14859	0.09497	-0.09274	0.094643	0.03606	0.10138	0.070138
CACNB4	0.023608	0.071632	0.130503	-0.08905	-0.04266	0.0638	0.285	-0.39355
CACNG1	-0.07042	0.210111	0.197312	-0.15239	0.267455	-0.14292	-0.07624	-0.19146
CACNG2	0.234615	0.269221	0.045185	-0.14525	-0.20218	-0.04386	-0.06921	0.2883
CACNG3	-0.0847	0.088514	0.241612	0.205919	0.28435	-0.09071	-0.11553	-0.17795
CACNG4	-0.07628	0.474449	0.343994	-0.24542	0.273461	-0.09145	0.30696	0.011105
CACNG5	0.306541	0.651789	0.288511	-0.03	0.16027	0.195969	0.339894	-0.19846
CACNG6	-0.23172	-0.18975	0.13669	-0.22767	0.021987	0.152874	-0.31268	-0.05199
CACNG7	0.291373	0.213821	0.119174	-0.03708	0.135712	0.234437	-0.00398	-0.12376
CACNG8	0.393002	0.450213	-0.22664	0.006163	0.020571	-0.19726	0.374776	0.401812
CACYBP	-0.3121	-0.65864	-0.33671	0.350008	-0.21055	-0.31543	-0.51501	-0.44615
CAD	0.429149	0.334245	0.107576	0.394184	0.376034	0.63251	0.557903	0.411301
CADM1	-0.1644	0.041851	0.266334	0.241162	-0.37857	0.171511	NA	NA
CADM2	0.610028	-0.27236	-0.17516	-0.26182	0.364548	0.000657	NA	NA
CADM3	0.246706	0.077309	-0.08917	0.039983	0.049314	0.107205	NA	NA
CADM4	0.003595	-0.01564	0.087259	-0.4751	0.224967	-0.24306	NA	NA
CADPS	0.343537	0.504631	0.14202	0.090071	0.332273	0.116263	0.203226	0.084646
CADPS2	0.138934	0.216994	0.319405	0.382352	-0.25257	-0.5647	-0.04886	0.018156
CAGE1	-0.004	0.636486	NA	NA	NA	NA	0.265484	0.369873
CALB1	0.020043	0.361055	-0.19456	0.530342	-0.24833	0.263838	-0.07981	0.003719
CALB2	-0.27179	0.118559	-0.06646	0.24105	-0.20682	-0.12961	0.128473	-0.30071
CALCA	-0.09513	0.23529	-0.01481	-0.08822	0.163749	0.059441	0.090272	0.354508
CALCB	0.12653	0.248071	NA	NA	NA	NA	0.254571	0.103994
CALCOCO1	0.208282	0.032474	0.056424	0.057088	0.195674	0.261353	0.207347	0.183167
CALCOCO2	-0.29587	0.284754	0.164632	-0.19064	-0.06083	-0.31996	0.018433	-0.20876
CALCR	0.262309	0.739639	-0.12583	-0.21626	0.0664	-0.20525	-0.07	-0.20751
CALCRL	-0.22821	-0.25648	0.051653	-0.16145	-0.12316	-0.18228	-0.26219	-0.12205
CALD1	-0.28322	-0.17912	-0.04521	-0.11591	-0.05615	0.154898	-0.41168	-0.49903
CALHM1	NA	NA	-0.14693	0.237398	0.086807	0.052032	NA	NA
CALHM2	NA	NA	0.182307	0.279171	-0.13213	0.345747	NA	NA
CALHM3	NA	NA	-0.17212	-0.21642	-0.12671	0.179302	NA	NA
CALM1	-0.59911	-0.32584	-0.30391	-0.32714	-0.05057	-0.28723	0.1043	0.174015
CALM2	0.032147	-0.18717	-0.09225	-0.23517	0.193716	-0.08278	-0.36437	-0.46124
CALM3	-0.02813	0.058014	0.112129	0.164964	-0.0105	9.59E-05	-0.28092	-0.2578
CALML3	0.00172	0.731952	0.007815	-0.37891	0.068319	0.079597	-0.22636	-0.25878
CALML4	-0.0968	0.114131	0.030656	-0.14494	0.018001	-0.38579	0.126552	-0.03226
CALML5	-0.01636	-0.04067	-0.17772	0.055199	-0.12459	0.182383	-0.38273	-0.46697
CALML6	-0.30225	0.09389	-0.0509	-0.11099	0.143957	-0.25531	-0.28244	-0.25153
CALN1	-0.15739	0.671229	0.250248	-0.19893	0.0061	0.063449	0.12524	0.164524
CALR	0.153345	0.348637	0.023022	0.315249	0.174105	-0.48462	-0.41066	-0.38131
CALR3	-0.12319	-0.00911	0.156721	0.027085	-0.24381	-0.12252	0.598861	0.433655
CALU	-0.24097	-0.17121	0.007526	0.172037	-0.1067	-0.00584	0.05832	-0.05965
CALY	NA	NA	0.247844	-0.07418	-0.10899	-0.23154	NA	NA
CAMK1	0.216429	0.138636	0.349688	-0.0345	0.059763	0.162749	-0.07999	0.183381
CAMK1D	-0.1018	-0.27755	-0.14869	-0.28829	0.149185	0.308425	-0.15857	-0.1606
CAMK1G	-0.19275	0.30253	0.16984	0.029735	-0.17082	0.20904	-0.44552	-0.46386
CAMK2A	-0.45607	0.178898	0.272354	-0.23405	-0.0111	0.273451	-0.49582	-0.249
CAMK2B	0.022189	0.615052	-0.31672	0.131668	-0.31627	0.135796	0.042819	0.302037
CAMK2D	-0.34806	-0.49028	-0.12799	-0.05752	0.018545	0.083333	-0.15379	-0.05513
CAMK2G	-0.15506	-0.18183	0.037346	-0.17749	0.243415	-0.41619	-0.35764	-0.29186
CAMK2N1	0.194539	0.011816	0.246067	-0.1677	0.412543	-0.06189	0.441158	-0.09801

CAMK2N2	-0.21625	-0.0601	0.029166	0.080184	-0.1198	0.065597	0.295887	0.247949
CAMK4	-0.07665	-0.01147	0.080521	0.457252	-0.21071	0.080269	-0.32707	-0.47854
CAMKK1	-0.30302	0.059686	0.027568	0.008816	0.278146	0.235528	0.293977	0.415841
CAMKK2	0.041845	-0.22151	0.033529	-0.1308	-0.06217	-0.04039	-0.14672	-0.372
CAMKV	0.323716	-0.13153	0.05064	0.128097	-0.06857	0.192917	0.12632	-0.18793
CAMLG	0.403743	0.16127	0.46874	-0.25994	0.294921	-0.11146	NA	NA
CAMP	-0.06867	-0.00505	0.130792	0.654922	-0.10444	-0.05976	-0.19967	0.202284
CAMSAP1	0.21428	-0.19593	-0.03723	-0.40429	0.320521	0.400714	-0.15183	-0.05474
CAMSAP1L	-0.13713	-0.16437	-0.05501	-0.29122	-0.22599	0.225428	-0.08101	-0.07484
CAMTA1	0.427115	-0.21005	0.261287	-0.23852	0.357713	0.0455	0.433738	0.056631
CAMTA2	-0.33344	0.398045	0.04814	0.013745	0.17408	0.175927	-0.22094	-0.03796
CAND1	-0.10936	-0.42238	-0.41037	-0.24494	-0.08846	-0.03131	-0.44706	-0.41986
CAND2	NA	NA	-0.08928	-0.01607	-0.29671	0.217057	-0.45631	-0.53555
CANT1	-0.20449	0.054323	0.327255	0.17302	-0.02348	-0.0239	0.557292	0.228539
CANX	-0.12356	-0.56907	0.146847	0.108562	0.095006	0.108121	-0.21502	0.267437
CAP1	-0.58688	-0.41432	0.049831	0.028078	-0.07211	0.108305	0.283276	0.471269
CAP2	-0.31131	-0.23078	0.146846	-0.20396	-0.23735	-0.17799	-0.22038	-0.33857
CAPG	-0.24504	0.065492	-0.03854	0.189424	-0.15215	-0.05312	-0.18795	-0.24204
CAPN1	0.022531	0.159001	0.213859	0.056843	0.206896	0.157859	-0.40247	-0.43311
CAPN10	0.435398	0.684488	0.377468	0.067137	0.442587	-0.22528	0.233326	-0.41638
CAPN11	-0.26666	0.261176	0.057146	-0.24572	-0.1268	-0.04146	-0.49878	-0.08205
CAPN12	0.044155	-0.0648	-0.11771	0.273804	-0.11063	0.324535	0.281486	0.282585
CAPN13	0.440298	-0.01853	0.199787	-0.08686	0.138652	-0.11432	0.036124	-0.03873
CAPN14	NA	NA	-0.04074	-0.13178	-0.13667	0.32775	NA	NA
CAPN2	-0.1307	0.24496	-0.21391	-0.09901	0.138975	-0.22274	-0.49702	-0.34219
CAPN3	0.290389	0.3115	-0.03131	0.066565	0.034161	0.412497	-0.11565	0.406447
CAPN5	-0.43593	0.231569	0.139574	0.124452	0.157972	-0.21782	-0.06025	-0.06976
CAPN6	0.337804	0.585595	0.005729	0.186745	-0.11429	0.004855	NA	NA
CAPN7	-0.22338	-0.62523	-0.24008	-0.07359	-0.00207	-0.1873	-0.62006	-0.59071
CAPN8	NA	NA	-0.30424	0.188589	0.101879	0.117229	NA	NA
CAPN9	-0.27857	0.132901	-0.34157	-0.07617	0.043741	-0.08711	0.135573	-0.00445
CAPNS1	0.000558	0.10959	0.309814	0.232485	0.148272	0.176348	0.53608	0.181578
CAPNS2	-0.15118	-0.58041	0.271211	0.061358	0.142601	-0.25009	0.119806	-0.25354
CAPRIN1	NA	NA	-0.31462	-0.00413	-0.01149	-0.17306	NA	NA
CAPRIN2	NA	NA	-0.18536	0.115328	-0.01054	0.177986	NA	NA
CAPS	-0.10049	-0.29281	0.079909	0.170456	0.029431	-0.08255	0.235479	-0.28653
CAPS2	-0.00824	-0.28339	0.326288	0.024685	0.091756	-0.19524	0.150211	0.041844
CAPSL	-0.17914	-0.28279	-0.09383	-0.05573	0.115827	0.067754	0.030117	0.025167
CAPZA1	-0.13185	-0.31197	-0.12975	0.043455	0.091638	-0.05183	NA	NA
CAPZA2	0.344871	-0.05767	-0.37069	-0.21206	-0.25249	-0.37822	0.145621	0.163804
CAPZA3	-0.0217	0.137961	0.14224	-0.2178	-0.02893	-0.18358	0.253064	0.023359
CAPZB	-0.57309	0.377118	0.194798	0.342975	0.14706	0.011726	-0.48034	-0.16083
CARD10	0.024905	0.176106	0.048343	-0.23233	0.518327	-0.06604	0.496582	0.397936
CARD11	0.23936	0.52617	-0.0429	0.758401	-0.04611	0.869076	-0.52675	-0.15026
CARD14	-0.00372	0.335044	0.371128	0.010785	0.361136	0.026155	-0.1001	-0.3716
CARD16	NA	NA	0.170467	-0.04054	-0.37527	-0.25506	NA	NA
CARD17	NA	NA	0.323074	-0.05623	-0.31864	0.040271	NA	NA
CARD18	NA	NA	-0.20361	0.462951	-0.02824	0.020975	NA	NA
CARD6	-0.1491	-0.30902	0.060324	0.087265	-0.13712	-0.26394	0.458242	0.215769
CARD8	0.135073	0.380111	-0.05839	0.555667	-0.21469	0.261733	-0.3158	0.122272
CARD9	-0.26988	0.003462	0.022969	0.093132	-0.31338	0.302052	-0.38868	-0.03804
CARHSP1	-0.00874	0.203223	0.137024	0.255067	0.008882	-0.12006	-0.18683	-0.18466

CARKD	NA	NA	0.079862	0.049966	0.365187	0.102635	NA	NA
CARM1	0.385527	0.393785	NA	NA	NA	NA	-0.08245	-0.60972
CARS	0.08757	-0.55105	-0.07268	0.130608	0.192588	-0.20529	-0.47459	-0.17295
CARS2	0.283792	-0.18066	0.04297	0.438355	0.545083	-0.20206	NA	NA
CARTPT	-0.16044	-0.03559	0.187174	-0.21151	-0.1881	-0.31925	NA	NA
CASC1	-0.01611	0.496172	-0.22789	-0.04445	-0.40082	-0.14302	-0.0504	-0.3679
CASC2	0.46869	0.455039	0.061183	-0.24225	0.06819	-0.04808	-0.57733	-0.56008
CASC3	0.224551	-0.0186	0.302687	0.194884	0.36989	0.307641	-0.06152	-0.06533
CASC4	-0.28703	-0.67187	-0.06019	-0.13988	-0.36349	-0.12934	0.42138	0.358501
CASC5	-0.13471	0.550883	-0.24409	0.188953	-0.18541	-0.15027	0.166857	0.37811
CASD1	0.000983	-0.65599	-0.21519	-0.10572	-0.24662	-0.30204	0.404565	0.054674
CASK	0.211825	-0.49453	-0.00734	0.004139	-0.03502	-0.15482	0.501963	0.273423
CASKIN1	0.382359	0.186563	0.025088	-0.09519	-0.02786	0.082682	-0.13448	0.079605
CASKIN2	0.141213	0.73206	0.31216	-0.16912	0.124823	0.144121	-0.45202	0.040012
CASP1	0.07148	0.067653	0.485453	0.028254	-0.15201	0.238961	-0.38032	0.492026
CASP10	0.18893	0.699644	0.180508	-0.14374	-0.22283	-0.08636	-0.04582	0.372904
CASP12	NA	NA	-0.0344	-0.26775	-0.22403	-0.14076	-0.04837	0.017213
CASP14	0.162755	0.565466	0.189544	-0.12241	0.14666	0.125378	0.05506	-0.27204
CASP2	0.156787	-0.12355	-0.00585	0.247437	0.174019	0.225727	0.430098	0.35715
CASP3	-0.49866	0.359256	0.096083	-0.28041	0.150963	-0.19696	0.210693	0.092845
CASP4	-0.56405	0.005343	0.33279	-0.14074	-0.13985	0.10057	-0.18289	0.342343
CASP5	-0.17387	0.184152	0.425509	-0.14836	-0.12734	0.142067	-0.2487	0.292899
CASP6	-0.20268	-0.21251	0.136783	-0.05345	0.063756	-0.02158	-0.08903	-0.29197
CASP7	-0.19768	-0.12209	-0.23313	-0.22229	0.0437	-0.55643	0.157624	-0.02909
CASP8	-0.09276	0.135689	-0.01637	0.024225	-0.10045	-0.03708	0.091207	0.243163
CASP8AP2	0.125632	0.229169	-0.26577	-0.27842	0.112564	0.018688	-0.18904	-0.36615
CASP9	-0.25791	0.398752	-0.03666	0.138249	0.08214	0.056926	0.059826	0.482391
CASQ1	-0.06186	-0.0886	0.339738	0.165978	-0.05045	0.170778	-0.30934	-0.14233
CASQ2	0.016395	-0.0793	0.09271	-0.11552	-0.06155	0.079481	-0.24245	-0.33
CASR	0.058788	-0.10061	0.006415	0.02253	-0.06256	-0.3482	-0.05415	0.060982
CASS4	NA	NA	-0.02906	-0.22701	0.034086	0.244429	NA	NA
CAST	-0.14549	-0.47649	-0.03938	-0.17044	-0.00568	-0.2382	-0.51571	-0.37449
CASZ1	-0.33466	0.142877	0.263885	-0.26102	0.111216	-0.06842	0.072664	-0.16237
CAT	0.023158	-0.43218	0.115169	0.058274	-0.08313	0.097219	-0.40312	-0.4331
CATSPER1	0.020342	0.470983	0.066274	0.023072	-0.1812	-0.10136	-0.00438	-0.07977
CATSPER2	-0.06535	-0.34745	0.238142	-0.00946	0.318354	0.371154	0.077857	0.557713
CATSPER3	0.07782	0.455552	-0.14433	0.101216	0.062206	-0.37106	-0.25339	0.124407
CATSPER4	0.16311	0.668886	0.167612	-0.06736	0.156828	0.178187	0.097883	0.072239
CATSPERB	NA	NA	-0.28542	-0.12486	0.033608	-0.1465	NA	NA
CAV1	-0.61849	-0.30863	-0.00035	-0.02051	-0.20845	0.10283	-0.07783	-0.07422
CAV2	-0.44391	-0.44607	-0.18027	-0.04904	-0.32708	-0.11149	-0.18638	-0.07371
CAV3	0.166035	-0.00442	-0.1381	-0.12218	0.032143	0.155698	-0.09259	0.297704
CBARA1	-0.36523	0.043615	-0.02523	-0.22064	0.051022	-0.47708	0.430015	0.409719
CBFA2T2	0.266959	-0.42168	0.24249	-0.1621	0.351808	-0.04012	0.423337	0.35771
CBFA2T3	-0.47821	0.708219	-0.11385	0.384232	-0.02436	0.250702	-0.17782	-0.32679
CBFB	0.166748	0.393262	-0.00528	0.046039	0.46194	0.540133	0.559494	0.209993
CBL	0.186558	-0.52822	0.097774	-0.11766	-0.06301	0.176925	-0.32997	-0.36923
CBLB	-0.29806	-0.04693	-0.08937	0.11679	-0.06947	0.398377	-0.32226	0.11421
CBLC	0.370413	0.349662	0.183047	-0.12887	0.235251	0.049532	0.394974	-0.00643
CBLL1	0.361598	-0.07701	-0.06921	-0.26607	0.092323	-0.04685	0.096598	-0.20599
CBLN1	-0.06334	-0.34396	0.221188	0.090853	0.202963	0.028213	0.361308	0.22358
CBLN2	0.107512	0.161715	0.050932	0.177312	-0.12951	0.039934	-0.38832	-0.45528

CBLN3	NA	NA	0.048426	-0.04997	0.308268	-0.35829	-0.51083	-0.06205
CBLN4	0.059074	-0.14965	-0.07812	-0.1982	-0.2299	0.148516	-0.06842	0.197267
CBR1	0.201646	-0.39215	0.035365	-0.31567	-0.24528	-0.40311	-0.25955	0.211741
CBR3	-0.00963	-0.03956	-0.23549	-0.15568	-0.20905	0.079668	-0.2446	0.099368
CBR4	0.09191	-0.5271	-0.02549	-0.04389	-0.1424	-0.36446	0.47986	0.214302
CBS	0.042215	0.093948	0.05383	-0.06787	0.242179	-0.18339	-0.30275	0.043153
CBWD1	-0.38935	0.289113	NA	NA	NA	NA	-0.42268	-0.44668
CBWD2	0.371059	0.324715	-0.39876	-0.14793	-0.12959	-0.1338	0.108157	0.039517
CBWD5	NA	NA	-0.32233	-0.12245	0.124793	0.23006	-0.16798	0.235122
CBX1	-0.41506	-0.30983	0.064209	0.043482	-0.07962	-0.32839	-0.33627	-0.22844
CBX2	0.034739	0.408332	0.121459	-0.16205	0.27886	-0.18512	0.480461	-0.04263
CBX3	0.155631	0.559748	-0.21248	0.117128	-0.03099	-0.11583	-0.29262	-0.20014
CBX4	0.19698	0.20095	0.201472	0.321663	0.364509	0.252791	0.517009	-0.07654
CBX5	0.064306	0.606293	-0.58728	0.092091	-0.14086	-0.03278	-0.2776	-0.23814
CBX6	-0.13128	-0.11397	0.377528	-0.14253	0.242263	0.111813	-0.15784	-0.4059
CBX7	-0.29106	-0.28821	0.293477	-0.00395	0.218564	-0.02309	-0.29782	-0.14939
CBX8	0.350387	0.185147	0.049133	0.130624	0.204003	-0.38206	0.429248	-0.47291
CBY1	-0.03765	-0.00401	0.092192	0.00262	0.11265	-0.27663	-0.5296	-0.4998
CC2D1A	0.33234	0.459336	0.308191	-0.20199	0.450552	0.250288	0.325032	0.380534
CC2D1B	-0.39455	0.090008	0.487684	0.019135	0.472433	0.103494	-0.40209	-0.00451
CC2D2A	NA	NA	-0.06119	-0.13225	-0.40995	-0.07659	NA	NA
CC2D2B	NA	NA	-0.21778	-0.15898	-0.05714	-0.24982	NA	NA
CCAR1	0.032723	-0.20474	-0.35951	0.185852	-0.03259	-0.12461	NA	NA
CCBE1	-0.15649	-0.19664	0.107896	0.14493	-0.25759	0.058152	-0.5708	-0.59056
CCBL1	0.020637	0.356635	0.533786	0.521987	0.35754	0.407759	0.284189	0.17507
CCBL2	-0.12381	0.374918	0.046505	-0.38105	0.10608	0.155416	0.249878	-0.21396
CCBP2	0.045805	0.291269	0.075991	-0.34829	-0.09869	0.182906	-0.19415	-0.13859
CCDC101	0.363704	0.282519	-0.08025	0.212059	0.190273	-0.17437	NA	NA
CCDC102A	0.269588	-0.09207	0.218072	0.182278	0.06704	0.488429	NA	NA
CCDC102B	0.091756	0.206902	-0.06801	-0.3744	-0.10385	0.120404	NA	NA
CCDC103	0.215288	-0.04353	-0.03253	0.030674	-0.21932	0.081089	NA	NA
CCDC104	0.026102	-0.50169	-0.0857	-0.06252	-0.13399	-0.42673	-0.6146	-0.34609
CCDC105	0.235637	-0.05214	-0.09658	0.269795	0.088044	-0.19667	NA	NA
CCDC106	0.007404	0.059248	0.180991	0.145379	0.110637	0.025883	NA	NA
CCDC107	-0.4726	-0.28028	0.219796	-0.147	-0.10605	-0.07916	NA	NA
CCDC108	-0.01349	-0.13358	0.011919	-0.01104	0.023275	0.269399	-0.24942	-0.29649
CCDC109A	-0.42677	0.015738	-0.2027	-0.27634	0.033852	-0.10615	NA	NA
CCDC109B	-0.25605	-0.07045	0.05327	0.376459	-0.13852	0.217468	NA	NA
CCDC11	-0.47085	0.077836	0.081674	-0.11119	-0.15843	-0.29787	0.197098	0.229965
CCDC110	-0.15066	0.258592	-0.20125	-0.13275	-0.05957	-0.28231	0.188202	0.301111
CCDC111	-0.24848	-0.49745	-0.29868	-0.21469	-0.05471	-0.42627	NA	NA
CCDC112	-0.19766	-0.48277	-0.20177	0.023541	-0.08538	-0.37389	NA	NA
CCDC113	0.347188	0.168282	0.07576	0.042875	0.140685	-0.24888	NA	NA
CCDC114	0.067232	0.318219	0.167577	-0.20911	0.01429	-0.22461	NA	NA
CCDC115	0.197704	-0.4492	0.194839	0.080125	0.123926	-0.06003	NA	NA
CCDC116	0.309901	0.373709	-0.46272	-0.13671	0.086376	-0.33585	NA	NA
CCDC117	-0.30241	-0.715	-0.08388	0.294495	0.121683	-0.32888	0.206516	0.36808
CCDC12	-0.00529	0.629968	-0.0979	0.136712	0.031803	-0.08736	0.199499	-0.01461
CCDC120	0.121845	0.556655	0.293887	-0.41691	0.289137	0.140122	NA	NA
CCDC121	-0.18437	-0.65774	-0.2485	-0.17389	-0.01109	-0.44779	-0.09838	-0.35442
CCDC122	0.3664	0.584749	-0.20557	-0.00492	-0.01516	-0.32845	NA	NA
CCDC123	0.377247	0.151605	-0.00662	-0.12033	-0.08091	0.021215	NA	NA

CCDC124	0.290644	0.357825	0.224205	0.103743	-0.00673	0.001269	NA	NA
CCDC125	0.153839	0.685961	-0.10286	-0.2615	-0.33948	-0.27269	NA	NA
CCDC126	-0.11768	-0.12514	-0.32094	-0.15446	-0.11707	-0.4494	NA	NA
CCDC127	0.071309	0.133875	0.043688	0.058072	-0.09385	-0.2196	0.200066	0.088705
CCDC129	0.156297	-0.13106	NA	NA	NA	NA	0.033132	-0.00506
CCDC13	-0.08694	0.467592	-0.49402	-0.11566	0.064266	-0.1738	0.269645	-0.09736
CCDC130	0.131981	-0.03799	-0.16668	0.095863	0.16996	0.313469	NA	NA
CCDC132	0.087016	-0.49073	-0.23056	-0.34518	0.076711	-0.39341	NA	NA
CCDC134	0.228048	0.316722	0.203666	0.204815	-0.02193	-0.3268	NA	NA
CCDC135	-0.11844	0.201968	0.049122	-0.08714	0.116579	0.159939	NA	NA
CCDC136	NA	NA	0.140949	-0.12098	0.202895	0.06499	NA	NA
CCDC137	NA	NA	0.109902	0.36169	0.075119	0.161437	NA	NA
CCDC138	NA	NA	-0.14173	0.091387	-0.06667	-0.15648	NA	NA
CCDC14	-0.03852	-0.08411	-0.23176	-0.03577	0.192474	0.096337	0.000756	0.056486
CCDC140	NA	NA	0.049125	-0.10092	-0.16559	-0.06653	NA	NA
CCDC141	NA	NA	-0.04501	0.162083	0.007065	0.027372	NA	NA
CCDC142	NA	NA	0.286694	-0.19558	0.309363	0.10292	NA	NA
CCDC144A	NA	NA	-0.08493	-0.11023	-0.01299	-0.02934	NA	NA
CCDC146	NA	NA	0.0626	-0.11779	0.064595	0.083144	NA	NA
CCDC148	NA	NA	-0.02935	0.01038	-0.26647	-0.19313	NA	NA
CCDC149	NA	NA	-0.08557	0.004492	0.144446	0.20807	NA	NA
CCDC15	0.009488	-0.44451	-0.0429	0.056106	-0.11613	-0.18399	NA	NA
CCDC150	NA	NA	-0.46202	0.055595	-0.1044	-0.01421	NA	NA
CCDC151	NA	NA	-0.02847	0.023189	-0.25224	-0.30086	NA	NA
CCDC153	NA	NA	0.293574	-0.28497	-0.19022	-0.44344	NA	NA
CCDC155	NA	NA	0.123155	0.21409	0.338573	0.038665	NA	NA
CCDC17	0.102765	0.705368	-0.10089	-0.15537	-0.13367	-0.30085	0.528886	0.396804
CCDC18	0.229857	0.301885	NA	NA	NA	NA	-0.30869	0.113311
CCDC19	-0.22896	0.537815	-0.35812	-0.08211	0.063116	-0.10068	NA	NA
CCDC21	-0.38042	-0.61008	0.076624	0.107896	0.382204	-0.12703	-0.10459	-0.23758
CCDC22	0.543691	-0.01882	0.277663	0.36588	0.246095	0.227719	-0.36143	-0.46753
CCDC23	-0.10101	0.085277	-0.06952	-0.08306	-0.05363	-0.29408	0.3	0.099229
CCDC24	0.451176	0.438924	0.273243	-0.12483	0.095457	-0.19648	0.049819	-0.29512
CCDC25	0.003853	-0.54611	0.092156	-0.24517	-0.21647	-0.29939	-0.24181	-0.2426
CCDC27	-0.07078	-0.2607	0.123954	-0.26614	0.072368	0.057527	0.204683	0.204071
CCDC28A	0.345789	0.066806	-0.07168	-0.31317	0.10242	-0.01206	-0.39676	-0.23041
CCDC28B	-0.01388	0.635851	-0.11132	0.377338	-0.20145	0.182196	-0.23068	-0.28715
CCDC3	-0.12415	-0.13433	-0.05938	0.26503	0.076759	0.003965	-0.03502	0.34707
CCDC33	0.25947	0.723986	NA	NA	NA	NA	0.518407	0.43573
CCDC34	0.243521	-0.06296	-0.107	0.431075	0.048217	-0.52591	0.350156	-0.08467
CCDC36	-0.08901	0.446667	-0.17077	-0.17845	-0.23413	-0.18458	0.129443	0.024586
CCDC37	-0.05116	0.44914	-0.0011	-0.18076	-0.1219	0.064868	0.468207	-0.09397
CCDC38	-0.02187	0.071894	0.301332	-0.17205	-0.1425	0.249249	0.176282	-0.1931
CCDC39	NA	NA	0.162478	0.270798	0.391776	0.089947	NA	NA
CCDC40	NA	NA	0.08452	0.190982	0.068515	-0.00046	NA	NA
CCDC41	0.286686	-0.0565	-0.45382	-0.2082	0.149319	-0.00612	-0.09379	-0.14999
CCDC42	0.589809	0.41328	0.249766	0.412742	-0.09295	-0.16727	-0.37428	-0.33406
CCDC43	0.320109	0.088298	-0.0149	0.161218	0.057553	-0.25707	-0.35114	-0.47951
CCDC44	0.359078	0.499734	-0.01278	0.060717	-0.13379	-0.45254	0.151876	-0.14118
CCDC45	0.179974	-0.1248	-0.02516	-0.06467	0.012222	0.378728	-0.25785	-0.07922
CCDC46	-0.0273	-0.12868	0.261678	-0.09542	-0.16092	-0.22412	-0.33153	-0.08224
CCDC47	0.029012	0.361766	0.193972	0.001387	-0.14897	-0.07255	0.308515	-0.10889

CCDC48	0.341316	0.7715	-0.30156	-0.28984	0.016639	0.17602	-0.08487	-0.16848
CCDC49	-0.03149	-0.25479	-0.10469	-0.01194	-0.11273	-0.2773	-0.1923	0.445863
CCDC5	-0.10192	-0.52543	-0.03611	0.144084	0.000889	-0.21861	-0.30256	-0.34134
CCDC50	-0.17161	-0.34046	-0.03533	-0.26223	-0.19799	-0.17364	-0.20999	0.290582
CCDC51	0.322725	0.502522	0.14177	-0.09595	-0.01877	-0.22698	0.236226	-0.29442
CCDC52	0.03399	-0.27604	-0.40824	-0.20949	-0.05221	-0.2169	-0.04135	-0.3592
CCDC53	0.131934	-0.27402	0.423265	-0.18223	0.250036	-0.29446	-0.35434	-0.32062
CCDC54	-0.02823	0.713539	-0.09468	-0.12864	-0.253	0.03407	0.017424	0.240654
CCDC55	0.151182	-0.54924	-0.12165	-0.02178	-0.16163	0.037025	-0.68	-0.41323
CCDC56	NA	NA	0.213612	0.034578	-0.14494	-0.5062	0.099496	-0.18935
CCDC57	0.28699	0.303258	0.388575	0.097042	0.016752	-0.64618	0.533359	0.547529
CCDC58	-0.10935	0.020736	-0.21833	-0.08021	-0.08008	-0.23085	0.199036	-0.0927
CCDC59	0.37396	-0.01529	-0.2638	-0.05004	0.06411	-0.2459	0.272364	0.201606
CCDC6	0.322131	-0.32642	-0.03974	-0.3244	0.254223	-0.28246	0.170069	0.084029
CCDC60	-0.09588	0.018799	-0.14527	0.167869	-0.1826	-0.50464	0.015433	0.006965
CCDC61	NA	NA	-0.03775	0.195819	0.050011	-0.04157	NA	NA
CCDC62	-0.13014	0.429466	-0.10555	0.028472	0.205092	0.235332	-0.30569	-0.19213
CCDC63	0.005463	0.434884	-0.02098	-0.42306	0.103524	-0.07552	-0.46582	-0.2768
CCDC64	-0.03201	0.143023	NA	NA	NA	NA	0.605164	0.201754
CCDC64B	NA	NA	0.057851	-0.00719	0.319406	-0.24352	NA	NA
CCDC65	-0.1359	-0.13636	-0.2719	0.145772	0.097192	0.086166	-0.09344	0.16851
CCDC66	0.142223	-0.50594	-0.22117	-0.23184	-0.21607	-0.01886	-0.22229	0.216824
CCDC67	-0.07196	0.172798	-0.24706	0.157054	-0.12214	-0.08548	-0.21734	0.112699
CCDC68	-0.23093	-0.0843	-0.30952	-0.03986	-0.29279	-0.25738	-0.44288	-0.6718
CCDC69	0.062255	0.115278	0.29055	0.078226	0.112058	-0.03649	-0.45626	-0.48233
CCDC7	0.330447	0.733919	0.276835	-0.29444	-0.20353	-0.00659	-0.09046	-0.30365
CCDC70	0.155853	0.639399	0.041375	-0.264	0.265071	-0.05391	-0.54852	-0.32388
CCDC71	-0.05555	-0.13156	0.140219	-0.24474	-0.01377	0.073561	0.292497	0.337022
CCDC72	-0.01082	-0.54658	-0.17897	-0.12376	-0.0637	-0.09422	-0.37382	-0.265
CCDC73	0.150047	0.171515	-0.08568	0.056351	-0.07748	0.174929	NA	NA
CCDC74A	0.354683	0.093946	NA	NA	NA	NA	-0.09062	-0.12119
CCDC74B	0.151958	-0.13905	-0.2467	0.08097	-0.11756	-0.18511	0.038011	0.046825
CCDC75	-0.04554	0.358296	-0.03811	-0.08881	-0.06237	-0.30855	0.221208	0.489415
CCDC76	0.071875	-0.46841	0.094211	0.048208	0.265625	0.167063	0.558093	0.346473
CCDC77	0.047785	0.266109	-0.33109	0.099763	-0.0414	0.089317	-0.05603	0.068526
CCDC78	-0.02059	0.515078	-0.10005	0.388471	0.012588	0.065194	NA	NA
CCDC79	NA	NA	0.035524	0.259376	0.243817	0.255959	NA	NA
CCDC8	0.175053	-0.25103	0.117222	0.048727	-0.14675	0.25895	-0.11358	-0.31748
CCDC80	-0.1988	-0.26533	0.297117	0.035176	-0.12568	0.1955	NA	NA
CCDC81	-0.01668	-0.22357	-0.19819	0.105812	0.205133	-0.14881	NA	NA
CCDC82	-0.20515	-0.45554	-0.15469	-0.11916	-0.05942	-0.03097	NA	NA
CCDC83	-0.00202	0.159594	-0.10562	-0.04104	-0.37373	0.114333	NA	NA
CCDC84	-0.08096	-0.13745	-0.15952	-0.06976	-0.01482	0.294204	NA	NA
CCDC85A	NA	NA	0.351641	0.173803	-0.03079	0.257044	-0.01329	0.264417
CCDC85B	-0.03597	0.595943	0.321693	-0.00152	0.318452	0.162407	NA	NA
CCDC86	0.141484	0.678207	-0.04741	0.056384	0.297526	0.304486	NA	NA
CCDC87	0.26672	-0.13637	-0.12707	-0.20235	-0.27721	-0.21738	NA	NA
CCDC88A	NA	NA	0.048413	0.367855	-0.2622	0.334085	NA	NA
CCDC88B	NA	NA	-0.00085	0.512488	0.134723	0.484525	NA	NA
CCDC88C	NA	NA	-0.116	0.272723	0.039557	0.449801	NA	NA
CCDC89	-0.42225	-0.52317	-0.26483	-0.11867	-0.18807	-0.41358	NA	NA
CCDC9	0.444721	0.535305	-0.12368	0.2016	0.221788	0.285835	0.503948	0.138819

CCDC90A	0.36861	0.198619	0.270892	-0.1439	0.028331	-0.47565	NA	NA
CCDC90B	-0.03957	-0.35868	-0.18867	-0.09459	-0.29189	-0.27253	0.073716	0.098941
CCDC91	0.294768	-0.33406	-0.21072	-0.17858	-0.20713	-0.1301	-0.55117	-0.33008
CCDC92	0.119308	0.1221	0.298429	0.052232	0.329433	0.197977	NA	NA
CCDC93	0.325876	0.046854	0.036206	-0.11767	0.165539	-0.1041	NA	NA
CCDC94	0.383932	0.68105	0.044778	0.06203	0.142709	-0.0281	NA	NA
CCDC96	0.069884	-0.17561	0.136919	-0.05646	-0.02545	-0.37255	NA	NA
CCDC97	0.428697	-0.01548	-0.16178	0.257195	0.187947	0.22166	NA	NA
CCDC99	0.225502	-0.37857	-0.34769	0.191059	-0.23651	-0.06719	NA	NA
CCHCR1	0.146027	0.171954	0.059264	0.15261	0.199297	0.024263	-0.01921	-0.25669
CCIN	-0.43246	0.085375	0.208497	-0.37412	-0.18065	0.104001	-0.04114	-0.06565
CCK	-0.03176	-0.0327	-0.18571	0.219762	-0.13181	-0.18429	-0.09857	0.181874
CCKAR	0.263738	0.369146	-0.02772	0.164322	-0.21875	0.288979	0.266455	0.062498
CCKBR	0.429707	0.667976	0.409352	-0.28071	0.337309	0.140958	-0.13909	0.16503
CCL1	-0.15561	-0.21312	-0.15923	-0.02841	0.016126	-0.00348	-0.08665	-0.01797
CCL11	0.158368	-0.07045	-0.11938	-0.09122	-0.1096	-0.1921	-0.13093	0.173593
CCL13	-0.39038	0.035764	0.109979	-0.12308	-0.26244	-0.28514	-0.2517	-0.05991
CCL15	0.02824	0.303598	0.29281	-0.31837	-0.25274	-0.43632	-0.33169	-0.29033
CCL16	0.314517	0.654344	-0.29549	-0.11474	0.125158	-0.04766	-0.65958	-0.34524
CCL17	-0.02968	0.096852	0.274946	0.7803	0.000746	0.445626	-0.04079	-0.0218
CCL18	-0.24452	-0.13734	0.198179	0.497268	-0.37424	0.091511	-0.23373	0.189254
CCL19	-0.10022	0.211286	0.102908	0.638515	-0.25264	0.661997	-0.45151	-0.13503
CCL2	-0.27016	-0.02789	0.019382	-0.04306	-0.2276	0.127156	-0.4611	0.07613
CCL20	0.303488	-0.05148	0.219673	0.207921	0.084458	0.03783	-0.25447	-0.01383
CCL21	0.120495	0.187583	0.098933	0.719801	-0.21552	0.500103	0.09176	0.17258
CCL22	-0.12499	0.520963	0.193505	0.339377	0.110327	0.236051	-0.45002	0.025891
CCL23	-0.31371	-0.06563	0.182725	0.372116	-0.30152	0.31756	-0.40667	-0.00604
CCL24	-0.30872	0.312463	0.16359	-0.14705	0.07704	-0.09982	0.293175	0.341574
CCL25	0.224255	-0.09645	0.17155	-0.14679	0.031978	0.141396	0.488015	0.494377
CCL26	-0.32623	0.354325	0.024935	-0.03897	-0.04219	-0.39026	-0.13953	-0.16245
CCL27	-0.05443	0.605288	-0.4519	0.088012	-0.32336	-0.19106	-0.04376	0.005448
CCL28	-0.00824	0.149831	-0.14875	0.020117	-0.1746	-0.36114	-0.20085	-0.41209
CCL3	-0.37527	-0.03012	0.141087	0.300938	-0.0671	0.107265	-0.14732	0.120978
CCL3L3	NA	NA	0.213697	0.392264	-0.06436	0.146894	-0.23453	0.02225
CCL4	-0.34319	-0.03695	0.215169	0.240741	-0.04634	0.155289	NA	NA
CCL5	-0.37927	-0.1239	0.412292	0.020408	-0.36722	0.257445	-0.60375	0.216186
CCL7	-0.38288	0.622859	-0.0172	-0.34048	-0.2571	-0.32226	-0.14545	-0.11368
CCL8	-0.22402	0.174102	-0.02946	-0.22274	-0.34018	-0.22163	-0.35691	0.03069
CCM2	-0.1976	-0.27148	0.270214	-0.02591	0.254125	0.255243	-0.64384	-0.07769
CCNA1	0.017292	-0.33099	-0.08535	0.261752	-0.19516	0.066713	0.570783	0.162811
CCNA2	-0.04639	0.360463	-0.26964	0.279906	-0.03409	-0.0413	0.296337	0.229914
CCNB1	-0.00852	0.351032	-0.14428	0.129535	-0.20398	0.002454	0.015312	-0.29749
CCNB1IP1	0.055453	0.228465	-0.19658	-0.29211	-0.05066	0.008235	0.066255	-0.20346
CCNB2	0.187119	0.149531	-0.44117	0.000171	-0.23086	-0.09878	0.299752	0.027303
CCNB3	0.149188	0.045988	-0.16915	-0.33481	0.122694	0.063799	0.307885	0.492239
CCNC	0.326697	-0.54083	-0.44467	-0.11478	-0.08285	-0.34379	0.060813	-0.13093
CCND1	-0.13781	0.194737	0.297599	-0.10466	0.39509	0.218022	0.347804	0.002928
CCND2	0.607001	-0.3705	-0.07386	0.125105	0.023962	0.410174	-0.0152	0.220854
CCND3	-0.27141	0.602691	0.238052	0.101403	0.237232	-0.15009	0.027511	-0.19435
CCNDBP1	-0.12318	-0.27933	-0.02677	-0.23473	-0.11785	-0.25778	0.095162	0.35729
CCNE1	0.071002	0.182576	0.058507	0.295338	0.173106	-0.03405	0.358323	0.188538
CCNE2	-0.30499	0.110455	-0.22879	0.018471	-0.04392	-0.33518	0.399594	0.264688

CCNF	0.082753	0.296185	-0.1526	0.314098	0.343074	-0.00654	0.400277	0.113423
CCNG1	0.120803	-0.49274	0.044404	-0.15316	-0.22572	-0.14087	-0.4143	-0.50839
CCNG2	-0.33201	-0.11497	-0.27246	-0.13066	-0.34865	-0.30961	-0.23353	-0.29282
CCNH	0.393469	-0.5013	0.355571	-0.33128	-0.0633	-0.23347	-0.4215	-0.31305
CCNI	-0.22337	-0.21936	0.191614	0.200226	0.183923	0.401877	0.400872	0.055354
CCNJ	-0.22499	-0.20474	-0.17587	-0.21453	0.213793	-0.20299	-0.0296	-0.23067
CCNJL	-0.27719	-0.20116	-0.06145	-0.04055	-0.24149	-0.39793	NA	NA
CCNK	0.181341	0.100811	-0.15352	-0.15228	-0.1411	-0.05837	0.102439	0.203906
CCNL1	-0.19919	-0.53981	-0.28947	-0.05753	-0.07727	0.169403	0.138749	0.516077
CCNL2	0.436753	0.744066	0.269916	0.098703	0.165272	0.062438	0.110824	0.235434
CCNO	NA	NA	0.238318	0.28619	0.258995	-0.06331	NA	NA
CCNT1	0.027488	0.376021	-0.19388	-0.24719	0.090236	-0.17289	-0.2591	-0.51131
CCNT2	-0.39385	-0.22749	-0.19376	-0.20954	-0.18941	-0.19463	0.204068	0.160816
CCNY	-0.01357	0.270364	0.391616	0.091685	0.176911	0.255068	0.052935	0.055926
CCNYL1	0.044721	-0.01191	-0.18805	-0.24791	-0.17262	-0.16091	-0.08724	-0.08053
CCPG1	-0.27964	-0.04608	-0.09131	-0.19715	-0.26412	-0.39872	-0.36971	0.002278
CCR1	-0.52232	0.207184	0.175237	0.058946	-0.22664	0.182628	-0.09421	0.509233
CCR10	0.33363	0.617728	0.203488	0.128828	0.004197	-0.24873	-0.34688	-0.28304
CCR2	-0.32033	0.168354	0.158971	0.072797	-0.17425	-0.03769	-0.16244	0.346363
CCR3	-0.18497	0.724027	-0.0384	0.041855	-0.25589	-0.09801	-0.18902	-0.01171
CCR4	0.309191	0.605789	0.347819	0.026823	-0.01051	0.438078	-0.13775	0.097308
CCR5	NA	NA	0.187244	0.189868	-0.33826	0.402223	-0.39535	-0.18276
CCR6	-0.10699	0.065612	-0.27702	0.519513	-0.03829	0.511655	0.55656	0.449349
CCR7	-0.19747	0.461308	0.107425	0.822261	0.003864	0.775847	-0.58992	-0.15664
CCR8	0.04739	0.251257	-0.07255	-0.12306	-0.23354	0.053372	-0.11392	-0.2917
CCR9	0.185642	0.684024	0.350734	0.449293	-0.12607	0.265473	0.196384	0.40315
CCRK	0.210461	-0.06229	-0.28083	-0.13743	-0.07068	-0.2459	-0.02429	0.273007
CCRL1	-0.11737	-0.16254	0.167293	-0.27368	-0.21153	-0.14118	-0.07389	0.162872
CCRL2	0.007315	0.220199	0.245039	-0.00055	0.134547	-0.24751	0.438326	0.486701
CCRN4L	-0.14941	0.316529	-0.17064	-0.21859	-0.24299	-0.17246	-0.05777	0.232789
CCS	0.010753	0.100694	0.297117	0.102373	0.202702	0.118353	-0.18654	-0.05334
CCT2	0.174546	-0.15027	-0.28164	-0.07364	0.201986	0.086459	0.633417	0.269244
CCT3	0.264325	-0.07347	0.042707	0.297796	0.012865	0.157371	0.240291	-0.3405
CCT4	0.511992	0.48035	-0.16531	0.165923	0.424837	0.057193	0.257295	0.121841
CCT5	0.226242	0.303797	-0.24177	-0.14306	0.22956	-0.38783	0.380379	-0.36169
CCT6A	0.080517	-0.63627	-0.14174	0.039836	0.119012	0.034746	0.230061	-0.06354
CCT6B	0.11544	-0.33473	-0.26261	-0.33586	-0.03982	-0.23637	-0.21053	-0.23336
CCT7	0.148802	-0.54884	-0.08135	0.481567	0.226441	0.169901	0.519183	-0.28222
CCT8	-0.12681	-0.72716	-0.0475	0.065232	-0.08783	0.050152	0.368853	0.275533
CD109	-0.01978	-0.30635	-0.19884	-0.27024	-0.1782	0.01039	-0.23647	-0.09396
CD14	-0.43855	-0.3029	0.315301	-0.34386	-0.03843	-0.00959	-0.49157	0.040928
CD151	-0.06324	-0.21251	0.137011	-0.06192	0.10062	-0.1844	0.066751	0.108287
CD160	0.082519	0.100986	0.066626	0.111686	-0.20246	-0.3591	-0.52971	0.04855
CD163	-0.56206	-0.34061	0.188569	-0.13588	-0.47932	0.329764	-0.2013	0.107807
CD163L1	0.153758	0.040904	0.010373	-0.38497	-0.01797	0.211247	NA	NA
CD164	-0.05816	-0.3295	-0.27116	-0.23369	0.044877	-0.22444	-0.32415	-0.31991
CD164L2	-0.33957	-0.06899	NA	NA	NA	NA	-0.23952	-0.01413
CD177	0.402223	0.063483	-0.17542	-0.38642	0.019392	-0.04183	-0.41716	-0.27569
CD180	-0.14262	0.01863	0.023675	0.485923	-0.34693	0.45051	-0.01355	-0.07299
CD19	-0.15835	0.412218	-0.0341	0.764336	-0.15138	0.613682	-0.11388	0.236217
CD1A	-0.01563	0.196076	0.079981	0.616472	-0.27791	0.479781	-0.09651	-0.11
CD1B	0.04516	0.059173	-0.08448	0.22274	-0.23792	0.439999	-0.15217	-0.03948

CD1C	-0.05828	0.026447	0.001763	0.669601	-0.05961	0.459284	-0.60353	-0.07553
CD1D	-0.12895	-0.17198	0.042029	0.343015	-0.08867	-0.11336	-0.37473	0.313052
CD1E	-0.12927	-0.08487	0.070439	0.426253	-0.2492	0.296838	-0.26215	-0.21247
CD2	-0.05138	0.024648	0.223446	0.576685	-0.2735	0.600765	-0.249	0.518586
CD200	-0.31874	0.414206	0.013644	0.214373	-0.43943	0.188426	0.060706	0.359273
CD200R1	0.167882	0.162705	0.023398	0.085648	0.010855	0.2421	-0.01524	0.501276
CD207	0.370704	0.749648	0.16015	0.026847	0.041958	0.094139	-0.0978	0.026162
CD209	-0.25063	0.048069	0.361454	-0.23897	-0.1864	0.302429	-0.43717	0.49811
CD22	0.047244	0.440957	0.125207	0.74996	-0.10882	0.605741	0.479291	0.289125
CD226	0.277137	0.414583	0.082686	0.33809	-0.20028	0.43597	-0.45819	0.126728
CD24	0.294031	-0.01334	-0.1623	0.028989	0.25868	0.336473	0.274909	-0.10739
CD244	-0.23238	0.529671	0.100116	0.087227	-0.1205	-0.12543	-0.13589	0.43623
CD247	-0.02899	0.176792	0.173645	0.54399	-0.26154	0.363927	-0.57907	0.228197
CD248	-0.27941	-0.17819	0.066131	0.029837	-0.11578	0.012478	-0.27951	-0.02562
CD27	0.120057	0.370542	0.191703	0.614054	-0.19832	0.122592	NA	NA
CD274	-0.25913	0.381468	-0.09499	-0.06183	-0.08126	0.19683	-0.04533	0.227409
CD276	-0.1973	0.601934	0.317088	0.154152	0.256821	-0.12353	-0.01902	-0.26583
CD28	-0.31927	0.007568	-0.06767	0.648043	-0.21533	0.669649	NA	NA
CD2AP	-0.13924	-0.41354	-0.4936	-0.06609	-0.09821	-0.18387	-0.05899	-0.17217
CD2BP2	0.042364	0.122704	0.373574	0.228265	0.589045	0.084548	0.249755	-0.02833
CD300A	-0.61771	-0.21299	-0.09069	0.257391	-0.13262	0.099743	-0.43001	0.517652
CD300C	-0.54035	-0.08421	0.108608	0.092854	-0.03268	-0.09702	-0.45033	0.096991
CD300E	-0.28008	-0.35197	0.215859	-0.0044	-0.10528	0.306933	-0.16201	-0.19575
CD300LB	0.285241	0.219287	0.025368	-0.14112	0.055204	-0.0532	-0.2277	0.24936
CD300LD	NA	NA	0.23611	-0.07876	-0.03271	-0.24399	NA	NA
CD300LF	-0.34491	0.39424	0.207596	0.444893	-0.25003	0.257143	-0.43918	0.195095
CD300LG	-0.25398	-0.09295	-0.08554	-0.1833	0.355252	0.221822	-0.70899	-0.10404
CD302	-0.00875	-0.51941	0.141613	-0.14596	-0.38042	-0.06918	-0.48368	-0.0341
CD320	0.106969	-0.15227	-0.04446	0.177331	0.208434	0.315732	0.509297	0.016487
CD33	-0.35955	-0.29817	0.220535	-0.12943	-0.41231	-0.17408	-0.55446	0.373541
CD34	-0.08576	-0.25664	-0.24155	0.131634	-0.07495	-0.03571	-0.31389	-0.05822
CD36	-0.32002	-0.30614	-0.00036	-0.38542	-0.26917	0.153938	-0.19171	0.45688
CD37	-0.53946	0.16842	0.217304	0.842055	-0.21674	0.687926	-0.39126	0.210103
CD38	-0.28052	-0.5247	0.244446	0.261985	-0.2385	-0.25027	-0.43998	-0.16871
CD3D	-0.08998	0.40758	0.30393	0.375099	-0.2417	0.413892	-0.63792	0.177276
CD3E	0.316657	0.708923	0.311924	0.6485	-0.1684	0.491973	-0.18116	0.508452
CD3EAP	0.373728	0.028734	-0.10317	0.072477	0.310565	0.309334	0.136558	-0.38347
CD3G	-0.04089	0.003978	0.17577	0.369516	-0.26561	0.406891	-0.63639	0.322295
CD4	-0.08979	0.278175	0.067915	0.009232	-0.29791	0.410259	-0.2538	0.32109
CD40	0.035238	-0.16805	0.240661	0.534334	0.059967	0.589758	-0.48196	0.198325
CD40LG	-0.02812	0.209392	0.133982	0.406579	-0.26662	0.276409	-0.36289	0.102377
CD44	-0.29085	-0.28634	-0.05637	0.366217	-0.03453	0.426689	-0.30372	0.00249
CD46	-0.07121	-0.65202	-0.09085	-0.03803	-0.01951	-0.30528	0.601075	0.316386
CD47	0.153668	-0.34643	-0.01926	-0.01978	0.196129	0.307753	-0.00113	-0.00804
CD48	0.173201	-0.08634	0.264808	0.502458	-0.38855	0.375916	-0.69739	-0.09548
CD5	0.006141	0.632558	0.212208	0.644402	0.032294	0.601974	-0.20153	0.186955
CD52	-0.35906	-0.00488	0.260602	0.582398	-0.46196	0.433517	-0.55268	0.056493
CD53	-0.4629	0.19454	0.235847	0.62256	-0.12839	0.671424	-0.55843	0.023959
CD55	-0.33497	-0.1741	0.116581	0.179911	-0.05283	0.224191	-0.24075	0.140198
CD58	-0.3192	-0.3327	0.302233	-0.23649	-0.08389	-0.08101	0.083704	-0.12083
CD59	-0.30967	-0.05626	-0.09288	-0.18744	-0.12158	-0.10657	-0.5669	-0.25287
CD5L	0.183415	0.327485	0.036889	-0.07333	0.181746	-0.05082	-0.69259	-0.20005

CD6	0.001493	0.296093	0.339803	0.781416	-0.0722	0.789592	-0.36112	0.291204
CD63	-0.13786	-0.2108	-0.12842	0.209799	0.025448	-0.37378	-0.45176	-0.51104
CD68	-0.29349	0.206282	0.365016	0.14788	-0.17258	0.002875	-0.20396	0.300523
CD69	-0.54754	-0.11714	-0.13164	0.235574	-0.12003	0.325821	-0.51189	0.051943
CD7	-0.02295	0.519052	0.300545	-0.05734	-0.19534	0.112931	-0.57959	0.148404
CD70	0.191885	0.550948	0.111425	0.732202	-0.00851	0.328988	NA	NA
CD72	-0.00224	0.225435	0.097626	0.750581	-0.28789	0.557875	-0.40629	0.19787
CD74	-0.14106	-0.23165	0.573484	0.500622	-0.25583	0.442528	-0.4319	0.219956
CD79A	-0.20753	0.380878	0.195462	0.819237	-0.13253	0.288677	-0.214	-0.13784
CD79B	-0.1641	0.265158	0.282289	0.778703	-0.16035	0.596469	-0.37929	-0.12784
CD80	-0.34002	0.239725	-0.10366	0.231071	-0.48654	0.379965	-0.33977	-0.05262
CD81	-0.09649	-0.07025	0.068536	0.064116	0.236181	0.671964	-0.06246	-0.24526
CD82	-0.50638	-0.11603	0.331398	0.615894	0.170464	0.467917	0.048113	0.459756
CD83	-0.39318	-0.02218	0.067156	0.734256	-0.02879	0.557635	-0.19341	0.092747
CD84	-0.43385	-0.15632	0.254636	0.218356	-0.30126	0.539318	-0.247	0.144002
CD86	-0.41854	0.105479	-0.04002	0.202671	-0.25781	0.043283	-0.51875	0.115518
CD8A	0.094494	0.050339	0.197159	0.072162	-0.3557	0.168823	-0.38981	0.509762
CD8B	-0.13341	0.409664	0.253847	0.098693	-0.20794	-0.06639	0.30153	0.727854
CD9	0.171099	-0.48979	-0.15795	-0.2118	-0.06739	-0.15279	-0.4403	-0.6561
CD93	-0.48061	-0.50886	-0.02716	0.216672	-0.01874	0.317346	NA	NA
CD96	-0.10588	0.032261	0.040373	0.287118	-0.27746	0.43483	-0.68238	0.291867
CD97	-0.10467	-0.03179	0.035026	0.053172	0.244199	0.170277	0.231073	-0.10482
CD99	-0.21308	-0.1802	0.47998	0.105124	-0.01025	-0.07619	0.010143	-0.16358
CD99L2	-0.19572	-0.05013	-0.14964	0.021263	0.305881	0.035807	-0.38258	-0.22468
CDA	-0.06241	0.236474	0.142488	-0.37405	-0.08975	-0.40648	0.129241	-0.03603
CDADC1	-0.12564	-0.05391	-0.20271	-0.08575	0.047201	-0.24127	0.385678	0.246245
CDAN1	0.212711	0.563607	-0.04147	-0.07927	0.031402	0.094421	-0.38735	-0.39421
CDC123	-0.15475	-0.41603	0.04151	0.145644	-0.18737	-0.21961	NA	NA
CDC14A	-0.25928	-0.14896	0.17699	-0.35918	-0.06218	-0.08005	-0.08453	0.103779
CDC14B	0.14829	-0.40234	0.053804	-0.17318	0.132911	-0.11443	0.038624	0.031195
CDC16	0.259626	-0.3413	0.092826	0.190417	0.336222	0.163865	0.15419	-0.17463
CDC2	-0.22749	-0.2879	-0.21275	0.121926	0.118791	-0.17008	0.153394	0.0128
CDC20	-0.18297	0.4272	-0.04009	0.284831	0.337171	0.036868	0.269425	-0.10312
CDC20B	0.055436	0.368976	-0.10394	0.135203	-0.13106	-0.0371	NA	NA
CDC23	0.396703	0.323709	-0.04256	-0.28727	-0.09081	-0.2865	-0.15435	-0.27387
CDC25A	-0.14712	0.063163	-0.18725	0.275586	0.32906	0.085562	0.456268	0.230327
CDC25B	0.181951	0.092174	0.09746	0.178268	0.372965	0.316819	0.515123	0.369503
CDC25C	0.349741	0.099501	0.136853	0.033191	0.122716	-0.24976	0.351505	0.151092
CDC26	-0.07784	0.161665	-0.06955	-0.14629	-0.39162	-0.25428	-0.5815	-0.33947
CDC27	0.171096	0.672949	-0.21025	0.027604	-0.34448	-0.26943	0.360185	0.423123
CDC2L1	-0.19717	-0.52186	0.270099	0.231888	0.162995	0.071082	0.30264	0.347776
CDC2L2	-0.17555	0.158167	0.029993	-0.04262	0.127515	-0.08472	-0.13393	0.100921
CDC2L5	0.650428	0.593779	-0.35381	0.113356	-0.00873	0.441285	-0.33087	-0.31456
CDC2L6	0.328394	0.184239	0.008439	-0.21023	0.251143	0.022636	0.281499	-0.04802
CDC34	-0.04687	0.064601	0.239727	-0.06568	0.252991	0.056172	-0.05714	-0.24991
CDC37	0.177785	0.562439	0.069305	0.247262	0.246321	0.20522	0.037886	0.242962
CDC37L1	-0.09921	-0.167	-0.23799	-0.12563	-0.17203	-0.13138	-0.53965	-0.33337
CDC40	0.478382	-0.51915	-0.5784	-0.06297	-0.02014	-0.20766	0.078142	0.1159
CDC42	-0.361	-0.43708	-0.10049	0.027295	-0.34345	-0.06777	-0.67562	-0.55893
CDC42BPA	-0.17578	0.344795	-0.0844	-0.27726	-0.01781	0.020779	-0.31684	0.115578
CDC42BPB	0.168218	0.337817	-0.08615	0.136257	0.056836	0.315097	0.257029	0.398659
CDC42BPG	0.237672	0.416402	-0.02909	-0.15125	0.078526	-0.08616	0.262461	0.140972

CDC42EP1	-0.278	0.45178	0.253461	0.208642	0.164607	0.109494	-0.22839	-0.30898
CDC42EP2	-0.24621	-0.30949	0.078448	0.054262	-0.00581	-0.00247	-0.30165	0.038837
CDC42EP3	-0.49786	-0.24579	0.150002	-0.01827	-0.17793	-0.15684	-0.13878	-0.12196
CDC42EP4	-0.07926	0.000481	0.302244	0.062472	0.198322	0.066681	0.225287	0.148782
CDC42EP5	-0.11164	0.233013	0.159617	-0.0425	0.045667	0.270519	-0.09421	-0.25056
CDC42SE1	-0.21453	-0.32438	-0.02253	0.130215	0.037704	-0.1322	0.138917	0.267326
CDC42SE2	-0.23549	-0.11445	0.078013	0.08667	-0.14054	-0.17005	0.087675	0.12556
CDC45L	0.145602	-0.0455	-0.25721	0.325471	0.327249	-0.04843	0.369831	0.211242
CDC5L	0.25159	-0.61413	-0.51816	0.102464	-0.08284	0.062396	0.083512	-0.05014
CDC6	0.191857	0.010898	-0.14618	0.305815	-0.01768	-0.0936	0.297863	0.209606
CDC7	-0.40492	0.090338	-0.14453	0.271523	0.103373	0.100734	-0.3167	-0.19484
CDC73	0.019658	-0.51543	-0.32985	-0.0285	-0.3411	0.10245	0.474558	0.468202
CDCA2	-0.12788	-0.11477	-0.22543	0.267521	-0.17561	-0.08354	0.360045	0.209814
CDCA3	0.431842	-0.15681	-0.31133	0.295088	-0.00332	-0.12139	0.227641	0.399471
CDCA4	-0.17811	0.060595	-0.06929	0.237889	0.097641	0.066293	0.042248	-0.31872
CDCA5	-0.21776	0.036664	-0.19717	0.338562	0.253763	-0.1618	0.418969	0.256033
CDCA7	0.192961	0.04737	-0.11801	0.344592	0.319164	0.204397	0.567019	0.348134
CDCA7L	0.060344	0.214572	-0.19397	0.280178	-0.07824	-0.01693	0.407256	0.223103
CDCA8	-0.37748	0.180093	-0.27684	0.248097	0.267545	-0.2455	0.555774	0.284948
CDCP1	-0.18115	0.259957	0.160522	-0.21808	0.302784	-0.08558	0.428419	0.337598
CDCP2	0.060928	-0.02443	0.263409	0.081064	-0.21886	0.244266	-0.01077	0.07613
CDGAP	0.298696	0.025336	0.280999	0.277506	-0.26874	0.185222	-0.47715	-0.44721
CDH1	-0.32167	0.122509	-0.06439	-0.03276	0.395228	-0.06887	0.582384	0.292343
CDH10	0.062344	-0.32471	0.310132	-0.05862	-0.28355	0.007556	-0.15409	-0.23989
CDH11	-0.2785	-0.2698	0.019428	0.211525	0.002338	0.527965	-0.11157	-0.07836
CDH12	0.163996	0.705595	0.256445	0.017249	-0.07496	-0.02326	0.340065	-0.03178
CDH13	-0.36955	-0.49736	-0.02987	-0.29592	0.072569	0.067944	-0.45517	-0.27309
CDH15	0.093867	0.112635	-0.26527	0.252968	-0.08505	-0.12655	0.225584	0.009345
CDH16	-0.30428	0.028519	0.085437	-0.20065	-0.09812	0.064144	0.338853	0.173761
CDH17	0.490488	0.085155	0.237854	-0.10267	0.140057	-0.0709	0.258795	0.105104
CDH18	-0.07011	-0.00993	0.483847	-0.26508	-0.01582	0.148192	0.013332	0.438764
CDH19	-0.48738	-0.34647	0.087339	-0.11153	0.023558	0.16488	-0.13295	0.201347
CDH2	-0.29684	-0.14867	-0.00887	-0.14022	-0.03564	-0.32208	-0.24292	-0.23625
CDH20	-0.22085	-0.24355	-0.04214	-0.06158	-0.11733	0.230362	-0.30863	0.102016
CDH22	-0.05904	0.551404	0.417331	0.160187	-0.23194	0.043277	0.214239	0.008877
CDH23	-0.312	0.005419	0.162334	-0.14334	0.094106	0.025292	-0.59275	0.347054
CDH24	-0.30179	-0.17402	-0.04155	0.015489	-0.0604	0.301488	0.539614	0.033205
CDH26	0.375137	-0.02971	0.301007	0.005914	0.037646	-0.23509	-0.15005	0.331352
CDH3	0.242459	-0.06738	0.133925	0.455675	0.32037	0.296659	0.314089	0.146133
CDH4	0.021125	0.507307	-0.01401	-0.23275	-0.16732	0.119022	-0.07725	0.23495
CDH5	-0.34185	-0.32128	0.257074	0.123548	0.322193	0.1676	-0.16457	0.254185
CDH6	-0.18441	-0.33899	-0.14527	-0.09644	-0.28337	-0.01936	-0.34515	0.087994
CDH7	0.452028	0.285889	0.182641	-0.09949	-0.00812	0.019894	-0.35842	-0.4733
CDH8	0.381146	-0.25437	-0.25773	-0.26755	-0.04885	-0.02076	-0.24675	-0.10418
CDH9	-0.00439	0.356446	0.215692	-0.35489	-0.08642	-0.12052	0.663504	0.37943
CDIPT	0.239327	0.66539	0.4159	0.030094	0.273826	-0.09586	0.034	-0.14251
CDK10	0.288424	0.101768	0.241513	0.169696	0.397245	0.068054	0.132725	0.179201
CDK2	-0.28348	-0.25	-0.13859	0.24298	0.32714	0.020795	0.400891	0.381858
CDK2AP1	-0.53917	-0.24164	0.110566	0.018711	0.043422	-0.04586	NA	NA
CDK2AP2	0.209739	0.524486	0.236095	0.235253	0.030963	-0.03332	0.235661	-0.1437
CDK3	-0.44299	-0.47913	0.067941	-0.08391	0.132182	-0.18089	0.314217	0.268028
CDK4	0.397658	0.111015	-0.21948	0.371249	-0.08047	0.01744	-0.13467	-0.29902

CDK5	0.443243	0.683489	0.153771	0.054585	0.086646	-0.45398	0.303861	-0.06568
CDK5R1	-0.04297	0.127911	0.243849	-0.03025	0.161914	-0.04045	0.09324	-0.03738
CDK5R2	0.187655	0.417058	0.263138	-0.21111	0.025767	-0.29455	0.485119	-0.06769
CDK5RAP1	0.528006	0.405317	0.222784	0.213848	0.371598	0.266329	0.28418	-0.09252
CDK5RAP2	-0.14278	0.119681	-0.13648	0.15711	-0.01164	0.172012	0.096568	-0.0037
CDK5RAP3	-0.19691	-0.27165	0.066926	0.361795	0.282517	0.312127	0.337332	0.466717
CDK6	-0.25719	-0.48472	0.131542	0.050449	0.085397	0.083225	0.334053	-0.16432
CDK7	0.326957	-0.276	0.13751	-0.49687	-0.23711	-0.30364	-0.01473	-0.41679
CDK8	0.157719	-0.40566	0.10499	-0.38722	-0.15334	-0.15023	0.217084	0.075777
CDK9	-0.13171	0.261233	0.059298	0.092404	-0.07734	0.377451	0.040656	0.24124
CDKAL1	0.243153	0.349705	-0.18968	0.124429	-0.07136	-0.2926	0.507397	0.291161
CDKL1	-0.4553	-0.45523	-0.19922	-0.23048	-0.29088	0.078452	0.21027	0.49376
CDKL2	-0.21618	-0.53197	-0.02416	-0.17605	-0.22196	-0.25638	0.07148	-0.00287
CDKL3	-0.1854	-0.34051	0.054245	-0.26442	-0.15567	-0.50215	-0.13508	-0.05761
CDKL4	-0.03002	0.296059	NA	NA	NA	NA	-0.10887	0.011065
CDKL5	0.23325	0.224289	-0.17285	-0.2323	-0.07641	-0.05047	0.028682	-0.10753
CDKN1A	-0.21499	0.47821	0.400787	-0.21507	-0.08146	-0.01634	-0.3904	-0.02807
CDKN1B	0.468671	-0.40392	-0.32806	-0.00387	0.152988	-0.26211	-0.15751	-0.23854
CDKN1C	0.021787	-0.2022	0.1443	-0.05881	-0.17819	-0.05081	0.190225	-0.02767
CDKN2A	0.047303	0.491345	-0.13619	-0.0861	-0.15585	-0.11228	-0.03186	-0.01488
CDKN2AIP	-0.18269	0.514827	-0.30187	-0.37751	-0.09477	0.004123	NA	NA
CDKN2AIPN	NA	NA	0.069058	0.079531	0.130032	-0.41684	NA	NA
CDKN2B	-0.26313	-0.19088	-0.08773	-0.01322	-0.24459	-0.47069	-0.09867	-0.07953
CDKN2C	-0.20076	-0.317	-0.13647	0.246796	0.23599	-0.14974	-0.28036	-0.17207
CDKN2D	-0.42209	0.28642	-0.13439	0.187327	0.033412	-0.24869	-0.25936	-0.08583
CDKN3	-0.14815	-0.02226	-0.41434	-0.01441	-0.11343	-0.21871	0.164654	-0.20181
CDO1	0.301758	-0.05015	-0.00202	0.214355	-0.15042	-0.19916	-0.46386	-0.06873
CDON	0.158977	0.327297	0.050379	-0.25418	0.1062	0.11396	-0.22788	-0.11007
CDR1	0.128692	0.158786	-0.39796	0.062149	-0.19874	-0.12198	-0.30699	0.050342
CDR2	-0.12975	-0.12975	-0.29121	-0.16671	0.260958	-0.17662	-0.04796	-0.00903
CDR2L	-0.43603	0.141771	0.177339	0.041748	0.042851	0.172947	-0.08477	0.156918
CDRT1	0.139224	-0.18938	-0.08404	-0.00519	-0.09615	0.114035	-0.29487	0.073526
CDRT15	0.015719	0.338519	-0.09194	-0.04475	0.167392	0.102998	NA	NA
CDRT4	-0.14531	0.012572	-0.03181	0.001266	0.117792	0.049536	NA	NA
CDS1	-0.04425	0.248425	-0.03627	-0.16685	0.049763	-0.58615	0.253521	0.074747
CDS2	0.172785	0.246732	-0.30447	-0.22066	-0.08384	-0.32968	0.670863	0.343997
CDSN	0.111429	0.318119	0.338955	-0.10587	-0.02415	-0.0957	0.124767	-0.16021
CDT1	-0.03267	0.566572	0.024007	0.240542	0.489852	0.144087	0.304103	-0.04736
CDV3	-0.24967	-0.54071	-0.18747	0.41583	-0.16416	0.212718	-0.08347	-0.00216
CDX1	0.490525	0.173478	0.265151	0.141483	0.091535	-0.10068	0.193632	-0.16637
CDX2	0.48284	0.153633	0.210076	0.197972	0.481527	-0.09568	0.2651	-0.0363
CDX4	0.304682	0.242949	-0.06925	-0.19041	-0.01215	0.111003	-0.33098	0.03459
CDY1B	0.125415	0.504948	NA	NA	NA	NA	-0.04657	-0.11105
CDY2A	0.117732	0.483321	0.126278	-0.04882	0.309567	0.035616	0.669894	0.254566
CDYL	0.286544	0.558595	-0.43868	0.039842	-0.15481	-0.2656	-0.12071	-0.19147
CDYL2	-0.08275	-0.02583	0.343841	0.560462	-0.00447	0.252905	0.762011	0.375535
CEACAM1	0.252338	0.212357	0.070767	0.011526	0.000587	0.091373	0.135258	0.014219
CEACAM19	0.053146	-0.05969	-0.06682	-0.28463	0.18803	-0.07901	-0.23006	-0.2626
CEACAM20	-0.16737	-0.08903	0.15363	-0.11799	0.088723	-0.30816	-0.50067	0.486274
CEACAM21	-0.19776	0.304047	0.3021	0.495692	-0.01158	0.34014	0.127392	0.545587
CEACAM3	-0.52879	0.100572	0.106909	0.080029	0.345569	-0.03966	-0.5002	-0.34318
CEACAM4	0.151256	0.555906	0.029825	0.108421	-0.07872	-0.12605	-0.32325	-0.00582

CEACAM5	0.102809	-0.10721	0.044775	0.231647	0.318087	0.263084	0.005645	-0.4475
CEACAM6	0.22329	0.119386	0.112518	0.172638	0.149681	-0.06927	0.229679	-0.05215
CEACAM7	-0.20564	0.119916	-0.08394	-0.06729	-0.07029	-0.00983	-0.11235	-0.39079
CEACAM8	-0.02173	-0.05955	0.025799	-0.31506	-0.02481	-0.22385	-0.04822	-0.14267
CEBPA	0.302984	0.083373	0.006654	0.032696	-0.13035	0.466331	0.161221	0.216826
CEBPB	0.015139	-0.087	0.339327	0.144526	0.37044	0.145301	-0.03155	0.010735
CEBPD	0.075568	0.028732	0.489469	-0.04785	-0.05076	0.218683	-0.30858	-0.07363
CEBPE	-0.1008	0.14817	0.445922	0.039757	0.350725	-0.07594	-0.30423	0.094104
CEBPG	0.144285	-0.03458	-0.42337	-0.11715	-0.17392	-0.15164	0.192896	0.237315
CEBPZ	0.456592	-0.23075	-0.20774	-0.1894	-0.02535	-0.14916	0.055043	-0.03297
CECR1	-0.16916	0.557392	0.328984	-0.05064	-0.17331	0.12577	-0.22761	0.351549
CECR2	NA	NA	0.41096	-0.20071	-0.14794	0.149516	NA	NA
CECR5	0.20127	0.405133	0.114362	0.366266	0.350214	0.291282	0.508078	0.189267
CECR6	-0.32392	-0.22224	-0.25195	0.090539	0.021801	0.173885	0.073585	0.048092
CEL	0.382566	0.59204	0.537339	-0.29493	0.142602	-0.16185	0.214065	0.081067
CELSR1	0.112829	0.166841	-0.29419	0.513487	0.179437	0.324968	-0.44596	-0.60546
CELSR2	0.028146	-0.24186	0.092655	-0.00985	0.37848	0.082949	0.264386	-0.2075
CELSR3	0.26209	0.546499	0.071031	0.059218	0.090262	0.160466	0.223366	-0.04666
CEMP1	NA	NA	-0.1359	0.199387	-0.04914	-0.07766	-0.06746	-0.29925
CEND1	-0.10985	-0.11524	-0.00429	-0.07554	-0.07241	0.210776	NA	NA
CENPA	0.004089	-0.15587	-0.30236	0.154058	0.054414	-0.10507	0.279452	-0.10578
CENPB	0.026579	0.385464	-0.00306	-0.00758	0.195879	0.07053	-0.05588	-0.40981
CENPC1	3.5E-05	-0.3801	-0.43612	-0.0458	-0.35015	-0.07049	-0.35517	-0.45709
CENPE	-0.30295	-0.34312	-0.32038	0.34795	0.033245	-0.02155	0.082338	-0.0687
CENPF	-0.04	0.114111	-0.47782	0.238644	-0.05649	-0.00966	0.411572	0.228623
CENPH	0.180647	-0.37364	0.073283	0.184846	0.169275	-0.07986	0.689992	0.371855
CENPI	-0.01159	-0.11877	-0.36409	0.13337	0.024424	-0.10478	0.061741	0.083579
CENPJ	0.073711	-0.71016	-0.02956	-0.00613	0.242655	-0.06383	0.064313	-0.11857
CENPK	-0.17847	-0.29041	-0.33751	0.098067	-0.0606	-0.14238	NA	NA
CENPL	-0.3858	-0.4144	-0.26744	0.074041	-0.08354	0.026824	NA	NA
CENPM	-0.27245	0.044006	-0.03126	0.289761	-0.03888	-0.09419	0.380728	0.090743
CENPN	0.549904	0.030084	-0.31591	0.064117	0.003439	-0.31896	NA	NA
CENPO	-0.38292	-0.04147	-0.13365	0.301502	0.074802	-0.06579	0.43641	0.372564
CENPP	0.18057	-0.01267	0.112523	0.246544	0.209456	-0.14364	NA	NA
CENPQ	0.091121	-0.44738	-0.33446	-0.07148	-0.14957	-0.17889	NA	NA
CENPT	0.066576	0.399936	0.072922	0.203343	0.156618	-0.24021	NA	NA
CENPV	NA	NA	-0.14871	0.140915	0.086022	-0.21936	NA	NA
CEP110	-0.23373	-0.72461	-0.32767	0.570507	0.09159	0.487536	NA	NA
CEP120	NA	NA	-0.16476	0.103566	0.038452	0.168422	NA	NA
CEP135	-0.20274	0.027807	-0.16363	0.181257	-0.13551	0.274766	0.185936	0.460908
CEP152	0.035514	-0.01203	-0.29052	0.11496	0.051774	-0.00858	0.210986	0.238176
CEP164	0.123507	0.484444	0.237285	0.249169	0.184652	0.584096	0.143997	0.461926
CEP170	-0.13797	0.556737	-0.06227	0.069182	-0.31193	0.258646	0.286727	0.217303
CEP192	-0.03445	-0.36131	-0.21984	-0.2009	0.127537	-0.0375	-0.12715	0.217777
CEP250	0.341662	-0.26085	0.318829	0.267738	0.441301	0.312581	0.332683	-0.00715
CEP27	-0.11228	0.505562	-0.21069	0.122794	-0.34371	-0.16528	-0.56786	-0.55808
CEP290	-0.15305	-0.56062	-0.03068	0.250588	0.134439	0.106261	0.074261	0.269673
CEP350	-0.18207	-0.65633	-0.20175	-0.14438	-0.01377	-0.00855	NA	NA
CEP55	-0.26914	-0.21217	-0.13266	0.023112	0.325713	-0.04885	0.178985	0.120001
CEP57	0.00489	-0.21952	-0.25434	-0.08139	0.048369	-0.14344	0.173473	0.038764
CEP63	0.090421	-0.57447	0.020827	0.168796	-0.0925	0.104674	-0.37198	0.089232
CEP68	0.344463	-0.33867	0.286175	-0.09024	0.242655	0.52825	0.270385	-0.22851

CEP70	-0.34456	0.422527	-0.32131	-0.21799	-0.24443	-0.46975	-0.07475	-0.15665
CEP72	0.045652	-0.55117	0.041063	0.141399	0.31307	0.216434	0.624182	0.412957
CEP76	0.107943	0.530115	-0.18614	-0.02684	-0.18844	-0.34342	0.197368	0.286166
CEP78	NA	NA	-0.15385	0.239952	0.095836	-0.07659	NA	NA
CEP97	NA	NA	-0.08538	-0.08701	-0.02729	0.121778	NA	NA
CEPT1	-0.26976	-0.19759	0.088381	-0.02907	0.09415	-0.29292	0.126317	0.088771
CER1	0.058843	0.059848	0.094907	-0.063	-0.12854	0.033345	0.489029	0.327567
CERCAM	NA	NA	0.127887	-0.0314	-0.26818	-0.06202	NA	NA
CERK	-0.15266	-0.39228	0.273672	0.14916	0.086309	-0.06018	0.074604	0.187486
CERKL	0.010142	0.568441	NA	NA	NA	NA	0.264409	0.397976
CES1	NA	NA	0.153812	-0.08715	0.147364	-0.10213	0.185846	-0.09599
CES2	0.400375	0.211122	0.026704	-0.06676	0.254696	0.046846	-0.25538	-0.04148
CES3	0.006426	-0.4107	-0.15819	0.11042	-0.03568	-0.19641	0.211911	0.071387
CES4	0.081465	0.103486	0.188712	-0.089	0.028184	-0.10962	0.449133	0.206586
CES7	0.073676	0.122845	0.111322	-0.31376	0.063517	0.0357	0.195047	-0.03652
CETN1	0.463061	0.707649	-0.02587	0.093788	0.510942	0.062263	0.186638	0.061153
CETN2	0.195653	-0.20316	-0.17361	-0.12528	-0.14363	-0.26476	-0.12223	-0.22917
CETN3	0.133998	-0.41186	0.066565	-0.06429	-0.26409	-0.39241	-0.00458	-0.34146
CETP	-0.46508	0.044728	0.431063	0.500306	0.059958	0.238221	-0.61003	0.176097
CFB	0.216094	-0.07988	0.538881	0.419629	0.370597	0.109563	0.028395	0.561462
CFC1	NA	NA	0.033083	0.161015	0.199208	-0.30879	0.07073	0.326919
CFD	-0.45386	-0.13094	0.141709	-0.25545	-0.25386	-0.1678	-0.29247	-0.01893
CFDP1	0.07494	-0.42843	-0.3193	0.251961	-0.00465	0.07119	-0.2051	-0.38209
CFH	-0.49181	-0.39715	0.125564	0.140609	-0.24221	0.266363	-0.33773	-0.21724
CFHR1	0.047522	-0.60596	-0.07425	-0.13267	-0.21832	0.234272	0.001552	-0.01037
CFHR2	0.076792	0.024989	-0.02561	0.078237	-0.20138	-0.06194	0.38553	0.401182
CFHR3	-0.08639	0.225476	0.21584	0.101017	-0.36214	0.289944	-0.0363	-0.11069
CFHR4	0.057957	-0.36768	-0.09908	0.13097	-0.30158	0.082337	0.563053	-0.07292
CFHR5	-0.20816	0.246784	0.119497	0.069445	0.106949	0.206233	-0.0957	0.121101
CFI	-0.24497	-0.12594	0.255464	0.261841	-0.17576	0.43551	-0.37252	-0.10973
CFL1	-0.15825	0.229475	-0.05328	0.271726	-0.0374	-0.18309	0.182844	0.041968
CFL2	-0.1021	0.177725	-0.26445	-0.11242	-0.23244	0.170628	-0.24328	-0.30827
CFLAR	-0.16338	0.570834	0.293911	0.137797	-0.12772	0.254614	-0.60691	-0.43665
CFP	-0.42712	0.239078	0.205825	0.480894	0.055343	0.339818	NA	NA
CFTR	0.537242	0.138418	0.170813	-0.264	0.056212	0.133573	0.03443	-0.23669
CGA	0.081977	0.136682	0.000316	0.241913	-0.50431	0.108999	-0.5155	-0.43104
CGB	NA	NA	-0.10884	-0.24064	0.115739	0.300132	0.626037	0.399495
CGB1	-0.12522	0.434956	-0.16011	0.133361	-0.1562	0.025139	0.44124	0.428747
CGB2	0.08585	0.067714	0.284475	-0.00343	-0.07284	0.146962	0.199907	0.370631
CGB5	0.078272	0.720708	NA	NA	NA	NA	0.503772	0.387737
CGB7	0.222809	-0.35495	NA	NA	NA	NA	-0.09986	-0.17064
CGGBP1	-0.17327	0.408643	-0.31669	-0.21496	-0.27372	0.015218	-0.16755	0.319564
CGN	-0.08798	0.202791	0.106857	-0.04479	0.382602	0.21925	0.456879	0.090643
CGNL1	0.009628	-0.22684	-0.26605	0.126172	-0.01369	0.264724	0.005106	0.012103
CGREF1	-0.01139	0.038101	-0.25536	0.234956	0.168829	-0.42117	0.324798	-0.22117
CGRRF1	-0.26893	-0.60576	-0.14638	-0.51792	-0.51038	-0.51273	-0.48245	-0.25135
CH25H	-0.01756	0.082885	-0.19315	0.083848	-0.05338	0.1313	-0.15806	0.170262
CHAC1	0.10044	0.421927	0.323698	0.3294	-0.11735	-0.23595	-0.46047	-0.30873
CHAC2	0.132365	0.217589	-0.3799	0.091267	-0.31284	-0.07376	-0.14376	-0.32939
CHAD	-0.02214	0.371962	0.190969	-0.20548	-0.03922	-0.48617	-0.01798	-0.19617
CHADL	NA	NA	0.010461	-0.18713	0.035329	-0.11652	NA	NA
CHAF1A	0.198384	0.642976	-0.37252	0.408997	0.214344	0.124505	-0.20934	-0.35466

CHAF1B	-0.12399	-0.20078	-0.18965	0.202485	0.115804	-0.01322	0.214709	0.024095
CHAT	0.289962	0.511043	-0.24174	-0.14616	-0.23192	-0.02094	0.154609	-0.1851
CHCHD1	0.058606	-0.60018	-0.23914	-0.02095	0.048352	-0.28837	0.266709	0.070841
CHCHD10	NA	NA	0.243803	0.084076	0.133548	-0.03491	NA	NA
CHCHD2	NA	NA	-0.14453	0.276936	-0.08013	-0.6117	-0.53372	-0.33222
CHCHD3	0.3188	-0.15204	-0.04399	0.275169	0.008993	-0.16591	-0.45807	-0.60793
CHCHD4	0.144824	0.133342	-0.15913	0.186509	-0.02997	-0.25004	-0.09714	-0.3041
CHCHD5	0.177692	0.39901	0.278506	0.091739	-0.14985	-0.14389	0.449831	0.265667
CHCHD6	0.385258	0.264837	0.37099	0.065191	0.047517	-0.04545	0.12452	-0.40782
CHCHD7	0.123065	-0.37289	0.107715	-0.23384	-0.13693	0.019902	0.322359	0.089081
CHCHD8	0.205337	-0.63646	-0.07947	0.023823	0.074568	0.044943	0.504964	0.303754
CHD1	0.010783	-0.09935	0.042256	-0.17369	-0.21308	0.065318	-0.43496	-0.30459
CHD1L	-0.27292	-0.40188	-0.21961	0.19124	0.057735	0.101249	0.37102	0.514701
CHD2	0.16135	0.088216	-0.28091	0.001555	0.261445	0.153483	0.073801	0.365943
CHD3	0.086303	-0.1161	-0.06999	0.132739	-0.09033	0.259254	-0.572	-0.40145
CHD4	0.488396	-0.11532	0.098608	0.207379	0.316581	0.491088	0.450181	0.18758
CHD5	0.08215	0.126073	-0.07135	-0.16413	-0.077	0.389528	-0.1302	-0.39344
CHD6	0.322969	0.036128	-0.03862	0.133856	0.134665	0.427233	-0.12642	-0.38081
CHD7	0.035856	-0.56202	0.08307	-0.01619	-0.08054	0.235824	0.658284	0.629147
CHD8	-0.08378	0.375732	-0.15522	0.40836	0.030498	0.406934	-0.36057	-0.32339
CHD9	0.185398	0.636575	0.062548	-0.23417	0.102467	-0.12413	-0.13733	-0.08392
CHDH	-0.00701	-0.04403	0.081708	0.173829	0.400585	0.045295	0.47084	0.378756
CHEK1	0.040521	-0.21491	-0.27031	0.139227	-0.06404	-0.19221	0.521957	0.537454
CHEK2	0.024286	0.075138	-0.20632	0.091891	0.123782	-0.25689	0.591225	0.17274
CHERP	0.392128	-0.183	-0.20077	0.191187	0.39272	0.35274	0.193074	0.027643
CHFR	-0.34719	-0.06058	0.036071	0.158206	0.016252	-0.36863	-0.20038	0.485478
CHGA	-0.15855	0.229757	0.001733	0.154654	-0.10336	-0.03539	0.108637	-0.10892
CHGB	0.046477	-0.1716	-0.24779	0.382958	-0.17735	-0.19577	0.198349	0.112765
CHI3L1	-0.12854	-0.02588	0.146506	0.327744	0.250265	0.023835	0.198357	-0.42285
CHI3L2	-0.45436	0.016504	0.160954	0.636414	0.095234	0.577485	-0.32384	0.30899
CHIA	-0.16526	0.223293	0.134998	0.187347	0.126099	0.140603	0.393088	0.11169
CHIC1	NA	NA	-0.22512	0.085529	-0.16124	-0.17984	0.144565	-0.00428
CHIC2	-0.4896	-0.00224	-0.11487	-0.09564	-0.17101	-0.35628	-0.28765	-0.07778
CHID1	-0.14949	-0.06206	0.075104	0.329499	0.139337	-0.06507	-0.41339	-0.25949
CHIT1	0.402026	0.544946	0.421824	0.078063	-0.29027	0.24276	NA	NA
CHKA	0.17251	0.140175	-0.13539	-0.17394	0.152912	0.027051	0.364099	0.218199
CHKB	-0.28292	-0.59923	0.123531	0.269036	-0.12017	0.264297	-0.07996	0.408481
CHL1	-0.17446	-0.39514	-0.06009	-0.0006	-0.29962	0.423425	0.098095	0.420551
CHM	0.093027	-0.16432	-0.32508	-0.26598	-0.1152	-0.28131	-0.17123	0.026562
CHML	-0.1761	0.400597	-0.03271	-0.12283	0.104894	7.26E-05	0.271614	0.153475
CHMP1A	NA	NA	0.235483	0.032076	0.242994	-0.0998	NA	NA
CHMP1B	0.193135	-0.42014	-0.24378	-0.07956	-0.25267	-0.1536	0.119673	0.329862
CHMP2A	0.020055	-0.21896	0.200838	-0.34081	-0.09699	-0.35728	0.174972	-0.10174
CHMP2B	0.016069	-0.3594	-0.49111	-0.39393	-0.25097	-0.32153	0.153629	0.037648
CHMP4A	0.087292	-0.05377	-0.2109	0.143452	0.140877	-0.06604	-0.52213	-0.34072
CHMP4B	0.565307	-0.0201	0.245612	0.099321	0.22862	-0.08669	0.539921	0.25212
CHMP4C	0.230782	-0.08885	0.149242	-0.09626	-0.12224	-0.3425	-0.02631	-0.30422
CHMP5	-0.25278	-0.59804	-0.34008	-0.25324	-0.22011	-0.48843	-0.37203	-0.15386
CHMP6	0.32057	0.84969	0.451252	-0.22751	0.168271	-0.01472	0.179335	0.065636
CHMP7	0.072827	0.689462	0.305098	0.26595	-0.03852	0.038331	0.352814	0.123747
CHN1	-0.1553	-0.10502	0.038624	0.097324	-0.12279	0.295923	-0.08208	-0.13446
CHN2	0.210453	-0.24692	0.169157	0.079198	0.247133	-0.44832	0.146836	0.567056

CHODL	0.102178	-0.31665	-0.06214	0.111088	-0.02184	0.490914	-0.53753	-0.03166
CHORDC1	0.013654	-0.67105	-0.36408	-0.09409	-0.01207	-0.20253	-0.7727	-0.56879
CHP	-0.17283	-0.06987	-0.2032	-0.16322	-0.18839	-0.45662	-0.42905	-0.58257
CHP2	NA	NA	0.249187	-0.16557	0.087772	-0.08213	NA	NA
CHPF	-0.14649	0.292231	0.564636	0.347875	0.280815	-0.21969	0.137901	0.191736
CHPT1	0.179274	-0.42993	0.190726	-0.08305	0.065187	-0.2611	-0.27979	-0.44311
CHRAC1	0.281213	-0.43566	-0.08811	0.007738	-0.16843	-0.14094	0.628496	0.519378
CHRD	-0.15336	0.282992	0.165249	0.131976	-0.05609	0.401563	-0.13437	0.130771
CHRDL1	0.498791	-0.0751	-0.0929	-0.06941	-0.08457	-0.06334	-0.05479	0.028977
CHRDL2	0.117299	-0.08853	0.056059	0.236581	0.084336	0.016603	-0.3664	-0.28518
CHRFAM7A	0.064646	0.213664	-0.17308	-0.08446	-0.37302	-0.28968	0.022238	0.482955
CHRM1	0.144819	0.178204	0.151968	-0.35676	0.01432	-0.06899	-0.0675	-0.25208
CHRM2	-0.17229	0.025901	0.059832	0.165552	-0.1912	0.294167	0.31754	0.505113
CHRM3	0.164987	0.128652	0.0926	0.29167	-0.13782	0.169473	NA	NA
CHRM4	0.095534	0.316446	-0.12909	-0.10343	-0.04366	-0.19264	0.12318	0.479231
CHRM5	0.370244	0.419773	0.010561	-0.02378	-0.09213	-0.04191	-0.28662	-0.26458
CHRNA1	0.193679	-0.07319	0.113269	-0.16051	0.102917	-0.38252	0.187767	-0.10211
CHRNA10	-0.02443	0.694603	-0.37776	-0.04562	0.121205	0.100092	-0.13595	0.361825
CHRNA2	0.508663	0.284488	-0.06132	-0.23909	-0.18306	0.259444	0.369098	0.415665
CHRNA3	-0.17127	-0.21823	0.109938	0.003721	-0.00849	0.06841	-0.27872	-0.45149
CHRNA4	0.226274	0.502098	0.001608	-0.16484	-0.01168	-0.12783	-0.17623	-0.34147
CHRNA5	-0.1842	0.402794	-0.32999	0.271855	-0.02463	-0.36581	-0.36651	-0.16712
CHRNA6	0.405283	-0.45921	0.314451	0.20438	-0.2154	-0.10136	0.286736	0.27146
CHRNA7	-0.17186	0.525193	-0.01351	0.055673	-0.29532	-0.15409	0.094812	0.631875
CHRNA9	0.004548	0.41655	0.048131	0.008315	-0.06602	0.183851	-0.26673	-0.22416
CHRNB1	-0.14125	-0.36781	0.301419	-0.09419	0.166265	-0.04454	0.137157	0.191037
CHRNB2	0.36549	-0.02907	0.278325	-0.27217	-0.16456	0.070345	0.023771	-0.25132
CHRNB3	0.203581	0.611276	-0.04128	-0.11889	-0.02183	-0.3962	0.146127	0.288373
CHRNB4	-0.06091	0.260616	0.139115	-0.17112	0.270128	0.175391	NA	NA
CHRND	0.183049	0.29731	0.033613	-0.11586	0.324778	0.17003	-0.55679	-0.42713
CHRNE	0.083056	0.537952	0.167685	0.080224	0.076701	0.112259	-0.53507	-0.36591
CHRNG	0.157128	0.488256	-0.16875	-0.02476	-0.19353	0.173828	0.174873	0.219344
CHST1	-0.29792	-0.32605	0.114593	0.031629	0.291415	0.04029	-0.17038	-0.0888
CHST10	0.268759	0.107256	0.278762	0.077002	-0.15219	0.201107	-0.11328	0.162102
CHST11	-0.14923	0.771758	0.383156	0.471022	0.017081	0.38283	-0.05375	0.0134
CHST12	-0.12612	0.595784	0.177582	0.042189	0.002539	0.190501	-0.63352	-0.36986
CHST13	-0.07556	0.111681	0.369776	0.065689	-0.28555	0.065811	0.14718	0.022744
CHST14	NA	NA	0.27296	0.236663	-0.0188	0.235317	NA	NA
CHST2	-0.31532	-0.26889	0.170888	0.686204	-0.14061	0.325557	-0.28807	-0.35767
CHST3	-0.12049	0.128789	-0.04392	0.099923	0.080739	0.175333	-0.16818	-0.06932
CHST4	0.226024	0.226829	-0.12807	0.300746	-0.00341	-0.45578	-0.2255	0.215993
CHST5	-0.09058	0.091592	-0.03381	-0.14198	-0.0768	-0.13179	-0.21038	-0.12009
CHST6	-0.36305	0.255137	-0.20452	-0.28828	-0.20659	-0.30649	-0.09504	0.243237
CHST7	-0.06525	-0.02664	-0.25849	0.026823	-0.07186	-0.26674	-0.20209	-0.1419
CHST8	0.153802	0.288023	0.178118	0.695242	-0.19535	0.223909	0.281589	-0.08496
CHST9	0.146646	0.069313	0.028949	-0.26993	-0.24677	-0.32392	-0.03382	-0.35302
CHSY1	-0.2501	-0.05959	0.001008	0.003519	0.070547	0.423065	-0.58484	-0.00273
CHSY3	NA	NA	-0.03268	0.080939	-0.11874	-0.07959	NA	NA
CHTF18	0.155595	-0.22012	-0.19596	0.305359	0.337828	0.175025	0.486777	0.411827
CHUK	-0.23078	0.145491	-0.13644	-0.07904	-0.01629	-0.24456	-0.40187	0.107644
CHURC1	-0.26383	-0.40622	-0.27403	-0.45755	-0.34236	-0.09781	-0.30623	0.193904
CIAO1	-0.28521	-0.65907	0.200148	-0.00611	-0.11324	0.027132	-0.40258	-0.29419

CIAPIN1	0.28308	-0.03712	0.125974	0.036671	0.247915	0.214466	0.397619	0.36411
CIB1	-0.03394	0.260482	-0.25717	0.069813	0.030485	-0.12336	0.184004	0.336374
CIB2	0.283329	0.570059	-0.0305	-0.21373	-0.15322	-0.39931	-0.12545	0.045706
CIB3	0.132069	0.234805	0.052269	-0.22165	0.004719	-0.1368	-0.21674	-0.01963
CIB4	-0.23818	0.19099	0.006557	-0.38565	-0.00294	-0.0381	0.267749	0.04892
CIC	0.152444	0.38175	0.051736	0.12886	-0.04201	0.436337	-0.54163	-0.55771
CIDEA	-0.07967	-0.06854	-0.15598	-0.13124	0.221277	-0.00193	0.168927	0.249074
CIDEB	-0.33957	-0.16148	0.306951	0.262512	-0.25164	0.207464	-0.06579	0.545036
CIDEC	0.145088	-0.04285	-0.14024	-0.14343	-0.09134	-0.36014	0.120744	0.290128
CIITA	0.129957	0.081234	0.313314	0.551375	-0.12404	0.705167	-0.36301	0.224576
CILP	0.033198	-0.13249	-0.19906	-0.02974	-0.10941	-0.14905	-0.18742	-0.37565
CILP2	0.223544	0.48733	-0.04792	-0.19172	-0.03927	-0.0185	-0.50009	-0.13437
CINP	0.151745	0.623234	-0.2768	-0.01391	-0.27374	-0.44915	0.045668	0.127533
CIP29	-0.40175	0.114608	-0.29612	-0.04192	-0.1611	-0.37846	-0.10348	-0.04868
CIR	0.15077	-0.62395	-0.13902	0.137753	-0.08698	-0.21457	-0.43083	-0.48878
CIRBP	-0.53243	-0.44601	0.102086	0.124635	0.208578	0.409484	-0.25211	0.04874
CIRH1A	0.371469	0.309822	0.106214	-0.03759	0.207824	0.301468	0.379304	-0.01801
CISD1	NA	NA	-0.05218	-0.39228	0.156129	-0.51974	NA	NA
CISD2	NA	NA	-0.16512	0.087398	-0.26023	-0.35497	NA	NA
CISD3	NA	NA	0.313542	0.087868	-0.27518	-0.17242	NA	NA
CISH	0.064525	0.418265	0.186101	0.243492	0.124949	-0.00674	0.139977	0.212203
CIT	-0.31228	-0.3391	-0.39438	0.00106	0.346023	-0.0762	-0.23399	-0.19959
CITED1	0.197127	0.655892	0.173241	-0.32631	0.134083	0.181278	0.04016	0.474209
CITED2	-0.04176	-0.59683	-0.32829	-0.24659	-0.25001	-0.40078	-0.4307	-0.45997
CITED4	-0.01167	0.001948	0.00719	-0.12513	0.124635	0.342024	-0.12646	-0.1861
CIZ1	0.3457	0.404311	0.397768	0.205038	0.426105	0.187534	0.458589	0.335263
CKAP2	0.219358	-0.25628	-0.35716	0.004219	0.036151	-0.23833	0.059671	-0.21575
CKAP2L	0.256384	-0.30158	-0.06985	0.040975	-0.04596	-0.04697	NA	NA
CKAP4	-0.49964	-0.42515	-0.25061	0.337477	0.088418	-0.3294	0.091997	0.03776
CKAP5	0.089386	-0.26776	-0.11142	0.163024	0.054085	0.134243	0.513272	0.294851
CKB	0.038974	0.159555	-0.1751	-0.09561	-0.10127	0.196725	-0.00151	-0.29927
CKLF	-0.31279	-0.2954	-0.06789	-0.03819	0.237134	-0.07618	-0.03265	0.125986
CKM	0.127974	0.080048	-0.2859	-0.0851	-0.05076	-0.15386	0.25785	-0.0608
CKMT1A	0.101793	0.033097	-0.01701	0.223148	0.123375	-0.00697	0.126065	0.01605
CKMT1B	0.13623	0.020593	-0.05385	-0.04949	0.299222	0.150946	0.035784	-0.06112
CKMT2	0.372715	0.260308	0.420728	-0.11215	0.15342	-0.03722	0.044961	-0.08638
CKS1B	-0.10083	-0.47734	-0.25984	-0.05288	-0.2293	-0.30945	0.383214	-0.02542
CKS2	0.230065	-0.12998	-0.19314	0.060692	-0.23535	-0.11989	0.24138	-0.09223
CLASP1	0.001969	0.00549	-0.12587	0.093616	0.005426	0.01658	0.35373	-0.01365
CLASP2	-0.14277	-0.65212	-0.38055	-0.20872	0.002741	0.146308	0.467571	0.331323
CLC	-0.21691	0.006006	0.031695	-0.24573	-0.22862	-0.26572	-0.3485	-0.10812
CLCA1	-0.20385	0.185398	-0.05471	0.173454	0.015533	0.212943	-0.16014	-0.11854
CLCA2	-0.08703	0.777861	-0.03531	-0.06544	-0.22825	0.082353	-0.24187	0.155492
CLCA3	0.175422	0.155263	-0.11185	-0.07556	-0.13336	-0.20414	0.231222	0.174558
CLCA4	-0.15041	-0.03939	-0.14867	-0.02515	0.059302	-0.07297	-0.09837	-0.3027
CLCC1	0.149379	-0.4131	0.280855	0.021092	0.377666	0.011358	0.081422	-0.07538
CLCF1	0.012253	0.320186	-0.31885	-0.07347	-0.01775	0.257222	0.153656	0.105097
CLCN1	0.086157	0.040227	0.222241	-0.25006	0.096416	-0.00562	-0.30562	-0.44335
CLCN2	0.338107	-0.08763	0.101032	-0.24963	0.452795	-0.06189	0.060627	-0.16516
CLCN3	-0.1529	0.013523	-0.10624	0.142671	-0.05657	-0.56717	0.100177	-0.18736
CLCN4	-0.06692	0.519213	-0.00598	-0.04201	-0.06012	0.11749	0.069565	0.2377
CLCN5	0.322968	0.480497	-0.32615	-0.17026	-0.01167	-0.5405	0.59403	0.644546

CLCN6	-0.14505	-0.08052	-0.17543	0.002392	0.365302	0.216965	-0.22769	0.194105
CLCN7	0.087319	0.40787	0.373092	-0.07747	0.419493	0.070264	-0.0851	0.132769
CLCNKA	0.01823	0.372957	0.163678	-0.10721	-0.0578	0.17894	-0.15382	0.036539
CLCNKB	0.037427	-0.14115	0.022	0.065339	-0.274	0.022623	0.015777	0.03963
CLDN1	0.12131	-0.21318	0.333831	-0.1404	-0.10262	0.051666	0.449359	0.088303
CLDN10	0.311012	0.415999	-0.06631	-0.04862	-0.30679	-0.0874	-0.12685	0.010255
CLDN11	-0.19481	-0.48351	-0.14363	0.030832	0.144736	-0.18652	NA	NA
CLDN12	0.193738	0.066039	-0.36684	-0.09836	0.125768	-0.23463	0.085545	-0.0583
CLDN14	-0.01125	0.476185	-0.14921	-0.21449	-0.16929	-0.25224	-0.32059	-0.07103
CLDN15	0.464846	0.287923	0.334093	-0.06057	0.370839	0.28387	0.188659	0.4072
CLDN16	-0.05854	-0.1921	0.244488	-0.09374	0.413788	-0.17531	-0.13717	-0.02363
CLDN17	0.064462	0.525548	0.270823	-0.4595	-0.37496	-0.14164	0.097646	0.018214
CLDN18	-0.511	0.229654	-0.15182	-0.13408	0.262349	0.076073	-0.16971	-0.46517
CLDN19	-0.10288	-0.26737	0.163749	0.016031	-0.19189	0.101105	-0.20068	-0.07886
CLDN2	-0.08749	0.30239	0.264082	0.098858	0.117559	-0.03171	0.255845	0.578605
CLDN20	0.170738	0.108571	NA	NA	NA	NA	0.087371	-0.45895
CLDN22	NA	NA	0.025996	0.031808	-0.32532	0.21227	NA	NA
CLDN23	-0.08321	0.141368	0.124367	-0.21829	0.020702	-0.00537	-0.0141	-0.04442
CLDN3	0.39959	0.252888	0.270345	-0.11209	0.079187	0.051385	-0.07161	-0.36266
CLDN4	-0.53003	-0.20879	0.1802	-0.18251	0.114365	0.072119	-0.18098	-0.48102
CLDN5	-0.33592	0.025187	0.084577	0.118822	0.040743	9.15E-05	-0.3728	-0.17723
CLDN6	0.502828	0.674602	0.007579	0.08767	0.008354	0.386111	-0.18593	-0.65369
CLDN7	-0.09554	0.132219	-0.12201	0.21379	-0.02555	0.206343	0.510563	0.253079
CLDN8	-0.04925	-0.22253	-0.01961	-0.24974	-0.11293	-0.29774	0.038996	-0.24449
CLDN9	0.198122	0.356301	-0.14175	-0.21334	0.031971	-0.44757	0.410913	0.306746
CLDND1	-0.48562	-0.1845	-0.21613	-0.17943	-0.19551	-0.03885	0.110996	0.119989
CLDND2	NA	NA	-0.14943	-0.34446	-0.05763	-0.24657	0.345215	0.262033
CLEC10A	0.286105	0.215422	0.088451	-0.35165	0.02433	0.01116	-0.53945	0.42884
CLEC11A	0.100041	-0.09404	0.076522	-0.01349	-0.03549	0.134249	0.188431	0.191426
CLEC12A	-0.20229	-0.0444	0.089728	-0.03299	0.003299	-0.15264	-0.2892	-0.05792
CLEC12B	0.217057	0.693278	-0.07821	0.197099	0.038509	-0.06879	0.085615	-0.41913
CLEC14A	-0.15304	-0.30514	-0.00174	0.21054	0.021189	0.054878	-0.3702	-0.1326
CLEC16A	NA	NA	-0.05555	-0.02816	0.18012	-0.01482	NA	NA
CLEC1A	-0.35463	-0.29672	0.173612	-0.12592	-0.37836	-0.21426	-0.13334	0.465488
CLEC1B	0.120941	-0.01102	-0.18721	-0.17768	-0.21716	-0.31294	-0.16014	0.009915
CLEC2A	-0.22034	0.251706	NA	NA	NA	NA	NA	NA
CLEC2B	-0.33215	-0.28816	0.086916	-0.07779	-0.1996	0.207385	-0.47035	0.004194
CLEC2D	-0.23541	-0.37178	-0.11202	0.627016	-0.17671	0.478152	-0.51348	-0.53929
CLEC2L	NA	NA	0.297265	-0.19497	0.316505	-0.03737	NA	NA
CLEC3A	-0.09762	0.406517	0.191399	0.025504	-0.09145	0.019409	-0.21013	-0.24603
CLEC3B	-0.37647	-0.31398	0.091876	-0.02726	-0.21861	-0.16157	-0.37391	-0.16326
CLEC4A	-0.63709	-0.29244	-0.10397	0.120612	-0.20654	-0.165	-0.51212	-0.08044
CLEC4C	-0.09465	0.640935	-0.00105	-0.20581	0.220667	0.089405	-0.3676	-0.02439
CLEC4D	-0.36526	-0.04778	0.111468	0.082378	-0.14984	-0.13002	-0.18472	-0.08337
CLEC4E	-0.54541	-0.09814	0.177687	-0.03219	-0.04811	0.01717	-0.27305	-0.06742
CLEC4F	-0.20385	-0.0487	0.448689	-0.03001	-0.19096	0.236029	0.00622	0.464531
CLEC4G	NA	NA	0.040146	-0.42522	-0.25987	-0.33933	-0.45774	0.249766
CLEC4M	NA	NA	-0.08397	-0.0253	0.035309	0.254162	0.389554	0.172975
CLEC5A	-0.35097	0.071157	0.040864	-0.41722	-0.27763	-0.13899	-0.22147	-0.08937
CLEC6A	0.083721	0.764001	NA	NA	NA	NA	-0.15941	-0.19792
CLEC7A	-0.54218	-0.06877	-0.00652	-0.11223	-0.15637	-0.20908	-0.42745	0.218933
CLEC9A	-0.0035	0.150889	NA	NA	NA	NA	0.136017	0.503711

CLECL1	NA	NA	-0.03872	0.133934	-0.32213	0.371158	NA	NA
CLGN	-0.11412	0.729716	-0.00941	0.067486	0.055747	-0.0345	-0.09469	-0.09104
CLIC1	0.081374	0.452852	-0.04453	0.230995	0.295108	0.090131	0.202338	-0.11621
CLIC2	-0.31974	-0.26719	-0.16853	-0.09403	-0.37373	0.03358	-0.6582	-0.61433
CLIC3	-0.3326	0.026355	0.139234	-0.07618	-0.11199	-0.07866	-0.00955	-0.25998
CLIC4	-0.23997	-0.60867	-0.27549	-0.23319	-0.05771	-0.30388	-0.38318	-0.34341
CLIC5	0.242853	0.046154	0.070934	-0.13903	-0.21467	-0.10493	-0.02971	0.025983
CLIC6	-0.04325	0.151168	0.128803	0.440301	0.057703	0.022575	-0.28483	-0.00161
CLINT1	-0.1625	-0.62229	0.07067	-0.00287	0.001517	0.104815	NA	NA
CLIP1	-0.31757	-0.1098	-0.02523	-0.23469	0.125065	0.102662	-0.54729	-0.25969
CLIP2	-0.04356	0.442386	0.465939	-0.02268	0.235841	-0.00912	0.222559	-0.1415
CLIP3	-0.16635	0.036503	0.049066	-0.06171	-0.0774	0.207861	NA	NA
CLIP4	-0.50057	-0.4209	-0.04119	-0.06483	-0.14089	0.156146	NA	NA
CLK1	-0.33497	-0.62858	-0.31616	0.007232	-0.06028	0.099249	-0.52036	-0.4376
CLK2	-0.05387	0.545609	-0.0692	0.347098	0.166761	0.135467	0.167495	0.40956
CLK3	-0.31562	-0.34843	0.156245	0.257721	-0.00202	-0.0261	-0.16433	-0.06119
CLK4	0.253224	-0.34279	-0.11515	-0.01904	-0.04121	0.138802	0.046763	0.151826
CLLU1	0.397066	0.203554	NA	NA	NA	NA	0.106852	0.262218
CLLU1OS	0.501654	0.58106	NA	NA	NA	NA	-0.24118	-0.05184
CLMN	0.188246	0.343663	-0.38322	0.071736	0.075797	-0.26786	-0.35593	-0.47723
CLN3	0.392129	0.243504	0.188294	-0.00295	0.501297	-0.05129	0.332357	0.164665
CLN5	0.227979	0.262902	0.143664	-0.29846	0.110024	-0.40164	0.19904	-0.13476
CLN6	0.273148	0.681515	0.066008	0.231734	0.397926	0.002865	0.224925	0.274015
CLN8	-0.29427	-0.36054	0.239765	0.054076	0.019595	0.23893	-0.47439	-0.23295
CLNS1A	0.392489	-0.29797	-0.07547	0.017532	0.144064	0.24339	0.589127	0.108594
CLOCK	-0.2208	0.443402	-0.11958	-0.07143	-0.04744	-0.17933	-0.13678	0.060134
CLP1	0.152918	0.286105	-0.22421	0.037852	-0.21345	-0.21447	NA	NA
CLPB	0.01321	-0.29268	0.28819	0.289884	0.19482	0.072175	0.226952	0.147755
CLPP	0.133997	0.429801	-0.16129	0.172357	0.09424	-0.22576	0.482699	0.190533
CLPS	0.062925	-0.00622	0.122884	-0.27584	0.01626	-0.1637	0.17311	0.012303
CLPTM1	-0.05183	0.627316	0.192435	0.230552	0.075077	0.126369	0.545302	0.39377
CLPTM1L	0.276623	0.235504	0.246919	0.436751	0.359032	0.025635	NA	NA
CLPX	0.177918	-0.57643	-0.27878	-0.11413	0.075887	0.025917	0.526483	0.252209
CLRN1	0.037534	0.709824	-0.14234	0.190243	0.001704	0.156187	NA	NA
CLRN3	-0.1873	-0.00133	0.36732	-0.221	0.034897	-0.18405	0.200995	-0.09615
CLSPN	0.203718	0.351233	-0.03319	0.406887	0.028445	-0.10999	-0.51223	-0.10494
CLSTN1	-0.23042	-0.2933	0.076301	0.307131	0.269073	0.337388	0.296638	0.037108
CLSTN2	0.165846	-0.39005	0.275381	-0.02332	-0.18933	0.230776	0.216615	-0.6151
CLSTN3	0.011935	-0.10401	0.073507	0.359413	-0.03084	0.295517	-0.07459	0.042031
CLTA	0.093683	-0.47807	0.153202	0.122565	0.067773	0.10502	0.247725	0.068183
CLTB	-0.25973	0.504742	0.431184	0.160401	-0.00095	-0.30435	-0.34619	-0.40967
CLTC	0.26121	-0.04826	0.14251	-0.35365	-0.12871	-0.02073	-0.48164	-0.27453
CLTCL1	-0.10356	-0.13393	0.298361	-0.32809	0.134068	0.097231	-0.03736	-0.0472
CLU	0.140655	0.194333	0.384072	0.575372	0.150227	0.49508	-0.34341	-0.12488
CLUAP1	0.354412	-0.33954	-0.0688	-0.10642	0.173054	-0.12543	0.589633	0.293413
CLUL1	-0.06485	0.759113	0.413807	0.043075	0.100189	0.035741	0.350857	0.208017
CLYBL	-0.18655	0.303837	0.214213	-0.13397	0.077025	-0.05419	0.236647	-0.02145
CMA1	0.119103	0.644305	-0.1442	-0.01473	0.263522	-0.25871	-0.17183	0.08613
CMAH	NA	NA	-0.18027	-0.20468	-0.42522	0.109621	-0.21457	0.159447
CMAS	0.258265	-0.28324	-0.44537	0.268667	-0.20743	-0.35619	0.309603	0.203422
CMBL	-0.42194	-0.01947	0.138693	-0.14599	-0.16942	0.203366	NA	NA
CMC1	NA	NA	-0.13677	-0.02583	-0.07426	-0.36658	NA	NA

CMIP	-0.20881	0.404238	-0.04557	0.003175	-0.15527	0.089198	-0.44487	-0.24695
CMKLR1	-0.47745	-0.38594	0.232227	-0.05299	-0.2779	0.143681	-0.13441	0.254064
CMPK1	NA	NA	0.200666	-0.05311	0.409541	-0.24367	NA	NA
CMTM1	0.18097	0.615265	-0.32711	-0.01368	0.180942	-0.19671	-0.01322	0.426919
CMTM2	-0.48168	0.400646	0.048029	0.007279	0.056232	-0.51506	-0.37856	0.188505
CMTM3	-0.20202	0.049615	0.121894	0.217752	-0.06008	0.490088	-0.04134	0.36399
CMTM4	-0.08669	0.142539	0.072404	0.005584	0.315993	-0.12954	0.211458	-0.04765
CMTM5	0.041921	0.654718	-0.02012	-0.08743	-0.19063	-0.23766	-0.2314	-0.44074
CMTM6	-0.46362	-0.59936	-0.16583	-0.02004	-0.04899	0.007858	NA	NA
CMTM7	-0.04204	-0.07618	0.245078	0.669531	0.112856	0.540213	-0.0007	0.038174
CMTM8	0.42298	0.071133	0.154935	0.144945	0.262038	-0.23492	0.606654	0.242392
CMYA5	0.007609	-0.34522	0.09629	-0.03897	-0.19164	-0.20933	-0.49456	-0.30169
CNBD1	0.250082	0.240719	0.050716	-0.04839	-0.1374	0.160893	-0.02091	0.077963
CNBP	-0.22426	-0.6869	-0.06595	-0.07964	0.104584	-0.26424	-0.09424	-0.0598
CNDP1	0.097013	0.194999	0.104641	-0.21184	-0.27855	0.047069	0.303944	0.628423
CNDP2	0.109837	0.035675	0.266548	0.216311	0.145377	0.015933	-0.08102	0.469641
CNFN	-0.13461	0.502799	0.298794	0.087997	0.112733	-0.22715	0.333157	0.259838
CNGA1	-0.13009	-0.12392	-0.20838	-0.39666	-0.11278	-0.50666	0.238009	0.121375
CNGA2	-0.06385	0.076883	NA	NA	NA	NA	0.598492	0.456755
CNGA3	-0.03086	-0.31381	0.058335	-0.14284	-0.23317	-0.29719	-0.25499	-0.25352
CNGA4	NA	NA	0.069351	-0.22117	0.139807	0.168131	0.488724	0.085034
CNGB1	-0.04614	-0.51052	-0.116	-0.09615	0.266486	-0.28359	-0.58233	-0.57248
CNGB3	0.062292	-0.05284	-0.14727	-0.03031	0.011993	-0.48694	-0.06993	0.327544
CNIH	-0.28342	-0.66489	-0.25134	-0.24068	-0.37365	-0.44086	0.257247	0.016998
CNIH2	0.234794	0.32023	-0.03964	-0.08464	-0.04504	-0.23388	0.087436	0.184723
CNIH3	-0.27792	-0.0635	-0.21114	-0.00613	-0.16587	-0.02929	-0.03891	-0.30919
CNIH4	0.042319	0.115823	-0.17925	-0.33989	-0.42618	-0.34337	-0.36223	-0.43352
CNKSR1	0.192594	0.177797	0.223562	0.147641	0.307015	-0.04203	0.575005	0.265459
CNKSR2	0.048415	-0.38184	0.032063	-0.22746	-0.01935	-0.17782	0.36726	0.116879
CNKSR3	0.395354	-0.16755	-0.178	-0.11048	0.406837	-0.14532	-0.23549	-0.13086
CNN1	-0.07579	-0.11424	0.212751	0.026526	0.105998	0.113407	-0.14369	-0.28362
CNN2	0.035528	-0.03951	0.255252	0.443125	0.373252	0.198453	-0.275	-0.29704
CNN3	0.124858	-0.12543	0.235366	-0.18341	-0.10577	-0.06505	-0.05606	0.054982
CNNM1	-0.00344	0.610164	-0.23318	-0.16932	-0.2884	-0.0466	0.393045	0.251476
CNNM2	-0.33209	0.114808	0.210778	-0.22505	-0.04229	-0.20533	-0.44072	0.035117
CNNM3	0.141372	0.646476	0.309394	0.202694	0.426282	0.286708	0.187074	-0.20643
CNNM4	0.313904	0.097713	-0.18733	0.065227	0.160625	-0.02244	0.17763	0.017456
CNO	0.193795	0.550429	0.017989	0.168013	0.212975	-0.03383	-0.31939	-0.22785
CNOT1	0.422895	0.205113	-0.13168	-0.08215	0.044731	0.014361	0.281312	0.040954
CNOT10	0.343095	0.458721	-0.34154	0.05716	0.016016	0.072964	0.666647	0.48404
CNOT2	-0.25853	-0.60829	-0.46706	-0.24816	-0.04962	-0.10427	0.256993	0.184107
CNOT3	0.242207	0.392941	-0.11485	0.20518	0.032754	0.197985	0.564728	0.396143
CNOT4	0.281955	0.473849	-0.11962	-0.11776	0.049654	0.485909	0.509914	0.098155
CNOT6	-0.11536	-0.42834	-0.28625	-0.08406	-0.109	-0.01014	0.255149	0.222811
CNOT6L	-0.03626	0.035878	-0.05495	0.007936	0.023621	0.066035	0.460282	0.418756
CNOT7	0.021808	-0.63812	0.053048	0.121692	-0.25075	-0.1029	0.162757	-0.00553
CNOT8	-0.00503	-0.69032	0.137025	0.175132	-0.06025	-0.29553	-0.12672	-0.39662
CNP	-0.01905	-0.31513	0.180972	0.019981	0.135413	0.052834	0.11191	0.106763
CNPY2	NA	NA	-0.23352	0.059991	-0.19101	-0.39872	NA	NA
CNPY3	NA	NA	0.276142	0.605227	0.167437	0.044909	NA	NA
CNPY4	NA	NA	0.089319	-0.10837	0.005138	-0.04532	NA	NA
CNR1	-0.58381	-0.29445	-0.01013	0.315737	-0.10798	0.627367	-0.31185	-0.39062

CNR2	0.345045	0.798602	0.097758	0.775863	-0.01888	0.627894	0.300751	-0.03261
CNRIP1	NA	NA	-0.04396	-0.1351	-0.17287	-0.15709	NA	NA
CNTD1	-0.17216	-0.09377	-0.10134	-0.20662	0.013388	-0.641	NA	NA
CNTD2	-0.247	0.139711	-0.08764	-0.1428	0.153733	0.062238	NA	NA
CNTF	-0.09447	0.208779	-0.36809	-0.11107	0.073153	0.166177	-0.23578	-0.45107
CNTFR	0.025134	0.522424	0.329914	-0.22689	-0.11661	0.020955	0.099929	0.275001
CNTLN	NA	NA	-0.16064	-0.0271	-0.19928	-0.24941	NA	NA
CNTN1	-0.22476	-0.44378	0.232929	-0.06373	-0.12241	-0.19916	-0.03439	0.102173
CNTN2	0.179432	-0.34947	0.211781	0.388086	-0.22526	0.601061	-0.28944	-0.1386
CNTN3	-0.03914	-0.36008	-0.3426	-0.22526	-0.32258	-0.1864	0.264703	0.191169
CNTN4	0.231462	0.026706	-0.01866	0.132961	-0.21481	0.113694	0.016931	0.118312
CNTN5	0.354454	0.20713	-0.25948	-0.01521	-0.14491	0.179597	0.390486	0.2082
CNTN6	0.223346	-0.30661	-0.17895	-0.18184	-0.05989	-0.19211	0.190042	-0.00379
CNTNAP1	-0.21365	-0.10733	0.233897	-0.02605	0.047651	0.029112	-0.3953	-0.2731
CNTNAP2	0.335868	0.00039	0.095503	0.081281	-0.14615	-0.15115	0.370728	0.256262
CNTNAP3	-0.3867	-0.1858	-0.22573	-0.07356	0.08949	-0.04682	-0.1367	0.063502
CNTNAP4	0.18664	0.267579	-0.00065	-0.14184	0.136626	0.043246	0.386129	0.250129
CNTNAP5	0.217398	0.035868	0.097451	-0.08283	-0.13048	-0.17143	0.459675	0.326286
CNTROB	-0.01665	-0.02428	0.203653	0.109539	0.102655	0.056294	0.079579	0.225457
COASY	-0.33411	-0.6344	0.258612	0.211033	0.255329	0.152771	-0.03416	-0.04167
COBL	0.300338	0.026775	0.033053	-0.25592	0.152911	-0.25387	0.347376	0.201404
COBLL1	0.324483	0.186384	0.32076	-0.09433	0.141686	-0.3206	0.189726	0.127453
COBRA1	-0.23309	0.286327	0.269249	0.086916	0.341737	0.136522	0.464853	0.215789
COCH	0.016379	-0.14741	-0.24801	-0.20163	-0.08856	-0.11831	0.110331	0.102116
COG1	-0.04404	-0.58459	0.347713	0.032509	0.064661	0.211275	0.619092	0.405134
COG2	0.267279	0.66275	-0.15729	-0.17118	0.064508	0.106711	0.386863	-0.04013
COG3	-0.04986	-0.63659	0.069775	-0.12675	0.2957	-0.00611	0.049799	-0.03371
COG4	0.210737	0.378522	-0.01189	0.088124	0.250228	0.131812	-0.01783	-0.06861
COG5	0.395633	0.621483	0.070915	-0.14535	0.094612	-0.57914	0.075626	0.002351
COG6	0.26058	-0.57321	0.039149	-0.16176	0.019234	-0.46699	0.409291	-0.01944
COG7	0.326555	0.691412	0.085419	-0.08465	0.200697	0.133936	0.410366	0.281932
COG8	0.078381	-0.53784	0.242096	0.226645	0.09768	0.121101	0.334341	0.174112
COIL	0.461156	0.307064	0.039742	0.127473	-0.00275	0.176511	0.511922	0.257466
COL10A1	-0.13152	-0.00032	0.078337	-0.39332	-0.31939	-0.33734	0.089883	0.102062
COL11A1	-0.16662	-0.18534	-0.0194	0.113826	-0.19378	0.129759	0.180843	-0.08532
COL11A2	0.195033	0.417099	-0.23768	0.693245	-0.16424	0.651833	-0.04413	-0.38232
COL12A1	-0.34564	-0.30717	-0.06486	-0.03021	-0.17209	0.213983	0.124053	0.034733
COL13A1	-0.24834	0.217566	-0.14743	-0.03457	-0.03252	0.302693	-0.46054	-0.30933
COL14A1	-0.09066	-0.28169	0.028984	0.064542	-0.2017	0.092287	NA	NA
COL15A1	-0.29656	-0.35055	0.056892	-0.00191	0.006781	0.28161	-0.24162	0.035259
COL16A1	-0.17079	0.003398	0.032284	0.271334	-0.04611	0.264282	-0.24763	-0.16119
COL17A1	0.128042	0.054147	0.249295	-0.29276	0.040084	-0.0147	-0.06305	0.048181
COL18A1	-0.27757	-0.0408	0.124736	0.062827	0.022352	0.053823	-0.28273	0.028131
COL19A1	-0.03639	0.10376	0.039034	0.114263	0.105772	0.360521	0.35806	0.240337
COL1A1	-0.18138	-0.21533	0.020516	0.179186	-0.07305	0.069196	0.061613	0.009356
COL1A2	-0.26991	-0.46426	0.022636	0.008229	-0.15942	0.216238	-0.16532	-0.23578
COL20A1	-0.03885	-0.19416	-0.15974	-0.15698	0.015198	-0.31092	-0.34332	-0.46858
COL21A1	0.021913	-0.48632	-0.27372	-0.20095	-0.20432	0.07675	-0.47122	-0.1997
COL22A1	-0.30699	0.207688	0.05861	0.380869	-0.01492	0.36493	-0.47336	-0.27363
COL23A1	-0.30644	0.395885	0.298173	0.039672	-0.14149	-0.10705	-0.08254	0.268072
COL24A1	-0.45469	-0.47747	0.021576	-0.07946	-0.34887	-0.00406	0.074212	0.273188
COL25A1	-0.22634	-0.03354	-0.27153	-0.0899	0.014727	0.157266	-0.34098	-0.0714

COL27A1	0.060077	-0.51604	0.127053	0.154896	0.137826	0.291682	0.006768	0.207018
COL2A1	0.501844	0.208042	-0.30571	0.173122	-0.13071	0.145544	-0.19281	-0.13146
COL3A1	-0.26516	-0.35489	0.011412	0.040795	-0.07926	0.037033	-0.04719	-0.0706
COL4A1	-0.2002	-0.14939	0.052576	0.035886	0.111704	-0.01889	-0.36018	-0.18946
COL4A2	-0.18418	-0.20064	0.110502	0.100986	0.149457	0.285407	-0.427	-0.3015
COL4A3	-0.00262	-0.014	0.039005	0.504797	0.098707	0.521046	0.1091	0.291931
COL4A3BP	-0.04266	-0.37282	0.004797	-0.10854	-0.12023	-0.04747	-0.25503	-0.29197
COL4A4	0.342405	-0.16796	0.123166	0.258233	0.103833	-0.01088	-0.50242	-0.2528
COL4A5	0.095154	-0.07658	-0.15104	-0.30861	-0.05306	0.063182	0.073002	0.339955
COL4A6	0.279964	0.24682	-0.09243	-0.31031	0.027686	0.047721	-0.0638	0.120791
COL5A1	-0.30339	-0.19968	0.072465	-0.11016	-0.07489	0.145049	-0.20657	-0.32146
COL5A2	-0.26675	-0.28109	0.036008	-0.09713	-0.18103	0.017924	-0.11312	-0.11849
COL5A3	-0.27017	-0.22909	0.029188	0.175909	-0.09382	0.274757	-0.07907	0.187743
COL6A1	-0.27642	-0.18075	0.052246	0.05685	-0.07499	0.414033	-0.12377	-0.03244
COL6A2	-0.30679	-0.14335	0.297699	-0.00941	0.073679	0.329032	-0.1896	-0.1423
COL6A3	-0.25346	-0.32932	0.056693	-0.02876	-0.12398	0.182975	-0.1009	-0.06935
COL6A6	NA	NA	-0.21183	0.274818	-0.05808	-0.22967	NA	NA
COL7A1	-0.51323	0.034347	-0.03622	0.339098	0.060497	0.264652	0.037409	0.288045
COL8A1	-0.09581	-0.09732	0.083872	-0.08221	-0.07984	-0.24705	-0.21147	-0.333
COL8A2	-0.01939	0.580354	-0.10089	0.002389	-0.11158	0.263055	-0.29601	-0.34049
COL9A1	0.224743	-0.01774	-0.21165	-0.04006	-0.03977	-0.01326	0.084101	-0.30586
COL9A2	0.01555	0.413045	-0.06648	0.488336	0.071306	0.226905	0.175113	0.192093
COL9A3	0.092986	-0.20882	0.250201	0.238176	-0.08365	0.060795	0.253535	0.145978
COLEC10	0.176478	0.295243	-0.39812	0.000683	0.031919	-0.08576	0.304736	-0.23228
COLEC11	0.210609	-0.284	-0.2083	-0.02054	0.098392	-0.33523	-0.15509	0.247336
COLEC12	-0.27731	-0.31318	-0.13816	-0.17945	-0.28644	0.193398	-0.26333	0.078663
COLQ	0.062546	0.540348	-0.08448	-0.11398	-0.15242	0.487576	-0.11674	0.2507
COMMD1	0.268998	0.448717	0.165631	-0.38161	-0.35925	-0.33807	-0.44374	-0.39411
COMMD10	0.168902	-0.53164	0.164193	-0.00179	-0.04773	-0.03859	0.409225	0.294227
COMMD2	-0.06875	0.218871	-0.2465	-0.3129	-0.18762	0.057878	0.062579	-0.31813
COMMD3	-0.33417	-0.54125	0.069282	-0.09933	-0.05191	0.028476	-0.11901	0.037377
COMMD4	0.093201	0.141382	-0.06237	-0.21662	-0.09318	-0.44157	0.231253	0.077979
COMMD5	0.266485	0.593056	0.274029	-0.09773	-0.00932	0.141248	0.377322	0.147218
COMMD6	0.34487	-0.50516	0.056748	-0.32026	-0.06408	-0.10374	0.021048	-0.30261
COMMD7	0.34978	-0.00124	0.02248	0.356315	0.206345	-0.044	0.323845	-0.35337
COMMD8	0.155277	-0.60239	-0.22136	-0.31937	-0.24838	-0.45202	-0.27693	-0.55031
COMMD9	0.105084	0.255733	0.182102	-0.42843	-0.05464	-0.2146	-0.16133	-0.10647
COMP	0.129965	-0.21495	0.310441	-0.19606	-0.0384	0.020261	-0.12739	-0.38507
COMT	-0.02884	-0.09362	0.33516	0.175688	0.17732	-0.14519	0.481262	0.036572
COMTD1	0.155783	0.347571	-0.00062	0.074799	0.343977	-0.43167	0.147572	0.125071
COPA	-0.08019	-0.12469	0.056337	0.102342	0.040596	-0.01115	0.286988	0.146621
COPB1	-0.12846	-0.62971	-0.07566	-0.07861	-0.09335	-0.21873	NA	NA
COPB2	-0.61269	-0.47621	-0.19605	-0.09175	-0.1187	-0.12988	0.385716	0.489726
COPE	0.265344	0.048324	0.074505	0.130407	0.049177	-0.23696	0.121855	0.017509
COPG	-0.31051	-0.1052	0.13272	0.401544	0.104984	-0.21953	0.472135	0.324717
COPG2	0.160412	0.008097	0.398506	-0.14908	0.084131	-0.30775	-0.06487	0.1529
COPS2	-0.05961	-0.50604	-0.0618	-0.09711	-0.19353	0.055326	-0.65748	-0.38318
COPS3	-0.13451	0.012418	-0.08494	0.02707	-0.36784	-0.0783	-0.04002	-0.04493
COPS4	-0.04338	-0.42908	-0.17306	-0.31447	-0.46386	-0.17968	-0.1001	-0.27634
COPS5	-0.00491	-0.68553	0.271177	-0.2381	0.006606	0.146024	0.501049	0.247463
COPS6	0.244787	0.184659	0.205206	-0.00296	0.260864	0.190484	0.562486	0.18389
COPS7A	-0.48345	-0.2154	0.054842	0.362552	0.069951	0.090225	-0.61216	-0.33056

COPS7B	0.388197	0.276699	0.177216	0.25024	0.246427	0.04834	0.512533	0.523266
COPS8	0.342687	-0.09849	0.036803	-0.09092	-0.16374	-0.41195	-0.01706	-0.25075
COPZ1	-0.2211	-0.20394	0.102792	0.211054	-0.09165	-0.36952	0.458957	0.418846
COPZ2	-0.37227	-0.06479	-0.01122	-0.01688	-0.30028	-0.47238	-0.32832	-0.19364
COQ10A	-0.10456	0.188864	-0.33875	0.497673	-0.22907	-0.27651	0.120437	0.410661
COQ10B	0.313216	0.259969	-0.02177	-0.06627	-0.27633	-0.19132	0.178313	0.343178
COQ2	-0.30844	-0.09643	-0.09976	0.193397	-0.18246	-0.18836	0.081107	0.098684
COQ3	0.520569	-0.12155	-0.40876	-0.2675	-0.04896	-0.07313	-0.33107	-0.29807
COQ4	0.273719	0.646389	0.260237	-0.10647	0.245173	-0.05785	NA	NA
COQ5	-0.14281	-0.36203	0.325571	-0.37286	0.087445	-0.10785	0.324821	0.315626
COQ6	0.064397	0.246049	0.037988	-0.03471	-0.24431	-0.19438	0.103836	0.600972
COQ7	-0.05721	0.018516	-0.26936	-0.08497	-0.07185	-0.39369	0.148866	0.223632
COQ9	0.078706	0.107639	0.10498	-0.04416	0.17233	-0.05581	0.308686	0.00405
CORIN	0.061968	0.152671	-0.00915	-0.00956	-0.06222	0.146833	0.332796	-0.19898
CORO1A	-0.27037	0.057633	0.251807	0.750556	0.053117	0.78597	-0.26576	0.147214
CORO1B	0.207438	0.350989	0.327035	0.115574	0.221401	-0.2132	0.461717	0.171159
CORO1C	-0.1956	-0.27156	-0.25634	-0.19684	-0.02932	-0.0329	-0.39709	-0.2144
CORO2A	0.047448	0.166309	0.239906	0.135896	0.169893	-0.29967	0.285064	0.277745
CORO2B	-0.07866	0.146916	0.303555	-0.09198	0.136276	-0.3435	-0.35305	0.273683
CORO6	-0.27149	0.067618	0.120749	-0.10491	-0.04675	-0.01135	-0.34894	-0.12913
CORO7	0.108193	0.292735	0.341954	0.109255	0.436953	0.227056	0.120767	0.264712
CORT	0.158067	-0.58346	-0.21521	0.050278	0.111857	-0.43292	-0.18394	-0.15484
COTL1	-0.09137	0.125815	-0.13952	0.589959	0.087654	0.36171	-0.229	0.052432
COX10	0.274136	0.191766	-0.06736	0.130537	0.03682	-0.03551	0.091424	-0.21091
COX11	0.241997	-0.39461	-0.14584	-0.04488	-0.2354	-0.29823	0.033751	0.199702
COX15	0.085391	0.685432	-0.31173	-0.04327	0.129764	-0.0365	0.421462	0.109518
COX16	NA	NA	-0.39436	-0.08333	-0.40895	-0.09414	NA	NA
COX17	-0.27915	-0.48011	-0.09698	-0.28815	-0.21647	-0.2713	0.037118	0.077553
COX18	-0.05385	-0.14526	-0.04802	-0.08691	-0.05033	-0.17283	NA	NA
COX19	0.052706	0.575239	-0.09139	-0.10583	0.433465	0.19884	NA	NA
COX4I1	0.194749	0.527166	0.193394	-0.01676	0.084912	-0.03216	0.321372	0.274432
COX4I2	-0.2047	-0.51085	0.121588	0.028586	-0.14258	-0.01854	0.05959	0.163671
COX4NB	0.123641	-0.48645	-0.08776	-0.0016	0.182991	0.094428	0.431871	0.276695
COX5A	0.284763	0.193654	-0.04563	-0.02152	-0.16347	-0.19143	-0.0284	-0.17091
COX5B	0.330904	-0.25646	0.264789	-0.3815	-0.04174	-0.38556	-0.0514	-0.25198
COX6A1	-0.12371	-0.10026	-0.18031	-0.20608	-0.117	-0.44219	-0.27388	-0.10681
COX6A2	0.414509	0.035674	0.036684	0.057193	-0.15687	0.252154	0.116358	0.494795
COX6B1	0.012359	-0.68867	0.239106	-0.29704	-0.25128	-0.28386	0.203912	-0.07599
COX6B2	-0.46381	0.033531	-0.01817	-0.27925	0.026159	-0.09632	-0.28476	-0.25902
COX6C	-0.02141	-0.56604	0.178136	-0.2872	-0.16818	-0.28793	-0.21002	-0.39719
COX7A1	-0.30475	-0.15205	0.082308	-0.07565	-0.22048	-0.16648	-0.32261	-0.30631
COX7A2	0.219416	-0.44801	-0.18969	-0.30495	0.098717	-0.51175	-0.01802	-0.06422
COX7A2L	0.125968	-0.63179	0.073527	-0.02914	-0.196	0.125488	NA	NA
COX7B	0.23382	-0.51959	-0.03235	-0.27967	-0.28264	-0.32296	-0.41396	-0.3549
COX7B2	0.507752	-0.02374	-0.15171	0.157901	-0.03456	-0.06515	-0.25673	-0.32734
COX7C	0.225345	-0.47379	-0.11805	-0.25877	-0.3989	-0.55503	0.098385	0.17118
COX8A	-0.23437	0.419346	0.189215	-0.00143	-0.14019	-0.21637	-0.04401	-0.04968
COX8C	0.211827	0.505888	NA	NA	NA	NA	-0.47686	-0.21415
CP	0.154682	-0.30076	0.068517	0.488268	-0.37222	0.492118	-0.03864	0.044333
CP110	-0.14091	-0.28433	-0.20055	-0.2473	0.079492	0.060876	0.10745	-0.0141
CPA1	0.179223	-0.18307	-0.13839	-0.08086	-0.04856	0.004401	0.06962	0.221501
CPA2	0.395402	0.266142	0.229881	0.124743	-0.17228	-0.06353	0.373558	0.263786

CPA3	-0.24799	-0.20376	0.096583	0.239462	0.013086	0.177358	-0.19033	0.039288
CPA4	-0.07236	-0.13636	0.284817	-0.16838	0.490261	0.233885	0.369209	0.144466
CPA5	0.502518	0.667616	-0.05921	-0.12289	-0.13763	-0.14845	-0.23822	-0.29851
CPA6	0.419004	0.145568	-0.16283	-0.18133	-0.29455	-0.04959	0.251687	-0.37216
CPAMD8	-0.18989	0.675484	-0.2174	-0.20438	-0.28406	-0.02678	-0.22785	-0.01271
CPB1	0.028567	0.179398	-0.11565	-0.13784	-0.28804	0.122736	-0.31044	0.079736
CPB2	-0.23551	0.117876	0.166479	-0.43942	-0.01149	0.19601	0.4099	0.558326
CPD	-0.06346	-0.24248	-0.13325	-0.2471	0.029257	-0.32193	-0.0514	-0.08508
CPE	-0.03572	-0.37038	0.025176	-0.01525	0.20653	-0.14074	-0.16932	-0.42977
CPEB1	-0.03773	-0.35703	-0.0216	-0.31523	0.075615	0.081335	-0.34047	-0.24504
CPEB2	0.093297	0.130535	-0.47533	-0.43209	-0.0578	-0.3536	0.349254	0.329248
CPEB3	-0.07797	0.205705	-0.12967	-0.2487	-0.06872	-0.33025	-0.5571	-0.18685
CPEB4	-0.42487	-0.22277	0.379204	-0.12711	0.034583	-0.10016	-0.21931	0.034184
CPLX1	0.174003	-0.07453	0.017429	0.104215	0.197359	0.122598	0.156657	0.341211
CPLX2	0.165431	0.567872	0.271121	-0.03457	0.147515	-0.12919	0.262424	-0.30043
CPLX3	0.251113	0.764302	-0.19603	-0.01896	-0.08508	-0.12197	0.261283	0.010111
CPLX4	0.282142	0.67833	-0.0307	-0.01178	0.035421	0.159302	NA	NA
CPM	-0.17825	0.694114	0.07315	-0.17314	-0.16473	-0.08856	0.358993	0.230969
CPN1	0.266571	0.542043	0.106326	-0.02251	0.306622	0.070847	0.340608	0.357329
CPN2	NA	NA	0.028231	-0.17102	-0.10012	-0.0206	0.335555	-0.01762
CPNE1	0.465729	0.197201	-0.00281	0.162226	0.529809	0.052589	0.477076	0.070566
CPNE2	0.187364	0.144809	0.413134	-0.05038	0.06914	-0.00958	0.603196	0.392797
CPNE3	0.060518	-0.5516	0.251582	-0.24708	-0.19016	-0.16559	0.586572	0.206611
CPNE4	0.186593	-0.27228	0.056961	0.144209	-0.27091	-0.10096	-0.1524	0.06728
CPNE5	0.085628	0.554273	-0.02435	0.494667	-0.23935	0.043329	-0.11404	0.208925
CPNE6	0.046455	-0.26532	-0.37179	-0.16591	0.061117	0.069693	-0.26921	0.053009
CPNE7	0.274358	-0.20049	0.311068	-0.20593	0.305198	0.184573	0.173063	-0.1803
CPNE8	-0.22095	-0.48572	-0.21794	-0.17843	-0.20849	-0.06143	-0.47406	-0.35147
CPNE9	0.439702	0.024377	-0.07907	-0.09398	-0.00112	-0.20636	-0.06344	-0.21715
CPO	0.058401	-0.20024	-0.36842	-0.04242	-0.05498	-0.20202	-0.2641	0.482325
CPOX	-0.419	0.290241	-0.39898	0.195971	0.119831	-0.28419	NA	NA
CPS1	0.027588	-0.17972	-0.04193	0.076073	0.022488	0.12472	-0.16438	0.437645
CPSF1	0.149589	0.607019	0.450307	0.248866	0.230116	-0.06438	0.216559	-0.21865
CPSF2	-0.28298	-0.28545	-0.45115	-0.06028	-0.17899	-0.17453	-0.08063	0.108768
CPSF3	0.27954	0.335137	-0.18144	0.202265	-0.11134	-0.18335	0.10066	-0.34605
CPSF3L	0.09622	0.324286	0.470863	0.068008	0.237311	0.085418	0.145189	0.084613
CPSF4	0.29904	0.654072	0.169283	-0.08612	0.388828	0.127095	0.126824	0.010044
CPSF6	0.24683	-0.30085	-0.47008	0.201116	-0.01249	-0.06448	0.228131	-0.10371
CPT1A	-0.13482	0.016553	0.072531	-0.09939	0.096844	-0.03581	-0.06941	0.29563
CPT1B	0.050984	0.211737	-0.18756	-0.10398	0.018055	0.222643	0.088836	0.399233
CPT1C	-0.21331	0.193644	0.02117	-0.00418	-0.06777	-0.17055	-0.30886	-0.0789
CPT2	-0.15656	-0.04754	0.1999	-0.17823	0.118404	-0.126	0.172318	0.071504
CPVL	-0.00239	-0.2907	-0.00931	-0.05949	-0.54866	0.137921	-0.41544	0.345913
CPXCR1	0.118394	-0.01938	-0.00584	-0.03089	0.21799	-0.12084	-0.3765	-0.48524
CPXM1	-0.22268	-0.16167	0.165039	0.08675	0.018384	0.279111	NA	NA
CPXM2	-0.26747	-0.19908	0.009999	-0.04135	0.017062	0.232946	-0.27421	-0.23717
CPZ	-0.22	-0.61038	0.182098	0.025331	-0.02138	0.199878	-0.17187	0.042012
CR1	0.293431	0.36929	0.227943	0.7469	-0.01393	0.640053	-0.37399	0.419732
CR2	0.214941	0.001309	0.05771	0.734458	-0.24058	0.614548	-0.10626	0.103109
CRABP1	0.477284	0.32136	-0.02211	-0.13932	-0.01949	0.012398	0.029908	-0.18201
CRABP2	-0.33043	-0.33753	-0.18232	-0.21719	-0.00151	-0.30741	0.008196	-0.31904
CRADD	-0.01588	-0.42583	0.133693	-0.59416	-0.13263	-0.43251	-0.07575	0.270997

CRAMP1L	0.141258	-0.28874	-0.18568	-0.01103	0.459536	0.300519	0.463672	0.417148
CRAT	-0.26116	0.692434	0.63621	-0.23956	0.079647	-0.23996	0.050863	0.179219
CRB1	0.347064	0.466549	-0.07829	-0.41074	0.029012	0.122957	-0.2443	-0.27785
CRB2	0.13088	0.370542	0.19539	0.139994	0.189496	0.012781	-0.02447	0.196507
CRB3	0.235658	0.340891	-0.00543	0.098898	0.054583	-0.16621	0.632942	0.240494
CRBN	0.168128	-0.51619	-0.26058	-0.25747	-0.12525	-0.34068	-0.27095	-0.41412
CRCP	NA	NA	0.118883	-0.05648	0.119981	0.278753	NA	NA
CRCT1	0.064579	0.323344	-0.40027	-0.35155	-0.0735	-0.04814	NA	NA
CREB1	0.033444	-0.63746	-0.24172	0.040322	-0.23332	-0.12233	-0.47548	-0.39347
CREB3	-0.08733	0.598721	0.07159	0.194385	-0.09312	-0.1271	-0.55118	-0.45173
CREB3L1	-0.35818	-0.24729	-0.01537	0.235388	-0.23156	0.008546	0.158195	0.096015
CREB3L2	-0.08141	-0.11599	0.005878	0.048512	-0.34425	-0.30119	0.569125	0.333097
CREB3L3	0.443796	-0.12046	0.290193	-0.43473	0.299547	-0.15	0.411507	0.294759
CREB3L4	-0.2403	-0.19223	-0.04467	0.184041	-0.00359	-0.29292	0.221337	-0.13543
CREB5	0.046175	-0.27564	0.077408	-0.22593	0.017364	0.114904	-0.18793	-0.0821
CREBBP	0.105725	-0.58261	-0.47394	-0.00918	0.152787	0.180872	0.159275	0.047523
CREBL2	0.281442	0.55421	-0.03433	-0.11157	-0.15326	-0.108	-0.45391	-0.32953
CREBZF	0.129146	-0.51993	-0.1793	0.04185	0.354587	-0.14969	NA	NA
CREG1	-0.06225	-0.31975	0.130377	-0.31676	-0.25299	-0.15388	0.501764	0.39187
CREG2	-0.35198	0.152406	0.225311	-0.32113	0.022937	0.027012	0.4843	-0.11104
CRELD1	0.016312	-0.07172	0.067996	0.185753	0.160197	0.190409	0.369807	0.112479
CRELD2	-0.52174	0.033104	-0.03505	0.495115	-0.0115	-0.0542	0.264734	0.16887
CREM	-0.07246	-0.22121	-0.06867	-0.07325	-0.26639	0.038815	-0.26245	-0.21747
CRH	0.166156	0.61992	-0.08092	-0.19645	-0.2145	0.005244	-0.63693	-0.40275
CRHBP	0.009539	-0.33266	-0.06295	0.167713	-0.22187	0.02749	-0.02241	-0.28002
CRHR1	0.153453	0.655982	0.196163	-0.00094	0.084977	-0.02392	-0.32272	-0.33768
CRHR2	0.456833	0.70568	0.105963	-0.0321	0.108565	0.007757	0.033558	0.286429
CRIM1	-0.06475	-0.25947	0.218697	-0.17354	0.108709	-0.08522	-0.24341	-0.11144
CRIP1	-0.30785	0.445311	-0.15271	-0.14403	-0.25047	-0.12254	-0.26638	-0.00217
CRIP2	-0.25998	-0.14005	0.160505	0.012146	0.004149	0.120225	-0.03319	-0.02898
CRIP3	-0.15809	0.039566	-0.30606	0.593007	0.134807	0.478619	-0.31284	-0.00138
CRIPAK	-0.13124	-0.15985	0.034127	-0.05607	0.235336	0.412939	0.263954	0.414622
CRIPT	0.35711	-0.20098	-0.09001	-0.44317	-0.15705	-0.38013	0.301503	-0.00539
CRISP1	0.104442	-0.17603	0.048285	-0.10619	0.014936	-0.16109	-0.1247	0.010468
CRISP2	-0.40637	-0.06129	0.205184	-0.05222	0.049758	0.38133	-0.35764	-0.06519
CRISP3	-0.33469	0.281503	-0.34741	-0.30037	-0.01903	0.14502	0.427659	0.221263
CRISPLD1	0.061141	0.122749	0.06542	0.062107	-0.01661	0.087877	-0.62843	-0.39286
CRISPLD2	-0.30412	-0.03983	0.236259	-0.01372	-0.22527	0.269278	-0.15727	0.130936
CRK	-0.38751	-0.31673	-0.11093	-0.01458	0.067416	-0.04653	0.176756	0.169406
CRKL	-0.39506	-0.28007	-0.40811	-0.04875	0.005029	-0.45835	0.568568	0.284253
CRKRS	0.264234	0.288523	-0.02804	-0.27318	0.085697	-0.00601	-0.35699	-0.3769
CRLF1	0.014638	-0.23723	-0.06026	-0.23956	0.012764	-0.44244	0.188519	0.13932
CRLF2	0.242645	0.647929	0.353507	-0.17266	-0.05001	0.162664	-0.0071	0.034778
CRLF3	-0.26402	0.197647	-0.08891	0.071412	-0.28694	0.126405	-0.1215	0.100579
CRLS1	0.129695	-0.648	-0.28168	-0.02028	-0.027	-0.30868	NA	NA
CRMP1	0.118824	0.189529	-0.02892	0.10871	0.172898	-0.05908	-0.12409	0.133238
CRNKL1	0.245227	-0.34494	-0.44802	-0.31464	-0.01765	-0.46189	0.59306	0.35723
CRNN	-0.0291	0.429211	-0.15802	-0.23821	-0.05186	-0.15652	-0.51025	-0.19888
CROCC	-0.03435	0.306087	0.39482	0.014048	0.200696	0.102724	-0.56907	-0.50408
CROP	-0.11015	-0.72907	-0.07638	0.020801	0.024837	-0.19424	-0.22201	0.034871
CROT	0.499943	0.439868	0.115266	-0.25468	0.089297	-0.32297	-0.14364	0.063612
CRP	0.098405	0.119485	0.079296	0.227626	-0.01659	0.264734	0.099381	0.199383

CRTAC1	0.407129	-0.36802	0.132443	-0.00329	0.141706	0.032663	-0.28498	-0.16661
CRTAM	0.142342	0.373659	0.027318	0.245156	-0.4469	0.139929	0.018635	0.238764
CRTAP	-0.20202	-0.31345	0.096467	0.047552	0.295637	-0.2538	-0.16045	-0.14707
CRTC1	0.535985	0.536589	0.16738	-0.00365	-0.00712	0.263735	0.17379	0.173791
CRTC2	-0.22461	-0.10174	0.037192	0.177724	0.029606	0.32934	-0.42404	-0.39175
CRTC3	NA	NA	0.133821	0.190355	0.541657	0.422359	NA	NA
CRX	0.435314	0.076305	0.427418	0.255014	0.368683	0.158669	0.224343	-0.41764
CRY1	-0.26114	-0.54297	-0.37007	0.06266	-0.02884	-0.03222	-0.39495	-0.07571
CRY2	0.089532	0.279132	0.20692	-0.18609	0.222522	-0.02471	-0.3673	-0.12475
CRYAA	0.185032	0.611638	-0.13789	-0.0744	0.161826	0.062281	-0.17576	0.399753
CRYAB	-0.23894	-0.14007	0.212497	-0.09773	-0.17225	0.08824	-0.11295	-0.16279
CRYBA1	-0.00264	-0.06595	0.28735	-0.11314	0.156617	-0.21992	0.279104	-0.11911
CRYBA2	-0.2761	0.099897	-0.08142	0.207668	-0.22991	-0.30642	0.479007	0.269359
CRYBA4	0.25762	-0.15951	0.006856	-0.10098	0.025872	-0.43601	-0.04972	-0.01949
CRYBB1	0.274023	0.414119	0.154018	-0.04133	-0.05692	-0.06172	0.192233	0.099044
CRYBB2	0.190056	-0.1613	-0.02026	0.013151	-0.12072	0.173577	0.521854	0.260135
CRYBB3	0.027433	-0.25623	-0.01845	-0.35982	0.067478	-0.16965	0.146912	0.158845
CRYBG3	NA	NA	-0.48244	-0.14876	0.103878	-0.07562	NA	NA
CRYGA	0.005708	0.089623	0.088823	-0.32208	-0.00655	-0.0459	0.396798	0.298486
CRYGB	-0.09295	-0.21938	-0.43032	-0.20983	0.172614	0.054619	-0.24512	0.461097
CRYGC	0.100503	0.09697	-0.2374	-0.22949	-0.10059	0.109655	0.077661	0.16265
CRYGD	0.142038	0.208391	-0.08287	-0.2936	0.107611	-0.07805	0.322126	0.425028
CRYGN	0.269328	0.422381	0.230834	0.012441	0.015383	0.045457	-0.05148	-0.01747
CRYGS	-0.01932	0.084594	-0.04914	-0.09331	0.004556	0.249365	-0.03801	0.345174
CRYL1	0.141501	0.240964	0.312443	-0.39216	0.319685	-0.16399	0.42911	0.353539
CRYM	-0.02647	0.105789	-0.25878	0.02016	-0.08233	-0.17129	0.173056	-0.25096
CRYZ	-0.20959	-0.31195	-0.07206	-0.18347	-0.10468	0.074224	0.072127	0.177195
CRYZL1	-0.23424	-0.31923	-0.23899	-0.32537	-0.18778	-0.17717	0.064252	-0.04355
CS	0.021153	0.418574	-0.29077	0.221693	0.166164	-0.16088	0.47263	0.456489
CSAD	0.068816	-0.09711	0.012147	-0.05899	0.256605	0.254492	-0.10181	0.407302
CSAG1	-0.30364	0.219862	-0.09736	0.13743	-0.09043	0.154355	0.072066	-0.05821
CSAG2	-0.1241	0.416042	-0.14304	0.159183	-0.08524	0.026949	0.074646	-0.1361
CSDA	0.532942	-0.18685	0.059739	0.218425	-0.12095	0.190498	-0.57517	-0.33591
CSDC2	0.286285	0.20449	-0.09904	-0.14222	-0.11687	-0.23664	-0.28148	-0.12852
CSDE1	-0.03449	0.009263	0.248078	-0.06306	0.115928	-0.1003	-0.4059	-0.51472
CSE1L	0.434001	-0.34905	0.050033	-0.1527	0.225785	0.191534	0.430792	-0.06672
CSF1	-0.18956	-0.26413	0.478993	0.038034	-0.28519	0.243659	0.184038	0.301835
CSF1R	-0.38137	0.039025	0.151606	-0.05202	-0.29398	0.402032	-0.52696	0.14716
CSF2	0.041447	0.113352	-0.0298	0.050661	0.026016	-0.0793	-0.5109	-0.2198
CSF2RA	0.161159	0.335519	0.181497	0.189465	-0.23596	0.36087	-0.64961	-0.08198
CSF2RB	-0.05941	0.745451	0.367594	0.373028	0.020495	0.021251	0.021666	0.298948
CSF3	0.049591	0.235331	0.00677	0.096085	0.122648	-0.07561	-0.33625	-0.03695
CSF3R	-0.5624	-0.245	0.295896	0.019258	0.113844	0.063462	-0.45767	0.201124
CSGALNAC	NA	NA	0.224816	0.317816	0.070155	0.484608	NA	NA
CSGALNAC	NA	NA	-0.05385	0.274215	-0.21182	0.262988	NA	NA
CSH1	0.230172	0.585515	-0.01596	0.197992	-0.16179	-0.00379	-0.18343	0.103863
CSH2	NA	NA	0.243714	0.221177	-0.20592	0.043065	0.251921	0.267259
CSHL1	0.207496	0.79256	0.386947	0.11376	-0.10562	-0.11387	-0.0799	0.158413
CSK	0.048191	-0.39375	0.321741	0.693932	0.058139	0.422046	0.231357	0.274255
CSMD1	-0.10897	0.655016	0.195369	0.096786	0.055256	-0.04775	0.432349	0.598809
CSMD2	-0.57637	-0.28305	0.2781	-0.24859	0.069718	-0.00119	-0.37711	-0.41698
CSMD3	0.032877	0.498575	-0.32146	-0.35681	0.114441	-0.00474	0.407165	0.180743

CSN1S1	-0.28021	-0.03158	-0.12591	-0.12747	0.218481	-0.10868	0.123805	0.00926
CSN2	0.149999	0.709657	-0.22132	0.024937	0.09855	0.208205	0.095754	0.050878
CSN3	-0.14633	0.504061	-0.22942	-0.07076	-0.04495	0.221939	0.07448	0.161375
CSNK1A1	-0.18209	-0.57125	0.064922	-0.05485	0.050436	0.147459	0.167	0.141232
CSNK1A1L	0.267341	0.646121	-0.08146	-0.12138	-0.02258	0.123088	-0.19877	0.196063
CSNK1D	-0.1313	-0.11444	0.104034	0.105558	0.246457	0.339415	0.394523	0.346939
CSNK1E	-0.06904	0.044765	0.359203	0.118146	0.640612	0.417376	0.224098	0.000901
CSNK1G1	0.095374	0.296535	-0.09859	-0.16694	-0.10767	0.112114	-0.22717	-0.16964
CSNK1G2	0.227109	0.52426	0.209156	0.255414	0.105893	-0.15685	-0.6224	-0.2861
CSNK1G3	0.350813	-0.41174	0.024783	-0.30187	-0.2541	-0.43684	0.338078	0.298456
CSNK2A1	0.246464	-0.15427	-0.2021	0.097555	-0.00202	0.007401	0.482898	-0.08168
CSNK2A2	0.310155	-0.11815	0.084211	-0.03143	0.427008	0.26682	0.461712	-0.10247
CSNK2B	0.458486	0.239304	0.061766	-0.0675	-0.01721	-0.21432	0.639245	0.21144
CSPG4	-0.33398	0.153125	0.139557	-0.07141	0.106901	0.22082	-0.45264	-0.36339
CSPG5	-0.27011	0.437921	-0.22966	0.157438	-0.03941	0.080243	0.241419	0.043134
CSPP1	0.008453	0.020237	0.143722	-0.18391	-0.05888	0.227036	0.397789	0.125861
CSRNP2	NA	NA	-0.06747	-0.28299	-0.06635	0.052269	NA	NA
CSRNP3	NA	NA	-0.04356	0.063304	-0.40699	-0.04552	NA	NA
CSRP1	-0.26769	-0.08435	0.306842	-0.07701	0.111769	0.081933	-0.19943	-0.23091
CSRP2	-0.43925	-0.25175	-0.10701	0.301204	-0.13564	0.342338	-0.44609	-0.6194
CSRP2BP	0.224086	-0.39197	-0.34578	-0.0376	0.189507	-0.35285	0.524466	0.297798
CSRP3	-0.04412	0.353661	-0.26286	0.098759	-0.04001	0.197129	0.527371	0.232572
CST1	-0.1375	0.032813	0.293981	-0.09715	-0.09863	0.127971	0.229374	-0.18311
CST11	0.171703	0.581371	0.117515	-0.17713	-0.05848	0.336251	-0.04482	0.071367
CST2	-0.00338	0.528678	0.249017	-0.25943	-0.12423	-0.18699	-0.10581	-0.16599
CST3	0.155459	-0.13753	0.031785	-0.08824	0.019302	-0.07999	0.298387	0.079482
CST4	-0.18413	0.1511	NA	NA	NA	NA	-0.0764	-0.14752
CST5	0.103386	-0.01226	0.03287	-0.1541	-0.00332	-0.08786	-0.14736	-0.024
CST6	-0.16992	0.21263	-0.07256	0.35982	-0.06119	-0.0652	0.481152	0.22038
CST7	0.009635	0.388674	0.299294	0.485745	-0.04816	0.084677	0.008643	0.413968
CST8	0.10559	0.096415	-0.26551	-0.19237	-0.02825	0.064606	0.439703	0.395378
CST9	0.183604	0.075629	0.066029	-0.20731	-0.11636	-0.21166	-0.32301	-0.45505
CST9L	0.10317	-0.134	-0.12798	-0.15709	0.006225	0.214295	0.138798	0.291777
CSTA	-0.10325	-0.21737	0.096169	0.285956	-0.22894	-0.02807	-0.26381	-0.02206
CSTB	-0.29473	-0.10992	-0.05122	-0.2092	-0.2722	-0.23189	0.113656	-0.02661
CSTF1	-0.00416	0.688879	-0.13181	-0.08668	0.054174	-0.30044	0.443213	0.219202
CSTF2	0.492624	0.54164	-0.32364	0.214323	0.13429	-0.42486	0.045366	-0.119
CSTF2T	0.505342	-0.0437	-0.25043	0.034488	0.216353	0.087983	-0.15847	0.025764
CSTF3	0.133767	-0.56299	-0.17304	-0.26124	-0.12605	-0.09618	0.345422	0.305559
CSTL1	0.361936	0.725261	-0.31894	-0.10562	-0.0239	0.123043	0.130645	-0.02871
CTAG1B	0.11606	0.319503	NA	NA	NA	NA	0.584111	-0.03558
CTAG2	0.110836	0.359499	0.211839	0.187695	0.14263	-0.05217	0.28944	0.361034
CTAGE1	0.314043	0.364468	-0.17128	0.031742	-0.1222	-0.41047	-0.39475	-0.37756
CTAGE5	-0.38219	0.21596	-0.47591	-0.11588	-0.23089	-0.03859	-0.1038	0.275127
CTBP1	-0.03382	-0.19423	0.136783	0.277196	0.305625	0.047047	0.140215	-0.11592
CTBP2	-0.20037	-0.00829	0.026526	0.123722	0.319909	0.001046	0.350626	0.083254
CTBS	-0.25188	-0.29775	0.163586	-0.28122	-0.01746	-0.31471	-0.57061	-0.4701
CTCF	0.067025	-0.65974	-0.57138	0.397004	0.156782	0.209164	0.713872	0.344301
CTCFL	0.354021	0.343888	0.153392	-0.1135	0.151301	0.063465	-0.1364	0.250437
CTDP1	-0.0923	0.267424	0.199314	0.036742	0.130744	0.08311	-0.22	-0.17611
CTDSP1	0.148832	-0.20738	0.147765	0.155356	-0.08618	-0.08584	-0.15378	-0.34236
CTDSP2	-0.01553	-0.4922	-0.01142	-0.00609	0.305064	0.215102	-0.27005	-0.03175

CTDSPL	-0.09894	-0.03978	0.141361	-0.19678	-0.00172	-0.11555	0.344262	0.195431
CTDSPL2	-0.14864	-0.60236	-0.31425	-0.31585	-0.10448	-0.33092	-0.0295	-0.00294
CTF1	-0.11686	0.41358	0.100388	-0.27747	-0.1938	0.014622	-0.33734	-0.35044
CTF8	0.417469	0.682605	0.34057	0.240151	0.174179	-0.02946	0.124441	0.158033
CTGF	-0.09847	-0.36524	0.022748	-0.0916	-0.03635	-0.02132	-0.32676	-0.17399
CTH	-0.18973	-0.13436	0.20132	0.124504	-0.12323	0.082699	0.154415	0.311883
CTHRC1	-0.24443	-0.31209	-0.01495	0.11071	-0.16446	-0.15766	-0.24675	-0.18953
CTLA4	-0.09226	0.468618	0.102738	0.492949	-0.30695	0.443949	-0.3097	0.042535
CTNNA1	-0.19616	-0.4074	0.332697	0.062198	0.226457	0.033628	0.389027	0.253584
CTNNA2	0.089053	-0.04896	0.256463	0.075654	-0.00086	-0.08894	0.561881	-0.0729
CTNNA3	0.088919	0.181928	0.677168	0.140743	-0.06826	0.130854	-0.38584	-0.14789
CTNNAL1	-0.2625	-0.47398	-0.30879	-0.11565	0.207136	-0.13993	-0.05254	-0.09668
CTNNB1	-0.00654	-0.47739	-0.04442	0.116348	0.389259	-0.15949	0.32838	0.047202
CTNNBIP1	-0.67168	0.314662	-0.03434	0.139554	0.110426	-0.18549	0.352681	0.290265
CTNNBL1	0.388945	-0.28101	0.254047	0.16589	0.185632	-0.0756	0.365115	-0.04242
CTNND1	-0.04699	-0.27911	-0.31252	0.002146	-0.00527	-0.12253	-0.11974	-0.08987
CTNND2	-0.31247	-0.07839	0.029468	0.147054	-0.0519	0.267222	-0.42562	-0.0153
CTNS	0.076394	0.443922	0.128748	0.125435	-0.24982	-0.17279	-0.48047	-0.17259
CTPS	-0.10103	-0.13579	-0.12805	0.206552	0.303303	0.0888	-0.05128	0.150786
CTPS2	0.329368	0.312405	-0.08739	-0.03257	0.209406	-0.09825	0.444346	0.374316
CTR9	-0.1462	-0.62062	-0.10932	0.209389	0.04905	0.411728	-0.31373	-0.25389
CTRB1	NA	NA	-0.06192	-0.19431	0.114248	0.13717	0.018701	-0.04438
CTRB2	0.291091	0.173429	0.104971	0.029741	-0.36678	0.218192	-0.49039	-0.23296
CTRC	-0.3497	-0.07172	0.233384	-0.28435	-0.07608	0.163989	-0.01716	-0.19507
CTRL	0.259348	0.480396	0.105989	-0.11156	0.113566	0.274611	-0.11064	0.243834
CTSA	0.230994	0.04091	0.36846	-0.34302	0.398752	-0.02515	NA	NA
CTSB	-0.38425	-0.40603	0.534582	0.044424	0.044441	0.131392	-0.34156	0.078345
CTSC	-0.21963	-0.32138	-0.02252	0.195293	-0.4248	0.220178	-0.24376	-0.01975
CTSD	-0.3008	0.458174	0.37304	0.080047	-0.00247	0.009614	0.092362	0.28017
CTSE	-0.66026	0.083302	0.010246	0.112315	-0.11119	-0.32688	-0.19651	-0.26136
CTSF	0.309188	0.459569	-0.06217	-0.00679	-0.09812	0.335944	-0.24242	-0.22763
CTSG	-0.19717	0.128971	0.117919	0.152945	-0.08811	-0.03835	-0.24418	-0.02967
CTSH	0.216871	0.104878	0.206161	0.48412	0.09916	0.529331	-0.21608	0.10552
CTSK	-0.43226	-0.60284	0.017497	0.026068	-0.03358	0.429061	-0.11859	-0.35636
CTSL1	-0.37327	-0.19325	-0.02223	0.005344	-0.20274	0.405122	-0.41115	-0.16755
CTSL2	0.145124	0.148896	-0.16109	-0.34298	0.066766	-0.29615	-0.07923	-0.48592
CTSO	-0.156	-0.5221	0.015603	-0.25043	-0.39212	-0.05658	-0.59952	-0.1703
CTSS	0.134373	0.050954	-0.01452	-0.07203	-0.33062	0.058505	-0.38872	-0.29229
CTSW	0.03451	0.518185	0.460943	0.167847	-0.14626	0.105613	-0.40108	0.018683
CTSZ	0.015335	-0.14415	0.231446	0.01565	-0.01519	0.158124	0.022838	0.118152
CTTN	-0.18837	-0.34978	-0.17611	0.229629	0.28799	-0.07652	0.396147	0.371924
CTTNBP2	0.340838	-0.18745	0.377578	-0.26619	0.0737	-0.09992	0.247084	0.186697
CTTNBP2NI	-0.12622	-0.24021	0.026982	-0.33656	0.143486	-0.2449	-0.2964	-0.34211
CTXN1	-0.08896	0.051125	-0.04066	-0.21667	-0.02042	-0.05375	-0.30184	-0.48178
CTXN3	NA	NA	-0.28654	-0.18128	-0.1377	0.346905	-0.25709	-0.13628
CUBN	0.156938	-0.37609	0.024019	-0.04515	-0.16004	-0.17268	-0.3365	0.531792
CUEDC1	-0.11619	0.085954	0.318028	-0.02626	0.214057	-0.14477	-0.04278	-0.39179
CUEDC2	0.118787	-0.05481	0.259294	0.182626	0.141056	0.220357	-0.20974	-0.24322
CUGBP1	0.100225	0.538327	-0.24733	-0.10903	-0.05507	0.2093	0.008428	0.135517
CUGBP2	-0.36201	-0.14611	-0.09991	0.050997	0.129038	0.493722	-0.2946	-0.19371
CUL1	-0.04909	-0.67458	-0.12738	-0.0447	-0.00151	0.22606	-0.54357	-0.41972
CUL2	0.045214	-0.09146	-0.25971	-0.23226	-0.13681	-0.27304	-0.10188	0.005172

CUL3	0.213309	-0.33209	-0.08154	-0.16344	0.243335	0.152554	-0.05142	-0.29549
CUL4A	0.230105	-0.57789	-0.03771	0.144965	0.390708	0.243914	0.196377	-0.06597
CUL4B	0.092942	-0.36118	0.055633	-0.15093	0.157698	-0.13825	0.109858	-0.0367
CUL5	-0.35477	-0.69894	-0.17713	-0.19123	-0.18316	-0.04925	-0.25564	-0.10519
CUL7	0.353028	0.600179	0.172614	0.269312	0.196201	0.319933	0.168186	0.081962
CUL9	NA	NA	0.044066	0.21998	0.283298	0.353498	NA	NA
CUTA	0.062486	0.101086	0.092151	0.065265	0.13837	-0.26347	0.431906	-0.09281
CUTC	-0.20913	-0.49162	-0.01614	0.268528	0.186166	0.196745	-0.23693	0.039167
CUX1	NA	NA	0.140218	-0.12655	0.198651	0.390293	NA	NA
CUX2	NA	NA	0.292233	-0.01159	0.145043	0.214872	NA	NA
CUZD1	0.182423	0.249485	-0.28905	-0.13474	0.079452	0.021713	0.318458	0.3115
CWC15	NA	NA	0.028032	-0.21886	-0.1458	-0.06762	NA	NA
CWC22	NA	NA	-0.42238	0.015822	-0.20311	0.084701	NA	NA
CWF19L1	0.123179	-0.53928	-0.10523	0.19961	0.077077	0.131028	-0.19322	-0.06442
CWF19L2	0.266847	-0.21172	-0.39968	-0.26841	-0.30971	-0.11835	0.217989	0.284844
CX3CL1	0.223582	-0.0102	0.110476	0.216596	-0.31026	0.508033	-0.38921	-0.14626
CX3CR1	-0.27779	-0.04489	-0.18501	0.287291	-0.05573	0.253931	-0.5604	0.047296
CXADR	0.304675	-0.09204	-0.04976	-0.07495	0.021879	-0.12462	0.301194	0.071242
CXCL1	0.021204	0.165386	0.049018	0.268292	0.117266	-0.03886	-0.4836	-0.062
CXCL10	-0.03062	-0.04034	-0.05325	-0.30508	-0.46605	0.303756	-0.27921	0.201434
CXCL11	0.236718	0.0681	0.031346	-0.26395	-0.38722	0.204507	-0.26273	-0.34651
CXCL12	-0.34443	-0.48698	0.109731	0.022809	-0.12534	0.402969	-0.12946	-0.00357
CXCL13	0.008131	0.329094	0.178176	0.617133	-0.27551	0.534328	-0.3018	-0.16154
CXCL14	0.267893	0.019455	0.368595	0.342738	0.156363	0.492822	0.210422	0.355066
CXCL16	-0.19191	0.192403	-0.15163	0.162185	-0.19024	-0.01414	-0.27552	0.260394
CXCL17	NA	NA	-0.48039	-0.17443	-0.30132	0.158602	NA	NA
CXCL2	-0.0962	-0.2629	0.020968	0.279941	0.028798	-0.08618	-0.43695	0.061646
CXCL3	0.059644	0.739715	-0.00311	0.320159	0.02572	-0.01723	-0.54541	-0.16405
CXCL5	-0.04171	-0.65537	0.122022	0.16242	0.13142	0.239793	-0.29814	-0.36344
CXCL6	-0.22463	-0.15449	0.062985	0.089859	-0.00471	-0.30247	-0.17024	0.597839
CXCL9	-0.05028	-0.07569	0.012185	-0.19017	-0.34313	0.276599	-0.1758	-0.12691
CXCR3	0.367659	0.462368	0.229084	0.403427	-0.06332	0.25956	-0.54311	-0.1341
CXCR4	-0.7304	-0.09106	0.238553	0.74303	-0.08076	0.564758	-0.00326	0.150564
CXCR5	NA	NA	0.012632	0.801608	-0.09046	0.586625	NA	NA
CXCR6	0.001358	-0.06361	0.191443	0.210373	-0.29838	0.070519	-0.26817	0.292082
CXCR7	0.007076	-0.39713	0.084083	-0.04115	-0.17148	0.021651	-0.12831	0.329968
CXorf1	NA	NA	-0.05554	-0.08669	-0.04775	-0.19655	0.4589	0.207205
CXorf21	NA	NA	0.025024	0.261318	-0.31006	0.267618	-0.38438	0.032577
CXorf22	NA	NA	-0.27347	-0.11519	-0.43948	-0.16979	0.168276	-0.14602
CXorf23	NA	NA	0.062272	-0.31381	0.178683	-0.48415	0.085751	0.108224
CXorf26	NA	NA	-0.08272	-0.14403	-0.10496	0.084426	-0.43723	-0.224
CXorf27	NA	NA	-0.26339	-0.07966	-0.04027	-0.27572	NA	NA
CXorf36	NA	NA	-0.02221	0.096541	-0.30577	-0.24361	-0.06595	0.1015
CXorf38	NA	NA	0.130483	-0.08213	0.034943	0.040855	0.0125	0.409803
CXorf40A	NA	NA	NA	NA	NA	NA	0.430977	0.406975
CXorf40B	NA	NA	-0.04653	0.055733	0.157582	-0.12161	0.180411	-0.03179
CXorf41	NA	NA	-0.05518	-0.09918	-0.25932	0.075202	0.608388	0.238519
CXorf48	NA	NA	-0.06155	0.140655	0.100968	-0.13798	-0.25053	-0.04249
CXorf56	NA	NA	-0.31477	-0.46866	-0.07177	-0.32403	-0.20954	-0.56315
CXorf57	NA	NA	0.071219	-0.02838	0.215519	-0.00475	NA	NA
CXorf58	NA	NA	-0.29136	0.060721	-0.10112	-0.4246	NA	NA
CXorf59	NA	NA	-0.26292	-0.05597	-0.03934	0.178771	NA	NA

CXorf61	NA	NA	0.142856	-0.14976	-0.22791	0.12548	NA	NA
CXXC1	-0.10293	0.647292	0.338519	0.164035	0.197613	0.220792	-0.12771	0.07168
CXXC4	0.048359	0.19658	0.122634	-0.16381	0.104204	-0.03655	-0.13184	-0.00422
CXXC5	0.160486	-0.35526	0.590956	0.408866	0.256793	0.187607	0.462314	0.068141
CYB561	-0.1422	0.050553	0.458861	0.41374	0.222575	-0.05017	0.399143	0.191638
CYB561D1	0.265583	0.623007	-0.08444	-0.05097	-0.17279	0.011747	NA	NA
CYB561D2	0.16572	0.194941	-0.07092	0.186387	0.140348	-0.49842	-0.03882	0.281308
CYB5A	0.190931	-0.2642	0.137968	-0.20005	-0.02012	-0.44788	0.098831	0.336541
CYB5B	0.216795	-0.00509	0.257785	0.002862	0.240385	-0.19494	NA	NA
CYB5D1	-0.1682	-0.08855	-0.18284	0.205659	-0.25309	-0.2848	0.50936	0.314093
CYB5D2	-0.41851	-0.24998	0.125851	-0.10229	-0.26797	-0.36132	-0.03285	0.118051
CYB5R1	-0.28591	0.360849	0.086276	0.254799	-0.22897	0.030801	-0.29056	-0.41463
CYB5R2	-0.38915	0.361345	-0.30651	0.289144	0.01927	-0.30511	-0.24409	-0.32434
CYB5R3	-0.24895	-0.10915	0.061071	0.011136	-0.06158	0.149199	-0.24726	-0.01821
CYB5R4	0.095766	-0.497	-0.17963	-0.08893	0.005495	-0.15421	0.293492	0.039003
CYB5RL	NA	NA	0.072835	0.122971	0.222327	0.174279	NA	NA
CYBA	0.148363	-0.13092	0.059401	0.23812	-0.03337	0.028638	0.069229	-0.06717
CYBASC3	0.053669	0.328539	0.391223	0.404781	0.318946	0.438819	0.141394	0.050555
CYBB	-0.38292	-0.19533	0.278439	-0.08401	-0.44237	0.321565	-0.55662	0.044786
CYBRD1	-0.01442	-0.56666	0.304791	-0.0994	-0.34778	-0.20066	-0.05932	0.365552
CYC1	0.278637	0.605228	0.265058	-0.01675	-0.21322	0.002345	0.364433	0.056994
CYCS	0.209282	-0.42389	-0.15857	-0.22684	-0.09129	0.090946	-0.03244	-0.14923
CYFIP1	0.176471	-0.35134	-0.09994	-0.07722	-0.03236	0.036786	0.448825	0.2924
CYFIP2	0.289905	0.375875	0.488523	0.780563	0.10285	0.662662	-0.25223	0.154561
CYGB	0.103072	0.606524	0.141431	-0.11735	-0.04501	0.048069	-0.05206	0.137991
CYHR1	-0.09746	0.535668	0.318704	-0.00427	0.112248	-0.10143	0.375255	0.315073
CYLC1	NA	NA	0.119915	0.086141	0.049134	-0.12053	0.056894	0.117152
CYLC2	-0.01829	0.542888	0.238567	0.182116	-0.27569	-0.1356	-0.21351	0.018122
CYLD	-0.04199	0.521668	0.236559	0.004095	-0.27058	0.347285	-0.10934	-0.23706
CYorf15A	NA	NA	0.07305	0.019425	-0.21983	-0.22561	0.286606	-0.09747
CYorf15B	NA	NA	0.108033	0.016816	-0.16877	-0.1653	0.301169	-0.128
CYP11A1	-0.2213	0.563527	0.327212	0.0043	-0.18519	-0.0362	0.055711	0.484741
CYP11B1	0.150564	0.448116	0.167482	-0.06285	-0.02114	-0.12775	0.255287	-0.02147
CYP11B2	0.345201	0.232445	0.204825	-0.05143	0.155728	0.015015	0.130688	0.212787
CYP17A1	0.023133	0.084484	0.328475	0.195327	0.098616	0.027409	-0.04125	-0.1234
CYP19A1	0.333081	0.696893	0.085082	0.135085	-0.03877	0.241109	0.039741	-0.04881
CYP1A1	-0.02996	-0.2293	0.064657	-0.3098	0.268866	0.272163	-0.17502	0.15482
CYP1A2	0.451214	0.197168	0.219707	-0.28489	0.211329	0.331862	0.304887	0.244922
CYP1B1	-0.1181	-0.4067	0.19002	-0.38963	0.112267	-0.19529	-0.27451	0.323307
CYP20A1	0.367123	-0.45025	0.119365	-0.02493	0.119414	0.138752	0.460638	0.360575
CYP21A2	-0.40534	0.298206	-0.18504	-0.13701	0.160907	0.158919	-0.14118	0.140194
CYP24A1	0.170953	0.298405	0.31447	-0.49236	0.099108	0.00293	-0.06174	-0.20096
CYP26A1	0.178151	0.678936	0.303308	0.125135	-0.06571	0.018838	-0.39653	-0.17474
CYP26B1	0.012719	-0.05888	-0.07487	0.196746	-0.06002	-0.1728	-0.00733	0.058864
CYP26C1	0.224783	0.458546	NA	NA	NA	NA	-0.00632	0.3778
CYP27A1	0.289222	-0.17805	0.519919	-0.15796	0.192373	0.049691	0.006689	0.29582
CYP27B1	0.048203	0.212295	-0.14518	0.341737	-0.06344	-0.23935	0.081253	0.102561
CYP2A13	0.183995	0.161254	0.047859	-0.24901	-0.03462	-0.32864	0.180971	0.09646
CYP2A6	0.220429	0.042942	-0.31023	-0.29847	-0.09927	-0.18644	0.410461	0.02837
CYP2A7	-0.30762	-0.01088	0.043431	-0.44781	-0.16597	-0.10415	0.508111	0.23888
CYP2B6	0.250681	0.003612	-0.00141	-0.23041	0.055356	-0.28175	0.298784	0.335213
CYP2B7P1	NA	NA	0.14167	-0.27607	-0.20718	-0.4561	0.24427	0.068741

CYP2C18	-0.07924	0.047621	-0.20535	-0.29864	-0.13719	-0.28076	0.064426	0.425961
CYP2C19	0.04046	-0.0305	0.101583	-0.36074	0.128384	-0.2053	0.292567	0.574518
CYP2C8	-0.1133	-0.19028	-0.24173	-0.05917	-0.03707	-0.26219	0.229475	0.468299
CYP2C9	0.084867	0.038053	0.03054	-0.2385	0.007102	-0.19989	0.096189	0.427991
CYP2D6	0.06924	0.398158	0.370273	-0.1947	0.323866	0.046864	0.526502	0.572768
CYP2E1	-0.24139	-0.09075	-0.08582	0.140341	-0.14058	0.324974	-0.17539	0.169588
CYP2F1	0.007067	0.498577	0.086964	-0.28541	0.231756	-0.01864	0.381947	-0.24667
CYP2J2	0.252277	0.019434	0.143874	0.072925	-0.30044	-0.33111	0.391473	0.327525
CYP2R1	-0.08162	0.176779	0.00993	-0.27289	0.16158	-0.16642	0.361343	0.519415
CYP2S1	0.219596	0.024693	0.173264	-0.01675	0.207276	0.175733	0.350866	0.33068
CYP2U1	0.250769	-0.47571	-0.02366	0.233009	-0.31162	0.048575	-0.53057	0.017913
CYP2W1	0.077182	0.056137	-0.02035	0.150252	-0.11857	-0.24501	0.287836	-0.2962
CYP39A1	0.313748	-0.51626	-0.0094	-0.05599	0.025098	-0.36522	0.38216	-0.1729
CYP3A4	8.59E-05	-0.12858	-0.0403	-0.30194	0.077014	-0.06946	0.388115	0.468835
CYP3A43	0.083757	0.48031	0.101966	-0.3945	-0.02167	0.018867	0.038691	0.264307
CYP3A5	-0.12898	0.084484	-0.17023	-0.20518	0.194521	-0.15828	0.083602	0.246662
CYP3A7	0.03534	-0.36068	-0.16427	-0.27207	0.15789	0.015311	-0.26288	0.333216
CYP46A1	-0.1318	-0.40099	0.085603	-0.26455	0.044453	-0.2689	-0.51215	-0.20026
CYP4A11	-0.00461	0.267018	0.031024	-0.21464	-0.14347	-0.06865	-0.20847	0.30673
CYP4A22	-0.40804	-0.01001	NA	NA	NA	NA	0.0242	0.340295
CYP4B1	0.09976	-0.0389	0.294816	0.232614	-0.17479	-0.5112	0.297285	0.062285
CYP4F11	0.554947	0.402694	-0.05286	0.030919	0.195159	0.437469	-0.05866	0.443231
CYP4F12	0.460172	0.142196	0.024622	0.117163	0.228748	0.015569	0.101708	0.367838
CYP4F2	0.303313	0.017102	0.13778	-0.1632	0.295064	-0.06184	-0.05429	0.456678
CYP4F22	NA	NA	0.142322	0.019567	-0.23836	0.183864	NA	NA
CYP4F3	0.412721	-0.1818	-0.12738	0.070741	0.172773	-0.47393	0.412699	0.598554
CYP4F8	0.002405	0.038155	0.069774	-0.01075	0.253302	-0.06299	0.075129	0.331451
CYP4V2	0.103948	-0.0045	-0.14451	-0.36441	0.02227	-0.11826	0.177103	0.348031
CYP4X1	0.000407	0.704509	0.252254	-0.16505	0.006767	-0.05649	0.150148	0.169049
CYP4Z1	-0.16728	0.104376	0.202519	0.149357	-0.08147	0.113844	0.423725	0.35431
CYP4Z2P	NA	NA	-0.05041	-0.02573	0.029822	-0.0237	0.123312	-0.16352
CYP51A1	0.180097	-0.12122	-0.20955	0.078769	0.031834	-0.06097	-0.24833	-0.33506
CYP7A1	-0.06582	0.36757	-0.23144	-0.26197	0.203418	-0.21722	-0.28459	-0.14751
CYP7B1	-0.08337	-0.22721	0.24959	0.285856	-0.03996	0.506824	-0.57277	-0.17411
CYP8B1	-0.05374	0.201712	0.29683	0.091762	0.141016	-0.29541	-0.21267	-0.26766
CYR61	-0.27956	-0.28766	0.005052	0.039733	-0.14337	0.064737	-0.03291	0.002625
CYS1	NA	NA	NA	NA	NA	NA	0.068213	-0.16171
CYSLTR1	-0.23278	-0.20604	0.175934	0.10901	-0.38209	0.113924	-0.42768	0.29195
CYSLTR2	0.460059	0.360951	-0.01453	0.075083	0.187211	0.218348	-0.51541	-0.43322
CYTH1	NA	NA	0.064899	0.174051	-0.04518	0.257959	NA	NA
CYTH2	NA	NA	0.056007	-0.01557	0.067887	0.13597	NA	NA
CYTH3	NA	NA	-0.03348	-0.21621	-0.00589	-0.14702	NA	NA
CYTH4	NA	NA	0.14584	0.56592	-0.04217	0.611813	NA	NA
CYTIP	NA	NA	0.153823	0.64132	-0.17484	0.311088	NA	NA
CYTL1	-0.32092	-0.25283	0.034641	-0.4257	-0.08084	0.062435	-0.25207	-0.01951
CYYR1	-0.45648	-0.44655	-0.01892	0.225212	-0.1568	0.053191	0.008967	0.395165
D2HGDH	NA	NA	0.077242	0.037192	0.318899	0.221815	-0.01882	0.332738
D4S234E	0.268729	-0.03675	-0.00477	-0.19651	0.13092	-0.08253	0.088444	0.277153
DAAM1	-0.19776	-0.45833	-0.3976	-0.15524	-0.04275	0.045319	-0.56539	-0.65713
DAAM2	0.038882	-0.01662	0.114948	-0.05274	-0.05418	0.551369	-0.28772	-0.0311
DAB1	0.093591	0.194441	0.120123	0.056141	-0.19168	-0.31209	0.307211	-0.04333
DAB2	-0.01542	9.42E-05	0.168452	0.002762	-0.04925	0.21776	-0.24796	-0.02935

DAB2IP	-0.04464	0.200173	0.219891	-0.18259	0.271469	0.076917	-0.10838	-0.34418
DACH1	-0.1097	-0.69146	0.454141	0.192439	0.31888	-0.06993	0.50846	0.141615
DACH2	-0.19589	0.147132	-0.04696	-0.32485	0.004037	-0.19959	NA	NA
DACT1	-0.3628	-0.44833	0.164126	-0.07561	-0.04556	0.360066	0.250153	0.146488
DACT2	0.140519	-0.0567	-0.29113	0.268441	0.097244	0.17376	0.296972	-0.02083
DACT3	-0.1154	-0.1412	-0.03839	-0.05826	-0.07431	-0.06062	NA	NA
DAD1	-0.10037	-0.63269	0.005841	-0.17506	-0.2416	-0.04575	0.023553	0.269468
DAG1	-0.1807	-0.09086	-0.05423	0.110587	0.425098	0.226039	0.009766	-0.31358
DAGLA	0.142417	-0.3285	-0.38514	0.016041	-0.00322	-0.18721	NA	NA
DAGLB	0.030489	0.3561	0.251142	-0.15411	0.395796	0.042321	NA	NA
DAK	0.277891	0.580502	0.401344	0.12459	0.373136	0.006075	0.451051	0.629852
DALRD3	0.255095	0.293598	0.144496	0.082363	0.086459	0.078318	0.319098	0.124676
DAND5	NA	NA	-0.29622	-0.29885	-0.11411	0.016351	-0.32079	-0.16454
DAO	0.456456	0.472345	-0.09023	-0.4297	-0.22084	-0.61192	0.03297	-0.03487
DAOA	0.515785	0.254951	-0.11175	-0.05065	-0.16279	-0.08808	0.448156	0.166951
DAP	-0.43499	0.017422	0.32268	0.071358	0.294003	-0.09928	0.462127	0.235855
DAP3	0.342382	-0.23272	-0.2197	0.023805	-0.08845	-0.12321	0.229655	-0.22635
DAPK1	-0.64345	-0.47813	-0.13626	-0.21794	-0.1844	0.185982	-0.53395	-0.06733
DAPK2	0.19396	0.346617	0.349856	0.182089	0.413795	-0.02912	0.164115	0.369418
DAPK3	-0.20529	0.077573	0.250013	-0.20345	0.306734	0.043252	-0.29213	-0.33667
DAPL1	NA	NA	0.072732	-0.33333	0.044875	-0.42287	NA	NA
DAPP1	-0.26821	0.004991	-0.3368	0.472954	-0.33512	0.519149	-0.58312	-0.54616
DARC	-0.3044	-0.27375	0.218374	0.325132	-0.13307	0.120031	-0.23178	-0.09852
DARS	0.405877	0.35526	0.115467	0.0828	0.331143	0.120043	0.401519	-0.16513
DARS2	0.127474	0.489154	-0.38927	0.107071	-0.17436	-0.09721	0.190395	-0.05225
DAXX	0.359547	0.314252	0.052357	0.324778	0.269935	0.205588	0.242612	0.164669
DAZ2	0.027724	0.555668	0.097461	0.140517	-0.09857	0.090217	0.462039	0.389504
DAZAP1	-0.16513	0.056888	-0.35098	0.194164	0.340817	0.16061	-0.24132	-0.02998
DAZAP2	-0.07177	-0.53697	-0.1709	-0.31043	0.092202	-0.14986	0.118179	0.471439
DAZL	0.149648	-0.13637	0.100525	0.709016	-0.21005	0.290626	-0.55056	-0.11509
DBC1	-0.09117	-0.56	0.055657	-0.09505	-0.08491	-0.07987	-0.21893	0.515956
DBF4	0.129057	-0.12022	-0.43243	0.111026	0.025016	-0.33206	0.34567	0.562401
DBF4B	0.423327	0.531348	0.31245	-0.12777	0.466838	0.170744	0.270953	0.451526
DBH	0.379082	0.664989	0.085232	-0.06047	0.135503	0.214791	-0.18067	-0.03033
DBI	0.351072	-0.30736	-0.05374	-0.09623	-0.01019	-0.11969	0.215018	-0.07229
DBN1	-0.21473	-0.02122	-0.08457	0.027113	0.009192	0.101468	-0.13113	-0.39294
DBNDD1	0.325217	0.267837	0.261449	-0.04909	0.390395	-0.39711	-0.02666	-0.53817
DBNDD2	NA	NA	0.27834	-0.22206	0.392353	0.100392	-0.28491	-0.39166
DBNL	0.134295	0.544564	0.315523	0.321006	0.261559	0.038683	-0.3536	-0.12692
DBP	0.157528	0.139038	0.360906	-0.2749	0.036925	0.015486	-0.0986	-0.12806
DBR1	-0.54575	-0.1061	-0.2037	0.022461	0.092239	0.136378	-0.04536	0.051851
DBT	-0.3888	0.266024	0.011267	-0.38511	-0.07394	-0.27846	-0.26075	-0.46978
DBX1	0.113097	0.55271	NA	NA	NA	NA	-0.62937	-0.09628
DBX2	0.184345	0.39383	0.106511	-0.11145	-0.21399	0.158164	-0.13071	-0.14257
DCAKD	0.04772	0.453919	0.219805	0.214004	0.108613	-0.16041	-0.30009	-0.37859
DCBLD1	-0.20383	0.493715	-0.06497	0.213621	-0.2494	-0.13883	-0.54284	-0.22796
DCBLD2	0.041834	-0.25943	-0.22734	-0.30571	-0.01809	-0.06264	-0.04261	-0.33759
DCC	-0.15637	-0.18071	-0.00929	-0.05377	0.109359	-0.03146	0.043615	-0.17444
DCD	-0.04923	0.173023	0.278894	-0.05624	-0.16324	-0.11132	0.211103	0.030638
DCDC1	0.120622	0.413021	-0.34381	-0.12709	-0.00924	-0.26558	-0.00211	0.091605
DCDC2	-0.05824	0.074301	-0.1813	0.136564	-0.33399	-0.31147	0.174467	-0.16786
DCHS1	0.220151	0.178316	0.233683	0.010052	0.07724	0.332284	-0.62231	-0.5657

DCHS2	0.198344	0.391769	0.08594	-0.13624	-0.34406	-0.40754	0.277139	-0.43891
DCI	-0.23823	0.450989	0.061126	-0.02965	0.06793	-0.25062	0.410347	-0.02694
DCK	-0.37536	-0.16779	-0.27037	0.283757	-0.09644	-0.10501	0.208197	0.121327
DCLK1	NA	NA	-0.04144	0.010924	-0.18531	0.01431	NA	NA
DCLK2	NA	NA	0.023562	-0.03953	-0.20704	0.237953	NA	NA
DCLK3	NA	NA	-0.38951	-0.21879	-0.06188	0.074245	NA	NA
DCLRE1A	0.16168	0.44083	-0.17033	-0.22079	0.107461	-0.22525	0.165048	0.003751
DCLRE1B	-0.18064	-0.22335	0.236278	0.177682	0.309453	-0.06619	0.455703	-0.08408
DCLRE1C	0.51671	0.675773	-0.22073	0.156654	0.013874	0.092672	-0.61185	-0.59432
DCN	-0.36605	-0.50374	0.047079	-0.09823	-0.11895	0.245599	-0.22506	-0.04914
DCP1A	0.105218	-0.25552	-0.24624	-0.15181	0.21293	0.098234	0.29485	-0.17674
DCP1B	0.556713	0.137807	-0.1072	0.054258	0.08163	-0.18301	0.131326	0.140758
DCP2	-0.05165	-0.28663	0.110351	-0.12828	-0.12665	-0.01085	-0.12427	0.005236
DCPS	-0.06768	0.342706	-0.2535	0.370048	-0.23335	-0.31149	0.33075	-0.259
DCST1	-0.14001	-0.07786	0.031876	-0.13984	-0.17414	-0.00298	0.009177	0.011201
DCST2	-0.15438	0.103406	0.185258	-0.11287	0.15566	-0.00027	0.202968	0.436999
DCT	-0.02377	-0.14431	0.160409	-0.20042	0.100817	0.021717	0.088035	-0.13141
DCTD	0.030593	-0.0993	-0.13611	0.349113	0.148779	-0.08192	0.480431	0.198687
DCTN1	0.227781	0.419451	0.375867	0.05471	0.207233	-0.07527	-0.19052	-0.49417
DCTN2	0.019523	0.430445	-0.37469	-0.06457	0.134767	-0.14568	0.000832	-0.10321
DCTN3	-0.05877	-0.32452	-0.02098	0.121173	-0.11688	-0.34668	0.25532	0.130381
DCTN4	0.295348	-0.11751	0.228512	-0.35442	0.049312	0.178956	-0.4599	-0.36879
DCTN5	0.116926	-0.24748	-0.01969	-0.092	0.133635	-0.17609	0.516328	0.121891
DCTN6	-0.10123	-0.47903	0.190396	-0.44457	-0.31929	-0.25334	-0.50007	-0.42185
DCTPP1	NA	NA	-0.10733	0.198498	0.300649	-0.15046	NA	NA
DCUN1D1	-0.15381	-0.57514	-0.37359	-0.17889	-0.2379	-0.52514	-0.09923	-0.11673
DCUN1D2	0.010179	-0.21418	-0.07637	-0.17958	0.33176	-0.0126	0.450097	0.440436
DCUN1D3	-0.43208	-0.03017	0.184568	-0.37484	-0.04458	-0.26113	-0.09884	-0.03651
DCUN1D4	-0.17633	-0.47583	-0.22273	-0.38188	-0.13201	-0.14892	-0.43272	-0.24036
DCUN1D5	0.095358	-0.56801	-0.15114	-0.08802	-0.09733	-0.07378	-0.04283	-0.12426
DCX	0.203657	-0.15814	-0.0219	-0.06484	0.004672	-0.11031	0.581516	0.311985
DCXR	-0.01082	0.631219	0.207573	0.24714	0.120736	-0.13219	0.751527	-0.02804
DDA1	NA	NA	-0.21832	-0.13444	-0.07713	-0.07853	-0.30932	0.021282
DDAH1	-0.16903	-0.13311	0.232527	-0.05292	0.385127	0.090306	0.367428	-0.14114
DDAH2	0.028455	-0.29133	-0.34986	-0.17064	0.037211	-0.52108	-0.2266	-0.43635
DDB1	-0.37033	0.238725	-0.0019	0.314668	-0.09653	0.270762	0.149931	-0.01697
DDB2	-0.44428	-0.38472	-0.03056	0.163838	-0.29078	-0.25116	-0.62531	-0.34267
DDC	0.603094	0.120051	0.12217	0.118579	0.255296	-0.15193	0.305105	0.43919
DDHD1	0.038732	-0.04582	0.188011	0.012947	-0.01521	0.521448	-0.13943	-0.02429
DDHD2	-0.31147	0.298983	0.086853	-0.04033	-0.0753	-0.14962	-0.09549	0.008444
DDI1	0.471379	0.354065	0.111244	-0.13037	0.001068	-0.11814	-0.09095	-0.16191
DDI2	0.112769	0.015106	-0.2348	0.16238	0.304236	-0.02714	0.129248	0.336133
DDIT3	-0.42803	-0.17514	-0.05374	-0.11596	-0.00651	-0.12096	-0.05444	0.125333
DDIT4	-0.32452	-0.1097	-0.19369	0.46353	0.233974	0.035375	-0.28988	0.021984
DDIT4L	0.120583	0.048441	0.095609	-0.18038	-0.07684	0.175932	0.123882	0.056694
DDN	0.416485	0.214721	0.182064	-0.32305	0.193677	-0.21174	0.227964	-0.21807
DDO	0.236926	-0.33665	-0.03241	0.015822	0.131383	-0.17468	0.215986	0.57157
DDOST	-0.39926	0.119734	0.379884	0.284769	0.364352	0.127853	-0.30366	-0.41005
DDR1	0.206501	0.187409	0.11152	0.2656	0.441203	-0.02062	0.502217	0.101625
DDR2	-0.17192	-0.04163	0.093285	-0.09468	-0.07723	-0.08369	-0.29323	-0.30422
DDRGK1	NA	NA	-0.06371	0.364716	0.041326	-0.06302	NA	NA
DDT	0.003498	-0.04448	0.141941	-0.01556	0.258054	-0.00058	0.24475	0.20252

DDX1	0.293767	-0.39607	-0.1564	-0.13765	0.207385	0.310959	0.63676	0.487234
DDX10	0.433571	0.50358	-0.12909	0.122991	0.13394	0.338346	0.403287	0.312906
DDX11	0.586822	0.635302	0.005942	0.405107	0.342951	0.218685	-0.00188	0.195714
DDX17	-0.47898	-0.17773	0.174686	0.008027	0.164383	0.332739	-0.25856	-0.17665
DDX18	0.196321	-0.19887	-0.30635	0.137978	-0.25201	-0.21575	0.707837	0.410031
DDX19A	-0.02493	-0.41669	-0.43725	0.339639	0.206118	-0.11908	-0.05849	-0.09485
DDX19B	0.009348	-0.51495	0.012891	0.150035	0.309737	-0.05274	0.322634	0.134439
DDX20	-0.09127	-0.01887	0.106806	0.093519	0.297829	0.256521	0.136412	0.232648
DDX21	0.151136	-0.48097	-0.05464	0.059202	0.318694	0.394412	0.478841	0.409616
DDX23	-0.07173	-0.45556	-0.08668	0.363908	0.200282	0.477305	0.351803	0.431899
DDX24	0.157162	-0.08152	-0.25735	0.207139	0.068107	0.135181	0.212748	0.197923
DDX25	-0.02492	0.296569	0.019975	-0.00914	-0.34359	-0.12979	-0.25188	-0.46593
DDX26B	0.361591	0.048536	-0.46755	0.071257	-0.18356	0.11685	-0.44843	-0.05582
DDX27	0.482898	0.546562	-0.16978	0.270839	0.365348	0.50249	-0.14147	-0.41804
DDX28	0.373645	0.506771	-0.19891	0.182115	0.117739	0.358391	-0.05316	-0.34788
DDX31	0.215448	0.626329	-0.03637	0.081375	0.342196	-0.16899	0.461531	0.205531
DDX39	0.06307	-0.05067	-0.13788	0.497247	0.058	0.169944	0.229693	0.142447
DDX3X	-0.02799	-0.65359	-0.27538	-0.05654	-0.27688	0.139509	0.085788	0.290342
DDX3Y	0.220934	0.063765	0.109079	-0.00409	-0.23493	-0.19689	0.202265	-0.12677
DDX4	0.175245	0.689677	-0.00953	0.292245	-0.03195	0.291377	0.213548	0.375246
DDX41	0.237202	0.599795	0.522781	0.224204	0.154021	0.148145	0.300582	0.307677
DDX42	0.008567	-0.06435	0.255117	0.220559	0.199674	0.210584	0.597853	0.311572
DDX43	-0.05753	0.40459	-0.31103	-0.21657	-0.22086	-0.12893	-0.09776	0.237069
DDX46	0.295484	-0.25064	-0.13738	-0.29706	0.194763	-0.05625	0.04681	-0.03085
DDX47	0.354121	-0.42681	-0.31135	-0.26948	-0.03094	0.287789	0.041691	-0.15723
DDX49	0.519496	-0.0075	0.093091	0.158221	0.247756	0.021084	-0.05092	-0.18864
DDX5	-0.33838	-0.62494	-0.08097	0.204452	-0.00742	0.406284	0.146678	0.430165
DDX50	0.085475	-0.37769	-0.2082	-0.23055	0.454958	-0.1484	-0.38774	-0.34965
DDX51	0.109156	-0.28781	0.240224	0.205904	0.490655	0.550448	-0.49496	-0.52028
DDX52	0.217986	-0.07836	0.006417	-0.03351	-0.08606	-0.14414	0.296473	0.434634
DDX53	-0.31402	-0.29213	-0.04697	-0.13032	0.234693	-0.06287	-0.33501	0.020088
DDX54	0.01462	0.632335	-0.05963	0.447494	0.334431	0.24202	-0.01834	-0.01938
DDX55	-0.17736	-0.05551	-0.24845	0.143843	0.17015	0.036336	-0.05736	0.173741
DDX56	0.541272	0.405576	0.106205	0.091364	0.257843	0.293455	0.574211	0.537959
DDX58	0.020441	0.013332	0.015547	-0.25795	-0.37306	-0.14125	-0.09155	0.36626
DDX59	-0.17121	-0.58389	-0.33288	0.167603	-0.09043	-0.32445	-0.26968	0.142748
DDX6	-0.12575	0.334	-0.04897	-0.03133	0.194347	0.111773	-0.17126	0.236973
DDX60	NA	NA	0.026379	-0.34956	-0.36577	-0.20963	NA	NA
DDX60L	NA	NA	-0.14605	-0.33159	-0.22501	-0.01556	NA	NA
DEAF1	0.180166	-0.04021	0.28339	-0.03863	0.300832	0.183938	0.365592	0.223525
DECR1	-0.10006	-0.04527	0.219567	-0.28274	-0.30751	-0.24358	-0.43787	-0.42008
DECR2	0.06603	0.15123	-0.04395	-0.21288	0.208051	0.00123	0.474411	0.089706
DEDD	0.203323	0.663791	0.046287	-0.04921	-0.04325	-0.0329	0.448783	0.334962
DEDD2	-0.14136	0.217625	0.289008	-0.10683	0.149284	0.141355	-0.31569	-0.39239
DEF6	0.097646	0.305543	0.341812	0.653894	0.120955	0.671428	-0.25805	-0.11047
DEF8	NA	NA	0.04738	0.30828	0.060149	-0.22632	NA	NA
DEFA3	NA	NA	0.276413	-0.13298	0.100009	-0.49296	-0.37356	-0.1186
DEFA4	-0.06512	-0.55698	0.228067	0.118269	0.258482	-0.433	0.071926	0.451055
DEFA5	0.132438	0.425135	0.18036	0.168598	0.019942	-0.20985	0.43991	0.416815
DEFA6	-0.01395	-0.38938	0.200758	0.217012	0.035157	-0.13706	0.394563	0.51937
DEFB1	0.091435	-0.06158	-0.06903	-0.13048	-0.27317	-0.27825	0.428119	0.156278
DEFB103A	NA	NA	0.150538	-0.17227	-0.01464	0.251632	0.339483	0.523811

DEFB104B	NA	NA	0.248443	0.074619	0.237563	0.062989	0.556391	0.274022
DEFB106B	NA	NA	0.057349	0.011637	0.252889	0.156331	0.237229	0.269668
DEFB108B	-0.11017	0.446333	NA	NA	NA	NA	-0.37063	-0.13199
DEFB110	NA	NA	NA	NA	NA	NA	-0.34913	-0.40049
DEFB112	NA	NA	NA	NA	NA	NA	0.388746	0.382618
DEFB114	NA	NA	-0.2406	0.064667	0.483531	0.142863	-0.06202	0.052043
DEFB118	0.179046	0.131284	-0.20369	-0.14798	-0.0291	0.278155	0.548231	0.307558
DEFB119	0.151388	0.509942	0.334625	0.3079	0.356803	0.100771	-0.06488	0.012263
DEFB121	-0.16266	-0.11434	NA	NA	NA	NA	-0.18292	-0.13168
DEFB123	-0.26931	0.189	0.139848	0.134426	-0.09141	-0.35687	-0.44207	-0.29327
DEFB124	NA	NA	NA	NA	NA	NA	-0.30777	0.170003
DEFB125	0.164609	0.351437	0.216699	0.096613	-0.21275	-0.4212	-0.46544	-0.44189
DEFB126	0.476422	0.749818	0.033478	0.036832	-0.27508	-0.17907	0.097764	-0.02792
DEFB127	0.249209	0.675686	0.401777	0.222168	0.21558	-0.1201	-0.33613	0.104999
DEFB128	NA	NA	NA	NA	NA	NA	-0.11452	0.020423
DEFB129	0.00632	0.509099	0.123987	-0.41931	-0.08799	-0.32801	-0.6201	-0.6038
DEFB131	NA	NA	NA	NA	NA	NA	-0.37818	-0.2767
DEFB134	0.439767	0.62139	NA	NA	NA	NA	0.185977	-0.09373
DEFB136	0.119616	0.029841	NA	NA	NA	NA	-0.16322	0.047236
DEGS1	-0.39535	-0.34453	0.057166	0.047186	0.005946	0.286815	-0.38413	-0.00906
DEGS2	-0.28279	0.331718	NA	NA	NA	NA	0.140691	0.416782
DEK	0.02516	-0.61901	-0.25722	-0.10056	0.170415	0.099153	0.18771	0.208092
DEM1	NA	NA	-0.05401	-0.06791	0.064333	0.067806	NA	NA
DENND1A	0.14681	-0.04062	0.056839	-0.13219	0.157065	-0.10333	0.155305	0.263497
DENND1B	0.064458	0.309962	0.020181	-0.24089	-0.07817	-0.09144	-0.17132	0.062515
DENND1C	0.123	0.691175	-0.05837	0.766686	-0.12209	0.773215	0.186393	0.607148
DENND2A	-0.28037	0.326289	0.099771	-0.058	-0.01054	-0.43403	-0.25402	-0.48851
DENND2C	-0.00723	0.236547	0.151175	-0.29126	-0.14014	0.190607	-0.28426	0.024676
DENND2D	-0.15582	-0.01959	-0.03469	0.553544	0.047148	-0.10434	0.056436	0.244493
DENND3	-0.37051	0.16427	0.033812	0.024007	-0.01459	0.584156	-0.46328	-0.00807
DENND4A	-0.51987	0.11284	-0.30817	0.133728	-0.24206	0.010932	-0.72176	-0.25468
DENND4B	NA	NA	0.210494	0.479281	0.267632	0.50952	NA	NA
DENND4C	0.206893	0.575038	0.060803	-0.20252	0.141281	0.337872	-0.49221	-0.40491
DENND5A	NA	NA	0.088673	0.01293	-0.03026	0.415144	NA	NA
DENND5B	NA	NA	0.317511	-0.11531	0.135712	-0.36117	NA	NA
DENR	0.299762	-0.32185	-0.26079	0.154571	0.098312	-0.11077	-0.47687	-0.62039
DEPDC1	-0.29992	-0.1893	-0.18931	0.040849	-0.03908	-0.06819	0.147234	0.109845
DEPDC1B	-0.00561	-0.0053	-0.17267	0.064752	-0.14888	-0.07898	0.006748	-0.24504
DEPDC4	-0.05148	0.145397	-0.11689	-0.04107	-0.08548	-0.16409	-0.21782	-0.54369
DEPDC5	0.245011	0.270086	-0.06768	0.058017	0.080447	0.074257	0.378964	0.29395
DEPDC6	0.262496	0.194036	-0.14344	-0.09953	-0.12753	-0.17832	-0.19625	-0.53377
DEPDC7	-0.00798	-0.62149	0.374414	-0.31107	0.09053	-0.17947	0.309857	0.330059
DERA	0.51819	-0.01508	-0.08271	-0.41883	0.025349	-0.35966	-0.07665	0.243691
DERL1	-0.19039	-0.42746	0.31731	0.320162	-0.2341	-0.28126	0.477291	0.429485
DERL2	-0.37804	-0.09609	-0.22238	0.0927	-0.53721	-0.28559	-0.11681	-0.07886
DERL3	-0.47278	0.289584	0.220228	0.198701	-0.14114	-0.19792	-0.05726	-0.05896
DES	0.066459	-0.12659	0.050495	0.041901	0.159068	0.130957	-0.21784	-0.31606
DET1	0.446639	0.271985	-0.071	0.023047	0.062724	-0.00571	0.257431	0.38548
DEXI	-0.02088	-0.25909	-0.02647	0.300094	0.151046	0.465364	-0.074	-0.13902
DFFA	0.081119	0.049447	0.247707	0.166113	0.329978	0.427792	0.232531	-0.10947
DFFB	-0.32045	-0.58906	0.040693	-0.00851	0.045013	0.202644	0.331884	0.478441
DFNA5	-0.098	-0.00924	0.149401	0.017358	-0.16371	-0.06245	-0.41016	0.242084

DFNB31	0.212484	0.115006	0.028557	-0.16303	-0.11241	-0.2435	0.027709	0.294214
DFNB59	NA	NA	-0.19898	-0.00171	0.132632	-0.07549	0.048441	0.043307
DGAT1	0.03785	0.47132	0.528051	-0.08356	0.161915	0.048164	0.452737	0.496659
DGAT2	0.32125	0.096411	0.115712	0.316041	0.252022	-0.10025	-0.11429	0.242771
DGAT2L3	0.389867	0.760457	-0.30771	0.295436	0.174592	-0.207	0.12661	0.091213
DGAT2L6	0.074086	-0.02552	NA	NA	NA	NA	0.343023	0.434966
DGCR14	0.24436	0.504584	0.347891	0.129784	0.138567	-0.0065	0.054655	-0.13041
DGCR2	-0.11211	-0.08752	-0.03758	-0.01826	0.352444	0.086856	0.458354	0.005674
DGCR5	NA	NA	0.042706	-0.20168	0.221353	0.0313	0.290777	0.425978
DGCR6	-0.27656	0.586005	0.289355	-0.04113	0.069081	-0.09681	0.457072	0.267892
DGCR6L	-0.28983	0.608115	0.324482	0.00184	0.164862	-0.03894	NA	NA
DGCR8	0.093483	0.285563	-0.01486	0.011531	0.359147	0.1825	0.408347	0.410531
DGKA	-0.35202	0.412978	0.108625	0.353725	-0.20171	0.152321	-0.38937	0.38087
DGKB	0.209944	0.217297	-0.07833	-0.12649	-0.05732	0.270579	-0.31661	-0.20012
DGKD	0.381863	0.241843	0.045395	0.075144	0.340438	0.32489	-0.40795	-0.10041
DGKE	0.22674	-0.01593	-0.06865	0.078547	-0.12453	-0.08547	-0.16794	-0.30546
DGKG	0.251375	-0.1408	0.079719	-0.08772	-0.0281	-0.05748	-0.45324	-0.22989
DGKH	-0.36617	-0.56855	-0.25724	-0.18473	-0.06733	-0.21965	0.076383	-0.36274
DGKI	-0.36726	-0.35315	-0.09904	-0.11869	-0.07477	0.245461	-0.51938	-0.25964
DGKK	0.039751	0.433377	NA	NA	NA	NA	0.150551	0.060905
DGKQ	-0.16485	0.107643	0.080521	-0.14462	0.11245	-0.27851	0.231988	0.164508
DGKZ	-0.32505	0.085644	0.388902	0.212709	0.323286	-0.06347	0.13821	0.080945
DGUOK	0.105106	0.444261	-0.16334	-0.09793	-0.28825	-0.35239	0.407616	-0.00766
DHCR24	-0.11639	0.275375	0.021665	0.124725	0.299332	-0.17765	8.23E-05	-0.31653
DHCR7	0.018472	0.306812	0.02944	0.312217	0.083093	0.15733	0.463815	0.279931
DHDDS	-0.35835	0.379967	0.021993	-0.04243	0.188934	-0.14037	0.124641	0.017803
DHDH	0.293765	0.579682	0.119421	-0.27903	-0.00664	0.07704	0.131379	0.43732
DHFR	0.25652	0.133046	-0.15542	0.219634	-0.03214	-0.28008	0.065471	0.3465
DHFRL1	-0.1961	0.415905	-0.07323	-0.04469	-0.14992	-0.32498	-0.16439	-0.01163
DHH	-0.11744	-0.37788	0.115057	-0.09023	0.073615	-0.23503	-0.2217	0.284618
DHODH	0.080331	-0.13504	-0.19174	0.468533	0.100812	0.218367	0.533277	0.428368
DHPS	-0.0718	-0.19789	0.196555	0.195833	0.031505	-0.18783	0.145081	0.146613
DHRS1	-0.09684	0.33927	0.023238	-0.02358	0.071996	0.150795	-0.23608	-0.14278
DHRS11	NA	NA	0.288622	-0.22823	0.095637	-0.12657	NA	NA
DHRS12	0.27494	-0.37664	0.286431	-0.18199	0.144612	-0.22833	NA	NA
DHRS13	-0.04043	0.757862	0.290788	0.336003	0.289036	0.166071	NA	NA
DHRS2	0.04775	0.421939	-0.15058	-0.12721	-0.15104	-0.36636	-0.20887	-0.18349
DHRS3	-0.41302	0.651689	0.139653	-0.04033	0.132811	-0.16836	-0.15084	0.080709
DHRS4	-0.2944	-0.44539	-0.01332	0.231692	-0.01511	-0.20767	0.16834	0.544616
DHRS4L2	-0.27075	-0.35675	NA	NA	NA	NA	0.021178	-0.06816
DHRS7	0.050613	-0.36948	-0.14666	0.138226	-0.15678	-0.38079	-0.28834	0.138479
DHRS7B	0.13182	-0.05679	-0.094	-0.10623	-0.10215	-0.46987	-0.18495	-0.1335
DHRS7C	NA	NA	-0.22573	-0.36337	-0.19523	0.050913	NA	NA
DHRS9	-0.15188	0.2135	-0.09855	-0.213	-0.05672	-0.03899	-0.10015	-0.01808
DHRSX	0.211055	-0.03889	0.021277	-0.03373	-0.16768	-0.32525	0.338847	0.267803
DHTKD1	-0.02745	0.093755	0.194261	-0.00671	0.351942	0.074703	0.026234	-0.44208
DHX15	0.297062	0.109103	-0.18712	-0.16084	0.063608	0.197892	0.055652	-0.11366
DHX16	0.464106	0.057575	-0.06096	0.118726	0.075086	0.195903	0.244338	0.554427
DHX29	-0.01962	-0.68698	0.022988	-0.18375	-0.19413	-0.28771	0.252084	0.255845
DHX30	0.322816	-0.01489	0.211741	0.175148	0.408247	0.481358	0.406323	0.255552
DHX32	-0.03302	-0.15052	0.076928	-0.18935	-0.01335	-0.24643	0.006121	-0.03734
DHX33	0.225961	0.039229	-0.31968	0.001198	0.089392	0.352195	0.607951	0.541392

DHX34	0.510534	0.68805	0.141671	0.005496	0.088193	0.022544	0.312602	0.252382
DHX35	0.367694	-0.55935	-0.09852	-0.21199	0.152875	-0.12323	0.591671	0.514394
DHX36	0.127398	-0.51359	-0.16652	-0.27978	0.009539	0.339579	0.37159	0.284605
DHX37	0.094278	-0.28147	0.207212	0.188215	0.407303	0.230173	-0.10819	0.039973
DHX38	0.2167	0.216063	0.192783	0.204978	0.423228	0.381755	0.439111	0.339372
DHX40	NA	NA	-0.13969	0.159621	-0.34543	-0.45261	0.553417	-0.03187
DHX57	0.281744	-0.14817	0.070811	0.123698	-0.01546	0.236261	0.384012	0.347312
DHX58	NA	NA	0.112532	0.321733	-0.1339	0.102957	NA	NA
DHX8	0.401923	0.681159	0.420718	-0.01626	0.05519	0.399456	-0.15677	0.283522
DHX9	-0.00272	-0.10759	-0.23932	0.41133	0.071043	0.124581	0.383571	0.150075
DIABLO	-0.2317	-0.18747	0.102173	-0.05404	0.005626	0.027391	0.181438	0.206908
DIAPH1	-0.21275	-0.07645	0.218652	0.068369	0.25125	0.318794	0.419802	0.394514
DIAPH2	0.176387	0.055751	-0.00089	-0.21334	0.005527	0.356632	-0.18261	-0.2551
DIAPH3	0.217995	0.243416	0.00804	0.041259	0.163358	-0.17509	0.343764	0.319311
DICER1	-0.3285	-0.73989	-0.0423	-0.18192	-0.00734	0.308035	-0.53099	-0.25777
DIDO1	0.308019	-0.4596	0.027083	-0.07103	0.313303	-0.27415	0.377328	0.372982
DIMT1L	0.485614	0.133752	-0.06029	0.281508	-0.07705	0.105493	NA	NA
DIO1	-0.00936	0.173649	0.200305	-0.15387	-0.29349	0.382066	0.237425	-0.13552
DIO2	-0.03591	-0.21574	0.231648	-0.21945	-0.01755	0.019885	-0.21733	0.042917
DIO3	0.11144	-0.11931	0.162729	0.029018	-0.13889	0.00394	-0.07626	-0.20597
DIO3OS	NA	NA	0.080132	-0.04116	-0.09767	-0.13233	NA	NA
DIP2A	0.058549	0.259142	0.143292	0.069649	0.260457	0.215952	0.105353	0.305936
DIP2B	-0.05104	-0.52393	-0.29684	-0.16442	-0.02609	0.055429	0.163165	-0.00624
DIP2C	-0.13659	-0.07524	0.325227	-0.17397	0.099762	0.125394	-0.21919	-0.12372
DIRAS1	-0.03258	-0.10975	-0.29372	0.03839	-0.12878	-0.16631	0.078853	-0.31686
DIRAS2	0.075793	0.398433	0.295651	0.141105	-0.11004	0.128048	-0.07678	0.006051
DIRAS3	-0.06444	-0.37172	0.005667	-0.03599	-0.27117	-0.09608	0.030728	0.053979
DIRC1	0.236548	0.273464	-0.35289	-0.00118	0.066933	0.051486	-0.11894	-0.1193
DIRC2	-0.0537	0.010146	-0.21613	-0.25075	-0.11326	-0.27397	-0.20368	0.108475
DIS3	0.191	-0.21639	0.022603	-0.26774	0.090732	0.044558	NA	NA
DIS3L	0.234102	0.290816	0.157703	0.17176	0.262365	0.487375	NA	NA
DIS3L2	0.321178	0.735029	-0.23449	0.206072	0.177093	0.119273	NA	NA
DISC1	-0.03845	0.54054	-0.27393	-0.16813	-0.40372	0.110523	-0.42517	-0.45881
DISP1	0.151143	0.073141	-0.13287	-0.02898	-0.22146	-0.43087	-0.44939	-0.32969
DISP2	-0.29637	0.632742	-0.09867	-0.06454	-0.17179	-0.17135	0.256148	0.148665
DIXDC1	-0.01647	-0.24136	0.261669	-0.23047	-0.18452	-0.05303	-0.14714	-0.17728
DKC1	0.474484	0.218505	-0.21035	0.075503	0.311268	0.087214	0.390435	0.191225
DKFZP434B	-0.20379	0.234978	0.253582	0.093819	0.205297	0.288881	-0.39493	-0.03408
DKFZP564C	-0.53094	-0.21693	0.057336	0.001374	-0.09832	-0.00693	0.190451	-0.18291
DKFZP586H	0.110611	0.107901	0.073009	-0.3035	-0.00824	0.232494	-0.28521	-0.29841
DKFZP586I:	NA	NA	-0.00878	-0.01086	0.102518	0.269052	NA	NA
DKFZP686I:	-0.25016	0.297895	-0.04751	0.226268	0.063179	-0.0847	NA	NA
DKFZp761E	NA	NA	0.06709	0.090099	0.164344	0.007503	-0.37943	-0.0554
DKK1	-0.23305	-0.06664	-0.10961	-0.06823	-0.24161	0.022981	0.335712	-0.08915
DKK2	-0.10351	-0.26662	-0.34359	-0.18102	-0.20902	-0.07022	-0.14784	-0.24065
DKK3	-0.19157	-0.49261	0.024486	-0.07252	-0.11108	0.089156	-0.30631	-0.54316
DKK4	-0.14327	-0.05846	0.434226	0.063263	0.053327	-0.28738	0.367968	0.457139
DKKL1	0.074397	0.697502	0.231836	0.126737	-0.09981	-0.17945	0.061707	-0.12854
DLAT	0.026351	0.265029	-0.268	-0.11663	-0.2966	-0.23549	0.328676	0.151359
DLC1	-0.23416	0.346159	0.030737	-0.08831	0.007329	0.384551	-0.30425	-0.00698
DLD	0.11725	-0.54601	-0.15925	-0.34487	0.063692	-0.32339	-0.33675	-0.48209
DLEC1	0.193889	0.196179	-0.23911	0.067071	-0.25612	0.199305	-0.12165	-0.05163

DLEU1	NA	NA	0.093393	0.033102	0.04684	-0.3054	0.045892	-0.42306
DLEU2	NA	NA	-0.08366	-0.08102	0.118233	-0.16396	NA	NA
DLEU7	0.022646	0.300713	-0.24363	-0.25807	0.328928	0.074849	-0.19616	-0.0325
DLG1	-0.0163	-0.10037	-0.31206	-0.25028	0.122699	-0.11289	-0.26452	-0.13278
DLG2	0.006286	0.063547	0.235023	-0.18163	-0.18197	0.096734	0.357632	-0.09287
DLG3	-0.16593	-0.06721	-0.14825	-0.14644	-0.05108	-0.02696	-0.02701	-0.12894
DLG4	-0.41815	-0.13819	0.258188	0.042592	-0.12065	0.137767	-0.41574	-0.06522
DLG5	0.134007	0.411566	0.114833	0.027443	0.427906	0.010354	-0.59722	-0.46442
DLGAP1	0.184179	0.30563	-0.23537	0.210971	0.001598	-0.14635	0.209848	-0.03448
DLGAP2	-0.15989	-0.18945	-0.00525	0.04045	-0.16229	0.153757	-0.32906	-0.53374
DLGAP3	NA	NA	0.09451	-0.04128	-0.08779	0.182583	-0.38335	0.001357
DLGAP4	0.089577	0.680578	-0.10099	-0.03388	0.064417	0.013747	0.329364	0.046324
DLGAP5	NA	NA	-0.59646	0.079326	-0.13932	-0.12376	NA	NA
DLK1	0.193764	0.233931	0.162538	-0.29829	0.004062	-0.04145	-0.09544	0.219387
DLK2	NA	NA	0.385451	-0.05505	-0.11626	-0.10153	NA	NA
DLL1	-0.37304	-0.52343	-0.13968	0.388756	-0.02877	0.046311	0.205778	0.235564
DLL3	0.161884	-0.27396	-0.33617	0.285398	-0.18534	0.021205	0.24925	0.47367
DLL4	0.18428	0.57744	0.219734	0.321947	0.293598	-0.06498	-0.28131	-0.25857
DLST	-0.33774	0.198289	-0.03831	0.063855	-0.03817	-0.09979	0.149761	0.307295
DLX1	0.066245	-0.14654	-0.03964	-0.26414	-0.00743	-0.1652	0.131777	-0.10547
DLX2	-0.05848	0.262978	0.170899	0.079934	0.034137	-0.28002	-0.2333	-0.45397
DLX3	-0.01287	0.007038	0.102063	-0.03987	-0.0041	-0.47704	0.461385	-0.20988
DLX4	0.500688	0.438205	-0.21327	0.275183	-0.06942	0.154563	0.368447	0.349353
DLX5	-0.32778	-0.41505	0.011488	-0.12061	-0.09194	-0.09364	-0.03008	-0.23038
DLX6	NA	NA	-0.16801	-0.21242	-0.04959	-0.04284	0.255815	0.174823
DMAP1	0.087674	0.201846	0.267388	-0.05519	0.202745	0.085956	-0.04679	-0.19105
DMBT1	-0.11512	0.579009	0.100297	-0.0432	0.12891	-0.19285	0.035689	0.176771
DMBX1	0.027454	0.091439	0.229107	-0.08157	0.258715	-0.06465	0.209959	-0.29486
DMC1	-0.49722	-0.26033	-0.11417	0.409302	-0.02338	-0.11876	-0.58244	-0.61706
DMD	-0.16323	-0.25352	0.029384	-0.25292	0.00039	0.333826	-0.19004	-0.34729
DMGDH	0.456493	0.617451	0.006396	-0.23748	-0.11609	0.00252	0.639221	0.253807
DMKN	0.196818	0.529611	-0.36475	0.058463	-0.27855	-0.34906	NA	NA
DMP1	-0.00335	0.001231	-0.04336	-0.14924	-0.27198	0.15455	-0.17214	-0.01289
DMPK	-0.10914	0.046539	-0.07787	-0.06217	-0.00562	0.193937	-0.13633	-0.07089
DMRT1	0.207269	0.497313	0.213154	-0.29481	-0.16353	-0.05236	-0.1114	0.211295
DMRT2	0.054859	0.03256	-0.32987	-0.06693	0.048674	-0.29113	0.020032	0.151756
DMRT3	0.047424	0.275667	0.16148	-0.09723	-0.20011	-0.1586	-0.59248	-0.23181
DMRTA1	-0.04414	0.264682	-0.12643	-0.04743	-0.29945	-0.32782	-0.3436	0.145453
DMRTB1	0.029432	-0.70391	-0.05016	0.14299	0.174099	0.014087	0.487243	-0.05383
DMRTC2	NA	NA	0.065382	-0.04793	-0.10528	0.164745	0.111978	0.294801
DMTF1	0.240601	-0.42961	-0.03968	-0.17291	0.05308	0.266471	-0.36515	0.023852
DMWD	0.152114	0.432405	0.155669	-0.0517	-0.06465	-0.1146	-0.22061	-0.2991
DMXL1	0.023866	-0.45079	0.032543	-0.30291	0.014582	-0.11737	-0.51847	-0.36113
DMXL2	-0.31437	-0.39829	-0.23706	-0.33318	-0.18745	-0.22347	0.194708	0.537991
DNA2	NA	NA	-0.36477	0.024541	0.208864	-0.10256	NA	NA
DNAH1	-0.10134	0.554212	0.105823	0.093512	0.324205	0.357173	-0.30625	0.364917
DNAH10	0.26516	0.742939	0.221727	0.11697	0.173774	0.001169	0.25668	0.050165
DNAH11	0.185379	0.327355	-0.15236	0.001238	-0.21832	0.052214	0.367977	0.517613
DNAH14	NA	NA	-0.07923	0.155541	-0.04475	-0.05621	NA	NA
DNAH17	-0.1442	0.55562	0.200882	-0.09321	-0.17734	-0.08175	0.064373	0.313504
DNAH2	-0.0241	0.136461	-0.1958	0.13588	-0.01083	0.367998	NA	NA
DNAH3	0.014261	0.278548	-0.1229	-0.43314	0.262525	-0.16093	-0.00748	-0.20991

DNAH5	0.563609	0.718396	-0.25915	-0.3119	0.075589	-0.0556	-0.30186	-0.47047
DNAH6	NA	NA	-0.15995	-0.23034	-0.05106	0.364279	NA	NA
DNAH7	0.149687	0.4683	-0.06282	-0.52132	-0.03207	-0.23678	0.354364	0.149469
DNAH8	-0.00673	0.159305	0.062473	0.344783	0.016074	0.236842	0.058934	-0.18483
DNAH9	0.162676	0.180178	-0.15368	-0.20871	-0.34409	0.058287	-0.24818	0.028107
DNAI1	0.045615	0.423207	-0.15662	-0.20858	0.079425	0.275518	0.096561	0.083705
DNAI2	-0.40128	-0.25396	-0.15997	0.276969	0.0019	-0.32056	-0.25499	-0.34669
DNAJA1	0.163798	-0.39612	-0.21853	0.065194	-0.05684	0.019543	0.016795	0.196528
DNAJA2	0.311261	0.098341	-0.31091	-0.1761	0.22724	-0.00839	-0.07439	-0.1163
DNAJA3	0.4705	-0.03864	-0.15349	0.145921	0.34056	0.212736	0.402601	-0.03747
DNAJA4	-0.24505	-0.27326	-0.12167	0.247935	0.197429	-0.00119	0.28881	0.137858
DNAJB1	0.167129	-0.17517	0.20453	0.15755	0.247809	0.091466	0.36896	-0.06339
DNAJB11	0.252796	-0.25319	-0.09879	0.261275	-0.01637	-0.06527	0.215497	0.505538
DNAJB12	0.146417	-0.30779	0.141948	-0.11021	0.382944	-0.22413	-0.42361	-0.13137
DNAJB13	-0.17419	0.09168	NA	NA	NA	NA	0.085975	0.570098
DNAJB14	-0.32873	-0.56097	-0.11531	-0.3155	-0.19954	-0.19219	-0.40482	-0.20297
DNAJB2	-0.12612	0.217347	0.523071	0.367114	0.26037	-0.06197	0.199475	0.008932
DNAJB3	NA	NA	-0.08969	-0.44845	0.124874	0.234903	NA	NA
DNAJB4	-0.31779	-0.54745	-0.15069	-0.14534	-0.34062	-0.56919	-0.27661	-0.26115
DNAJB5	-0.16863	0.157139	0.090155	-0.02466	-0.0134	0.194998	-0.24368	-0.10146
DNAJB6	-0.01902	-0.33999	-0.01362	0.009452	0.004136	0.291911	0.140984	0.058276
DNAJB7	0.326722	0.343161	0.063229	-0.2413	-0.00193	0.008128	0.35446	0.360962
DNAJB8	-0.21202	0.123238	-0.22413	-0.1362	0.044288	0.116009	-0.17654	-0.54833
DNAJB9	-0.15626	0.257331	0.412292	0.012246	0.102024	-0.06042	0.273082	0.244928
DNAJC1	-0.14986	-0.41057	0.179356	0.293422	-0.07416	-0.32427	-0.2566	-0.20377
DNAJC10	-0.27737	-0.63826	-0.12161	0.260575	-0.04147	-0.30397	0.002883	-0.21093
DNAJC11	-0.14575	-0.40029	0.044257	0.175168	0.27473	0.017341	0.080397	0.074258
DNAJC12	0.280133	-0.00168	-0.13013	0.168777	-0.16903	-0.28369	-0.02038	-0.15484
DNAJC13	0.413422	0.518959	-0.19126	-0.40155	-0.28816	-0.09667	0.036518	0.307349
DNAJC14	0.329295	-0.50657	-0.14201	0.332154	-0.06932	-0.111	0.546231	0.511314
DNAJC15	0.216798	0.180493	0.492839	-0.0225	-0.3498	0.21002	-0.25079	-0.36919
DNAJC16	-0.13316	-0.10849	-0.03825	0.198929	0.117367	-0.36567	0.40035	0.405289
DNAJC17	0.264358	0.725446	0.228418	-0.09646	-0.23488	0.134192	-0.28976	-0.01638
DNAJC18	0.19724	-0.04248	0.030866	0.094448	-0.36449	-0.23423	-0.20591	-0.38739
DNAJC19	0.250375	-0.49383	-0.21475	-0.40142	-0.14907	-0.44389	0.01629	-0.13576
DNAJC2	NA	NA	-0.20774	0.03549	0.15108	0.060016	NA	NA
DNAJC21	NA	NA	-0.05793	-0.22914	0.234576	-0.06278	NA	NA
DNAJC22	NA	NA	0.159513	0.006489	0.021889	-0.1828	NA	NA
DNAJC24	NA	NA	-0.29639	-0.41264	-0.17531	-0.20381	NA	NA
DNAJC25	NA	NA	-0.35997	-0.22212	0.035615	-0.52974	NA	NA
DNAJC27	NA	NA	0.122015	-0.46047	0.005397	0.097235	NA	NA
DNAJC28	NA	NA	-0.27409	-0.32146	-0.44872	-0.39954	NA	NA
DNAJC3	-0.11084	0.17631	0.345396	0.189315	0.263318	-0.0003	-0.05745	-0.01468
DNAJC30	NA	NA	0.279917	0.161736	-0.04681	0.1016	NA	NA
DNAJC4	0.17309	0.544547	0.286775	-0.03669	-0.07538	-0.12993	-0.40401	-0.23204
DNAJC5	0.163525	0.711138	0.243714	-0.07658	0.355681	-0.02535	0.478432	0.339455
DNAJC5B	-0.29034	0.011314	0.079728	0.282411	-0.39301	0.168961	-0.06371	0.422394
DNAJC5G	-0.35214	0.074044	-0.29521	-0.0925	0.012156	0.028199	-0.25834	-0.45474
DNAJC6	0.058487	0.195154	0.044895	0.05972	0.166345	0.095122	-0.32273	0.14584
DNAJC7	-0.07991	-0.51708	0.176869	0.174297	-0.1387	-0.09559	-0.27744	-0.33846
DNAJC8	0.020655	0.32167	-0.20356	0.043692	-0.17434	-0.02012	0.104125	0.253829
DNAJC9	-0.07262	-0.15526	-0.24474	0.214669	0.210901	-0.07062	0.172376	0.135663

DNAL1	0.019327	0.344515	-0.3062	-0.24338	-0.51145	-0.47543	-0.31972	-0.06003
DNAL4	-0.17837	0.088928	0.268126	0.013187	0.087698	-0.33428	0.500845	0.052387
DNALI1	0.155952	0.086292	-0.19969	0.126561	-0.10009	-0.30294	-0.33892	-0.11968
DNASE1	0.47209	0.129077	0.154465	-0.18222	0.067629	-0.18779	0.246617	0.424017
DNASE1L1	-0.09685	0.450983	0.268758	0.122433	0.040498	-0.25615	0.313756	0.463843
DNASE1L2	-0.00504	0.40592	-0.13108	-0.12707	0.166986	-0.28527	0.301569	0.407031
DNASE1L3	-0.01939	0.042349	0.115503	0.078045	-0.03158	0.483655	-0.29048	0.003357
DNASE2	0.057745	0.116306	0.175296	0.156835	0.175334	0.258682	-0.523	-0.18943
DNASE2B	-0.10211	0.091281	-0.22449	0.133	0.28683	-0.41961	-0.08142	-0.34521
DND1	0.112679	0.601328	-0.24703	0.145137	-0.07797	0.253761	-0.17039	-0.16432
DNER	-0.27964	-0.34615	0.064069	0.095211	0.066498	-0.1583	-0.11632	-0.30534
DNHD1	-0.23826	0.786406	-0.24797	0.004622	0.01483	0.123112	-0.26319	0.390122
DNHD2	0.017781	0.097322	0.169461	-0.20479	-0.0919	-0.01446	0.473947	0.53156
DNLZ	NA	NA	0.028827	0.003509	0.107183	0.249695	NA	NA
DNM1	0.240444	0.076479	0.091185	0.036956	0.056505	0.22753	0.239695	0.50188
DNM1L	0.251967	-0.5655	-0.36559	-0.28538	-0.13085	0.014283	-0.40045	-0.07076
DNM1P35	NA	NA	0.103243	-0.02026	-0.12732	0.273264	NA	NA
DNM2	-0.18299	0.230476	0.078774	0.15898	0.18109	0.081915	0.108699	0.1104
DNM3	0.131656	-0.52849	0.056828	-0.0284	0.01957	0.129217	0.109222	0.040588
DNMBP	0.16908	-0.05471	0.024544	-0.29236	0.386434	0.135399	0.250569	0.172071
DNMT1	0.129662	-0.44583	-0.07118	0.349502	0.425158	0.186772	-0.00093	-0.07979
DNMT3A	0.26245	0.216677	-0.27599	0.133467	0.082936	0.292383	-0.04535	-0.12704
DNMT3B	0.110511	-0.11784	-0.00801	0.124493	0.052994	-0.00647	0.553776	0.338582
DNMT3L	0.174093	0.13915	0.1819	0.265162	0.160432	0.060372	0.375085	0.425649
DNPEP	0.380716	0.41709	0.37778	0.10796	0.399655	0.086193	0.176236	0.173154
DNTT	0.10176	0.274256	0.002682	-0.10539	0.132286	-0.05073	-0.00474	0.21033
DNTTIP1	0.451684	-0.3484	-0.05954	-0.05783	0.297023	-0.00151	0.206631	-0.33556
DNTTIP2	-0.07002	-0.51357	-0.01507	-0.05442	0.069981	0.082314	-0.55239	-0.15101
DOC2A	0.472578	0.702539	-0.13895	0.202771	0.365274	0.003333	-0.10174	-0.20793
DOC2B	0.06813	-0.15965	0.306796	0.042095	0.300977	-0.09867	-0.11195	0.153799
DOCK1	-0.38524	-0.79655	-0.26912	-0.1016	0.271533	-0.11207	0.256395	0.315026
DOCK10	-0.50311	-0.22408	0.121792	0.389544	-0.33035	0.573668	-0.64287	0.167425
DOCK11	-0.00182	-0.31552	-0.0957	0.214282	-0.10602	0.463262	-0.46456	-0.2377
DOCK2	-0.51768	0.063752	0.350332	0.597206	-0.04852	0.702841	-0.58736	0.202147
DOCK3	0.0796	-0.14068	-0.09573	-0.14015	-0.21023	-0.08117	-0.22703	-0.07641
DOCK4	-0.2688	0.016435	0.095258	-0.22713	-0.25824	0.36415	-0.07579	-0.00462
DOCK5	0.1636	0.592346	0.166743	-0.19906	-0.23286	-0.16802	0.252773	0.500259
DOCK6	0.484477	0.377929	0.17031	-0.05888	0.374005	0.242105	0.45728	0.340508
DOCK7	-0.27297	-0.65706	0.339562	-0.3268	0.016232	0.116936	0.397734	0.217634
DOCK8	-0.49796	-0.29472	-0.11976	0.526337	-0.14081	0.624053	-0.38702	0.302209
DOCK9	0.11738	-0.61798	-0.03278	0.113417	0.253509	0.089753	0.189585	-0.03748
DOHH	-0.00352	0.27415	0.356047	0.314598	0.330475	0.349783	NA	NA
DOK1	0.313077	0.701862	0.239265	0.300974	0.327539	0.238607	0.174211	0.366422
DOK2	-0.42491	0.094685	0.420052	0.270887	-0.14365	0.367196	-0.57302	0.00544
DOK3	-0.36831	0.05075	0.039371	0.748622	-0.07212	0.627014	-0.31958	0.346962
DOK4	-0.19837	0.032303	0.293206	0.058993	0.213507	-0.26414	0.407983	0.051241
DOK5	-0.12844	-0.06448	0.172102	0.067427	-0.07005	-0.03251	-0.00459	0.221582
DOK6	0.342436	0.007432	-0.0619	0.087292	-0.21992	-0.04283	0.322909	0.029057
DOK7	0.18703	0.117626	-0.1506	0.287645	-0.17272	0.230621	NA	NA
DOLK	0.309468	-0.17754	0.008942	0.159471	0.268215	0.139824	NA	NA
DOLPP1	-0.29999	0.196931	0.216628	-0.03981	0.297709	-0.29217	0.021293	0.064227
DOM3Z	0.5267	-0.04261	0.090706	0.275282	0.192342	0.020919	0.51596	0.599423

DONSON	-0.26632	0.557162	-0.2667	0.2636	0.160065	-0.22162	0.401185	0.524044
DOPEY1	0.243258	-0.53386	-0.1343	-0.07501	0.218098	-0.19113	0.447247	0.507044
DOPEY2	-0.01658	-0.09236	-0.00956	0.093112	0.083879	-0.18783	-0.36866	-0.21548
DOT1L	0.168102	0.63272	0.055144	0.022051	0.331145	0.208777	-0.27573	-0.11554
DPAGT1	-0.08637	0.344314	0.27075	0.310873	0.179839	0.170613	0.407696	0.467901
DPCR1	0.011262	0.404227	0.289438	0.034338	-0.16516	0.107889	-0.4976	-0.51666
DPEP1	0.382759	0.618558	0.519062	-0.21772	0.31285	-0.20457	0.522766	0.508882
DPEP2	-0.47183	0.515599	0.27709	0.243717	-0.12132	0.077136	-0.45442	0.418021
DPEP3	0.178975	0.728494	0.175532	-0.07789	-0.05038	-0.08194	-0.67281	-0.03649
DPF1	-0.10317	0.552175	-0.15471	-0.03634	-0.11245	0.214077	0.01334	-0.04695
DPF2	0.098742	-0.03653	0.078705	-0.03148	0.205813	0.096501	0.110112	0.031948
DPF3	-0.23806	-0.22912	-0.38673	-0.12685	-0.45101	0.055448	0.460707	0.405171
DPH1	-0.08909	-0.13149	NA	NA	NA	NA	-0.10518	0.040742
DPH2	0.311619	0.338722	0.234137	0.274744	0.486045	0.300789	0.296299	0.156104
DPH3	0.542329	-0.25347	-0.29856	-0.30072	-0.26506	-0.37732	0.094758	0.237886
DPH5	0.066013	-0.38563	0.419482	-0.00971	0.175199	-0.084	-0.1281	-0.4702
DPM1	0.392514	-0.67218	-0.09566	-0.14733	0.373188	-0.09797	0.097593	-0.35879
DPM2	0.152158	0.663319	0.187207	0.271277	0.196792	-0.206	0.592919	0.17993
DPM3	-0.34495	0.716078	-0.04722	0.052225	-0.04793	-0.20118	0.371565	0.032008
DPP10	0.134446	-0.11635	0.089552	-0.14843	-0.0405	-0.27995	0.168043	0.008436
DPP3	0.180083	-0.07252	0.288859	0.158857	0.413523	0.119495	0.445372	0.112493
DPP4	0.186011	-0.24428	0.111852	-0.08339	-0.07794	-0.41602	-0.0487	0.114156
DPP6	0.25341	0.213486	-0.05352	-0.064	-0.14091	-0.27875	-0.37165	-0.3181
DPP7	-0.3932	-0.09002	0.357762	0.229436	0.409072	0.212545	-0.34512	-0.26153
DPP8	0.158087	-0.32875	-0.18988	-0.18894	-0.18422	-0.00535	-0.52817	-0.54488
DPP9	0.066797	0.522315	0.146778	0.021837	0.324613	0.068451	0.2017	0.29541
DPPA2	0.014697	0.396885	-0.26869	-0.13484	-0.0028	0.059191	-0.1289	-0.27298
DPPA3	0.040164	0.078176	0.04039	-0.106	0.282566	-0.18018	-0.02157	-0.29677
DPPA4	-0.37989	0.041304	-0.00758	0.209641	0.01001	0.294958	0.242594	0.369365
DPPA5	-0.00378	-0.45233	0.294109	0.439802	0.071863	-0.25507	-0.17859	0.105359
DPRX	0.016976	0.045958	NA	NA	NA	NA	-0.06637	0.332571
DPT	0.007414	-0.0324	0.094641	-0.08511	-0.32887	0.05796	-0.22968	-0.29286
DPY19L1	NA	NA	-0.3195	-0.01719	0.127447	0.167842	NA	NA
DPY19L2	-0.23312	-0.19798	0.018216	0.128161	-0.18742	0.250704	-0.15924	0.316219
DPY19L2P2	NA	NA	-0.08922	0.381992	0.266024	0.196175	NA	NA
DPY19L3	-0.01566	-0.32468	-0.36214	-0.08358	-0.02099	-0.15517	0.433416	0.195572
DPY19L4	0.140761	-0.52553	-0.09679	-0.38256	-0.01494	-0.11754	0.341114	-0.21462
DPY30	NA	NA	-0.00761	-0.17324	0.091184	-0.15501	NA	NA
DPYD	-0.41938	-0.63937	0.027371	-0.29338	-0.442	0.243215	-0.37292	0.076518
DPYS	0.185887	-0.11747	-0.14336	-0.164	-0.25505	0.285759	-0.60313	0.044449
DPYSL2	-0.3534	-0.54986	0.216806	0.057882	-0.09385	0.046881	0.336227	-0.03076
DPYSL3	-0.13273	-0.22655	0.234018	0.105294	0.0969	0.295785	-0.33366	-0.36567
DPYSL4	-0.08597	0.346908	-0.15794	0.262314	-0.2367	0.102159	-0.16032	-0.05135
DPYSL5	0.304255	0.265176	-0.17004	0.040724	-0.06263	0.054747	-0.1064	0.07924
DQX1	-0.08004	-0.10525	-0.10536	-0.33477	0.013223	-0.11939	0.230981	0.130581
DR1	-0.1164	-0.70482	-0.06404	-0.02379	-0.14781	-0.30016	0.122863	-0.23113
DRAP1	0.258637	0.259475	0.176191	0.465042	0.182647	0.213131	0.065565	0.028526
DRD1	-0.07828	-0.45278	0.168833	0.07764	-0.35849	-0.10686	-0.43548	0.170551
DRD2	0.261044	-0.22171	-0.15586	-0.24384	0.378492	-0.16554	0.066655	-0.45096
DRD3	0.21131	0.624205	-0.14839	-0.30711	-0.19096	-0.06282	-0.38566	-0.36434
DRD4	0.146302	0.748759	0.128568	0.012007	0.138852	0.25857	0.142027	0.342622
DRD5	0.274899	0.66404	0.116039	-0.09149	-0.07978	-0.2084	0.291881	0.142961

DRG1	0.290152	-0.28994	0.104216	-0.01182	-0.1944	0.05631	0.694365	0.126753
DRG2	0.1838	0.62014	0.135518	0.224582	-0.14032	-0.14388	0.371468	0.336464
DRP2	0.270768	0.285248	0.067566	-0.17415	-0.163	0.164716	0.561815	0.276044
DSC1	0.318317	0.116416	0.116466	-0.03466	-0.01967	-0.00989	-0.08401	0.262453
DSC2	0.039588	0.054692	-0.10665	0.004815	-0.21279	0.038609	0.34392	0.010944
DSC3	0.307168	0.045067	-0.25597	0.583065	-0.0944	-0.20787	0.330587	0.122315
DSCAM	0.017037	0.021929	-0.09454	0.043085	-0.15222	0.027699	-0.03565	-0.057
DSCAML1	-0.21574	-0.1291	-0.06396	-0.15078	-0.18432	0.0113	0.240873	0.274586
DSCC1	NA	NA	-0.06938	0.16753	0.055521	-0.11643	NA	NA
DSCR10	0.064742	0.744689	-0.1193	-0.02571	-0.04877	-0.10542	-0.10673	-0.54927
DSCR3	-0.00545	-0.4007	-0.05244	-0.19982	-0.33777	-0.46622	0.426606	0.449376
DSCR4	-0.0277	0.072261	0.241381	0.035197	0.040695	0.04086	-0.18214	-0.22716
DSCR6	0.30043	-0.0794	0.16312	0.001667	0.050789	-0.38262	0.0946	0.140104
DSCR8	-0.27378	-0.16649	-0.17555	-0.0861	-0.11826	0.04844	0.588682	0.214461
DSCR9	0.108249	0.640351	-0.16886	0.180445	0.095177	-0.18156	0.206614	-0.02915
DSE	-0.32634	-0.19901	0.266382	0.069903	-0.14682	0.391478	NA	NA
DSEL	-0.26883	0.199529	-0.17646	-0.06972	-0.31607	-0.06887	-0.10401	-0.00452
DSG1	0.017152	-0.39047	0.035075	-0.2513	-0.21342	0.051295	-0.1144	-0.08461
DSG2	-0.11288	-0.11732	0.016421	0.053999	0.06012	-0.22836	0.433182	0.18516
DSG3	-0.02272	-0.10068	0.204377	0.179039	0.024977	0.075272	0.230163	0.537731
DSG4	0.331118	0.431139	-0.00016	0.075717	-0.21012	0.235627	-0.59603	-0.52715
DSN1	0.161342	-0.39313	0.285073	0.099133	0.505917	0.088823	NA	NA
DSP	-0.16687	-0.17556	-0.13649	0.103922	0.160405	0.151816	-0.15857	-0.2346
DSPP	0.008492	0.473504	0.192332	-0.01766	-0.13693	-0.11418	-0.11113	-0.22003
DST	0.278064	0.492101	-0.18851	-0.55272	-0.24775	-0.09789	-0.1346	-0.08743
DSTN	0.0561	-0.16191	-0.29296	-0.37423	-0.00551	-0.32376	-0.33508	-0.64104
DSTYK	NA	NA	0.148442	-0.04186	-0.11216	0.196032	NA	NA
DTD1	0.287769	0.209247	-0.13255	0.059233	-0.14284	-0.28931	NA	NA
DTL	0.111704	0.375841	-0.25613	0.12646	0.258544	-0.0332	0.502572	0.501911
DTNA	0.117628	0.056356	-0.09092	-0.1189	-0.03301	-0.15657	-0.19141	0.086218
DTNB	0.256187	-0.09916	0.355484	0.138171	0.483283	0.120353	0.422135	0.409521
DTNBP1	0.113951	-0.45786	-0.05632	0.019855	0.333414	0.308175	0.295524	0.12863
DTWD1	-0.0647	-0.58424	-0.14287	-0.32325	-0.04234	-0.19808	-0.5782	-0.13779
DTWD2	-0.19878	-0.1556	-0.01055	-0.31162	-0.26931	-0.27266	-0.59241	-0.58288
DTX1	-0.10037	0.172212	0.122782	0.772394	0.200882	0.574509	0.026166	0.068159
DTX2	0.264864	-0.15449	0.22374	0.092565	0.177695	0.239558	-0.09079	-0.05199
DTX3	0.438635	0.006431	-0.01417	0.045658	-0.04842	0.289553	-0.10858	-0.09269
DTX3L	-0.10456	-0.33586	-0.25508	-0.17922	-0.15103	0.111473	0.446981	0.327992
DTX4	NA	NA	-0.07428	-0.17327	0.120442	-0.11519	NA	NA
DTYMK	-0.01276	-0.50918	-0.22423	0.405664	0.086489	-0.05268	-0.33196	-0.08145
DULLARD	-0.20439	-0.18116	0.095283	0.060578	0.090358	-0.11805	0.18396	-0.01651
DUOX1	-0.18233	0.524649	-0.21179	0.153597	-0.0666	0.41231	0.043677	0.044138
DUOX2	-0.1673	0.036987	-0.0409	0.367326	0.061885	0.258287	-0.05832	0.16999
DUOXA1	-0.06329	0.477412	-0.08886	0.19199	-0.17851	0.315175	NA	NA
DUOXA2	-0.20828	0.267195	0.097888	0.39135	-0.02487	0.139889	NA	NA
DUPD1	0.272362	0.480411	NA	NA	NA	NA	0.042569	-0.45402
DUS1L	0.307328	0.368203	0.261338	0.471	0.388925	0.008641	0.008827	-0.23685
DUS2L	0.236682	0.764174	0.21509	0.356192	0.218041	0.18474	-0.23724	-0.33982
DUS3L	0.371469	0.282054	0.090949	-0.09078	0.295553	0.250309	0.499099	0.523826
DUS4L	0.414252	0.229675	-0.28844	-0.11655	0.06386	-0.51608	0.275016	0.022944
DUSP1	-0.3818	-0.64278	0.047861	-0.09229	-0.0563	-0.25358	-0.10348	-0.06099
DUSP10	0.334513	0.193968	-0.13196	0.114725	-0.20883	-0.1276	0.273764	-0.02218

DUSP11	-0.07705	-0.63527	-0.25868	-0.18063	-0.2587	-0.19963	-0.20423	-0.3692
DUSP12	-0.2218	-0.63528	-0.20629	-0.05881	-0.17144	-0.03231	0.42004	0.245519
DUSP13	0.304241	0.607473	-0.1203	-0.01844	-0.11394	-0.09541	0.232661	-0.05069
DUSP14	-0.02218	0.045614	0.230626	-0.01896	-0.12858	0.082713	0.007999	0.01294
DUSP15	0.354636	0.416302	0.055611	-0.105	0.01776	-0.03309	-0.47486	-0.44966
DUSP16	0.673092	0.036703	-0.19398	-0.04805	0.281916	-0.18418	0.192218	0.036289
DUSP18	0.231717	0.24597	0.2525	-0.1163	0.252073	-0.05713	0.515738	0.512604
DUSP19	-0.06067	0.390947	0.021787	-0.22128	-0.26967	-0.39276	-0.41464	-0.17749
DUSP2	-0.29339	-0.0698	-0.03406	0.696169	0.042962	0.304062	-0.05931	0.02278
DUSP21	-0.11554	0.67261	0.049047	-0.44366	0.218253	0.046056	-0.07958	-0.0578
DUSP22	-0.17965	-0.3242	0.193133	0.22297	0.168474	0.008419	0.342366	0.079192
DUSP23	0.040503	0.239501	-0.1077	-0.08795	-0.00028	-0.23143	0.143235	-0.10281
DUSP26	-0.06325	0.324905	0.039576	-0.1037	-0.15289	-0.12824	-0.18024	-0.06656
DUSP27	NA	NA	-0.38515	0.012664	0.109597	-0.25052	-0.1752	-0.00438
DUSP28	0.152588	0.39514	NA	NA	NA	NA	NA	NA
DUSP3	-0.01543	0.158217	0.262169	-0.29228	0.026214	-0.35575	-0.39677	-0.25259
DUSP4	-0.38678	-0.072	0.091994	0.165158	-0.01502	-0.14786	0.084649	0.466441
DUSP5	-0.10533	-0.09287	-0.00645	-0.13084	0.058331	0.058818	-0.41605	0.2517
DUSP5P	NA	NA	0.105202	0.004785	0.041247	-0.19719	-0.24676	-0.39808
DUSP6	-0.22498	-0.17414	-0.18971	-0.24068	-0.27017	0.030207	-0.41826	-0.23579
DUSP7	-0.28375	0.707221	-0.09771	0.068773	0.201762	0.04817	0.296509	-0.07191
DUSP8	0.080149	0.056652	0.236099	-0.04177	0.508251	0.113829	-0.14164	-0.12715
DUSP9	0.241186	0.721181	-0.20439	-0.26961	0.344212	-0.26805	0.087913	-0.35244
DUT	0.507993	0.490601	-0.05196	0.359359	0.073805	-0.01188	0.038904	-0.25439
DUX1	-0.00638	0.475158	NA	NA	NA	NA	0.028619	-0.1157
DUX2	NA	NA	-0.03616	-0.0788	-0.22429	0.030985	0.114527	-0.13791
DUX4	-0.22757	-0.25035	0.061981	0.005436	-0.07613	0.191233	0.542549	0.113855
DUXA	-0.00624	0.235422	NA	NA	NA	NA	0.326787	0.037923
DVL1	-0.2398	-0.20159	0.436564	0.103241	0.364246	0.141881	0.521111	0.442616
DVL2	0.161853	0.235392	-0.1215	0.155865	-0.1956	-0.0174	0.200652	0.16283
DVL3	-0.21853	-0.46932	0.350413	-0.01472	0.308754	0.27467	-0.11166	-0.01118
DYDC1	0.211917	0.648652	0.248392	-0.24756	-0.26411	-0.2038	-0.31502	-0.24197
DYDC2	0.124654	0.599094	0.135529	0.099364	0.117773	-0.20125	NA	NA
DYM	0.103227	-0.40156	0.002489	0.119776	-0.1771	-0.35223	0.35903	0.123104
DYNC1H1	-0.22662	0.112141	0.062589	0.077942	0.210861	0.533835	-0.2914	-0.20782
DYNC1I1	-0.14942	-0.26803	0.085081	-0.1534	-0.35221	-0.03681	-0.21334	-0.26298
DYNC1I2	-0.24028	-0.69619	0.025995	-0.05173	-0.11033	-0.41454	0.645255	0.251765
DYNC1LI1	-0.12804	-0.55079	-0.1649	0.040366	-0.03105	-0.25076	0.183166	-0.13994
DYNC1LI2	0.083255	0.205598	-0.00055	-0.20637	0.43101	-0.1158	-0.16135	0.034931
DYNC2H1	NA	NA	0.235761	-0.19747	-0.21612	0.517995	-0.27899	-0.14216
DYNC2LI1	0.306112	0.178588	0.139328	-0.25237	0.035907	-0.48453	0.295269	0.452238
DYNLL1	NA	NA	-0.44887	0.024146	0.126393	-0.36282	-0.31446	-0.34743
DYNLL2	0.271311	0.445591	0.175992	-0.1814	-0.19144	-0.03629	-0.07957	0.13406
DYNLRB1	0.263099	-0.63689	0.255162	-0.16141	0.166263	-0.42939	0.171707	-0.10091
DYNLRB2	0.512861	0.312064	0.281884	-0.33475	-0.07691	-0.43167	-0.20484	-0.55814
DYNLT1	0.121693	-0.11301	-0.04505	-0.36805	0.087155	-0.4089	-0.15635	0.002448
DYNLT3	0.156164	-0.37586	-0.00928	-0.38517	-0.18764	-0.54319	-0.2021	-0.34845
DYRK1A	-0.08239	0.160874	-0.32738	-0.33607	0.163614	-0.06236	-0.00594	-0.11027
DYRK1B	0.294342	0.454992	0.081865	0.073342	-0.0346	-0.09488	0.125019	0.016931
DYRK2	-0.1137	-0.28231	-0.13543	-0.39105	-0.14386	0.035897	0.103974	0.115544
DYRK3	0.509228	0.230026	9.9E-05	0.175529	-0.09531	0.062618	-0.10903	-0.06006
DYRK4	0.010288	-0.02192	-0.04968	0.237683	-0.38083	-0.37249	-0.23919	-0.17455

DYSF	-0.32212	-0.01996	0.198312	0.036955	-0.01179	0.185356	-0.29343	-0.12021
DYX1C1	0.11415	0.46451	-0.25051	0.018137	-0.34638	-0.15459	0.102708	0.343792
DZIP1	0.118937	0.121647	-0.03733	0.089742	-0.18254	0.075057	-0.45416	-0.20663
DZIP1L	NA	NA	-0.03899	-0.01665	-0.23601	0.049518	-0.29884	-0.40288
DZIP3	-0.43967	-0.41857	-0.24726	-0.15944	-0.15641	0.266352	-0.1039	-0.27089
E2F1	0.234214	0.610286	-0.08538	0.332602	0.378444	-0.15289	0.128626	0.521044
E2F2	-0.24772	0.264331	-0.40531	0.29572	0.261957	-0.04966	0.085608	-0.01282
E2F3	-0.10212	-0.01609	-0.25915	-0.08225	0.035321	0.18113	0.074958	-0.10083
E2F4	0.389809	0.423106	0.044024	0.2398	0.333829	-0.01815	0.562104	0.459393
E2F5	0.325834	-0.23762	-0.00842	0.336985	-0.21117	0.379066	0.51948	0.324014
E2F6	0.132348	0.377648	0.014896	-0.07026	0.059065	0.132065	0.579442	0.526589
E2F7	-0.04154	0.021554	-0.19422	0.093846	0.101066	-0.18858	0.260905	0.361168
E2F8	0.382545	0.474143	-0.50302	0.200157	0.102405	-0.14212	0.182182	-0.08534
E4F1	-0.24641	0.314418	0.058838	0.004791	0.237306	0.015045	0.047592	0.008312
EAF1	-0.26998	-0.46213	-0.47358	0.087788	-0.01848	-0.43055	-0.52527	-0.44198
EAF2	-0.20581	-0.26089	0.08242	0.212929	-0.1809	-0.15148	-0.12563	-0.00232
EAPP	NA	NA	-0.46966	-0.24607	-0.26301	-0.42199	NA	NA
EARS2	0.055481	-0.42762	-0.07001	0.073278	-0.0008	0.200676	NA	NA
EBAG9	-0.09782	-0.26672	0.098698	0.117819	-0.14468	-0.19162	0.320108	0.192381
EBF1	-0.39493	-0.44377	-0.04281	0.449197	0.005958	0.469051	NA	NA
EBF2	NA	NA	-0.15476	0.023188	0.11861	0.020648	-0.02225	0.318644
EBF3	-0.12095	-0.1078	-0.04201	0.013753	-0.07436	0.15726	-0.32668	-0.07662
EBF4	NA	NA	-0.10741	-0.06258	-0.11696	0.177982	NA	NA
EBI3	0.246491	0.283163	0.369179	0.525698	0.026114	0.586717	-0.27283	0.359253
EBNA1BP2	0.157	-0.24682	-0.06723	0.365439	0.361042	-0.00975	0.509467	0.209043
EBP	0.266548	0.282235	-0.08141	0.207258	0.105831	-0.33955	0.3363	-0.00428
EBPL	0.584035	0.046246	0.006362	-0.09835	0.183096	-0.02534	NA	NA
ECD	0.111217	-0.46524	-0.11337	-0.18386	0.1885	0.127248	0.380117	0.286397
ECE1	-0.17177	-0.49697	0.401406	-0.17925	0.39616	0.272483	0.085696	0.045989
ECE2	-0.08386	-0.32321	-0.11725	0.102151	0.027478	-0.11677	0.334453	0.241425
ECEL1	0.273365	0.198052	0.311183	-0.11798	-0.10974	0.113333	-0.25473	-0.03887
ECH1	0.020594	-0.1926	-0.32803	0.075556	-0.02314	-0.17043	0.560006	0.431417
ECHDC1	0.108838	-0.39788	-0.17704	-0.23534	-0.07336	-0.12929	-0.20837	-0.0737
ECHDC2	0.171194	0.0192	0.22438	-0.0535	0.420882	0.050688	-0.23305	0.311357
ECHDC3	-0.18508	0.257937	-0.1658	-0.11184	-0.04332	0.007249	0.169236	0.209782
ECHS1	-0.28993	0.498629	0.027104	-0.00865	0.154354	-0.04333	-0.1653	-0.08743
ECM1	-0.25397	-0.43535	0.207044	-0.17342	0.043678	0.176023	-0.2235	-0.29147
ECM2	-0.42162	-0.359	-0.0242	-0.12869	-0.22741	-0.03622	-0.23585	0.286504
ECOP	0.000372	-0.15531	0.197164	0.518805	0.184043	-0.20164	0.602885	0.283815
ECSIT	0.574859	0.555068	0.13701	0.259421	0.068004	-0.044	0.094605	-0.28985
ECT2	-0.27793	-0.00179	-0.42642	-0.05799	-0.16772	-0.19986	NA	NA
EDA	0.298389	0.058175	-0.1544	-0.29304	0.205039	-0.02283	-0.21609	-0.27508
EDA2R	0.039559	-0.1091	0.130294	0.198852	0.378844	0.004682	-0.16271	-0.04044
EDAR	0.061891	-0.01556	0.378435	0.200798	-0.11988	0.104404	0.407179	0.431188
EDARADD	-0.05958	0.326025	0.124652	0.26921	0.204772	0.20897	0.24686	0.474344
EDC3	0.323887	0.651244	0.24461	-0.22003	0.010788	0.274644	NA	NA
EDC4	0.23488	0.473054	0.237184	-0.06307	0.457471	0.182415	NA	NA
EDEM1	-0.58821	-0.27506	-0.15911	0.278485	-0.16361	-0.16966	-0.09544	-0.1961
EDEM2	0.415091	0.262741	0.387745	0.34283	0.183782	-0.36757	NA	NA
EDEM3	0.314569	0.132587	-0.30435	-0.15907	-0.20439	-0.34446	-0.04735	-0.33233
EDF1	-0.13139	-0.05341	0.025764	0.155334	0.058951	0.023422	-0.56881	-0.20375
EDIL3	-0.5182	-0.51805	0.034167	-0.14377	-0.09848	-0.23836	-0.10642	0.041796

EDN1	0.101501	-0.14036	0.103028	-0.07396	-0.39682	-0.38582	-0.19945	-0.3604
EDN2	-0.08898	0.299932	0.048758	-0.08797	-0.05858	-0.30876	0.352131	0.283973
EDN3	0.070728	-0.1362	-0.16542	-0.1668	-0.15815	-0.46786	0.12492	0.087705
EDNRA	-0.02895	-0.04402	0.171486	0.348704	-0.13648	0.007288	-0.39471	-0.30331
EDNRB	-0.28047	-0.14609	-0.17405	-0.47421	-0.06117	-0.03228	-0.12348	0.559348
EEA1	0.096671	-0.64289	-0.42726	-0.11969	0.250364	0.002821	0.375705	0.37459
EED	-0.1555	-0.35472	-0.41207	-0.06172	-0.0765	-0.12217	-0.14107	-0.22825
EEF1A1	-0.18839	-0.69684	-0.05626	0.224984	0.286173	0.002737	0.801012	0.548682
EEF1A2	-0.19739	0.049614	0.08576	-0.1328	0.239056	0.091664	-0.48959	-0.41488
EEF1B2	-0.04436	-0.65059	0.144178	0.260018	0.051496	-0.18291	-0.16611	-0.31869
EEF1D	0.117464	-0.28442	0.492229	0.306433	0.221119	0.403507	-0.08979	-0.26707
EEF1E1	-0.03029	-0.32013	-0.13099	0.047179	-0.15084	-0.07259	-0.53573	-0.16669
EEF1G	NA	NA	0.273937	0.432885	0.209436	0.372084	0.028613	-0.04569
EEF2	0.219381	0.198352	0.312729	0.258906	0.318267	0.281776	-0.41763	-0.66107
EEF2K	0.107045	0.007121	-0.65543	0.004578	0.090427	-0.18758	0.12971	-0.3576
EEFSEC	0.36655	0.501076	0.008256	0.308722	0.269823	-0.09866	0.28254	0.009697
EEPD1	NA	NA	0.155744	-0.39038	0.353234	0.060841	NA	NA
EFCAB1	0.117537	-0.17523	0.045858	-0.48392	-0.14612	0.084435	0.104229	0.15989
EFCAB2	-0.01376	-0.43174	-0.16219	-0.16328	-0.22315	-0.2018	0.071183	-0.37417
EFCAB3	0.035619	0.009235	0.046175	0.217879	-0.10249	0.01399	-0.12823	-0.13212
EFCAB4A	-0.33162	0.289189	-0.01357	0.111082	-0.1166	0.004662	NA	NA
EFCAB4B	0.184238	0.636702	-0.22752	0.18714	-0.09531	-0.32278	NA	NA
EFCAB5	0.344646	0.421432	NA	NA	NA	NA	NA	NA
EFCAB6	NA	NA	0.021368	-0.08597	0.092199	-0.2251	NA	NA
EFCAB7	NA	NA	0.053429	-0.11399	0.03464	-0.20848	NA	NA
EFEMP1	0.082862	-0.17515	0.062899	0.255812	-0.19552	0.208347	-0.12583	0.240245
EFEMP2	-0.23388	-0.01929	0.309836	0.020861	0.061589	0.260731	-0.30463	-0.20237
EFHA1	0.273246	-0.01095	0.065445	-0.25212	0.300346	-0.01867	0.14132	-0.28162
EFHA2	-0.14718	-0.35078	0.098986	-0.02354	-0.11851	0.436583	-0.46463	-0.03602
EFHB	0.476878	0.398736	-0.12329	-0.31248	0.064868	-0.12903	0.073267	0.222986
EFHC1	0.242004	-0.05838	0.109262	0.029915	0.024147	-0.09768	0.352725	0.264636
EFHC2	-0.01616	0.081202	-0.07025	-0.30651	0.052736	-0.35018	-0.32539	-0.29132
EFHD1	-0.12524	-0.25753	0.160246	0.008948	-0.18779	-0.18013	-0.24599	-0.2587
EFHD2	-0.3365	0.230595	0.041925	0.147548	0.216112	0.004718	0.261575	0.585711
EFNA1	0.008054	-0.0521	-0.13088	-0.01594	0.15992	-0.21884	0.126912	0.042046
EFNA2	0.250537	0.23027	0.122874	-0.15627	0.158379	0.085178	0.255885	0.290573
EFNA3	-0.20423	0.539437	0.141789	0.225165	0.061596	-0.23246	0.379673	-0.14774
EFNA4	0.04558	0.161034	0.087487	0.042372	0.170213	-0.233	0.479611	-0.06342
EFNA5	-0.1281	-0.17643	-0.30904	0.198017	-0.51804	-0.2953	0.32132	0.321105
EFNB1	0.34687	0.383673	-0.03015	0.161327	0.248398	0.18702	0.143034	0.084438
EFNB2	-0.01107	0.047536	-0.11772	-0.18743	0.015283	0.103768	0.482813	0.006333
EFNB3	0.051607	0.186679	0.18796	0.002457	-0.09398	0.094944	0.159981	0.327949
EFR3A	NA	NA	0.301264	-0.29732	-0.17806	0.057031	NA	NA
EFR3B	NA	NA	-0.25404	-0.10413	0.095868	-0.2369	NA	NA
EFS	-0.1619	0.01583	0.031119	0.085878	-0.06358	0.081856	-0.25042	-0.05436
EFTUD1	-0.03102	-0.38793	-0.15696	-0.18118	-0.12923	0.05149	0.278325	0.285433
EFTUD2	0.410038	-0.04047	-0.06897	0.364404	0.280529	0.320042	0.519711	0.299094
EGF	0.014757	-0.09718	0.021043	-0.09252	-0.30267	-0.00942	0.022308	-0.13666
EGFL6	-0.59714	-0.1388	-0.09271	0.174155	-0.28515	0.094343	-0.23119	-0.00339
EGFL7	-0.35936	0.033513	0.005039	0.038955	0.07744	-0.06158	-0.44505	0.305216
EGFL8	0.20034	-0.25885	-0.05834	-0.06325	0.044037	0.174029	-0.43686	-0.31292
EGFLAM	-0.32997	0.045993	0.043752	-0.07586	0.080189	0.45703	-0.14224	-0.06053

EGFR	0.177425	0.159054	-0.26847	-0.3535	0.06344	-0.33252	0.388268	0.145479
EGLN1	-0.05343	0.522149	-0.1801	-0.27152	-0.1255	-0.4392	-0.04685	-0.05739
EGLN2	-0.12973	0.037727	0.363275	0.063775	0.158947	0.122301	-0.31895	-0.53718
EGLN3	-0.44612	0.147339	-0.24327	0.114993	-0.25238	-0.17976	-0.39999	-0.06244
EGR1	-0.28606	-0.45377	-0.23101	0.004941	-0.05216	-0.0839	0.100532	0.174703
EGR2	-0.13624	-0.27792	-0.2279	0.222308	-0.24561	0.041552	-0.15258	-0.01459
EGR3	-0.27972	0.243521	-0.24728	0.344781	-0.16691	0.243483	-0.09748	-0.11494
EGR4	0.22266	0.257955	0.063278	0.24135	0.023915	0.13841	-0.03834	-0.24686
EHBP1	-0.05561	-0.35036	-0.14935	-0.23524	-0.17959	0.233831	0.026634	-0.11261
EHBP1L1	NA	NA	0.251512	0.035822	0.169064	0.287736	NA	NA
EHD1	-0.49408	0.260654	0.159354	-0.02566	0.04338	0.232175	-0.56014	-0.26355
EHD2	-0.2605	-0.07853	0.066426	-0.03134	-0.00838	0.047551	-0.367	-0.17155
EHD3	-0.25462	-0.48521	0.268229	0.517401	0.145552	0.555828	-0.12096	0.235576
EHD4	-0.01896	0.072816	0.130143	-0.09974	-0.13071	-0.02458	0.367279	0.402138
EHF	-0.37172	-0.06071	0.047403	-0.0782	0.139235	-0.02209	0.261315	0.147277
EHHADH	-0.43759	-0.30769	-0.41564	-0.10107	-0.2081	-0.28394	0.26943	0.206154
EHMT1	-0.29572	-0.27045	-0.00707	0.273068	0.342293	0.086076	0.267823	0.263398
EHMT2	0.28875	0.220499	0.138445	0.201906	0.316264	0.287182	0.383297	0.104413
EI24	-0.1011	-0.64938	0.095245	0.107332	-0.05784	-0.05677	-0.31579	-0.46695
EID1	0.292892	-0.24453	0.193227	0.067372	-0.00905	0.182906	NA	NA
EID2	-0.09391	-0.66178	-0.44966	0.216378	-0.05824	-0.43166	NA	NA
EID2B	-0.08543	-0.34788	-0.01343	0.14603	-0.01988	0.096798	-0.55851	-0.52901
EID3	-0.09348	-0.31911	-0.09545	0.085988	-0.16841	0.228605	-0.65741	-0.16936
EIF1	-0.33309	-0.29595	0.146471	0.345773	0.19933	0.108746	-0.20026	0.151228
EIF1AD	NA	NA	-0.09051	-0.05701	0.063137	-0.11933	NA	NA
EIF1AX	0.164387	-0.70301	-0.08171	-0.13403	0.306371	0.046938	-0.05326	-0.24249
EIF1AY	-0.06654	0.125603	0.109288	-0.0039	-0.17478	-0.27051	0.268586	-0.0798
EIF1B	0.067627	-0.61537	-0.26488	-0.19069	-0.09347	-0.23371	-0.55516	-0.31693
EIF2A	0.14113	0.056607	0.043723	-0.16896	-0.03195	-0.16676	NA	NA
EIF2AK1	0.170434	0.102582	0.107785	0.064453	0.243013	0.123131	0.196555	-0.27721
EIF2AK2	0.181582	0.21206	0.194368	-0.29812	0.05597	-0.08647	-0.29442	-0.32545
EIF2AK3	-0.2314	-0.4125	-0.1721	-0.07937	-0.14948	-0.37224	NA	NA
EIF2AK4	0.201092	-0.62029	0.084182	0.028488	-0.4927	-0.13714	-0.52811	-0.59026
EIF2B1	-0.27194	-0.50295	-0.15775	0.125197	-0.13257	0.133119	0.5333	0.448128
EIF2B2	-0.11447	-0.34177	-0.28548	-0.07398	-0.08397	0.04396	0.242984	0.117525
EIF2B3	-0.07373	-0.05849	0.134919	0.008903	0.029026	0.098852	NA	NA
EIF2B4	0.238888	0.674528	0.207184	0.128618	0.249053	0.108803	0.605533	0.261755
EIF2B5	0.082387	0.6157	-0.194	0.297599	0.173447	0.280572	-0.45306	-0.21987
EIF2C1	-0.01339	0.168052	0.025079	-0.07351	0.252021	0.360572	0.38074	0.20176
EIF2C2	0.079475	0.433617	-0.00353	-0.11732	-0.05801	0.312169	-0.05514	-0.35122
EIF2C3	-0.14822	0.22106	0.192796	-0.18939	-0.06865	0.300625	0.277036	0.395637
EIF2C4	-0.14551	-0.17205	0.013292	-0.42931	0.066403	0.009327	-0.41465	0.052904
EIF2S1	-0.03983	0.334099	-0.36299	0.119882	0.017142	0.202671	-0.44912	-0.21694
EIF2S2	0.351298	0.00659	0.065799	-0.0098	0.122972	-0.62626	0.467057	0.347988
EIF2S3	0.241998	-0.27617	0.341756	0.075262	0.34053	0.125427	0.428864	0.288437
EIF3A	NA	NA	-0.21059	0.151285	0.240988	0.326831	NA	NA
EIF3B	NA	NA	0.194057	0.219529	0.392261	0.362576	NA	NA
EIF3D	NA	NA	0.057569	0.325695	0.34562	0.355899	NA	NA
EIF3E	NA	NA	0.247981	-0.10471	0.032764	-0.1501	NA	NA
EIF3F	NA	NA	0.233036	0.166673	0.153897	0.265281	NA	NA
EIF3G	NA	NA	0.30217	0.208074	0.222292	0.086946	NA	NA
EIF3H	NA	NA	0.141874	0.094658	0.064754	-0.27719	NA	NA

EIF3I	NA	NA	0.264764	0.198601	-0.00573	0.070146	NA	NA
EIF3J	NA	NA	-0.14541	0.037269	0.109954	0.086494	NA	NA
EIF3K	NA	NA	-0.04096	0.034513	-0.19533	-0.4047	NA	NA
EIF3L	NA	NA	0.336179	-0.12194	0.154636	-0.00502	NA	NA
EIF3M	NA	NA	-0.2126	-0.13232	-0.02776	0.075486	NA	NA
EIF4A1	-0.19692	-0.5067	-0.15463	0.312992	-0.22905	0.151392	0.332669	0.41557
EIF4A2	0.412208	0.158813	0.047241	0.030154	0.033102	-0.07689	-0.26113	-0.07664
EIF4A3	-0.07157	-0.1051	-0.09719	0.175074	0.290959	-0.02004	NA	NA
EIF4B	-0.15007	-0.58845	0.195008	0.375949	0.1113	0.103471	0.261185	0.180554
EIF4E	-0.26573	0.556102	-0.29279	-0.1364	-0.06661	-0.25176	-0.35011	-0.35156
EIF4E2	0.341167	-0.15271	-0.14539	0.024114	0.037956	-0.37202	0.10384	-0.03563
EIF4E3	-0.32911	-0.40133	-0.30354	-0.5087	-0.34957	-0.54146	-0.46783	0.015592
EIF4EBP1	-0.10886	-0.18674	0.27585	0.158257	0.060112	-0.32393	0.088367	-0.16343
EIF4EBP2	0.036457	-0.48997	-0.24033	-0.19305	0.362838	-0.39263	0.663991	0.315062
EIF4EBP3	0.572641	-0.14228	NA	NA	NA	NA	-0.36234	-0.60257
EIF4ENIF1	-0.18722	-0.68801	-0.05133	0.005359	0.290908	-0.04066	0.54025	0.127481
EIF4G1	0.253764	0.688105	0.17075	0.283917	0.340766	0.321132	0.186203	0.021113
EIF4G2	0.057563	-0.74435	-0.11415	-0.25988	0.281077	0.135686	0.411359	0.01265
EIF4G3	-0.34254	-0.21045	-0.0757	-0.13614	0.120782	0.00889	-0.29918	-0.1244
EIF4H	-0.08569	-0.68459	0.230199	0.066973	0.138237	-0.03173	0.242105	-0.00334
EIF5	-0.3365	-0.58283	-0.6103	0.000578	-0.07622	-0.27738	0.211127	0.145991
EIF5A	-0.07927	0.627657	0.058053	0.334984	-0.04627	0.02978	0.132694	-0.07564
EIF5A2	-0.23098	0.194151	-0.28171	0.015233	-0.18311	0.005314	0.116596	-0.07899
EIF5B	-0.18284	-0.29322	-0.22717	0.261342	0.000701	0.040006	0.623972	0.273829
EIF6	NA	NA	0.416143	-0.06468	0.299115	0.064383	NA	NA
ELA1	-0.13135	0.193921	-0.03742	0.017612	-0.43698	-0.02683	-0.64922	-0.29786
ELA2	0.043329	0.460411	0.259881	-0.34747	-0.23793	-0.37976	-0.32944	0.060131
ELA2A	0.061498	0.703204	0.144887	-0.11066	-0.39878	-0.0998	-0.04902	-0.64157
ELA3B	0.223104	0.598696	-0.21011	-0.48119	-0.17039	-0.28776	-0.43262	-0.18709
ELAC1	-0.25935	-0.5908	-0.12436	0.108648	-0.25632	-0.16923	0.288379	0.530087
ELAC2	0.020983	-0.41585	-0.36881	0.453629	-0.14373	0.14246	0.394094	0.296454
ELAVL1	-0.16489	-0.32071	-0.27266	0.292624	0.123529	0.104519	-0.06936	-0.08674
ELAVL2	-0.00913	0.369389	0.09236	-0.13864	0.07095	0.005078	-0.06594	0.413053
ELAVL3	0.32821	0.43094	-0.04741	-0.04602	-0.23415	0.307957	0.205974	-0.14167
ELAVL4	-0.18142	-0.07384	0.202005	-0.0679	-0.43968	0.116118	0.221807	-0.05003
ELF1	0.044101	-0.48258	-0.06395	0.038944	0.125695	0.175192	0.150227	-0.04469
ELF2	-0.44542	-0.43686	0.083456	-0.14713	-0.24047	-0.04868	-0.0248	-0.05345
ELF3	0.280353	0.086591	0.18419	0.267393	0.359653	-0.00992	0.410232	0.046766
ELF4	0.238557	0.34487	-0.1358	0.183423	0.178876	-0.0167	0.149037	0.112882
ELF5	-0.06618	0.635102	0.081493	-0.07512	-0.22767	-0.13988	0.165465	0.057248
ELFN1	NA	NA	-0.11313	0.115042	-0.30158	0.201151	NA	NA
ELFN2	NA	NA	0.209077	-0.35309	0.10703	0.192117	NA	NA
ELK1	0.186756	0.302402	0.176168	0.106993	0.330027	0.056316	-0.36969	-0.24689
ELK3	-0.55649	-0.17149	0.010686	0.317731	-0.22963	0.378084	-0.08077	0.022925
ELK4	-0.1337	-0.28331	-0.17362	0.023437	-0.0645	-0.01042	0.160236	0.394007
ELL	-0.27891	-0.47967	0.142489	0.002536	0.205122	0.232668	-0.43278	0.100886
ELL2	-0.51755	-0.33359	-0.0303	-0.08799	-0.14065	0.167407	NA	NA
ELL3	0.021188	-0.15443	-0.21095	0.311526	-0.09697	-0.16887	0.146773	0.065514
ELMO1	-0.10407	0.282276	0.125784	0.248588	-0.15481	0.346133	-0.23669	0.276987
ELMO2	0.182693	-0.32664	-0.16695	0.335806	0.263273	-0.02937	0.568855	0.294378
ELMO3	-0.2863	-0.29608	0.155417	0.192246	0.316468	-0.02179	0.471692	0.273313
ELMOD1	-0.65267	-0.31389	0.000339	-0.22221	-0.03717	-0.07536	0.067102	0.001741

ELMOD2	0.092776	0.303583	-0.28525	-0.38595	-0.26027	-0.54128	0.111984	0.183554
ELMOD3	NA	NA	0.177812	-0.19499	0.393722	0.295194	NA	NA
ELN	-0.21835	-0.25137	0.276978	0.006524	0.041623	-0.08046	-0.04799	-0.17065
ELOF1	0.330762	0.39695	0.080322	0.298433	-0.07925	-0.04122	-0.24795	-0.50636
ELOVL1	-0.3828	0.268021	0.189803	0.059989	0.238999	0.02248	-0.05731	-0.10873
ELOVL2	-0.30687	-0.01681	-0.15731	0.010476	-0.11945	-0.02777	-0.54718	-0.21452
ELOVL3	0.225056	0.578295	-0.16133	-0.257	0.035946	0.092339	-0.17009	0.24066
ELOVL4	0.053383	0.135803	-0.20173	-0.10079	-0.45916	0.144379	-0.49664	-0.35194
ELOVL5	0.131105	-0.44227	0.242276	0.401599	-0.07869	0.45555	0.013682	0.142091
ELOVL6	-0.17186	0.00325	-0.25606	-0.04703	-0.16973	0.036436	0.099071	0.005667
ELOVL7	-0.10002	-0.06259	0.204675	0.050031	-0.17929	-0.02543	0.056699	0.106059
ELP2	NA	NA	0.236139	-0.22583	-0.1145	0.389042	NA	NA
ELP3	0.13295	-0.08138	0.043735	0.060828	-0.19755	-0.12302	0.133201	0.016786
ELP4	0.30203	0.453226	-0.31334	-0.29601	-0.23363	-0.26199	0.446627	-0.3175
ELSPBP1	0.087368	0.68592	0.071575	-0.44051	-0.00145	-0.06871	0.390332	0.183247
ELTD1	NA	NA	-0.03253	0.14658	-0.04336	0.349219	NA	NA
EMB	0.156335	-0.11512	NA	NA	NA	NA	NA	NA
EMCN	-0.2393	-0.4291	-0.12395	0.108143	-0.02787	0.184378	-0.42996	-0.11376
EMD	0.066232	0.156669	0.203679	0.165785	0.332036	0.109496	-0.40766	-0.35552
EME1	-0.2833	-0.45114	0.217124	-0.11401	-0.03486	-0.30428	0.225386	0.023883
EME2	0.169098	0.686587	0.064716	0.149624	0.215646	0.06971	-0.05705	-0.06607
EMG1	0.622975	0.149648	-0.23201	0.175642	-0.08869	0.031045	0.40853	0.124255
EMID1	-0.01448	-0.08764	0.248899	0.078376	0.121659	0.203236	0.136961	0.293147
EMID2	NA	NA	0.25552	-0.14698	0.004473	0.013412	NA	NA
EMILIN1	-0.15692	0.046564	0.231411	0.020961	0.060667	0.368326	-0.22344	-0.03802
EMILIN2	-0.08623	-0.11278	0.052953	0.110396	-0.16775	0.262929	-0.63088	0.161516
EMILIN3	0.335479	-0.17265	0.225021	-0.1228	0.159898	-0.1972	-0.03443	-0.20573
EML1	-0.07281	-0.19542	0.056157	0.017524	-0.11096	0.22977	-0.24957	-0.14841
EML2	-0.35954	0.066512	0.194934	0.39404	0.235545	0.15264	NA	NA
EML3	-0.13892	0.459385	0.427391	0.356845	0.194887	0.080149	-0.30345	0.124579
EML4	0.32459	-0.26123	-0.0518	-0.07234	0.109384	0.140326	0.22616	0.207528
EML5	-0.41791	-0.31635	NA	NA	NA	NA	0.214143	0.380798
EMP1	-0.08278	-0.19155	-0.02176	-0.12665	-0.18674	-0.04018	-0.09408	4.52E-05
EMP2	-0.13869	0.436069	-0.13974	-0.35782	0.008312	0.176819	-0.47021	0.087643
EMP3	-0.36271	-0.10326	0.34656	0.195082	-0.20965	0.321704	-0.28757	-0.10031
EMR1	-0.2772	-0.11647	0.137054	0.042982	-0.27754	0.141776	-0.46553	0.019549
EMR2	-0.28913	-0.00285	0.049368	0.106179	-0.17327	0.171688	-0.41169	0.461295
EMR3	0.191095	-0.53609	-0.02375	0.007391	-0.08088	-0.27972	-0.37715	0.215034
EMX1	-0.24157	0.399538	-0.13743	0.037697	-0.04279	-0.16167	0.127915	0.050265
EMX2	-0.24035	-0.11465	0.138014	-0.2024	0.378545	0.219963	-0.31716	-0.08746
EN1	-0.00139	0.456056	-0.1038	-0.35834	0.049681	0.079252	-0.08837	-0.19551
EN2	0.039126	0.683721	-0.15454	-0.03553	-0.11436	0.134837	-0.03367	-0.26865
ENAH	0.46945	-0.09354	-0.08383	-0.19621	0.225516	0.064796	-0.07394	-0.28323
ENAM	0.419244	-0.00109	-0.09897	0.061634	-0.06754	0.115501	0.139353	0.255037
ENC1	0.153308	-0.04228	0.204251	0.097667	-0.0817	0.397755	0.486098	0.433405
ENDOD1	NA	NA	-0.09046	-0.1639	-0.17302	-0.39674	NA	NA
ENDOG	-0.30811	-0.13648	0.34692	-0.09301	0.212754	-0.02013	0.435964	-0.02789
ENG	-0.25784	-0.40059	0.418012	0.131573	0.118655	0.444876	-0.30424	0.014524
ENHO	NA	NA	0.148397	-0.24532	0.107276	-0.14179	NA	NA
ENO1	-0.15439	0.275238	0.100478	0.283628	0.210781	0.137403	0.210181	0.397977
ENO2	-0.40151	0.136036	-0.00371	0.262659	0.073455	0.49666	-0.06552	-0.07236
ENO3	0.063379	0.472525	-0.13503	0.089448	-0.16323	-0.01633	0.133143	0.391946

ENOPH1	-0.06272	-0.48126	-0.2087	0.190087	0.107591	-0.29196	NA	NA
ENOSF1	-0.01643	-0.23671	-0.15458	0.199014	-0.28074	0.12013	-0.29093	-0.07719
ENOX1	NA	NA	-0.04061	0.119811	-0.20277	0.356405	NA	NA
ENOX2	NA	NA	-0.06652	0.229106	0.125365	-0.22364	NA	NA
ENPEP	-0.41299	-0.26901	0.035621	-0.38418	-0.07622	0.090654	-0.24388	0.337155
ENPP1	0.004685	-0.21419	0.031311	-0.30776	0.326749	-0.32535	-0.25846	-0.25345
ENPP2	-0.42091	-0.61111	0.075191	0.374304	-0.16328	0.687432	-0.36131	0.312755
ENPP3	0.293255	-0.03248	-0.04282	0.129201	0.20144	-0.45047	0.10268	0.385226
ENPP4	0.289526	-0.06236	-0.08849	-0.51209	0.119011	-0.08865	0.222378	0.286989
ENPP5	0.261846	-0.15901	0.033047	-0.10853	-0.03497	-0.21094	-0.05278	-0.29778
ENPP6	-0.33533	-0.59231	-0.01542	-0.32053	-0.13828	0.019017	0.320765	0.452664
ENPP7	-0.1442	0.014074	-0.08753	-0.10209	-0.17643	0.032291	0.010367	0.257856
ENSA	-0.23026	-0.48359	0.007333	0.19275	-0.21215	-0.32993	-0.13494	-0.3352
ENTHD1	-0.19272	-0.29093	0.108325	0.505967	0.005846	0.494719	NA	NA
ENTPD1	-0.19044	-0.07641	0.212171	0.004169	-0.22369	0.276721	-0.20515	-0.08096
ENTPD2	-0.12932	0.00401	0.011559	-0.40812	0.101904	-0.17564	0.38599	0.306249
ENTPD3	-0.14085	-0.35485	-0.14871	-0.38633	-0.13767	-0.43567	-0.29344	-0.71543
ENTPD4	-0.16181	-0.22071	0.218213	0.198345	-0.27125	-0.18225	-0.13703	0.307598
ENTPD5	0.128442	0.229145	-0.34131	-0.10731	-0.17088	-0.358	0.226735	-0.16368
ENTPD6	0.135651	-0.27782	0.062987	0.013148	0.207033	0.17433	0.603494	0.43107
ENTPD7	-0.6886	-0.07755	0.154272	0.112281	0.062623	-0.15153	-0.05064	0.284307
ENTPD8	-0.52119	0.378929	-0.02478	-0.1281	-0.16482	-0.28756	0.01766	0.053493
ENY2	0.009214	-0.47491	-0.04057	-0.286	-0.31236	-0.31789	0.015082	-0.10578
EOMES	-0.15026	0.392025	-0.00258	0.460982	-0.20969	0.557422	-0.39904	0.131388
EP300	0.236635	-0.46356	0.037444	-0.03277	-0.12132	0.329124	0.688416	0.39146
EP400	-0.07839	-0.59114	0.05686	0.120984	0.284887	0.49587	0.266546	0.253505
EP400NL	0.175461	-0.33188	-0.00154	0.183197	0.07702	0.134503	0.223069	0.391707
EPAS1	-0.20882	-0.51248	0.258959	0.002791	0.264141	0.160105	0.163962	0.365606
EPB41	0.013032	-0.22562	-0.01149	0.031865	0.01781	0.213102	-0.33514	-0.20823
EPB41L1	0.172588	0.103467	0.29052	-0.0736	0.465843	-0.00722	0.489235	0.080095
EPB41L2	0.47398	-0.6125	0.000997	0.2713	0.139475	0.29476	-0.139	-0.49052
EPB41L3	-0.35531	0.234688	-0.04275	-0.41582	-0.2114	-0.25379	-0.25999	0.484683
EPB41L4A	-0.03513	0.162377	-0.08627	-0.19105	-0.28385	-0.38962	NA	NA
EPB41L4B	-0.13007	0.046052	-0.15045	-0.19975	0.24196	-0.3764	0.054375	0.036031
EPB41L5	0.073822	-0.58051	-0.05572	-0.47788	0.141	-0.432	0.27904	0.208292
EPB42	0.153683	0.268531	-0.00897	0.293565	0.112449	-0.43439	0.269617	0.181393
EPB49	0.284473	0.46419	0.198046	-0.13941	0.115848	-0.17724	0.532426	0.044749
EPC1	-0.09722	-0.54484	-0.21706	-0.22675	-0.16952	0.041919	0.049167	0.156109
EPC2	0.429846	0.529863	-0.04822	0.007646	0.015697	-0.12277	-0.28215	-0.00029
EPCAM	NA	NA	0.019711	0.038948	0.403697	-0.06308	NA	NA
EPDR1	0.361843	-0.29774	0.245291	-0.12002	0.470897	-0.24874	0.19961	0.181378
EPGN	0.448255	0.274377	-0.06062	-0.30331	0.214173	0.046778	-0.54718	-0.46786
EPHA1	0.327464	0.35116	0.255493	0.138202	0.158318	0.05981	0.442889	0.42918
EPHA10	-0.14803	0.218667	0.225773	-0.14413	0.081626	0.160548	0.519056	0.492496
EPHA2	-0.20841	0.301569	0.242367	0.027193	0.391778	0.089792	0.044851	0.184124
EPHA3	0.124219	-0.21205	0.300039	-0.08846	-0.11078	0.353253	-0.27752	-0.17297
EPHA4	-0.46858	-0.53288	-0.13957	-0.09857	-0.42882	0.248371	0.128753	-0.03539
EPHA5	0.193503	-0.278	0.116312	-0.25454	0.220806	0.068958	-0.34888	-0.45946
EPHA6	NA	NA	-0.1258	-0.24442	0.144287	0.097608	0.003854	-0.04062
EPHA7	-0.24966	0.26266	-0.14284	0.129392	-0.02605	-0.00262	-0.16939	-0.09417
EPHA8	-0.4174	0.332791	0.083552	-0.07252	-0.08159	-0.05986	0.199778	-0.19844
EPHB1	0.15368	-0.27687	0.227402	-0.05417	-0.07217	-0.09842	0.234521	-0.04545

EPHB2	0.21738	-0.06795	0.378095	0.296093	0.462042	0.290509	0.553186	0.005733
EPHB3	0.015861	0.178834	-0.08973	0.175015	0.275979	0.148248	0.187853	0.201658
EPHB4	0.122868	0.275544	0.284765	0.109949	0.473064	0.181674	0.548481	0.270363
EPHB6	-0.33085	0.246246	0.133099	0.451033	-0.1289	0.627614	0.200658	0.158691
EPHX1	-0.10437	0.091377	0.382681	-0.1015	0.304578	0.006066	0.165616	0.123778
EPHX2	-0.20961	-0.18991	0.282919	-0.20272	-0.05068	0.064972	-0.08282	-0.16098
EPM2A	-0.06859	-0.32854	-0.24262	-0.07673	-0.01182	-0.00394	-0.38259	-0.24113
EPM2AIP1	0.098962	-0.02834	-0.20406	0.022933	0.162776	0.368394	0.32056	0.453769
EPN1	0.27258	0.475494	0.077474	-0.05425	0.139569	0.003369	0.180677	0.012465
EPN2	-0.16542	0.425059	0.136833	-0.0804	0.055507	0.16963	-0.0702	-0.00036
EPN3	-0.05496	0.400148	-0.02764	0.16284	-0.22431	-0.29452	0.04602	-0.5317
EPO	0.408207	0.765105	-0.22326	-0.25923	0.17473	-0.0063	-0.24947	-0.17271
EPOR	0.0833	-0.38826	-0.18598	0.321769	-0.24198	0.013541	0.075056	0.079575
EPPK1	NA	NA	-0.05212	-0.36085	0.133639	0.192327	NA	NA
EPRS	-0.06745	-0.1555	-0.00515	0.103443	-0.00571	0.27988	0.165602	0.183123
EPS15	0.015852	-0.38044	0.009586	-0.15726	0.074153	-0.14444	-0.7358	-0.12269
EPS15L1	0.307671	0.397116	-0.16548	0.175016	0.105568	-0.49599	0.309251	0.243938
EPS8	0.264623	-0.18395	-0.05794	-0.25041	-0.11031	0.045869	NA	NA
EPS8L1	-0.23815	0.463496	0.104395	0.059446	0.012977	0.205595	0.39517	0.146962
EPS8L2	0.403913	0.353181	0.042348	-0.02814	0.454557	0.061533	0.300899	0.199648
EPS8L3	0.39342	0.490198	0.319906	0.166749	0.480946	-0.06478	0.533676	0.304243
EPSTI1	0.106646	-0.18661	0.327202	-0.27755	-0.03904	0.078387	0.045677	0.427712
EPX	0.126675	0.403943	0.159499	0.401897	0.131076	0.024607	-0.02659	-0.06949
EPYC	0.018083	-0.08373	0.18888	-0.25448	-0.31366	0.091006	NA	NA
ERAF	0.084086	0.06615	-0.02523	0.030437	0.074138	-0.47337	-0.54888	-0.22424
ERAL1	0.419321	0.66043	0.186373	0.217971	0.030647	-0.17961	0.390329	-0.05354
ERAP1	NA	NA	0.393256	-0.06512	0.104016	0.132718	NA	NA
ERAP2	NA	NA	0.196272	-0.14663	0.109883	0.270638	NA	NA
ERAS	0.452096	0.708076	0.419686	0.179005	-0.13916	-0.23274	0.248991	-0.00694
ERBB2	-0.01531	0.428997	0.156235	0.062559	0.135835	-0.07128	0.589022	0.269866
ERBB2IP	-0.17594	-0.0341	0.37608	-0.1321	0.004678	-0.0159	0.192992	0.108737
ERBB3	0.151623	-0.04884	0.076318	-0.11981	0.352947	-0.15147	0.438478	0.310713
ERBB4	0.081378	0.187794	-0.0412	0.197717	-0.14133	0.205369	0.43166	0.378863
ERC1	0.248921	0.280789	0.025892	-0.15892	0.001596	0.07183	-0.52078	-0.04531
ERC2	0.060624	-0.1243	0.125542	-0.12935	-0.08689	-0.21809	NA	NA
ERCC1	0.431192	-0.19948	0.406976	0.171552	0.033349	-0.07443	0.462339	0.05591
ERCC2	0.270942	0.473072	0.255029	-0.06395	0.289856	0.07797	0.329428	-0.30184
ERCC3	0.388781	-0.41492	0.059733	0.237572	-0.06236	0.128888	0.473757	0.528466
ERCC4	0.017895	-0.20845	-0.32679	-0.02767	-0.06062	-0.11701	0.359598	0.355122
ERCC5	-0.03972	-0.32838	-0.0122	0.098503	0.062544	0.144703	0.262481	0.405708
ERCC6	0.23873	0.563212	-0.32828	-0.30315	-0.02691	-0.0272	-0.02965	0.208442
ERCC6L	NA	NA	-0.24008	0.169111	0.133271	-0.14882	NA	NA
ERCC8	0.076176	-0.17484	-0.24265	-0.30205	-0.25265	-0.5377	0.294565	0.082988
EREG	0.266789	-0.23518	0.221867	-0.19269	0.158715	-0.26337	0.289622	0.294349
ERF	0.085789	0.033987	0.001622	0.161287	0.1612	0.191825	-0.23383	-0.13962
ERG	-0.08177	0.542256	-0.00386	0.209781	0.024238	0.014442	-0.41462	0.013103
ERGIC1	-0.32916	0.428753	0.234064	0.227283	0.147882	-0.24784	-0.49001	-0.44696
ERGIC2	0.124912	-0.28995	0.172585	-0.14471	-0.0719	0.081365	-0.73089	-0.56366
ERGIC3	0.508508	0.77836	0.36865	0.323416	0.328949	-0.05252	0.485369	-0.0931
ERH	-0.18474	-0.60268	-0.09099	-0.25053	-0.28533	-0.08779	-0.458	-0.30726
ERI1	NA	NA	-0.13511	0.006608	-0.20921	-0.14202	NA	NA
ERI2	NA	NA	-0.41559	-0.3021	-0.01568	-0.24079	NA	NA

ERI3	NA	NA	0.121881	0.100872	0.109622	-0.06485	NA	NA
ERICH1	0.087534	-0.7318	0.075359	0.140444	-0.06989	-0.1973	0.022948	0.099016
ERLIN1	0.112983	0.517201	0.053325	0.24289	0.327286	-0.11792	NA	NA
ERLIN2	-0.07103	0.304106	0.311065	-0.41134	-0.07855	-0.37588	0.617114	0.364795
ERMAP	-0.26987	-0.64408	0.112684	0.024163	0.160398	-0.19001	-0.0635	-0.15258
ERMN	NA	NA	-0.28063	0.196189	-0.00375	0.514329	NA	NA
ERMP1	NA	NA	-0.17953	-0.14124	0.178867	-0.49789	NA	NA
ERN1	0.311635	0.479649	0.431622	-0.08949	0.126088	-0.02515	-0.22563	-0.01351
ERN2	-0.36418	0.261144	-0.01767	-0.03392	-0.10584	0.015338	0.09156	-0.13574
ERO1L	-0.1808	0.548733	-0.34184	0.034756	-0.10875	-0.30811	0.042864	0.162335
ERO1LB	-0.30157	-0.28415	-0.04165	0.372839	-0.46511	-0.24761	0.152562	0.29534
ERP27	NA	NA	0.172045	-0.1918	0.024381	-0.0152	NA	NA
ERP29	NA	NA	0.089869	0.436211	0.253222	0.027396	0.147369	0.218791
ERRFI1	-0.22118	-0.32475	-0.2352	-0.10502	0.02358	-0.09454	-0.0065	0.15948
ERVWE1	0.115923	-0.40601	0.049626	-0.21794	-0.25471	0.328907	-0.26705	-0.38871
ESAM	-0.5491	-0.41802	-0.21644	0.221882	-0.11692	-0.04635	-0.39278	-0.07298
ESCO1	-0.1952	-0.54505	-0.31547	0.187697	-0.13786	0.261976	-0.39338	-0.34343
ESCO2	-0.10923	-0.33447	-0.07951	0.056377	-0.00166	-0.08532	-0.21586	-0.01751
ESD	0.405556	-0.49206	0.012632	-0.34115	0.039353	-0.36466	-0.4531	-0.64298
ESF1	0.230351	-0.64588	-0.17563	-0.12415	-0.19104	-0.12643	NA	NA
ESM1	-0.09312	-0.19028	-0.32828	-0.00361	0.168153	0.265328	-0.02986	0.086211
ESPL1	-0.03333	0.595602	0.016326	0.293132	0.323564	0.04732	0.007319	0.147082
ESPN	0.381522	0.165953	-0.00129	-0.29099	0.417932	-0.2608	0.369668	0.2103
ESPNL	0.300059	-0.07477	-0.16955	0.477347	-0.07016	0.445717	-0.35555	-0.1622
ESR1	-0.1913	-0.49821	0.233526	-0.04112	-0.39145	0.312216	-0.1362	0.459848
ESR2	0.110426	0.671519	0.02921	0.233071	0.034625	0.189184	0.116251	-0.01865
ESRRA	0.116633	0.10266	0.031378	0.09721	0.437651	-0.3021	-0.07237	0.167684
ESRRB	0.185584	0.054803	-0.20741	-0.12707	-0.11965	-0.11796	0.032942	0.291788
ESRRG	0.188228	0.116862	0.303126	-0.11962	0.205268	0.063763	0.309722	0.375062
ESX1	-0.00349	0.618631	0.275404	-0.25423	0.179802	-0.10357	-0.36785	0.137316
ETAA1	-0.2791	-0.56196	-0.12072	0.007463	-0.22528	-0.54028	NA	NA
ETF1	0.232522	-0.5318	0.201699	-0.20906	0.124021	0.247335	-0.3359	-0.34378
ETFA	-0.15245	-0.51544	-0.12164	-0.01598	-0.30926	-0.13041	0.332549	0.31371
ETFB	0.144998	-0.58046	0.133514	-0.05716	0.406148	-0.13359	0.489092	0.235688
ETFDH	-0.26457	-0.15732	-0.2663	-0.21802	-0.25953	-0.39493	0.201277	0.320015
ETHE1	0.166679	0.389035	-0.34357	-0.17561	-0.15522	-0.44364	0.003974	-0.3037
ETNK1	0.048274	0.223826	-0.40273	-0.10927	-0.24017	-0.3614	0.333665	0.130495
ETNK2	0.205082	0.178858	0.194415	0.412115	0.070314	-0.16742	0.171328	-0.13471
ETS1	-0.51562	-0.51854	-0.00841	0.632921	-0.0035	0.751633	-0.53566	-0.07958
ETS2	0.281797	0.082688	0.160321	0.068142	0.256733	0.141685	0.393843	0.325999
ETV1	-0.20839	0.011341	0.092856	-0.28439	-0.16719	-0.08679	0.288964	0.157836
ETV2	0.097248	0.539823	NA	NA	NA	NA	-0.26012	-0.25138
ETV3	-0.23659	-0.11174	0.096411	0.059212	0.063993	0.061796	0.289912	0.3012
ETV3L	NA	NA	-0.01845	0.048925	0.112786	0.102977	NA	NA
ETV4	0.552661	0.376143	0.466067	0.404376	0.484768	-0.08261	0.452937	0.398863
ETV5	-0.33461	-0.08113	0.214536	0.029397	-0.13339	0.287117	-0.04336	0.205539
ETV6	0.553734	0.461169	-0.22669	-0.11767	-0.03087	0.065966	-0.71952	-0.52882
ETV7	-0.01478	-0.53031	0.466842	-0.22146	0.092575	0.094417	NA	NA
EVC	-0.21484	-0.08342	0.231494	-0.06627	-0.08703	0.362441	-0.30052	-0.20785
EVC2	-0.07795	-0.20519	0.283878	0.237623	-0.08752	0.174686	-0.29703	-0.36119
EVI1	-0.03663	0.095416	0.015425	-0.26947	-0.17335	-0.086	0.28435	0.125693
EVI2A	-0.43683	-0.11433	0.216246	0.181617	-0.17817	0.261628	-0.60391	-0.35636

EVI2B	-0.61111	-0.0282	0.232169	0.236218	-0.2741	0.170851	-0.63292	-0.2936
EVI5	-0.16013	0.504625	-0.08116	-0.359	-0.10561	-0.49566	NA	NA
EVI5L	-0.06058	0.364069	0.083071	-0.03412	0.211608	-0.21844	-0.28649	-0.4354
EVL	-0.18759	0.360293	0.44059	0.52872	-0.19673	0.866052	-0.55294	0.046838
EVPL	0.219305	0.616095	0.385582	0.041455	0.089685	0.121844	0.198326	-0.03807
EVX1	0.184034	0.686244	0.034343	-0.09572	-0.20588	0.261148	-0.34382	-0.03724
EVX2	NA	NA	NA	NA	NA	NA	0.089864	0.072906
EWSR1	0.045157	0.353689	0.153436	0.330177	0.317816	0.131552	0.087065	-0.16395
EXDL1	0.362445	0.131261	0.097134	-0.12936	0.020168	0.15437	-0.53835	-0.18839
EXDL2	0.21134	-0.21609	-0.10372	-0.01066	-0.03503	-0.42149	0.389607	0.128578
EXO1	-0.18422	0.091621	-0.40405	0.22813	-0.05079	-0.17065	0.286587	-0.10382
EXOC1	0.07971	0.166495	-0.2412	-0.14642	-0.10795	0.158744	0.38629	0.185591
EXOC2	0.277009	-0.48504	0.052047	-0.10055	0.077534	0.222249	0.077412	-0.11552
EXOC3	0.24395	0.183424	0.064957	-0.05699	0.249694	-0.41254	NA	NA
EXOC3L	NA	NA	0.224981	0.004108	0.147562	-0.26874	-0.0371	0.249465
EXOC3L2	0.244264	0.238905	-0.08527	0.022438	-0.14383	0.296301	NA	NA
EXOC4	0.141807	0.511223	0.169145	0.015807	0.088449	0.393794	0.273214	0.048541
EXOC5	-0.32148	-0.47036	-0.43171	-0.3557	-0.48716	-0.18006	NA	NA
EXOC6	-0.20103	-0.20887	-0.1949	-0.05412	0.01712	-0.10489	-0.03526	0.189897
EXOC6B	NA	NA	0.334277	-0.31395	0.324804	0.070902	NA	NA
EXOC7	-0.14372	-0.60906	0.264359	-0.14913	0.089315	0.345639	0.17613	0.168224
EXOC8	-0.26389	-0.43086	-0.13619	-0.24828	-0.0016	0.004354	-0.11429	-0.35589
EXOG	NA	NA	-0.04794	0.166415	-0.1954	0.225475	NA	NA
EXOSC1	0.225178	0.626149	0.094256	0.094773	0.106369	-0.20851	0.207619	-0.00179
EXOSC10	-0.23325	-0.6644	0.071781	0.303101	0.090854	0.015967	-0.14671	-0.05019
EXOSC2	-0.04961	-0.34574	-0.21117	-0.03384	0.025897	0.14101	0.324359	0.175427
EXOSC3	0.432121	-0.33721	-0.31075	0.220321	0.052651	-0.08842	-0.13856	-0.20458
EXOSC4	0.230055	0.09692	0.352175	-0.13037	-0.09406	0.013071	0.222503	-0.11547
EXOSC5	0.616114	0.268373	-0.14723	0.347829	0.068053	0.250575	0.622449	0.070293
EXOSC6	0.012656	0.571306	-0.1279	0.227645	0.517717	0.221746	0.339428	0.21267
EXOSC7	0.148588	0.26135	-0.298	0.202754	-0.10135	0.174901	0.208931	-0.08751
EXOSC8	-0.28449	0.605563	0.015261	0.056265	0.132796	0.00801	-0.26181	-0.33896
EXOSC9	-0.14296	-0.47716	-0.08143	0.152552	-0.03878	0.039837	-0.52482	-0.45006
EXPH5	-0.35057	0.014726	-0.05009	-0.16822	-0.17147	0.052536	0.370291	-0.2093
EXT1	-0.41375	0.184043	0.35113	-0.17486	-0.00803	-0.05726	0.24256	0.416006
EXT2	0.278916	0.242858	0.114324	0.163931	0.222346	-0.05303	-0.2454	0.112291
EXTL1	0.492664	0.182786	0.029017	0.034546	-0.36824	-0.4136	-0.4881	-0.05565
EXTL2	-0.04002	-0.26328	-0.19306	-0.33098	-0.11186	-0.01409	0.292234	-0.02241
EXTL3	-0.04373	0.014133	0.471844	-0.02197	0.209139	0.20553	0.267476	0.195727
EYA1	0.21869	0.078226	0.534655	0.024096	-0.04855	-0.27466	0.090976	0.211147
EYA2	-0.1142	0.707616	0.211496	0.0953	0.20242	0.088507	0.359414	0.017728
EYA3	0.110871	-0.09847	-0.21668	0.067834	0.093356	0.345922	0.274263	0.2406
EYA4	0.222122	-0.0648	0.031505	-0.01624	0.004538	-0.10197	-0.18141	0.276071
EYS	NA	NA	0.050747	-0.21191	-0.24636	-0.21931	NA	NA
EZH1	0.256925	0.752616	0.454938	0.078489	0.136236	0.306239	0.224417	0.349558
EZH2	0.146277	-0.01587	-0.40067	0.205176	0.096591	-0.0814	0.273183	0.268038
EZR	NA	NA	-0.40171	0.010888	0.087442	0.124727	NA	NA
F10	0.283797	0.032061	0.267387	0.16066	0.24243	0.346328	0.514729	0.423636
F11	0.127826	0.456794	0.102367	-0.08179	-0.10662	0.018715	0.510916	0.708196
F11R	-0.05642	-0.06152	-0.20872	0.010346	0.128283	-0.06328	0.087591	-0.16131
F12	-0.05818	0.512703	0.266931	0.319144	0.197432	-0.00376	0.121959	-0.00707
F13A1	-0.15674	-0.22734	0.103083	-0.30204	-0.07957	0.115816	-0.28091	-0.07041

F13B	-0.00039	0.531329	0.144808	0.012746	0.009813	-0.08459	0.145233	0.254376
F2	0.205767	-0.04984	-0.29582	0.036023	-0.01558	-0.20938	NA	NA
F2R	-0.19487	-0.39726	-0.00878	-0.25689	-0.1326	0.166234	0.764351	0.56667
F2RL1	0.00746	-0.04248	-0.11187	-0.19337	-0.0988	-0.12112	0.186499	-0.04742
F2RL2	0.173102	-0.18271	-0.07272	-0.37504	0.0344	0.061745	-0.37959	0.302081
F2RL3	-0.0218	0.531576	-0.02845	-0.11164	0.005508	0.113748	0.013399	0.468259
F3	-0.54996	-0.37599	-0.05785	-0.06988	0.080172	0.012411	0.146194	0.092338
F5	0.023892	-0.24043	-0.10317	0.151999	-0.21524	-0.27081	-0.13833	0.123246
F7	0.174742	0.206165	0.041193	-0.40819	0.275339	0.047107	0.424484	0.36512
F8	0.13317	-0.1975	0.139871	-0.1575	0.174803	-0.04429	0.039414	0.135487
F8A1	0.306218	0.695082	-0.0039	0.237979	0.113064	0.00068	0.491938	0.370378
F9	-0.07295	-0.0204	-0.38032	0.082832	-0.00328	-0.34274	0.441375	0.261948
FA2H	-0.09831	0.362623	-0.14749	0.160892	0.275145	-0.06319	0.296047	0.137774
FAAH	0.437538	0.249092	0.353375	0.069926	0.393812	-0.13924	0.507184	0.351623
FAAH2	0.162773	-0.04442	-0.21673	-0.03409	-0.00526	-0.14186	NA	NA
FABP1	0.168247	0.020694	-0.17224	0.017501	-0.08927	0.043028	-0.39676	-0.52662
FABP12	NA	NA	0.223275	-0.05914	-0.20717	-0.20936	NA	NA
FABP2	-0.42149	0.495002	-0.12568	0.046413	-0.04899	-0.34378	-0.0173	0.324703
FABP3	0.02968	-0.23165	-0.06288	-0.35223	0.023581	0.108954	-0.31324	-0.59214
FABP4	0.139918	-0.19802	0.218675	-0.10529	-0.07223	0.272094	-0.3234	-0.04664
FABP5	NA	NA	-0.36131	0.054086	-0.55593	-0.08821	-0.63706	-0.51581
FABP6	0.322173	0.335532	0.03017	-0.06718	-0.00629	-0.10749	0.119561	0.538851
FABP7	-0.26289	0.404086	0.383799	-0.00595	0.075896	-0.08892	-0.29539	-0.08559
FADD	0.132692	0.329837	-0.1016	0.189198	0.014138	-0.05233	0.019974	-0.11957
FADS1	-0.11391	-0.32667	-0.12402	0.083775	-0.27818	0.336308	-0.08482	-0.09553
FADS2	-0.2239	0.0658	-0.11552	0.01295	-0.33592	-0.00183	-0.14778	-0.21095
FADS3	-0.05546	-0.06777	0.205536	0.321506	0.142153	0.218895	-0.36656	-0.07443
FADS6	-0.22288	0.632327	0.316691	-0.30361	0.109751	-0.19936	-0.28539	0.01138
FAF1	-0.09467	0.063629	0.284033	0.112068	0.318125	0.001395	0.465598	0.218126
FAF2	NA	NA	0.18043	0.021725	-0.07124	-0.30795	NA	NA
FAH	0.570109	-0.32674	0.21914	0.070235	0.351354	0.07695	0.114624	0.289425
FAHD1	0.217091	-0.2818	-0.28094	-0.16904	0.065944	-0.34957	-0.38059	-0.4177
FAHD2A	0.042898	0.356343	0.160764	-0.10597	0.211095	-0.29799	-0.40129	-0.39711
FAHD2B	NA	NA	NA	NA	NA	NA	0.30579	-0.06211
FAIM	-0.16756	-0.58088	-0.2234	0.157863	-0.22024	-0.04694	0.072792	-0.26023
FAIM2	0.299802	0.228568	0.251973	0.00887	-0.06277	-0.02937	-0.2333	-0.16048
FAIM3	-0.18613	0.372498	0.048702	0.868551	-0.08117	0.760206	-0.33478	0.276873
FAM100A	0.187623	0.154681	-0.12078	0.1098	0.067115	0.086796	0.480346	0.23846
FAM100B	-0.03814	0.372673	0.030835	0.195396	-0.0897	-0.24415	0.472938	0.114048
FAM101A	0.027259	0.305231	0.103433	0.029022	0.134805	-0.1713	-0.18238	-0.41969
FAM101B	-0.38948	-0.31685	0.175398	0.007569	0.059245	0.322773	-0.26271	0.23246
FAM102A	-0.04021	0.114428	0.280834	-0.10646	0.395405	-0.08099	-0.58235	-0.13208
FAM102B	-0.19696	-0.48641	0.061271	-0.21606	-0.04607	-0.11478	-0.13666	-0.37337
FAM103A1	0.210439	-0.21585	-0.18084	-0.27586	-0.02798	-0.18054	-0.52233	-0.37826
FAM104A	-0.32438	0.324911	0.098835	-0.10003	0.04808	-0.22491	0.080879	0.225756
FAM104B	0.189706	0.542525	-0.04637	-0.26859	-0.1385	-0.41108	NA	NA
FAM105A	0.102593	0.080392	0.042401	-0.24999	0.109301	-0.20173	0.102115	-0.41833
FAM105B	-0.11136	0.179411	-0.0286	-0.03976	0.07026	-0.08602	0.577133	0.464077
FAM106A	0.249414	-0.06459	0.407233	-0.00977	-0.13498	0.061202	NA	NA
FAM107A	-0.45847	-0.00613	0.082657	0.340217	-0.37122	0.296796	-0.12032	-0.28496
FAM107B	-0.24212	-0.32852	-0.01286	0.172222	-0.00687	0.243541	-0.38602	0.108596
FAM108A1	-0.37874	-0.0222	0.289846	-0.02374	0.092283	0.148707	0.357471	-0.20347

FAM108B1	NA	NA	-0.5525	0.011438	-0.25878	-0.03246	NA	NA
FAM108C1	NA	NA	-0.3244	-0.06998	0.024036	-0.47331	NA	NA
FAM109A	-0.21493	-0.07129	0.232127	0.06754	0.330695	0.193216	0.449495	0.430441
FAM109B	-0.3829	0.689472	0.300674	-0.0143	0.073849	-0.05807	0.370565	0.296091
FAM110A	NA	NA	-0.05617	0.393817	0.039698	-0.09766	NA	NA
FAM110B	NA	NA	-0.01899	0.160738	-0.07072	0.007147	NA	NA
FAM110C	NA	NA	0.324884	-0.36899	0.074178	-0.32648	0.179557	0.22461
FAM111A	-0.36932	-0.52642	-0.19983	0.035126	0.069037	-0.02906	-0.01558	0.272721
FAM111B	0.054696	0.134076	-0.0861	0.003483	-0.01662	-0.02042	0.14806	-0.03151
FAM113A	0.256973	0.298652	0.034425	0.344803	0.05432	0.155077	-0.37024	-0.04423
FAM113B	0.152814	0.427816	0.247129	0.599185	-0.28678	0.617929	-0.30104	-0.261
FAM114A1	-0.51966	-0.51365	0.03004	0.019362	-0.37164	-0.56937	NA	NA
FAM114A2	NA	NA	0.038037	0.013601	-0.16601	-0.13662	NA	NA
FAM115A	NA	NA	-0.10948	0.237419	-0.04691	-0.18372	NA	NA
FAM115C	NA	NA	-0.01292	0.183133	-0.16054	0.151899	NA	NA
FAM116A	NA	NA	-0.1706	-0.10978	0.057813	0.055034	NA	NA
FAM116B	-0.16145	0.552163	-0.04952	0.002099	-0.05131	-0.18806	NA	NA
FAM117A	0.254194	-0.61253	0.03242	0.138642	-0.22699	0.29911	NA	NA
FAM117B	NA	NA	-0.11439	-0.21272	0.045939	0.167992	NA	NA
FAM118A	0.030082	0.181511	-0.30572	-0.10927	0.107366	-0.06149	NA	NA
FAM118B	0.048987	0.480462	-0.14909	-0.04404	-0.17614	-0.1157	NA	NA
FAM119A	0.367175	0.075703	0.074371	-0.16578	0.001848	0.085339	-0.52462	-0.40906
FAM119B	-0.13336	0.492701	0.350036	-0.20715	0.506021	0.379366	NA	NA
FAM120A	-0.1877	0.208472	-0.00314	0.053885	0.200656	-8.8E-05	-0.53155	-0.52585
FAM120AO	0.026437	0.079101	-0.22185	-0.08805	0.080435	-0.3389	NA	NA
FAM120B	0.043276	0.048941	0.157495	-0.05026	0.161042	0.319734	NA	NA
FAM120C	-0.042	0.326321	-0.34711	0.202345	0.08215	-0.07246	0.091431	-0.00844
FAM122A	-0.22027	-0.5534	-0.12037	0.231037	-0.12679	-0.26661	-0.52135	-0.2098
FAM122B	0.226145	-0.64875	-0.32689	-0.08825	0.176312	-0.282	0.428354	0.463934
FAM122C	0.291802	-0.41202	-0.29719	-0.19869	0.143502	-0.12574	0.29616	0.476534
FAM123A	0.077103	0.579515	0.224746	-0.21354	-0.12021	0.025966	-0.38002	-0.25965
FAM123B	0.494537	0.508762	-0.20967	-0.1448	0.285143	0.291005	NA	NA
FAM123C	NA	NA	-0.2597	0.192738	-0.1189	-0.23713	NA	NA
FAM124A	0.259494	0.605712	0.151066	-0.14928	-0.22554	-0.07853	NA	NA
FAM124B	0.20619	0.61865	-0.00444	0.062334	-0.10176	-0.09556	NA	NA
FAM125A	0.256659	0.657632	0.292864	-0.16662	0.177711	-0.08611	-0.2135	-0.19777
FAM125B	0.329918	0.65003	0.022042	-0.18198	0.347833	0.079958	-0.02663	-0.04956
FAM126A	0.191427	0.661329	-0.03618	0.263741	-0.0718	0.123761	0.1923	-0.07184
FAM126B	-0.62801	-0.01992	-0.05877	-0.37463	-0.26065	-0.18862	-0.0387	-0.23904
FAM127A	-0.15547	-0.08647	-0.02886	-0.1023	0.030432	-0.09046	NA	NA
FAM127B	NA	NA	-0.10431	-0.26594	0.095431	-0.16853	0.218576	0.062017
FAM127C	NA	NA	NA	NA	NA	NA	-0.25557	-0.24492
FAM128B	-0.07039	0.076469	0.259132	0.014137	0.2716	0.251305	NA	NA
FAM129A	-0.50519	-0.18769	-0.09483	0.016042	-0.09784	0.032227	NA	NA
FAM129B	-0.47464	-0.18751	-0.00491	0.069525	-0.03151	0.371827	-0.10218	-0.01732
FAM129C	-0.08312	0.690914	0.104832	0.774486	-0.10395	0.704343	NA	NA
FAM12A	-0.19886	0.610851	-0.13612	0.155559	0.116151	0.141014	0.549972	0.432884
FAM12B	0.104572	0.412127	-0.0431	-0.01961	0.119168	-0.04382	0.506647	0.330706
FAM131A	NA	NA	0.376446	-0.21932	0.114036	0.084499	NA	NA
FAM131B	NA	NA	0.103244	-0.21545	0.095648	-0.32446	NA	NA
FAM131C	NA	NA	-0.07274	-0.09325	-0.15604	0.148725	NA	NA
FAM132A	NA	NA	0.217113	-0.1756	-0.11122	-0.4004	NA	NA

FAM133A	NA	NA	-0.20251	-0.22402	0.082002	-0.09952	NA	NA
FAM134A	NA	NA	0.229997	-0.08747	0.244361	-0.14833	NA	NA
FAM134B	NA	NA	-0.13702	-0.04158	0.064208	0.06378	NA	NA
FAM134C	NA	NA	0.367794	0.1693	0.381719	-0.0505	NA	NA
FAM135A	NA	NA	-0.36555	-0.30786	0.079434	-0.19951	NA	NA
FAM135B	NA	NA	0.083325	-0.05132	-0.19229	0.046075	NA	NA
FAM136A	NA	NA	-0.2134	-0.00971	0.097153	-0.31245	NA	NA
FAM13A1	-0.33666	-0.19448	-0.20503	-0.16336	-0.16339	-0.04717	-0.10513	0.041074
FAM13C1	0.154959	-0.60817	-0.25431	0.082308	-0.22816	0.092649	-0.36563	-0.05625
FAM149A	NA	NA	-0.24949	-0.08371	-0.09062	-0.13369	NA	NA
FAM149B1	NA	NA	-0.14468	0.077124	0.149634	-0.27806	NA	NA
FAM150A	NA	NA	0.124924	0.088624	-0.04917	-0.30966	NA	NA
FAM150B	NA	NA	-0.12762	-0.43324	-0.11679	0.008485	NA	NA
FAM151A	NA	NA	-0.23768	-0.27727	-0.06759	-0.25339	NA	NA
FAM153A	NA	NA	-0.03418	-0.29226	-0.07956	-0.10412	NA	NA
FAM153B	NA	NA	-0.06859	-0.21403	-0.30936	0.034279	NA	NA
FAM153C	NA	NA	-0.29204	-0.25242	-0.42235	0.016164	NA	NA
FAM154A	NA	NA	0.313916	0.138319	-0.18014	-0.12418	NA	NA
FAM154B	NA	NA	-0.18773	0.239112	-0.30296	-0.15116	NA	NA
FAM155A	NA	NA	0.033411	0.131271	-0.20121	0.042801	NA	NA
FAM155B	NA	NA	-0.08465	0.546635	0.366673	-0.10709	NA	NA
FAM159A	NA	NA	-0.24707	0.53965	-0.00081	0.44193	NA	NA
FAM160A1	NA	NA	-0.1227	-0.16099	-0.20303	0.021722	NA	NA
FAM160A2	NA	NA	0.009779	-0.12248	0.130527	0.247355	NA	NA
FAM160B1	NA	NA	-0.10998	-0.35671	0.043143	0.119136	NA	NA
FAM160B2	NA	NA	0.309038	-0.06633	0.090941	0.242756	NA	NA
FAM161A	NA	NA	-0.08267	-0.0154	0.213937	0.093156	NA	NA
FAM161B	NA	NA	-0.15563	0.032175	-0.03836	-0.09454	NA	NA
FAM162A	NA	NA	-0.21955	-0.14147	-0.341	-0.4945	NA	NA
FAM162B	NA	NA	-0.10339	0.027853	-0.26593	0.033147	NA	NA
FAM163A	NA	NA	0.178016	0.290248	0.102819	-0.13816	NA	NA
FAM165B	NA	NA	-0.0327	0.09232	-0.10612	-0.26222	NA	NA
FAM166B	NA	NA	-0.08522	-0.08553	-0.18145	-0.23929	NA	NA
FAM167A	NA	NA	0.254971	0.283105	0.134583	-0.19416	NA	NA
FAM167B	NA	NA	0.139613	0.038647	-0.01219	-0.02528	NA	NA
FAM168A	NA	NA	-0.10788	-0.1799	0.247196	-0.0127	NA	NA
FAM168B	NA	NA	0.095135	0.200481	0.392044	0.214605	NA	NA
FAM169A	NA	NA	0.123476	0.290453	-0.08362	-0.24324	NA	NA
FAM169B	NA	NA	-0.08315	0.053847	0.305821	-0.31573	NA	NA
FAM170A	NA	NA	0.069367	0.431004	0.173146	0.177848	NA	NA
FAM171A1	NA	NA	-0.16029	0.073162	0.230951	0.453046	NA	NA
FAM171B	NA	NA	-0.04603	-0.15004	-0.26445	0.208711	NA	NA
FAM172A	NA	NA	0.175786	-0.23367	-0.10511	-0.17951	NA	NA
FAM173A	NA	NA	-0.02374	0.060821	0.180692	-0.1705	NA	NA
FAM173B	NA	NA	-0.0085	-0.19008	-0.12055	-0.38998	NA	NA
FAM174A	NA	NA	-0.12094	-0.10181	-0.10195	-0.31958	NA	NA
FAM174B	NA	NA	-0.33218	0.364737	0.076489	-0.32467	NA	NA
FAM175A	NA	NA	0.067613	-0.29127	0.131173	-0.17385	NA	NA
FAM175B	NA	NA	-0.22018	-0.34168	0.021697	-0.41023	NA	NA
FAM176A	NA	NA	0.248502	0.203932	0.084183	0.182899	NA	NA
FAM176B	NA	NA	0.320667	-0.19611	-0.04523	-0.08777	NA	NA
FAM177A1	NA	NA	-0.16706	-0.2942	-0.21433	-0.53178	NA	NA

FAM178A	NA	NA	-0.19289	-0.16621	0.063843	-0.17401	NA	NA
FAM178B	NA	NA	0.164877	-0.28392	-0.086	0.054817	NA	NA
FAM179A	NA	NA	-0.22036	-0.00269	-0.07692	0.032162	NA	NA
FAM179B	NA	NA	-0.17091	-0.17926	-0.10351	-0.05799	NA	NA
FAM180A	NA	NA	0.036829	-0.32382	-0.01285	-0.08626	NA	NA
FAM181A	NA	NA	0.045219	-0.22812	-0.01658	-0.04059	NA	NA
FAM181B	NA	NA	0.059374	0.022528	0.009135	-0.00158	NA	NA
FAM183A	NA	NA	0.259702	-0.37748	-0.06036	0.175838	NA	NA
FAM183B	NA	NA	0.20229	-0.3181	-0.08835	0.307422	NA	NA
FAM184A	NA	NA	0.239775	0.032918	0.115567	0.336335	NA	NA
FAM184B	NA	NA	-0.0915	0.251961	-0.18358	0.138316	NA	NA
FAM186B	NA	NA	0.029101	-0.32951	-0.13493	0.327532	NA	NA
FAM187B	NA	NA	-0.24427	-0.22638	-0.03858	-0.01067	NA	NA
FAM18B	-0.41473	-0.56137	0.105786	-0.16485	-0.2785	-0.3943	0.196258	-0.10113
FAM18B2	-0.12306	0.601512	0.075019	0.020471	-0.25414	-0.41479	0.693249	0.109477
FAM19A1	0.421485	-0.19297	0.182164	0.01136	-0.42259	-0.21298	-0.18088	0.264156
FAM19A2	0.147328	0.268085	-0.28553	-0.31554	-0.26786	-0.06154	0.607167	0.291072
FAM19A3	0.040232	-0.01236	-0.36456	-0.06602	0.079635	0.212942	-0.36709	-0.13721
FAM19A4	0.299841	-0.16708	0.157265	0.012485	0.089455	-0.05289	-0.06304	0.184525
FAM19A5	0.25867	-0.42087	0.21252	0.366943	-0.01999	0.219835	-0.38597	-0.05926
FAM20A	-0.30638	-0.19866	0.14146	0.325717	-0.10935	0.308881	-0.49302	0.111116
FAM20B	-0.25054	-0.33531	-0.44228	-0.04378	-0.0776	-0.12687	-0.00384	-0.18298
FAM20C	-0.21872	-0.04954	0.282868	0.067625	0.106835	0.544601	-0.29559	0.134647
FAM21C	-0.14709	-0.24348	-0.09193	-0.08821	0.031719	-0.06172	-0.20541	-0.02731
FAM22D	NA	NA	-0.33327	0.105848	-0.04426	-0.25968	NA	NA
FAM22G	NA	NA	NA	NA	NA	NA	-0.30048	0.216634
FAM24A	0.148578	0.670129	0.181364	0.155001	-0.37988	0.164234	0.039547	0.004239
FAM24B	0.086019	-0.03402	-0.1266	-0.19648	0.078585	0.138414	0.144597	0.442604
FAM26D	NA	NA	-0.10497	-0.00046	0.027542	0.191425	NA	NA
FAM26E	NA	NA	0.150762	-0.07724	-0.23367	0.183351	NA	NA
FAM26F	NA	NA	0.110411	-0.05739	-0.36985	0.304968	NA	NA
FAM27L	0.062784	0.368534	0.08313	-0.23929	0.123537	0.201609	-0.35064	-0.50971
FAM29A	0.200242	-0.48634	-0.38338	-0.17592	0.081234	0.047379	-0.42329	0.115766
FAM32A	0.026449	0.33197	-0.18228	-0.08341	0.032825	-0.02311	NA	NA
FAM33A	-0.01416	0.270307	-0.18714	-0.08801	-0.17343	-0.38227	-0.2323	-0.18354
FAM35A	-0.25454	-0.66483	-0.23702	-0.22085	0.024266	-0.30518	-0.32841	-0.27951
FAM36A	-0.21668	-0.11166	0.206081	-0.19296	-0.14328	0.002173	-0.49216	-0.58613
FAM38A	0.24008	-0.29056	0.074133	0.030599	0.456169	0.248532	-0.01113	0.083939
FAM38B	-0.16383	-0.28262	-0.08828	-0.44275	0.07496	0.031174	-0.0443	-0.10198
FAM3A	0.249449	0.509815	0.230667	-0.15345	0.113387	-0.39711	0.071468	0.038315
FAM3B	0.202015	0.040095	0.190097	-0.00833	0.083683	-0.05179	0.010845	0.311263
FAM3C	0.143923	-0.67687	0.062538	0.077415	-0.2772	-0.00866	0.26388	0.509
FAM3D	-0.30232	0.113283	-0.04753	0.275928	-0.1814	-0.16684	0.167786	-0.14305
FAM40A	0.018531	0.342259	0.335431	0.346418	0.372434	0.363171	-0.1538	-0.21062
FAM40B	-0.18301	-0.08628	-0.02649	0.250033	0.210359	0.081505	-0.61009	-0.61537
FAM43A	0.106808	0.524466	0.413393	-0.17203	0.321281	-0.2971	-0.49275	-0.3777
FAM43B	0.311134	0.339033	0.08595	-0.16495	-0.17423	-0.20353	-0.26732	0.15146
FAM44A	-0.18895	0.382353	-0.06175	0.045234	-0.11225	0.192923	-0.15319	-0.03334
FAM44B	0.563719	0.31593	-0.03914	0.061426	-0.1804	-0.32958	-0.2624	-0.3704
FAM45A	-0.17901	-0.02712	-0.29547	-0.38249	-0.10489	-0.51874	-0.36416	-0.30452
FAM45B	0.051235	0.454964	-0.27396	-0.25556	-0.22051	-0.36344	0.154113	0.432303
FAM46A	-0.0883	0.039514	-0.00374	-0.00458	-0.13375	-0.13834	-0.05675	-0.03108

FAM46B	-0.0928	-0.02047	-0.18992	-0.14926	0.029029	0.282151	-0.1655	-0.26894
FAM46C	-0.58221	0.308575	0.075578	0.357773	-0.19312	-0.17099	-0.11566	-0.26088
FAM46D	-0.05614	0.186711	-0.01283	0.056887	0.005053	0.248765	0.146407	-0.17772
FAM47A	-0.05068	-0.03256	0.110447	-0.02275	0.109906	-0.01149	0.388692	-0.09102
FAM47B	0.230454	-0.0751	0.083631	-0.34899	-0.07904	0.090878	0.456191	0.116505
FAM47C	-0.15907	0.735109	-0.02316	-0.18515	-0.10992	-0.06922	0.521843	0.404493
FAM47E	NA	NA	-0.48737	-0.31551	-0.17842	-0.37508	NA	NA
FAM48A	0.429046	-0.42582	-0.01933	-0.07535	0.144132	-0.0436	0.46501	0.453486
FAM49A	-0.28853	0.505455	0.157861	0.139172	-0.13362	0.359809	-0.42502	-0.13589
FAM49B	-0.15165	-0.41369	-0.0913	0.074719	-0.12219	-0.04662	-0.44265	-0.12249
FAM50A	-0.04032	0.339271	0.143289	0.18954	0.346556	0.125626	-0.11407	0.154051
FAM50B	-0.10977	0.089825	0.011611	0.076803	-0.06329	0.079282	0.032706	0.236653
FAM53A	0.254121	-0.33635	NA	NA	NA	NA	-0.2507	-0.20286
FAM53B	-0.44622	-0.52938	0.162623	0.114642	0.315276	0.314948	-0.32524	-0.29268
FAM53C	-0.13452	0.267239	0.05229	0.038835	-0.05517	-0.10932	-0.22441	-0.26662
FAM54A	0.030905	0.205841	-0.37208	0.063751	0.224935	-0.17801	-0.08229	0.078658
FAM54B	-0.18805	-0.31577	0.10095	-0.09759	0.01242	-0.19477	NA	NA
FAM55A	0.001745	0.028332	-0.22933	-0.01283	-0.34026	-0.20205	-0.13585	-0.24318
FAM55B	-0.154	0.199888	-0.29878	-0.22109	-0.27599	-0.34993	0.145869	-0.00172
FAM55C	-0.24235	-0.31676	-0.03611	0.363981	-0.04669	0.094785	-0.53096	-0.11516
FAM55D	0.008376	-0.03527	-0.23335	-0.19749	-0.24519	-0.28697	-0.02987	-0.28197
FAM57A	-0.14295	-0.14481	0.027984	0.35941	-0.04583	0.073613	0.481335	0.374902
FAM57B	-0.47716	0.196403	-0.12639	-0.0955	0.19901	-0.07502	NA	NA
FAM58A	0.19515	0.331975	-0.39215	0.087064	0.142908	-0.44586	0.132518	-0.14892
FAM59A	-0.08908	-0.01896	0.116332	-0.26779	0.055117	-0.3099	-0.2456	-0.28576
FAM5B	0.147928	0.657786	-0.16172	0.025516	0.064295	0.023051	-0.45194	-0.30201
FAM5C	-0.2847	0.005184	-0.07162	0.182218	-0.19968	0.04664	-0.21179	0.016296
FAM60A	0.470687	0.052541	-0.20277	0.067738	0.051435	-0.29182	NA	NA
FAM62A	-0.21617	0.156973	0.099438	0.13561	0.478144	0.349637	-0.41568	-0.27362
FAM62B	0.132827	0.708054	-0.16721	-0.17456	0.037551	0.343161	0.258681	0.292383
FAM62C	-0.08227	-0.29788	-0.03432	0.005787	-0.1037	0.134201	0.076138	-0.19593
FAM63A	-0.23692	0.171796	0.069893	-0.13986	-0.10496	-0.39613	-0.46714	-0.47529
FAM63B	-0.17239	-0.24158	0.144666	-0.20221	-0.20746	0.108021	-0.58568	-0.13228
FAM64A	0.029764	-0.05201	-0.5539	0.113878	-0.18949	-0.26676	0.46221	0.25677
FAM65A	-0.06274	0.058993	0.158771	0.156732	0.118924	0.451033	-0.33014	-0.24637
FAM65B	NA	NA	0.173687	0.722446	-0.04928	0.769065	NA	NA
FAM65C	NA	NA	-0.17279	0.316559	0.122015	0.139481	NA	NA
FAM69A	0.275514	0.740526	0.061246	-0.08907	-0.16539	-0.42546	0.119894	-0.44893
FAM69B	-0.00344	-0.30258	0.135487	0.116118	-0.07592	0.036725	-0.23903	-0.32317
FAM70A	-0.07315	-0.17194	0.167783	0.018938	-0.02126	0.342401	0.070649	-0.14743
FAM70B	-0.00074	0.37148	0.349726	-0.03388	0.092937	0.089793	0.011184	0.225833
FAM71A	-0.02486	0.219484	0.06523	-0.07323	-0.33233	-0.14316	-0.35365	-0.04275
FAM71B	0.274578	0.186652	-0.03889	-0.0067	-0.07843	0.004855	NA	NA
FAM71C	0.15648	-0.0649	0.013558	-0.16615	-0.12435	-0.35061	0.55734	0.130187
FAM71D	NA	NA	-0.28176	0.007975	-0.01872	0.110523	NA	NA
FAM71E1	NA	NA	-0.13416	0.050857	0.157309	-0.466	NA	NA
FAM71E2	NA	NA	-0.11312	-0.05542	0.391267	0.134102	NA	NA
FAM71F1	NA	NA	-0.45215	-0.13024	0.024013	-0.3584	NA	NA
FAM72A	-0.21467	-0.01094	NA	NA	NA	NA	-0.15488	-0.17538
FAM73A	-0.53479	-0.34737	0.049131	-0.37368	0.138079	-0.25292	-0.58659	-0.5987
FAM73B	-0.00166	0.044561	0.114276	-0.09579	0.339664	-0.17219	-0.13832	0.369782
FAM76A	-0.35715	0.702145	-0.23566	-0.36655	0.134958	-0.55278	0.170079	0.256155

FAM76B	-0.18282	-0.60756	-0.44868	0.069576	-0.02118	-0.20785	0.49521	0.425536
FAM78A	-0.43898	-0.06121	0.148532	0.614022	-0.31611	0.337445	-0.02815	0.40207
FAM78B	-0.21454	-0.51692	NA	NA	NA	NA	0.112411	0.243512
FAM81A	0.323612	0.626736	-0.18918	0.157638	0.018195	-0.35303	0.031533	-0.08011
FAM81B	0.240102	0.185391	-0.30074	-0.13194	0.070507	0.207564	0.05323	-0.19634
FAM82A1	NA	NA	-0.17093	-0.08824	-0.08352	-0.18144	NA	NA
FAM82A2	NA	NA	0.03181	-0.06703	-0.05704	-0.04627	NA	NA
FAM82B	NA	NA	0.242772	-0.44526	-0.15024	-0.4807	NA	NA
FAM83A	-0.19845	-0.00779	-0.33606	0.172187	0.165553	-0.10395	0.01535	-0.04596
FAM83B	-0.11131	0.110017	-0.14725	-0.25864	-0.4773	-0.17326	NA	NA
FAM83C	0.146445	0.121631	-0.2666	-0.14603	0.192657	0.120361	NA	NA
FAM83D	0.283715	-0.22345	-0.292	-0.04087	0.082274	-0.06043	NA	NA
FAM83E	-0.35428	0.143931	-0.04286	0.015314	0.173679	-0.03763	0.263323	0.164844
FAM83F	-0.03132	0.643504	0.331358	-0.12169	0.272882	-0.23951	-0.14846	-0.33371
FAM83G	NA	NA	NA	NA	NA	NA	0.127533	0.207058
FAM83H	0.271497	0.534517	0.237461	-0.00586	0.065908	0.397767	NA	NA
FAM84A	0.070412	0.163054	0.356341	0.079275	0.056825	0.141678	-0.04512	-0.29237
FAM84B	-0.05708	-0.34627	-0.27457	0.035222	-0.10736	-0.22977	0.390631	0.248196
FAM86A	0.359633	-0.51644	-0.37736	-0.13369	-0.19614	-0.05219	0.388443	0.140267
FAM86C	-0.10097	-0.29401	-0.0213	0.112721	0.168751	0.025023	NA	NA
FAM89A	0.20602	-0.3561	0.286627	-0.04006	0.183814	0.371958	0.377706	0.194084
FAM89B	-0.07688	0.480982	0.14265	-0.035	-0.0517	-0.19216	0.068594	-0.03944
FAM8A1	0.003176	-0.19108	-0.19305	-0.41552	-0.26738	-0.46717	-0.36861	-0.4771
FAM90A1	0.044726	-0.19954	-0.29803	-0.17921	0.015299	0.190351	-0.07802	0.522063
FAM91A1	-0.16505	-0.08542	-0.1331	-0.16524	-0.30371	-0.29777	0.41773	0.363226
FAM92A1	NA	NA	-0.09843	0.077652	-0.1816	-0.26721	NA	NA
FAM92B	0.21454	0.047847	0.224183	0.162413	-0.25595	-0.32808	-0.26405	-0.09585
FAM96A	0.283769	-0.28109	-0.10385	-0.03326	-0.13028	-0.31135	-0.19553	-0.26362
FAM96B	0.450641	0.647286	0.207051	-0.0811	0.191739	0.053577	0.397081	-0.02677
FAM98A	0.021626	-0.14587	0.075171	-0.06803	-0.06147	-0.04317	-0.56903	-0.43337
FAM98B	-0.01401	0.168889	-0.32435	-0.04795	-0.08565	0.390575	-0.29304	0.483106
FAM98C	0.032626	0.406212	0.151558	-0.10498	0.035942	-0.13963	0.448772	0.340189
FAM9A	0.330972	0.308799	0.18626	0.115562	-0.04451	0.236577	0.144894	-0.01117
FAM9B	-0.0838	-0.12878	0.314453	0.005648	-0.01516	0.167936	0.239531	0.267735
FAM9C	-0.24638	0.364399	-0.01173	-0.42279	0.117234	0.118138	0.202233	0.238566
FANCA	0.017388	0.162235	-0.24629	0.469559	0.294742	0.133549	0.384551	0.400784
FANCB	0.1477	-0.18709	-0.0346	0.059073	0.24216	0.112297	-0.0313	-0.44542
FANCC	0.077491	0.591042	-0.07315	0.193569	0.242101	0.013873	-0.52108	-0.42342
FANCD2	-0.12381	0.084057	-0.34715	0.285893	0.249743	0.054916	0.26456	0.211421
FANCE	0.039442	0.485081	-0.3773	0.383366	0.159689	0.027337	0.055361	-0.13647
FANCF	0.309026	0.050155	-0.07487	0.154091	0.293388	-0.18614	0.402561	0.224621
FANCG	-0.10681	0.418893	-0.35483	0.542865	0.21066	-0.18794	0.039867	-0.0936
FANCI	NA	NA	-0.27806	0.099857	0.175534	-0.07553	NA	NA
FANCL	-0.07981	-0.58566	-0.22324	-0.14761	0.155581	0.008348	-0.11748	-0.6183
FANCM	0.002671	0.47578	-0.50888	0.046472	-0.20283	-0.01695	-0.12119	-0.27807
FANK1	-0.28254	-0.29631	-0.15175	-0.07422	-0.08307	-0.34408	0.213112	0.355381
FAP	-0.23407	-0.25739	-0.08098	0.051685	-0.02807	-0.21807	0.001431	0.043837
FAR1	NA	NA	-0.42744	-0.18809	-0.27601	-0.00103	NA	NA
FAR2	NA	NA	-0.24313	0.092339	-0.07425	-0.31032	NA	NA
FARP1	0.199241	-0.00067	0.161121	-0.12697	0.448032	0.09763	0.38853	-0.13238
FARP2	0.255581	0.300715	0.094339	-0.09386	0.32405	-0.36823	0.327425	0.039332
FARS2	0.506187	0.008924	-0.22963	-0.03595	-0.20641	0.039795	0.39187	-0.04245

FARSA	0.46616	0.679426	0.178133	0.272653	0.312088	0.252224	NA	NA
FARSB	NA	NA	0.053074	0.049259	0.118809	0.008028	0.329428	0.515685
FAS	0.000149	-0.02291	0.08768	-0.12697	-0.66037	-0.38081	-0.34178	-0.13036
FASLG	0.047625	0.073485	0.282397	0.049501	-0.35848	0.095245	-0.28811	0.376255
FASN	0.209939	0.513736	0.106568	0.203262	0.179758	0.071891	0.293351	-0.14095
FASTK	-0.12773	0.093647	0.060095	0.139287	0.027674	-0.51171	0.004278	0.250066
FASTKD1	-0.0739	-0.5546	-0.06817	-0.14702	-0.02909	-0.14997	NA	NA
FASTKD2	0.224238	-0.54609	-0.12446	-0.27309	-0.06342	-0.37836	0.612356	0.343359
FASTKD3	0.255259	0.556346	-0.33158	-0.33899	0.016383	-0.29039	0.640802	0.348232
FASTKD5	0.176203	-0.58774	-0.31514	-0.14616	-0.15093	-0.21066	NA	NA
FAT1	NA	NA	0.193254	-0.16922	0.125861	0.21696	NA	NA
FAT2	0.336568	0.75298	-0.07715	0.057152	-0.1296	0.003063	-0.08925	0.227749
FAT3	NA	NA	0.36288	-0.13816	-0.17509	0.040847	NA	NA
FAT4	-0.05635	-0.35308	-0.17515	-0.15625	-0.06551	-0.05032	-0.37084	-0.48064
FATE1	-0.42259	-0.25888	-0.0957	-0.2207	-0.02461	-0.15205	-0.34407	0.13524
FAU	0.058309	-0.35148	0.000347	0.324984	-0.02053	-0.08343	-0.17902	-0.14195
FBF1	NA	NA	0.161528	0.186232	0.338572	0.103648	0.032791	0.017489
FBL	0.235113	0.408861	-0.37598	0.428837	0.209692	0.329875	0.321245	-0.31451
FBLIM1	-0.02536	0.120415	0.188503	-0.27587	0.228381	-0.04313	0.205196	0.161791
FBLN1	-0.04245	-0.2961	0.16109	0.098969	0.017432	-0.05318	0.047095	0.075317
FBLN2	-0.26198	-0.28899	0.086681	0.041622	-0.20057	-0.0564	-0.23886	-0.01162
FBLN5	-0.08973	-0.32269	0.103181	0.157651	-0.14285	0.03617	-0.1698	-0.06578
FBLN7	NA	NA	0.340059	0.361313	0.047643	0.314301	NA	NA
FBN1	-0.07211	-0.10043	0.083815	-0.092	-0.11562	-0.06534	-0.31762	0.115172
FBN2	-0.05056	-0.09279	-0.07708	-0.22491	-0.22199	0.00585	0.101216	-0.02662
FBN3	0.106698	0.126953	0.165744	-0.228	0.31084	0.004096	0.488056	0.07134
FBP1	-0.09992	0.143064	-0.1129	0.03471	-0.13226	-0.13871	-0.04431	0.49216
FBP2	0.1359	0.61757	-0.20485	0.430627	-0.02497	-0.05795	0.046036	0.114959
FBRS	NA	NA	0.09263	0.071428	0.380986	0.086509	NA	NA
FBXL10	0.1705	0.082759	-0.02332	0.690305	0.559221	0.51309	0.045183	0.157324
FBXL12	0.146296	-0.61957	-0.10951	-0.04158	0.085881	-0.20597	-0.25063	-0.18211
FBXL13	-0.33884	-0.49262	0.127397	-0.29023	0.125896	-0.174	-0.46881	-0.18078
FBXL14	0.074531	0.07459	-0.33237	-0.28058	-0.16185	-0.25218	-0.08086	-0.05491
FBXL15	0.109731	0.616735	0.112179	0.014405	0.169813	-0.28527	0.268417	0.265257
FBXL16	0.140442	-0.2126	-0.27878	0.382975	0.046267	0.101376	0.269571	0.195667
FBXL17	0.064771	-0.3438	0.050886	-0.1021	0.074209	0.216794	-0.06933	0.082798
FBXL18	0.399304	-0.13053	0.013403	-0.25476	0.182901	0.147127	-0.07116	-0.08637
FBXL19	0.248164	0.536059	0.123845	0.260095	0.117769	-0.22344	NA	NA
FBXL2	0.05842	-0.21502	-0.15224	-0.1461	-0.18039	-0.08809	0.159863	-0.2373
FBXL20	0.398469	-0.21056	-0.01277	0.243202	0.024918	-0.30532	0.040161	-0.15118
FBXL21	-0.11317	-0.20078	0.081935	0.379759	0.398438	-0.04896	-0.36157	-0.42446
FBXL22	0.260027	0.244658	0.019603	-0.22272	-0.04438	-0.01657	-0.19445	-0.22006
FBXL3	0.141871	-0.66997	-0.1385	-0.11697	0.200715	-0.13006	-0.52957	-0.19929
FBXL4	0.168716	0.036767	-0.14393	-0.20899	-0.09038	-0.06669	-0.0046	0.21175
FBXL5	0.069961	0.267752	0.075759	-0.509	-0.28199	-0.07232	-0.19897	0.051821
FBXL6	0.087511	0.59447	0.439683	0.06151	0.249795	-0.13472	0.544088	0.423067
FBXL7	-0.34297	-0.29228	-0.04457	-0.07185	-0.03022	0.146477	-0.45493	-0.11008
FBXL8	-0.0933	0.035425	0.088076	-0.11852	0.196206	-0.1288	0.266999	0.104639
FBXO10	NA	NA	-0.29846	0.299643	-0.17327	0.112692	-0.04855	0.114368
FBXO11	0.286699	-0.64599	-0.26696	-0.18696	-0.05612	0.29309	0.126617	0.117137
FBXO15	-0.23456	0.679347	-0.15082	0.300976	-0.18032	0.181587	0.042174	0.422874
FBXO16	0.214025	0.15048	0.174919	0.078116	-0.35594	-0.4256	0.142458	-0.2654

FBXO17	-0.01347	-0.27888	0.074164	-0.14778	-0.06908	0.061731	-0.05896	-0.10014
FBXO18	0.018128	0.710949	0.076155	0.270239	-0.0116	-0.12712	-0.54646	-0.50416
FBXO2	0.140101	-0.18628	0.339798	0.33474	0.305066	0.339119	0.202083	-0.29494
FBXO21	-0.13715	-0.17532	-0.19202	-0.01299	0.466538	0.26117	-0.02909	-0.49895
FBXO22	0.183243	-0.42726	-0.10847	-0.16933	-0.28258	-0.52096	0.009904	0.176086
FBXO24	-0.04017	0.377911	-0.27762	0.1235	-0.19929	-0.09044	-0.04943	-0.16795
FBXO25	0.217196	0.377265	0.114791	-0.15627	-0.1685	-0.37826	-0.58416	-0.52982
FBXO27	0.072244	0.10257	0.157047	-0.13014	-0.0601	-0.10453	-0.11588	-0.07013
FBXO28	-0.31848	-0.44456	-0.20071	-0.39385	-0.35668	-0.41754	0.176064	0.135081
FBXO3	0.059088	-0.19432	-0.23745	-0.45719	-0.2737	-0.52766	-0.02905	-0.06898
FBXO30	0.313711	-0.37585	-0.37992	-0.34505	0.291988	0.028421	-0.23214	-0.17186
FBXO31	0.143664	0.143966	-0.29807	-0.12799	0.387823	-0.20513	0.254373	0.093399
FBXO32	-0.2158	-0.32329	0.157279	-0.30069	-0.16175	-0.02222	-0.12638	-0.48616
FBXO33	-0.06536	-0.10685	-0.27147	-0.16314	-0.18619	-0.15033	-0.36833	-0.11214
FBXO34	0.007721	-0.1311	-0.35625	-0.15608	-0.26522	-0.52644	0.109186	0.025009
FBXO36	0.0447	-0.63169	-0.25705	-0.34228	-0.0766	-0.38037	-0.5514	-0.36423
FBXO38	0.153615	0.683367	0.065924	-0.06508	-0.12505	-0.11046	-0.41927	-0.1805
FBXO39	0.133041	-0.25117	-0.09507	-0.10719	-0.14608	0.161046	0.217081	0.187171
FBXO4	-0.05868	0.334094	-0.01189	0.040346	-0.08349	-0.41188	0.366425	0.231323
FBXO40	0.250603	0.455176	0.050251	0.057113	0.292022	0.091724	-0.47636	-0.2637
FBXO41	NA	NA	-0.19652	0.639934	0.071031	0.514253	NA	NA
FBXO42	-0.12462	0.017954	-0.42736	0.142459	-0.06774	-0.13701	-0.08111	-0.00282
FBXO43	-0.11334	0.714798	NA	NA	NA	NA	0.34272	0.088237
FBXO44	-0.23826	0.285652	0.461007	0.336785	0.246764	0.197077	0.363304	0.617961
FBXO45	NA	NA	-0.38436	0.190482	0.067244	-0.01702	NA	NA
FBXO46	NA	NA	-0.00042	-0.05475	0.159613	0.062281	0.499206	0.261416
FBXO47	0.064762	0.170839	NA	NA	NA	NA	-0.04923	0.127164
FBXO5	0.171146	0.414942	-0.45532	0.187205	-0.02807	-0.21373	0.211476	0.050802
FBXO6	-0.47829	0.242645	0.256587	0.031492	0.175386	0.182	-0.44708	-0.07161
FBXO7	0.216644	0.027962	0.218238	0.202623	0.134879	0.21964	0.416706	0.335981
FBXO8	-0.08736	-0.42404	-0.20194	-0.42697	-0.25784	-0.64667	-0.42891	-0.45665
FBXO9	-0.02861	-0.18003	-0.29849	-0.35285	-0.09852	-0.22339	-0.60085	-0.35491
FBXW10	-0.02704	0.700255	-0.21353	0.289107	-0.06424	-0.12345	0.084201	-0.04244
FBXW11	0.197436	-0.56617	0.269077	-0.43638	-0.08783	0.024748	0.158278	-0.05234
FBXW12	0.183937	0.580461	NA	NA	NA	NA	-0.15629	0.157244
FBXW2	0.240854	0.588622	0.180909	-0.1124	-0.11597	-0.2396	0.370395	0.557926
FBXW4	-0.27145	0.138091	0.269315	-0.01879	0.370392	0.062893	-0.25781	-0.29802
FBXW4P1	NA	NA	0.13271	0.125134	0.470617	0.083448	NA	NA
FBXW5	-0.14144	0.796605	0.178523	-0.13379	0.151194	-0.16207	-0.24114	0.077219
FBXW7	0.205638	0.226865	-0.24346	-0.03272	0.0092	-0.00371	-0.10448	-0.03132
FBXW8	0.237515	0.643192	-0.20808	0.058553	0.350652	-0.03189	0.160466	-0.04025
FBXW9	0.454995	0.521118	0.08835	0.109581	0.190698	0.190049	0.079881	-0.14316
FCAMR	0.143195	-0.0052	-0.17929	0.75957	-0.06794	0.238989	-0.09116	-0.13956
FCAR	-0.29798	0.55869	0.125448	-0.03457	-0.00304	0.082496	-0.58204	-0.31758
FCER1A	-0.26185	-0.30301	0.103166	-0.26992	-0.02741	-0.37182	NA	NA
FCER1G	-0.29711	-0.04552	0.247645	-0.36301	-0.41973	0.021239	-0.33297	0.332522
FCER2	0.013423	0.769378	0.042704	0.618743	0.047903	0.120494	-0.04185	0.37794
FCF1	0.276838	0.767252	-0.21675	-0.34588	0.151858	0.214573	-0.53941	-0.27221
FCGBP	NA	NA	-0.12492	0.009565	0.03176	0.161217	0.00212	0.046945
FCGR1A	NA	NA	NA	NA	NA	NA	-0.55134	0.171557
FCGR1B	NA	NA	0.096507	-0.15141	-0.35818	0.048053	-0.32537	0.278929
FCGR2A	-0.5556	-0.29025	0.052199	-0.07726	-0.23049	-0.05667	-0.48477	0.19702

FCGR2B	-0.48689	-0.41658	0.249254	-0.00186	-0.27758	0.02828	-0.45533	0.020391
FCGR3A	0.323791	-0.02622	0.174977	-0.07822	-0.02174	0.034373	-0.64139	-0.11548
FCGR3B	-0.62798	0.127367	0.164034	-0.02187	-0.06308	-0.09523	-0.1607	0.180605
FCGRT	0.204676	0.057728	0.057361	-0.11647	0.140033	0.216184	0.381637	0.381784
FCHO1	0.294952	0.583391	0.055205	0.621039	0.193864	0.392301	-0.15553	0.484377
FCHO2	-0.22991	-0.54186	0.059359	-0.25044	-0.31911	0.209072	-0.33238	-0.35633
FCHSD1	-0.02263	0.271036	0.198587	0.121517	0.358186	-0.18686	0.293903	0.427651
FCHSD2	-0.14758	0.100337	0.210702	0.445451	0.132223	0.566902	-0.58893	-0.44821
FCN1	-0.24211	0.040971	0.150139	0.180778	-0.02895	0.001904	-0.5474	0.175266
FCN2	0.214113	0.56266	0.082078	0.042407	0.001507	0.052894	0.186025	0.395425
FCN3	-0.30462	0.00509	0.114708	0.404122	-0.08218	-0.26672	-0.38956	0.0852
FCRL1	0.373533	0.817754	0.108823	0.714372	0.012562	0.67308	0.257083	0.127466
FCRL2	-0.38574	0.380495	0.023027	0.755333	-0.22299	0.397079	0.038784	0.22666
FCRL3	-0.12038	0.551066	-0.1436	0.69066	-0.12417	0.617109	-0.29878	0.265498
FCRL4	0.160193	0.226908	0.02971	0.629061	-0.03816	0.484748	-0.31276	0.037745
FCRL5	NA	NA	0.104979	0.513133	-0.06656	0.380986	-0.11085	0.409991
FCRL6	-0.011	0.379518	NA	NA	NA	NA	-0.24697	0.2928
FCRLA	-0.18236	0.351871	0.140113	0.775167	-0.18754	0.624604	NA	NA
FCRLB	0.085912	-0.2665	-0.01786	0.235646	-0.24297	0.211437	NA	NA
FDFT1	0.027288	-0.11051	0.0032	0.00879	-0.21946	-0.02877	0.085026	0.017136
FDPS	0.043666	0.656699	0.023185	0.154274	-0.18431	0.090259	NA	NA
FDX1	-0.01969	-0.24455	0.054832	-0.22587	-0.34342	-0.37904	0.025498	0.165642
FDX1L	NA	NA	-0.33201	0.185255	-0.00661	-0.40064	NA	NA
FDXACB1	NA	NA	-0.06197	0.155419	-0.12993	-0.15791	NA	NA
FDXR	-0.01657	0.617737	0.305495	0.103773	-0.00602	-0.35161	0.178643	0.033547
FECH	-0.16118	0.327748	-0.01999	-0.14002	-0.17806	-0.42426	-0.2466	-0.08389
FEM1A	0.132483	0.451572	-0.19849	0.337418	0.287689	0.171418	0.410321	0.284239
FEM1B	-0.31933	-0.25608	-0.23804	-0.34012	-0.23095	-0.57353	0.114217	0.196511
FEM1C	-0.09282	0.199878	-0.20307	-0.32536	-0.17985	-0.26316	-0.06962	-0.17996
FEN1	0.162079	0.74263	-0.2137	0.319122	0.330879	0.060742	0.406258	0.190634
FER	-0.17378	-0.10589	-0.22942	-0.17566	-0.37148	0.038871	-0.14753	-0.23729
FER1L4	NA	NA	-0.15	0.347891	-0.03149	-0.08146	0.101435	0.076652
FER1L5	NA	NA	0.098257	-0.02754	0.025953	-0.14299	NA	NA
FERD3L	0.039493	0.686523	-0.00655	-0.12775	-0.16909	0.187462	0.401326	-0.15305
FERMT1	NA	NA	-0.17319	-0.05404	0.164012	0.253222	NA	NA
FERMT2	NA	NA	-0.04968	-0.07574	-0.19241	0.152873	NA	NA
FERMT3	NA	NA	0.301111	0.536984	-0.08429	0.589705	NA	NA
FES	-0.12002	0.37793	0.136605	0.15257	0.170872	0.288995	-0.28958	0.404505
FETUB	0.351864	0.380703	-0.06115	-0.24733	-0.21724	-0.13168	0.551083	-0.07402
FEV	0.021172	0.639327	-0.00751	0.128733	-0.15088	-0.05785	0.409692	0.374069
FEZ1	-0.25468	-0.24998	0.418401	0.06808	0.025089	0.027053	-0.23871	-0.12336
FEZ2	0.186246	-0.51538	-0.24375	-0.34587	-0.02642	-0.00139	0.090084	0.230519
FEZF1	0.229925	0.092093	NA	NA	NA	NA	NA	NA
FEZF2	0.301979	0.391579	-0.16871	0.125651	0.253188	0.256072	NA	NA
FFAR1	-0.05613	0.11389	-0.06943	0.026428	-0.23442	0.087957	0.265284	0.475329
FFAR2	0.030856	0.094345	0.158652	0.141634	0.089929	-0.17478	-0.15484	0.677555
FFAR3	-0.12048	0.560741	-0.00566	-0.34933	0.198431	-0.00613	0.452123	0.597341
FGA	0.464556	0.596421	0.057373	-0.20855	0.149056	-0.08525	-0.06597	-0.4715
FGB	0.198897	-0.06952	0.18865	0.125544	0.068243	0.182997	-0.10842	-0.06164
FGD1	0.34284	0.11706	0.101861	-0.19749	0.258619	-0.16835	-0.13506	-0.03773
FGD2	-0.00402	0.126931	0.286286	0.572975	0.038012	0.429988	0.087938	0.028691
FGD3	-0.34468	0.342471	0.383782	0.624385	-0.23838	0.655228	-0.46332	0.086452

FGD4	-0.50929	-0.19424	-0.19045	-0.18914	-0.1652	-0.07318	0.240038	0.142176
FGD5	-0.28506	-0.19658	0.428208	0.182414	0.004482	0.301859	-0.30145	0.200716
FGD6	-0.11191	-0.27952	-0.19329	-0.26124	-0.11204	-0.10242	0.204948	0.262859
FGF1	0.227154	0.107784	-0.05601	-0.22691	-0.18401	-0.12863	0.005633	-0.18642
FGF10	-0.04479	0.094393	-0.10848	-0.23237	0.131795	-0.20156	-0.40211	0.084418
FGF11	-0.37117	-0.0905	0.152005	0.127902	0.385538	-0.01832	-0.42397	0.071777
FGF12	0.065525	0.275808	-0.08856	-0.15647	0.073149	-0.15298	-0.25123	-0.35279
FGF13	0.328188	-0.35429	-0.29437	-0.13833	0.089424	-0.44534	-0.29807	-0.00165
FGF14	0.333685	0.387801	-0.09666	0.153946	0.010494	-0.25387	-0.55934	-0.05739
FGF16	-0.03636	0.287287	0.139857	-0.06468	-0.01995	-0.1302	0.062616	0.16274
FGF17	0.363382	0.565598	0.018532	-0.06048	0.074463	-0.03108	0.229282	0.178309
FGF18	0.340629	0.789004	0.544906	0.035883	-0.04058	-0.01356	0.419507	0.251552
FGF19	0.118774	0.761482	0.33893	0.150719	-0.09591	-0.07056	0.576696	0.530234
FGF2	0.316456	-0.33494	0.092691	-0.1365	0.145163	0.372596	-0.43287	-0.30666
FGF20	0.334078	0.69817	0.462314	0.168245	-0.24938	-0.16626	0.225499	-0.3367
FGF21	-0.00637	0.386909	0.014871	-0.31012	0.140757	-0.3528	-0.44132	-0.19055
FGF22	-0.0149	0.504072	-0.13183	-0.14594	0.014634	-0.16942	-0.21264	-0.01036
FGF23	-0.1258	0.560468	-0.31659	-0.12422	-0.20863	-0.04962	0.668743	0.357505
FGF3	0.022835	0.264589	0.274168	-0.04316	0.11935	0.086403	0.313818	-0.10892
FGF4	-0.03504	0.268094	0.098491	-0.40616	-0.04176	0.039147	0.21078	0.490804
FGF5	-0.4344	-0.14954	-0.24945	0.048986	0.232611	-0.20652	0.110773	0.056663
FGF6	0.16876	-0.0615	0.038157	0.083699	-0.03099	-0.07068	-0.1576	0.134128
FGF7	-0.15469	-0.25888	-0.10011	-0.10053	-0.07524	0.20087	-0.4966	-0.1518
FGF8	0.2488	0.141542	0.114078	-0.51915	0.192643	-0.04943	0.044328	0.327796
FGF9	-0.10312	0.099341	0.333109	-0.23474	-0.07844	-0.05228	0.292002	0.228898
FGFBP1	-0.024	0.215495	-0.40919	0.006981	-0.33895	0.040627	-0.06551	-0.02438
FGFBP2	-0.39399	-0.41456	-0.17309	-0.183	-0.22297	-0.01202	NA	NA
FGFBP3	0.110884	0.502369	-0.23398	0.460566	-0.03769	-0.39218	NA	NA
FGFR1	0.194676	0.197017	0.236133	0.110856	-0.0482	0.420026	-0.23158	-0.11125
FGFR1OP	0.038943	-0.47017	-0.43953	0.0015	-0.11015	-0.6657	0.570003	0.399849
FGFR1OP2	0.089058	-0.16706	-0.29155	-0.25964	-0.40606	-0.33344	-0.6336	-0.2939
FGFR2	-0.17895	0.327065	-0.2539	-0.36787	0.1833	-0.34546	0.049484	-0.27805
FGFR3	-0.0639	0.252152	-0.06282	0.095295	0.212331	-0.03115	0.359723	0.031215
FGFR4	0.389717	0.085094	0.360651	0.201691	0.391422	0.090533	0.683955	0.3639
FGFRL1	0.124268	0.25682	0.182215	0.086135	0.489601	-0.1391	0.485352	0.335372
FGG	0.260848	0.748201	0.054547	-0.05906	-0.18704	-0.03943	-0.13698	-0.12624
FGGY	NA	NA	0.149418	-0.0866	0.18531	-0.22747	NA	NA
FGL1	-0.09633	-0.47352	-0.06719	0.207724	-0.181	-0.07111	0.137091	0.416592
FGL2	-0.18918	-0.06353	0.039975	-0.41056	-0.36764	0.065325	-0.46698	0.186556
FGR	-0.48167	0.024585	0.262293	0.348371	0.052855	0.284846	-0.34085	0.040926
FH	-0.1078	-0.01123	0.101824	-0.04251	-0.06803	-0.09073	-0.27144	-0.37932
FHAD1	NA	NA	-0.21197	-0.06162	-0.32258	-0.10593	NA	NA
FHDC1	NA	NA	-0.20304	-0.20402	0.065022	-0.07953	NA	NA
FHIT	0.452955	0.684924	-0.06012	0.010461	-0.15463	0.176871	0.059777	-0.35719
FHL1	0.003322	-0.26843	0.237503	-0.01299	0.070294	0.066112	-0.28905	-0.28694
FHL2	-0.30192	-0.16444	-0.20599	-0.2921	0.371729	0.104706	-0.07095	0.433381
FHL3	-0.05057	-0.01455	0.275491	-0.09833	0.052409	0.220231	-0.11936	-0.26242
FHL5	0.099607	-0.02623	0.002168	-0.00181	-0.38595	-0.20541	-0.2448	-0.08385
FHOD1	-0.35597	-0.20676	0.057689	0.222887	0.227553	0.492844	0.287324	0.436058
FHOD3	0.232558	-0.11417	0.526698	0.120077	0.237847	0.218622	-0.09554	-0.16423
FIBCD1	0.114449	0.592856	0.133185	0.078459	0.27168	-0.45254	0.086534	0.001171
FIBIN	NA	NA	0.05335	-0.08785	0.077106	-0.22078	NA	NA

FIBP	0.112983	0.671862	0.163138	0.168898	-0.00732	-0.18955	-0.01492	-0.02949
FICD	NA	NA	0.367967	0.166352	0.037105	-0.15465	NA	NA
FIG4	NA	NA	-0.09805	-0.1536	-0.04419	-0.25632	NA	NA
FIGF	-0.24812	-0.31585	-0.12516	-0.08365	-0.11002	-0.24016	-0.10092	-0.0413
FIGLA	0.3416	0.10436	NA	NA	NA	NA	-0.10158	-0.17893
FIGN	-0.07163	0.128029	-0.31506	-0.16716	-0.16678	-0.02044	-0.17144	-0.40556
FIGNL1	-0.08059	-0.42869	-0.47435	0.034658	0.026174	0.030324	0.212147	0.222505
FILIP1	-0.39885	-0.06247	-0.04229	-0.1317	-0.01827	0.019005	-0.28455	-0.16578
FILIP1L	-0.12376	-0.01072	0.294014	-0.13171	-0.02541	0.505161	-0.20404	-0.0442
FIP1L1	-0.32273	-0.58947	-0.15506	0.303061	0.142685	0.151932	0.159707	0.08847
FIS1	0.430001	0.051628	0.280027	-0.03799	-0.13436	-0.15617	-0.18042	-0.289
FIZ1	0.287107	0.463439	-0.01044	0.313984	0.216135	0.211383	NA	NA
FJX1	0.194883	-0.22529	-0.07587	0.259215	0.023296	-0.12899	0.042816	0.109897
FKBP10	0.056866	-0.10876	-0.04504	0.120776	-0.13301	0.093431	-0.20753	-0.04619
FKBP11	-0.20383	0.152275	0.133537	0.434899	-0.02078	-0.39028	0.197059	0.080385
FKBP14	-0.30187	-0.10339	0.023244	-0.12117	0.045917	0.088012	-0.51375	-0.35852
FKBP15	NA	NA	0.242172	0.256172	0.086712	0.350435	NA	NA
FKBP1A	0.21339	-0.36666	-0.29648	0.047617	-0.26077	-0.38169	0.642612	0.24523
FKBP1B	NA	NA	0.016078	-0.12353	-0.05225	-0.09301	-0.44673	-0.19142
FKBP2	-0.30343	0.023372	0.101166	0.079124	-0.2064	-0.52086	-0.2806	-0.13864
FKBP3	0.115159	-0.4498	-0.43663	-0.11431	-0.22009	-0.32298	-0.42969	-0.3912
FKBP4	0.153573	0.685983	-0.02306	0.369938	0.170846	-0.05992	0.197468	-0.11327
FKBP5	-0.09654	-0.07951	-0.17913	0.111837	-0.11734	-0.25925	-0.03082	-0.2016
FKBP6	0.096541	0.100749	-0.12111	-0.07566	-0.1883	0.016748	0.471889	0.310375
FKBP7	-0.39588	-0.02379	-0.15334	-0.10307	-0.35878	-0.41645	0.210269	0.457803
FKBP8	0.317893	0.726464	0.201897	-0.09692	0.1471	-0.28711	0.195113	-0.10717
FKBP9	-0.03087	-0.1693	0.220903	-0.02604	0.322242	-0.02121	0.616574	0.136183
FKBP9L	0.021437	0.265518	0.268737	0.096925	0.366123	-0.22469	0.405306	0.09582
FKBPL	0.189729	-0.0979	0.066041	-0.03963	-0.05454	-0.3983	0.541791	0.288166
FKRP	0.445953	0.201804	0.319521	0.074396	0.389451	0.172549	0.050679	0.061703
FKSG2	0.168981	-0.14286	-0.2021	0.238817	-0.11198	-0.15569	0.012502	-0.11129
FKSG24	-0.23961	-0.59032	0.069018	0.23271	-0.05643	-0.20226	-0.20981	0.105483
FKSG29	NA	NA	0.270488	-0.37966	-0.16477	0.315649	NA	NA
FKSG83	-0.06031	0.096627	-0.07828	-0.31368	0.21668	-0.12693	NA	NA
FKTN	NA	NA	-0.03456	-0.17263	0.057663	-0.12298	NA	NA
FLAD1	0.322018	0.55711	0.090403	0.120579	-0.07399	-0.23532	0.271594	-0.0846
FLCN	-0.28121	-0.34219	0.209177	-0.07317	0.055009	-0.27754	0.419815	0.590409
FLG	NA	NA	0.141502	-0.03158	-0.0588	0.262712	0.106264	0.126816
FLG2	0.273297	0.252002	NA	NA	NA	NA	-0.16286	0.02915
FLI1	-0.39361	-0.27784	0.056159	0.548312	-0.28473	0.377552	-0.6119	-0.33316
FLII	-0.0234	0.11638	0.382761	0.194314	0.093988	0.512875	0.128222	-0.03454
FLJ10213	0.014434	0.552861	-0.03583	-0.22314	0.239921	0.118534	-0.04381	-0.35645
FLJ10357	0.033058	0.140631	-0.08821	-0.03248	0.127474	0.013197	0.248054	0.208297
FLJ10490	0.167698	-0.099	0.050563	0.099572	0.014792	0.070697	0.466017	0.581233
FLJ10661	NA	NA	NA	NA	NA	NA	0.487083	0.33502
FLJ11151	0.248143	-0.1068	-0.19903	-0.27014	0.178032	-0.51584	-0.43098	-0.06911
FLJ11235	NA	NA	-0.0352	-0.11669	-0.36241	-0.36153	NA	NA
FLJ12331	-0.10323	-0.44819	0.26556	0.262069	0.332405	0.171281	-0.15591	0.031701
FLJ12529	0.171993	-0.11118	-0.12629	0.185057	0.139509	0.148295	-0.1973	-0.17232
FLJ14107	-0.0034	-0.2027	0.241013	-0.11046	0.174596	-0.08574	0.3373	0.149515
FLJ14213	0.200223	0.058881	-0.19416	-0.02492	0.053777	-0.21336	-0.08707	-0.10009
FLJ14816	0.212463	0.525313	0.136576	-0.11995	0.266624	0.04386	0.469541	0.276804

FLJ16124	0.298016	0.25107	NA	NA	NA	NA	-0.02059	-0.59929
FLJ16165	0.006993	0.377403	0.186679	-0.27278	-0.05164	-0.24904	-0.38686	0.19528
FLJ20184	0.325761	0.455399	-0.14723	0.1222	-0.21217	-0.27868	0.161206	-0.0645
FLJ20323	0.07801	-0.28963	-0.2167	-0.27346	0.035494	0.146199	0.09801	0.088484
FLJ20433	-0.43583	0.678115	0.137736	-0.35248	0.242827	0.291265	-0.10528	0.249165
FLJ20674	0.23182	0.030876	-0.0695	-0.22833	0.347913	-0.59144	0.513853	0.312237
FLJ21075	0.264676	-0.00831	0.154978	-0.11045	-0.02091	0.146335	-0.32603	0.505517
FLJ21511	0.185504	-0.23372	-0.20767	-0.20693	-0.26647	-0.06014	0.09397	-0.13046
FLJ21865	0.07562	-0.05634	0.288644	0.093077	0.432037	0.439427	0.411021	0.53138
FLJ22167	0.013391	-0.26104	-0.12062	-0.20011	0.257295	-0.18213	0.299042	0.324541
FLJ22184	NA	NA	0.081661	-0.03038	-0.06881	0.237023	0.524728	-0.12108
FLJ22374	0.481883	0.282273	-0.15407	0.104379	0.108515	-0.11681	-0.22066	-0.38416
FLJ22662	0.091351	-0.21527	-0.18398	0.138848	-0.29128	-0.31943	0.013735	0.297764
FLJ23834	0.116758	-0.13293	-0.45782	-0.11872	-0.02533	-0.36819	0.36191	0.048811
FLJ25006	0.230259	0.540489	-0.15447	0.092695	-0.05463	0.468793	0.199495	0.613121
FLJ25328	0.137111	0.442091	-0.09603	-0.0878	0.180249	0.071534	-0.11516	0.115395
FLJ25758	-0.14241	0.551367	-0.36544	-0.05949	-0.18171	0.113185	NA	NA
FLJ25770	0.364925	0.548241	0.090137	-0.17974	0.064046	-0.15955	-0.16247	-0.11791
FLJ25996	-0.36618	0.205175	NA	NA	NA	NA	0.061425	0.34126
FLJ26850	0.22919	0.286127	NA	NA	NA	NA	-0.51677	-0.54595
FLJ32063	NA	NA	-0.51438	-0.03874	-0.05595	-0.05731	0.269534	-0.05406
FLJ32679	0.55136	0.004265	-0.01843	0.417688	0.033842	0.390495	-0.29255	-0.36673
FLJ33360	0.091064	0.501946	0.21179	0.053853	0.135807	0.144679	-0.30092	-0.40668
FLJ33534	0.118509	0.001683	0.147092	0.518783	0.122884	0.350995	NA	NA
FLJ33590	-0.1038	0.274691	0.034898	-0.21455	0.243669	0.047845	-0.47506	-0.12918
FLJ34503	-0.04307	-0.15049	NA	NA	NA	NA	0.172447	0.130051
FLJ35220	-0.30343	0.324398	-0.21068	-0.01931	-0.16661	-0.04819	-0.27658	-0.05536
FLJ35409	0.183037	0.198266	0.066498	-0.02044	0.22646	-0.15194	0.404796	0.292693
FLJ35767	0.039992	0.572768	-0.28005	0.037968	-0.1271	-0.04322	-0.01511	-0.39769
FLJ36031	0.198729	-0.10848	0.165299	-0.02497	0.292586	0.063154	-0.09968	0.047539
FLJ36070	0.362529	0.190893	-0.00847	-0.34814	-0.13987	-0.16235	0.244283	-0.39901
FLJ36208	0.056567	-0.28412	0.271659	0.21512	-0.35684	0.013207	0.057523	-0.09336
FLJ37078	-0.0803	0.560458	0.200969	0.031547	-0.01481	-0.32395	0.264921	0.055088
FLJ37464	-0.10291	0.692501	0.074526	0.408501	0.16214	-0.27987	-0.22741	0.106557
FLJ37543	0.11295	0.741516	-0.04123	-0.06886	0.079587	0.056455	NA	NA
FLJ38723	0.204683	0.625284	0.108501	0.03726	0.305081	0.16357	-0.12279	-0.18678
FLJ39653	-0.15728	-0.57542	-0.01355	-0.1882	0.201198	0.095119	NA	NA
FLJ40113	-0.17949	-0.24252	0.069341	0.115114	0.210708	0.128155	-0.20599	-0.21312
FLJ40125	-0.31395	-0.0999	-0.30359	0.459406	-0.0157	0.209722	0.224294	0.379265
FLJ40142	0.056207	0.285923	-0.16003	-0.16866	0.135431	-0.1349	0.109101	0.226907
FLJ40235	0.084102	0.365392	0.072993	0.023332	0.161615	-0.0492	-0.08976	-0.23035
FLJ40288	-0.02987	0.653263	NA	NA	NA	NA	-0.02764	0.17468
FLJ40448	-0.03268	-0.09857	NA	NA	NA	NA	-0.54832	-0.32032
FLJ40504	-0.03115	0.012924	0.057665	0.245944	0.340759	0.134151	0.112386	-0.24259
FLJ40852	-0.0827	-0.48012	-0.21445	-0.20687	-0.1122	0.144022	0.228776	0.33359
FLJ41423	0.112333	0.322662	NA	NA	NA	NA	-0.24375	-0.58508
FLJ41603	-0.17288	0.087745	0.624145	-0.15941	0.089481	-0.28041	-0.29017	-0.17901
FLJ41649	0.101604	0.608529	NA	NA	NA	NA	-0.2273	-0.30504
FLJ42102	0.127283	0.490488	NA	NA	NA	NA	0.108642	0.053197
FLJ42280	0.194305	0.331704	NA	NA	NA	NA	0.168865	-0.27344
FLJ42289	-0.0846	-0.30069	NA	NA	NA	NA	-0.18779	0.048141
FLJ42393	-0.13175	-0.06116	0.27951	-0.06734	0.130098	-0.03322	NA	NA

FLJ42709	NA	NA	-0.01392	-0.16663	-0.22455	-0.16713	NA	NA
FLJ43093	0.106315	0.55986	-0.05597	-0.04404	-0.07292	-0.15217	-0.22594	0.227115
FLJ43276	0.06296	0.096292	-0.1153	-0.16636	0.04094	0.227921	-0.49578	-0.22449
FLJ43692	0.51263	-0.67945	-0.02845	-0.06097	-0.12434	-0.07132	0.453848	0.207173
FLJ43860	0.094702	0.46488	-0.21255	-0.19811	0.139168	0.249716	0.101269	0.622212
FLJ44048	-0.13194	0.089761	-0.06113	0.030242	0.001294	0.009457	-0.17664	-0.04402
FLJ44635	-0.30239	0.444071	NA	NA	NA	NA	0.215894	0.18336
FLJ44653	-0.01517	0.500588	NA	NA	NA	NA	0.148945	-0.36263
FLJ45079	-0.02068	0.34066	NA	NA	NA	NA	-0.08011	0.205452
FLJ45422	0.101473	0.310631	0.39529	0.231777	0.263535	-0.11854	-0.13679	-0.11927
FLJ45445	NA	NA	NA	NA	NA	NA	-0.31204	-0.40944
FLJ45717	-0.02368	0.010804	-0.02113	-0.02813	-0.10679	0.264805	0.045274	-0.10328
FLJ45803	0.071538	-0.14628	0.077117	-0.01827	-0.4632	-0.06249	-0.24321	0.097291
FLJ45974	-0.1243	0.466084	NA	NA	NA	NA	0.198194	-0.0026
FLJ45983	0.287159	0.639251	NA	NA	NA	NA	-0.28904	-0.08481
FLJ46257	0.068589	0.826165	NA	NA	NA	NA	0.100285	-0.00237
FLJ46321	0.096043	0.189958	NA	NA	NA	NA	0.078196	-0.05711
FLJ90757	-0.16594	0.614007	NA	NA	NA	NA	0.195903	-0.22015
FLNA	-0.07106	-0.0824	0.058933	0.008746	0.086545	0.345307	-0.48524	-0.4768
FLNB	-0.25413	-0.20252	-0.13896	-0.30076	0.173011	0.396037	0.390135	0.346414
FLNC	-0.11077	-0.09346	0.293776	-0.06719	0.082897	0.15618	-0.53313	-0.4612
FLOT1	0.173909	0.231181	0.392732	0.161715	0.130273	-0.08585	0.334467	0.213821
FLOT2	0.113749	0.617732	0.46014	0.221021	0.147648	0.276059	0.063107	-0.24121
FLRT1	0.291275	-0.17496	-0.20479	-0.09442	0.148373	-0.01009	-0.09812	-0.1407
FLRT2	-0.15344	-0.40042	0.104104	0.053447	-0.14278	0.209959	-0.36369	0.035871
FLRT3	0.064425	-0.00376	-0.1462	-0.33157	-0.19303	-0.26323	0.220202	-0.07573
FLT1	-0.4851	-0.44577	-0.26445	0.141415	-0.06052	0.272346	-0.07907	-0.38978
FLT3	-0.21524	0.493314	-0.01856	0.301889	0.031935	0.337163	-0.36855	0.010262
FLT3LG	-0.09298	0.502141	0.224607	0.392851	-0.19032	0.459473	-0.2615	0.291053
FLT4	-0.22707	0.327394	0.139387	0.003601	0.121363	0.201625	-0.2804	-0.11406
FLVCR1	NA	NA	-0.21586	-0.10411	0.045186	-0.25184	NA	NA
FLVCR2	NA	NA	-0.05493	-0.4818	-0.21229	-0.22596	NA	NA
FLYWCH1	0.036442	0.29896	0.43538	-0.19413	0.296311	0.28509	-0.37031	-0.33124
FLYWCH2	NA	NA	-0.09918	0.009702	0.124496	-0.28125	NA	NA
FMN1	NA	NA	0.131564	-0.24521	-0.13935	-0.17243	NA	NA
FMN2	-0.07491	-0.22756	0.095879	-0.17225	-0.06894	-0.18713	-0.12458	-0.27315
FMNL1	-0.63293	0.649437	0.457769	0.537705	0.097989	0.659958	-0.29349	0.159807
FMNL2	0.079764	-0.36292	-0.15349	-0.01617	0.044255	0.398235	0.025767	-0.04301
FMNL3	0.019608	-0.2445	0.40871	0.391759	-0.1488	0.331494	0.07831	0.251426
FMO1	0.136	-0.12917	0.059658	0.167494	-0.20613	0.007921	-0.08123	0.516365
FMO2	-0.25197	0.08409	0.295387	-0.03159	-0.07458	-0.2281	-0.35562	0.105342
FMO3	-0.51926	-0.35462	-0.05953	-0.07329	-0.12188	-0.0693	0.061121	0.074872
FMO4	0.27208	0.062453	0.082171	-0.44383	-0.08771	-0.51183	0.080782	0.176084
FMO5	-0.01037	-0.06117	-0.06648	-0.33841	0.063392	-0.38879	0.345219	0.334891
FMOD	-0.37206	-0.28634	0.001518	0.214253	-0.13949	-0.05554	-0.07133	-0.16968
FMR1	0.108705	-0.05185	-0.11027	-0.15228	0.176773	-0.12573	0.150272	-0.06732
FMR1NB	-0.11619	0.587553	-0.21139	0.176237	-0.01311	-0.04108	0.366606	0.553361
FN1	-0.03591	-0.30732	0.082108	-0.2191	-0.00065	0.256484	-0.19667	-0.15031
FN3K	0.113897	0.496745	0.208699	-0.11273	0.16801	0.013689	0.581213	0.295668
FN3KRP	-0.25421	-0.07191	0.070053	0.400602	0.04434	0.163866	-0.06761	-0.23934
FNBP1	-0.01932	-0.11535	-0.11839	0.098473	-0.07791	0.350183	-0.55297	-0.39541
FNBP1L	-0.30401	-0.10703	0.23037	-0.18292	0.107677	-0.21752	0.184099	-0.21331

FNBP4	0.238708	0.470498	-0.2016	-0.07722	0.144271	0.1986	0.559701	0.568548
FNDC1	-0.16928	-0.21661	0.108641	-0.11323	-0.02172	0.22114	-0.34596	-0.34294
FNDC3A	0.051828	-0.64023	0.117971	-0.13524	0.183713	0.124995	0.011406	-0.20022
FNDC3B	0.171413	0.686295	0.132176	-0.28827	-0.04486	-0.20382	NA	NA
FNDC4	-0.37612	-0.07196	0.185784	0.109385	0.159099	0.327957	-0.24305	0.128848
FNDC5	-0.02824	-0.08757	-0.1299	-0.07225	-0.03626	0.134398	-0.19537	-0.07468
FNDC7	0.244372	0.229578	-0.10797	-0.21642	-0.02401	-0.15845	0.391839	-0.12795
FNDC8	0.225014	0.187739	-0.01813	0.013582	-0.0703	0.252199	0.057003	0.513942
FNIP1	0.17221	-0.4156	0.148545	-0.19778	-0.27548	0.112415	NA	NA
FNIP2	NA	NA	-0.09101	-0.40649	-0.09643	-0.09954	NA	NA
FNTA	-0.00625	-0.70523	0.331785	-0.20047	0.007462	0.274196	0.431723	0.201642
FNTB	0.176103	0.215999	-0.13243	-0.06647	-0.08234	-0.14152	-0.28125	-0.17865
FOLH1	-0.24074	-0.04142	-0.27083	-0.2404	-0.20748	-0.08301	0.561267	0.534976
FOLR1	0.097406	0.707346	-0.06555	-0.41329	-0.03588	0.032273	0.479082	0.151551
FOLR2	-0.44777	0.002004	0.302373	-0.09521	-0.24643	0.309795	-0.41463	-0.01237
FOLR3	-0.09125	-0.08632	-0.17624	-0.19017	0.09703	-0.03636	-0.16765	-0.0673
FOLR4	NA	NA	0.068395	0.058617	0.203429	-0.41386	NA	NA
FOS	-0.30506	-0.61736	-0.30159	0.0137	-0.04677	-0.19143	-0.09878	-0.13793
FOSB	-0.1101	-0.19772	-0.20467	-0.0489	-0.19265	-0.09948	0.127704	-0.12269
FOSL1	0.150831	0.17482	-0.08579	-0.03105	0.114771	0.22524	0.281177	0.159542
FOSL2	-0.40088	0.419142	0.153665	0.082509	0.085903	0.007526	-0.53609	-0.4374
FOXA1	-0.25324	0.1151	-0.2091	0.165835	-0.09962	-0.27519	0.165367	0.073132
FOXA2	0.078611	0.250971	-0.27339	0.160172	0.324155	-0.04114	0.515983	-0.17219
FOXA3	-0.37895	0.288473	0.001736	0.308297	-0.01008	-0.10436	0.172757	-0.00242
FOXB1	-0.06604	0.096564	0.007578	0.047842	-0.09223	0.299574	0.135081	0.41188
FOXB2	0.220242	0.174741	NA	NA	NA	NA	NA	NA
FOXC1	-0.09554	-0.02325	0.189268	-0.01438	-0.0376	-0.1197	0.022967	-0.07296
FOXC2	-0.0429	-0.26398	-0.11931	-0.03141	-0.17591	-0.02565	-0.39979	-0.33251
FOXD1	-0.06142	0.390833	0.042836	-0.07909	-0.21485	-0.18594	0.001117	0.447935
FOXD2	0.12581	0.15542	0.090155	0.113202	0.314528	-0.04957	0.128179	-0.22503
FOXD3	0.54693	0.548279	0.219117	-0.05987	0.287571	-0.03749	-0.2079	-0.48483
FOXD4	-0.28232	-0.09215	-0.28263	0.134442	-0.20729	0.407681	0.189156	0.035168
FOXD4L1	-0.4913	-0.02075	NA	NA	NA	NA	-0.18305	-0.22802
FOXE1	-0.12482	0.142147	-0.25278	-0.01574	-0.19485	0.149716	0.321132	0.300276
FOXE3	0.416033	0.548129	-0.37749	0.006754	0.271296	-0.11689	-0.29786	-0.25528
FOXF1	0.032338	-0.17602	0.096811	-0.0533	0.023542	0.351638	-0.3762	-0.41554
FOXF2	0.1312	-0.2741	-0.05391	-0.04457	-0.13015	0.242999	0.159626	-0.18177
FOXG1	NA	NA	-0.24174	0.086881	0.113326	-0.14416	NA	NA
FOXH1	0.195253	0.575919	0.029491	-0.14441	0.200057	-0.17798	-0.38225	-0.3128
FOXI1	0.106415	0.502663	-0.07486	-0.239	-0.09138	-0.14103	0.024934	-0.1533
FOXI3	NA	NA	0.108038	-0.16582	-0.11689	0.181143	NA	NA
FOXJ1	-0.21794	0.454973	0.030782	-0.09613	-0.10382	0.134826	-0.35962	-0.5008
FOXJ2	0.194535	0.276972	-0.05507	0.065037	0.383064	0.122893	-0.22974	-0.12601
FOXJ3	-0.28998	-0.50095	0.061352	-0.21047	0.436114	0.274507	0.439919	0.041034
FOXK1	NA	NA	0.051106	-0.11646	0.453408	0.55763	0.080542	0.112306
FOXK2	NA	NA	0.154002	0.006462	0.403445	0.106298	-0.01018	0.055063
FOXL1	-0.16334	0.441688	0.187796	-0.12508	0.221198	-0.06554	-0.14301	-0.31913
FOXL2	-0.01205	-0.1055	-0.23179	-0.18392	-0.00438	-0.39428	-0.23244	0.183649
FOXM1	0.252721	-0.18714	-0.11443	0.33849	0.242026	-0.02829	0.363724	0.219017
FOXN1	-0.01896	0.336397	0.184754	-0.16213	0.135754	0.126212	-0.52827	-0.24412
FOXN2	0.372287	0.195113	0.185023	-0.21401	-0.09673	0.147601	NA	NA
FOXN3	NA	NA	-0.04303	-0.16282	0.157796	0.297549	NA	NA

FOXN4	-0.1528	0.498715	-0.11462	-0.24275	-0.26698	-0.0575	0.159641	0.140862
FOXO1	NA	NA	0.162406	0.169208	0.077986	0.273754	NA	NA
FOXO3	NA	NA	0.274099	0.021168	0.469878	0.388161	NA	NA
FOXO4	NA	NA	0.205505	-0.17872	-0.0766	-0.32494	NA	NA
FOXP1	-0.49462	0.389477	-0.34171	-0.00807	-0.14696	0.3478	-0.58978	-0.42619
FOXP2	0.422709	-0.30358	0.0645	-0.33953	-0.24379	0.114662	-0.34575	-0.3029
FOXP3	0.062451	0.736028	0.108316	0.015815	-0.30183	0.267927	-0.33335	-0.21679
FOXP4	0.17609	0.015908	-0.0376	0.141434	0.296363	0.186741	0.596261	-0.006
FOXQ1	0.145987	-0.01657	0.124759	-0.07699	0.041917	0.16578	0.440431	-0.12095
FOXR1	0.126852	0.603748	-0.00491	0.060042	-0.02318	-0.48352	0.421579	0.032816
FOXR2	0.230636	0.680665	NA	NA	NA	NA	0.429242	0.266717
FOXRED1	0.149458	0.67761	-0.08972	0.155907	0.008092	-0.07924	0.112315	0.133081
FOXRED2	-0.05008	0.296345	-0.09918	-0.01932	0.291922	0.16516	0.083705	-0.44171
FOXS1	NA	NA	0.123339	-0.24401	-0.15794	-0.41921	NA	NA
FPGS	0.194615	0.488689	0.32866	0.124726	0.332568	0.346041	-0.2071	-0.11142
FPGT	-0.09512	-0.42999	0.026948	-0.5247	0.105464	-0.33055	0.171582	0.161113
FPR1	-0.47851	0.123559	0.191077	0.0425	0.024651	-0.12701	-0.31138	0.159893
FPR2	NA	NA	0.054191	0.056749	0.069191	0.038094	NA	NA
FPR3	NA	NA	0.248721	-0.39735	-0.40725	0.199325	NA	NA
FRAG1	-0.11584	0.155501	-0.03129	-0.04145	-0.11388	-0.35783	-0.10769	-0.28537
FRAP1	-0.28362	-0.53977	0.013346	0.359085	0.237414	0.286779	0.31301	0.392974
FRAS1	-0.23131	0.013982	-0.22417	-0.11665	-0.40006	-0.08762	0.408929	-0.14827
FRAT1	-0.06817	-0.17312	0.154942	0.235662	0.183328	-0.15335	0.306118	0.08128
FRAT2	0.358498	0.024772	-0.13368	0.069289	0.170812	-0.17851	0.301481	-0.10199
FREM1	0.176069	-0.03727	0.41538	-0.29713	-0.06816	-0.02032	-0.1928	0.136759
FREM2	NA	NA	NA	NA	NA	NA	-0.03989	-0.29231
FREQ	-0.25283	0.109623	0.162507	-0.06273	0.263058	0.045906	-0.18376	-0.30053
FRG1	-0.0743	-0.15697	-0.14669	-0.06824	0.094924	-0.21478	-0.66168	-0.51935
FRG2	-0.02576	0.243569	-0.26723	-0.08981	0.247289	0.091906	0.38093	0.15336
FRK	0.005874	0.220468	-0.36081	-0.27602	-0.09747	-0.41286	0.073232	-0.00892
FRMD1	-0.14491	0.289736	0.183859	-0.39461	0.080347	-0.22085	0.106842	-0.09418
FRMD3	-0.05602	-0.38132	-0.12051	0.225676	-0.25546	-0.24094	NA	NA
FRMD4A	-0.2603	-0.61528	-0.12856	-0.16263	-0.07726	0.326588	-0.33504	-0.11779
FRMD4B	NA	NA	-0.15138	-0.19019	-0.37529	-0.12013	NA	NA
FRMD5	0.192501	0.477971	0.017788	-0.03704	-0.22556	-0.12828	0.273515	-0.03852
FRMD6	-0.26859	-0.2423	-0.05215	-0.21844	-0.19463	0.103479	-0.09344	-0.04144
FRMD7	-0.30128	-0.19741	-0.04398	-0.22633	0.080866	0.198819	NA	NA
FRMD8	NA	NA	-0.00201	-0.10564	0.130131	0.214761	NA	NA
FRMPD1	0.124799	0.022655	0.329633	-0.0108	-0.16041	0.060762	0.154348	-0.03247
FRMPD2	0.219199	0.673411	0.081158	0.097312	0.005738	-0.06581	0.292856	-0.11262
FRMPD4	-0.15872	-0.03849	0.007153	-0.14516	-0.14224	0.112368	-0.23545	-0.21345
FRRS1	0.307215	0.369459	-0.04371	-0.21292	-0.36731	-0.52473	-0.13356	0.12772
FRS2	0.186479	0.565824	-0.12124	-0.23804	-0.19083	-0.26939	0.377276	0.467096
FRS3	0.160211	0.005092	0.221888	-0.12837	0.225196	-0.24071	-0.26132	-0.23786
FRY	-0.12287	-0.28319	-0.15062	-0.06555	-0.08218	-0.16213	-0.07699	-0.01484
FRYL	NA	NA	-0.18524	-0.2593	-0.07454	-0.02422	0.292996	0.226294
FRZB	-0.15305	-0.00097	-0.17771	-0.05834	-0.14115	-0.07622	-0.29197	-0.53594
FSCB	NA	NA	0.015424	0.144658	-0.18353	-0.05077	NA	NA
FSCN1	-0.51932	-0.21841	-0.15168	0.34927	-0.1505	0.335783	-0.17553	-0.14172
FSCN2	-0.14255	0.322794	0.103893	-0.05756	0.07797	-0.33427	0.266882	0.218343
FSCN3	0.274751	0.631637	0.181104	0.148752	0.095152	0.014268	0.319113	0.004621
FSD1	0.134562	-0.08998	0.038237	0.081687	0.086799	-0.08471	-0.04245	0.218521

FSD1L	0.575275	0.210046	0.161857	-0.14508	0.067965	-0.13391	NA	NA
FSD2	-0.19224	0.253651	-0.03442	-0.22817	0.382799	0.056531	-0.03432	0.24687
FSHB	0.084063	0.56831	0.224593	-0.2741	0.201762	0.151442	0.506328	0.195389
FSHR	0.448798	0.133055	0.027447	-0.2208	-0.13074	0.146656	0.498026	0.432192
FSIP1	0.165956	-0.09127	-0.34613	-0.19286	-0.01937	-0.41108	-0.14615	-0.06111
FSIP2	0.208398	-0.18254	NA	NA	NA	NA	-0.14152	-0.36435
FST	-0.23582	-0.01496	0.005953	-0.08513	0.156908	0.170269	-0.02453	0.055099
FSTL1	-0.27932	-0.24322	0.156287	0.009025	-0.06498	0.177689	-0.23176	-0.13405
FSTL3	-0.12949	0.127752	-0.03936	-0.01089	0.132606	0.241882	-0.06307	-0.11011
FSTL4	0.229879	0.11048	-0.06955	-0.03625	-0.03801	-0.22775	0.41764	0.208532
FSTL5	0.142557	0.056008	-0.17233	-0.03513	0.092839	-0.13384	-0.16615	-0.06148
FTCD	-0.33323	0.331576	0.009949	0.255711	-0.05718	-0.19754	-0.29724	0.000172
FTH1	-0.29783	-0.2004	0.047773	-0.36389	0.066245	-0.02298	NA	NA
FTHL17	-0.02731	0.665274	0.118064	-0.22712	0.010845	-0.01177	0.006273	0.029692
FTL	-0.00421	-0.43933	0.427593	-0.03257	0.081123	0.029608	0.588642	0.638314
FTMT	0.138999	0.72019	0.089033	-0.50812	-0.0364	-0.04944	0.467715	0.206786
FTO	NA	NA	-0.06104	0.086542	0.062627	-0.0488	0.274423	0.054697
FTSJ1	0.078996	-0.58733	0.099973	0.214162	0.146148	0.028472	0.360883	-0.05183
FTSJ2	0.170545	-0.08928	-0.00206	0.207675	0.220013	-0.0191	-0.26396	-0.44604
FTSJ3	0.458444	0.201899	0.051116	0.300442	-0.16138	0.24809	-0.42168	-0.20664
FTSJD1	NA	NA	-0.24459	-0.33138	-0.13423	-0.06744	NA	NA
FTSJD2	NA	NA	0.21573	0.066183	0.310879	0.72497	NA	NA
FUBP1	0.179792	-0.00411	-0.11957	0.189831	0.096162	0.081162	0.309318	0.254587
FUBP3	NA	NA	-0.38568	0.193337	0.252353	-0.16842	0.664563	0.332539
FUCA1	0.019262	-0.31506	0.130407	-0.19842	0.170131	-0.03689	-0.17551	-0.06921
FUCA2	0.402333	-0.46713	-0.00292	0.20897	0.182796	-0.2734	0.336698	0.4792
FUK	0.308879	0.379348	0.208883	0.149144	0.232363	0.258089	0.072736	-0.01152
FUNDC1	0.079105	-0.64852	-0.12723	-0.16992	-0.07804	-0.30594	-0.14301	-0.48748
FUNDC2	0.449181	0.628065	-0.12971	-0.06381	-0.14349	-0.40758	-0.0727	0.124827
FURIN	-0.06846	0.291969	0.174525	0.128939	0.270469	-0.11926	0.06927	0.173361
FUS	NA	NA	-0.02897	0.494928	0.399837	0.46247	-0.24087	-0.16
FUSIP1	-0.24257	-0.5317	-0.09495	-0.04588	0.008801	-0.18582	0.156299	-0.2727
FUT1	0.189815	-0.03791	-0.21696	-0.12372	0.178418	-0.15168	-0.3421	-0.31928
FUT10	0.036562	-0.15776	0.059508	-0.5599	-0.33618	-0.28163	0.42594	0.649758
FUT11	0.088875	-0.18946	-0.33018	0.124998	0.005091	-0.29947	-0.38575	-0.19845
FUT2	-0.18355	0.156985	0.307071	0.081786	0.328332	-0.18162	0.333175	0.337076
FUT3	-0.39373	0.120392	0.222594	0.203518	-0.02988	-0.04023	0.306234	0.121499
FUT4	-0.40048	0.077685	0.071654	0.03736	0.286901	0.194541	0.262397	0.313094
FUT5	0.211394	0.275995	0.339549	0.035362	0.217867	0.093683	-0.29785	-0.17582
FUT6	-0.19208	-0.01473	0.112615	0.024353	0.187113	0.233439	-0.05307	-0.11247
FUT7	0.056941	-0.07114	0.155275	0.221233	-0.01995	-0.14615	0.664305	0.441275
FUT8	-0.12669	-0.51638	-0.30038	0.314549	-0.55888	-0.01766	-0.23212	0.184197
FUT9	-0.30967	0.63288	0.114122	-0.16103	-0.0519	-0.12936	-0.16829	-0.22784
FUZ	0.452813	-0.4917	0.217309	0.093197	-0.03913	0.221619	NA	NA
FXC1	0.160942	0.450314	-0.02594	-0.17834	-0.07249	-0.16675	0.378745	0.496992
FXN	-0.15905	0.03952	-0.17515	0.135953	-0.39629	-0.18298	-0.63161	-0.2113
FXR1	0.011895	-0.27335	0.190353	-0.12638	-0.14874	-0.14203	0.087694	-0.01759
FXR2	-0.2188	-0.1275	0.106805	0.169769	0.192175	-0.19386	0.109896	0.362064
FXYD1	-0.13913	0.083295	0.09874	-0.09433	-0.22427	-0.03977	-0.25184	-0.28376
FXYD2	0.294158	0.352009	0.104439	-0.0426	-0.08398	-0.38007	0.145616	-0.15457
FXYD3	-0.44633	0.101666	0.006631	-0.19644	-0.31395	-0.39449	0.200573	-0.15407
FXYD4	0.173798	-0.01889	-0.01539	0.095038	-0.09476	-0.08955	-0.10829	-0.17618

FXYD5	0.119406	0.22643	0.237199	0.570045	-0.12712	0.470107	0.367506	-0.16468
FXYD6	0.105445	0.01652	0.297104	0.014632	-0.11827	0.304031	-0.27653	-0.36059
FXYD7	0.138961	-0.08426	0.257363	0.249092	-0.43927	0.132267	-0.04265	-0.28087
FYB	-0.56525	-0.27481	0.13861	0.544136	-0.26804	0.507706	-0.45222	0.339709
FYCO1	0.010777	-0.14742	-0.03379	-0.12078	0.097234	0.081301	-0.47457	-0.30556
FYN	-0.07426	0.291406	0.083042	0.592986	-0.34086	0.737422	0.076114	0.341342
FYTTD1	-0.12217	0.40515	-0.24027	-0.25184	-0.09907	0.131314	-0.72691	-0.71136
FZD1	-0.27865	-0.65634	-0.04515	0.059074	0.08836	-0.08937	-0.59655	-0.10004
FZD10	0.232859	0.147126	0.355719	0.282132	0.368342	0.050057	0.316526	0.262928
FZD2	-0.13867	0.099835	-0.00782	0.009476	-0.30794	0.034974	-0.08965	-0.16308
FZD3	0.203122	-0.21273	0.214789	-0.1935	-0.03078	-0.21924	0.467261	-0.32811
FZD4	-0.31477	-0.35394	-0.17817	0.142077	-0.08296	-0.12722	-0.13215	0.129551
FZD5	-0.12194	0.053837	-0.26865	-0.22293	0.232624	-0.22923	-0.04501	0.233388
FZD6	-0.05841	-0.66224	-0.15247	-0.4757	-0.10437	-0.08186	0.126144	-0.09418
FZD7	-0.04421	-0.46938	-0.03828	0.010473	-0.1252	0.352402	0.368061	-0.09109
FZD8	0.085454	-0.18801	-0.16172	0.126215	0.020441	-0.19385	-0.34926	-0.36682
FZD9	0.00394	0.468563	-0.04039	0.134859	0.093543	-0.02007	0.107458	0.494879
FZR1	0.249465	0.132849	0.196774	0.099884	0.314198	0.121982	-0.45085	-0.31891
G0S2	-0.46664	-0.24913	0.06842	0.209045	0.126116	0.117317	-0.32993	0.324851
G3BP1	0.318988	0.365421	0.24555	-0.12654	-0.09058	0.141315	0.289829	0.335914
G3BP2	-0.17189	-0.60057	-0.32673	-0.03278	-0.22025	-0.09028	0.165707	-0.0056
G6PC	-0.16779	-0.14143	0.222953	-0.17873	0.353714	0.335974	0.203836	0.498791
G6PC2	-0.08272	0.251223	-0.04481	-0.21449	-0.1591	-0.12124	0.23602	0.083423
G6PC3	0.363525	0.631283	0.202059	0.174418	0.03758	-0.19157	0.548323	0.285904
G6PD	0.203881	0.313125	0.120949	0.235991	0.236388	0.054606	0.256887	0.049808
GAA	-0.08916	0.234046	0.361781	0.268607	0.188388	0.1466	-0.01697	-0.12307
GAB1	-0.20626	-0.4619	-0.20962	-0.26043	-0.28262	-0.31314	-0.02378	-0.0948
GAB2	-0.25714	0.155589	0.295487	-0.02032	0.164622	0.251517	0.025703	0.258124
GAB3	0.136437	-0.39526	0.127882	0.467566	-0.25147	0.593927	-0.63843	0.578443
GABARAP	-0.42998	0.497177	0.123298	-0.34729	-0.46353	-0.01152	-0.59097	-0.63504
GABARAPL1	-0.27492	-0.13249	0.297757	-0.12718	-0.04368	-0.19948	-0.23055	0.113052
GABARAPL2	-0.25327	-0.67675	-0.00577	-0.14577	-0.1422	-0.49954	-0.65986	-0.52891
GABARAPL3	NA	NA	0.336166	-0.28626	-0.015	0.104994	NA	NA
GABBR1	0.174118	0.144183	0.092162	0.100371	0.24619	0.380119	-0.0119	0.39756
GABBR2	-0.10195	-0.04935	-0.02696	0.083626	0.171273	-0.17592	0.22977	0.21097
GABPA	0.153828	-0.56935	-0.2031	-0.33368	-0.28642	-0.25471	-0.02675	-0.26732
GABPB1	NA	NA	-0.27399	-0.26072	-0.03876	-0.11115	NA	NA
GABPB2	-0.11552	-0.07149	-0.13401	-0.25443	0.191002	-0.00279	0.186583	0.278076
GABRA1	-0.10814	0.561477	0.013964	-0.21444	0.084448	-0.05893	0.41641	0.556656
GABRA2	-0.07416	-0.12602	0.214922	-0.27698	-0.02559	-0.10205	0.37106	0.281726
GABRA3	0.329783	0.720479	0.023662	0.13421	-0.19784	0.259518	-0.50776	-0.05761
GABRA4	0.250416	0.122012	0.114536	0.091054	0.135686	0.234396	0.124064	0.016971
GABRA5	-0.25975	0.375861	0.245432	-0.18526	0.351695	0.15772	0.081608	-0.33576
GABRA6	0.1038	0.564452	0.142137	-0.19779	0.4483	-0.06336	-0.6791	-0.32546
GABRB1	-0.0721	0.387567	0.26166	0.156092	-0.37979	-0.16759	-0.07498	-0.02366
GABRB2	0.343775	0.675036	-0.07818	0.175372	-0.19568	-0.12446	-0.10402	-0.21058
GABRB3	-0.07191	0.641042	-0.03476	0.051164	0.053442	-0.0383	-0.28048	-0.23181
GABRD	0.142862	0.291366	0.087133	-0.17766	0.120993	0.032262	0.09227	-0.0607
GABRE	0.121198	0.248911	-0.05894	-0.19219	0.387223	0.326746	0.277809	0.431903
GABRG1	0.320502	0.092487	0.078507	0.127687	-0.14675	-0.29239	-0.26358	-0.00151
GABRG2	0.131418	-0.18294	0.240424	-0.05876	0.114502	-0.08188	0.34881	-0.41615
GABRG3	0.101631	0.273567	0.07102	-0.03176	0.0099	0.114894	-0.34199	0.261187

GABRP	-0.30902	0.11762	0.131515	0.383988	-0.30688	0.301357	NA	NA
GABRQ	0.438971	0.560413	0.020115	-0.00131	0.033421	0.080811	0.070023	0.250601
GABRR1	0.242302	0.286917	0.001644	-0.0966	0.02981	-0.06655	0.232717	0.408052
GABRR2	0.005076	0.584236	-0.0957	-0.44998	0.173743	0.020634	-0.10793	-0.08141
GABRR3	NA	NA	0.017448	-0.10962	-0.04603	-0.18946	NA	NA
GAD1	-0.09953	0.357054	0.381429	0.088427	0.159677	-0.54691	0.391235	0.281588
GAD2	-0.22849	-0.38147	-0.07277	0.235468	0.069853	0.21693	0.666199	0.540924
GADD45A	-0.3128	-0.22307	0.483277	-0.08858	0.11315	0.22548	-0.26404	-0.11657
GADD45B	-0.2881	0.21099	0.082951	0.201164	0.124784	0.284669	-0.35973	-0.26065
GADD45G	0.023047	0.161016	0.331736	-0.02323	0.215705	0.10217	-0.44349	-0.17609
GADD45GIF	0.040221	-0.14267	0.098237	0.038361	-0.12498	-0.03505	-0.64167	-0.52763
GADL1	0.294134	0.002936	NA	NA	NA	NA	0.264274	0.246181
GAGE1	0.024826	0.212988	0.051673	-0.11927	-0.11699	-0.10011	0.072583	0.115266
GAGE6	0.232156	0.563596	NA	NA	NA	NA	-0.22796	-0.17154
GAGE7	-0.10574	0.215576	-0.04581	0.358248	0.124471	0.107881	-0.35349	-0.37089
GAK	-0.00961	0.315846	0.243876	0.016632	0.299959	0.331599	0.287131	0.177012
GAL	0.219206	-0.06587	-0.3068	0.179291	-0.14202	-0.08068	-0.00034	-0.17009
GAL3ST1	0.197412	-0.134	-0.0091	0.03776	0.017369	-0.2966	0.471121	0.108764
GAL3ST2	0.226163	0.738607	-0.06079	-0.19656	0.058378	-0.07235	-0.1437	-0.46926
GAL3ST3	0.388825	0.155062	-0.02843	-0.1156	0.271025	0.021546	0.065769	-0.06635
GAL3ST4	-0.1473	0.05436	0.142571	-0.08816	0.125027	-0.09447	-0.59239	-0.38236
GALC	0.250564	-0.40184	-0.32714	-0.28213	-0.17412	0.01116	0.194644	0.465756
GALE	-0.07943	0.39352	0.316518	0.174514	0.188902	-0.10665	0.206139	-0.02657
GALK1	-0.03232	0.320519	0.311025	0.055338	0.049107	-0.12756	0.290268	0.257329
GALK2	0.2164	-0.48592	-0.06937	0.038555	-0.13402	-0.33331	0.296226	0.178726
GALM	-0.15547	0.054948	-0.03434	0.078686	0.084823	-0.04899	0.09729	0.163059
GALNAC4S-	-0.28924	-0.20915	0.032646	0.114453	-0.03038	0.071506	-0.33035	-0.05019
GALNS	0.042399	-0.36333	0.379334	0.071563	0.279839	0.126878	-0.5214	-0.24455
GALNT1	-0.24118	-0.25691	-0.13791	-0.29351	-0.07396	-0.13012	-0.21272	-0.28856
GALNT10	-0.12132	-0.60003	0.334164	0.119363	0.104955	0.017684	0.011093	0.276644
GALNT11	-0.09334	-0.49516	0.229756	0.129557	-0.01114	0.099171	-0.18419	-0.33629
GALNT12	-0.4149	-0.45277	-0.19429	0.11521	0.099314	-0.31621	-0.11769	0.067448
GALNT13	0.014211	-0.08768	0.007882	-0.19064	-0.07211	-0.1185	0.134131	0.471331
GALNT14	0.103867	-0.25352	0.100222	0.646714	0.058626	0.181205	-0.50908	-0.22998
GALNT2	-0.05243	-0.10364	0.209591	0.142972	0.233246	0.05708	0.037071	-0.19832
GALNT3	-0.15523	0.366167	-0.09692	0.018997	-0.09008	-0.49136	-0.57594	-0.47931
GALNT4	-0.19993	-0.26888	-0.24547	0.027718	0.271403	-0.11453	0.171793	0.172477
GALNT5	0.005147	-0.17422	-0.2854	0.213626	-0.18781	-0.46714	0.288329	0.045431
GALNT6	-0.19706	0.36774	0.180228	-0.08391	0.193634	-0.09677	-0.06247	0.051711
GALNT7	-0.27029	-0.01534	-0.32881	-0.17866	-0.02278	-0.26097	-0.15943	-0.29146
GALNT8	-0.12801	0.229166	-0.28408	0.393639	-0.01409	-0.15702	0.387292	0.320676
GALNT9	-0.11849	0.397279	0.333641	-0.09866	0.005728	-0.00588	-0.07744	-0.18266
GALNTL1	0.101011	0.070832	-0.08316	-0.01177	0.010441	0.125166	0.039385	-0.038
GALNTL2	-0.47788	-0.32162	0.21405	0.045739	-0.18498	-0.10588	-0.39799	-0.153
GALNTL4	0.210815	-0.36249	-0.001	0.104505	0.039474	0.500572	-0.25287	-0.19415
GALNTL5	-0.15004	0.066192	-0.02855	-0.18857	0.137562	0.165628	0.159143	0.269235
GALP	0.102806	0.292038	0.01467	-0.06624	0.1489	-0.13148	0.315699	-0.13581
GALR1	-0.09756	0.317971	0.077643	-0.12085	-0.31751	-0.38884	0.215884	0.116217
GALR2	0.151535	0.225807	0.205349	-0.19929	0.089462	0.048462	-0.17475	-0.21325
GALR3	0.354294	-0.06032	-0.03891	0.088329	-0.09563	0.230168	-0.40344	0.185455
GALT	0.436166	0.588843	0.376245	-0.2121	0.192927	0.181722	-0.05171	0.179651
GAMT	0.349049	-0.55379	0.210657	0.064131	-0.04219	0.090162	0.064557	-0.0293

GAN	0.485411	0.581379	-0.39085	-0.38718	-0.14325	-0.08469	-0.32855	0.064748
GANAB	-0.28834	0.113533	0.180776	0.436553	0.289772	0.063096	0.0271	-0.30869
GANC	0.013375	-0.35724	NA	NA	NA	NA	-0.08688	0.222537
GAP43	0.102027	0.236261	-3.5E-06	0.066114	-0.03075	-0.26712	-0.33348	-0.05344
GAPDH	0.236052	0.451404	0.120597	-0.00256	0.091157	-0.13065	-0.08354	-0.26153
GAPDHS	0.28431	-0.05417	0.070551	0.027655	0.110979	-0.00068	0.015169	0.027036
GAPT	NA	NA	0.064704	0.452182	-0.07496	0.545552	NA	NA
GAPVD1	-0.03211	-0.55552	-0.05217	-0.35608	0.002642	0.178609	-0.5484	-0.21709
GAR1	NA	NA	-0.13767	0.279014	0.14636	0.251914	NA	NA
GARNL1	0.039097	-0.30897	-0.29399	-0.36033	-0.21336	0.107637	-0.50255	-0.35687
GARNL3	0.308883	0.1248	-0.299	-0.05819	0.322566	0.138927	0.256576	0.179998
GARNL4	0.044189	0.523378	0.171091	-0.09391	0.06571	0.125801	-0.00183	-0.13138
GARS	0.160086	-0.38492	-0.08363	0.162792	0.039927	-0.24612	-0.04848	0.029156
GART	0.443117	-0.03758	-0.1874	0.175223	0.133677	0.099016	0.447845	0.435294
GAS1	-0.27961	-0.4037	0.050319	0.008727	-0.11103	-0.06069	-0.35988	-0.33208
GAS2	0.453832	0.412473	0.151629	-0.26454	0.082114	-0.54264	0.377883	0.147917
GAS2L1	-0.29286	-0.09624	0.085796	-0.10932	0.042756	-0.28264	-0.20991	0.062424
GAS2L2	0.127558	-0.25707	0.187927	-0.25685	0.155584	0.071791	0.381165	0.054993
GAS2L3	-0.10006	-0.08703	-0.27109	-0.15231	0.077778	-0.01822	0.282341	-0.01469
GAS6	0.063225	0.041603	0.46505	0.005336	0.243962	0.262006	0.013732	0.231922
GAS7	-0.10619	-0.12266	0.231777	-0.09651	-0.13882	0.331295	-0.17825	-0.02085
GAS8	0.013007	-0.07452	0.014116	0.040913	0.451129	-0.25397	0.568639	0.320076
GAST	0.056225	0.411859	0.194034	-0.26395	0.041436	-0.01612	-0.04253	-0.09601
GATA1	0.222249	0.484451	0.411752	-0.07764	-0.16305	0.003823	-0.30303	-0.29959
GATA2	-0.12389	0.41497	0.216628	0.276659	0.435256	-0.05034	-0.38126	0.102053
GATA3	0.304838	-0.05014	0.402325	0.777933	-0.05595	0.657848	-0.05514	0.203196
GATA4	-0.28637	-0.48429	0.140504	-0.04489	-0.21339	-0.14603	0.422422	0.523576
GATA5	0.253575	0.025439	-0.09968	0.045017	-0.14414	-0.21396	0.506228	0.526037
GATA6	0.149954	0.142488	-0.28396	0.047733	0.086157	-0.26176	-0.18358	-0.0685
GATAD1	0.354234	-0.17477	0.124651	-0.2375	0.232206	0.099922	-0.35577	-0.26449
GATAD2A	0.367965	0.259664	-0.09285	0.076909	0.353546	0.167542	-0.0191	0.375137
GATAD2B	0.214999	-0.55323	-0.01678	0.159781	-0.07834	0.158558	0.259456	-0.1979
GATC	NA	NA	-0.063	-0.17033	0.044545	-0.10453	NA	NA
GATM	-0.01738	-0.4008	-0.20432	-0.21178	0.038474	0.303014	0.180762	0.52341
GATS	0.021451	0.151513	NA	NA	NA	NA	-0.1609	-0.13938
GBA	0.355562	-0.61806	0.244088	-0.11332	0.235744	-0.19163	0.411776	0.269479
GBA2	-0.0608	0.398039	0.360963	0.148463	0.276349	-0.18693	-0.03424	0.283819
GBA3	-0.05045	0.077147	-0.16192	-0.26174	-0.1313	-0.76106	0.044596	0.239999
GBAS	0.40109	-0.16062	-0.05873	-0.52596	-0.10351	-0.26337	-0.1802	-0.48661
GBE1	-0.46892	-0.60286	-0.26273	-0.17336	0.126294	-0.03743	0.250308	0.325299
GBF1	-0.20547	0.536598	0.0948	0.102245	0.23238	0.174155	-0.07181	0.012871
GBGT1	0.086273	0.279517	0.502718	0.069469	0.257342	0.339267	-0.43108	-0.19339
GBL	0.262198	0.624677	0.263259	0.059732	0.357921	0.05579	0.561908	0.123532
GBP1	0.022529	-0.41001	0.082283	-0.19884	-0.22073	0.176612	-0.33776	0.325265
GBP2	0.052357	0.342637	0.333882	-0.08331	0.019154	0.181632	-0.47641	-0.06425
GBP3	-0.09336	-0.32351	-0.06277	0.024619	0.185428	-0.30695	-0.60707	-0.31453
GBP4	-0.03845	-0.0882	0.270081	-0.105	-0.29157	0.374225	-0.23853	0.414904
GBP5	-0.38308	-0.11943	0.200857	0.001063	-0.20786	0.455993	-0.36294	0.207211
GBP6	0.278785	0.032625	0.079974	-0.00378	0.046404	-0.19303	-0.33861	-0.25295
GBP7	-0.21616	0.461851	0.099595	0.322444	-0.16455	0.027966	0.044887	0.344021
GBX1	NA	NA	-0.0812	0.205045	-0.36572	0.307774	NA	NA
GBX2	-0.05194	0.19881	0.355997	-0.15595	-0.03183	0.101231	0.41335	-0.22346

GC	-0.47239	-0.07381	0.065214	-0.11238	0.317805	0.06517	0.124005	0.415498
GCA	-0.35229	-0.52361	0.173066	0.060105	-0.03593	-0.04741	0.122665	0.067174
GCAT	-0.0672	-0.01172	0.262328	0.128702	0.45965	-0.11604	0.0328	-0.24858
GCC1	0.282284	0.542458	0.070774	0.085455	0.22707	-0.12188	-0.38221	-0.35414
GCC2	-0.03095	0.195771	-0.12973	0.079037	-0.18598	-0.17394	0.280474	0.297056
GCDH	0.457753	0.212536	-0.00334	0.41673	0.261637	0.217511	0.031832	-0.1974
GCET2	-0.04072	-0.067	-0.04401	0.61897	-0.09761	0.388947	-0.33895	-0.38772
GCG	-0.1376	0.022845	-0.06095	-0.016	-0.06191	-0.01211	-0.01693	0.26153
GCGR	-0.05194	0.160409	-0.17281	-0.1081	0.287676	-0.062	-0.29045	-0.06231
GCH1	-0.33946	-0.05999	-0.35782	0.035394	-0.1393	-0.13896	-0.00245	0.412499
GCHFR	0.26353	0.405378	0.15998	0.365556	-0.06921	-0.12557	0.315163	0.346184
GCK	0.002071	0.382282	-0.19803	0.00604	-0.11981	-0.2175	0.417487	0.483755
GCKR	0.019481	0.053622	-0.22192	0.280701	-0.16448	0.09125	0.411776	0.516549
GCLC	0.436868	-0.39381	-0.42564	-0.10198	-0.01251	0.18835	-0.56535	-0.22926
GCLM	-0.2497	-0.26868	-0.05595	0.073775	0.000648	-0.35063	-0.68495	-0.49676
GCM1	-0.19276	-0.23672	-0.08052	-0.28138	-0.08005	-0.21504	0.392579	0.465296
GCM2	-0.1815	0.415992	-0.23658	-0.15034	0.285242	0.009294	0.393316	-0.08273
GCN1L1	-0.07026	0.458482	-0.37764	0.386547	0.177092	0.089866	-0.62207	-0.52324
GCNT1	0.227064	-0.03209	-0.35815	0.176687	-0.07196	-0.20007	-0.31959	0.025037
GCNT2	0.010962	0.545762	-0.13246	-0.16898	-0.36617	-0.1921	-0.2917	0.113058
GCNT3	-0.09839	0.164373	-0.03597	-0.14947	-0.07247	-0.16832	-0.01583	0.207434
GCNT4	0.160022	0.725691	-0.07468	-0.28697	-0.3342	-0.34625	NA	NA
GCOM1	0.171618	-0.06557	-0.17588	-0.25878	-0.16075	-0.2823	NA	NA
GCS1	0.227804	-0.0666	0.307474	0.353254	0.398445	0.102957	0.598117	0.434194
GCSH	NA	NA	-0.02038	-0.11714	0.14951	-0.3047	0.126447	-0.27886
GDA	0.265938	-0.10961	-0.05127	-0.06514	-0.06494	-0.22167	0.060494	0.488448
GDAP1	-0.18937	-0.25751	-0.10906	0.307092	-0.19177	-0.3716	0.38005	0.501246
GDAP1L1	0.423905	-0.05338	0.170269	0.087073	0.432589	0.230093	-0.03629	0.282589
GDAP2	0.107432	0.018156	0.080617	-0.25863	0.019591	-0.31901	0.571613	0.520302
GDE1	NA	NA	-0.16314	-0.06769	-0.28186	-0.19522	NA	NA
GDEP	NA	NA	-0.41714	-0.05208	0.141567	0.109877	NA	NA
GDF1	-0.23307	0.601995	0.064645	-0.24983	-0.12747	0.06646	-0.05889	-0.39843
GDF10	0.143612	-0.18845	0.280119	0.020715	0.412863	-0.17757	0.004335	-0.00937
GDF11	0.040299	0.505226	0.101259	0.212541	0.133657	0.233966	0.025873	-0.0303
GDF15	-0.16162	0.185751	0.204177	0.312258	0.231468	-0.09468	-0.15554	-0.05218
GDF2	0.29633	0.698695	0.188511	0.126342	-0.21108	0.103027	0.458396	0.016651
GDF3	0.20752	-0.07915	0.134152	-0.12595	0.279403	-0.35366	0.278606	0.201583
GDF5	0.176935	0.178534	-0.2767	-0.19474	0.077506	0.036618	-0.44515	-0.21223
GDF6	-0.01678	-0.15698	0.27643	0.008722	-0.13167	-0.07079	-0.13304	-0.48863
GDF7	-0.15625	0.625915	NA	NA	NA	NA	-0.16097	-0.00277
GDF9	-0.17701	0.400721	-0.233	-0.09575	0.246457	-0.28703	0.350923	0.357037
GDI1	0.011934	0.114897	0.0632	0.348243	0.102428	0.021651	0.343582	0.251144
GDI2	0.359749	0.054123	0.166949	0.181265	0.29123	0.098582	0.009053	-0.30423
GDNF	0.27951	0.404494	0.132564	-0.15651	0.027557	0.228036	-0.45419	-0.33656
GDPD1	-0.2765	-0.36381	0.327708	-0.0569	-0.09999	-0.15246	-0.5849	-0.58241
GDPD2	-0.25337	0.017944	0.030346	-0.14136	-0.12753	-0.42451	0.029436	0.345298
GDPD3	-0.22256	0.354263	-0.23028	0.047773	-0.08718	-0.26701	-0.02673	-0.00728
GDPD4	0.307757	0.649747	-0.02249	-0.36686	0.152742	0.051113	-0.2585	0.16368
GDPD5	0.378089	0.122939	0.073296	0.384003	0.292191	0.444255	0.338111	0.329769
GEFT	0.056625	0.050826	0.078885	-0.08239	0.014738	-0.01074	-0.3513	-0.36187
GEM	-0.06352	-0.19993	0.048061	0.124654	-0.1014	0.222406	-0.0745	-0.32821
GEMIN4	0.018115	0.218296	-0.40649	0.426214	-0.14417	0.337039	0.267341	0.128766

GEMIN5	0.245557	-0.05625	0.236522	-0.04178	0.015483	0.390692	0.418946	0.145945
GEMIN6	0.555125	0.13447	-0.01121	-0.17917	-0.22592	-0.24309	0.165386	-0.00598
GEMIN7	0.326376	0.717333	-0.277	0.167917	-0.2003	-0.38492	-0.0992	-0.32336
GEMIN8	0.335413	-0.00026	0.286198	0.154662	0.302615	-0.17541	-0.0929	-0.42819
GEN1	NA	NA	-0.30339	0.087848	-0.10556	-0.03253	NA	NA
GFAP	0.290994	0.618147	-0.23189	-0.2925	-0.07234	0.031272	-0.54748	-0.2664
GFER	0.188555	-0.17755	-0.17919	-0.24229	0.114955	-0.52554	-0.27523	-0.30905
GFI1	-0.58675	-0.07965	-0.05955	0.335438	-0.16242	0.031427	-0.08001	0.431299
GFI1B	0.101811	0.20008	0.052351	-0.08005	0.186219	0.161953	0.494649	0.443955
GFM1	0.257312	0.243252	-0.50785	0.010009	-0.04919	-0.4507	0.003045	-0.10815
GFM2	-0.01414	-0.2086	-0.02904	-0.40532	0.051415	-0.46357	0.230091	0.145987
GFOD1	-0.18599	0.509678	-0.13584	0.266409	0.404519	-0.07521	-0.48626	-0.37476
GFOD2	-0.06873	0.59125	-0.06283	0.113416	0.354296	-0.25826	NA	NA
GFPT1	-0.27681	-0.31985	-0.0712	-0.0626	-0.23464	-0.08461	0.209426	-0.09415
GFPT2	-0.17597	-0.09133	0.079523	0.059258	-0.00718	0.184722	-0.09785	0.32271
GFRA1	-0.21043	0.10044	0.036428	0.08025	0.060656	0.227188	0.090268	-0.1708
GFRA2	-0.37312	0.403285	0.058808	-0.28751	-0.35075	0.226046	-0.27567	-0.07265
GFRA3	0.165283	-0.12632	0.086346	0.061435	-0.29997	-0.0153	-0.28232	-0.00389
GFRA4	0.00783	0.674168	-0.01853	-0.00552	-0.15549	0.218386	0.425031	0.426041
GFRAL	-0.02663	0.019806	0.092094	0.044091	0.275752	-0.27933	NA	NA
GGA1	0.319806	0.078565	0.005494	0.054903	0.390767	0.282575	0.480122	0.607167
GGA2	-0.07274	-0.10288	-0.06712	0.673613	0.356708	0.575888	-0.23013	0.050216
GGA3	0.00065	-0.21104	0.088136	0.30677	-0.09322	0.292766	0.443537	0.306479
GGCT	NA	NA	-0.13054	0.149284	0.17115	-0.24999	NA	NA
GGCX	-0.05939	0.520244	-0.02321	0.195182	-0.16924	-0.13283	0.110554	0.183564
GGH	0.097119	-0.35184	0.287138	0.018666	0.151064	-0.11073	0.187056	-0.35756
GGN	0.266069	0.677774	-0.11756	-0.06232	-0.17173	-0.50249	0.472152	0.46672
GGNBP1	NA	NA	0.099884	-0.09574	0.089905	-0.09741	NA	NA
GGNBP2	NA	NA	0.071444	0.031377	0.061735	0.025656	NA	NA
GGPS1	-0.16233	-0.50152	-0.20095	-0.1296	-0.44779	-0.41403	0.154652	-0.17222
GGT1	0.067962	-0.34847	0.377854	-0.26758	0.006995	-0.3652	0.331528	0.452566
GGT5	NA	NA	0.286985	0.325142	0.015048	0.30078	NA	NA
GGT6	0.103766	0.065736	0.157343	-0.21011	-0.01541	0.093314	0.171481	0.090799
GGT7	NA	NA	0.229414	-0.00509	0.271411	0.104535	NA	NA
GGTA1	NA	NA	0.122002	-0.30094	-0.28343	-0.09111	0.543763	0.464326
GH1	-0.10316	0.653653	0.144709	-0.12233	0.017341	0.032428	0.162686	0.127869
GH2	-0.20612	0.467082	0.09169	-0.10205	-0.30298	0.007369	0.076477	0.106568
GHDC	0.389893	0.027888	0.462905	0.360453	0.140769	-0.07151	NA	NA
GHITM	0.088575	-0.12349	-0.25278	-0.0985	-0.02419	-0.34988	-0.04176	0.100446
GHR	-0.02562	-0.25554	0.023085	-0.3704	-0.06991	-0.61625	-0.30198	-0.08058
GHRH	0.042945	0.208785	-0.20198	-0.31749	0.059758	-0.2387	0.203359	-0.13178
GHRHR	0.375882	0.689997	0.090361	-0.44015	0.106052	-0.05792	-0.25371	-0.10273
GHRL	-0.42055	-0.04259	0.190474	0.536375	0.016942	0.45482	-0.13966	0.258131
GHSR	0.152656	0.106084	0.140296	-0.26822	0.118373	-0.14437	0.161165	-0.27198
GIF	-0.09921	0.652728	-0.21065	0.231131	0.109677	-0.02247	0.228373	0.423983
GIGYF1	NA	NA	0.062594	0.026311	0.307193	0.195037	NA	NA
GIGYF2	NA	NA	0.053115	-0.07836	0.026624	0.232271	NA	NA
GIMAP1	0.024541	0.185022	0.152625	0.454923	-0.26028	0.444885	-0.61062	-0.32191
GIMAP2	0.416312	0.449395	0.237359	0.159705	-0.16251	0.080686	-0.49185	-0.03534
GIMAP4	-0.39538	0.244754	-0.05915	0.383633	-0.35426	0.231715	-0.45662	0.137191
GIMAP5	-0.29838	0.248299	0.263889	0.508962	-0.16449	0.614255	-0.35256	0.503085
GIMAP6	0.063812	-0.07664	0.108403	0.408258	-0.22345	0.45674	-0.31544	0.359484

GIMAP7	-0.67718	-0.50709	0.132519	0.530032	-0.19324	0.586746	-0.70255	-0.46121
GIMAP8	-0.40922	-0.01502	0.108379	0.352511	-0.15198	0.436447	-0.7331	-0.1521
GIN1	NA	NA	-0.22684	-0.40559	-0.22048	-0.48294	NA	NA
GINS1	0.231129	0.690034	-0.34866	0.083384	0.060371	0.106862	NA	NA
GINS2	0.267172	0.49682	-0.10092	0.207505	0.103397	-0.10948	NA	NA
GINS3	-0.00453	-0.29789	-0.45391	0.339265	-0.16265	-0.09141	NA	NA
GINS4	-0.10006	-0.25841	0.138931	0.154738	0.277109	0.110767	NA	NA
GIP	-0.04821	0.513824	0.159694	-0.16452	-0.00972	-0.17703	-0.01382	-0.26697
GIPC1	-0.01612	0.181626	0.149021	0.0427	-0.07922	-0.26742	0.450714	-0.17575
GIPC2	0.368967	-0.08329	0.201714	-0.05543	0.017304	-0.44873	0.229746	0.424011
GIPC3	0.340352	0.562121	0.071722	-0.26564	0.399462	0.147361	0.333595	0.089812
GIPR	0.086005	0.431837	0.183164	-0.20415	0.114019	-0.04082	0.098351	0.015261
GIT1	0.287058	0.476107	0.502513	0.125138	0.38437	-0.08092	0.315989	0.075502
GIT2	-0.31232	-0.49227	-0.06736	0.206385	-0.03993	0.184285	-0.05048	0.546393
GJA1	-0.29289	-0.42598	-0.16689	-0.17791	0.06917	0.184551	-0.11795	0.235101
GJA10	0.22276	-0.02015	0.396382	0.059424	-0.03819	-0.1984	0.259018	0.158668
GJA3	0.602746	0.623112	0.093989	0.160192	0.085723	0.290236	0.031328	-0.02731
GJA4	-0.17359	-0.04682	0.213682	0.357397	-0.05436	0.087475	-0.19	-0.07516
GJA5	0.043739	0.503333	-0.04487	-0.01977	-0.10919	-0.01754	0.3187	0.101854
GJA8	0.012006	0.279188	0.050358	-0.16374	0.214352	0.014018	0.132464	-0.19264
GJA9	-0.14342	-0.4216	-0.11283	-0.17927	-0.14874	-0.52777	NA	NA
GJB1	0.377726	0.514852	0.136068	0.183234	0.254278	-0.35174	0.278442	-0.16989
GJB2	-0.06859	-0.01526	-0.32267	0.012507	-0.16592	-0.50685	0.131551	-0.01152
GJB3	-0.04031	0.52484	0.178553	-0.05146	-0.2356	0.180833	0.084193	-0.08347
GJB4	-0.1479	-0.04879	0.093767	-0.02237	-0.06389	-0.04689	-0.22999	-0.3036
GJB5	-0.17734	0.698292	0.219003	-0.12405	-0.193	0.090598	-0.11535	0.064562
GJB6	-0.05602	0.308358	-0.12435	-0.08406	0.072417	0.144926	-0.09033	-0.35975
GJB7	-0.06249	0.678646	-0.12368	0.293734	0.024252	-0.17636	-0.01523	-0.11316
GJC1	0.26594	-0.05412	-0.01266	-0.1121	-0.12152	0.279227	-0.00424	0.321331
GJC2	NA	NA	0.042918	-0.1371	-0.20729	0.139528	NA	NA
GJC3	NA	NA	0.116176	-0.06127	0.0997	-0.09972	NA	NA
GJD2	NA	NA	0.247744	-0.12957	-0.16905	0.290355	NA	NA
GJD3	NA	NA	0.02649	-0.2252	0.100426	-0.0756	NA	NA
GJD4	NA	NA	0.385625	-0.22631	-0.06338	-0.41198	NA	NA
GK	-0.39455	-0.03982	0.004726	-0.23814	0.22257	-0.29	-0.21364	0.379576
GK2	0.228139	0.307824	0.049754	0.004979	0.142713	-0.45827	-0.32057	-0.34885
GK5	-0.20014	-0.63652	-0.11975	-0.12006	-0.10273	-0.22446	NA	NA
GKAP1	0.079836	-0.37386	0.111898	-0.08667	0.350163	-0.31284	-0.37857	-0.33398
GKN1	-0.03093	0.178086	-0.07851	-0.06728	-0.44444	0.366278	-0.4016	-0.49201
GKN2	-0.07027	-0.17343	0.138897	-0.22912	0.221168	-0.0024	NA	NA
GLA	0.101948	0.125571	0.02171	-0.05809	-0.0285	-0.28175	-0.11628	0.25565
GLB1	0.407155	0.259202	-0.42348	0.146889	0.190782	-0.3539	0.720931	0.210317
GLB1L	-0.24647	-0.32597	0.287234	-0.18205	0.115535	0.143562	-0.15604	0.363916
GLB1L2	NA	NA	-0.15776	-0.04045	0.169876	-0.13273	NA	NA
GLB1L3	-0.04807	0.235805	-0.06599	0.272059	0.124913	0.115193	NA	NA
GLCCI1	0.171515	0.172515	0.025291	0.455641	0.09189	0.246506	0.329419	0.392012
GLCE	0.246874	-0.54695	0.266733	-0.24904	0.402602	-0.16236	0.491283	0.302262
GLDC	0.123136	-0.05348	0.056396	-0.24568	0.144368	-0.13234	0.403788	0.114939
GLDN	0.015112	-0.11689	-0.2082	-0.27873	-0.10977	-0.49534	-0.16662	-0.18343
GLE1	NA	NA	-0.0208	0.254025	0.249088	-0.21089	NA	NA
GLG1	-0.27684	0.011716	0.172717	0.380652	0.243363	0.468972	-0.45846	-0.40037
GLI1	0.120774	-0.3948	-0.10394	0.357748	-0.13148	-0.02242	0.171125	0.026518

GLI2	-0.25513	-0.10577	0.164171	0.021714	-0.04014	0.156158	-0.05004	-0.02268
GLI3	-0.40755	-0.28385	0.081518	0.080419	-0.07013	0.152751	-0.23377	-0.10716
GLI4	-0.18387	0.30163	0.276966	-0.20102	-0.01146	0.005805	0.086556	0.147252
GLIPR1	0.042529	0.086016	-0.02758	0.013822	-0.28902	-0.0942	-0.27349	0.101559
GLIPR1L1	-0.1708	0.076765	-0.07927	0.132257	0.234794	-0.18092	NA	NA
GLIPR1L2	0.111006	0.515931	0.247703	-0.10014	-0.07785	-0.21062	NA	NA
GLIPR2	NA	NA	0.014202	-0.17993	-0.21028	0.05244	NA	NA
GLIS1	0.384252	0.850776	-0.13092	-0.25023	-0.08943	0.174956	-0.08064	0.390835
GLIS2	0.059951	0.308311	0.280946	-0.07912	0.375892	-0.06121	0.141522	0.253035
GLIS3	0.058356	0.018252	-0.07364	0.097501	-0.21565	-0.06144	-0.16173	-0.07055
GLMN	-0.30375	0.288825	0.102615	-0.06609	-0.00193	0.135509	0.177363	0.15108
GLO1	0.09412	-0.54975	-0.23678	-0.37869	-0.16829	-0.57465	0.611768	0.438746
GLOD4	NA	NA	-0.36696	-0.13284	-0.32185	-0.02102	NA	NA
GLP1R	0.203559	0.293924	0.07687	-0.15461	-0.05668	-0.072	-0.06187	-0.22829
GLP2R	0.250951	-0.34773	-0.1249	-0.38058	0.061502	-0.02254	0.388717	0.264239
GLRA1	0.352177	0.692522	0.039272	0.125861	-0.14112	0.007768	-0.35691	-0.11851
GLRA2	0.336491	-0.22479	-0.20741	-0.16895	-0.20425	-0.20998	-0.07264	-0.08452
GLRA3	0.381687	0.61976	-0.00531	0.103379	-0.01806	-0.03327	-0.17693	-0.0387
GLRA4	NA	NA	0.049669	-0.00061	-0.22069	-0.19869	NA	NA
GLRB	-0.08363	0.142211	0.19139	-0.15748	0.066363	-0.14829	-0.01072	-0.10267
GLRX	-0.34521	-0.08078	0.205268	0.063322	-0.23773	-0.18448	-0.29482	0.011969
GLRX2	-0.19347	-0.56955	0.049751	-0.14002	-0.21198	-0.22638	-0.03648	-0.01205
GLRX3	NA	NA	-0.18167	0.129324	0.085286	-0.16099	NA	NA
GLRX5	-0.07591	-0.05643	-0.59663	0.273537	-0.12153	-0.24225	-0.2587	-0.27169
GLS	0.040319	0.112789	0.114644	-0.28636	0.116428	0.311821	0.020156	-0.06411
GLS2	0.446934	0.367511	0.022091	0.488673	0.08376	0.007411	0.253675	-0.05378
GLT1D1	0.33041	0.508104	0.09186	-0.01762	0.085943	-0.10604	-0.25028	-0.30888
GLT25D1	-0.30284	-0.14937	-0.02261	0.225615	0.381583	0.17422	0.321911	0.379839
GLT25D2	0.043971	0.019706	0.100043	-0.137	-0.37132	-0.11901	0.369544	-0.0488
GLT6D1	0.05962	0.477308	NA	NA	NA	NA	0.231932	0.299369
GLT8D1	-0.27039	-0.37774	-0.19424	0.148267	0.038283	-0.17821	-0.25068	-0.47512
GLT8D2	-0.1759	-0.3821	-0.0015	-0.11742	-0.28346	0.104008	-0.13843	-0.07344
GLTP	-0.41723	-0.07788	-0.05679	-0.06886	0.123487	-0.14998	-0.23013	0.128324
GLTPD1	NA	NA	0.377041	-0.1752	-0.02899	0.151889	NA	NA
GLTPD2	NA	NA	0.236782	-0.20475	0.117965	-0.05661	NA	NA
GLTSCR1	0.461574	0.441406	-0.23885	0.142894	-0.19128	0.026016	-0.0633	-0.11811
GLTSCR2	0.539175	0.258774	0.30622	0.273295	0.303851	0.239504	0.118101	-0.15507
GLUD1	-0.21605	-0.17397	-0.14304	-0.307	0.006841	-0.29996	0.334358	0.467749
GLUD2	-0.24703	0.327858	-0.28868	-0.06347	0.19883	-0.20276	-0.3562	-0.5449
GLUL	-0.50208	-0.00094	-0.3634	0.279304	-0.25783	-0.09209	0.531951	0.271837
GLYAT	0.254266	-0.00433	-0.0128	-0.12386	0.356118	0.104233	-0.3973	0.020177
GLYATL1	0.339135	0.511298	0.232223	0.198144	-0.21083	0.007074	0.389877	0.392515
GLYATL2	0.297811	0.690507	0.133698	0.096993	0.30975	0.017577	-0.21923	-0.17218
GLYCAM1	NA	NA	0.189022	-0.11572	0.134852	0.06042	0.159669	0.014554
GLYCTK	0.284269	0.191662	0.120889	0.020031	0.339705	-0.24544	0.333992	0.540092
GM2A	-0.24954	0.100485	0.454027	-0.00725	-0.14904	0.181612	-0.2599	-0.00966
GMCL1	-0.01144	-0.15511	-0.17812	-0.20262	-0.08356	-0.38092	0.021116	-0.0245
GMCL1L	0.405048	0.422857	0.075441	-0.1281	-0.22512	-0.11515	0.046606	0.271668
GMDS	-0.34875	0.260307	0.001282	0.422061	0.138191	0.024533	0.252199	0.178391
GMEB1	-0.15917	-0.4194	-0.33901	0.329099	0.316571	0.185677	0.128357	0.276492
GMEB2	0.34319	0.358204	0.165006	0.047086	0.389708	0.062092	0.105473	0.009426
GMFB	-0.1425	0.39951	-0.44431	-0.40378	-0.37253	-0.48982	0.084402	0.085099

GMFG	-0.53429	0.158405	0.486741	0.382158	-0.11941	0.44818	-0.7395	-0.1388
GMIP	0.208233	0.390608	0.315863	0.28462	0.241936	0.304003	0.286138	0.325391
GML	-0.18505	0.468588	0.012803	0.030259	-0.23009	-0.01726	-0.42511	-0.10518
GMNN	-0.08689	-0.43581	-0.42933	-0.11901	-0.21571	-0.30598	0.256906	-0.31926
GMPPA	-0.03706	0.474843	0.221946	0.190277	0.140342	-0.24309	0.412684	0.304389
GMPPB	-0.0667	0.570452	0.227477	0.267112	0.27615	0.06949	0.157174	0.247611
GMPR	-0.20036	0.191097	0.30403	-0.008	-0.07614	-0.19047	-0.0217	-0.19312
GMPR2	0.306713	-0.14162	-0.16386	-0.10588	-0.05187	-0.14662	-0.55826	-0.41311
GMPS	-0.03573	-0.33294	-0.16915	0.169072	0.155221	-0.17938	NA	NA
GNA11	-0.07013	0.372662	0.285836	-0.07572	0.318214	0.089273	0.262261	0.371644
GNA12	0.245985	0.187103	0.131431	0.173912	0.233856	0.293796	-0.46776	-0.22403
GNA13	0.161887	0.254184	-0.16096	-0.42411	-0.29542	-0.22826	-0.64209	-0.27856
GNA14	-0.41283	-0.12853	-0.21874	0.125561	-0.21231	-0.3901	-0.07451	-0.215
GNA15	-0.4213	0.099267	0.255507	0.244299	0.048592	0.078556	-0.24773	0.169362
GNAI1	-0.08025	-0.38472	-0.16946	-0.2795	-0.06693	0.21465	-0.25616	-0.29574
GNAI2	-0.31978	-0.19327	0.189229	0.186941	-0.07686	0.097702	-0.08946	0.009383
GNAI3	-0.36838	0.076144	-0.06368	-0.15466	-0.17926	-0.15821	-0.3587	-0.11359
GNAL	0.112944	0.317529	-0.06308	-0.08902	-0.07369	-0.38934	-0.16836	0.001568
GNAO1	0.42086	0.053101	0.058192	-0.12773	0.089586	0.022417	-0.39668	-0.27188
GNAQ	-0.49182	-0.47859	-0.28661	-0.41146	0.085325	-0.24154	0.360359	0.294722
GNAS	0.360909	-0.12283	0.127914	0.063782	0.042872	0.148407	0.465725	0.175312
GNAT1	0.097327	0.659257	-0.01492	0.025847	-0.07451	0.037055	0.02855	-0.14635
GNAT2	0.077485	-0.08522	NA	NA	NA	NA	-0.15048	0.316009
GNAZ	0.050946	-0.03795	0.023515	-0.1586	0.433608	0.049326	-0.22611	-0.39092
GNB1	-0.4504	0.342121	0.203361	0.158835	0.117348	-0.1668	-0.28105	-0.11543
GNB1L	0.02718	-0.08591	0.324199	0.108453	0.167637	0.288167	-0.08571	-0.03558
GNB2	0.251657	0.668258	0.284993	0.062869	0.469194	0.016053	-0.13135	-0.18571
GNB2L1	0.205079	0.751414	0.436899	0.41017	0.198333	0.336547	0.339911	-0.33137
GNB3	-0.11814	-0.21011	-0.0874	0.311075	-0.09692	-0.08754	-0.31753	-0.13281
GNB4	-0.50003	-0.37502	0.015195	-0.08209	-0.2742	0.333604	-0.64163	-0.62387
GNB5	0.33399	0.34318	0.026629	0.233775	0.09649	0.003585	-0.35028	-0.22976
GNE	-0.48994	-0.33568	-0.04617	-0.20006	-0.16286	0.087311	-0.26624	-0.43431
GNG10	-0.20674	0.075743	0.034026	-0.36491	-0.15445	-0.36226	-0.26785	-0.09882
GNG11	-0.37613	-0.54662	-0.11336	-0.00675	-0.03121	-0.11203	-0.42318	-0.1752
GNG12	-0.09933	-0.26565	0.150743	-0.3438	0.077438	-0.21601	0.488168	0.457212
GNG13	0.286088	0.714102	-0.004	0.06157	-0.33033	0.005119	0.076105	0.14634
GNG2	-0.63443	-0.60413	-0.09261	0.156617	-0.14203	0.030703	-0.48628	0.106193
GNG3	0.06858	0.212449	-0.0127	-0.02876	0.006495	0.126357	-0.56441	-0.44598
GNG4	0.666227	0.541026	0.372412	0.076252	0.405092	-0.51104	0.283902	0.559624
GNG5	0.182055	0.109062	0.107455	-0.24365	-0.28831	-0.33399	-0.19242	-0.3307
GNG7	-0.05476	0.542402	0.055544	0.335177	-0.10877	0.226947	-0.51619	-0.14741
GNG8	0.069618	0.541772	-0.04662	0.01105	-0.11966	-0.41093	-0.09824	-6.5E-05
GNGT1	0.141206	0.141497	-0.16187	0.104562	0.161183	-0.11494	0.023071	-0.01253
GNGT2	-0.25654	-0.10422	0.039886	0.311535	-0.38739	0.161706	-0.36681	0.273717
GNL1	0.397995	0.591536	0.258143	-0.01889	0.35034	0.386434	0.179985	-0.11764
GNL2	-0.33126	-0.25818	0.156739	0.000389	0.189708	0.187501	-0.21394	-0.04239
GNL3	0.058906	-0.08601	-0.31741	0.05063	0.217072	0.209848	0.358443	0.160418
GNL3L	0.379975	0.300752	0.156257	-0.14069	0.200917	0.420903	-0.14446	-0.03004
GNLY	-0.16107	-0.0489	0.268234	0.342391	-0.32081	0.276637	-0.23192	0.381814
GNMT	0.358326	0.746038	-0.37242	-0.11526	0.080335	0.109174	0.327188	0.153581
GNPAT	-0.19781	-0.66631	0.367752	-0.11416	-0.1318	0.041509	0.528681	0.108324
GNPDA1	0.344394	0.089794	0.496237	0.027475	0.353793	0.133236	0.254288	0.330629

GNPDA2	0.084732	-0.32703	-0.02346	-0.29761	-0.11575	-0.3297	-0.53683	-0.3584
GNPNAT1	-0.11863	-0.09379	-0.44512	0.045846	-0.31909	-0.37727	-0.332	-0.52653
GNPTAB	-0.3797	-0.29724	-0.02831	-0.216	-0.19552	-0.38791	0.406775	0.128923
GNPTG	0.002878	0.388602	0.043389	-0.00617	-0.09196	0.094439	0.602893	0.392045
GNRH1	-0.10265	0.258377	-0.12998	0.06672	-0.13652	-0.03913	0.034386	0.185482
GNRH2	-0.07791	0.614513	0.123992	-0.04336	0.110138	0.029035	-0.31963	-0.06819
GNRHR	0.279143	0.569297	0.078277	0.106879	0.042629	-0.21311	-0.32049	-0.42865
GNRHR2	NA	NA	-0.3948	-0.12809	-0.28591	-0.17634	-0.34516	-0.04802
GNS	-0.49492	0.018487	0.105552	-0.17277	-0.21477	-0.15665	-0.30956	0.443558
GOLGA1	-0.19708	-0.17062	0.125635	0.036125	0.241406	0.103945	-0.25353	0.10878
GOLGA2	-0.45645	0.006665	0.084314	0.098446	0.297778	0.084482	-0.02618	-0.16336
GOLGA2L1	-0.2659	-0.11848	-0.05473	-0.04642	0.227326	0.123395	-0.16782	0.145672
GOLGA3	-0.5593	-0.51057	-0.03178	0.199	0.144137	-0.01606	-0.24335	0.025706
GOLGA4	0.108465	-0.6665	-0.2817	0.335027	0.098506	-0.1846	-0.29924	-0.04609
GOLGA5	-0.14607	-0.67613	-0.11241	-0.33112	-0.21008	-0.16887	NA	NA
GOLGA6	-0.06702	-0.38335	-0.01063	0.078176	0.260163	0.296089	0.41373	-0.50582
GOLGA7	-0.1824	-0.5434	-0.12653	-0.03541	-0.05156	-0.42912	0.312393	0.163508
GOLGA8A	-0.18159	-0.54046	-0.21217	0.372442	0.061934	0.44498	-0.35176	-0.34317
GOLGA8B	0.159566	-0.06138	NA	NA	NA	NA	0.061119	0.288902
GOLGA8E	-0.00768	0.542381	-0.3811	0.036665	-0.07604	0.1745	-0.11507	-0.08243
GOLGA8F	NA	NA	0.012017	-0.23329	0.030243	-0.31815	0.272964	0.423187
GOLGA8G	0.393757	0.657706	NA	NA	NA	NA	0.156823	-0.12182
GOLGB1	0.147964	-0.31366	-0.19323	0.002057	-0.1281	-0.13056	-0.68295	-0.40525
GOLIM4	NA	NA	0.063606	0.003135	-0.07068	0.004527	NA	NA
GOLM1	NA	NA	-0.00974	0.077372	0.071276	-0.07561	NA	NA
GOLPH3	-0.26694	-0.77377	0.031192	-0.09483	0.18191	0.005191	0.378621	0.233088
GOLPH3L	-0.01757	-0.25584	-0.21297	-0.12792	-0.16359	-0.40962	0.402728	-0.0288
GOLT1A	0.15145	0.038945	0.115754	0.071737	-0.19682	0.015886	0.542802	0.388473
GOLT1B	0.243133	-0.61566	-0.30354	-0.21093	-0.27438	-0.47722	-0.55614	-0.3785
GON4L	NA	NA	-0.05132	0.010511	0.069587	0.649244	NA	NA
GOPC	0.078871	-0.60823	-0.38927	0.046661	-0.19388	-0.24767	-0.3438	-0.43311
GORASP1	0.012328	-0.16411	0.053882	0.057996	0.330943	0.045842	0.335427	0.344982
GORASP2	0.01989	0.41803	0.005399	0.204798	-0.00867	0.02011	-0.02534	-0.32423
GOSR1	0.315988	0.68158	0.156922	0.031974	-0.19825	0.296412	0.119662	0.048716
GOSR2	0.260376	-0.44231	0.149837	0.043672	0.106066	-0.33892	0.242819	0.46282
GOT1	-0.19364	0.302854	0.115627	0.132526	0.151634	-0.0657	-0.05509	0.017319
GOT1L1	0.312675	-0.29829	0.013582	-0.375	0.089927	-0.08729	-0.1003	-0.14212
GOT2	0.434438	0.425448	-0.21636	0.143138	0.213527	0.063216	0.570697	0.150935
GP1BA	0.119554	0.235404	0.342241	0.770786	-0.22417	0.694485	0.20398	-0.0556
GP1BB	-0.3443	0.00433	NA	NA	NA	NA	NA	NA
GP2	NA	NA	-0.1218	0.160421	-0.07988	0.043065	0.126167	-0.14836
GP5	0.303372	0.334212	0.103954	-0.15168	-0.05804	0.233776	NA	NA
GP6	-0.42004	0.056789	-0.29525	0.051128	-0.0595	-0.06354	-0.69257	-0.5525
GP9	0.049509	0.352633	0.279127	-0.25388	0.109214	0.001424	0.104167	-0.19987
GPA33	-0.00974	0.093845	0.029639	-0.06968	-0.02876	-0.19336	0.120464	0.175754
GPAA1	-0.02868	-0.42779	0.338201	0.100842	0.218326	-0.26217	0.462194	0.406103
GPAM	0.437816	-0.04328	-0.3235	-0.44816	0.109802	0.145639	-0.02193	0.016907
GPATCH1	0.336032	0.105811	-0.08756	-0.29582	-0.13683	-0.00012	NA	NA
GPATCH2	0.088489	-0.43953	-0.24815	-0.28019	-0.27909	-0.12274	NA	NA
GPATCH3	-0.02756	0.056794	0.051048	0.063266	0.311527	0.013335	NA	NA
GPATCH4	0.289399	0.273613	-0.23437	0.076051	0.132571	0.108397	0.330198	0.003665
GPATCH8	0.257899	-0.34087	-0.11206	0.001591	0.036791	0.166319	NA	NA

GPBAR1	0.032758	0.221087	0.250741	-0.15468	-0.1856	-0.40879	-0.11407	0.2058
GPBP1	-0.23291	-0.70798	-0.23565	-0.14375	-0.03995	-0.25314	-0.45602	-0.33649
GPBP1L1	-0.15256	-0.04469	0.140396	0.182189	0.252454	-0.10891	-0.54604	-0.44108
GPC1	0.249968	-0.06907	0.3367	0.109925	0.237912	0.26811	0.168615	-0.02908
GPC2	0.016039	-0.28543	-0.37914	0.043626	0.245793	-0.03412	-0.03748	-0.14342
GPC3	-0.02895	0.044885	0.037935	-0.21922	0.03105	-0.05192	-0.00911	0.468641
GPC4	0.360092	-0.23696	0.077597	-0.14193	0.279743	0.237969	0.26533	-0.02918
GPC5	-0.25997	0.269913	0.179973	0.090638	-0.24288	-0.08359	-0.24949	-0.34919
GPC6	0.177478	0.285956	0.130413	-0.02553	-0.05802	0.311485	-0.43152	-0.3433
GPD1	0.182858	0.102189	0.426599	-0.1244	0.158907	-0.15886	0.201761	0.547977
GPD1L	0.019873	0.222582	-0.13216	-0.20625	0.043653	-0.35038	-0.3387	-0.21057
GPD2	-0.14747	-0.14382	-0.06959	-0.22535	-0.10399	-0.1674	-0.16622	-0.34059
GPER	NA	NA	-0.07602	-0.01447	0.054749	-0.36291	NA	NA
GPHA2	0.501569	0.582931	-0.18972	0.16534	0.180349	0.185704	-0.29444	-0.34317
GPHB5	0.223966	0.425694	0.006891	-0.23257	-0.22709	-0.11709	0.719359	0.523012
GPHN	0.095145	-0.23265	-0.16372	0.281973	-0.11095	-0.12809	0.206262	0.286946
GPI	-0.07322	0.503795	-0.37746	0.276384	-0.07936	0.14417	0.534109	0.251414
GPIHBP1	NA	NA	-0.04456	-0.05199	0.037698	0.180928	NA	NA
GPKOW	0.360822	0.56481	0.091596	-0.0219	0.052894	-0.12874	-0.60654	-0.63039
GPLD1	0.189958	-0.06707	0.04133	-0.12117	0.237921	0.096055	-0.15627	-0.38228
GPM6A	-0.10816	-0.23695	-0.14289	-0.16912	0.277277	-0.02157	-0.35449	-0.42992
GPM6B	0.032552	-0.39952	-0.16415	-0.19533	-0.15608	0.03337	-0.26406	-0.35179
GPN1	NA	NA	0.135893	-0.23267	0.035252	-0.24807	NA	NA
GPN2	NA	NA	0.129701	-0.23642	0.026514	-0.12422	NA	NA
GPN3	NA	NA	-0.31275	0.051418	-0.2366	-0.43354	NA	NA
GPNMB	-0.33029	-0.47462	0.027748	-0.24232	-0.41874	0.18731	-0.26635	-0.23395
GPR1	0.033806	0.069293	0.273562	-0.29746	-0.42524	-0.15334	-0.61117	-0.56122
GPR101	0.228211	-0.11744	-0.24222	0.087786	-0.11063	0.134646	-0.04512	0.050551
GPR107	0.216344	0.388708	-0.32328	0.145528	-0.08065	0.105757	0.247641	0.30379
GPR108	NA	NA	0.296736	0.420016	0.080427	-0.17377	0.16626	0.076439
GPR109A	-0.12491	-0.682	0.121213	0.032709	-0.02422	0.177814	-0.15905	0.328032
GPR109B	-0.54724	0.336531	0.005435	0.100073	0.086603	0.110654	0.025341	0.24578
GPR110	0.12718	-0.02968	-0.26465	0.051441	-0.36482	-0.03775	-0.1527	0.14779
GPR111	0.508785	0.30891	-0.20218	-0.12686	-0.09753	0.184341	-0.35585	-0.18176
GPR112	-0.32334	0.034158	0.081656	-0.14976	0.003508	0.021764	0.301973	0.443021
GPR113	-0.23763	0.632219	0.337575	0.185097	0.358971	-0.06649	0.468362	0.249256
GPR114	0.010184	0.067563	0.026248	0.716163	0.175609	0.524266	0.10434	0.289916
GPR115	0.438108	0.274502	0.105683	-0.05294	0.067113	0.005188	0.022386	0.089276
GPR116	-0.423	-0.30038	0.162611	0.236394	0.126793	0.288516	-0.30044	0.213669
GPR119	0.060165	0.004229	0.39434	-0.04945	0.057797	-0.10259	-0.37447	-0.31886
GPR12	-0.12106	0.670522	0.082343	-0.20855	0.038992	-0.10099	-0.21822	0.095173
GPR120	-0.33805	0.349578	-0.15482	-0.00716	-0.03497	0.132686	0.091854	-0.14634
GPR123	0.161167	0.467255	0.163266	-0.1079	-0.10906	-0.15211	-0.02259	-0.16374
GPR124	-0.26205	0.084317	0.025014	0.265182	-0.0619	0.220658	-0.33591	-0.29013
GPR125	-0.02376	-0.12328	-0.06645	-0.2185	0.09382	-0.09945	-0.05839	0.197544
GPR126	-0.48782	-0.61407	-0.22024	-0.13503	-0.32067	-0.07328	0.069373	0.440474
GPR128	0.290174	-0.10329	0.213347	-0.43905	-0.30711	0.047264	-0.06865	0.250046
GPR132	-0.22131	-0.06172	0.287958	0.602245	-0.07071	0.597542	-0.27056	0.450818
GPR133	0.097298	0.652258	-0.13812	-0.04174	-0.02287	0.005443	0.203341	0.210787
GPR135	-0.0604	0.58895	-0.04178	-0.28182	-0.08468	0.256273	-0.26467	-0.06219
GPR137	-0.04905	0.729718	-0.01929	0.370715	0.086296	-0.23698	-0.39058	-0.22693
GPR137B	-0.0645	0.060296	0.066798	0.085565	-0.27363	0.209253	-0.2264	0.17789

GPR137C	NA	NA	-0.13956	0.028917	-0.25494	-0.04283	NA	NA
GPR139	0.118199	0.573488	NA	NA	NA	NA	-0.25183	-0.35114
GPR141	-0.25776	0.337242	0.021208	-0.01635	0.004409	0.244244	0.088405	0.071899
GPR142	0.089513	0.107708	0.039012	-0.09736	0.019871	-0.11879	-0.08008	-0.15964
GPR143	-0.1938	0.120168	0.308054	-0.34151	0.163128	-0.10545	-0.09788	-0.54148
GPR144	0.370405	0.222578	NA	NA	NA	NA	NA	NA
GPR146	-0.21787	0.318405	0.133092	-0.06163	0.062608	-0.15401	-0.25785	-0.30172
GPR148	-0.13183	0.299537	NA	NA	NA	NA	-0.42506	-0.28396
GPR149	NA	NA	0.070108	-0.21513	-0.0507	0.118309	-0.50035	-0.1714
GPR15	-0.38135	-0.14781	0.188575	-0.03209	0.122425	-0.09146	0.196556	0.166762
GPR150	-0.02894	-0.04199	0.045691	-0.04732	-0.13131	0.282983	0.302818	0.070064
GPR151	-0.27343	0.29982	NA	NA	NA	NA	-0.38131	-0.18034
GPR152	0.344557	0.627006	NA	NA	NA	NA	-0.18371	-0.02833
GPR153	0.2188	0.743853	0.076574	0.030482	-0.10723	0.244015	0.271429	0.563972
GPR155	0.058497	-0.47551	0.100109	-0.24022	-0.11258	0.182226	-0.02305	0.074137
GPR156	0.063597	0.460001	0.05735	-0.22429	-0.09605	-0.0617	0.287053	0.4045
GPR157	0.163687	0.587869	0.22286	-0.15322	0.42286	0.516745	0.502654	0.363638
GPR158	-0.05601	0.277382	-0.39437	0.025998	-0.12569	-0.0805	-0.35772	-0.26933
GPR160	0.314747	0.182654	0.052224	-0.31385	0.076398	-0.00859	0.043176	0.018764
GPR161	-0.11458	-0.17098	0.248774	-0.20753	-0.15066	0.036683	-0.06359	-0.29855
GPR162	-0.48632	0.47096	-0.01266	-0.07715	0.011848	0.061935	-0.46495	-0.18534
GPR17	-0.05576	0.230453	-0.1812	-0.31686	-0.25905	-0.27778	0.0828	-0.17659
GPR171	-0.21988	0.172328	0.313084	0.094711	-0.15648	0.469884	-0.10596	-0.39635
GPR172A	0.042238	0.524253	0.533855	0.1823	0.276418	0.192845	0.632114	0.352345
GPR172B	0.019592	0.289196	0.188072	-0.19916	-0.18132	-0.11037	0.171608	0.544181
GPR173	0.259702	0.739988	0.378926	-0.2488	-0.133	0.147597	-0.31513	-0.23827
GPR174	0.138921	0.48366	0.109577	0.278965	-0.16021	0.267976	-0.46288	-0.41693
GPR175	0.276554	0.592067	0.271961	0.000715	0.269552	-0.01201	0.193714	0.115387
GPR176	-0.05	0.322967	0.240486	-0.11218	0.065855	-0.10268	0.03022	0.059582
GPR177	0.142621	-0.44363	0.563064	-0.1141	-0.07311	0.056645	-0.22226	-0.45311
GPR179	0.017226	0.117992	NA	NA	NA	NA	0.638913	0.528118
GPR18	0.01878	0.029973	0.164397	0.702468	-0.24819	0.636957	-0.32833	0.307537
GPR180	0.115529	0.035322	-0.06777	-0.09494	0.107264	0.028301	NA	NA
GPR182	NA	NA	0.141887	-0.18995	-0.04561	0.032339	NA	NA
GPR183	NA	NA	0.094691	0.511894	-0.36837	0.462638	NA	NA
GPR19	0.370159	0.682536	-0.32849	0.418334	0.120023	0.099028	-0.18197	-0.08711
GPR20	0.146281	-0.1865	0.120371	-0.13544	-0.09183	0.183755	-0.13837	-0.09426
GPR21	0.040458	0.741316	0.21293	-0.04726	-0.13067	0.400451	0.472025	0.217828
GPR22	0.212809	0.218736	-0.12078	-0.09583	0.372868	-0.11987	0.059658	0.268259
GPR25	0.029216	0.114763	0.153945	-0.17187	0.093614	0.047617	-0.46801	0.009538
GPR26	0.07465	0.14631	-0.20635	0.219196	0.058607	-0.25025	-0.43781	-0.13665
GPR27	-0.17005	-0.00502	0.110994	0.327794	-0.17378	-0.08613	-0.37209	-0.6462
GPR3	0.14324	0.353709	0.077545	-0.15407	-0.07118	0.112676	-0.07088	0.143859
GPR31	-0.01991	0.526014	0.06163	-0.07189	-0.08768	-0.00668	-0.11681	-0.56941
GPR32	0.129996	0.664905	0.274214	-0.0999	-0.11309	0.346349	-0.0319	0.277748
GPR34	-0.3398	-0.07728	-0.01926	-0.25417	-0.27874	-0.00408	-0.61123	-0.1683
GPR35	0.365327	0.422877	0.394777	-0.14306	0.285519	0.165871	0.11945	-0.16875
GPR37	0.001877	0.237939	-0.00803	0.342545	-0.23576	0.046076	0.183032	0.359573
GPR37L1	0.439784	0.611991	0.305935	-0.10672	0.03232	-0.01695	-0.12156	-0.44287
GPR39	0.277709	-0.02752	-0.09072	-0.02747	0.154598	-0.16138	0.616651	0.039542
GPR4	-0.43287	-0.01413	0.135652	0.154095	0.015627	0.375954	-0.2599	0.106925
GPR44	-0.24416	0.055653	-0.02209	-0.09752	-0.08132	-0.09636	0.277465	-0.07493

GPR45	-0.03765	0.543796	0.066463	0.074176	-0.31909	-0.39932	0.426777	0.007163
GPR50	-0.31621	0.45238	0.121785	0.020811	-0.06769	-0.01335	-0.1682	-0.15564
GPR52	-0.0458	0.45284	0.037743	-0.17607	0.087737	0.180441	-0.21361	0.234992
GPR55	-0.12318	0.073205	-0.12628	-0.17728	-0.40239	-0.14252	-0.32633	0.174885
GPR56	-0.10648	0.062996	0.190939	0.304735	0.431144	-0.01079	0.712294	0.2513
GPR6	-0.20067	0.370699	0.063211	-0.4615	0.060103	-0.06418	0.339529	0.46555
GPR61	0.066594	0.142094	0.09118	-0.09138	0.189354	0.015412	-0.34524	-0.05489
GPR62	-0.05432	-0.39955	-0.27377	-0.14998	0.262018	0.13289	0.024654	-0.01577
GPR63	-0.10623	-0.49366	-0.05941	0.141482	-0.04364	0.050317	0.093995	-0.07296
GPR64	-0.05598	0.45713	-0.133	0.589995	0.141119	0.072635	0.127622	0.486009
GPR65	-0.56897	-0.18047	0.168021	0.223613	-0.1941	0.42905	-0.61554	-0.01802
GPR68	-0.25517	-0.35577	0.060382	0.264232	-0.03854	0.471382	-0.22718	0.262042
GPR75	-0.31757	0.145143	0.053434	0.308502	-0.14448	0.077203	0.435178	0.45062
GPR77	0.026886	0.125274	0.30795	-0.33039	0.039282	0.128033	0.038919	0.22766
GPR78	-0.03845	-0.08421	-0.17642	-0.02556	-0.21725	0.033356	0.035071	0.344567
GPR81	0.132965	-0.23623	0.024361	-0.14451	-0.06791	0.166572	-0.02483	0.194061
GPR82	0.316401	0.739418	0.084131	0.139386	0.370859	-0.00319	-0.27233	0.271477
GPR83	0.164676	-0.04231	0.186908	0.110878	-0.20399	-0.04966	-0.0403	-0.12675
GPR84	-0.4959	-0.16553	0.136971	0.11429	-0.05412	-0.07486	-0.31741	0.314366
GPR85	0.061708	0.092102	-0.03878	0.119148	-0.00445	0.038964	-0.3044	-0.14323
GPR87	0.007866	0.11238	-0.07926	-0.24307	-0.36257	0.113665	0.45033	0.299602
GPR88	0.268439	0.120394	0.25089	0.200914	0.131232	0.094777	-0.49422	-0.06831
GPR89A	NA	NA	NA	NA	NA	NA	0.609314	0.724167
GPR97	-0.54401	-0.03044	0.522284	-0.04729	0.050384	0.149714	-0.40814	0.07255
GPR98	0.331427	-0.16414	-0.27208	-0.18915	-0.30116	-0.17077	-0.3029	-0.08654
GPRASP1	-0.24109	-0.18754	-0.21348	-0.02593	0.0582	0.502137	-0.69698	-0.43947
GPRASP2	0.228084	0.141554	-0.22029	-0.08234	-0.08241	-0.20941	0.004446	-0.1607
GPRC5A	0.084468	0.169475	-0.15286	0.085359	0.090014	0.077963	0.474523	0.331687
GPRC5B	0.083746	0.648515	-0.41058	0.2505	-0.31897	0.472211	-0.19389	0.431779
GPRC5C	0.29373	0.344034	0.17731	0.026493	0.085964	-0.21626	-0.01118	-0.15813
GPRC5D	0.118807	0.448783	-0.08638	0.003951	0.067427	-0.15934	0.174628	0.076075
GPRC6A	0.112954	0.655662	0.060795	0.018891	-0.04539	0.086507	0.235338	0.078442
GPRIN1	0.292347	0.442711	0.155568	0.144107	0.295195	-0.17458	NA	NA
GPRIN2	0.300062	0.190189	0.071076	0.101467	0.102312	-0.15946	NA	NA
GPRIN3	-0.32535	0.080893	0.042281	-0.05546	-0.24931	0.032407	NA	NA
GPS1	0.118008	0.743815	0.33436	0.164069	0.264472	0.139118	0.434958	-0.10091
GPS2	-0.03572	0.266631	0.035804	0.179922	-0.12754	-0.35051	0.157153	0.058356
GPSM1	0.036307	0.37902	-0.2128	0.014098	0.011242	0.361145	-0.30393	-0.25837
GPSM2	NA	NA	0.32667	-0.31729	0.223131	-0.1675	0.518422	0.107317
GPSM3	-0.37842	0.223308	0.243076	0.154338	-0.07818	0.118344	-0.48901	-0.03704
GPSN2	0.409562	0.184206	0.181644	0.31702	0.200897	0.082661	0.484835	0.120259
GPT	-0.08409	0.251377	-0.06147	0.070442	0.001848	-0.0318	0.008738	0.128232
GPT2	0.096932	0.652821	0.192344	0.121891	0.462559	-0.28363	0.195145	0.128858
GPX1	-0.23233	0.549261	0.035066	0.228042	0.049836	-0.27172	-0.17631	0.02882
GPX2	0.445315	0.159009	0.046203	0.076848	0.079702	0.152801	0.356344	0.092823
GPX3	-0.16336	0.08436	0.660231	0.074598	-0.25574	-0.23691	-0.04672	0.180248
GPX4	-0.10809	-0.33755	0.196194	0.102426	0.213024	0.13346	-0.23259	0.110262
GPX5	-0.09243	0.010803	0.175051	-0.31248	0.263436	-0.14086	0.322097	0.066723
GPX6	0.195438	0.443101	-0.04072	-0.03254	-0.28668	-0.32233	0.577002	0.445954
GPX7	-0.15371	-0.33642	-0.08563	0.401493	-0.17972	-0.13822	-0.24367	-0.20709
GPX8	NA	NA	-0.2191	0.016446	-0.43325	-0.38138	NA	NA
GRAMD1A	-0.23025	0.177048	0.257638	0.217337	0.277719	0.469625	-0.13434	-0.34708

GRAMD1B	NA	NA	0.163917	0.210405	-0.10032	0.280098	NA	NA
GRAMD1C	-0.21894	-0.23951	0.400052	-0.20494	-0.10426	-0.31587	0.540507	0.385053
GRAMD2	0.502646	0.056825	0.076289	-0.24092	0.268916	-0.29983	0.05031	0.59446
GRAMD3	0.274372	0.435772	0.198296	-0.17176	-0.20941	-0.13922	0.34213	0.162674
GRAMD4	NA	NA	-0.01526	0.139909	0.140683	-0.20582	NA	NA
GRAP	NA	NA	0.451437	0.826982	0.347213	0.75385	-0.05703	0.181308
GRAP2	0.259788	0.032714	0.147363	0.464345	0.020062	0.325239	-0.74205	-0.04762
GRASP	-0.06188	-0.00544	-0.12953	0.109377	-0.03068	0.194431	-0.04317	0.30865
GRB10	0.080203	-0.00633	-0.12424	-0.15797	-0.0979	-0.08365	-0.06029	-0.05112
GRB14	0.520473	0.707935	-0.12685	-0.17961	-0.24758	-0.06003	-0.25545	-0.26592
GRB2	-0.19849	-0.54835	0.28007	0.268327	-0.0486	0.350732	-0.08029	0.264025
GRB7	0.41927	-0.01403	0.38291	-0.12407	0.413265	0.041792	0.350801	0.011677
GREB1	-0.04202	0.041188	-0.04291	0.391198	-0.00776	0.312355	0.075838	-0.21501
GREM1	0.137528	0.400029	0.032923	0.365389	-0.09717	0.196653	-0.39368	-0.36789
GREM2	-0.09228	-0.29719	-0.15559	0.148033	-0.13834	0.313921	0.02757	0.365572
GRHL1	-0.15197	-0.04543	-0.19717	-0.04567	-0.05583	-0.20661	0.184737	0.167755
GRHL2	-0.05568	-0.05188	-0.00526	-0.18234	0.047222	-0.26094	0.600739	0.302249
GRHL3	0.030983	0.098557	-0.12381	-0.21388	-0.26576	0.255262	0.30104	0.071934
GRHPR	0.103963	0.203419	0.313517	0.164104	-0.00951	0.179847	-0.33671	-0.25953
GRIA1	0.11246	-0.00209	-0.01082	0.023	-0.13824	0.104568	0.173599	0.014602
GRIA2	0.584816	0.525452	0.017789	-0.12888	0.334199	-0.10567	0.052935	-0.21295
GRIA3	-0.4579	0.034753	0.188021	0.022245	-0.0837	0.133272	-0.18239	0.389836
GRIA4	-0.0509	-0.18031	0.328154	0.154272	0.301078	0.054578	-0.34352	0.431831
GRID1	0.16677	0.66659	-0.1719	0.105794	-0.15943	0.201754	-0.24607	0.213764
GRID2	0.160185	0.418504	0.076629	-0.10844	0.19594	-0.24034	-0.01983	-0.1072
GRIK1	-0.16126	0.264268	0.127184	0.148308	-0.08025	0.002612	-0.30412	-0.07611
GRIK2	0.233692	0.370035	-0.14013	-0.14519	0.11166	-0.01188	0.201275	-0.04477
GRIK3	0.222146	0.066328	-0.08305	-0.11576	-0.06336	0.188921	0.250226	0.086547
GRIK4	-0.13187	0.036512	-0.03136	0.362963	0.29327	0.177298	-0.30312	-0.52624
GRIK5	0.307766	0.394582	0.004214	-0.27427	0.224937	-0.15962	-0.22707	-0.06212
GRIN1	-0.53875	0.380279	-0.12758	-0.02359	-0.16119	0.143429	-0.06986	-0.27247
GRIN2A	-0.25179	0.280506	0.279237	-0.09016	-0.07614	-0.08402	-0.2305	0.179059
GRIN2B	0.398422	0.664036	0.410783	0.049586	0.038135	0.049132	-0.3295	-0.11469
GRIN2C	0.17312	0.085803	-0.01531	-0.13443	3.96E-05	-0.19227	-0.04182	0.026154
GRIN2D	0.21578	0.427451	0.013939	0.025095	-0.15178	0.218384	0.300451	-0.01909
GRIN3A	0.046074	0.074454	-0.12808	-0.09799	-0.24655	-0.24685	-0.53692	-0.119
GRIN3B	0.171665	-0.09629	0.002732	-0.45789	0.001262	-0.22355	-0.07359	0.396423
GRINA	0.219144	0.172752	0.620964	0.070031	0.167572	0.295389	0.172643	0.185219
GRINL1A	-0.02811	-0.29167	-0.31789	0.192118	-0.12742	-0.12085	-0.36069	-0.08502
GRIP1	NA	NA	NA	NA	NA	NA	-0.21002	-0.08873
GRIP2	NA	NA	-0.10147	0.093876	-0.26757	0.151256	0.060278	0.239952
GRIPAP1	0.229268	0.036524	0.257709	0.335554	0.367107	0.361124	-0.38105	0.022379
GRK1	-0.11031	-0.20949	-0.10624	-0.1516	0.053872	-0.28994	0.318021	0.097889
GRK4	-0.09785	-0.42481	-0.27997	-0.04218	-0.16634	-0.05587	-0.22348	0.082958
GRK5	-0.24235	0.038431	-0.0587	0.154532	0.151175	-0.33179	-0.54072	-0.38056
GRK6	-0.21385	0.67754	0.442868	0.302384	0.246974	0.394446	0.391497	0.245429
GRK7	-0.20932	0.003602	0.052537	0.172976	-0.12515	0.08866	-0.51965	-0.33022
GRLF1	0.337086	0.567747	0.023188	-0.09759	0.111581	0.05966	-0.35359	0.143868
GRM1	-0.00699	0.201347	0.110003	-0.09374	-0.18878	0.061614	0.022747	0.158771
GRM2	-0.16309	0.143972	0.032777	0.037307	0.211835	-0.00574	-0.11394	-0.19549
GRM3	0.001687	0.155198	-0.19048	-0.19126	0.014064	0.238749	0.013139	-0.15396
GRM4	0.069007	-0.20517	0.060997	-0.10758	0.193333	-0.21708	-0.15931	-0.06444

GRM5	-0.13738	-0.00859	0.089713	-0.0455	-0.01244	-0.09789	-0.26709	-0.24958
GRM6	0.271104	0.383238	NA	NA	NA	NA	0.230753	0.074666
GRM7	0.375111	0.604937	0.002897	0.089016	-0.20317	-0.01698	0.689223	0.684142
GRM8	0.21644	-0.163	0.199534	0.223111	0.03578	-0.06828	0.107422	-0.29678
GRN	-0.25551	-0.11031	0.181965	0.018778	0.076756	-0.01366	-0.1845	-0.2914
GRP	0.007406	0.03484	0.059449	-0.1684	-0.20038	-0.08672	0.034745	-0.28734
GRPEL1	0.011585	0.066582	-0.12254	-0.04152	-0.03172	-0.19943	0.198652	0.337936
GRPEL2	0.199837	-0.39228	-0.02667	-0.01019	-0.08421	-0.05816	-0.08123	0.178622
GRPR	0.012058	-0.01095	0.3752	-0.23397	0.368341	-0.07992	0.089414	-0.22707
GRRP1	-0.47177	-0.17456	0.0575	-0.0019	0.135179	0.072202	0.015348	0.111222
GRSF1	-0.06972	0.167293	-0.23701	-0.20461	0.119623	-0.17523	0.18244	-0.08348
GRTP1	0.082097	-0.00899	0.20384	-0.04236	0.251297	-0.30349	0.674445	0.546114
GRWD1	0.146769	0.558114	-0.05016	0.390545	0.187277	0.313781	0.555204	-0.0785
GRXCR1	NA	NA	-0.28179	-0.20483	0.131097	0.241542	NA	NA
GSC	0.186451	-0.15792	0.239036	-0.13269	0.030265	0.218676	-0.45437	0.085014
GSC2	NA	NA	0.276885	-0.16194	0.003307	0.117392	NA	NA
GSDMA	NA	NA	0.493671	0.04254	0.143638	-0.12867	NA	NA
GSDMB	NA	NA	0.448676	0.001347	0.019917	0.238115	NA	NA
GSDMC	NA	NA	-0.23772	-0.25851	-0.33691	-0.15422	NA	NA
GSDMD	NA	NA	0.499039	0.133963	-0.0481	0.06732	NA	NA
GSG1	0.369546	0.65352	0.064838	0.221974	-0.17481	-0.50491	0.347992	0.359265
GSG1L	0.203595	0.464038	-0.0133	-0.2636	0.228787	0.081579	NA	NA
GSG2	-0.02993	0.139653	-0.49072	0.326546	-0.18371	-0.05571	0.297678	0.119
GSK3A	0.243492	0.463823	-0.21611	0.169373	0.031372	0.083919	-0.63281	-0.56359
GSK3B	0.241736	0.546072	0.117832	-0.20812	0.06158	0.191029	-0.06438	0.038268
GSN	-0.38435	-0.30888	0.08691	-0.10885	-0.04364	-0.13567	-0.12826	-0.23296
GSPT1	0.516669	0.581615	-0.4808	0.128309	0.19698	-0.05102	0.397045	-0.03345
GSPT2	0.26635	-0.14282	0.111824	0.152465	0.138011	0.420836	-0.07068	0.073134
GSR	0.036865	0.43257	0.075361	0.20062	-0.08297	-0.40802	-0.0798	-0.45458
GSS	0.397052	0.22436	0.391065	0.375335	0.454745	-0.03397	0.550186	0.271733
GSTA1	0.156282	-0.03661	-0.24577	-0.14694	0.012358	-0.17051	-0.01813	0.459993
GSTA2	0.150039	0.053213	-0.19149	-0.15557	0.074923	-0.08102	0.034316	0.487983
GSTA3	0.130206	-0.34532	-0.18383	-0.11461	0.10941	-0.15191	0.194603	0.499367
GSTA4	-0.17129	-0.3479	-0.39898	-0.38754	-0.29196	-0.10127	-0.35505	-0.11415
GSTA5	0.316525	0.047811	-0.19533	-0.15746	0.055899	-0.09955	-0.35267	0.466663
GSTCD	0.32452	0.219043	-0.19651	-0.27607	-0.06865	0.081317	0.43902	0.022188
GSTK1	0.495606	0.280652	0.327756	-0.0808	-0.03512	-0.25772	0.309668	0.305321
GSTM1	0.096711	-0.20016	0.109547	0.027506	0.007579	-0.32528	-0.02096	0.185115
GSTM2	-0.01074	-0.12781	0.314464	0.022878	0.0653	0.171029	-0.19736	-0.03349
GSTM3	-0.17078	0.128246	0.069077	-0.12169	0.042144	-0.01978	-0.297	-0.15369
GSTM4	-0.03267	0.169306	0.554262	0.229525	0.219897	-0.31495	0.303685	0.330437
GSTM5	0.283148	-0.15334	0.162154	0.159252	-0.07709	-0.16913	-0.05097	0.171633
GSTO1	-0.0377	-0.66797	0.098002	-0.23276	-0.06912	-0.04846	-0.58521	-0.17255
GSTO2	-0.02135	0.502225	-0.14277	-0.22437	-0.1605	-0.32792	0.101272	-0.08779
GSTP1	-0.25932	0.512493	-0.02673	0.21963	0.057862	-0.30684	0.037361	-0.00225
GSTT1	0.050758	0.258476	0.016528	-0.33634	-0.17111	0.152124	0.047099	0.093262
GSTT2	0.102489	0.311968	-0.18905	-0.12612	0.05102	-0.04915	0.051327	0.193516
GSTTP1	NA	NA	-0.26525	-0.01189	0.157774	0.176507	NA	NA
GSTZ1	0.078912	0.576129	0.009004	0.021543	-0.22921	-0.2206	-0.27157	-0.15712
GSX1	NA	NA	0.014222	-0.22921	0.088711	-0.10853	NA	NA
GSX2	NA	NA	0.120446	-0.03635	0.375366	0.163684	NA	NA
GTDC1	0.26668	-0.50447	-0.01405	-0.16259	0.072087	-0.2946	-0.35954	-0.09784

GTF2A1	0.134331	0.531859	-0.3495	0.030755	-0.19722	0.005746	-0.48625	-0.44971
GTF2A2	0.256418	0.092978	-0.07989	-0.22971	-0.2921	-0.18144	-0.30067	-0.24222
GTF2B	-0.33968	-0.37075	0.259533	-0.24779	0.061937	-0.39717	0.286644	0.274715
GTF2E1	0.11169	-0.73502	-0.30466	-0.08938	-0.04801	-0.18301	0.413341	0.332001
GTF2E2	0.001658	-0.17952	0.060635	0.04748	-0.23033	-0.35966	0.2551	0.449693
GTF2F1	0.519654	0.497368	0.427895	0.27106	0.315688	0.216519	0.410996	0.243135
GTF2F2	0.475815	-0.23876	0.015287	0.085386	0.163818	0.023558	0.183718	-0.29326
GTF2H1	0.126904	-0.35419	-0.35421	-0.07557	-0.15069	-0.50114	-0.28311	-0.35109
GTF2H2	0.165327	-0.37032	NA	NA	NA	NA	-0.16833	-0.11618
GTF2H3	-0.04419	-0.43093	-0.17835	-0.10659	-0.00318	-0.50994	-0.21624	0.082087
GTF2H4	0.404863	0.415263	-0.0385	0.294441	0.097232	0.141022	0.388289	0.483263
GTF2H5	0.19097	-0.53761	0.047319	-0.26615	-0.13547	-0.20284	-0.15159	-0.25211
GTF2I	0.022671	-0.14569	0.096792	-0.07766	-0.08357	0.287471	0.137173	0.385598
GTF2IRD1	0.604803	0.416465	0.407469	-0.0254	0.410584	0.371789	0.505039	0.263685
GTF2IRD2	0.298765	0.039498	0.027003	-0.10802	-0.19882	0.116446	NA	NA
GTF3A	0.326107	-0.47532	0.129417	0.452032	0.16197	-0.35106	NA	NA
GTF3C1	0.251132	-0.29463	0.220563	0.155358	0.423849	0.071259	0.259831	0.089908
GTF3C2	0.106274	-0.24054	-0.1189	0.009641	0.090549	0.186925	0.297042	-0.18368
GTF3C3	0.262121	-0.31752	-0.09791	-0.28709	-0.07855	0.227353	0.233822	0.026479
GTF3C4	0.219677	0.006856	-0.32523	-0.16801	0.003655	-0.04926	0.021337	-0.19033
GTF3C5	0.285433	-0.05668	0.553126	-0.01449	0.457308	0.235775	0.401932	0.302643
GTF3C6	NA	NA	-0.31339	-0.00107	-0.04833	-0.3325	NA	NA
GTPBP1	0.101816	-0.04623	0.355317	-0.08233	0.210945	0.388738	-0.09198	0.191077
GTPBP10	0.083122	-0.07129	-0.11942	-0.33224	-0.14284	-0.46852	-0.14873	-0.03932
GTPBP2	0.334859	0.493299	-0.0472	-0.09792	0.186398	-0.15889	0.300541	0.402074
GTPBP3	0.061984	0.188125	-0.29221	0.200771	0.193206	-0.02815	-0.12282	0.236291
GTPBP4	-0.02544	-0.06169	-0.10938	0.080733	0.304251	0.106456	0.179487	0.110849
GTPBP5	-0.01656	-0.06411	0.248982	-0.04746	0.438238	0.340697	-0.1427	-0.25732
GTPBP6	0.35239	0.736231	0.267609	0.226726	0.30568	-0.08926	-0.27142	-0.35533
GTPBP8	-0.11177	0.462988	-0.03195	-0.0561	-0.10513	0.119575	-0.27353	-0.36206
GTSE1	0.135257	0.707332	-0.10245	0.258819	0.226613	0.119861	0.357993	0.128591
GTSF1	NA	NA	-0.08945	-0.00328	-0.24825	0.015786	NA	NA
GTSF1L	NA	NA	-0.04178	0.455284	0.115952	0.065677	NA	NA
GUCA1A	-0.15171	0.669589	-0.03706	-0.21511	-0.27872	0.020685	0.088289	-0.12328
GUCA1B	0.129223	0.504604	-0.35625	0.021696	-0.14352	-0.37544	-0.10723	0.143254
GUCA1C	-0.08372	-0.1158	-0.23924	-0.28118	0.266716	0.020181	0.223313	0.355766
GUCA2A	0.161884	0.247819	-0.04346	-0.41303	-0.04247	-0.5486	0.115688	0.019645
GUCA2B	-0.01804	0.157504	-0.06571	-0.3879	-0.07805	-0.32865	-0.0026	-0.13019
GUCY1A2	0.076226	-0.18321	0.142318	-0.06355	-0.41136	0.084135	-0.08131	0.308196
GUCY1A3	-0.31429	-0.41677	0.113713	-0.08034	-0.02924	0.191481	-0.37248	-0.39522
GUCY1B2	0.165769	-0.05769	0.138292	-0.10908	0.04481	0.011705	NA	NA
GUCY1B3	-0.35844	-0.36389	-0.04789	0.001156	-0.14078	0.367774	-0.38816	-0.21972
GUCY2C	0.563008	0.05741	0.073653	0.003743	0.066505	-0.18375	0.064552	0.111013
GUCY2D	-0.43181	0.543985	0.189772	-0.23424	0.194071	-0.16723	0.26101	0.071689
GUCY2F	-0.04106	-0.10122	0.300009	0.144433	0.061515	-0.13009	-0.51061	-0.08483
GUF1	0.187703	0.579847	-0.48523	0.023535	0.001451	-0.25068	0.30093	0.309007
GUK1	0.194284	0.197544	-0.06771	-0.1104	-0.25368	-0.43405	0.070463	-0.02913
GULP1	-0.13845	-0.47959	-0.02435	-0.24047	-0.0909	0.075553	0.029525	0.200949
GUSB	0.180474	0.126854	-0.21297	0.151251	0.067592	0.030458	0.161505	0.107905
GUSBL1	0.101507	-0.47524	0.150964	0.008214	0.240965	0.378104	NA	NA
GUSBL2	-0.03801	-0.21279	-0.00638	-0.1147	0.090116	0.24193	-0.25463	-0.35347
GYG1	NA	NA	0.074457	-0.19551	0.030831	-0.19637	-0.17375	-0.20463

GYG2	0.302769	-0.27073	0.234696	-0.12734	0.063366	-0.27082	0.172266	0.278909
GYLTL1B	0.34184	0.341194	0.353709	0.327794	0.220506	-0.02408	0.523864	-0.23126
GYPA	0.061447	0.54104	0.286702	0.06701	-0.03375	-0.23043	0.231735	0.494392
GYPB	0.051496	0.107978	-0.02557	0.103351	-0.02905	-0.10477	-0.01568	0.279187
GYPC	-0.34337	-0.45243	0.321962	0.046314	-0.01576	0.160087	-0.35467	-0.03728
GYPE	0.41519	0.336517	0.067423	0.042936	0.29492	-0.04642	-0.48565	0.509534
GYS1	0.237886	0.557294	0.238222	-0.08361	0.187089	0.258175	0.369984	0.177777
GYS2	0.187239	0.545689	0.154994	-0.50586	0.071161	-0.23393	-0.01147	0.298782
GZF1	-0.12951	-0.20276	-0.34413	-0.18264	0.053319	-0.2745	NA	NA
GZMA	0.066752	0.039445	0.323804	-0.21236	-0.32751	-0.13147	-0.58747	0.212941
GZMB	0.103213	0.425174	0.148889	-0.16801	-0.09689	-0.00183	-0.34445	0.320422
GZMH	0.002634	0.080719	0.108989	-0.05305	-0.26945	0.229511	-0.33536	0.104062
GZMK	-0.02327	0.083206	0.114239	0.552329	-0.32162	0.601607	-0.38837	0.165108
GZMM	0.359064	0.28466	0.165768	0.363761	-0.30072	0.307955	-0.52727	0.223294
H19	NA	NA	-0.00904	-0.23273	-0.14439	-0.02844	0.085833	0.033268
H1F0	-0.17797	0.677221	0.086921	-0.03948	-0.01984	-0.18633	0.393172	0.065655
H1FNT	-0.07717	-0.65716	0.066583	-0.39901	-0.29358	-0.38032	NA	NA
H1FOO	-0.03041	0.318284	0.356625	0.176776	-0.0795	0.118613	0.165273	0.287885
H1FX	-0.015	0.167073	0.158839	0.06733	0.121239	0.090331	0.322003	0.0689
H2AFB1	0.366492	0.113251	NA	NA	NA	NA	-0.04732	-0.08232
H2AFB2	NA	NA	-0.18098	-0.35336	-0.06207	-0.24623	0.008566	0.14393
H2AFJ	0.431412	-0.05011	0.386785	-0.14074	0.275222	-0.30275	-0.27848	-0.41345
H2AFV	0.052445	-0.42834	-0.51311	0.179993	-0.01678	-0.2958	-0.34207	0.117596
H2AFX	NA	NA	-0.14612	0.40476	-0.1034	0.100912	0.255144	0.143594
H2AFY	-0.05971	-0.60651	0.504657	0.270743	0.391974	0.379969	0.080515	0.27673
H2AFY2	0.326926	0.19279	0.040904	0.369239	0.164676	-0.0201	-0.11732	0.35898
H2AFZ	-0.19806	-0.51809	-0.24765	0.052925	-0.05206	-0.13149	0.111828	-0.12315
H2BFWT	0.126182	0.061734	NA	NA	NA	NA	-0.08293	-0.16472
H2BFXP	NA	NA	0.308107	-0.08403	0.098757	0.085864	0.080015	-0.38129
H3F3A	NA	NA	-0.18864	-0.02961	0.09051	-0.27221	0.573129	0.407792
H3F3B	-0.4158	-0.34273	0.187392	0.242997	0.061654	0.206563	-0.0741	0.228438
H6PD	-0.10529	0.132552	0.271773	-0.05921	0.333484	0.081865	0.351594	0.367828
HAAO	0.374023	0.575059	0.192732	-0.02776	0.000875	-0.29129	0.192843	0.09342
HABP2	0.140118	0.512581	-0.14776	-0.17389	0.116022	-0.00285	0.04011	-0.29016
HABP4	0.242499	-0.36555	0.281541	0.137614	0.225908	0.365509	-0.30123	-0.25754
HACE1	0.278335	0.546245	-0.22141	-0.28392	0.183915	0.111519	-0.48329	-0.45234
HACL1	0.463437	0.597329	-0.37088	-0.12932	-0.01912	-0.07933	NA	NA
HADH	-0.08023	-0.11041	-0.28888	0.103849	0.035934	-0.19454	NA	NA
HADHA	-0.16636	0.670254	0.483413	-0.103	-0.02316	-0.42664	0.259147	0.226925
HADHB	NA	NA	-0.12457	-0.29288	-0.21765	0.135953	-0.50404	-0.3632
HAGH	-0.05958	0.102049	0.361014	-0.05805	0.173333	-0.19561	-0.21137	-0.12856
HAGHL	-0.10701	0.439154	0.197437	0.012438	0.007657	0.249549	0.290698	0.264763
HAL	-0.11321	0.634219	-0.40822	0.287158	0.057528	0.065756	0.026665	0.668975
HAMP	-0.17962	0.267546	0.185609	0.285914	-0.03907	0.028036	-0.24468	-0.00356
HAND1	0.041775	-0.11998	0.289377	-0.10779	-0.08215	0.032798	-0.22631	-0.3565
HAND2	0.165732	0.756674	0.20531	-0.08751	-0.02793	-0.0826	-0.2883	-0.34392
HAO1	0.523591	-0.16034	-0.00415	-0.01009	-0.14101	-0.1302	-0.3783	-0.12072
HAO2	0.086683	0.530692	0.025878	0.045301	0.170557	0.114478	-0.36121	-0.5988
HAP1	0.350917	0.589025	4.83E-05	-0.17494	0.119858	0.191272	0.1687	0.116068
HAPLN1	-0.20039	-0.08482	0.266705	-0.17313	-0.32504	-0.11093	-0.06557	-0.17745
HAPLN2	0.278968	0.147763	0.243156	-0.14424	0.216943	0.153101	0.191973	0.010487
HAPLN3	-0.30222	-0.17242	0.148784	0.304438	0.00053	0.61987	-0.39273	0.151722

HAPLN4	0.138635	0.689754	-0.00137	-0.36595	0.08938	-0.16322	0.585382	0.604088
HARBI1	NA	NA	-0.19896	-0.08645	-0.11796	-0.40664	NA	NA
HARS	0.206962	0.735136	0.545726	0.199147	0.218964	0.111854	0.041949	-0.23793
HARS2	0.098449	-0.39427	0.41517	0.087427	0.218661	0.207745	0.389955	0.213977
HAS1	0.342102	-0.13456	0.311762	-0.20717	0.17202	0.08608	-0.44099	0.137689
HAS2	-0.21737	-0.23951	-0.01087	-0.03336	-0.28086	0.01351	-0.15206	0.074494
HAS3	0.132426	0.372936	0.053589	-0.36834	0.051046	0.018137	-0.17565	0.109899
HAT1	-0.08521	-0.62538	-0.08127	-0.08543	0.07212	-0.19369	-0.32491	-0.36776
HAVCR1	0.023381	0.010286	-0.23327	-0.34361	-0.25658	-0.14612	0.001087	0.011115
HAVCR2	-0.31014	0.176738	0.244322	0.093479	-0.34885	0.348237	-0.45886	0.112259
HAX1	0.160506	0.750053	0.260187	-0.0775	-0.1042	-0.24038	0.095354	-0.15791
HBA1	-0.02771	0.043812	0.017758	0.019062	0.082876	-0.18459	NA	NA
HBA2	NA	NA	0.007598	-0.0313	0.039345	-0.24391	-0.04951	-0.1767
HBB	-0.02815	-0.06474	-0.02139	0.095858	0.125689	-0.20063	0.048127	-0.11861
HBD	-0.14518	0.445211	-0.03923	0.006072	0.054527	-0.37605	-0.03056	-0.20144
HBE1	0.008414	0.287394	-0.25248	-0.20792	-0.01035	0.09941	-0.18891	-0.24928
HBEGF	-0.19676	-0.42273	0.404403	0.036864	0.10214	-0.03337	-0.21899	0.100923
HBG1	0.163253	0.069478	-0.1029	-0.11653	-0.21336	-0.50116	-0.48808	-0.35158
HBG2	-0.37086	-0.09609	NA	NA	NA	NA	0.036953	-0.27785
HBM	NA	NA	-0.0159	-0.18608	-0.00386	-0.41988	-0.25802	-0.23012
HBP1	0.184332	-0.45539	-0.14058	-0.12735	-0.27464	-0.29717	-0.24839	-0.2387
HBQ1	0.012073	-0.07161	-0.01749	-0.19834	0.045284	-0.33454	0.164489	-0.00153
HBS1L	-0.11162	-0.58598	-0.32176	-0.33049	-0.01784	-0.23919	-0.24853	0.092893
HBXIP	-0.00957	0.257634	0.286136	-0.2469	-0.09129	-0.0225	0.538619	0.400808
HBZ	0.074228	0.147751	-0.15609	-0.00534	0.152308	-0.10604	-0.33373	-0.45056
HCCA2	0.250799	0.325592	0.137097	-0.16856	0.21532	0.046293	-0.14456	-0.21685
HCCS	0.212179	-0.42511	-0.13174	-0.27437	0.076008	-0.46923	-0.22088	-0.30481
HCFC1	0.369892	0.180927	0.064321	0.305916	0.429807	0.05054	-0.26422	-0.62353
HCFC1R1	0.148223	0.379366	0.056331	-0.0812	-0.23053	-0.03792	-0.37483	-0.37118
HCFC2	-0.39319	-0.18431	-0.21756	-0.35344	-0.17631	-0.46657	-0.3199	0.058767
HCG27	-0.30144	-0.15118	0.064681	-0.29959	-0.09994	-0.12716	0.146351	0.336447
HCG4	NA	NA	0.26238	0.158213	-0.32454	-0.20251	-0.16713	0.062955
HCG9	-0.09237	0.606378	-0.37907	-0.20822	0.384941	0.09875	-0.63129	-0.24516
HCK	-0.42459	0.088416	0.348109	0.300599	0.1025	0.350152	-0.38516	0.543809
HCLS1	-0.49681	0.194409	0.140396	0.643351	0.008887	0.737067	-0.4204	0.177036
HCN1	0.134501	0.008459	-0.09053	-0.1705	-0.1495	-0.24255	0.07706	0.190961
HCN2	0.215545	0.516223	0.122724	0.001514	0.01657	0.31786	-0.37539	-0.56486
HCN3	0.038333	0.410986	0.137382	0.02337	0.434981	-0.01792	0.475509	0.437449
HCN4	0.486651	0.469718	0.029403	-0.01948	-0.07351	0.271538	-0.38929	-0.15152
HCP5	0.289553	0.105049	0.117216	-0.04443	0.038333	-0.11566	0.053899	0.09151
HCRT	0.148469	0.415453	0.280219	-0.07611	0.163301	-0.03517	-0.21065	-0.23957
HCRTR1	-0.00249	0.550679	-0.18721	0.185941	0.001411	-0.08406	-0.14504	0.060933
HCRTR2	0.068036	0.570176	-0.23345	-0.09193	-0.17638	-0.14789	0.523364	0.238423
HCST	-0.38146	0.244201	0.292584	0.252959	-0.39547	0.054666	-0.57748	0.144238
HDAC1	0.036273	0.422265	0.311026	0.144862	0.153716	0.168678	0.233817	0.05838
HDAC10	NA	NA	0.151929	0.204166	0.419632	0.437634	0.643697	0.537308
HDAC11	0.024906	0.678361	0.314522	-0.17394	0.422941	-0.19034	-0.10138	-0.26093
HDAC2	0.384464	-0.62161	-0.40131	-0.12834	0.146053	0.027545	0.189118	-0.30173
HDAC3	0.386474	0.290345	0.275822	0.095474	0.115677	0.092454	0.379046	0.025601
HDAC4	-0.17122	-0.25911	0.08471	-0.16146	-0.0237	0.136581	-0.53532	-0.32826
HDAC5	-0.04757	0.364353	0.419004	-0.05428	0.158588	0.080798	-0.199	-0.0399
HDAC6	0.253354	0.40593	0.258936	0.205959	0.259842	0.189423	0.075457	0.225674

HDAC7	NA	NA	0.209138	0.334054	0.225204	0.591008	NA	NA
HDAC8	-0.04199	-0.70532	-0.1493	-0.10234	-0.0464	-0.46608	0.010497	0.040136
HDAC9	0.008431	-0.10092	-0.08487	-0.31198	-0.22666	0.099186	-0.13399	-0.04851
HDC	-0.29096	0.12332	-0.05326	0.269461	0.055714	0.112116	-0.3206	0.418086
HDDC2	-0.28237	0.276112	-0.00512	0.0912	-0.09715	-0.14482	-0.51348	-0.42274
HDDC3	0.322856	0.457489	-0.01537	0.324361	0.092506	-0.18154	0.445774	0.344159
HDGF	0.005632	-0.07029	0.067389	0.147973	0.158677	0.087907	0.789738	0.552732
HDGF2	0.311519	0.29118	0.13383	0.267968	0.345375	0.043897	0.28515	-0.187
HDGFL1	0.014357	0.314247	0.31897	-0.18154	0.089672	0.162598	-0.27611	-0.40054
HDGFRP3	0.207514	-0.40478	-0.27722	0.03771	0.039737	-0.06265	-0.42293	-0.29064
HDHD1A	0.121526	-0.13223	-0.04263	0.040272	0.040912	-0.00092	-0.00979	0.051743
HDHD2	0.082274	0.389846	0.055196	-0.16202	0.004545	-0.06684	-0.5015	-0.56722
HDHD3	0.163548	0.386435	0.177422	-0.14033	0.162136	-0.21608	0.173131	0.265872
HDLBP	0.415274	0.47181	0.220695	0.215478	0.143389	-0.14367	0.318271	0.130712
HDX	NA	NA	-0.27779	-0.40584	-0.49621	-0.08827	NA	NA
HEATR1	0.335749	-0.17851	-0.30188	0.152047	-0.09727	0.338585	0.509068	0.132918
HEATR2	0.507548	0.509023	0.259696	0.33553	0.34968	0.254911	NA	NA
HEATR3	0.036378	-0.33415	-0.03492	-0.13139	0.254595	-0.05998	NA	NA
HEATR4	0.087423	0.188649	0.006752	0.274762	0.268781	0.30912	NA	NA
HEATR5A	NA	NA	-0.4247	-0.40942	-0.13062	-0.34459	NA	NA
HEATR5B	-0.11128	-0.36482	-0.23736	-0.1172	-0.00636	-0.16295	0.519586	0.406709
HEATR6	NA	NA	0.25407	0.178926	-0.04154	0.13104	NA	NA
HEATR7A	NA	NA	0.384656	-0.10497	0.407727	0.27904	NA	NA
HEATR7B2	NA	NA	-0.28229	-0.21071	0.025887	-0.1118	NA	NA
HEBP1	0.467398	0.048867	0.16587	0.009795	0.078525	0.129719	-0.42059	-0.04533
HEBP2	0.139567	0.374511	0.056882	-0.14525	0.054674	-0.1824	-0.28381	-0.49425
HECA	-0.0414	-0.55363	0.295506	-0.29418	0.316509	0.355332	-0.1911	-0.11083
HECTD1	-0.26255	-0.72898	-0.38896	-0.225	0.04165	0.226121	0.374254	0.358805
HECTD2	-0.3831	-0.23765	-0.27553	-0.25273	-0.07662	0.09898	-0.2754	-0.16653
HECTD3	-0.05108	0.394149	0.300409	-0.0438	0.106221	-0.09842	-0.06923	0.024722
HECW1	-0.35134	0.296758	0.053342	-0.02765	0.097777	-0.04277	-0.25032	0.029164
HECW2	-0.47208	-0.21684	-0.09858	0.573624	-0.08111	0.346285	-0.17855	0.038447
HEG1	NA	NA	0.030341	0.108647	0.014786	0.253002	NA	NA
HEL308	-0.28078	-0.70598	-0.24302	-0.16737	-0.24349	-0.18563	0.458162	0.610587
HELB	0.164479	0.214792	0.090401	0.12163	0.008987	0.116892	0.27417	0.29519
HELLS	-0.08794	-0.1082	-0.36768	0.250935	0.262033	0.087052	0.138849	-0.00386
HELT	0.02944	0.41716	NA	NA	NA	NA	0.473678	0.38082
HELZ	0.296592	-0.36739	0.344869	-0.16276	-0.14911	-0.084	0.283792	-0.12891
HEMGN	0.305873	0.517251	0.035662	0.160685	-0.20869	-0.00924	0.259757	0.279048
HEMK1	0.101274	0.036816	0.125292	0.235196	0.25703	-0.0759	-0.00772	0.54322
HEPACAM	NA	NA	0.049414	0.215075	-0.36208	0.203632	0.272409	0.173732
HEPACAM2	NA	NA	-0.05857	-0.17361	0.025302	-0.52689	NA	NA
HEPH	0.133951	0.322591	-0.1011	-0.05647	0.074562	-0.01199	-0.33039	-0.29817
HEPN1	-0.02366	-0.3116	0.068242	0.21363	-0.15191	-0.03049	NA	NA
HERC1	0.034965	-0.37629	-0.11726	0.110148	-0.03249	0.151966	0.287356	0.360741
HERC2	0.326597	-0.19354	0.318781	-0.10528	0.092222	0.285602	0.367234	0.156483
HERC2P2	0.319536	0.401009	-0.06358	0.038908	0.044662	0.130095	0.126043	0.329662
HERC2P4	NA	NA	0.06969	-0.01163	-0.03882	0.041338	-0.14748	-0.0871
HERC3	-0.19129	-0.03421	0.005593	0.108986	-0.32081	-0.17376	0.307404	0.335639
HERC4	0.060374	-0.50305	-0.31194	-0.20999	0.231133	-0.08859	-0.04489	0.101636
HERC5	-0.05388	0.150477	0.10052	-0.32087	-0.19887	0.056452	-0.24911	0.149635
HERC6	0.004376	-0.22755	0.085091	-0.34685	-0.26628	-0.00514	0.151432	0.537124

HERPUD1	-0.43303	-0.51106	0.025657	0.181961	0.089795	-0.17357	0.083401	-0.12209
HERPUD2	0.013973	-0.56773	0.0376	0.136726	0.164012	0.077624	-0.43409	-0.37544
HERV-FRD	0.217583	0.479378	-0.23064	-0.11547	0.144949	0.004272	-0.26392	0.218959
HES1	-0.03047	-0.30036	-0.16823	0.075053	-0.09279	-0.37835	0.373114	-0.27672
HES2	-0.00571	-0.07983	-0.15764	0.098061	0.07819	-0.16864	0.014091	-0.15434
HES3	-0.14568	0.225249	NA	NA	NA	NA	NA	NA
HES4	-0.35218	0.004399	0.028674	-0.13276	0.043892	-0.16817	-0.32125	-0.25981
HES5	-0.05404	0.361646	-0.15834	0.297576	0.049697	-0.02829	NA	NA
HES6	-0.12375	0.118821	-0.02033	-0.01207	0.266453	-0.3124	0.247488	-0.24642
HES7	0.309274	0.3163	0.156272	0.014172	-0.00164	0.240003	0.206735	0.199683
HESX1	-0.18856	0.024189	-0.37162	-0.06269	0.011781	-0.16133	0.319055	0.643026
HEXA	0.076453	0.290531	0.207984	0.031053	0.109098	-0.31097	-0.21422	-0.04316
HEXB	-0.22427	-0.5564	0.360189	0.170502	0.097122	-0.14458	0.452454	0.37278
HEXDC	0.021037	0.324343	0.219573	0.313557	0.147481	0.321158	-0.60246	-0.35996
HEXIM1	0.285717	0.604524	-0.16725	-0.08307	-0.17404	-0.17722	-0.31906	-0.06091
HEXIM2	0.161512	0.187272	0.13289	0.086068	-0.21256	-0.09692	0.034471	-0.15348
HEY1	-0.25643	-0.21024	-0.06978	0.114712	-0.14525	-0.19686	0.055814	-0.37828
HEY2	0.15811	0.341076	-0.12782	-0.05418	-0.11235	-0.11057	0.133778	-0.20248
HEYL	-0.24315	-0.00763	0.089894	0.072602	0.032474	0.13806	-0.03159	-0.14869
HFE	-0.02421	0.669457	0.35791	-0.29504	0.131781	-0.24886	0.129785	-0.22256
HFE2	0.208501	0.140861	0.023413	0.138885	-0.01264	0.001554	-0.08354	-0.22445
HFM1	-0.56754	0.094018	0.017334	-0.32926	0.097497	-0.25837	NA	NA
HGC6.3	NA	NA	0.190267	0.010924	0.159615	-0.16768	NA	NA
HGD	-0.24749	0.314559	-0.10213	0.031264	-0.15359	-0.50069	0.200947	0.147992
HGF	-0.02423	-0.16097	0.172143	0.030697	0.155287	0.093803	-0.36052	0.048944
HGFAC	0.156278	0.667265	-0.17422	0.024918	-0.04645	-0.47345	0.166156	-0.09053
HGS	0.145901	0.553039	0.376758	0.25157	0.415868	0.350666	0.129	0.187736
HGSNAT	NA	NA	0.303313	0.152126	0.219216	0.204622	NA	NA
HHAT	-0.00132	0.209481	-0.18096	0.091172	-0.14928	-0.08716	-0.44586	-0.24901
HHATL	0.297201	0.646794	-0.31449	-0.19634	-0.11945	-0.10158	NA	NA
HHEX	-0.32151	-0.4767	0.143647	0.673372	-0.14986	0.412152	-0.68858	-0.4308
HHIP	0.124405	0.122783	0.088268	-0.13023	-0.09746	-0.29274	0.246883	0.169809
HHIPL1	NA	NA	0.195851	0.054611	-0.11367	0.03266	NA	NA
HHIPL2	NA	NA	-0.39664	0.102431	-0.08913	-0.25443	NA	NA
HHLA1	0.125786	0.468366	0.296536	-0.02648	0.40295	0.156917	0.492275	0.543677
HHLA2	0.026835	0.059272	-0.20054	-0.22338	-0.12795	-0.50624	-0.11638	0.015136
HHLA3	-0.02101	-0.45781	-0.10967	0.077346	0.059846	-0.25027	0.15292	-0.10024
HIAT1	-0.38165	-0.5371	0.178258	-0.33292	-0.09972	-0.07452	-0.41102	-0.2606
HIATL1	-0.06056	-0.43964	0.231491	-0.10906	0.040952	0.161037	0.262045	0.392842
HIBADH	0.406986	-0.17405	-0.0794	-0.06891	0.066022	-0.19563	0.602841	0.269195
HIBCH	-0.02158	-0.06234	0.133003	0.044599	-0.11703	-0.13875	0.384192	-0.00983
HIC1	-0.35635	0.019071	0.2854	0.077676	-0.08394	0.17007	0.240528	0.420592
HIC2	-0.02651	0.057841	-0.11639	-0.29508	0.121388	0.007859	0.530404	0.066283
HIF1A	-0.45807	-0.30156	-0.217	-0.03117	-0.14946	-0.05703	-0.53607	-0.0158
HIF1AN	0.098517	0.14348	0.114252	0.285757	0.253907	0.013246	-0.28702	0.025137
HIF3A	0.085109	0.3384	-0.2512	-0.1178	0.242158	-0.02938	0.128461	0.024885
HIG2	-0.117	0.16362	0.092891	-0.10255	-0.05453	-0.22546	-0.31697	-0.30547
HIGD1A	-0.18313	-0.37863	-0.32894	-0.24149	-0.10832	-0.43297	-0.39137	-0.46834
HIGD1B	-0.35025	0.018774	-0.04497	0.26812	-0.05067	-0.18158	-0.19007	0.074559
HIGD2A	0.142097	0.541447	0.377006	-0.09114	-0.19871	-0.33342	-0.08956	-0.3495
HILS1	0.184716	0.648241	0.001102	-0.10283	-0.14782	0.101018	0.40735	0.12382
HINT1	0.088493	-0.50692	0.266977	-0.11648	0.03064	-0.33617	0.183976	-0.03666

HINT2	-0.16678	0.395088	0.084443	0.048543	-0.06428	-0.32864	-0.02063	-0.07356
HINT3	0.203908	0.087952	-0.12768	-0.22483	-0.13805	-0.42679	-0.15474	0.130397
HIP1	0.09876	-0.45902	0.153959	-0.07038	-0.0257	0.292071	0.34279	0.097961
HIP1R	0.346819	0.568314	-0.17229	0.427052	0.350921	0.07448	0.334123	0.293216
HIPK1	0.16949	0.756885	0.212202	-0.16653	0.240165	-0.16153	-0.69437	-0.46048
HIPK2	-0.47694	0.072383	-0.23827	0.407211	0.24081	0.173414	-0.31407	-0.1184
HIPK3	0.035579	0.473417	-0.12814	-0.26206	-0.03302	0.025282	0.052888	0.200377
HIPK4	0.365774	0.581416	0.060769	-0.00761	-0.18	0.459326	0.227563	0.00766
HIRA	0.150022	0.739999	0.00519	0.160213	0.284983	0.211324	-0.27626	-0.16619
HIRIP3	0.009582	0.294636	-0.17315	0.252623	0.334276	-0.17369	-0.24458	-0.36369
HISPPD1	0.647983	0.50165	-0.24762	-0.20877	-0.14147	-0.30567	0.567592	0.320235
HISPPD2A	-0.00167	0.136655	0.148762	0.0912	0.140014	0.146467	0.516843	0.707009
HIST1H1A	-0.13333	0.335535	0.121693	0.283531	-0.29813	0.143187	-0.39203	-0.33171
HIST1H1B	0.407416	0.011054	-0.21024	0.066352	-0.01367	-0.2486	-0.13411	-0.05532
HIST1H1C	-0.03768	0.176112	0.071475	-0.25361	-0.3306	-0.26007	-0.37944	-0.25327
HIST1H1D	-0.02742	-0.14385	0.076874	-0.03462	-0.29143	-0.27546	-0.09703	-0.15695
HIST1H1E	0.348797	0.349238	0.052525	-0.24522	-0.36388	-0.32763	-0.00483	-0.1234
HIST1H1T	-0.12146	0.408915	-0.11278	0.207699	0.348383	-0.21656	0.112736	0.006471
HIST1H2AA	0.220618	0.533878	-0.03335	-0.13052	-0.13645	0.169959	0.239797	0.198069
HIST1H2AB	-0.39595	-0.48589	0.032577	-0.06384	0.001667	-0.3222	-0.09273	-0.08964
HIST1H2AC	-0.10911	-0.3811	0.005128	-0.24351	-0.42304	-0.35568	0.025737	-0.03362
HIST1H2AD	0.066218	-0.49538	0.017228	0.11015	-0.39044	-0.41909	0.397912	-0.05623
HIST1H2AE	-0.03303	0.588881	-0.09394	-0.1322	-0.32858	-0.47432	0.371335	0.243352
HIST1H2AG	-0.07256	-0.12524	0.225036	0.012702	0.044073	-0.33993	0.365894	0.142294
HIST1H2AH	0.56939	0.418148	0.175754	0.083021	-0.06035	-0.30022	-0.0243	0.033832
HIST1H2AI	0.262006	0.299102	NA	NA	NA	NA	-0.57515	-0.3039
HIST1H2AJ	0.228516	0.494324	0.217217	-0.16799	-0.09221	-0.34247	0.351411	0.546878
HIST1H2AK	0.450327	0.550828	0.222234	-0.00443	-0.15473	-0.29839	0.050019	0.229717
HIST1H2AL	0.12515	0.656519	-0.36087	0.091837	-0.01955	-0.34502	NA	NA
HIST1H2AM	-0.45177	0.134663	-0.03104	-0.14167	-0.19242	-0.28532	0.118108	-0.1183
HIST1H2BA	0.434951	0.033571	0.558127	-0.16072	-0.00367	-0.02465	0.338223	0.1308
HIST1H2BB	-0.2778	-0.11954	-0.12351	0.024401	-0.40327	-0.43151	0.047223	0.221507
HIST1H2BC	-0.01178	-0.22983	-0.0727	-0.03048	-0.36171	-0.36636	-0.5166	-0.34502
HIST1H2BD	-0.15265	0.109821	-0.17879	0.001249	-0.45034	-0.46788	0.172084	-0.08986
HIST1H2BE	-0.13749	-0.31836	-0.07734	-0.03917	-0.45462	-0.38793	-0.42932	-0.38305
HIST1H2BF	0.310349	-0.05299	-0.08427	-0.01086	-0.44794	-0.39378	0.283286	0.10332
HIST1H2BG	-0.00766	-0.09397	-0.08168	-0.05126	-0.38716	-0.35994	0.109858	-0.07219
HIST1H2BH	0.45254	0.319909	-0.08205	-0.11387	-0.45011	-0.30651	0.001781	-0.21514
HIST1H2BI	0.302705	0.439201	-0.01593	-0.02981	-0.33726	-0.21567	0.329662	0.182916
HIST1H2BJ	-0.00483	0.30044	-0.13731	-0.07346	-0.45598	-0.35651	-0.45725	-0.44001
HIST1H2BK	-0.31977	-0.10975	-0.02428	0.020657	-0.42321	-0.14867	-0.22396	-0.09046
HIST1H2BL	-0.08729	-0.14796	-0.08826	0.069311	-0.45074	-0.29948	0.22385	0.083039
HIST1H2BM	0.349039	0.320715	-0.00525	-0.03086	-0.33529	-0.22204	-0.32373	-0.36716
HIST1H2BN	-0.36159	0.183361	-0.15445	0.078124	-0.4054	-0.29403	NA	NA
HIST1H2BO	0.371488	0.730804	-0.11263	0.064175	-0.45099	-0.32126	-0.11425	0.180045
HIST1H3A	0.216943	0.424226	0.187343	-0.14635	0.198064	-0.07372	-0.21647	-0.07909
HIST1H3B	-0.12472	0.023028	-0.05759	-0.04896	-0.24688	-0.16051	-0.28114	-0.14295
HIST1H3C	0.010167	-0.14016	0.395155	0.025452	0.091845	0.248152	-0.18919	-0.25752
HIST1H3D	0.005007	0.534205	-0.11768	0.05576	-0.31102	-0.24547	-0.13614	-0.22104
HIST1H3E	-0.13868	-0.14613	-0.16219	-0.02035	0.037769	-0.30529	NA	NA
HIST1H3F	0.193371	0.021771	-0.31434	-0.2004	-0.37212	-0.31603	-0.28407	-0.25218
HIST1H3G	0.293248	0.603088	0.26672	0.128565	-0.08371	0.044207	-0.06033	-0.10749

HIST1H3H	-0.17699	-0.2261	-0.252	-0.22754	-0.15532	-0.40548	0.184938	0.041961
HIST1H3I	0.235447	0.466684	-0.29262	0.286342	-0.05822	0.053822	0.308044	0.501438
HIST1H3J	0.428288	0.269503	-0.12219	-0.13787	-0.16139	-0.28652	-0.12949	0.003071
HIST1H4A	0.023183	0.343847	-0.25492	-0.20329	-0.17205	-0.21224	-0.39701	0.043829
HIST1H4B	0.401705	0.119725	-0.05405	0.101536	-0.0215	-0.23548	-0.01677	-0.07247
HIST1H4C	0.115791	-0.43553	-0.0789	0.079966	-0.18995	-0.10098	0.025987	-0.18965
HIST1H4D	0.474144	0.765993	-0.12959	0.040845	-0.1854	-0.31409	0.205008	0.032459
HIST1H4E	0.251274	-0.31585	-0.0943	-0.27389	-0.29333	-0.19172	0.004595	-0.10885
HIST1H4F	0.16022	0.362071	-0.24208	-0.28747	-0.18981	-0.49845	0.438251	0.28769
HIST1H4G	0.295372	0.29982	-0.24596	0.077913	-0.0585	-0.24113	0.058568	-0.11756
HIST1H4H	-0.11607	-0.34172	-0.2789	-0.20806	-0.35224	-0.4996	-0.18303	0.19714
HIST1H4I	0.297548	-0.19314	-0.19378	-0.33703	-0.2489	-0.43545	-0.20089	-0.15644
HIST1H4J	NA	NA	-0.19705	-0.29667	-0.15933	-0.34854	-0.18487	-0.23043
HIST1H4K	0.442481	-0.00456	-0.28517	-0.25929	-0.24974	-0.40765	NA	NA
HIST1H4L	0.404146	0.101063	-0.05875	0.097444	-0.11124	-0.16228	-0.27234	-0.28513
HIST2H2AB	-0.17495	-0.0925	-0.06369	-0.03773	-0.4369	-0.45015	-0.39789	-0.01118
HIST2H2AC	-0.4085	0.196416	-0.05797	-0.10226	-0.42196	-0.33157	0.015207	0.143366
HIST2H2BE	-0.35557	-0.42541	-0.02006	0.228665	-0.33102	-0.30803	0.108296	0.215508
HIST2H2BF	-0.33195	-0.11975	NA	NA	NA	NA	0.26542	0.489131
HIST2H3C	0.339725	0.073334	NA	NA	NA	NA	-0.06692	0.020206
HIST2H4A	0.070825	-0.40736	NA	NA	NA	NA	0.017914	0.227382
HIST3H2A	0.194568	0.727989	0.252658	-0.12871	-0.14331	-0.28472	0.413712	-0.12917
HIST3H2BB	-0.18146	0.278738	-0.15015	0.046798	-0.40684	-0.35134	0.105171	0.423616
HIST3H3	0.389536	0.587729	0.056429	0.127273	0.017109	0.405115	-0.50452	-0.59596
HIST4H4	NA	NA	-0.07584	-0.20481	-0.03552	0.21032	0.188515	0.320594
HIVEP1	0.115904	-0.51049	-0.1159	-0.3294	-0.17392	0.130159	0.389084	0.390429
HIVEP2	0.013564	0.152805	-0.06268	0.089517	0.40779	0.172907	-0.56379	-0.49058
HIVEP3	-0.30973	-0.01922	0.284517	0.219054	-0.09582	0.494424	-0.04503	0.071511
HJURP	NA	NA	-0.00423	0.180223	0.06672	0.032572	NA	NA
HK1	-0.57745	0.379552	0.155581	0.247156	0.281757	0.414924	0.129584	0.090161
HK2	-0.33257	-0.15418	-0.29624	-0.02609	-0.18358	0.014383	-0.35909	-0.48621
HK3	-0.45198	-0.12831	0.492293	-0.1042	-0.16198	0.256496	-0.18496	0.45266
HKDC1	-0.2366	0.108769	0.26617	-0.31425	0.474319	-0.20792	0.410221	0.409777
HKR1	0.390554	0.731188	0.06739	-0.06194	0.100166	0.384814	-0.26294	-0.08774
HLA-A	0.079727	-0.21079	0.405545	0.067118	0.116378	0.141595	0.323975	0.501654
HLA-B	0.187346	-0.30766	0.411684	0.125951	0.303305	0.171855	NA	NA
HLA-C	-0.13886	-0.38762	0.262881	0.210449	0.26147	0.181258	-0.45113	-0.19317
HLA-DMA	0.000394	0.456635	0.587431	0.174184	-0.3185	0.486982	-0.55165	0.106656
HLA-DMB	-0.21807	0.157653	0.289078	0.32357	-0.47017	0.390108	-0.65848	0.309416
HLA-DOA	-0.13849	-0.00061	0.43676	0.337802	-0.43877	0.553862	-0.37992	0.030972
HLA-DOB	0.039794	0.355557	0.25308	0.669583	-0.35098	0.419989	-0.21789	0.204437
HLA-DPA1	-0.18044	-0.23747	0.417477	-0.01592	-0.42615	0.410536	-0.54392	-0.04133
HLA-DPB1	-0.28546	0.212948	0.458074	0.154995	-0.30376	0.496209	-0.49125	0.301074
HLA-DPB2	NA	NA	0.314206	0.26676	-0.37132	0.252613	-0.27943	0.319255
HLA-DQA1	NA	NA	-0.05583	-0.18098	-0.38281	0.149125	-0.46795	0.25191
HLA-DQA2	0.328134	0.485464	-0.11636	-0.0409	-0.40759	0.199679	-0.07848	0.215957
HLA-DQB1	-0.28684	-0.18788	-0.04158	-0.3023	-0.25898	0.183041	0.080477	0.250519
HLA-DQB2	0.009661	0.084247	0.100676	-0.12779	-0.32663	0.283178	-0.24927	0.10557
HLA-DRA	-0.18851	-0.31406	0.357955	0.150314	-0.41875	0.401918	-0.44129	0.270861
HLA-DRB1	0.144876	0.128611	0.463675	0.000931	-0.35615	0.649981	-0.62881	-0.37253
HLA-DRB3	-0.141	-0.25171	0.55895	0.002242	-0.32206	0.472452	-0.58393	-0.2219
HLA-DRB4	0.338088	0.333801	0.471083	0.219854	-0.33492	0.552476	-0.28478	0.382663

HLA-DRB5	0.168552	0.227444	0.231555	0.098801	-0.2639	0.395141	-0.24543	-0.36078
HLA-DRB6	NA	NA	0.307531	-0.03997	-0.15495	0.024073	-0.66574	-0.16646
HLA-E	-0.05377	0.10735	0.435825	0.013615	0.231571	0.114788	-0.50913	-0.32859
HLA-F	-0.03636	-0.34985	0.317869	-0.01966	0.207195	0.191038	-0.07389	-0.01714
HLA-G	0.062018	-0.07033	-0.10341	0.156633	0.249648	-0.01424	-0.24726	-0.10495
HLA-H	NA	NA	0.358534	0.087318	0.282894	0.155397	-0.0177	0.134364
HLCS	-0.14093	-0.39745	-0.10341	0.102937	-0.07407	-0.09446	-0.2772	-0.21892
HLF	0.374318	-0.17941	-0.19538	-0.25954	0.164072	0.009334	-0.17746	-0.25122
HLTF	-0.29544	-0.53983	-0.0492	0.001554	0.054812	0.194857	NA	NA
HLX	NA	NA	0.296069	0.001217	0.099511	0.355141	NA	NA
HM13	0.32961	-0.30176	0.259763	0.166045	0.12923	0.241212	0.503639	0.412109
HMBOX1	0.123674	-0.25091	-0.04704	0.094326	-0.16194	-0.07304	0.042891	-0.07201
HMBS	-0.01575	0.339171	-0.18758	0.231652	-0.10371	-0.18949	-0.01547	-0.08175
HMCN1	0.15322	-0.44975	-0.03454	-0.13299	-0.29753	0.10943	-0.39565	-0.16036
HMG20A	0.41009	0.234287	-0.09073	-0.12779	-0.072	0.172678	0.633615	0.466934
HMG20B	-0.11437	0.646164	0.152403	0.013657	0.149216	-0.21984	-0.36886	-0.35555
HMGA1	0.296321	0.635775	-0.0524	0.221303	0.04119	0.148511	0.440189	0.246193
HMGA2	-0.14408	0.34532	-0.23008	-0.11683	-0.1191	-0.33858	0.034277	0.31893
HMGB1	NA	NA	-0.33628	0.093342	0.143317	-0.06172	-0.39228	-0.42731
HMGB2	-0.16733	-0.57424	-0.41058	0.319813	0.112437	-0.05982	0.11057	-0.2748
HMGB3	-0.22469	0.304789	-0.41379	-0.0478	0.214918	0.081175	0.386926	0.115923
HMGB4	0.526289	0.45509	0.138494	-0.10231	-0.21485	0.08247	-0.16153	-0.26819
HMGCL	-0.15859	0.41549	0.20214	-0.29319	0.032169	-0.24014	0.290816	-0.00093
HMGCLL1	0.143412	-0.14892	0.302324	-0.18703	0.019304	-0.07338	0.227262	-0.2893
HMGCR	0.093801	0.234756	-0.1161	-0.17398	0.03275	0.043689	0.252392	0.052421
HMGCS1	-0.04474	0.40769	-0.32067	0.027861	-0.0889	0.051491	0.171441	0.006693
HMGCS2	-0.1559	0.047732	-0.13287	0.020401	-0.2057	-0.0472	0.296827	-0.02769
HMGN1	NA	NA	-0.43853	0.352087	-0.15157	-0.18937	0.222773	0.248531
HMGN2	-0.64305	-0.45972	-0.3318	0.386592	0.163983	-0.01549	-0.41383	-0.52131
HMGN3	-0.03831	-0.55791	-0.19495	-0.03439	-0.24896	0.159407	-0.76938	-0.39882
HMGN4	0.187213	-0.22684	-0.10535	-0.11484	-0.02917	0.127477	-0.41237	-0.34918
HMGXB4	NA	NA	-0.04249	-0.21935	0.238029	-0.2144	NA	NA
HMHA1	0.04676	0.288367	NA	NA	NA	NA	-0.59731	-0.07292
HMHB1	-0.2048	-0.0766	0.392064	0.291932	0.169645	0.148464	NA	NA
HMMR	-0.09208	-0.13155	0.056694	0.115372	0.108219	-0.14917	0.200832	-0.04358
HMOX1	-0.45777	-0.26488	0.057448	0.04583	-0.04122	-0.03088	-0.37523	0.104786
HMOX2	0.086082	-0.08371	0.082722	-0.01844	0.185673	0.214515	-0.18964	-0.18026
HMP19	-0.15152	0.109049	-0.144	0.11758	-0.29075	-0.10918	-0.5133	-0.32901
HMX1	-0.16928	0.08186	-0.11777	-0.1594	0.080536	0.090305	0.320743	0.266902
HMX2	0.324672	-0.04613	0.116261	-0.23035	0.128136	0.175172	0.353121	0.361594
HN1	-0.23143	0.100172	0.183771	0.174729	-0.09782	-0.01591	-0.16082	-0.21172
HN1L	-0.21687	0.374982	-0.34112	0.362872	0.320437	0.314091	NA	NA
HNF1A	NA	NA	0.076204	0.09098	0.22117	0.038237	NA	NA
HNF1B	NA	NA	-0.12886	-0.01049	0.310425	0.01529	NA	NA
HNF4A	0.394324	0.307769	0.123373	-0.19491	0.411849	0.094496	0.279303	0.258746
HNF4G	0.079037	-0.02701	-0.11948	0.008781	-0.33641	-0.39936	0.418947	0.510825
HNMT	0.198347	-0.55389	-0.01633	-0.20404	-0.21463	-0.27343	-0.11218	-0.24123
HNRNPA0	NA	NA	0.042936	0.314279	0.163005	0.471583	NA	NA
HNRNPA1	NA	NA	-0.18134	0.311212	0.152752	0.128704	NA	NA
HNRNPA1L	NA	NA	-0.09125	0.332947	0.176139	0.213337	NA	NA
HNRNPA2B	NA	NA	-0.21191	0.182594	-0.00884	-0.0164	NA	NA
HNRNPA3	NA	NA	-0.09968	0.327207	0.084047	0.19683	NA	NA

HNRNPAB	NA	NA	0.100075	0.213142	0.167288	0.152657	NA	NA
HNRNPC	NA	NA	-0.43171	0.34084	-0.12588	-0.01334	NA	NA
HNRNPCL1	NA	NA	-0.29952	0.276232	0.01962	0.122964	NA	NA
HNRNPD	NA	NA	-0.3562	0.354815	0.293124	0.255305	NA	NA
HNRNPF	NA	NA	-0.41783	0.259087	0.190057	0.188073	NA	NA
HNRNPH1	NA	NA	-0.09215	0.431938	0.09766	0.32346	NA	NA
HNRNPH2	NA	NA	-0.2776	-0.16021	0.083468	-0.09018	NA	NA
HNRNPH3	NA	NA	-0.41997	0.261836	0.125579	0.077172	NA	NA
HNRNPK	NA	NA	-0.2532	0.27409	0.275153	-0.23705	NA	NA
HNRNPL	NA	NA	0.063101	0.282524	0.167131	0.104979	NA	NA
HNRNPM	NA	NA	-0.22831	0.282011	0.441655	-0.05057	NA	NA
HNRNPR	NA	NA	-0.43248	0.369363	0.069264	0.049927	NA	NA
HNRNPU	NA	NA	-0.07815	0.263782	0.120765	0.39369	NA	NA
HNRNPUL1	NA	NA	-0.00266	0.193237	0.10359	0.106292	NA	NA
HNRNPUL2	NA	NA	-0.12373	0.317815	0.056093	0.149389	NA	NA
HNRPDL	-0.38353	-0.50214	-0.28102	0.200564	0.207651	0.120722	0.084627	0.114063
HNRPLL	0.310399	-0.37351	-0.09547	-0.05094	-0.04246	-0.02879	-0.458	-0.40584
HOMER1	0.200637	-0.19059	-0.21804	-0.16348	-0.1737	0.171364	0.484134	0.118319
HOMER2	0.075866	0.127671	-0.0391	-0.13068	-0.07497	-0.07399	0.005358	-0.11887
HOMER3	-0.37067	-0.52033	-0.01898	-0.00566	-0.04292	0.102037	-0.04419	0.054991
HOMEZ	0.289565	0.144751	-0.03258	-0.06479	-0.01987	0.07262	NA	NA
HOOK1	0.120765	0.222506	-0.06539	-0.12235	-0.09846	-0.15314	0.276781	-0.07009
HOOK2	0.258101	0.144422	0.191885	-0.06656	0.339306	0.007213	0.366373	0.541494
HOOK3	-0.13923	-0.17136	0.434531	-0.22665	0.029149	0.23401	0.516504	0.152806
HOPX	NA	NA	0.056029	0.120319	-0.18882	-0.23803	NA	NA
HORMAD1	-0.19725	0.13264	-0.21054	-0.37253	-0.32931	0.161775	0.022607	-0.36387
HORMAD2	-0.04486	0.343943	0.065943	-0.29779	0.132693	0.204358	0.241585	0.289822
HOXA1	0.104927	0.086317	-0.05675	0.155927	0.095861	-0.20534	0.192547	0.276346
HOXA10	0.12637	-0.35096	-0.09089	0.031443	0.005375	-0.11945	0.181573	-0.42658
HOXA11	0.214317	-0.13544	-0.03133	0.211367	-0.01132	-0.29812	0.265692	-0.18671
HOXA13	0.184534	-0.40045	-0.12256	-0.12047	-0.05854	-0.21184	0.181697	-0.34263
HOXA2	0.088895	-0.09103	-0.27891	-0.16911	0.098557	-0.01202	-0.0138	-0.0725
HOXA3	0.170576	0.657865	-0.0778	-0.06589	0.165576	0.119927	0.031288	-0.30676
HOXA4	0.198811	-0.33524	-0.29891	-0.11766	0.101757	-0.0354	0.393344	0.262054
HOXA5	0.246836	-0.00915	-0.0473	0.008078	0.124673	0.193848	0.075403	-0.10831
HOXA6	0.011662	0.229499	0.101967	-0.20815	0.112343	0.075119	-0.51612	-0.55815
HOXA7	0.185922	0.051695	-0.12046	0.166148	-0.02078	0.134568	0.296191	0.017017
HOXA9	0.197665	0.059877	-0.21883	0.120621	0.008112	-0.26477	0.19652	-0.05512
HOXB1	0.132727	0.371653	-0.03887	0.107966	-0.03021	0.02536	-0.22303	-0.334
HOXB13	0.036684	-0.18044	-0.1106	0.161683	-0.11837	-0.16486	0.064822	-0.19653
HOXB2	-0.30753	-0.21048	-0.18321	0.089337	0.004943	-0.13385	-0.46694	-0.2857
HOXB3	-0.13681	-0.51945	-0.35336	-0.22416	-0.18054	0.310703	0.314726	0.342549
HOXB4	-0.2518	0.057242	-0.00069	0.237177	-0.43265	0.151623	0.176072	0.351546
HOXB5	-0.06693	0.205645	-0.033	0.134561	0.002197	0.102611	0.145581	-0.20085
HOXB6	-0.21124	0.487017	0.034887	0.144965	-0.24019	-0.17285	0.195065	-0.21277
HOXB7	-0.01836	0.220209	-0.22298	0.308615	-0.2411	-0.37002	0.543009	0.398148
HOXB8	-0.06876	0.446031	0.060112	0.001599	-0.05889	0.396617	0.147252	0.176991
HOXB9	-0.03742	0.066656	0.189659	0.371112	1.73E-05	0.270635	0.328362	0.003389
HOXC10	0.399323	0.641967	-0.26209	0.062732	0.200512	-0.00514	-0.41057	-0.44069
HOXC11	0.101377	0.228387	0.214285	-0.05818	0.110578	-0.05319	-0.44159	-0.15686
HOXC12	-0.08913	0.206011	-0.12455	-0.03373	0.023429	0.225361	-0.53557	0.107417
HOXC13	0.00819	0.534049	0.12289	-0.41255	0.058841	0.017852	0.120838	0.075614

HOXC4	-0.51729	-0.06213	-0.10267	0.003605	-0.1796	0.028242	0.008394	0.165493
HOXC5	-0.4495	-0.17272	-0.10428	-0.0999	-0.06346	-0.4378	0.223687	0.141181
HOXC6	-0.38396	0.184588	0.065747	-0.28526	0.010647	0.177987	0.063369	0.487426
HOXC8	-0.19333	0.09034	0.028429	0.080319	-0.02085	-0.04707	0.01128	0.58722
HOXC9	-0.20332	0.464221	-0.21467	-0.03461	-0.07228	-0.00691	-0.0525	0.256005
HOXD1	-0.19786	-0.06133	-0.05708	-0.22081	-0.33896	0.005041	-0.1665	0.327072
HOXD10	0.315156	0.10886	0.229934	0.106261	-0.00953	0.092478	-0.15582	-0.18765
HOXD11	0.271278	0.329222	0.220632	0.06887	0.011593	0.014543	-0.09777	-0.10035
HOXD12	-0.00802	0.241446	-0.05305	0.240459	-0.12678	-0.2802	-0.67408	-0.36468
HOXD13	0.291518	-0.09788	-0.00418	0.101784	-0.16034	-0.05003	-0.46362	-0.22233
HOXD3	0.079341	0.479445	-0.03577	-0.18095	-0.07093	-0.01091	0.449898	0.168862
HOXD4	0.073638	0.019175	0.158883	0.030122	-0.02524	0.093593	-0.44386	0.223277
HOXD8	-0.11939	-0.50496	-0.00112	0.163936	-0.07519	-0.2006	-0.2758	-0.28151
HOXD9	0.32366	0.155233	0.346035	-0.04949	-0.00862	0.01802	-0.16891	-0.23644
HP	0.345723	-0.02956	0.135443	-0.03641	-0.02683	-0.26417	0.148515	0.280344
HP1BP3	-0.0294	-0.55329	0.086404	0.077653	0.12474	0.234516	0.297738	0.456758
HPCA	-0.13202	0.439902	0.284012	0.171667	0.226823	-0.20145	0.497217	0.312646
HPCAL1	-0.31778	0.631164	0.322967	0.31198	0.323279	-0.00885	0.17661	0.32736
HPCAL4	-0.22383	0.285272	0.027007	0.162987	-0.03955	0.101852	-0.20552	-0.33239
HPD	-0.02048	-0.22776	0.329029	0.077787	0.109611	-0.30429	-0.4084	-0.27536
HPDL	NA	NA	-0.0701	0.142126	0.17384	0.191331	NA	NA
HPGD	NA	NA	-0.10624	-0.39986	-0.13321	-0.58106	-0.2894	-0.07951
HPN	-0.10852	0.349473	0.051002	0.404087	-0.34699	-0.22247	0.188103	-0.00382
HPR	0.281689	0.244766	-0.07079	-0.12674	-0.04972	-0.11765	0.184924	-0.13454
HPRT1	0.006953	-0.61483	-0.32151	-0.01067	0.053929	-0.2526	0.411585	0.336617
HPS1	0.002018	0.337496	-0.02879	-0.10601	0.109764	-0.07679	0.21678	0.331406
HPS3	-0.1267	-0.62396	-0.3	-0.21679	-0.05774	-0.23587	0.321551	0.385989
HPS4	0.056525	0.208193	0.230142	0.337356	0.386281	0.265946	0.441453	0.370531
HPS5	-0.09481	0.012871	-0.02488	0.007474	-0.18287	0.242983	-0.1136	0.455661
HPS6	-0.16713	0.154958	-0.22314	0.098031	0.296613	0.106555	0.180511	0.018014
HPSE	-0.56662	-0.16417	-0.07464	-0.07619	-0.44009	-0.41606	-0.16019	-0.27478
HPSE2	0.501896	0.111598	-0.07921	-0.09846	-0.16843	0.287957	-0.46412	-0.11796
HPX	-0.41104	-0.1413	-0.08253	-0.26694	-0.3052	0.003824	-0.12642	0.463037
HR	0.254369	0.558697	0.137224	0.045236	0.287872	-0.00872	0.090599	-0.06025
HRAS	-0.09475	0.213941	0.189244	0.060043	0.120367	-0.17315	-0.03933	-0.0532
HRASLS	0.208656	-0.00361	-0.30967	0.108855	-0.07382	-0.49253	-0.1324	-0.04846
HRASLS2	-0.28625	-0.02727	-0.1376	-0.39828	-0.16326	-0.28873	-0.00918	0.118272
HRASLS5	-0.29352	0.011813	-0.20278	0.109804	-0.21575	-0.00307	0.402527	0.293443
HRC	0.066725	0.310094	-0.00981	-0.19032	-0.04915	0.098843	-0.21451	0.074099
HRCT1	NA	NA	0.077478	-0.13653	0.080969	-0.14191	NA	NA
HRG	0.133693	0.031225	-0.35051	0.021798	0.144116	-0.09377	-0.08649	0.216858
HRH1	-0.07026	0.142602	0.101223	-0.2014	-0.063	0.043863	-0.50054	-0.02997
HRH2	-0.06877	0.077009	0.046055	-0.22583	-0.00261	-0.11069	-0.36482	0.237035
HRH3	-0.18713	0.011995	0.237708	-0.19008	0.100479	0.208855	0.096762	-0.02224
HRH4	-0.03221	0.480235	0.152786	-0.02639	0.026139	-0.24053	0.265589	0.111191
HRK	-0.16402	-0.28111	0.027847	-0.0353	-0.15073	0.116751	-0.09112	-0.45416
HRNR	0.301965	0.382249	NA	NA	NA	NA	-0.07013	-0.45738
HRSP12	0.137024	-0.60905	-0.15927	-0.24873	-0.2598	-0.36034	-0.16792	-0.47979
HS1BP3	0.218311	-0.268	0.404063	-0.18781	0.401528	-0.24598	-0.01906	-0.13489
HS2ST1	-0.06942	-0.47962	0.25523	-0.43806	0.142155	0.024008	-0.45376	-0.4906
HS3ST1	-0.06149	0.102757	-0.00641	-0.04615	-0.06857	0.165626	0.316515	0.618409
HS3ST2	-0.01556	-0.08735	0.048628	0.017739	-0.24737	0.058795	0.044523	-0.02362

HS3ST3A1	-0.18856	0.294072	-0.05772	0.258958	-0.09699	0.301327	-0.13882	-0.17319
HS3ST3B1	0.261957	0.567643	-0.05464	0.301104	-0.25844	0.320937	-0.51036	0.198145
HS3ST4	-0.0219	0.413428	0.176744	-0.02566	0.06717	0.071914	-0.15578	0.010882
HS3ST5	-0.29899	-0.26241	0.0523	-0.12123	0.277608	0.020875	0.089421	0.214042
HS3ST6	-0.08888	0.315423	NA	NA	NA	NA	-0.25584	-0.34747
HS6ST1	-0.14887	0.374272	-0.10299	-0.20818	0.353908	-0.31832	0.373468	0.297661
HS6ST2	-0.04545	-0.42136	-0.01496	0.108544	-0.09353	0.088565	0.369606	0.159202
HS6ST3	NA	NA	-0.02028	-0.10865	-0.26961	-0.07909	0.158064	0.349933
HSBP1	-0.1981	-0.0724	-0.15793	-0.17462	0.01162	-0.50351	0.392889	0.406116
HSCB	0.043979	0.296267	-0.1683	0.112895	-0.10022	-0.16403	-0.62139	-0.51253
HSD11B1	-0.18271	-0.20063	0.113379	0.103741	-0.01394	-0.18812	-0.21564	0.476624
HSD11B1L	0.417517	0.61063	0.201862	-0.323	0.174334	-0.11269	0.454579	0.168155
HSD11B2	0.053171	0.127968	0.058752	0.168505	0.149037	-0.28089	0.170706	-0.17999
HSD17B1	-0.32048	-0.23288	0.161917	-0.04057	0.016798	0.107533	-0.15649	-0.12053
HSD17B10	0.129453	0.455073	-0.2105	-0.00803	0.033	-0.22686	NA	NA
HSD17B11	-0.19749	-0.40727	0.158019	-0.37368	-0.09409	-0.46349	NA	NA
HSD17B12	-0.00082	-0.29291	-0.40333	0.320912	-0.1622	-0.34908	-0.15999	-0.21009
HSD17B13	-0.13983	-0.24808	-0.21421	-0.0738	-0.44759	0.016709	-0.05833	0.118547
HSD17B14	-0.17835	0.332429	0.148073	-0.15399	-0.20201	0.090149	NA	NA
HSD17B2	-0.49372	0.093266	-0.0511	-0.30835	0.057366	-0.28546	0.185072	0.092375
HSD17B3	0.128339	0.478949	0.433119	-0.23737	0.397206	-0.13696	0.064036	0.325351
HSD17B4	0.351498	-0.41116	0.359452	0.346947	0.226833	0.011166	0.260612	0.40195
HSD17B6	-0.17241	-0.33324	-0.11568	-0.08007	0.036405	-0.06899	-0.30415	-0.10522
HSD17B7	-0.17656	0.212177	-0.1107	0.029032	-0.22037	0.013905	NA	NA
HSD17B7P2	-0.12341	0.098519	-0.13719	0.109938	-0.12498	0.003903	NA	NA
HSD17B8	0.217161	0.182845	0.029428	0.180637	-0.21346	-0.08581	0.306082	0.098883
HSD3B1	0.440693	0.460129	0.364215	-0.01012	0.198534	-0.32321	0.534456	0.481849
HSD3B2	-0.25755	0.145212	0.20658	0.122306	0.013485	-0.28747	0.082454	-0.08686
HSD3B7	-0.35561	0.143149	0.330855	0.034969	0.111461	0.008407	-0.07558	0.429507
HSDL1	0.190721	-0.04617	0.10977	0.031793	0.022148	-0.565	0.358326	0.220839
HSDL2	0.096561	0.002534	0.038213	-0.28402	0.147523	-0.24363	0.246275	-0.02739
HSF1	0.196126	-0.05973	0.121445	0.084263	0.027154	-0.02745	0.622156	0.240809
HSF2	0.205106	-0.05772	-0.02649	-0.20025	0.254403	0.08818	0.329661	0.111978
HSF2BP	0.444876	0.083634	-0.2744	0.116848	-0.06365	-0.16469	0.397692	0.270953
HSF4	-0.06532	-0.51573	-0.08052	0.147961	0.220954	0.187752	0.093425	0.278688
HSF5	NA	NA	0.072025	0.381954	0.243055	-0.06576	-0.25301	-0.11526
HSFX1	0.301811	0.06761	0.066648	-0.57355	0.285876	0.059359	-0.04918	0.085412
HSFY1	-0.25107	0.323295	NA	NA	NA	NA	0.152336	0.298549
HSFY2	0.359985	0.120268	-0.31134	-0.02508	-0.18352	-0.10156	0.195699	0.421949
HSH2D	-0.11756	-0.09793	0.305054	0.178434	0.036368	0.421895	-0.15486	0.257466
HSN2	0.127062	-0.03652	NA	NA	NA	NA	0.185276	0.006501
HSP90AA1	0.046321	-0.67742	-0.2993	0.247999	-0.01936	0.149308	0.15709	0.06395
HSP90AB1	0.531889	-0.44732	0.046825	0.430825	0.160295	0.155857	0.403124	0.244709
HSP90AB2F	0.018574	0.708065	NA	NA	NA	NA	-0.30758	-0.05324
HSP90AB6F	0.131192	0.264349	-0.33754	-0.1129	-0.3838	-0.01916	NA	NA
HSP90B1	-0.25249	-0.03115	-0.04844	0.311524	0.06117	0.074799	0.320346	0.267003
HSP90B3P	NA	NA	NA	NA	NA	NA	-0.26168	0.008858
HSPA12A	NA	NA	0.303926	-0.0709	0.294937	0.1544	0.019423	0.042232
HSPA12B	-0.36061	-0.15527	0.118672	0.106609	0.001041	0.143408	-0.24113	0.118863
HSPA13	NA	NA	-0.19035	-0.07264	-0.28628	-0.4264	NA	NA
HSPA14	-0.08706	-0.56726	-0.31547	-0.10338	0.213862	-0.1036	0.171603	0.207963
HSPA1A	-0.3915	-0.33248	0.156138	0.189457	0.221779	0.193154	-0.29317	-0.14865

HSPA1B	0.356873	-0.20112	NA	NA	NA	NA	0.410496	-0.08726
HSPA1L	-0.02808	-0.0088	-0.02674	0.124725	0.002806	-0.14052	-0.27755	-0.00615
HSPA2	-0.40401	-0.52738	-0.05123	-0.19176	0.10743	-0.01401	-0.31869	-0.2569
HSPA4	0.157391	0.25299	0.026325	0.060416	0.012471	-0.05125	-0.0523	-0.12282
HSPA4L	-0.11565	-0.15214	-0.13844	0.173125	-0.21497	-0.31654	0.132961	-0.05602
HSPA5	-0.22378	0.226941	0.02	0.315289	-0.04421	0.22562	0.15647	0.115736
HSPA6	-0.38413	-0.02354	0.06776	0.037361	0.06011	0.114682	NA	NA
HSPA8	0.280239	-0.51945	-0.07613	0.219993	-0.00947	-0.0207	0.200081	0.274628
HSPA9	0.247391	-0.20516	0.071415	0.067509	-0.03857	-0.16842	NA	NA
HSPB1	-0.13869	-0.05088	0.106834	-0.06792	-0.00689	-0.08608	-0.29925	-0.5084
HSPB11	NA	NA	0.429542	-0.1598	0.193078	-0.29533	NA	NA
HSPB2	-0.03679	0.047722	0.026085	-0.07524	-0.18052	-0.19358	-0.30168	-0.21855
HSPB3	-0.04083	-0.02289	-0.13545	-0.11572	0.197324	-0.12394	-0.29937	-0.41948
HSPB6	-0.05261	0.02175	-0.15026	-0.19003	0.083291	0.007384	-0.19732	-0.1762
HSPB7	0.039495	-0.00048	0.099697	-0.08295	0.12091	0.002774	-0.25992	-0.07151
HSPB8	-0.15701	-0.22609	0.081609	-0.04703	0.058216	0.310667	-0.38716	-0.55321
HSPB9	-0.24034	-0.0792	0.295806	-0.09393	-0.07943	-0.18347	0.077946	-0.44662
HSPBAP1	0.329702	0.450304	-0.1482	0.0398	-0.23366	0.116331	-0.3408	0.099226
HSPBP1	0.206675	0.174527	-0.01935	0.282314	0.190441	0.063074	0.458063	0.261485
HSPC072	-0.20556	0.358812	0.256311	-0.16026	-0.20457	0.172187	0.239712	0.169957
HSPC152	-0.15629	-0.63463	-0.02485	0.198617	-0.13227	0.065441	-0.37197	-0.2413
HSPC159	-0.00692	0.316662	0.099906	-0.19	-0.14839	-0.58141	-0.12056	-0.2673
HSPD1	0.066794	-0.36961	-0.12643	0.306932	0.049463	0.077335	0.039151	-0.20174
HSPE1	0.180019	-0.64822	0.086622	0.038172	0.010376	-0.08545	-0.12132	-0.36508
HSPG2	-0.41384	0.107689	-0.03745	-0.12886	0.071517	0.099578	-0.08583	-0.16048
HSPH1	0.34162	-0.30086	-0.06392	0.089357	0.45806	0.063778	0.419607	0.039044
HTATIP2	0.38329	0.173492	-0.0556	-0.40514	0.146362	-0.37833	0.359703	0.21708
HTATSF1	-0.16942	-0.64524	-0.28559	0.269671	0.166535	0.309819	-0.25752	-0.23834
HTN1	0.057943	0.142725	-0.01035	-0.10897	0.244315	-0.01198	0.317299	-0.20445
HTN3	0.298803	-0.33166	-0.17594	-0.02956	-0.21195	-0.08014	0.499714	0.1894
HTR1A	0.108787	0.45571	-0.01057	-0.27929	0.033718	0.384978	-0.21751	-0.33044
HTR1B	0.065124	0.100311	0.153628	-0.07366	0.197354	-0.12197	0.332049	0.045421
HTR1D	-0.29296	0.503096	0.296005	0.003003	0.056509	0.034123	0.245706	0.488291
HTR1E	0.320886	0.657654	-0.02579	0.102133	0.312036	-0.16119	-0.4031	-0.36822
HTR1F	0.247598	0.173478	0.039643	-0.07435	-0.0395	-0.23751	0.110864	0.202928
HTR2A	0.040078	0.420138	0.083724	0.141767	-0.07207	0.010601	-0.58891	-0.40448
HTR2B	0.105419	-0.02093	0.060781	-0.21796	-0.28524	0.094151	0.006644	-0.31593
HTR2C	-0.09926	0.041378	0.132338	0.136727	-0.10077	0.170351	0.178452	-0.16961
HTR3A	0.133519	0.383174	0.110438	0.583845	-0.09541	0.29441	-0.10062	-0.27877
HTR3B	0.00925	0.230908	0.364226	-0.14155	-0.22464	-0.11727	0.190968	0.065161
HTR3C	-0.14461	0.541151	-0.30914	-0.2054	-0.14814	0.096543	0.038236	0.30991
HTR3D	0.12362	0.210363	-0.0432	-0.11799	0.312781	-0.0734	0.226019	-0.1934
HTR3E	-0.24512	0.062383	-0.23795	-0.04202	-0.05162	0.185697	0.077387	0.376503
HTR4	0.191389	0.220935	0.448161	-0.01586	0.143685	-0.14647	-0.07516	0.335586
HTR5A	-0.18801	0.511484	-0.00389	0.175784	-0.27018	-0.00341	0.013351	0.347766
HTR6	-0.29601	0.138922	0.001301	-0.12225	0.044007	0.164099	-0.13809	-0.26793
HTR7	-0.0867	0.552896	-0.00033	-0.32053	-0.21781	0.077477	-0.25811	-0.13963
HTRA1	-0.17481	-0.22461	0.070511	-0.01023	0.032265	0.003945	-0.03794	0.009989
HTRA2	0.325039	-0.34165	0.273385	0.240575	0.026032	0.189588	0.124239	-0.17259
HTRA3	-0.09032	-0.31025	0.132595	-0.03921	-0.03354	0.057641	-0.17911	0.201189
HTRA4	0.095485	0.511735	0.346809	0.347318	-0.1723	0.03338	-0.5643	-0.05187
HTT	NA	NA	0.403588	0.056151	0.301274	0.174238	NA	NA

HUNK	-0.19039	0.363416	0.456337	0.130949	0.343574	-0.01456	0.326392	0.167416
HUS1	0.046688	0.50625	-0.114	-0.19277	-0.25047	-0.50115	-0.10717	0.694277
HUS1B	-0.22431	0.023759	-0.04786	-0.60049	0.09329	0.109361	-0.51246	-0.35652
HUWE1	0.390923	-0.29415	0.006435	0.108418	0.150215	0.370848	0.074648	-0.22708
HVCN1	0.069176	0.433647	0.108713	0.79523	-0.22475	0.699711	NA	NA
HYAL1	-0.50353	0.500723	-0.1393	-0.03677	-0.32607	-0.06993	-0.16504	0.041556
HYAL2	0.020403	-0.19696	0.370258	0.088056	0.299211	0.162096	0.238509	0.057634
HYAL3	0.022513	0.171416	-0.09102	0.11802	0.019479	0.042943	0.05918	-0.0295
HYAL4	0.046354	0.256493	0.054314	0.143589	0.078055	0.151872	-0.58674	-0.44889
HYDIN	0.393294	0.443748	-0.01562	-0.30862	-0.05613	-0.08319	0.16505	0.566047
HYI	-0.12948	0.42579	0.142498	0.13898	0.395279	0.051799	0.189687	0.194239
HYLS1	-0.29209	-0.5806	-0.05331	-0.29949	-0.11143	-0.48064	-0.03325	-0.04226
HYOU1	-0.15614	-0.01773	0.303767	0.326625	0.22514	-0.01869	0.61238	0.19827
HYPK	-0.28315	-0.31931	-0.12307	0.351257	-0.23043	-0.07105	-0.44275	-0.35463
IAH1	NA	NA	0.242329	-0.42465	-0.10387	-0.14904	0.509282	0.091844
IAPP	-0.02498	0.179791	0.174238	-0.1562	-0.00036	0.067073	-0.06198	-0.04305
IARS	-0.0852	-0.49207	-0.1761	0.246345	-0.00708	0.191842	0.25933	0.175165
IARS2	-0.19483	-0.69646	-0.05681	0.015768	-0.18195	0.067635	0.390325	0.100432
IBSP	-0.37995	0.028701	-0.06014	-0.04943	-0.31158	0.091686	0.156205	-0.02073
IBTK	-0.1261	-0.27165	-0.24812	-0.00281	0.089466	0.027722	-0.15333	-0.03591
ICA1	-0.19857	0.084847	0.094541	0.0225	0.07994	-0.23103	0.320206	-0.03117
ICA1L	-0.42921	-0.15725	0.293369	-0.21713	0.166705	0.166124	0.031296	-0.11599
ICAM1	-0.12544	-0.01296	0.065891	0.274178	-0.01833	0.51871	-0.33788	-0.13243
ICAM2	0.110406	-0.2507	0.046535	0.652609	-0.30439	0.192449	0.143797	0.254114
ICAM3	-0.21335	0.38379	-0.08026	0.56696	0.080275	0.213203	0.142301	0.136561
ICAM4	0.024201	0.550204	-0.24617	-0.0991	-0.04321	-0.06782	-0.31889	-0.12806
ICAM5	-0.05049	0.404556	-0.25949	0.012477	0.021247	0.109712	-0.46987	-0.22101
ICK	-0.10385	0.040723	-0.35156	-0.19085	0.00588	-0.3671	-0.03791	-0.27745
ICMT	-0.23893	0.301523	-0.10804	0.003437	0.14639	-0.45888	0.174241	0.00207
ICOS	-0.08887	0.395685	0.198606	0.650713	-0.35111	0.674741	-0.42728	0.116794
ICOSLG	0.329411	0.426785	0.231394	0.020831	0.013825	0.073846	-0.31384	0.209925
ICT1	0.363086	0.174713	0.107967	0.123683	-0.11809	-0.07276	-0.12653	-0.3497
ID1	0.320196	-0.10102	0.140531	0.005881	0.206867	-0.00288	0.078556	-0.06875
ID2	0.126307	-0.59161	-0.25035	-0.20834	-0.44493	-0.25413	-0.47363	-0.54725
ID3	0.129625	-0.35585	0.153387	0.113028	-0.07604	0.176566	-0.08245	-0.2597
ID4	-0.09879	-0.44357	0.252037	0.060273	-0.14188	0.01457	-0.57565	-0.25941
IDE	-0.06957	-0.41184	-0.02485	-0.00181	0.121145	-0.44266	0.277766	0.148418
IDH1	-0.167	-0.35533	0.079907	-0.06571	-0.06565	-0.32787	0.539851	0.338753
IDH2	0.084281	0.389425	0.266641	0.200838	0.495148	0.282396	0.216358	0.180488
IDH3A	0.299271	0.263886	-0.22474	-0.09043	-0.07307	-0.39729	0.376004	0.531993
IDH3B	0.381005	0.485978	-0.193	0.397568	-0.10791	-0.03541	0.562432	0.273634
IDH3G	0.278449	0.375046	0.177673	0.038209	0.161861	0.05346	0.312766	0.374013
IDI1	0.105934	-0.4133	-0.22512	-0.01587	-0.25229	0.139241	-0.16361	-0.25036
IDI2	-0.18897	-0.22852	-0.14629	-0.24348	0.333172	0.348823	0.543482	0.445673
IDO1	NA	NA	0.065504	0.024901	-0.33972	0.357497	NA	NA
IDO2	NA	NA	-0.05913	0.501001	-0.03235	0.504176	NA	NA
IDS	-0.15761	-0.61721	0.034077	-0.12763	0.010953	-0.37848	-0.08594	0.230196
IDUA	0.095547	-0.11827	0.381105	0.224906	0.198422	0.261856	0.29183	0.614869
IER2	0.045663	0.240801	-0.36961	0.095693	0.20437	0.02875	-0.43719	-0.26914
IER3	-0.26323	-0.0058	-0.0016	0.068874	0.009351	0.043946	-0.36751	-0.23019
IER3IP1	-0.03859	-0.66573	0.089257	-0.17802	-0.13008	-0.33829	-0.27934	-0.26995
IER5	-0.38893	-0.16093	-0.12386	0.115882	-0.13703	0.135985	0.156503	-0.15633

IER5L	0.09541	-0.05619	0.52277	-0.19246	0.143819	0.398897	-0.16914	-0.20606
IFFO1	NA	NA	0.095574	0.153351	-0.20431	0.52364	NA	NA
IFFO2	NA	NA	0.216382	-0.10793	0.339992	0.401724	NA	NA
IFI16	-0.17812	-0.32139	0.108433	0.263258	-0.62251	0.373704	-0.3444	0.075543
IFI27	0.130648	-0.24273	-0.11519	-0.31481	-0.36239	0.227556	-0.10542	-0.07036
IFI27L1	NA	NA	0.206465	-0.22719	-0.03993	-0.4267	NA	NA
IFI27L2	NA	NA	0.124946	0.144722	0.05121	-0.06898	NA	NA
IFI30	-0.33391	-0.02669	0.316255	0.089534	-0.23477	0.429143	-0.13919	0.558996
IFI35	0.06535	0.292222	0.593948	-0.13173	-0.03653	-0.41611	-0.19911	0.131582
IFI44	-0.35922	-0.42776	0.052358	-0.23393	-0.36522	0.458652	-0.17686	0.495199
IFI44L	-0.12521	-0.2731	0.122739	-0.31193	-0.38018	0.38217	-0.1758	0.206354
IFI6	-0.27619	-0.03481	0.161068	0.008251	-0.20942	0.013043	-0.00907	0.090206
IFIH1	-0.04416	-0.4693	-0.00821	-0.10399	-0.33797	-0.08532	-0.53229	-0.30793
IFIT1	-0.2511	-0.23976	0.061231	-0.43649	-0.38234	-0.20858	-0.25988	-0.12094
IFIT2	-0.3019	-0.35805	0.186371	-0.24658	-0.27233	-0.34646	-0.32328	0.088732
IFIT3	-0.35034	-0.13457	0.330226	-0.33472	-0.23993	-0.00325	-0.23178	0.399629
IFIT5	0.35022	0.108508	0.113507	-0.33646	-0.20507	-0.05613	-0.4261	0.120301
IFITM1	0.16292	0.242903	0.440137	-0.01042	0.225026	0.5271	0.078678	0.252597
IFITM2	0.099151	0.160655	0.499994	0.036942	0.038717	0.109162	-0.42742	-0.05818
IFITM3	0.208622	0.014991	0.496284	0.056149	0.244984	0.281563	-0.25186	0.01898
IFITM4P	NA	NA	0.504973	-0.03414	0.295915	0.143284	-0.27014	0.075797
IFITM5	0.361219	-0.03089	-0.01121	0.056581	-0.14924	0.128169	0.330425	0.190168
IFLTD1	-0.24788	-0.24977	0.313946	0.373587	-0.26791	-0.12996	NA	NA
IFNA1	0.296263	-0.38096	NA	NA	NA	NA	0.111198	-0.19193
IFNA10	-0.27368	0.277002	-0.06806	0.135204	-0.15799	0.070135	-0.14497	-0.40487
IFNA13	0.216586	0.397205	NA	NA	NA	NA	-0.28307	-0.18854
IFNA14	0.027683	0.116643	NA	NA	NA	NA	-0.04618	0.07466
IFNA16	-0.05891	0.454096	-0.31241	0.110699	0.128814	-0.13172	0.541936	0.409935
IFNA17	-0.20693	0.240403	NA	NA	NA	NA	0.078188	-0.18911
IFNA2	0.094806	0.517747	0.35802	-0.26008	-0.05393	0.0672	0.072942	0.067294
IFNA21	-0.4618	0.232044	0.167503	-0.07211	-0.34379	0.073764	0.527609	0.012977
IFNA4	0.196646	0.652663	-0.05373	-0.35473	0.085003	-0.03724	-0.40568	-0.37771
IFNA5	0.266836	0.470938	-0.06605	0.30974	-0.18766	-0.02678	-0.55678	-0.25299
IFNA6	0.268819	0.187082	-0.0018	-0.14509	-0.11329	-0.17368	0.088803	-0.08943
IFNA7	0.026117	0.256902	NA	NA	NA	NA	-0.08066	-0.28703
IFNA8	0.144193	0.159343	-0.14787	0.017682	0.093181	-0.05129	-0.61557	-0.60258
IFNAR1	-0.4643	-0.35745	-0.09245	0.108412	-0.12866	-0.16113	0.110351	0.272824
IFNAR2	0.25186	0.117096	0.310248	0.250062	0.169061	0.425104	-0.4641	0.069064
IFNB1	-0.13987	-0.11405	0.165927	-0.10639	-0.16564	0.002553	0.123788	-0.20302
IFNE	NA	NA	-0.16538	-0.08867	0.018176	0.164474	NA	NA
IFNG	0.274483	0.168893	0.093586	-0.11638	-0.26676	0.083055	-0.34275	-0.03035
IFNGR1	0.05765	-0.25753	0.056691	-0.23537	0.138165	0.123228	-0.13338	0.542661
IFNGR2	-0.00201	-0.01492	0.05567	-6.6E-05	-0.12555	0.005817	0.021438	0.414763
IFNK	0.130824	0.266866	-0.09224	0.165207	0.065476	-0.42621	-0.24481	-0.09783
IFNW1	-0.16834	-0.01596	0.057153	0.148305	-0.01545	-0.19956	0.618859	-0.09865
IFRD1	0.312554	-0.11914	-0.11652	0.186904	-0.16292	-0.14779	-0.29206	-0.09278
IFRD2	0.220483	-0.28976	0.105011	0.253053	0.439215	0.089414	0.523943	0.363651
IFRG15	0.056151	-0.09618	-0.16317	-0.32274	-0.22722	0.00877	0.300204	0.400826
IFT122	-0.08683	0.628467	0.241573	0.04023	0.106952	0.251886	0.14471	0.012075
IFT140	-0.10893	0.30191	-0.00554	0.374542	0.213642	0.293227	0.35532	0.227612
IFT172	0.290177	-0.26174	0.179787	-0.23829	0.241087	-0.23608	0.089093	-0.14173
IFT20	0.160718	0.263082	-0.05787	0.034352	-0.38054	-0.43976	NA	NA

IFT52	0.568454	-0.20122	-0.05713	-0.15337	0.079839	-0.42998	0.237206	-0.47065
IFT57	0.2032	0.242343	-0.22197	-0.30737	-0.04604	0.190486	-0.57235	-0.22338
IFT74	0.012352	0.297229	-0.25206	0.019513	-0.06207	-0.49263	-0.48764	-0.46628
IFT80	-0.10397	-0.27888	-0.16328	-0.06289	-0.09093	0.157033	-0.0206	-0.34276
IFT81	-0.14136	-0.11419	-0.25904	-0.13592	0.00433	-0.51139	-0.33972	-0.18364
IFT88	0.37814	-0.46665	0.138769	-0.38065	0.10678	-0.12393	0.203883	0.005753
IGBP1	0.511174	-0.23465	0.137477	-0.00834	0.008953	-0.34984	-0.12276	-0.42886
IGDCC3	NA	NA	0.246969	0.145963	-0.02577	0.04292	NA	NA
IGDCC4	NA	NA	-0.14085	0.290663	0.019587	0.39152	NA	NA
IGF1	-0.29173	-0.27195	0.10523	-0.13375	-0.27747	-0.16233	-0.38777	-0.00167
IGF1R	0.20483	0.713806	-0.11574	0.07529	0.047521	0.137856	0.103432	-0.00264
IGF2	0.117681	-0.13674	0.043195	0.079751	-0.00356	-0.16818	0.356383	0.359152
IGF2AS	0.080484	0.509735	0.092595	-0.01857	-0.02333	-0.21245	0.134485	-0.06009
IGF2BP1	0.31685	0.799313	-0.06385	0.137808	-0.28428	-0.10677	-0.72399	-0.44405
IGF2BP2	0.463967	0.274913	-0.24307	-0.04352	0.053202	-0.22969	0.202249	-0.17057
IGF2BP3	0.148975	-0.33539	-0.39085	0.379767	-0.18328	0.189595	-0.33092	-0.16406
IGF2R	0.314943	0.349081	0.241459	-0.08855	0.428374	0.18811	0.141636	-0.08346
IGFALS	0.341473	0.751267	0.02899	0.338746	-0.05805	-0.3133	-0.07402	-0.18647
IGFBP1	0.059956	0.219324	-0.11193	-0.00794	-0.08714	-0.19305	-0.06449	-0.31423
IGFBP2	-0.16984	0.175406	-0.25278	0.19932	-0.0147	-0.00704	0.014777	0.074297
IGFBP3	-0.17863	-0.21615	0.071655	0.380574	0.025013	0.330614	-0.13571	-0.49303
IGFBP4	0.132713	0.037341	0.492297	0.068979	0.192312	-0.13339	0.012441	0.091703
IGFBP5	0.198114	0.765016	0.067188	0.094875	0.017718	-0.18882	-0.37758	-0.40385
IGFBP6	-0.1466	-0.43036	0.223628	-0.11014	-0.04262	-0.00518	0.062757	0.032398
IGFBP7	-0.17522	-0.38433	-0.00622	0.207088	0.035065	0.230275	-0.07305	-0.08302
IGFBPL1	0.390211	0.799693	-0.09895	-0.19317	-0.29376	0.016361	-0.0525	-0.09758
IGFL1	0.267104	0.368618	NA	NA	NA	NA	0.480365	0.316842
IGFL2	0.112605	0.047225	0.09313	0.37563	-0.13689	-0.30138	-0.05484	-0.02423
IGFL3	-0.34076	0.147934	NA	NA	NA	NA	-0.06482	-0.09272
IGFL4	-0.19411	0.334128	NA	NA	NA	NA	-0.2576	-0.13594
IGFN1	NA	NA	-0.18268	0.05939	0.124595	0.022266	NA	NA
IGHMBP2	-0.04582	0.638312	0.07051	0.063261	0.422838	-0.30262	0.096518	0.302482
IGJ	-0.30872	-0.04934	0.049801	0.042231	0.004566	0.184712	-0.24068	-0.28313
IGLL1	0.287394	0.431484	0.32403	0.340562	0.006552	0.143821	-0.20852	-0.16895
IGLON5	NA	NA	-0.04596	-0.20613	-0.12769	-0.12765	NA	NA
IGSF1	-0.049	-0.1936	-0.2233	0.020665	0.398838	0.12412	0.228157	0.044989
IGSF10	-0.00194	-0.41605	0.233822	0.039942	-0.19395	-0.07327	-0.20461	-0.40006
IGSF11	0.010265	-0.06321	0.384889	0.073596	-0.13451	0.147848	-0.0093	-0.15206
IGSF2	-0.24024	-0.24322	-0.1603	0.142441	0.055296	0.019413	0.441145	0.460042
IGSF21	-0.55513	-0.15973	0.171567	0.124672	-0.21041	-0.18968	-0.09279	0.038502
IGSF22	0.448521	0.136693	0.235829	0.245406	0.202725	-0.06937	0.182055	0.272576
IGSF3	0.401527	0.10554	0.156509	-0.13678	0.240886	-0.31551	0.381122	0.119299
IGSF5	NA	NA	-0.22136	0.063979	-0.20839	-0.41754	NA	NA
IGSF6	-0.53473	0.064646	0.390675	-0.19748	-0.21054	0.222303	-0.59599	-0.28883
IGSF8	-0.10793	0.438699	0.486873	0.320854	0.24978	-0.12686	0.401799	0.042756
IGSF9	-0.03671	0.10737	-0.14729	-0.13672	-0.1081	-0.19649	0.173543	0.123259
IHH	0.297743	-0.08813	NA	NA	NA	NA	0.235302	-0.14971
IK	0.084671	-0.63837	0.125659	0.17049	0.124017	0.060067	-0.01208	0.170256
IKBKAP	0.265956	0.302771	-0.09366	0.000181	0.139153	0.041711	-0.24714	0.154025
IKBKB	-0.02775	0.304422	0.219578	0.197445	0.230181	0.5645	-0.25277	-0.1791
IKBKE	0.016957	-0.0353	0.145571	0.416022	0.347532	-0.0075	0.542974	0.424788
IKBKG	0.069044	0.578458	0.115311	0.205308	0.283656	0.093924	-0.62725	-0.29859

IKIP	-0.49186	-0.15806	-0.02342	0.218318	-0.15064	-0.52602	-0.24776	0.046188
IKZF1	-0.29556	0.442731	0.116649	0.732781	-0.13705	0.793009	NA	NA
IKZF2	-0.30718	0.103874	0.026177	-0.12276	-0.39379	-0.04534	NA	NA
IKZF3	0.192803	0.678472	0.108905	0.581251	-0.33147	0.253031	-0.28265	0.016529
IKZF4	0.365649	0.509434	-0.33593	-0.13031	0.429354	-0.14149	NA	NA
IKZF5	-0.25543	-0.52069	-0.16893	-0.26678	0.163139	-0.17513	NA	NA
IL10	-0.24268	0.177619	0.061874	0.069241	-0.32584	0.022981	-0.58671	-0.60276
IL10RA	-0.45448	-0.00419	0.279861	0.456179	-0.23137	0.324846	-0.49981	0.247697
IL10RB	-0.32764	-0.28635	0.026688	-0.14279	-0.05383	-0.00787	-0.2392	-0.10735
IL11	0.145077	0.02439	-0.20698	0.083816	0.023473	-0.22868	-0.14303	0.078239
IL11RA	-0.07679	-0.25156	0.112838	0.036414	-0.08477	0.297371	-0.24931	0.054403
IL12A	0.330052	-0.27323	0.033901	0.102575	0.060592	0.056926	-0.12303	0.078662
IL12B	0.185192	0.687405	-0.07938	0.171397	-0.18184	0.135499	-0.20037	0.004724
IL12RB1	0.08462	0.173844	0.387024	0.336509	0.005962	0.213946	-0.43391	0.260091
IL12RB2	0.1786	-0.18154	0.20671	0.052345	-0.35207	0.07345	-0.14522	-0.14139
IL13	0.330755	0.499946	-0.05397	0.097902	0.07565	0.163135	-0.11276	-0.02786
IL13RA1	-0.20953	-0.65141	-0.11495	-0.11637	-0.0992	-0.26647	-0.04045	0.309782
IL13RA2	0.233657	-0.26285	-0.19354	0.087121	0.074383	-0.2527	-0.33052	-0.31569
IL15	-0.0899	0.48791	0.262766	-0.32691	-0.13337	-0.37196	-0.45754	0.146978
IL15RA	0.186451	-0.06146	0.576595	-0.05153	0.095412	0.17067	-0.05362	0.364011
IL16	-0.40466	-0.08414	0.165952	0.687976	-0.28629	0.543503	-0.45624	0.197401
IL17A	0.063154	-0.05008	0.232015	0.099609	0.02903	-0.32985	NA	NA
IL17B	0.072295	-0.08455	0.058452	0.047194	-0.07741	-0.00803	-0.37322	-0.14812
IL17C	0.19787	0.602095	0.137386	0.208645	-0.16423	0.2632	-0.14219	-0.20277
IL17D	0.069384	0.632015	-0.20166	-0.15061	0.100695	-0.13557	0.241055	0.100973
IL17F	-0.08751	-0.17128	0.2829	0.110385	0.233021	-0.01831	-0.03192	0.390634
IL17RA	-0.40035	-0.46578	0.40146	0.01987	0.270483	0.588075	NA	NA
IL17RB	0.036777	-0.15668	-0.27654	0.103665	0.11496	-0.17979	0.601858	0.350066
IL17RC	0.149789	0.575875	0.331911	-0.17814	0.30313	-0.00464	-0.1147	0.340327
IL17RD	0.027754	0.293209	-0.05868	-0.10332	0.028602	0.243274	-0.45747	-0.58311
IL17RE	0.178103	0.183282	-0.01739	-0.02076	0.067605	-0.12269	0.410687	0.112863
IL18	-0.0634	0.599836	-0.07469	-0.23396	-0.32949	-0.07359	-0.50091	-0.31989
IL18BP	0.348418	0.468768	0.262081	-0.03356	-0.22594	0.417641	-0.46608	0.161057
IL18R1	-0.36651	-0.17035	0.030154	0.553453	-0.20351	0.4413	-0.25505	0.51801
IL18RAP	-0.55868	0.304782	0.011116	0.133028	-0.07896	0.215586	-0.45571	0.765901
IL19	-0.0442	0.460552	-0.0552	0.270206	-0.1515	-0.37936	0.127619	0.16731
IL1A	-0.2341	0.05051	0.100151	0.420137	0.06944	0.156388	-0.23062	0.045277
IL1B	-0.28532	-0.08169	0.041944	0.274029	0.008123	0.12415	-0.21411	0.150939
IL1F10	-0.09953	-0.0384	0.145679	-0.22231	-0.13808	0.026027	0.23709	-0.19633
IL1F5	0.156164	0.818575	-0.06453	-0.19149	-0.23853	0.155812	-0.2654	-0.17827
IL1F6	0.019953	-0.04522	0.371247	0.007425	-0.08929	0.08604	-0.17471	-0.17838
IL1F7	0.204853	0.250742	-0.27645	-0.07177	-0.08996	0.069502	-0.12512	-0.28067
IL1F8	0.451019	0.450159	-0.01605	0.040041	-0.08217	0.070782	-0.08403	0.172818
IL1F9	-0.04473	0.810645	-0.27192	0.009585	-0.16785	-0.30248	0.123978	0.428867
IL1R1	-0.3961	-0.45256	-0.06303	0.022526	-0.28259	0.087437	-0.57535	-0.1323
IL1R2	-0.51467	0.200261	-0.19516	-0.0763	-0.35793	-0.21232	-0.0005	-0.36879
IL1RAP	-0.55221	-0.2273	-0.23326	-0.22581	-0.03645	-0.16478	-0.08417	-0.04495
IL1RAPL1	-0.10097	0.319693	0.09907	0.006026	0.099172	0.029535	-0.15123	-0.03147
IL1RAPL2	-0.32598	0.188373	0.163526	-0.02489	-0.28683	0.49523	0.21438	-0.05777
IL1RL1	0.050505	0.235643	0.263177	0.20608	0.189974	0.035114	0.053728	0.423076
IL1RL2	0.110708	0.183417	-0.10409	-0.2312	-0.00268	-0.02808	-0.0027	0.008866
IL1RN	-0.51374	-0.12109	-0.05415	0.191493	-0.04899	-0.01027	-0.14937	0.322645

IL2	-0.22085	0.496974	0.612377	0.318137	-0.06941	0.336654	0.290766	0.342967
IL20	0.021856	0.106646	-0.1775	-0.23076	0.029218	-0.15913	NA	NA
IL20RA	0.384723	0.245282	-0.08264	-0.12623	0.104915	-0.28374	0.315558	-0.33721
IL20RB	-0.13667	0.150884	-0.53791	0.017265	-0.35456	-0.07563	NA	NA
IL21	0.273736	0.267595	0.06463	0.294012	-0.12124	-0.17698	0.10763	0.218598
IL21R	0.026967	0.645966	0.2537	0.759973	-0.29681	0.639467	-0.45432	0.044823
IL22	0.146451	0.359157	0.425451	-0.05537	0.361547	0.135831	0.487426	0.536457
IL22RA1	0.435162	0.117351	0.212731	-0.40229	0.483696	0.072589	0.441195	0.317837
IL22RA2	0.359677	0.74793	-0.06664	0.417197	-0.10657	0.616333	-0.26382	-0.12432
IL23A	0.127204	0.122508	-0.05579	0.589219	0.055927	0.338102	-0.01612	0.156782
IL23R	0.133121	-0.0216	-0.10039	-0.03789	0.144918	-0.1532	0.034307	-0.03883
IL24	0.129301	0.456962	0.001537	-0.12368	0.139596	0.145776	-0.26824	0.179365
IL25	0.310843	0.237178	0.096504	-0.1873	0.185933	-0.25605	0.090748	-0.24981
IL26	0.146853	0.791387	0.015714	-0.12064	-0.0432	-0.08843	0.234099	0.61566
IL27	0.119556	-0.10494	-0.09343	0.040805	0.063306	0.128458	-0.38427	0.243309
IL27RA	0.084474	0.268639	-0.05719	0.849394	-0.15669	0.71386	0.19167	0.394518
IL28A	-0.1646	0.045537	0.125213	-0.08209	-0.35965	-0.22295	0.308918	-0.06611
IL28B	0.125141	0.30897	0.212659	-0.18086	-0.32492	0.004351	-0.37146	-0.35128
IL28RA	0.212293	0.135341	0.079557	-0.25532	0.34722	0.180991	0.076668	0.034224
IL29	-0.0632	0.279378	0.180432	-0.21172	-0.13017	-0.26379	-0.05833	0.472422
IL2RA	-0.0226	0.514754	0.100341	0.384969	-0.12977	0.403295	0.199276	0.119226
IL2RB	-0.42355	0.027615	0.150352	0.627487	-0.29367	0.544262	-0.6454	0.212031
IL2RG	0.236976	-0.04569	-0.07848	0.071236	-0.30748	-0.02001	0.464095	0.363065
IL3	0.317087	0.74858	-0.07217	0.044418	0.018891	0.079517	0.121891	-0.02595
IL31	0.138914	0.043683	0.201908	-0.15807	0.144348	0.030311	0.000562	-0.22149
IL31RA	-0.25815	0.092535	-0.12572	0.189794	-0.03377	0.018966	0.600422	0.49638
IL32	0.453167	0.241723	0.042151	0.019749	-0.05881	-0.06124	-0.22297	0.093627
IL33	0.040563	-0.27855	0.171652	-0.1076	-0.32589	0.411896	-0.13594	-0.30269
IL34	NA	NA	0.206096	0.59794	0.045032	0.135752	NA	NA
IL3RA	0.032925	0.364453	0.130674	0.1397	-0.33406	0.355852	-0.47149	-0.39681
IL4	0.266943	0.527811	-0.1082	0.371591	0.138668	-0.04475	-0.25957	-0.18246
IL4I1	-0.30012	0.164288	-0.01554	0.552436	-0.2193	0.754287	-0.42321	-0.02096
IL4R	-0.30447	0.301921	0.010506	0.095724	0.249439	0.351747	-0.49367	-0.21096
IL5	0.183956	-0.20938	-0.15161	0.016395	0.209032	0.343178	0.359355	0.410548
IL5RA	-0.20442	0.340021	-0.07809	-0.33041	-0.09124	-0.28167	0.040082	0.070241
IL6	-0.06013	-0.20428	-0.08251	0.134575	0.087971	0.131472	-0.47617	-0.09291
IL6R	-0.02822	0.482198	0.072293	-0.1124	-0.0526	0.281903	-0.18917	0.268265
IL6ST	-0.02862	-0.24553	0.016819	-0.07252	-0.30696	0.169851	-0.63895	-0.21803
IL7	-0.01708	-0.22669	0.200417	0.172233	-0.11249	-0.21842	0.005517	-0.0714
IL7R	-0.391	0.054507	0.244751	0.576741	-0.27064	0.569598	-0.2427	0.228668
IL8	-0.25951	-0.22269	-0.19365	0.361015	-0.02183	0.094377	-0.1629	-0.01915
IL8RA	-0.44277	0.053808	0.194514	0.075951	0.08041	-0.31741	-0.3946	0.168425
IL8RB	-0.49078	-0.1774	0.201945	0.021002	0.078983	-0.0967	-0.29737	0.037813
IL9	-0.07187	0.379614	-0.04686	-0.06809	0.047868	-0.33625	0.304112	-0.38734
IL9R	0.244058	0.456719	0.097216	0.296532	0.26199	0.219377	0.529225	0.413522
ILDR1	-0.0432	0.022935	-0.02904	0.01376	0.099903	0.436975	0.341832	-0.2463
ILF2	0.021297	-0.41533	-0.43975	0.072723	-0.27047	-0.33463	0.634643	0.348866
ILF3	0.016823	-0.30817	-0.09872	0.547684	0.439102	0.500615	0.327692	0.508959
ILK	-0.09894	-0.07479	0.242962	-0.11217	0.001709	0.121603	-0.20959	-0.32605
ILKAP	0.219454	0.578796	-0.1833	0.363923	0.189595	0.154713	-0.18002	-0.15692
ILVBL	0.173906	0.212566	0.192177	0.010864	0.230363	-0.41559	0.065565	0.056412
IMMP1L	0.332401	-0.18755	-0.13628	-0.29337	-0.19659	-0.11025	NA	NA

IMMP2L	0.421802	-0.26741	-0.03927	-0.1972	0.100078	-0.25243	0.162416	0.06671
IMMT	0.076451	-0.6082	-0.20882	0.013426	0.117315	-0.07177	-0.32741	-0.31041
IMP3	0.199521	-0.12966	-0.03338	0.42136	-0.22822	0.037654	-0.02264	-0.0662
IMP4	0.388664	0.285961	-0.20467	0.232402	-0.05071	0.147401	-0.00781	-0.18686
IMP5	-0.21452	0.35407	0.207156	-0.27523	-0.04157	-0.30551	-0.27831	-0.13443
IMPA1	0.044349	0.22772	-0.00696	-0.18181	-0.36249	-0.23279	-0.18159	-0.11163
IMPA2	0.141533	0.502371	-0.21261	0.025737	0.222908	0.169626	0.416843	0.185892
IMPACT	-0.19033	-0.09363	-0.11768	-0.14445	-0.20274	-0.37539	0.173533	0.444407
IMPAD1	-0.2658	-0.60659	-0.03735	-0.01821	-0.21543	-0.16109	-0.06155	-0.07392
IMPDH1	0.152753	0.138675	-0.06363	0.206524	0.068896	-0.00291	0.188307	0.062193
IMPDH2	0.180608	-0.12001	0.057525	0.464365	0.315902	0.225282	0.302701	-0.33834
IMPG1	0.410164	0.78299	0.069951	-0.12783	-0.20404	0.136751	0.213287	0.07028
IMPG2	-0.09875	-0.05854	-0.15568	-0.11958	-0.06662	-0.21789	0.273515	0.245216
INA	0.130615	-0.11975	0.042658	-0.00154	0.116503	-0.06617	-0.42717	-0.18829
INADL	-0.13998	0.097496	0.439979	-0.18582	0.259267	-0.05226	0.35829	0.039882
INCA1	-0.05562	0.408096	NA	NA	NA	NA	-0.05828	0.187776
INCENP	0.102991	0.342181	-0.41171	0.262489	-0.03416	0.099814	0.26969	-0.08551
INE1	-0.70319	-0.14923	0.03755	-0.003	0.075353	0.399648	NA	NA
INF2	NA	NA	-0.02737	-0.30999	0.232567	-0.03073	NA	NA
ING1	0.394112	0.508347	-0.17776	0.052337	0.345054	-0.20226	0.579995	0.171871
ING2	-0.30443	-0.38985	-0.16228	-0.26255	0.056081	-0.24781	-0.04132	-0.0008
ING3	0.094611	-0.15222	-0.21258	0.057399	-0.13069	-0.02933	0.302293	0.258774
ING4	0.252071	0.394297	0.251957	-0.03406	-0.0026	-0.06541	-0.60638	-0.40594
ING5	-0.01172	-0.36825	0.007177	0.078494	0.081218	0.295836	-0.39049	-0.09714
INGX	NA	NA	-0.2967	-0.23796	0.176182	0.329993	-0.14895	-0.10344
INHA	0.222888	0.46861	0.287326	-0.13927	-0.02171	0.002939	-0.29902	-0.16221
INHBA	0.266896	0.728562	0.165752	-0.06713	-0.09051	0.369895	-0.28464	0.244648
INHBB	0.018912	-0.02799	0.197746	0.27423	0.227958	0.213728	0.483637	0.131724
INHBC	0.091522	-0.12853	0.049371	-0.1605	0.061917	-0.12741	-0.24513	-0.01086
INHBE	0.308697	0.180895	0.148894	-0.02102	-0.34767	-0.29282	-0.12101	-0.31854
INMT	0.097965	-0.0139	0.172978	-0.27474	-0.05257	0.292173	-0.30282	-0.17515
INO80	NA	NA	-0.00844	0.112216	0.099653	0.188571	NA	NA
INO80B	NA	NA	0.181711	0.191741	-0.04007	-0.07225	NA	NA
INO80C	NA	NA	-0.03292	0.090201	-0.09297	-0.28543	NA	NA
INO80D	NA	NA	0.126493	0.04585	-0.09233	0.137523	NA	NA
INO80E	NA	NA	0.11868	0.012901	0.133403	0.340278	NA	NA
INPP1	-0.24012	0.33869	-0.06258	-0.02851	-0.0232	0.176634	-0.0609	0.315317
INPP4A	-0.2011	0.142377	0.187423	0.142618	-0.0596	0.084456	-0.11186	-0.30185
INPP4B	0.179756	0.613516	0.273593	0.308843	-0.06953	-0.11422	0.26137	0.219942
INPP5A	-0.59132	-0.19939	-0.02387	-0.12598	0.075652	-0.13823	-0.34492	-0.27761
INPP5B	-0.06418	-0.56377	-0.1869	0.463386	0.061999	0.121124	-0.16692	0.32549
INPP5D	0.182558	0.129776	-0.12936	0.76895	0.386854	0.749309	-0.17472	0.029694
INPP5E	0.259377	-0.0073	-0.10706	0.304804	0.261874	0.154134	0.162771	0.256811
INPP5F	0.190921	0.594146	-0.01591	-0.36247	0.136081	-0.09262	0.377701	0.375145
INPP5J	NA	NA	0.226723	-0.14423	0.556494	-0.1853	NA	NA
INPP5K	NA	NA	-0.08695	-0.01663	-0.29494	-0.17556	NA	NA
INPPL1	-0.18884	0.202949	0.251928	0.135361	0.4609	0.512271	0.023679	0.164981
INS	0.188136	0.166896	-0.1905	-0.1318	-0.17218	-0.28548	0.06536	0.337444
INS-IGF2	NA	NA	NA	NA	NA	NA	-0.00652	-0.00391
INSC	-0.31485	0.015968	NA	NA	NA	NA	-0.22791	0.174694
INSIG1	-0.26731	-0.24956	-0.04945	0.040802	-0.28508	-0.04997	-0.00359	0.147323
INSIG2	-0.3195	-0.69189	-0.06899	-0.11972	-0.17862	-0.50983	0.617451	0.472273

INSL3	-0.22553	0.061914	0.132618	0.76476	-0.08657	0.486098	0.035317	0.432367
INSL4	-0.5257	0.124071	-0.03132	-0.00181	-0.15719	-0.26999	0.153354	0.301299
INSL5	0.035841	0.013346	-0.10636	0.027701	-0.02428	-0.33973	-0.12198	-0.05592
INSL6	0.20388	0.215852	-0.09453	-0.13732	-0.39307	0.121036	-0.08918	0.121459
INSM1	-0.15922	0.656886	-0.00522	0.06503	-0.24804	-0.03019	0.11067	-0.00436
INSM2	0.038508	0.32007	0.05589	-0.11243	-0.05011	-0.24057	0.016959	-0.42702
INSR	0.174842	0.746338	-0.03827	-0.12843	0.250707	0.305736	0.068246	0.07142
INSRR	-0.08238	0.128825	0.070832	-0.26619	0.334574	0.150587	-0.49928	-0.34804
INTS1	NA	NA	-0.1432	-0.0713	0.156915	-0.00452	-0.324	-0.36559
INTS10	0.021258	-0.40486	0.278644	0.002092	-0.07921	0.20139	NA	NA
INTS12	-0.02615	0.21498	0.002347	-0.45789	-0.1766	0.071426	NA	NA
INTS2	-0.00727	-0.58475	-0.19024	0.072054	-0.01371	0.050384	NA	NA
INTS3	0.124944	-0.26306	-0.09193	0.20554	0.054107	0.272423	NA	NA
INTS4	-0.07437	-0.11883	-0.0469	0.284704	-0.16731	-0.0371	NA	NA
INTS5	0.146383	0.651924	0.256433	0.185518	0.330492	0.155383	NA	NA
INTS6	0.086988	0.404958	-0.09846	-0.07931	0.357383	0.072234	0.362571	0.548505
INTS7	-0.04726	-0.42075	-0.2052	-0.07501	-0.04671	-0.08843	NA	NA
INTS8	-0.28194	0.177125	0.086858	-0.06976	0.010228	-0.29984	NA	NA
INTS9	NA	NA	0.226586	0.309424	-0.24408	0.353709	NA	NA
INTU	0.224028	-0.32104	-0.1268	-0.04768	-0.21252	0.286604	-0.3503	0.019695
INVS	0.024422	-0.51491	-0.14516	0.218175	-0.21058	0.130573	-0.02854	0.358415
IP6K1	NA	NA	-0.10462	-0.05688	0.077362	-0.20368	NA	NA
IP6K2	NA	NA	-0.17188	0.135981	0.126358	-0.35396	NA	NA
IP6K3	NA	NA	0.129107	0.102776	0.264997	-0.20957	NA	NA
IPMK	0.146906	0.187696	-0.08579	-0.29863	-0.02327	-0.24247	-0.20455	0.08092
IPO11	0.502722	0.221456	-0.25975	0.080387	0.011227	-0.19425	0.196634	-0.01942
IPO13	-0.02433	0.334477	0.49816	0.161343	0.329029	-0.0473	-0.29416	-0.01914
IPO4	0.242559	0.454611	0.04665	0.254526	0.263316	0.299654	0.579861	0.311937
IPO5	NA	NA	0.192364	0.01263	0.370836	-0.14325	NA	NA
IPO7	0.249231	-0.30577	-0.00498	0.030717	-0.0251	0.20947	0.363104	0.108196
IPO8	0.254939	-0.21916	-0.33481	-0.04684	-0.0277	-0.23679	-0.24093	0.167127
IPO9	0.161381	-0.55248	-0.1385	0.175527	-0.03615	0.308226	0.263162	-0.00709
IPP	-0.27919	0.251278	0.283203	-0.14278	0.44479	-0.14767	-0.6067	-0.64161
IPPK	-0.37638	-0.56599	-0.38386	0.095958	-0.03643	-0.25428	-0.084	0.203213
IPW	NA	NA	-0.06	0.005095	-0.02042	-0.01032	NA	NA
IQCA1	NA	NA	0.070004	0.274943	-0.04138	0.310706	NA	NA
IQCB1	0.322411	-0.38314	0.015487	-0.34723	0.263511	0.034149	0.365307	0.243737
IQCC	-0.33682	0.244354	0.185685	0.191761	0.365684	0.032543	-0.30202	-0.32662
IQCD	-0.05659	-0.14275	-0.30837	-0.06892	-0.07692	-0.30001	-0.10614	0.089498
IQCE	0.386456	-0.21568	0.216483	0.091687	0.400723	-0.24059	0.149137	0.38701
IQCF1	0.497785	0.717917	-0.36232	-0.26454	0.097	-0.00243	0.09474	-0.28052
IQCF2	0.278722	0.636423	0.143907	0.014517	0.041497	0.148945	0.152291	0.046132
IQCF3	NA	NA	0.068866	-0.32031	0.301546	-0.18401	NA	NA
IQCF5	NA	NA	0.258063	0.096809	-0.21837	0.203448	NA	NA
IQCF6	NA	NA	0.212674	-0.14095	-0.13703	-0.03404	NA	NA
IQCG	-0.38453	0.026838	-0.07832	-0.08034	-0.25699	-0.25793	-0.51023	-0.22458
IQCH	0.289234	0.65759	-0.11811	-0.39969	0.217715	-0.53226	0.248786	0.443964
IQCK	0.012583	-0.68699	-0.62312	-0.43472	-0.27428	-0.69681	0.033743	-0.32545
IQGAP1	-0.18415	-0.75985	-0.28783	-0.09668	-0.01505	0.071044	-0.32141	-0.14381
IQGAP2	-0.19072	0.427589	-0.04307	0.103427	-0.18868	-0.01004	-0.34038	-0.20849
IQGAP3	-0.23724	0.419136	-0.10778	-0.1193	0.004846	-0.25861	0.575057	0.128691
IQSEC1	-0.28354	-0.33651	0.1339	0.22396	0.114171	0.489346	0.108554	0.03569

IQSEC2	0.229591	0.663018	0.027818	0.041538	-0.12696	0.15561	-0.19779	0.214258
IQSEC3	0.267103	0.696047	-0.2441	-0.26427	-0.26314	0.21347	-0.48379	-0.36377
IQUB	0.395685	-0.3047	0.010203	-0.25907	-0.1852	-0.29306	NA	NA
IQWD1	-0.13358	-0.58131	-0.39642	-0.28329	-0.14969	-0.33402	0.056332	0.00943
IRAK1	0.12864	-0.45843	0.255939	0.169031	0.575841	0.036945	0.137858	0.098183
IRAK1BP1	0.470899	-0.20437	-0.16211	0.152586	0.04232	-0.11817	-0.01382	-0.45422
IRAK2	-0.07538	0.000771	-0.07903	-0.16357	0.40976	-0.04542	-0.25985	0.39864
IRAK3	-0.43755	0.059885	0.022625	0.219722	-0.18568	0.034586	-0.46342	0.159045
IRAK4	-0.1845	-0.3258	0.140881	-0.04875	0.132839	-0.21994	0.050182	0.503693
IREB2	0.147575	-0.34887	-0.48038	-0.16512	-0.28224	-0.30961	-0.24589	-0.20318
IRF1	0.22051	0.252975	0.469262	-0.12847	0.179187	0.223711	-0.40639	-0.12248
IRF2	0.199438	-0.26671	-0.22609	0.133912	-0.16752	-0.38452	-0.60767	-0.31077
IRF2BP1	0.404766	0.626033	-0.12489	0.111805	-1.5E-05	0.058088	0.620959	0.347256
IRF2BP2	0.241071	-0.61772	0.182138	0.020131	0.541571	0.214829	0.627585	0.106985
IRF3	0.333148	0.515739	0.203806	-0.01801	0.411319	0.26368	0.31605	0.347208
IRF4	-0.27491	-0.10038	0.220135	0.413648	-0.06759	0.191572	-0.59924	-0.2915
IRF5	-0.03158	0.08077	-0.19521	0.408819	-0.09906	-0.10377	0.009669	0.18894
IRF6	0.169287	0.073832	0.034661	0.003026	-0.18475	-0.12668	0.123258	-0.19185
IRF7	-0.1922	0.380789	-0.06647	-0.15315	-0.21906	-0.17072	-0.37394	0.305423
IRF8	0.071851	-0.10231	0.172393	0.531358	-0.006	0.20142	-0.36083	0.18757
IRF9	NA	NA	-0.03215	-0.28088	-0.1033	0.363396	NA	NA
IRGC	0.30573	0.626187	-0.14504	-0.07088	-0.10267	-0.05575	0.088849	-0.01114
IRGQ	-0.13986	-0.10106	NA	NA	NA	NA	0.529642	0.140073
IRS1	0.118767	0.269163	-0.14503	-0.32867	-0.37531	0.035884	0.030968	-0.05254
IRS2	0.096378	-0.56925	0.223569	0.020299	0.21259	0.15551	0.422053	0.116043
IRS4	-0.5281	-0.02919	-0.01036	-0.39531	0.246032	0.164323	0.274017	0.031367
IRX1	0.27258	0.71703	0.321564	-0.43871	0.254863	0.10996	-0.12171	-0.28151
IRX2	-0.13676	-0.04279	0.042028	-0.11673	0.000831	-0.06773	0.331939	-0.17933
IRX3	-0.59268	-0.24166	-0.24834	0.030723	-0.28767	0.081048	0.382302	-0.07908
IRX4	-0.05574	0.266727	-0.01054	-0.05328	-0.04984	0.201777	0.290281	0.153617
IRX5	-0.07155	0.398377	-0.19552	0.153044	-0.12677	-0.07303	0.333147	-0.30859
IRX6	-0.38774	-0.35722	0.158771	-0.21456	-0.0546	-0.01922	-0.58987	-0.12553
ISCA1	-0.08392	-0.27031	-0.04088	-0.2588	-0.07503	-0.61391	-0.31576	-0.29538
ISCA2	0.102583	0.034061	-0.08148	-0.12643	-0.0979	-0.46108	0.117258	0.159749
ISCU	0.239975	-0.0689	-0.31952	0.004779	-0.27227	-0.46521	-0.15594	0.053087
ISG15	-0.17083	-0.0499	0.235194	-0.34063	-0.28055	-0.17951	-0.1474	0.058382
ISG20	-0.25132	0.266428	0.062107	0.15032	-0.24394	-0.01292	-0.34844	0.128205
ISG20L2	-0.2489	-0.04667	-0.02523	-0.05478	0.148894	-0.00968	0.188105	0.187457
ISL1	-0.47818	-0.14779	-0.10361	-0.01962	-0.23002	-0.05903	NA	NA
ISL2	-0.06472	-0.16287	0.191706	-0.06002	0.06945	0.089452	-0.3026	-0.28213
ISLR	-0.10809	-0.0815	0.033196	0.178051	-0.07356	0.386055	-0.15902	-0.24392
ISLR2	-0.27074	-0.35663	0.258995	0.027723	0.148502	0.318399	-0.19263	0.107963
ISM1	NA	NA	0.065723	-0.19524	-0.25779	-0.09513	NA	NA
ISM2	NA	NA	0.158051	-0.26036	0.152941	0.104178	NA	NA
ISOC1	0.007834	-0.65239	-0.2117	0.301348	-0.05328	-0.26222	-0.14557	-0.37547
ISOC2	-0.06636	0.161119	0.061472	0.117694	0.09684	-0.06442	-0.36065	-0.2513
ISX	0.201029	0.023178	-0.37191	-0.01853	0.101303	-0.34938	NA	NA
ISY1	-0.23536	-0.55759	-0.14758	0.118484	-0.136	-0.06027	NA	NA
ISYNA1	-0.10449	-0.38986	-0.20234	0.357667	-0.10223	0.365575	-0.2329	0.201917
ITCH	0.172048	-0.37939	-0.05428	-0.44741	0.192179	-0.28734	0.329562	0.139319
ITFG1	-0.15711	-0.5168	0.214668	-0.40652	0.173783	-0.33822	-0.50244	-0.35433
ITFG2	0.500863	0.143287	-0.05378	0.335174	0.032734	0.406839	NA	NA

ITFG3	0.154903	0.138916	-0.12665	0.066386	0.170511	0.087179	NA	NA
ITGA1	-0.12458	-0.21259	-0.04524	-0.15557	-0.24449	-0.1944	-0.31082	-0.28228
ITGA10	-0.17003	0.528716	-0.01518	0.400089	0.181418	0.153021	0.145575	0.080851
ITGA11	-0.00971	-0.37894	0.062292	-0.29993	-0.0311	-0.13907	0.244544	0.219357
ITGA2	0.09635	-0.12611	0.00238	-0.0235	-0.15854	0.045551	0.143565	0.147758
ITGA2B	-0.24203	0.172787	0.146299	-0.1136	-0.09297	-0.12866	-0.60607	-0.17702
ITGA3	-0.18373	0.312267	0.352347	0.069811	0.054394	-0.05576	-0.17642	-0.0762
ITGA4	-0.32623	-0.08799	0.092147	0.010593	-0.41486	0.095785	-0.00544	0.383077
ITGA5	-0.3631	0.006627	0.17058	0.027059	0.011873	0.279711	-0.30902	-0.25551
ITGA6	0.2545	-0.19231	-0.13922	-0.11008	0.354937	0.386765	0.044481	0.005783
ITGA7	0.080218	0.116327	-0.00592	0.007426	0.099437	0.265692	-0.27947	-0.03062
ITGA8	0.004919	0.359909	0.199431	-0.13501	-0.25639	-0.14381	-0.42168	-0.51667
ITGA9	0.307912	0.175792	-0.05065	-0.19704	-0.23792	0.062223	NA	NA
ITGAD	0.170559	0.657922	0.076431	0.682909	-0.16971	0.318999	-0.292	-0.27784
ITGAE	-0.03673	-0.3541	-0.21988	-0.11257	-0.40359	-0.29522	-0.59703	-0.31189
ITGAL	0.186568	0.717754	0.329964	0.710708	-0.05802	0.808446	-0.4725	0.478243
ITGAM	-0.4597	-0.31452	0.185099	0.420275	-0.15788	0.396583	-0.25009	0.192905
ITGAV	-0.51796	-0.71758	-0.06955	-0.15697	-0.16706	0.154827	0.027797	0.127229
ITGAX	-0.19934	0.556248	0.16587	0.529635	0.005286	0.529601	-0.39509	0.231203
ITGB1	-0.36162	-0.43096	0.050745	-0.15421	0.012289	0.083124	-0.00116	0.034665
ITGB1BP1	-0.43259	-0.51271	0.020891	0.022366	0.081923	-0.00016	-0.49074	-0.0122
ITGB1BP2	-0.06245	0.096399	-0.32505	-0.13873	-0.23946	-0.24996	-0.1263	-0.1738
ITGB1BP3	0.078507	-0.13608	-0.15791	-0.19135	-0.1847	0.163452	-0.06061	-0.03998
ITGB2	-0.53654	-0.05212	0.155837	0.486537	-0.18982	0.479723	-0.44234	0.197476
ITGB3	0.309522	0.612056	0.038748	-0.07572	0.010019	-0.12898	-0.41159	-0.11125
ITGB3BP	0.041087	-0.31051	-0.07842	-0.03481	0.031653	-0.32528	0.086061	-0.25672
ITGB4	-0.111	0.466837	0.46481	0.050118	0.29757	0.26818	0.327567	0.159875
ITGB5	-0.04504	-0.56044	0.330821	-0.03275	0.301834	0.187138	-0.08463	-0.30715
ITGB6	-0.07853	0.20816	0.283589	-0.09548	-0.0553	-0.49488	-0.28388	-0.13235
ITGB7	-0.30001	0.347145	0.055105	0.31439	-0.15283	0.345352	-0.1408	0.392051
ITGB8	-0.41209	0.006519	-0.16235	-0.33591	-0.15386	0.025532	0.119177	0.280349
ITGBL1	-0.04521	-0.55689	0.250691	-0.06717	-0.07861	-0.13992	-0.42739	-0.14923
ITIH1	0.039067	0.567067	0.120987	0.439306	-0.00533	0.322639	-0.22604	-0.11882
ITIH2	-0.34369	0.08819	-0.23771	-0.33663	0.009807	0.060716	0.335429	0.18547
ITIH3	0.493549	0.139507	0.315872	0.316207	-0.17454	-0.04382	-0.14823	-0.13994
ITIH4	-0.29817	0.389086	0.006172	0.209209	-0.36757	0.130598	-0.31846	-0.03318
ITIH5	-0.02779	-0.17182	-0.13573	-0.02289	0.011449	0.468952	0.141867	0.173187
ITIH5L	0.061752	0.04727	0.015484	-0.07136	-0.16939	0.120986	-0.17202	-0.3149
ITK	-0.27437	0.121346	0.160649	0.665892	-0.11286	0.737738	-0.2743	0.680419
ITLN1	-0.26956	0.320769	-0.02733	0.254786	0.085863	-0.09687	0.039192	0.074301
ITLN2	0.047715	0.216563	-0.07926	0.262928	0.069554	-0.04654	-0.07492	0.590405
ITM2A	0.127449	-0.43966	0.189286	0.005919	-0.29013	-0.17236	-0.72692	-0.43211
ITM2B	-0.18504	-0.63589	-0.35431	-0.05556	-0.16788	-0.26179	-0.75886	-0.48721
ITM2C	-0.32763	0.188926	0.017271	0.242927	-0.15067	-0.07058	0.065403	-0.09212
ITPA	0.183054	0.468796	-0.08789	0.128401	-0.00851	0.175134	0.268299	-0.17318
ITPK1	-0.00403	-0.12776	-0.13132	-0.2423	0.065562	0.053062	-0.36323	0.003804
ITPKA	-0.04631	0.241904	0.019512	-0.0521	0.134899	-0.34789	0.117399	0.052237
ITPKB	0.03466	-0.01713	0.318952	0.306928	0.067996	0.631363	-0.37518	-0.16065
ITPKC	0.254243	0.566352	0.036515	-0.4586	0.145336	0.152177	0.063418	-0.18359
ITPR1	-0.10442	-0.31036	-0.00711	0.066659	-0.14181	0.590566	-0.25313	0.004979
ITPR2	0.239198	-0.59738	-0.03862	0.018434	-0.15752	0.010006	0.256261	0.19628
ITPR3	-0.33456	0.154195	0.09983	0.257324	0.479046	0.279753	0.177246	0.154893

ITPRIP	NA	NA	-0.12126	0.084378	-0.09136	0.295352	NA	NA
ITPRIPL1	NA	NA	-0.05162	0.441714	-0.17346	0.014793	NA	NA
ITPRIPL2	NA	NA	-0.00276	-0.14293	0.119002	0.170008	NA	NA
ITSN1	0.134677	-0.26596	-0.20177	-0.29678	-0.07188	0.087879	0.089858	0.241187
ITSN2	-0.03679	0.21251	0.178933	0.318315	-0.03753	0.450256	-0.52871	-0.06941
IVD	0.108171	0.425267	0.099787	0.111502	0.015207	0.200388	0.064847	0.163816
IVL	0.289854	0.444469	0.072677	-0.12327	-0.18908	0.076754	0.041844	0.250641
IVNS1ABP	-0.45041	-0.38154	-0.39602	-0.0091	-0.22661	-0.09684	0.010404	0.042936
IWS1	0.043626	-0.73922	-0.09458	0.172457	0.057617	0.17939	NA	NA
IYD	0.15402	0.436334	-0.06422	-0.19715	0.409758	-0.10859	NA	NA
IZUMO1	0.592019	0.590257	0.114607	-0.36304	0.097893	0.171938	-0.21453	-0.06352
JAG1	-0.10346	0.530053	-0.10278	-0.20493	-0.22171	-0.01804	-0.1061	0.25187
JAG2	-0.18479	0.265571	0.027526	0.10883	0.119256	0.196958	0.217371	0.196413
JAGN1	0.145002	0.457767	-0.28303	0.012919	-0.01123	-0.25595	NA	NA
JAK1	-0.35611	-0.64062	0.203445	0.263481	0.013382	0.161313	0.202914	0.101966
JAK2	-0.60813	-0.45083	0.018689	-0.04891	-0.31243	-0.11896	-0.28169	0.240103
JAK3	0.159693	0.130683	0.110034	0.779597	-0.03608	0.450398	-0.37601	-0.24352
JAKMIP1	0.064426	0.596583	0.117046	0.320733	-0.18345	0.310946	-0.05776	-0.23915
JAKMIP2	-0.10733	0.219209	0.23901	0.055413	-0.38248	-0.03556	-0.28363	-0.24045
JAKMIP3	NA	NA	0.047121	-0.21006	-0.18749	-0.05893	NA	NA
JAM2	-0.49146	-0.20259	-0.00205	0.015065	-0.13681	0.100671	-0.14959	-0.02004
JAM3	-0.21154	-0.0412	-0.0362	0.029865	-0.05294	0.075276	-0.39562	-0.40762
JARID1A	0.288113	0.418762	-0.01332	-0.09839	-0.02487	0.100011	-0.31805	-0.26013
JARID1B	-0.11754	-0.09	-0.19601	-0.22841	-0.36863	0.425141	0.376315	0.265426
JARID2	0.276611	-0.35598	-0.24358	0.090743	0.032996	0.208786	0.23811	-0.07338
JAZF1	-0.38734	-0.38294	-0.15785	-0.04335	-0.35368	-0.17064	-0.52505	-0.47366
JDP2	-0.03173	0.70426	-0.06717	-0.03238	-0.19459	-0.05081	-0.40552	-0.35144
JHDM1D	NA	NA	0.278854	-0.14974	0.212289	0.353474	NA	NA
JMJD1A	-0.12409	-0.51357	-0.41979	-0.11178	-0.13054	-0.25504	-0.21676	-0.17746
JMJD1B	0.296105	-0.26988	-0.01191	0.138737	-0.03082	0.03434	0.370512	0.006022
JMJD1C	0.007204	-0.53969	0.077276	-0.38746	0.046445	0.242099	-0.23818	0.08498
JMJD2A	0.009078	-0.04269	0.247633	0.07198	0.21353	-0.03198	-0.03993	0.164353
JMJD2C	-0.18414	-0.56845	-0.23918	-0.07424	0.068091	-0.11674	-0.04088	0.167833
JMJD2D	0.025475	0.438863	-0.15207	0.224077	-0.16159	0.048446	-0.10183	-0.10091
JMJD4	0.191915	0.691937	0.089423	0.1199	0.090895	-0.09864	0.572012	0.420019
JMJD5	-0.04244	0.30162	-0.2058	0.126342	0.200226	-0.11626	-0.22694	0.468079
JMJD6	-0.33303	-0.12353	0.232566	0.053677	0.146509	0.004384	NA	NA
JMJD8	NA	NA	-0.09587	0.110108	0.212343	-0.3721	NA	NA
JMY	0.170465	-0.41446	-0.07816	-0.15816	-0.06672	0.138165	-0.10918	0.12824
JOSD1	-0.39655	0.422608	0.043897	-0.05158	0.075594	0.014976	-0.58233	-0.32161
JOSD2	0.445188	0.572734	0.305115	-0.05552	0.029168	-0.23625	0.162611	-0.22046
JPH1	-0.21496	0.529278	-0.22991	0.079787	-0.13958	0.020647	0.499963	0.185698
JPH2	-0.05706	-0.09583	0.186041	-0.04953	0.187525	0.047217	-0.35486	-0.34235
JPH3	0.247571	0.364113	-0.05865	0.032011	-0.08122	-0.33813	-0.41473	-0.07559
JPH4	0.25537	0.398117	NA	NA	NA	NA	-0.42023	-0.32757
JRK	0.128585	-0.32756	0.291846	0.033094	0.367752	0.333523	0.419198	0.251511
JRKL	0.201795	-0.16938	-0.00234	-0.39641	0.054536	-0.34005	0.458176	0.335645
JSRP1	-0.16556	0.416266	0.123685	0.216187	-0.42507	-0.09979	-0.18606	-0.29386
JTB	0.254919	0.748737	-0.15992	0.076407	-0.29984	-0.33777	0.661513	0.509975
JTV1	0.283326	-0.23021	-0.05734	0.031667	0.021901	0.019073	0.068357	0.016635
JUB	0.19039	0.165355	-0.17832	0.022192	0.10046	0.010823	-0.04624	0.045468
JUN	0.504887	-0.02852	0.156377	-0.05778	-0.04328	-0.09965	0.270173	0.019386

JUNB	-0.28171	0.256051	0.065512	0.18061	0.04307	-0.07067	-0.10745	-0.08615
JUND	0.101129	0.468952	0.08542	-0.02214	-0.07566	-0.18896	-0.09846	0.155265
JUP	0.324952	0.058787	0.249111	-0.02881	0.137152	0.151429	0.57404	0.177486
KAAG1	-0.26269	0.080865	-0.11166	-0.03659	-0.18298	0.093591	0.055929	-0.00577
KAL1	0.04599	0.301528	-0.03805	0.032009	-0.33882	-0.19215	0.054375	0.113313
KALRN	0.103587	0.482618	-0.35005	0.01672	-0.0617	-0.21932	0.445274	0.677081
KANK1	NA	NA	0.283266	-0.11451	0.242999	0.444253	NA	NA
KANK2	NA	NA	0.13469	-0.0902	0.214578	0.290181	NA	NA
KANK3	NA	NA	0.089184	-0.01522	-0.05905	0.262469	NA	NA
KANK4	NA	NA	-0.07955	0.012567	-0.18032	0.19488	NA	NA
KARS	0.46417	-0.01962	-0.00347	0.274124	0.153947	-0.00223	0.523917	0.116836
KAT2A	NA	NA	0.114091	0.297781	0.167894	0.276849	NA	NA
KAT2B	NA	NA	-0.086	-0.26519	-0.01535	-0.43206	NA	NA
KAT5	NA	NA	0.044373	0.246001	0.176768	0.385597	NA	NA
KATNA1	0.272946	-0.60358	-0.46589	-0.20776	0.103782	-0.26316	0.538705	0.343499
KATNAL1	-0.22029	0.016352	0.260457	-0.04628	0.12426	0.046539	-0.22974	-0.30336
KATNAL2	0.213499	-0.051	-0.42185	-0.01973	-0.09609	0.149613	-0.40081	-0.18249
KATNB1	0.463397	0.679868	0.441659	0.087843	0.456392	0.088078	0.121179	-0.06899
KAZALD1	0.256214	0.247894	-0.31665	0.046732	0.073373	-0.43822	0.172081	0.30079
KBTBD10	-0.07166	-0.29024	0.241682	-0.15757	0.330429	-0.06396	-0.1115	0.046667
KBTBD11	0.025905	0.180339	-0.03038	0.048269	0.188145	0.268675	-0.02948	0.09196
KBTBD2	0.343854	0.194689	-0.19789	0.065702	0.010913	0.060582	-0.36385	-0.39605
KBTBD3	0.167115	-0.02163	-0.02153	-0.36964	-0.32821	-0.25124	0.102378	0.171949
KBTBD4	0.465519	-0.45351	-0.09461	0.207073	0.004357	-0.15132	0.672099	0.223712
KBTBD5	-0.03867	0.6552	-0.10965	-0.04124	0.160225	-0.18413	-0.26024	-0.14324
KBTBD6	0.109261	-0.20387	-0.01824	-0.16358	0.125645	0.086985	0.006156	-0.22733
KBTBD7	0.062347	-0.47498	-0.11949	-0.33211	0.055458	-0.12231	0.228049	-0.37828
KBTBD8	0.232396	0.530624	-0.40711	0.219666	-0.1317	0.025055	-0.26965	0.032282
KCMF1	-0.23878	-0.59391	-0.0913	-0.35937	0.09699	-0.15695	-0.04246	-0.18385
KCNA1	0.063438	0.169856	0.095134	-0.10619	-0.04088	-0.00653	-0.15333	-0.05314
KCNA10	-0.13085	0.667218	-0.15223	0.013813	0.186501	-0.16766	-0.01741	0.253054
KCNA2	0.310409	0.211466	0.120134	-0.07718	0.084727	0.249359	0.335602	-0.12011
KCNA3	-0.03927	0.443042	-0.03996	0.22465	-0.28061	0.405638	0.065703	0.080169
KCNA4	-0.22813	0.037605	-0.00887	-0.24447	-0.23672	0.12896	-0.44624	-0.31597
KCNA5	-0.1746	0.007332	-0.05254	-0.15446	-0.05131	-0.17265	-0.04165	-0.17572
KCNA6	0.231317	-0.09868	0.035346	0.165169	0.341991	0.107949	0.175292	0.200643
KCNA7	-0.3625	-0.22037	-0.00731	-0.14607	-0.09445	0.145054	0.000563	0.168438
KCNAB1	-0.26009	0.11325	-0.17933	-0.20191	-0.00605	0.139947	-0.09121	-0.11774
KCNAB2	0.046201	0.117204	0.19256	0.54441	0.185237	0.371341	0.23967	0.052573
KCNAB3	0.221335	0.046615	-0.24353	0.14523	-0.05989	0.072262	0.075989	0.074534
KCNB1	-0.08508	0.071546	0.048396	-0.14318	0.016082	0.071301	-0.32381	-0.25421
KCNB2	-0.12745	0.430546	0.243776	0.026953	-0.13122	0.041247	0.136498	0.277904
KCNC1	0.041422	0.534932	0.009688	0.024674	-0.0381	0.014905	-0.19048	-0.04311
KCNC2	0.193801	0.796199	-0.16675	0.030933	0.074884	-0.11131	-0.26358	-0.13794
KCNC3	0.135607	0.214035	0.176259	-0.0951	0.305484	0.171048	0.218504	0.0953
KCNC4	0.203093	0.638955	0.272165	0.148595	0.337582	0.254361	0.100074	0.281615
KCND1	-0.05087	-0.17552	-0.02901	0.025465	0.236824	0.108157	-0.5901	-0.31266
KCND2	-0.5208	-0.38857	0.13152	-0.31963	-0.04809	0.018529	-0.22422	0.196693
KCND3	0.062487	-0.05889	0.080842	0.019384	-0.10568	0.229685	0.059538	-0.17017
KCNE1	NA	NA	-0.35321	0.030657	-0.08877	-0.38825	0.35515	-0.07586
KCNE1L	0.306968	0.629579	0.079677	0.288621	-0.00696	-0.14907	0.078247	-0.44704
KCNE2	0.317462	0.321056	-0.40718	0.056698	-0.1594	0.05241	-0.27518	-0.28148

KCNE3	-0.03524	0.045959	0.080218	0.088171	0.194933	0.094471	0.265795	0.166473
KCNE4	-0.37226	0.099752	0.133757	-0.19317	-0.06118	-0.19918	-0.30976	0.072715
KCNF1	-0.04994	0.36787	0.052138	-0.12003	0.204774	0.149707	-0.10158	0.028998
KCNG1	0.068352	0.333153	-0.15554	-0.04155	-0.13299	-0.16277	-0.13105	0.004984
KCNG2	-0.09651	0.223581	-0.20956	-0.10904	-0.27019	0.277188	0.01758	0.254299
KCNG3	0.254735	0.266012	-0.09067	-0.22777	-0.23662	-0.37999	-0.01404	0.010234
KCNG4	0.129588	0.339795	0.114452	-0.04331	0.06715	-0.06505	0.453697	0.290637
KCNH1	-0.11502	0.431103	0.236549	-0.1119	0.264212	-0.05452	-0.12317	0.071398
KCNH2	0.212744	-0.1993	0.121162	-0.07439	0.273454	-0.31457	-0.04941	-0.16259
KCNH3	0.235501	-0.1569	0.050397	-0.18511	-0.05151	0.202321	0.246814	0.249114
KCNH4	0.270393	0.674099	0.037141	-0.05071	-0.14605	-0.05312	0.068621	0.077416
KCNH5	0.256358	0.463856	0.071568	-0.38839	0.073251	0.006144	-0.02687	0.148624
KCNH6	0.29896	0.007679	0.023262	-0.04127	-0.10844	-0.06929	-0.51526	-0.27027
KCNH7	0.115876	0.34278	-0.38205	0.032675	0.027473	-0.10786	-0.40821	-0.19622
KCNH8	0.238109	-0.18571	-0.3829	0.108058	0.211973	-0.08737	0.240897	0.151666
KCNIP1	0.265695	-0.03754	-0.08536	0.192765	0.279294	-0.02048	-0.56245	-0.13877
KCNIP2	-0.04886	0.430538	0.178551	0.285825	0.095134	-0.10218	-0.36763	0.141772
KCNIP3	0.004369	-0.03793	-0.00379	-0.02619	-0.10602	0.1919	-0.06104	-0.3426
KCNIP4	-0.04079	-0.03246	-0.15104	-0.08413	0.118083	0.040518	-0.30948	-0.20365
KCNJ1	-0.07818	-0.12011	-0.10539	0.031427	-0.17549	-0.29364	0.391521	0.550641
KCNJ10	0.130057	0.638353	0.171919	0.585563	-0.07113	0.171522	-0.29342	-0.31722
KCNJ11	0.235607	0.599339	0.204013	-0.08197	0.062606	-0.21099	0.378311	0.305515
KCNJ12	-0.06508	0.568166	-0.26212	0.198151	-0.04306	-0.08006	-0.16132	0.075757
KCNJ13	0.022166	0.048767	-0.10186	-0.23891	0.084337	0.015598	0.059672	0.493288
KCNJ14	0.153721	0.582889	-0.14677	0.108562	0.303494	-0.08433	0.363683	-0.2072
KCNJ15	-0.65801	-0.14791	0.131572	0.155157	-0.01231	-0.14785	-0.03082	0.347922
KCNJ16	0.091562	0.807587	0.100452	0.005297	-0.23733	-0.29691	0.089782	0.518385
KCNJ2	-0.10442	-0.05441	0.39886	-0.07983	0.073293	-0.098	0.105936	-0.33091
KCNJ3	-0.24358	0.379884	0.52131	-0.10874	-0.15333	-0.33093	0.018003	0.567611
KCNJ4	0.101933	0.683184	0.097945	-0.27849	0.24492	0.226848	0.24862	0.033529
KCNJ5	0.04426	-0.11735	0.415256	0.11973	-0.19183	0.14498	-0.17487	0.012433
KCNJ6	-0.15585	-0.07657	-0.19222	-0.14575	-0.23672	-0.07003	0.163277	0.156369
KCNJ8	-0.25576	-0.10104	0.025591	0.012726	-0.25393	-0.10742	-0.24419	-0.08695
KCNJ9	0.222056	0.632582	0.104485	-0.22047	0.207376	-0.20571	0.049617	0.017418
KCNK1	-0.38501	-0.04616	-0.21005	-0.0292	-0.15129	-0.08474	-0.19328	-0.24904
KCNK10	0.270171	0.196291	-0.12994	-0.20302	-0.26571	0.049445	-0.09359	-0.25198
KCNK12	-0.28762	0.71633	0.143357	0.620919	0.126839	0.169885	-0.27014	-0.13386
KCNK13	-0.03241	0.471655	0.254548	0.330175	-0.29208	0.246215	-0.25806	0.057495
KCNK15	0.280027	0.244389	0.399251	-0.11535	0.159927	-0.05458	-0.41697	-0.21738
KCNK16	-0.17648	0.268717	-0.04021	-0.00646	-0.21195	0.125635	-0.09309	0.053169
KCNK17	-0.21235	-0.53882	0.306321	-0.28256	0.121126	-0.05773	-0.02387	0.079025
KCNK18	0.389425	0.418913	0.003843	0.051696	-0.28579	0.014007	0.360578	0.513294
KCNK2	0.137324	0.477664	0.17356	0.230829	-0.24558	0.119136	-0.11971	0.409058
KCNK3	-0.07682	0.364295	0.018449	-0.10972	0.062675	-0.13745	-0.39406	-0.27827
KCNK4	0.208279	0.514713	-0.09531	-0.23519	-0.16653	0.175583	0.136131	0.262302
KCNK5	0.099472	0.136584	-0.2038	-0.45916	0.424825	-0.02699	-0.14416	-0.03784
KCNK6	-0.43199	0.137698	0.205639	0.136917	0.035424	-0.12777	-0.66316	-0.60858
KCNK7	-0.15181	0.761184	0.221394	0.062813	0.035305	-0.12471	-0.0199	-0.00501
KCNK9	0.027356	0.475427	0.085717	0.026189	-0.01198	0.049274	-0.0473	-0.14899
KCNMA1	0.018035	0.007503	-0.10509	-0.18048	-0.09765	-0.29201	-0.31739	-0.36236
KCNMB1	-0.20735	-0.14331	0.041285	-0.10281	-0.12218	-0.16426	-0.23139	-0.30216
KCNMB2	-0.11669	0.003754	-0.20454	-0.33856	-0.48848	-0.05777	0.034145	-0.1433

KCNMB3	0.156722	-0.54031	-0.43666	-0.20555	0.026076	0.022861	0.435265	0.385102
KCNMB4	0.105944	0.132804	0.417136	-0.12362	0.13002	-0.04154	-0.40589	-0.51063
KCNN1	0.078122	0.658257	-0.08216	0.05008	0.059879	-0.09772	0.311286	-0.08949
KCNN2	0.085607	-0.38342	-0.22099	0.131565	-0.21111	-0.14347	-0.18523	-0.18284
KCNN3	0.028074	-0.23436	0.231396	0.041213	-0.11185	-0.20584	-0.37076	-0.29778
KCNN4	0.198226	0.433417	-0.25302	0.549106	0.15846	0.60584	0.182499	0.033076
KCNQ1	0.418593	0.45537	0.282915	0.512589	0.459453	0.132627	-0.07518	-0.08451
KCNQ1DN	-0.3732	0.050443	-0.04933	-0.06594	-0.07928	-0.02016	-0.15578	-0.03532
KCNQ1OT1	NA	NA	-0.36245	-0.03537	-0.07976	-0.03012	NA	NA
KCNQ2	-0.39453	-0.35075	-0.01685	-0.17582	-0.00943	-0.08623	-0.42056	-0.32192
KCNQ3	0.297101	0.557085	-0.02212	0.046857	-0.22694	-0.16734	-0.31238	-0.44444
KCNQ4	0.068038	-0.19757	-0.02655	-0.27462	0.204628	-0.02583	-0.57257	-0.15429
KCNQ5	-0.17354	0.434244	-0.03888	0.337734	-0.00292	-0.27181	0.569427	0.09595
KCNRG	-0.16936	0.093912	-0.23716	-0.19444	0.133336	0.291937	0.431951	0.154559
KCNS1	0.340191	-0.17894	-0.22505	-0.01349	0.115439	-0.00287	-0.38026	-0.22563
KCNS2	0.228356	0.3897	-0.17346	0.131952	-0.26942	-0.29219	0.454321	0.374186
KCNS3	-0.42927	-0.1079	-0.22374	0.119984	-0.20569	-0.0202	-0.3049	-0.18006
KCNT1	0.079595	-0.35519	0.02308	-0.17798	-0.13438	0.269589	-0.10283	-0.22412
KCNT2	0.398154	0.469321	NA	NA	NA	NA	-0.25885	-0.00765
KCNV1	0.09036	0.0926	0.084738	-0.13841	-0.31329	-0.15389	0.034681	0.105966
KCNV2	-0.03744	0.523916	-0.06058	-0.14472	-0.07322	-0.02903	NA	NA
KCTD1	-0.06341	-0.43728	-0.205	-0.14101	-0.49435	-0.36869	-0.18625	-0.49289
KCTD10	-0.36557	-0.36554	-0.36639	-0.17308	-0.05071	-0.00783	-0.44568	-0.39742
KCTD11	-0.30739	0.511328	0.152309	0.3738	-0.00211	0.234866	-0.27238	-0.01951
KCTD12	-0.10516	-0.27408	-0.27313	0.004003	-0.20484	0.212839	-0.62808	-0.4935
KCTD13	0.206035	0.475912	0.174512	0.236495	0.15116	-0.05426	0.250646	0.379512
KCTD14	-0.08541	-0.2972	0.137434	0.098947	-0.01338	-0.28827	0.519561	0.32051
KCTD15	-0.31731	0.094033	-0.26725	0.099353	-0.15792	0.06096	-0.11056	-0.04452
KCTD16	0.18429	0.278764	0.044039	-0.12782	-0.01614	-0.0736	0.490154	0.196893
KCTD17	0.293018	0.466486	0.506559	-0.0674	0.389231	0.012413	-0.33557	-0.28792
KCTD18	-0.02887	-0.61062	0.082448	-0.05255	0.333461	0.042702	0.02745	0.067756
KCTD19	NA	NA	-0.0862	0.172168	0.145137	0.299785	NA	NA
KCTD2	-0.19511	0.361537	0.453429	-0.05154	0.219024	-0.0293	0.143909	0.296372
KCTD20	-0.32323	-0.19753	0.30657	-0.22946	0.192963	0.151219	NA	NA
KCTD21	-0.01774	-0.1837	0.212505	0.021417	0.291124	-0.1347	NA	NA
KCTD3	-0.4573	-0.68761	0.045299	-0.30909	0.047444	-0.22439	0.041882	0.078
KCTD4	0.235517	0.320452	-0.06133	-0.07392	-0.2464	-0.29498	0.235499	0.267703
KCTD5	0.031649	0.218288	0.125884	-0.05235	0.321355	0.21126	0.372953	0.070351
KCTD6	0.069277	-0.65855	-0.52731	-0.38366	-0.06519	-0.1688	-0.27815	-0.10994
KCTD7	0.016243	0.325111	0.350248	-0.12487	-0.20093	0.058574	-0.69036	-0.57128
KCTD8	-0.12127	0.069072	0.016262	-0.1406	-0.04062	-0.13362	-0.38833	-0.36896
KCTD9	-0.06704	0.389903	-0.08931	-0.49782	-0.3151	-0.22447	-0.38072	-0.32393
KDELC1	0.049915	-0.41573	-0.0116	-0.20446	0.028304	-0.27933	-0.14791	-0.03494
KDELC2	-0.31182	-0.30026	-0.22736	0.155386	-0.17828	-0.07548	-0.24477	0.082601
KDELR1	0.151199	0.558269	0.171739	0.222078	0.05047	-0.09631	0.011373	-0.29019
KDELR2	0.025703	-0.06594	-0.22785	0.176286	-0.08677	-0.3382	0.321812	0.20482
KDELR3	-0.54427	-0.31819	0.019123	0.159293	-0.30881	-0.30341	0.273706	0.112955
KDR	-0.12854	0.093427	-0.06715	0.294436	0.031934	0.217099	-0.00123	-0.14042
KDSR	NA	NA	0.182617	-0.09717	0.139378	0.167086	NA	NA
KEAP1	0.383899	0.578273	0.009933	0.36391	0.422199	0.429387	0.227908	-0.33103
KEL	-0.21369	-0.13879	-0.12513	0.380957	0.108923	0.339881	0.294143	0.042454
KERA	0.131664	0.374479	0.175511	-0.01835	-0.28253	-0.05393	-0.48034	-0.08221

KGFLP1	NA	NA	-0.33255	-0.07794	-0.1989	0.01172	-0.01268	0.149354
KGFLP2	NA	NA	-0.22811	-0.0945	-0.02087	0.026828	NA	NA
KHDC1	NA	NA	-0.03547	0.316951	-0.04322	-0.12266	NA	NA
KHDRBS1	-0.1264	-0.2639	-0.00225	0.287298	0.226207	0.300541	0.339079	-0.15246
KHDRBS2	0.014193	0.03949	-0.10946	0.401881	-0.23128	0.022525	-0.49643	0.088461
KHDRBS3	0.441295	-0.24925	0.055078	0.063842	0.057965	-0.01867	-0.15935	-0.40772
KHK	0.076367	-0.10016	0.404796	0.306106	0.281943	-0.07046	0.378102	0.507132
KHSRP	0.418889	0.623908	0.00733	0.372937	0.330626	0.144564	0.628741	0.054848
KIAA0020	0.289847	-0.57895	-0.34994	-0.09267	0.06797	0.254181	0.302014	0.252799
KIAA0040	-0.3179	0.522989	-0.04235	-0.00081	-0.20667	0.207978	NA	NA
KIAA0090	-0.11728	-0.44576	-0.0857	-0.00676	0.239047	0.104792	0.084584	0.002724
KIAA0100	0.432015	0.16354	0.379586	0.336803	0.0078	0.258416	-0.59783	-0.50599
KIAA0101	-0.17681	0.084843	-0.27534	0.214087	-0.0526	-0.19693	0.369189	0.248184
KIAA0125	-0.29402	0.067416	0.27685	0.273941	-0.11748	0.203867	NA	NA
KIAA0141	0.178415	-0.48247	0.399517	0.156638	0.207132	0.246568	0.201327	0.470967
KIAA0146	NA	NA	0.333032	0.382859	0.247493	0.147181	NA	NA
KIAA0174	-0.12225	-0.18011	-0.07449	0.047747	0.272317	-0.1695	-0.04002	-0.04974
KIAA0182	-0.12336	0.002091	-0.00515	0.116876	0.341479	0.080989	0.140428	-0.07482
KIAA0195	-0.36147	0.256422	0.27673	0.116427	0.328765	-0.03932	0.527112	0.439111
KIAA0196	0.02928	-0.63625	0.1076	-0.38156	-0.14997	0.032466	0.601311	0.338588
KIAA0226	NA	NA	-0.23385	0.109525	0.036303	0.241215	NA	NA
KIAA0232	0.289112	-0.30063	-0.11878	-0.23115	0.138654	-0.21835	0.015325	0.171196
KIAA0240	0.39606	0.315879	-0.07252	0.093681	0.189546	0.006407	-0.01271	-0.22018
KIAA0247	-0.43276	-0.14197	0.034525	0.075922	0.036848	0.282423	-0.43678	0.207445
KIAA0256	0.019733	0.143668	-0.25881	-0.42892	-0.18771	-0.30718	-0.07117	-0.03666
KIAA0284	0.054718	0.491553	-0.10313	0.074448	0.116236	0.280514	0.625686	0.482977
KIAA0317	0.023275	0.326926	0.058199	-0.09021	-0.30098	0.114718	NA	NA
KIAA0319	-0.58639	-0.16623	-0.30018	-0.0161	-0.35155	0.061088	-0.01534	0.009552
KIAA0319L	-0.36109	-0.1229	0.288439	0.092212	0.414706	0.309322	0.482552	0.090971
KIAA0323	0.247868	0.303348	-0.00975	0.0168	0.114823	-0.13416	0.312766	0.296299
KIAA0329	0.111683	0.542747	0.006308	-0.03334	0.049243	0.162131	-0.20423	0.12726
KIAA0355	0.036318	-0.58509	-0.14167	-0.03335	0.06768	0.054816	0.224567	0.195985
KIAA0368	NA	NA	0.006627	-0.06121	0.030845	-0.2229	0.330329	0.11583
KIAA0391	0.126915	0.528485	-0.34323	0.136586	-0.31859	-0.4377	-0.35812	-0.29307
KIAA0406	0.467967	-0.01612	0.199677	0.304449	0.272211	0.112367	0.507422	0.584801
KIAA0408	-0.01597	0.23191	0.099496	-0.16536	-0.02074	0.097821	-0.6434	-0.55601
KIAA0409	0.039241	0.214405	-0.46634	0.155063	0.307601	-0.00154	-0.28497	-0.46915
KIAA0415	NA	NA	0.336708	-0.04215	0.37882	0.306972	-0.09462	0.174605
KIAA0427	-0.106	-0.05951	0.384446	-0.08212	0.068685	0.1109	-0.05017	-0.05624
KIAA0430	-0.22932	-0.58333	-0.31696	0.023498	0.088672	-0.18524	NA	NA
KIAA0467	-0.04086	0.602458	0.124135	0.148595	0.296164	0.226908	-0.47098	-0.31209
KIAA0494	-0.21921	-0.65654	0.317498	-0.18402	0.022023	-0.23888	0.456099	0.262567
KIAA0495	-0.30863	-0.23169	0.357248	0.210668	0.124071	0.476472	-0.58732	-0.20046
KIAA0513	-0.44535	0.29048	0.007699	-0.23269	-0.17221	-0.03023	-0.25286	0.023604
KIAA0528	0.487549	0.409442	-0.46389	-0.13717	-0.14182	-0.33222	-0.2491	-0.13626
KIAA0556	0.162001	-0.38589	0.207646	-0.01409	0.358054	0.209344	-0.32199	-0.47066
KIAA0562	NA	NA	-0.18878	-0.19607	0.435873	0.062715	0.58301	0.282281
KIAA0564	0.295957	-0.50803	0.123576	-0.16422	0.095851	0.024556	0.157452	0.068291
KIAA0586	-0.14123	-0.46878	-0.37837	-0.03608	-0.07686	-0.0163	-0.53324	-0.27897
KIAA0644	-0.06741	-0.01067	-0.10048	0.056682	-0.36613	-0.1845	-0.05549	0.092275
KIAA0649	0.153881	-0.12818	0.033634	-0.07474	0.48519	0.312085	0.191915	-0.15101
KIAA0652	0.163159	0.492492	0.270681	0.000913	0.091597	-0.00906	-0.03266	0.166541

KIAA0664	0.025461	0.143272	0.13031	0.134724	0.204773	0.20719	0.387301	0.257143
KIAA0746	-0.21584	0.028752	0.140559	0.231403	0.138727	-0.35388	0.313483	0.018767
KIAA0753	0.293001	-0.44171	-0.297	-0.20802	-0.07957	0.375763	-0.37329	-0.38792
KIAA0754	NA	NA	0.128436	-0.40431	0.24161	0.542132	NA	NA
KIAA0774	-0.04917	-0.07884	-0.00618	-0.18081	-0.14639	-0.42252	-0.20064	-0.05228
KIAA0776	-0.11675	-0.46145	-0.17076	-0.31864	0.120956	-0.06979	0.413126	0.222913
KIAA0802	0.071581	-0.35065	-0.21678	0.043149	-0.0618	-0.37484	0.08955	-0.07187
KIAA0831	-0.0875	-0.02986	-0.53141	0.042602	-0.22388	-0.1394	-0.64911	-0.54616
KIAA0859	0.503809	0.206265	-0.18378	0.41162	0.028913	0.215329	0.407177	0.144962
KIAA0892	0.34436	-0.02497	0.039149	-0.03698	0.062962	0.180188	0.253513	0.61844
KIAA0895	-0.13899	-0.37102	-0.20562	-0.03027	-0.10247	-0.40812	0.182213	-0.10961
KIAA0895L	NA	NA	-0.15599	0.071204	0.277405	0.136183	NA	NA
KIAA0907	0.153088	-0.30842	-0.06757	-0.04471	0.052435	-0.1177	0.097118	-0.05253
KIAA0913	-0.47123	0.157357	0.017216	-0.04892	0.030859	0.196189	-0.19371	0.369653
KIAA0922	-0.1928	-0.22127	-0.09987	0.662243	-0.01265	0.574619	0.207021	0.188123
KIAA0947	NA	NA	-0.30881	-0.24518	0.027369	0.317665	NA	NA
KIAA0999	-0.11963	-0.16186	-0.20118	-0.11679	-0.07683	0.339762	-0.59406	-0.5061
KIAA1009	0.329784	0.17414	-0.29017	-0.01194	0.150936	-0.28478	NA	NA
KIAA1012	0.040925	-0.32603	-0.03304	-0.33445	-0.36457	-0.19437	-0.1421	0.205187
KIAA1024	0.081288	-0.48036	NA	NA	NA	NA	-0.06146	0.121122
KIAA1033	-0.01231	-0.50095	-0.04226	-0.23722	0.262896	0.318288	-0.40741	-0.38815
KIAA1045	NA	NA	0.080664	0.521367	-0.21195	0.409514	NA	NA
KIAA1107	NA	NA	0.052419	-0.11613	0.211911	-0.10909	NA	NA
KIAA1109	NA	NA	0.062012	-0.34574	-0.17353	-0.0959	NA	NA
KIAA1128	0.03811	-0.47723	0.015709	-0.25877	0.191208	0.093273	0.252618	0.083576
KIAA1143	-0.05774	0.074227	-0.29553	-0.17668	-0.24177	-0.28883	0.246918	0.390839
KIAA1147	NA	NA	-0.14604	0.226639	0.344928	-0.03416	0.397264	0.310777
KIAA1161	0.229728	0.302606	0.093899	0.099085	0.122746	-0.18468	-0.31439	0.031807
KIAA1191	-0.01679	-0.14348	0.093978	-0.20792	-0.02725	-0.16039	-0.01715	-0.00946
KIAA1199	-0.07066	-0.21451	0.194188	0.194605	0.370099	0.476695	-0.08887	-0.00994
KIAA1210	NA	NA	0.281517	0.091453	0.057518	-0.00238	NA	NA
KIAA1211	NA	NA	-0.0561	-0.13675	-0.29943	-0.08518	NA	NA
KIAA1217	-0.05232	0.049777	0.166855	-0.27055	0.13376	-0.16594	0.22103	0.164526
KIAA1219	0.257428	-0.33159	0.187567	-0.13485	0.391794	0.244437	0.505729	0.463313
KIAA1239	NA	NA	-0.36993	-0.32454	0.096136	-0.32867	NA	NA
KIAA1244	-0.26332	-0.28667	-0.36153	-0.15794	0.073225	-0.19572	-0.00789	-0.4733
KIAA1257	NA	NA	-0.19591	-0.18859	0.065018	0.097005	NA	NA
KIAA1267	0.175013	-0.31065	-0.11782	0.026526	-0.0706	-0.05959	-0.3863	-0.41856
KIAA1274	0.335061	-0.22525	0.168438	0.06419	0.420167	0.360083	0.042178	-0.21175
KIAA1279	-0.20211	-0.62307	-0.14186	-0.26574	-0.04032	-0.32499	-0.70538	-0.29312
KIAA1324	-0.19773	0.369085	-0.1028	0.164571	0.103655	0.055375	0.215657	0.09439
KIAA1324L	0.339067	-0.27078	0.16585	-0.17008	0.058881	0.329507	0.292277	-0.1485
KIAA1328	0.265546	0.583299	0.154815	-0.17949	-0.1165	-0.18013	-0.49201	-0.3269
KIAA1333	0.277814	0.409761	-0.55683	-0.14022	-0.22797	-0.1362	-0.25241	-0.14063
KIAA1370	-0.28898	-0.57858	-0.08584	-0.01343	-0.15413	-0.07783	-0.33861	-0.15764
KIAA1377	0.214712	-0.30118	-0.02271	-0.07567	-0.071	-0.28737	-0.27264	0.038536
KIAA1383	NA	NA	-0.28319	-0.00186	-0.04008	0.073017	NA	NA
KIAA1407	0.176834	0.011125	0.036691	-0.18443	0.105368	0.382158	0.078688	0.278907
KIAA1409	0.296359	-0.26855	0.174318	-0.07751	0.03539	0.267546	-0.36533	0.113144
KIAA1429	-0.01547	-0.32818	-0.07974	-0.2717	-0.15353	0.078489	0.659193	0.271749
KIAA1430	NA	NA	-0.23362	-0.04069	0.103784	0.062698	NA	NA
KIAA1432	0.211489	0.724826	-0.23664	-0.34549	-0.21422	-0.49908	0.079695	0.483799

KIAA1456	0.367784	0.332783	NA	NA	NA	NA	0.078667	0.005073
KIAA1462	NA	NA	0.03396	-0.01569	-0.04763	0.208752	NA	NA
KIAA1467	0.492335	-0.27535	0.333835	0.430996	-0.30537	-0.20096	0.082736	0.378498
KIAA1468	-0.04575	-0.3964	-0.17744	-0.20405	-0.06614	-0.08157	-0.05677	0.236802
KIAA1522	-0.52893	0.052908	0.075442	-0.0979	0.091348	-0.07722	0.198435	-0.12638
KIAA1524	-0.27588	-0.02602	-0.28485	-0.00838	0.006506	0.265881	0.215443	-0.10656
KIAA1529	0.096744	0.351503	0.012807	-0.00505	-0.03434	-0.12475	-0.14789	-0.1499
KIAA1530	0.028588	0.555445	-0.21522	0.173813	0.120243	0.292048	-0.41447	-0.13227
KIAA1539	-0.20635	0.531916	0.209129	-0.10739	0.059329	-0.1045	-0.27859	0.142528
KIAA1543	NA	NA	0.190018	0.103445	0.397025	0.217446	0.631896	0.170424
KIAA1549	NA	NA	0.148561	0.055853	0.400221	-0.00953	NA	NA
KIAA1586	-0.16421	-0.27985	-0.06196	0.060251	-0.17479	-0.14679	0.01893	-0.10152
KIAA1598	-0.00013	-0.20285	-0.24107	-0.06808	0.265358	-0.05819	0.525553	0.434158
KIAA1609	-0.02331	0.00994	0.224926	-0.13237	0.166799	-0.0535	0.204981	0.165065
KIAA1614	NA	NA	0.290417	-0.14532	-0.24147	0.365272	NA	NA
KIAA1618	-0.18523	0.474465	0.469231	0.022679	0.073213	0.595649	0.077608	-0.00417
KIAA1627	0.075243	0.195016	0.072083	-0.0729	-0.00446	0.388408	-0.2774	-0.44328
KIAA1632	-0.20074	-0.46748	-0.05961	-0.17155	-0.08621	-0.11672	-0.14371	0.148599
KIAA1683	-0.03453	0.372189	-0.32468	-0.1944	-0.14732	-0.18185	-0.59345	-0.12656
KIAA1688	0.006032	0.532908	0.1276	-0.00441	0.094226	0.030136	0.354539	-0.21634
KIAA1704	0.400102	0.48896	-0.05618	-0.08932	0.172965	-0.02784	0.251308	-0.28253
KIAA1712	0.285847	0.228641	-0.4595	-0.20429	-0.03662	-0.47718	0.083294	0.0208
KIAA1715	0.104187	0.230429	-0.04458	-0.17836	-0.04714	-0.2862	0.154713	0.150179
KIAA1731	NA	NA	-0.29889	0.083458	-0.03186	0.013854	NA	NA
KIAA1737	0.061877	-0.51852	-0.1244	-0.36121	0.070879	-0.23971	-0.20599	-0.28199
KIAA1751	NA	NA	0.251353	0.146466	-0.18525	-0.37773	-0.5595	-0.5593
KIAA1755	0.117851	-0.5482	0.167572	0.094232	-0.04017	0.316456	-0.09824	-0.20226
KIAA1772	0.370824	-0.01727	0.226169	-0.32645	0.354473	0.109825	0.095616	0.12984
KIAA1787	-0.22168	0.134016	0.044417	0.052485	0.011825	0.410373	0.378081	0.489961
KIAA1797	0.169912	0.321566	-0.00617	0.083052	0.143796	-0.03054	0.298581	-0.00702
KIAA1804	-0.20157	0.308532	-0.4807	0.082803	0.139122	-0.27976	0.141803	-0.06756
KIAA1826	0.194588	-0.29894	-0.001	-0.30936	-0.18098	-0.27016	0.289755	0.093843
KIAA1841	-0.30196	-0.03893	-0.04976	-0.2608	0.30835	-0.39381	0.227	0.713395
KIAA1853	0.053301	0.386441	0.107496	-0.48296	0.370811	-0.03587	-0.30678	-0.0744
KIAA1875	0.219029	0.527379	-0.10046	0.052007	-0.12368	0.121355	0.03845	0.323608
KIAA1919	-0.05151	0.174385	0.154627	-0.44198	0.253302	0.202643	-0.42895	-0.45275
KIAA1958	0.360853	0.553836	-0.01178	-0.37839	0.201211	-0.29783	-0.06809	-0.29475
KIAA1967	0.024049	0.088664	0.17222	0.179766	0.123708	0.105592	0.141832	-0.21379
KIAA1984	-0.01189	0.400468	-0.05076	-0.11374	0.258813	0.224726	0.203212	-0.07986
KIAA2013	0.02075	0.262324	0.106446	-0.27816	0.320227	-0.20635	0.459894	0.216031
KIAA2018	0.160643	0.457696	-0.24563	-0.62914	-0.17511	-0.36307	-0.06856	-0.11345
KIAA2022	-0.23486	-0.29166	-0.05582	-0.09465	-0.16108	0.150371	-0.4447	-0.29801
KIAA2026	-0.12018	-0.62694	-0.30085	-0.24491	-0.01753	0.104217	0.414787	0.441892
KIDINS220	-0.06471	-0.71696	-0.11258	-0.44864	-0.06228	0.208918	0.504701	0.246602
KIF11	-0.35733	-0.11558	-0.34185	0.214829	0.044315	-0.09022	0.20631	0.022716
KIF12	-0.00169	0.077825	0.139107	0.219921	0.244183	-0.27089	0.601296	0.624174
KIF13A	-0.09887	0.378165	-0.14264	-0.50311	0.033751	0.188724	-0.45591	0.02849
KIF13B	-0.06867	-0.06701	0.118514	-0.04756	0.003871	0.281472	0.530812	0.297666
KIF14	-0.20181	0.464534	-0.37143	0.264606	-0.06029	-0.12824	0.316873	-0.10287
KIF15	-0.00546	-0.28554	-0.41969	0.040376	0.054484	-0.02774	0.259286	0.121071
KIF16B	NA	NA	-0.12679	-0.10546	-0.02403	-0.42206	NA	NA
KIF17	0.08835	0.004965	0.045223	-0.25117	-0.07893	-0.14876	-0.19801	0.059794

KIF18A	0.13623	0.855822	-0.49555	0.098077	-0.11814	-0.11936	0.21187	-0.05347
KIF18B	NA	NA	-0.36507	0.306343	0.030572	-0.02469	NA	NA
KIF19	-0.24036	0.010223	-0.07269	-0.02404	-0.13297	0.00446	0.002117	0.270498
KIF1A	0.045756	0.151589	0.203746	0.13982	-0.01648	-0.22227	-0.23404	-0.43321
KIF1B	-0.42547	0.439426	0.083473	-0.44557	0.072376	0.237047	-0.2899	-0.19392
KIF1C	-0.10889	-0.18769	0.127269	0.034562	-0.1191	0.03966	-0.51026	-0.57824
KIF20A	-0.02757	-0.14246	-0.23159	0.205175	-0.05969	-0.07188	0.450794	0.188094
KIF20B	NA	NA	-0.22408	0.290987	-0.02127	-0.10328	NA	NA
KIF21A	-0.18614	-0.25252	-0.31402	-0.03514	-0.18117	-0.07783	-0.25423	-0.4408
KIF21B	NA	NA	0.376107	0.494156	0.412469	0.353798	-0.36383	0.027885
KIF22	0.062653	-0.06997	-0.04019	0.284185	0.31305	0.117126	NA	NA
KIF23	-0.18975	0.14142	-0.45086	0.049128	0.041861	-0.04886	0.166025	-0.00492
KIF24	NA	NA	-0.29945	-0.00792	0.059996	-0.08919	NA	NA
KIF25	-0.04696	-0.14408	-0.0606	0.163832	0.133785	0.148576	-0.19587	0.061849
KIF26A	NA	NA	-0.41207	0.159685	-0.04728	-0.03995	NA	NA
KIF26B	NA	NA	0.075967	-0.16386	-0.11118	0.302513	0.060177	0.017674
KIF27	-0.04583	-0.53109	-0.13476	-0.09522	0.056983	0.085668	-0.16115	0.102804
KIF2A	0.264287	-0.24218	-0.09797	-0.29643	0.010438	-0.03768	NA	NA
KIF2B	0.392576	0.77232	0.483617	-0.0582	-0.12478	0.056763	-0.163	-0.20476
KIF2C	-0.26635	-0.09518	-0.16167	0.235188	0.227947	-0.01713	0.244589	-0.05806
KIF3A	-0.05678	0.507867	0.349509	0.113359	0.063631	0.42917	0.069604	-0.13607
KIF3B	0.515892	-0.43931	0.200021	-0.21999	0.225824	0.073365	0.536696	0.522767
KIF3C	0.180231	-0.40198	0.183056	0.060153	0.137248	0.172632	-0.04544	-0.22197
KIF4A	0.161116	-0.23649	-0.32393	0.230263	0.113125	-0.02294	0.401862	0.159376
KIF5A	-0.11992	0.308693	-0.0202	0.04948	0.032564	0.041094	-0.42839	-0.46639
KIF5B	0.049363	-0.19891	-0.17895	-0.11027	-0.02482	-0.2666	0.234892	0.377505
KIF5C	NA	NA	-0.05854	-0.48521	-0.11833	0.004199	-0.44636	-0.26892
KIF6	-0.05241	0.279771	-0.13528	-0.04632	-0.33418	0.035311	-0.14233	-0.21198
KIF7	0.140027	-0.11504	0.242872	-0.05859	0.252604	0.306529	-0.30633	-0.00666
KIF9	-0.19229	0.477001	-0.07047	-0.17774	-0.0875	-0.20056	0.182458	0.184252
KIFAP3	0.24112	-0.45133	-0.14191	0.006832	-0.21181	0.455273	0.288973	-0.05569
KIFC1	0.157262	0.189423	-0.32662	0.378062	0.261907	0.05966	0.572438	0.348469
KIFC2	-0.14472	0.279658	0.148328	0.184024	0.219001	-0.05248	0.293929	0.253959
KIFC3	-0.29082	-0.21619	0.020832	-0.09321	0.12271	0.472866	-0.11451	-0.1423
KIN	0.183398	0.743568	-0.03991	-0.37559	0.082312	-0.15692	0.703223	0.537312
KIR2DL1	-0.19249	0.503185	NA	NA	NA	NA	-0.21715	0.251217
KIR2DL2	0.023829	0.146678	0.160761	0.073453	0.03166	-0.14702	NA	NA
KIR2DL3	-0.17953	0.149689	NA	NA	NA	NA	-0.57998	0.323213
KIR2DL4	-0.10598	0.208967	0.368131	0.055769	-0.19457	-0.18756	0.357573	0.721566
KIR2DL5A	0.165545	0.218668	-0.06881	0.016491	-0.16177	0.147888	-0.15887	0.374421
KIR2DL5B	-0.45328	0.521706	NA	NA	NA	NA	-0.43501	-0.17457
KIR2DS1	NA	NA	0.290594	0.103188	0.211412	0.112763	0.141801	0.417122
KIR2DS2	0.608824	0.202503	0.293857	0.195137	-0.21011	-0.06928	NA	NA
KIR2DS3	0.276943	0.631431	NA	NA	NA	NA	-0.01253	0.462098
KIR2DS4	0.243742	-0.07609	0.045194	-0.02425	0.017383	0.044015	0.118837	-0.18959
KIR2DS5	0.062095	-0.19856	NA	NA	NA	NA	-0.45638	0.327647
KIR3DL1	-0.03738	0.745735	0.144068	0.196686	-0.30202	-0.15988	-0.32762	0.455032
KIR3DL2	-0.47266	0.051071	0.032595	0.04547	-0.13777	0.156692	0.06434	0.538384
KIR3DL3	0.25012	0.539012	0.165127	-0.22961	-0.07247	0.22529	-0.35749	0.083872
KIR3DX1	-0.17053	0.420339	0.028419	-0.04388	0.131518	-0.12666	NA	NA
KIRREL	0.230334	-0.24338	0.221218	-0.03004	-0.14468	0.296956	-0.57099	-0.40359
KIRREL2	0.42745	0.711096	-0.20166	0.711803	-0.02234	0.032005	0.298607	-0.13692

KIRREL3	0.026834	0.127006	-0.36718	-0.01395	-0.2918	-0.30398	-0.39267	-0.32876
KISS1	-0.02157	0.13828	-0.30453	-0.03838	-0.26791	0.293728	0.446472	0.710931
KISS1R	0.097584	0.337629	-0.06759	0.011439	-0.18529	0.249726	0.154084	0.36954
KIT	-0.49237	-0.51376	-0.08169	0.095083	-0.25597	0.23912	-0.16404	-0.04866
KITLG	-0.30774	-0.43338	-0.46447	-0.10376	-0.25218	-0.36444	-0.10166	-0.15055
KL	-0.01767	-0.38303	0.212509	0.114088	-0.08484	-0.20545	-0.23408	0.122069
KLB	-0.04868	0.344211	0.000248	0.039547	-0.13271	-0.51037	0.385849	0.29585
KLC1	NA	NA	0.095908	0.030002	0.236544	0.477491	NA	NA
KLC2	0.196718	0.536173	0.219668	-0.0135	0.189555	-0.20755	0.042521	0.161688
KLC3	0.084344	0.282371	0.010629	-0.09237	0.071751	0.396414	0.333695	0.366585
KLC4	0.473852	0.431825	0.202247	-0.00169	0.035225	-0.51121	0.263755	0.002144
KLF1	0.023612	0.722634	0.000576	-0.25902	0.231066	-0.12519	-0.03017	0.323205
KLF10	-0.15022	-0.42394	0.034484	-0.22544	-0.24557	-0.14965	0.320683	-0.04897
KLF11	-0.32977	-0.18189	-0.27495	-0.13984	-0.08358	-0.10099	-0.54217	-0.27257
KLF12	-0.14345	-0.14412	-0.26378	0.09141	-0.24777	0.438749	0.287631	-0.13948
KLF13	-0.21047	-0.21925	0.161808	-0.03846	-0.04844	0.260334	0.069782	0.378481
KLF14	0.588199	0.227285	-0.06065	-0.11861	-0.12148	-0.34214	0.216947	0.109415
KLF15	0.353824	-0.26147	-0.09031	0.134443	-0.02043	-0.08358	-0.13907	-0.55533
KLF16	0.370043	0.266559	0.188855	0.000484	-0.02194	0.287127	-0.45843	-0.39587
KLF17	0.12868	0.506025	-0.02403	-0.08806	-0.0067	-0.06646	-0.29515	-0.02622
KLF2	-0.51298	-0.07212	-0.0677	0.231838	-0.12326	0.016093	-0.56249	-0.39971
KLF3	0.340078	0.312807	-0.26985	0.122434	0.009183	-0.21199	0.114915	0.135855
KLF4	-0.39131	-0.4057	-0.15356	-0.09163	-0.10181	-0.09633	-0.04488	-0.0906
KLF5	0.09695	-0.13315	-0.12826	-0.02679	0.252989	0.331614	0.047868	-0.35185
KLF6	-0.485	-0.41653	0.2713	-0.02947	0.029646	0.076178	-0.15594	-0.12904
KLF7	-0.03108	0.103584	-0.187	0.031236	0.004207	-0.03209	-0.01723	-0.52106
KLF8	0.187481	0.014928	-0.30034	-0.03212	-0.18786	0.191159	-0.27731	0.109186
KLF9	-0.30285	-0.47192	-0.13807	-0.30783	0.049905	-0.13053	-0.13195	-0.13813
KLHDC1	-0.3036	-0.5294	0.125481	-0.257	-0.33754	-0.0035	-0.25948	-0.096
KLHDC10	NA	NA	-0.07738	-0.12575	-0.1739	-0.00156	NA	NA
KLHDC2	0.112689	-0.18938	-0.2029	-0.21768	-0.24203	-0.15233	-0.40021	-0.37398
KLHDC3	0.203407	0.270374	0.044878	0.114355	0.019655	-0.30465	-0.43173	-0.50321
KLHDC4	0.217843	-0.00315	0.084221	0.347176	0.311659	0.12711	-0.07125	-0.05481
KLHDC5	0.040135	-0.21111	0.064851	-0.50879	0.321228	0.137238	-0.26566	-0.33091
KLHDC6	0.177822	-0.0062	-0.07004	-0.21802	0.083534	0.097975	-0.05915	-0.21491
KLHDC7A	0.096372	0.229069	0.259487	-0.29167	-0.13154	0.11324	0.594267	0.514128
KLHDC7B	0.466532	0.669863	-0.01944	0.432697	-0.39575	0.443435	-0.28583	-0.26535
KLHDC8A	0.027089	0.103304	-0.27561	-0.12169	-0.00488	0.110228	0.077992	0.19908
KLHDC8B	-0.13244	-0.14388	0.466542	-0.10236	0.042352	0.133243	-0.15111	-0.22554
KLHDC9	NA	NA	-0.14001	-0.08178	-0.22547	-0.31848	NA	NA
KLHL1	0.034275	0.17376	0.173527	0.002395	-0.03572	-0.18025	0.376139	0.2017
KLHL10	0.10655	0.027684	-0.24647	-0.16011	0.087629	-0.51061	-0.08147	0.065462
KLHL11	0.511136	0.34675	-0.11169	0.082979	-0.4678	-0.1863	0.630296	0.312074
KLHL12	0.000586	-0.4141	-0.1999	-0.1162	0.048864	-0.50143	0.395925	0.015947
KLHL13	0.06103	-0.24774	0.038348	-0.41464	-0.29951	-0.30276	-0.23181	-0.17753
KLHL14	-0.23933	0.414786	-0.25877	0.691044	0.147884	0.391863	0.344901	0.179734
KLHL15	0.335165	0.548278	0.004574	-0.12234	0.109845	-0.0555	-0.23371	-0.14563
KLHL17	0.326034	0.000547	0.305507	0.075553	0.274268	-0.0793	0.07708	0.391917
KLHL18	-0.01502	-0.44013	-0.15461	-0.15289	-0.096	-0.10277	-0.44673	0.15353
KLHL2	-0.60841	-0.68885	-0.02468	-0.259	0.01349	-0.19266	-0.46492	-0.20506
KLHL20	-0.29109	0.034923	0.029168	-0.29983	0.000156	0.300006	0.219263	-0.00387
KLHL21	-0.45226	-0.16998	-0.01767	-0.11194	0.265457	-0.04269	0.003639	-0.1318

KLHL22	-0.33978	0.116853	0.123535	0.138661	0.342443	0.100634	-0.33868	-0.26862
KLHL23	0.055147	-0.19024	0.013797	-0.55806	0.158155	-0.038	0.254943	-0.09669
KLHL24	0.107374	-0.68123	-0.03431	-0.07501	-0.02026	-0.11887	NA	NA
KLHL25	-0.298	0.122975	-0.18567	0.111951	0.323237	0.018774	0.393709	-0.15371
KLHL26	0.121529	-0.31896	-0.06141	0.119289	0.359411	-0.05154	0.031106	0.051063
KLHL28	-0.39195	-0.42106	-0.06697	-0.2379	-0.23977	0.285201	NA	NA
KLHL29	NA	NA	-0.02482	0.052675	0.516361	0.521819	NA	NA
KLHL3	0.532012	-0.27539	-0.11596	0.297394	-0.31586	0.135253	-0.19896	0.210491
KLHL30	-0.15723	-0.25379	0.293279	-0.22168	-0.25411	-0.00174	NA	NA
KLHL31	0.047666	0.519974	NA	NA	NA	NA	NA	NA
KLHL32	0.212868	-0.35921	-0.24917	0.077903	0.258084	-0.09987	NA	NA
KLHL33	NA	NA	0.126688	-0.37246	-0.06925	0.205416	NA	NA
KLHL34	0.241952	-0.16953	0.073148	0.044995	0.174432	0.057974	NA	NA
KLHL35	NA	NA	0.013099	0.134028	0.174612	0.022288	NA	NA
KLHL36	NA	NA	0.189962	-0.0367	0.319739	0.241156	NA	NA
KLHL4	-0.01564	0.093113	0.204205	0.074875	-0.355	-0.23619	-0.06043	0.067891
KLHL5	0.001699	-0.12377	-0.11595	0.231602	-0.13257	0.201595	-0.20619	-0.14416
KLHL6	-0.42395	-0.17848	0.157657	0.533464	-0.34277	0.42756	NA	NA
KLHL7	0.226586	-0.47586	-0.06251	-0.60268	-0.1056	-0.30266	0.076505	0.235126
KLHL8	-0.21652	-0.59136	-0.00819	-0.29457	-0.23362	-0.25986	-0.22745	-0.15025
KLHL9	0.108931	-0.63794	-0.23719	-0.4745	0.129051	0.127991	-0.12887	-0.21113
KLK1	-0.12527	0.718589	-0.16154	-0.03875	-0.01287	-0.31489	-0.24607	-0.3211
KLK10	0.051069	0.765413	-0.00982	-0.12756	-0.10892	-0.01513	0.250028	0.409237
KLK11	-0.22477	0.498626	-0.00541	0.334042	-0.18089	-0.20829	0.305217	0.197339
KLK12	-0.07419	0.268973	-0.14537	0.291275	-0.03675	-0.32163	0.27726	0.487625
KLK13	-0.04125	0.625482	-0.28068	-0.22424	-0.05108	0.099068	-0.22724	0.09523
KLK14	0.41605	0.375173	0.083401	0.010587	0.121735	-0.15885	0.370807	0.051938
KLK15	0.035267	0.183367	-0.07016	-0.28875	-0.16775	-0.50137	0.023323	-0.20463
KLK2	-0.0436	0.545935	0.013552	-0.20614	-0.23858	-0.12397	-0.51144	0.121947
KLK3	-0.04873	-0.06748	-0.07536	-0.0167	-0.15983	-0.0794	-0.0074	-0.47739
KLK4	-0.04667	-0.06402	0.092325	0.054289	-0.07343	-0.31593	0.262018	-0.09117
KLK5	-0.0193	0.756183	-0.16747	-0.12197	0.011296	0.104927	-0.10832	-0.33117
KLK6	0.014582	-0.01801	0.214889	-0.28999	0.395609	0.137042	0.226537	0.13691
KLK7	-0.15646	0.043448	0.117431	-0.15963	0.037658	0.100389	0.07854	0.510552
KLK8	-0.02025	0.132401	0.067557	-0.16669	0.074741	-0.12557	-0.25139	-0.16455
KLK9	0.194978	0.439428	0.277463	-0.40627	-0.00053	-0.36328	-0.14416	-0.02739
KLKB1	-0.39352	0.351826	-0.03529	-0.2312	-0.11294	-0.38058	-0.27286	0.40201
KLKP1	NA	NA	NA	NA	NA	NA	-0.0063	-0.36195
KLRA1	-0.01862	-0.43286	-0.25293	-0.18178	-0.31408	-0.12788	0.149399	0.524501
KLRB1	-0.08997	-0.13873	0.251792	0.355338	-0.12392	0.335822	-0.32944	0.20042
KLRC1	0.08531	0.234386	0.356108	-0.18901	-0.40652	-0.05821	-0.20922	0.052642
KLRC2	0.343017	0.383253	0.199071	-0.19572	-0.22055	-0.08117	-0.35435	0.099665
KLRC3	0.282455	0.166412	0.189174	-0.00767	-0.42701	-0.25288	-0.1849	0.45099
KLRC4	0.123717	0.017443	0.334682	-0.06614	-0.35578	0.093571	0.147605	0.085752
KLRD1	-0.14029	0.114801	0.115221	-0.34818	-0.37042	-0.22014	-0.4784	0.473677
KLRF1	-0.15344	-0.00408	-0.03456	0.138439	-0.23645	0.485146	-0.46667	-0.02237
KLRG1	0.204094	0.273983	-0.04184	0.565709	-0.29797	0.549504	0.111853	-0.05963
KLRG2	NA	NA	-0.2062	0.08774	0.0223	-0.23906	0.081264	-0.11193
KLRK1	-0.36985	-0.19679	0.208802	0.10612	-0.21247	0.110752	0.289112	0.479992
KMO	-0.3651	0.036637	0.225139	0.447111	-0.46871	0.516233	-0.53703	-0.11104
KNCN	NA	NA	0.089788	-0.57924	0.354608	0.216158	NA	NA
KNDC1	0.145384	0.622616	0.027722	-0.04623	0.121728	0.301089	-0.33383	-0.36448

KNG1	-0.12448	0.210051	0.150579	-0.18644	-0.12986	-0.27772	-0.03305	-0.09416
KNTC1	-0.10808	-0.0116	-0.16255	0.239361	0.10216	0.190171	0.340483	0.183779
KPNA1	-0.30755	-0.25866	-0.3859	0.061179	-0.25608	-0.0364	NA	NA
KPNA2	-0.18098	0.010133	-0.11304	0.221963	-0.11155	0.126108	-0.26059	-0.21537
KPNA3	0.357697	-0.4515	0.03709	-0.34113	0.013979	-0.07572	0.060847	-0.32811
KPNA4	0.207849	-0.27716	-0.3208	-0.31304	-0.16981	0.022676	-0.1265	-0.11709
KPNA5	-0.18642	-0.44417	-0.07799	-0.11797	0.08644	0.145734	-0.04022	-0.16724
KPNA6	0.115542	0.727157	0.11865	0.120554	0.37114	0.125708	0.48961	0.284205
KPNB1	0.079023	-0.61558	-1E-04	0.423446	0.260535	0.31533	-0.25728	-0.22246
KPRP	-0.32066	0.168037	NA	NA	NA	NA	NA	NA
KPTN	0.409497	0.293554	0.028244	0.236419	-0.04424	-0.06311	-0.10075	-0.17008
KRAS	0.07633	-0.01131	-0.45982	-0.19586	-0.2635	-0.14632	0.367021	0.204489
KRBA1	-0.33182	0.250433	0.077255	0.098801	0.061946	0.430359	NA	NA
KRBA2	-0.30995	-0.34237	NA	NA	NA	NA	NA	NA
KRCC1	-0.27898	-0.1773	-0.38622	-0.06784	-0.09666	-0.17669	-0.07327	-0.00347
KREMEN1	-0.04523	0.678277	0.331374	-0.11907	0.269928	0.351648	0.149785	-0.48911
KREMEN2	-0.24103	0.505185	-0.29847	0.429834	0.22061	-0.35414	0.238727	0.048338
KRI1	NA	NA	0.118221	0.239123	0.249813	0.3233	NA	NA
KRIT1	0.199809	-0.41108	-0.10874	-0.03711	0.098145	0.096595	0.498087	0.317048
KRR1	0.207804	-0.01628	-0.11562	-0.16682	-0.02345	-0.0712	NA	NA
KRT1	-0.06382	0.150629	-0.04052	0.045609	-0.00445	0.165013	0.020717	0.181785
KRT10	0.285528	-0.38256	0.241573	-0.02895	-0.07418	0.175657	0.587391	0.313113
KRT12	0.151894	-0.08176	-0.3172	-0.02702	0.066307	-0.20307	0.068961	-0.058
KRT13	-0.02718	0.345319	-0.07353	-0.01634	0.019301	-0.28947	-0.13912	-0.13141
KRT14	-0.03177	0.165961	-0.1419	-0.17968	-0.27126	-0.25343	-0.07695	0.371687
KRT15	0.215905	0.314229	0.179078	0.078206	0.04516	-0.22509	0.133362	0.358687
KRT16	-0.13981	0.026332	-0.06479	-0.26865	-0.18196	0.117045	-0.01283	-0.28651
KRT17	0.077202	0.100442	-0.08315	-0.05041	-0.31029	0.13958	0.166919	-0.11474
KRT18	-0.27901	-0.11399	0.009073	0.072204	0.277786	0.077157	0.054051	-0.26208
KRT19	0.244024	0.054959	0.002765	0.123243	-0.13213	-0.06743	0.373958	0.081512
KRT2	0.049028	0.228564	-0.02209	0.252593	-0.01248	0.118832	NA	NA
KRT20	0.276414	-0.06268	-0.03094	-0.05333	-0.27865	-0.20244	-0.29331	-0.36568
KRT23	0.33851	0.375832	0.297485	-0.16655	0.268992	-0.60895	0.320007	-0.46807
KRT24	-0.02739	-0.1872	-0.21261	0.038622	-0.29479	-0.19559	-0.17621	-0.38469
KRT25	-0.06281	-0.13094	-0.09464	0.008777	-0.04664	-0.21428	NA	NA
KRT26	0.017796	0.608082	0.260824	0.078505	-0.16388	-0.13923	-0.07023	-0.08075
KRT27	-0.10034	-0.38538	0.01842	0.109024	-0.0231	0.116466	NA	NA
KRT28	-0.0158	0.369564	-0.09971	0.194693	-0.1082	-0.4844	NA	NA
KRT3	0.387844	0.731048	0.034482	-0.14691	-0.02719	0.03297	-0.15321	-0.14777
KRT31	0.177714	0.727652	0.324483	-0.1695	-0.11513	-0.12292	NA	NA
KRT32	-0.24464	0.455665	0.146222	-0.18766	0.179924	-0.32861	NA	NA
KRT33A	0.408347	0.743928	0.262443	-0.1075	-0.12013	-0.03345	-0.30861	-0.19013
KRT33B	0.073883	-0.00882	0.219868	-0.00648	-0.01023	-0.12308	NA	NA
KRT34	-0.40226	0.110764	-0.17362	-0.0591	-0.11365	0.199246	NA	NA
KRT35	0.016886	0.170564	-0.15868	0.097266	-0.18559	-0.09863	NA	NA
KRT36	0.303725	0.756965	0.117711	-0.02674	-0.04289	-0.30857	NA	NA
KRT37	-0.01749	0.086309	0.146855	0.262657	-0.07543	0.061548	NA	NA
KRT38	0.264605	0.084257	-0.05575	-0.06589	0.280335	-0.18473	NA	NA
KRT39	0.236702	0.533674	NA	NA	NA	NA	-0.02044	-0.05556
KRT4	-0.01898	0.733032	0.107762	-0.32492	-0.1899	-0.01926	-0.28061	-0.12498
KRT40	NA	NA	0.275794	-0.04738	0.179163	-0.06498	-0.21818	-0.20133
KRT5	-0.04717	-0.10555	0.031	0.484015	-0.20058	0.393523	-0.05251	-0.34187

KRT6A	NA	NA	-0.49278	0.334872	0.003673	-0.02253	0.180744	0.066561
KRT6B	0.021029	0.767931	-0.54558	0.239531	0.035929	-0.1756	-0.05335	-0.06886
KRT6C	-0.13643	-0.32241	-0.40236	0.274344	-0.1045	-0.01827	NA	NA
KRT7	-0.20161	0.216862	0.205325	-0.1357	-0.14082	0.25451	-0.08552	0.198868
KRT71	0.461277	0.089921	0.073411	0.042766	-0.12159	0.123315	NA	NA
KRT72	0.322369	0.320925	-0.14947	0.357007	0.09103	0.596674	NA	NA
KRT73	0.140571	0.399844	0.404662	-0.19675	-0.29911	-0.06257	NA	NA
KRT74	0.009418	-0.08018	-0.14461	-0.2843	0.014198	0.053103	NA	NA
KRT75	0.357782	0.301856	0.038862	0.003786	-0.11434	-0.1205	NA	NA
KRT76	0.357664	-0.31014	0.367494	0.017197	0.096868	-0.12918	NA	NA
KRT77	0.000914	0.184782	-0.12943	0.022855	-0.25577	0.004143	NA	NA
KRT78	0.002774	0.303152	-0.04987	0.095716	-0.24263	-0.15801	NA	NA
KRT79	NA	NA	0.009872	0.274448	0.142367	-0.33837	NA	NA
KRT8	-0.18407	0.420387	0.139337	0.289462	0.111511	-0.12309	0.346591	0.052776
KRT80	0.208748	0.372186	-0.12207	-0.07653	0.046888	-0.14469	NA	NA
KRT81	-0.27749	-0.29442	0.193864	0.260065	-0.16685	-0.11151	NA	NA
KRT82	0.160164	0.06401	-0.0383	0.07448	-0.17565	-0.15342	NA	NA
KRT83	0.120965	0.213738	0.082032	-0.04888	-0.18977	-0.08736	NA	NA
KRT84	-0.2746	0.072027	NA	NA	NA	NA	NA	NA
KRT85	0.05298	0.599996	-0.04975	-0.10666	0.117008	-0.23026	NA	NA
KRT86	NA	NA	0.012024	0.225269	-0.27258	0.317954	0.007161	-0.04533
KRT9	-0.04378	0.37273	0.108453	-0.19536	-0.27058	-0.39381	-0.43711	-0.24511
KRTAP1-1	0.477613	0.671899	0.106541	-0.00636	0.024152	-0.16183	-0.38204	-0.35662
KRTAP1-3	NA	NA	-0.03062	-0.14239	0.064075	0.260992	0.071078	-0.11608
KRTAP1-5	-0.43779	0.558236	0.122221	-0.23984	-0.18394	0.293366	NA	NA
KRTAP10-1	0.123961	-0.30551	NA	NA	NA	NA	-0.25299	-0.26582
KRTAP10-1(	0.264621	0.686465	0.085848	0.018027	0.173152	0.25227	-0.10241	-0.12235
KRTAP10-1	0.274371	0.68641	NA	NA	NA	NA	-0.44202	-0.55347
KRTAP10-1:	0.060115	0.355054	NA	NA	NA	NA	0.18895	0.391331
KRTAP10-2	0.179928	-0.12129	NA	NA	NA	NA	0.046123	-0.0526
KRTAP10-3	NA	NA	NA	NA	NA	NA	0.101911	0.187878
KRTAP10-4	0.58781	0.68379	NA	NA	NA	NA	-0.16106	-0.24473
KRTAP10-5	0.342888	0.274999	NA	NA	NA	NA	-0.21867	-0.26903
KRTAP10-7	0.235251	0.551742	NA	NA	NA	NA	0.365996	0.25394
KRTAP10-8	0.313228	0.463554	NA	NA	NA	NA	0.281546	0.120747
KRTAP10-9	0.26254	-0.16362	NA	NA	NA	NA	0.572457	0.467087
KRTAP11-1	0.055164	0.09691	-0.05233	-0.01642	0.345909	-0.07262	0.108467	0.065022
KRTAP12-1	0.034271	0.362684	0.11461	-0.11125	-0.09089	-0.36981	0.427686	0.396323
KRTAP12-2	0.242129	0.516732	-0.14879	-0.01763	-0.14459	0.097837	0.464933	0.51319
KRTAP12-3	-0.09108	0.272828	NA	NA	NA	NA	0.29883	0.256234
KRTAP12-4	-0.23541	0.268314	NA	NA	NA	NA	-0.10066	0.123597
KRTAP13-1	0.228019	0.322899	0.115075	-0.18399	-0.10073	-0.17268	0.619824	0.129386
KRTAP13-2	0.101255	-0.36308	0.106495	-0.19122	-0.16723	-0.17304	-0.02399	-0.07622
KRTAP13-3	0.028038	0.010986	0.285224	-0.13531	-0.16463	-1.4E-06	0.229778	0.292175
KRTAP13-4	0.088473	0.027375	-0.01248	-0.27128	-0.01533	-0.29176	0.412203	0.496478
KRTAP15-1	-0.21464	0.259995	0.138617	-0.19581	0.031823	-0.07934	-0.13729	0.130663
KRTAP17-1	0.117141	0.447419	-0.17004	0.063661	-0.35817	-0.40567	0.196496	0.152244
KRTAP19-1	0.08081	-0.05764	0.149053	0.025275	0.11558	-0.17714	-0.41789	-0.36284
KRTAP19-2	0.109335	-0.03885	NA	NA	NA	NA	0.2545	0.11492
KRTAP19-3	0.206663	0.584547	NA	NA	NA	NA	-0.15037	-0.25387
KRTAP19-4	0.071748	0.428256	NA	NA	NA	NA	-0.31479	-0.29849
KRTAP19-5	0.044875	0.331783	NA	NA	NA	NA	-0.39445	-0.20549

KRTAP19-6	-0.16274	0.528132	NA	NA	NA	NA	-0.45655	-0.43954
KRTAP19-7	0.332596	0.280686	NA	NA	NA	NA	0.044861	-0.00222
KRTAP2-1	NA	NA	0.198552	-0.29902	0.021143	-0.19023	NA	NA
KRTAP2-4	NA	NA	0.014969	-0.05438	-0.10325	0.150858	NA	NA
KRTAP20-1	0.354034	0.415171	NA	NA	NA	NA	-0.02676	0.336371
KRTAP20-2	-0.34071	-0.38374	NA	NA	NA	NA	-0.52106	0.007391
KRTAP21-1	0.013937	-0.11091	NA	NA	NA	NA	0.475356	0.333175
KRTAP21-2	0.024689	0.473987	NA	NA	NA	NA	0.459997	0.267454
KRTAP22-1	0.155533	0.539611	NA	NA	NA	NA	-0.60922	0.012808
KRTAP23-1	0.523627	0.518982	NA	NA	NA	NA	0.170947	-0.1016
KRTAP26-1	-0.06967	0.185826	NA	NA	NA	NA	-0.07133	-0.0969
KRTAP27-1	NA	NA	NA	NA	NA	NA	0.246513	0.199153
KRTAP3-1	-0.32328	0.045157	0.262767	-0.14887	-0.17656	0.27716	0.115077	0.270775
KRTAP3-2	NA	NA	0.132955	-0.05885	0.351482	0.209941	-0.01415	0.172871
KRTAP3-3	0.479106	0.651841	-0.00711	-0.15827	-0.07065	-0.23622	NA	NA
KRTAP4-1	NA	NA	0.185527	-0.12691	0.150788	0.173724	NA	NA
KRTAP4-11	NA	NA	0.03966	-0.03601	-0.0058	-0.26208	NA	NA
KRTAP4-12	-0.12514	0.07656	0.073353	0.24115	-0.1514	-0.20332	-0.24927	0.236656
KRTAP4-2	-0.03337	0.265647	-0.08008	0.098855	-0.23891	0.066311	0.31338	0.211546
KRTAP4-3	NA	NA	0.134213	-0.15251	-0.12943	0.055008	NA	NA
KRTAP4-4	0.178414	0.077832	0.022782	0.065289	-0.10562	0.035192	-0.21178	0.328577
KRTAP4-5	-0.29699	0.022446	0.098834	-0.19267	0.147904	0.016428	0.286117	-0.2515
KRTAP4-7	0.293991	0.350148	-0.11336	-0.0227	-0.15255	-0.0314	-0.61427	-0.38991
KRTAP5-1	-0.09782	0.238276	NA	NA	NA	NA	-0.25386	-0.37375
KRTAP5-10	-0.22175	0.215516	NA	NA	NA	NA	-0.24064	-0.29248
KRTAP5-11	-0.13583	-0.25015	NA	NA	NA	NA	-0.59096	0.1009
KRTAP5-2	0.245702	0.333061	NA	NA	NA	NA	-0.2257	0.073124
KRTAP5-3	0.29649	0.008448	NA	NA	NA	NA	0.152218	0.13629
KRTAP5-4	0.149342	0.011452	NA	NA	NA	NA	-0.49063	-0.05338
KRTAP5-5	NA	NA	NA	NA	NA	NA	0.126889	0.217649
KRTAP5-6	-0.16475	0.129814	NA	NA	NA	NA	0.108336	0.196262
KRTAP5-7	0.122327	0.691834	NA	NA	NA	NA	0.515966	-0.01159
KRTAP5-8	NA	NA	0.001233	-0.00256	0.195116	0.041772	0.508166	0.152222
KRTAP5-9	0.269776	-0.20724	0.058364	-0.00176	0.208664	-0.02288	-0.23802	-0.18007
KRTAP6-1	0.278991	0.606542	NA	NA	NA	NA	-0.3625	-0.14048
KRTAP6-2	0.074398	0.375432	NA	NA	NA	NA	-0.01468	-0.0411
KRTAP6-3	0.01458	0.279556	0.137746	-0.24452	0.008217	-0.24319	0.398103	0.240834
KRTAP7-1	NA	NA	0.07765	-0.24987	0.007023	-0.18054	NA	NA
KRTAP8-1	0.025401	-0.16336	-0.02942	-0.09439	0.171649	-0.29336	-0.09438	0.020637
KRTAP9-2	0.02987	0.612149	0.181336	-0.239	-0.29244	0.032805	NA	NA
KRTAP9-3	-0.07533	0.695469	-0.16596	-0.08604	0.126915	-0.38507	-0.22897	0.100153
KRTAP9-4	0.00189	0.594818	0.365231	-0.0648	0.093279	0.132065	0.081612	-0.00252
KRTAP9-8	NA	NA	0.215928	0.280051	0.096751	-0.01041	NA	NA
KRTAP9-9	NA	NA	0.016868	-0.04617	-0.17147	0.115668	NA	NA
KRTCAP2	0.051953	0.732051	0.212963	0.125083	-0.17669	-0.12745	0.090078	-0.10178
KRTCAP3	0.201804	0.238608	0.0433	0.343253	0.074913	-0.09339	0.054937	-0.33462
KRTDAP	NA	NA	-0.43107	-0.06358	-0.2363	-0.07267	NA	NA
KSR1	-0.06896	-0.68964	-0.07961	0.110285	0.267571	0.219895	0.111175	0.304294
KSR2	-0.01009	0.208877	-0.11098	0.086134	-0.22125	0.206718	0.10356	-0.00869
KTELC1	-0.1158	-0.09648	-0.45608	0.161798	0.024585	0.106468	NA	NA
KTI12	0.309875	0.366012	-0.23804	0.29272	0.153194	-0.29061	0.219317	0.118013
KTN1	0.126236	-0.24933	-0.13427	-0.21092	-0.13221	0.107458	0.02566	0.181904

KY	0.178287	0.200033	-0.04825	-0.22115	-0.15604	-0.11229	-0.50496	-0.61821
KYNU	-0.33165	-0.16531	0.008654	0.230375	-0.19745	0.505895	-0.43493	0.15876
L1CAM	0.321139	-0.3556	0.02489	-0.05327	0.262953	0.183933	0.160587	-0.53846
L1TD1	-0.40087	0.769014	-0.17882	0.066321	-0.01116	-0.09982	NA	NA
L2HGDH	-0.06394	0.576539	-0.2493	-0.04761	-0.11747	0.108245	NA	NA
L3MBTL	0.40365	-0.36166	-0.03185	0.073125	0.263254	0.298448	0.412855	-0.1483
L3MBTL2	0.150305	0.535168	0.267303	0.015935	0.00416	0.252288	0.562737	0.536847
L3MBTL3	0.403098	0.386964	-0.11756	0.08442	-0.22315	0.122713	-0.11686	0.086265
L3MBTL4	0.226832	-0.09919	0.479255	0.163766	0.185066	-0.23912	-0.02906	0.423466
LACE1	0.452003	0.420405	-0.08302	-0.27043	-0.23757	-0.45476	-0.00363	0.077587
LACRT	0.186224	0.633801	0.335039	-0.17675	-0.01893	0.087582	-0.2778	-0.32568
LACTB	-0.19062	-0.20994	-0.28331	0.016398	-0.27777	-0.38662	-0.18274	0.071465
LACTB2	0.043513	0.127991	0.104701	-0.11897	-0.12184	-0.41644	0.067379	0.058218
LAD1	0.112702	0.213596	-0.27505	0.319051	-0.08573	0.198968	0.423118	-0.00964
LAG3	-0.0165	0.51427	0.17852	0.246849	-0.35207	0.431027	-0.44666	0.182055
LAGE3	0.218758	-0.07498	-0.1542	0.395298	0.252134	0.089582	NA	NA
LAIR1	-0.46848	0.323849	0.462107	0.256525	-0.43082	0.387494	0.523615	0.613642
LAIR2	0.067835	-0.12974	0.278635	0.374177	-0.12533	-0.41337	-0.33878	0.112199
LALBA	0.281899	0.609582	0.000785	0.274378	0.120796	0.004009	-0.24837	-0.33185
LAMA1	-0.33659	-0.15792	-0.27929	-0.13129	-0.27151	-0.34986	-0.26324	0.051582
LAMA2	-0.38645	-0.4198	0.02565	-0.29812	-0.45119	-0.44116	-0.00345	-0.05957
LAMA3	-0.04408	0.198362	-0.15414	-0.06105	-0.21252	0.267609	0.235359	0.335733
LAMA4	-0.42673	-0.278	0.090235	-0.15766	-0.02887	0.076498	-0.66117	-0.60782
LAMA5	-0.04242	-0.27791	0.103284	-0.10341	-0.07868	0.31964	0.064635	-0.32998
LAMB1	-0.17045	-0.49274	-0.25468	-0.04214	0.000371	0.011288	-0.55942	-0.31636
LAMB2	-0.16848	-0.08019	0.156992	0.026161	0.135173	0.412101	-0.33993	-0.43542
LAMB3	-0.11098	0.052829	-0.13049	-0.11882	0.145295	0.145378	0.520825	0.248165
LAMB4	0.049823	0.360718	-0.20219	-0.15422	0.311808	-0.07146	0.512951	0.531624
LAMC1	-0.26183	-0.13967	0.045734	-0.14895	-0.18803	0.395558	-0.27071	-0.14494
LAMC2	-0.4689	0.254015	-0.21479	0.12195	-0.14781	0.09908	0.16441	0.039082
LAMC3	-0.07831	0.51256	0.09749	-0.01969	0.083761	0.123493	-0.22634	-0.30789
LAMP1	0.006631	0.240326	0.252013	0.168076	0.539156	0.147443	0.214827	0.106194
LAMP2	0.163198	-0.36268	-0.05135	-0.27066	0.108724	-0.24994	-0.03732	-0.01957
LAMP3	-0.16741	0.33334	0.171551	0.46545	-0.16873	0.681304	-0.36365	0.235115
LANCL1	-0.00968	-0.53729	0.075268	-0.24432	-0.16303	0.199738	-0.50351	-0.38945
LANCL2	0.301628	0.138539	-0.03318	0.250012	0.059056	-0.06649	-0.19065	0.021134
LANCL3	0.238969	0.201591	-0.15453	-0.3255	-0.45424	0.160442	-0.29765	-0.13499
LAP3	0.039793	-0.42806	0.180689	-0.14469	-0.22261	-0.02777	-0.46706	-0.31589
LAPTM4A	-0.01697	-0.4524	0.056822	-0.31716	-0.04512	-0.24784	-0.60941	-0.6102
LAPTM4B	0.169644	-0.44806	0.112931	-0.08607	0.190332	0.147242	0.420318	0.576578
LAPTM5	-0.5444	-0.11728	0.223909	0.492419	-0.03949	0.57461	-0.30612	0.289769
LARGE	-0.03225	0.29329	0.129119	0.099324	-0.04788	-0.26323	-0.46603	-0.16105
LARP1	0.492781	-0.19955	0.343724	0.333241	0.31489	0.256598	0.453732	0.315561
LARP2	-0.26827	0.065904	-0.18954	-0.01766	0.181585	-0.16866	0.060576	0.006121
LARP4	0.2	0.695603	-0.23228	-0.1784	-0.12423	0.103239	-0.45198	-0.20533
LARP5	-0.22408	-0.13248	0.2043	0.061599	0.294957	0.342796	0.080521	0.097708
LARP6	0.236535	-0.34881	-0.02485	-0.00198	0.114629	-0.01592	-0.09148	-0.08888
LARP7	-0.19771	-0.04914	-0.19725	-0.01405	-0.09018	0.060752	0.477209	0.487383
LARS	-0.24731	-0.52206	0.164615	-0.20315	0.006441	-0.07194	0.427995	0.252786
LARS2	0.503027	-0.53613	-0.17879	0.480275	0.287261	0.167092	0.530149	0.267389
LAS1L	0.536174	0.470863	-0.03218	0.279614	0.271606	0.383595	-0.09871	-0.3643
LASP1	-0.15488	0.092625	0.269871	0.214495	0.242538	0.157537	0.114618	0.171664

LASS1	0.571795	0.618153	NA	NA	NA	NA	-0.24169	-0.10554
LASS2	-0.21761	-0.06903	0.338265	0.14127	0.326292	0.137107	0.3445	0.058537
LASS3	-0.17091	-0.14792	0.085423	-0.0569	-0.13058	0.274612	0.342585	0.155679
LASS4	-0.14521	0.320175	0.333855	0.618109	0.148043	0.57211	-0.0814	0.396118
LASS5	0.118083	0.328574	0.29435	-0.05506	0.141408	0.228069	0.320299	0.32967
LASS6	-0.17948	0.053789	0.265066	-0.09963	0.132895	-0.17364	0.532186	0.340283
LAT	-0.03887	0.177972	-0.03762	0.653572	-0.18482	0.58559	-0.23325	0.377736
LAT2	-0.18005	0.586397	0.397339	0.668593	-0.22889	0.403445	-0.43958	0.188827
LATS1	-0.22737	-0.27879	-0.01908	-0.2742	-0.085	-0.01987	-0.38416	-0.0524
LATS2	-0.02626	-0.26801	0.043211	-0.18621	-0.01091	0.264288	-0.11155	-0.0747
LAX1	-0.53595	0.052279	0.255422	0.461511	-0.12004	0.425571	-0.30576	0.04333
LAYN	-0.33012	-0.01966	0.297438	0.07204	0.028167	0.236818	-0.24485	-0.14265
LBH	0.041257	0.370316	0.047861	0.128256	-0.24137	0.412975	-0.23287	-0.02528
LBP	0.173745	-0.2418	0.194846	0.05969	0.389741	0.068022	-0.42996	0.084966
LBR	0.057066	-0.36242	-0.27795	0.024899	0.246241	0.17156	0.479497	0.265239
LBX1	0.384708	0.742014	0.114723	-0.01063	-0.08419	0.248326	-0.09883	-0.44021
LBX2	-0.04149	-0.09737	0.204981	-0.16394	-0.09388	-0.21633	0.345522	0.586071
LBXCOR1	0.183872	0.119399	NA	NA	NA	NA	-0.12763	0.223808
LCA5	NA	NA	-0.14968	-0.09647	-0.17918	-0.01209	NA	NA
LCA5L	NA	NA	-0.14683	-0.25201	-0.29628	-0.31275	NA	NA
LCAT	-0.22522	0.146204	0.196961	-0.01592	0.073634	0.480468	-0.38198	-0.19335
LCE1A	0.375667	0.759202	0.248805	0.044054	0.081837	-0.00188	-0.04875	0.534697
LCE1B	-0.02651	0.642044	-0.03258	0.199658	-0.15054	0.041332	0.162065	-0.15664
LCE1C	-0.0643	0.736674	-0.00653	-0.02191	-0.1739	0.204977	NA	NA
LCE1D	-0.00085	0.361596	-0.03295	-0.03914	-0.18174	0.236506	0.002059	0.162789
LCE1E	0.210528	0.399538	0.092809	-0.20921	0.089651	0.271438	-0.07399	-0.00195
LCE1F	0.050846	0.56428	-0.0041	-0.17169	0.228422	-0.10508	0.021874	0.296569
LCE2A	0.05693	0.573892	0.190012	-0.0129	0.023293	0.050755	0.050055	0.282626
LCE2B	-0.10103	0.072601	0.000331	-0.33556	0.280106	-0.16553	0.403763	0.478957
LCE2C	0.096647	0.558065	0.159952	-0.39158	0.234456	0.041866	-0.3058	0.010218
LCE2D	0.055336	-0.01285	-0.13185	0.186879	-0.12159	0.198721	0.015477	-0.26435
LCE3A	-0.0204	-0.60327	-0.11236	-0.1136	0.138642	0.012267	-0.2807	-0.19827
LCE3B	0.558354	0.708713	0.054773	-0.21441	0.055429	-0.00068	-0.44544	-0.19983
LCE3C	0.08818	0.219223	0.090016	0.132464	-0.05781	-0.04006	0.278977	0.326928
LCE3D	0.002129	0.056753	0.03178	-0.07397	0.387796	-0.06134	-0.35908	-0.17045
LCE3E	0.116978	0.668736	0.291513	-0.00932	-0.08855	-0.10341	0.239528	0.058503
LCE4A	0.039595	0.374864	0.239436	-0.20895	-0.25266	-0.12222	NA	NA
LCE5A	-0.0932	0.217653	0.11891	-0.00847	-0.04725	0.270446	-0.20126	0.184001
LCK	-0.07229	0.439324	0.109474	0.787374	-0.187	0.804483	0.111135	0.202733
LCLAT1	NA	NA	-0.23756	-0.25627	0.365149	-0.0524	NA	NA
LCMT1	0.429282	0.0933	0.161046	-0.10856	0.296079	-0.02171	0.619176	0.013731
LCMT2	0.314401	-0.57339	0.044391	0.027671	-0.00486	0.015555	0.460961	0.528649
LCN1	0.351491	0.249918	0.146939	-0.25987	0.044215	0.091748	-0.07692	0.34482
LCN10	0.008905	0.218616	NA	NA	NA	NA	0.029017	0.285972
LCN12	0.246105	-0.23228	0.085476	0.076926	0.033824	-0.20275	0.340472	-0.17558
LCN2	-0.14551	0.225901	0.277214	0.385682	0.036509	-0.00567	-0.11011	-0.10318
LCN6	-0.32589	-0.32311	0.102871	0.254728	-0.1204	-0.30508	-0.1304	0.118471
LCN8	0.392029	0.307856	-0.09115	-0.16394	0.247717	-0.01611	-0.29382	-1.9E-05
LCN9	0.071094	0.158948	NA	NA	NA	NA	0.328797	0.329543
LCOR	0.029009	-0.19955	-0.06578	-0.26599	0.204338	-0.02206	-0.12289	-0.09685
LCORL	-0.1475	0.365298	-0.39232	-0.02597	0.16647	-0.26458	-0.04396	0.111516
LCP1	-0.54643	-0.03575	-0.15385	0.659758	-0.16258	0.685182	-0.63074	0.201936

LCP2	-0.56781	-0.09125	0.101589	0.349298	-0.0008	0.61145	-0.32359	0.439484
LCT	-0.21782	0.381263	-0.32114	-0.01169	0.117453	0.150098	0.530782	0.515819
LCTL	0.091464	0.05016	NA	NA	NA	NA	-0.01624	-0.29775
LDB1	0.218109	0.689696	0.219415	0.012963	0.205645	0.084868	0.115494	-0.05317
LDB2	-0.43663	-0.30658	0.009498	0.190991	-0.1098	0.168182	-0.14646	0.189566
LDB3	0.215707	-0.23719	-0.14341	-0.11665	0.22231	-0.05746	0.349182	0.408716
LDHA	0.013137	0.049154	-0.13051	0.138973	0.068823	-0.05485	0.084972	0.674944
LDHAL6A	-0.07917	-0.07975	-0.03766	-0.11211	-0.0508	0.173424	-0.11877	0.177742
LDHAL6B	-0.00606	0.516025	0.037636	-0.04196	-0.03906	0.101332	-0.03127	0.291398
LDHB	0.421206	-0.42179	-0.01327	0.157713	-0.15118	0.219056	-0.34395	-0.51293
LDHC	-0.1927	0.289044	-0.15003	0.067123	-0.0781	-0.00461	-0.11095	-0.13781
LDHD	0.031877	0.177455	-0.10586	-0.33255	-0.12931	-0.51396	0.035548	-0.15902
LDLR	0.016776	0.152077	-0.08945	0.021326	0.210073	0.178569	-0.14676	0.117226
LDLRAD1	0.228601	0.284759	-0.02905	-0.13813	0.05859	0.078146	0.311097	0.102222
LDLRAD2	-0.0554	0.112263	NA	NA	NA	NA	0.055586	0.328695
LDLRAD3	0.33567	0.084195	-0.06906	0.297624	0.050094	0.017935	0.437403	-0.04592
LDLRAP1	0.09133	0.513257	0.152046	0.258423	0.346548	-0.01523	0.174844	-0.18225
LDOC1	-0.1948	-0.03263	0.118642	-0.06737	0.170015	-0.23066	0.011157	0.015528
LDOC1L	0.212763	-0.43229	-0.30116	0.002582	0.021508	-0.05558	-0.06396	-0.189
LEAP2	NA	NA	-0.18705	-0.11302	-0.07868	-0.03589	0.071695	0.236041
LECT1	0.038012	0.222353	0.00894	0.152088	-0.0316	0.454894	0.508379	0.483181
LECT2	0.354092	0.343174	0.244352	-0.22028	-0.08506	-0.01928	0.10547	0.030793
LEF1	-0.07216	-0.31864	0.28357	0.71618	-0.13378	0.737259	0.381272	0.289009
LEFTY1	-0.11934	-0.03398	-0.48441	0.181088	-0.15456	-0.15684	0.253589	-0.3539
LEFTY2	0.343208	0.175994	0.255066	-0.04435	-0.15374	-0.13771	-0.04206	-0.12466
LELP1	0.215887	0.346567	0.074118	-0.11462	-0.20007	0.22562	-0.03475	0.428656
LEMD1	-0.28139	0.221425	0.382391	-0.27084	-0.1028	-0.24569	0.223212	-0.10461
LEMD2	0.035375	0.473485	0.052769	0.003697	0.137953	0.16591	-0.41302	-0.21092
LEMD3	0.178503	-0.16666	-0.04331	-0.17235	0.015961	0.29767	0.140808	-0.3332
LENEP	0.094397	-0.11133	-0.13282	-0.26458	0.435334	0.160514	-0.30983	0.138809
LENG1	0.494513	0.620806	-0.11866	0.002124	-0.10668	-0.29226	-0.31741	-0.43847
LENG8	0.528027	-0.03302	-0.12353	0.096863	0.050573	0.346803	0.18927	0.250935
LENG9	0.092821	-0.17665	0.193722	0.032945	0.077344	-0.0666	0.344176	0.349272
LEO1	-0.12789	-0.58848	-0.2119	0.051067	0.013118	-0.24008	-0.29685	-0.1441
LEP	-0.31344	-0.06834	-0.05317	-0.14046	0.010505	-0.17426	-0.64521	-0.59397
LEPR	-0.43421	-0.35768	-0.28046	-0.03933	0.062413	-0.12276	-0.39272	-0.17362
LEPRE1	-0.25206	0.090528	0.338811	0.216992	0.003122	0.111397	0.069496	0.396054
LEPREL1	-0.54173	0.270573	-0.15661	-0.04733	-0.22233	-0.02503	-0.33674	-0.23557
LEPREL2	-0.19769	-0.00705	0.171227	0.195242	0.017396	0.03939	-0.32088	-0.20147
LEPROT	-0.11996	-0.19104	0.291882	0.042858	-0.00063	0.116782	-0.05085	-0.4743
LEPROTL1	-0.26339	-0.48421	0.161598	-0.36437	-0.13735	-0.27499	-0.08083	0.015981
LETM1	0.060793	0.426286	-0.3606	0.054341	-0.0001	-0.10236	0.160501	0.066027
LETM2	-0.2699	0.390656	-0.2908	-0.36577	0.119599	-0.30479	-0.08301	-0.17864
LETMD1	-0.02949	-0.62095	0.065238	-0.16943	0.175702	0.151977	0.453772	0.552795
LFNG	0.157223	-0.08864	0.172618	0.054097	0.166284	-0.09551	0.279007	-0.12909
LGALS1	-0.19665	-0.04488	0.17994	-0.20553	-0.28535	0.128541	-0.31861	-0.22559
LGALS12	0.082216	-0.15738	-0.00758	0.035151	-0.00895	-0.20867	-0.53016	0.028155
LGALS13	0.388628	0.603728	-0.06922	-0.14445	0.171348	0.045398	-0.4949	-0.32611
LGALS14	0.125322	0.515635	-0.0029	0.076856	0.236501	-0.08066	0.254646	-0.38314
LGALS2	0.034392	-0.15997	0.031442	-0.06762	-0.23445	0.242814	-0.21293	-0.05008
LGALS3	-0.30763	-0.35677	-0.10271	-0.20255	-0.18436	-0.29353	-0.40756	-0.43268
LGALS3BP	0.276376	0.055454	0.439556	0.160886	0.084017	0.02946	0.417422	0.092355

LGALS4	-0.23832	0.028852	0.005944	0.027583	0.061674	-0.06201	0.22146	0.095927
LGALS7	0.09105	0.238538	-0.21279	-0.216	-0.17309	-0.06007	0.106726	-0.26422
LGALS8	0.013471	0.317544	0.053867	-0.20083	-0.38018	-0.39672	-0.26356	0.021385
LGALS9	0.18868	0.246108	0.339013	0.210182	-0.0617	-0.24516	0.060975	-0.03304
LGI1	0.17028	-0.07091	-0.15704	0.033887	-0.05978	0.204405	-0.47209	-0.4471
LGI2	0.438653	-0.06206	-0.15456	0.348433	-0.10209	0.410252	-0.15029	-0.13711
LGI3	-0.29928	-0.02342	0.236937	-0.09597	0.225777	-0.08079	-0.11097	-0.22963
LGI4	-0.06395	-0.03077	-0.16243	-0.06865	-0.3473	0.025555	-0.55433	-0.51204
LGMN	-0.11415	-0.42584	0.130182	-0.21584	-0.18985	0.126618	-0.18866	0.369653
LGR4	-0.18969	-0.06581	-0.42946	-0.13365	-0.29923	-0.01178	0.297852	0.272173
LGR5	0.238808	0.413431	-0.29233	0.262129	-0.20803	0.313725	0.460493	0.508658
LGR6	0.218305	0.195377	0.306018	0.200891	0.077587	0.379719	-0.03967	-0.3027
LGSN	NA	NA	-0.05464	-0.21866	-0.01703	-0.12256	NA	NA
LGTN	0.27222	0.294307	0.003477	-0.09103	-0.20558	-0.14285	0.615292	0.206984
LHB	-0.05885	0.758308	0.214985	-0.13536	0.106797	-0.22088	0.105187	0.020774
LHCGR	-0.05596	0.00837	0.081075	-0.04436	0.099511	0.305303	-0.09224	-0.36191
LHFP	-0.36738	-0.44447	0.013276	-0.06168	-0.09034	0.257027	-0.12594	0.067702
LHFPL1	0.240083	0.768734	0.019845	0.401138	-0.02931	-0.12178	-0.06031	0.146227
LHFPL2	-0.46162	-0.06992	0.306178	-0.32332	-0.20778	-0.18989	-0.25233	0.21109
LHFPL3	0.17156	0.056181	0.023661	-0.12674	-0.25423	-0.15029	-0.30214	-0.39998
LHFPL4	-0.13433	0.392633	-0.05804	-0.1826	-0.0497	-0.02483	NA	NA
LHFPL5	0.120182	0.100916	0.213046	-0.22577	0.088224	-0.09486	0.405182	0.362654
LHPP	-0.12666	0.013428	0.248247	-0.02475	0.126358	-0.25841	-0.50389	-0.2115
LHX1	-0.40152	-0.44192	0.102185	-0.20783	0.090937	-0.03699	0.565908	0.352677
LHX2	-0.47275	0.317817	-0.15158	0.151835	-0.17402	0.235831	-0.2753	-0.1502
LHX3	-0.00479	0.572297	0.195467	-0.07944	-0.10068	0.051266	0.221589	-0.31901
LHX4	-0.01127	0.224578	-0.00707	-0.06312	-0.12077	-0.44118	0.101713	-0.31986
LHX5	-0.13363	-0.40201	0.119151	-0.03113	-0.28843	-0.00306	-0.34844	-0.4791
LHX6	-0.11351	0.003352	-0.20035	-0.07887	0.000547	0.252828	-0.13555	-0.22027
LHX8	0.328859	0.114034	NA	NA	NA	NA	-0.14646	0.174016
LHX9	0.169275	-0.10274	-0.0736	-0.26157	0.245064	-0.13525	0.379187	0.568583
LIAS	0.229171	0.309443	0.024429	-0.18998	0.018539	-0.42039	-0.13667	-0.20234
LIF	-0.1214	-0.13073	0.138111	0.10004	0.257597	0.113381	-0.01098	0.4194
LIFR	-0.09506	-0.63546	-0.00613	0.204681	-0.40028	0.207594	-0.3552	-0.15443
LIG1	0.08118	-0.41297	-0.11612	0.526782	0.452536	0.353951	0.596829	0.298955
LIG3	0.451432	0.27798	0.168401	0.24779	0.11038	0.057534	0.13524	0.262387
LIG4	-0.05052	0.538613	-0.08016	-0.07543	-0.13528	-0.28437	-0.39496	-0.29602
LILRA1	0.195107	0.634179	0.126018	0.21755	-0.05564	-0.29861	-0.04204	-0.38831
LILRA2	-0.32194	-0.30042	0.211567	-0.09985	-0.02269	-0.03153	-0.51571	-0.05602
LILRA3	-0.3698	-0.1529	0.180469	0.131034	-0.05421	0.060385	-0.2848	0.272403
LILRA4	-0.13857	-0.29108	0.42794	0.160836	-0.03126	0.053454	-0.10293	0.056615
LILRA5	-0.43374	-0.09956	0.186432	0.011549	-0.06179	-0.17485	-0.44398	0.530725
LILRA6	0.086282	0.255711	NA	NA	NA	NA	0.04228	-0.04829
LILRB1	0.387053	-0.09125	0.198938	0.44375	-0.33857	0.343406	-0.51569	-0.43891
LILRB2	0.188248	0.155929	0.082066	0.060405	-0.15473	0.219151	-0.4599	0.535769
LILRB3	-0.46925	0.02041	0.216528	-0.00629	0.001345	0.090542	-0.37115	0.271124
LILRB4	-0.20259	-0.349	-0.07582	-0.08312	0.214723	0.39062	-0.3346	0.129539
LILRB5	-0.2827	-0.2105	0.110735	-0.41103	-0.26641	-0.05303	-0.49419	0.240457
LILRP2	NA	NA	0.299533	0.053446	-0.0169	-0.03175	NA	NA
LIM2	0.025129	0.206942	-0.17355	-0.27266	-0.11426	0.116417	0.203245	0.016038
LIMA1	-0.23134	-0.3274	-0.02326	-0.24605	-0.0684	-0.04031	-0.02017	0.387766
LIMCH1	NA	NA	-0.03044	-2.6E-05	-0.11533	-0.10517	NA	NA

LIMD1	0.341548	0.795204	-0.2837	-0.01671	0.173731	-0.01092	0.090178	0.068153
LIMD2	0.199302	0.802138	0.349897	0.841166	-0.16998	0.737779	-0.28609	0.14887
LIME1	0.037099	0.512806	0.265439	0.148351	0.241462	-0.1286	-0.06042	-0.24482
LIMK1	0.188287	0.347446	0.112872	0.602527	-0.19267	0.146414	-0.05945	-0.20752
LIMK2	-0.42169	-0.03936	0.180989	0.325954	0.143691	0.074326	0.308655	0.083299
LIMS1	-0.31226	-0.05599	-0.14887	-0.11913	-0.1165	0.270083	-0.1636	-0.28551
LIMS2	-0.06735	-0.01518	0.124174	-0.06976	0.067922	0.141728	-0.36159	-0.42303
LIMS3	0.156208	0.099347	-0.05601	-0.16144	-0.01732	-0.23472	-0.42746	-0.25205
LIN28	-0.14837	-0.06523	-0.48446	0.045305	-0.14707	-0.34462	-0.03953	0.064814
LIN28B	0.34491	0.639761	-0.15904	0.054467	0.216486	-0.29002	-0.22843	-0.15509
LIN37	NA	NA	0.120095	0.034376	-0.34731	-0.25056	NA	NA
LIN52	-0.2219	-0.14933	-0.1558	-0.03714	-0.00552	-0.39677	NA	NA
LIN54	-0.45646	0.117256	-0.23555	-0.34002	-0.3524	-0.64232	NA	NA
LIN7A	-0.01487	-0.23087	-0.2079	-0.03755	-0.12823	-0.22086	-0.01606	0.455565
LIN7B	0.03633	-0.3677	0.133392	0.161594	0.151289	-0.23444	0.082423	0.060862
LIN7C	-0.1169	-0.22661	-0.42486	-0.05333	-0.1565	-0.29413	-0.34972	-0.14322
LIN9	0.013549	0.321045	-0.38783	-0.21318	-0.12773	-0.17654	0.422096	0.306307
LINGO1	0.340318	0.073084	-0.21937	-0.08121	-0.0493	-0.28746	NA	NA
LINGO2	0.188291	0.549615	0.115323	-0.16485	0.047213	-0.06964	NA	NA
LINGO4	-0.18424	0.550572	-0.19872	-0.31596	0.171353	-0.14236	0.299845	-0.19249
LINS1	-0.12839	0.267524	-0.2082	-0.20562	-0.12516	0.145983	-0.32171	-0.02732
LIPA	0.129909	-0.2233	0.249813	-0.45315	-0.17489	-0.38838	-0.15739	0.457445
LIPC	0.333198	0.303599	-0.10839	-0.12554	-0.14043	-0.58536	0.340433	-0.32705
LIPE	0.235391	-0.12307	-0.16532	0.400862	0.184191	0.235786	0.586325	0.436493
LIPF	-0.33651	0.132037	0.156331	0.077815	-0.45713	0.038552	0.631596	0.517826
LIPG	-0.25924	-0.08736	-0.22956	0.072488	0.008746	0.287692	0.112594	0.452069
LIPH	-0.23875	0.28857	-0.20265	-0.175	-0.08509	-0.30735	-0.12506	-0.22011
LIPI	-0.05926	0.062019	0.033505	0.037593	0.280519	-0.12827	-0.25818	-0.1744
LIPJ	0.344123	0.724805	NA	NA	NA	NA	NA	NA
LIPK	NA	NA	NA	NA	NA	NA	-0.14107	-0.13675
LIPT1	0.003423	-0.40865	0.139194	-0.39471	-0.08634	-0.15162	0.592732	0.632555
LITAF	-0.39051	-0.42958	0.324196	0.098717	-0.01603	-0.25693	0.057043	0.064643
LIX1	-0.09032	-0.11566	0.175971	-0.13186	-0.06606	0.091469	0.057576	-0.01526
LIX1L	-0.23818	0.092917	0.093364	0.166505	-0.19786	0.288523	-0.33442	-0.24284
LLGL1	-0.30101	-0.05803	0.249501	0.032428	-0.32499	-0.08015	0.185139	0.018498
LLGL2	NA	NA	0.336366	-0.0691	0.408646	0.160627	0.501558	0.044684
LLPH	NA	NA	-0.25374	-0.09136	-0.17005	-0.38156	NA	NA
LMAN1	-0.27189	0.309744	0.046738	0.20258	-0.18443	-0.4097	-0.22862	0.14087
LMAN1L	-0.26711	0.327371	0.319978	-0.1674	0.22386	-0.00092	-0.28101	-0.4321
LMAN2	0.003649	0.358454	0.472816	0.302212	0.226158	0.024737	0.327438	-0.06318
LMAN2L	0.204677	0.604318	0.253347	-0.01144	0.197226	-0.1264	0.359277	-0.2523
LMBR1	0.384275	-0.22572	-0.1668	-0.17539	0.012806	-0.44086	0.524535	0.441122
LMBR1L	0.0776	0.585088	0.317412	0.003558	0.133302	0.059393	-0.16578	0.247206
LMBRD1	0.14403	-0.58208	0.088169	-0.38514	0.15854	-0.16237	0.312077	0.122295
LMBRD2	-0.32499	-0.37264	-0.22126	-0.27321	0.110666	-0.51257	0.055282	0.113109
LMCD1	0.300166	0.457907	0.065872	0.224141	-0.00139	0.347744	-0.30814	-0.02996
LMF1	NA	NA	0.425672	0.072652	0.119909	0.019548	NA	NA
LMF2	NA	NA	0.397034	0.064037	0.163173	-0.11332	NA	NA
LMLN	-0.20884	-0.47235	-0.2806	0.097546	0.002234	-0.29704	-0.02007	-0.10533
LMNA	-0.07063	0.254751	0.130099	0.026748	0.0462	0.225895	0.109134	-0.2085
LMNB1	-0.24019	-0.02567	0.032822	0.201849	0.003484	-0.03091	0.310892	0.149071
LMNB2	0.089025	0.243954	-0.3732	0.342063	-0.01953	-0.07703	0.479936	0.316554

LMO1	-0.23568	0.180741	-0.1807	-0.3015	0.116014	-0.11117	-0.16283	0.05008
LMO2	-0.43106	-0.40601	0.021866	0.479878	-0.24619	0.132584	-0.54419	-0.28629
LMO3	0.03483	-0.15912	0.21661	-0.16175	0.033512	0.051257	-0.13629	-0.24591
LMO4	-0.51763	-0.123	0.165474	0.182289	-0.07535	0.205501	-0.59324	-0.02093
LMO7	0.44058	0.317288	0.036084	-0.22493	0.197812	0.062057	0.373668	0.351476
LMOD1	0.147283	-0.10087	0.067879	-0.07652	0.050468	-0.02898	-0.28218	-0.30401
LMOD2	NA	NA	-0.16014	-0.1231	-0.18735	0.030384	NA	NA
LMOD3	-0.08899	-0.00499	0.093371	-0.35495	-0.15833	-0.12147	-0.45025	-0.42734
LMTK2	0.121783	0.01085	0.191444	-0.23732	0.454337	0.126544	-0.23176	-0.26041
LMTK3	NA	NA	0.147174	-0.01863	0.081034	-0.16269	0.098009	0.232962
LMX1A	0.567364	0.342674	0.096021	-0.14332	0.281171	-0.20521	0.029802	0.242492
LMX1B	0.39718	0.794379	0.0085	0.213945	-0.08419	-0.14824	-0.48884	-0.33632
LNPEP	0.114701	0.516222	0.083897	0.056878	-0.17289	-0.25004	0.351217	0.410239
LNX1	-0.04097	-0.04776	0.071557	-0.1312	0.038425	-0.40681	0.38637	0.28939
LNX2	0.440715	-0.15154	0.167708	-0.1366	0.312771	-0.52109	0.555834	0.001785
LOC100130	NA	NA	0.095767	-0.07742	-0.12064	0.113981	NA	NA
LOC100130	NA	NA	-0.31071	0.07185	-0.01378	-0.32569	NA	NA
LOC100130	NA	NA	0.050997	0.147504	-0.08296	-0.34089	NA	NA
LOC100132	NA	NA	0.076311	-0.13378	0.290059	0.11604	NA	NA
LOC100133	NA	NA	-0.13684	0.004296	-0.05815	0.083527	NA	NA
LOC100192	NA	NA	-0.30109	0.214619	-0.08743	-0.29495	NA	NA
LOC100216	NA	NA	-0.03956	0.260096	-0.16609	-0.16856	NA	NA
LOC116236	0.063738	-0.24326	0.319361	0.001571	0.192032	-0.37738	-0.29822	0.012638
LOC123688	0.279468	-0.09026	0.06817	-0.35122	-0.08353	-0.5844	-0.55061	-0.48204
LOC124220	-0.0946	0.258078	0.205152	0.430726	0.086293	-0.14959	0.052485	-0.13785
LOC126536	NA	NA	-0.45816	-0.15582	-0.15393	-0.12138	0.090532	0.058245
LOC136242	-0.01559	0.323336	0.194415	-0.15793	-0.02832	-0.13841	0.007441	0.031318
LOC143188	0.332163	0.432217	NA	NA	NA	NA	-0.16882	0.460817
LOC147804	-0.34743	-0.37681	-0.29988	0.310327	-0.0475	-0.27097	NA	NA
LOC148413	NA	NA	0.384155	0.286958	0.217963	0.250702	NA	NA
LOC149134	-0.14547	-0.22771	-0.27915	-0.38357	-0.07963	-0.13183	NA	NA
LOC149950	-0.1785	0.155104	NA	NA	NA	NA	-0.58551	-0.41502
LOC150568	NA	NA	-0.24372	0.385782	0.034849	0.207574	NA	NA
LOC150763	-0.09162	0.449157	-0.24049	-0.06272	-0.17363	-0.09605	-0.54022	-0.63581
LOC151121	0.174862	0.42008	NA	NA	NA	NA	-0.07654	0.410354
LOC153328	-0.11494	0.508735	0.050151	0.109649	-0.01594	-0.37188	-0.329	-0.45494
LOC153364	0.154088	-0.50903	0.26077	0.13233	0.01285	0.061659	0.279086	0.160307
LOC154761	NA	NA	0.20873	0.120935	-0.31289	0.145764	NA	NA
LOC158381	0.362864	0.447777	-0.24345	-0.30388	-0.08957	-0.25544	0.542068	0.389307
LOC158830	-0.15136	-0.04919	-0.37324	0.076572	-0.449	0.185815	0.061456	0.128772
LOC196913	0.067	0.566112	-0.28346	-0.25529	0.143749	0.075392	0.132188	0.277905
LOC200261	0.112778	0.210467	0.239851	0.012089	-0.08868	-0.2608	NA	NA
LOC200810	-0.15716	-0.23929	-0.31316	0.272787	0.031507	-0.4055	-0.21859	-0.38818
LOC201175	-0.22101	0.299188	0.281444	-0.16086	0.20837	0.024921	0.245929	-0.12304
LOC202181	NA	NA	0.131233	-0.28749	0.183926	0.144472	NA	NA
LOC220729	NA	NA	0.014998	0.167939	0.220146	0.043551	NA	NA
LOC221442	-0.27864	0.41571	0.163868	0.410599	0.170122	0.043716	NA	NA
LOC255374	0.112462	-0.1843	0.039898	0.005125	0.252173	-0.10947	-0.06998	0.092805
LOC257358	0.073156	0.036552	-0.19999	0.055362	0.022698	-0.01375	0.071992	-0.07704
LOC283174	-0.17712	-0.26628	-0.41982	0.027745	0.036834	0.351413	-0.08114	0.065366
LOC283392	0.203833	0.325851	NA	NA	NA	NA	0.544249	0.485751
LOC284009	0.498434	0.747664	NA	NA	NA	NA	0.276861	-0.06021

LOC284402	-0.23547	0.365814	-0.01737	-0.09001	-0.07552	-0.12375	0.156248	0.072054
LOC284441	NA	NA	-0.09017	0.001802	-0.15215	0.13233	0.162818	0.396208
LOC284751	0.135796	-0.20395	NA	NA	NA	NA	-0.12251	-0.02711
LOC284757	-0.15193	-0.39844	NA	NA	NA	NA	-0.50869	0.02117
LOC284912	0.139394	0.716299	0.207053	-0.15372	0.180127	-0.23963	-0.49129	-0.56439
LOC285033	NA	NA	-0.03257	0.038987	0.106968	-0.29026	0.311136	0.105069
LOC285074	-0.00054	-0.3235	0.098893	-0.13314	0.037542	0.197903	-0.16891	0.104201
LOC285908	0.106325	0.397677	-0.03624	0.141559	0.267255	0.318777	0.145916	0.306648
LOC338579	NA	NA	0.015565	-0.06691	-0.22129	0.179276	NA	NA
LOC338799	NA	NA	-0.02002	0.142176	0.02111	-0.10715	0.278528	0.548441
LOC339240	NA	NA	-0.29399	-0.02237	-0.15531	0.102263	0.139625	0.409055
LOC339344	0.180547	0.327345	-0.00939	0.046582	-0.07266	0.229843	0.016392	-0.18108
LOC339524	0.095293	0.129146	0.233676	0.035416	-0.02759	-0.06522	NA	NA
LOC339803	NA	NA	0.334006	0.241211	-0.20531	0.156649	NA	NA
LOC348174	-0.17367	0.106718	-0.0574	0.122248	0.247827	-0.02408	-0.41609	-0.22785
LOC348262	0.178973	0.04361	0.231371	0.102529	-0.03789	0.01871	-0.03927	-0.10282
LOC348840	0.023655	0.359344	0.145532	0.041804	0.23177	-0.16859	-0.29378	-0.22104
LOC349196	0.13922	0.462172	0.098272	-0.01046	-0.07666	0.147259	NA	NA
LOC375190	0.045188	0.650957	NA	NA	NA	NA	NA	NA
LOC388503	-0.17747	0.487349	-0.0887	-0.19477	0.176818	0.171707	0.036191	0.043579
LOC388796	NA	NA	0.029874	-0.07277	0.116522	0.462652	NA	NA
LOC388882	-0.29322	-0.14818	NA	NA	NA	NA	-0.37784	-0.09924
LOC388965	-0.06605	0.207082	0.040373	-0.13745	0.060296	-0.30264	-0.29498	-0.15523
LOC389199	-0.40817	0.300668	0.101452	-0.18005	-0.13366	0.056494	NA	NA
LOC389458	0.185491	-0.10892	0.183134	0.057821	0.066287	-0.37904	-0.11087	-0.1169
LOC389517	-0.2064	-0.4832	0.116925	-0.20888	0.090603	0.333682	0.545783	0.491797
LOC389634	-0.08466	-0.09781	-0.372	-0.227	0.379195	-0.28312	NA	NA
LOC389705	NA	NA	-0.05487	-0.20703	-0.19641	-0.32032	NA	NA
LOC389791	0.150953	0.254776	0.308184	0.053593	0.180852	0.007198	NA	NA
LOC389834	0.029628	-0.15479	NA	NA	NA	NA	0.141053	-0.05209
LOC399744	0.173566	0.047541	NA	NA	NA	NA	0.01299	0.472499
LOC399900	-0.2889	-0.00369	-0.03674	-0.1995	0.124556	-0.03587	-0.19181	0.069033
LOC400464	-0.3672	-0.09708	-0.03376	0.200376	-0.21512	0.609039	-0.30722	-0.02121
LOC400657	0.097166	-0.33608	0.101929	0.196778	0.096846	-0.27853	0.256009	0.556656
LOC400891	0.060764	0.113525	NA	NA	NA	NA	0.034433	-0.31232
LOC400986	-0.29435	0.015525	0.022433	-0.12729	0.078332	0.422969	-0.5448	-0.55573
LOC401010	NA	NA	0.189463	-0.02647	0.297026	-0.02629	-0.26521	-0.38309
LOC401052	-0.14317	-0.0348	0.100834	-0.15048	-0.03445	-0.29089	0.067846	-0.2315
LOC401233	-0.17387	-0.16682	0.013841	-0.06019	0.404758	-0.04195	0.310105	-0.01328
LOC401296	0.380318	0.466274	NA	NA	NA	NA	0.395777	0.332175
LOC401357	0.18373	0.788856	0.060063	-0.15974	0.078819	0.37028	0.330583	0.308247
LOC401431	0.296327	-0.26136	NA	NA	NA	NA	0.155711	0.249463
LOC402176	-0.02507	-0.23328	0.217686	-0.17598	0.046898	-0.0784	-0.26094	-0.03474
LOC407835	NA	NA	0.309827	0.237477	0.32051	-0.03355	0.399774	0.238171
LOC440040	NA	NA	0.362811	0.303744	-0.15397	0.092145	NA	NA
LOC440093	-0.47222	-0.61331	0.224154	0.061447	0.10074	0.21754	-0.69085	-0.52017
LOC440173	NA	NA	-0.10374	-0.24433	0.17966	-0.22609	NA	NA
LOC440248	0.026603	0.103774	NA	NA	NA	NA	0.35529	0.113215
LOC440295	0.053837	0.069758	-0.05601	-0.08998	0.227442	0.172151	-0.32061	-0.32231
LOC440313	0.387087	0.745475	NA	NA	NA	NA	-0.34153	-0.42901
LOC440337	0.20066	0.058402	-0.4213	-0.13342	-0.11302	0.210164	NA	NA
LOC440354	NA	NA	-0.26136	-0.04009	-0.12751	0.027495	0.288885	-0.21073

LOC440905	-0.0582	0.057348	NA	NA	NA	NA	NA	NA
LOC440925	-0.02057	-0.01705	NA	NA	NA	NA	0.54358	0.101353
LOC440944	0.058689	0.187476	NA	NA	NA	NA	-0.03971	0.143068
LOC441046	-0.14572	-0.07945	-0.3526	0.331272	-0.09251	0.010242	0.22951	0.133884
LOC441108	0.037195	-0.08287	0.481958	-0.21229	0.18609	0.363806	0.089579	0.247461
LOC441150	0.11967	0.447096	-0.02492	-0.31273	-0.06918	-0.31111	0.027219	0.311606
LOC441178	0.076808	0.531986	-0.15286	0.220566	0.486954	0.077693	-0.23202	-0.06665
LOC441208	0.10246	0.151595	0.170515	0.158971	0.072387	-0.10716	0.259969	0.113271
LOC441242	NA	NA	NA	NA	NA	NA	0.293459	0.310491
LOC441294	0.383577	0.69287	-0.34045	-0.03879	-0.06631	-0.10867	0.207984	0.302536
LOC441376	0.138982	0.029778	0.454722	-0.0075	-0.1584	0.037666	-0.18638	-0.3558
LOC441426	0.118367	0.343485	NA	NA	NA	NA	-0.16587	-0.50207
LOC441601	NA	NA	0.147154	-0.07802	0.053284	0.226758	-0.10808	-0.18129
LOC442459	NA	NA	-0.16293	0.020313	-0.08157	-0.04391	NA	NA
LOC492311	0.068718	-0.39116	0.149981	-0.25869	0.079322	-0.49647	-0.54431	-0.45529
LOC493754	NA	NA	-0.22676	0.027212	0.06753	0.362752	NA	NA
LOC497190	0.381299	0.284059	-0.33242	0.084286	0.096811	-0.37173	0.254709	-0.00677
LOC51057	-0.08506	-0.16581	-0.13022	0.215183	-0.12545	0.090192	0.098495	-0.14745
LOC51233	0.180298	0.772866	-0.19918	-0.01908	-0.23831	0.004553	0.453874	0.154358
LOC541469	0.087465	0.244628	-0.24186	-0.23457	-0.2764	-0.32945	-0.13295	-0.00579
LOC541473	-0.09628	0.544322	0.024026	-0.15927	0.025289	-0.05336	0.071287	0.064458
LOC554235	-0.14979	-0.23526	0.234254	-0.07942	-0.18577	-0.06861	0.068345	0.323615
LOC55908	0.154371	0.736227	0.233263	-0.06171	0.034697	0.172424	-0.05737	0.16988
LOC57228	-0.11883	0.194257	0.022155	0.01252	0.048585	-0.06925	0.423882	0.069557
LOC595101	NA	NA	0.194944	-0.15734	0.352474	0.316317	0.207333	0.089903
LOC63920	0.372635	0.441428	0.265517	-0.13272	-0.01118	-0.25326	-0.04633	-0.12035
LOC643923	NA	NA	NA	NA	NA	NA	-0.04938	0.224824
LOC645676	0.219356	-0.34721	-0.05101	-0.19047	-0.02993	-0.05963	NA	NA
LOC646627	NA	NA	-0.07026	-0.31958	-0.07903	-0.20813	NA	NA
LOC650293	NA	NA	-0.38183	-0.09763	-0.10555	-0.22511	0.358534	0.021228
LOC728613	NA	NA	0.262951	0.253974	0.125361	-0.18476	NA	NA
LOC729603	NA	NA	-0.02074	-0.40962	0.238512	0.337557	-0.52981	-0.56905
LOC81691	-0.23975	0.448024	-0.1561	0.273099	0.211223	-0.33991	0.47022	0.395618
LOC90826	-0.13322	-0.47732	-0.27331	-0.24562	-0.28819	0.050236	-0.04724	-0.19297
LOC90835	0.039662	0.030124	-0.08478	0.365175	0.149404	-0.17355	0.100092	0.091218
LOC90925	0.05714	0.691215	NA	NA	NA	NA	-0.22364	-0.04251
LOC91431	-0.25408	-0.58884	-0.38064	0.167516	0.217877	0.178512	-0.27465	-0.02487
LOC92270	-0.24601	-0.13088	0.090877	0.009816	-0.07337	-0.05065	0.223896	-0.21091
LOC93622	-0.06094	0.337167	NA	NA	NA	NA	0.233344	-0.12836
LOH12CR1	0.08653	-0.41876	-0.13942	0.012852	-0.08891	-0.09358	-0.4269	-0.51129
LOH3CR2A	-0.17826	-0.20542	-0.11128	-0.01373	-0.06579	0.360195	-0.22719	0.220173
LONP1	0.46461	-0.06803	-0.01991	0.165457	0.302182	0.305449	NA	NA
LONP2	0.157861	0.021349	-0.25699	0.076176	0.392807	-0.09221	-0.64019	-0.5234
LONRF1	0.223624	-0.43194	0.119459	-0.1293	0.063767	0.265597	0.398591	0.142523
LONRF2	-0.0723	-0.15792	-0.10678	-0.08402	0.023308	0.101919	-0.43005	-0.17417
LONRF3	-0.25941	-0.2213	-0.51953	-0.09184	-0.2482	-0.0435	0.057362	0.196179
LOR	0.105736	0.334688	0.017164	0.581445	0.116851	0.426182	-0.14989	0.149655
LOX	-0.32562	-0.3285	-0.10282	-0.01613	-0.1266	-0.1289	-0.04855	0.031366
LOXHD1	0.009443	0.777667	-0.14932	-0.15066	0.303266	0.032771	-0.03793	-0.0178
LOXL1	0.072053	-0.19804	0.02741	0.137419	-0.01237	0.19724	-0.1525	0.009146
LOXL2	0.184721	0.652251	0.027666	-0.00063	0.063961	0.24555	0.228585	0.250267
LOXL3	0.159295	0.088749	-0.19195	-0.1227	0.000749	0.159812	0.06497	0.193552

LOXL4	-0.20832	-0.59969	0.287306	0.071676	-0.0702	0.247722	-0.31823	-0.09431
LPA	NA	NA	0.073356	0.218252	-0.02037	0.214021	0.274117	0.341755
LPAL2	-1.2E-05	0.505881	0.171206	0.209729	0.177279	0.208411	0.453448	0.429555
LPAR1	NA	NA	-0.04079	-0.12475	-0.22014	-0.03063	NA	NA
LPAR2	NA	NA	0.297921	0.037831	0.243681	0.254423	NA	NA
LPAR3	NA	NA	-0.20943	0.016324	-0.26153	0.200125	NA	NA
LPAR4	NA	NA	-0.01552	-0.07872	0.050185	-0.01392	NA	NA
LPAR5	NA	NA	0.20402	0.474337	-0.05997	0.198689	NA	NA
LPCAT1	NA	NA	0.213809	0.523173	0.119731	0.309699	NA	NA
LPCAT2	NA	NA	-0.00264	-0.31247	0.089615	-0.385	NA	NA
LPCAT3	NA	NA	0.32437	-0.10795	0.116341	-0.02071	NA	NA
LPCAT4	NA	NA	0.135376	0.026799	0.040314	-0.19718	NA	NA
LPGAT1	-0.44276	-0.49575	0.023988	-0.02534	-0.01034	0.056761	0.473087	0.579356
LPHN1	0.078839	0.086368	-0.23859	-0.17592	0.276852	0.039269	0.275552	0.255383
LPHN2	-0.07849	0.064724	-0.0512	-0.09134	-0.17807	-0.30538	-0.46851	-0.45037
LPHN3	0.439034	-0.60598	-0.12667	-0.10688	0.082991	-0.16828	0.033961	-0.07544
LPIN1	-0.14265	-0.40996	0.111901	0.131016	0.259551	0.224755	-0.27432	0.135774
LPIN2	-0.06209	-0.44317	0.057584	-0.33704	0.034704	-0.3493	-0.59607	-0.39319
LPIN3	-0.05359	-0.25905	0.343237	-0.16539	0.11964	-0.26859	0.564869	0.303235
LPL	0.133088	-0.30193	-0.06349	0.191962	0.097133	0.041109	-0.13263	0.158841
LPO	-0.0999	0.13229	-0.06896	-0.18318	0.066307	-0.23417	0.332232	0.416356
LPP	-0.09896	-0.08633	-0.00337	-0.18201	-0.08545	0.104007	-0.34757	-0.36815
LPPR2	-0.0766	-0.08972	0.053097	-0.17484	0.251682	-0.00634	-0.30341	-0.01765
LPPR4	-0.41373	-0.38144	-0.19275	-0.06759	-0.39719	0.199775	-0.28822	0.043353
LPXN	-0.09854	-0.04594	0.014655	0.521695	-0.23016	0.38786	-0.64933	0.077878
LRAT	-0.36883	-0.31463	-0.11631	-0.17262	-0.18232	0.069718	-0.16776	0.243176
LRBA	-0.14104	-0.60245	-0.02754	-0.20506	-0.08201	0.1663	0.502043	0.192735
LRCH1	0.209049	0.674365	0.344196	-0.12489	0.418244	0.144901	-0.28514	-0.27562
LRCH2	-0.0774	-0.18438	0.0566	-0.08855	-0.19749	0.114791	-0.25373	-0.18863
LRCH3	-0.23643	-0.73039	-0.31544	0.092521	-0.21717	0.13143	0.180089	0.102443
LRCH4	-0.31513	0.343525	NA	NA	NA	NA	-0.49235	-0.10565
LRDD	0.117601	0.581958	0.243281	0.23226	0.37227	0.039272	0.061074	0.190152
LRFN1	NA	NA	0.010518	-0.16899	0.109153	0.002625	NA	NA
LRFN2	-0.35802	-0.3267	-0.39201	0.095839	-0.05375	-0.38837	0.354158	-0.0144
LRFN3	0.086393	0.503189	0.03423	0.135893	0.100493	0.012464	0.156622	0.09538
LRFN4	0.132994	0.6618	0.094291	-0.00646	0.389277	0.344183	0.489122	0.03062
LRFN5	-0.17528	0.018467	0.170983	-0.07152	0.007665	-0.26431	-0.32061	-0.04315
LRG1	-0.1531	0.445979	-0.09665	0.094307	0.049073	0.080844	-0.3057	-0.19354
LRGUK	0.215793	-0.03464	-0.0772	-0.17577	-0.09984	-0.04794	NA	NA
LRIG1	-0.07233	-0.36041	0.164886	-0.00588	0.467093	0.072576	0.01638	-0.14649
LRIG2	-0.24518	-0.58408	0.067031	-0.42537	0.250882	0.241545	0.274373	0.245976
LRIG3	0.032127	-0.56992	-0.28373	-0.11373	-0.05691	-0.24672	0.100703	0.009779
LRIT1	NA	NA	-0.22212	-0.07936	-0.2265	0.054077	NA	NA
LRIT2	NA	NA	-0.00582	-0.10734	2.45E-05	-0.22978	NA	NA
LRMP	-0.3336	0.241498	0.068928	0.661483	-0.18531	0.448539	-0.37293	0.262564
LRP1	0.250169	-0.14504	0.270958	-0.00154	0.421335	0.112444	-0.52406	0.032423
LRP10	-0.14297	0.193716	0.18271	0.024828	0.080432	0.009875	-0.57294	-0.57255
LRP11	-0.06181	-0.46704	-0.15239	-0.04763	0.083648	0.030835	0.608968	0.002131
LRP12	0.180845	0.740441	-0.1659	-0.28225	-0.09876	-0.01303	-0.01868	0.007949
LRP1B	0.104699	-0.23751	-0.12057	-0.19175	0.188925	-0.40956	-0.14044	0.040486
LRP2	0.055608	0.669667	-0.05771	-0.03219	0.022788	-0.19084	0.406818	0.252407
LRP2BP	-0.06323	0.180775	-0.19831	-0.21928	-0.08001	0.008185	-0.15393	-0.32327

LRP3	NA	NA	-0.00114	-0.06937	-0.11692	0.226537	0.094998	-0.06726
LRP4	0.164132	-0.22131	-0.11304	-0.05852	0.160418	-0.02835	0.320052	0.32803
LRP5	0.095988	0.554358	0.285753	0.207716	0.409035	0.139597	0.510714	0.438875
LRP5L	-0.14882	0.212239	-0.18059	-0.0337	0.076999	0.143045	0.419276	0.116712
LRP6	0.255294	0.671644	-0.19956	-0.33766	-0.26914	-0.27554	0.520473	0.100002
LRP8	-0.02088	-0.39766	-0.2201	0.261337	0.401787	0.164741	0.13667	0.216502
LRPAP1	-0.09587	0.028931	0.274041	0.312613	-0.13258	-0.08965	-0.10458	-0.25215
LRPPRC	0.350554	0.196597	-0.27008	0.198755	0.247174	0.065152	0.658207	0.414852
LRRC1	-0.13874	-0.41075	-0.39205	-0.48919	0.077693	-0.1115	-0.07553	-0.14556
LRRC10	-0.10541	0.010393	NA	NA	NA	NA	0.260274	-0.00835
LRRC14	0.296208	0.326204	-0.09095	0.286557	-0.20193	0.017452	-0.11005	-0.10562
LRRC15	0.179577	0.198326	0.179599	0.298482	-0.01346	0.114676	-0.30016	-0.37808
LRRC16A	NA	NA	-0.19373	0.029647	0.11224	0.016352	NA	NA
LRRC16B	NA	NA	-0.20093	0.264089	0.104102	-0.21686	NA	NA
LRRC17	0.232613	-0.07782	-0.39398	-0.13444	-0.41186	-0.06774	-0.11898	-0.02117
LRRC18	-0.11123	-0.44775	0.076893	-0.05096	0.097799	-0.09981	0.047998	0.058785
LRRC19	0.035817	-0.00421	-0.15584	-0.14352	0.031285	-0.39425	-0.30925	-0.23261
LRRC2	0.052243	0.185902	-0.11096	0.338999	-0.18383	-0.05203	0.00968	0.101929
LRRC20	-0.05387	0.11404	0.030109	-0.06577	0.522641	0.064299	0.020505	-0.21875
LRRC23	0.374514	-0.08977	0.136789	-0.05032	0.023247	-0.14374	0.047265	-0.20344
LRRC24	0.160265	0.009677	NA	NA	NA	NA	-0.09815	-0.24049
LRRC25	0.069006	-0.1877	0.15033	0.193852	-0.15922	0.061058	-0.27668	0.371673
LRRC27	NA	NA	-0.21016	0.012936	0.018892	-0.17297	-0.51779	-0.34878
LRRC28	0.173161	0.662819	0.148784	-0.44493	0.054172	-0.38544	-0.24376	0.145968
LRRC29	0.329854	0.581429	0.303877	-0.18625	0.192236	0.06558	0.13392	0.35386
LRRC3	-0.3618	-0.2919	0.090455	-0.30946	-0.05483	-0.09269	0.057432	-0.0739
LRRC31	0.268565	0.143383	-0.21291	-0.19937	-0.14082	-0.55709	0.052045	0.012183
LRRC32	-0.02502	-0.08127	0.265258	0.009895	-0.07771	0.211266	-0.38058	-0.29884
LRRC33	0.009819	-0.00708	0.288624	0.466808	-0.24231	0.081678	-0.29122	0.220963
LRRC34	0.407118	-0.40809	-0.18111	0.007055	0.011375	-0.07246	-0.35415	-0.66905
LRRC36	0.402128	0.475454	-0.01545	0.054218	0.289418	-0.17486	0.018059	-0.21872
LRRC37A2	NA	NA	-0.07792	0.037057	0.14796	0.257	-0.18968	-0.07509
LRRC37A3	0.634852	0.136264	-0.16316	0.125562	0.03143	-0.0853	NA	NA
LRRC37B	0.323884	0.061156	0.310148	-0.12574	-0.07531	-0.37396	-0.45982	0.00138
LRRC39	-0.155	-0.31346	-0.24377	0.140351	0.134506	-0.21147	-0.40358	-0.38597
LRRC3B	0.202286	0.081738	0.100222	0.009503	-0.19619	-0.09116	0.186261	0.228422
LRRC4	0.078687	0.628726	-0.45186	0.041221	-0.13151	-0.30485	-0.2625	-0.20272
LRRC40	-0.00523	0.458133	-0.32157	-0.39936	0.011742	-0.27806	-0.21207	-0.08471
LRRC41	0.15805	0.593133	0.32272	-0.13091	0.102828	-0.28218	-0.51774	-0.64587
LRRC42	-0.06749	0.245824	0.387213	0.022401	0.279243	-0.13647	0.218301	-0.44644
LRRC43	-0.10154	-0.0294	0.148268	-0.19768	0.256967	-0.01521	0.054764	0.03248
LRRC45	-0.09886	-0.37259	0.325606	0.031508	0.397212	-0.02061	0.596773	0.332365
LRRC46	0.402163	-0.34923	0.091901	0.104858	-0.08709	-0.40922	0.150821	-0.29677
LRRC47	-0.31054	-0.20856	0.097361	0.273646	0.484619	0.347215	0.162346	-0.29682
LRRC48	0.025416	0.453496	-0.05715	0.276994	-0.27151	0.115554	0.32546	0.206489
LRRC49	0.723199	0.137264	0.181585	-0.40701	0.108322	0.106464	0.182802	-0.0627
LRRC4B	NA	NA	0.16432	-0.18063	-0.09271	-0.3328	-0.15252	0.330484
LRRC4C	-0.16189	-0.23655	0.211274	-0.02253	-0.28729	-0.0181	-0.31724	-0.36658
LRRC50	-0.36133	0.204446	-0.11105	-0.03425	-0.23598	0.156679	0.044868	-0.30969
LRRC52	-0.09646	0.01987	NA	NA	NA	NA	-0.08871	0.089107
LRRC55	0.126297	0.737536	0.167877	-0.26847	0.168355	-0.33063	0.471688	0.120858
LRRC56	-0.14581	0.441359	-0.34055	-0.17239	0.145435	-0.1616	0.071246	-0.13363

LRRC57	0.116942	0.137159	-0.32577	-0.18262	-0.235	-0.41868	-0.25934	-0.20652
LRRC58	NA	NA	0.017636	-0.05168	0.007196	0.235329	NA	NA
LRRC59	-0.22688	-0.63228	-0.17855	0.24157	-0.1986	-0.30625	-0.42707	-0.3827
LRRC6	0.447808	0.067888	-0.0614	-0.35407	-0.34954	-0.17033	-0.08361	-0.00516
LRRC61	0.489689	0.634997	0.145245	0.156751	-0.05406	-0.19399	0.57843	0.33592
LRRC7	0.015919	0.176606	-0.26217	-0.16531	0.280849	0.263688	-0.08997	0.23932
LRRC8A	-0.38271	0.076295	0.161227	-0.12478	0.317237	0.150893	-0.08089	-0.13065
LRRC8B	-0.04646	0.536762	0.172163	-0.24834	0.318673	0.163482	0.237365	0.397165
LRRC8C	-0.19173	-0.40472	0.040907	0.241987	-0.31053	0.490628	-0.16536	0.310548
LRRC8D	-0.08675	-0.36036	0.232606	0.119355	0.344413	-0.18993	0.550919	0.213221
LRRC8E	-0.16134	0.375246	0.067833	-0.09309	0.269347	0.137855	0.169781	-0.60793
LRRCC1	-0.00133	-0.21801	-0.19124	0.05279	-0.0749	-0.39472	-0.13302	-0.29699
LRRFIP1	-0.06695	0.200881	-0.01279	-0.20459	0.07516	0.344111	-0.52649	-0.38108
LRRFIP2	-0.13276	-0.31646	-0.23441	-0.02054	0.094702	-0.39394	0.171559	0.292015
LRRIQ1	0.109742	0.347476	-0.24035	0.066161	-0.22401	-0.01237	-0.15526	-0.17316
LRRIQ3	NA	NA	-0.10207	0.155498	0.167553	-0.04887	NA	NA
LRRK1	0.148673	-0.17897	0.199636	0.217565	0.203015	0.109082	-0.70626	-0.5419
LRRK2	NA	NA	0.030388	0.075789	0.036808	0.477815	-0.28382	-0.03695
LRRN1	0.014727	-0.34273	-0.13785	0.423976	-0.13512	0.046915	0.308172	0.505354
LRRN2	0.305939	0.175643	-0.21853	-0.11931	0.013796	-0.24236	NA	NA
LRRN3	-0.04508	-0.2516	0.22486	-0.1388	-0.37185	0.122472	-0.40426	0.456539
LRRN4	NA	NA	-0.0563	0.13951	-0.04564	-0.02762	NA	NA
LRRTM1	0.302609	0.264804	-0.12822	-0.02872	-0.27201	-0.00685	0.316235	0.194142
LRRTM2	-0.26071	0.559914	0.222577	-0.22599	-0.20053	0.13655	0.086396	0.391018
LRRTM3	-0.23801	0.291176	0.050773	0.199095	-0.14485	0.146549	-0.2566	-0.41445
LRRTM4	0.102551	-0.03991	-0.00592	-0.23943	0.057218	0.039727	-0.67949	-0.46249
LRSAM1	-0.11327	0.330622	0.186327	-0.04073	0.170972	0.330934	-0.01796	-0.38112
LRTM1	0.022463	0.588187	0.053546	-0.06627	-0.28745	0.136904	0.025689	0.018621
LRTM2	NA	NA	0.3142	0.071023	0.247544	-0.11392	0.261069	-0.29561
LRTOMT	NA	NA	0.193835	-0.15192	-0.04406	-0.48626	NA	NA
LRWD1	NA	NA	-0.02318	0.246025	0.411158	0.022121	NA	NA
LSAMP	0.319104	0.144176	0.059081	0.283118	0.047079	0.388921	-0.21134	-0.10436
LSG1	0.402512	0.663381	-0.23586	0.103725	-0.04863	0.185172	0.05031	0.163697
LSM1	0.025033	-0.64521	0.025316	-0.34346	0.082628	-0.23001	0.355594	0.256805
LSM10	0.056687	-0.48777	0.26962	0.070437	-0.12714	-0.25503	-0.44409	-0.37342
LSM11	0.179486	0.631108	0.11209	-0.34022	-0.07448	-0.09526	-0.12156	0.059648
LSM12	-0.13383	-0.41409	-0.05965	0.129077	-0.15315	-0.0676	0.250928	0.061327
LSM14A	0.224257	-0.22104	-0.02928	0.115424	0.088586	0.185869	0.329808	0.140056
LSM14B	0.114351	-0.31223	0.101299	0.306049	0.185313	0.158234	-0.40878	-0.43671
LSM2	0.397249	0.4631	-0.01517	0.001577	-0.26723	-0.09431	-0.21302	-0.43504
LSM3	0.199511	-0.70189	-0.36118	-0.20196	-0.3401	-0.32268	-0.26395	-0.27434
LSM4	0.044954	0.199272	0.104063	0.272944	0.074864	-0.17012	0.413104	0.130083
LSM5	0.451589	-0.45315	-0.39837	-0.19245	-0.23839	-0.32898	-0.23285	-0.45957
LSM6	0.137587	-0.27777	-0.17527	0.209948	-0.29281	-0.17543	-0.41854	-0.42658
LSM7	0.3208	-0.03907	-0.45543	0.303237	-0.01005	-0.24638	0.412221	0.038109
LSM8	0.195939	-0.58653	-0.03059	-0.05836	-0.12175	-0.31108	0.482687	0.358961
LSMD1	-0.26733	0.58856	-0.15794	-0.55413	-0.40776	0.027603	-0.48367	-0.52032
LSP1	-0.35106	-0.26514	0.215051	0.568789	-0.04133	0.502341	0.31928	0.230474
LSR	0.144729	0.265329	0.286921	0.107764	0.250951	0.042477	0.564001	-0.08895
LSS	0.033682	-0.119	-0.14403	0.106113	-0.08864	0.04587	0.182047	0.130994
LST-3TM12	NA	NA	0.292047	-0.13132	-0.13336	-0.21156	0.164885	-0.24556
LST1	-0.31421	0.090416	0.205936	0.072145	-0.17552	0.076419	-0.6055	0.212044

LTA	0.31948	0.327216	0.254204	0.482184	0.114029	0.489786	-0.38338	0.031798
LTA4H	0.158184	-0.06603	0.411465	-0.08316	0.086911	0.082649	-0.28029	-0.24744
LTB	-0.21708	0.422056	-0.00688	0.793677	-0.16983	0.749414	-0.58485	0.036674
LTB4R	0.112192	0.372253	0.008491	0.332536	0.320313	0.214527	-0.26149	0.448044
LTB4R2	0.184048	0.481339	-0.24515	-0.25712	0.166355	-0.06656	0.166915	0.248517
LTBP1	-0.0814	0.282318	0.039678	-0.06524	-0.02127	-0.02294	-0.3881	-0.39825
LTBP2	-0.27508	-0.27146	0.266363	0.117043	0.101111	0.598502	NA	NA
LTBP3	-0.17653	-0.33728	0.140151	0.015157	0.147552	0.310101	0.040599	0.073625
LTBP4	0.012905	0.284139	0.120526	0.104643	0.017748	0.198984	0.057166	0.168211
LTBR	-0.07972	-0.22848	0.292783	0.058149	0.308424	0.176617	0.181242	0.355077
LTC4S	-0.17174	-0.31565	0.185055	-0.03507	-0.03166	-0.06628	-0.26324	0.210207
LTF	0.440051	0.733908	-0.00313	0.693283	-0.09976	0.484902	-0.29211	-0.04921
LTK	0.120911	0.48752	-0.0433	-0.02113	-0.08959	-0.28065	0.205314	0.417315
LTV1	0.386826	-0.27969	-0.35035	0.049356	0.229864	0.035972	-0.1696	-0.14148
LUC7L	0.267485	0.518079	-0.16591	0.33286	0.14314	0.082028	0.473551	0.514518
LUC7L2	0.14458	0.553035	-0.26874	0.044703	0.277529	0.110989	0.052104	0.062857
LUM	-0.26318	-0.58742	0.096107	-0.33604	-0.14269	0.084519	-0.48607	-0.45167
LUZP1	-0.10935	0.318349	0.170286	-0.38113	0.390622	0.278277	-0.11021	0.344329
LUZP2	0.181294	0.140275	-0.317	-0.351	-0.14933	-0.29857	-0.40685	-0.07193
LUZP4	0.092557	0.41394	-0.27813	0.028045	-0.00054	0.036592	-0.20434	0.039866
LXN	-0.26573	-0.31962	0.11492	-0.20932	-0.18299	-0.39456	-0.46124	-0.15797
LY6D	-0.04156	0.55863	0.13405	0.086284	-0.07416	-0.47733	0.284771	-0.05023
LY6E	-0.21452	-0.17052	0.086716	-0.01248	-0.28765	0.18403	-0.09158	0.039805
LY6G5B	0.073076	-0.29622	-0.05148	-0.0404	-0.03085	0.19321	0.371951	0.393632
LY6G5C	0.140771	-0.22409	-0.14793	-0.04263	0.006184	-0.28337	0.409598	0.127144
LY6G6C	-0.20181	0.094062	0.127303	0.028778	0.001626	-0.02637	-0.33337	0.264001
LY6G6D	0.371416	-0.20795	NA	NA	NA	NA	0.17451	-0.33387
LY6G6E	0.488914	0.570154	0.074347	-0.08708	0.428009	-0.23251	NA	NA
LY6G6F	NA	NA	-0.09102	-0.05009	0.040692	0.099925	NA	NA
LY6H	-0.29308	0.007221	-0.13512	-0.06548	0.115596	-0.09796	-0.4176	-0.2722
LY6K	0.268531	0.416611	-0.18195	0.083299	-0.12059	-0.01538	0.222883	0.29795
LY75	-0.25581	-0.424	0.273018	-0.13481	0.08474	0.012315	-0.23944	-0.28366
LY86	-0.37604	0.156614	0.147872	0.565121	-0.40744	0.506874	-0.49423	0.215586
LY9	-0.04278	0.423291	0.205345	0.6113	-0.30387	0.525468	-0.30471	0.22896
LY96	-0.42836	0.168719	0.113181	-0.05593	-0.28795	-0.08216	-0.56781	-0.05763
LYAR	0.238947	-0.31382	-0.30724	0.248854	0.10365	0.166256	-0.24211	-0.095
LYG1	NA	NA	-0.35898	-0.02332	0.008294	0.001862	NA	NA
LYG2	0.183369	-0.19334	-0.31617	0.042972	0.073777	0.251484	-0.05725	-0.25309
LYL1	-0.40593	-0.17672	0.396394	0.731412	-0.00781	0.614755	-0.5873	-0.14112
LYN	-0.33501	-0.15148	0.410797	0.501396	-0.01998	0.501494	-0.16861	0.581072
LYNX1	0.061601	0.03545	0.185392	-0.03127	-0.05541	0.281232	-0.31999	-0.07293
LYPD1	-0.27837	-0.23305	0.056873	0.036692	-0.141	0.031811	-0.47052	-0.2426
LYPD2	-0.16668	0.30858	0.114479	-0.06987	0.121294	-0.17137	-0.50853	-0.66726
LYPD3	-0.11415	0.571463	0.120213	0.129099	0.172916	0.17322	0.050125	-0.19848
LYPD4	-0.17494	0.075933	-0.14315	0.020885	0.087278	-0.02617	-0.217	-0.02832
LYPD5	0.155216	0.385465	-0.15225	-0.24738	0.084795	0.178514	0.222992	0.162881
LYPD6	0.086865	0.01847	0.109152	-0.03491	0.113117	-0.30929	NA	NA
LYPD6B	NA	NA	0.007218	-0.18766	-0.09554	-0.14567	NA	NA
LYPLA1	0.035522	0.087343	0.158639	-0.07228	0.01879	-0.35599	-0.27756	-0.41245
LYPLA2	0.336002	0.219331	0.207005	0.268293	0.150795	-0.29453	0.526523	0.257911
LYPLAL1	-0.02396	-0.49719	0.019735	-0.2348	-0.27074	-0.1699	-0.16155	0.243933
LYRM1	0.083346	0.208229	0.312995	-0.12351	0.002552	-0.07037	NA	NA

LYRM2	0.253448	-0.54153	-0.19427	-0.18786	-0.18026	-0.01412	NA	NA
LYRM4	0.510685	0.144167	0.121545	0.30423	-0.21834	-0.12187	NA	NA
LYRM5	0.290254	0.054413	-0.28474	-0.26412	-0.34618	-0.45573	NA	NA
LYRM7	NA	NA	-0.13592	-0.08383	0.023778	-0.17336	NA	NA
LYSMD1	-0.20813	-0.51563	-0.05377	-0.29542	0.206531	-0.231	-0.00774	-0.43172
LYSMD2	0.323521	-0.40372	0.160976	0.415651	0.024146	0.469892	-0.32221	-0.03212
LYSMD3	NA	NA	0.227027	-0.26144	-0.14539	-0.02407	-0.51205	-0.31867
LYSMD4	-0.2923	0.325459	-0.1317	-0.33073	0.01292	-0.2807	-0.05822	0.400133
LYST	-0.41544	-0.43382	-0.23905	-0.11087	-0.1357	-0.11623	-0.49041	-0.00909
LYVE1	NA	NA	0.080644	0.093527	-0.23633	-0.30582	NA	NA
LYZ	-0.28221	-0.29392	0.069209	0.192314	-0.15302	0.044933	0.17009	0.504363
LYZL1	-0.14573	0.024188	-0.00979	-0.09148	-0.03518	-0.13962	-0.54779	-0.02385
LYZL2	0.291162	0.701801	-0.02281	-0.14988	-0.0779	0.062842	0.28992	0.306841
LYZL4	0.231946	0.572251	0.044727	0.380457	0.098817	-0.30586	0.589817	0.13024
LYZL6	0.252667	0.303338	0.255889	-0.07259	0.38461	0.01986	0.628052	0.484668
LZIC	-0.16128	-0.04158	-0.06762	-0.34361	-0.09144	-0.4818	0.153219	0.025242
LZTFL1	0.423501	0.08508	-0.28157	-0.1352	-0.0919	-0.30995	-0.21954	-0.31446
LZTR1	-0.15488	0.253503	0.450216	-0.01512	0.356071	0.116592	0.112968	0.071095
LZTS1	-0.25692	0.131281	0.067226	0.191916	-0.03745	0.493095	-0.01873	0.174741
LZTS2	0.036416	0.226121	-0.0324	-0.03769	0.117459	0.489956	-0.16405	-0.18852
M6PR	0.465401	0.13825	-0.14789	0.270281	-0.16438	-0.14145	0.14512	0.472583
M6PRBP1	-0.16586	-0.14823	0.139424	-0.0792	0.276966	0.035223	-0.61014	-0.0812
MAB21L1	0.043446	0.610775	0.191078	0.017559	0.022987	0.04598	0.094696	0.187153
MAB21L2	0.099735	-0.01895	-0.36178	-0.15544	-0.1981	-0.07136	-0.27979	-0.38213
MACC1	NA	NA	0.064859	-0.18167	0.206886	0.026397	NA	NA
MACF1	-0.58205	-0.23962	0.27891	-0.26211	0.075368	-0.00424	-0.16065	-0.03249
MACROD1	NA	NA	0.147892	0.090731	0.194927	-0.32886	NA	NA
MACROD2	NA	NA	-0.27457	-0.0436	-0.00486	0.205097	NA	NA
MAD1L1	0.095605	0.305405	0.219889	0.304504	0.311144	0.088415	0.407141	0.168296
MAD2L1	-0.12404	-0.30942	-0.25974	0.105707	0.067123	-0.16944	-0.02282	-0.16459
MAD2L1BP	0.001299	-0.33335	-0.08321	-0.39037	-0.09481	-0.11667	0.187458	-0.04939
MAD2L2	-0.13454	0.004768	0.17283	0.377946	0.302887	0.218065	0.386385	0.069768
MADCAM1	-0.13684	0.12366	0.046577	0.353365	-0.04283	0.35584	0.143304	0.014222
MADD	0.123527	0.494736	0.34113	0.175044	0.310214	0.386626	-0.08299	0.0231
MAEA	-0.22838	-0.45659	-0.27505	0.137023	0.031963	-0.25722	0.36714	0.212525
MAEL	-0.36674	-0.21053	-0.01895	-0.21074	-0.34526	0.036762	-0.33779	-0.22318
MAF	-0.28821	-0.32112	0.106788	-0.0268	-0.32917	0.043212	-0.57457	0.435116
MAF1	0.151786	0.153344	0.31636	-0.00812	-0.19042	-0.21258	0.315348	0.05345
MAFA	-0.35098	0.26382	0.291194	-0.21531	0.022588	0.030945	-0.27448	-0.26831
MAFB	-0.45562	-0.40261	0.311205	-0.04753	-0.34975	0.356254	-0.43014	-0.00437
MAFF	-0.43693	0.009394	0.106058	0.041034	0.195653	0.095878	-0.74006	-0.58397
MAFG	-0.12666	-0.248	0.402085	-0.03703	0.314966	-0.03306	-0.09689	0.126117
MAFK	-0.07701	0.710173	0.178057	-0.19765	0.287192	-0.03521	-0.02829	0.28663
MAG	0.425691	0.707296	0.021951	0.16116	0.01554	0.327932	-0.18143	0.379918
MAGEA1	-0.08085	0.530654	-0.19916	-0.12525	0.076508	-0.0609	-0.11953	0.016806
MAGEA10	0.118338	0.435664	0.323774	0.104655	0.066298	0.350622	0.413241	0.157139
MAGEA11	0.283585	0.72183	0.178419	-0.04561	-0.16474	-0.20496	-0.55046	-0.34358
MAGEA12	-0.1384	0.567496	-0.07705	-0.22026	-0.1396	0.140131	0.088522	0.153281
MAGEA2	0.152556	0.469684	NA	NA	NA	NA	0.027354	0.164423
MAGEA2B	0.425191	0.397233	-0.0997	-0.23619	-0.16111	-0.01276	-0.52554	-0.32432
MAGEA3	0.018217	0.364436	NA	NA	NA	NA	0.240441	0.258965
MAGEA4	0.122725	0.664453	-0.08117	-0.14027	-0.12542	-0.15039	-0.00413	0.178675

MAGEA5	-0.21651	0.183237	0.208556	-0.18177	-0.06167	-0.04921	0.283719	0.281123
MAGEA6	0.170208	0.679371	-0.04822	-0.37259	-0.11029	-0.0941	0.139351	0.183099
MAGEA8	-0.08645	0.211948	-0.17	0.281002	0.022252	-0.0618	0.143859	-0.14013
MAGEA9	0.237678	-0.00831	0.258179	0.116742	-0.17692	0.337357	NA	NA
MAGEB1	-0.01725	0.358367	-0.13107	-0.08664	-0.1002	-0.05548	0.196408	0.428299
MAGEB10	0.047896	0.137733	0.064881	-0.29917	0.063765	0.263263	0.015221	0.307326
MAGEB18	-0.02698	0.214785	-0.01166	-0.02749	-0.27871	-0.27612	-0.14806	0.158166
MAGEB2	0.305807	0.231811	-0.14456	0.159973	0.094647	-0.02894	-0.07616	0.073082
MAGEB3	0.015524	0.465206	0.014356	0.234469	0.123399	-0.01031	-0.64062	-0.28763
MAGEB4	0.195514	0.444743	-0.00339	0.165855	0.045506	0.114895	-0.23915	0.029458
MAGEB6	0.309337	0.064323	-0.28388	0.155952	-0.12099	0.22089	-0.36353	-0.41832
MAGEC1	0.360913	0.467408	-0.09561	-0.0413	0.074922	0.149488	-0.06427	0.124147
MAGEC2	0.208861	0.433235	-0.0176	0.296834	0.101839	-0.38622	-0.22583	-0.21466
MAGEC3	0.142893	0.04381	0.161305	-0.10184	-0.11408	-0.02587	-0.27481	-0.18586
MAGED1	0.111374	-0.37188	-0.00833	0.171208	-0.01686	-0.04825	0.382223	0.070825
MAGED2	0.358426	-0.07252	0.284421	0.08661	0.191815	0.256294	-0.03759	-0.49276
MAGEE1	-0.11928	-0.16207	-0.10175	-0.03723	0.243371	0.20153	-0.39027	-0.26859
MAGEE2	-0.1056	-0.364	-0.2674	-0.06942	0.065877	-0.18568	0.049138	0.129747
MAGEF1	-0.04461	0.700621	-0.2395	0.435747	-0.07487	-0.04497	0.072488	-0.25076
MAGEH1	-0.02083	-0.33595	0.08021	0.073819	-0.15228	-0.07992	0.050398	0.050934
MAGEL2	-0.00182	-0.09211	-0.0503	-0.01475	-0.34561	0.12123	NA	NA
MAGI1	-0.24067	0.274608	-0.19373	-0.37426	0.191376	-0.10082	0.2849	0.271724
MAGI2	0.121006	-0.36011	-0.43554	-0.41447	-0.07002	-0.12416	-0.51277	-0.30983
MAGI3	-0.01429	-0.09283	0.273994	-0.17427	0.225064	-0.49148	0.012009	0.296322
MAGIX	NA	NA	-0.1425	-0.24872	0.135113	-0.10069	NA	NA
MAGOH	-0.06063	-0.65263	-0.06848	-0.14769	-0.08417	-0.05523	-0.02857	-0.20928
MAGOHB	NA	NA	-0.29503	0.187802	-0.13764	-0.19569	NA	NA
MAGT1	NA	NA	-0.16093	0.016159	-0.18648	-0.40984	NA	NA
MAK	-0.09963	-0.28151	-0.07624	-0.14311	0.064049	0.206599	0.0824	-0.39177
MAK10	-0.07321	-0.41985	0.097804	-0.25733	0.000983	0.196839	-0.54489	-0.48923
MAK16	NA	NA	0.018957	-0.21324	-0.26039	0.129837	NA	NA
MAL	-0.06305	-0.09273	-0.06097	0.127901	-0.16358	0.348718	-0.50537	-0.5675
MAL2	-0.49399	-0.14306	0.152032	-0.02161	-0.05726	-0.29549	0.480248	0.045916
MALAT1	NA	NA	0.055406	-0.28135	0.121007	0.017485	-0.0385	0.250306
MALL	0.205286	-0.1662	0.008644	-0.09107	0.013857	-0.1744	0.039637	0.12359
MALT1	0.112391	-0.06222	-0.1076	0.230365	-0.21002	0.231475	-0.48876	-0.12135
MAMDC2	0.189885	-0.27726	-0.12007	-0.08599	-0.11663	0.123266	-0.06692	-0.18112
MAMDC4	-0.19694	0.463905	-0.13225	0.12482	0.075007	-0.1557	0.204789	0.467786
MAML1	0.334999	0.139742	-0.07889	0.316738	0.205617	0.297452	0.172296	0.289453
MAML2	-0.02493	-0.02656	0.065079	-0.01241	-0.24942	0.066459	0.373185	0.183838
MAML3	0.343351	0.745126	-0.11555	0.111767	-0.13424	0.078533	-0.16773	-0.28308
MAMLD1	NA	NA	-0.27772	0.021867	-0.14221	0.098509	NA	NA
MAN1A1	-0.15183	-0.58985	0.379626	-0.02091	-0.04953	-0.2242	-0.47306	-0.23443
MAN1A2	-0.286	-0.41815	0.192468	-0.26425	-0.01418	-0.17154	0.009712	0.18088
MAN1B1	-0.36115	0.433572	0.276769	-0.07784	0.284334	0.027755	-0.12566	-0.01635
MAN1C1	-0.08257	-0.50823	0.171702	0.194269	-0.04702	0.219555	-0.2932	0.251122
MAN2A1	0.27955	-0.15896	0.078953	-0.09858	0.20881	-0.04495	-0.21878	-0.15899
MAN2A2	-0.14463	-0.31363	-0.03517	0.300409	0.427365	0.357358	-0.35759	0.336346
MAN2B1	-0.21798	0.714543	0.375672	0.175513	0.022078	0.298982	-0.28207	0.273005
MAN2B2	-0.10856	-0.1914	0.293305	0.369688	0.352417	0.219328	-0.64671	-0.63622
MAN2C1	0.196247	0.603448	0.314447	-0.16738	0.180904	0.183152	0.142683	0.62487
MANBA	-0.49926	-0.4746	0.178231	0.242254	-0.05113	0.486233	0.069294	-0.23825

MANBAL	0.257425	-0.06004	0.129945	-0.15513	0.174523	-0.02506	-0.30896	-0.40895
MANEA	-0.00247	0.486728	-0.28467	-0.19202	-0.05517	-0.00165	0.355855	0.234179
MANEAL	0.275766	0.533256	0.362735	0.38533	0.356149	-0.03633	0.219176	-0.22517
MANSC1	0.137248	-0.30737	-0.02767	0.191668	0.050123	-0.2865	0.297747	-0.01286
MAOA	-0.21869	0.282517	-0.00831	-0.17747	-0.17515	-0.37089	-0.01775	0.138123
MAOB	-0.22708	-0.15602	-0.07879	-0.00714	-0.16465	0.031379	-0.13066	0.1148
MAP1A	-0.03436	0.041796	0.229881	-0.06254	0.050685	-0.01527	-0.1773	-0.11921
MAP1B	0.313479	-0.56131	-0.00774	-0.12185	0.036787	-0.16478	-0.21183	-0.17573
MAP1D	0.345149	-0.22111	-0.06674	0.008113	-0.01491	-0.06582	-0.51903	-0.05553
MAP1LC3A	0.073319	0.195151	0.43208	0.105924	0.220403	-0.14544	0.253708	0.312382
MAP1LC3B	-0.11095	-0.2528	-0.08506	-0.29123	0.003333	-0.45333	0.024442	-0.27746
MAP1LC3C	-0.13759	-0.14602	-0.21005	0.306282	-0.44079	0.069125	0.18279	0.351844
MAP1S	0.195663	0.347281	0.168135	-0.07413	0.294924	0.1562	NA	NA
MAP2	0.435902	-0.08657	0.043291	0.027288	-0.40583	-0.03094	0.162284	0.147593
MAP2K1	-0.20233	-0.48156	-0.23052	0.396567	0.404656	0.126021	-0.08429	0.084698
MAP2K2	0.182121	0.430082	0.412685	0.08461	0.33245	-0.04493	NA	NA
MAP2K3	0.08776	0.52764	0.08795	-0.08397	0.18572	0.205179	0.341584	0.229156
MAP2K4	-0.15354	-0.64862	-0.38401	-0.26833	-0.24375	-0.21024	0.462696	0.203831
MAP2K5	0.59737	0.646837	0.213019	0.128179	0.049374	-0.30782	-0.41542	-0.52231
MAP2K6	0.579832	0.394066	0.341479	-0.16001	-0.23316	-0.27398	0.138256	-0.0188
MAP2K7	0.493912	0.651777	-0.01924	-0.10558	0.103627	0.066896	0.236324	0.124425
MAP3K1	NA	NA	0.132838	0.281999	0.125377	0.471982	NA	NA
MAP3K10	0.035541	0.640693	-0.29901	0.139773	-0.02141	-0.00407	-0.56883	-0.45836
MAP3K11	0.036315	0.538961	0.328393	-0.10572	0.144144	-0.10247	0.086603	0.319749
MAP3K12	-0.14498	0.096108	0.120443	0.060225	0.031391	0.23348	-0.57673	-0.15707
MAP3K13	0.155969	-0.13812	0.092514	-0.15552	0.17861	-0.2833	0.105087	-0.02805
MAP3K14	0.021066	0.460355	0.069827	0.135062	0.368317	0.410691	-0.46105	-0.0387
MAP3K15	0.222732	0.277323	-0.19822	0.027506	-0.30784	0.033826	0.172624	0.279472
MAP3K2	0.116793	-0.60789	-0.13343	-0.24661	-0.13125	-0.35088	-0.27286	-0.24895
MAP3K3	-0.11774	-0.04337	0.442361	0.083965	0.010027	0.184772	-0.27964	0.008205
MAP3K4	0.003293	-0.15567	-0.22129	0.355909	0.236691	0.097024	0.511969	0.396556
MAP3K5	-0.0837	-0.03437	-0.17685	0.009982	-0.00485	-0.12739	NA	NA
MAP3K6	-0.6112	-0.10331	-0.14025	0.127997	-0.03614	0.459837	-0.57556	-0.27892
MAP3K7	0.586608	-0.45953	-0.37867	-0.00304	0.154673	0.14613	0.193717	0.10606
MAP3K7IP1	0.212201	0.395745	0.383154	0.159453	0.471255	0.267252	0.286309	-0.01555
MAP3K7IP2	0.066882	-0.53561	-0.26458	-0.10267	0.215508	0.217097	-0.29063	-0.28131
MAP3K8	-0.36107	-0.26802	0.344183	0.215123	-0.07792	0.271107	-0.34063	-0.0537
MAP3K9	-0.19857	0.13482	-0.06791	-0.09072	-0.10996	-0.04756	0.33154	0.112815
MAP4	-0.23266	0.194325	0.054467	0.067777	0.426292	0.345913	-0.21883	-0.22255
MAP4K1	-0.19669	0.3891	0.348585	0.842731	-0.003	0.769096	-0.42934	0.243536
MAP4K2	-0.03856	0.707902	0.235559	0.198123	0.096231	0.027684	-0.0558	-0.18653
MAP4K3	0.025848	-0.62373	-0.1357	-0.41453	-0.04685	-0.23276	0.396697	0.32063
MAP4K4	-0.09901	-0.46465	-0.03699	0.45374	-0.05283	0.401408	0.175972	0.158904
MAP4K5	0.567781	-0.11973	-0.22085	-0.30758	-0.16745	0.143457	-0.63645	-0.07663
MAP6	0.225028	0.094396	0.121474	-0.15842	0.007833	0.132791	-0.41287	-0.30311
MAP6D1	-0.02733	0.37812	-0.25922	0.254081	-0.02519	-0.40283	-0.04842	0.281359
MAP7	0.292482	-0.03319	-0.50839	0.053234	0.154636	-0.28242	0.413933	0.080631
MAP7D1	-0.49672	-0.2446	0.396946	-0.04542	0.19009	0.266142	NA	NA
MAP7D2	0.210619	-0.16334	-0.05316	-0.1123	-0.00095	-0.02137	0.030909	0.503459
MAP7D3	NA	NA	-0.11438	0.080006	-0.32035	0.384755	NA	NA
MAP9	-0.12808	-0.43825	0.039448	0.005169	0.073213	0.151854	NA	NA
MAPK1	-0.08018	-0.0386	-0.02562	-0.02833	-0.17306	-0.05739	0.193863	-0.01196

MAPK10	-0.02467	-0.18668	0.213436	-0.01652	-0.16775	-0.13204	-0.24741	0.073417
MAPK11	-0.39081	-0.12014	0.184848	0.406024	-0.06795	0.228466	-0.32009	0.307935
MAPK12	-0.03304	-0.33733	0.332287	0.14727	0.039401	0.087621	-0.45136	-0.19719
MAPK13	0.245117	0.136796	0.132491	0.219464	0.12867	0.015015	0.393753	0.080094
MAPK14	0.248629	0.531258	-0.05069	-0.11066	-0.03803	-0.48495	0.6868	0.490266
MAPK15	-0.00196	0.083021	-0.10661	-0.15284	0.157869	0.094065	0.030435	-0.05553
MAPK1IP1L	NA	NA	-0.32105	-0.12651	-0.17974	0.029759	NA	NA
MAPK3	-0.28483	0.327019	0.326276	0.068421	0.071526	-0.26789	0.26629	0.04095
MAPK4	-0.14299	0.241075	-0.29253	-0.07189	0.153364	0.240848	-0.3943	-0.1655
MAPK6	0.049089	-0.0715	-0.41031	-0.2426	-0.06519	-0.51108	0.207119	0.200939
MAPK7	-0.16156	0.349247	0.119523	-0.05856	0.163531	-0.1271	0.131666	0.287922
MAPK8	-0.21508	0.802751	-0.31726	-0.31233	-0.10074	-0.09171	-0.14946	0.103772
MAPK8IP1	0.346728	0.348677	0.213404	0.091411	-0.18067	0.125653	-0.09595	-0.00823
MAPK8IP2	0.004258	0.644865	0.005935	-0.06671	0.152963	0.084936	0.051716	0.215736
MAPK8IP3	0.06586	0.331407	0.032983	-0.09587	-0.08578	0.013418	0.353894	0.463374
MAPK9	-0.07978	-0.72746	0.116716	0.163405	0.053951	-0.19247	0.392953	0.302994
MAPKAP1	0.359511	-0.4182	0.177532	0.285831	0.149588	-0.09816	0.147353	0.252845
MAPKAPK2	-0.24406	0.291561	0.32912	0.244785	0.198208	0.055788	-0.60292	-0.4514
MAPKAPK3	0.295878	0.593185	0.218421	0.360993	0.084151	-0.07673	-0.07805	-0.07573
MAPKAPK5	0.295134	-0.1183	-0.2174	0.050018	0.173777	-0.64235	-0.28504	-0.41108
MAPKBP1	0.201646	0.310228	0.240105	-0.11612	0.01433	0.381008	-0.12959	0.015267
MAPRE1	0.378923	-0.50332	0.01715	-0.17764	-0.00588	-0.19862	0.422475	-0.03304
MAPRE2	-0.4162	-0.38132	0.120649	-0.01552	-0.27808	-0.50122	0.219572	0.405644
MAPRE3	0.004724	0.574226	0.214885	-0.30141	-0.19187	-0.0452	0.103648	0.032603
MAPT	-0.07965	0.129749	0.224202	-0.05664	-0.06538	0.031309	-0.17273	-0.21209
MARCKS	-0.10684	-0.51946	-0.34391	0.096916	-0.03341	0.036249	-0.52825	-0.39885
MARCKSL1	-0.01796	-0.01346	0.162527	0.466991	0.148318	0.206642	0.623355	0.115416
MARCO	-0.52923	-0.28099	0.229116	0.076605	-0.10345	-0.08634	-0.18024	0.150311
MARK1	0.065237	-0.15933	0.250906	-0.01688	-0.05551	0.164578	0.044978	-0.0968
MARK2	-0.11565	-0.26473	0.224784	-0.11083	0.361671	0.325162	0.122193	-0.07916
MARK3	0.007795	-0.49399	-0.00923	-0.08578	0.023758	0.449631	0.319592	0.528211
MARK4	0.166781	0.159859	0.214344	-0.04089	0.181866	0.15445	-0.26119	-0.16132
MARS	0.020983	-0.14123	-0.20743	0.440835	0.297106	0.026593	-0.51916	-0.21408
MARS2	NA	NA	-0.18854	-0.02361	0.009503	0.032244	-0.24018	-0.20712
MARVELD2	0.275502	0.127569	0.04207	-0.05238	0.276502	-0.31044	0.323909	-0.00077
MARVELD3	-0.15205	0.148216	0.088125	-0.18506	0.303704	-0.23834	0.254672	0.137306
MAS1	-0.02559	0.037256	0.046595	-0.39265	0.285565	-0.01945	-0.21858	-0.0846
MAS1L	0.150297	0.134899	0.091756	-0.07669	-0.03476	-0.12844	-0.0441	0.348327
MASP1	0.036241	-0.09004	0.176235	0.012815	-0.1692	0.203339	-0.34095	-0.27077
MASP2	0.071428	-0.00815	0.178296	-0.11202	-0.24277	0.200177	0.377998	0.268347
MAST1	0.077513	-0.17879	-0.00167	-0.00845	0.065193	-0.28432	-0.3892	-0.15564
MAST2	-0.08239	0.332368	0.333849	-0.03118	0.444678	0.066954	-0.35903	-0.11387
MAST3	NA	NA	0.24467	0.171956	0.228873	0.011947	NA	NA
MAST4	NA	NA	0.12413	-0.25628	0.10195	0.431836	NA	NA
MASTL	-0.44141	-0.54197	-0.47866	0.080883	-0.26778	-0.21949	-0.11331	0.150984
MAT1A	0.068209	0.489067	-0.25662	0.006355	0.030665	-0.23656	-0.01387	0.191527
MAT2A	-0.01434	0.250799	-0.08957	-0.02515	0.266552	0.413907	0.442844	0.567915
MAT2B	0.144093	-0.25727	0.187919	0.121718	-0.10405	0.003377	-0.10779	-0.06285
MATK	0.149097	0.399606	0.335899	0.154315	-0.10373	0.570922	-0.69772	0.481308
MATN1	0.424514	0.803812	0.013191	0.231508	0.149962	0.155205	0.03042	0.215399
MATN2	-0.36938	-0.4906	-0.19377	-0.03407	-0.09328	0.166326	-0.12358	0.283518
MATN3	0.154914	0.615864	-0.09954	-0.01901	0.103059	0.201823	0.123788	-0.36717

MATN4	0.297002	0.596031	-0.30909	-0.01474	0.051194	-0.06621	0.703287	0.171723
MATR3	0.23146	-0.4281	-0.15121	-0.12722	-0.10995	-0.0093	0.174577	-0.08639
MAX	0.154244	0.272606	0.072438	-0.11061	-0.17712	-0.28871	-0.28471	-0.13945
MAZ	0.18983	0.569287	0.118927	0.26083	0.157056	-0.04059	0.508579	0.088327
MB	-0.14771	0.358213	-0.24037	0.069419	-0.05313	-0.0888	-0.06595	-0.12549
MBD1	-0.03215	0.488312	0.060804	0.135648	0.213926	-0.28107	0.206354	0.468427
MBD2	0.039591	0.609702	0.105056	0.074856	-0.07487	-0.05471	0.055101	0.15199
MBD3	-0.2309	0.341149	0.251327	-0.04919	0.148039	0.110076	-0.12993	0.078193
MBD3L1	0.616436	0.327096	-0.00752	-0.08325	-0.01758	0.097757	-0.59326	-0.29482
MBD3L2	NA	NA	0.037574	-0.19212	-0.30632	-0.15286	0.768746	0.33754
MBD4	-0.1097	-0.17942	-0.39885	0.206207	-0.33309	-0.02951	-0.46934	-0.28304
MBD5	0.254223	-0.33198	-0.07231	-0.19177	-0.22411	-0.29917	0.298331	0.031479
MBD6	-0.03985	0.277186	-0.1025	0.113159	0.214754	-0.00835	-0.04415	0.209073
MBIP	-0.09351	-0.65937	-0.32665	-0.4263	-0.39597	-0.11961	0.351556	0.331709
MBL2	-0.14381	0.109289	0.411658	-0.21074	0.239349	-0.14822	-0.11283	-0.13053
MBNL1	-0.1348	-0.08835	0.016119	-0.28541	0.009416	0.367779	-0.50738	-0.4281
MBNL2	0.082603	-0.3634	0.355147	-0.24263	0.367027	-0.28656	-0.02382	-0.23908
MBNL3	-0.13842	0.078783	-0.20687	-0.43699	-0.22298	-0.61383	0.286026	0.187126
MBOAT1	NA	NA	-0.37998	0.055718	-0.28852	-0.2806	-0.25814	0.022401
MBOAT2	NA	NA	-0.03152	-0.1723	-0.12188	-0.32059	-0.38527	-0.57175
MBOAT4	NA	NA	-0.1483	-0.13674	-0.15916	-0.02395	NA	NA
MBOAT7	NA	NA	0.197673	-0.13005	0.218114	0.069515	NA	NA
MBP	-0.2362	-0.1767	-0.04861	-0.08121	0.016713	-0.02882	0.069036	-0.13564
MBTD1	0.205586	0.096747	0.110107	-0.18149	0.025748	0.339968	-0.42347	-0.5091
MBTPS1	-0.30254	-0.72435	0.081403	0.229474	0.290846	-0.0952	0.360822	0.167401
MBTPS2	0.023806	0.302335	-0.08995	-0.13683	-0.05787	-0.29779	0.450341	0.585673
MC1R	-0.26931	0.503841	-0.15573	0.032127	0.154805	-0.36887	-0.3646	-0.1137
MC2R	0.580077	0.781731	0.090322	-0.39041	-0.22463	-0.04339	-0.28753	-0.46934
MC3R	0.38214	0.246813	-0.11346	-0.16905	0.183641	-0.0732	-0.17667	-0.00946
MC4R	-0.12996	0.139779	-0.08241	-0.15108	0.45155	0.059894	-0.17149	-0.23806
MC5R	-0.05927	0.256305	0.216601	-0.04618	0.421522	0.009973	-0.64284	-0.22442
MCAM	-0.44368	-0.1506	0.116856	0.015188	0.200613	0.273249	-0.61843	-0.46496
MCART1	-0.20626	0.420354	-0.20052	0.247898	-0.14651	-0.58179	-0.36463	0.342887
MCART2	NA	NA	-0.16127	-0.31739	-0.33821	-0.40921	0.066244	-0.15865
MCART6	-0.23544	-0.15682	NA	NA	NA	NA	-0.06622	0.317103
MCAT	0.138884	-0.57573	-0.0799	0.010317	0.132906	0.209417	NA	NA
MCC	-0.67921	-0.43832	0.091437	-0.07473	-0.03338	0.373728	-0.19474	0.171608
MCCC1	-0.05909	-0.42304	-0.09378	0.077883	0.115718	0.135729	0.516775	0.409814
MCCC2	0.091157	-0.52743	-0.18238	0.23653	-0.00134	-0.25566	0.488319	0.418238
MCCD1	-0.29899	0.132923	-0.25902	-0.2793	0.057544	-0.03402	-0.306	-0.18168
MCEE	-0.35336	-0.40561	-0.09215	-0.10516	-0.12859	-0.31278	0.186839	-0.06045
MCF2	0.265379	0.560961	0.096311	0.110782	0.086744	-0.04698	-0.12535	-0.14011
MCF2L	0.342071	0.160804	0.026124	0.237119	0.383674	0.064844	0.189827	0.414416
MCF2L2	-0.15869	-0.02506	-0.1852	-0.01728	0.095068	0.104905	0.455384	0.229197
MCFD2	0.164296	-0.69974	0.017886	0.026757	-0.08553	-0.58867	-0.4316	-0.04096
MCHR1	0.111981	0.782026	-0.12928	0.246405	0.170754	-0.09279	NA	NA
MCHR2	-0.29383	0.035722	0.154689	-0.15144	0.38364	-0.10977	-0.60885	-0.51723
MCL1	-0.28542	0.05322	-0.19726	-0.09357	-0.25416	0.174277	-0.14649	0.086909
MCM10	-0.30053	0.360176	-0.34235	0.199195	-0.03632	-0.1371	0.39467	0.322585
MCM2	0.003199	-0.28754	0.070517	0.324143	0.22009	0.198759	0.50114	0.36145
MCM3	0.103621	-0.53052	-0.35481	0.449532	0.130037	0.238543	0.458392	0.397671
MCM3AP	0.277115	0.492945	0.264288	0.314253	0.131711	0.564411	-0.42968	-0.29112

MCM3APAS	NA	NA	-0.21565	-0.2162	0.058482	0.446524	0.500102	0.24413
MCM4	-0.0932	-0.23879	-0.04996	0.296101	0.188655	0.141776	0.360921	0.143284
MCM5	0.064966	0.287566	0.077419	0.522613	0.476099	0.477589	0.569576	0.431101
MCM6	-0.06327	-0.17427	-0.20059	0.320142	0.139413	0.040139	0.336597	0.199884
MCM7	0.176015	0.428076	-0.08303	0.235579	0.350456	0.315287	0.43001	0.243641
MCM8	-0.24409	0.266192	-0.28145	0.177168	0.080982	-0.05886	-0.43549	-0.51742
MCM9	NA	NA	-0.29575	0.02472	0.244817	-0.21019	NA	NA
MCOLN1	-0.58582	-0.02923	0.297925	-0.14934	-0.01259	-0.04653	-0.33724	0.064273
MCOLN2	0.187967	-0.04409	0.438882	0.091485	-0.22988	-0.18174	-0.32463	-0.0215
MCOLN3	-0.01351	-0.44696	0.181367	0.109888	-0.16551	-0.07954	0.214526	0.689686
MCPH1	-0.11523	-0.04795	0.112783	-0.07943	-0.08507	-0.0911	0.091861	0.164841
MCRS1	0.367142	0.71594	0.059524	0.216641	0.08553	-0.04294	0.202696	0.296345
MCTP1	-0.06847	0.380835	0.116927	-0.26967	-0.23287	0.140169	-0.12085	0.340518
MCTP2	-0.22786	0.519051	-0.26519	-0.16798	-0.12524	-0.36888	0.416726	0.101323
MCTS1	0.064876	0.508089	-0.08911	-0.17058	0.225547	0.010911	-0.30422	-0.38453
MDC1	0.179134	-0.30698	-0.27572	0.41319	0.307763	0.256663	-0.13342	-0.48714
MDFI	-0.27434	0.688107	0.210748	0.116416	-0.06671	0.064459	0.031277	0.148255
MDFIC	-0.49883	-0.13735	-0.00521	0.169406	-0.45897	0.193582	-0.09602	0.179104
MDGA1	-0.10701	0.379295	0.068877	-0.10894	-0.05836	0.406692	-0.4512	-0.6061
MDH1	-0.24218	-0.56227	0.079199	-0.34096	-0.07379	-0.36673	0.262013	0.031266
MDH1B	0.095298	-0.05016	0.160555	-0.32257	-0.18383	0.124926	-0.54346	-0.4601
MDH2	0.279876	0.34753	0.160654	0.253635	0.048855	-0.09529	0.033404	-0.11252
MDK	0.147472	0.020686	0.021848	-0.07705	0.030779	-0.44886	0.032877	0.312194
MDM1	-0.19324	0.387897	-0.32389	-0.0477	-0.1275	-0.35786	-0.22206	-0.29224
MDM2	0.10643	0.631553	-0.19309	-0.13753	-0.24842	-0.15549	0.235032	0.069027
MDM4	0.004353	-0.39419	-0.23639	-0.06996	-0.28694	0.008042	0.326549	0.374491
MDN1	0.105514	-0.56058	-0.27912	0.214566	0.275255	0.167779	0.468803	0.229265
MDP-1	-0.01539	-0.18803	-0.0227	-0.2694	-0.09127	-0.20793	-0.43147	-0.35572
MDS1	0.230196	0.470193	-0.10289	-0.37091	0.065255	0.068425	0.073221	-0.38595
ME1	-0.06618	-0.11312	-0.17472	0.058783	-0.24957	-0.1496	-0.43216	-0.18604
ME2	-0.12975	-0.46673	0.047437	-0.27547	-0.29403	-0.29499	-0.1247	0.115487
ME3	0.266432	0.542721	-0.02417	-0.09451	0.189496	-0.43505	0.256799	0.198727
MEA1	0.165104	0.024071	0.086457	0.023197	-0.10956	-0.22251	0.542122	0.022567
MECP2	0.267733	-0.00572	-0.09429	0.027042	0.277124	0.270957	0.078207	-0.10891
MECR	-0.11763	-0.26356	0.230391	-0.01904	0.119287	-0.20082	0.222676	0.395848
MED1	NA	NA	0.179031	0.134822	0.060391	0.375454	NA	NA
MED10	0.059065	-0.063	-0.16367	-0.24866	-0.06454	-0.29552	NA	NA
MED11	-0.30668	-0.38899	-0.12125	-0.02631	-0.34197	-0.16308	NA	NA
MED12	0.485818	0.54187	0.11878	0.187213	0.26027	0.35977	0.261195	0.277385
MED12L	0.478813	0.437353	0.349234	0.359271	-0.03199	-0.04095	0.268785	0.310032
MED13	NA	NA	0.12517	-0.36083	0.136747	0.036972	NA	NA
MED13L	NA	NA	0.082548	0.123338	0.267773	0.310719	NA	NA
MED14	NA	NA	-0.1775	-0.20723	-0.16349	-0.02754	NA	NA
MED15	NA	NA	0.288596	-0.18817	0.435631	0.213522	NA	NA
MED16	NA	NA	0.184659	0.100943	0.256436	0.064113	NA	NA
MED17	NA	NA	-0.35322	-0.02257	0.075503	0.122073	NA	NA
MED18	-0.14666	0.003242	0.060078	0.046361	-0.18375	0.09298	-0.70617	-0.36677
MED19	-0.09182	-0.15007	-0.00713	-0.01614	-0.1803	-0.2722	0.215962	0.055309
MED20	NA	NA	-0.39308	0.016515	-0.06655	-0.24587	NA	NA
MED21	NA	NA	-0.20491	-0.29544	-0.37937	-0.4099	NA	NA
MED22	NA	NA	0.132118	0.006369	0.515795	0.237147	NA	NA
MED23	NA	NA	-0.22386	-0.19588	-0.0189	0.074127	NA	NA

MED24	NA	NA	0.48902	0.316562	0.228628	-0.00487	NA	NA
MED25	0.497109	0.723214	0.137762	-0.07088	0.139107	0.10657	0.601527	0.127532
MED26	NA	NA	-0.16359	0.195073	0.263843	-0.03685	NA	NA
MED27	NA	NA	-0.17902	0.034997	0.133412	0.344175	NA	NA
MED28	0.134228	0.622231	-0.24466	0.138851	-0.18999	-0.14126	0.342402	-0.15722
MED29	NA	NA	0.081781	0.05712	0.21353	0.018432	NA	NA
MED30	NA	NA	-0.02058	-0.20853	-0.11731	-0.39334	NA	NA
MED31	0.138726	0.630388	-0.09017	-0.30639	-0.41931	-0.35692	-0.01302	-0.16833
MED4	0.227258	0.207739	-0.23995	-0.25104	0.214842	-0.27259	0.592271	0.47329
MED6	0.30217	0.484866	-0.3595	0.006969	-0.31803	-0.3157	-0.05326	0.10765
MED7	NA	NA	0.134556	-0.50204	-0.1519	-0.44073	NA	NA
MED8	-0.45801	-0.5831	-0.11011	0.026675	0.037996	-0.59011	0.104323	0.090397
MED9	-0.15476	0.562839	0.026415	-0.16455	-0.19933	-0.27685	-0.25369	-0.52672
MEF2A	-0.41122	-0.39671	-0.11661	-0.22937	-0.38567	0.299864	-0.00973	0.285005
MEF2B	0.297385	0.836142	-0.03051	0.105617	0.017521	-0.38309	0.422231	0.2839
MEF2C	-0.05187	0.267918	0.131293	0.377354	-0.36269	0.283612	-0.58784	-0.32653
MEF2D	-0.21706	-0.00609	0.263295	-0.03853	0.091391	-0.06981	-0.27649	-0.48038
MEFV	-0.24444	0.054761	0.431845	-0.43853	0.028646	0.004073	-0.35187	0.307627
MEG3	NA	NA	0.030388	0.0895	-0.03443	0.192585	-0.33625	0.277261
MEGF10	0.072879	-0.32729	-0.01189	-0.25732	-0.02934	0.000121	0.097038	-0.28466
MEGF11	0.234072	0.45571	0.221466	0.067265	0.22133	0.031816	-0.31885	-0.26346
MEGF6	NA	NA	-0.15586	0.183152	-0.12659	0.416855	NA	NA
MEGF8	0.188494	-0.18179	0.007886	0.026312	0.091059	0.179786	0.00966	-0.15618
MEGF9	NA	NA	0.403526	-0.3025	0.064781	0.010253	-0.43556	-0.77819
MEI1	NA	NA	0.132834	0.133022	-0.2096	-0.17308	-0.24281	0.171421
MEIG1	NA	NA	-0.37632	0.239197	-0.22427	-0.50671	0.067999	0.042737
MEIS1	-0.21406	-0.26457	-0.06887	-0.09202	-0.10335	-0.05002	NA	NA
MEIS2	-0.39139	-0.22232	0.031193	-0.19794	-0.08423	-0.09323	-0.1818	-0.2643
MEIS3	0.056369	-0.07653	0.169598	-0.10942	0.245024	-0.10472	-0.43263	-0.25608
MEIS3P1	NA	NA	-0.06554	-0.09044	0.131235	0.051812	-0.31425	-0.77658
MELK	-0.00478	-0.13118	-0.29844	-0.0217	-0.04731	-0.21035	0.32307	0.241385
MEN1	0.211186	0.171502	0.364288	0.193525	0.221267	0.073977	0.59167	0.373526
MEOX1	-0.28518	-0.35129	0.254494	0.242342	-0.04402	0.362293	-0.31446	0.15875
MEOX2	-0.17908	-0.41849	0.197033	0.138132	-0.29343	-0.23176	-0.26407	-0.19715
MEP1A	0.467567	0.125325	0.156332	-0.36025	0.220856	-0.20837	0.160966	0.180581
MEP1B	-0.29259	0.36472	0.126996	-0.24953	0.037203	-0.3196	-0.37527	0.386162
MEPCE	NA	NA	0.133987	0.356912	0.473615	0.201665	NA	NA
MEPE	-0.08436	0.102438	0.038934	0.048831	-0.21064	-0.24371	0.257152	0.144677
MERTK	0.034786	-0.10442	0.186849	-0.17871	0.008147	0.302416	-0.16761	0.197492
MESDC1	-0.20586	0.150893	-0.18799	0.17856	0.080418	-0.04516	0.372701	0.440343
MESDC2	0.011003	-0.17356	-0.12554	0.533502	-0.07989	-0.25457	0.024099	-0.23894
MESP1	0.203043	0.443908	0.143036	-0.23576	0.191029	-0.14657	-0.47642	-0.33753
MEST	0.290258	0.561122	-0.21284	0.06065	-0.08651	-0.32873	0.309901	0.140802
MESTIT1	NA	NA	0.287074	0.013826	0.45428	0.15272	NA	NA
MET	0.447692	-0.30264	-0.27983	0.112159	-0.02896	0.033321	-0.27543	-0.4483
METAP1	-0.21625	-0.48962	-0.25538	0.154799	0.027965	0.049245	0.509237	0.249624
METAP2	0.097948	0.29173	-0.30577	-0.02208	0.123078	0.108072	-0.48703	-0.37527
METRN	-0.33739	-0.25371	0.124195	-0.08472	0.089841	-0.07613	0.091327	-0.23339
METRNL	-0.44618	-0.3966	-0.00875	-0.13267	0.063761	-0.13598	-0.40949	0.204039
METT10D	0.022475	0.388793	-0.17658	0.233401	0.041919	0.136194	-0.03083	-0.11634
METT11D1	0.040825	-0.26116	-0.21241	0.077424	0.103648	0.196709	-0.01463	0.399004
METT5D1	0.222844	-0.34828	-0.30277	-0.17663	-0.15738	-0.21068	-0.47981	-0.32356

METTL1	0.055883	0.334481	-0.12968	0.26603	0.197012	-0.12159	-0.17784	0.027187
METTL10	NA	NA	-0.28122	0.057443	0.083321	0.028279	NA	NA
METTL2A	-0.08159	-0.09709	-0.03685	-0.09587	-0.22821	-0.13588	0.090408	-0.02746
METTL2B	0.195577	-0.21526	0.027479	-0.01368	-0.11833	0.114378	-0.53797	-0.62973
METTL3	-0.07574	-0.70886	-0.28951	0.181659	0.194193	0.434263	0.466142	0.597414
METTL4	0.4271	-0.52387	-0.40175	-0.02125	-0.37415	-0.52196	0.44287	0.472358
METTL5	0.004687	-0.59227	0.17137	-0.35261	0.091604	-0.19401	0.163376	-0.188
METTL6	0.083943	-0.53121	-0.05688	-0.02222	-0.09277	-0.10358	-0.31146	-0.38302
METTL7A	-0.20075	-0.42726	-0.1934	-0.15165	-0.22919	-0.00416	-0.38178	-0.5318
METTL7B	0.318593	0.325601	0.318661	0.001365	-0.03656	-0.32959	0.513652	0.377291
METTL8	-0.12503	-0.16967	-0.19194	-0.06911	-0.40924	0.076041	NA	NA
METTL9	0.40499	-0.3108	0.029222	-0.14109	0.082639	-0.37539	-0.04167	-0.19988
MEX3A	NA	NA	-0.12221	0.119247	-0.06867	0.056366	NA	NA
MEX3B	NA	NA	-0.0845	0.010481	-0.15765	-0.03537	NA	NA
MEX3C	NA	NA	-0.35644	-0.06242	-0.09264	-0.13111	NA	NA
MEX3D	NA	NA	0.006187	-0.04852	0.029678	0.128022	NA	NA
MFAP1	-0.02473	-0.64235	-0.14563	-0.26536	-0.39127	-0.44305	-0.40849	-0.20567
MFAP2	-0.21937	-0.22582	0.216103	0.075196	-0.08496	-0.09346	0.262761	0.103599
MFAP3	-0.09381	-0.36376	0.110004	-0.36279	-0.18356	-0.45823	0.159591	0.456261
MFAP3L	0.125376	0.434622	0.03145	0.017823	-0.15934	-0.36903	-0.01134	0.024767
MFAP4	-0.00615	-0.12057	0.037775	0.152685	-0.09386	0.349213	-0.14533	-0.22192
MFAP5	-0.11949	-0.21687	0.209125	-0.14773	-0.24388	-0.3158	-0.38039	-0.06297
MFF	NA	NA	0.053265	-0.45226	0.058188	-0.28882	NA	NA
MFGE8	0.382916	-0.07186	0.065879	0.065898	0.142576	0.146694	-0.14495	-0.37096
MFHAS1	-0.03259	-0.58232	0.345735	0.059938	0.10723	0.249871	0.05546	-0.25194
MFI2	-0.22399	-0.00181	0.092277	-0.24402	0.055716	-0.36215	0.512008	0.39844
MFN1	0.124556	0.730299	-0.13215	-0.32317	-0.10138	-0.24979	-0.16718	-0.06472
MFN2	-0.16478	0.20136	-0.07089	-0.09723	0.351274	-0.02991	-0.19726	-0.08559
MFNG	-0.17119	0.337703	0.28208	0.672791	-0.00658	0.574492	-0.55306	-0.11605
MFRP	0.16446	0.050242	0.01032	-0.20801	0.126091	-0.3379	-0.23195	-0.09473
MFSD1	-0.383	-0.21774	-0.06578	-0.17169	-0.21848	-0.12901	-0.70983	-0.39291
MFSD10	NA	NA	0.331134	0.259881	0.179383	0.003486	NA	NA
MFSD11	0.14788	-0.38505	0.248314	-0.20905	-0.34885	-0.20665	NA	NA
MFSD2	-0.34689	0.4372	0.17563	-0.06009	0.172734	0.108656	-0.1407	0.511583
MFSD3	-0.06606	0.41791	0.282873	0.101263	-0.11525	-0.32532	0.367344	0.051521
MFSD4	0.085954	-0.20143	-0.20006	-0.16845	-0.2367	-0.3387	-0.19166	-0.16667
MFSD5	0.193076	0.253334	0.132236	0.143024	0.22608	-0.00105	0.48172	0.26478
MFSD6	NA	NA	-0.09014	0.077758	-0.0175	-0.29363	NA	NA
MFSD6L	NA	NA	0.00125	0.150067	-0.28436	-0.19344	NA	NA
MFSD7	0.12681	0.361448	0.099802	-0.06249	-0.45637	-0.20718	-0.04423	0.391997
MFSD8	0.174564	0.58165	-0.34215	-0.18648	-0.02291	0.158221	0.28634	0.531894
MFSD9	-0.13686	0.54565	-0.03805	-0.0729	-0.21236	-0.17197	NA	NA
MGA	NA	NA	0.187062	0.149542	0.255362	0.416442	-0.4399	-0.4211
MGAM	-0.26069	0.314912	0.153207	0.174402	0.040667	0.001291	-0.28134	0.445761
MGAT1	-0.30285	0.151266	0.459667	0.204857	0.074906	0.309749	-0.08484	0.293587
MGAT2	-0.38041	0.274239	-0.34757	-0.04816	-0.24779	-0.4864	-0.26657	-0.28897
MGAT3	-0.09231	0.475426	0.171007	0.205455	0.036885	-0.0385	-0.04929	0.074635
MGAT4A	0.057122	0.304541	-0.12889	-0.30526	-0.19174	-0.6206	-0.16724	0.248467
MGAT4B	0.506159	0.05568	0.373964	0.046384	0.387626	0.037828	0.545134	0.434939
MGAT4C	0.071221	-0.05406	-0.03596	-0.16885	0.239384	0.31452	-0.21046	-0.36214
MGAT5	0.579513	0.071887	0.251584	-0.33824	-0.00844	0.022041	0.285816	0.100512
MGAT5B	0.251347	0.673352	-0.32586	0.231773	-0.1334	0.388957	-0.45269	-0.41719

MGC13057	-0.42869	0.187926	-0.03379	-0.33503	-0.2346	-0.57165	-0.16738	-0.08888
MGC16169	0.021113	-0.70632	0.136651	-0.19158	0.168901	0.121304	0.633893	0.262949
MGC16291	0.010073	0.34278	-0.00739	-0.05827	-0.00266	0.125515	0.139786	0.391825
MGC16703	0.041264	-0.34236	0.135572	0.032725	0.035016	0.027775	0.519152	0.240012
MGC23270	NA	NA	-0.22803	-0.06759	0.349124	0.212767	NA	NA
MGC24975	0.108251	0.110467	-0.39421	0.042834	0.011758	0.178427	0.133452	0.143705
MGC26718	0.445338	-0.13036	-0.00366	0.035998	-0.0798	0.244242	0.065154	-0.10468
MGC27345	NA	NA	-0.141	0.080159	0.213749	0.335068	NA	NA
MGC2752	0.436676	-0.03661	0.25219	0.020496	0.069775	-0.01223	0.15235	0.069888
MGC29506	NA	NA	0.245793	0.385923	-0.13238	-0.13112	-0.15536	0.100821
MGC3207	0.205002	0.244206	-0.35517	0.125805	0.1653	-0.05955	0.278282	0.524145
MGC33407	0.328427	0.745884	0.078998	-0.13415	-0.01005	0.147461	-0.1206	0.018084
MGC33894	0.095491	0.162953	0.230134	0.016106	0.067126	-0.1422	0.120479	0.170658
MGC34774	-0.2645	-0.03342	0.18388	-0.021	-0.16154	0.077023	0.40575	-0.13838
MGC39372	NA	NA	0.075988	0.173228	-0.15116	0.320858	NA	NA
MGC39545	-0.37145	0.678152	-0.22952	0.091922	-0.01519	-0.24887	NA	NA
MGC42105	0.082474	-0.26143	-0.24769	-0.141	-0.19302	-0.11485	-0.62612	-0.37732
MGC45800	-0.33308	0.061328	NA	NA	NA	NA	0.182437	0.148052
MGC45922	NA	NA	0.067896	-0.04759	-0.1108	0.18762	NA	NA
MGC50722	0.251724	0.712073	-0.17627	-0.14091	-0.11948	-0.26803	0.476733	0.404106
MGC70863	-0.46476	-0.14719	-0.10211	-0.14815	-0.33625	-0.14967	0.312959	0.182206
MGC70870	0.102174	0.387759	-0.33981	0.270212	-0.11394	-0.06077	0.151272	0.347385
MGC87631	-0.05556	-0.00337	0.070133	-0.13371	-0.03485	0.121447	0.390461	0.323636
MGEA5	-0.0993	-0.55176	-0.25192	0.014975	0.231321	0.186949	-0.19598	0.113892
MGLL	-0.29027	-0.21447	0.108657	-0.09724	0.154452	0.166028	0.021381	0.007472
MGMT	-0.10438	0.107261	0.149132	0.015866	0.242433	0.011981	-0.31169	-0.36748
MGP	-0.20118	-0.50335	0.31528	0.047472	-0.04934	-0.21648	-0.18023	-0.24945
MGRN1	0.460566	0.249121	-0.23021	0.123449	0.204079	0.282557	0.380193	0.254126
MGST1	0.071347	-0.0791	-0.30708	-0.20305	-0.37373	-0.19052	0.03978	0.083551
MGST2	0.04015	-0.00871	0.172625	-0.05981	-0.21513	-0.1322	0.206767	0.17513
MGST3	0.440145	0.13112	-0.24878	-0.45294	-0.48082	-0.37084	0.26004	0.327385
MIA	-0.20468	0.266407	-0.19533	0.389478	-0.41202	0.25967	-0.30127	-0.31814
MIA2	-0.25966	-0.14485	0.17943	-0.12098	-0.02901	-0.10935	0.140306	0.206172
MIA3	NA	NA	-0.33115	-0.06026	-0.17999	-0.43122	0.265174	0.485959
MIB1	-0.01537	-0.41082	-0.03688	-0.25351	-0.2647	-0.00553	0.238963	0.176278
MIB2	-0.35131	0.216176	0.229883	-0.10126	0.077062	0.153379	-0.05538	0.245084
MICA	-0.08588	0.135575	-0.00549	-0.36939	-0.16771	-0.04337	-0.2902	-0.01172
MICAL1	0.294088	0.353093	0.222808	0.430926	0.393372	0.213503	0.338338	0.511832
MICAL2	0.010056	-0.53247	-0.0688	-0.28771	0.152138	0.120422	-0.47934	-0.23192
MICAL3	NA	NA	-0.03127	-0.11468	0.347574	0.42303	NA	NA
MICALCL	0.24688	-0.13704	0.165155	-0.24625	0.201121	-0.29638	0.301842	0.086899
MICALL1	0.300814	0.407552	0.269567	-0.05915	0.488943	-0.04709	NA	NA
MICALL2	0.148736	0.139801	0.018784	0.475764	0.060342	0.092761	NA	NA
MICB	0.120739	0.397076	-0.0731	0.008086	-0.23955	0.236256	-0.34196	0.061195
MID1	-0.12578	0.102441	0.109854	-0.26301	0.192163	-0.24816	-0.07811	-0.19909
MID1IP1	0.033054	-0.28409	0.023337	0.307991	-0.02421	0.144657	-0.54343	-0.35523
MID2	-0.02653	-0.13628	-0.00765	0.135328	-0.17439	-0.20469	-0.3116	0.01294
MIDN	0.00412	0.528678	-0.10842	0.078288	0.382658	0.017612	-0.3476	-0.1425
MIER1	-0.34934	0.097828	0.073797	-0.43033	-0.27376	-0.23435	-0.40298	-0.15869
MIER2	0.130185	0.462244	0.197493	0.089738	0.091448	-0.03629	NA	NA
MIER3	-0.41783	0.068014	-0.32876	-0.39313	-0.11493	-0.18809	NA	NA
MIF	-0.06692	0.545938	-0.07893	0.148672	0.246256	0.016095	-0.08846	-0.24664

MIF4GD	-0.26499	0.280283	0.314486	0.051898	0.063567	-0.23072	-0.50177	-0.5135
MINA	0.188009	-0.34938	-0.24784	0.134741	0.0178	-0.05243	-0.22575	-0.35183
MINK1	-0.16253	0.167535	-0.10582	0.218817	-0.05077	-0.05406	-0.10736	-0.15202
MINPP1	0.212438	-0.426	-0.00788	-0.09777	-0.0473	-0.19749	-0.06603	-0.23458
MIOX	-0.07432	-0.05486	0.132204	0.030058	0.152087	-0.09602	0.248213	-0.06685
MIP	0.018991	0.577911	-0.23496	-0.1998	-0.07226	-0.34478	0.093623	-0.03358
MIPEP	0.451854	-0.36142	0.392736	0.044906	0.28949	-0.24157	0.488477	0.085371
MIPOL1	0.330453	0.012864	-0.13922	-0.09603	-0.3351	0.148011	0.005956	0.025338
MIS12	-0.30087	-0.12062	-0.35647	-0.08134	-0.37785	-0.15769	-0.00541	0.109594
MITD1	0.195809	-0.746	-0.08478	0.037216	-0.03886	-0.08548	NA	NA
MITF	-0.17694	-0.14758	0.081241	-0.30539	-0.31339	0.070058	-0.36503	-0.38356
MIXL1	-0.01476	0.39775	0.096046	-0.09267	-0.20541	-0.2798	0.235838	-0.18644
MIZF	-0.00918	-0.07643	-0.03092	-0.11557	-0.10346	-0.14244	0.451831	0.293648
MKI67	0.153441	0.190651	-0.3187	0.170536	0.313083	0.133387	0.43064	0.23352
MKI67IP	0.173867	-0.33821	-0.12602	-0.08012	0.129108	0.273519	0.438903	0.020352
MKKS	0.205249	-0.35531	-0.34641	-0.28747	-0.28316	-0.40361	-0.11061	-0.27556
MKL1	-0.14814	0.239292	0.121596	0.211095	0.210969	0.286594	0.106811	0.126633
MKL2	0.061838	-0.74937	-0.25531	-0.10732	0.190928	-0.14572	0.094581	-0.22289
MKLN1	0.236039	-0.51164	-0.00748	-0.32225	-0.07786	0.072832	0.476866	0.393773
MKNK1	0.14723	-0.07209	0.434289	-0.2701	0.148447	0.421267	0.185768	0.54884
MKNK2	0.147054	-0.28156	-0.01074	0.069053	0.149803	-0.11381	-0.37563	-0.42733
MKRN1	-0.09033	0.09995	-0.07182	-0.1522	0.153666	-0.2342	0.222397	0.14352
MKRN2	0.360707	0.027689	-0.17937	0.064121	-0.17124	-0.07693	0.669087	0.242151
MKRN3	-0.10235	-0.11648	-0.29922	0.111958	0.259766	-0.33401	0.053532	-0.37285
MKS1	0.299479	0.366268	0.17103	0.269958	0.230681	-0.02081	0.692112	0.37817
MKX	0.154226	-0.25255	-0.28794	-0.23503	0.067113	-0.19668	NA	NA
MLANA	0.142587	0.432586	0.115378	-0.10434	-0.07979	-0.21926	0.335104	0.469988
MLC1	0.623355	0.542684	-0.00311	0.492678	0.028344	0.133389	-0.1643	-0.21823
MLEC	NA	NA	0.112704	0.26537	0.252897	0.056962	NA	NA
MLF1	-0.30564	-0.25288	-0.1012	-0.04914	-0.32471	0.011715	-0.0538	-0.11755
MLF1IP	-1.3E-05	0.32943	-0.2079	0.129076	0.128572	0.034411	-0.02832	-0.0706
MLF2	0.487855	0.21609	0.123125	0.225262	0.029225	0.196635	0.30747	0.307392
MLH1	-0.1037	-0.31195	-0.3478	-0.00511	0.111475	0.084613	-0.15825	-0.10391
MLH3	0.044076	-0.23002	-0.40231	-0.09364	-0.05785	-0.04237	0.05222	-0.05001
MLKL	0.03688	0.154655	-0.1618	0.079139	-0.12524	0.154792	-0.20843	-0.09212
MLL	0.047279	-0.51971	0.065702	0.008671	0.002343	0.421537	-0.0807	0.184127
MLL2	0.36887	0.199555	0.03517	-0.16913	0.140191	0.097287	-0.53722	-0.19813
MLL3	-0.08564	-0.17659	0.053849	-0.05447	0.177104	0.449648	0.019616	0.065168
MLL4	0.181026	0.54849	0.049796	0.283261	0.241257	0.20147	0.376182	0.397197
MLL5	-0.0022	-0.56571	0.131293	-0.06669	0.072175	0.686996	0.114512	-0.11071
MLLT1	0.252479	-0.0616	0.283603	-0.03341	0.289328	0.075651	0.34836	-0.32387
MLLT10	-0.25943	-0.4811	0.340603	-0.26262	0.179532	0.38189	0.012884	-0.023
MLLT11	0.114268	-0.12902	0.243579	-0.0388	-0.13646	-0.08234	-0.04536	-0.15808
MLLT3	0.212966	0.618019	-0.21586	-0.09745	-0.25038	-0.53086	-0.08832	-0.34384
MLLT4	0.130757	0.758998	-0.2664	-0.20596	0.353788	0.047472	NA	NA
MLLT6	0.365965	-0.07546	0.530893	0.249958	0.450096	0.568411	0.146762	0.175144
MLN	-0.07832	0.566039	0.099288	-0.00428	0.147896	-0.03755	-0.05722	-0.12195
MLNR	0.353977	0.208322	-0.24027	0.123604	-0.05851	0.106775	0.380648	0.300686
MLPH	-0.377	0.120456	-0.12799	0.168871	-0.01264	-0.20605	0.103289	0.067109
MLX	-0.14205	0.078967	0.277261	-0.10154	-0.01521	-0.4642	0.12971	0.057143
MLXIP	0.140253	0.378943	0.12154	-0.0664	0.314921	0.090315	-0.33237	-0.14884
MLXIPL	0.439069	0.050149	0.313227	0.095433	0.325361	0.28548	0.151447	0.452906

MLYCD	0.22438	-0.099	0.019141	0.134566	0.306048	-0.22255	0.135697	-0.20743
MMAA	-0.28686	0.513639	-0.10425	-0.16413	-0.33437	-0.07372	-0.38118	-0.3667
MMAB	-0.05499	0.107793	-0.12804	0.013304	0.18834	0.082392	0.126794	0.330926
MMACHC	0.221783	0.46588	0.158502	-0.14057	0.149384	-0.08554	0.30457	0.042212
MMADHC	NA	NA	-0.17779	-0.18285	-0.13348	-0.34478	NA	NA
MMD	0.281566	-0.57896	-0.19392	0.00615	-0.12066	-0.10774	-0.11031	0.162839
MMD2	-0.10514	0.723279	-0.18757	0.009148	-0.10641	-0.09406	0.261892	0.028484
MME	-0.28622	0.362616	0.321284	0.055841	0.127073	0.238593	0.467289	0.586797
MMEL1	0.135584	0.094669	0.149827	-0.11996	-0.08708	-0.34533	-0.16786	-0.26903
MMGT1	NA	NA	0.002504	-0.25944	0.158593	0.102206	NA	NA
MMP1	0.003636	-0.01048	-0.09995	-0.05701	0.011579	-0.04962	-0.32304	-0.18708
MMP10	-0.08664	0.198713	-0.08815	0.207469	0.084595	0.164516	-0.05222	0.131767
MMP11	-0.03887	0.200014	-0.02447	-0.10144	0.015032	0.147915	0.355164	0.261817
MMP12	-0.09427	0.162288	-0.11944	0.181227	-0.27549	0.385613	-0.11413	-0.09444
MMP13	-0.43494	0.042449	0.142063	-0.00379	0.021473	-0.16298	0.120078	0.254865
MMP14	-0.46129	-0.25775	0.215343	0.133242	-0.02767	0.104759	-0.28548	-0.29695
MMP15	0.048901	0.223975	0.265935	-0.04123	0.431881	0.087156	0.437673	0.109621
MMP16	0.144433	0.138779	0.135298	-0.15662	-0.23545	0.180629	-0.02034	-0.45446
MMP17	0.092907	0.583282	0.149324	-0.0395	-0.23098	0.161689	0.087271	-0.16497
MMP19	-0.16771	-0.00258	0.047265	0.431119	-0.30541	0.250808	-0.08526	0.18891
MMP2	-0.27415	-0.19645	0.119987	0.055331	0.072939	0.114852	-0.17912	0.138446
MMP20	-0.0757	0.527097	-0.07074	0.176409	-0.34906	0.241639	-0.11462	-0.13628
MMP21	-0.15182	-0.28798	-0.33977	-0.16206	0.010186	0.291215	0.30256	0.172029
MMP23A	-0.23235	0.027415	NA	NA	NA	NA	-0.42715	-0.03007
MMP23B	-0.19542	-0.46497	-0.01559	0.159292	-0.45617	0.040093	-0.41129	-0.08985
MMP24	0.035349	0.041112	0.120969	-0.18644	0.01127	-0.12117	0.29283	0.065061
MMP25	-0.50784	0.342354	0.255926	-0.00384	0.167502	0.281963	-0.36021	0.339657
MMP26	0.276586	0.390831	-0.10922	0.063415	-0.09584	-0.05694	0.356819	-0.17684
MMP27	0.038847	0.069714	0.007704	-0.04201	-0.0667	0.089067	0.162822	0.245596
MMP28	-0.12478	0.570645	0.019295	-0.24447	-0.17032	-0.41835	-0.2066	-0.27443
MMP3	0.192283	0.125905	-0.02293	0.122985	0.109151	0.113185	-0.30478	-0.13986
MMP7	0.099819	0.049926	-0.24025	0.331909	-0.03511	0.25687	0.082097	-0.03259
MMP8	0.013748	0.121246	0.322844	-0.03967	-0.05932	0.340122	-0.10578	-0.04056
MMP9	-0.22709	0.325469	0.16947	0.693616	-0.14262	0.666207	0.007542	0.20839
MMRN1	-0.29125	-0.01153	-0.16598	0.353104	-0.34217	0.135404	0.072446	-0.08372
MMRN2	-0.34777	-0.36057	0.251239	0.14225	-0.04131	0.044842	-0.57718	-0.4127
MMS19	NA	NA	0.185847	0.115884	0.312383	0.126274	NA	NA
MN1	0.301248	-0.28627	-0.05394	0.107728	-0.09941	-0.11206	-0.09123	-0.35053
MNAT1	0.149352	-0.51751	-0.38296	-0.10574	-0.20217	-0.36426	-0.48451	-0.42744
MND1	-0.165	-0.27922	-0.27981	0.208268	0.164262	-0.28155	0.148446	-0.0832
MNDA	-0.46005	-0.21057	0.002584	-0.07807	-0.02243	0.065357	-0.40571	0.382682
MNS1	-0.0138	-0.51338	0.015219	0.282865	0.026089	-0.21893	-0.0251	0.042542
MNT	0.119275	0.462149	0.145435	-0.09074	0.067882	0.29005	-0.31715	-0.02562
MNX1	NA	NA	-0.06706	0.25637	0.314961	-0.07053	NA	NA
MOAP1	0.022183	-0.48289	0.06902	-0.18171	0.173494	0.058211	0.136041	0.031756
MOBKL1A	0.026783	-0.67328	0.076165	-0.03603	-0.02691	0.103514	-0.51333	-0.51193
MOBKL2A	-0.13028	0.632251	0.112469	0.402905	-0.02535	0.361483	-0.64425	-0.41998
MOBKL2B	-0.12489	-0.34687	-0.34302	-0.27687	-0.14287	-0.04433	0.080417	0.387362
MOBKL2C	-0.45681	-0.08081	0.410333	-0.15883	0.214374	0.115357	-0.31417	0.209101
MOBP	0.044218	0.36784	0.245388	-0.14259	0.142403	0.066923	-0.22597	0.026322
MOCOS	-0.05898	0.290048	-0.05573	0.173402	0.019806	-0.37129	0.057072	0.408387
MOCS1	0.397868	0.090351	0.488892	-0.25519	0.134565	0.048542	-0.36578	-0.24841

MOCS2	-0.2084	-0.39818	-0.02419	0.028185	-0.24472	-0.26602	-0.24588	-0.29468
MOCS3	0.503246	-0.27132	-0.06132	0.104749	0.309095	-0.16525	0.467398	0.459166
MOG	0.060984	-0.1165	0.186403	0.214454	-0.02562	-0.05094	0.524815	0.547518
MOGAT1	0.272064	-0.08509	-0.41936	-0.26106	-0.13163	-0.45587	-0.04837	0.090852
MOGAT2	-0.17166	-0.02014	-0.18477	-0.06483	-0.25511	-0.56026	0.003165	0.229727
MOGAT3	0.573624	0.36031	0.25201	-0.11545	0.278632	-0.44039	0.337214	0.312082
MON1A	0.053022	-0.14903	0.138349	0.071267	0.161385	-0.00342	-0.37214	0.215797
MON1B	0.3835	0.038164	-0.31021	0.096494	0.168658	-0.18359	0.512279	0.384628
MON2	-0.36563	-0.58511	-0.23497	-0.26605	-0.00603	0.13027	-0.13293	0.255151
MORC1	0.054195	0.584857	-0.28512	-0.25795	0.274798	-0.18826	-0.34442	-0.33707
MORC2	0.085843	0.093577	-0.01808	0.228275	0.284985	0.275774	0.611517	0.014491
MORC3	-0.40071	-0.47942	-0.15529	-0.34097	-0.09533	0.350935	0.241532	0.264243
MORC4	-0.17262	-0.00516	0.007265	0.006378	0.08451	-0.31986	-0.14335	-0.16843
MORF4	0.125085	0.221767	-0.25586	0.080975	-0.37003	-0.22105	-0.32182	-0.19264
MORF4L1	-0.25639	-0.65734	-0.1336	0.034717	-0.25004	-0.01133	-0.23906	-0.25827
MORF4L2	-0.00958	-0.58461	-0.11033	0.050992	-0.1479	-0.1276	NA	NA
MORG1	0.024522	-0.31341	0.116013	0.162909	0.23072	0.072106	0.027129	0.031831
MORN1	-0.10804	0.481183	0.040999	0.098422	0.158095	-0.24759	-0.10037	0.114035
MORN2	-0.03911	-0.04129	NA	NA	NA	NA	NA	NA
MORN3	0.216046	0.029618	-0.06573	0.160393	-0.14196	0.14877	0.360999	-0.15571
MORN4	NA	NA	-0.03293	-0.23091	0.096747	-0.4815	NA	NA
MORN5	NA	NA	0.030892	-0.19009	0.158242	-0.22875	NA	NA
MOS	0.165685	0.717428	0.205008	-0.16041	0.109704	-0.17089	-0.32549	-0.53152
MOSC1	-0.13473	-0.11073	-0.2066	0.344192	-0.09614	-0.35207	0.482504	0.145991
MOSC2	-0.07133	-0.51623	0.015681	-0.10249	-0.3078	-0.22276	-0.11447	0.304442
MOSPD1	0.289391	-0.03403	-0.1375	-0.49052	-0.06015	-0.04272	NA	NA
MOSPD2	0.208684	-0.11417	-0.03195	-0.06408	-0.06635	-0.42084	-0.43983	-0.3293
MOSPD3	0.294658	0.776222	0.261139	0.080355	0.08579	0.045108	0.748455	0.17562
MOV10	0.128715	0.377115	0.365557	-0.16536	0.399061	0.163558	0.139571	0.373786
MOV10L1	0.462556	-0.22523	-0.08606	-0.13699	0.010687	-0.01067	-0.14878	-0.26016
MOXD1	0.008687	0.050269	0.047936	0.05104	-0.02927	0.298471	-0.11151	0.060894
MPDU1	-0.26138	0.47422	0.025828	0.23897	-0.17792	-0.30527	0.117177	0.204793
MPDZ	-0.17728	-0.328	-0.15795	-0.04507	-0.14004	-0.02159	-0.33462	-0.23269
MPEG1	NA	NA	0.080985	-0.05209	-0.26031	0.382361	NA	NA
MPG	0.109183	0.506397	0.196296	0.060365	0.161695	0.020911	0.385897	-0.07714
MPHOSPH1	-0.07406	-0.34002	0.003532	0.24984	0.059985	0.277043	-0.424	-0.32522
MPHOSPH6	-0.00976	-0.60855	-0.27191	-0.03154	-0.03882	-0.44619	-0.49051	-0.40836
MPHOSPH8	NA	NA	0.224448	0.074653	0.205372	0.253174	NA	NA
MPHOSPH9	-0.19039	-0.45118	-0.05582	0.076126	0.096025	0.03215	NA	NA
MPI	0.044321	0.514069	0.086789	-0.14884	-0.1817	-0.1062	0.117167	0.406386
MPL	-0.00694	0.256665	-0.29554	-0.33245	-0.09051	0.074526	-0.21948	-0.39147
MPN2	0.146469	0.162551	-0.04348	-0.16659	-0.13313	0.037629	-0.31813	-0.23916
MPND	0.156703	0.56627	0.348168	-0.05618	0.200742	-0.06271	0.577238	0.155725
MPO	-0.05008	-0.27007	-0.19104	0.03221	-0.17129	0.134689	-0.16251	0.391417
MPP1	-0.06214	-0.35029	0.133264	-0.20375	0.153681	-0.14631	-0.30173	0.397533
MPP2	0.406123	0.173814	0.017505	-0.11992	-0.11342	0.085676	-0.41212	-0.31456
MPP3	-0.02479	-0.39172	-0.06745	0.142093	0.029697	0.062982	0.199046	0.129708
MPP4	-0.00452	0.383215	-0.21867	-0.19801	-0.00366	0.176432	0.388716	0.561016
MPP5	0.139104	-0.07488	-0.28084	-0.51741	-0.26642	-0.26312	0.430101	0.307472
MPP6	0.14521	-0.5209	-0.37872	0.153157	-0.03643	-0.01581	0.236636	0.521408
MPP7	0.070638	-0.11889	0.018111	-0.41572	-0.13554	0.034152	-0.07599	0.129942
MPPE1	0.00887	0.052215	-0.01251	-0.28602	-0.21952	-0.47299	-0.46752	0.079564

MPPED1	0.041892	-0.28232	-0.13279	-0.10939	0.166731	0.12267	0.129564	-0.02562
MPPED2	-0.0405	0.12544	0.21824	-0.05344	-0.15107	0.000105	-0.17681	-0.23712
MPRIP	NA	NA	0.126482	0.124447	0.098647	0.247579	NA	NA
MPST	0.167542	0.379587	0.438902	0.097549	0.501666	0.102933	0.232692	-0.12246
MPV17	0.274087	0.046728	0.383251	0.245793	0.168351	-0.23987	0.249275	0.129549
MPV17L	0.066436	-0.10463	0.056003	0.009869	-0.03639	0.018015	NA	NA
MPZ	0.04483	-0.20676	-0.08539	-0.19225	0.037397	0.047441	0.002166	0.144306
MPZL1	0.084379	-0.24316	-0.38698	0.27503	-0.20372	-0.39132	0.260907	0.212272
MPZL2	NA	NA	-0.06134	-0.19566	-0.31715	-0.27852	NA	NA
MPZL3	NA	NA	-0.00238	-0.34943	-0.10633	-0.19458	NA	NA
MR1	-0.1673	0.009078	0.122038	0.077816	-0.31164	0.238205	-0.00719	0.326135
MRAP	0.45895	-0.10862	0.125287	-0.10488	-0.11906	-0.01375	-0.29769	0.225078
MRAP2	NA	NA	-0.18368	0.287928	0.015262	-0.49324	NA	NA
MRAS	-0.50646	-0.4466	0.168476	-0.05687	-0.33439	0.365284	-0.33079	0.047418
MRC1	-0.01267	0.151074	NA	NA	NA	NA	-0.07503	0.001098
MRC1L1	-0.3357	0.061701	0.089709	-0.47751	-0.55732	0.106317	-0.11975	-0.16952
MRC2	-0.25954	-0.28909	0.163184	-0.14879	0.080455	0.233476	-0.1605	0.120601
MRCL3	0.385421	0.485101	-0.12227	-0.34907	-0.3338	-0.30885	-0.34115	-0.15372
MRE11A	-0.21789	-0.54664	-0.36262	0.069629	0.11299	-0.01438	0.488138	0.221637
MREG	-0.23059	-0.06531	-0.23275	-0.01421	-0.35936	0.005075	NA	NA
MRFAP1	-0.45939	-0.60815	0.010789	0.209789	0.194281	0.102489	0.202358	0.084377
MRFAP1L1	-0.15334	-0.38387	-0.04332	0.227466	0.131527	0.006366	0.063738	-0.22225
MRGPRD	-0.06994	0.023002	0.208422	0.143066	-0.30732	0.145042	-0.47131	0.127631
MRGPRE	NA	NA	0.191921	-0.22938	0.103149	-0.21848	0.177748	-0.05751
MRGPRF	-0.03318	-0.14763	0.200877	-0.04492	-0.02735	0.204513	-0.17204	-0.23485
MRGPRX1	0.108425	0.359835	0.1222	0.187409	0.366307	0.120458	-0.01849	0.211538
MRGPRX2	0.115959	0.233125	-0.14882	-0.08075	0.065643	-0.16651	0.165842	0.501679
MRGPRX3	-0.21853	-0.29892	-0.10288	0.058596	-0.00698	-0.16063	-0.31977	-0.25995
MRGPRX4	0.230708	0.536444	-0.06607	0.326359	-0.19129	0.072262	-0.36661	-0.35975
MRLC2	0.137058	0.02546	-0.10435	-0.27208	-0.12329	-0.18791	0.055232	0.181264
MRM1	0.269721	0.166894	0.074001	0.243274	0.171407	-0.05988	0.301651	0.116525
MRO	0.125397	0.143458	-0.03162	-0.17257	0.10636	-0.04398	0.350589	0.586403
MRP63	-0.28051	-0.33691	0.157256	0.372027	0.190317	-0.30166	-0.55344	-0.60914
MRPL1	-0.07392	-0.15623	-0.12595	-0.17493	-0.18331	0.158411	-0.33421	-0.60045
MRPL10	0.345811	0.233505	0.332612	0.130321	0.047935	0.208316	-0.46663	-0.64085
MRPL11	0.207181	0.411015	0.103128	-0.22622	-0.18281	-0.36899	0.01934	-0.26197
MRPL12	0.307562	0.260144	0.174608	0.161398	-0.08459	0.152414	0.489262	0.048694
MRPL13	0.38044	0.246948	-0.05585	-0.38271	-0.1353	-0.33892	-0.23959	-0.52192
MRPL14	0.376207	0.019397	-0.11117	-0.20454	0.012747	-0.12755	0.187019	0.024776
MRPL15	0.052365	-0.59247	0.133828	-0.22294	-0.01514	-0.13778	-0.29127	-0.60126
MRPL16	0.067732	-0.05302	-0.22512	0.243581	-0.07032	-0.13144	-0.39702	-0.49945
MRPL17	0.047656	0.408976	0.00421	-0.12085	-0.1996	-0.01643	-0.30255	-0.30758
MRPL18	0.08665	-0.64142	-0.27636	0.236165	0.117726	-0.14125	-0.38501	-0.5444
MRPL19	0.200822	-0.27502	-0.21757	0.005433	-0.19804	-0.21325	-0.58124	-0.5775
MRPL2	0.245099	0.551409	0.077919	0.175965	-0.00739	-0.34205	0.543082	0.309616
MRPL20	-0.22427	-0.71034	0.150756	-0.21853	-0.03631	-0.10938	-0.28875	-0.25956
MRPL21	0.179023	-0.35564	-0.02059	-0.2181	-0.27861	-0.03685	-0.06653	-0.26178
MRPL22	0.105312	-0.51574	0.133549	0.096947	-0.01849	-0.36437	0.036753	-0.06667
MRPL23	0.127397	-0.11021	0.117921	0.090596	-0.07241	-0.22839	0.478647	-0.04639
MRPL24	-0.25028	-0.23181	0.310492	0.34534	-0.05349	0.087267	-0.0332	-0.31282
MRPL27	-0.33652	0.102111	0.26709	-0.08694	-0.1902	-0.0827	-0.23258	-0.2645
MRPL28	0.193096	0.510705	-0.04961	-0.13796	0.02742	0.107001	0.366678	0.123657

MRPL3	0.337449	-0.26615	-0.29642	0.075695	-0.03017	-0.15446	-0.38793	-0.51737
MRPL30	0.306868	-0.59935	-0.03284	-0.25933	-0.21498	-0.24038	-0.2906	-0.41719
MRPL32	0.283552	-0.45368	-0.13543	-0.35204	-0.11329	-0.06365	-0.19635	-0.31737
MRPL33	0.019863	-0.48097	-0.0388	-0.2063	-0.18598	-0.3942	-0.03129	-0.06892
MRPL34	0.12999	-0.20069	0.024319	-0.22822	-0.09267	-0.1893	-0.28642	-0.11484
MRPL35	0.149776	0.152411	-0.34915	-0.18389	-0.16672	-0.33226	0.155074	-0.05694
MRPL36	0.216659	-0.06733	-0.05219	-0.04005	-0.03493	-0.30638	0.317556	-0.18768
MRPL37	0.166055	0.595297	0.466077	0.11231	0.137862	-0.34798	0.28073	-0.15646
MRPL38	0.244052	0.430612	0.240285	0.295315	0.068686	-0.03283	-0.33421	-0.56065
MRPL39	-0.01972	-0.55865	-0.15781	-0.15174	-0.33904	-0.22256	0.093056	0.01786
MRPL4	0.474578	0.658043	0.026003	0.079584	0.113078	-0.04375	0.097159	-0.29042
MRPL40	0.101225	-0.45355	0.126783	-0.24487	-0.01151	-0.39249	0.083617	-0.27065
MRPL41	-0.20536	-0.36231	0.259568	-0.00416	0.008991	-0.28215	-0.16545	-0.28919
MRPL42	0.03261	-0.42395	-0.18693	-0.25206	-0.1305	-0.50348	0.129518	-0.07544
MRPL42P5	NA	NA	0.06485	0.001461	-0.20968	0.203917	0.11191	0.21823
MRPL43	-0.2506	-0.31654	0.087371	0.141439	0.348261	-0.12346	-0.18075	-0.13108
MRPL44	0.190584	0.037705	-0.21962	0.140969	-0.43523	-0.26935	-0.3616	-0.21009
MRPL45	0.243269	-0.51762	0.314104	-0.21574	-0.13438	-0.36835	0.125768	-0.33464
MRPL46	0.273307	-0.22249	-0.36082	-0.10864	-0.15407	0.067561	0.182256	0.167398
MRPL47	0.069615	-0.5413	-0.33594	-0.29741	-0.30383	-0.33986	-0.20756	-0.34038
MRPL48	-0.08944	-0.56825	-0.20739	-0.06271	-0.06308	-0.19588	0.389361	-0.02165
MRPL49	-0.02341	-0.37066	0.003072	0.249091	0.240503	-0.15834	0.607274	0.50179
MRPL50	0.042718	-0.42117	-0.38661	-0.20551	-0.07217	-0.20935	-0.40614	-0.47785
MRPL51	0.511899	0.024637	-0.3938	0.349827	-0.31521	-0.24659	0.099975	-0.05127
MRPL52	-0.09345	-0.08693	-0.28918	0.13079	-0.23079	-0.44264	-0.17921	-0.36237
MRPL53	-0.04944	0.567413	0.275422	0.044481	-0.13131	-0.15904	0.164625	-0.08942
MRPL54	-0.13662	-0.21954	-0.23468	0.099338	-0.31205	-0.29818	-0.47004	-0.20415
MRPL55	-0.06828	0.147761	-0.01429	0.148332	-0.14104	-0.24917	-0.03232	-0.30754
MRPL9	0.557624	0.142851	-0.17089	0.455066	-0.1558	-0.06616	0.530981	0.203419
MRPS10	0.05651	-0.11594	-0.14237	-0.17362	-0.07525	-0.29218	-0.2345	-0.42762
MRPS11	0.196907	0.636092	-0.3793	-0.05841	-0.01939	-0.3487	0.273793	0.095534
MRPS12	0.257409	0.554982	-0.12533	-0.00695	-0.09581	-0.19099	0.287305	-0.15029
MRPS14	0.34938	0.411684	-0.15136	-0.23649	-0.26924	-0.26735	0.166647	0.119642
MRPS15	0.14857	0.164772	0.231059	0.03138	-0.18677	-0.36821	0.198232	0.010295
MRPS16	0.114019	0.544533	0.211731	-0.18631	-0.16331	-0.26774	-0.40114	-0.38362
MRPS17	0.388529	0.033142	-0.23407	-0.06553	-0.1427	-0.21005	-0.02893	-0.17439
MRPS18A	0.283852	0.770342	-0.1622	0.196413	-0.07769	0.103707	0.022531	-0.18483
MRPS18B	0.077218	0.08702	-0.24368	0.32023	-0.07777	-0.26046	-0.28615	-0.45322
MRPS18C	0.038335	-0.52905	-0.19716	-0.21608	-0.26792	-0.39324	0.101118	0.04266
MRPS2	0.398891	0.639138	0.029249	0.16538	0.104623	0.242871	0.243125	0.172966
MRPS21	-0.17884	-0.60538	-0.05245	-0.0818	-0.44642	-0.14262	-0.05495	0.025523
MRPS22	0.19737	0.391235	-0.17646	0.015762	-0.20729	-0.30865	0.232275	0.181929
MRPS23	0.336784	-0.4286	0.06174	0.055926	-0.10879	-0.09684	-0.20258	-0.53691
MRPS24	0.377634	-0.2855	-0.11097	0.022369	-0.11066	-0.46208	0.27979	-0.01795
MRPS25	0.577505	0.091024	-0.18857	0.352832	-0.01883	-0.23998	NA	NA
MRPS26	0.294358	0.365642	-0.21738	0.169789	-0.01638	0.33726	0.296307	-0.32406
MRPS27	0.012529	-0.50473	0.246999	0.466379	-0.17853	0.004714	0.223486	-0.0971
MRPS28	0.054168	-0.64918	0.019845	-0.17006	-0.35911	-0.15946	0.045309	-0.03108
MRPS30	0.030883	-0.46853	-0.00846	0.044969	-0.17999	0.101478	0.667433	0.26945
MRPS31	0.383833	-0.17257	0.035608	0.043114	0.17007	-0.04359	0.146426	-0.02612
MRPS33	0.340281	-0.55377	0.226107	-0.0946	-0.12732	-0.00286	-0.03194	-0.26513
MRPS34	0.165166	0.506989	-0.36528	0.184525	0.071496	-0.04789	0.505374	0.137161

MRPS35	0.374461	-0.06961	-0.57924	-0.00594	-0.06986	-0.37465	0.069654	-0.15309
MRPS36	0.092655	0.612535	0.144888	-0.30228	-0.23284	-0.39344	-0.00078	-0.18849
MRPS5	0.000317	-0.3647	0.158593	0.183529	-0.03834	-0.1095	0.384058	0.210345
MRPS6	0.071293	-0.28679	-0.1672	0.043186	-0.17447	-0.10513	-0.14766	-0.198
MRPS7	-0.20732	-0.01137	-0.09883	0.283878	-0.2237	-0.11021	-0.17428	-0.38585
MRPS9	0.493682	-0.03117	-0.06182	0.107429	0.061186	-0.05619	0.730964	0.444266
MRRF	-0.08627	0.038478	-0.24116	-0.05241	-0.20657	-0.56512	0.213095	0.196748
MRS2	NA	NA	-0.19068	-0.25265	-0.10328	-0.37037	NA	NA
MRTO4	0.061102	0.263536	0.072854	0.117559	0.144636	0.30092	NA	NA
MRVI1	0.245371	-0.25859	-0.17062	-0.09641	-0.07292	-0.15426	-0.22255	-0.09276
MS4A1	-0.22064	0.212146	0.051998	0.695891	-0.16217	0.460899	-0.16499	-0.02913
MS4A10	0.131574	0.248663	NA	NA	NA	NA	0.315699	0.477595
MS4A12	-0.0768	-0.05783	0.057902	-0.30095	-0.13931	-0.15766	-2.8E-05	-0.38577
MS4A13	0.305804	0.417899	NA	NA	NA	NA	-0.51908	-0.37759
MS4A14	NA	NA	-0.1344	0.065216	-0.26388	0.060288	NA	NA
MS4A15	NA	NA	-0.3167	-0.04418	-0.07506	0.031754	NA	NA
MS4A2	-0.16008	0.051206	-0.02232	0.181573	-0.06272	0.154259	-0.20987	0.287561
MS4A3	-0.21626	0.409254	0.009539	0.037021	0.158603	-0.02681	0.057265	-0.05155
MS4A4A	-0.30859	0.065158	0.064075	-0.35326	-0.49918	-0.02892	-0.48628	0.032438
MS4A5	-0.09012	0.561593	0.161718	-0.01906	0.289889	-0.43084	-0.41762	0.15803
MS4A6A	-0.45207	-0.21511	0.113308	-0.36201	-0.30802	0.023207	-0.49372	0.125633
MS4A6E	0.018949	-0.23657	0.086883	-0.40449	-0.02692	0.024281	-0.33028	-0.13553
MS4A7	-0.48381	-0.4184	0.136442	-0.10021	-0.46095	0.37625	-0.42058	0.25179
MS4A8B	-0.28458	0.33332	-0.12522	-0.03606	-0.32512	-0.28455	0.306723	0.438317
MSC	-0.13563	-0.03927	0.198394	0.269326	0.13404	0.217513	-0.07787	0.472237
MSH2	0.186682	-0.19581	-0.09618	-0.16942	0.027953	0.129034	-0.26888	-0.33912
MSH3	0.267398	-0.5402	0.313616	-0.01718	-0.21911	-0.34256	0.279562	0.246949
MSH4	-0.07886	-0.20346	0.175446	0.244103	0.107235	-0.48313	0.14655	0.1818
MSH5	0.206785	-0.40324	NA	NA	NA	NA	0.493032	0.316659
MSH6	0.437608	-0.23631	-0.24889	0.287251	0.153901	0.175318	-0.01926	-0.32865
MSI1	-0.12989	0.179061	0.101081	-0.22317	0.207034	-0.15797	0.212056	0.297625
MSI2	0.049058	0.178755	-0.17895	0.24555	-0.10915	-0.37214	0.542535	0.06404
MSL1	NA	NA	0.259247	0.331758	0.010296	-0.13568	NA	NA
MSL2	NA	NA	-0.1879	0.09255	0.314367	0.185513	NA	NA
MSL3	NA	NA	-0.13028	0.268378	-0.12356	-0.29959	NA	NA
MSLN	-0.33517	0.142516	0.150693	0.054679	-0.04206	0.140113	0.276759	-0.36617
MSMB	-0.32956	0.269926	0.03057	-0.19089	-0.07099	-0.35718	-0.088	0.080753
MSN	-0.51798	-0.33202	0.02254	0.507682	-0.12818	0.687729	-0.31002	0.086197
MSR1	-0.28836	-0.25187	0.049603	-0.35236	-0.3153	0.085135	-0.37682	0.090984
MSRA	-0.04374	0.163768	0.099267	-0.24104	-0.0228	-0.14992	0.240436	0.42801
MSRB2	-0.19729	-0.20613	0.388632	0.030786	0.022306	0.111588	-0.13026	-0.17198
MSRB3	-0.24397	-0.30547	0.081102	-0.07129	-0.15923	0.100732	-0.1945	-0.23047
MST1	-0.03369	-0.17999	0.193498	0.153954	0.163371	-0.14733	NA	NA
MST150	-0.22361	0.047407	0.231455	-0.16793	-0.07045	-0.24703	0.118733	0.135797
MST1R	0.260511	0.265351	-0.05963	0.011889	0.241919	0.097022	0.343043	0.312197
MST4	0.514353	0.549052	NA	NA	NA	NA	0.448787	0.059035
MSTN	NA	NA	0.13932	0.089003	-0.04703	0.050014	NA	NA
MSTO1	-0.06549	0.021812	0.234962	0.357041	0.012267	0.032145	NA	NA
MSX1	0.141776	-0.33655	0.162507	0.077971	0.164256	0.130543	0.202855	0.102503
MSX2	0.12327	-0.14985	0.220976	0.132608	-0.09641	0.179641	0.088453	-0.25309
MT1A	-0.15746	0.243934	0.151128	-0.12197	-0.1409	-0.23547	-0.01526	0.069094
MT1B	0.111821	0.259427	0.125506	-0.09721	-0.21529	-0.30287	0.363078	0.306794

MT1E	-0.19193	-0.15418	0.106386	-0.14019	-0.24785	-0.28102	-0.18843	-0.16532
MT1F	0.010663	0.153776	0.226502	-0.07483	-0.21153	-0.14418	0.205249	0.046054
MT1G	0.041915	0.219448	0.14802	-0.1043	-0.23802	-0.26096	0.059107	0.096208
MT1H	0.090715	0.030684	0.133996	-0.09174	-0.22866	-0.2514	0.113319	0.111451
MT1JP	-0.26066	-0.07442	0.182836	-0.0539	-0.23903	-0.1064	NA	NA
MT1M	-0.14209	0.05993	0.047771	-0.04788	-0.29657	-0.06525	-0.10379	-0.29155
MT1X	-0.22644	0.175465	0.101436	-0.11276	-0.20107	-0.3252	0.205587	0.004017
MT2A	-0.21971	0.208584	0.131826	-0.10974	-0.19658	-0.19166	0.040808	0.147267
MT3	-0.13102	0.431547	0.375684	0.195326	-0.23097	-0.0717	0.101844	-0.21721
MT4	0.11071	0.802512	0.255153	0.070798	-0.1897	0.10173	-0.03045	-0.11578
MTA1	0.122577	-0.07131	0.037136	0.151003	0.140489	0.181172	0.243879	0.250855
MTA2	-0.12015	0.590528	-0.16644	0.217191	0.033884	-0.19395	0.286838	0.068761
MTA3	0.240439	-0.05691	0.001105	0.116583	-0.2071	-0.48714	0.350299	0.09757
MTAP	-0.16216	-0.37986	-0.35171	-0.30383	-0.17621	0.12917	0.220968	0.153362
MTBP	0.263747	-0.49531	-0.33657	-0.01804	-0.22943	-0.20321	0.296713	0.146649
MTCH1	0.154328	-0.28649	0.075302	0.11905	0.208156	0.062254	-0.15716	-0.03546
MTCH2	0.027028	0.546836	-0.03611	0.186557	0.167488	-0.32823	-0.1494	-0.11268
MTCP1	0.042072	-0.33993	-0.10727	0.190649	0.153356	-0.54086	-0.04903	-0.14805
MTDH	0.057907	-0.46841	0.184654	0.189086	-0.12272	-0.0056	-0.15885	-0.3801
MTERF	0.15288	-0.25458	-0.27723	-0.13399	-0.03412	-0.15651	0.45659	0.385236
MTERFD1	0.0072	-0.63297	-0.02068	-0.11661	-0.19015	-0.02498	-0.12933	-0.3568
MTERFD2	-0.02106	-0.03487	-0.18242	0.088316	-0.17947	-0.03695	-0.64582	-0.51933
MTERFD3	0.568257	0.584851	-0.21942	-0.18302	0.183232	-0.25333	0.109443	0.278616
MTF1	-0.55431	-0.25502	-0.03799	-0.24523	0.051895	0.078754	-0.15285	0.030653
MTF2	0.114493	0.476055	0.012996	-0.03167	-0.07889	0.240725	-0.44725	-0.38344
MTFMT	0.28492	0.06799	-0.01673	-0.22591	-0.01313	-0.34659	0.324358	-0.11381
MTFR1	0.048047	-0.21682	-0.0773	0.119777	-0.22131	-0.52412	0.385902	0.334209
MTG1	0.079447	0.459835	-0.07618	0.129749	0.327271	-0.04691	0.085482	0.443298
MTHFD1	0.046493	-0.27888	-0.33444	0.290787	0.122193	0.235834	0.455134	0.533118
MTHFD1L	0.359997	-0.21536	-0.17321	0.333435	0.216479	-0.167	0.42585	0.25508
MTHFD2	-0.19778	0.07767	0.003273	0.118398	-0.03992	-0.38397	0.211617	0.217023
MTHFD2L	0.054094	0.087563	-0.33449	-0.14633	-0.20686	-0.27148	0.094455	0.110977
MTHFR	-0.49975	-0.07773	0.125362	-0.19299	-0.09895	0.179393	-0.33984	0.363052
MTHFS	-0.0031	-0.57464	-0.31693	0.440194	-0.23276	-0.33404	0.268268	0.208592
MTHFSD	0.304724	0.224217	-0.2697	-0.09019	0.147351	-0.01422	NA	NA
MTIF2	0.369689	0.192154	-0.04714	-0.02875	0.122576	-0.18992	0.096542	0.016642
MTIF3	0.267692	-0.15909	0.282106	0.021428	-0.01273	-0.33632	-0.18164	-0.35571
MTL5	0.235002	0.639201	-0.15724	-0.03088	0.106941	-0.02686	0.197908	-0.16082
MTM1	-0.05359	-0.43535	-0.24022	-0.17204	-0.07289	-0.19994	0.376416	0.115993
MTMR1	0.334616	0.157244	-0.139	-0.0052	0.340458	0.096308	0.548645	0.562255
MTMR10	-0.09531	0.146606	0.23241	-0.38133	0.184618	0.107386	NA	NA
MTMR11	0.406622	0.0333	-0.17031	-0.01865	0.162126	-0.06249	-0.02736	0.210107
MTMR12	-0.04597	-0.4212	-0.28192	0.03746	0.097897	-0.38466	0.548281	0.17824
MTMR14	0.233774	0.5461	-0.02805	0.145646	0.167655	-0.05225	0.299793	0.369919
MTMR15	NA	NA	0.134582	0.015892	0.115731	0.353105	0.242585	0.143097
MTMR2	0.157833	0.436188	-0.1066	-0.19436	-0.07013	-0.28141	-0.08624	-0.14204
MTMR3	-0.48526	-0.46738	0.006214	-0.21313	-0.0344	0.297377	0.270093	0.288515
MTMR4	-0.26048	-0.21194	-0.09734	0.121939	-0.09229	0.053983	0.285896	0.157317
MTMR6	0.043037	-0.47069	0.176098	-0.29543	-0.03336	-0.01992	-0.10853	-0.19829
MTMR7	-0.43639	-0.19049	-0.39616	-0.03635	-0.37091	-0.19813	-0.17774	0.147227
MTMR8	0.166316	0.629224	0.015161	-0.29147	-0.00331	0.09545	0.106062	-0.1099
MTMR9	-0.00698	-0.20671	-0.03875	-0.08345	-0.22115	-0.1143	-0.22794	-0.14395

MTNR1A	-0.00408	-0.03059	-0.21254	0.230784	0.130563	0.242849	0.251462	0.21827
MTNR1B	0.162981	0.62212	0.066057	-0.04234	-0.04986	0.325411	0.554425	0.344186
MTO1	0.297193	-0.5061	-0.21054	0.110605	0.079187	-0.01725	0.474646	0.498986
MTP18	-0.28108	0.323068	0.014338	0.254212	0.043462	0.102546	0.228123	-0.06166
MTPAP	NA	NA	-0.30485	0.025253	-0.04981	0.09063	NA	NA
MTPN	-0.08456	-0.71428	0.20095	-0.13936	-0.0753	0.201438	-0.17915	-0.2289
MTR	0.291969	-0.08266	-0.31425	-0.03772	-0.04425	0.251334	-0.542	-0.55485
MTRF1	0.298283	0.567848	0.248379	0.060971	0.11068	-0.28036	-0.26831	-0.33196
MTRF1L	0.21314	-0.28104	-0.15485	-0.24606	0.02368	-0.4257	-0.31588	-0.38823
MTRR	0.106333	0.02373	0.212153	-0.14051	-0.10508	0.194262	0.231536	-0.08649
MTSS1	-0.07977	-0.37476	0.110926	0.007132	0.071165	-0.1532	0.027411	-0.29168
MTSS1L	NA	NA	0.278085	0.032027	0.274538	0.198369	NA	NA
MTTP	0.419544	-0.08392	0.448953	-0.04517	0.170725	0.349089	0.164016	0.410363
MTUS1	-0.47224	-0.61716	-0.0546	0.010144	-0.13391	-0.14575	0.111134	0.502876
MTX1	0.07393	0.344811	0.211194	0.112703	-0.09177	-0.08005	0.665671	0.076428
MTX2	0.199973	-0.60652	-0.15498	-0.25171	-0.09802	0.039578	-0.01743	-0.45758
MTX3	0.051368	-0.37463	0.088211	0.165832	-0.08596	-0.13876	0.117092	0.43195
MUC1	-0.46741	-0.44063	-0.06438	0.199087	-0.23405	0.123536	-0.04939	-0.35066
MUC12	NA	NA	-0.06627	0.031176	0.081648	0.173046	NA	NA
MUC13	-0.27352	0.094756	0.156539	0.090302	0.181389	0.125441	0.236062	0.14701
MUC15	-0.00585	0.459525	-0.17447	-0.44071	-0.13087	-0.12576	-0.25393	-0.04233
MUC16	NA	NA	-0.05	0.089038	0.204311	0.01776	-0.18767	-0.17987
MUC17	-0.20487	0.122134	0.241948	-0.05129	0.027917	-0.13343	0.094081	0.549447
MUC2	NA	NA	-0.06519	0.143937	0.013271	0.036164	0.003163	-0.24622
MUC20	0.322165	0.184813	0.079	0.005945	0.160204	-0.03958	0.261145	-0.11181
MUC4	0.256826	-0.2002	-0.05925	0.091083	-0.07503	0.246895	-0.15121	-0.238
MUC6	NA	NA	0.119072	-0.18805	-0.10634	-0.30382	NA	NA
MUC7	0.032414	-0.14348	0.3468	-0.20573	0.366693	-0.11141	0.274563	0.039195
MUCL1	NA	NA	0.151745	-0.014	-0.18115	0.343876	NA	NA
MUL1	NA	NA	0.263323	-0.09396	0.166019	-0.02415	NA	NA
MUM1	-0.10155	-0.08508	0.013886	0.216042	0.40318	0.247131	0.090249	0.202621
MUM1L1	0.310476	0.314669	0.070874	-0.1171	0.007528	-0.08152	0.060679	0.322881
MUS81	-0.05896	0.299594	-0.04458	0.344663	0.355259	0.211646	-0.48401	-0.06827
MUSK	0.264691	-0.03672	-0.13027	0.128446	0.091595	0.354256	-0.00215	-0.09277
MUSTN1	-0.1412	0.046488	0.135289	0.129771	-0.15642	0.033636	-0.39959	-0.11263
MUT	0.217997	-0.28115	-0.31555	-0.24738	0.00334	-0.24763	-0.31923	-0.2415
MUTED	0.217207	0.392374	-0.23116	-0.10857	-0.13411	-0.32863	-0.02454	-0.1687
MUTYH	-0.23036	0.229484	0.294234	0.317597	0.33907	-0.21255	0.354223	0.247952
MVD	-0.08078	0.308603	0.350448	0.118381	0.222631	0.146611	0.150895	0.254152
MVK	-0.09112	-0.20858	-0.27222	0.142449	0.337386	-0.15546	0.267702	0.393043
MVP	0.0468	0.18566	0.223057	0.143574	0.32518	0.06119	0.160053	0.099355
MX1	-0.20492	0.136827	0.093015	-0.27076	-0.18402	0.280679	-0.18548	0.137459
MX2	0.136005	-0.18802	0.198044	0.175609	-0.25977	0.589988	-0.01469	0.619224
MXD1	-0.3241	-0.00779	-0.02507	-0.14258	-0.155	-0.04902	-0.10268	0.12494
MXD3	-0.05099	0.353081	0.361357	0.11563	0.199988	-0.07574	0.387143	0.220227
MXD4	0.015596	0.120919	0.216492	0.019059	0.006354	-0.16436	-0.48988	-0.42127
MXI1	0.287825	0.003146	-0.22874	-0.17759	-0.21783	-0.29119	-0.20768	-0.11293
MXRA5	-0.22755	-0.15709	0.183563	0.104082	0.013267	0.532251	-0.26059	-0.03702
MXRA7	-0.37328	-0.16483	0.052619	-0.00755	-0.10975	-0.0008	-0.2586	-0.26109
MXRA8	NA	NA	0.176912	0.001515	-0.03071	0.139445	0.039199	0.131401
MYADM	-0.26548	-0.23017	-0.02939	-0.11056	0.124173	0.181065	-0.05206	-0.2751
MYADML	0.388952	0.566893	-0.013	-0.20891	-0.02089	0.203739	-0.55137	0.238968

MYB	0.475849	0.09732	-0.29475	0.060886	0.241269	0.164303	0.287884	0.016222
MYBBP1A	0.290526	-0.12651	-0.04322	0.280365	0.209702	0.33008	0.083624	-0.0403
MYBL1	NA	NA	0.086595	0.585239	0.042077	0.431128	NA	NA
MYBL2	-0.27517	0.733863	0.143565	0.43647	0.494823	0.019557	-0.22339	-0.24749
MYBPC1	-0.20203	-0.73873	-0.48741	-0.29487	-0.42561	-0.67019	-0.04312	-0.40765
MYBPC2	-0.20246	0.277118	0.072768	0.528633	-0.14905	0.028104	0.024952	0.098464
MYBPC3	-0.27207	0.114923	-0.16986	0.165504	-0.03963	-0.30067	-0.18322	0.548281
MYBPH	0.064113	0.763383	-0.16143	-0.00016	0.041402	-0.11291	0.138256	0.458889
MYBPHL	0.007744	0.345492	NA	NA	NA	NA	0.55034	0.222353
MYC	-0.11906	0.029459	-0.084	0.001775	0.139024	0.341654	0.070206	-0.15849
MYCBP	0.115859	0.161621	-0.21438	-0.0239	-0.08	-0.66167	0.202464	0.035227
MYCBP2	0.516761	-0.07389	0.031459	0.275094	-0.06755	0.203856	-0.00312	0.147619
MYCBPAP	-0.13551	0.518145	-0.2498	0.03103	-0.30548	-0.4072	-0.40751	-0.28835
MYCL1	0.256332	0.040053	-0.16723	-0.00299	0.09504	-0.15548	0.007098	0.012725
MYCN	-0.03625	-0.26185	-0.0744	0.01872	-0.2672	0.269431	0.217945	-0.09748
MYCT1	-0.20371	0.22195	-0.0984	0.167814	-0.07345	-0.01317	-0.69631	-0.25048
MYD88	-0.17506	0.107911	0.10728	-0.03862	0.140185	0.086318	0.395974	0.408278
MYEF2	0.243089	0.741502	0.013214	-0.05318	0.041917	0.113039	0.090705	-0.08721
MYEOV	-0.03426	0.580855	0.081241	-0.17996	0.062525	0.031979	0.174167	0.19841
MYEOV2	0.18242	0.612686	-0.09593	-0.1668	-0.30094	-0.20146	-0.2211	-0.36866
MYF5	-0.17738	0.441492	0.157891	-0.07663	-0.13821	-0.14755	0.183126	0.071045
MYF6	-0.20161	0.018883	0.01583	0.11272	0.125176	-0.12133	-0.47642	-0.0135
MYH1	0.074721	-0.0016	-0.03557	0.133729	-0.07125	0.057776	-0.01119	-0.12928
MYH10	-0.14376	-0.1607	-0.11491	0.076079	-0.1603	-0.00495	-0.16852	-0.15468
MYH11	0.013253	0.046208	0.206185	0.027687	0.186255	0.338506	-0.18892	-0.43148
MYH13	-0.12205	0.646107	0.001941	-0.09468	0.012088	-0.08053	-0.35303	-0.08641
MYH14	0.490991	0.333402	0.228998	-0.15863	0.263414	0.170454	0.467063	0.232698
MYH15	NA	NA	-0.26365	-0.39488	-0.33605	-0.37695	NA	NA
MYH2	0.094623	-0.02194	-0.14687	-0.07092	0.137969	0.111563	0.351111	-0.06851
MYH3	-0.14422	0.124104	-0.04748	-0.22593	0.084298	0.257489	-0.18265	0.007822
MYH4	0.255548	0.257949	-0.04228	-0.2196	0.105908	0.037658	-0.09774	-0.41402
MYH6	-0.29763	0.426965	-0.17837	-0.09436	-0.00262	-0.08621	0.414828	0.209372
MYH7	0.022086	0.007421	-0.35375	0.143804	-0.12422	0.308122	0.01848	0.268689
MYH7B	0.285321	0.16552	0.204662	0.042099	-0.02508	0.085778	0.473352	-0.21762
MYH8	0.328947	-0.17593	0.131346	0.104079	0.175806	-0.18436	0.201658	0.042404
MYH9	-0.2648	-0.39404	0.174418	0.136659	0.292009	0.307228	0.461828	0.328972
MYL1	-0.10826	-0.07735	0.019781	-0.05279	-0.06629	0.09646	-0.13784	-0.47846
MYL2	0.059202	0.150956	0.188395	-0.19751	-0.22349	0.085479	-0.36808	-0.20313
MYL3	-0.198	-0.14434	0.225565	-0.21709	-0.11425	-0.04299	NA	NA
MYL4	-0.067	0.10799	0.019154	-0.18301	-0.04332	-0.47082	0.122588	0.247491
MYL5	NA	NA	0.192079	-0.04145	0.036632	-0.06523	0.067985	0.548295
MYL6	-0.00693	0.625606	-0.3636	-0.21563	-0.02618	-0.16351	-0.08859	0.156365
MYL6B	0.414773	0.081295	0.085105	0.139715	-0.05487	0.043117	NA	NA
MYL7	0.247218	0.737168	0.03644	-0.15251	0.012689	0.092086	-0.36384	-0.19874
MYL9	-0.04332	-0.23486	0.259062	-0.01656	0.061566	0.100879	-0.23076	-0.33332
MYLC2PL	0.049113	-0.12838	0.282916	-0.06683	-0.04309	-0.05729	-0.24252	0.080197
MYLIP	-0.04769	-0.65851	0.196794	0.041116	0.092494	0.308973	0.145465	-0.19883
MYLK	-0.18481	-0.30244	-0.00786	-0.14468	0.019506	-0.08973	-0.22882	-0.34856
MYLK2	0.506304	0.728592	-0.32858	-0.21901	-0.06032	0.138738	0.583575	0.155102
MYLK3	NA	NA	-0.0679	-0.09934	0.300858	-0.14894	NA	NA
MYLPF	-0.05785	0.172821	-0.28811	-0.08572	0.089073	0.030418	0.329928	0.168882
MYNN	-0.44007	-0.29124	-0.43014	-0.14794	-0.31212	-0.38935	-0.14664	-0.2314

MYO10	-0.14837	0.210926	-0.04362	0.130867	0.345009	0.046693	0.080774	-0.37284
MYO15A	-0.0548	-0.19093	-0.038	-0.03102	-0.26355	-0.0815	-0.45443	-0.20513
MYO16	-0.09596	-0.05411	0.089149	0.369675	-0.01883	0.069589	NA	NA
MYO18A	-0.12458	0.268214	0.408456	0.217638	0.27491	0.256694	-0.4005	-0.01536
MYO18B	0.199074	0.02772	0.22722	-0.01328	0.172532	0.224847	-0.07183	-0.00586
MYO19	NA	NA	-0.26846	0.264465	0.052095	-0.04054	NA	NA
MYO1A	0.258204	0.23024	-0.05191	-0.01846	0.058727	-0.07415	0.181538	0.367011
MYO1B	-0.26773	0.111399	-0.25456	-0.11373	-0.07641	0.100106	-0.34265	-0.35443
MYO1C	-0.22662	-0.00949	0.214575	-0.12138	0.254412	0.295766	-0.09599	-0.11539
MYO1D	0.074767	0.036679	0.168299	-0.08779	0.128409	0.064339	0.354156	0.052726
MYO1E	-0.06562	-0.11957	0.091309	0.092376	0.045233	0.157579	-0.0995	-0.10965
MYO1F	-0.48346	-0.16545	0.367398	0.031785	0.05848	0.618828	-0.44533	0.512497
MYO1G	-0.48884	0.227161	0.434561	0.508753	-0.02025	0.811054	-0.53165	0.410424
MYO1H	NA	NA	0.300853	0.02025	-0.25633	0.019194	NA	NA
MYO3A	-0.57694	0.486864	0.3831	-0.0285	0.189803	0.2597	-0.31941	0.379485
MYO3B	0.004	0.132589	-0.14693	-0.0577	0.241782	-0.20438	-0.51203	-0.48615
MYO5A	-0.33411	-0.11596	0.095209	0.077601	-0.27177	0.234804	-0.63016	-0.32484
MYO5B	NA	NA	0.233537	-0.01137	0.026377	0.274229	-0.02132	-0.17801
MYO5C	-0.04786	-0.04225	-0.22456	0.199416	0.088174	-0.53915	0.251172	-0.10695
MYO6	0.235526	0.105782	-0.17149	-0.01267	0.233228	0.23297	0.523469	0.275812
MYO7A	0.065968	0.194243	0.249525	-0.10476	0.080196	0.033169	0.219957	0.443707
MYO7B	NA	NA	0.179609	0.067579	0.114787	-0.00805	0.489385	0.329196
MYO9A	0.044889	0.021984	-0.09492	-0.02866	0.259043	-0.19537	0.201353	0.008344
MYO9B	-0.17827	0.251273	0.049574	0.065233	0.089968	0.562017	-0.21228	0.142087
MYOC	0.123641	-0.37048	-0.17701	-0.1821	0.145058	-0.19229	-0.29644	-0.26518
MYOCD	0.089055	0.279929	0.020451	-0.0963	-0.22106	0.121969	-0.44052	-0.39628
MYOD1	0.13688	0.546613	0.142643	-0.15149	-0.14413	-0.05522	0.442153	0.30327
MYOF	NA	NA	-0.12903	0.107212	-0.20559	-0.05444	NA	NA
MYOG	0.02767	0.273226	0.244142	-0.30329	0.128124	-0.2091	0.409156	0.634777
MYOM1	-0.19038	0.055959	-0.24264	-0.28597	-0.05007	-0.33983	-0.30821	-0.29711
MYOM2	0.00903	0.02242	-0.15977	-0.03924	-0.0824	-0.05926	-0.11525	-0.07039
MYOM3	0.510048	-0.22043	0.2246	-0.17589	0.096486	-0.22473	0.268173	0.470063
MYOT	0.136652	-0.17489	-0.04088	-0.21023	-0.19097	0.091602	-0.18755	-0.28687
MYOZ1	0.294559	0.594386	-0.0509	0.006062	-0.27412	-0.04209	0.03704	-0.13234
MYOZ2	NA	NA	0.042631	-0.40835	-0.04851	0.025465	0.089555	0.152062
MYOZ3	-0.05515	-0.26133	0.088486	-0.04011	-0.27097	-0.29826	-0.18654	0.137607
MYPN	0.571537	0.394284	-0.11766	-0.07938	-0.22585	-0.25585	-0.30097	0.087654
MYRIP	0.33024	-0.39354	-0.06868	-0.0281	0.221235	-0.33041	-0.02113	-0.25238
MYSM1	NA	NA	-0.0814	-0.36867	0.030989	0.086299	NA	NA
MYST1	0.081537	0.459567	0.05496	0.193534	0.205785	0.129193	0.007529	-0.1081
MYST2	0.405436	0.72745	0.109444	0.165895	0.220652	0.315167	0.368591	0.164577
MYST3	0.106262	0.397222	0.048096	-0.03707	0.084049	0.365921	0.136407	0.081953
MYST4	-0.33218	-0.46292	-0.26255	0.015041	0.055619	-0.10283	-0.40099	-0.2039
MYT1	0.127877	-0.17841	-0.1362	0.075293	0.182039	0.052361	0.65026	-0.01345
MYT1L	0.337297	-0.15046	-0.06628	-0.04655	0.142923	0.146742	0.001329	0.17507
MZF1	0.084826	-0.21634	0.133211	-0.02267	0.219384	0.146669	NA	NA
N-PAC	0.143764	-0.26095	-0.04301	-0.02876	0.282436	-0.05376	0.008501	-0.30346
N4BP1	0.176464	0.452489	0.082361	0.022504	0.123936	0.156207	-0.03647	0.074227
N4BP2	-0.33776	0.324526	0.128108	-0.12415	-0.13816	-0.10256	-0.57946	-0.58025
N4BP2L1	NA	NA	0.126986	0.010808	0.059286	0.276784	NA	NA
N4BP2L2	NA	NA	0.098283	-0.15639	0.231029	-0.06918	NA	NA
N4BP3	-0.48116	0.065104	0.223605	0.004721	0.042486	0.093953	-0.23237	-0.1305

N6AMT1	-0.22829	-0.20168	-0.02958	-0.04481	-0.25054	-0.33947	-0.06851	0.175809
N6AMT2	0.310586	0.101787	0.083337	-0.14403	0.197749	-0.18568	NA	NA
NAAA	NA	NA	-0.09888	-0.18533	-0.06138	-0.48211	NA	NA
NAALAD2	0.278279	0.027589	-0.40902	0.043204	-0.04536	-0.16641	-0.46305	-0.31034
NAALADL1	0.13953	0.13864	0.391785	-0.422	0.195005	-0.33021	0.101893	0.36283
NAALADL2	0.090346	0.324877	NA	NA	NA	NA	-0.36677	-0.4219
NAB1	-0.21189	-0.40008	-0.16957	-0.39949	-0.19035	-0.21642	0.012402	-0.05062
NAB2	0.148616	0.58762	0.186904	0.100052	0.187923	0.247653	-0.26624	-0.05251
NACA	-0.03542	-0.70775	-0.22019	0.145099	0.223268	-0.21794	-0.51375	-0.43717
NACA2	NA	NA	-0.19605	0.060193	0.257829	-0.10577	NA	NA
NACAD	NA	NA	0.228088	-0.10938	-0.05383	0.33259	NA	NA
NACAP1	NA	NA	-0.51572	0.061739	-0.25889	-0.18841	-0.54081	-0.59586
NACC1	NA	NA	-0.34324	0.225912	-0.11017	-0.07633	NA	NA
NACC2	NA	NA	-0.28514	-0.20624	0.074601	0.04447	NA	NA
NADK	0.164915	-0.21583	0.344125	0.177466	0.264888	-0.1886	NA	NA
NADSYN1	0.109369	0.129728	-0.01984	0.038983	0.323095	0.0631	-0.22908	0.052677
NAE1	NA	NA	-0.17907	-0.4249	0.230468	-0.15325	NA	NA
NAF1	NA	NA	-0.33603	0.227755	-0.0505	-0.10208	NA	NA
NAG	0.09372	0.412982	0.438642	0.278937	0.023185	-0.05703	0.153154	-0.27107
NAGA	-0.33793	0.108857	0.178927	0.225468	0.058387	-0.15773	0.179212	0.452585
NAGK	-0.46164	0.112549	0.039796	0.281707	-0.14863	0.11842	-0.29142	0.101426
NAGLU	-0.03563	0.483967	0.4614	-0.03711	0.073752	-0.26389	-0.50269	-0.15786
NAGPA	0.319293	0.181544	0.101965	-0.18648	0.235167	-0.06596	0.393884	0.298275
NAGS	0.19423	0.467259	0.349652	-0.31539	-0.13729	-0.11015	-0.57908	0.051479
NAIF1	NA	NA	0.102703	0.123751	-0.05531	-0.41954	NA	NA
NAIP	0.091049	0.583924	-0.0466	0.087164	-0.08196	0.188297	-0.36754	0.20342
NALCN	NA	NA	-0.02038	-0.08675	0.142001	0.272756	NA	NA
NAMPT	NA	NA	0.09291	0.042954	0.096508	0.248101	NA	NA
NANOG	-0.07053	0.129795	-0.0767	-0.17605	0.191555	0.095547	0.454544	0.272573
NANOS1	-0.27556	-0.12065	-0.25073	0.026088	0.084276	-0.06386	-0.52199	-0.18105
NANOS2	-0.011	0.17815	NA	NA	NA	NA	-0.44366	-0.09831
NANOS3	NA	NA	-0.24155	-0.18118	0.003002	-0.09573	NA	NA
NANP	-0.14889	0.138064	-0.21704	-0.10722	-0.06525	-0.054	0.261659	-0.23476
NANS	-0.43712	-0.2051	0.089514	0.206277	0.028834	-0.22812	-0.34327	-0.51215
NAP1L1	0.257579	-0.57535	-0.22879	0.1459	0.162475	0.338189	-0.12351	-0.10969
NAP1L2	-0.17116	0.714426	-0.00772	-0.44081	-0.23607	0.036465	0.345205	0.036717
NAP1L3	-0.22321	-0.26802	0.028094	0.078263	0.009111	-0.03546	-0.35838	-0.36801
NAP1L4	-0.07099	-0.24752	0.083649	0.079025	0.399914	0.312186	0.22226	0.275808
NAP1L5	0.0935	0.089972	-0.17077	-0.16657	0.07458	-0.12649	-0.20592	-0.3521
NAPA	0.396632	0.272246	-0.02956	-0.09229	-0.0476	-0.22738	-0.35126	-0.30723
NAPB	0.245875	0.209422	-0.16123	0.301075	0.036083	-0.01875	0.446459	0.163953
NAPEPLD	NA	NA	-0.30481	-0.04311	0.2127	-0.17441	NA	NA
NAPG	0.081109	0.612864	-0.10138	-0.28245	-0.16123	-0.48329	-0.44399	-0.57175
NAPRT1	-0.02018	0.425354	0.281085	-0.08477	-0.0415	-0.17191	0.118525	0.062756
NAPSA	0.179585	-0.31549	0.153489	0.815989	-0.01222	0.537214	-0.28241	0.143455
NAPSB	NA	NA	0.146775	0.783572	-0.10312	0.632314	-0.5583	0.245834
NARF	-0.23954	0.509672	-0.02027	0.160359	-5.2E-05	0.236499	0.415416	0.363007
NARFL	0.339636	0.631865	0.330583	-0.01231	0.306725	-0.06748	-0.02905	-0.38423
NARG1	0.22555	-0.3032	-0.36873	-0.17776	-0.06358	0.086008	0.175372	0.003498
NARG1L	-0.00462	-0.60646	0.137927	-0.17194	0.425401	0.382629	0.434043	0.238869
NARG2	-0.16354	-0.13239	-0.3128	0.016536	0.081039	0.272278	-0.13808	-0.00244
NARS	-0.0504	0.049521	-0.17662	-0.15043	-0.08835	-0.45325	-0.24931	-0.21471

NARS2	0.296899	-0.30474	-0.20777	0.483369	0.125377	-0.01449	0.149694	-0.07901
NASP	-0.18669	0.407971	-0.04197	0.501514	0.250568	0.172341	0.155002	0.328059
NAT1	-0.0661	-0.08189	0.020383	-0.20825	-0.38572	-0.38839	-0.02546	-0.0248
NAT10	0.239806	0.171371	0.152216	0.194311	0.321649	0.402683	0.513541	0.140467
NAT11	-0.20641	-0.39906	-0.22691	-0.01399	0.21001	-0.14417	0.165745	0.49419
NAT12	0.20325	-0.5505	-0.3333	-0.28617	-0.34441	-0.3129	0.470976	0.193885
NAT14	0.211577	0.147573	0.008258	-0.01251	0.169718	0.29685	NA	NA
NAT2	0.320096	0.159311	0.122335	-0.3297	-0.22921	-0.31513	-0.01239	0.002746
NAT5	0.269258	0.202448	-0.05429	-0.14851	0.063618	0.140682	0.494079	0.240389
NAT6	0.242872	0.671452	0.232143	0.360562	0.226824	0.007731	0.392107	0.052397
NAT8	0.02135	0.607116	0.114691	-0.29786	-0.16979	-0.40952	0.01981	0.454581
NAT8B	-0.24817	0.381378	0.041166	-0.20896	-0.16661	-0.51001	NA	NA
NAT8L	0.176732	0.621533	-0.2588	0.292123	-0.10896	0.036228	NA	NA
NAT9	0.207406	0.418444	0.459115	0.07262	0.179858	-0.13779	0.098493	0.235056
NAV1	-0.23426	-0.53782	0.064526	-0.085	-0.11703	0.036402	-0.04945	-0.12692
NAV2	0.424147	-0.08901	0.247303	-0.15359	0.075437	0.542239	-0.42095	-0.32261
NAV3	0.216137	-0.19669	0.202529	-0.07874	-0.46385	0.167793	0.037603	0.125973
NBEA	-0.20691	-0.39176	-0.03838	-0.08005	-0.13654	0.16056	-0.45354	-0.4358
NBEAL1	-0.2963	-0.1728	NA	NA	NA	NA	0.133463	0.232529
NBEAL2	NA	NA	0.186001	0.388354	0.35876	0.421558	NA	NA
NBL1	0.090304	0.685241	0.171329	0.181469	0.028003	-0.30778	-0.00012	0.305856
NBLA00301	NA	NA	0.146957	-0.09984	-0.07636	0.026234	NA	NA
NBN	0.107586	-0.31317	-0.16048	0.010752	-0.18214	0.001315	-0.38945	-0.20004
NBPF1	NA	NA	0.008471	-0.13573	0.239566	-0.15758	0.345964	0.244344
NBPF10	NA	NA	-0.00916	0.034659	-0.05863	0.441903	-0.49801	-0.50897
NBPF11	0.060943	0.255586	0.006254	-0.07617	0.003248	0.482784	0.14683	0.251178
NBPF14	-0.27759	-0.16503	-0.05315	0.136708	0.144931	0.22229	0.112852	0.308258
NBPF15	-0.11857	-0.32673	-0.21347	-0.02038	0.071278	0.140492	0.191285	-0.05805
NBPF3	-0.15657	-0.38932	-0.04387	0.052007	0.071847	0.218348	0.115584	0.289617
NBPF4	0.009855	-0.16195	NA	NA	NA	NA	NA	NA
NBPF7	NA	NA	NA	NA	NA	NA	-0.01085	-0.12714
NBR1	0.066815	0.242226	0.185031	-0.16106	0.06761	-0.17117	0.409584	0.358864
NBR2	0.088273	0.264355	0.228407	-0.08664	0.024795	-0.51881	-0.28091	-0.52524
NCALD	0.02736	-0.06252	0.146347	-0.22848	0.175788	-0.39569	-0.32056	-0.12162
NCAM1	0.436795	-0.04462	0.176835	-0.17402	-0.17536	-0.0461	-0.23909	-0.31245
NCAM2	0.105982	-0.17961	-0.02549	-0.03014	-0.22181	-0.01251	-0.21366	-0.24407
NCAN	0.3507	0.697255	-0.06483	-0.14212	0.000728	-0.07214	NA	NA
NCAPD2	0.013335	-0.10419	-0.2343	0.319863	0.159875	0.102676	NA	NA
NCAPD3	-0.03764	-0.39813	-0.36738	0.294604	-0.01155	0.067986	NA	NA
NCAPG	-0.11013	0.146315	-0.35676	0.238647	-0.00608	-0.10136	NA	NA
NCAPG2	NA	NA	-0.33808	0.296459	0.109249	-0.08147	0.533336	0.448405
NCAPH	0.381757	0.831533	-0.45735	0.295169	0.010797	-0.19257	NA	NA
NCAPH2	0.152191	0.640213	0.186652	0.142806	0.370359	0.081163	NA	NA
NCBP1	0.601564	0.465845	-0.41387	-0.10063	0.019148	-0.31379	-0.29868	-0.302
NCBP2	-0.22962	-0.75017	-0.25139	0.070468	-0.14785	-0.27449	-0.02201	-0.00313
NCDN	0.061612	0.473586	0.421315	0.274806	0.182395	0.226712	0.484356	0.324417
NCF1	-0.39631	-0.02987	0.426449	0.58399	-0.00887	0.453883	-0.29501	0.19862
NCF2	-0.61949	-0.25457	0.048637	0.082716	-0.02686	-0.07141	-0.5493	-0.36411
NCF4	-0.25664	0.355019	0.309421	0.30193	0.085889	0.193863	-0.55905	0.274569
NCK1	-0.18718	-0.48499	-0.0402	-0.20964	-0.28913	-0.32606	0.113747	0.128313
NCK2	NA	NA	0.341579	0.068867	0.060872	-0.10692	0.171488	-0.28019
NCKAP1	0.158492	-0.07391	-0.17188	-0.45493	-0.21475	-0.36486	0.390441	0.095427

NCKAP1L	-0.40453	0.316472	0.371266	0.503961	-0.10884	0.538051	NA	NA
NCKIPSD	0.048119	0.170349	0.150935	0.094213	0.404523	-0.184	-0.50327	-0.42051
NCL	0.268286	-0.3342	-0.07635	0.482044	0.076243	0.225536	-0.06218	-0.22683
NCLN	0.07989	0.597738	0.195798	0.339854	0.349276	0.090619	NA	NA
NCOA1	-0.10861	-0.33305	0.020035	-0.03867	-0.11589	0.165784	-0.46917	-0.25585
NCOA2	-0.14653	0.140163	-0.12467	-0.20476	0.037829	-0.01035	0.443037	0.378264
NCOA3	0.353508	0.540502	-0.0077	0.122281	0.149522	0.129087	-0.21859	-0.36896
NCOA4	-0.28684	-0.0984	-0.2624	-0.22757	-0.26551	-0.17624	-0.05511	0.357343
NCOA5	0.370431	0.214814	-0.08348	0.394061	0.2276	0.036742	0.624051	0.421039
NCOA6	0.270826	-0.65944	0.173826	-0.16004	0.446658	0.243802	0.201514	-0.09776
NCOA7	0.368983	-0.16344	0.015392	-0.08853	0.07069	-0.04966	-0.0483	0.370335
NCOR1	-0.11495	-0.36271	-0.03001	0.031099	-0.26018	0.130247	0.192496	0.25087
NCOR2	0.073055	-0.48328	0.077892	0.092138	0.235499	0.264456	0.058844	0.061099
NCR1	0.005602	0.117368	0.186657	0.33408	-0.16445	0.161533	-0.32101	-0.14435
NCR2	-0.05035	0.193455	-0.09119	-0.04009	-0.17027	0.192487	-0.33034	-0.44106
NCR3	0.10378	-0.20179	0.195431	0.703465	-0.17885	0.550656	-0.30181	0.196219
NCSTN	-0.23632	0.320278	-0.06703	0.18551	-0.09967	0.046169	0.211837	-0.01146
NDC80	-0.2297	-0.3267	-0.32638	0.142038	-0.06567	-0.27788	NA	NA
NDE1	-0.30683	0.330559	-0.08751	0.347991	0.387402	0.58857	-0.28116	-0.42041
NDEL1	-0.48461	0.135694	0.0322	0.205867	-0.06494	0.013012	0.005373	0.298985
NDFIP1	-0.41019	0.102671	0.546137	0.166089	-0.14972	-0.42509	0.161505	0.2035
NDFIP2	0.453478	0.018445	-0.01053	-0.07934	0.067896	-0.60645	0.551812	0.299086
NDN	-0.04797	-0.05725	0.070918	-0.04222	-0.13851	-0.15957	-0.31697	-0.18093
NDNL2	0.186649	-0.47451	0.067909	0.333862	0.17767	0.06398	-0.56067	-0.28662
NDOR1	0.286657	0.54129	0.108693	-0.04819	-0.10759	0.139817	-0.22133	-0.0514
NDP	0.031513	-0.41467	0.268851	-0.01138	-0.06274	0.037458	0.308681	-0.03233
NDRG1	-0.45899	0.15248	0.04081	0.254625	0.026306	0.024102	-0.43781	-0.26125
NDRG2	0.07574	0.07261	0.15199	0.340695	0.119427	0.172954	-0.08137	-0.40767
NDRG3	0.230915	-0.52665	0.136401	0.013672	0.309128	-0.02641	0.258024	-0.12319
NDRG4	-0.11606	0.044069	-0.02639	0.113374	-0.13132	0.049353	-0.08417	-0.05047
NDST1	-0.20403	-0.25643	0.111453	-0.1744	0.033968	-0.11128	-0.27362	0.157616
NDST2	0.039433	0.257331	0.170199	0.335081	0.178736	0.532534	0.146347	0.391911
NDST3	-0.1683	0.086879	-0.02143	0.025786	0.286563	0.008076	0.186631	0.169588
NDST4	0.164657	0.246001	0.202544	-0.28534	0.029627	0.072774	0.550454	0.107519
NDUFA1	-0.25385	-0.56575	-0.12624	-0.23343	-0.2693	-0.25126	0.006	0.198785
NDUFA10	NA	NA	-0.09919	0.21986	-0.05706	-0.1969	0.24729	-0.16038
NDUFA11	0.281156	-0.30805	0.117701	-0.16268	0.028304	0.042753	0.425289	0.096862
NDUFA12	0.078946	-0.43312	-0.049	-0.21744	-0.06624	-0.29959	0.151736	0.070311
NDUFA13	0.507843	0.581534	0.260087	-0.32168	-0.15597	-0.11779	-0.2513	-0.18814
NDUFA2	0.001976	-0.20606	0.15313	-0.34345	-0.14022	-0.43689	NA	NA
NDUFA3	0.54888	0.371398	0.133754	-0.13062	-0.31685	-0.16638	0.252412	0.101758
NDUFA4	-0.14069	-0.52872	0.128856	-0.32865	-0.27166	-0.08366	-0.54601	-0.31593
NDUFA4L2	0.243864	0.705863	0.099346	-0.00851	-0.29181	0.053058	NA	NA
NDUFA5	-0.01131	-0.48624	-0.20332	-0.32253	-0.25698	-0.34382	-0.59495	-0.60016
NDUFA6	-0.33822	-0.16286	-0.13891	-0.12531	-0.26433	-0.32521	NA	NA
NDUFA7	0.378478	0.6482	-0.00587	-0.26476	-0.11735	-0.26746	0.076071	-0.23119
NDUFA8	0.341399	0.07248	-0.00196	-0.2982	0.123755	-0.07243	-0.14325	-0.39354
NDUFA9	0.38902	0.180402	-0.12239	0.069243	-0.31044	-0.05288	0.02804	-0.03677
NDUFAB1	0.173619	0.012782	0.013945	0.032901	0.027943	-0.10962	-0.30208	-0.43509
NDUFAF1	0.185394	-0.35903	-0.07529	0.226178	-0.08953	-0.01868	-0.2152	-0.17993
NDUFAF2	NA	NA	-0.02869	0.051394	-0.28914	-0.2214	NA	NA
NDUFB1	-0.15598	-0.49443	-0.18185	-0.27571	-0.38438	-0.28191	-0.24709	0.007197

NDUFB10	-0.00282	0.042144	-0.09683	0.039024	0.003676	-0.3601	-0.57379	-0.59378
NDUFB11	0.076004	-0.0365	0.060329	0.088286	-0.18322	-0.15894	0.166426	-0.19732
NDUFB2	0.249299	-0.2667	-0.00356	-0.10667	-0.07496	-0.30037	-0.00927	-0.08377
NDUFB3	-0.28943	-0.72614	0.040238	-0.21684	-0.19122	-0.28578	-0.07054	-0.21116
NDUFB4	NA	NA	-0.15444	-0.07183	-0.25351	-0.35747	-0.41417	-0.13979
NDUFB5	-0.06344	-0.52278	-0.02168	-0.24706	-0.29724	-0.21415	0.20451	0.114982
NDUFB6	0.250396	-0.42997	-0.21371	-0.0198	-0.19083	-0.46852	-0.31306	-0.05463
NDUFB7	0.410007	0.221541	0.23244	-0.0108	-0.08188	-0.3383	-0.00247	-0.14822
NDUFB8	0.110764	0.463744	-0.0531	-0.2491	0.009253	-0.31821	-0.25257	0.108756
NDUFB9	-0.14569	-0.45408	-0.08728	-0.12428	-0.49288	-0.46993	NA	NA
NDUFC1	-0.36619	-0.35262	0.188913	-0.1555	-0.26801	-0.22579	-0.276	-0.33337
NDUFC2	0.233768	0.236566	-0.24303	-0.11433	0.181666	-0.50629	-0.00287	-0.22855
NDUFS1	0.339029	-0.33546	-0.1949	-0.15303	-0.18988	-0.20322	0.388031	-0.08741
NDUFS2	-0.14121	0.334269	0.038601	0.113111	-0.17191	-0.11935	0.162957	-0.12518
NDUFS3	-0.15959	0.025732	-0.03876	-0.04247	-0.0721	-0.19861	-0.33404	-0.34126
NDUFS4	-0.05331	-0.28608	-0.16051	-0.47809	-0.2235	-0.12801	-0.38601	-0.39275
NDUFS5	-0.28648	-0.62936	0.079253	-0.42535	-0.04358	-0.38616	-0.08012	-0.24649
NDUFS6	-0.00858	0.555823	-0.00598	0.212885	0.088337	-0.15524	-0.17349	-0.14143
NDUFS7	0.143876	0.746554	0.121474	-0.04618	0.016502	-0.13266	-0.15616	-0.11118
NDUFS8	-0.13536	0.593144	0.354453	0.010746	-0.0663	0.022096	-0.22707	-0.2011
NDUFV1	-0.23299	-0.15891	0.386378	0.081258	0.226632	0.230066	0.328033	0.257825
NDUFV2	-0.00192	0.394933	-0.14296	-0.14842	-0.22214	-0.13467	-0.30744	-0.18409
NDUFV3	0.279576	0.113361	-0.02302	0.101533	-0.15717	0.092139	-0.09064	-0.08427
NEB	0.179063	0.034675	-0.15688	0.150656	-0.1701	0.144327	0.095009	0.412665
NEBL	0.02101	0.308515	-0.21558	0.325752	0.075055	-0.17968	0.38551	-0.14498
NECAB1	NA	NA	0.364508	-0.19309	-0.28484	-0.10843	NA	NA
NECAB2	NA	NA	0.155017	-0.03373	-0.02698	0.343782	NA	NA
NECAB3	NA	NA	0.46777	-0.26532	0.377177	-0.42675	NA	NA
NECAP1	-0.16056	-0.32878	-0.1253	0.04123	-0.15084	-0.53996	0.018915	0.371095
NECAP2	-0.25417	-0.16551	-0.03243	0.309499	0.031127	0.078554	0.349161	0.456596
NEDD1	-0.16445	-0.57196	-0.2196	0.061469	-0.07256	-0.21059	-0.00525	-0.38866
NEDD4	0.274632	-0.20813	0.420831	-0.09226	0.251819	0.216586	0.264914	0.483444
NEDD4L	-0.14559	-0.08426	-0.05825	-0.3888	0.151539	0.094742	0.336589	-0.01526
NEDD8	-0.04082	-0.67618	-0.27826	0.070415	-0.18827	0.170396	-0.14081	-0.01613
NEDD9	-0.09177	-0.08335	-0.441	-0.06363	-0.25067	-0.3518	0.092157	-0.10353
NEFH	-0.15056	0.216468	0.031514	0.139489	0.149155	0.558402	-0.58717	-0.1974
NEFL	0.364625	0.316389	-0.28281	-0.08764	-0.24032	0.047824	0.154974	-0.07691
NEFM	0.125685	0.618597	0.227224	-0.35109	0.081959	-0.11826	-0.2609	-0.19623
NEGR1	0.384724	-0.27658	-0.15765	-0.20918	0.033282	-0.05207	-0.12422	-0.31571
NEIL1	0.217329	0.632299	0.107354	0.159086	0.152947	0.205659	0.250912	-0.23904
NEIL2	0.011778	-0.12953	0.402846	0.376681	0.063621	0.018021	-0.0399	0.020649
NEIL3	0.234552	0.796611	-0.28836	0.091461	-0.04855	-0.13788	-0.21898	-0.26253
NEK1	-0.14443	-0.58202	-0.28648	-0.12221	-0.29503	0.139979	0.101196	0.106634
NEK10	0.002487	0.130624	-0.27498	0.010623	-0.12793	0.011921	-0.01221	0.023453
NEK11	0.137495	0.254502	-0.37724	-0.39959	-0.06747	-0.44281	-0.01098	-0.38364
NEK2	-0.01983	0.099451	-0.42953	0.180473	-0.13916	-0.10342	0.282495	-0.03988
NEK3	0.48437	0.022407	0.020645	-0.10942	0.45083	-0.09037	0.088198	-0.09181
NEK4	-0.07972	-0.43727	-0.50491	-0.00969	0.067124	-0.58629	0.453624	0.354251
NEK5	NA	NA	NA	NA	NA	NA	-0.04633	-0.24174
NEK6	0.033324	0.26916	0.014266	0.371929	0.154409	0.055479	0.00368	0.246014
NEK7	0.030529	0.330879	-0.35911	-0.18695	-0.34281	-0.36137	0.142108	0.199
NEK8	-0.30069	-0.1814	0.287361	-0.00988	0.35037	0.000274	-0.13278	-0.19588

NEK9	-0.01887	-0.14826	-0.14097	0.028923	0.103616	0.199017	0.146353	0.298861
NELF	-0.2342	0.64557	0.081536	0.024275	0.271321	0.073346	-0.44385	-0.24614
NELL1	0.105306	-0.30659	-0.15768	0.012468	-0.07197	-0.07308	-0.05309	-0.15283
NELL2	0.131002	-0.29825	0.223038	0.00558	-0.35492	0.045499	0.277772	0.18865
NENF	0.154572	0.205335	0.206177	0.055351	-0.25477	-0.34857	-0.03472	-0.33699
NEO1	0.004768	0.390139	0.079001	0.087498	0.095872	0.109999	-0.27282	-0.42534
NES	0.035091	0.165569	-0.12707	0.157376	0.165711	0.375453	0.302316	0.143121
NET1	-0.30266	-0.07972	-0.41197	-0.27001	0.068048	-0.27213	0.512039	0.229694
NETO1	0.262628	0.768793	0.121485	0.224027	0.095742	0.04386	-0.36187	-0.34017
NETO2	0.195817	0.152788	0.004286	0.037684	0.286849	0.145062	0.248302	0.013157
NEU1	0.317318	-0.04622	0.187129	0.015625	0.165686	-0.0112	0.50365	0.309305
NEU2	-0.03406	0.397267	-0.26787	-0.44652	-0.0391	-0.00935	0.359513	0.1942
NEU3	-0.3315	-0.0123	0.175523	-0.25017	-0.24721	0.245282	0.216924	0.518898
NEU4	-0.54363	0.20209	-0.0236	0.084078	-0.04217	-0.24532	0.049862	-0.10948
NEURL	0.092351	0.739507	-0.14371	0.193837	-0.04038	-0.39426	0.319489	0.363608
NEURL2	0.364108	-0.44418	0.179234	-0.39246	0.327225	-0.38442	0.135301	0.448941
NEUROD1	0.034152	0.09589	0.061062	0.008136	-0.11573	-0.14325	0.309823	0.303227
NEUROD2	0.404354	0.765381	0.22493	-0.10674	0.181959	0.116953	0.398057	0.122248
NEUROD4	0.097435	0.188268	-0.19653	-0.06495	-0.08198	0.015919	-0.2093	0.415274
NEUROD6	-0.07455	0.079177	-0.03043	-0.3293	0.329715	0.131505	-0.01065	-0.17851
NEUROG1	-0.0345	0.125435	-0.15873	-0.00271	-0.1297	0.208896	0.483604	0.132949
NEUROG2	0.126393	0.458862	-0.00165	-0.03887	0.322264	-0.29431	0.190989	0.325389
NEUROG3	-0.02407	0.441554	0.173617	-0.11553	-0.07787	0.158674	0.202081	-0.06271
NEXN	-0.12647	-0.10005	0.028091	0.047871	-0.14563	-0.00851	-0.31627	-0.34913
NF1	0.336925	-0.22018	-0.05084	-0.04325	-0.04784	-0.35792	0.381464	0.401592
NF2	-0.14077	0.684885	0.24071	0.392911	0.391552	0.044285	0.449776	0.407718
NFAM1	0.433604	0.592735	0.076107	0.003727	-0.26667	0.159709	-0.25695	0.123732
NFASC	0.25122	0.356236	NA	NA	NA	NA	-0.35841	-0.18984
NFAT5	0.219251	-0.13235	0.262377	-0.34805	0.240285	0.439077	-0.19067	-0.27511
NFATC1	-0.42998	0.090682	0.080415	0.634114	-0.1597	0.771335	-0.65721	-0.2243
NFATC2	0.403967	0.443919	-0.23118	-0.10507	-0.09249	0.346203	0.434074	0.116471
NFATC2IP	NA	NA	-0.0898	0.213594	0.442561	0.190521	-0.37564	-0.0564
NFATC3	0.341413	-0.01823	0.31385	0.268519	0.497757	0.616268	0.16715	0.34425
NFATC4	0.223005	0.20461	0.35717	-0.17214	0.000611	-0.08699	-0.27	-0.15904
NFE2	-0.09902	0.343839	0.257482	0.119829	-0.02784	-0.37402	0.17481	0.231887
NFE2L1	-0.17672	-0.17327	-0.00971	0.152256	0.112314	0.125365	0.037133	0.220345
NFE2L2	-0.07677	-0.62807	-0.08146	-0.2532	-0.02614	-0.20146	-0.05636	0.207109
NFE2L3	0.40654	-0.275	-0.11592	0.110913	0.131269	0.410836	0.223466	0.062649
NFIA	0.111978	-0.13752	0.269066	0.018436	0.03224	0.132837	-0.08007	-0.41709
NFIB	0.1606	-0.08393	-0.13853	0.084179	0.097996	0.111791	-0.34706	0.010351
NFIC	0.040952	0.136072	0.047427	-0.11337	0.051884	0.079555	-0.24022	-0.24615
NFIL3	-0.38816	-0.07619	-0.12719	-0.02249	0.023209	-0.08103	-0.43984	-0.28331
NFIX	0.522897	-0.10676	0.289064	-0.02828	0.448758	0.485626	-0.14117	-0.11956
NFKB1	-0.28803	-0.13509	-0.08226	0.229502	0.090623	0.409444	-0.36127	0.263858
NFKB2	-0.15933	0.325795	0.210274	0.277093	0.089705	0.475089	-0.50706	0.008879
NFKBIA	-0.36268	0.091992	-0.40379	0.429747	0.106571	0.086561	NA	NA
NFKBIB	0.370295	0.33029	0.139935	0.199733	0.123798	-0.1244	-0.03318	-0.27403
NFKBID	NA	NA	0.020786	0.178976	0.043198	-0.10983	NA	NA
NFKBIE	0.214576	0.458649	-0.16676	0.473303	-0.12598	0.163536	-0.60045	-0.16094
NFKBIL1	0.363134	0.750598	0.185442	0.08597	0.136154	-0.2298	0.196502	0.061436
NFKBIL2	0.283521	0.799245	0.156178	0.254465	0.083408	-0.08325	0.40939	0.52114
NFKBIZ	-0.39167	0.292339	0.067216	0.249821	0.096049	0.01154	-0.32582	0.055134

NFRKB	-0.07973	-0.46183	0.128061	-0.01435	0.331843	0.209785	0.427255	0.380994
NFS1	0.354089	-0.17687	0.197524	-0.0533	0.171956	0.043177	-0.61663	0.036106
NFU1	0.122514	-0.62221	-0.10221	-0.03131	-0.20912	-0.31169	0.088163	-0.14251
NFX1	-0.04445	0.616286	0.02622	-0.11134	0.27844	0.207919	-0.48689	-0.16965
NFXL1	0.218853	0.28244	-0.22781	-0.12854	0.001294	0.026105	0.122265	-0.11156
NFYA	0.163386	-0.09989	-0.40209	-0.01813	-0.24128	-0.21808	0.507682	0.456844
NFYB	0.309481	0.786282	-0.28608	0.007774	-2.3E-05	-0.16176	-0.7153	-0.61366
NFYC	-0.34087	-0.25695	0.002615	-0.03324	0.112872	-0.34113	0.381044	0.419768
NGB	-0.1107	-0.01785	0.026108	-0.14683	-0.12428	0.097581	-0.61984	-0.41727
NGDN	NA	NA	-0.32073	-0.14652	-0.04429	0.237011	0.215075	0.598444
NGEF	0.529297	0.122447	0.00688	0.070733	0.414558	-0.28167	0.298392	0.145684
NGF	NA	NA	0.206855	-0.30726	-0.09316	-0.16564	NA	NA
NGFR	0.076323	0.032583	-0.08085	0.269505	0.058619	0.228914	-0.61371	-0.10157
NGFRAP1	-0.0009	-0.33064	-0.58197	-0.02278	-0.21733	-0.32218	-0.23377	-0.44048
NGLY1	0.010455	-0.54871	-0.10316	0.086642	0.061442	0.31994	-0.1667	-0.30273
NGRN	-0.10002	-0.49385	-0.10023	0.197898	0.085282	0.144848	0.168681	-0.0075
NHEDC1	-0.4213	0.190761	0.014445	-0.1788	-0.15287	0.046282	NA	NA
NHEJ1	0.561831	0.791834	0.123739	0.05107	-0.12758	-0.28435	NA	NA
NHLH1	-0.08926	0.531968	-0.30374	-0.0972	0.022	0.263506	0.073381	0.181022
NHLH2	0.076417	0.127877	0.392279	-0.01903	0.123772	0.153694	-0.02007	-0.14825
NHLRC1	-0.03032	0.02418	NA	NA	NA	NA	0.120755	0.045313
NHLRC2	-0.15477	0.489821	-0.40198	-0.35247	0.055282	-0.278	0.295008	0.349974
NHLRC3	NA	NA	0.042528	-0.25291	0.276347	-0.02654	NA	NA
NHP2	NA	NA	0.315609	0.388912	-0.03652	0.066922	NA	NA
NHP2L1	-0.26252	-0.5539	0.170218	0.218884	-0.03579	0.136687	0.127198	-0.31945
NHS	0.411821	0.557356	0.268107	-0.06764	0.007345	0.424646	0.346042	0.454285
NICN1	0.176899	0.316404	0.001094	0.011294	0.1692	0.014605	-0.13829	-0.04299
NID1	-0.00862	-0.40311	-0.01418	0.031316	0.075795	0.25746	0.052638	0.072989
NID2	-0.03476	-0.43769	-0.17078	-0.11177	-0.09114	0.167465	-0.49272	-0.18332
NIF3L1	0.255748	-0.06694	-0.14597	-0.19319	-0.33216	-0.19667	0.077209	-0.16285
NIN	-0.06412	-0.29057	0.133805	0.283074	-0.24872	0.513689	-0.46262	0.104716
NINJ1	-0.32608	0.067867	0.398054	-0.09951	-0.18872	0.11291	-0.34253	0.108062
NINJ2	0.200356	0.468312	-0.37668	-0.03358	-0.12163	0.054914	-0.19712	0.079654
NIP30	0.572474	0.172998	-0.17067	-0.09111	0.161104	-0.43675	0.649129	0.490449
NIP7	0.381936	0.429302	-0.05631	-0.08652	0.145116	0.177979	0.220383	-0.19594
NIPA1	-0.26173	-0.05658	-0.02254	0.086301	0.132123	-0.13358	-0.53434	-0.38723
NIPA2	0.0198	-0.12166	-0.17366	-0.00547	-0.16678	-0.14973	-0.05376	0.205302
NIPBL	0.305993	0.421415	-0.22225	0.012321	-0.2456	0.063721	0.498173	0.138218
NIPSNAP1	0.261261	0.472267	0.252189	0.140708	0.110969	-0.12278	0.460048	0.021334
NIPSNAP3A	0.001268	-0.27122	-0.00983	-0.54237	-0.03201	-0.20761	-0.66143	-0.50482
NIPSNAP3B	0.006395	-0.43548	-0.04679	-0.4254	-0.0547	-0.25677	0.159948	-0.59747
NISCH	-0.10932	0.082698	0.152691	0.001002	0.337197	0.428024	-0.08006	0.141842
NIT1	0.101412	0.345562	0.248177	-0.11531	0.018622	-0.14667	-0.04455	0.060412
NIT2	0.450206	0.330431	-0.17592	-0.1206	-0.14001	-0.37108	0.160214	-0.04127
NKAIN1	NA	NA	-0.09983	-0.16327	0.054215	-0.12365	NA	NA
NKAIN2	NA	NA	0.087889	0.099013	-0.20017	0.1028	NA	NA
NKAIN3	NA	NA	0.027406	-0.29738	-0.20868	0.22092	NA	NA
NKAIN4	NA	NA	-0.04127	-0.14539	0.301979	-0.03791	NA	NA
NKAP	0.080643	-0.60133	-0.20099	0.275781	-0.0163	-0.3034	-0.30422	-0.29276
NKAPL	NA	NA	0.212912	-0.10616	0.244257	-0.04537	NA	NA
NKD1	0.247911	-0.29118	0.537873	-0.12569	0.394521	0.239911	0.640359	0.113447
NKD2	0.289352	0.073109	0.110599	-0.12902	-0.06625	0.166517	0.366628	0.194667

NKG7	-0.06923	0.193011	0.163328	0.247492	-0.39963	0.078772	-0.4145	0.426485
NKIRAS1	-0.09455	-0.09565	-0.07036	-0.09459	-0.08481	-0.11998	-0.07652	-0.11107
NKIRAS2	-0.10339	0.354344	0.452926	-0.15585	0.202227	-0.0542	-0.17944	-0.27918
NKPD1	0.156039	0.541523	-0.03486	-0.11807	0.235356	0.125945	0.053238	-0.26225
NKRF	0.456674	-0.10669	-0.12286	0.038311	0.09148	0.379958	0.396649	0.077718
NKTR	-0.3426	-0.65905	-0.10404	0.05444	0.186907	0.520608	0.132636	0.406379
NKX1-2	NA	NA	-0.03477	0.036228	-0.18752	0.229493	NA	NA
NKX2-1	NA	NA	-0.11363	0.089474	-0.04609	-0.00794	NA	NA
NKX2-2	-0.37305	0.059669	0.039057	-0.14666	-0.07097	-0.2909	0.313496	0.359672
NKX2-3	-0.16658	-0.09817	0.160631	0.202187	-0.07377	0.051966	-0.08042	0.028737
NKX2-5	0.119183	0.189075	0.031839	-0.10907	0.025334	-0.1365	0.036532	0.143782
NKX2-8	-0.33313	0.278535	0.343539	-0.15473	0.101282	0.104554	-0.06678	-0.00092
NKX3-1	-0.40478	0.066438	-0.11647	0.009368	-0.16122	0.249282	-0.12826	-0.14302
NKX3-2	NA	NA	0.210228	-0.1003	-0.17259	0.09096	NA	NA
NKX6-1	-0.00343	0.550328	0.211426	0.299948	-0.11203	-0.19586	-0.42009	-0.08716
NKX6-2	-0.00589	0.547697	0.029056	-0.14634	0.274538	0.126352	0.005442	0.190629
NKX6-3	NA	NA	-0.18936	0.465024	-0.4114	0.007973	-0.06036	-0.18787
NLE1	0.426451	0.698684	0.47174	0.24439	0.257202	0.275056	0.289924	0.114747
NLGN1	-0.01369	0.071451	0.233128	-0.23422	0.167732	-0.32997	-0.27328	-0.31584
NLGN2	-0.35228	0.072562	0.111611	0.198151	-0.02044	0.535662	-0.19472	-0.11665
NLGN3	0.258386	0.459764	0.000476	0.138945	-0.20608	0.23059	-0.29579	-0.09426
NLGN4X	-0.32283	-0.50758	-0.09744	0.432135	-0.32673	0.124315	-0.18511	-0.27306
NLGN4Y	0.210735	0.015089	-0.09143	-0.09349	-0.26688	0.096694	0.047677	-0.16747
NLK	0.208154	-0.52424	-0.08991	0.02528	-0.23872	-0.48064	-0.31359	-0.09972
NLN	0.010389	-0.28688	-0.24204	-0.43744	-0.23273	-0.32412	0.359587	0.37315
NLRC3	0.400169	0.711668	0.018515	0.61485	-0.16027	0.72515	NA	NA
NLRC4	-0.33083	0.004523	-0.00365	0.077346	-0.23792	-0.00336	NA	NA
NLRC5	-0.09934	0.09156	0.173973	0.020724	0.248289	0.315287	NA	NA
NLRP1	NA	NA	0.033683	0.491307	-0.01142	0.663538	-0.30662	0.20258
NLRP10	0.092867	0.419463	0.035509	-0.30761	-0.03568	-0.33954	NA	NA
NLRP11	0.661724	0.463297	-0.19292	0.014247	-0.18949	-0.00657	NA	NA
NLRP12	0.297958	0.388115	0.159071	-0.22678	0.021479	0.054435	NA	NA
NLRP13	0.225939	0.363874	0.171544	-0.13935	0.046202	-0.09085	NA	NA
NLRP14	-0.01058	-0.3636	-0.2008	0.037833	0.096114	0.019261	NA	NA
NLRP2	-0.32606	-0.16808	-0.15176	-0.08804	-0.04637	-0.19366	NA	NA
NLRP3	-0.36957	-0.0111	0.053956	-0.05598	0.063441	0.217599	-0.4413	0.212821
NLRP4	-0.11539	0.413072	-0.10231	0.451402	0.059777	0.304689	NA	NA
NLRP5	0.144337	0.398034	-0.30037	-0.1413	-0.13638	0.113583	NA	NA
NLRP6	0.168546	0.275572	0.246902	-0.11105	0.207887	0.028455	NA	NA
NLRP7	-0.16344	-0.04484	0.011204	0.222829	0.001681	-0.13815	NA	NA
NLRP8	-0.10038	0.360083	-0.16153	0.037655	-0.41949	0.134814	-0.56476	-0.57555
NLRP9	0.322448	0.61562	0.20873	0.114697	0.296286	0.188726	NA	NA
NLRX1	0.014918	0.550114	0.067556	-0.09711	-0.07863	-0.14212	NA	NA
NMB	0.390984	-0.31536	-0.04514	0.122193	0.001215	-0.22415	0.556284	0.315389
NMBR	0.333972	0.676769	0.293456	0.02824	-0.06677	-0.20362	0.438549	0.057804
NMD3	-0.18031	-0.69793	-0.21054	-0.32643	-0.24745	-0.29574	0.059359	-0.2309
NME1	-0.08769	-0.28286	0.353628	0.273402	-0.16714	-0.42599	0.346239	0.069169
NME1-NME	-0.21128	-0.5702	NA	NA	NA	NA	0.054368	-0.19534
NME2	0.250166	-0.47758	0.198207	0.349602	-0.09641	-0.08767	-0.45207	-0.57697
NME3	-0.20894	0.44642	0.08571	0.128214	0.066426	-0.0474	0.091452	0.098997
NME4	0.179009	-0.04963	0.429377	0.023753	0.186387	-0.04563	0.177962	-0.14517
NME5	0.503927	-0.22537	0.126722	-0.41952	-0.22854	-0.19789	-0.17333	-0.11368

NME6	-0.12108	-0.24028	0.133554	-0.17844	0.217145	-0.02289	0.069064	0.197638
NME7	0.055953	-0.18971	0.053985	-0.37367	-0.26339	-0.12189	-0.16823	-0.35986
NMI	0.300023	-0.31971	-0.0819	-0.02638	-0.07725	-0.04957	-0.38554	-0.21366
NMNAT1	-0.14108	0.014764	-0.02217	-0.14222	-0.14291	-0.35013	-0.15825	0.372718
NMNAT2	0.131763	-0.12388	-0.1103	0.091529	-0.13629	0.147134	0.083851	-0.04555
NMNAT3	-0.05066	-0.31543	0.56446	-0.17275	0.111614	0.158352	0.223688	-0.2088
NMRAL1	0.022114	0.7098	0.259398	0.228644	0.340846	0.166561	NA	NA
NMS	0.045476	0.126992	NA	NA	NA	NA	-0.0791	-0.01602
NMT1	0.19192	0.468886	0.212889	0.48743	0.115285	0.016486	-0.56119	-0.15925
NMT2	0.073075	0.114127	-0.17033	-0.17435	-0.01669	-0.25137	0.079402	0.138472
NMU	-0.26044	-0.45834	0.026825	0.19414	-0.17517	-0.16917	0.176088	0.311929
NMUR1	-0.11786	0.05115	0.008635	-0.23513	-0.07709	0.069361	0.149102	0.598584
NMUR2	-0.40556	0.218437	0.156029	-0.19616	-0.17425	0.022332	0.091901	0.033679
NNAT	0.136216	-0.05709	-0.24594	-0.11968	0.274675	-0.18294	-0.24666	-0.24779
NNMT	-0.41889	-0.29156	0.138509	0.182163	0.061167	0.354078	-0.3936	-0.04738
NNT	0.025117	-0.29912	-0.3421	-0.01547	0.016174	-0.33387	0.108444	-0.43146
NOB1	0.443171	-0.29999	0.283552	0.050257	0.272024	0.212067	NA	NA
NOBOX	NA	NA	NA	NA	NA	NA	0.18733	0.393851
NOC2L	0.068158	0.444254	0.284093	0.469768	0.316467	0.383798	0.294509	-0.1062
NOC3L	0.131176	-0.24109	-0.31776	-0.00078	0.088254	0.293422	-0.0221	0.207464
NOC4L	0.279217	0.538791	0.303964	0.095977	0.37444	0.203102	0.241774	0.001059
NOD1	0.122257	0.441386	0.305636	0.049426	-0.00968	0.318338	0.05043	0.398151
NOD2	-0.10303	-0.24844	0.236005	0.165768	0.34533	0.093351	NA	NA
NODAL	0.187245	0.191665	-0.01622	0.117626	0.090354	-0.09097	-0.10485	-0.2751
NOG	0.118035	-0.07831	0.209192	-0.18019	-0.14019	0.081982	0.47575	-0.12802
NOL10	0.230527	0.30549	-0.23314	-0.1575	0.130461	-0.02137	-0.01193	-0.27161
NOL11	0.386535	-0.27308	-0.00013	-0.17227	-0.06859	0.084273	0.123962	-0.17366
NOL12	NA	NA	-0.03132	0.152607	0.259407	-0.23323	0.226329	0.118523
NOL3	-0.34771	-0.17524	-0.08585	0.009248	0.377487	-0.09758	0.179122	-0.00765
NOL4	0.348404	-0.37149	0.031605	0.018079	-0.24587	-0.20043	0.277327	0.049385
NOL6	-0.02499	-0.14183	0.117612	0.276795	0.248976	-0.06239	-0.13128	0.025386
NOL7	-0.0331	-0.56552	-0.24581	0.294591	-0.09053	-0.09014	-0.04316	-0.12546
NOL8	0.289151	-0.59512	-0.12353	0.044601	-0.07852	0.278213	-0.01429	0.137407
NOL9	0.047101	0.264544	-0.0715	0.142322	0.183075	0.384176	0.214031	-0.07062
NOLC1	0.137037	-0.3209	-0.37495	0.306107	0.173417	0.359192	0.36931	0.414961
NOM1	0.157452	-0.24346	-0.01723	-0.0877	0.072319	0.13169	-0.69555	-0.57117
NOMO1	-0.31189	-0.65929	0.210712	0.117674	0.397387	0.134372	0.095486	-0.33805
NOMO2	-0.01369	0.354216	NA	NA	NA	NA	-0.54409	-0.43628
NOMO3	-0.14453	-0.06678	NA	NA	NA	NA	0.436758	0.237216
NONO	0.529802	0.023166	0.018096	0.382062	0.221464	0.263398	0.227518	-0.03779
NOP10	NA	NA	-0.08454	-0.3195	-0.56722	-0.27214	NA	NA
NOP14	NA	NA	0.01909	0.317559	0.313026	0.286397	NA	NA
NOP16	NA	NA	0.123986	0.015346	0.080903	0.195292	NA	NA
NOP2	NA	NA	-0.01784	0.221447	0.109054	0.347557	NA	NA
NOP5/NOP	0.554615	0.141842	0.057901	0.203122	0.126762	0.343261	-0.52921	-0.48184
NOP56	NA	NA	-0.27973	0.335622	-0.02042	0.21631	NA	NA
NOS1	0.23994	0.200603	0.217742	0.143394	-0.09346	-0.28324	0.306209	0.379039
NOS1AP	-0.06447	0.227059	0.012403	-0.08585	0.042364	-0.33509	0.151849	-0.36767
NOS2	NA	NA	0.26265	0.215453	-0.02725	0.213948	NA	NA
NOS3	0.074078	0.146802	0.1741	-0.25876	-0.32946	-0.22051	-0.04097	0.05453
NOSIP	0.168669	0.567054	0.020576	-0.07531	0.192424	-0.17928	-0.01692	-0.3704
NOSTRIN	-0.15622	0.066097	0.079589	-0.09093	-0.09719	-0.02319	0.261721	0.409286

NOTCH1	-0.19713	-0.20459	0.218528	0.221857	0.444048	0.287816	-0.18432	-0.37606
NOTCH2	0.36483	0.391734	-0.30569	0.184869	-0.03349	0.282484	-0.21211	0.052285
NOTCH2NL	-0.16715	0.054127	-0.17533	-0.29397	-0.07957	0.335747	0.117071	-0.24751
NOTCH3	-0.29378	-0.50335	0.131266	0.028936	-0.04905	-0.23372	-0.16645	-0.09985
NOTCH4	-0.29595	0.127948	-0.03059	0.079484	0.111543	0.317109	0.000376	0.354142
NOTUM	0.187778	0.436012	0.459364	0.003928	0.210876	-0.05545	0.682973	0.309898
NOV	-0.30028	-0.45417	-0.23433	0.273415	-0.43305	-0.02925	-0.03854	-0.11809
NOVA1	0.124772	-0.30541	0.46261	-0.02749	0.048814	0.293373	-0.32737	-0.34043
NOVA2	-0.06146	0.246136	0.130672	-0.09281	-0.04298	-0.09337	-0.16336	-0.40439
NOX1	0.286853	0.202436	-0.12893	0.00526	0.160751	-0.16912	0.032532	-0.03409
NOX3	0.263764	0.596308	-0.1261	0.32305	-0.10529	-0.21353	0.39488	0.029591
NOX4	-0.21388	-0.18745	0.060952	-0.01221	-0.13002	-0.10298	-0.0424	0.146333
NOX5	-0.28323	0.202541	-0.14198	-0.24581	0.179258	0.003623	0.066266	0.34235
NOXA1	-0.16943	0.35569	-0.05342	-0.13163	-0.01358	-0.24213	0.328326	0.397734
NOXO1	-0.06439	0.220283	-0.4667	0.138571	0.181904	-0.34339	0.289727	-0.11734
NP	-0.23459	0.042194	-0.23934	-0.1181	-0.09221	-0.31544	0.180444	0.388193
NPAL2	-0.09135	-0.1947	0.128996	-0.2234	-0.07328	-0.18155	0.282784	0.07478
NPAL3	0.007856	0.074051	0.02629	-0.13454	0.206588	-0.00664	0.072406	0.159409
NPAS1	-0.23119	0.732049	-0.03751	0.058042	-0.13271	-0.19824	0.135149	-0.1564
NPAS2	-0.00317	-0.41424	0.248167	0.106495	0.361995	-0.15615	0.265581	-0.04791
NPAS3	-0.24243	0.351954	-0.04185	-0.04954	-0.16425	0.224118	-0.16593	0.004791
NPAS4	0.059976	0.368428	-0.07616	-0.20151	-0.02392	-0.38109	-0.34757	-0.1047
NPAT	0.163687	-0.32597	-0.07275	-0.03465	-0.32382	0.118075	-0.32822	-0.18607
NPB	0.17442	0.702758	0.164216	0.000581	-0.02639	-0.10631	-0.47337	-0.11664
NPBWR1	-0.17807	-0.12214	-0.14696	-0.04568	-0.02377	0.103448	0.377006	-0.17509
NPBWR2	-0.06822	0.333459	0.154593	-0.10838	-0.03501	-0.19551	-0.35399	0.018727
NPC1	-0.03588	-0.17427	-0.12873	-0.1622	-0.15035	0.326298	-0.12224	0.421076
NPC1L1	0.186772	0.339899	-0.2672	-0.24091	-0.04914	-0.31954	0.273579	0.084487
NPC2	-0.21558	0.048897	0.192887	-0.3612	-0.34916	0.04673	-0.06078	0.161352
NPDC1	-0.24583	0.357875	-0.00914	0.202824	0.202745	0.100256	0.318285	0.013605
NPEPL1	0.157504	0.173898	0.061906	0.172148	0.311848	0.21368	-0.04869	-0.15487
NPEPPS	-0.16068	-0.41538	0.094027	-0.18979	-0.09649	0.173034	0.28091	-0.02995
NPFF	-0.07542	0.489736	-0.13628	0.035162	0.110623	0.102648	-0.35672	0.001627
NPFFR1	0.409276	-0.23345	0.092397	-0.14099	0.128427	-0.11485	-0.1868	-0.22616
NPFFR2	-0.1678	0.181392	0.028907	0.492536	0.081616	0.443136	0.32559	0.283273
NPHP1	0.2824	-0.29122	0.10869	-0.05151	0.131404	0.308574	-0.30451	-0.34504
NPHP3	-0.24238	-0.67166	-0.165	-0.20533	0.251502	0.15054	0.335488	0.548345
NPHP4	0.07976	0.453192	-0.27374	0.128604	0.106751	-0.06115	0.276855	0.471463
NPHS1	0.042852	0.606685	-0.26166	0.698288	0.138265	0.572311	-0.09865	-0.02157
NPHS2	0.43842	0.61002	-0.16746	0.139858	-0.06178	-0.47519	-0.24263	-0.08711
NPIP	-0.17699	-0.54733	-0.0579	0.073475	0.295041	0.402409	-0.20554	-0.00031
NPL	-0.26453	-0.04325	0.036747	0.01466	-0.21584	0.030318	-0.13716	0.056353
NPLOC4	-0.12053	0.615919	0.454229	-0.13577	0.359455	0.390534	0.276383	0.208567
NPM1	NA	NA	0.197499	0.294251	0.051315	0.06176	0.189184	0.030897
NPM2	0.40461	0.147588	0.019995	-0.0381	-0.09067	-0.04974	0.30785	0.164581
NPM3	0.326548	0.688246	-0.11771	0.468874	0.076175	-0.18707	0.353563	-0.00787
NPNT	-0.44538	-0.48073	0.183819	-0.03312	0.002944	0.036316	0.005617	-0.1601
NPPA	-0.38269	-0.51517	-0.00334	-0.13764	0.136172	0.321539	-0.37446	-0.25491
NPPB	-0.19546	0.347793	0.130761	-0.1961	0.267517	-0.10759	-0.21025	-0.13513
NPPC	0.319949	0.106743	0.268935	0.218469	-0.06003	0.332249	-0.49013	-0.08122
NPR1	0.105786	0.724013	0.203267	0.284555	0.161144	0.458811	-0.62001	-0.2369
NPR2	-0.02985	-0.33513	0.088906	-0.06048	-0.20582	0.243547	-0.23324	0.035861

NPR3	0.006134	0.199791	0.239546	-0.07864	-0.32116	-0.20575	-0.44111	-0.22196
NPSR1	-0.19555	0.513027	-0.18857	-0.04482	0.081982	-0.34882	NA	NA
NPTN	-0.223	-0.61976	-0.10242	-0.07677	0.089768	-0.14564	-0.11877	0.047212
NPTX1	0.17662	-0.08789	-0.02892	-0.09432	-0.04234	0.128065	-0.21729	-0.369
NPTX2	0.220737	-0.38157	0.261568	-0.06006	-0.05531	0.134983	0.153547	-0.01774
NPTXR	0.209182	0.609505	0.156543	-0.17248	0.405821	0.14013	0.092734	-0.39164
NPVF	-0.04901	0.252499	0.104719	-0.02138	-0.03209	0.197841	NA	NA
NPW	NA	NA	-0.23771	0.152198	-0.24796	-0.06747	NA	NA
NPY	0.108853	0.032561	-0.15685	-0.41595	-0.08564	0.147736	-0.23282	0.124419
NPY1R	-0.25262	-0.16301	-0.16963	-0.54386	-0.24712	-0.23383	NA	NA
NPY2R	0.223886	-0.10896	0.223012	-0.06165	0.103634	-0.1924	0.290238	-0.04187
NPY5R	-0.03526	0.258517	-0.15698	-0.45247	-0.31909	0.014421	0.050756	-0.20024
NPY6R	NA	NA	-0.08263	0.302966	0.280238	-0.00532	0.097169	0.490127
NQO1	-0.11106	0.151432	-0.11169	-0.42706	0.181507	-0.638	0.182025	-0.04479
NQO2	-0.23985	-0.41083	0.17933	-0.00181	-0.09622	-0.09758	-0.40521	-0.03474
NR0B1	-0.06625	-0.28916	0.039673	-0.03201	-0.26207	-0.06366	0.310431	0.573477
NR0B2	-0.1541	-0.05575	-0.01496	-0.07769	0.116451	-0.2295	0.390745	0.545699
NR1D1	0.278651	0.178128	0.398202	-0.30012	0.361508	0.129466	0.014208	-0.21254
NR1D2	0.266302	-0.41204	0.053542	-0.4817	0.334779	-0.06373	0.382577	-0.12961
NR1H2	0.094976	0.673529	0.265473	-0.15125	0.062873	-0.46111	-0.04888	0.085692
NR1H3	0.185225	0.43028	0.358777	0.14861	-0.28845	-0.01348	-0.27565	-0.14556
NR1H4	-0.15713	0.055564	0.012423	-0.20106	-0.18716	-0.0835	0.22895	0.315768
NR1I2	0.215927	0.314327	0.199196	-0.07657	0.277246	0.050952	-0.00081	0.290618
NR1I3	0.150374	0.325101	-0.05404	-0.38231	0.303255	-0.16234	0.360621	0.43956
NR2C1	-0.24835	-0.49183	-0.31701	-0.33957	0.184896	-0.09021	0.086178	0.164065
NR2C2	0.216645	0.427622	-0.25792	-0.14364	-0.25109	-0.04102	0.355556	0.372164
NR2C2AP	NA	NA	-0.08681	0.230803	0.284519	0.163549	NA	NA
NR2E1	0.299443	0.629318	0.085849	0.056271	0.25685	0.277832	-0.12062	-0.09179
NR2E3	-0.21287	0.027323	-0.15855	-0.18298	-0.14868	-0.10902	0.119339	0.098096
NR2F1	0.300668	-0.15229	0.110514	0.085062	-0.19388	0.164345	-0.14263	-0.2779
NR2F2	-0.14423	0.080543	-0.24809	0.193769	0.080705	0.279799	-0.12761	-0.50951
NR2F6	0.160233	0.263965	0.113963	-0.08864	0.320919	-0.19264	0.25507	-0.14566
NR3C1	-0.23215	-0.28529	-0.04487	-0.11949	-0.42577	0.02003	-0.22151	0.184151
NR3C2	-0.22791	-0.17537	-0.11462	-0.12444	-0.20652	-0.07235	-0.0664	-0.20028
NR4A1	0.043987	-0.07661	-0.27504	0.080264	-0.05361	0.029864	-0.05783	0.00181
NR4A2	-0.2527	-0.12025	-0.08325	0.153317	-0.14477	-0.09149	-0.23382	-0.20385
NR4A3	-0.0669	-0.25654	0.003153	-0.0037	-0.07435	0.263089	-0.31889	-0.38261
NR5A1	-0.08569	0.762068	-0.04952	0.094675	-0.05296	-0.19225	0.356826	0.334848
NR5A2	-0.05687	-0.13503	-0.02428	-0.25923	-0.24349	-0.23487	0.028811	0.131869
NR6A1	0.353209	0.331572	0.36642	-0.34399	0.692259	0.10071	0.588164	0.257868
NRAP	-0.40614	0.301618	-0.34266	0.077408	-0.10937	-0.222	0.580225	0.243124
NRAS	0.209146	0.005979	-0.04011	-0.15111	-0.25718	-0.38772	0.288791	0.260674
NRBF2	-0.1466	-0.39309	-0.22345	-0.28717	0.012044	-0.45165	NA	NA
NRBP1	0.111437	0.619501	0.327973	-0.02816	0.308965	-0.08493	-0.49126	-0.50694
NRBP2	-0.16535	-0.1385	0.196176	-0.07671	0.253561	0.199786	0.173236	0.15211
NRCAM	-0.45522	-0.37965	0.23373	0.054671	-0.15458	0.097223	0.015209	-0.35002
NRD1	-0.17714	-0.61044	0.263381	0.106758	0.157969	-0.31824	0.276909	0.347496
NRF1	0.307156	-0.45851	-0.27019	-0.0797	0.185907	0.079876	-0.28705	0.165541
NRG1	0.196957	0.254409	0.034678	-0.33275	0.164453	0.196602	-0.05868	0.186223
NRG2	0.236415	0.246908	-0.01391	0.087862	0.040897	0.130459	-0.04195	-0.01214
NRG3	0.318601	0.203881	NA	NA	NA	NA	-0.43673	-0.44667
NRG4	0.128848	0.429549	0.008185	-0.52605	-0.07463	-0.5409	0.174477	-0.07256

NRGN	-0.17028	-0.11802	-0.17776	0.107789	-0.21534	-0.17341	-0.6061	-0.2019
NRIP1	-0.0475	-0.60553	-0.08654	-0.25784	-0.19831	-0.18024	0.365799	0.195535
NRIP2	-0.22563	-0.5256	-0.06374	0.013723	0.323252	0.300793	-0.38244	-0.06756
NRIP3	-0.37776	0.310935	-0.22722	-0.13987	-0.20383	0.256762	-0.36759	-0.38109
NRK	0.18232	0.475254	NA	NA	NA	NA	0.174681	0.224338
NRL	0.084235	0.505472	-0.39787	0.211318	-0.22051	-0.20506	0.204924	0.23445
NRM	0.123235	0.509672	-0.01496	0.28245	0.108437	0.205683	0.316058	0.263568
NRN1	0.337296	-0.40236	-0.12008	0.022355	0.140638	-0.10632	0.23924	-0.40439
NRN1L	0.063552	-0.0929	0.022956	0.064868	0.077438	-0.02654	NA	NA
NRP1	-0.55838	-0.44152	0.062813	-0.1649	-0.15161	0.143039	-0.3131	0.353473
NRP2	-0.28738	-0.201	0.132916	-0.17644	-0.09498	0.221674	-0.054	-0.27247
NRSN1	-0.06569	-0.16667	0.175213	0.152125	0.242271	0.20742	NA	NA
NRSN2	0.203386	0.122866	0.072857	-0.08613	-0.1574	0.317423	NA	NA
NRTN	0.267512	0.122479	-0.1064	-0.22413	0.059988	0.028936	0.076489	-0.06563
NRXN1	-0.0494	0.124211	-0.13381	-0.20743	-0.22321	0.047678	-0.37337	-0.42732
NRXN2	-0.02283	0.097042	0.114786	-0.10463	0.37783	0.051132	-0.35139	-0.12369
NRXN3	0.084748	-0.25655	0.240454	0.036973	-0.22414	0.234469	0.192285	-0.06485
NS3BP	NA	NA	0.187187	-0.03051	0.34622	0.291485	NA	NA
NSBP1	-0.10978	-0.10596	-0.37469	0.090376	-0.25278	-0.20631	0.090416	0.257863
NSD1	-0.09385	-0.23681	0.234773	0.197193	-0.01247	0.122285	-0.01192	-0.34776
NSDHL	0.354755	0.573566	-0.08829	0.106261	0.173332	0.147407	0.030617	0.070235
NSF	-0.08751	-0.18619	0.049901	0.090445	-0.17809	0.038275	0.19823	0.147367
NSFL1C	0.049035	0.012954	-0.21872	0.191215	0.034755	0.014477	0.234131	0.277177
NSL1	0.146458	0.26468	-0.24858	-0.04952	-0.18608	-0.02444	-0.31666	0.098432
NSMAF	0.128508	0.04233	0.089648	0.03007	-0.08414	0.337193	0.121352	0.147266
NSMCE1	0.399577	0.320798	0.438253	0.165113	0.228567	-0.28296	0.363319	-0.20459
NSMCE2	0.271179	0.562271	0.05998	-0.24143	-0.22529	-0.17516	NA	NA
NSMCE4A	-0.144	-0.46148	-0.22337	-0.05352	0.154814	0.068003	NA	NA
NSUN2	-0.35922	-0.42346	-0.15784	0.227643	0.301402	0.435471	0.153975	-0.27116
NSUN3	-0.14545	-0.33101	-0.35139	-0.04342	-0.19321	-0.44755	-0.19303	-0.2619
NSUN4	-0.07564	-0.14137	0.285526	0.056618	0.005318	0.304336	-0.07911	-0.06433
NSUN5	0.175378	0.29659	0.222699	0.196283	0.22531	0.411211	0.482395	0.467205
NSUN5B	0.078949	-0.1615	0.150446	0.316161	0.212911	0.567959	0.618383	0.541288
NSUN5C	0.214509	-0.57663	0.101039	0.363255	0.2502	0.367562	0.122792	0.248482
NSUN6	-0.09984	-0.25817	-0.25796	0.142102	0.134945	-0.02287	-0.29308	-0.36285
NSUN7	0.184731	-0.16714	-0.23009	-0.28418	-0.02496	-0.14079	0.353723	0.14377
NT5C	-0.0656	0.519256	0.328077	0.096575	0.109362	-0.40506	-0.07007	-0.05579
NT5C1A	0.237848	0.729251	-0.11796	-0.17047	0.503719	0.412239	0.344328	0.425704
NT5C1B	0.184647	0.382447	0.012339	0.122999	0.018009	0.100643	0.276143	0.370242
NT5C2	-0.1322	0.372957	-0.17995	-0.11755	0.20184	0.076757	-0.52289	-0.3443
NT5C3	0.229367	-0.32167	0.138423	-0.16511	-0.16389	0.009704	0.323605	0.01556
NT5C3L	0.060734	0.21678	0.192255	0.221592	-0.01544	-0.36615	0.119905	-0.15155
NT5DC1	0.510033	-0.60367	-0.08721	-0.03853	0.140065	-0.28183	-0.21506	-0.19161
NT5DC2	-0.16193	0.421914	0.170449	0.429652	0.127286	-0.36046	0.376342	0.195538
NT5DC3	-0.30319	0.365653	-0.13344	-0.3384	0.275677	-0.05636	0.196999	-0.34611
NT5E	0.028012	-0.82588	-0.40058	-0.06545	-0.13852	-0.33281	-0.07909	-0.36669
NT5M	0.036564	0.311138	0.163249	-0.09256	0.027499	-0.16821	0.141659	-0.15721
NTAN1	-0.21324	-0.51296	-0.00196	-0.23497	-0.47536	-0.42395	-0.37071	-0.50134
NTF3	-0.37778	-0.31564	-0.26493	0.082012	-0.2587	-0.07058	0.056181	-0.12703
NTF4	NA	NA	0.035319	-0.13139	-0.32145	-0.06357	NA	NA
NTHL1	-0.53056	-0.48222	-0.05635	0.190353	0.228445	0.269168	0.498466	-0.33769
NTM	NA	NA	0.02147	-0.02565	-0.26413	-0.01153	NA	NA

NTN1	-0.13259	0.22714	-0.34868	0.010257	-0.13245	0.130041	0.260952	0.460228
NTN3	NA	NA	0.08873	-0.18224	0.152459	-0.26603	NA	NA
NTN4	0.049848	0.162891	-0.28859	-0.0186	0.046235	-0.20324	-0.18324	0.007324
NTN5	NA	NA	-0.13189	0.226331	-0.22915	0.29455	NA	NA
NTNG1	0.013293	-0.30186	-0.0205	-0.05744	0.041626	-0.01463	-0.4923	0.051997
NTNG2	-0.21913	0.09919	0.170306	0.105643	-0.04581	0.204054	-0.07636	-0.02572
NTRK1	-0.01275	0.325146	0.322277	-0.07562	0.14616	-0.05365	-0.05735	0.31265
NTRK2	-0.25283	-0.12356	-0.10698	0.029634	-0.08984	0.32083	0.07589	0.041208
NTRK3	-0.24451	0.017426	0.216185	-0.12982	0.025316	-0.15594	-0.34482	0.381749
NTS	0.051912	-0.24402	-0.01553	-0.20671	-0.07583	-0.16319	0.179538	0.365584
NTSR1	-0.05573	0.054289	-0.29431	-0.12286	-0.03174	-0.14102	-0.09811	-0.38441
NTSR2	0.314458	0.238033	0.236532	0.131458	-0.47108	0.03296	0.157565	0.246351
NUAK1	-0.2971	-0.17229	0.005815	-0.06008	-0.13909	-0.17452	-0.33365	-0.27421
NUAK2	0.147505	-0.22013	0.002914	-0.03882	0.204489	-0.02033	0.547546	0.401241
NUB1	NA	NA	0.168955	0.020495	-0.11143	-0.1923	0.389352	0.527777
NUBP1	0.153991	0.229556	-0.09965	0.096066	0.085764	0.132171	0.39738	0.323814
NUBP2	0.264574	0.385825	0.130075	0.201229	0.276983	0.107216	0.433923	0.19935
NUBPL	-0.46112	-0.28615	-0.18671	-0.05419	-0.05967	0.019295	-0.49219	-0.49608
NUCB1	-0.05581	0.492473	0.249937	0.21801	0.03884	-0.07229	0.206063	0.580415
NUCB2	-0.36286	-0.21058	-0.01791	0.071693	-0.0937	-0.3813	-0.09398	-0.08783
NUCKS1	-0.22596	0.206573	-0.39128	-0.12803	-0.02478	0.228078	-0.43527	-0.59226
NUDC	0.315532	0.162689	0.136636	0.402133	0.401735	0.243268	0.077355	-0.22626
NUDCD1	0.398638	-0.39953	0.094524	-0.33572	-0.12653	0.065904	0.04425	-0.24425
NUDCD2	-0.2397	-0.06065	-0.01422	0.100044	-0.07959	-0.44746	-0.2268	-0.41221
NUDCD3	0.407022	0.199472	-0.07963	0.17301	0.277754	-0.03244	0.214831	0.042036
NUDT1	0.230403	0.38408	-0.02614	0.172523	0.227009	-0.14114	0.186881	-0.17432
NUDT10	0.094042	0.000841	0.037232	0.126345	0.001943	0.035418	-0.6259	-0.21895
NUDT11	0.214083	-0.33668	-0.24956	-0.00808	0.06267	0.019759	-0.22841	-0.22896
NUDT12	0.213647	-0.03974	-0.1235	-0.30694	-0.03029	-0.55444	-0.54238	-0.63538
NUDT13	-0.27403	-0.18538	0.156197	-0.15573	0.146048	-0.19151	-0.46699	-0.18391
NUDT14	0.220477	0.300264	0.266516	-0.05972	0.024827	0.173163	0.556548	0.192639
NUDT15	0.009467	-0.10496	-0.29253	0.134014	-0.00294	-0.3071	0.01303	-0.34088
NUDT16	-0.01441	0.401639	-0.09227	0.042158	0.012311	-0.32882	0.219024	0.421617
NUDT16L1	0.010917	0.19335	0.199546	0.262014	0.226893	0.103579	0.604969	0.0158
NUDT17	-0.36704	0.314677	NA	NA	NA	NA	0.285081	0.216293
NUDT18	0.094837	0.265953	0.370039	0.008723	-0.04066	-0.36728	0.039014	0.21784
NUDT19	NA	NA	-0.25884	-0.03979	0.173231	0.145318	NA	NA
NUDT2	0.03436	-0.05906	0.127449	-0.03713	-0.23212	-0.35748	-0.30872	-0.13091
NUDT21	-0.13512	-0.73707	-0.13087	0.083459	0.106491	0.137042	-0.2388	-0.17674
NUDT22	0.136389	0.379106	0.291548	0.004473	0.062757	-0.04343	-0.1497	-0.20352
NUDT3	-0.07667	0.225084	-0.03173	0.151008	-0.127	-0.32686	0.284627	0.068985
NUDT4	0.023997	-0.15307	-0.22079	-0.31812	0.128614	-0.34765	0.236809	0.032354
NUDT5	-0.11299	-0.2323	-0.12377	0.021679	-0.06794	-0.11351	0.141274	-0.02431
NUDT6	-0.02436	-0.18822	-0.22201	-0.20871	-0.4137	-0.37632	0.044947	-0.18373
NUDT7	NA	NA	0.041117	-0.17028	0.01094	-0.44265	NA	NA
NUDT8	-0.27316	0.42625	0.11729	0.146463	-0.1392	-0.37154	0.180711	0.395894
NUDT9	0.129532	-0.67045	-0.2889	-0.07238	-0.21868	-0.54087	-0.0708	-0.07663
NUDT9P1	NA	NA	-0.18248	0.244357	-0.02948	0.285926	0.106277	0.170701
NUF2	-0.30343	-0.12994	-0.29268	0.255762	0.056071	-0.07341	0.139367	0.078628
NUFIP1	0.536049	-0.12519	-0.16709	0.075925	0.128035	0.017655	-0.04182	-0.2252
NUFIP2	-0.0052	-0.0704	0.13951	-0.29123	0.021801	0.089675	-0.37161	-0.16177
NUMA1	0.201428	0.463331	0.354528	0.036362	0.438574	0.213403	-0.1884	-0.35743

NUMB	-0.30979	-0.4626	-0.40089	0.06825	-0.2874	-0.35447	-0.35957	0.081417
NUMBL	-0.12478	0.35428	-0.03647	-0.02941	-0.11963	-0.26662	-0.45333	-0.05923
NUP107	0.292286	-0.32087	-0.24446	-0.08082	0.23657	0.245426	-0.1835	-0.26894
NUP133	0.033687	-0.32092	-0.29389	0.190843	0.07996	-0.25021	0.491683	0.183823
NUP153	-0.04504	-0.66049	-0.24093	-0.08736	-0.05802	0.252788	0.387166	0.278809
NUP155	0.172259	0.333183	-0.03644	-0.0593	0.190785	0.19433	0.651973	0.287046
NUP160	0.130908	-0.68617	-0.39265	0.023481	0.001255	0.299203	0.637088	0.452931
NUP188	-0.05067	0.329972	0.194328	0.291892	0.456739	0.318291	0.408529	0.240726
NUP205	0.305698	0.06052	-0.27408	0.218303	-0.0068	0.248112	0.20744	-0.04728
NUP210	0.27038	0.532668	-0.22171	0.649126	0.351805	0.689919	0.078958	0.300139
NUP210L	-0.15103	0.580277	0.205062	-0.20046	-0.11391	0.238616	-0.01255	0.163501
NUP214	0.227275	0.222213	0.336142	0.00407	0.374442	0.290459	-0.08533	-0.0313
NUP35	-0.04008	-0.53802	-0.26888	-0.24334	-0.12735	0.027587	0.094963	-0.17896
NUP37	0.084594	-0.23717	-0.2853	-0.04974	0.118324	-0.19125	0.301308	0.277992
NUP43	0.480017	0.057118	-0.34205	0.218593	0.154848	0.257016	-0.36119	-0.13861
NUP50	-0.05443	0.296111	-0.50054	0.049789	-0.04907	-0.2189	-0.07913	0.180087
NUP54	-0.08145	-0.62859	-0.2336	-0.33145	-0.00883	0.039807	0.407443	0.102877
NUP62	0.026909	-0.45859	-0.38165	0.287729	0.170213	0.446132	-0.13584	-0.25071
NUP62CL	-0.08203	0.25716	-0.18171	-0.05303	0.16967	-0.52347	NA	NA
NUP85	-0.20052	-0.75368	0.384726	0.024148	0.014602	0.125731	0.60133	0.411428
NUP88	0.503359	-0.26932	-0.40647	0.153246	-0.38107	-0.09469	0.121007	0.062551
NUP93	0.290402	-0.00694	-0.13379	0.254577	0.263244	0.108676	0.708926	0.480864
NUP98	-0.05533	-0.39	-0.10709	0.117062	0.104432	0.315237	0.438134	0.309332
NUPL1	-0.20267	0.326326	-0.04288	-0.19664	0.090317	-0.25496	0.004798	0.11979
NUPL2	0.354087	-0.56892	-0.2976	-0.00868	-0.03943	-0.15255	0.378687	0.556355
NUPR1	0.028459	0.032767	0.12879	-0.09726	-0.0411	-0.29916	-0.40983	-0.49122
NUS1	NA	NA	-0.33253	0.139556	-0.03258	-0.05944	-0.42045	-0.21381
NUSAP1	-0.23652	0.250752	-0.32649	0.282145	-0.29335	0.01914	0.206619	0.066218
NUTF2	0.232337	0.140973	0.178453	0.267078	0.221111	-0.07614	-0.41979	-0.50595
NVL	0.233702	0.447304	-0.17986	-0.1199	0.030836	-0.34385	0.423411	-0.03878
NWD1	0.014043	0.122408	NA	NA	NA	NA	NA	NA
NXF1	-0.29802	-0.36844	0.125136	0.243624	0.129855	0.63281	0.401629	0.498188
NXF2	-0.21154	-0.15765	-0.11203	0.317681	-0.18934	-0.38548	-0.01685	0.412029
NXF3	-0.16524	-0.43226	0.088147	0.037709	-0.03392	0.182411	0.033199	0.263356
NXF5	0.397174	0.723026	-0.12417	0.134456	-0.17012	0.100981	-0.23849	-0.16948
NXN	-0.20143	-0.0765	0.157624	-0.05386	-0.04176	0.064914	-0.21256	-0.27228
NXNL1	NA	NA	0.190993	-0.04006	0.211801	0.050722	NA	NA
NXNL2	NA	NA	0.158207	0.031149	0.191303	-0.32728	NA	NA
NXPH1	-0.25665	-0.32058	-0.04808	0.014135	-0.07991	-0.2647	0.248252	0.238709
NXPH2	-0.33118	0.514244	-0.38924	0.294002	-0.04316	0.095473	0.337097	-0.16344
NXPH3	-0.12501	-0.11865	0.610296	-0.12674	0.206988	0.163804	0.031753	-0.40148
NXPH4	0.103359	0.324627	-0.02467	-0.00842	-0.21371	-0.17469	0.206436	-0.27928
NXT1	0.115317	0.282019	-0.28433	0.264338	0.221571	0.103736	0.3852	-0.01647
NXT2	0.27356	0.178374	-0.04615	-0.32973	0.025404	-0.33573	-0.31903	-0.17735
NYX	0.068102	0.144524	0.275462	0.085115	-0.04173	-0.11946	-0.25429	-0.21995
OAF	0.195965	-0.06452	0.341957	-0.18054	-0.02637	-0.25298	0.459539	0.400881
OAS1	0.006762	0.094572	0.123337	0.070122	0.078393	0.04465	0.182588	-0.01245
OAS2	-0.14148	0.168777	0.062788	-0.02478	-0.30944	0.49434	-0.12154	0.353676
OAS3	-0.32653	-0.27766	0.146906	-0.15696	0.079585	0.072538	0.036931	0.202991
OASL	-0.16815	0.486293	0.090921	-0.12929	-0.24677	0.201166	-0.023	0.107346
OAT	-0.09536	-0.50014	0.096792	-0.1887	-0.02466	-0.30116	0.263596	0.571733
OAZ1	0.152142	0.003848	-0.27765	0.237853	0.117851	-0.0266	0.198012	0.346569

OAZ2	-0.36593	-0.29121	0.145727	0.195915	0.000358	0.554597	NA	NA
OAZ3	-0.19426	0.149326	-0.11734	-0.01277	-0.40165	-0.45205	-0.59507	-0.58083
OBFC1	-0.25537	0.137858	0.038601	0.249647	0.015805	-0.05968	-0.07913	-0.04894
OBFC2A	-0.33264	-0.14226	0.259795	0.279768	0.021763	0.184729	-0.25094	0.501346
OBFC2B	-0.01286	-0.28094	-0.02195	0.125531	0.121291	-0.28617	0.343638	0.406631
OBP2A	-0.08851	0.553816	0.392446	-0.18902	0.117973	-0.08724	0.28167	-0.15255
OBP2B	-0.1912	0.007885	0.51381	-0.01217	0.262224	-0.07849	NA	NA
OBSCN	-0.55358	-0.12272	0.036357	-0.00986	-0.00127	0.44855	-0.19188	0.333625
OBSL1	NA	NA	0.196031	0.017996	-0.07611	0.350774	NA	NA
OC90	NA	NA	NA	NA	NA	NA	-0.11139	0.1207
OCA2	0.281278	0.1787	-0.06151	0.48736	0.089486	0.579929	-0.21972	0.055355
OCEL1	-0.46294	-0.05524	0.179911	0.032823	-0.06496	-0.1025	NA	NA
OCIAD1	-0.06707	0.380512	-0.04199	-0.04203	-0.17779	-0.23751	-0.43703	-0.41307
OCIAD2	0.014276	0.076954	0.013231	-0.28019	-0.18406	-0.289	0.163386	0.104073
OCLM	-0.00888	0.007219	0.200678	-0.08708	0.224722	0.106719	0.356977	0.422544
OCLN	-0.26636	0.139072	-0.21473	-0.34993	-0.08539	-0.46853	0.332721	0.154856
OCM	0.20179	0.779198	NA	NA	NA	NA	0.569102	0.333148
OCRL	0.529219	-0.32425	-0.27067	-0.36835	0.163293	-0.08309	0.068138	0.09379
ODAM	0.119615	-0.3462	-0.08865	-0.06679	-0.26365	-0.09603	NA	NA
ODC1	-0.06956	-0.20137	-0.27504	0.24529	-0.02372	0.138893	0.234564	0.214026
ODF1	-0.06508	-0.10959	0.149342	-0.41022	-0.06759	0.306642	0.165989	0.090925
ODF2	0.315057	0.146394	-0.13616	0.365749	0.241572	-0.06211	0.51428	0.37794
ODF2L	-0.00979	-0.39894	-0.08493	-0.01254	-0.22765	0.030785	-0.05966	0.362877
ODF3	0.124694	0.737546	-0.08949	0.004917	0.040047	0.07002	NA	NA
ODF3L1	-0.05147	-0.6955	-0.04135	-0.25832	-0.09464	0.156069	0.342454	0.118172
ODF3L2	NA	NA	-0.18114	-0.30365	0.052132	-0.09276	NA	NA
ODF4	0.2048	0.552599	0.105594	-0.12584	-0.17889	0.033794	-0.30125	-0.29482
ODZ1	0.413115	0.67716	-0.21093	-0.16943	0.01387	-0.02754	0.108138	0.319398
ODZ2	NA	NA	0.356567	-0.10755	-0.03443	0.170037	0.446419	0.23163
ODZ3	NA	NA	0.15416	-0.1239	-0.33643	0.127455	-0.20454	-0.24608
ODZ4	NA	NA	0.194988	0.54069	-0.13586	0.456403	NA	NA
OFCC1	0.091736	0.661213	0.28216	-0.01642	1.85E-05	-0.46555	0.09787	0.271641
OFD1	0.384066	-0.27931	0.063364	0.272571	0.310913	0.339346	-0.0334	0.06393
OGDH	-0.02818	0.255769	0.327711	-0.03024	0.343622	0.063646	0.025227	0.544071
OGDHL	0.268641	-0.29934	0.017216	-0.01394	-0.03568	0.080645	-0.24008	0.056302
OGFOD1	0.026863	0.10788	0.015212	-0.20737	-0.08723	-0.04434	0.294757	0.161863
OGFOD2	-0.23024	-0.22662	0.173758	0.150794	0.176598	-0.12007	0.202449	0.024815
OGFR	0.349442	0.351522	0.186166	0.045174	0.068879	0.286917	0.396331	0.438349
OGFRL1	-0.10352	0.19512	-0.14555	0.193759	-0.18488	-0.05473	-0.4547	-0.35227
OGG1	0.253907	0.089064	0.165868	0.515841	0.394202	-0.20362	0.362227	0.22091
OGN	0.240693	-0.47887	0.193304	-0.04822	-0.31631	-0.01193	-0.30817	-0.39139
OGT	0.260574	-0.47342	0.101901	0.14855	0.131935	0.398526	0.139212	0.377414
OIP5	-0.02504	0.068241	-0.3107	0.237345	-0.11841	-0.34754	0.250257	0.036406
OIT3	0.040289	0.571297	-0.33752	0.223434	-0.0142	-0.1608	-0.29067	-0.37289
OLA1	NA	NA	0.128002	0.150035	0.23458	-0.2577	NA	NA
OLAH	-0.14907	0.599781	-0.12003	-0.11162	-0.12604	0.013324	NA	NA
OLFM1	0.061965	-0.18096	0.001519	0.31433	-0.0256	-0.18061	-0.46439	-0.58523
OLFM2	NA	NA	0.066371	-0.06791	-0.0291	-0.18294	-0.4641	-0.42895
OLFM3	0.047612	0.096544	-0.01027	-0.10608	-0.12753	-0.18497	-0.18649	-0.22448
OLFM4	0.078155	-0.107	-0.01809	0.255456	0.024792	0.004431	0.11643	0.083377
OLFML1	-0.06468	-0.04056	0.247939	0.083742	-0.19416	0.363161	-0.38794	-0.17682
OLFML2A	-0.2254	-0.17296	0.001066	-0.20391	-0.04643	0.335584	-0.12361	-0.24496

OLFML2B	-0.12154	-0.11088	0.167017	0.128136	-0.13636	0.065553	-0.16769	-0.0737
OLFML3	0.092034	-0.08748	0.293767	-0.00575	-0.16891	0.0988	-0.16686	-0.30805
OLIG1	-0.06415	0.331	0.092015	-0.09106	0.014368	-0.37681	NA	NA
OLIG2	-0.55526	0.223807	0.109192	-0.09511	-0.16291	0.311425	-0.19187	-0.03411
OLIG3	0.188395	0.241774	-0.0916	-0.23303	0.305323	-0.02642	0.343793	0.113734
OLR1	-0.42353	-0.10693	-0.00853	-0.0047	-0.11383	0.154641	-0.15397	-0.0062
OMA1	0.043185	-0.54874	0.350434	-0.06849	0.189091	0.168848	-0.56996	-0.46054
OMD	0.260827	-0.18986	0.363731	-0.17385	-0.12723	-0.09849	0.082918	-0.16205
OMG	-0.5025	-0.38234	-0.30668	0.158959	-0.21193	0.172637	-0.53018	-0.35079
OMP	-0.25191	-0.23644	-0.13643	-0.30261	0.153231	-0.20627	-0.19657	0.108116
ONECUT1	0.105378	0.309702	-0.28472	-0.20659	0.015433	0.10896	0.23771	-0.05808
ONECUT2	-0.00341	0.320044	0.146432	-0.50054	0.165247	-0.16899	-0.2387	0.044124
ONECUT3	NA	NA	NA	NA	NA	NA	0.160286	0.407096
OOEP	NA	NA	-0.22185	-0.0203	-0.2391	-0.15801	NA	NA
OPA1	0.213236	-0.45366	-0.35588	-0.28572	-0.07588	0.026326	0.171091	0.072044
OPA3	0.061014	-0.09029	-0.02243	-0.05319	-0.1686	0.09598	-0.40542	-0.41323
OPALIN	NA	NA	0.163953	-0.30336	0.112469	-0.11456	NA	NA
OPCML	0.366571	0.336286	0.021373	-0.30448	0.153191	-0.13495	-0.01456	-0.37179
OPHN1	0.188367	0.35652	0.038493	-0.11066	0.119624	-0.24168	0.317096	0.145908
OPLAH	-0.07121	0.412317	0.241948	-0.08416	0.04146	0.090851	-0.05453	-0.16542
OPN1LW	0.071404	0.458759	-0.1847	-0.09058	-0.11382	-0.16959	0.264081	0.075937
OPN1MW	NA	NA	-0.03505	-0.17036	-0.01548	-0.10831	-0.06328	0.122095
OPN1SW	-0.07757	-0.19598	0.126863	0.041217	-0.04841	0.166751	0.113	-0.07767
OPN3	-0.06414	-0.20218	-0.42434	-0.18532	-0.38773	-0.41569	0.49643	0.073618
OPN4	-0.06904	0.158891	0.191281	-0.1101	0.121405	-0.13479	0.014379	-0.08403
OPN5	0.016667	0.615791	NA	NA	NA	NA	0.130185	-0.07581
OPRD1	0.015542	-0.27761	-0.19598	-0.45177	0.092302	0.054454	0.171224	-0.14722
OPRK1	0.297649	0.04823	0.144297	-0.31262	0.08659	-0.26467	0.164296	0.135472
OPRL1	-0.2474	-0.07667	0.155217	0.481562	0.152447	0.075198	-0.28092	-0.40328
OPRM1	0.068356	0.42558	-0.09147	0.105721	-0.04201	-0.09333	0.009679	-0.47134
OPTC	0.366957	0.67867	-0.07926	-0.14441	0.169634	0.271065	-0.03682	-0.13081
OPTN	-0.14372	-0.26828	0.208699	-0.33727	-0.05821	-0.16281	-0.27463	0.110173
OR10A2	0.114526	0.387064	0.270695	0.137766	-0.03821	-0.22115	0.186652	-0.43109
OR10A3	0.094029	0.450647	NA	NA	NA	NA	-0.24418	-0.03326
OR10A4	0.335439	0.720176	0.06942	-0.03502	0.159506	-0.34599	-0.07591	0.090691
OR10A5	0.248098	0.421192	-0.25035	-0.02684	0.271884	-0.07299	0.466349	0.537866
OR10A6	0.433776	0.29243	NA	NA	NA	NA	0.132559	0.022901
OR10A7	0.271031	0.510884	NA	NA	NA	NA	-0.4607	-0.33344
OR10AD1	0.036781	0.34784	NA	NA	NA	NA	-0.17175	-0.06512
OR10AG1	-0.264	0.096242	NA	NA	NA	NA	0.094885	-0.25295
OR10C1	0.341903	0.040387	-0.10708	-0.21985	0.159916	-0.32014	-0.2837	-0.27142
OR10G2	0.016077	0.102989	NA	NA	NA	NA	-0.0704	0.156228
OR10G3	-0.1418	0.281596	NA	NA	NA	NA	-0.00656	-0.03594
OR10G4	0.236191	0.276277	NA	NA	NA	NA	-0.2506	-0.01931
OR10G7	0.225384	0.696229	NA	NA	NA	NA	0.389787	0.469461
OR10G8	0.330977	0.291942	-0.01177	-0.38187	-0.30044	-0.0547	-0.10516	-0.01244
OR10G9	0.052756	0.507805	NA	NA	NA	NA	0.224441	0.039101
OR10H1	0.223888	0.537328	-0.2617	-0.22341	0.104004	-0.04611	0.517842	0.395139
OR10H2	0.040758	0.584796	0.060436	-0.14525	-0.25339	-0.00116	0.361607	0.347353
OR10H3	0.023439	0.374297	0.033438	-0.07015	0.160027	-0.08635	0.274179	0.172664
OR10H4	0.067955	0.566457	0.355121	-0.03379	-0.00545	-0.30782	0.093161	-0.00614
OR10H5	0.323857	0.447207	NA	NA	NA	NA	-0.03938	-0.06723

OR10J1	0.173567	0.2103	-0.04638	-0.16084	-0.25552	0.3374	0.481026	0.543325
OR10J3	0.357342	0.73476	0.289224	-0.03268	0.146431	0.111814	0.174422	-0.09409
OR10J5	-0.00943	0.411684	-0.00652	-0.26446	-0.08069	-0.40176	0.05312	0.488293
OR10K1	0.431169	-0.1901	NA	NA	NA	NA	0.48871	-0.02456
OR10K2	0.189912	0.021245	NA	NA	NA	NA	-0.16168	-0.24083
OR10P1	-0.04035	0.259698	0.289031	-0.32789	-0.14305	-0.03256	0.093619	0.041722
OR10Q1	0.150253	0.290365	NA	NA	NA	NA	0.259476	0.417514
OR10R2	-0.19045	0.618242	0.033739	0.35852	0.473421	-0.21952	-0.11279	-0.04413
OR10S1	-0.09494	0.425117	NA	NA	NA	NA	0.433138	0.379459
OR10T2	0.053721	-0.11984	0.15021	0.178066	-0.19157	-0.0857	0.000515	-0.32988
OR10V1	0.223728	0.045034	NA	NA	NA	NA	-0.31369	-0.286
OR10W1	0.17993	0.34792	NA	NA	NA	NA	0.511927	0.43209
OR10X1	-0.01784	0.030992	NA	NA	NA	NA	-0.4605	-0.34255
OR10Z1	0.172594	0.126233	NA	NA	NA	NA	0.070931	0.127009
OR11A1	0.473848	0.186592	0.087993	-0.2385	-0.20425	-0.15741	0.046378	-0.20321
OR11G2	0.1366	-0.36831	0.030828	-0.27078	-0.11328	0.27799	-0.28027	-0.29946
OR11H1	-0.25707	0.541546	NA	NA	NA	NA	-0.24467	-0.48745
OR11H12	NA	NA	0.091164	0.02261	0.030888	-0.19423	-0.39294	-0.06703
OR11H4	-0.0756	0.006027	NA	NA	NA	NA	-0.16511	-0.19838
OR11H6	-0.09184	0.707335	NA	NA	NA	NA	0.400184	0.040912
OR11L1	0.263323	0.579027	NA	NA	NA	NA	-0.50572	-0.63875
OR12D2	0.192333	0.662291	0.09674	0.099381	-0.17821	-0.38043	0.265758	0.139098
OR12D3	0.388386	0.71585	0.098804	-0.35764	0.076592	0.123346	-0.2806	-0.38492
OR13A1	-0.26899	-0.25611	NA	NA	NA	NA	0.41812	0.060848
OR13C2	0.332922	0.312599	NA	NA	NA	NA	0.088089	0.40068
OR13C3	0.263073	0.651657	NA	NA	NA	NA	-0.17613	-0.16375
OR13C4	0.081448	0.028431	-0.15352	0.234327	0.078341	0.129005	0.25	0.199708
OR13C5	0.061401	0.199304	NA	NA	NA	NA	-0.08881	0.144921
OR13C8	-0.01361	-0.15836	NA	NA	NA	NA	0.135969	-0.11851
OR13C9	-0.08883	0.088728	NA	NA	NA	NA	0.441488	0.061448
OR13D1	-0.12324	-0.04279	0.023083	0.245668	0.180289	0.199705	0.291544	0.631391
OR13F1	0.212313	0.134917	NA	NA	NA	NA	-0.22555	-0.21547
OR13G1	0.313924	0.450596	NA	NA	NA	NA	0.595673	0.38766
OR13H1	0.131294	0.473922	NA	NA	NA	NA	-0.16377	0.102492
OR13J1	0.352266	-0.32199	NA	NA	NA	NA	0.267815	-0.09669
OR1A1	-0.51931	0.303283	0.089635	-0.05074	0.220149	0.016573	-0.20869	-0.07238
OR1A2	0.248174	0.372407	0.121395	0.04656	0.039795	-0.26153	-0.28429	-0.34401
OR1B1	0.227253	0.25349	NA	NA	NA	NA	0.32505	0.352178
OR1C1	-0.21366	0.520877	-0.05029	-0.01973	-0.06508	-0.01625	0.081476	-0.381
OR1D2	-0.37878	-0.08807	0.042773	-0.20841	-0.38409	0.036538	-0.12896	0.155249
OR1D4	NA	NA	0.005888	0.110435	0.077539	-0.21048	-0.21729	-0.19722
OR1E1	0.394594	0.753874	0.234364	0.241767	0.320215	0.256384	-0.10342	-0.13133
OR1E2	0.07228	0.184322	NA	NA	NA	NA	-0.04918	0.159709
OR1F1	-0.07344	0.490524	0.081835	-0.30165	0.384894	0.108805	0.068349	-0.29855
OR1G1	0.387897	0.395324	-0.1485	-0.31461	0.41846	0.055047	0.156998	-0.38727
OR1I1	-0.02164	0.14024	NA	NA	NA	NA	-0.19795	-0.21114
OR1J1	-0.04047	0.213677	NA	NA	NA	NA	-0.65493	-0.57514
OR1J2	0.02603	0.301347	NA	NA	NA	NA	0.350943	0.344676
OR1J4	-0.33152	-0.31709	NA	NA	NA	NA	0.424527	0.317826
OR1K1	0.002413	-0.0361	NA	NA	NA	NA	-0.14801	-0.21424
OR1L1	0.290868	0.432298	NA	NA	NA	NA	-0.24417	-0.06669
OR1L3	0.0489	0.622849	0.187204	0.213774	-0.18431	-0.16681	-0.03433	0.133439

OR1L4	0.210701	0.527837	0.006934	-0.03472	0.018778	0.240818	0.421772	0.075443
OR1L6	0.100763	0.325639	NA	NA	NA	NA	-0.1945	-0.17879
OR1L8	0.094617	0.619446	NA	NA	NA	NA	-0.14536	-0.24195
OR1M1	0.119587	0.272545	NA	NA	NA	NA	-0.49704	-0.31379
OR1N1	-0.07388	0.191102	NA	NA	NA	NA	0.127799	-0.02669
OR1N2	0.128427	0.486319	-0.048	0.003855	-0.19925	-0.2663	-0.30895	-0.05639
OR1Q1	0.096979	-0.26678	-0.16127	-0.29162	-0.04199	0.173956	-0.02754	-0.07715
OR1S1	0.201715	0.740354	NA	NA	NA	NA	-0.28806	-0.10954
OR1S2	-0.07303	0.286156	0.030734	-0.02503	-0.05723	0.014048	0.223979	0.229018
OR2A12	0.16251	0.440296	NA	NA	NA	NA	0.079174	-0.22572
OR2A14	0.172652	0.638314	-0.07954	0.097702	0.280166	0.235639	-0.43737	-0.64244
OR2A2	0.285048	0.672277	NA	NA	NA	NA	0.214427	0.284731
OR2A20P	NA	NA	-0.38341	-0.11505	0.225464	0.197376	0.171357	0.127953
OR2A25	-0.01976	-0.11897	0.226068	-0.27959	0.137825	-0.21215	0.323839	0.348392
OR2A5	0.112003	0.239179	NA	NA	NA	NA	-0.58775	-0.71803
OR2A7	NA	NA	-0.34722	-0.03956	0.091245	0.107148	0.316764	0.257379
OR2A9P	NA	NA	-0.35779	-0.17965	-0.2254	0.015674	0.44756	0.451293
OR2AE1	0.076607	0.689563	NA	NA	NA	NA	-0.00534	-0.06158
OR2AG1	0.254801	-0.01366	NA	NA	NA	NA	0.493044	0.158151
OR2AG2	-0.23652	0.601664	NA	NA	NA	NA	-0.19754	0.075099
OR2AK2	0.073209	0.463431	NA	NA	NA	NA	-0.54899	-0.53118
OR2AT4	-0.07014	0.481744	NA	NA	NA	NA	-0.35048	0.264369
OR2B11	0.255906	0.556503	NA	NA	NA	NA	0.129341	0.217171
OR2B2	0.161998	0.458688	0.197956	-0.17554	0.206907	0.008465	-0.1999	-0.40662
OR2B3	0.171716	0.702805	NA	NA	NA	NA	-0.44129	-0.38923
OR2B6	-0.06193	0.065112	-0.09869	-0.18483	-0.32002	0.215041	0.334615	-0.08328
OR2C1	0.1124	0.617816	-0.01719	-0.14315	-0.08146	-0.08805	0.194823	-0.36788
OR2C3	0.207679	0.627951	-0.11685	-0.10998	0.173662	-0.10551	0.104928	0.301667
OR2D2	0.294756	0.041127	NA	NA	NA	NA	0.513435	0.48133
OR2D3	-0.07731	-0.07516	NA	NA	NA	NA	-0.41066	-0.44343
OR2F1	0.257836	0.784331	-0.23386	-0.07172	-0.12874	-0.09202	0.097744	-0.20477
OR2F2	-0.07638	0.194343	NA	NA	NA	NA	-0.18191	-0.17823
OR2G2	-0.23688	-0.10857	NA	NA	NA	NA	-0.31835	-0.34534
OR2G3	0.249649	0.165074	NA	NA	NA	NA	0.586517	0.518507
OR2G6	0.056001	0.54675	NA	NA	NA	NA	0.464048	0.034009
OR2H1	0.213774	0.562966	0.108889	0.005218	-0.09458	-0.03422	0.320143	0.058845
OR2H2	-0.04427	0.20606	-0.11707	-0.36661	0.060844	-0.0218	-0.5085	-0.27009
OR2J2	-0.03466	0.507265	0.254192	-0.0766	0.333703	-0.05468	0.133978	0.066851
OR2J3	0.560199	0.442043	NA	NA	NA	NA	0.519019	0.10449
OR2K2	-0.12837	0.21579	NA	NA	NA	NA	-0.0547	-0.13362
OR2L13	-0.24145	0.355181	-0.03869	-0.21016	0.059762	0.01854	0.197962	0.123454
OR2L2	0.148954	0.34525	NA	NA	NA	NA	0.49379	0.123803
OR2L3	0.182479	0.602009	NA	NA	NA	NA	0.249408	-0.11047
OR2M2	0.291498	0.679144	0.26639	0.018022	-0.1051	0.37506	0.009773	0.06601
OR2M3	-0.06734	0.520438	NA	NA	NA	NA	0.195917	0.147998
OR2M4	0.049219	0.025485	NA	NA	NA	NA	0.051715	0.184221
OR2M5	0.165508	0.536358	NA	NA	NA	NA	-0.31606	-0.21585
OR2M7	0.212089	0.697906	NA	NA	NA	NA	0.283739	0.144215
OR2S2	0.185262	0.525003	-0.162	0.005547	-0.17571	0.317438	-0.1467	0.000354
OR2T1	0.287653	0.248392	NA	NA	NA	NA	-0.1154	-0.18443
OR2T10	0.251142	0.330529	NA	NA	NA	NA	-0.12679	0.249913
OR2T11	-0.16934	0.289295	NA	NA	NA	NA	-0.0686	-0.26255

OR2T12	-0.16292	0.636637	NA	NA	NA	NA	-0.25365	-0.17796
OR2T2	-0.55852	0.133048	NA	NA	NA	NA	NA	NA
OR2T27	0.051308	-0.07166	NA	NA	NA	NA	0.149978	0.306811
OR2T3	-0.08956	0.434982	NA	NA	NA	NA	-0.17259	0.20891
OR2T33	-0.21388	0.476459	NA	NA	NA	NA	0.479193	0.222592
OR2T34	-0.381	0.688967	NA	NA	NA	NA	NA	NA
OR2T35	0.203665	0.430976	NA	NA	NA	NA	-0.34022	-0.50459
OR2T4	0.414257	0.523883	NA	NA	NA	NA	-0.49997	-0.27714
OR2T5	NA	NA	-0.26289	-0.03066	0.26933	0.060394	-0.18102	-0.15846
OR2T6	-0.26053	0.51133	NA	NA	NA	NA	0.065081	-0.08003
OR2T8	-0.04364	0.507212	-0.07206	-0.34258	-0.14203	-0.31361	-0.30282	-0.35319
OR2V2	0.129306	0.684697	-0.11165	0.222144	-0.13032	-0.22689	-0.28646	-0.28216
OR2W1	-0.05675	0.11597	0.006963	-0.31573	0.098771	-0.22753	0.389855	0.359784
OR2W3	0.027774	0.410686	-0.31416	0.058293	0.086244	0.011583	-0.20024	-0.10491
OR2W5	0.413604	0.247556	NA	NA	NA	NA	0.363257	0.404783
OR2Y1	0.319508	0.062929	NA	NA	NA	NA	-0.404	-0.31799
OR2Z1	-0.31241	0.221028	NA	NA	NA	NA	0.473875	0.304517
OR3A1	0.160338	0.191885	-0.16955	0.254874	-0.03462	-0.20494	-0.53336	-0.15856
OR3A2	0.088737	0.589534	-0.08613	0.058584	0.087074	-0.27495	0.084234	-0.38829
OR3A3	0.063445	0.427049	0.048911	-0.22737	-0.07163	-0.32661	0.100947	0.328981
OR4A15	NA	NA	0.072159	-0.41277	0.321912	-0.04192	-0.61402	-0.26542
OR4A16	0.009479	0.537618	NA	NA	NA	NA	-0.03427	0.100962
OR4A47	0.233852	-0.07901	NA	NA	NA	NA	-0.1342	-0.4418
OR4A5	0.050559	0.312835	NA	NA	NA	NA	0.306397	0.437775
OR4B1	0.213417	0.741226	NA	NA	NA	NA	0.572691	0.29832
OR4C11	0.149768	0.36295	NA	NA	NA	NA	0.324928	-0.0337
OR4C12	-0.03582	0.152034	NA	NA	NA	NA	-0.03762	0.271957
OR4C13	-0.41169	0.565429	NA	NA	NA	NA	-0.6607	-0.21539
OR4C15	0.191351	0.308276	0.008762	-0.00285	0.369218	-0.01702	0.514909	0.494212
OR4C16	0.302829	0.488095	NA	NA	NA	NA	-0.44474	-0.4792
OR4C3	0.121544	0.159531	0.208293	0.169908	-0.03402	0.507956	0.220957	-0.0512
OR4C45	-0.29232	0.343275	NA	NA	NA	NA	0.442301	0.598486
OR4C46	0.503119	0.4541	0.241955	-0.11004	0.191189	-0.48081	-0.07111	0.176378
OR4C6	0.169757	0.773208	NA	NA	NA	NA	0.021528	-0.46666
OR4D1	0.304038	0.009311	NA	NA	NA	NA	-0.2398	-0.41235
OR4D10	0.0928	0.655344	NA	NA	NA	NA	-0.14862	-0.14213
OR4D11	0.326333	0.511494	NA	NA	NA	NA	-0.24858	-0.04002
OR4D2	-0.11527	0.416292	0.072543	-0.20203	-0.05834	-0.0216	-0.26159	-0.48875
OR4D5	0.166169	0.668464	0.168344	0.13558	0.126332	0.086008	-0.04601	-0.07146
OR4D6	0.288062	0.145291	NA	NA	NA	NA	0.061083	-0.177
OR4D9	-0.15203	0.566859	NA	NA	NA	NA	0.118723	0.394276
OR4E2	-0.03181	0.335308	NA	NA	NA	NA	-0.04162	-0.01292
OR4F15	0.192949	0.118192	NA	NA	NA	NA	-0.33132	-0.03461
OR4F21	0.163025	0.732693	NA	NA	NA	NA	-0.44164	0.02654
OR4F4	0.195435	0.423652	0.188204	-0.0537	-0.03541	0.025255	-0.18607	-0.11632
OR4F5	0.087288	0.603846	NA	NA	NA	NA	0.184644	0.167009
OR4F6	0.110012	0.162217	NA	NA	NA	NA	0.055785	0.277493
OR4K1	-0.16698	-0.09903	0.020827	0.098284	0.136492	0.001162	-0.35914	-0.39146
OR4K13	-0.01542	0.347846	NA	NA	NA	NA	-0.2203	-0.13382
OR4K14	-0.28114	-0.2397	NA	NA	NA	NA	0.315043	-0.12464
OR4K15	0.38217	0.707224	NA	NA	NA	NA	-0.16822	0.088239
OR4K17	0.211469	0.605086	0.181396	0.116545	-0.28502	-0.133	0.105298	-0.01118

OR4K2	0.135336	0.481771	NA	NA	NA	NA	0.155017	0.054012
OR4K5	NA	NA	NA	NA	NA	NA	0.294572	0.394946
OR4L1	0.031295	0.538963	NA	NA	NA	NA	-0.28641	-0.11083
OR4M1	0.009753	0.221326	NA	NA	NA	NA	0.461256	0.306016
OR4M2	0.201996	0.491874	NA	NA	NA	NA	-0.2287	0.293139
OR4N2	-0.00386	-0.19967	NA	NA	NA	NA	-0.16939	-0.31571
OR4N4	0.123293	0.379485	0.051355	-0.35185	-0.13469	0.279029	0.223333	0.187308
OR4N5	-0.10994	-0.06731	NA	NA	NA	NA	0.189617	0.362259
OR4P4	-0.1195	0.45729	NA	NA	NA	NA	-0.01517	0.078512
OR4Q3	0.082601	0.318499	NA	NA	NA	NA	-0.06825	-0.14726
OR4S1	0.229634	0.877397	-0.05964	0.062024	0.144807	-0.02999	-0.50315	-0.36101
OR4S2	-0.00552	0.413878	NA	NA	NA	NA	-0.31814	-0.00963
OR4X1	-0.05793	0.365693	NA	NA	NA	NA	0.093121	-0.03413
OR4X2	-0.25021	0.518375	0.127718	-0.0755	0.055984	-0.44269	0.260183	-0.11976
OR51A2	0.332579	0.00682	NA	NA	NA	NA	-0.23326	-0.17285
OR51A4	0.219803	-0.02964	NA	NA	NA	NA	0.066441	-0.00197
OR51A7	-0.09397	0.171529	0.316418	0.1743	0.088473	0.036662	-0.12567	0.249212
OR51B2	0.353489	0.527256	-0.09409	0.044694	-0.31714	0.073938	-0.43327	-0.30974
OR51B4	-0.08687	-0.09975	0.20177	0.086495	-0.14503	0.189048	0.063874	-0.1327
OR51B5	0.185949	-0.38799	0.056597	-0.00637	0.012163	-0.08045	-0.15221	-0.36421
OR51B6	0.087079	0.648237	NA	NA	NA	NA	-0.13456	-0.12825
OR51D1	0.050564	-0.28709	-0.19814	-0.33363	0.203883	-0.03375	0.441532	0.25472
OR51E1	0.01109	-0.03014	0.169553	-0.13868	0.168274	-0.20516	-0.16399	-0.14031
OR51E2	-0.19782	0.181349	-0.1004	-0.07078	-0.16413	-0.14554	0.1861	-0.11117
OR51F1	0.051183	0.342587	NA	NA	NA	NA	-0.03173	0.032414
OR51F2	-0.17905	-0.06782	NA	NA	NA	NA	0.198976	0.205257
OR51G1	0.435008	0.618444	0.340781	0.118821	-0.08394	0.01702	-0.07371	-0.3845
OR51G2	0.157886	0.638282	NA	NA	NA	NA	-0.21974	-0.33114
OR51I1	0.050318	-0.14565	NA	NA	NA	NA	0.13236	0.12579
OR51I2	-0.27149	0.170589	NA	NA	NA	NA	-0.08396	0.23675
OR51L1	0.359483	0.194767	NA	NA	NA	NA	-0.06405	0.137114
OR51M1	0.16978	0.621513	NA	NA	NA	NA	-0.32887	0.401361
OR51Q1	-0.22256	0.065767	0.0267	-0.06418	-0.25747	0.153468	-0.26037	-0.29842
OR51S1	-0.48048	0.0962	NA	NA	NA	NA	-0.20093	-0.18138
OR51T1	-0.69874	0.118868	NA	NA	NA	NA	-0.57591	-0.58199
OR51V1	-0.13902	0.266873	NA	NA	NA	NA	0.310019	0.424091
OR52A1	0.066388	-0.12319	-0.23355	-0.00188	-0.09782	-0.5165	-0.37821	0.158962
OR52A4	0.260372	0.599269	NA	NA	NA	NA	-0.05465	0.321212
OR52A5	-0.31244	0.008691	NA	NA	NA	NA	-0.38557	-0.40882
OR52B2	0.213457	0.632226	0.06501	0.220441	-0.09929	-0.1733	0.155527	0.218754
OR52B4	0.297211	0.491738	NA	NA	NA	NA	-0.19575	-0.00975
OR52B6	0.41684	0.109276	NA	NA	NA	NA	-0.27113	-0.37941
OR52D1	0.050522	0.162821	NA	NA	NA	NA	-0.5837	-0.52196
OR52E2	0.317152	0.36898	NA	NA	NA	NA	-0.00728	0.026271
OR52E4	0.348782	0.595488	NA	NA	NA	NA	-0.1893	0.285013
OR52E6	-0.27928	0.715145	NA	NA	NA	NA	0.42246	0.091441
OR52E8	-0.00716	0.057754	NA	NA	NA	NA	0.215962	0.243772
OR52H1	-0.41185	-0.11967	NA	NA	NA	NA	0.429926	0.152022
OR52I1	0.087232	0.662498	NA	NA	NA	NA	0.273584	0.293158
OR52I2	-0.04249	-0.11729	NA	NA	NA	NA	-0.40032	0.091204
OR52J3	-0.02311	0.19152	NA	NA	NA	NA	0.233424	0.307328
OR52K1	0.157768	0.288186	NA	NA	NA	NA	0.349855	0.269473

OR52K2	0.404114	0.602374	0.362619	-0.09416	0.002327	0.189467	-0.1165	-0.06162
OR52L1	0.144226	0.191199	NA	NA	NA	NA	0.453066	0.289647
OR52M1	0.304035	0.691428	NA	NA	NA	NA	-0.02094	0.196452
OR52N1	0.198775	0.171608	NA	NA	NA	NA	-0.20941	0.349062
OR52N2	0.273684	0.771954	NA	NA	NA	NA	0.216831	0.392359
OR52N4	-0.01952	0.626267	NA	NA	NA	NA	0.070209	0.068113
OR52N5	-0.04831	0.35564	NA	NA	NA	NA	-0.06092	-0.10001
OR52R1	0.198051	0.447458	NA	NA	NA	NA	-0.33094	-0.29072
OR52W1	0.067681	0.459724	NA	NA	NA	NA	-0.42035	-0.24503
OR56A1	-0.0907	0.738671	NA	NA	NA	NA	0.471909	-0.15882
OR56A3	0.017571	0.080622	NA	NA	NA	NA	0.210757	-0.00051
OR56A4	0.139388	0.262309	NA	NA	NA	NA	0.545028	0.252482
OR56B1	-0.06585	-0.2052	-0.11474	0.346483	0.079724	-0.18317	-0.047	-0.24843
OR56B4	0.255499	-0.1767	NA	NA	NA	NA	-0.31232	-0.36482
OR5A1	0.071569	0.662113	0.30076	-0.19636	0.207063	0.316187	-0.01787	0.405325
OR5A2	0.305257	-0.0711	NA	NA	NA	NA	-0.21522	-0.12066
OR5AC2	0.009309	0.101662	NA	NA	NA	NA	-0.03111	-0.27747
OR5AK2	0.272928	0.032449	-0.22489	-0.19987	0.047337	-0.19189	0.277203	0.194482
OR5AN1	-0.16578	-0.13583	NA	NA	NA	NA	0.50741	-0.33935
OR5AP2	-0.0707	0.673904	0.032998	0.086786	-0.11294	0.263908	-0.23266	-0.07491
OR5AR1	-0.39255	0.563975	NA	NA	NA	NA	-0.44065	-0.32511
OR5AS1	0.336912	0.658096	NA	NA	NA	NA	0.173521	-0.24364
OR5AU1	0.200179	0.145802	NA	NA	NA	NA	0.277247	0.270968
OR5B12	-0.19194	0.147683	NA	NA	NA	NA	0.155316	-0.23097
OR5B17	0.006953	0.59384	NA	NA	NA	NA	0.226442	0.398357
OR5B2	-0.01017	0.153677	NA	NA	NA	NA	-0.22635	0.045871
OR5B21	0.030279	0.446346	NA	NA	NA	NA	0.340703	0.307291
OR5B3	0.049942	0.697793	NA	NA	NA	NA	-0.28378	-0.13122
OR5C1	0.055711	0.250279	NA	NA	NA	NA	0.570266	0.258439
OR5D13	0.141532	0.279695	NA	NA	NA	NA	-0.07476	0.029744
OR5D14	-0.35263	0.566346	NA	NA	NA	NA	-0.26542	-0.06938
OR5D16	0.137998	0.256014	NA	NA	NA	NA	0.109403	0.234574
OR5D18	-0.03789	0.765397	NA	NA	NA	NA	-0.21471	0.244402
OR5F1	-0.0112	0.620725	0.015106	-0.16114	-0.02119	-0.28288	-0.1614	-0.16456
OR5H1	0.45754	0.242848	0.199745	0.005262	0.231164	0.029706	0.052711	-0.29331
OR5H14	0.232891	0.161716	NA	NA	NA	NA	0.228234	-0.0561
OR5H15	0.20913	0.555409	NA	NA	NA	NA	0.060608	0.063025
OR5H2	-0.0491	0.400448	NA	NA	NA	NA	-0.07753	-0.04699
OR5H6	-0.37064	0.472725	NA	NA	NA	NA	0.418579	-0.29472
OR5I1	0.044764	0.192769	0.135225	0.267165	0.180702	0.034469	0.171276	0.293762
OR5J2	-0.05044	0.1062	NA	NA	NA	NA	-0.5191	-0.12427
OR5K1	0.123351	0.495295	NA	NA	NA	NA	0.291189	0.394681
OR5K2	0.364517	0.054782	NA	NA	NA	NA	0.504379	0.454304
OR5K3	-0.11228	0.365444	NA	NA	NA	NA	-0.05652	0.17879
OR5K4	-0.42312	0.300953	NA	NA	NA	NA	-0.25801	-0.07682
OR5L1	0.175976	0.130991	NA	NA	NA	NA	-0.14114	-0.28124
OR5L2	0.243986	0.136348	0.135304	-0.20345	-0.15273	-0.28049	0.152002	0.041512
OR5M1	-0.24054	-0.04364	NA	NA	NA	NA	NA	NA
OR5M10	0.040168	0.405628	NA	NA	NA	NA	-0.4567	-0.09729
OR5M11	0.138357	0.793494	NA	NA	NA	NA	0.334623	0.043249
OR5M3	-0.00519	0.062406	NA	NA	NA	NA	-0.30734	-0.58725
OR5M8	0.489543	0.652037	NA	NA	NA	NA	-0.15585	-0.1518

OR5M9	-0.01026	-0.05162	NA	NA	NA	NA	-0.21732	-0.41473
OR5P2	0.309086	0.666829	0.245398	0.381569	0.388197	0.279372	0.051687	0.413148
OR5P3	-0.28057	-0.28865	0.068341	-0.08137	-0.26696	0.165907	-0.02651	-0.10994
OR5R1	-0.08715	0.314471	NA	NA	NA	NA	0.447706	0.162498
OR5T1	-0.37643	0.004254	NA	NA	NA	NA	-0.12037	-0.13081
OR5T2	-0.13819	0.444961	-0.12015	-0.0574	0.086627	-0.01259	0.258142	0.50114
OR5T3	0.220548	0.18541	NA	NA	NA	NA	0.121454	-0.0658
OR5V1	0.188842	0.060287	0.183899	-0.22885	0.218255	-0.30424	0.320898	0.06907
OR5W2	0.144069	0.017042	NA	NA	NA	NA	0.099452	0.115022
OR6A2	0.26975	0.385284	0.060777	0.142999	0.278668	-0.24473	0.374499	0.01424
OR6B1	0.32722	0.604795	NA	NA	NA	NA	-0.42982	-0.30061
OR6B2	0.057612	0.134581	NA	NA	NA	NA	-0.35524	-0.30211
OR6B3	-0.15678	0.006084	NA	NA	NA	NA	0.256502	-0.32705
OR6C1	0.485386	0.457529	NA	NA	NA	NA	-0.33183	-0.2398
OR6C2	-0.2451	-0.25353	NA	NA	NA	NA	0.411233	0.095893
OR6C3	0.068219	0.070623	NA	NA	NA	NA	-0.34988	0.23376
OR6C4	0.066682	0.17737	NA	NA	NA	NA	-0.2216	-0.16175
OR6C6	-0.21361	0.455424	NA	NA	NA	NA	0.317482	0.229502
OR6C65	0.176436	0.394464	NA	NA	NA	NA	0.345423	-0.10747
OR6C68	0.217075	0.655245	NA	NA	NA	NA	0.124362	0.010399
OR6C70	-0.2261	-0.04236	NA	NA	NA	NA	0.036169	-0.01076
OR6C74	-0.11592	0.239711	NA	NA	NA	NA	0.223967	0.09828
OR6C75	0.001984	0.136861	NA	NA	NA	NA	0.363581	-0.00944
OR6C76	0.068667	0.099511	NA	NA	NA	NA	-0.31299	-0.40066
OR6F1	-0.28827	-0.26149	NA	NA	NA	NA	0.174753	-0.09336
OR6K2	0.148961	0.3963	0.287871	-0.00488	0.067638	-0.05029	-0.09317	-0.0971
OR6K3	0.341073	0.737638	NA	NA	NA	NA	-0.12205	-0.6434
OR6K6	0.040763	0.252951	NA	NA	NA	NA	0.233983	0.031483
OR6M1	0.29756	0.445975	0.028763	-0.13667	0.018767	0.037796	-0.42625	-0.13656
OR6N1	0.201451	-0.04186	0.23485	-0.15916	-0.06042	0.028093	-0.32844	0.03418
OR6N2	0.106516	0.338346	NA	NA	NA	NA	-0.4023	-0.22293
OR6Q1	0.12209	0.663243	NA	NA	NA	NA	0.023691	-0.36963
OR6S1	0.074776	0.719045	NA	NA	NA	NA	0.170565	0.152363
OR6T1	0.117331	0.643516	NA	NA	NA	NA	0.328936	-0.1806
OR6V1	0.400644	-0.0841	NA	NA	NA	NA	-0.08978	-0.1707
OR6W1P	NA	NA	0.326053	-0.2923	-0.07428	0.031777	0.174365	-0.09598
OR6X1	-0.03798	-0.37201	NA	NA	NA	NA	0.142167	0.143146
OR6Y1	0.338091	0.720663	-0.05982	-0.25431	-0.09939	0.10571	0.134079	0.525087
OR7A10	0.249893	0.718554	-0.16067	0.115873	-0.25149	-0.28848	0.342853	0.220243
OR7A17	0.314101	0.04048	0.075276	0.027141	0.140765	0.124861	-0.08031	-0.11517
OR7A5	0.277575	0.392247	0.045525	-0.14661	0.142633	-0.06008	0.375769	0.341762
OR7C1	0.11589	0.42698	NA	NA	NA	NA	-0.20109	0.05891
OR7C2	-0.17097	0.585098	-0.07365	0.155906	0.051617	-0.27103	-0.11363	-0.20225
OR7D2	-0.20013	-0.20424	-0.02968	-0.23633	-0.02063	0.280891	0.226315	-0.13835
OR7D4	0.212815	0.19913	0.050498	-0.04562	0.094138	-0.06065	0.267053	0.200644
OR7E156P	NA	NA	0.259487	-0.28536	0.115053	-0.61123	0.54102	0.29667
OR7E24	NA	NA	0.183767	-0.3253	0.05722	-0.40599	-0.26836	-0.45223
OR7E91P	NA	NA	-0.16598	-0.35288	-0.07479	-0.17389	0.267515	0.170397
OR7G1	-0.32389	-0.29697	NA	NA	NA	NA	0.293163	0.140376
OR7G2	0.149589	-0.07947	NA	NA	NA	NA	-0.14129	0.222862
OR7G3	0.289194	-0.0146	-0.13574	-0.33531	-0.29888	-0.34492	-0.11875	0.120397
OR8A1	-0.02475	0.389713	NA	NA	NA	NA	-0.2281	-0.2979

OR8B12	NA	NA	NA	NA	NA	NA	-0.53345	-0.31393
OR8B2	0.543364	0.347769	NA	NA	NA	NA	0.038562	0.11766
OR8B3	0.241318	-0.21466	NA	NA	NA	NA	0.142249	0.281298
OR8B4	-0.08311	-0.37956	NA	NA	NA	NA	0.239819	-0.17026
OR8B8	0.156117	0.692731	0.410364	-0.09425	0.22064	-0.06952	-0.10505	-0.33492
OR8D1	0.023484	0.508237	-0.0186	0.104644	0.05345	-0.12191	-0.25413	-0.48713
OR8D2	0.072531	0.09676	-0.08762	-0.15972	-0.0165	-0.0604	0.041219	0.055223
OR8D4	0.375475	0.341218	NA	NA	NA	NA	0.463249	-0.1237
OR8G1	-0.10325	0.480009	0.093442	-0.20213	-0.37569	0.261598	-0.09683	0.080341
OR8G2	-0.07001	0.510794	-0.02231	0.266774	0.095503	-0.25095	-0.3221	0.025646
OR8G5	0.304685	0.425497	NA	NA	NA	NA	0.39854	-0.18551
OR8H1	-0.03056	0.561546	0.190539	0.048259	-0.32786	0.119579	-0.35638	-0.12017
OR8H2	0.385597	0.276299	NA	NA	NA	NA	-0.47918	0.455263
OR8H3	-0.06166	0.245257	NA	NA	NA	NA	-0.55303	-0.55974
OR8I2	0.361603	0.346543	NA	NA	NA	NA	-0.17163	-0.26258
OR8J1	0.117109	0.20659	-0.07215	-0.18969	-0.01159	0.164629	0.250529	0.517679
OR8J3	0.080968	0.280316	NA	NA	NA	NA	0.506499	0.596624
OR8K1	-0.09804	0.029713	NA	NA	NA	NA	0.521808	0.504918
OR8K3	0.33417	0.537506	0.252324	0.315072	0.092722	-0.02119	-0.20278	-0.18628
OR8K5	0.219074	0.501614	NA	NA	NA	NA	-0.37709	-0.35139
OR8S1	0.096513	0.349503	NA	NA	NA	NA	-0.53876	-0.23059
OR8U1	NA	NA	0.316617	-0.15167	0.288012	-0.06954	-0.34603	-0.08647
OR8U8	-0.15577	0.117324	NA	NA	NA	NA	0.342617	0.108138
OR8U9	0.014983	0.711671	NA	NA	NA	NA	0.399389	0.541598
OR9A2	0.513245	0.175581	NA	NA	NA	NA	-0.04778	0.236947
OR9A4	-0.07599	0.641419	NA	NA	NA	NA	-0.16685	-0.02508
OR9G1	0.381099	0.577448	NA	NA	NA	NA	-0.41666	-0.18805
OR9G4	0.1951	0.553619	NA	NA	NA	NA	0.325012	0.014678
OR9I1	-0.11364	0.371596	NA	NA	NA	NA	0.223659	0.231471
OR9K2	0.469595	0.143475	NA	NA	NA	NA	-0.03621	0.131208
OR9Q1	-0.17727	0.078255	NA	NA	NA	NA	-0.07925	-0.10124
OR9Q2	0.101563	0.592533	NA	NA	NA	NA	0.088979	0.293537
ORAI1	NA	NA	-0.01	0.522011	0.040335	0.298737	NA	NA
ORAI2	NA	NA	0.370523	0.565193	0.307074	0.463956	NA	NA
ORAI3	NA	NA	0.157599	-0.00696	0.106323	-0.1868	NA	NA
ORAOV1	-0.11737	0.371747	-0.28523	0.118559	-0.02612	-0.26746	0.117663	0.354208
ORC1L	-0.19839	-0.33658	0.075175	0.191767	0.35038	0.019586	0.497499	0.390085
ORC2L	-0.02782	0.600273	-0.01945	0.110398	0.148889	0.289365	0.024722	0.045778
ORC3L	0.601392	-0.52341	-0.3305	-0.16598	-0.02519	-0.13905	0.073077	-0.35984
ORC4L	0.562619	-0.45061	-0.20015	-0.31166	-0.21448	-0.38506	0.183203	0.046804
ORC5L	0.176126	0.302013	-0.07119	-0.2153	0.140186	-0.00157	-0.06345	-0.22124
ORC6L	0.040351	0.765478	-0.24847	-0.03989	0.403906	0.201502	0.629286	0.449229
ORM1	-0.31057	0.253334	-0.0252	0.067187	0.247266	0.32999	0.394478	0.082179
ORM2	-0.19925	0.223773	-0.12036	-0.15105	-0.04596	0.067597	0.437421	0.023147
ORMDL1	-0.07157	-0.43257	0.121633	-0.16423	0.066245	-0.19759	-0.01703	0.022307
ORMDL2	0.082157	0.50314	-0.01155	-0.18764	-0.2728	-0.57057	-0.33657	-0.26266
ORMDL3	-0.04354	0.49964	0.560438	0.20999	0.017017	-0.15267	0.135482	0.030133
OS9	-0.40155	0.020245	0.20815	0.281854	0.038898	0.035573	-0.0233	-0.08627
OSBP	-0.28062	0.226402	0.115266	0.137974	-0.02267	-0.12822	0.100018	0.169912
OSBP2	0.24916	0.594183	0.252183	-0.20706	0.523437	0.406151	0.320505	0.239641
OSBPL10	0.090261	-0.12883	-0.18745	0.449805	0.162545	0.082339	0.372616	0.115657
OSBPL11	0.100984	-0.4742	-0.22557	-0.41606	0.090023	-0.16169	-0.61726	-0.25596

OSBPL1A	0.409914	0.054697	0.019699	-0.42617	0.042848	-0.34289	-0.41183	-0.36257
OSBPL2	0.205133	-0.15382	-0.151	0.028095	0.375923	-0.12622	0.061973	-0.64298
OSBPL3	0.377752	0.624259	-0.16066	0.448563	-0.06806	0.336826	0.452403	0.459997
OSBPL5	-0.10561	0.60112	-0.01134	0.148359	0.193053	0.202716	-0.25313	0.035293
OSBPL6	-0.43819	-0.00073	0.170606	0.162877	-0.23054	-0.10369	-0.0551	0.044772
OSBPL7	-0.0957	0.046194	0.106287	0.06822	0.092605	-0.10562	0.345398	0.173105
OSBPL8	-0.12348	-0.45279	-0.23761	-0.17229	-0.10754	0.296967	-0.66371	-0.22598
OSBPL9	-0.1567	-0.43188	0.229719	-0.23693	0.108807	0.138914	0.42802	0.412607
OSCAR	-0.49649	-0.34429	0.282517	-0.26802	-0.09756	0.301235	-0.33278	0.129699
OSGEP	-0.46643	-0.57596	-0.14882	0.398282	-0.23419	-0.19467	-0.27497	-0.08646
OSGEPL1	-0.03888	-0.10591	-0.18979	0.115682	-0.00647	-0.04375	0.214205	0.297404
OSGIN1	NA	NA	0.465569	-0.11766	0.188939	-0.10561	0.416143	0.39466
OSGIN2	0.150637	0.204501	0.035479	-0.25456	-0.06689	-0.10658	NA	NA
OSM	-0.46489	-0.20307	-0.01024	0.280787	0.094835	0.064159	-0.4154	-0.12424
OSMR	-0.23252	-0.39107	0.023569	0.229689	0.105073	0.491968	-0.37431	0.190532
OSR1	0.112586	0.192285	0.077861	-0.03561	-0.39163	-0.10702	-0.14747	-0.01471
OSR2	0.065787	-0.08576	0.034333	0.073943	-0.22456	0.026583	0.416285	0.621903
OSTalpha	NA	NA	0.009387	-0.27182	-0.04002	-0.10819	0.215547	0.226482
OSTBETA	0.263682	-0.69488	NA	NA	NA	NA	NA	NA
OSTC	NA	NA	0.104252	-0.08991	-0.08809	-0.03693	NA	NA
OSTF1	-0.50677	-0.15577	0.035452	-0.11465	0.023936	-0.499	0.043851	0.372324
OSTM1	0.42109	-0.46799	-0.27072	-0.35881	0.110534	-0.27843	-0.15639	0.100378
OSTN	0.210729	0.771031	NA	NA	NA	NA	-0.3965	0.218265
OTC	0.231163	0.33024	0.180237	0.183193	0.145855	-0.17285	0.005563	0.382076
OTOA	-0.17655	0.456075	-0.0832	-0.11634	0.33316	0.383461	0.264871	-0.07509
OTOF	-0.02144	0.477593	0.060884	-0.06052	0.160161	-0.03389	0.08396	-0.12456
OTOL1	NA	NA	0.243985	-0.12123	0.30143	0.05394	NA	NA
OTOP1	0.285025	0.544249	0.0973	0.187488	-0.18618	0.154511	-0.11356	-0.48633
OTOP2	-0.10349	-0.02411	-0.0525	-0.28217	0.39575	-0.17515	-0.0734	-0.28132
OTOP3	0.225173	0.567902	0.018554	-0.24199	-0.03926	0.061209	0.17579	0.353122
OTOR	-0.07158	0.26274	0.019059	-0.13464	-0.06438	0.076412	0.262826	0.032709
OTOS	0.013148	0.106448	0.116441	-0.31558	0.049604	-0.36666	0.229643	0.274541
OTP	-0.21667	0.318964	-0.16256	-0.20417	0.378056	-0.25977	-0.47082	-0.46162
OTUB1	0.214248	0.644944	0.199763	0.07639	-0.13601	-0.09488	0.162068	-0.09515
OTUB2	0.269447	-0.23791	0.170095	0.086969	0.152686	-0.10655	0.339491	0.226481
OTUD1	NA	NA	0.371188	-0.05596	-0.01257	0.011261	NA	NA
OTUD3	NA	NA	-0.19101	0.033097	0.090394	0.081871	NA	NA
OTUD4	-0.31431	-0.58505	-0.2792	-0.09057	-0.20821	-0.00112	0.498829	0.415559
OTUD5	0.246268	0.574087	0.152583	0.170619	0.244097	0.322098	-0.53602	-0.35117
OTUD6A	0.257348	0.217888	-0.14452	-0.09887	-0.1297	-0.40707	0.277292	-0.15945
OTUD6B	0.030305	-0.52295	-0.14277	0.027379	-0.00819	0.135263	0.241146	-0.16827
OTUD7A	0.074672	0.588005	0.098481	-0.1596	0.032336	-0.02042	-0.39309	-0.05412
OTUD7B	0.06084	0.172877	-0.12572	-0.20861	-0.00661	0.1311	NA	NA
OTX1	-0.22866	0.260379	0.088191	-0.2421	-0.01586	-0.08042	-0.01537	-0.25291
OTX2	-0.02882	0.398852	-0.2211	0.035888	0.021076	-0.36564	0.456384	0.322825
OVCA2	-0.20781	-0.36042	-0.08691	0.100971	-0.20393	0.109324	0.032226	-0.10332
OVCH1	0.081613	0.232977	NA	NA	NA	NA	-0.2295	0.072115
OVCH2	0.099543	0.634469	NA	NA	NA	NA	-0.03047	-0.36176
OVGP1	0.103469	0.267746	0.274951	-0.12242	0.144132	-0.03129	-0.06033	-0.20019
OVOL1	0.278105	0.29271	-0.10318	0.117227	0.507556	-0.03863	0.162089	0.018322
OVOL2	-0.02817	0.17399	-0.00352	-0.05742	-0.14235	0.173503	0.524963	0.077606
OVOS2	NA	NA	-0.21961	0.595354	0.056849	0.22549	-0.34126	-0.32715

OXA1L	0.077873	-0.19617	-0.01667	0.298438	-0.06931	0.262985	0.30433	0.327628
OXCT1	-0.13698	-0.248	-0.29727	0.14136	0.225092	-0.06281	0.079076	0.061233
OXCT2	0.027847	0.266438	0.16096	-0.01715	0.207846	0.151978	-0.18981	0.099005
OXER1	0.506912	0.717439	0.121917	0.076525	0.009786	-0.08112	-0.13427	0.226852
OXGR1	0.169385	-0.18155	0.005618	-0.10575	0.044637	-0.04422	0.12317	0.058611
OXNAD1	0.053553	-0.19455	-0.35219	0.037842	-0.09131	-0.21215	NA	NA
OXR1	0.160516	-0.01998	0.240134	-0.32222	-0.15822	-0.39018	0.3359	-0.10659
OXSM	0.150267	0.230195	-0.12957	-0.36109	-0.00594	-0.04898	-0.2002	-0.15591
OXSR1	-0.31185	-0.4193	-0.32207	-0.22077	0.095576	0.250998	0.463112	0.314719
OXT	0.047375	0.15787	-0.15987	0.044107	-0.26823	-0.13381	0.028357	0.466717
OXTR	0.050614	-0.19314	-0.18602	0.057309	0.21291	-0.3843	0.017425	-0.12434
P11	-0.17929	0.152951	-0.41472	0.352724	-0.3183	0.223583	0.410162	0.11692
P2RX1	0.405172	0.172392	-0.07531	0.005244	-0.07746	0.441772	-0.24275	0.440847
P2RX2	0.28345	0.733211	-0.04416	-0.01781	-0.04295	-0.02161	-0.04792	0.231744
P2RX3	0.306124	0.649547	0.127575	-0.13274	0.199044	-0.29003	-0.15665	-0.14673
P2RX4	-0.32897	0.119168	0.177022	-0.0896	0.08662	-0.35194	0.281411	0.110088
P2RX5	0.281744	0.456009	-0.04361	0.627355	0.041558	0.591426	-0.18078	0.242499
P2RX6	NA	NA	0.106806	-0.19967	0.035786	-0.13879	NA	NA
P2RX7	0.089007	0.600127	0.270785	-0.32524	-0.22854	0.326468	-0.49337	-0.61804
P2RY1	-0.10699	0.539078	-0.1993	-0.44377	-0.31783	-0.23193	0.016772	0.209071
P2RY10	-0.41327	0.253676	0.317491	0.370042	-0.2568	0.474699	-0.49784	0.171017
P2RY11	-0.02864	0.250194	NA	NA	NA	NA	-0.26888	-0.14193
P2RY12	-0.27294	-0.06259	0.225894	0.094174	-0.36712	0.14569	NA	NA
P2RY13	-0.52017	0.490213	0.328328	0.034346	-0.07843	-0.07954	-0.54739	-0.0717
P2RY14	-0.47741	-0.40369	-0.01463	-0.20855	-0.12834	-0.18598	0.012253	0.14542
P2RY2	0.187275	0.704959	-0.07264	0.0282	0.192219	-0.26276	-0.00963	0.271398
P2RY4	0.461526	0.631913	-0.00204	-0.25304	0.143522	-0.28281	-0.15786	-0.09008
P2RY5	-0.04414	-0.33859	0.183691	-0.43451	-0.27108	0.149015	-0.17016	0.371964
P2RY6	0.105013	0.302346	0.188589	-0.39618	-0.07553	0.00902	-0.5613	-0.13331
P2RY8	-0.162	-0.00267	0.174687	0.738459	-0.20989	0.594282	-0.28381	0.260469
P4HA1	-0.47145	-0.44606	-0.23318	-0.00191	-0.12102	-0.23342	-0.04535	-0.14114
P4HA2	-0.44609	-0.62411	0.005704	0.120137	0.084436	-0.34278	0.183878	-0.08637
P4HA3	0.348566	0.772635	0.153346	-0.13777	0.084613	-0.3467	-0.49866	-0.3055
P4HB	-0.50631	0.26559	0.306186	0.389845	0.309885	0.19628	NA	NA
P4HTM	NA	NA	0.068588	0.163837	0.30314	0.195176	NA	NA
P53AIP1	0.100356	0.66498	-0.13792	-0.09799	0.130133	0.225895	0.311719	0.346491
PA2G4	0.346045	0.489707	-0.38488	0.414911	0.114994	0.19233	0.12516	-0.06529
PAAF1	NA	NA	0.238628	-0.13321	0.023362	-0.06291	NA	NA
PABPC1	0.266864	-0.53714	0.257497	0.301057	0.222837	0.532293	-0.48294	-0.59585
PABPC1L	NA	NA	0.214751	0.273122	0.213298	0.45754	NA	NA
PABPC1L2B	NA	NA	0.405575	0.009162	-0.01895	0.263964	NA	NA
PABPC3	0.129413	-0.50624	-0.06439	0.11604	-0.18686	0.153918	0.027348	0.337009
PABPC4	NA	NA	0.169636	0.37289	0.163347	-0.04038	0.67155	0.327087
PABPC4L	NA	NA	-0.0034	-0.20242	-0.12662	0.306495	NA	NA
PABPC5	0.296819	0.265521	0.403252	0.023711	0.022426	0.156988	-0.08953	0.040619
PABPN1	-0.00298	0.568016	-0.14549	0.559681	0.151597	0.415407	0.290312	0.443052
PACRG	0.298622	-0.23682	0.031383	-0.374	-0.07233	-0.28768	-0.09721	-0.4493
PACRGL	NA	NA	-0.26208	0.041599	-0.19987	-0.2203	NA	NA
PACS1	-0.23289	0.104887	0.190182	0.356748	0.188101	0.654706	0.007065	0.145757
PACS2	0.038385	0.074122	0.08562	-0.00961	0.151267	0.057456	0.178891	0.166831
PACSIN1	-0.31164	-0.16004	-0.09197	0.63135	-0.19208	0.682772	-0.42198	-0.32189
PACSIN2	-0.42386	-0.04488	-0.01906	0.022966	-0.00814	-0.11988	0.141015	-0.24824

PACSIN3	0.027114	-0.02061	0.262462	-0.16634	0.281678	0.098686	0.019814	-0.28837
PADI1	0.058562	0.565859	-0.06529	-0.03465	0.068093	-0.19845	-0.14005	-0.2336
PADI2	0.092282	0.185981	0.042266	-0.03272	-0.1557	-0.28138	-0.19683	-0.16586
PADI3	-0.16019	-0.4314	-0.09838	-0.06427	0.048511	-0.26926	-0.05106	-0.47869
PADI4	-0.40187	0.187319	0.350736	-0.08999	-0.17474	-0.10433	-0.38641	0.182467
PADI6	0.058465	0.627014	NA	NA	NA	NA	0.044772	0.221828
PAEP	-0.07853	0.287083	-0.1352	-0.18091	0.048712	0.235784	-0.01179	-0.17926
PAF1	0.000248	0.72141	0.129918	0.335333	0.025034	0.362056	-0.0861	-0.40432
PAFAH1B1	-0.33184	-0.63686	-0.32116	-0.12361	-0.31683	-0.20099	-0.31378	-0.45987
PAFAH1B2	-0.44545	-0.42098	-0.22088	-0.17369	-0.3746	0.064356	-0.04324	0.433413
PAFAH1B3	0.216251	0.471685	-0.07784	0.261525	0.235817	-0.1769	0.518679	-0.00768
PAFAH2	-0.50007	0.070541	0.165566	0.005872	-0.0585	-0.37465	-0.0291	0.192902
PAG1	-0.43844	-0.18208	-0.22603	0.038171	-0.24729	-0.19063	-0.47697	-0.16776
PAGE1	-0.13651	0.321741	-0.32787	-0.26825	0.037671	0.059668	0.357276	-0.03728
PAGE2	0.273538	-0.10527	-0.30217	-0.21058	0.142027	0.024486	-0.20042	-0.02036
PAGE2B	0.341395	0.240305	-0.5772	0.074692	0.09357	-0.06672	-0.14265	-0.31689
PAGE3	-0.25709	-0.18375	NA	NA	NA	NA	0.432227	0.48851
PAGE4	-0.10668	0.033391	-0.23528	-0.21514	0.13169	0.024916	-0.20556	-0.39872
PAGE5	0.238767	0.4622	-0.38839	-0.04062	0.110807	-0.27776	0.110136	-0.4323
PAH	0.183496	0.278144	0.162899	-0.22195	0.243035	-0.16224	-0.02691	0.048683
PAICS	0.076523	-0.22423	-0.14889	0.240895	0.008354	0.201018	0.552799	0.413194
PAIP1	0.304003	0.209165	-0.02407	-0.44451	0.288927	-0.22943	0.27046	-0.01363
PAIP2	-0.07592	-0.63578	0.315856	-0.04368	0.025206	-0.07445	0.139446	-0.19335
PAIP2B	NA	NA	0.135779	0.036453	-0.08457	0.169473	NA	NA
PAK1	0.049602	0.154156	0.106757	0.21218	0.12399	-0.34396	0.573255	0.098236
PAK1IP1	-0.01717	-0.42446	-0.14557	-0.07319	-0.08326	0.163762	-0.43147	-0.41755
PAK2	-0.15442	-0.36284	-0.10319	0.245136	0.188745	-0.2569	0.321857	0.294661
PAK3	-0.30087	-0.38085	-0.16644	0.023758	0.034184	0.165016	0.498885	0.416883
PAK4	-0.11205	0.404512	0.1351	0.008184	0.008095	-0.14796	0.250652	-0.15296
PAK6	-0.05582	-0.06982	-0.09607	0.247765	-0.07023	-0.09637	0.167651	0.226885
PAK7	0.059113	0.685395	-0.00282	0.398429	-0.01484	-0.09089	0.492033	-0.06782
PALB2	0.453721	0.566736	-0.04227	-0.12701	0.232737	0.139335	NA	NA
PALLD	-0.37407	-0.23475	-0.13203	-0.06664	-0.21442	0.168522	-0.40604	-0.42222
PALM	0.24846	-0.02387	0.24381	-0.05017	0.020111	0.027855	-0.16721	-0.10489
PALM2	0.246621	0.04804	0.114548	0.072836	-0.07157	0.193404	0.040278	0.175192
PALM2-AK/	NA	NA	-0.18694	0.255056	-0.14613	-0.02915	0.255326	-0.12034
PALM3	NA	NA	0.306682	-0.018	-0.17641	-0.08803	NA	NA
PALMD	0.147211	-0.15573	-0.10843	0.126346	-0.18337	-0.26526	-0.00882	0.071187
PAM	-0.23719	-0.57659	0.186982	-0.21106	-0.21002	-0.14212	0.180653	-0.07839
PAN2	NA	NA	-0.08308	0.330498	0.34359	0.105115	NA	NA
PAN3	0.274658	-0.43215	0.258941	0.048501	0.312351	0.421653	0.669955	0.439308
PANK1	0.277972	0.021223	-0.29573	0.081257	0.105534	-0.25455	0.167208	0.009959
PANK2	0.229608	-0.11831	0.076323	0.131469	0.199799	0.188196	0.264537	0.248412
PANK3	0.153816	0.332578	-0.43882	-0.29008	-0.38003	-0.51498	0.307197	0.301184
PANK4	0.034429	-0.30276	0.011628	0.203995	0.243149	0.166469	-0.09585	0.229414
PANX1	-0.17728	-0.28743	-0.08569	0.08203	-0.20916	0.067154	0.132405	0.106277
PANX2	-0.40097	-0.03159	-0.10961	-0.12456	-0.13262	0.228244	-0.56067	-0.03462
PANX3	-0.24696	0.315887	-0.12484	-0.24478	-0.10026	-0.03126	0.623327	0.511384
PAOX	-0.01751	0.036257	0.283143	0.027803	0.238338	-0.08564	-0.4001	0.314
PAP2D	-0.03419	0.003626	-0.11222	-0.05979	-0.3991	0.147129	0.144345	0.026608
PAPD4	-0.13108	-0.67143	-0.12841	-0.08426	-0.26029	-0.08881	0.423835	0.39244
PAPD5	0.20817	0.289973	0.048356	-0.14946	0.237025	-0.00737	-0.49038	-0.51789

PAPLN	-0.07199	-0.67711	0.097569	0.72757	-0.11546	0.676063	-0.40462	-0.24587
PAPOLA	0.192662	-0.30995	-0.35886	-0.13324	0.033601	-0.26995	-0.15729	-0.0148
PAPOLB	-0.22052	0.015279	0.061426	-0.13938	-0.04746	-0.14289	0.227125	0.158573
PAPOLG	0.003104	-0.28182	-0.2637	-0.34475	-0.02006	0.165325	0.311728	0.102706
PAPPA	-0.22442	0.125262	0.020195	0.132062	0.000625	-0.26834	-0.13848	-0.19693
PAPPA2	-0.12326	0.524005	-0.18995	-0.16061	0.056954	-0.02248	0.461491	0.271836
PAPSS1	-0.39621	-0.53421	-0.02682	0.261191	-0.17599	-0.03267	-0.30222	-0.34831
PAPSS2	-0.28169	0.090987	-0.15046	-0.22171	-0.0189	0.205179	-0.05017	0.184971
PAQR3	-0.00901	-0.24799	-0.21423	-0.36347	0.070234	-0.36377	0.079595	0.069199
PAQR4	0.180648	0.492304	0.117731	0.226607	0.430118	-0.07425	0.260715	-0.11187
PAQR5	-0.18365	-0.16767	-0.12016	-0.27124	-0.19255	-0.15172	-0.03662	0.079826
PAQR6	-0.30826	-0.30639	-0.25318	0.192838	-0.0364	-0.29945	0.191723	0.129746
PAQR7	-0.12577	0.103025	0.31326	0.19175	0.407714	-0.17724	-0.12647	0.107732
PAQR8	0.159909	0.214598	0.119504	-0.00129	-0.20195	-0.04188	0.03102	-0.16159
PAQR9	-0.16988	0.026469	-0.07374	0.181357	-0.1417	0.164852	0.447178	0.057246
PARD3	0.288108	-0.26838	-0.256	-0.03555	0.102288	-0.10939	-0.31534	-0.28105
PARD3B	0.101934	0.483322	0.450845	-0.51473	0.080677	-0.17273	0.261935	0.021229
PARD6A	0.217132	0.327527	0.242139	0.160389	0.236105	0.0343	-0.05813	-0.43389
PARD6B	0.413902	-0.03833	-0.24431	-0.36096	0.454252	-0.1579	0.168152	-0.36674
PARD6G	-0.42684	-0.21946	0.115714	0.041278	-0.06434	0.194982	-0.1935	-0.04946
PARG	NA	NA	-0.28956	-0.08293	0.016325	0.013856	0.457713	0.316254
PARK2	0.404835	0.576916	0.197623	-0.53215	0.364593	0.130007	0.153992	0.108345
PARK7	-0.12075	-0.45618	-0.03012	0.023765	0.24039	-0.40327	-0.10333	-0.25764
PARL	-0.05139	0.029259	-0.00215	0.141262	-0.09604	0.250967	-0.20031	-0.25173
PARN	0.43285	0.211829	-0.3429	0.091019	0.065814	-0.16889	-0.49121	-0.56968
PARP1	-0.11222	-0.08511	-0.13998	0.417214	0.109288	0.224305	-0.09446	-0.27511
PARP10	-0.05234	0.264589	0.084052	0.00187	-0.00895	0.259408	-0.50823	-0.09311
PARP11	NA	NA	-0.11819	0.082315	-0.11825	0.15783	0.229043	0.257323
PARP12	-0.10715	0.050603	0.274208	-0.21198	-0.15952	-0.10351	-0.12277	0.276453
PARP14	-0.05695	0.129698	0.334262	-0.19982	-0.24147	0.042362	-0.4355	-0.13871
PARP15	-0.06164	-0.14566	-0.06081	0.633526	-0.22558	0.624084	-0.20861	-0.21138
PARP16	0.158297	-0.00242	0.116916	0.019311	0.148631	0.012465	0.059626	0.191735
PARP2	-0.06789	-0.37142	-0.6243	0.221144	-0.27835	-0.09808	0.09434	0.269918
PARP3	0.447589	0.68983	0.077223	0.187367	0.274347	-0.14471	-0.16239	0.385535
PARP4	0.199611	-0.16329	0.246535	-0.01151	0.064033	-0.22014	0.446828	0.153975
PARP6	-0.17645	0.014935	0.118055	0.180922	0.084326	0.357727	-0.05417	0.297613
PARP8	-0.10437	-0.50795	0.198439	-0.16975	-0.40011	-0.15417	0.054235	-0.12927
PARP9	-0.20069	-0.52786	0.352773	-0.2386	-0.24127	0.224436	-0.68194	-0.40205
PARS2	0.429758	-0.59335	0.313131	-0.046	0.31707	-0.1501	-0.07918	-0.56133
PART1	0.217684	-0.01328	0.134647	0.06518	-0.20827	-0.03689	-0.06548	-0.32141
PARVA	-0.30369	0.045914	-0.25459	0.050987	-0.02436	0.055602	-0.34185	-0.28967
PARVB	-0.03115	0.096665	0.271497	0.229219	0.052233	0.054587	-0.19052	0.07928
PARVG	-0.43518	0.232065	0.435749	0.015941	-0.19283	0.759052	-0.31525	0.534417
PASD1	0.167294	0.209554	-0.10972	0.504947	-0.24693	0.000863	-0.21997	0.127967
PASK	0.120165	0.344608	0.022587	0.746002	0.398164	0.459225	0.337448	0.176848
PATE1	NA	NA	0.0686	-0.05816	0.206996	-0.40398	NA	NA
PATL1	NA	NA	-0.11023	0.016255	0.030294	0.197159	NA	NA
PATZ1	0.00451	0.702573	0.211534	0.440349	0.371689	0.339737	0.077077	-0.15465
PAWR	-0.07513	0.223039	-0.07225	0.065925	0.077643	-0.09297	NA	NA
PAX1	-0.24308	0.364487	-0.12512	0.037819	0.174309	-0.08279	0.623341	0.235606
PAX2	0.19205	-0.14229	-0.27823	-0.15123	0.091099	-0.11744	0.600133	0.439515
PAX3	0.15453	0.591856	0.218936	-0.1966	-0.12516	0.219233	-0.34942	0.040313

PAX4	-0.17543	0.338169	0.336235	-0.35834	0.113106	0.132692	0.560644	0.014269
PAX5	-0.19115	0.002869	0.21594	0.076979	0.091184	0.125136	0.26729	0.231666
PAX6	-0.29547	0.399722	-0.06994	-0.0131	-0.0003	-0.06541	-0.3015	0.373949
PAX7	-0.03836	0.217512	0.242179	-0.32714	0.186028	0.024103	-0.3126	-0.52803
PAX8	0.334862	0.666238	0.067163	-0.13217	0.002948	-0.3892	0.02819	0.543183
PAX9	-0.39889	0.123692	-0.18961	-0.18792	-0.25985	-0.33339	-0.06395	-0.25095
PAXIP1	0.142865	0.093376	-0.02297	0.030402	0.20903	0.247014	0.258548	0.351386
PBK	-0.05897	0.107193	-0.17371	0.138413	-0.09311	-0.13126	0.247199	-0.06083
PBLD	-0.12774	0.047121	-0.06244	-0.28674	0.089864	-0.28492	NA	NA
PBOV1	-0.2394	0.115991	0.141007	0.068098	-0.35413	-0.16339	-0.27372	-0.26247
PBRM1	NA	NA	-0.37293	-0.00439	0.108378	-0.23868	NA	NA
PBX1	0.241164	0.164966	0.113504	-0.15055	-0.03571	0.038569	-0.2247	-0.29972
PBX2	0.331428	0.24141	0.018865	0.385145	0.004651	0.324424	-0.15901	0.197245
PBX3	-0.07237	-0.09951	0.089275	0.077479	0.181913	0.199646	-0.43695	-0.38753
PBX4	-0.0348	-0.00756	0.161606	0.276418	0.248051	0.591496	0.131196	0.582229
PBXIP1	-0.08962	0.105915	0.39288	0.057698	0.000595	0.281991	0.16274	0.1293
PC	-0.4355	0.566222	-0.07194	-0.11653	0.355709	-0.03142	-0.18879	0.184082
PCBD1	0.261061	0.16043	0.275554	0.105856	-0.01374	-0.22274	0.690817	-0.00823
PCBD2	-0.0942	-0.44136	0.241838	-0.15395	0.240138	0.01653	0.261998	0.310337
PCBP1	0.140935	-0.63136	0.077883	0.092577	0.22833	0.409267	-0.35821	-0.51904
PCBP2	0.065331	-0.06146	0.220488	0.001624	0.100874	-0.16477	0.072504	-0.14684
PCBP3	-0.11933	-0.22625	0.174919	0.413545	0.280192	0.219939	-0.37171	0.081403
PCBP4	0.052152	0.27128	-0.00319	-0.05296	0.341847	-0.28657	-0.44237	-0.58482
PCCA	-0.43651	0.201926	0.007417	-0.19903	0.006439	-0.20646	0.008808	-0.12075
PCCB	-0.17919	0.143756	-0.06528	0.161562	-0.17882	-0.00029	0.061027	0.128973
PCDH1	0.117532	-0.2332	0.322289	-0.07972	0.404882	0.036971	0.3035	0.041077
PCDH10	0.270463	0.529523	-0.10236	-0.19942	0.122092	0.201179	0.014253	0.032168
PCDH11X	0.003492	0.528771	-0.0871	0.161744	-0.11473	-0.14146	0.607896	0.345085
PCDH11Y	0.188397	0.648457	-0.06068	0.331318	0.093469	0.268403	0.551086	0.386419
PCDH12	-0.35256	-0.30854	0.178972	0.215534	0.182786	0.369306	-0.40013	-0.01605
PCDH15	-0.13441	0.075781	0.004867	0.48599	-0.00824	-0.25548	0.104527	-0.12237
PCDH17	-0.20542	-0.41407	-0.00576	0.251613	-0.09132	0.162162	-0.51759	-0.33029
PCDH18	0.116573	-0.51101	-0.16704	-0.17021	-0.25128	0.059792	-0.36059	-0.20488
PCDH19	0.096693	-0.35341	0.293835	0.034947	0.017766	0.165688	0.593008	0.059654
PCDH20	-0.13074	-0.10208	-0.08926	-0.18909	0.162541	0.137121	0.183598	-0.1207
PCDH21	0.025373	0.034335	0.131727	0.011575	0.170515	0.1815	-0.24664	-0.18369
PCDH7	-0.06732	-0.06875	0.027141	-0.03043	-0.12634	0.199739	-0.16012	-0.08031
PCDH8	0.030849	-0.41416	-0.10553	-0.25262	-0.14273	-0.12868	0.325376	0.115743
PCDH9	-0.14058	0.12761	-0.20196	-0.15877	0.014848	0.062105	-0.32434	-0.27367
PCDHA1	0.147348	0.271316	-0.16776	0.110184	0.235601	0.075163	0.160259	-0.02051
PCDHA10	-0.02169	0.532369	NA	NA	NA	NA	0.132117	0.059462
PCDHA11	-0.21691	0.155423	-0.2828	0.502808	0.050505	0.516541	-0.18921	-0.26571
PCDHA12	0.12132	0.2736	NA	NA	NA	NA	0.420527	-0.375
PCDHA13	0.378836	0.353416	NA	NA	NA	NA	0.085832	0.015418
PCDHA2	0.462753	0.710421	NA	NA	NA	NA	-0.0473	-0.14208
PCDHA3	-0.18283	-0.32095	NA	NA	NA	NA	-0.23015	-0.48208
PCDHA4	0.309328	0.184924	NA	NA	NA	NA	0.137516	-0.06925
PCDHA5	-0.08521	-0.09495	-0.27502	0.19742	0.213954	0.000176	-0.72508	-0.59052
PCDHA6	0.195151	0.344649	NA	NA	NA	NA	0.480061	0.156718
PCDHA7	0.236364	0.372974	NA	NA	NA	NA	0.408397	0.310974
PCDHA8	0.236299	-0.40802	-0.02817	-0.47729	0.081355	-0.04379	-0.00885	0.266798
PCDHA9	0.267046	-0.11718	-0.23984	0.314779	0.064406	0.096178	0.129219	0.107156

PCDHAC1	0.269026	-0.08434	-0.04816	0.255979	0.112532	0.358671	-0.1512	-0.28981
PCDHAC2	0.472908	0.763225	0.318335	0.16143	-0.216	0.044649	0.0806	-0.00494
PCDHB1	0.091042	0.402414	0.236555	-0.18835	0.20213	-0.06822	0.404933	0.651909
PCDHB10	-0.05801	-0.0438	-0.10362	-0.05508	-0.044	0.13504	-0.04259	-0.13396
PCDHB11	0.131359	0.548662	0.031606	-0.08973	0.147588	0.267284	0.085553	-0.0338
PCDHB12	-0.13576	-0.22728	0.150851	-0.0502	-0.18213	0.013108	-0.2771	-0.18949
PCDHB13	-0.24621	-0.39523	0.185028	-0.04241	-0.22934	-0.06578	0.146758	-0.12845
PCDHB14	0.164703	0.595872	-0.03477	-0.16272	0.083542	0.183935	-0.00029	-0.12095
PCDHB15	-0.12483	-0.27365	0.16317	-0.02491	0.412109	0.425238	-0.48567	-0.22792
PCDHB16	0.014144	-0.25205	-0.06176	-0.06216	-0.19608	0.032874	-0.048	-0.21609
PCDHB17	NA	NA	-0.05121	-0.44109	-0.07536	0.230027	0.059553	0.13042
PCDHB18	NA	NA	-0.36789	-0.15994	-0.10438	0.25371	-0.25514	-0.31242
PCDHB2	-0.15205	0.100735	0.096366	0.227771	-0.01445	0.108789	0.086313	0.006318
PCDHB3	-0.25518	-0.28565	NA	NA	NA	NA	-0.53899	-0.32895
PCDHB4	-0.04359	-0.05614	-0.11921	-0.12641	-0.30648	0.092779	0.075258	-0.03752
PCDHB5	-0.23637	-0.28018	0.041046	-0.19384	-0.13456	0.281687	-0.14071	0.045207
PCDHB6	-0.03244	0.287555	0.098901	-0.23041	0.149592	0.095233	0.190302	-0.27581
PCDHB7	-0.10451	0.016369	0.172602	-0.08296	-0.14892	-0.03427	-0.05871	-0.11035
PCDHB8	0.207199	0.169849	-0.17529	-0.12176	-0.10753	-0.01766	0.378642	-0.05643
PCDHB9	0.093948	-0.14486	-0.08984	-0.32779	-0.04671	0.174992	-0.5742	-0.55655
PCDHGA1	0.444148	0.572241	NA	NA	NA	NA	-0.11406	-0.17029
PCDHGA10	-0.16441	-0.19648	NA	NA	NA	NA	-0.11892	-0.24736
PCDHGA11	0.131655	-0.13841	NA	NA	NA	NA	-0.07748	0.006105
PCDHGA12	0.450007	-0.08585	0.154246	-0.43767	0.043708	0.227452	-0.26576	-0.06847
PCDHGA2	0.097262	0.13252	0.024925	-0.42622	-0.06437	-0.07222	0.589054	0.558908
PCDHGA3	0.07887	0.444533	-0.31479	-0.30496	-0.0733	-0.10532	0.224162	-0.01475
PCDHGA4	0.398085	0.49226	NA	NA	NA	NA	0.231204	0.289456
PCDHGA5	-0.16182	0.400485	-0.14454	-0.36135	0.102861	-0.29847	-0.08985	-0.24184
PCDHGA6	0.099818	-0.00142	NA	NA	NA	NA	0.580086	0.523622
PCDHGA7	0.339562	0.306056	-0.15313	-0.27748	0.085038	0.045712	-0.17374	-0.02152
PCDHGA8	-0.02145	0.385915	0.227532	0.195126	0.156184	0.305549	-0.11584	-0.16513
PCDHGA9	0.54288	0.374803	-0.25843	-0.08813	0.0842	0.034597	-0.40066	0.294872
PCDHGB1	0.098865	0.477996	-0.0541	-0.34132	-0.03646	0.003772	0.130793	-0.22545
PCDHGB2	0.029778	-0.01663	-0.30148	-0.02813	0.323799	0.144223	-0.38568	-0.11885
PCDHGB3	0.410567	0.157312	-0.02566	0.216051	-0.06861	0.07787	0.30606	0.062173
PCDHGB4	0.097342	0.397603	-0.31168	-0.12074	0.132098	-0.04933	-0.41943	-0.40565
PCDHGB5	0.361203	0.650733	NA	NA	NA	NA	0.063138	-0.09637
PCDHGB6	0.453396	0.674069	-0.0262	-0.07253	0.016027	0.238556	0.195538	0.286679
PCDHGB7	0.217803	0.402825	-0.02647	0.268722	0.202382	-0.1096	0.514183	0.133472
PCDHGC3	0.232147	0.574321	0.026009	0.261433	-0.08377	-0.13272	-0.15305	0.127419
PCDHGC4	0.087297	0.393053	-0.14502	0.117181	-0.12741	-0.0223	0.249772	0.272076
PCDHGC5	0.251167	0.740235	0.268566	-0.19279	-0.23465	0.124887	0.488717	0.340859
PCF11	-0.1978	-0.32904	-0.17113	-0.07353	-0.23288	0.027007	0.103219	0.30035
PCGF1	0.345803	-0.19776	-0.02029	-0.00539	-0.15384	-0.3055	0.583183	0.272059
PCGF2	-0.36455	-0.0672	0.122367	-0.22901	0.117877	-0.23046	0.344754	0.172509
PCGF3	0.122112	0.123449	-0.12103	-0.12717	0.296726	-0.10048	-0.31383	-0.50015
PCGF5	0.059683	-0.51102	-0.12516	-0.39182	0.1882	-0.6573	-0.06451	0.216118
PCGF6	-0.17875	-0.54178	-0.2531	-0.35168	0.230566	-0.04949	0.065894	0.06323
PCID2	0.288313	-0.43789	0.049359	0.207967	0.391448	0.365018	NA	NA
PCIF1	NA	NA	0.418679	0.007698	0.221958	0.051743	NA	NA
PCK1	0.132591	-0.2653	-0.02285	-0.0308	0.121378	-0.26663	-0.11313	-0.30159
PCK2	0.431729	-0.01446	-0.08603	-0.02569	0.130263	-0.06886	0.317256	0.505762

PCLO	NA	NA	0.013738	-0.08474	0.144504	-0.61683	NA	NA
PCM1	-0.05266	-0.63338	0.217734	-0.25754	-0.12078	0.057095	-0.44051	-0.48528
PCMT1	0.437656	0.136363	-0.27308	-0.0036	-0.05277	-0.32301	0.299995	-0.28913
PCMTD1	0.048113	-0.34346	0.322954	-0.00873	-0.08037	-0.20452	-0.41044	-0.60535
PCMTD2	0.211339	-0.74664	0.10569	-0.10134	0.121751	0.423266	0.750346	0.50392
PCNA	0.023407	-0.58368	-0.41007	0.303617	0.009083	0.007026	0.336287	-0.0562
PCNP	-0.13805	-0.52623	-0.12071	-0.15077	0.112622	0.19202	0.259569	0.034704
PCNT	-0.24039	0.171002	-0.31194	0.234702	0.033167	0.116647	NA	NA
PCNX	-0.03941	0.649211	0.329235	-0.21828	-0.06036	0.535561	-0.22425	0.205393
PCNXL2	0.336506	0.721445	-0.31567	0.507797	0.091171	0.324589	0.368064	0.252494
PCNXL3	NA	NA	0.119331	0.067168	0.308533	-0.03826	NA	NA
PCOLCE	-0.25928	-0.23576	0.199889	0.305667	-0.07527	0.539977	-0.07993	0.031811
PCOLCE2	0.130334	-0.26705	0.155637	0.067313	-0.42254	0.053729	-0.15754	-0.04151
PCP2	-0.05677	0.08689	-0.25014	0.067415	0.196193	-0.03271	0.321749	0.067985
PCP4	0.039057	-0.14197	0.040677	-0.08351	0.108625	-0.22948	-0.01179	-0.34611
PCP4L1	NA	NA	-0.00154	-0.02774	-0.00517	0.010592	NA	NA
PCSK1	-0.25126	-0.29574	-0.18733	0.298326	0.098925	-0.19614	-0.02535	0.07357
PCSK1N	-0.09008	-0.01962	0.055214	0.021241	-0.11973	0.110261	0.087006	0.160336
PCSK2	-0.01275	-0.46469	-0.08093	-0.12011	0.003613	-0.02327	-0.31994	-0.49205
PCSK4	-0.28598	0.158285	-0.08108	-0.13382	-0.00614	-0.15964	-0.0696	0.055581
PCSK5	-0.15153	-0.19068	0.132402	-0.47517	0.11481	0.227741	NA	NA
PCSK6	-0.05804	0.16274	0.007882	0.102091	-0.14722	-0.16568	-0.14107	0.043141
PCSK7	-0.06611	0.242874	0.213148	0.1507	0.186988	-0.1111	0.269966	0.22644
PCSK9	-0.07807	0.546143	-0.38321	0.121023	-0.1768	-0.02804	-0.05866	-0.17821
PCTK1	0.276368	0.580084	0.05668	0.02119	0.15214	-0.13476	0.430134	0.430022
PCTK2	-0.27304	-0.36691	-0.18236	-0.04932	-0.17209	-0.14933	-0.49601	-0.28426
PCTK3	0.49662	0.355041	0.159458	0.109691	0.123229	-0.14713	0.629848	0.503443
PCTP	0.188144	-0.61304	0.013677	-0.18471	-0.16235	-0.40895	-0.04787	-0.40008
PCYOX1	-0.37485	-0.31812	-0.12094	-0.25716	0.030752	-0.14535	-0.51665	-0.60079
PCYOX1L	0.05761	-0.38684	0.381794	0.389311	-0.01941	0.220284	NA	NA
PCYT1A	0.017236	0.662913	-0.29001	-0.09941	-0.32463	-0.15724	-0.12088	0.244378
PCYT1B	-0.1564	0.202853	-0.00468	0.184611	-0.12962	-0.07673	-0.03725	-0.40784
PCYT2	NA	NA	0.317195	0.171612	0.329374	-0.0709	-0.2572	-0.01449
PDAP1	0.068374	-0.16959	0.221886	0.286026	0.257266	-0.0838	-0.22489	-0.42335
PDC	0.166662	-0.00458	-0.23214	-0.15026	-0.07604	0.284716	0.363156	0.251709
PDCD1	0.125617	0.266094	0.421667	0.785246	-0.25569	0.682723	-0.07663	0.237906
PDCD10	0.033028	-0.00437	0.114147	-0.35521	-0.01226	0.0084	-0.35448	-0.34793
PDCD11	0.355323	-0.35193	NA	NA	NA	NA	NA	NA
PDCD1LG2	-0.17249	0.116131	0.157239	0.010646	-0.38182	-0.24593	-0.26927	-0.15562
PDCD2	0.294878	-0.49303	-0.17376	0.008954	0.098666	-0.33323	0.682583	0.440028
PDCD2L	0.540862	0.182834	-0.08585	0.227398	-0.11267	0.186975	NA	NA
PDCD4	-0.06678	-0.47475	-0.13053	0.057639	0.048193	-0.17567	-0.46975	-0.29918
PDCD5	0.578343	-0.19554	-0.24791	0.127107	-0.25122	-0.24422	-0.06852	-0.16147
PDCD6	-0.2281	-0.6174	-0.35463	0.188992	-0.06705	-0.22274	0.33358	-0.04342
PDCD6IP	0.033774	-0.61933	-0.32845	0.016856	0.094392	-0.33217	-0.03654	-0.11412
PDCD7	-0.17257	-0.19183	-0.0034	0.24504	0.266751	0.157727	-0.49462	-0.57114
PDCL	-0.24206	0.161292	-0.33427	-0.29199	-0.10387	-0.4456	0.12071	0.295741
PDCL2	0.246273	0.666391	0.118299	-0.0947	-0.11428	0.204807	-0.1626	-0.37031
PDCL3	0.046416	-0.02019	-0.05547	-0.24821	0.245817	0.068968	-0.26518	-0.33388
PDDC1	0.257224	0.3377	0.070237	-0.01259	0.408034	0.186909	0.548624	0.483844
PDE10A	0.163101	0.168076	0.091329	0.286436	-0.19181	-0.12713	0.094319	0.075175
PDE11A	0.009594	0.550969	0.057461	-0.13331	0.082543	-0.2486	0.258054	0.206263

PDE12	NA	NA	-0.45975	-0.03065	0.230497	0.088454	NA	NA
PDE1A	-0.19976	-0.24237	0.160165	-0.17713	-0.26858	0.07234	-0.21391	-0.19576
PDE1B	-0.26486	0.402594	0.204299	0.319096	-0.31711	0.086563	-0.36531	0.134741
PDE1C	-0.11142	0.418254	-0.12053	-0.15987	0.017691	0.089864	-0.0141	0.436832
PDE2A	-0.33717	0.163607	0.033497	-0.06445	-0.12088	0.097184	-0.31132	0.051459
PDE3A	0.211213	0.618915	-0.17526	-0.2723	-0.19953	-0.51997	-0.13293	-0.0226
PDE3B	0.053642	-0.19969	-0.49056	-0.08957	-0.24979	-0.30062	0.329654	-0.00971
PDE4A	0.010501	0.230252	0.066086	-0.03149	0.08315	0.231415	-0.60991	-0.43436
PDE4B	-0.58888	-0.3504	-0.07194	0.342686	0.028861	0.243064	-0.3407	0.21691
PDE4C	-0.07704	0.084866	0.088432	-0.11134	0.174624	0.426151	-0.34827	-0.02544
PDE4D	-0.43731	-0.58406	-0.06914	-0.44254	-0.46671	-0.12986	-0.16188	0.093646
PDE4DIP	-0.12393	-0.37492	-0.04372	-0.17102	-0.2213	-0.32394	0.122144	0.023549
PDE5A	-0.38107	-0.07453	0.005295	-0.20434	-0.24783	0.142051	-0.33221	-0.37313
PDE6A	0.185322	0.216666	-0.27297	-0.212	-0.18935	-0.24049	-0.10685	-0.18882
PDE6B	0.199327	0.64064	0.200829	0.623503	-0.22952	0.446106	-0.45317	0.30046
PDE6C	0.143017	0.585508	-0.13958	-0.18984	0.17707	-0.0245	-0.53033	-0.2248
PDE6D	0.449326	0.49009	-0.07608	-0.05569	-0.15247	-0.14556	-0.54963	-0.4239
PDE6G	0.253002	0.65677	0.143358	0.266658	-0.27315	0.270882	-0.10771	0.232918
PDE6H	0.062937	0.535925	0.193709	-0.10363	-0.23682	0.18281	-0.2035	-0.50009
PDE7A	-0.32053	0.00994	0.303327	0.462261	0.149181	0.693447	-0.02453	0.35336
PDE7B	-0.2654	-0.19288	0.306614	-0.36071	-0.29788	0.069343	-0.42487	-0.21915
PDE8A	0.128811	-0.32512	-0.39987	0.053142	0.033921	0.055639	-0.25704	-0.15119
PDE8B	0.266542	-0.00616	0.059985	0.069246	-0.13436	0.107376	0.23786	0.06799
PDE9A	0.169312	0.41853	-0.15798	-0.18581	-0.06433	-0.14186	-0.14839	-0.22578
PDF	0.104254	-0.15681	NA	NA	NA	NA	-0.53522	-0.35121
PDGFA	NA	NA	0.058168	-0.29528	0.436103	-0.01439	-0.20806	-0.19718
PDGFB	-0.02621	-0.29198	0.096388	-0.05634	0.059403	0.101857	-0.32545	0.027427
PDGFC	-0.2135	-0.25265	-0.00296	-0.32876	-0.16755	-0.0461	-0.42598	-0.43694
PDGFD	-0.04859	-0.27995	-0.10416	-0.28643	-0.18086	0.210327	0.103718	0.134594
PDGFRA	-0.01746	-0.72889	0.00137	-0.4536	-0.01899	0.249202	-0.38246	-0.33678
PDGFRB	-0.24346	-0.28908	-0.00641	0.21763	-0.06427	0.303603	-0.06287	0.09321
PDGFRL	-0.05441	-0.12281	0.027776	-0.13094	-0.46448	-0.44996	-0.14222	-0.1056
PDHA1	0.271056	-0.06824	-0.09663	0.170797	0.150991	-0.13446	-0.24169	-0.43683
PDHA2	0.198157	0.312256	0.107933	-0.20729	0.106676	0.062077	-0.14327	-0.04353
PDHB	0.046101	-0.26262	-0.33781	-0.23214	-0.31217	-0.22083	-0.36589	-0.09384
PDHX	-0.02213	-0.54027	-0.28612	-0.03733	-0.26089	-0.00794	0.050091	-0.18337
PDIA2	-0.21521	0.187175	-0.26098	0.173207	-0.08125	0.155824	0.193275	0.608313
PDIA3	-0.07351	-0.00292	0.091538	0.295675	-0.15763	0.194497	0.065965	0.261839
PDIA4	-0.21578	0.260165	-0.08609	0.458108	0.015604	-0.20737	0.367466	-0.02488
PDIA5	-0.38412	-0.43409	0.05555	0.236895	-0.19251	-0.02064	0.266093	0.06101
PDIA6	-0.32851	-0.57178	0.102335	0.339688	0.227612	0.156285	0.307755	-0.2248
PDIK1L	-0.42393	-0.55862	-0.24933	-0.07797	0.020131	-0.39808	0.386936	0.093606
PDILT	-0.00478	0.369065	0.014414	0.000358	0.057541	-0.03285	0.184481	0.221532
PDK1	-0.42813	-0.13466	-0.13892	-0.10107	-0.1758	-0.17445	0.273824	0.105422
PDK2	0.289198	0.441153	0.437616	-0.1484	0.088484	-0.10062	-0.26536	-0.24645
PDK3	0.218601	0.224071	0.176664	-0.0412	-0.18612	-0.19802	-0.04554	0.051885
PDK4	0.133927	-0.2647	0.20369	-0.2164	-0.15828	-0.07239	0.051005	-0.26265
PDLIM1	-0.19604	-0.04353	0.243465	-0.10762	0.094348	-0.18688	0.325028	0.264033
PDLIM2	0.033137	0.55008	0.276629	0.005847	-0.0367	0.156859	-0.18654	0.114232
PDLIM3	-0.43754	-0.25906	0.02309	-0.03857	-0.13559	0.128987	-0.20379	-0.30083
PDLIM4	-0.06637	0.038584	0.331211	0.024143	-0.15187	0.255181	-0.26024	-0.07286
PDLIM5	-0.39875	-0.13332	-0.10494	-0.49245	-0.16496	0.093262	0.069423	0.284037

PDLIM7	-0.23207	0.049896	0.209691	-0.05445	-0.08461	0.227362	-0.26548	-0.28836
PDP2	0.398507	0.489142	-0.05594	-0.24542	0.032662	-0.36161	-0.47386	-0.39697
PDPK1	0.412798	-0.35723	0.083146	-0.11222	0.227242	0.142637	0.287713	0.116237
PDPN	-0.21818	-0.03763	-0.05483	0.168352	-0.02983	0.154243	-0.27831	-0.02704
PDPR	-0.0865	0.043682	0.13142	-0.17951	0.162023	0.419883	-0.079	0.093515
PDRG1	0.455724	0.296238	0.22328	-0.08531	0.203534	0.073313	0.443392	0.335824
PDS5A	NA	NA	-0.0896	-0.04605	-0.10622	-0.11033	NA	NA
PDS5B	NA	NA	-0.16329	-0.39648	0.198155	-0.06815	NA	NA
PDSS1	-0.13582	-0.36512	-0.12451	-0.06937	-0.02182	-0.07032	0.422584	0.281338
PDSS2	0.402747	0.019631	-0.23658	-0.17744	0.075899	-0.2569	-0.35635	-0.60967
PDX1	0.293271	0.749975	0.275179	0.037511	0.121517	0.047968	NA	NA
PDXDC1	NA	NA	-0.05422	0.108694	0.328133	-0.0599	NA	NA
PDXK	-0.18512	0.437283	0.180862	0.121125	0.069768	0.043606	0.041938	0.34965
PDXP	-0.08693	0.39403	0.424562	-0.1376	0.37921	-0.37757	0.060887	-0.00102
PDYN	0.114461	0.574852	0.114741	-0.06407	0.106372	-0.11941	-0.59688	-0.17935
PDZD11	0.100339	0.366392	0.108501	-0.36694	-0.06022	-0.52176	0.364454	0.154985
PDZD2	0.057622	-0.25779	-0.28442	-0.08065	-0.1579	0.054137	NA	NA
PDZD3	-0.01101	-0.0231	0.081058	-0.01278	-0.00945	-0.24575	0.135839	0.34026
PDZD4	0.019864	0.013431	-0.00947	-0.12517	0.142299	0.193251	-0.27435	-0.21994
PDZD7	0.108645	0.115738	0.098366	-0.13626	0.18976	0.430604	0.474105	0.448872
PDZD8	-0.11895	0.201419	0.082241	-0.19041	-0.13045	-0.05652	0.184174	0.17292
PDZK1	0.24058	-0.22892	-0.02371	-0.314	-0.34338	0.163522	0.273556	0.423715
PDZK1IP1	-0.22679	0.11151	0.172288	0.207723	0.214692	-0.03859	-0.00237	-0.08841
PDZRN3	-0.30175	-0.43091	-0.03055	-0.04142	0.049562	-0.02708	-0.32073	-0.5487
PDZRN4	0.286566	-0.11994	0.155669	-0.13624	-0.37721	-0.20592	-0.3227	-0.44155
PEA15	-0.51937	-0.138	0.162123	0.245341	0.007324	0.289946	-0.41412	-0.13941
PEAR1	NA	NA	0.07764	0.026448	-0.02258	-0.15381	-0.20599	-0.30845
PEBP1	-0.23395	-0.2876	-0.23889	0.109795	0.407507	-0.22922	-0.56204	-0.30881
PEBP4	0.256077	0.236947	-0.13774	-0.09151	-0.44388	-0.04139	-0.11311	-0.30596
PECAM1	-0.41972	-0.23203	0.106685	0.123077	-0.18254	0.104321	-0.38556	0.102458
PECI	-0.33042	0.264471	-0.17555	-0.19319	-0.40455	-0.24814	-0.31613	-0.31662
PECR	0.487983	0.320706	0.123656	-0.26733	-0.04359	-0.35988	0.388233	0.300955
PEF1	0.11445	0.574801	0.164452	0.072241	0.228923	-0.11727	-0.09361	-0.17466
PEG10	NA	NA	-0.2805	0.258561	-0.26769	0.468161	-0.21313	-0.26628
PEG3	-0.18253	0.025307	-0.33974	-0.01092	-0.00735	-0.04379	-0.28821	-0.33787
PELI1	-0.51939	-0.57317	0.089449	-0.23201	-0.16613	-0.08894	0.433802	0.407199
PELI2	0.42744	-0.57746	-0.27503	-0.44731	0.08775	0.258587	-0.16761	0.245913
PELI3	-0.06494	-0.24783	0.284521	-0.07655	0.155589	-0.08816	-0.18384	-0.05045
PELO	-0.09486	-0.3103	-0.20128	-0.16786	-0.12741	-0.04003	0.043125	0.049573
PELP1	0.149558	0.371465	-0.0964	0.462499	0.267776	-0.01104	0.332958	-0.15559
PEMT	-0.03639	0.327663	0.162816	-0.00843	-0.1213	0.001266	0.38938	0.260216
PENK	0.449912	-0.17252	0.298492	0.058012	0.088446	0.046106	-0.38865	-0.2031
PEPD	0.310468	-0.34653	-0.27361	-0.2005	-0.2124	-0.38768	0.289556	0.516066
PER1	-0.21827	0.174002	0.253807	-0.10747	0.071573	0.094698	-0.14612	-0.07873
PER2	-0.02622	0.194347	0.067045	-0.1876	0.445056	0.157894	0.039416	-0.15514
PER3	-0.148	-0.02738	0.155733	-0.43506	-0.26889	-0.16293	NA	NA
PERLD1	0.068427	0.424492	0.449327	0.100144	0.173477	0.001112	0.319582	0.082313
PERP	0.304529	-0.33869	0.194023	-0.21143	0.278111	-0.42825	0.146791	-0.31437
PES1	0.204805	0.564476	0.291671	0.348327	0.241155	0.09685	-0.48641	-0.4891
PET112L	0.097335	0.24334	-0.0294	-0.17734	-0.32535	-0.48123	-0.3672	-0.4654
PEX1	0.158173	-0.67946	-0.10495	-0.24933	0.242993	-0.14595	0.409132	0.399373
PEX10	0.134532	0.649073	0.00491	0.014224	-0.08065	0.013074	0.25368	-0.16254

PEX11A	0.218277	0.26176	0.183027	-0.0275	0.084924	-0.36744	-0.21515	-0.13679
PEX11B	-0.0259	0.048388	-0.20776	0.055013	-0.19506	-0.4818	0.706585	0.451578
PEX11G	0.202466	0.587891	0.036948	-0.3853	0.003097	-0.1467	-0.09577	-0.38529
PEX12	0.13128	-0.02613	-0.04254	-0.02568	-0.30627	-0.33873	0.03556	-0.17466
PEX13	-0.12365	-0.29209	-0.3696	-0.39446	-0.18267	-0.59029	-0.58952	-0.3186
PEX14	0.247942	0.437189	-0.13409	-0.02608	-0.24218	-0.36137	0.18303	0.3027
PEX16	0.407935	0.495732	0.210713	0.021045	0.327686	-0.06335	0.114991	0.08592
PEX19	-0.10674	-0.02631	-0.32235	-0.14733	-0.21328	-0.30441	0.226467	0.059361
PEX26	0.216221	0.252897	-0.07084	-0.22012	-0.0505	-0.09677	0.180717	-0.19505
PEX3	0.617137	-0.03756	-0.24631	-0.11621	0.151802	-0.0016	0.186647	0.080725
PEX5	0.483992	-0.61531	0.02804	0.182485	0.228049	0.132529	0.068978	-0.24269
PEX5L	0.251826	0.590932	-0.22588	-0.0262	-0.10926	0.582673	0.050832	0.204043
PEX6	0.257744	0.430058	-0.03753	0.456997	0.161882	-0.14257	0.358949	0.294834
PEX7	0.399477	-0.18608	0.020763	-0.23593	0.210419	-0.17115	0.325473	-0.02429
PF4	0.166472	0.288174	-0.13836	0.095831	-0.31565	-0.31059	0.250013	-0.05192
PF4V1	0.353274	0.653526	-0.18015	-0.23059	-0.22243	-0.20383	-0.12533	0.09826
PFAS	0.137243	0.286052	-0.36624	0.4791	0.098873	0.20488	-0.09656	-0.37806
PFDN1	0.522938	-0.28306	0.133684	-0.53315	-0.30844	-0.15876	0.425968	0.300925
PFDN2	0.251456	0.609248	-0.06716	-0.19517	0.005467	0.172476	-0.29628	0.135511
PFDN4	0.382305	-0.44301	0.12842	-0.32119	0.066923	-0.22674	0.10722	-0.16061
PFDN5	0.168928	0.18995	0.242494	0.015067	-0.16854	0.141201	-0.6945	-0.49276
PFDN6	0.234069	0.055072	-0.13889	0.374809	0.054789	-0.05552	-0.12383	-0.10986
PFKFB1	0.312088	-0.06834	0.107623	-0.10343	0.058663	-0.43241	0.135598	0.385738
PFKFB2	-0.18929	-0.08257	-0.01613	-0.10675	0.029349	-0.28574	0.316573	0.243759
PFKFB3	-0.58425	-0.07176	0.12495	0.139724	0.076723	-0.0818	-0.12956	0.30832
PFKFB4	-0.45146	0.050122	-0.01352	-0.16796	-0.09266	-0.26792	0.08631	0.534397
PFKL	0.233486	0.560452	0.237096	0.063227	-0.00309	0.002956	0.587833	0.473866
PFKM	0.50204	0.026981	-0.00499	-0.20881	0.02499	0.190649	-0.28622	-0.41792
PFKP	-0.3705	0.265664	-0.01586	0.151917	0.239625	0.22946	0.010508	0.344471
PFN1	0.115406	-0.64885	0.166749	0.244378	-0.14858	-0.1195	-0.32661	-0.11603
PFN2	-0.02666	-0.36818	-0.17362	0.105581	-0.01133	0.057958	0.228275	0.051395
PFN3	0.225783	0.62603	-0.00731	0.050996	0.198969	0.083152	-0.09935	-0.20578
PFN4	0.388565	0.319816	0.411592	0.064408	-0.12169	0.104504	-0.01562	0.099026
PFTK1	-0.30132	-0.39815	-0.01585	0.179777	-0.2712	0.46843	-0.13844	-0.10325
PGA3	NA	NA	0.325511	-0.14593	-0.1305	0.014144	0.150277	-0.05806
PGA5	0.156872	-0.27115	NA	NA	NA	NA	-0.38051	0.086143
PGAM1	NA	NA	-0.06835	0.021361	-0.07575	-0.23659	0.252829	0.422329
PGAM2	-0.05418	-0.13392	-0.10422	-0.0334	-0.1844	-0.11211	0.099011	-0.00402
PGAM4	NA	NA	NA	NA	NA	NA	-0.67747	-0.5568
PGAM5	0.294613	0.684424	0.107528	0.191636	0.171234	-0.04961	-0.0662	0.180936
PGAP1	-0.10261	-0.43592	-0.30889	-0.50091	-0.26016	0.150807	0.427469	0.470312
PGBD1	0.246529	-0.20422	-0.16141	-0.09288	-0.25714	-0.22435	0.032904	-0.03731
PGBD2	-0.19182	-0.05434	-0.44863	0.267152	-0.30999	-0.24161	0.266512	0.20779
PGBD3	-0.14607	-0.47602	-0.2737	-0.30194	-0.14748	-0.34423	-0.36178	0.017366
PGBD4	-0.20013	-0.2542	0.302633	-0.18315	-0.06782	0.230973	0.080664	0.407673
PGBD5	0.364824	0.69294	-0.01705	0.268644	0.17086	0.155944	0.042911	0.170301
PGC	0.25431	0.747432	-0.37606	-0.22622	0.165022	-0.44166	0.349798	0.103955
PGCP	-0.20268	-0.54402	0.245036	-0.05609	-0.17333	0.050013	-0.14703	-0.16441
PGD	-0.38583	-0.22027	0.078702	0.413059	0.248249	-1.1E-05	0.205755	0.365424
PGDS	0.038533	-0.15303	0.078294	-0.03224	-0.19537	0.068528	0.057405	0.082697
PGF	-0.31222	0.01487	0.245042	0.117991	0.250991	0.216822	-0.33862	-0.30826
PGGT1B	-0.18354	0.547151	-0.18693	-0.24886	0.083572	-0.32205	0.565103	0.496782

PGK1	-0.16368	-0.32352	-0.10711	-0.07212	0.03824	-0.00784	-0.07325	0.117792
PGK2	-0.02753	-0.22942	0.010103	-0.04191	-0.14221	0.083709	0.298419	0.597132
PGLS	0.608659	0.256549	0.263291	-0.12818	0.204682	0.215049	0.196637	0.04974
PGLYRP1	-0.12475	0.636653	0.229132	-0.21613	-0.00142	-0.12654	-0.31247	0.117397
PGLYRP2	-0.19373	0.139592	-0.14934	-0.0261	-0.26331	0.113475	0.454147	0.513911
PGLYRP3	-0.02075	0.033103	-0.23857	-0.27077	0.075193	-0.29902	0.185936	0.564412
PGLYRP4	0.170223	0.758383	-0.27696	0.109016	-0.14265	0.409151	-0.09308	-0.36717
PGM1	-0.39053	-0.2944	0.15287	-0.01226	0.054085	0.151967	-0.00131	0.221575
PGM2	0.082908	-0.70626	-0.28408	-0.12516	-0.1167	-0.32653	0.326895	0.115405
PGM2L1	-0.14206	-0.5045	0.261619	0.020642	-0.01635	0.084985	-0.15899	-0.03965
PGM3	-0.07045	-0.03985	-0.11811	-0.06939	0.100212	-0.27351	0.221319	0.273428
PGM5	0.143439	-0.15948	0.23797	-0.11607	0.09247	0.205534	-0.09094	-0.28871
PGP	NA	NA	-0.12369	0.034098	0.321166	-0.12985	NA	NA
PGPEP1	0.167237	0.092273	0.237542	-0.15943	0.121545	-0.30683	-0.01499	0.057264
PGR	0.26553	-0.02659	0.117025	-0.15024	-0.2779	0.324344	-0.3273	-0.09627
PGRMC1	0.467434	-0.67485	-0.36214	0.058452	-0.24021	-0.65824	0.175651	0.346189
PGRMC2	-0.2822	0.270053	-0.14438	0.113335	0.081096	-0.24497	-0.00836	0.481879
PGS1	NA	NA	0.418558	0.155211	0.185201	0.176303	0.033976	0.511451
PHACTR1	-0.5887	0.258299	0.123581	0.539697	-0.09846	0.492734	-0.43866	-0.21521
PHACTR2	0.412383	-0.48734	-0.25918	-0.31151	0.167942	-0.17878	0.503577	0.274077
PHACTR3	0.505536	-0.24019	-0.04379	0.079393	-0.17387	-0.22572	0.10742	-0.19392
PHACTR4	-0.3738	-0.64451	-0.00625	-0.28952	0.176638	0.154283	0.238023	0.18338
PHAX	NA	NA	-0.05823	-0.15995	-0.16564	-0.29092	NA	NA
PHB	0.416262	0.482183	0.263478	0.273694	0.044236	0.145115	-0.0808	-0.38518
PHB2	0.589227	-0.03769	-0.17688	0.15074	-0.14155	-0.14074	-0.32303	-0.4058
PHC1	0.505881	-0.04085	0.085545	0.28645	0.291038	0.304049	-0.11397	0.092465
PHC2	-0.13013	0.355948	0.394377	-0.14015	0.340285	0.424807	0.18233	0.053014
PHC3	-0.144	-0.59477	-0.16388	-0.25069	-0.02993	0.14598	-0.04435	0.206564
PHEX	0.097215	-0.11011	-0.45609	0.226421	-0.14113	0.130481	-0.20035	0.359858
PHF1	0.121994	0.181681	0.349126	0.102046	0.030932	0.063635	-0.25641	0.05613
PHF10	-0.2295	-0.62789	-0.34738	0.374974	0.11582	-0.20778	0.474504	0.133106
PHF11	0.095183	-0.20861	0.059943	0.022554	-0.02416	0.155262	-0.14886	0.327781
PHF12	0.408476	-0.1382	-0.17771	-0.39615	0.202168	0.100703	0.193438	0.190231
PHF13	-0.12204	-0.05557	0.2498	-0.1807	0.436787	0.293084	-0.08702	-0.18408
PHF14	0.263639	-0.42753	-0.3303	-0.13545	0.053515	0.192509	-0.17756	-0.31225
PHF15	0.221197	0.464474	0.288045	0.530289	0.196876	0.517209	0.208228	0.299365
PHF16	0.45519	-0.32993	-0.16156	-0.05435	0.284338	0.115656	0.374866	0.063897
PHF17	0.040039	0.536441	0.163502	0.133442	0.323121	0.495215	0.026183	-0.01039
PHF19	0.184745	0.057073	0.157715	0.284892	0.176688	0.039227	0.060098	-0.23498
PHF2	-0.34732	-0.30319	-0.07953	-0.12063	0.187132	0.177297	0.150842	0.238609
PHF20	0.185228	-0.31033	0.051258	-0.04117	0.443093	0.548151	0.068869	-0.07943
PHF20L1	-0.05687	-0.48583	0.168535	-0.20386	-0.02225	0.260578	0.07237	0.186606
PHF21A	-0.14127	-0.24364	-0.11176	0.122281	-0.19859	-0.20203	-0.28889	-0.22787
PHF21B	0.343574	0.231559	0.072915	0.277351	0.075905	0.304987	0.244375	0.389486
PHF23	-0.2707	0.141914	0.016816	-0.10762	-0.06787	0.064871	-0.66029	-0.66902
PHF3	0.265644	-0.54092	-0.41338	-0.04863	0.028811	0.054884	-0.4922	-0.32892
PHF5A	0.022119	-0.56469	-0.04784	-0.11136	-0.20184	-0.24214	0.025225	-0.30158
PHF6	-0.16212	0.435962	-0.27112	0.003114	-0.00881	-0.16233	0.353009	0.501473
PHF7	-0.27737	0.137287	0.007201	-0.21935	-0.02884	-0.50914	0.104621	0.512187
PHF8	NA	NA	-0.10735	0.242825	0.415009	0.35443	-0.20443	-0.03345
PHGDH	0.054676	0.192126	0.056183	0.190263	0.249654	-0.04398	-0.08285	-0.10073
PHIP	-0.02237	-0.45053	-0.21144	-0.06551	0.084043	-0.0071	-0.44008	-0.6236

PHKA1	0.122839	-0.23339	-0.09025	-0.06316	0.142837	-0.04809	0.059877	0.212657
PHKA2	0.201284	0.523947	0.104964	0.262929	0.418259	0.227484	0.311472	0.368618
PHKB	-0.3015	-0.5067	0.001302	-0.31567	0.093436	-0.12242	0.204412	0.127243
PHKG1	0.095166	-0.21392	-0.13435	-0.10724	0.04572	0.064459	-0.1345	0.010342
PHKG2	-0.12541	0.319745	0.163851	0.045333	0.264005	-0.09538	0.292338	0.286865
PHLDA1	-0.04368	-0.13063	-0.22055	0.041939	-0.06357	0.058459	0.248815	0.228938
PHLDA2	-0.14392	0.591502	-0.16566	-0.08723	-0.05347	0.108062	-0.09006	-0.15089
PHLDA3	-0.03948	0.077203	0.396991	0.023891	-0.20338	-0.07297	-0.26483	-0.3002
PHLDB1	-0.28876	0.210747	0.250596	-0.11392	0.014339	-0.06074	-0.32337	-0.28604
PHLDB2	-0.58004	-0.45171	0.136733	-0.16121	-0.07292	0.135135	-0.31983	-0.10927
PHLDB3	0.0841	0.171984	-0.16228	0.043745	0.089087	-0.15179	-0.18102	-0.40133
PHLPP	0.166143	0.297733	0.043999	0.216634	0.120312	0.013635	0.500234	0.057612
PHLPPL	0.075687	-0.04112	-0.23626	-0.43533	0.164274	-0.15713	0.305764	0.292466
PHOSPHO1	0.214518	0.052467	0.145208	-0.06401	0.109411	-0.11807	0.622729	0.020151
PHOSPHO2	0.390788	-0.44234	0.148977	-0.26727	0.012799	-0.20935	-0.35229	-0.31883
PHOX2A	-0.06343	0.709403	0.076069	0.00845	-0.13868	0.231819	0.337509	0.334457
PHOX2B	-0.15204	-0.46692	0.062283	-0.01464	-0.41074	0.079843	-0.56611	-0.54288
PHPT1	-0.25789	-0.23385	0.310176	0.121929	0.099691	-0.41451	-0.34798	-0.24414
PHRF1	NA	NA	-0.08446	0.269341	0.35152	0.431327	NA	NA
PHTF1	-0.0065	-0.63742	-0.09577	-0.07	-0.01712	-0.11284	0.033111	0.187811
PHTF2	-0.16886	0.135936	-0.27242	0.006804	-0.40532	-0.06713	0.181827	0.115344
PHYH	-0.10015	0.336694	-0.20554	-0.21388	0.024913	-0.47006	0.234959	0.243859
PHYHD1	-0.11598	0.356549	0.146408	-0.01842	-0.24187	-0.07247	-0.40989	0.049718
PHYHIP	0.058791	0.511429	0.123478	-0.35628	0.179345	0.104602	-0.37041	-0.27797
PHYHIPL	0.336693	0.461434	0.061486	-0.07591	0.099289	0.143987	0.240675	0.399872
PI15	0.00619	-0.114	0.135275	-0.13458	-0.207	-0.19644	-0.32561	-0.49804
PI16	-0.0998	-0.03065	0.23803	-0.06295	-0.08355	-0.14538	-0.18456	0.121023
PI3	-0.3095	0.049663	-0.13928	0.207125	-0.30043	-0.04422	-0.14748	-0.20178
PI4K2A	NA	NA	0.287766	-0.12837	0.210824	0.012441	NA	NA
PI4K2B	-0.28349	-0.38555	-0.04322	0.032026	0.107094	-0.3749	0.054302	0.155355
PI4KA	NA	NA	0.21809	-0.11203	0.193339	0.166883	NA	NA
PI4KAP2	NA	NA	0.132164	-0.26637	0.211151	-0.01048	NA	NA
PI4KB	NA	NA	0.307316	-0.02461	0.048527	0.104526	NA	NA
PIAS1	-0.11615	-0.41955	-0.02487	-0.24357	-0.15023	-0.02435	0.055451	-0.24763
PIAS2	-0.04108	-0.0852	-0.34673	-0.20175	-0.19579	0.230455	-0.3539	0.229505
PIAS3	-0.53049	0.031591	-0.11723	0.107824	-0.02149	0.050526	0.275762	0.10192
PIAS4	-0.00142	0.50917	0.17647	0.207193	0.144345	0.048212	-0.4079	-0.50826
PIBF1	NA	NA	-0.11927	0.02318	0.103634	-0.30674	NA	NA
PICALM	-0.43347	-0.12841	-0.10445	-0.01831	0.111749	-0.00398	-0.34591	0.008499
PICK1	0.168915	0.297821	0.291562	0.216039	0.316378	0.027574	-0.0188	-0.13003
PID1	0.044211	-0.30914	0.075405	-0.05481	-0.21048	-0.11017	NA	NA
PIF1	NA	NA	-0.24725	0.202229	0.08707	0.077615	0.162969	0.255643
PIGA	-0.17466	0.085596	-0.3707	-0.0691	-0.04665	-0.24056	-0.18359	0.188067
PIGB	0.155908	-0.03709	-0.02879	-0.33526	-0.26034	-0.5031	0.318637	0.601624
PIGC	-0.1972	-0.68565	-0.2432	-0.21295	-0.36117	0.038588	0.617169	0.605853
PIGF	0.210798	-0.10863	0.088084	-0.20084	0.007728	-0.40577	0.214287	0.076126
PIGG	0.352677	-0.01633	0.202222	-0.00432	0.11281	0.226141	0.17952	0.140564
PIGH	-0.06581	-0.32456	-0.30315	-0.21274	-0.41275	-0.16504	-0.02253	0.21484
PIGK	-0.31696	-0.66663	-0.07217	-0.48305	0.016337	-0.23377	0.247326	-0.30057
PIGL	0.324557	-0.62911	-0.30572	-0.13738	-0.28227	-0.18274	0.110066	0.446854
PIGM	0.120728	0.072237	-0.10591	0.171024	-0.02716	0.0067	-0.06716	-0.37221
PIGN	-0.00508	0.158487	0.051341	-0.15001	-0.02622	-0.36637	0.26347	0.241142

PIGO	0.17035	0.031585	0.012257	0.228882	0.121711	-0.01968	0.337915	0.28135
PIGP	0.314184	-0.05133	-0.15355	-0.25276	-0.22546	-0.23602	NA	NA
PIGQ	0.065853	0.625068	0.214394	0.13005	0.460544	-0.07153	-0.37842	-0.39422
PIGR	-0.24501	0.051812	-0.06434	0.397216	-0.31826	0.233278	-0.05082	-0.00176
PIGS	-0.10182	-0.09675	0.251714	0.039095	0.273173	-0.11538	0.251156	0.207896
PIGT	0.36914	-0.33236	0.050346	0.364424	0.551338	-0.54808	0.6086	0.025478
PIGU	0.423624	0.644536	0.207737	0.101662	0.329587	-0.01776	NA	NA
PIGV	0.143648	0.416866	0.04642	-0.04133	0.092258	-0.61874	-0.45641	-0.37782
PIGW	-0.01279	-0.34497	-0.18457	0.164539	-0.10134	0.106736	-0.24837	-0.29559
PIGX	-0.14723	0.422837	0.091099	0.062143	0.029144	-0.18893	-0.60839	-0.61744
PIGY	-0.129	-0.69373	-0.15144	-0.19162	-0.05428	-0.08487	0.124577	0.009151
PIGZ	-0.24135	0.091982	-0.1836	-0.16129	0.032562	-0.1665	0.270116	0.143164
PIH1D1	0.116973	-0.36956	-0.03649	0.242527	0.090477	-0.03494	NA	NA
PIH1D2	0.110891	0.248192	-0.11905	-0.32759	-0.00632	-0.54757	NA	NA
PIK3AP1	-0.38999	-0.284	-0.16871	0.442494	-0.2696	0.368794	-0.64734	-0.31956
PIK3C2A	-0.12059	-0.49082	-0.12078	-0.22285	-0.03568	-0.21787	0.293234	0.210003
PIK3C2B	0.146207	0.711116	-0.27286	0.24809	0.028047	-0.08769	0.490875	0.202321
PIK3C2G	-0.17014	0.264864	-0.39433	-0.31871	0.138699	-0.34456	-0.02993	0.293873
PIK3C3	0.353555	-0.49445	-0.00454	-0.40191	-0.18769	0.099506	-0.60304	-0.59552
PIK3CA	0.107866	-0.60365	-0.2617	0.025028	-0.23113	0.03767	-0.58591	-0.31139
PIK3CB	-0.30159	-0.44723	-0.26939	0.028687	-0.05045	-0.42376	-0.08102	-0.09674
PIK3CD	-0.60436	0.191444	0.20976	0.82038	0.120652	0.786052	-0.4627	0.467587
PIK3CG	-0.51692	-0.02293	-0.01257	0.219636	-0.31699	0.379583	-0.56065	-0.01567
PIK3IP1	NA	NA	0.414288	0.150253	0.052979	0.180856	NA	NA
PIK3R1	0.282284	0.135589	0.097319	-0.15145	-0.08738	0.321892	-0.0138	-0.44146
PIK3R2	0.022879	0.365182	0.305124	0.018528	0.437146	0.003126	-0.20086	-0.10369
PIK3R3	0.00852	0.668265	-0.21289	0.017079	-0.17481	-0.3216	-0.55252	-0.24862
PIK3R4	-0.13205	-0.37299	-0.40022	-0.31871	-0.05564	-0.17295	0.12249	0.112936
PIK3R5	0.032212	0.605372	0.4371	0.251921	-0.03863	0.30483	-0.34911	0.173976
PIK3R6	NA	NA	0.360482	0.48996	-0.15936	0.10928	NA	NA
PILRA	-0.35928	0.24267	0.105803	0.208959	0.505149	0.151016	-0.5121	-0.337
PILRB	0.055414	-0.08706	-0.01771	0.247358	0.495015	0.263196	0.119332	0.134766
PIM1	-0.00217	0.171933	-0.1004	0.111705	0.001716	0.057609	-0.07434	0.367313
PIM2	-0.25552	0.17473	0.204885	0.214081	-0.06555	0.195224	-0.26623	0.0592
PIM3	-0.42377	0.013257	-0.05696	0.255704	0.205764	0.152554	0.083786	0.079771
PIN1	0.113999	0.277707	0.174178	0.101664	0.101056	-0.13985	0.00887	-0.23735
PIN4	0.195574	0.131274	-0.30715	0.043541	-0.1466	-0.41759	NA	NA
PINK1	-0.54778	-0.29034	0.2407	-0.25558	0.183183	-0.11263	-0.42432	-0.56715
PINX1	0.293179	0.256225	0.105381	0.04774	-0.18113	0.124094	0.255427	-0.00352
PION	NA	NA	0.130688	0.097673	-0.1472	0.296762	NA	NA
PIP	0.360081	0.358011	-0.01109	0.380011	-0.31081	0.418142	0.079342	0.253747
PIP3-E	0.125335	0.621609	-0.13498	0.544385	-0.15496	0.001597	-0.48972	-0.18746
PIP4K2A	NA	NA	0.116065	0.52698	-0.11111	0.417987	NA	NA
PIP4K2B	NA	NA	0.042721	0.142335	0.051076	-0.0655	NA	NA
PIP4K2C	NA	NA	0.286082	-0.03028	0.211684	-0.21533	NA	NA
PIP5K1A	0.264063	0.700729	-0.00062	0.140893	0.149705	0.2373	-0.15415	-0.09038
PIP5K1B	0.033595	-1E-05	-0.06253	-0.23891	0.152365	-0.41966	-0.12655	-0.09769
PIP5K1C	-0.12404	0.117575	-0.12933	-0.04159	-0.09528	0.278781	-0.07211	-0.01851
PIP5K3	0.431263	0.671553	-0.07427	0.000634	-0.07915	0.14546	0.337425	0.290875
PIP5KL1	-0.09587	0.775354	0.102378	0.048003	-0.02602	0.070049	0.525175	0.465097
PIPOX	0.291301	0.227484	0.180845	-0.34162	0.170781	-0.19829	0.029673	-0.25513
PIR	-0.12872	-0.53258	0.273193	-0.15832	-0.00296	-0.22716	-0.22269	-0.18907

PISD	-0.47114	0.41485	0.248649	0.125923	0.404923	0.186219	0.318821	0.443215
PITPNA	0.0419	-0.29211	-0.03845	0.095581	-0.16741	0.149214	-0.31966	0.000202
PITPNB	-0.01781	-0.1875	-0.12452	0.097451	0.097295	0.295003	0.483128	0.200181
PITPNC1	0.302736	-0.00994	0.309223	0.334707	0.161284	0.243084	-0.064	0.169148
PITPNM1	0.229131	0.288288	0.340098	0.129992	0.275681	0.144653	0.326229	0.379369
PITPNM2	0.313628	0.366786	-0.15869	-0.18849	0.322251	-0.08821	-0.35513	0.156648
PITPNM3	0.024653	-0.18409	0.023419	0.044683	0.091891	-0.10466	0.212013	0.34319
PITRM1	-0.45367	-0.24949	0.330103	0.265934	0.077383	0.022957	0.338525	-0.05793
PITX1	0.191534	0.600347	0.294357	0.185531	0.069719	0.070018	0.394173	-0.09618
PITX2	0.136384	0.482205	0.129433	-0.00877	0.267083	-0.36985	0.298642	0.061406
PITX3	0.261833	0.000235	0.112246	-0.16983	0.140661	-0.24377	-0.15554	0.171927
PIWIL1	-0.02377	0.673567	0.085133	0.081784	0.015402	-0.19348	0.002883	-0.13832
PIWIL2	0.152349	-0.25076	0.335975	-0.40534	0.153422	-0.15455	0.266495	0.207869
PIWIL3	0.121381	-0.02265	-0.07283	0.182981	0.227943	-0.07987	-0.03989	-0.22163
PIWIL4	-0.32303	-0.342	-0.28793	-0.05898	0.030998	-0.11276	-0.05044	0.046487
PJA1	0.304033	-0.33372	-0.23886	0.151148	0.106829	0.074533	0.571567	0.184931
PJA2	-0.14546	0.123154	0.258103	-0.27686	-0.12208	0.029	-0.62608	-0.54509
PKD1	-0.03587	-0.01452	-0.024	-0.12294	0.240791	0.30315	-0.04502	0.117151
PKD1L1	0.379687	0.016085	-0.26421	-0.29653	0.231645	0.002901	0.172741	0.524341
PKD1L2	-0.13758	-0.12325	-0.22853	-0.09992	0.080932	0.082407	0.196609	0.139069
PKD1L3	0.17389	0.620596	0.14685	0.019918	0.058013	0.020298	-0.04423	-0.42668
PKD2	-0.31331	-0.58666	-0.00456	-0.17273	-0.02105	0.483678	-0.27416	-0.17486
PKD2L1	0.034386	0.204951	-0.04057	0.034412	-0.30315	-0.32046	-0.03254	-0.00064
PKD2L2	0.318569	0.313561	0.062366	-0.16644	-0.24854	0.142237	0.462637	0.490964
PKDREJ	-0.13813	-0.38687	-0.24778	-0.01835	-0.16917	-0.08693	-0.17533	0.211857
PKHD1	-0.21444	0.381208	-0.13507	0.096869	0.308753	0.235577	-0.09928	0.198105
PKHD1L1	-0.39976	-0.13398	-0.07582	0.246598	-0.09208	-0.35711	0.106972	-0.03081
PKIA	-0.27386	-0.4133	0.160501	0.373061	-0.30134	0.025718	-0.18826	-0.36662
PKIB	-0.03004	0.123779	-0.13	-0.20778	-0.25492	-0.3458	-0.05605	-0.17647
PKIG	0.438406	0.018795	0.328262	0.088979	-0.11695	-0.04809	-0.44989	-0.45936
PKLR	0.193245	0.11895	0.346589	-0.3495	0.116996	-0.22813	0.212536	0.348056
PKM2	-0.0641	0.012286	0.106195	0.418875	0.055898	0.164922	0.253116	0.055046
PKMYT1	-0.11997	-0.17645	-0.16272	0.338913	0.401311	-0.01614	0.542121	0.215204
PKN1	0.184118	-0.0069	0.385754	0.244662	0.315436	0.109697	-0.00299	-0.13643
PKN2	-0.15576	-0.29668	0.085153	-0.27346	0.095996	0.278506	-0.44256	-0.33394
PKN3	-0.32017	-0.26698	0.275076	0.066472	0.338019	0.194602	0.119804	0.204881
PKNOX1	-0.04608	0.556628	-0.48379	-0.07572	0.099239	0.05615	-0.25571	0.071248
PKNOX2	-0.48667	-0.16651	-0.11302	0.120133	-0.17476	0.326728	-0.09908	-0.02358
PKP1	0.270594	0.477154	-0.08779	-0.03898	0.286049	-0.05512	-0.1396	0.459722
PKP2	0.276023	-0.10851	-0.51541	-0.01463	-0.31563	0.180321	0.470971	0.288214
PKP3	-0.02925	-0.07894	-0.07185	0.162452	0.209217	-0.00752	0.370292	0.135386
PKP4	0.298238	-0.02741	0.016165	0.175802	0.372072	0.083449	0.418221	-0.0969
PLA1A	0.076063	-0.39601	0.590644	0.219642	-0.13496	-0.18993	-0.19593	0.095519
PLA2G10	-0.1837	0.106674	-0.16175	-0.22347	-0.11317	-0.46311	0.08072	-0.19622
PLA2G12A	-0.18122	-0.45268	-0.18173	-0.17052	0.050175	-0.38338	-0.21132	-0.33635
PLA2G12B	0.423619	0.144807	0.293047	-0.2364	0.200435	-0.41855	0.240944	0.311528
PLA2G15	NA	NA	0.432536	-0.0596	0.273047	0.02117	NA	NA
PLA2G16	NA	NA	0.284745	-0.30807	0.066235	-0.00011	NA	NA
PLA2G1B	-0.00748	0.387159	0.241153	-0.22915	0.061381	-0.37278	0.293434	0.314326
PLA2G2A	-0.29978	0.138581	-0.09178	0.204256	-0.22283	-0.10034	-0.12555	0.217089
PLA2G2D	0.303633	0.385393	0.166868	0.473506	-0.18079	0.336209	-0.57814	-0.5421
PLA2G2E	0.173886	0.454841	-0.04197	-0.09018	0.216219	0.259213	0.426899	-0.06093

PLA2G2F	-0.31325	-0.30735	-0.21845	-0.14428	-0.03845	-0.02578	0.037565	-0.13491
PLA2G3	0.059132	0.039381	-0.11353	0.081486	-0.24948	-0.15418	-0.13213	-0.0209
PLA2G4A	-0.03808	-0.58469	0.499608	-0.53186	-0.26117	-0.18684	0.137419	0.118979
PLA2G4B	-0.2642	0.388779	NA	NA	NA	NA	-0.35067	0.21173
PLA2G4C	0.034276	0.147939	-0.01009	0.119286	-0.07272	0.186367	-0.57329	0.052832
PLA2G4D	0.56702	0.339317	0.245577	-0.17913	0.104037	-0.11016	-0.15629	-0.10577
PLA2G4E	0.034526	0.505204	NA	NA	NA	NA	-0.10593	-0.04827
PLA2G4F	-0.03254	0.359147	0.326662	-0.07581	0.123702	-0.39953	0.282792	0.006149
PLA2G5	-0.30309	-0.08126	0.243714	0.004474	-0.03417	0.055662	-0.28369	-0.17463
PLA2G6	-0.05954	0.505317	0.2109	0.123958	0.242955	0.128991	0.309304	0.024195
PLA2G7	-0.22657	-0.02701	-0.00959	0.18317	-0.42511	0.218644	-0.28956	0.180786
PLA2R1	0.036458	-0.19341	0.542173	-0.32677	0.088627	-0.09661	0.122792	0.275092
PLAA	0.126733	0.436394	-0.35628	-0.21797	0.198612	0.109365	-0.33528	-0.29485
PLAC1	0.231832	0.705443	-0.12164	-0.34434	-0.19065	-0.39389	0.2642	0.357853
PLAC1L	0.030309	-0.08373	-0.04011	-0.07869	0.382046	-0.41313	NA	NA
PLAC2	-0.18702	-0.06867	-0.25414	-0.05696	-0.06035	-0.46057	-0.46898	-0.56519
PLAC4	0.355095	0.456805	-0.01162	0.073273	-0.27485	0.053711	0.07636	-0.09159
PLAC8	-0.37187	-0.0306	-0.03191	-0.09808	-0.2375	-0.27512	-0.11156	-0.14331
PLAC8L1	0.011742	-0.52721	NA	NA	NA	NA	0.287655	0.539125
PLAC9	-0.18536	-0.31684	NA	NA	NA	NA	-0.38342	-0.11742
PLAG1	0.505138	0.348075	0.135277	-0.02454	-0.045	0.499957	0.318122	0.161886
PLAGL1	0.042821	-0.18495	-0.07308	-0.00391	0.0299	0.477621	0.254069	0.125276
PLAGL2	0.452654	0.103062	0.027976	0.062337	0.14981	0.05827	0.480632	0.493577
PLAT	0.088686	-0.20389	-0.07934	-0.09945	0.033788	0.308329	0.123608	0.201059
PLAU	-0.40487	-0.18709	0.05638	0.089832	0.060396	0.285628	-0.31338	0.363153
PLAUR	-0.24736	0.036937	0.063973	0.075201	0.041876	0.062522	-0.14038	0.091312
PLB1	0.073322	0.174213	0.290719	-0.40571	0.129184	-0.48498	-0.4264	0.5612
PLCB1	0.256998	0.202344	-0.00927	-0.20171	-0.17642	0.193543	-0.03419	-0.41283
PLCB2	-0.17725	0.27954	-0.02255	0.55022	-0.22473	0.332314	-0.34717	0.355552
PLCB3	-0.18202	-0.08747	0.112774	0.213272	0.324851	-0.11666	0.116845	0.020371
PLCB4	0.331493	0.09996	-0.08291	-0.04936	-0.04057	-0.02001	0.107031	-0.34978
PLCD1	0.026951	0.21404	0.046345	-0.3733	-0.01742	-0.12205	0.144832	-0.10874
PLCD3	-0.09715	0.247493	0.283578	-0.23774	0.048179	-0.13411	-0.17928	-0.27341
PLCD4	-0.14061	0.164259	0.077634	-0.05435	0.111918	0.119568	-0.44754	-0.23105
PLCE1	-0.06839	0.087589	-0.25687	-0.04304	-0.18677	-0.54061	-0.32054	-0.47662
PLCG1	0.279198	0.255296	0.243256	0.2269	0.494628	0.335755	0.087885	-0.07139
PLCG2	-0.68224	0.048499	0.453795	0.798159	-0.18619	0.503091	-0.50089	0.198216
PLCH1	-0.26339	-0.11502	-0.38259	-0.05765	-0.25762	-0.43414	0.347374	0.024133
PLCH2	NA	NA	0.040834	0.309837	0.033344	0.299583	0.042355	0.126567
PLCL1	0.121834	-0.44852	-0.14576	-0.11331	-0.07404	0.08718	-0.34052	-0.25796
PLCL2	-0.28578	0.000263	-0.02151	0.074166	-0.47526	-0.41538	-0.54682	-0.08653
PLCXD1	-0.1154	0.516244	0.143203	-0.1742	0.325854	0.141814	0.193618	0.435295
PLCXD2	0.432931	-0.01075	0.088633	-0.22411	0.020327	-0.21358	-0.31365	-0.36367
PLCXD3	0.076988	0.62704	-0.17013	-0.12514	-0.17493	-0.12951	-0.06774	0.203228
PLCZ1	0.020176	-0.32004	0.154503	0.099443	0.293903	0.030874	-0.13668	-0.43886
PLD1	0.020473	0.272606	-0.58714	-0.30618	-0.09342	-0.41181	-0.26792	-0.17592
PLD2	-0.24172	0.465632	0.225109	-0.04935	-0.13002	-0.11482	-0.04183	0.255193
PLD3	-0.19091	0.425089	0.232894	-0.16915	-0.10212	0.161819	-0.38208	-0.48216
PLD4	-0.35685	0.516443	0.301066	0.586662	0.007636	0.462175	-0.02052	0.120576
PLD5	0.038304	-0.08893	0.057676	-0.15746	-0.21981	-0.01948	0.124104	-0.40695
PLD6	NA	NA	-0.19101	-0.02264	0.084581	-0.08084	NA	NA
PLDN	-0.1028	-0.31737	0.262711	0.119869	-0.15416	0.223459	-0.55465	-0.50943

PLEC1	0.273287	0.719712	0.30874	-0.2057	0.031196	0.196104	0.236943	0.095201
PLEK	-0.48654	-0.12122	0.036595	0.477814	0.017186	0.484147	-0.4139	0.271509
PLEK2	-0.04198	0.230345	-0.38351	0.160087	-0.33566	-0.09425	0.280116	0.21528
PLEKHA1	-0.07282	-0.54968	0.027641	-0.34438	0.286166	-0.13822	-0.19342	-0.16039
PLEKHA2	NA	NA	0.310658	0.381455	0.093349	0.539468	NA	NA
PLEKHA3	-0.1653	-0.35824	-0.24071	-0.02699	-0.13688	-0.44452	0.611198	0.355444
PLEKHA4	-0.12259	0.212078	0.083227	0.056778	-0.02208	0.361486	-0.01054	-0.04708
PLEKHA5	0.520336	0.507867	-0.06702	-0.35517	0.146467	0.040618	-0.22928	-0.3877
PLEKHA6	-0.22168	0.023233	-0.21953	0.335017	0.05511	-0.05679	-0.03035	-0.31697
PLEKHA7	0.283718	0.233046	-0.36703	-0.02078	0.269372	-0.30412	0.449423	0.314722
PLEKHA8	0.261993	0.478266	-0.18289	0.146867	0.007589	-0.40249	-0.24506	-0.19005
PLEKHB1	0.24096	0.109719	-0.10674	0.185842	0.299835	-0.01909	0.474459	-0.07929
PLEKHB2	-0.31021	-0.22885	0.365621	-0.0433	-0.03903	-0.17731	-0.19712	-0.18163
PLEKHF1	0.175356	0.353747	0.202207	0.090977	-0.14206	0.30678	-0.27937	0.061201
PLEKHF2	-0.13231	-0.375	0.149234	0.065324	0.116231	-0.20359	0.40589	0.202292
PLEKHG1	-0.03334	-0.08545	-0.06639	0.311124	0.020926	0.384996	-0.46754	-0.04751
PLEKHG2	0.171914	0.299726	-0.04271	0.157688	-0.09187	0.548904	0.17991	0.184887
PLEKHG3	-0.23679	0.161033	-0.17458	-0.24153	0.100237	0.262698	-0.17389	-0.43046
PLEKHG4	0.102408	0.079159	-0.19776	0.167299	0.195087	0.202405	0.433108	0.315502
PLEKHG4B	NA	NA	0.036632	-0.15999	0.028735	-0.03927	NA	NA
PLEKHG5	0.51535	0.562162	0.030524	-0.32241	0.102821	-0.24785	-0.10271	-0.3663
PLEKHG6	0.644156	0.211444	0.255482	-0.06381	0.364205	-0.13603	0.368615	0.137326
PLEKHH1	0.035144	0.066404	-0.29608	0.080588	-0.05247	-0.11833	0.206306	0.216946
PLEKHH2	0.081316	0.05313	-0.21201	0.09315	-0.13196	0.191832	-0.2183	-0.04937
PLEKHH3	-0.03441	0.245614	0.114727	-0.09147	0.114181	0.009425	-0.64201	-0.52046
PLEKHJ1	0.475074	0.226074	0.189943	0.133374	0.253778	-0.17605	0.085877	-0.00848
PLEKHM1	-0.25271	0.301539	0.025927	-0.1029	-0.01879	0.242138	-0.07235	0.241519
PLEKHM1P	NA	NA	0.130951	-0.18859	0.245286	-0.13795	NA	NA
PLEKHM2	NA	NA	0.403895	0.129841	0.391509	0.171385	NA	NA
PLEKHM3	NA	NA	-0.10451	-0.26317	-0.23957	-0.1322	NA	NA
PLEKHN1	-0.29011	0.009154	0.217162	0.132594	0.087395	0.172986	-0.51051	-0.09068
PLEKHO1	-0.40193	0.048949	0.20135	0.220269	-0.04688	0.710078	-0.62869	-0.53201
PLEKHO2	NA	NA	0.344466	0.145582	-0.10931	0.492795	NA	NA
PLG	0.03887	-0.0037	-0.05172	-0.00885	0.043013	0.184708	-0.1287	0.092026
PLGLB1	NA	NA	-0.20444	-0.27517	-0.11539	0.109825	0.444077	0.063436
PLGLB2	-0.09239	0.193819	NA	NA	NA	NA	NA	NA
PLIN	0.053734	-0.19546	-0.00247	-0.05278	0.325757	-0.42177	-0.03846	0.262674
PLK1	0.202078	-0.25223	-0.25932	0.299582	0.133747	0.047927	0.502178	0.167854
PLK2	-0.48625	-0.04996	-0.35142	0.029449	-0.18507	-0.1819	-0.3846	-0.47288
PLK3	-0.37639	0.4155	0.219514	0.172679	0.036452	0.123082	-0.2477	-0.27368
PLK4	-0.21817	0.266969	-0.3398	0.067409	-0.02631	-0.11538	0.491463	0.345731
PLLP	NA	NA	-0.17626	0.04052	-0.12411	-0.14535	NA	NA
PLN	-0.19922	-0.32698	0.093235	-0.02362	-0.04804	-0.2464	-0.32285	-0.46434
PLOD1	-0.43107	-0.05666	0.362707	0.047192	0.323512	0.023417	-0.21439	-0.17398
PLOD2	-0.47159	-0.38985	0.13941	-0.12615	-0.1238	-0.22735	-0.26905	0.032724
PLOD3	0.388477	-0.38084	0.328658	0.210528	0.464577	0.149375	0.584722	0.335533
PLP1	0.05169	-0.21971	-0.11492	-0.12308	-0.08363	0.104343	-0.37988	-0.34908
PLP2	0.572115	0.2722	0.188188	0.316878	-0.09738	-0.02979	0.020221	-0.30991
PLRG1	-0.24025	0.161211	-0.07721	-0.15527	-0.14796	0.224461	0.187526	0.276854
PLS1	-0.40346	-0.24357	-0.18412	-0.15115	-0.0581	-0.2696	0.245476	0.204598
PLS3	0.086457	-0.45614	-0.06467	-0.29982	-0.16527	-0.14104	-0.43487	-0.55376
PLSCR1	-0.21916	-0.53353	-0.22611	-0.13764	-0.20396	-0.03675	-0.05202	0.164298

PLSCR2	-0.26409	0.200936	-0.18866	-0.09409	-0.24399	-0.10341	0.074456	0.077229
PLSCR3	-0.05131	-0.29551	0.071873	0.329669	-0.04251	0.35077	-0.05229	-0.16177
PLSCR4	-0.02032	0.126199	0.038733	-0.41584	-0.1769	-0.3014	-0.26732	-0.05246
PLTP	0.21	-0.08941	0.153868	0.115418	0.184277	0.08959	-0.07941	0.243749
PLUNC	0.127095	0.294568	0.155881	-0.10431	0.113836	-0.16705	0.370723	0.148868
PLVAP	-0.34188	0.070533	0.122209	0.157491	0.188927	0.383802	-0.15775	0.319408
PLXDC1	-0.12496	0.373028	-0.09408	-0.03391	0.005635	0.230089	0.165934	0.366781
PLXDC2	-0.55332	-0.00581	0.002612	0.026487	-0.4742	-0.11516	-0.46909	-0.33437
PLXNA1	-0.07756	0.326998	-0.1141	0.101582	0.201569	0.448942	0.058497	0.343422
PLXNA2	-0.47319	-0.03476	0.088119	-0.0722	0.182289	-0.00945	-0.42117	-0.3684
PLXNA3	0.15369	0.414178	-0.0633	-0.21315	0.272836	-0.01518	0.729104	0.394449
PLXNA4	NA	NA	0.336957	0.230482	-0.06435	-0.02462	NA	NA
PLXNB1	0.342891	0.422061	0.23095	-0.04048	0.498126	0.263797	0.535879	0.33428
PLXNB2	NA	NA	0.217979	0.15681	0.347524	0.256058	NA	NA
PLXNB3	0.003999	0.029425	0.06886	-0.20459	0.244484	0.245491	-0.06853	-0.06709
PLXNC1	-0.04391	0.740557	0.123855	-0.05533	-0.27264	0.147885	0.062508	0.470146
PLXND1	-0.62486	0.345617	0.218266	0.309469	-0.07973	0.22882	0.1608	0.361226
PM20D1	NA	NA	0.075882	-0.10912	-0.21708	-0.0575	NA	NA
PM20D2	NA	NA	-0.2326	0.023685	0.375538	-0.057	NA	NA
PMAIP1	0.100633	0.20488	0.155741	0.217935	0.020796	-0.05824	-0.24924	-0.11105
PMCH	NA	NA	-0.03421	0.063891	-0.32776	-0.02157	0.250127	0.280029
PMCHL1	-0.38	0.026829	-0.08935	0.245522	-0.05486	-0.05865	0.516936	0.034969
PMCHL2	-0.21072	0.269955	NA	NA	NA	NA	NA	NA
PMEPA1	NA	NA	0.085583	0.009023	0.378492	0.181864	NA	NA
PMF1	0.052269	0.701068	0.193013	0.131067	-0.3832	0.144077	NA	NA
PMFBP1	0.221145	0.020466	-0.38406	-0.26944	0.080347	-0.3209	0.291353	0.054521
PML	-0.21111	-0.25423	0.167538	0.132419	0.25081	0.494049	-0.13322	0.320703
PMM1	-0.10857	-0.07041	0.098899	-0.08819	0.115236	-0.16921	0.298865	0.107218
PMM2	0.474433	0.470322	0.022393	-0.01576	0.200969	0.390573	0.343696	0.233866
PMP2	-0.0173	-0.30334	0.136858	-0.01968	0.166302	0.111414	-0.42261	-0.20399
PMP22	-0.43707	-0.36958	0.112898	-0.04974	-0.28881	0.172653	-0.1899	0.485279
PMP22CD	0.045362	0.668217	0.211743	-0.38884	0.063371	-0.09386	0.29783	-0.01393
PMPCA	0.090274	0.686027	0.038778	0.207174	0.194817	0.314718	0.560156	0.369261
PMPCB	0.4664	-0.25991	-0.03295	-0.29299	0.154946	-0.07135	0.010691	-0.2319
PMS1	0.006863	-0.58208	-0.18077	0.195792	-0.0316	0.251711	-0.04149	0.137324
PMS2	-0.02045	-0.62084	0.015639	-0.02336	0.000686	0.186459	0.42333	0.047537
PMS2CL	NA	NA	-0.0503	-0.20564	0.327945	0.301968	0.649346	0.555158
PMS2L2	-0.02664	-0.15462	0.24548	-0.02468	0.022559	0.252802	-0.24789	-0.55366
PMS2L3	0.423493	0.780132	0.063817	0.338989	-0.03266	0.121039	0.007219	0.147471
PMS2L5	-0.10527	-0.53075	0.249416	-0.05967	-0.10649	0.098474	-0.33205	-0.4459
PMVK	-0.24583	0.106354	0.01651	0.27552	-0.11226	-0.04833	-0.26029	-0.28631
PNCK	-0.23824	-0.07635	-0.33567	-0.21592	0.068034	0.034504	-0.19201	-0.31028
PNKD	-0.16216	-0.55765	0.302832	0.092079	0.001531	-0.25929	0.517847	0.349297
PNKP	0.147809	0.658757	0.425637	-0.03871	0.37936	0.02896	0.483557	0.362008
PNLDC1	0.372618	0.76024	-0.25231	0.279913	-0.02221	-0.28068	-0.23292	-0.56232
PNLIP	-0.27368	-0.10897	-0.07795	-0.01754	0.139962	-0.31452	-0.44199	0.147474
PNLIPRP1	0.077191	0.020904	0.028769	0.033502	0.218121	-0.28754	0.361757	0.312392
PNLIPRP2	0.397071	0.204303	-0.01278	-0.05599	0.156771	-0.10007	0.135314	-0.20095
PNLIPRP3	0.411902	0.371987	NA	NA	NA	NA	0.495252	-0.11268
PNMA1	-0.49308	-0.40947	-0.25546	-0.08613	-0.02205	0.061027	-0.2834	-0.29309
PNMA2	-0.37017	-0.4872	-0.17775	-0.30918	-0.14528	0.002337	0.007725	0.03081
PNMA3	0.093964	0.016633	-0.31815	0.527896	-0.15319	0.654115	0.366243	0.288526

PNMA5	-0.24328	-0.26896	-0.17578	0.403172	-0.00091	0.349007	0.497552	0.451376
PNMA6A	0.13059	-0.10231	-0.19802	-0.1073	-0.08012	0.104526	-0.07057	-0.18882
PNMAL1	NA	NA	-0.10235	-0.02817	-0.2095	0.02763	NA	NA
PNMAL2	NA	NA	0.111382	-0.11099	0.239283	0.105454	NA	NA
PNMT	0.246115	-0.13924	-0.16472	-0.23893	-0.07819	-0.41337	0.617395	0.197507
PNN	-0.03423	-0.54067	-0.28496	0.216857	-0.01585	0.068433	0.040302	0.342213
PNO1	0.047181	-0.34823	-0.16197	0.047216	0.048242	0.242092	NA	NA
PNOC	-0.14778	0.245594	0.040064	0.6138	-0.20265	-0.16072	-0.20245	0.073998
PNPLA1	0.400902	0.425598	-0.12919	0.068684	0.159445	-0.10524	-0.25781	0.449939
PNPLA2	0.14473	0.148704	0.026658	-0.0071	0.096087	-0.19414	0.495667	0.381823
PNPLA3	0.00263	0.537703	-0.16962	0.262442	-0.32591	0.083276	NA	NA
PNPLA4	0.055942	-0.53375	0.090404	-0.16595	0.094703	-0.19261	0.080925	0.216413
PNPLA5	0.107002	0.522468	0.204793	0.091545	-0.10155	-0.442	0.191413	0.26125
PNPLA6	0.210693	0.635516	0.479085	0.327469	0.166894	0.09714	NA	NA
PNPLA7	-0.32138	0.469828	0.069264	-0.09189	0.111403	0.064285	NA	NA
PNPLA8	-0.06771	-0.4422	0.018205	-0.26639	-0.08607	-0.3682	-0.51869	-0.25643
PNPO	0.184	-0.05958	0.312772	0.400857	0.096872	-0.08109	0.173994	0.010456
PNPT1	0.094242	-0.38886	-0.17596	-0.17172	0.093112	0.180954	-0.50674	-0.62964
PNRC1	0.342644	0.332639	-0.06049	0.239709	0.027786	0.161779	0.224279	0.064205
PNRC2	0.094061	0.228973	0.038244	-0.00361	-0.11546	0.13586	-0.09963	-0.33618
PODN	0.027768	-0.03649	0.296716	0.000951	0.11514	0.104028	-0.17111	-0.06346
PODNL1	0.098655	0.410493	-0.04386	0.194031	-0.24481	0.019534	NA	NA
PODXL	-0.21964	-0.4005	-0.20747	0.165579	-0.12016	0.043819	0.11801	0.076564
PODXL2	0.130513	0.62546	-0.22565	0.174096	0.28198	-0.30224	0.580943	0.119219
POF1B	-0.11799	0.138817	-0.24272	-0.06998	-0.10177	-0.37705	-0.03967	-0.25168
POFUT1	0.343831	-0.13074	0.082346	0.34411	0.111259	-0.12553	-0.1601	-0.37797
POFUT2	-0.0963	0.038883	-0.01597	0.054708	0.048851	0.0357	0.12445	0.423308
POGK	0.077803	-0.45711	-0.37425	0.10253	0.095691	0.056714	0.745062	0.492122
POGZ	-0.21835	-0.27597	0.086668	-0.07945	0.00173	-0.2001	0.003382	-0.33253
POLA1	NA	NA	0.1056	0.102436	0.26423	-0.05436	0.030895	-0.43132
POLA2	0.062129	0.460596	-0.09548	0.383939	0.338559	0.169123	0.097931	0.000632
POLB	0.091615	0.424239	0.052446	0.020543	-0.28563	-0.38077	0.420444	0.033902
POLD1	0.294334	-0.57166	-0.12282	0.455634	0.572688	0.35944	0.427582	-0.01086
POLD2	0.360856	0.483817	-0.04432	0.238773	0.134464	-0.09651	NA	NA
POLD3	-0.21817	-0.12288	-0.43633	0.088194	0.078131	-0.11611	0.069059	0.083115
POLD4	0.063876	0.167242	0.156917	-0.05458	-0.05175	-0.25524	-0.10981	0.00992
POLDIP2	0.536244	0.303976	0.128577	0.388701	0.170361	-0.03009	0.320336	0.169695
POLDIP3	-0.09476	0.488395	0.284943	0.284808	0.266157	0.526521	0.568826	0.374365
POLE	-0.35443	0.312632	0.287807	-0.00795	0.428145	-0.24366	0.438916	0.368205
POLE2	-0.01251	0.264557	-0.27197	-0.01128	-0.04659	-0.13399	0.201692	0.270034
POLE3	-0.13631	-0.22504	-0.28527	0.235759	0.091951	0.189112	0.131054	0.144491
POLE4	-0.01499	-0.04259	-0.24575	0.035855	-0.45495	-0.43459	0.269586	0.096165
POLG	-0.25507	0.662951	-0.24098	0.427876	-0.02482	0.061028	0.235769	0.240154
POLG2	0.223163	-0.51082	-0.09926	0.259883	-0.14086	0.150352	-0.49087	-0.44998
POLH	-0.41977	-0.36032	-0.15171	0.417368	-0.06936	-0.14166	-0.71983	-0.58221
POLI	0.05543	-0.46592	-0.16707	-0.06104	-0.11698	-0.12228	-0.14986	-0.02491
POLK	0.006652	-0.39037	0.084777	-0.36862	-0.10273	0.027733	-0.44175	-0.21278
POLL	0.10903	-0.39441	0.344068	-0.02441	0.232499	-0.03863	-0.00503	-0.0202
POLM	0.47338	0.259924	0.347203	0.007212	0.060582	-0.19566	0.156359	0.552353
POLN	0.093754	-0.28082	-0.05958	0.157969	-0.25497	0.186488	0.145777	0.180771
POLQ	0.037153	0.230613	-0.38527	0.067681	0.103118	-0.18647	0.328476	0.226442
POLR1A	0.332704	0.512671	0.219514	-0.12088	0.412687	0.23509	0.249064	-0.18401

POLR1B	0.2014	0.536862	-0.2917	-0.17744	0.033442	0.210932	0.114769	-0.12547
POLR1C	0.274194	0.041537	-0.32636	0.095874	-0.03256	0.353433	0.257069	0.210402
POLR1D	0.49244	0.06222	0.404947	0.117645	0.3689	-0.20858	0.410574	0.079778
POLR1E	-0.12057	-0.23263	-0.01644	0.157394	0.2314	0.373514	-0.50343	-0.49826
POLR2A	-0.55849	-0.14638	-0.02511	-0.12057	-0.14764	-0.2662	0.042776	0.167613
POLR2B	-0.07145	-0.52724	-0.0863	-0.01552	0.009726	0.29149	0.233287	-0.05968
POLR2C	0.23469	-0.01571	0.078949	0.043849	-0.0587	0.269617	0.37838	0.191845
POLR2D	0.491287	0.05246	0.103137	0.267808	0.050166	0.138154	0.584878	0.324076
POLR2E	0.291856	0.208171	-0.09845	0.082032	0.106149	0.089129	NA	NA
POLR2F	0.479091	0.156875	0.245965	-0.06611	0.192007	-0.02048	0.467644	0.124656
POLR2G	0.030253	-0.42975	-0.03778	0.171235	-0.0765	-0.04147	-0.14438	-0.33493
POLR2H	0.048659	0.150963	-0.41224	-0.00489	-0.10292	-0.49359	-0.29006	-0.19086
POLR2I	0.584124	0.236586	-0.00965	-0.03253	-0.03967	-0.0207	-0.15994	-0.33359
POLR2J	0.289628	0.537414	-0.08031	-0.07364	-0.08836	-0.17049	0.306313	0.388412
POLR2J2	0.181285	-0.0756	-0.06669	-0.11517	-0.1106	-0.34398	-0.02627	0.277339
POLR2J3	0.189313	-0.69033	NA	NA	NA	NA	-0.42221	-0.45044
POLR2K	0.145312	0.200574	0.218497	-0.41067	-0.13684	0.017502	-0.44227	-0.58736
POLR2L	-0.0035	0.355442	-0.08437	0.040829	-0.20333	-0.19335	0.097387	0.106131
POLR3A	0.11403	0.504458	0.073501	-0.13541	0.466979	0.188109	0.279465	0.367041
POLR3B	-0.08521	0.648321	-0.37768	0.034636	0.09221	-0.09626	-0.13982	-0.11997
POLR3C	0.225433	-0.00254	-0.12839	-0.06103	-0.04748	-0.07347	0.408627	0.248063
POLR3D	-0.02938	-0.45072	0.266604	0.291687	0.150514	-0.13107	0.214816	-0.01647
POLR3E	0.458386	-0.53671	0.220405	0.096148	0.364838	0.4597	NA	NA
POLR3F	0.305372	0.328311	-0.38845	-0.21967	-0.0458	-0.04257	-0.13479	-0.34191
POLR3G	-0.35652	-0.0363	-0.05763	0.063318	-0.34565	-0.09127	0.216543	0.043806
POLR3GL	-0.4169	-0.21872	-0.0393	0.063578	-0.18403	-0.2901	-0.60044	-0.47652
POLR3H	-0.13556	0.1309	0.131428	0.247412	0.234325	0.321834	0.1171	0.026727
POLR3K	0.1773	0.762431	-0.08323	-0.02095	0.069578	-0.01752	0.198921	-0.25044
POLRMT	0.115424	0.154564	0.236838	0.121708	0.273879	0.370461	0.352201	0.512661
POLS	0.144877	0.276135	-0.18737	0.086745	0.205759	0.324924	0.140669	0.211856
POM121	NA	NA	0.028917	0.249091	0.116866	0.456786	0.007675	-0.20345
POMC	-0.16473	-0.17777	0.0814	0.155024	-0.23038	-0.37521	-0.3214	-0.21685
POMGNT1	-0.09422	0.267237	0.44165	0.243306	0.432679	0.095742	-0.19421	0.016115
POMP	0.362183	-0.1867	0.366534	-0.29989	-0.03396	-0.08835	0.021194	-0.21714
POMT1	0.034087	0.666781	-0.13228	-0.00479	0.208658	0.104287	0.188527	0.233293
POMT2	-0.12847	0.101822	-0.07081	0.242465	-0.10515	0.054813	-0.3138	0.042568
POMZP3	0.247076	0.053522	0.440089	-0.05955	-0.07539	-0.03201	-0.34132	0.04602
PON1	0.127885	-0.08555	-0.13285	-0.15928	-0.13302	-0.25598	-0.04427	-0.22156
PON2	0.143947	-0.5802	-0.05325	-0.01372	0.0138	-0.4899	0.095835	-0.25136
PON3	0.25775	-0.23096	-0.17239	0.239331	-0.08915	0.098628	0.153178	0.080295
POP1	-0.23587	-0.33917	-0.02951	-0.04599	-0.24049	0.13528	0.440873	0.493581
POP4	0.369208	-0.29277	-0.15172	-0.3254	-0.26069	-0.59688	-0.49979	-0.48259
POP5	0.050685	0.176504	-0.22579	-0.09056	-0.25099	0.001769	0.214092	0.162147
POP7	0.334812	-0.40805	-0.1961	0.246473	-0.01473	-0.40937	0.404134	-0.14392
POPDC2	-0.31259	-0.18444	-0.10787	-0.18642	0.003571	-0.15756	-0.33158	-0.33062
POPDC3	0.098584	0.36321	-0.08711	0.14362	-0.2364	-0.3524	-0.0869	-0.01756
POR	0.200574	0.357431	0.408066	-0.02667	0.348483	0.042666	0.37586	0.346827
PORCN	0.210861	0.710041	-0.03462	0.08357	0.203871	-0.02757	0.006621	0.059386
POSTN	-0.35058	-0.38419	-0.07233	-0.21538	-0.23388	-0.32573	-0.31004	-0.43846
POT1	0.147081	-0.30566	-0.17308	0.003967	-0.06203	-0.06181	0.115678	-0.03464
POTEB	NA	NA	-0.03324	-0.12561	-0.14921	0.153729	NA	NA
POTEC	NA	NA	-0.16586	0.201867	-0.07353	-0.12821	NA	NA

POTEG	NA	NA	0.139633	-0.12424	-0.11893	-0.12501	NA	NA
POU1F1	-0.30689	0.016113	0.029634	-0.12771	0.20536	0.021266	0.080358	0.210442
POU2AF1	-0.40065	0.064261	0.099794	0.626078	-0.06414	0.392731	-0.11602	0.079596
POU2F1	0.156237	0.741365	-0.27143	0.09778	0.011116	-0.06842	0.133263	-0.33616
POU2F2	-0.44241	0.605822	0.229352	0.686069	-0.05519	0.660918	-0.08127	0.069256
POU2F3	0.230819	-0.09571	0.221393	-0.12242	0.088145	0.138995	-0.34107	0.151741
POU3F1	0.328099	0.151616	0.302905	-0.11301	0.176044	0.091088	0.048587	-0.22157
POU3F2	0.190287	-0.04076	-0.18744	0.073306	-0.18175	-0.46856	-0.37172	-0.34658
POU3F3	0.371523	0.64276	-0.02994	-0.04987	-0.13142	0.182241	0.183826	-0.45403
POU3F4	-0.00113	0.72935	0.045997	-0.28959	-0.08596	-0.0106	-0.43901	-0.42013
POU4F1	0.27805	0.687707	-0.25452	0.088243	0.307097	-0.01424	0.157572	0.278821
POU4F2	0.007768	0.059846	-0.0608	-0.12952	0.054567	-0.07937	0.307382	0.01621
POU4F3	-0.01725	0.437101	-0.06105	-0.02677	0.010116	-0.0092	-0.19662	-0.18949
POU5F1	-0.05976	-0.0653	0.109216	-0.03929	0.191094	-0.02602	0.170276	0.387457
POU5F1P3	NA	NA	0.173665	-0.07974	0.2263	0.056743	NA	NA
POU5F2	NA	NA	-0.22483	-0.03975	-0.0488	0.254048	NA	NA
POU6F1	-0.19912	0.574457	-0.09397	0.273649	-0.07252	0.585542	-0.54842	-0.61097
POU6F2	0.306875	0.757605	-0.17272	-0.32572	0.063541	0.162054	0.142732	-0.23358
PPA1	0.0504	-0.56858	-0.10125	0.155402	0.160175	-0.17161	0.161459	0.029822
PPA2	0.109994	-0.19597	-0.27035	0.050014	-0.25951	-0.2969	-0.51263	-0.5189
PPAN	0.341593	0.664992	0.141779	0.199183	0.286608	0.355291	0.581659	0.390187
PPAN-P2RY	NA	NA	0.05582	-0.12178	0.204577	0.219633	0.12112	-0.08515
PPAP2A	-0.28536	-0.59937	0.29834	-0.42579	-0.36923	-0.19402	-0.29933	-0.02531
PPAP2B	-0.19965	-0.61513	0.188686	-0.04555	-0.04053	0.44941	0.276031	0.293368
PPAP2C	-0.37623	0.17848	0.342034	-0.05423	0.264382	-0.08934	0.034223	-0.19789
PPAPDC1A	-0.07985	0.701043	0.078653	0.084467	-0.20495	-0.15392	0.078521	-0.27677
PPAPDC1B	-0.46497	-0.44741	-1.6E-05	0.051989	-0.04234	-0.3871	-0.15024	-0.11175
PPAPDC2	0.379574	-0.20226	-0.07304	-0.0504	0.231647	-0.30953	0.282119	0.260426
PPAPDC3	-0.27866	-0.03313	-0.06248	-0.11474	-0.16589	-0.0583	-0.3329	-0.13876
PPARA	0.198383	0.018571	0.028764	-0.21575	0.338972	0.089968	0.15415	0.160497
PPARD	-0.2463	0.364615	-0.03699	-0.29974	0.039705	0.193814	0.199141	0.258499
PPARG	0.412945	0.070772	-0.19042	-0.1317	-0.00556	-0.14833	0.038284	-0.20942
PPARGC1A	0.191374	-0.69227	0.094982	-0.22744	-0.00575	-0.04287	-0.05008	-0.12725
PPARGC1B	0.276295	0.138911	0.34701	-0.12956	0.301498	-0.05255	-0.27125	-0.20458
PPAT	0.00726	-0.51584	0.071697	-0.22598	0.026577	-0.00746	0.588561	0.196672
PPBP	-0.44431	0.267446	-0.0137	-0.178	-0.06152	-0.50243	-0.39	-0.23754
PPCDC	0.197034	-0.31061	0.243054	-0.08582	-0.01995	-0.2602	0.044585	0.056429
PPCS	0.001658	0.263111	0.11233	-0.00369	0.096727	-0.41802	-0.35637	-0.2585
PPEF1	-0.13934	0.08181	0.158518	-0.0275	-0.14768	-0.08588	-0.05125	0.012778
PPEF2	0.213873	0.718834	-0.22835	-0.03008	0.085343	-0.39153	0.324314	-0.22617
PPFIA1	-0.05844	0.216198	-0.14865	-0.20248	0.163811	0.31779	0.053453	0.070672
PPFIA2	-0.16659	-0.18345	0.059556	-0.02807	-0.31227	-0.14511	-0.29393	0.12781
PPFIA3	0.223446	0.46003	0.033218	0.115206	0.363789	-0.29249	0.274459	-0.08211
PPFIA4	-0.44528	-0.20561	-0.40376	-0.15902	0.088824	-0.10608	0.144036	0.0868
PPFIBP1	0.13597	-0.17808	-0.16004	-0.0946	-0.13	-0.12473	-0.51088	-0.58417
PPFIBP2	0.169275	0.044235	-0.18751	0.253866	0.007508	-0.05347	0.161631	0.089326
PPHLN1	0.317851	-0.52721	-0.12821	-0.11251	0.023681	0.012853	0.204326	0.50821
PPIA	-0.07727	-0.40412	-0.1322	0.212779	0.006064	-0.32476	-0.48648	-0.41892
PPIB	-0.05758	-0.18264	0.209245	0.504984	-0.2125	-0.14237	-0.68259	-0.44316
PPIC	-0.11824	-0.41859	0.215148	0.06184	-0.00329	-0.50204	-0.07461	-0.07791
PPID	0.059721	-0.52919	-0.2041	0.103335	0.16046	-0.24817	0.358091	0.152404
PPIE	-0.33737	-0.23759	0.331436	-0.03899	0.094874	-0.23612	0.076631	-0.06526

PPIF	-0.3144	-0.35595	-0.00422	0.333184	0.190865	0.308498	0.364593	0.22376
PPIG	-0.17471	-0.66155	-0.0468	0.166864	0.051707	-0.1664	-0.4612	-0.13872
PPIH	0.313191	0.167446	-0.10556	0.084084	-0.03572	-0.2562	0.242813	-0.10719
PPIL1	-0.04659	-0.51635	-0.31679	0.003359	-0.31764	-0.10798	0.51708	0.275109
PPIL2	-0.08043	-0.00496	0.131481	0.264067	0.361363	-0.14541	-0.04735	-0.01417
PPIL3	-0.17521	0.189536	0.082306	-0.27085	-0.07201	0.045497	-0.25759	0.18068
PPIL4	0.33429	-0.38878	-0.50498	0.293189	0.067212	0.119517	-0.29143	0.050666
PPIL5	-0.05565	-0.17677	-0.37064	-0.11541	-0.2471	-0.35272	0.202738	0.208743
PPIL6	0.205856	-0.21749	-0.17182	0.004759	-0.00999	-0.40911	0.00752	-0.59821
PPL	0.037666	-0.39332	0.267685	-0.20664	0.238666	0.249803	0.424312	-0.1805
PPM1A	-0.22257	-0.21684	-0.34965	-0.4878	-0.16765	-0.2496	0.269555	0.360362
PPM1B	0.187868	0.359931	-0.28589	-0.18937	0.053267	-0.30312	-0.08132	0.102442
PPM1D	-0.05232	0.030315	-0.24535	-0.29633	-0.14586	-0.26332	-0.42857	-0.22415
PPM1E	0.149073	0.171	0.169767	-0.2419	-0.17518	0.254149	0.4996	0.352347
PPM1F	0.128728	0.0867	0.093348	0.319047	0.124878	0.091717	-0.46339	-0.04256
PPM1G	0.254815	0.392114	0.326083	0.38347	0.443193	0.114666	0.399687	0.273772
PPM1H	NA	NA	0.001103	0.369196	0.111762	-0.13402	NA	NA
PPM1J	-0.01557	0.252989	0.238203	0.219384	0.220354	0.14582	0.105258	0.233482
PPM1K	-0.18649	-0.21738	0.010503	0.382552	-0.231	0.211476	-0.50463	-0.5657
PPM1L	-0.1617	0.181947	-0.03462	-0.07682	0.314802	-0.39337	-0.32295	-0.3267
PPM1M	-0.28384	0.241342	0.285597	0.240263	-0.06692	0.432554	-0.34055	-0.0333
PPM2C	-0.12274	-0.55187	0.288314	-0.41125	-0.17833	0.047938	0.039462	-0.09194
PPME1	0.106073	0.552901	0.219286	0.121653	0.306809	-0.037	0.606368	0.241551
PPOX	0.066268	0.330916	0.368565	0.098656	0.171468	0.116611	0.436568	-0.00031
PPP1CA	0.029738	-0.29924	0.26716	0.286882	0.270493	0.001664	0.473559	0.295087
PPP1CB	-0.0622	-0.52221	-0.10852	-0.33066	0.162105	-0.24523	-0.38787	-0.4123
PPP1CC	-0.15538	-0.47429	-0.26124	-0.01064	0.070247	-0.09079	-0.20487	-0.01419
PPP1R10	-0.10351	-0.15399	0.060068	0.365019	0.064632	0.092826	-0.287	-0.23212
PPP1R11	0.168491	0.594924	0.073958	0.088638	-0.08394	-0.22683	-0.0462	0.266082
PPP1R12A	-0.07693	-0.44571	-0.33224	-0.30477	-0.05255	0.113228	-0.59353	-0.3788
PPP1R12B	0.042801	-0.17925	-0.20592	-0.31006	-0.33382	0.091749	-0.19554	-0.2643
PPP1R12C	0.25836	-0.11464	0.123103	-0.10255	0.358956	0.053615	-0.47383	-0.37601
PPP1R13B	-0.17491	0.56835	-0.07133	-0.11166	0.214539	0.299855	0.011605	0.025806
PPP1R13L	0.001892	0.415801	0.037292	-0.25047	0.004508	-0.0145	0.509424	0.192013
PPP1R14A	0.104375	0.06067	0.035171	-0.10108	-0.01587	-0.0679	-0.28874	-0.39146
PPP1R14B	-0.19071	0.448454	-0.09559	0.435833	0.146037	0.191511	0.596943	0.275511
PPP1R14C	0.493062	0.021786	0.451077	0.020744	0.318976	-0.15671	0.129711	-0.03493
PPP1R14D	0.673293	0.149144	0.018967	0.038943	-0.09348	-0.5052	0.006915	-0.01591
PPP1R15A	-0.35041	0.008887	-0.00841	0.063401	0.069357	-0.07189	-0.00905	-0.05958
PPP1R15B	-0.1196	-0.53482	-0.54205	-0.12509	-0.28936	-0.15637	-0.6222	-0.36588
PPP1R16A	0.166472	0.283871	0.146199	0.089699	0.234907	-0.08685	-0.14885	-0.19958
PPP1R16B	0.016683	0.522505	0.251179	0.600556	-0.03806	0.574288	-0.62521	0.21113
PPP1R1A	NA	NA	0.253325	-0.21969	0.036039	-0.06111	-0.36415	0.001741
PPP1R1B	-0.08967	0.467969	0.230362	0.292336	0.123274	0.220428	0.265674	0.08251
PPP1R1C	NA	NA	0.034711	-0.06528	-0.34741	-0.2256	0.132702	-0.14907
PPP1R2	0.178837	-0.4785	-0.00745	-0.28494	-0.0136	-0.18508	0.187964	0.084049
PPP1R2P9	NA	NA	-0.47564	0.054731	0.209553	0.13807	0.457765	0.084177
PPP1R3A	0.254923	0.712964	0.138472	0.235758	-0.0034	0.123229	-0.01026	0.123679
PPP1R3B	0.09848	-0.01663	0.003342	-0.29945	-0.16586	-0.19799	0.101808	-0.08934
PPP1R3C	-0.32744	-0.27431	-0.00693	-0.09009	-0.20068	0.044516	-0.15769	-0.24179
PPP1R3D	0.426692	-0.02316	0.289223	0.058647	0.190429	-0.19331	0.248781	0.227459
PPP1R3E	NA	NA	-0.31182	0.219908	-0.15072	-0.16442	NA	NA

PPP1R3F	0.286023	0.122255	-0.20985	-0.02998	-0.1108	-0.2387	-0.23645	-0.02928
PPP1R7	0.230146	-0.15024	0.129732	0.21534	0.060981	-0.28324	-0.02906	-0.28926
PPP1R8	0.204707	0.707414	-0.1696	0.170041	0.016939	-0.11878	0.155422	0.104372
PPP1R9A	NA	NA	-0.31403	-0.1833	-0.34278	0.019937	-0.18847	-0.59155
PPP1R9B	0.228748	0.637484	0.225366	0.388933	0.250253	0.356773	-0.24921	-0.23221
PPP2CA	-0.04672	0.585	-0.03224	0.026089	-0.02428	0.169599	0.185041	0.175631
PPP2CB	-0.18474	-0.22581	0.138146	-0.32812	-0.14176	-0.11325	-0.20361	-0.09145
PPP2R1A	0.222439	0.607038	0.289995	0.25884	0.256255	0.06052	0.590372	0.425613
PPP2R1B	0.148893	-0.03047	-0.05585	-0.16537	-0.17713	-0.5005	-0.17031	0.169305
PPP2R2A	0.134996	0.437061	0.080609	-0.01719	-0.15211	0.120103	0.451659	0.33767
PPP2R2B	0.096866	0.376455	-0.08907	-0.15822	-0.43428	0.176903	-0.38071	-0.24963
PPP2R2C	-0.12919	0.005931	0.306345	-0.08562	-0.03184	-0.28026	0.257703	0.194383
PPP2R2D	-0.33973	0.23454	-0.06383	-0.2085	0.218905	0.300947	0.127527	0.146219
PPP2R3A	0.010775	0.652992	-0.417	-0.46765	-0.3381	-0.45706	-0.62394	-0.26864
PPP2R3B	-0.00454	-0.51628	0.178709	0.122268	0.342357	0.038485	0.231305	0.294101
PPP2R3C	-0.24493	-0.66465	-0.17845	-0.40332	-0.20206	-0.17246	-0.65118	-0.27231
PPP2R4	-0.10415	0.219862	0.414645	0.051754	0.091375	-0.11917	-0.65766	-0.45329
PPP2R5A	0.01451	0.013879	-0.22354	-0.1731	0.015473	-0.04377	0.533206	0.306866
PPP2R5B	0.002441	0.087248	0.322708	-0.08575	0.241253	-0.05984	-0.46883	0.201293
PPP2R5C	-0.14329	-0.08515	-0.32696	0.083997	0.089665	-0.27753	0.316559	0.391867
PPP2R5D	0.06582	-0.22325	0.180455	0.006381	0.240612	-0.04965	0.314458	0.24382
PPP2R5E	0.126813	-0.21514	-0.4118	-0.25413	0.004391	-0.10828	-0.24163	-0.25842
PPP3CA	-0.46424	-0.03069	-0.289	0.279143	-0.00562	0.384302	-0.49639	-0.48241
PPP3CB	-0.21168	-0.00633	-0.09498	-0.13791	0.179288	0.018406	0.064769	0.166546
PPP3CC	0.111226	-0.1173	0.008235	0.1525	-0.02482	-0.19659	-0.34366	-0.02905
PPP3R1	-0.40472	-0.54529	-0.06937	-0.09924	0.210187	0.035161	0.462328	0.37826
PPP3R2	0.060204	0.120181	-0.00309	0.040266	0.126842	-0.22974	0.431244	0.139571
PPP4C	0.194697	-0.09456	0.037992	0.164582	0.129832	-0.02929	NA	NA
PPP4R1	-0.06292	-0.10415	-0.1853	-0.17524	0.125049	-0.17433	-0.287	0.011611
PPP4R1L	0.063263	0.117886	-0.36316	0.142763	0.252805	-0.05032	-0.32225	-0.14082
PPP4R2	-0.25381	-0.22477	-0.23154	-0.12895	-0.06408	-0.36676	-0.08379	-0.20269
PPP4R4	NA	NA	-0.15714	0.323184	-0.15632	0.214427	NA	NA
PPP5C	0.117566	0.626017	0.166657	0.13831	0.134258	-0.21378	0.042148	-0.19438
PPP6C	-0.13065	0.405881	-0.26731	-0.06921	-0.01091	-0.57449	-0.30433	-0.15429
PPRC1	0.099001	0.274979	-0.14588	0.199751	0.267616	0.434049	-0.03673	0.165047
PPT1	-0.35851	-0.24103	0.145208	0.076931	0.041481	0.235509	-0.30923	-0.33485
PPT2	0.136323	0.309043	-0.11149	-0.01619	-0.14125	-0.36864	0.394264	0.10666
PPTC7	-0.04893	-0.08527	-0.30312	-0.03758	0.129627	-0.17027	NA	NA
PPWD1	-0.17243	-0.68467	-0.09289	0.006945	-0.15854	0.105826	0.497311	0.520412
PPY	0.017791	0.615752	-0.05652	-0.25283	-0.1337	-0.1988	0.223564	-0.08221
PPY2	NA	NA	-0.26263	-0.32277	-0.30398	-0.19625	-0.19459	-0.16281
PPYR1	0.070903	0.010937	0.37028	-0.12323	0.254437	0.013962	0.29072	0.018617
PQBP1	0.28718	-0.40017	-0.17522	0.303499	0.120746	0.056598	-0.10768	-0.11143
PQLC1	-0.49282	-0.28492	0.289662	-0.00881	0.202997	-0.097	-0.10314	-0.20193
PQLC2	-0.12941	0.075256	0.012281	-0.18843	0.459069	-0.00747	-0.36534	-0.3087
PQLC3	-0.30798	-0.65061	0.066018	-0.43359	-0.25701	-0.26293	-0.10106	-0.12248
PRAC	-0.06789	-0.20703	0.079694	-0.31786	-0.16187	-0.26973	-0.04421	-0.18124
PRAF2	0.119045	0.325157	0.258312	0.205723	-0.06657	-0.19434	-0.22997	-0.05754
PRAM1	-0.38643	-0.35599	0.200736	-0.15449	-0.05228	0.054304	-0.10781	0.54119
PRAME	-0.13002	0.372257	-0.02997	-0.32035	-0.1756	-0.01627	0.412241	0.337002
PRAMEF1	-0.1719	0.279982	0.039324	-0.09517	-0.13119	-0.11612	0.646761	0.100141
PRAMEF10	NA	NA	-0.13456	-0.2797	-0.00821	0.018326	0.009489	0.10476

PRAMEF16	NA	NA	0.055042	0.119516	-0.05311	-0.26747	0.636366	0.273704
PRAMEF2	-0.05496	0.713632	-0.04042	-0.1033	0.021916	0.363633	NA	NA
PRAMEF3	-0.2342	0.285322	-0.16823	-0.07485	-0.03998	-0.04614	NA	NA
PRAMEF4	0.076262	0.184451	NA	NA	NA	NA	-0.14986	0.048025
PRAMEF8	-0.14781	0.320106	0.252491	-0.06232	-0.12321	0.098281	0.462646	0.357175
PRAP1	0.40428	0.483804	0.473279	-0.47695	0.256696	-0.18638	0.153707	0.253663
PRB1	-0.11153	0.165019	0.111721	0.321171	-0.17051	0.050991	0.297639	0.130093
PRB2	0.065369	-0.2869	0.018816	0.354657	-0.04939	0.182441	0.512562	0.030335
PRB3	0.416266	0.101506	-0.00428	-0.24185	-0.2324	-0.16453	-0.04161	-0.42665
PRB4	-0.338	-0.35119	0.01687	-0.21818	-0.05933	0.101327	0.2584	-0.15404
PRC1	-0.26231	0.206529	-0.2591	0.150311	0.244123	0.066547	0.366984	0.194425
PRCC	-0.01572	-0.0208	-0.30326	0.382966	0.025336	-0.13657	0.563555	0.175315
PRCD	NA	NA	0.067494	-0.00131	0.099633	0.043417	NA	NA
PRCP	-0.0743	-0.10949	0.243724	-0.12098	-0.04463	0.10781	-0.20111	-0.18563
PRDM1	-0.11516	0.089317	0.014515	0.229265	-0.31705	0.002606	-0.3418	-0.21572
PRDM10	0.044089	-0.40879	-0.4248	-0.10792	-0.06215	-0.18918	-0.02903	0.250972
PRDM11	-0.14666	0.775893	-0.32291	0.082943	-0.0508	0.123397	-0.22952	-0.13525
PRDM12	-0.11467	0.205856	-0.32705	0.202389	0.006406	-0.25322	-0.08889	-0.16097
PRDM13	0.339672	-0.00678	-0.389	0.066928	0.06798	0.083794	-0.05353	0.148471
PRDM14	0.329434	0.295141	-0.06585	-0.1409	-0.10277	0.01145	0.260325	0.481377
PRDM15	0.300505	0.377663	-0.16669	0.457103	0.115866	-0.05883	0.224736	0.311173
PRDM16	-0.21731	0.023882	-0.16599	0.204164	0.156709	0.094048	0.070054	-0.02513
PRDM2	0.157683	0.208935	0.025739	0.293037	0.198139	0.341924	-0.18341	-0.026
PRDM4	0.132011	0.411847	-0.03311	0.018202	0.197632	-0.37042	-0.40837	-0.21327
PRDM5	-0.21166	0.604208	-0.10954	-0.4264	0.023906	-0.0831	-0.04849	-0.11174
PRDM6	NA	NA	0.118143	-0.07369	-0.11737	-0.04768	NA	NA
PRDM7	0.142485	0.181132	-0.12716	-0.15948	-0.10919	-0.03414	0.562128	0.436172
PRDM8	-0.38838	-0.29732	-0.01232	-0.12087	-0.12869	0.189741	-0.46165	-0.27828
PRDM9	-0.18344	0.736026	0.17472	-0.10675	0.017337	0.108992	0.441547	0.317683
PRDX1	-0.3259	0.376031	0.281523	0.057099	0.418241	-0.15611	0.043194	0.04881
PRDX2	-0.09502	-0.69133	0.143674	0.071089	0.057887	-0.18689	0.302994	0.097135
PRDX3	-0.22976	-0.58971	-0.18843	-0.24092	0.058274	0.093481	0.42697	0.291184
PRDX4	0.107471	-0.39978	-0.02384	0.362225	0.123912	-0.46852	-0.05273	-0.27767
PRDX5	0.210023	-0.2142	0.181286	0.133556	-0.08581	0.136446	0.053568	0.074035
PRDX6	-0.21858	-0.13896	-0.2629	-0.37763	-0.41943	-0.49437	0.428729	-0.34965
PREB	-0.09779	0.120192	0.543978	0.254384	0.414437	-0.03826	0.447907	0.11365
PRELID1	0.430449	0.585161	0.418152	-0.04151	-0.0198	-0.11486	0.303456	0.414943
PRELID2	0.098112	-0.10087	0.103813	-0.02658	0.155437	-0.24998	0.055236	0.052782
PRELP	0.165006	0.056364	0.363761	-0.01525	0.015227	0.259937	-0.13663	-0.09561
PREP	-0.03891	-0.06654	-0.15098	0.146224	0.156812	0.450147	0.129807	-0.08682
PREPL	0.140678	-0.57694	-0.33585	-0.31551	-0.11271	-0.33871	0.626768	0.383245
PREX1	-0.31491	-0.43368	0.033598	0.495884	0.015234	0.569323	-0.50027	0.177512
PREX2	NA	NA	0.158649	-0.21649	0.185582	-0.02603	NA	NA
PRF1	-0.45318	0.01421	0.536047	0.183567	0.042311	0.239336	-0.10349	0.602244
PRG2	-0.39729	-0.07498	-0.09576	-0.23493	0.017064	0.032449	-0.10918	0.170778
PRG3	-0.12279	0.196654	-0.29374	-0.1867	-0.11595	-0.28015	-0.08981	-0.44173
PRG4	0.006458	-0.30183	-0.03566	0.007566	-0.06343	0.246807	NA	NA
PRH1	0.426662	0.403062	NA	NA	NA	NA	-0.6447	-0.29095
PRH2	-0.04067	0.10968	-0.14958	-0.21913	-0.2081	-0.27255	-0.1404	-0.26227
PRIC285	0.187302	0.259767	0.238334	-0.13446	0.076291	-0.18536	-0.10113	-0.08309
PRICKLE1	-0.1435	0.044481	0.231528	0.046769	-0.17553	0.366288	-0.31352	-0.20275
PRICKLE2	-0.08839	-0.27333	0.115501	-0.4476	-0.30157	0.193537	-0.26689	-0.22344

PRICKLE3	NA	NA	0.083181	0.07007	0.283695	0.057718	NA	NA
PRIM1	0.173424	-0.1456	-0.1421	-0.06723	0.101413	0.00506	0.329011	0.211248
PRIM2	NA	NA	-0.30162	0.04589	-0.0634	-0.42029	NA	NA
PRIMA1	-0.02026	-0.08117	-0.04662	-0.09408	0.005299	0.009141	-0.13026	-0.17913
PRKAA1	0.119614	-0.23789	-0.09072	-0.17685	-0.20323	-0.60123	0.076962	-0.00318
PRKAA2	-0.14721	-0.0952	0.272954	-0.26922	-0.15902	-0.1621	0.104313	-0.07083
PRKAB1	-0.13488	0.371173	0.021677	-0.05383	0.306745	0.170705	-0.0213	-0.08326
PRKAB2	-0.21659	-0.23509	-0.23283	-0.51182	-0.04782	-0.15832	0.23688	0.118588
PRKACA	0.096134	0.712595	0.394292	-0.06385	0.142396	-0.00542	-0.14382	0.242919
PRKACB	0.138078	0.148384	-0.12289	-0.27473	-0.14725	-0.528	0.046782	-0.02014
PRKACG	0.227008	0.237375	-0.10731	-0.28839	0.061289	-0.19231	0.048414	-0.09084
PRKAG1	0.526249	0.302505	0.183486	0.082134	0.049625	-0.02103	0.283325	0.374393
PRKAG2	-0.23636	-0.34264	0.060059	-0.137	-0.086	-0.30815	-0.05802	0.132155
PRKAG3	0.240761	0.765015	0.065543	-0.34052	0.103971	0.011994	-0.01619	-0.33845
PRKAR1A	-0.26758	-0.61796	-0.04575	0.176098	-0.0637	0.048527	-0.14456	-0.48106
PRKAR1B	0.295357	0.620253	0.149641	0.371384	0.331096	-0.25148	0.415607	0.190577
PRKAR2A	0.201959	-0.49822	0.28859	-0.1253	0.259207	-0.20862	0.059324	0.097732
PRKAR2B	0.118499	0.333752	-0.13478	-0.22674	-0.19017	0.018822	-0.29093	0.010281
PRKCA	-0.06194	-0.2264	0.202462	0.03748	0.278196	-0.04702	0.157692	0.093886
PRKCB	NA	NA	0.215256	0.681719	-0.03398	0.683859	NA	NA
PRKCD	-0.34364	0.039979	0.02535	0.116795	0.263306	0.068099	-0.04944	0.136886
PRKCDBP	-0.24508	0.01137	0.016496	-0.03638	-0.13782	-0.01159	-0.28285	-0.30264
PRKCE	0.059938	0.522489	0.217719	-0.01844	0.37832	-0.21977	0.074302	0.056711
PRKCG	0.035952	-0.18915	-0.17142	0.071444	0.216504	-0.13428	0.004524	0.069042
PRKCH	-0.30946	-0.50167	0.361554	0.61828	0.058307	0.633591	-0.23036	0.611028
PRKCI	-0.1005	-0.39015	0.078094	-0.22342	-0.05573	-2E-05	-0.31388	-0.32907
PRKCQ	0.439711	0.142397	0.340153	0.097387	0.243621	0.597429	-0.22387	-0.0738
PRKCSH	0.191701	0.491012	0.274421	0.346226	0.396617	0.014512	0.338075	-0.15648
PRKCZ	-0.14021	-0.00158	0.257521	-0.00022	0.283972	-0.00725	0.435419	-0.02724
PRKD1	-0.07889	-0.14644	-0.06309	0.184049	-0.084	0.151863	-0.31262	-0.12691
PRKD2	0.060364	0.405486	0.34537	0.11043	0.284952	0.443558	-0.10254	-0.23687
PRKD3	0.418995	0.333661	-0.05524	-0.04231	-0.15105	-0.09162	0.035663	0.140857
PRKDC	0.053468	0.189694	0.173788	0.40833	0.188008	0.359968	0.402165	0.255464
PRKG1	0.026352	0.6658	0.114524	-0.3486	-0.10277	-0.03041	0.358855	0.192592
PRKG2	0.206578	-0.00113	0.118355	-0.3724	0.019694	-0.08909	-0.02333	0.328207
PRKRA	0.193623	0.284642	-0.30834	0.060844	-0.1017	-0.2759	NA	NA
PRKRIP1	0.105073	0.446083	-0.05457	0.266921	0.357746	0.169985	-0.10751	0.099781
PRKRIR	0.035796	-0.51752	-0.17917	-0.19763	0.063801	-0.10605	0.353988	0.096751
PRKX	-0.09591	0.082715	0.120706	0.117783	0.049885	0.307717	-0.35354	0.153243
PRKY	0.126697	0.01218	0.090172	0.078856	-0.21664	-0.02114	0.15609	-0.10048
PRL	-0.13186	0.003902	-0.0173	0.038823	0.015136	0.368317	-0.26962	0.222652
PRLH	0.127103	-0.07536	0.060166	-0.27976	0.357178	-0.13002	0.379575	0.245417
PRLHR	0.307029	0.739182	-0.14569	-0.06784	0.127498	-0.18037	0.173927	0.159622
PRLR	0.272998	0.504214	0.087287	-0.04941	-0.12547	-0.27244	0.191059	0.200142
PRM1	-0.19577	0.143713	0.204839	-0.08743	0.339335	0.005724	0.114441	-0.06283
PRM2	0.003225	-0.05818	0.227022	-0.21597	0.034237	0.060251	0.027982	0.146916
PRM3	-0.30973	-0.36284	-0.15354	-0.07598	-0.16947	0.03095	0.335075	0.31557
PRMT1	0.139737	-0.05282	-0.05971	0.313376	0.091041	0.110098	0.435932	0.124125
PRMT2	-0.09886	0.031339	0.111285	0.111689	-0.00021	-0.07041	-0.43058	-0.29714
PRMT3	0.113988	-0.43563	-0.1395	0.011782	0.109138	0.318599	NA	NA
PRMT5	0.132243	0.548212	-0.25031	0.222784	0.026509	0.033427	0.065523	-0.25617
PRMT6	0.240738	-0.46552	0.11069	0.407999	0.289135	0.296571	0.404842	0.465186

PRMT7	0.188626	0.474978	0.149363	0.288419	0.519463	0.08723	0.619091	0.224371
PRMT8	-0.10868	-0.11956	-0.16565	0.032611	-0.19075	-0.32136	-0.4058	-0.41546
PRND	-0.35675	-0.03752	-0.22833	-0.12931	-0.05487	-0.11973	-0.51602	-0.11792
PRNP	0.119533	-0.18985	-0.26552	-0.01644	0.045994	0.215294	-0.36342	-0.19466
PRNT	0.211798	0.314529	0.213358	-0.0729	0.27195	-0.06215	-0.20622	-0.20458
PROC	-0.28466	-0.2822	-0.12361	-0.32769	-0.13598	-0.02411	0.32122	0.014335
PROCA1	0.329572	0.725045	-0.06089	0.228755	0.058475	0.037193	-0.2097	-0.16446
PROCR	0.272743	-0.09496	0.265011	0.044074	0.327717	0.118029	-0.21203	0.036527
PRODH	-0.07926	0.168971	-0.31998	-0.00202	0.053298	-0.06991	0.408351	0.444247
PRODH2	0.0951	0.509801	-0.17166	0.30486	-0.19945	0.060472	-0.14888	-0.3622
PROK1	0.120167	-0.12499	0.378064	0.054332	-0.17363	-0.17018	-0.28362	-0.00073
PROK2	-0.54555	-0.22328	-0.03811	0.252545	0.092003	0.001864	-0.19041	0.005458
PROKR1	0.315232	0.379841	0.102732	-0.05685	-0.11923	-0.1325	0.135617	0.10469
PROKR2	0.182814	0.687283	0.014146	-0.21802	-0.1087	-0.04051	-0.33472	-0.24595
PROL1	0.293778	0.351108	0.040376	-0.13193	0.209702	0.073537	0.538451	0.322018
PROM1	-0.17851	0.347413	-0.36362	-0.17134	0.008382	-0.18539	0.244877	0.102964
PROM2	-0.03078	0.343393	0.08898	0.094163	0.029519	-0.17858	0.160102	-0.19715
PROP1	-0.0021	0.109236	0.225331	-0.08822	0.062323	-0.16135	-0.39995	-0.35999
PROS1	-0.14002	-0.15117	0.06417	-0.3099	-0.00854	0.05932	-0.08194	0.061953
ProSAPiP1	NA	NA	-0.06317	-0.02249	0.013819	-0.252	0.500664	0.413051
PROSC	0.005589	-0.32388	0.366929	0.167641	0.021291	-0.35011	0.647377	0.411403
PROX1	-0.1829	-0.61174	0.133976	-0.14569	0.135611	0.029849	0.229953	-0.29377
PROX2	NA	NA	0.156231	0.35961	-0.06747	-0.04285	NA	NA
PROZ	-0.03752	0.326174	-0.15266	-0.031	-0.14248	-0.20225	0.268802	0.334325
PRPF18	-0.14038	-0.52023	-0.24131	-0.14515	-0.20934	-0.37525	-0.2676	-0.09734
PRPF19	0.169668	0.691324	0.202496	0.198735	0.107837	0.371246	0.212908	-0.18018
PRPF3	-0.05379	-0.37264	-0.07654	0.410526	0.024366	0.181978	0.339853	0.5395
PRPF31	0.374803	-0.48653	-0.25098	0.315677	0.178453	-0.24511	0.366405	-0.03876
PRPF38A	0.110026	0.259284	0.0256	0.236503	0.423335	-0.11373	-0.19995	-0.27542
PRPF38B	-0.11991	-0.61749	-0.25452	0.171293	0.11791	0.371996	0.359302	0.41514
PRPF39	-0.39569	-0.54774	-0.36839	-0.06736	-0.05564	-0.01002	0.00164	0.026452
PRPF4	-0.02806	-0.40991	0.054512	0.175297	0.127898	0.319414	0.45424	0.314249
PRPF40A	NA	NA	-0.03518	-0.04167	0.116655	-0.07605	NA	NA
PRPF40B	0.382915	-0.16359	0.213266	0.101875	0.483329	0.12813	-0.27112	-0.21258
PRPF4B	-0.11805	-0.31019	-0.36577	0.038469	-0.16171	0.099509	0.311607	0.327346
PRPF6	0.415642	0.137936	0.354184	0.355369	0.499357	0.57228	NA	NA
PRPF8	0.160752	0.191913	0.10327	0.325289	0.030183	0.462039	0.002196	-0.20259
PRPH	-0.30064	0.051213	-0.12354	0.09273	-0.21765	-0.185	-0.22019	-0.49769
PRPH2	-0.15792	-0.19786	0.001226	0.163554	-0.18285	-0.14234	NA	NA
PRPS1	0.110217	-0.38804	-0.08492	0.363433	0.048924	0.202793	0.207806	0.077709
PRPS1L1	0.535802	0.404092	0.048676	0.363451	0.065346	0.039289	-0.31815	0.104195
PRPS2	-0.19021	-0.46568	-0.30259	0.1146	0.174352	-0.05507	-0.30987	-0.58529
PRPSAP1	-0.233	-0.28175	0.223728	0.118121	-0.07185	-0.43993	0.338702	0.087555
PRPSAP2	-0.22234	-0.51409	-0.21953	0.546827	-0.25418	0.048994	0.281544	0.01839
PRR11	-0.0777	0.349046	0.069703	-0.16974	-0.05944	0.478199	0.295196	0.037563
PRR12	NA	NA	-0.04157	0.329824	0.165323	0.32216	NA	NA
PRR13	-0.04021	-0.26895	-0.13387	0.130695	0.093849	-0.32836	-0.07716	0.185595
PRR14	-0.04052	0.610546	0.280467	0.222701	0.278548	0.289808	NA	NA
PRR15	0.367552	0.120404	0.036442	-0.09734	0.199989	-0.20369	-0.35313	-0.55662
PRR16	-0.14221	-0.20193	0.029168	-0.09069	-0.03265	0.011144	NA	NA
PRR18	NA	NA	0.132605	-0.35253	0.064544	0.116705	NA	NA
PRR3	0.159683	0.304668	0.131606	0.303918	0.230797	0.059702	-0.40782	-0.37979

PRR4	0.266571	0.791217	-0.01811	-0.23586	-0.32054	0.057491	0.491053	0.567303
PRR5	-0.00377	0.188469	0.124294	0.053182	0.113329	0.292036	0.514063	0.629519
PRR7	0.284148	-0.11038	0.303715	0.081105	0.136189	0.293971	0.406658	0.179839
PRRC1	-0.33939	-0.6902	0.356544	-0.05956	-0.08428	-0.03124	NA	NA
PRRG1	0.04353	-0.10403	0.045414	-0.23327	-0.1163	-0.16868	0.095808	0.309584
PRRG2	0.034497	-0.17472	0.213932	-0.2393	0.147092	-0.39636	0.327457	-0.02372
PRRG3	0.269845	0.247541	0.106312	-0.28151	0.20111	-0.13646	-0.08802	0.045386
PRRG4	-0.06914	-0.08702	0.025826	-0.09851	0.142389	0.023979	-0.55466	-0.51445
PRRT1	0.300821	0.293896	-0.08476	-0.01715	-0.06498	-0.09197	-0.5008	-0.33697
PRRT2	-0.05136	0.124086	0.044386	-0.16155	-0.02666	0.159618	-0.45241	-0.34818
PRRT3	-0.00466	0.126819	-0.01998	0.005662	0.002932	-0.17216	-0.66094	-0.5
PRRX1	-0.27435	-0.44816	-0.09167	0.402903	-0.35306	0.185561	-0.24224	0.045701
PRRX2	-0.16978	0.040002	0.051521	0.03161	-0.28259	-0.24241	-0.27881	-0.07279
PRSS1	0.141133	0.65644	-0.19594	-0.38593	-0.04114	-0.28916	0.202267	-0.32124
PRSS12	-0.12932	-0.20756	-0.10009	-0.10302	-0.08506	-0.42098	0.050107	0.266541
PRSS16	0.227836	0.329457	-0.0661	0.149411	-0.11575	-0.19857	0.511202	0.49353
PRSS2	0.001645	0.022733	-0.09199	-0.12688	-0.03907	-0.26892	0.07287	0.276905
PRSS21	-0.24572	0.379057	-0.08083	0.157439	0.129098	0.335772	0.291889	0.190241
PRSS22	-0.15859	0.115532	0.149411	0.025322	0.07888	-0.00843	0.349116	-0.10501
PRSS23	-0.18315	-0.01133	0.228116	-0.21932	0.096079	-0.27461	-0.28663	-0.56249
PRSS27	0.00549	0.216994	-0.15355	-0.29156	-0.083	0.170741	0.005214	0.464945
PRSS3	0.380725	0.109076	-0.055	-0.09615	-0.07347	-0.00688	0.309992	0.262337
PRSS33	0.177338	0.767096	0.306481	-0.22276	0.198601	-0.14899	0.134719	0.056282
PRSS35	-0.37283	-0.10621	-0.1161	-0.01204	-0.08265	0.306169	-0.32842	-0.52594
PRSS36	0.244131	0.632013	-0.16516	0.014069	-0.01529	0.121283	NA	NA
PRSS7	-0.07571	0.052125	0.184408	-0.08212	0.05567	-0.07866	0.256868	0.163092
PRSS8	-0.01997	0.196449	0.323747	0.097197	0.372048	-0.15999	0.099026	-0.24688
PRSSL1	0.071108	0.457784	-0.08913	-0.06015	0.075514	-0.04472	-0.29509	0.062232
PRTFDC1	0.188232	-0.22644	-0.37389	-0.235	-0.07327	-0.06461	-0.27072	-0.3295
PRTG	NA	NA	-0.2025	0.200575	-0.1968	0.063753	-0.09499	0.088165
PRTN3	-0.36156	0.116844	0.087609	0.043411	0.191011	-0.22911	0.425121	0.169607
PRUNE	-0.23226	-0.33097	0.065694	0.119238	-0.27378	-0.37157	0.57875	0.342522
PRUNE2	-0.35676	-0.22857	0.083147	-0.19859	0.227375	0.069416	-0.38087	-0.46862
PRX	-0.13955	0.170933	0.064811	0.050963	0.007472	0.176502	-0.11748	-0.32297
PRY2	0.075418	-0.10721	-0.08308	-0.12491	-0.56126	-0.09598	NA	NA
PSAP	-0.37839	0.105174	0.365706	0.15657	0.275193	0.229128	-0.60954	-0.49086
PSAPL1	NA	NA	-0.42059	-0.14558	-0.25074	0.17286	NA	NA
PSAT1	-0.06035	0.364015	-0.1032	0.265401	0.081967	-0.569	0.078366	0.122022
PSCA	-0.28555	0.033942	-0.08835	0.034347	-0.40955	-0.36582	0.085796	-0.22086
PSD	0.342746	0.694067	-0.00865	-0.13598	-0.26708	-0.01712	-0.23381	-0.21998
PSD2	-0.32268	0.505929	-0.43104	-0.13094	0.259518	0.421636	0.36777	0.164605
PSD3	-0.24691	-0.42643	0.407256	-0.31323	-0.23422	-0.3598	0.156194	-0.19015
PSD4	0.157118	0.180287	0.348719	0.398367	0.259094	0.133251	0.407029	0.18832
PSEN1	-0.18046	-0.01791	-0.11221	-0.34494	-0.23449	0.131463	-0.05209	0.096786
PSEN2	0.373281	0.219076	0.435278	0.093516	0.148909	-0.09757	0.231794	0.216613
PSENEN	0.323138	0.617486	0.10705	0.033414	-0.23294	-0.31205	0.52714	0.005731
PSG1	0.052283	-0.11519	0.147936	0.004657	0.159284	-0.2139	0.085032	-0.27305
PSG11	-0.02275	-0.04122	-0.11276	-0.181	0.169095	0.197598	-0.3049	-0.22705
PSG2	-0.0588	0.608936	-0.0203	0.259871	-0.21186	-0.26832	0.058149	0.215617
PSG3	-0.08858	-0.04389	0.23847	0.294728	0.030852	0.041491	-0.07438	0.303911
PSG4	0.290657	0.016608	0.073292	-0.08945	0.113127	-0.13456	0.104341	-0.08105
PSG5	-0.1968	0.304025	-0.08909	-0.12031	0.157409	-0.11005	-0.08771	0.286086

PSG6	0.172056	0.180662	-0.0971	0.04757	0.26257	-0.01706	-0.04003	-0.18467
PSG7	-0.00636	-0.10881	0.070883	-0.0951	-0.31837	0.248719	0.091828	0.171763
PSG8	-0.23883	0.311486	-0.21252	-0.10262	0.14305	-0.07711	0.557744	0.447737
PSG9	0.08655	-0.01645	0.098191	-0.14494	0.046319	-0.26772	-0.07505	-0.10437
PSIP1	0.132054	-0.3328	-0.28513	0.18592	0.135294	0.327507	-0.09981	0.087373
PSKH1	0.229884	0.363902	-0.26014	0.150321	0.164647	-0.33646	-0.14395	-0.13
PSKH2	0.067511	0.572517	-0.16402	-0.27595	0.336465	0.074322	-0.66161	-0.24195
PSMA1	-0.01779	-0.70609	0.017548	0.002849	-0.01806	-0.27482	-0.21383	-0.36742
PSMA2	0.384961	-0.53218	-0.18947	-0.12295	-0.09373	-0.2053	-0.10557	-0.04724
PSMA3	0.084553	-0.65272	-0.32248	-0.08978	-0.32216	-0.18435	-0.60978	-0.45573
PSMA4	0.013565	-0.63423	-0.03104	-0.11027	-0.15376	0.211123	-0.60759	-0.45444
PSMA5	0.081459	-0.26573	0.232079	-0.05204	0.234239	-0.08329	0.214959	0.115082
PSMA6	-0.1771	-0.66095	-0.19881	-0.27291	-0.17688	-0.29414	-0.12418	0.273084
PSMA7	0.273297	-0.60563	0.128459	0.281087	0.187809	0.029973	NA	NA
PSMA8	0.296643	0.629615	0.24315	0.420721	-0.02751	-0.10704	-0.33638	-0.03141
PSMAL	-0.25691	0.098518	-0.15359	-0.04836	-0.21716	-0.06548	-0.53581	0.170333
PSMB1	0.348273	-0.44917	-0.30393	-0.06559	0.241429	0.180491	-0.54737	-0.32248
PSMB10	0.453534	0.258002	0.383549	0.030506	0.125648	-0.04762	-0.36097	-0.16763
PSMB2	-0.04473	-0.31662	0.266275	0.200776	-0.06376	-0.25751	0.259009	0.217526
PSMB3	0.255461	0.399087	0.18381	-0.06574	-0.32404	-0.29395	0.222338	0.128089
PSMB4	-0.12285	-0.33168	-0.14581	0.118523	-0.07144	-0.23254	0.302571	0.020934
PSMB5	0.015353	-0.49219	-0.04811	0.073408	0.034768	-0.02954	-0.17683	-0.26632
PSMB6	-0.21572	-0.58064	0.07448	0.209198	-0.27095	-0.22053	-0.04726	-0.00648
PSMB7	0.114318	-0.44578	-0.01794	-0.04141	-0.03455	-0.38404	0.26018	0.107328
PSMB8	0.396533	-0.20409	0.226363	0.089389	-0.00636	0.105343	-0.06159	0.030969
PSMB9	0.154272	0.019341	0.391472	-0.19207	-0.05657	0.272906	0.05034	0.157749
PSMC1	0.045538	0.502844	-0.45093	-0.17229	-0.10952	-0.31542	-0.19451	0.101155
PSMC2	0.128421	-0.48954	-0.08724	-0.04052	0.122003	0.115397	-0.02413	-0.04389
PSMC3	0.127089	0.457425	0.159456	0.233358	0.016241	-0.00794	-0.5825	-0.40644
PSMC3IP	-0.11905	0.509306	-0.07137	0.313301	0.215994	-0.02337	0.139311	0.014851
PSMC4	-0.25752	-0.64587	0.14229	0.101333	-0.01529	0.006397	0.38049	-0.03475
PSMC5	-0.26225	-0.25137	-0.03016	0.400197	0.040197	0.030425	-0.70561	-0.57985
PSMC6	-0.08974	-0.74292	-0.43828	-0.11822	-0.22958	-0.16974	-0.59825	-0.46796
PSMD1	-0.00646	-0.54558	-0.17538	-0.03481	-0.07383	0.017422	0.275286	0.415518
PSMD10	0.011374	-0.58526	-0.2701	0.014122	-0.21246	-0.4711	0.250099	0.000848
PSMD11	-0.03593	-0.4898	0.437213	0.296724	0.192018	-0.01569	-0.26859	-0.05705
PSMD12	NA	NA	-0.08971	-0.09612	-0.15829	0.049323	0.196116	0.144604
PSMD13	0.0216	0.025724	0.219005	0.144165	0.067336	0.202037	-0.25189	-0.45216
PSMD14	0.0881	-0.61341	0.125767	-0.01144	-0.03227	0.205033	-0.21395	-0.36768
PSMD2	-0.15917	-0.03216	0.131566	0.303962	0.221725	0.009291	-0.00195	-0.25508
PSMD3	0.144789	-0.13791	0.101527	0.348481	0.232928	0.123097	-0.07558	-0.0894
PSMD4	-0.42441	-0.55929	-0.05518	0.336382	-0.18777	-0.03088	0.433377	0.08625
PSMD5	0.181947	0.642729	-0.14062	-0.17994	-0.06144	-0.00556	0.129523	-0.16066
PSMD6	-0.0253	-0.52391	-0.2095	-0.00445	0.129282	0.078845	-0.0282	-0.04981
PSMD7	0.082566	-0.56936	-0.09083	-0.47352	0.239046	-0.12128	0.554851	0.359612
PSMD8	-0.23986	0.396065	-0.23773	0.094711	-0.1618	-0.19304	-0.49496	-0.23298
PSMD9	-0.29246	-0.23972	-0.17352	0.282247	0.163735	-0.23486	0.002747	0.479174
PSME1	0.293057	-0.36456	-0.06741	0.300789	-0.07437	0.053198	-0.24413	0.149045
PSME2	0.037098	-0.36881	-0.17888	0.120093	-0.20057	-0.10429	-0.5769	-0.09861
PSME3	0.388386	0.304464	-0.04766	0.210542	-0.10579	0.006945	0.0664	0.122618
PSME4	0.015111	-0.14851	-0.04724	-0.01266	-0.00546	0.140707	-0.34081	-0.13885
PSMF1	0.19905	0.091868	-0.07374	0.388045	0.105076	0.186286	0.186445	-0.04486

PSMG1	NA	NA	-0.03291	0.176595	0.055721	0.148912	NA	NA
PSMG2	NA	NA	-0.04855	-0.14815	-0.23796	-0.06692	NA	NA
PSMG3	NA	NA	-0.09658	0.322176	0.172909	-0.03012	NA	NA
PSORS1C1	-0.03977	0.562666	-0.04695	-0.19041	0.1096	-0.32698	-0.39104	-0.47091
PSORS1C2	0.362319	0.648594	-0.11105	0.077739	-0.15378	0.126901	-0.06861	-0.01323
PSPC1	-0.01606	-0.53538	-0.09404	0.089591	0.108363	0.059992	0.028009	-0.16257
PSPH	0.138369	-0.46233	0.074608	0.181237	-0.00166	-0.08755	0.151577	-0.08616
PSPN	0.429563	0.434883	0.365632	-0.08826	0.018391	-0.20054	-0.27532	-0.09068
PSRC1	-0.1778	0.086804	0.032702	0.213744	0.126284	-0.24565	0.273278	-0.10968
PSTK	NA	NA	-0.28444	0.137439	-0.11814	-0.42823	NA	NA
PSTPIP1	-0.13504	0.446958	0.208487	0.283671	-0.17051	-0.14659	-0.3648	0.48373
PSTPIP2	0.164601	-0.41802	0.09734	0.098812	0.085818	0.065695	-0.15554	-0.20358
PTAFR	-0.65336	-0.24684	0.265763	0.133746	-0.10957	0.658416	-0.64032	-0.01073
PTAR1	NA	NA	-0.16238	-0.21135	0.059934	0.127212	NA	NA
PTBP1	0.308473	0.537073	-0.27723	0.138912	0.1618	-0.05912	-0.00236	0.110554
PTBP2	-0.45735	-0.37781	-0.18013	-0.10991	0.191899	0.104181	-0.21899	-0.25743
PTCD1	0.318339	0.466639	-0.1244	0.261816	0.373701	0.136579	-0.02192	-0.05996
PTCD2	0.402676	-0.11233	0.056304	0.06361	-0.08026	0.126177	0.532883	0.353746
PTCD3	0.211529	-0.33307	-0.12468	-0.08844	0.153386	0.167868	NA	NA
PTCH1	0.304194	-0.24423	0.241444	0.314363	0.205988	0.077113	NA	NA
PTCH2	-0.33923	-0.20934	0.040452	-0.31673	0.15147	-0.17483	-0.49231	0.061734
PTCHD1	-0.38479	-0.01057	0.033079	-0.1032	-0.17478	0.142335	-0.23544	-0.29616
PTCHD2	NA	NA	0.004297	0.039508	0.078598	0.064534	NA	NA
PTCHD3	NA	NA	NA	NA	NA	NA	0.420615	0.462658
PTCRA	-0.31813	0.040619	0.126993	-0.12106	-0.17068	-0.31701	-0.21502	0.06571
PTDSS1	0.082419	0.067461	0.198044	0.454901	0.112942	0.213315	0.604879	0.479814
PTDSS2	0.268727	0.524041	-0.17268	0.058075	0.065815	-0.34724	-0.13104	-0.27357
PTEN	-0.30791	-0.39955	-0.2527	-0.05683	0.024534	-0.5406	0.301381	0.289341
PTER	-0.022	-0.27912	-0.11365	-0.19228	0.189765	0.062645	-0.16573	0.119322
PTF1A	0.146379	0.063811	0.298449	-0.21717	0.160269	-0.44747	0.198967	0.125029
PTGDR	0.076562	-0.05843	-0.02768	-0.14756	-0.27364	0.051521	-0.16911	0.072014
PTGDS	-0.28503	0.180464	-0.00429	0.757191	-0.0554	0.353663	-0.22261	0.060794
PTGER1	0.239338	0.328881	-0.17525	0.2787	-0.0215	-0.03664	-0.34252	0.167328
PTGER2	-0.51609	-0.32327	-0.11826	0.191758	-0.13041	-0.0711	0.27162	0.511971
PTGER3	0.21016	0.074277	0.125711	0.015176	-0.19371	0.296475	-0.15755	0.040604
PTGER4	-0.45532	-0.13232	-0.085	-0.0522	-0.18124	-0.10629	-0.37634	-0.25937
PTGES	0.073085	-0.08247	0.11499	0.289462	0.322859	0.02713	-0.1887	0.466366
PTGES2	0.125304	0.521638	0.394416	0.250015	0.16867	0.009497	0.315378	0.030073
PTGES3	0.102596	-0.06131	-0.427	0.196671	0.115923	0.04227	0.094016	0.087675
PTGFR	-0.2255	-0.02686	0.132382	-0.10153	-0.22547	0.172515	-0.5668	-0.39237
PTGFRN	0.102225	-0.4511	-0.21134	-0.08959	0.129992	-0.18369	-0.61884	-0.60752
PTGIR	0.038016	0.347468	0.248086	0.309498	0.187644	0.23002	-0.18069	-0.13788
PTGIS	0.317453	-0.02991	0.024293	0.138664	0.185318	-0.19747	-0.05011	-0.15844
PTGR1	NA	NA	0.152339	-0.09843	0.170579	0.152337	NA	NA
PTGR2	NA	NA	-0.40217	0.035214	-0.22344	-0.14712	NA	NA
PTGS1	-0.25967	-0.08595	0.066284	0.160754	-0.07735	0.337909	-0.47225	-0.31958
PTGS2	-0.14948	-0.31268	-0.10569	0.00535	0.098167	0.116641	-0.34399	-0.29678
PTH	-0.11697	0.591708	0.043819	-0.09705	-0.17456	0.007125	-0.54801	-0.16897
PTH1R	NA	NA	-0.13538	-0.04582	0.115251	0.010675	NA	NA
PTH2	NA	NA	-0.25738	-0.00255	-0.06615	-0.14844	NA	NA
PTH2R	NA	NA	0.090017	0.304532	-0.10416	-0.29665	NA	NA
PTHLH	0.061062	-0.30371	0.144048	-0.20513	-0.06038	0.119655	-0.1266	-0.13367

PTK2	0.017892	-0.22084	0.086697	-0.15115	-0.05947	-0.16129	0.466334	0.337246
PTK2B	0.019003	0.280931	0.383687	0.467643	0.003393	0.048292	0.061628	-0.15855
PTK6	0.222667	0.27252	0.263813	-0.08162	0.406515	-0.19243	0.211902	0.120369
PTK7	-0.04235	-0.26696	0.483939	0.245655	0.347048	0.292276	0.372498	0.064871
PTMA	0.088649	-0.59248	-0.28331	0.399634	-0.25434	0.215028	-0.74289	-0.55672
PTMS	0.125732	0.079625	0.005864	-0.05668	-0.0889	0.207022	0.300662	0.099747
PTN	0.06434	0.260949	0.107135	-0.18377	-0.44335	0.267994	-0.44543	-0.35059
PTOV1	0.14258	0.395671	0.241442	0.106144	0.31908	0.184793	0.428313	0.150609
PTP4A1	-0.01434	-0.29286	-0.48545	-0.02221	0.03217	-0.03887	-0.32818	-0.29535
PTP4A2	NA	NA	0.240791	-0.08102	-0.00622	-0.0554	-0.26577	-0.00071
PTP4A3	-0.05593	-0.00556	0.494123	-0.11477	0.130034	0.297564	-0.09594	-0.02752
PTPDC1	-0.05543	-0.20965	-0.05466	-0.22236	0.159914	-0.65764	0.094831	-0.07191
PTPLA	-0.04249	-0.24399	0.13452	-0.08842	-0.148	-0.17526	-0.20725	-0.20218
PTPLAD1	-0.0309	-0.40304	-0.12288	-0.0711	0.011782	0.103757	0.37335	0.256735
PTPLAD2	-0.26356	0.07705	0.021414	-0.22097	-0.40361	-0.12009	-0.65213	-0.60859
PTPLB	-0.27809	-0.48551	-0.45416	-0.11901	-0.14963	-0.21375	-0.01448	-0.11894
PTPMT1	NA	NA	0.017441	0.127137	-0.07274	-0.28375	NA	NA
PTPN1	0.271219	-0.3919	-0.18106	0.171563	-0.02929	0.190864	-0.28311	-0.06275
PTPN11	0.349454	0.017753	-0.27674	-0.23651	0.153394	-0.02215	-0.53089	-0.46687
PTPN12	-0.15526	-0.81533	-0.24338	-0.53925	-0.19035	0.067682	-0.30892	-0.25144
PTPN13	-0.11413	-0.47991	0.294011	-0.01299	-0.31096	0.163751	-0.11337	-0.17956
PTPN14	-0.19093	-0.68116	0.271584	-0.13096	-0.29595	-0.07339	0.483033	-0.02557
PTPN18	0.365086	0.669012	-0.1621	0.487126	0.154962	-0.05144	-0.04501	0.220399
PTPN2	0.071063	0.080978	-0.13114	0.044527	-0.1715	-0.16156	0.065292	0.329254
PTPN20B	-0.02883	-0.00979	-0.26941	-0.30761	-0.05223	0.123118	0.297536	-0.11281
PTPN21	-0.32868	-0.41626	-0.27215	-0.22116	-0.18122	-0.25799	-0.45127	-0.24257
PTPN22	-0.23217	0.050595	0.062358	0.195119	-0.33262	0.193898	-0.34682	0.380298
PTPN23	0.091486	0.684306	0.04747	0.140797	0.150728	0.290423	0.698564	0.372316
PTPN3	0.283718	0.075283	0.276582	0.043682	0.257323	-0.01599	0.259255	0.286061
PTPN4	0.120046	0.540516	-0.11196	0.041395	-0.035	0.0478	0.16782	0.276487
PTPN5	-0.2032	0.173417	0.24999	-0.21675	0.292862	0.230419	-0.12858	-0.03771
PTPN6	0.165182	0.19347	0.491662	0.677481	-0.06374	0.469979	0.046469	0.431823
PTPN7	-0.08478	0.516561	0.26155	0.734093	-0.02172	0.731667	0.078324	0.545336
PTPN9	0.235295	0.654297	0.299682	0.02288	0.132224	-0.14242	0.528232	0.508442
PTPRA	0.137598	-0.34178	-0.3268	0.172071	-0.02151	0.039247	0.090309	-0.56677
PTPRB	-0.37984	-0.74275	-0.20919	-0.50635	-0.14871	-0.21643	0.299008	0.233048
PTPRC	-0.51696	0.243624	0.093867	0.651162	-0.21213	0.723848	-0.61282	-0.21081
PTPRCAP	-0.14854	0.472591	0.35113	0.857175	-0.16003	0.419968	-0.2481	0.262153
PTPRD	0.325003	-0.13377	-0.25045	-0.24256	-0.07743	-0.02271	0.099315	0.336464
PTPRE	-0.56975	-0.38119	0.22267	-0.11605	-0.10992	0.080549	-0.08743	0.428993
PTPRF	-0.17057	0.441575	0.212315	-0.00558	0.473833	0.009179	0.142037	-0.04147
PTPRG	0.058567	-0.41235	-0.2185	-0.29247	0.077848	0.132499	-0.27638	-0.2375
PTPRH	0.150254	0.115585	-0.0031	-0.10881	0.109508	-0.01173	0.01446	0.192089
PTPRJ	0.468666	0.773315	-0.1563	0.054554	0.095263	-0.16378	NA	NA
PTPRK	0.424283	-0.30646	-0.18677	-0.17095	0.080767	0.113339	0.41602	0.330748
PTPRM	-0.45424	-0.61686	0.053227	-0.03795	-0.02432	0.242881	-0.18255	0.110618
PTPRN	0.022711	0.28567	0.453758	0.352828	0.105104	0.043187	-0.20709	-0.10683
PTPRN2	0.081824	-0.23092	0.158739	0.149342	0.363507	-0.38357	0.211543	-0.12557
PTPRO	0.370269	-0.09893	-0.03391	0.100007	0.075641	-0.07663	0.055217	-0.08893
PTPRR	0.156474	0.037532	-0.09596	-0.18197	-0.1021	-0.27262	0.31851	0.101859
PTPRS	-0.32349	-0.02492	0.26524	0.098943	0.200111	-0.12437	0.50603	0.217291
PTPRT	-0.20277	0.14226	0.252907	0.299934	0.064682	0.148999	0.02063	0.075817

PTPRU	-0.13097	-0.26025	0.128518	0.13121	0.072153	0.089876	-0.10936	-0.07837
PTPRZ1	0.112635	-0.41076	0.114616	-0.13711	0.026704	-0.04654	-0.30528	-0.18098
PTRF	-0.19647	0.167486	0.11284	0.052767	0.040362	0.182037	-0.40538	-0.41221
PTRH1	0.354786	0.444664	0.257243	0.345916	0.140084	-0.16192	0.26246	-0.06995
PTRH2	-0.00598	0.012368	0.074679	-0.28989	-0.08468	0.103313	0.445328	0.391355
PTS	-0.23029	-0.62034	-0.19539	-0.08386	-0.14946	-0.4381	-0.66761	-0.49003
PTTG1	-0.16845	-0.08553	0.092983	0.070383	-0.06486	-0.08195	0.236404	0.002048
PTTG1IP	-0.33506	-0.68816	0.153346	-0.10598	-0.20292	-0.04003	-0.43815	-0.31159
PTTG2	-0.28069	-0.1198	0.174264	0.079089	-0.12184	-0.20172	-0.63594	-0.46027
PTX3	0.056466	0.206874	0.058533	-0.01842	-0.00247	0.224277	-0.03014	0.147866
PUF60	NA	NA	0.212679	0.403188	0.041736	0.334167	NA	NA
PUM1	-0.09778	-0.60554	-0.03664	0.041105	0.181217	0.342634	0.497229	0.133066
PUM2	0.30825	-0.42354	-0.11346	-0.27388	-0.02566	0.144414	0.602291	0.383104
PURA	-0.05968	-0.63648	0.44829	-0.1003	0.348678	0.469099	0.128024	-0.11604
PURB	-0.48283	0.054327	-0.2803	-0.3619	0.037828	0.335939	0.222924	0.135734
PURG	0.232274	0.772535	-0.10311	0.116535	0.01872	0.204258	0.472955	0.316414
PUS1	0.080924	0.505825	-0.0751	0.248314	0.432492	0.527074	0.375598	0.41374
PUS10	NA	NA	-0.29096	-0.09725	0.011892	-0.51138	NA	NA
PUS3	0.260987	0.230499	-0.05646	-0.25243	-0.19619	-0.2519	-0.32935	-0.19029
PUS7	0.382773	0.064831	-0.26249	0.025479	0.27314	0.290448	0.311502	0.100217
PUS7L	0.566169	-0.29822	-0.29402	-0.12075	-0.02154	0.074955	0.222786	0.012211
PUSL1	-0.20081	-0.08141	0.073505	0.119161	-0.01661	-0.31284	0.494113	0.295128
PVALB	-0.15469	-0.26362	-0.15281	0.209778	-0.13445	0.097526	-0.08487	0.333308
PVR	0.263246	0.361127	0.192465	0.261479	0.421395	0.189329	0.003507	-0.21811
PVRIG	-0.06626	-0.12255	0.142685	0.741383	-0.04795	0.666918	NA	NA
PVRL1	-0.02254	0.463722	0.131587	0.384858	0.242211	0.028126	0.365962	0.031819
PVRL2	-0.15831	0.138739	-0.03016	0.027065	0.101877	0.080128	0.540021	0.375219
PVRL3	-0.08267	-0.0077	0.242233	-0.14377	-0.21068	-0.17872	0.010811	-0.24795
PVRL4	-0.3892	0.063606	0.36442	0.314382	-0.01143	0.255276	0.029429	-0.10395
PWP1	-0.08843	-0.36094	-0.27922	-0.42202	0.164179	-0.1879	0.373548	0.251996
PWP2	0.10003	-0.28627	0.033948	0.27039	0.017181	0.50724	NA	NA
PWWP2A	NA	NA	0.222117	0.104099	0.09897	0.069343	NA	NA
PWWP2B	NA	NA	0.158965	0.08067	0.174424	-0.03093	NA	NA
PXDN	NA	NA	0.046979	-0.20232	0.140171	0.26984	-0.4781	-0.44286
PXDNL	0.290932	0.114616	-0.10928	0.227885	-0.31203	0.322315	-0.14994	0.323871
PXK	-0.1293	-0.53239	0.04689	0.378079	0.14547	0.383732	0.344807	0.386535
PXMP2	-0.33506	0.081663	0.068849	-0.18122	0.219629	-0.31328	0.392542	0.147775
PXMP3	-0.11574	-0.52068	0.367335	-0.08553	-0.33139	-0.15522	0.527738	0.158793
PXMP4	0.350395	0.45831	0.317544	-0.11936	-0.01598	-0.68134	0.039001	-0.16854
PXN	-0.19139	-0.07833	-0.06635	-0.22426	0.272896	0.450488	-0.00574	0.156451
PXT1	0.071229	0.642393	-0.37249	0.17026	0.160627	-0.25483	-0.14171	0.0117
PYCARD	0.063717	0.026662	0.427106	-0.16425	0.083733	0.15559	0.281373	0.060323
PYCR1	-0.20195	0.398541	0.334639	0.241268	0.372293	-0.19476	0.083209	0.045121
PYCR2	-0.00767	-0.62255	-0.14193	0.56963	0.233594	0.157971	0.165085	-0.23295
PYCRL	0.107764	0.18147	0.290443	0.191565	-0.06143	0.050372	-0.00801	-0.14731
PYDC1	0.387721	0.197157	0.290789	-0.2057	0.108917	-0.0437	0.125312	0.429471
PYGB	-0.37237	-0.18521	0.105106	0.0075	0.243951	-0.06096	0.076698	-0.35677
PYGL	0.064509	-0.31446	-0.30296	-0.09078	-0.25219	0.408091	-0.53448	-0.48788
PYGM	0.279265	0.293864	-0.18776	-0.14251	-0.18477	0.111702	-0.38663	-0.21548
PYGO1	-0.19509	-0.26445	-0.06667	-0.00906	-0.04836	-0.01699	0.023021	-0.2881
PYGO2	0.189366	0.335878	0.076772	0.484004	0.008112	0.309864	0.485567	0.271328
PYHIN1	-0.13254	0.053937	0.207367	0.629769	-0.20956	0.709919	-0.38013	0.397586

PYROXD1	NA	NA	-0.27618	-0.09101	-0.24488	-0.27999	NA	NA
PYY	-0.12368	0.267374	0.044715	0.012613	-0.13289	-0.05308	0.102981	0.129242
PYY2	-0.0153	0.096611	0.157227	-0.04021	-0.2295	-0.21942	-0.26966	-0.42462
PZP	-0.06914	-0.32487	-0.141	0.183724	0.167612	0.057641	-0.61964	-0.07531
QARS	0.299699	0.607501	0.254504	0.149129	0.337965	-0.03893	0.045497	-0.29886
QDPR	-0.14245	-0.38483	-0.02364	0.015482	0.140674	-0.42895	0.55191	0.188452
QKI	-0.43024	-0.54605	-0.0184	-0.02627	-0.20392	0.381169	-0.49993	-0.38404
QPCT	0.392715	-0.26425	0.248732	0.066528	0.025318	-0.33058	-0.17061	-0.38576
QPCTL	0.06081	0.465725	0.048888	-0.05897	0.359651	-0.08048	0.403164	0.248882
QPRT	0.4206	-0.15436	0.357286	-0.18578	0.249276	-0.15424	0.312677	-0.06543
QRFPR	NA	NA	-0.13068	0.058941	0.320728	-0.4052	NA	NA
QRICH1	0.083405	0.622687	-0.29096	0.176058	0.098367	0.06752	0.352854	0.128012
QRICH2	0.29637	0.546435	0.01467	-0.17325	0.462517	0.063984	0.414553	0.392294
QRSL1	0.323572	-0.23957	-0.04675	0.238769	0.256129	0.03438	0.165019	0.143144
QSER1	0.262846	0.609568	-0.35905	-0.08933	-0.14347	-0.35596	-0.35771	0.031081
QSOX1	NA	NA	0.012253	0.307509	0.063408	-0.20348	NA	NA
QSOX2	NA	NA	-0.01913	0.410853	0.349055	0.46344	NA	NA
QTRT1	0.215346	-0.35209	-0.0089	0.357965	0.26886	0.240289	0.181834	-0.11181
QTRTD1	0.034256	-0.62074	-0.44529	0.207008	0.213125	0.142531	-0.09694	-0.29068
R3HCC1	NA	NA	0.423408	-0.00125	0.076969	-0.00578	NA	NA
R3HDM1	0.083892	0.298166	-0.18977	0.239075	0.11482	-0.12996	-0.38539	-0.36725
R3HDM2	0.30466	0.595986	0.052192	0.042194	0.489225	-0.11285	-0.11898	0.161985
R3HDML	-0.00189	0.613632	-0.11675	0.05396	0.190334	-0.437	-0.15814	-0.39506
RAB10	0.412722	0.206183	-0.15746	-0.08901	-0.04705	-0.21908	-0.06642	-0.14408
RAB11A	-0.22685	-0.44276	-0.10472	0.049556	-0.17377	-0.17604	0.265101	0.105439
RAB11B	0.432561	0.004459	-0.00423	-0.05556	-0.00938	0.02322	0.22284	0.084168
RAB11FIP1	0.268378	-0.01581	0.013067	-0.31287	0.144147	-0.08019	0.209106	-0.08549
RAB11FIP2	0.090082	-0.56531	-0.02216	-0.08134	0.11398	0.045906	0.1499	-0.15388
RAB11FIP3	0.142813	0.086309	0.266337	0.062449	0.497384	0.442398	0.040983	0.107129
RAB11FIP4	0.156062	0.461658	0.40934	0.187725	0.407071	0.109219	0.351519	0.423756
RAB11FIP5	0.010826	0.328917	0.293733	-0.05767	0.171592	0.2792	-0.17017	-0.46027
RAB12	-0.34371	-0.53142	-0.2374	-0.0774	-0.09361	0.08181	-0.04309	0.161354
RAB13	-0.35873	0.024877	0.258452	0.025567	-0.15763	-0.079	-0.51562	-0.6045
RAB14	-0.18715	-0.31996	-0.10665	-0.26217	-0.02015	-0.1097	-0.47784	-0.26875
RAB15	-0.35275	-0.14335	-0.09119	0.083031	0.233482	0.170428	-0.25522	-0.38749
RAB17	0.514301	-0.09331	0.242914	-0.12522	0.29263	-0.29233	0.335851	0.172956
RAB18	-0.07937	-0.56944	-0.04541	-0.4606	-0.44824	-0.19184	-0.00343	0.104031
RAB1A	-0.05172	-0.25249	-0.09227	-0.05958	-0.15583	0.019459	-0.09969	-0.16527
RAB1B	0.146516	-0.27521	0.241548	0.034456	-0.05782	-0.18966	0.376723	0.189403
RAB20	0.108072	0.089487	0.324353	0.022801	0.328579	0.295074	0.091972	-0.03552
RAB21	-0.02764	-0.49863	-0.06779	0.020548	0.047365	-0.03312	-0.35309	-0.11787
RAB22A	0.20995	-0.65519	0.031309	-0.14578	0.219065	0.1559	0.304996	-0.05755
RAB23	0.048884	-0.04587	0.24267	-0.20755	-0.18785	-0.02682	-0.30318	-0.2518
RAB24	-0.00644	0.057302	0.449408	0.21677	0.138355	0.186713	-0.43437	0.142066
RAB25	0.180798	0.189249	0.277042	0.163056	-0.11508	-0.08072	0.515122	0.094016
RAB26	-0.37755	0.265194	-0.02351	0.003814	-0.07639	-0.20906	0.335364	0.364361
RAB27A	-0.4539	-0.16678	-0.25035	-0.05675	-0.28694	-0.35439	-0.50638	-0.31394
RAB27B	-0.47154	0.055132	0.104228	0.024172	-0.46671	-0.46097	-0.34972	-0.13164
RAB28	-0.01258	-0.20732	-0.08827	-0.0028	-0.03308	-0.50213	0.361972	0.44149
RAB2A	-0.0804	-0.03949	0.078054	-0.19538	-0.28031	-0.42507	NA	NA
RAB2B	-0.02469	-0.28112	-0.29201	-0.36002	-0.15886	0.004786	-0.43514	-0.06976
RAB30	0.01525	0.174315	0.072802	0.155557	-0.06345	0.065758	0.251248	0.109135

RAB31	-0.32522	-0.15839	0.16656	-0.12148	-0.24905	0.435429	-0.16301	-0.10755
RAB32	0.376113	0.105405	0.051891	0.181282	-0.09409	-0.14746	-0.00245	-0.05527
RAB33A	-0.12591	0.021689	0.146861	0.471078	-0.25875	0.283253	-0.18201	0.152728
RAB33B	-0.32868	0.049249	-0.3578	0.319429	-0.19428	0.147877	-0.39654	-0.10533
RAB34	-0.23955	-0.1577	0.172757	0.01633	-0.13107	0.158787	-0.36391	-0.24603
RAB35	-0.35229	0.414261	-0.22871	0.044967	0.204851	0.194184	-0.61756	-0.42875
RAB36	0.393706	0.283871	0.059515	0.074916	0.405376	0.281827	0.088058	-0.28296
RAB37	0.373184	-0.13173	0.155218	-0.12165	-0.09739	-0.08427	-0.34463	0.152938
RAB38	0.040447	0.255309	-0.27995	-0.06539	-0.57309	0.062167	-0.2311	0.166084
RAB39	0.141226	-0.06403	0.084209	-0.29759	-0.29522	-0.0753	-0.43949	-0.13892
RAB39B	0.402259	0.508195	0.231174	0.564736	-0.26303	-0.13125	0.260419	0.116396
RAB3A	0.400266	0.086797	0.051423	0.114557	0.077998	-0.42876	-0.02768	-0.32271
RAB3B	-0.36624	0.255928	-0.00304	-0.0272	-0.19733	-0.33734	0.009015	-0.11651
RAB3C	0.221388	-0.27643	-0.06115	0.309706	-0.09542	-0.18377	-0.29163	0.041571
RAB3D	0.22448	0.152078	-0.30849	0.250392	0.048175	-0.29875	0.398021	-0.17758
RAB3GAP1	0.297196	-0.13247	-0.03794	-0.21225	-0.40006	0.141502	-0.22024	-0.25457
RAB3GAP2	-0.16655	-0.53459	-0.10611	-0.18986	-0.02406	0.243871	NA	NA
RAB3IL1	-0.04911	0.061325	0.171171	0.032495	-0.22285	-0.02293	-0.21289	0.090469
RAB3IP	0.052818	0.182933	0.077206	-0.07698	0.310769	0.192293	0.564284	0.06058
RAB40A	-0.06913	-0.18675	NA	NA	NA	NA	0.048466	-0.06759
RAB40B	-0.22633	-0.14717	0.166256	0.013812	0.211717	0.110898	0.050862	-0.38187
RAB40C	0.113232	0.160487	0.116167	-0.02582	0.320967	-0.21605	0.364225	0.090402
RAB41	0.297881	0.706207	NA	NA	NA	NA	-0.20873	-0.30478
RAB42	0.113979	0.646468	0.170937	-0.08646	-0.23468	0.21453	0.589806	0.519389
RAB43	-0.20341	0.615171	-0.305	0.0005	0.025725	-0.13277	0.305835	0.292546
RAB4A	0.174227	-0.62402	-0.30371	-0.14071	-0.06303	-0.51745	0.673831	0.04596
RAB4B	0.236562	0.606936	0.128091	0.138991	-0.09513	-0.36743	0.132756	0.202567
RAB5A	-0.16929	-0.52581	-0.53821	-0.07801	-0.20423	-0.29572	0.223273	0.177758
RAB5B	0.03615	-0.10515	-0.05528	0.094013	0.027056	-0.12992	-0.05932	0.196464
RAB5C	0.156298	-0.04805	0.32182	0.086468	0.071169	-0.25888	-0.09246	-0.13452
RAB6A	0.117208	0.006655	0.112875	-0.22017	0.139294	-0.44639	-0.17194	-0.44851
RAB6B	-0.16698	0.563559	-0.04251	-0.27745	-0.10055	0.026464	-0.40252	-0.3566
RAB6C	NA	NA	0.049002	-0.0551	0.176712	-0.14863	0.187329	-0.02657
RAB7A	0.182842	0.19046	0.053323	-0.00557	-0.23211	-0.0619	NA	NA
RAB7B	-0.04294	0.142907	0.106813	-0.12038	-0.42529	0.246778	-0.40324	0.260621
RAB7L1	-0.21244	-0.15678	0.242646	0.191601	-0.01367	0.357875	0.037705	0.428491
RAB8A	0.255229	0.271575	-0.21156	0.134818	-0.00406	-0.13668	0.180837	0.514517
RAB8B	0.281424	0.702747	-0.00212	0.285893	-0.25841	0.463477	-0.57702	-0.06274
RAB9A	0.025856	-0.4169	0.050312	-0.15803	0.005735	-0.12909	-0.09931	-0.35572
RAB9B	0.273011	-0.07464	-0.39379	-0.16671	-0.16434	-0.12117	-0.35047	-0.34633
RABAC1	-0.2315	-0.66564	-0.01119	-0.09198	-0.21715	-0.40575	0.066846	0.012226
RABEP1	-0.29701	-0.57313	-0.36789	-0.28175	-0.31309	0.11722	0.332146	0.212553
RABEP2	0.408999	0.295414	0.24015	0.284331	0.460954	0.199961	0.320473	-0.29985
RABEPK	0.159877	-0.16356	0.113773	0.252794	-0.02884	-0.32716	-0.50428	-0.4753
RABGAP1	-0.16985	0.032929	0.295574	-0.43294	0.038885	0.208691	-0.12718	-0.32185
RABGAP1L	-0.18396	-0.37745	0.166952	0.026415	-0.29361	-0.0572	NA	NA
RABGEF1	0.240059	-0.21544	-0.26702	-0.14109	0.000212	0.07637	-0.52329	-0.32669
RABGGTA	-0.15274	0.628558	0.328087	0.054113	0.14641	-0.19133	0.465995	0.324681
RABGGTB	-0.0976	-0.56884	0.397699	0.11437	0.194406	-0.07691	-0.37587	-0.16786
RABIF	0.216942	0.311286	-0.01016	-0.2089	-0.36512	-0.43469	-0.3247	-0.24496
RABL2A	0.017487	-0.18271	-0.11538	0.203351	0.194801	-0.20852	0.193833	0.185451
RABL2B	-0.37418	0.248525	NA	NA	NA	NA	-0.18045	-0.23136

RABL3	-0.25263	-0.51697	-0.18766	-0.30018	-0.34303	-0.36781	0.167965	0.413283
RABL4	0.017075	0.182322	0.02708	0.091873	-0.01609	-0.10648	-0.32763	-0.28023
RABL5	0.159455	-0.15903	-0.0607	-0.20303	0.059593	-0.39288	-0.52256	-0.49081
RAC1	0.197074	-0.18994	0.106166	-0.1281	0.310675	0.390651	0.246192	-0.10963
RAC2	-0.22195	0.136343	0.479869	0.868598	-0.07395	0.553304	-0.59493	-0.08266
RAC3	-0.07415	0.147715	0.111632	-0.22977	0.179384	-0.37421	0.558462	0.467738
RACGAP1	0.190425	0.518183	-0.47343	-0.01864	-0.02839	-0.28147	0.30074	-0.03668
RAD1	0.115613	-0.06235	-0.14152	-0.12107	-0.09265	-0.23613	-0.33235	-0.26956
RAD17	0.211915	0.695726	0.224803	-0.02099	-0.14032	-0.26169	0.500819	0.344209
RAD18	0.310243	-0.05757	-0.3905	-0.29519	0.197029	-0.42406	0.180201	0.245127
RAD21	0.020982	0.306085	-0.12256	-0.14478	-0.04175	-0.03781	-0.22682	-0.30687
RAD21L1	NA	NA	-0.17135	-0.11013	0.140827	0.168829	NA	NA
RAD23A	-0.10598	-0.13405	0.304663	0.073066	0.349831	0.208956	0.197946	0.072134
RAD23B	-0.23757	-0.45842	0.03222	-0.18887	0.262683	0.027052	0.26787	0.33229
RAD50	0.385564	0.323667	0.309309	0.315492	0.207942	0.076069	0.085521	-0.02781
RAD51	-0.21145	-0.22203	-0.37993	0.403609	-0.16353	-0.34076	-0.63677	-0.50363
RAD51AP1	0.058209	-0.28867	-0.40598	0.070555	-0.1623	-0.07053	0.275832	0.156486
RAD51C	-0.05437	-0.39653	0.175678	0.10868	-0.01801	-0.13492	0.034064	-0.17637
RAD51L1	0.190451	-0.42414	-0.11079	-0.25782	0.038772	-0.01754	-0.20257	-0.37747
RAD51L3	0.364356	-0.39565	0.127347	0.356422	0.191586	-0.05694	0.430919	0.483287
RAD52	0.060738	0.496793	-0.14641	-0.14303	-0.07875	-0.34564	0.248586	0.165187
RAD54B	0.04465	-0.08065	-0.20618	0.005787	-0.17217	-0.06791	0.395849	0.302771
RAD54L	-0.08957	-0.17567	-0.12505	0.436759	0.332366	-0.19309	0.049156	-0.18447
RAD54L2	-0.00252	-0.2963	0.099908	0.232697	0.252896	0.548678	NA	NA
RAD9A	-0.21722	0.256409	0.000424	-0.18122	0.00136	0.024895	0.373377	0.377986
RAD9B	0.217457	0.584916	-0.3955	0.27315	0.169483	-0.14323	6.8E-05	-0.02957
RADIL	NA	NA	-0.22058	0.043425	-0.0944	-0.11106	NA	NA
RAE1	0.384515	0.371733	-0.08503	0.141002	0.204321	0.012775	0.533634	0.403948
RAET1E	0.096258	0.147674	0.1304	-0.13224	-0.18378	-0.42669	-0.31076	-0.03963
RAET1G	-0.05605	-0.14486	NA	NA	NA	NA	0.122229	0.098444
RAET1L	NA	NA	NA	NA	NA	NA	0.349488	0.174471
RAF1	0.018861	0.152125	-0.02039	0.114447	0.163357	0.161247	-0.15289	0.18493
RAG1	0.110181	-0.34807	-0.17078	-0.43648	-0.12937	-0.24822	-0.27518	-0.08732
RAG1AP1	-0.53472	-0.05505	-0.01741	0.170436	-0.21847	-0.24786	-0.42381	-0.37927
RAG2	0.525843	0.316865	-0.11356	0.056806	0.158231	-0.14896	0.298099	0.127482
RAGE	-0.2689	0.368467	-0.17862	-0.15322	-0.01421	-0.36043	0.121081	0.028599
RAI1	-0.02457	-0.09836	-0.02498	0.111594	0.045315	0.448564	0.038756	-0.16918
RAI14	-0.02577	-0.0665	0.028978	-0.11568	0.058489	0.207534	-0.10664	-0.18195
RAI2	0.298011	-0.13419	0.278918	0.141415	0.113072	0.205161	NA	NA
RALA	0.095657	0.641143	0.012691	-0.01814	-0.0219	-0.34181	0.152303	0.070349
RALB	-0.10796	-0.57224	-0.08888	0.107565	-0.17673	-0.24021	0.567625	0.238408
RALBP1	0.240975	0.22631	-0.10942	0.169065	-0.16189	-0.17957	-0.70217	-0.55325
RALGDS	-0.41255	-0.02674	-0.01683	0.113086	0.161463	0.214485	-0.49048	0.063358
RALGPS1	0.194497	0.292192	0.302639	-0.30963	0.3451	0.026515	0.035119	0.213006
RALGPS2	-0.14294	0.237797	-0.01382	-0.1447	-0.31879	0.300549	0.05784	0.182084
RALY	0.46438	0.490426	0.357055	-0.02643	0.395731	-0.04017	0.549655	0.212692
RALYL	NA	NA	-0.10948	-0.12674	0.293766	-0.23632	NA	NA
RAMP1	0.018735	0.133681	0.111088	0.019337	0.034001	-0.08706	-0.33706	-0.23872
RAMP2	-0.15283	0.0378	-0.15243	-0.00636	-0.15379	-0.07248	-0.02199	0.187402
RAMP3	-0.19575	0.152757	0.422208	0.285108	-0.05241	0.074261	-0.31173	-0.06599
RAN	0.042814	-0.34485	-0.25066	0.038765	0.195659	-0.05637	0.219121	-0.00158
RANBP1	0.075967	-0.56018	-0.28848	0.273764	0.283376	-0.23899	0.156226	-0.23745

RANBP10	0.510302	-0.09982	0.219443	0.080746	0.474963	-0.20545	0.25013	0.028901
RANBP17	-0.13392	0.608167	-0.37791	-0.15822	0.151432	-0.03156	0.163496	-0.49229
RANBP2	0.208472	-0.46074	-0.09168	-0.15736	-0.03808	0.030774	0.139791	-0.06603
RANBP3	0.266533	-0.0564	-0.37332	0.374568	0.186511	-0.04145	0.437368	0.09053
RANBP3L	0.144694	0.368417	0.002804	-0.23143	0.199466	-0.1343	-0.27767	-0.12316
RANBP6	-0.1333	-0.5252	-0.32183	-0.20943	0.222374	0.261533	-0.21719	-0.34944
RANBP9	0.100494	-0.31683	-0.0347	-0.24361	0.206506	-0.05703	-0.32416	-0.19977
RANGAP1	-0.12729	0.491439	0.123012	0.340443	0.403627	0.049816	0.31069	0.076057
RANGRF	NA	NA	-0.164	0.232355	-0.10758	-0.27939	NA	NA
RAP1A	-0.42783	-0.38881	-0.0092	-0.03412	-0.04509	-0.19532	-0.13532	-0.15262
RAP1B	-0.01399	0.119934	-0.31109	-0.26582	-0.15661	-0.02963	-0.73558	-0.40642
RAP1GAP	-0.28787	0.33502	-0.06085	0.270612	0.179529	-0.28598	NA	NA
RAP1GDS1	-0.11489	-0.24533	-0.24782	0.095594	0.082089	0.074468	0.371691	-0.02105
RAP2A	0.064199	-0.64594	-0.09358	-0.05159	0.305153	-0.19922	0.528067	0.258139
RAP2B	-0.34453	0.603712	-0.13861	-0.23428	0.247073	-0.19242	-0.17087	0.057327
RAP2C	0.07276	-0.32731	-0.35228	-0.18219	-0.15754	-0.32352	-0.27201	-0.13658
RAPGEF1	-0.3394	0.516403	0.322492	0.298257	0.341463	0.188456	-0.37689	0.157991
RAPGEF2	NA	NA	-0.00582	-0.60463	0.112604	0.220365	NA	NA
RAPGEF3	-0.28335	0.387598	-0.02368	0.076477	-0.08113	0.170949	-0.18569	0.398407
RAPGEF4	-0.02478	-0.58849	-0.02759	0.01264	-0.16459	0.04076	-0.10792	0.012174
RAPGEF5	NA	NA	-0.03777	-0.25753	0.026194	-0.1571	NA	NA
RAPGEF6	0.208206	0.291844	-0.14838	0.033073	0.088806	0.153724	0.289111	0.186843
RAPGEFL1	-0.17252	-0.34196	0.063659	-0.00645	-0.0215	-0.20939	0.239359	-0.00205
RAPH1	-0.15006	-0.13819	-0.07671	-0.29655	0.187859	0.109721	0.293843	0.070361
RAPSN	0.338711	0.765047	0.071207	-0.22305	-0.12206	-0.11017	-0.0654	-0.28408
RARA	0.001796	0.475644	0.272396	-0.17022	0.07078	-0.04451	-0.50442	0.044193
RARB	-0.25172	-0.18146	0.085525	-0.0494	-0.10645	0.094342	-0.05292	0.097833
RARG	0.348797	0.223717	0.215388	0.058659	0.064845	0.157402	0.420418	0.281838
RARRES1	-0.1254	-0.22376	-0.1736	-0.2437	-0.16916	-0.25392	-0.37732	0.03019
RARRES2	-0.03156	-0.06181	0.119604	-0.0386	0.002002	0.05544	-0.12299	-0.00629
RARRES3	-0.05806	-0.35305	0.264411	-0.48704	-0.41072	-0.05955	-0.34154	0.295253
RARS	-0.11543	-0.43725	-0.00872	-0.15703	-0.03249	-0.05543	-0.22936	0.04181
RARS2	-0.61543	-0.3035	-0.19221	-0.43179	0.256931	-0.04367	NA	NA
RASA1	0.266218	0.099836	0.221149	-0.2206	-0.03888	0.226276	0.284078	0.176436
RASA2	-0.37374	-0.56134	-0.01214	-0.07528	-0.16358	0.335523	0.015638	-0.02244
RASA3	-0.02594	-0.21751	0.254124	0.143894	0.240523	0.134799	-0.00658	-0.12173
RASA4	0.007479	0.069589	-0.21434	0.019621	-0.26376	0.090876	0.013309	0.400518
RASAL1	-0.28035	0.493849	-0.16343	0.252639	-0.13447	0.106884	0.14063	-0.14939
RASAL2	0.062504	-0.27611	0.058877	-0.13425	-0.21606	-0.04137	-0.14326	-0.31448
RASAL3	NA	NA	0.196506	0.79343	-0.20338	0.59854	NA	NA
RASD1	-0.14566	-0.55915	-0.08102	0.147221	-0.02736	-0.3602	-0.05306	-0.10941
RASD2	0.067781	0.397318	-0.12048	0.172319	0.141147	-0.07392	-0.22253	-0.65917
RASEF	-0.10288	0.225062	0.02636	0.061672	0.080617	-0.01422	0.31877	-0.29203
RASGEF1A	-0.44721	0.402526	-0.2192	0.185609	0.089705	0.296024	-0.11386	-0.31502
RASGEF1B	-0.26328	0.274576	0.118572	0.281797	-0.35305	-0.06169	-0.01875	0.110252
RASGEF1C	0.178745	0.583346	-0.12652	-0.16579	-0.14069	-0.29614	-0.2373	-0.44313
RASGRF1	0.363617	0.694229	0.18221	0.65187	0.311745	0.408294	0.103809	-0.34254
RASGRF2	0.097547	-0.23173	0.201784	-0.03542	-0.01524	0.126138	0.251202	0.391969
RASGRP1	-0.28548	-0.12496	0.110269	0.535193	-0.25386	0.707884	-0.63061	-0.14005
RASGRP2	-0.34507	0.113088	0.037202	0.669552	-0.12817	0.748233	-0.45852	-0.01048
RASGRP3	-0.17042	-0.02699	-0.0417	0.327477	-0.26177	0.028644	-0.44052	-0.00122
RASGRP4	0.161283	0.654826	0.221885	0.089001	0.034867	-0.16376	0.283495	0.237095

RASIP1	-0.27102	-0.05084	-0.25414	0.130164	-0.03607	0.196451	-0.20097	-0.03461
RASL10A	-0.2061	0.356089	0.170202	0.119241	0.060687	-0.06453	NA	NA
RASL10B	0.390984	0.195911	-0.07024	-0.22282	0.045944	-0.28709	0.192751	0.268836
RASL11A	0.417883	0.013392	-0.11709	0.110329	-0.02803	-0.30714	-0.05367	-0.0514
RASL11B	0.157261	-0.58169	-0.03374	0.332491	-0.18477	-0.0024	0.467652	-0.15772
RASL12	-0.24698	-0.07641	0.162923	-0.13522	-0.00261	0.174602	-0.31021	-0.37771
RASSF1	-0.5496	-0.38299	-0.07025	0.428122	0.43257	0.353982	0.305625	0.586967
RASSF2	-0.6209	0.00065	0.237776	0.347824	-0.01897	0.678539	-0.52481	-0.21686
RASSF3	-0.09601	0.025605	0.135007	-0.32877	-0.2272	-0.50188	-0.30253	-0.37534
RASSF4	-0.28728	0.160285	0.140031	0.0116	-0.41754	0.169336	-0.37045	0.096683
RASSF5	0.088221	0.231442	0.101473	0.714503	-0.15403	0.587098	-0.25041	0.140773
RASSF6	-0.57336	-0.3493	-0.17767	-0.17154	-0.18759	-0.4909	-0.26012	-0.16738
RASSF7	-0.06557	0.256652	-0.02137	0.043938	0.083306	-0.19217	0.47071	0.164017
RASSF8	-0.07032	0.664303	0.018487	-0.17556	0.046824	0.208027	-0.45642	-0.54208
RASSF9	NA	NA	0.045794	-0.05411	-0.36268	-0.14058	NA	NA
RAVER1	0.442728	0.608657	-0.04496	0.00518	-0.05182	0.338305	0.664254	0.379349
RAVER2	0.212992	0.151258	0.035348	-0.13439	-0.14108	-0.43833	0.347781	0.221777
RAX	-0.31595	0.457464	0.128469	-0.06973	-0.2487	0.251997	0.022607	0.233184
RAX2	NA	NA	-0.16705	-0.1717	0.006492	0.383557	NA	NA
RB1	0.007275	-0.06944	-0.27048	-0.19774	0.175893	0.124516	0.542209	0.380444
RB1CC1	0.128558	-0.37038	0.199033	-0.2529	0.014223	-0.00958	-0.02926	-0.19527
RBAK	0.3012	0.647207	-0.20318	-0.15354	0.000829	-0.08177	0.275254	0.252596
RBBP4	-0.13667	-0.34808	0.072556	0.269994	0.231953	0.21045	0.244752	0.020976
RBBP5	-0.11429	-0.38264	-0.32325	0.08807	-0.20557	-0.36855	0.199595	0.381132
RBBP6	0.203158	-0.1081	-0.05007	0.123741	0.176874	0.188443	0.153715	0.012331
RBBP7	-0.00716	-0.54233	-0.00421	0.187818	0.284591	0.196591	-0.54195	-0.63073
RBBP8	0.352179	0.427615	-0.19451	0.007708	-0.05769	-0.34276	-0.0534	0.309595
RBBP9	0.195656	-0.10186	-0.08889	-0.29852	-0.10525	-0.19164	0.055502	-0.23454
RBCK1	-0.09938	-0.48321	-0.01651	0.064578	0.258596	0.098095	0.251413	-0.09902
RBKS	0.419618	0.505429	0.470331	-0.33863	-0.00259	-0.55791	0.349983	0.120932
RBL1	0.281039	-0.32339	-0.14619	0.126233	0.162663	-0.07361	0.375205	0.378786
RBL2	-0.09543	0.307474	-0.08434	0.199096	0.320334	-0.11203	0.434362	0.401995
RBM10	0.200612	-0.06534	0.154439	0.379884	0.297246	0.395354	0.217935	-0.06863
RBM11	0.274771	0.273326	-0.14329	0.061068	-0.11079	-0.02114	-0.17743	-0.08183
RBM12	0.45367	0.161921	0.012844	-0.24743	0.411825	0.343237	0.263655	-0.05151
RBM12B	0.238289	0.22376	0.001816	-0.0354	-0.06406	-0.03972	-0.29219	-0.56864
RBM14	0.303597	0.567988	0.008459	0.374154	0.428819	0.239114	0.290574	-0.05256
RBM15	0.098271	0.511861	0.072384	-0.07962	0.362523	0.082092	0.234123	-0.00517
RBM15B	0.114482	0.385584	-0.091	0.199398	0.308438	0.174559	0.259567	-0.02998
RBM16	0.197918	-0.65324	-0.50022	-0.05919	0.14703	0.07219	NA	NA
RBM17	0.050557	-0.26054	0.010444	0.135604	0.382464	0.322189	0.327948	0.336663
RBM18	-0.12304	-0.46573	-0.23347	-0.35881	-0.25788	-0.57802	0.179885	0.258213
RBM19	-0.10322	0.129336	-0.00553	0.436306	0.405349	0.476954	0.346732	0.180561
RBM20	NA	NA	-0.06341	-0.13019	-0.10392	-0.12478	NA	NA
RBM22	0.006505	-0.10203	-0.0455	0.36188	-0.18081	-0.0983	0.185572	0.294221
RBM23	0.148035	0.498636	-0.11368	0.07008	0.018381	-0.16183	-0.05746	0.336797
RBM24	-0.21536	-0.34189	0.302018	-0.43927	0.05294	-0.47571	-0.515	-0.21106
RBM25	-0.02873	0.096206	-0.32555	0.257425	-0.08959	0.22139	-0.51699	-0.14198
RBM26	0.124356	-0.68613	-0.06631	0.309441	0.181732	0.300971	NA	NA
RBM27	NA	NA	0.15134	-0.23491	-0.03572	-0.0236	NA	NA
RBM28	0.489877	-0.44784	-0.32351	0.249113	0.000655	0.201377	0.596895	0.442763
RBM3	-0.02461	0.502399	-0.0159	-0.11928	0.034827	0.015118	-0.27622	-0.31369

RBM33	-0.24722	-0.46905	-0.01799	0.142541	-0.13874	-0.06617	0.028609	0.341314
RBM34	-0.24141	-0.76151	-0.24859	0.011135	-0.30041	-0.33662	-0.36278	-0.37714
RBM35B	-0.32637	0.019337	-0.38035	0.019362	0.195498	0.033261	0.255623	-0.04532
RBM38	0.165812	0.352119	0.108838	-0.00976	0.088468	0.189601	-0.28614	-0.41975
RBM39	0.184809	-0.54613	0.047683	0.138815	0.339061	0.326155	0.190807	-0.15673
RBM4	-0.14495	-0.68729	0.107884	0.314895	0.073533	0.047962	0.361373	0.186072
RBM41	0.003815	-0.47992	-0.25584	0.102319	-0.03528	-0.31817	0.149891	-0.20009
RBM42	NA	NA	0.268763	0.150643	0.11148	0.307736	NA	NA
RBM43	0.213191	0.180079	0.248225	-0.20394	0.042835	0.072843	NA	NA
RBM45	NA	NA	-0.17367	-0.31599	-0.11335	-0.20565	NA	NA
RBM46	NA	NA	-0.15195	-0.07337	-0.3512	-0.00296	NA	NA
RBM47	NA	NA	-0.25667	-0.0672	-0.00657	-0.12139	NA	NA
RBM4B	-0.25053	-0.07221	-0.02159	0.178711	-0.09048	0.197104	0.393754	0.265217
RBM5	-0.33932	-0.72715	0.046493	0.02452	0.206615	0.522283	0.18752	0.436111
RBM6	0.290597	-0.3546	-0.08009	0.356363	0.165192	0.443517	-0.11371	0.398254
RBM7	-0.08538	-0.38003	-0.22971	-0.23933	-0.25909	-0.24348	-0.11926	-0.14849
RBM8A	0.137676	-0.001	-0.66339	0.021074	-0.19038	-0.15797	0.255716	0.072216
RBM9	-0.05928	-0.15942	0.03624	-0.05323	0.195358	0.026843	-0.02718	-0.13419
RBMS1	-0.0388	-0.30138	0.328197	-0.0168	-0.06529	0.66211	-0.00036	0.12018
RBMS2	0.426495	0.438996	-0.18089	-0.26991	-0.0845	-0.02238	-0.3409	-0.22644
RBMS3	0.00891	-0.00884	0.135559	0.039337	-0.01403	0.390065	-0.28852	-0.02049
RBMX	0.530715	-0.22169	-0.3898	0.35695	0.018114	-0.16307	0.479397	0.164413
RBMX2	0.406642	0.641895	-0.08285	0.266469	0.069547	-0.00722	0.027725	-0.3236
RBMXL1	0.119665	-0.40952	NA	NA	NA	NA	-0.38971	-0.13525
RBMXL2	NA	NA	0.166806	-0.22327	-0.07322	-0.19106	NA	NA
RBMY1A1	-0.0035	-0.07243	NA	NA	NA	NA	0.293458	0.240713
RBMY1B	0.070986	-0.22937	0.28329	-0.12039	-0.0773	0.042139	0.044521	0.226714
RBMY1E	0.211968	0.573524	0.17371	-0.11593	-0.05045	-0.23848	0.668959	0.258705
RBMY2EP	NA	NA	0.034841	-0.07535	-0.23975	0.030643	0.372722	0.071216
RBMY2FP	NA	NA	0.251921	-0.08785	0.193825	0.121067	0.149612	-0.04007
RBP1	0.215982	-0.06805	0.342402	-0.17052	-0.22752	0.160039	-0.16236	-0.15253
RBP2	0.189922	0.143535	0.216247	-0.27367	-0.27415	-0.48192	0.409797	0.50704
RBP3	-0.19959	0.338141	NA	NA	NA	NA	-0.27352	-0.32656
RBP4	0.187181	-0.10445	-0.51395	-0.0053	0.096848	-0.42042	0.069627	0.005183
RBP5	0.112305	0.538606	-0.01299	0.779221	-0.224	0.754889	-0.54422	-0.31377
RBP7	-0.15987	-6.1E-05	-0.13312	0.121724	-0.17866	-0.00466	-0.38576	0.003863
RBPJ	-0.25201	-0.071	-0.10244	-0.03997	-0.05814	0.19848	0.257274	0.16826
RBPJL	-0.02961	-0.22431	-0.159	0.05306	0.164116	-0.08634	NA	NA
RBPMS	-0.02718	-0.26867	0.256441	-0.01689	-0.10559	0.333284	-0.16628	-0.20851
RBPMS2	-0.06558	-0.0987	0.129604	-0.09301	-0.05304	0.110483	-0.25829	-0.32731
RBX1	-0.35689	-0.65813	-0.02578	-0.19944	-0.32498	-0.52461	-0.40788	-0.33534
RC3H1	0.041368	0.767391	-0.07239	-0.23481	-0.11477	0.060552	-0.48611	-0.01137
RC3H2	-0.11678	-0.34217	0.10353	-0.12739	0.166045	-0.44465	NA	NA
RCAN1	NA	NA	-0.32825	-0.15456	-0.16971	-0.23858	NA	NA
RCAN2	NA	NA	-0.08605	0.025719	-0.06098	0.062789	NA	NA
RCAN3	NA	NA	-0.00756	0.365432	0.267176	0.137752	NA	NA
RCBTB1	0.16966	-0.47528	0.24221	-0.27007	0.207558	-0.1552	0.459343	0.424554
RCBTB2	-0.20486	-0.59767	-0.15507	-0.2212	-0.2407	0.143139	-0.07443	0.232391
RCC1	NA	NA	0.291846	0.020303	0.004482	-0.27567	0.293771	0.181699
RCC2	-0.42366	-0.16373	0.074698	0.374829	0.308307	0.583338	0.232123	-0.08309
RCCD1	0.148865	0.356603	-0.21407	0.461829	0.34181	0.010075	0.226504	0.16273
RCE1	0.12972	0.635328	0.211461	0.257599	0.163301	-0.02924	0.578246	0.386714

RCHY1	-0.29461	-0.21283	-0.07433	-0.12195	-0.10225	0.01327	0.411824	0.468604
RCL1	0.191179	-0.51518	-0.1781	0.204667	0.056003	0.229933	0.571492	0.457947
RCN1	NA	NA	-0.09296	0.462784	0.08902	-0.24805	-0.31905	0.079757
RCN2	-0.17205	-0.50371	0.294573	-0.13626	0.081046	-0.0765	-0.49752	-0.52071
RCN3	-0.09263	0.134929	0.141249	0.02359	-0.0062	-0.02703	-0.31223	-0.08302
RCOR1	-0.16943	0.331306	-0.32733	-0.08256	-0.06678	0.221853	0.040204	-0.0233
RCOR2	0.046037	0.759047	0.05227	0.018628	-0.05728	0.268253	0.22884	-0.04212
RCOR3	-0.1164	-0.24804	-0.32247	-0.29709	0.032626	-0.12856	0.338693	0.178714
RCSD1	-0.58728	-0.1086	0.115061	0.570214	-0.08862	0.521228	-0.55875	-0.38868
RCVRN	-0.01863	-0.33823	-0.08028	0.11387	0.045416	0.120987	NA	NA
RD3	-0.07133	0.413028	NA	NA	NA	NA	NA	NA
RDBP	0.214467	0.744246	-0.06934	0.241267	0.140716	0.009463	0.181844	0.085419
RDH10	0.008319	-0.42677	-0.02546	-0.1154	0.141069	-0.26408	0.254712	0.377167
RDH11	-0.0465	0.480455	-0.39592	0.179754	-0.41132	-0.24524	-0.54627	-0.4807
RDH12	0.002711	0.123847	-0.10596	-0.08461	-0.09204	-0.05375	0.006084	0.267814
RDH13	-0.05162	0.470753	0.163644	-0.03185	0.380956	0.297922	0.473026	0.189158
RDH14	-0.06399	-0.68534	0.013815	-0.07678	-0.11971	-0.20438	-0.1639	-0.29244
RDH16	-0.38915	0.078065	-0.11707	-0.04182	0.051111	0.218823	-0.14964	0.404578
RDH5	-0.19753	0.460923	-0.05408	-0.28782	-0.12612	-0.24208	-0.21923	-0.0374
RDH8	-0.04895	0.293471	0.011343	-0.40306	-0.00142	-0.10535	0.053165	0.158471
RDM1	0.111417	0.498284	0.123005	0.020867	-0.03659	0.020105	0.371282	0.384297
RDX	-0.15544	-0.57473	0.019135	-0.4069	-0.27712	0.119917	NA	NA
REC8	NA	NA	-0.18579	-0.21789	-0.19772	0.348327	NA	NA
RECK	-0.26023	-0.49582	0.06731	-0.11064	-0.24111	0.244888	-0.5096	-0.38022
RECQL	0.147473	-0.1376	-0.03538	0.026645	-0.19946	0.224117	-0.47366	-0.21121
RECQL4	-0.03583	0.186324	0.161504	0.29759	0.241077	0.099965	0.407786	0.303264
RECQL5	0.311793	0.274616	0.34918	0.050903	0.296501	0.308597	0.267022	0.234213
REEP1	0.09707	-0.17943	0.537657	-0.12691	0.439573	-0.2803	-0.20427	-0.28504
REEP2	-0.06958	0.046386	-0.00194	-0.29328	-0.00533	-0.10745	-0.45749	-0.38368
REEP3	-0.07433	-0.28341	-0.1506	-0.38258	-0.14203	-0.48023	0.212703	0.452553
REEP4	-0.07534	0.23693	0.372229	0.038195	-0.01585	0.138767	0.2546	0.0775
REEP5	-0.02991	-0.11793	0.447017	-0.26177	-0.08533	-0.31094	-0.49553	-0.15443
REEP6	-0.30001	0.022531	0.170132	-0.06041	0.171765	-0.09435	0.308203	0.439446
REG1A	-0.38975	0.402491	0.075314	-0.21271	0.047322	-0.00364	0.136538	0.38272
REG1B	-0.25194	0.288668	0.06684	-0.16515	0.063243	0.037591	0.095204	0.471741
REG1P	NA	NA	-0.21607	-0.04313	-0.11183	-0.00155	NA	NA
REG3A	0.656962	-0.26837	0.258991	0.080117	0.005414	-0.00878	0.143823	0.445833
REG3G	0.152094	0.473445	0.215329	0.091143	-0.07194	0.047227	-0.07697	0.482425
REG4	-0.48524	0.261516	-0.07285	0.310414	0.072868	-0.17821	0.034847	0.260183
REL	-0.18273	-0.34549	0.086792	-0.02021	0.03229	0.209132	-0.51196	-0.41347
RELA	0.19402	-0.41708	0.285029	-0.07754	0.357102	0.481262	0.14646	0.145514
RELB	0.123937	0.051793	-0.31455	0.229103	0.037682	0.186293	-0.18181	0.501934
RELL1	NA	NA	-0.17065	-0.53475	0.062678	-0.45757	NA	NA
RELL2	NA	NA	0.391506	0.268018	0.30106	-0.05878	NA	NA
RELN	0.158967	0.051479	-0.21414	0.219519	-0.16547	0.276258	-0.4292	-0.54137
RELT	NA	NA	0.025879	0.5894	0.021529	0.346473	NA	NA
REM1	-0.13964	0.193916	0.095787	-0.05531	0.000103	0.0278	-0.42276	-0.09651
REM2	0.272591	0.742214	0.283827	-0.14621	0.252754	-0.01795	NA	NA
REN	-0.09221	0.164773	-0.05652	-0.21693	-0.16035	-0.21458	-0.09363	0.159537
RENBP	-0.23766	0.328872	0.220673	0.016599	-0.01771	0.142839	-0.33231	0.168041
REP15	-0.14897	0.044035	NA	NA	NA	NA	0.107504	-0.01466
REPIN1	0.364137	-0.08152	0.120784	0.151898	0.311538	0.137343	0.245362	-0.0636

REPS1	0.388557	0.217169	-0.19458	0.197605	0.388825	0.038903	0.590602	0.468252
REPS2	0.1662	-0.1392	0.072507	-0.20022	0.004515	-0.32638	0.133667	-0.17951
RER1	-0.36501	0.051756	0.232973	-0.06899	0.089342	-0.47905	-0.33274	-0.36788
RERE	-0.20688	-0.45556	0.145772	-0.09554	0.181315	0.006181	0.040469	-0.16023
RERG	-0.26471	-0.45168	0.05123	-0.11136	-0.01036	-0.13678	-0.22098	-0.1877
RERGL	NA	NA	0.053449	0.170514	-0.28364	-0.14144	NA	NA
RESP18	0.28999	0.118497	0.17545	-0.275	-0.14601	-0.31119	0.170522	-0.14467
REST	-0.28313	-0.17945	0.102544	-0.21821	0.040605	0.071448	0.400839	0.344904
RET	0.068424	0.084426	-0.02425	0.150366	-0.24586	-0.12369	0.139512	0.207232
RETN	-0.65899	-0.08021	-0.07302	-0.21494	-0.03106	-0.18832	-0.13425	-0.46873
RETNLB	0.190321	0.273104	-0.22844	-0.18971	-0.0999	-0.10453	-0.18997	0.24381
RETSAT	-0.43349	0.010018	0.118317	0.037404	0.240735	-0.16092	NA	NA
REV1	-0.31021	-0.5568	-0.29567	-0.4861	0.022201	-0.12995	-0.31553	-0.17969
REV3L	0.138265	-0.51271	-0.20837	-0.24461	-0.02881	-0.00394	0.241565	0.369999
REXO1	0.145385	0.364791	-0.08859	0.041437	0.150895	-0.22934	-0.3668	-0.35493
REXO1L1	0.083011	0.320698	0.0443	-0.31095	0.103335	-0.06475	-0.04399	-0.03408
REXO2	-0.24641	-0.21618	0.00826	-0.12622	-0.21634	0.04757	-0.53082	-0.37833
REXO4	0.318635	0.601685	-0.03003	0.376198	0.329473	-0.13347	-0.38715	-0.24277
RFC1	-0.02587	-0.28495	0.133577	0.175616	-0.1446	0.018746	0.033231	-0.24347
RFC2	-0.12594	-0.47886	0.116206	0.303085	0.021909	0.123727	0.026105	0.02687
RFC3	0.282604	-0.43573	-0.23583	0.189478	0.005248	-0.05408	0.564392	0.343089
RFC4	-0.08668	-0.35058	-0.21117	0.387572	0.058224	-0.06711	0.219122	0.093206
RFC5	0.038924	0.229102	-0.49619	0.085403	0.237296	-0.24185	0.221383	-0.05481
RFESD	-0.04484	-0.28624	0.16179	-0.11864	-0.29907	-0.35602	0.284169	0.306045
RFFL	0.174563	-0.42088	-0.04923	-0.18204	0.112549	-0.42849	0.023597	-0.11872
RFK	0.082869	-0.05123	-0.17706	-0.26235	0.016348	-0.33821	-0.20739	-0.2436
RFNG	-0.15365	-0.0894	0.379283	-0.05776	0.420968	-0.12638	0.451441	0.35456
RFPL1	0.022289	-0.04368	-0.2005	-0.07345	-0.09273	0.157192	NA	NA
RFPL2	-0.14361	0.441152	0.040556	-0.2617	-0.32404	-0.17161	NA	NA
RFPL3	0.216464	-0.03794	-0.17833	0.00055	-0.22727	-0.07911	NA	NA
RFPL4A	NA	NA	0.01834	0.011135	-0.24098	0.139843	NA	NA
RFPL4B	0.110186	0.539742	0.245514	-0.26602	-0.08976	-0.32712	-0.2993	-0.35521
RFT1	-0.10173	0.715403	-0.04059	0.256865	0.081349	0.202569	-0.31627	-0.47569
RFTN1	-0.28072	-0.06907	0.018417	0.748294	-0.05965	0.549609	NA	NA
RFTN2	0.046538	-0.15903	-0.16662	-0.18318	-0.31568	-0.23372	NA	NA
RFWD2	0.11715	0.160163	-0.16714	-0.20138	-0.26716	-0.32299	0.263142	0.055874
RFWD3	0.337651	0.247975	-0.06558	-0.12628	0.14211	-0.05359	0.422842	0.176598
RFX1	0.163636	0.544203	-0.1554	0.196321	-0.14048	0.245987	0.222966	0.08891
RFX2	0.146062	0.110098	0.308532	-0.14506	0.136268	0.257427	0.066118	0.038997
RFX3	-0.08316	-0.57543	-0.19612	-0.17433	-0.15662	0.270555	0.014226	-0.04806
RFX4	0.336423	0.677504	-0.34171	-0.12542	0.142094	-0.27235	-0.66203	-0.53414
RFX5	-0.06132	-0.31027	-0.00123	0.288384	-0.32301	-0.21589	-0.03372	0.407833
RFX6	NA	NA	-0.00544	-0.05439	-0.08425	-0.39821	NA	NA
RFX7	NA	NA	0.064787	-0.14007	-0.26751	0.047738	NA	NA
RFXANK	0.287032	-0.43639	-0.00674	0.272289	0.011286	-0.08068	0.029263	-0.01973
RFXAP	0.083794	-0.21677	-0.02773	-0.06204	0.269193	-0.06096	-0.34447	-0.39504
RG9MTD1	0.3028	-0.39964	-0.36276	-0.29295	-0.0167	0.136119	-0.05945	-0.19311
RG9MTD2	-0.10246	-0.41495	0.018111	-0.31695	-0.08555	-0.19157	-0.13562	0.082055
RG9MTD3	-0.20011	-0.29306	-0.07552	-0.14233	0.050399	0.173835	-0.06425	0.377515
RGAG1	-0.2537	-0.19938	-0.25759	-0.07995	0.171239	-0.08565	-0.45135	0.078842
RGAG4	0.176595	0.03704	0.089675	0.071841	-0.18611	0.087907	-0.42623	-0.41813
RGL1	-0.22254	-0.17546	0.514405	-0.10102	-0.35325	0.109964	-0.34993	-0.17625

RGL2	-0.0722	0.1276	0.304222	0.165627	0.093804	0.369664	0.407019	0.204033
RGL3	NA	NA	-0.25448	0.060917	-0.39031	0.023382	0.219563	0.089629
RGL4	NA	NA	0.238839	0.538449	-0.0142	0.349443	NA	NA
RGMA	0.026435	-0.19093	0.172899	0.260488	-0.12242	0.264945	-0.19948	-0.10949
RGMB	0.06576	0.184156	-0.1239	0.260372	0.06558	-0.18539	0.000568	0.334985
RGN	0.147912	-0.26085	-0.024	-0.07758	0.054256	-0.0091	0.115115	-0.03214
RGNEF	NA	NA	0.284446	0.034691	0.178362	-0.12891	0.321843	0.269885
RGP1	NA	NA	-0.32899	0.051381	0.124555	-0.0363	NA	NA
RGPD1	-0.13376	0.378515	0.097057	0.107452	-0.17429	0.066954	0.072562	0.543534
RGPD2	NA	NA	0.036722	0.057978	-0.18213	0.047027	-0.18942	-0.10205
RGPD5	NA	NA	-0.21268	-0.16105	-0.13287	-0.20225	0.36084	-0.06458
RGR	0.062153	0.285481	-0.05282	0.013925	-0.03043	0.056319	0.303473	0.048156
RGS1	-0.32559	-0.43749	0.109268	0.105971	-0.23959	-0.02662	-0.42496	-0.40468
RGS10	-0.27747	-0.20919	-0.12388	-0.06537	-0.1911	-0.00159	-0.62952	-0.49454
RGS11	-0.30942	0.199824	-0.04813	-0.08442	0.094061	0.160923	-0.41152	-0.11004
RGS12	-0.19223	0.603874	0.15718	-0.00635	0.232132	0.421302	-0.20357	0.035852
RGS13	0.270868	0.069607	-0.10144	0.49767	-0.29257	0.326331	0.012477	0.123286
RGS14	-0.11095	-0.29954	0.354851	0.488628	0.22842	0.333855	0.000826	0.646874
RGS16	-0.14847	-0.00923	0.273441	0.342759	-0.12942	0.113775	-0.00131	0.03661
RGS17	-0.08151	-0.02244	-0.30455	-0.42133	-0.14035	-0.09996	0.06143	0.130235
RGS18	-0.05149	0.318304	0.041923	-0.09692	-0.10854	-0.19809	-0.50506	0.401239
RGS19	-0.09878	-0.15157	0.416086	0.543076	0.188143	0.505965	0.163058	0.40863
RGS2	-0.60987	-0.55675	0.153155	-0.06521	-0.05137	0.007571	-0.38244	-0.11253
RGS20	0.269095	0.503125	-0.34645	-0.12814	-0.22942	0.089212	0.431414	0.398139
RGS22	-0.26385	0.213508	-0.38893	-0.10656	-0.35089	0.097384	0.352495	0.411588
RGS3	-0.03333	0.770693	0.057056	-0.04615	0.166074	0.092631	-0.12476	0.20383
RGS4	0.21714	-0.11255	0.029732	-0.25098	-0.27381	-0.26392	0.20378	-0.01738
RGS5	-0.05306	-0.52277	-0.0747	-0.07882	0.009605	0.023355	-0.42758	-0.48961
RGS6	0.037419	0.662849	-0.36324	-0.20581	-0.14254	0.321903	0.072802	-0.05272
RGS7	-0.221	0.117937	-0.13983	0.233634	-0.1656	-0.20241	0.179811	0.14738
RGS7BP	-0.01002	-0.51018	0.016632	0.294303	0.107499	-0.2856	NA	NA
RGS8	0.282072	0.382659	-0.197	0.039825	0.043183	-0.0009	-0.07252	0.141267
RGS9	0.097084	0.417832	0.282846	0.133628	-0.1959	0.49999	-0.41097	-0.1756
RGS9BP	0.012609	0.11514	0.137625	0.172604	-0.06386	-0.1592	0.467098	0.189172
RGSL1	-0.05014	0.112876	-0.08897	0.212615	-0.10229	-0.22379	NA	NA
RHAG	0.126485	-0.00805	0.244075	0.132075	0.08966	-0.02758	0.204313	-0.18748
RHBDD1	0.378517	0.195779	0.244959	0.229312	-0.05998	-0.20444	-0.12782	-0.02273
RHBDD2	-0.08117	0.453031	0.329374	0.075926	-0.04597	-0.05252	0.483726	0.431294
RHBDD3	-0.158	0.153502	0.095724	0.00182	0.209309	0.09477	-0.06166	-0.19844
RHBDF1	-0.04953	0.114489	0.141831	0.007387	0.383023	0.311673	0.162287	-0.07643
RHBDF2	-0.12623	0.256852	0.135095	0.458156	-0.10799	0.61205	-0.20642	0.19129
RHBDL1	-0.00795	0.445346	-0.05314	-0.00246	-0.07514	0.167605	0.561925	0.314754
RHBDL2	-0.35566	-0.10043	-0.14807	0.042939	-0.16142	-0.22105	-0.51209	-0.59872
RHBDL3	-0.34127	0.261006	-0.14816	0.019588	-0.10251	-0.27069	0.026903	0.281398
RHBG	0.126332	0.20161	0.104256	-0.20268	-0.07716	-0.22846	-0.1122	-0.18992
RHCE	-0.19321	0.515474	0.055272	-0.12502	-0.10374	-0.35876	-0.06427	-0.17763
RHCG	0.019896	0.473366	-0.09049	0.249531	-0.1207	0.106501	-0.30239	-0.30073
RHD	0.108643	0.350687	-0.00078	0.093008	0.370992	0.33341	-0.06556	0.230559
RHEB	0.301392	-0.61347	-0.17342	0.021602	0.00317	-0.17883	0.045055	-0.13883
RHEBL1	0.17427	0.302375	0.111699	0.232829	0.043197	-0.07673	-0.1327	0.119832
RHO	0.214598	0.362674	-0.26751	0.105102	-0.19875	-0.16812	0.076453	0.448163
RHOA	-0.09067	-0.27133	-0.11799	0.230095	-0.07578	-0.00963	-0.66541	-0.64939

RHOB	0.170311	-0.39358	-0.01729	-0.13277	0.049122	-0.27877	-0.53661	-0.51355
RHOBTB1	-0.02025	-0.04403	0.300203	0.106324	-0.13065	-0.30583	-0.45348	-0.42404
RHOBTB2	-0.20864	0.597766	0.477623	0.356713	-0.07458	0.44442	0.269629	0.510143
RHOBTB3	-0.06421	-0.26499	0.262375	-0.11008	-0.21739	-0.03864	0.262779	0.139768
RHOC	-0.35267	0.55812	0.306419	-0.06119	-0.00504	-0.32104	-0.60556	-0.47983
RHOD	NA	NA	0.180272	-0.22439	0.049813	-0.28268	0.361614	0.201953
RHOF	0.083151	0.383665	0.17185	-0.15737	-0.09587	0.020713	0.019081	0.171545
RHOG	-0.11505	0.37896	0.390812	0.072337	-0.04283	0.183193	0.140312	0.287834
RHOH	-0.58353	0.226005	0.068455	0.762918	-0.03613	0.530797	-0.29661	-0.19956
RHOJ	-0.18504	-0.11171	-0.06548	-0.1199	-0.21417	-0.01971	-0.48011	-0.46274
RHOQ	-0.09472	0.000922	-0.03029	0.004684	-0.17701	0.01842	-0.3446	-0.15352
RHOT1	0.284397	-0.53979	0.249109	-0.27503	0.072239	0.091715	0.076968	0.039597
RHOT2	0.21717	0.239858	0.192999	0.115014	0.394773	0.318742	0.503714	0.522228
RHOU	0.06962	0.044833	0.264234	-0.54878	0.067935	-0.33067	NA	NA
RHOV	-0.26915	-0.10943	-0.13562	0.270193	-0.15361	-0.14341	-0.19846	-0.37967
RHOXF1	NA	NA	-0.13156	-0.1507	-0.36639	-0.0375	NA	NA
RHPN1	0.154347	0.470424	0.091842	-0.12386	-0.02093	-0.03367	-0.02136	-0.12336
RHPN2	0.132474	-0.42625	-0.33276	-0.08773	0.048637	0.032382	0.423194	0.152105
RIBC1	-0.21806	0.315208	0.071137	-0.33692	0.066063	-0.37876	0.003952	-0.36462
RIBC2	-0.35361	-0.00066	-0.13509	0.124008	0.175978	-0.13893	0.234089	0.411739
RIC3	0.060533	-0.09174	-0.12337	0.26773	-0.05132	0.407973	-0.38607	-0.27328
RIC8A	0.035908	-0.55356	-0.16902	0.248495	0.22759	0.169927	0.540091	0.207295
RIC8B	-0.04807	0.176141	-0.46429	0.105859	-0.09228	-0.18991	-0.60277	-0.6195
RICS	-0.54263	-0.28087	0.066438	-0.16614	-0.04634	-0.04882	0.415935	0.285605
RICTOR	-0.25285	-0.45284	-0.02093	-0.22035	0.113147	0.294191	-0.06347	-0.22954
RIF1	-0.23994	-0.33008	-0.38091	-0.07995	-0.05492	0.247989	0.230655	0.105736
RILP	0.33769	0.711156	0.121457	-0.24889	-0.16549	-0.13931	-0.63268	-0.28317
RILPL1	NA	NA	0.253603	0.001872	0.201825	0.242975	NA	NA
RILPL2	NA	NA	0.080591	0.288479	0.021876	0.342562	NA	NA
RIMBP2	0.238091	-0.16322	0.217914	-0.11347	-0.21143	-0.02658	0.17232	0.593022
RIMKLA	NA	NA	0.043075	-0.3199	0.238039	-0.44732	NA	NA
RIMKLB	NA	NA	-0.30261	-0.05752	-0.11226	0.226679	NA	NA
RIMS1	-0.10338	0.259659	0.186957	0.033206	0.20538	-0.0796	-0.23279	0.04952
RIMS2	0.285685	-0.13329	0.252073	-0.15328	-0.28631	0.094178	-0.40009	0.158897
RIMS3	-0.35582	0.315089	0.214724	-0.44154	0.220194	-0.12734	-0.55331	-0.38048
RIMS4	0.233744	-0.05656	0.069267	-0.37853	0.148235	0.021874	-0.34816	-0.26725
RIN1	0.220904	0.335416	0.26812	0.080321	0.357548	0.055406	-0.07504	-0.0804
RIN2	0.079054	-0.41629	-0.38388	0.161735	-0.00187	0.111566	0.250008	-0.04882
RIN3	0.114407	0.149134	0.217534	-0.02224	0.174456	0.479208	-0.17292	-0.14976
RING1	0.228634	0.36651	0.234778	0.156803	-0.00344	0.098147	-0.43132	-0.14816
RINT1	0.27743	-0.37233	-0.20801	0.156736	-0.13421	-0.20787	NA	NA
RIOK1	-0.11559	0.457755	-0.22849	0.204073	-0.01241	0.264639	0.036194	0.074157
RIOK2	-0.19142	-0.73131	0.135037	-0.18045	-0.16685	-0.26453	0.286458	0.208283
RIOK3	-0.4699	-0.29303	-0.13217	-0.18049	-0.26468	-0.30473	0.098761	0.183165
RIPK1	-0.39556	0.484205	-0.06917	-0.2651	-0.1817	-0.19249	0.522937	0.485282
RIPK2	-0.2884	-0.08517	-0.02829	-0.04381	-0.09073	-0.14693	0.261431	0.383096
RIPK3	-0.0054	0.268533	0.010548	-0.01617	0.219458	-0.01298	0.24264	0.245885
RIPK4	0.297078	0.410494	0.029997	-0.09203	0.16064	-0.13034	0.485001	0.202549
RIPPLY1	NA	NA	-0.25798	-0.19548	0.317777	0.09873	NA	NA
RIPPLY2	NA	NA	-0.13713	0.051822	-0.19478	0.082643	NA	NA
RIT1	-0.74047	-0.485	-0.2276	-0.0717	-0.40971	-0.26359	-0.13089	-0.05551
RIT2	-0.49194	-0.06991	0.212522	0.15035	-0.2262	0.265156	-0.00801	0.280858

RLBP1	-0.26715	0.469394	-0.11764	-0.02552	-0.44445	-0.21678	-0.10608	0.143434
RLF	-0.45705	0.243367	-0.18424	-0.47195	-0.06081	-0.05509	-0.5624	-0.39776
RLN1	0.290205	0.157944	0.063854	-0.4486	-0.05923	-0.14534	-0.00193	0.317024
RLN2	0.376385	0.50767	0.151233	-0.05259	-0.11707	0.034781	0.00709	-0.21898
RLN3	0.395126	0.426971	-0.0604	-0.07851	0.014093	-0.15658	0.008405	-0.1458
RLTPR	0.13071	0.176308	0.343039	0.022634	-0.0787	-0.17139	0.126059	0.198884
RMI1	0.384016	0.050729	-0.29702	-0.03306	0.197876	-0.39059	0.053283	-0.04843
RMND1	0.255582	-0.3788	-0.06502	-0.20398	0.06749	-0.24796	NA	NA
RMND5A	0.099058	-0.3124	-0.43323	-0.09527	-0.01523	-0.43566	NA	NA
RMND5B	0.331366	0.646542	0.437442	0.050005	0.162841	0.124316	NA	NA
RNASE1	-0.34698	0.096558	0.303379	-0.25599	-0.46963	-0.25239	-0.41595	-0.11913
RNASE10	-0.05285	0.700473	NA	NA	NA	NA	0.538717	0.435759
RNASE11	0.455347	-0.22698	-0.06824	-0.07715	-0.36928	0.098694	0.215455	0.535575
RNASE12	0.266808	0.576601	NA	NA	NA	NA	0.40773	0.44279
RNASE13	0.422879	0.113442	NA	NA	NA	NA	-0.19253	-0.01014
RNASE2	-0.28714	-0.12821	0.107922	-0.02786	-0.34532	-0.24027	-0.4078	-0.00261
RNASE3	0.024795	0.519955	0.058249	-0.14142	-0.26858	0.247171	-0.63126	0.021126
RNASE4	-0.26949	-0.30126	-0.26389	0.103184	-0.07361	-0.09956	0.062793	0.132775
RNASE6	-0.07049	-0.24073	0.220952	-0.19728	-0.54695	0.304932	0.076937	0.48454
RNASE7	-0.11519	0.126046	-0.00354	0.24362	-0.05586	-0.33595	-0.03999	-0.14748
RNASE8	-0.21982	0.078649	0.178647	-0.07608	0.122906	0.127782	-0.0376	0.034514
RNASE9	0.049672	0.397616	NA	NA	NA	NA	-0.03019	0.257155
RNASEH1	0.151469	-0.1842	-0.08528	0.1878	-0.07774	0.128349	0.34623	0.011873
RNASEH2A	0.308715	0.470959	0.162808	0.336013	0.194899	-0.11486	0.221009	0.014209
RNASEH2B	0.447503	-0.11828	-0.08269	0.367953	0.050631	-0.40091	NA	NA
RNASEH2C	0.118615	0.210363	0.177092	-0.10394	0.068772	-0.1429	NA	NA
RNASEK	NA	NA	-0.11032	0.219482	-0.28484	-0.29952	NA	NA
RNASEL	-0.29269	0.146859	-0.14533	-0.16649	-0.14584	-0.33644	-0.37107	-0.2157
RNASEN	-0.11906	-0.71606	-0.32732	0.142116	0.146029	0.26194	-0.34325	-0.39575
RNASET2	0.162661	0.273164	-0.19967	0.245046	0.320308	-0.00306	-0.05158	-0.19281
RND1	-0.51597	0.208483	-0.08567	0.093247	-0.05158	-0.01581	-0.07198	0.074652
RND2	0.662621	0.308353	-0.18159	-0.26794	0.160945	-0.58858	-0.11806	-0.24741
RND3	-0.20662	-0.03937	0.049239	-0.49838	-0.12463	-0.31936	0.049499	0.024602
RNF10	-0.28371	0.222367	-0.05907	-0.10301	0.03427	-0.09741	-0.04769	0.41773
RNF103	-0.07458	0.585384	-0.00552	-0.16676	0.068959	-0.38138	NA	NA
RNF11	-0.30756	0.609976	-0.08069	-0.36251	-0.11934	-0.45522	0.33895	0.274089
RNF111	-0.43871	-0.59525	-0.0341	-0.32003	-0.22099	0.254675	0.274278	0.175797
RNF112	NA	NA	-0.34261	0.365427	-0.16778	-0.07886	NA	NA
RNF113A	0.564956	0.65108	-0.01986	0.231758	0.19035	-0.11182	-0.34195	-0.20302
RNF113B	-0.14695	0.395128	-0.1394	-0.05	0.362238	-0.11055	0.41887	0.468202
RNF114	NA	NA	-0.1168	-0.04657	0.407471	0.128062	NA	NA
RNF115	NA	NA	-0.12089	0.069715	-0.25935	0.370871	NA	NA
RNF12	-0.01352	0.664897	-0.33502	-0.3028	-0.1085	-0.37416	-0.13662	-0.34488
RNF121	-0.09071	0.379576	0.001752	-0.01405	-0.10667	0.00323	-0.1384	0.337504
RNF122	-0.28139	0.33235	-0.25271	-0.01761	-0.19855	0.108639	-0.55074	-0.42632
RNF123	0.48969	0.79114	0.182869	0.064482	0.372638	-0.08548	0.235301	0.280199
RNF125	-0.1987	-0.09794	0.192801	-0.09262	-0.04778	0.098775	-0.34326	0.137134
RNF126	0.132528	0.536183	-0.45849	0.065749	-0.01303	-0.23181	-0.26779	-0.41167
RNF128	-0.05133	0.13457	0.089121	-0.2167	0.108211	-0.49438	0.274602	0.082964
RNF13	-0.19966	-0.44245	0.064198	-0.51397	-0.31741	-0.07171	0.067082	0.238781
RNF130	-0.00065	-0.32564	0.151784	-0.17638	-0.0786	-0.2919	-0.23263	-0.47405
RNF133	0.10829	0.507987	0.371557	-0.06634	-0.20354	-0.14862	0.207193	0.075221

RNF135	0.084063	-0.55913	0.290624	-0.28163	-0.19059	-0.18294	-0.19771	0.403996
RNF138	-0.10786	0.267762	-0.14631	-0.30391	-0.23633	-0.33994	-0.1151	-0.24665
RNF139	-0.01384	0.639583	-0.08099	-0.27586	-0.31801	-0.23859	-0.17163	-0.12979
RNF14	-0.18101	-0.569	0.281258	-0.28458	-0.04518	-0.61885	0.01226	0.051081
RNF141	0.066917	0.690266	-0.3518	-0.04203	-0.28278	-0.3246	-0.23275	0.074384
RNF144A	NA	NA	-0.00504	-0.41364	-0.32813	-0.21581	NA	NA
RNF144B	NA	NA	0.209206	0.229449	-0.06734	-0.10054	NA	NA
RNF145	NA	NA	0.014317	0.199675	-0.23215	0.240082	NA	NA
RNF146	0.317817	0.5734	-0.12062	0.085533	-0.02421	-0.22963	0.122603	-0.19222
RNF148	-0.22947	0.699813	0.070363	-0.02033	-0.33128	-0.2643	-0.07367	-0.1204
RNF149	-0.15529	0.171673	0.186032	-0.39284	0.215649	-0.15263	-0.34354	0.019857
RNF150	-0.0783	-0.03007	0.039367	0.031151	-0.34216	0.230974	-0.30917	-0.34251
RNF151	NA	NA	-0.19576	-0.08744	0.121862	0.071677	-0.47481	-0.39878
RNF152	0.234921	0.726452	-0.3104	-0.26915	-0.36529	-0.59328	0.10557	0.140249
RNF157	-0.14147	0.534742	0.283748	-0.26367	0.358979	-0.07459	-0.11501	-0.02079
RNF165	0.041161	0.073452	NA	NA	NA	NA	-0.52857	-0.17107
RNF166	-0.04104	-0.30215	0.287005	0.430383	0.318876	0.524136	-0.43531	0.01662
RNF167	-0.0154	0.404935	-0.06033	0.306743	-0.20954	-0.35941	-0.39634	-0.45657
RNF168	-0.22471	-0.35278	-0.31996	-0.16101	-0.11673	-0.30058	-0.30032	0.19315
RNF169	NA	NA	-0.15731	-0.09346	0.055498	0.416492	NA	NA
RNF17	0.222196	0.495275	0.202725	0.125691	0.119733	0.234148	-0.22863	0.207884
RNF170	-0.20118	-0.1328	0.366507	-0.2187	0.002934	-0.56516	0.278451	-0.04212
RNF175	-0.3064	-0.18544	0.157858	0.049203	-0.16982	0.212145	-0.17199	-0.13447
RNF180	-0.46843	-0.15758	-0.07886	-0.07004	-0.11027	0.199001	-0.01563	-0.04356
RNF181	NA	NA	0.322869	-0.21261	0.059009	0.00697	NA	NA
RNF182	0.138043	0.032175	0.341545	-0.06175	0.225529	-0.25936	NA	NA
RNF183	0.134331	0.7343	-0.34649	0.113856	0.057612	0.083517	0.118792	0.077131
RNF185	0.010273	-0.02082	0.261651	-0.23419	0.191534	-0.29502	0.498787	0.3686
RNF186	0.117542	0.241235	-0.09225	0.101688	-0.1647	-0.3376	-0.25927	-0.35695
RNF187	NA	NA	0.27986	0.206044	0.040449	0.123909	NA	NA
RNF19A	NA	NA	-0.06955	-0.12041	-0.4155	0.093581	NA	NA
RNF19B	NA	NA	0.156158	0.009233	-0.00592	-0.18129	NA	NA
RNF2	-0.49768	-0.42578	-0.37337	-0.09198	-0.43646	-0.48925	-0.2299	-0.2955
RNF20	-0.00927	-0.26278	0.136052	0.163534	0.203282	-0.01729	-0.35467	-0.35773
RNF207	-0.28868	0.330537	0.264694	0.077957	0.05485	-0.09643	NA	NA
RNF208	-0.073	0.141493	0.443454	0.012863	0.357295	-0.10187	NA	NA
RNF212	0.005651	-0.52652	-0.23396	0.378462	-0.0305	-0.25393	NA	NA
RNF213	0.402284	0.696111	0.271779	0.026853	-0.07964	0.439568	NA	NA
RNF214	-0.00128	0.013812	-0.21176	0.071724	-0.0938	-0.26364	NA	NA
RNF215	NA	NA	0.010825	0.106398	0.004915	-0.25872	NA	NA
RNF216	NA	NA	0.339148	-0.12275	0.308512	0.587315	NA	NA
RNF217	NA	NA	0.033464	-0.11285	0.125873	0.252658	NA	NA
RNF219	NA	NA	-0.09037	-0.24744	0.191192	0.146387	NA	NA
RNF220	NA	NA	0.263406	0.410888	0.467489	0.325874	NA	NA
RNF24	-0.01811	0.076547	-0.13998	-0.0132	-0.08735	0.149205	0.076623	0.192944
RNF25	0.21053	0.022739	0.133183	0.091888	0.22456	0.119024	0.346663	0.251056
RNF26	0.166561	0.34622	-0.04691	0.313946	0.047585	-0.00029	-0.09437	-0.28521
RNF31	-0.05052	0.162215	-0.03657	-0.15133	0.078905	0.35968	0.070711	0.023349
RNF32	-0.08374	0.334169	-0.07013	0.009864	0.200249	-0.41114	-0.09818	-0.09872
RNF34	-0.20739	0.019171	-0.35124	0.048232	0.451652	0.108222	0.312915	0.319634
RNF38	-0.02771	-0.45432	-0.13724	-0.32244	0.059661	-0.00681	0.301032	0.085633
RNF39	0.308428	-0.0426	0.100673	-0.06917	0.369882	-0.16306	0.304432	-0.01185

RNF4	-0.32912	-0.67857	-0.28844	0.20346	-0.08112	-0.15079	0.254483	0.251735
RNF40	0.12528	0.464232	0.272919	0.323499	0.411667	0.043192	0.411075	0.094125
RNF41	0.164562	0.270864	0.111188	-0.19687	0.157291	0.006274	-0.05559	-0.10472
RNF43	0.562131	-0.12199	0.417226	0.155468	0.57141	0.264759	0.273746	-0.25472
RNF44	0.559785	-0.09094	0.130049	0.111939	0.168601	0.22476	0.427421	0.085384
RNF5	0.128447	-0.12789	-0.09715	-0.03167	-0.0376	-0.32401	0.333036	0.072445
RNF6	-0.00592	0.505231	0.00171	-0.18227	-0.17878	-0.61113	0.446214	0.536818
RNF7	-0.22745	-0.01601	-0.14275	0.043966	-0.02529	-0.11333	-0.33733	-0.23697
RNF8	0.06639	-0.48802	0.082665	0.025498	0.235152	0.102883	0.060672	-0.18302
RNFT1	NA	NA	0.117822	-0.16412	-0.12691	-0.34163	NA	NA
RNFT2	NA	NA	-0.22625	0.468983	0.024534	0.128566	NA	NA
RNGTT	0.200228	0.058247	-0.31217	0.286187	-0.08444	0.339614	-0.26806	-0.24834
RNH1	-0.05396	0.162102	0.203078	0.167919	0.19686	0.204199	-0.01199	0.092052
RNMT	0.246819	0.140948	-0.31637	-0.15478	-0.34998	0.047286	0.187231	0.292188
RNMTL1	0.091534	0.50704	-0.2776	0.188811	-0.38525	0.164615	0.100286	-0.2075
RNPC3	0.156454	-0.53208	0.32551	-0.36714	-0.12164	-0.42934	-0.32915	-0.21099
RNPEP	-0.55333	0.165418	0.172842	0.12209	0.308872	-0.17945	0.029011	-0.2172
RNPEPL1	0.084498	0.756449	0.464649	0.101349	0.346244	-0.02505	0.052049	0.17408
RNPS1	0.200928	-0.0008	-0.10855	0.258531	0.348596	0.246252	0.687863	0.294037
ROBO1	-0.01292	-0.50193	-0.01297	0.493697	0.073737	0.53733	-0.52879	-0.27668
ROBO2	0.263951	0.264438	-0.08204	0.206357	-0.22899	0.030835	0.310508	0.358113
ROBO3	0.161619	-0.03144	-0.20499	-0.09253	-0.37155	0.007741	-0.20546	0.371597
ROBO4	-0.18952	0.04877	0.066627	0.1678	0.113239	0.262637	-0.42587	0.102984
ROCK1	-0.19965	-0.00195	-0.24802	0.117164	-0.23421	0.230514	-0.21892	0.058528
ROCK2	-0.19361	-0.62781	0.313626	-0.19046	0.257227	-0.1145	0.613578	0.319523
ROD1	0.085606	-0.10483	0.142583	-0.12791	0.180551	0.064289	-0.32328	-0.33141
ROGDI	0.000743	0.130774	0.238284	-0.23796	0.170997	-0.05551	NA	NA
ROM1	0.084167	0.42319	-0.05561	0.145369	-0.12207	-0.4491	-0.23336	-0.07276
ROMO1	NA	NA	0.213718	-0.1581	-0.02011	-0.27536	NA	NA
ROPN1	0.003999	0.560983	-0.20843	-0.1319	-0.28147	0.146939	-0.31836	-0.15441
ROPN1B	0.302517	0.770257	-0.1366	-0.03263	-0.08883	-0.00028	-0.25713	-0.268
ROPN1L	-0.2584	0.351085	-0.03394	0.331845	-0.18228	-0.36785	0.29719	0.336957
ROR1	-0.10661	-0.34177	-0.13089	-0.26196	-0.29276	-0.06231	-0.38263	-0.32739
ROR2	0.108413	-0.00193	-0.00845	-0.03078	0.25446	0.034201	-0.32542	-0.34839
RORA	0.005079	0.514577	0.213356	-0.03791	0.016844	0.563083	-0.43204	-0.41713
RORB	0.077703	-0.14307	0.214448	0.087564	-0.25415	0.166245	-0.3527	-0.14279
RORC	-0.29065	0.382502	0.203294	0.205603	0.083118	-0.08746	0.212258	0.039859
ROS1	-0.04352	-0.09192	-0.02577	-0.11354	-0.0247	0.011352	0.026968	-0.03703
RP1	0.018474	0.201257	0.12709	-0.19467	-0.09728	0.121953	0.006511	-0.04047
RP11-529I1	-0.04996	0.451913	-0.21162	-0.32662	0.027873	-0.60674	0.120385	-0.21403
RP1L1	0.26422	0.470056	-0.08604	-0.28207	-0.08104	0.05837	0.413346	0.252141
RP2	-0.03863	-0.38547	-0.33293	-0.28627	-0.13154	-0.19925	0.015881	0.349472
RP9	0.383233	0.633167	0.037606	0.062673	0.101337	0.109332	0.100724	0.053492
RPA1	-0.08699	-0.62623	-0.00392	0.229455	-0.18602	0.241689	-0.23659	-0.08823
RPA2	-0.45137	-0.07213	-0.09461	0.43516	-0.01861	0.271575	0.139961	-0.01388
RPA3	0.161187	-0.18305	-0.1462	-0.22784	-0.08868	-0.18708	0.273085	-0.14386
RPA4	-0.02599	-0.33981	-0.06307	-0.18136	-0.15364	0.095563	-0.00053	-0.18126
RPAIN	0.135445	-0.48561	-0.2734	-0.0148	-0.32587	-0.14184	0.323129	0.574432
RPAP1	0.360757	0.062674	-0.06598	0.258128	-0.17849	0.291763	-0.36814	-0.51514
RPAP2	NA	NA	0.21362	-0.12904	0.053602	0.206373	NA	NA
RPAP3	NA	NA	-0.42071	0.154728	-0.07196	-0.08029	NA	NA
RPE	0.333721	-0.59162	-0.20929	0.004666	-0.34831	-0.53767	0.451446	0.264197

RPE65	0.142404	0.293161	-0.02474	0.102965	-0.00086	-0.29911	-0.07088	-0.0245
RPGR	0.338886	-0.67695	-0.12137	-0.0717	-0.10032	-0.08335	-0.24112	0.000113
RPGRIP1	-0.09088	0.111756	-0.04959	0.279451	-0.11272	0.350674	-0.13069	0.485559
RPGRIP1L	NA	NA	-0.36848	-0.03669	0.279269	-0.04589	NA	NA
RPH3A	0.086007	-0.1659	-0.02893	-0.25154	-0.03424	-0.27026	0.371433	0.085023
RPH3AL	-0.00608	0.277712	0.01274	0.488252	-0.13338	0.173306	0.461571	0.376363
RPIA	0.388918	-0.01303	-0.09827	0.335569	0.472465	0.23479	-0.00836	-0.33007
RPL10	0.001533	0.354919	0.16346	0.218778	0.238764	0.176958	-0.29065	-0.05514
RPL10A	-0.09182	-0.70002	-0.19149	0.198009	-0.00408	-0.27154	0.420867	-0.24391
RPL10L	0.068689	0.469153	0.372277	0.070154	0.286151	0.142213	0.34402	0.039466
RPL11	-0.26006	-0.67963	-0.2717	0.250237	0.083257	0.076565	-0.06139	-0.47565
RPL12	0.34083	-0.48899	0.240502	0.390832	0.119453	-0.1006	0.575832	0.229925
RPL13	0.271872	0.018274	0.215551	0.358896	0.334395	-0.05185	0.117538	-0.45105
RPL13A	0.189348	-0.55062	0.127872	-0.08221	0.118671	0.151761	-0.27957	-0.54626
RPL14	0.195134	-0.19828	-0.21527	0.191908	0.087159	0.164475	0.031514	-0.21823
RPL15	0.119928	-0.56877	-0.11073	-0.09564	0.143169	0.276574	NA	NA
RPL17	-0.04413	-0.59069	0.240291	-0.04293	-0.23042	0.119507	-0.00871	-0.08511
RPL18	NA	NA	-0.06872	0.464743	0.164002	-0.16465	0.835309	-0.08918
RPL18A	NA	NA	0.121914	0.138705	0.038851	0.168999	0.39951	-0.07798
RPL19	0.341102	-0.66255	0.29874	0.159769	0.075735	-0.07737	0.207119	-0.20391
RPL21	NA	NA	0.23594	0.090769	0.188011	-0.08784	-0.49844	-0.58773
RPL22	0.069383	-0.49097	-0.02097	0.128978	0.093625	-0.04895	0.478376	0.232268
RPL22L1	NA	NA	0.054631	0.016356	-0.16243	-0.12816	NA	NA
RPL23	-0.19675	-0.35624	0.262832	-0.13108	0.007831	0.026875	-0.42682	-0.52846
RPL23A	0.290484	0.170943	0.131991	0.080521	-0.15802	-0.12503	-0.08995	-0.42714
RPL23AP7	NA	NA	-0.14512	-0.04981	-0.41055	-0.27962	-0.50873	-0.52209
RPL24	NA	NA	-0.2468	0.028503	-0.17774	-0.16902	-0.27956	-0.2895
RPL26	-0.04149	-0.59796	-0.03106	0.155741	0.118679	0.040098	-0.47529	-0.5158
RPL26L1	0.132609	-0.66499	-0.07491	-0.16273	-0.3455	-0.29185	0.289297	0.252186
RPL27	0.401343	-0.26944	0.075234	-0.16184	-0.088	-0.29468	0.468203	0.112714
RPL27A	-0.05491	-0.33395	-0.21221	0.153953	-0.0404	-0.55977	-0.40863	-0.46408
RPL28	0.211981	0.488777	0.072611	0.286611	0.117908	0.108683	0.064525	0.009286
RPL29	-0.01567	-0.40949	0.019895	0.013715	-0.07262	-0.10344	-0.02212	-0.15446
RPL29P2	NA	NA	0.209501	-0.13877	-0.02405	-0.19577	0.219074	0.083255
RPL3	0.158717	-0.32446	0.251261	0.435225	0.079386	0.297856	-0.34901	-0.64793
RPL30	-0.14662	-0.43221	0.136572	-0.16659	0.038233	-0.12085	-0.03198	-0.33918
RPL31	0.023431	-0.49884	-0.2038	-0.14904	-0.18519	-0.21888	-0.37058	-0.55501
RPL32	-0.27139	0.032162	-0.11606	-0.10691	0.041788	-0.06621	0.365596	0.312348
RPL32P3	NA	NA	-0.13522	-0.19718	0.170729	0.272029	0.362333	0.413452
RPL34	0.16107	-0.50048	-0.02297	-0.13652	-0.07398	-0.03339	0.367953	0.03434
RPL35	NA	NA	0.05661	0.341354	0.000539	-0.01583	-0.46799	-0.48014
RPL35A	0.124042	0.014984	0.183293	-0.22556	0.087147	-0.16154	0.30313	0.107483
RPL36	0.546654	-0.29084	0.045787	0.317318	0.05628	-0.01518	0.185319	-0.2375
RPL36A	0.035271	0.613199	-0.20624	-0.00465	-0.03588	0.086115	-0.10492	-0.19771
RPL36AL	-0.42844	-0.695	-0.11173	0.007751	-0.27149	-0.15318	-0.72282	-0.38718
RPL37	-0.09205	-0.23731	-0.12093	-0.09816	-0.04419	-0.15972	-0.0852	-0.38024
RPL37A	0.121745	0.008017	0.19471	-0.1539	-0.10866	0.121622	-0.48661	-0.52672
RPL38	0.189317	-0.53508	0.212047	0.128826	0.078577	-0.31715	0.673733	0.214596
RPL39	0.371784	-0.50637	0.09701	-0.24064	0.018296	-0.18772	0.198345	-0.17955
RPL39L	-0.25664	-0.27147	0.101454	0.092077	-0.01419	0.002727	-0.44421	-0.27459
RPL3L	0.298855	0.422926	0.118595	-0.40484	0.044702	-0.16953	0.05329	0.257534
RPL4	-0.12988	0.11537	-0.19041	0.217054	0.115093	0.159253	0.1741	-0.20848

RPL41	-0.20928	-0.37645	-0.24637	0.010861	-0.01626	-0.3375	0.012285	-0.20923
RPL5	0.348377	0.379653	0.278113	0.23514	0.026311	-0.02564	-0.41763	-0.59481
RPL6	0.154116	0.350512	-0.2327	0.137956	-0.00653	-0.32192	0.383472	0.231942
RPL7	0.198573	-0.61128	0.03022	-0.02394	-0.17113	-0.28491	-0.32636	-0.60818
RPL7A	0.167161	-0.37303	-0.02098	0.473612	0.228639	-0.20206	-0.41223	-0.60959
RPL7L1	NA	NA	-0.05698	-0.04946	0.06549	0.290854	-0.52344	-0.56556
RPL8	0.010197	-0.58167	0.24298	0.33912	0.072072	0.102415	0.539326	0.04716
RPL9	0.10745	-0.56994	-0.1	-0.06681	-0.05655	-0.00601	-0.29342	-0.44904
RPLP0	-0.20853	-0.65498	0.165179	0.150379	0.251687	0.084856	0.342933	0.110628
RPLP0P2	NA	NA	-0.12	-0.1804	0.304617	-0.12795	0.080097	0.02227
RPLP1	NA	NA	-0.14768	0.26431	-0.06784	-0.0857	-0.51418	-0.51776
RPLP2	0.342203	0.406322	-0.13133	0.251591	-0.11346	-0.01014	0.501064	-0.19214
RPN1	-0.22264	-0.09101	-0.09743	0.302568	-0.12946	-0.38311	0.34094	0.346378
RPN2	0.329739	-0.23416	0.268607	0.187126	0.279671	-0.02795	-0.13312	-0.41922
RPP14	0.114735	0.460029	-0.1907	-0.16955	0.059543	0.084094	-0.34805	-0.04187
RPP21	0.344665	0.376671	-0.02086	0.04942	-0.08329	-0.15632	0.13485	-0.11602
RPP25	0.158619	0.056656	0.02578	0.106138	0.252326	-0.09199	0.228415	-0.00402
RPP30	-0.07408	0.102439	-0.17166	-0.24575	-0.13558	-0.26129	0.49325	0.309683
RPP38	-0.26119	-0.59105	-0.19456	0.094903	-0.2354	-0.1729	0.433395	0.2435
RPP40	0.369803	0.25828	-0.24538	0.016447	-0.10295	-0.01636	0.14193	-0.18574
RPRD1A	NA	NA	-0.35697	-0.30874	-0.13716	-0.54132	NA	NA
RPRD1B	NA	NA	-0.07765	-0.11927	0.096551	-0.3893	NA	NA
RPRD2	NA	NA	0.102347	0.279577	0.145083	0.248434	NA	NA
RPRM	0.145829	-0.0454	-0.18012	-0.0615	-0.08656	-0.02396	-0.346	-0.24905
RPRML	0.341847	0.788999	-0.31966	-0.02331	0.06264	-0.25144	-0.06961	0.027721
RPS10	0.198982	0.69285	-0.21958	-0.18119	0.009464	-0.11566	0.462711	-0.18708
RPS11	0.233657	-0.3713	-0.2444	-0.04807	0.189303	-0.06517	0.006129	-0.25739
RPS12	0.292723	-0.46587	-0.15232	-0.16023	0.084621	-0.08053	0.786028	0.16374
RPS13	0.272471	-0.59302	0.001718	-0.17805	0.019775	-0.03297	-0.04496	-0.28528
RPS14	0.325916	0.033261	0.129573	0.093962	-0.07333	-0.10183	-0.15272	-0.36284
RPS15	-0.02541	-0.30514	-0.06994	0.424549	0.172243	-0.14637	-0.20946	-0.28815
RPS15A	0.205416	-0.61314	-0.12316	-0.12666	0.107535	0.030056	0.178637	-0.23205
RPS16	0.025496	0.260902	0.20912	0.24537	0.129743	0.205725	0.73894	-0.00126
RPS17	0.13013	-0.61987	-0.26038	0.351406	0.057766	-0.47887	0.688822	-0.08749
RPS18	0.054461	-0.17845	0.036481	-0.02294	-0.02373	-0.18976	NA	NA
RPS19	0.126563	0.645124	0.156233	0.099131	-0.09482	-0.0382	-0.18559	-0.56959
RPS19BP1	0.085582	0.512732	0.143909	0.174551	0.138542	-0.16499	-0.14334	-0.29396
RPS2	NA	NA	-0.27105	0.278096	0.242284	-0.14559	0.141668	-0.35892
RPS20	0.141453	-0.66309	0.306486	-0.1734	0.170658	0.021316	-0.44478	-0.37469
RPS21	0.357459	-0.61053	-0.09146	-0.10615	0.20193	-0.09222	0.094722	-0.11837
RPS23	0.007891	0.029779	0.237796	0.140359	0.207551	-0.18135	-0.1712	0.124209
RPS24	0.114778	0.41729	0.152939	-0.0303	0.337607	0.031347	-0.23571	-0.33535
RPS25	-0.09024	-0.63435	0.013925	0.002428	-0.12812	-0.13988	0.686266	0.29233
RPS26	0.26714	0.030645	-0.38557	-0.06324	-0.14532	-0.38992	-0.02281	-0.09526
RPS27	NA	NA	0.02441	0.109715	0.163182	0.062978	0.809746	0.450969
RPS27A	-0.01766	0.27969	-0.14251	-0.04591	-0.17697	-0.31646	0.778855	0.373996
RPS27L	-0.04432	-0.575	0.005142	-0.2021	-0.26323	-0.17358	0.443665	0.112294
RPS28	0.023182	0.247275	0.138987	-0.1003	-0.07442	0.051515	-0.44975	-0.39545
RPS29	0.173774	0.71002	-0.11851	-0.08194	-0.12479	-0.24019	0.049702	-0.1125
RPS3	0.224633	-0.4282	0.133982	0.240688	0.016009	0.148696	0.405664	-0.19257
RPS3A	NA	NA	-0.30822	0.023363	-0.1931	0.008316	-0.33708	-0.55483
RPS4X	0.043386	-0.6827	0.129921	0.14474	0.272253	0.148445	0.269453	0.121441

RPS4Y1	0.132689	0.193937	0.086562	0.02048	-0.1431	-0.2282	0.261107	-0.13727
RPS4Y2	NA	NA	0.076343	0.023514	-0.14109	-0.22529	0.157297	-0.20411
RPS5	0.284961	-0.34008	-0.06961	0.409008	0.14277	-0.29162	0.276464	-0.25863
RPS6	-0.19789	-0.69293	-0.13589	0.307318	0.110264	-0.16932	-0.40305	-0.57451
RPS6KA1	-0.1027	0.382139	0.175373	0.157896	0.301704	0.007067	0.030848	0.107803
RPS6KA2	-0.06267	0.149137	0.215446	0.24268	0.277225	0.146253	0.182114	0.200602
RPS6KA3	0.291904	0.42482	-0.0969	-0.11981	0.22728	0.327901	-0.21482	-0.0273
RPS6KA4	0.258454	0.366166	-0.05718	0.1352	0.160324	0.01293	-0.22321	-0.04916
RPS6KA5	0.185044	0.076413	-0.18182	-0.4241	-0.12262	0.030094	-0.38914	-0.38597
RPS6KA6	0.245797	0.140748	-0.01729	-0.10505	-0.19505	-0.33412	-0.321	-0.07942
RPS6KB1	-0.28969	-0.04349	0.067834	-0.25873	-0.24001	0.271579	-0.24246	0.001134
RPS6KB2	-0.13638	0.06387	0.186164	0.081896	0.065236	-0.2923	-0.22454	-0.30003
RPS6KC1	-0.07834	-0.46919	0.027729	-0.27284	-0.44355	0.093648	-0.43006	-0.15662
RPS6KL1	0.422847	0.680775	-0.01053	0.04222	0.479164	0.163323	0.468433	0.310489
RPS7	0.281502	0.683317	-0.04206	-0.07555	0.04413	-0.16475	-0.18579	-0.28249
RPS8	0.233604	0.490823	-0.14624	0.145143	0.111026	-0.05427	NA	NA
RPS9	0.12887	-0.58496	-0.15777	0.024766	0.051448	-0.24	0.446088	-0.13765
RPSA	-0.0086	-0.54971	-0.34662	0.069656	0.079607	-0.20446	0.170634	-0.36547
RPTN	NA	NA	0.005266	-0.45996	-0.13151	-0.15611	NA	NA
RPUSD1	0.376339	0.733907	0.296788	0.162647	0.336748	0.157321	0.568798	0.275386
RPUSD2	0.402351	0.634558	-0.31146	0.086572	-0.20691	-0.05992	0.279485	0.1333
RPUSD3	0.353948	0.641853	0.100973	0.124947	0.202359	0.155193	0.350126	-0.27066
RPUSD4	0.001006	0.181285	-0.10802	0.379906	-0.00943	0.201393	0.045826	-0.0547
RQCD1	-0.01134	-0.34834	-0.16813	0.123049	-0.0576	-0.22322	0.438661	0.430647
RRAD	-0.31143	-0.12748	0.000475	-0.06237	-0.16881	0.230844	-0.32483	-0.33321
RRAGA	0.118532	-0.33737	0.054241	-0.06324	-0.10102	0.227193	0.197212	0.199921
RRAGB	-0.018	-0.10484	-0.32988	-0.26482	0.056694	-0.26462	-0.12978	-0.10933
RRAGC	-0.3114	0.575107	0.145414	-0.2531	-0.03677	0.042259	-0.44744	-0.35639
RRAGD	0.015825	0.141441	0.05239	0.060906	-0.25043	-0.17901	-0.18444	-0.27929
RRAS	0.07566	0.188823	0.236126	-0.02062	-0.068	-0.06017	-0.17842	-0.25129
RRAS2	0.196458	-0.43995	-0.4653	0.169895	-0.09783	0.03155	-0.56433	0.061278
RRBP1	0.085299	0.260838	0.097281	0.263215	0.322274	0.265783	0.336565	-0.06913
RREB1	-0.17281	0.141232	0.113264	-0.06208	0.219288	0.124125	0.609004	0.460226
RRH	0.08333	0.250202	-0.1818	-0.30014	0.073373	-0.0894	0.42505	0.609869
RRM1	-0.23523	0.11171	-0.32655	0.077245	0.293015	-0.20577	0.376275	0.192595
RRM2	-0.3309	-0.04176	-0.15484	0.369755	0.235371	-0.14002	0.348143	0.394505
RRM2B	-0.11049	-0.59984	0.378911	-0.24799	-0.12818	-0.25657	-0.29013	-0.21309
RRN3	0.099776	-0.04637	-0.26297	0.219254	0.090177	0.236522	-0.33606	-0.43244
RRP1	NA	NA	-0.00846	0.308172	0.146093	0.316076	NA	NA
RRP12	0.265511	-0.07248	-0.18654	0.352265	0.442894	0.298149	NA	NA
RRP15	0.198444	0.140319	-0.23277	-0.07879	-0.00968	0.127335	NA	NA
RRP1B	NA	NA	-0.22068	0.241123	-0.14358	0.057678	NA	NA
RRP7A	NA	NA	0.325382	0.269584	-0.08866	0.199191	NA	NA
RRP9	0.513482	0.544301	0.209899	0.283557	0.425312	0.174219	NA	NA
RRS1	0.274453	-0.32589	-0.02864	0.235868	0.152565	0.268792	0.43547	0.217154
RS1	-0.01092	0.599713	-0.13362	-0.04873	-0.20102	0.127451	-0.09243	-0.02846
RSAD1	0.010538	-0.36407	0.449573	0.214897	-0.02244	0.134864	-0.58697	-0.30186
RSAD2	-0.30999	-0.31429	0.041727	-0.31129	-0.43279	-0.00554	-0.20326	0.137028
RSBN1	-0.18993	-0.32312	-0.27464	-0.32277	-0.18314	-0.26371	0.181353	-3.5E-05
RSBN1L	0.191738	-0.20519	0.195267	-0.21619	-0.11452	0.075153	-0.40537	-0.41418
RSC1A1	-0.18969	-0.51753	-0.06026	-0.27572	-0.10933	-0.04971	0.333148	0.386293
RSF1	-0.11255	-0.27	-0.31437	-0.05756	0.011262	0.234355	NA	NA

RSHL1	0.243357	0.344776	-0.0826	-0.29925	-0.05503	-0.06424	0.144406	-0.06818
RSHL3	-0.22833	0.132811	-0.24514	-0.16783	-0.17277	0.134284	0.292906	0.44622
RSL1D1	0.063814	-0.11058	-0.42987	0.139325	0.229352	0.211718	0.454558	0.311456
RSPH1	NA	NA	-0.09075	0.078575	0.10384	-0.49462	NA	NA
RSPH3	NA	NA	-0.32836	-0.12672	-0.18455	-0.50863	NA	NA
RSPO1	0.170375	-0.21646	0.050971	0.349109	-0.14402	-0.10505	-0.35144	-0.14152
RSPO2	-0.01582	-0.38749	0.051078	0.117193	-0.06713	0.14803	0.208688	0.207038
RSPO3	-0.03117	-0.24324	-0.12954	0.251694	-0.08604	-0.11433	-0.38022	0.244877
RSPO4	0.105508	0.685023	NA	NA	NA	NA	-0.61228	-0.23311
RSPRY1	-0.18774	-0.26171	-0.10206	-0.11497	0.126507	-0.22816	0.600855	0.310254
RSRC1	-0.06693	-0.46805	-0.48543	-0.09801	-0.19633	-0.44555	0.261516	0.109868
RSRC2	-0.3155	-0.63489	-0.33882	0.19918	0.042647	-0.2549	-0.6209	-0.43132
RSU1	-0.03727	-0.44006	-0.06557	-0.36916	-0.00634	0.000576	-0.59701	-0.61663
RTBDN	0.16639	-0.2081	0.146202	-0.16402	0.102159	-0.16122	0.06306	-0.38722
RTCD1	-0.04514	-0.62265	0.056244	0.062294	0.206264	-0.55437	-0.46901	-0.38384
RTDR1	0.043412	-0.02177	0.325038	-0.24436	0.308717	-0.11352	0.429734	0.212699
RTEL1	0.1123	-0.0789	0.153162	0.238983	0.164053	0.066748	-0.1165	-0.11677
RTF1	-0.08787	0.43391	-0.11842	-0.01901	-0.08505	0.075401	0.331318	0.375706
RTKN	0.220133	0.55007	0.440078	0.10357	0.484723	0.227998	0.554259	0.287916
RTKN2	NA	NA	-0.27938	0.056244	-0.17623	-0.28066	NA	NA
RTN1	0.099225	-0.43787	0.033657	-0.05747	-0.1485	0.423444	0.141871	0.112948
RTN2	0.048742	0.187178	-0.07359	-0.00668	-0.24667	-0.07124	-0.16126	-0.34268
RTN3	-0.43275	-0.20156	-0.1834	0.041485	-0.13655	-0.06559	0.413686	0.198646
RTN4	0.018933	-0.5354	0.267165	-0.22837	-0.11331	-0.17549	-0.37033	0.18103
RTN4IP1	-0.30487	-0.16631	-0.06555	-0.10636	0.234631	-0.15819	-0.02021	-0.17001
RTN4R	-0.26296	0.765926	0.108939	0.330309	0.111229	-0.00926	0.143731	-0.1686
RTN4RL1	0.043099	0.500919	0.124997	-0.07488	0.003409	-0.31938	-0.35918	-0.19581
RTN4RL2	0.020282	0.113398	0.206186	-0.17147	-0.08651	-0.29342	0.548356	0.044088
RTP1	0.106409	0.451206	0.155529	0.053766	0.166712	-0.13872	0.323031	0.279207
RTP2	0.083703	0.610553	NA	NA	NA	NA	-0.13266	-0.27325
RTP3	-0.57623	-0.36494	0.090387	0.064757	-0.02944	0.100231	-0.20135	-0.33967
RTP4	0.205129	0.330384	-0.07778	-0.20492	-0.21089	-0.46594	-0.06178	0.210391
RTTN	0.038114	-0.20356	-0.28575	-0.07287	0.099478	-0.04826	NA	NA
RUFY1	-0.05235	0.067708	0.215992	0.120006	0.10049	0.124852	0.374217	0.351642
RUFY2	-0.01	-0.2823	-0.06578	-0.24019	0.022578	-0.01311	-0.24818	-0.27069
RUFY3	-0.10244	-0.46872	0.084403	-0.07218	0.130679	0.254735	-0.5008	-0.22573
RUFY4	-0.00999	0.619262	NA	NA	NA	NA	NA	NA
RUNDC1	-0.19548	-0.1045	0.178897	0.018948	0.358685	-0.32644	0.396778	0.219533
RUNDC2A	-0.15918	0.389305	0.264433	0.186136	0.039715	0.644232	-0.19571	-0.12653
RUNDC3A	NA	NA	0.090424	0.012441	-0.23997	-0.1436	NA	NA
RUNDC3B	NA	NA	0.08554	-0.5247	-0.017	-0.14543	NA	NA
RUNX1	0.006336	-0.0484	0.176921	-0.19702	-0.0379	0.251918	0.089465	0.445162
RUNX1T1	0.041531	0.029952	0.138249	0.107468	-0.16522	0.102378	-0.02049	-0.03386
RUNX2	-0.03941	-0.15327	0.16	0.327747	-0.09444	0.369303	0.22355	-0.23751
RUNX3	-0.42645	0.130661	0.312875	0.685281	0.052509	0.632408	-0.04764	0.159806
RUSC1	-0.36732	0.483553	0.411383	0.078952	0.093876	-0.12533	0.365919	0.382996
RUSC2	0.148161	0.078349	0.310753	-0.08057	0.307017	-0.08711	-0.41111	-0.17082
RUVBL1	0.305982	-0.46721	-0.17556	0.504634	0.231892	-0.02817	0.326633	0.116312
RUVBL2	0.321195	-0.38565	0.002967	0.310142	0.304697	0.181672	0.544391	-0.13804
RWDD1	0.018508	-0.07997	-0.05377	-0.20525	0.178668	-0.38472	0.031879	0.227623
RWDD2A	NA	NA	0.126962	-0.12316	0.069653	-0.51521	NA	NA
RWDD2B	NA	NA	-0.15807	0.151755	-0.30762	-0.28053	NA	NA

RWDD3	-0.11418	-0.4555	0.237739	-0.32285	0.109562	-0.24704	NA	NA
RWDD4A	0.047264	0.38829	-0.22361	-0.07885	0.206862	-0.274	0.271189	-0.02239
RXFP1	0.280637	0.189803	-0.10775	0.055263	-0.10398	-0.32757	NA	NA
RXFP2	0.397648	0.445323	0.227415	0.062784	-0.47629	0.135308	NA	NA
RXFP3	0.11839	0.514241	0.103756	-0.26678	0.076413	-0.08337	NA	NA
RXFP4	0.204	0.250987	-0.10092	0.003968	0.047713	0.072609	NA	NA
RXRA	-0.48419	-0.13343	0.207808	-0.09673	0.264907	0.099037	0.035938	0.125474
RXRB	-0.09291	0.46762	0.078517	0.338572	0.202434	0.090586	-0.50754	-0.44576
RXRG	0.359385	0.648487	-0.0407	-0.01552	0.151756	-0.28521	-0.16388	-0.3549
RYBP	-0.10547	0.479291	-0.10049	-0.05856	-0.17098	-0.12189	-0.15839	-0.41713
RYK	-0.06575	-0.16872	-0.2915	0.010645	-0.04485	0.091138	0.413222	0.121547
RYR1	0.279283	0.099818	-0.06548	0.188208	-0.09305	0.355809	-0.18362	0.103607
RYR2	0.299234	0.287416	-0.40851	-0.14362	-0.26958	0.132412	-0.35571	-0.11604
RYR3	0.142986	0.728858	0.30825	-0.04548	-0.15919	0.012371	-0.31622	-0.18024
S100A1	-0.05987	0.134105	-0.14979	0.390825	-0.10337	0.141901	-0.1333	-0.13155
S100A10	-0.19637	-0.18074	-0.3087	-0.09565	-0.41046	-0.31554	0.563998	0.208993
S100A11	-0.01579	0.217962	0.122219	0.263247	-0.27973	0.020533	-0.1381	-0.02514
S100A12	-0.45444	-0.11262	0.105829	0.088994	0.011816	-0.40307	-0.296	0.044592
S100A13	-0.18606	-0.29161	-0.24189	-0.24049	-0.44172	-0.21837	-0.30451	-0.3055
S100A14	-0.21853	0.10665	-0.24669	0.126584	-0.26558	-0.47715	-0.03874	-0.27277
S100A16	-0.29106	0.194079	-0.14995	0.085921	-0.35125	0.14433	0.351168	0.191255
S100A2	0.211434	0.317372	-0.31749	-0.18704	-0.25085	-0.04389	-0.08887	-0.67537
S100A3	-0.17998	0.032372	-0.0974	0.194331	-0.15093	0.431457	-0.23321	-0.37464
S100A4	-0.28196	-0.62102	-0.04313	0.02733	-0.01212	0.083293	-0.00492	0.000733
S100A5	-0.00277	0.057935	0.012585	-0.14445	-0.25353	-0.03401	-0.34864	-0.45425
S100A6	-0.04758	0.458693	-0.43424	0.04323	-0.24341	-0.33279	-0.45955	-0.54543
S100A7	-0.10635	-0.36949	0.081084	-0.1022	0.032518	-0.24937	-0.23427	0.257863
S100A7A	-0.09927	-0.09398	-0.08592	0.1031	-0.09729	-0.3497	NA	NA
S100A7L2	NA	NA	NA	NA	NA	NA	-0.57117	-0.46317
S100A8	-0.32689	0.051751	0.096492	0.094147	0.070575	-0.27321	-0.534	-0.01387
S100A9	-0.24727	0.274944	0.043413	0.030666	-0.12537	-0.29246	-0.4489	0.017857
S100B	0.257552	-0.20751	0.031731	0.055497	-0.05149	-0.03681	-0.21324	-0.53823
S100G	0.052904	0.075753	0.087987	0.179622	0.081684	-0.03909	-0.38788	0.231291
S100P	-0.50557	0.041008	-0.127	0.131583	-0.01573	-0.14012	0.018729	-0.04147
S100PBP	-0.22694	-0.50599	0.210329	0.002646	0.188848	-0.13773	-0.64077	-0.41285
S100Z	-0.13114	0.556134	-0.20383	0.238577	-0.34433	0.046748	-0.16292	0.032235
S1PR1	NA	NA	-0.00083	0.455595	-0.12147	0.43088	NA	NA
S1PR2	NA	NA	0.187769	0.496966	-0.08213	0.331523	NA	NA
S1PR3	NA	NA	0.219438	-0.03996	0.319844	0.038399	NA	NA
S1PR4	NA	NA	0.237033	0.713676	-0.1009	0.410219	NA	NA
S1PR5	NA	NA	0.115527	-0.08933	0.040682	0.47147	NA	NA
SAA1	-0.22214	-0.06983	0.279176	0.09039	0.217882	-0.04222	-0.14321	0.19239
SAA2	-0.13694	-0.12864	0.310489	0.073773	0.163735	-0.1749	-0.14658	0.152331
SAA3P	NA	NA	-0.05093	-0.22785	0.00912	0.001132	NA	NA
SAA4	0.031059	0.169121	0.427796	0.056188	0.20673	0.179918	-0.30863	0.249314
SAAL1	0.26356	0.045342	-0.2436	0.184744	-0.14801	-0.18448	0.33604	-0.18375
SAC3D1	0.015707	0.386182	0.114298	0.316315	0.195143	-0.2156	NA	NA
SACM1L	0.1548	-0.19599	-0.23158	-0.07985	-0.05777	-0.31131	-0.46433	-0.23851
SACS	-0.0838	-0.2739	-0.20587	0.159579	-0.07585	0.514693	-0.46138	-0.13922
SAE1	0.151764	-0.15598	-0.32538	0.005464	0.112858	-0.24301	0.364358	-0.11571
SAFB	0.299738	0.23326	-0.08997	0.476643	0.276623	0.232293	-0.02133	0.107572
SAFB2	-0.14376	0.19764	-0.24505	0.437125	0.023409	0.205766	-0.13145	-0.07057

SAG	-0.21728	-0.3096	-0.4124	-0.23594	-0.21778	-0.3073	-0.54592	-0.22371
SAGE1	0.306902	0.149139	0.149078	-0.11232	0.101585	-0.19479	-0.28002	-0.23429
SALL1	-0.07508	-0.36906	-0.18249	-0.06238	-0.14398	0.109132	-0.17923	-0.14722
SALL2	-0.02774	-0.12682	-0.07502	0.036224	-0.05165	0.144286	-0.13938	-0.0626
SALL3	-0.05195	0.016032	0.342186	0.139524	0.086633	0.216329	-0.26805	-0.70721
SALL4	0.231317	-0.16614	0.049767	0.026363	0.013866	0.246613	0.169876	0.26352
SAMD1	0.286694	-0.4545	0.122608	0.389179	0.36735	0.000581	0.183764	-0.13935
SAMD10	0.300719	0.696698	-0.16414	-0.14967	0.09762	-0.01208	0.491408	-0.03324
SAMD11	0.069474	0.220524	0.356288	0.358396	-0.09088	0.070894	0.450984	0.415046
SAMD12	-0.37669	-0.23245	-0.13438	0.068078	-0.1305	-0.17986	-0.29709	-0.1762
SAMD13	-0.02483	-0.08306	-0.11216	-0.19561	-0.18855	-0.31337	0.353565	-0.15732
SAMD14	-0.21081	0.632066	0.247002	0.153729	0.309536	0.281476	-0.12563	0.129703
SAMD3	0.261468	-0.78008	0.097431	0.297827	-0.27218	0.095215	0.02448	0.148759
SAMD4A	0.133692	0.1666	-0.02914	-0.18093	0.044545	0.136512	-0.20629	0.009557
SAMD4B	-0.41633	-0.52597	-0.03053	-0.10954	0.23867	0.342815	0.289925	0.113097
SAMD5	NA	NA	0.19606	0.000383	-0.04171	-0.09454	NA	NA
SAMD7	0.188155	0.420832	0.024367	0.023482	0.134213	-0.10799	-0.53583	-0.37726
SAMD8	0.233851	0.123439	-0.11423	-0.29412	0.087879	-0.07315	-0.33843	-0.13367
SAMD9	-0.10702	0.044653	-0.12894	-0.16176	-0.45466	-0.20049	-0.30511	0.020124
SAMD9L	-0.19765	-0.12281	0.028576	-0.113	-0.49401	-0.05645	-0.18799	0.587149
SAMHD1	0.334511	-0.19079	0.00415	-0.16949	-0.24435	0.150694	-0.21534	-0.34506
SAMM50	0.211146	0.744141	0.28635	0.16023	0.068177	0.150814	0.396897	0.3699
SAMSN1	-0.6085	-0.09151	0.07604	0.380811	-0.06935	0.393775	-0.5832	-0.10186
SAP130	0.352493	0.024428	0.11993	-0.18272	0.184611	0.081377	0.673411	0.313949
SAP18	-0.3672	-0.34137	0.043695	-0.42127	0.198411	-0.33989	0.208548	-0.15921
SAP30	-0.16415	-0.28165	-0.41008	0.079693	0.299604	-0.4653	-0.39451	-0.51938
SAP30BP	0.022898	0.598091	-0.07533	-0.00102	-0.29543	-0.16118	0.162459	0.269966
SAP30L	0.475194	0.586599	0.274757	-0.43749	0.164238	-0.37997	NA	NA
SAPS1	0.124458	0.66837	-0.12807	0.406388	-0.17843	-0.02632	0.152815	0.269367
SAPS2	0.325752	0.75669	0.160512	-0.06663	0.279927	0.022807	-0.11869	0.218122
SAPS3	-0.27934	-0.39404	-0.31933	-0.20379	0.071742	-0.08645	0.392013	0.310134
SAR1A	-0.11954	-0.4687	-0.22304	-0.0952	0.080834	-0.06877	-0.64616	-0.55936
SAR1B	-0.02872	0.167575	0.114455	-0.19104	-0.21594	-0.51443	-0.07112	0.18192
SARDH	0.273557	0.656541	0.448675	-0.26154	-0.01363	0.376	0.256814	0.374753
SARM1	0.340895	0.234746	0.21217	0.186763	0.149351	0.003466	-0.1147	0.35045
SARS	-0.01764	0.559693	0.4013	-0.01222	0.277598	-0.03203	-0.31011	-0.57367
SARS2	0.159999	0.621946	0.244521	0.239847	-0.03277	0.231648	0.199875	0.032688
SART1	0.072766	-0.09499	0.193682	0.379108	0.446276	0.351869	-0.51215	-0.15997
SART3	-0.103	-0.21675	-0.10703	0.104305	0.424214	0.060278	0.151253	0.061703
SASH1	-0.36654	-0.51714	0.078294	-0.12524	-0.11735	0.308753	-0.46428	-0.27165
SASH3	NA	NA	0.313917	0.821159	-0.13481	0.709221	NA	NA
SASS6	-0.06139	0.134536	-0.14855	0.131238	0.207277	-0.2072	0.244944	0.256717
SAT1	0.272615	-0.3599	-0.0516	-0.2534	0.008166	-0.1342	NA	NA
SAT2	-0.0332	-0.37621	0.161494	0.241989	-0.21363	-0.46616	-0.28624	0.139926
SATB1	0.39077	-0.55252	-0.21158	-0.09406	-0.12439	0.432721	-0.20466	-0.52018
SATB2	0.376566	-0.02771	-0.44298	-0.14902	0.185104	0.047883	0.318733	0.042967
SATL1	0.133838	0.64683	NA	NA	NA	NA	0.540475	0.236654
SAV1	-0.22237	-0.54224	-0.33036	0.114417	-0.3325	-0.16715	0.091925	0.312402
SBDS	-0.21558	-0.54329	-0.22673	-0.21357	-0.10509	-0.51427	-0.16893	-0.18382
SBF1	-0.18498	0.501891	0.205915	0.152429	0.333849	0.207626	-0.00075	0.006575
SBF2	-0.06685	0.177362	0.187661	-0.559	0.07362	0.059954	0.553081	0.03377
SBK1	0.208829	0.348141	0.175985	-0.20247	0.315977	-0.01331	0.42459	-0.11222

SBK2	NA	NA	0.2664	-0.11983	0.05226	0.195991	NA	NA
SBNO1	-0.14631	-0.35112	-0.33007	0.086704	0.002625	-0.0753	-0.15721	0.075227
SBNO2	0.046452	0.507716	0.271475	-0.01602	0.287678	0.260199	NA	NA
SBSN	-0.04735	-0.04093	NA	NA	NA	NA	-0.13456	-0.14846
SC4MOL	-0.21178	-0.09343	-0.05638	0.019236	0.019811	0.078654	0.145223	0.257892
SC5DL	0.136549	0.770529	-0.32202	-0.10939	-0.24235	-0.23207	0.124224	0.038439
SC65	0.07566	0.064823	0.056342	0.114606	0.297174	-0.17656	0.28426	0.171681
SCAF1	NA	NA	0.115814	0.264732	0.268002	0.4099	NA	NA
SCAMP1	0.130194	0.357354	0.019268	-0.26045	-0.14515	-0.21772	0.262149	0.016851
SCAMP2	0.056323	0.576063	0.181184	0.160023	-0.00117	0.214961	0.254926	0.39417
SCAMP3	-0.04575	0.582913	0.378559	0.225687	0.081867	-0.07221	0.472616	0.33506
SCAMP4	0.126805	-0.19184	0.211137	0.010977	0.370821	0.337147	-0.31283	-0.31408
SCAMP5	-0.27067	0.123523	0.288868	-0.12497	0.036551	-0.16572	-0.01825	0.424413
SCAND1	0.344125	-0.19526	0.390292	-0.19976	0.269922	-0.15941	0.363633	0.074846
SCAND2	0.386294	-0.51427	-0.07837	-0.00118	0.200193	0.176527	NA	NA
SCAND3	NA	NA	-0.07793	-0.12945	-0.25598	-0.12388	NA	NA
SCAP	0.279507	0.399089	0.224988	0.205351	0.635892	0.222949	-0.32473	-0.41184
SCAPER	NA	NA	0.078773	-0.50301	-0.1391	-0.22154	NA	NA
SCARA3	0.125555	0.014526	0.419552	0.353833	0.031597	0.319049	-0.28698	-0.12714
SCARA5	0.308649	0.533462	0.212419	-0.07923	-0.03419	0.163328	0.110067	0.264643
SCARB1	0.21389	0.112423	0.188673	-0.00539	0.381716	0.038349	0.492201	0.106408
SCARB2	-0.33685	-0.21875	0.301173	-0.14893	-0.31641	-0.0968	0.132665	0.36026
SCARF1	-0.1954	0.48922	0.005458	-0.03886	0.014815	0.185244	-0.2834	-0.02978
SCARF2	-0.0763	0.050799	0.160816	-0.09062	-0.13941	0.226934	-0.24757	-0.33069
SCCPDH	-0.02072	0.312712	-0.17029	-0.26912	-0.15975	-0.12763	0.203209	0.090209
SCD	0.083405	-0.37871	-0.01412	0.030617	0.04397	0.067163	0.234228	-0.02075
SCD5	0.026829	0.443436	0.056834	-0.0272	0.095273	-0.21429	0.379101	-0.27179
SCEL	-0.15343	-0.225	-0.4975	-0.22078	-0.26632	-0.38058	0.463662	0.016935
SCFD1	-0.29562	-0.35861	-0.43349	-0.21959	-0.2692	-0.36945	-0.36436	-0.03638
SCFD2	0.175734	0.206766	0.108625	0.124335	-0.09045	-0.32058	-0.27738	-0.43633
SCG2	-0.18978	-0.36309	-0.37929	-0.07049	-0.23825	-0.27807	-0.16482	-0.17724
SCG3	0.171522	0.012515	-0.23798	0.148584	-0.11191	-0.28711	0.363208	0.044617
SCG5	-0.02545	-0.3367	0.033241	0.19544	-0.43686	-0.39821	-0.11148	0.131029
SCGB1A1	0.139212	0.232074	0.052293	-0.05672	-0.26087	-0.13927	-0.40782	-0.14142
SCGB1C1	0.380079	0.511829	-0.13718	-0.26559	-0.24817	-0.15544	0.01111	-0.27212
SCGB1D1	0.049794	0.4258	-0.0535	-0.00454	0.164715	0.264689	-0.09528	-0.45577
SCGB1D2	0.262215	0.199529	-0.00189	-0.0526	0.096669	0.263678	0.282576	0.023898
SCGB1D4	0.03641	0.450449	NA	NA	NA	NA	0.305008	-0.06655
SCGB2A1	-0.14717	0.04559	-0.17512	0.081284	-0.19152	-0.39288	0.150619	-0.14345
SCGB2A2	-0.01735	0.280874	-0.005	-0.08472	-0.17289	-0.15085	0.091868	-0.08893
SCGB3A1	0.424929	-0.49537	-0.2126	0.210895	0.090716	0.16637	-0.12583	-0.23603
SCGB3A2	0.201004	-0.07152	-0.47735	-0.13692	-0.32032	0.053407	-0.30252	-0.12721
SCGN	-0.11101	-0.18595	-0.20977	-0.16134	-0.18946	-0.25649	0.499684	0.007785
SCHIP1	-0.51477	-0.42765	-0.10781	-0.1586	0.007128	0.280991	-0.38281	-0.20383
SCIN	-0.17958	-0.06086	-0.08598	-0.24195	-0.10838	-0.3788	0.10516	0.303312
SCLT1	-0.23674	-0.29901	-0.12795	0.031182	-0.40743	-0.2039	NA	NA
SCLY	0.579604	0.068247	0.296688	0.237307	0.204213	0.164136	0.394968	0.573314
SCMH1	0.001018	0.07962	0.326024	-0.08309	0.447832	0.070235	-0.2749	-0.24341
SCML1	0.411096	-0.59982	0.019382	-0.28794	0.361132	-0.13093	0.415546	0.481795
SCML2	0.215716	0.457139	-0.24757	0.145797	-0.11112	0.338227	0.28341	0.255509
SCML4	0.213418	-0.0927	-0.00731	0.539984	-0.11853	0.432386	0.378217	0.362784
SCN10A	0.278178	0.644974	0.150549	-0.17969	0.194178	-0.01255	-0.07188	0.024685

SCN11A	-0.05515	-0.41685	0.059121	0.042384	-0.28583	0.268031	-0.22173	-0.21072
SCN1A	0.208402	0.549124	-0.01647	-0.0351	-0.14895	0.09305	-0.20563	-0.04984
SCN1B	0.098663	0.02047	-0.08046	0.048286	0.070707	-0.29938	0.097011	0.261858
SCN2A	0.032814	0.258664	-0.08857	-0.14445	-0.15729	-0.18501	NA	NA
SCN2B	0.504832	-0.08327	0.106394	-0.06143	-0.14419	0.020873	0.015435	0.003385
SCN3A	0.000471	-0.08935	0.037615	-0.15447	-0.0606	0.0722	-0.4279	-0.32233
SCN3B	0.040081	-0.04257	-0.12245	0.376995	0.045273	-0.10525	0.030299	0.056217
SCN4A	0.667332	-0.08225	0.134056	0.034016	-0.26792	-0.03781	-0.41758	0.19101
SCN4B	-0.05626	-0.45738	-0.01735	-0.29168	-0.128	0.278259	-0.05633	-0.05701
SCN5A	0.121742	0.028886	0.067076	-0.19133	0.35116	0.330776	0.429644	0.363089
SCN7A	-0.07593	0.522799	-0.22374	-0.17231	-0.02554	0.131678	0.11815	-0.0441
SCN8A	0.129884	0.012443	-0.30309	-0.1202	0.082248	-0.09896	0.191471	-0.02583
SCN9A	-0.33704	-0.21983	-0.18645	-0.34597	-0.09162	-0.06923	-0.18262	-0.23995
SCNM1	-0.00955	0.015129	-0.16467	0.156076	-0.19685	-0.46473	0.228631	0.049766
SCNN1A	-0.13327	0.120632	-0.015	0.045955	0.003581	0.02934	0.247034	-0.1694
SCNN1B	-0.00902	0.142843	0.012585	-0.02247	-0.11849	-0.44952	0.021999	-0.39159
SCNN1D	0.009434	0.021892	0.202747	-0.33114	0.09815	0.105742	-0.3781	0.319244
SCNN1G	0.006218	-0.02473	-0.16975	0.066699	0.015993	-0.27717	-0.18201	-0.25177
SCO1	-0.0091	-0.25936	-0.26765	-0.12856	-0.4296	-0.21311	0.174033	0.0819
SCO2	-0.09108	0.019041	0.175671	-0.02388	-0.15024	-0.14583	0.163111	0.345005
SCOC	-0.1251	-0.70005	-0.19455	-0.06778	-0.24448	-0.47294	-0.05559	-0.35717
SCP2	-0.3181	0.337322	0.023014	-0.0538	0.191338	-0.34202	0.129672	0.177538
SCPEP1	-0.16872	-0.40996	0.348984	0.066834	-0.23234	0.27599	-0.15705	0.350018
SCRG1	0.001432	-0.25231	0.009425	-0.05066	-0.07717	-0.17383	-0.24035	-0.26622
SCRIB	-0.04031	0.162359	0.126726	0.102925	0.086603	0.117982	0.076247	-0.3531
SCRN1	-0.01008	-0.2135	-0.11477	0.154255	0.079076	0.23419	0.107856	-0.18392
SCRN2	-0.02206	0.608339	0.465139	-0.19669	0.161488	-0.2598	0.223736	0.019729
SCRN3	0.039509	-0.60668	-0.18328	-0.39552	-0.11667	-0.30863	0.215641	0.24665
SCRT1	0.154101	0.52986	0.186331	-0.31034	-0.00607	-0.19169	0.226339	0.007426
SCRT2	0.110757	0.687343	0.022854	-0.22023	-0.19084	0.053973	-0.07181	-0.1942
SCT	0.183091	-0.0046	0.115211	-0.31712	-0.00892	-0.33805	-0.0219	-0.07502
SCTR	-0.00731	0.48306	-0.04815	-0.103	0.041201	0.041032	0.121481	0.202379
SCUBE1	-0.13143	-0.00081	0.018047	0.053187	-0.18102	-0.48997	0.404987	0.031881
SCUBE2	-0.08634	0.133065	-0.00838	-0.07057	-0.16822	-0.01888	-0.21292	-0.15745
SCUBE3	0.112747	0.081688	0.04128	0.094006	0.02308	-0.04296	0.021871	0.302048
SCYE1	-0.03051	-0.44383	-0.40246	0.131818	-0.00769	-0.19705	-0.30306	-0.27184
SCYL1	-0.11813	0.074802	0.261618	0.250587	0.123827	0.029965	-0.473	-0.43307
SCYL1BP1	-0.23514	-0.42209	-0.28305	-0.19901	-0.38417	-0.47253	0.134563	0.003288
SCYL2	-0.21208	-0.02617	-0.33896	-0.25853	-0.02216	-0.15396	-0.30112	-0.14164
SCYL3	-0.05186	-0.05125	-0.28789	-0.19438	-0.38252	-0.60015	-0.14876	-0.07571
SDAD1	0.294729	0.459271	-0.07473	0.12145	-0.20002	-0.04004	0.248775	0.261513
SDC1	-0.43148	0.197117	0.140482	0.296177	0.552786	-0.15174	0.097328	-0.06956
SDC2	-0.24302	-0.03375	-0.10379	-0.04287	-0.36821	0.111304	-0.53753	-0.49462
SDC3	0.197943	-0.24691	0.320972	0.094271	0.165249	0.055421	-0.18244	-0.08252
SDC4	0.246893	0.431134	0.191007	0.006266	0.353588	-0.33499	0.374936	-0.22541
SDCBP	-0.35563	-0.63293	0.150164	-0.07367	-0.21182	0.222986	0.170939	0.052984
SDCBP2	-0.1423	0.018604	-0.0195	-0.08944	-0.06544	0.214059	0.10062	0.127559
SDCCAG1	-0.30968	-0.46747	-0.26212	-0.13766	-0.11502	0.240347	0.264389	0.134523
SDCCAG10	0.55765	-0.13499	-0.17083	0.108805	-0.18447	-0.33562	-0.0468	-0.29895
SDCCAG3	0.187433	-0.24853	-0.08424	0.317693	0.192408	0.191028	0.396775	0.484272
SDCCAG8	-0.376	-0.44678	0.041282	-0.04499	-0.23395	-0.12317	0.21657	0.202631
SDF2	0.181968	-0.2255	0.039719	-0.17978	-0.29508	-0.4645	0.022027	-0.34893

SDF2L1	-0.37581	0.475504	0.162418	0.359117	-0.0695	-0.10702	0.292806	0.083799
SDF4	-0.42855	-0.02492	0.315613	0.367289	0.128934	0.033001	0.265159	0.257658
SDHA	-0.3313	-0.58201	0.178177	0.219226	0.157949	0.221669	0.504194	0.401497
SDHAP3	NA	NA	NA	NA	NA	NA	0.422096	0.133567
SDHB	-0.083	0.008345	0.090878	0.063428	-0.11241	0.086919	0.261632	0.043831
SDHC	0.017144	-0.39585	0.093053	0.176138	-0.18208	-0.07323	-0.08708	-0.15281
SDHD	0.01551	-0.24484	-0.12224	-0.39123	-0.4254	-0.46705	-0.49734	-0.53029
SDK1	0.322957	0.136777	0.045998	0.158738	-0.00195	0.200796	-0.33818	0.118246
SDK2	-0.04816	0.336463	0.02681	0.072511	-0.06291	-0.00681	-0.43557	-0.44186
SDPR	0.0067	-0.1465	-0.41194	0.050418	-0.36365	-0.38785	-0.30978	-0.48867
SDR16C5	NA	NA	0.278043	0.07348	-0.05948	0.049807	NA	NA
SDR39U1	NA	NA	-0.11214	0.053857	-0.081	-0.14654	NA	NA
SDR42E1	NA	NA	-0.28921	-0.15776	-0.08153	-0.21765	NA	NA
SDR9C7	NA	NA	0.251452	-0.02458	0.274824	0.185331	NA	NA
SDS	-0.23912	0.442445	0.326622	-0.22876	0.103237	0.170047	-0.39527	0.025582
SDSL	-0.18159	-0.31691	0.222951	0.031908	-0.09518	-0.24707	-0.33209	-0.21606
SEC11A	-0.01185	-0.26447	-0.25854	0.031057	-0.10169	-0.02218	NA	NA
SEC11C	-0.14436	-0.28893	-0.04035	0.251651	-0.02685	-0.29931	NA	NA
SEC13	-0.0663	-0.09203	-0.08949	0.313353	0.167318	-0.17084	NA	NA
SEC14L1	0.222673	0.407455	0.198013	0.124404	-0.08433	0.134754	-0.30071	0.201055
SEC14L2	-0.08004	0.001615	0.369102	0.109085	0.178098	-0.21378	0.261759	0.489929
SEC14L3	0.023287	-0.06144	0.12188	-0.17703	-0.13489	0.067154	-0.43782	-0.31775
SEC14L4	0.283602	0.03685	-0.18274	-0.32434	0.0895	0.260903	0.204962	0.292289
SEC14L5	NA	NA	0.101304	0.164348	-0.09092	-0.2135	NA	NA
SEC16A	NA	NA	0.006114	0.326144	0.254842	0.285608	NA	NA
SEC16B	NA	NA	0.052524	0.032159	-0.21987	-0.22488	NA	NA
SEC22A	-0.01651	0.412189	-0.13833	-0.1867	-0.07478	-0.08495	NA	NA
SEC22B	-0.01592	0.502599	0.02149	-0.00621	-0.17753	-0.33177	NA	NA
SEC22C	-0.13868	-0.2597	0.050572	0.320313	-0.03288	-0.16404	0.183685	0.334313
SEC23A	-0.0581	-0.39133	-0.3818	-0.33033	-0.20496	-0.37998	-0.76431	-0.49664
SEC23B	0.073872	-0.36662	-0.09838	0.220797	0.108523	-0.15963	0.501447	0.136148
SEC23IP	-0.18906	-0.73522	-0.37611	0.054196	0.166566	0.005414	0.540156	0.447109
SEC24A	NA	NA	-0.06043	-0.24123	-0.01734	-0.29527	-0.3458	-0.0709
SEC24B	-0.26327	-0.54999	-0.19244	-0.2191	-0.17749	-0.36586	-0.21418	-0.29897
SEC24C	-0.44595	0.694104	0.245464	0.263585	0.358821	0.131637	0.453738	0.547079
SEC24D	-0.36632	-0.58251	0.107237	0.004935	-0.27681	-0.34481	0.015371	0.123241
SEC31A	-0.32478	-0.20493	-0.20132	0.077534	-0.27896	-0.13559	NA	NA
SEC31B	-0.03934	-0.09536	-0.2528	0.270522	0.02342	0.288795	NA	NA
SEC61A1	-0.47216	-0.08609	0.352539	0.092268	0.250638	-0.0338	-0.16677	0.059273
SEC61A2	-0.12199	-0.50717	-0.1574	-0.00995	0.04262	-0.19424	0.258169	0.411932
SEC61B	-0.04116	0.075654	-0.1579	0.179667	-0.1873	-0.1266	-0.49212	-0.20823
SEC61G	-0.17514	-0.70562	-0.08567	-0.15847	-0.21637	-0.40828	-0.07715	-0.14005
SEC62	NA	NA	-0.16107	-0.01533	-0.08667	-0.31032	NA	NA
SEC63	0.11965	0.400144	-0.01016	-0.03907	0.215628	0.270377	-0.24078	-0.18471
SECISBP2	-0.03413	-0.41202	0.181198	0.049037	0.138231	0.027808	0.315604	0.420583
SECTM1	-0.28645	0.174157	0.085359	-0.46656	-0.15177	-0.24064	-0.26448	0.116966
SEH1L	0.240364	-0.61057	-0.42284	-0.0603	-0.02269	0.024554	0.472761	0.443278
SEL1L	-0.19814	0.370039	-0.15935	-0.13435	-0.23311	-0.39311	-0.29537	0.116207
SELE	-0.22175	-0.05489	-0.13153	0.260577	0.040693	0.156037	-0.11521	0.0773
SELENBP1	0.388529	0.084996	0.246716	0.180234	0.174815	0.23428	0.123574	-0.12447
SELI	0.311446	0.110679	-0.10109	-0.12905	0.115321	-0.22097	0.246223	0.324994
SELK	-0.54878	-0.40772	0.040615	-0.08036	-0.12877	-0.2875	-0.18576	-0.35703

SELL	-0.38451	-0.13662	0.057243	0.623539	-0.13416	0.676774	-0.41533	0.094182
SELM	-0.22762	0.144124	0.22043	0.090419	0.070525	-0.03771	-0.24701	-0.19508
SELO	0.028104	0.649677	NA	NA	NA	NA	0.452281	0.499497
SELP	-0.47125	-0.37943	0.154646	0.298736	-0.07158	0.008669	-0.44933	-0.28213
SELPLG	0.246737	0.521758	0.479321	0.527528	0.001407	0.591021	-0.41421	0.226967
SELS	-0.52714	-0.41364	0.080045	0.044241	-0.02002	-0.15702	-0.07215	0.081821
SELT	-0.29134	-0.26789	-0.02455	-0.01801	-0.30746	-0.27768	-0.43948	-0.3094
SELV	0.13604	0.024794	-0.21579	0.123143	0.075444	0.064804	-0.13316	-0.38086
SEMA3A	0.243157	-0.35653	0.086935	-0.18652	-0.1476	0.382794	0.125844	-0.37493
SEMA3B	-0.05487	0.207547	0.236688	0.005766	0.224743	-0.04955	-0.19982	0.222249
SEMA3C	0.110298	-0.63968	0.251756	-0.29799	0.097877	0.03714	-0.2583	-0.25131
SEMA3D	0.34798	0.587248	0.068173	-0.04159	-0.24181	0.087334	-0.31533	-0.3437
SEMA3E	0.148323	-0.35046	0.124509	-0.37752	0.021636	0.261774	-0.50233	-0.34797
SEMA3F	-0.33587	0.022772	0.13396	0.24981	0.097824	0.150412	0.005234	0.123763
SEMA3G	-0.25275	-0.39309	-0.02602	0.266725	0.046481	0.133507	-0.36875	0.272073
SEMA4A	-0.25703	0.429862	0.32123	0.391092	-0.00478	-0.09291	-0.11375	0.080354
SEMA4B	-0.21587	0.428446	0.244563	-0.09987	0.244792	0.131356	0.133355	0.387121
SEMA4C	0.371144	0.601501	0.237595	0.132244	0.208882	0.514836	-0.45631	-0.43852
SEMA4D	-0.21289	0.050617	0.241462	0.604297	0.431457	0.431668	0.134533	0.371463
SEMA4F	0.137485	-0.34689	0.160806	0.002162	0.297476	-0.10754	0.350725	-0.05612
SEMA4G	0.088069	0.313722	-0.10802	0.040031	-0.05874	-0.09153	0.280407	0.265885
SEMA5A	0.071658	-0.05326	0.067031	-0.00149	0.057403	0.239315	-0.01492	-0.02829
SEMA5B	-0.01798	0.20561	0.014772	0.083565	0.021222	0.011243	0.143242	0.545863
SEMA6A	0.164309	0.19234	-0.07148	-0.19901	0.130763	-0.11205	NA	NA
SEMA6B	0.391263	0.590849	0.28608	-0.31793	-0.00134	-0.1697	-0.34652	-0.16324
SEMA6C	-0.17763	-0.40262	0.249541	-0.19406	0.127631	-0.00372	-0.07045	0.235798
SEMA6D	0.295825	0.075516	0.018935	-0.39592	-0.10741	0.131267	0.106573	0.279667
SEMA7A	-0.16458	-0.30915	-0.13833	0.671413	-0.12058	0.409305	0.120963	0.17805
SEMG1	-0.18645	0.315982	-0.07739	0.045082	-0.21273	-0.33034	-0.03829	-0.32342
SEMG2	-0.07297	0.138859	0.262997	-0.1932	0.114747	-0.16743	0.109161	-0.00078
SENP1	0.221879	-0.52522	-0.49796	-0.08197	0.108282	-0.51388	NA	NA
SENP2	0.163398	-0.48653	-0.40208	-0.01136	0.047736	-0.26082	-0.25667	-0.20247
SENP3	0.170517	0.088578	0.054411	0.181816	0.08084	0.452187	-0.49999	-0.10051
SENP5	-0.05009	-0.31858	-0.42567	-0.42832	0.061237	0.004974	-0.20928	-0.16292
SENP6	0.169474	-0.62718	-0.40525	-0.14361	0.182442	0.333402	0.006213	0.055394
SENP7	-0.01573	0.442154	-0.23255	0.015766	-0.2346	0.166204	-0.37509	0.007234
SENP8	-0.03078	-0.28767	-0.18551	-0.3835	-0.20719	-0.69496	-0.43042	-0.07972
SEPHS1	0.06947	-0.37823	-0.11838	0.103062	0.082037	-0.27332	0.151949	-0.10476
SEPHS2	0.180577	0.315938	0.192286	-0.09556	0.287089	-0.32508	-0.36706	-0.42338
SEPN1	-0.22678	0.038238	0.318301	0.253058	0.142964	0.065909	-0.01329	-0.02
SEPP1	0.039496	-0.50115	0.026654	-0.1284	-0.08571	-0.35383	-0.59818	-0.4533
SEPSECS	NA	NA	-0.01692	-0.26246	-0.06485	-0.28204	NA	NA
SEPW1	0.318141	-0.28551	-0.10346	-0.21013	0.088219	-0.32598	0.227275	-0.09275
SEPX1	0.093702	0.061474	0.200084	-0.49657	-0.04378	-0.45992	0.252223	-0.18834
SERAC1	0.023155	0.245005	-0.23024	-0.05132	0.025494	-0.35508	-0.09091	0.000262
SERBP1	0.000342	0.242699	-0.00854	0.24082	0.265868	0.126905	NA	NA
SERF1A	0.331158	0.212067	-0.05298	-0.23595	-0.16553	-0.22366	0.0804	-0.28041
SERF1B	0.332095	-0.36359	-0.04333	-0.23031	-0.14572	-0.12404	0.058914	0.414549
SERF2	-0.09543	-0.49088	-0.12229	0.061263	-0.2843	-0.46053	-0.11445	-0.1554
SERGEF	-0.03274	-0.01269	0.025718	0.028392	0.038542	-0.33607	0.042416	-0.18557
SERHL	-0.09771	0.037531	-0.16589	-0.00377	0.393538	-0.15321	-0.40218	0.032245
SERHL2	-0.06475	-0.23602	-0.15691	-0.02712	-0.01741	-0.21314	-0.32372	-0.47817

SERINC1	0.110058	-0.5949	-0.29314	-0.29216	-0.21771	-0.40418	-0.47207	-0.47659
SERINC2	-0.0392	0.181707	0.209005	0.107048	0.194287	-0.04642	0.589669	0.213242
SERINC3	0.444833	-0.14383	0.309274	-0.16086	0.315352	-0.45128	0.208482	-0.17302
SERINC4	-0.14638	0.526291	NA	NA	NA	NA	-0.1919	0.106571
SERINC5	0.264195	-0.31948	0.513656	0.081145	0.375197	0.150512	0.473566	0.14824
SERP1	-0.18241	-0.54277	-0.37036	0.148867	-0.13984	-0.19935	-0.30635	-0.38288
SERP2	NA	NA	-0.00595	-0.10016	0.038567	0.092903	NA	NA
SERPINA1	-0.31688	0.034776	0.195693	0.219761	0.18698	-0.15577	0.043881	0.444174
SERPINA10	0.482779	0.504473	-0.32386	-0.0511	0.319993	-0.01042	0.088881	-0.34816
SERPINA11	NA	NA	-0.54504	0.15799	0.215402	0.009798	-0.02558	0.13862
SERPINA12	0.199137	0.765216	-0.02604	-0.06587	0.060019	-0.33063	0.403179	-0.02408
SERPINA13	-0.03679	0.190466	-0.11117	0.040059	0.112108	-0.01431	-0.28296	0.05936
SERPINA3	-0.45029	-0.01703	0.037746	0.499303	-0.00959	0.349666	-0.1176	-0.16948
SERPINA4	0.274468	0.245507	0.087891	0.089753	0.051446	-0.32677	-0.28603	-0.46922
SERPINA5	-0.06554	-0.3287	-0.0464	0.687988	-0.06824	0.223804	-0.28854	-0.40916
SERPINA6	0.274011	0.253944	-0.22071	0.047241	0.032433	-0.27851	0.195593	-0.42315
SERPINA7	0.229541	0.117809	0.156363	0.226157	-0.09534	0.001716	0.267819	0.30571
SERPINA9	-0.14148	-0.0448	0.144306	0.697247	-0.18913	0.295182	0.30902	0.076071
SERPINB1	-0.16869	0.001225	-0.09447	-0.20207	-0.22927	-0.01018	0.1757	0.245027
SERPINB10	NA	NA	-0.04826	0.487602	0.183065	0.175301	0.457254	-0.34887
SERPINB11	0.171588	0.040009	-0.08724	-0.16392	-0.03986	0.099802	-0.04219	0.001203
SERPINB12	-0.2386	0.139573	0.164953	-0.11163	0.238125	-0.03176	0.240828	0.114275
SERPINB13	-0.00996	0.499605	0.076073	0.072119	0.122132	-0.09549	-0.3159	-0.21683
SERPINB2	0.032078	0.133831	-0.00142	-0.07959	-0.04948	-0.19188	0.052741	-0.14297
SERPINB3	-0.01622	-0.05571	0.150424	0.030825	0.013009	0.002868	0.006971	-0.05874
SERPINB4	-0.02573	0.027536	0.076746	-0.0928	0.00778	0.06232	0.237052	0.293508
SERPINB5	-0.29717	-0.01175	-0.04189	0.048999	-0.34635	0.112401	0.250892	0.240061
SERPINB6	-0.3472	0.666637	0.071073	0.110832	-0.22621	-0.40157	-0.17775	0.012664
SERPINB7	-0.42622	-0.03162	0.094997	0.321161	-0.03063	0.111166	-0.04894	-0.21627
SERPINB8	-0.25198	-0.0442	0.065122	-0.09988	-0.33593	0.127291	-0.00444	0.490416
SERPINB9	0.170438	-0.07986	0.005866	0.482468	-0.09911	0.613185	-0.17	0.147012
SERPINC1	-0.25059	0.191146	-0.17953	0.101236	-0.21783	-0.39281	0.567753	0.507084
SERPIND1	0.312092	0.38761	0.204879	-0.23628	-0.03413	0.02783	0.729549	-0.44933
SERPINE1	-0.37997	-0.25397	0.066969	-0.03596	0.093283	0.139652	-0.12254	0.068661
SERPINE2	0.462678	-0.2566	0.272337	0.075454	0.271845	0.15319	-0.1074	0.095566
SERPINE3	NA	NA	-0.17155	0.002019	0.260653	0.005309	NA	NA
SERPINF1	-0.15601	0.103947	0.264248	0.042609	-0.07409	0.53838	-0.40447	-0.06435
SERPINF2	0.357407	0.308731	0.263445	0.411857	0.161022	0.025909	0.223241	0.222177
SERPING1	-0.11005	-0.15448	0.230766	0.110242	-0.1956	0.491715	-0.29133	-0.01868
SERPINH1	-0.21277	-0.58815	0.108001	0.203053	0.093393	0.100593	-0.01707	0.046278
SERPINI1	-0.07496	-0.35809	0.213642	-0.02349	-0.39383	-0.03719	-0.32413	-0.33544
SERPINI2	0.103395	0.330062	0.147382	-0.28768	0.03127	0.05386	-0.16898	-0.10415
SERTAD1	-0.17486	0.386239	-0.23278	0.01468	-0.25667	-0.07723	0.096889	0.08799
SERTAD2	-0.67097	-0.62947	-0.17939	-0.12527	-0.24243	0.049927	-0.1756	0.001881
SERTAD3	-0.10914	-0.08337	-0.1337	-0.01351	-0.2337	-0.25202	0.204332	-0.2173
SERTAD4	-0.24174	-0.08566	-0.03264	0.016193	-0.24562	-0.05731	-0.5175	-0.50253
SESN1	0.204276	-0.34363	0.340278	-0.12533	0.212626	-0.10836	0.34712	0.072913
SESN2	-0.56429	-0.44235	0.059422	0.035405	0.16592	0.038452	-0.34829	0.167452
SESN3	0.213982	-0.01451	0.176739	0.200983	-0.41638	-0.3896	0.06969	0.384578
SESTD1	-0.06683	-0.20734	-0.02908	-0.27829	0.140201	-0.21087	0.362128	0.255345
SET	0.14248	-0.51718	-0.40197	0.358584	-0.0974	-0.04668	-0.13879	-0.46732
SETBP1	-0.27094	0.225389	-0.11299	0.087691	-0.02976	0.121323	-0.21753	-0.35939

SETD1A	0.237943	0.40876	0.154847	0.086848	0.328622	0.088595	0.495782	0.422955
SETD1B	NA	NA	0.001201	-0.08138	0.214812	0.060095	NA	NA
SETD2	0.088151	-0.57736	-0.19394	0.024791	0.103812	0.33875	NA	NA
SETD3	-0.34818	-0.00407	-0.32993	0.124566	-0.05746	-0.17957	0.150372	0.287245
SETD4	0.176297	0.107703	-0.25338	-0.25981	-0.03329	0.058114	0.285468	0.324268
SETD5	NA	NA	0.136064	0.075755	0.325327	0.576409	NA	NA
SETD6	0.485868	0.08579	-0.35135	0.116466	-0.04297	0.06297	-0.37874	-0.01002
SETD7	-0.09202	-0.10714	-0.20784	0.138195	-0.01385	-0.27576	NA	NA
SETD8	-0.38708	0.175347	-0.0664	0.077634	0.259509	-0.18596	0.032232	0.129058
SETDB1	0.317869	0.418756	0.02851	0.291922	0.090691	0.558559	0.631982	0.490233
SETDB2	0.298466	0.429674	0.296828	-0.07923	0.052944	-0.14044	-0.47128	-0.44282
SETMAR	0.156363	-0.26932	-0.35783	-0.22313	-0.15033	-0.26511	0.488712	0.366792
SETX	-0.01157	-0.00557	-0.06582	-0.15363	-0.04635	0.334681	-0.65544	-0.40107
SEZ6	0.07519	0.023767	0.277891	-0.09922	0.198757	-0.0528	-0.30672	-0.46192
SEZ6L	-0.22345	0.140343	0.195156	-0.14486	-0.06126	-0.33994	0.15015	0.421048
SEZ6L2	0.274746	0.437029	0.198959	0.437518	0.385205	0.10146	0.396182	-0.10186
SF1	0.093744	-0.42349	0.169072	0.106084	0.021287	0.557273	-0.35543	0.062868
SF3A1	0.194594	-0.47502	0.010633	0.528467	0.186824	0.256199	0.55942	0.094831
SF3A2	-0.26816	-0.25347	-0.12489	0.015823	-0.14502	0.207164	0.461564	-0.03838
SF3A3	0.026101	0.359466	0.217476	0.158969	0.220815	0.250927	-0.40824	-0.39422
SF3B1	0.03083	-0.39112	-0.05435	-0.23283	-0.05995	0.252531	0.529366	0.356883
SF3B14	0.310112	-0.71538	-0.09665	-0.19998	0.128294	-0.24606	-0.4597	-0.52367
SF3B2	-0.07587	0.574154	-0.05481	0.481969	0.306907	0.473141	-0.0432	-0.16972
SF3B3	0.462636	0.558223	-0.24677	0.369257	0.267065	0.299882	0.137468	-0.32915
SF3B4	0.356671	0.519998	-0.08465	0.169917	-0.06116	0.050476	0.12822	-0.09472
SF3B5	0.408508	0.136191	-0.33495	0.122202	-0.0021	-0.09837	-0.05571	-0.21275
SF4	0.420115	0.261487	0.235883	0.390285	0.387795	0.347953	0.368367	0.402413
SFI1	-0.04446	-0.08304	0.155237	0.051378	0.380896	0.178109	0.218481	0.219664
SFMBT1	-0.32085	-0.48673	-0.10268	0.138019	0.079664	0.073165	0.459511	0.309741
SFMBT2	0.022592	0.532653	0.136621	-0.12861	-0.16468	0.197715	-0.51944	0.060947
SFN	-0.02501	0.331413	0.320939	-0.04475	0.018202	0.116321	0.371302	0.042117
SFPQ	0.117517	-0.04001	-0.25121	0.357955	0.172011	0.1312	-0.37688	-0.3926
SFRP1	-0.00538	-0.30291	0.254402	0.101074	-0.16187	0.00551	-0.1247	0.309379
SFRP2	-0.09197	-0.35729	0.097511	-0.03182	-0.15338	-0.12928	-0.06503	-0.07112
SFRP4	0.020175	-0.17396	0.089751	-0.02502	-0.00516	-0.17065	-0.16011	0.312336
SFRP5	-0.02763	-0.27236	0.171792	-0.02067	0.328079	0.250208	0.51729	0.417084
SFRS1	0.039395	-0.69704	-0.1751	0.306844	0.243679	0.169893	-0.33952	-0.36814
SFRS10	-0.10232	0.345667	-0.21755	0.093682	-0.02358	0.268634	0.053293	0.086895
SFRS11	-0.21486	-0.48717	0.039852	0.148113	0.230079	0.443843	-0.52546	-0.16351
SFRS12	-0.03662	-0.47318	-0.05083	-0.10492	0.029413	0.052501	-0.00792	-0.04534
SFRS14	0.065988	-0.60637	0.010294	0.089177	0.334281	0.373648	-0.33397	0.18204
SFRS15	0.211522	-0.37233	-0.27363	0.469922	0.121586	0.23935	0.221422	-0.19008
SFRS16	0.040518	0.278723	0.081279	0.122142	0.31733	0.372849	-0.35353	0.152657
SFRS2	-0.27977	-0.69143	-0.18976	0.196079	-0.08825	0.249192	0.505131	0.466953
SFRS2B	-0.05429	-0.54625	-0.25149	-0.0234	0.043331	0.020992	NA	NA
SFRS2IP	0.257937	-0.15073	-0.14942	-0.13772	-0.01532	0.246181	0.170557	0.218141
SFRS3	0.102203	-0.508	-0.49842	0.055999	-0.04527	0.185283	-0.55542	-0.24624
SFRS4	NA	NA	-0.13332	0.027652	0.128473	0.280868	0.348895	0.389154
SFRS5	-0.28354	-0.39665	-0.30975	0.092581	-0.15304	0.253032	0.270357	0.406575
SFRS6	0.375948	0.36618	-0.40065	-0.03389	0.330946	-0.05187	0.468744	0.437596
SFRS7	0.395038	-0.38988	-0.32413	-0.00488	0.171883	0.262129	0.332235	0.222513
SFRS8	0.050279	0.35266	0.24124	0.108448	0.28066	0.367128	-0.49266	-0.08369

SFRS9	-0.12133	-0.6178	-0.16092	0.461395	0.218963	0.039141	0.45892	0.507662
SFT2D1	0.038767	-0.44195	-0.20794	-0.0908	-0.02873	-0.23447	0.525611	0.418086
SFT2D2	-0.27091	-0.18274	-0.00935	-0.1683	-0.2891	-0.14252	0.15105	0.078913
SFT2D3	0.369536	-0.41635	-0.00964	-0.01477	0.16062	-0.3323	0.667834	0.203974
SFTA2	NA	NA	0.157877	-0.21009	-0.05144	-0.06691	NA	NA
SFTA3	NA	NA	-0.05593	0.0406	-0.12359	0.090083	NA	NA
SFTPA1	NA	NA	-0.07006	-0.08472	0.405661	-0.14504	0.048845	0.041719
SFTPA2	NA	NA	NA	NA	NA	NA	0.185013	0.094663
SFTPB	0.54178	0.446936	-0.11505	-0.02206	0.081438	0.111692	0.197297	0.300991
SFTPC	-0.23303	-0.46589	0.202674	0.192803	-0.18669	-0.07976	0.332806	0.271586
SFTPD	0.363683	0.036214	-0.12255	-0.13031	-0.07855	-0.00133	-0.12311	-0.22627
SFXN1	0.088553	0.16699	-0.0704	-0.12273	-0.26467	-0.45391	0.344573	0.207713
SFXN2	0.223472	-0.05223	0.018852	0.413105	0.384262	-0.01914	0.264474	0.134809
SFXN3	-0.08352	-0.2539	0.376177	-0.00569	0.164434	0.094603	0.167573	0.276573
SFXN4	-0.18177	0.323161	0.144427	0.057222	0.26011	0.135391	0.040943	-0.08684
SFXN5	0.005043	0.377029	0.038145	-0.12444	0.445373	-0.35606	0.312547	0.446327
SGCA	0.316885	0.12218	-0.07928	-0.08456	-0.02341	0.132994	-0.154	0.000572
SGCB	-0.16728	0.405906	-0.02577	-0.1568	-0.21086	-0.37661	-0.58522	-0.42036
SGCD	-0.27859	-0.20458	0.081002	-0.03371	-0.22159	-0.09776	-0.31555	-0.22139
SGCE	0.015236	-0.44761	-0.01051	-0.08054	-0.19295	0.196801	0.00014	-0.08078
SGCG	-0.04566	-0.19014	-0.15321	-0.14025	0.129175	-0.15938	0.050944	0.282128
SGCZ	-0.17255	-0.20923	-0.15218	0.083428	-0.01504	0.017796	0.254628	0.203638
SGEF	-0.02095	0.086034	0.136151	-0.19014	-0.16087	-0.00013	-0.09325	-0.15179
SGIP1	-0.34391	-0.44443	-0.14021	0.219518	-0.10035	0.020053	-0.11108	0.155595
SGK1	NA	NA	0.060857	-0.25977	-0.11855	-0.30252	NA	NA
SGK196	NA	NA	0.010172	-0.22794	-0.10852	0.078482	NA	NA
SGK2	0.28979	0.177391	0.005703	-0.09831	0.07653	-0.12536	0.493393	0.210661
SGK3	-0.10902	0.40414	0.134267	-0.20346	0.10688	-0.02151	-0.26317	-0.19152
SGMS1	NA	NA	-0.10763	-0.21891	-0.24122	-0.24144	NA	NA
SGMS2	NA	NA	-0.2886	-0.24118	-0.42425	-0.07188	NA	NA
SGOL1	-0.13862	-0.0765	-0.48017	0.1635	-0.02301	-0.13679	0.31138	0.450016
SGOL2	0.020614	-0.1852	-0.34919	0.341626	-0.06187	-0.00604	-0.26565	-0.23169
SGPL1	0.090141	-0.09989	0.366785	0.093785	-0.16011	-0.10008	0.745168	0.479106
SGPP1	-0.00458	0.246399	-0.14023	0.096973	-0.2083	0.183844	0.149612	0.330246
SGPP2	-0.06687	0.185091	0.417298	-0.24413	-0.02322	-0.07099	0.194751	0.384623
SGSH	0.167441	0.653248	0.491675	-0.00719	0.325215	0.372978	0.415777	0.419347
SGSM1	NA	NA	-0.05967	0.174147	0.064156	-0.00288	NA	NA
SGSM2	NA	NA	0.263264	0.136042	0.085706	0.357162	NA	NA
SGSM3	NA	NA	0.132177	0.46266	0.057153	0.043893	NA	NA
SGTA	-0.08582	-0.04677	0.110429	0.053213	0.287006	-0.03254	-0.07666	-0.06707
SGTB	0.020758	0.367092	0.119401	0.281515	-0.25337	0.01469	-0.39869	-0.34661
SH2B1	-0.22119	0.357643	0.303728	0.147346	0.337141	0.125794	0.489557	0.417845
SH2B2	-0.09472	0.49763	0.340016	0.623308	0.206852	0.260405	NA	NA
SH2B3	0.135146	-0.25998	-0.04609	0.108042	-0.229	0.011389	NA	NA
SH2D1A	-0.186	0.325457	0.157837	0.674739	-0.33914	0.666323	-0.62945	-0.27512
SH2D1B	-0.29532	0.132228	-0.20698	-0.06276	-0.21698	-0.03046	-0.68483	0.03956
SH2D2A	0.276655	0.314773	0.083017	0.349587	-0.2367	0.407469	-0.51773	-0.25639
SH2D3A	0.214087	0.237658	0.035315	-0.07243	0.278679	-0.00069	0.481961	0.26343
SH2D3C	-0.33632	-0.05331	0.400661	0.489249	0.094985	0.004502	-0.1409	0.488596
SH2D4A	-0.14568	0.373927	0.10511	0.058666	-0.1968	-0.23589	0.200837	-0.01472
SH2D4B	0.284164	0.703064	0.036276	-0.14773	0.281146	-0.15411	-0.08945	-0.23871
SH2D5	NA	NA	0.043854	-0.40672	0.134118	-0.01713	NA	NA

SH2D6	0.344066	0.73795	-0.05884	0.004901	-0.17098	0.245155	NA	NA
SH3BGR	0.108058	0.016676	-0.16125	-0.23668	-0.0963	-0.38671	-0.19211	-0.23908
SH3BGRL	0.02228	-0.45574	-0.02378	-0.11722	-0.39389	-0.15718	-0.47146	-0.30934
SH3BGRL2	0.170121	0.197808	-0.41413	-0.35216	0.070449	-0.42759	0.253188	0.010942
SH3BGRL3	-0.29178	-0.00875	0.207507	0.046677	-0.006	0.077887	0.255767	-0.01175
SH3BP1	0.09234	0.284646	0.017469	0.023891	0.355502	-0.03512	0.366638	0.203189
SH3BP2	-0.66484	0.07704	0.485224	-0.05382	0.247217	0.102745	0.135226	0.317827
SH3BP4	0.15369	0.165783	0.119963	0.261561	0.345177	0.145361	0.398031	-0.07154
SH3BP5	-0.17975	0.216764	0.243056	0.372145	-0.07086	0.262723	0.531416	0.593334
SH3BP5L	-0.3235	-0.28495	0.16858	0.159727	-0.03279	0.247158	0.431735	0.263119
SH3D19	-0.08439	-0.6508	-0.37592	-0.22705	-0.14617	-0.24299	0.316743	0.232116
SH3GL1	-0.00599	0.046245	0.048146	0.102121	0.163665	0.126877	-0.60079	-0.37342
SH3GL2	-0.23798	-0.15212	0.018221	0.097003	-0.26435	-0.13941	-0.00348	0.336143
SH3GL3	0.158224	-0.31621	0.06486	-0.27591	0.154538	-0.18959	0.058266	-0.08854
SH3GLB1	-0.13269	-0.08356	0.174127	-0.17628	-0.06307	-0.50336	0.442587	0.492614
SH3GLB2	-0.00778	0.419643	0.451435	-0.05739	0.297986	-0.16197	0.284409	0.363354
SH3KBP1	-0.30341	-0.02329	0.160671	0.093735	-0.36194	-0.0594	0.161423	-0.02532
SH3PXD2A	-0.53469	-0.34949	0.07186	-0.10087	0.144953	0.003824	-0.26672	-0.04778
SH3PXD2B	-0.10967	-0.01908	0.209562	-0.12198	0.03483	0.006935	-0.34041	-0.12927
SH3RF1	-0.31726	-0.30917	-0.29312	-0.14793	0.179635	0.14835	NA	NA
SH3RF2	0.20755	0.226492	0.21979	0.094597	0.056564	-0.2846	-0.04021	0.065025
SH3RF3	NA	NA	0.178587	0.217549	0.167041	0.080422	NA	NA
SH3TC1	0.265999	0.757032	0.339225	0.446672	0.306232	0.437663	-0.32994	0.170549
SH3TC2	0.358727	0.45372	0.109362	0.39347	-0.04494	0.317624	0.232172	0.349964
SH3YL1	-0.03914	0.224382	-0.30129	-0.02959	-0.21932	-0.19156	0.037894	-0.40641
SHANK1	-0.06208	0.188777	-0.19378	-0.05567	-0.08004	-0.0872	-0.15248	-0.33116
SHANK2	0.060301	-0.1894	-0.17036	-0.25767	0.387561	0.214389	-0.06303	-0.51176
SHANK3	NA	NA	0.010406	0.085655	0.093875	0.360178	0.036669	0.334488
SHARPIN	0.211209	0.617785	0.421889	0.122959	0.137312	0.009956	0.253015	-0.21997
SHB	-0.23124	0.348774	0.276975	-0.08875	0.150809	-0.01624	0.416307	0.399909
SHBG	-0.23099	-0.11781	0.041963	-0.15398	0.29158	-0.19767	0.211491	0.417887
SHC1	-0.31166	-0.21336	0.381985	-0.04665	0.037004	0.143834	0.158704	0.535277
SHC3	0.206381	-0.54101	-0.00731	-0.05555	0.120366	0.070874	-0.38461	-0.26212
SHC4	-0.08754	-0.36629	-0.09828	-0.29097	-0.15968	0.33416	-0.09029	-0.05178
SHCBP1	-0.04399	0.082667	-0.46492	0.374486	0.187696	0.01326	-0.51162	-0.59818
SHD	-0.17098	0.380742	-0.28291	-0.02639	0.129912	-0.34374	-0.02654	-0.17603
SHE	-0.55947	-0.2455	-0.0216	-0.01842	-0.14008	-0.06496	-0.55434	-0.15991
SHF	-0.14657	0.309556	-0.0113	0.169925	-0.05248	0.452732	-0.21739	0.144174
SHFM1	-0.14196	-0.62479	0.179512	-0.27528	0.164934	-0.04727	0.485996	0.405034
SHH	0.391411	0.578295	0.234007	0.148189	0.132303	-0.10729	0.113529	0.002674
SHISA2	NA	NA	0.081337	0.184908	0.00148	0.255945	NA	NA
SHISA3	NA	NA	-0.18754	-0.11768	-0.08127	0.051171	NA	NA
SHISA4	NA	NA	0.225652	-0.09371	0.058568	-0.15042	NA	NA
SHISA5	NA	NA	0.222158	0.260449	0.27348	0.099856	NA	NA
SHKBP1	0.509346	0.673324	0.184037	0.186271	0.056898	-0.03336	0.694952	0.257944
SHMT1	0.13615	0.238823	0.125499	0.112161	0.197827	0.048027	0.227671	0.063607
SHMT2	0.035319	0.412092	-0.08149	0.43363	0.354679	-0.03362	0.397333	0.2913
SHOC2	NA	NA	-0.17812	-0.22169	-0.1539	-0.39337	-0.30751	-0.48453
SHOX	0.233547	0.497748	0.179281	-0.25655	0.01756	0.019595	0.229806	-0.15897
SHOX2	-0.60427	-0.26612	-0.02634	0.068645	-0.08036	-0.51545	0.20958	0.202737
SHPK	NA	NA	0.238754	0.026294	-0.44336	0.123207	NA	NA
SHPRH	0.528411	0.110601	-0.20082	-0.15316	0.27573	0.3487	0.251884	0.1263

SHQ1	0.347148	0.266708	-0.29656	0.129439	1.95E-05	-0.07963	0.422082	0.264317
SHROOM1	-0.03111	-0.06703	0.452334	-0.05447	0.463666	0.21583	NA	NA
SHROOM2	0.30976	-0.10617	0.285694	-0.37533	-0.0547	-0.19416	NA	NA
SHROOM3	-0.23966	0.085317	-0.27833	0.05112	-0.04009	-0.19291	NA	NA
SHROOM4	NA	NA	0.089595	-0.0723	0.066774	0.033333	-0.42302	-0.3344
SI	-0.04685	-0.22983	0.006194	0.36327	-0.13776	0.171453	-0.0484	0.117519
SIAE	-0.21883	-0.13411	0.081871	-0.24096	-0.03694	0.017678	-0.21804	-0.21901
SIAH1	-0.01668	-0.51665	-0.18286	-0.19031	0.178858	-0.11743	0.495751	0.093622
SIAH2	-0.2112	-0.67283	0.094901	0.192447	0.163946	-0.07295	0.566976	0.24615
SIAH3	NA	NA	-0.1787	-0.22486	0.132779	-0.0607	NA	NA
SIDT1	-0.29985	-0.15176	-0.2294	-0.05336	-0.32916	-0.13789	0.154161	0.165331
SIDT2	-0.47118	0.311839	0.087505	-0.01298	-0.0284	0.125959	-0.29449	0.136094
SIGIRR	-0.1007	0.485894	0.110611	0.238798	-0.11886	0.183271	0.465871	0.158189
SIGLEC1	0.066755	-0.09557	0.115915	-0.29699	-0.37562	0.153986	NA	NA
SIGLEC10	-0.26546	0.397634	0.341587	0.639725	0.141365	0.644513	-0.34877	0.069884
SIGLEC11	-0.21622	-0.26768	0.221616	-0.44653	0.062617	0.008526	-0.29111	-0.15348
SIGLEC12	0.275665	0.571979	0.073789	-0.29798	-0.04442	0.066493	-0.02776	0.186502
SIGLEC15	NA	NA	0.360191	0.137269	-0.07794	0.128917	NA	NA
SIGLEC5	-0.50835	0.010073	0.081321	0.247706	-0.00431	0.230303	-0.35392	0.237045
SIGLEC6	0.122926	0.525243	0.274948	0.440543	0.175034	0.257999	-0.12121	0.040054
SIGLEC7	-0.33113	0.259525	0.159529	0.040219	-0.28014	0.085198	-0.22254	0.137861
SIGLEC8	-0.04899	0.397644	0.339552	0.077163	-0.09712	0.236785	0.328835	0.306531
SIGLEC9	-0.39548	-0.5197	0.406891	-0.00041	-0.04722	0.190725	-0.19514	0.226649
SIGLECP3	NA	NA	-0.00124	0.166834	-0.19057	-0.18293	-0.34716	0.119357
SIGMAR1	NA	NA	-0.04052	0.240566	0.110834	-0.15763	NA	NA
SIK1	NA	NA	0.094606	0.294947	0.004805	0.141135	NA	NA
SIK2	NA	NA	0.291832	-0.19428	0.051876	-0.0012	NA	NA
SIL1	-0.25563	0.582283	0.674958	0.275747	0.130931	-0.32757	-0.16765	-0.14958
SILV	0.051715	0.427593	0.261479	-0.12531	0.196144	-0.2821	0.148052	0.572198
SIM1	0.02227	0.586785	0.161107	-0.12551	0.263789	0.039367	-0.0304	-0.01813
SIM2	0.282393	0.236262	-0.00342	-0.27861	0.026542	0.181853	0.27476	-0.32014
SIN3A	-0.03282	0.175018	-0.1164	-0.02022	0.0018	0.201463	0.294207	0.297977
SIN3B	0.184	0.407317	-0.15328	0.286667	-0.05433	-0.07449	-0.0776	0.2402
SIP1	-0.03274	-0.54636	-0.16355	-0.29683	-0.24566	-0.08943	0.345645	0.357048
SIPA1	-0.41315	0.6005	0.293057	0.498128	0.060766	0.294983	-0.64037	-0.06065
SIPA1L1	-0.24763	0.531204	-0.0287	-0.17342	0.036296	0.15694	-0.40952	-0.3289
SIPA1L2	-0.67226	-0.23641	-0.21729	0.195093	-0.30826	-0.08569	-0.44751	-0.37061
SIPA1L3	0.501954	0.725893	-0.25865	-0.10426	0.079099	-0.3416	-0.19158	-0.40281
SIRPA	-0.3639	0.120106	0.197057	0.00747	-0.00158	0.045925	NA	NA
SIRPB1	-0.38177	0.381986	0.43842	0.404196	-0.02193	0.135961	-0.38762	-0.17901
SIRPB2	NA	NA	0.241453	0.267726	0.028581	0.227885	-0.04124	0.330021
SIRPD	0.318542	0.735976	0.106265	-0.35214	-0.26186	-0.47729	0.426307	0.29263
SIRPG	0.21351	0.26114	0.378811	0.714189	-0.24981	0.507556	-0.32909	-0.36461
SIRT1	0.025829	-0.48671	-0.23179	-0.07454	0.192472	0.04307	0.122377	0.07172
SIRT2	0.236788	0.344788	0.201216	-0.04221	-0.03735	-0.19376	-0.23512	-0.30172
SIRT3	0.03328	0.174767	-0.35171	-0.02381	-0.04894	-0.28999	-0.2779	-0.37828
SIRT4	-0.34607	-0.2815	-0.11578	-0.13578	-0.21158	-0.53657	-0.39413	-0.28355
SIRT5	0.349924	-0.28714	-0.29438	0.046963	-0.08513	-0.30719	0.342729	0.132691
SIRT6	0.577884	0.278204	0.042859	-0.19341	0.046372	0.058057	-0.06874	-0.07385
SIRT7	-0.20301	0.307919	0.346244	0.044582	0.251424	0.070023	NA	NA
SIT1	0.275165	0.441976	0.021178	0.780814	-0.16146	0.60542	-0.47644	-0.06008
SIVA1	-0.13104	-0.33454	-0.19091	-0.00738	-0.17244	-0.21838	NA	NA

SIX1	0.260095	0.285539	0.044309	-0.09436	-0.16395	0.243339	0.187376	-0.37157
SIX2	-0.27649	-0.10676	-0.27088	-0.23859	-0.00886	-0.20513	-0.23087	0.360572
SIX3	-0.11456	-0.16218	-0.0045	-0.18285	0.022681	0.048705	0.148048	-0.2232
SIX4	0.137851	-0.32167	-0.13636	-0.13003	-0.33857	0.384427	-0.03781	0.145639
SIX5	-0.39006	0.176698	0.095947	-0.04346	0.450568	0.072498	-0.37062	-0.41884
SIX6	0.094672	0.154669	0.272356	0.238171	-0.08268	0.071382	0.032043	0.06578
SKAP1	0.233403	0.666284	0.309149	0.598375	-0.00404	0.379038	0.082831	0.287811
SKAP2	0.092578	-0.5245	-0.15966	0.070395	-0.13568	-0.25954	-0.21357	-0.34983
SKI	-0.24196	0.329029	0.253865	0.008085	-0.05158	0.255822	0.034354	-0.09831
SKIL	0.006714	-0.06165	-0.4626	0.079059	-0.12831	-0.02193	0.417364	0.376392
SKIV2L	0.38706	0.42692	0.305261	0.273857	0.328138	0.148991	0.298213	0.353407
SKIV2L2	0.171366	-0.63829	-0.05397	-0.08463	-0.00298	0.140068	0.170675	-0.09817
SKP1	NA	NA	0.338897	-0.36658	0.03041	-0.30442	NA	NA
SKP2	-0.07971	-0.08678	-0.30486	0.131582	-0.09737	-0.09948	0.486658	0.276584
SLA	-0.42379	0.02215	0.328875	0.650319	-0.01469	0.722293	-0.61469	-0.23247
SLA2	0.084635	0.256001	0.423831	0.344384	0.231842	0.259777	-0.35162	0.633429
SLAIN1	0.172464	0.179649	-0.35995	0.169228	-0.35441	0.168093	NA	NA
SLAIN2	NA	NA	-0.32595	-0.30017	-0.23819	-0.50224	NA	NA
SLAMF1	-0.282	-0.02526	0.236133	0.348629	-0.1305	0.461124	-0.27828	0.450975
SLAMF6	0.229376	0.185759	0.084203	0.595042	-0.13329	0.386554	-0.56495	-0.00173
SLAMF7	-0.36824	-0.07866	0.161404	0.133894	-0.33417	0.179094	-0.60988	-0.06323
SLAMF8	-0.2986	-0.06178	0.144225	0.154956	-0.35565	0.379046	-0.53646	-0.1537
SLAMF9	-0.42312	0.353719	0.126888	-0.24521	-0.1564	-0.16356	-0.24552	-0.52319
SLBP	0.129116	-0.23654	-0.00487	0.12253	0.172554	-0.02715	-0.421	-0.56609
SLC10A1	-0.23608	0.039547	-0.18112	-0.02533	0.068978	0.268159	0.131464	0.138104
SLC10A2	0.200992	0.593751	-0.08171	-0.17911	0.041818	0.136245	0.264185	0.501571
SLC10A3	0.317093	0.572831	-0.11594	0.066235	0.270302	-0.1598	0.37327	-0.04381
SLC10A4	0.095548	-0.3574	-0.41035	-0.08584	-0.10839	-0.1406	-0.4333	-0.42616
SLC10A5	-0.25495	0.273836	NA	NA	NA	NA	0.377061	0.336848
SLC10A6	0.604852	0.384835	NA	NA	NA	NA	0.066249	0.113833
SLC10A7	-0.35186	-0.38262	-0.25413	0.051785	-0.20064	-0.26621	0.076919	-0.26111
SLC11A1	-0.52229	0.068667	0.242112	-0.10004	-0.03375	-0.12782	-0.20427	0.163124
SLC11A2	0.078016	0.273614	0.083065	0.036792	0.014777	-0.23706	0.16831	0.222288
SLC12A1	0.084043	0.325251	0.088026	-0.33251	-0.04208	0.010138	0.19196	0.079457
SLC12A2	-0.10177	-0.45308	-0.04363	0.068123	0.121436	0.309258	-0.03225	-0.16256
SLC12A3	-0.01917	0.133924	0.06345	0.339476	0.000263	0.117685	0.121398	0.386396
SLC12A4	NA	NA	0.292979	0.036464	0.239755	0.226863	-0.31005	-0.24908
SLC12A5	0.154117	-0.07361	0.117947	0.084198	-0.26803	0.045035	-0.04168	-0.17329
SLC12A6	-0.37441	0.453869	0.099362	-0.22128	-0.22891	-0.39501	-0.21039	0.168629
SLC12A7	0.083963	-0.03314	0.332384	0.069167	0.541634	0.073634	0.180406	-0.02964
SLC12A8	-0.2022	0.078246	0.096729	0.076261	0.28841	-0.04654	-0.33676	-0.17382
SLC12A9	0.353653	0.46297	0.287906	0.259395	0.494196	0.149202	-0.05568	-0.03984
SLC13A1	0.047142	-0.03987	-0.06923	-0.44285	0.108603	-0.21303	0.59952	0.578202
SLC13A2	0.380361	-0.16172	-0.03653	-0.00704	0.226047	-0.12435	-0.15752	0.249827
SLC13A3	0.076502	0.568376	0.236743	-0.05222	0.354012	-0.01592	0.279049	0.368487
SLC13A4	0.121974	0.517125	-0.2638	-0.39787	0.040259	-0.00946	0.407166	-0.03146
SLC13A5	-0.14994	0.092666	0.036523	0.04227	0.021873	0.33121	-0.0676	-0.34646
SLC14A1	0.230224	0.353186	0.230578	0.501199	-0.25917	-0.23315	-0.68754	-0.51321
SLC14A2	0.069981	0.670176	-0.02071	-0.05416	-0.13894	-0.28007	0.66228	0.212961
SLC15A1	0.266557	-0.14645	-0.0143	-0.17568	0.087298	-0.22609	0.186924	0.340558
SLC15A2	0.286325	0.291715	0.122254	0.156042	-0.22525	0.071448	0.149602	0.284081
SLC15A3	-0.33221	0.187203	0.357396	0.090401	-0.25188	0.584841	-0.46699	-0.07652

SLC15A4	-0.13054	0.034237	0.430112	0.358923	0.169279	0.543191	-0.30525	0.030161
SLC16A1	0.423628	0.263579	0.102932	-0.40783	0.102665	0.116713	-0.48569	-0.33114
SLC16A10	-0.07921	-0.10669	-0.00899	-0.07331	-0.04067	-0.08562	-0.04612	-0.07952
SLC16A11	0.244758	-0.28937	0.181078	0.054013	0.126312	0.072159	-0.00911	-0.09204
SLC16A12	0.099469	0.511214	-0.26769	-0.12996	0.06106	0.001608	-0.47738	-0.5757
SLC16A13	-0.17999	-0.1496	0.413528	-0.00307	0.090382	0.246984	-0.20661	-0.05245
SLC16A14	0.170534	-0.31332	-0.01583	-0.02663	0.051056	-0.31567	-0.15508	0.084001
SLC16A2	-0.09494	-0.22814	0.094749	0.20681	-0.1551	0.410197	0.038731	0.23597
SLC16A3	-0.63604	0.065311	0.05485	0.196576	0.16697	-0.07715	-0.34517	-0.18115
SLC16A4	-0.16978	-0.28714	0.286659	-0.19735	-0.03556	-0.08107	0.018005	0.421036
SLC16A5	-0.30365	0.272883	0.196053	0.086818	0.224663	-0.0863	0.186692	0.138809
SLC16A6	-0.1778	0.17597	-0.30646	0.12549	0.013425	0.208364	-0.0449	0.305804
SLC16A7	-0.2899	0.622305	-0.19778	0.060486	0.056815	-0.05215	0.127536	0.253113
SLC16A8	0.10117	0.691661	-0.25872	-0.38826	0.041249	-0.05541	-0.30979	-0.21391
SLC16A9	0.314928	0.139398	-0.25409	-0.11192	0.149414	-0.31311	0.069309	-0.29022
SLC17A1	0.321336	0.38885	-0.28557	0.02222	0.123091	0.052108	-0.42661	-0.09021
SLC17A2	0.042919	-0.34212	0.193543	0.25123	-0.1711	-0.41993	0.254743	-0.21954
SLC17A3	-0.14416	0.033496	-0.31026	0.065166	-0.2045	0.110389	0.580865	0.517528
SLC17A4	0.255925	0.087136	-0.20487	-0.30949	-0.11668	-0.29194	0.273416	0.270643
SLC17A5	0.020167	-0.17113	-0.106	-0.117	-0.12201	-0.08075	0.228611	-0.0048
SLC17A6	0.036728	0.510966	-0.03003	0.183716	-0.1138	-0.07725	-0.26999	-0.01019
SLC17A7	-0.28093	-0.35574	0.404472	-0.14123	0.117878	-0.16527	-0.04075	0.393395
SLC17A8	-0.04162	0.094822	-0.223	-0.29514	-0.15106	0.062054	0.179599	0.39957
SLC18A1	-0.05027	0.107573	-0.07688	0.336725	-0.17128	-0.11615	0.270404	0.325532
SLC18A2	-0.10869	-0.03568	-0.15462	0.070777	-0.1399	0.102314	0.176716	0.376738
SLC18A3	NA	NA	-0.12092	-0.15904	0.124298	0.199425	0.155566	-0.22663
SLC19A1	0.280475	0.605957	-0.02119	0.081521	0.205559	0.111517	0.351215	0.244029
SLC19A2	0.127665	0.571289	-0.19876	-0.29436	0.103491	-0.16566	0.009385	-0.31646
SLC19A3	0.433353	0.086303	0.126596	0.049959	0.144986	0.009165	-0.08175	0.191924
SLC1A1	-0.3551	0.349221	-0.0916	-0.52083	-0.05117	-0.35144	-0.06148	0.251976
SLC1A2	0.041592	0.755826	-0.09408	0.626432	-0.25642	0.408653	-0.30014	-0.32413
SLC1A3	-0.23914	0.087639	0.017464	0.195308	-0.35691	0.473446	-0.16873	0.069898
SLC1A4	-0.05665	0.474332	0.218503	0.426045	0.426728	-0.11697	0.295762	0.327589
SLC1A5	0.341588	0.445139	0.299923	0.269582	0.436256	0.060906	0.590447	-0.00149
SLC1A6	-0.31057	0.304379	-0.04667	-0.29265	0.130688	0.088707	0.067149	-0.13737
SLC1A7	0.307345	-0.08945	-0.08045	-0.20464	0.080467	-0.52548	0.183636	0.165192
SLC20A1	-0.38235	0.083979	-0.09221	-0.0459	0.09298	0.110463	0.284894	0.174601
SLC20A2	0.212587	0.384303	0.020079	-0.51259	0.273999	-0.20772	0.176605	-0.08131
SLC22A1	-0.07221	0.09767	-0.32335	-0.11863	0.088732	-0.2294	-0.04122	-0.09732
SLC22A10	NA	NA	0.106805	0.271054	-0.00837	-0.24106	-0.01171	-0.29323
SLC22A11	0.226807	0.293558	0.20112	0.052293	0.033773	-0.0838	-0.07922	-0.28912
SLC22A12	0.526706	0.590819	0.18787	0.152353	-0.19203	0.079231	-0.31797	0.102737
SLC22A13	0.204175	0.314734	0.102796	-0.13463	0.292903	0.096741	0.525745	0.323037
SLC22A14	-0.01821	0.047101	0.246062	-0.38737	0.179842	-0.02251	-0.01559	-0.39662
SLC22A15	-0.19622	0.743346	0.071034	0.02695	0.083391	-0.24014	0.105712	0.351152
SLC22A16	-0.23446	-0.09535	-0.33096	-0.36169	-0.20459	-0.33532	-0.08061	0.172526
SLC22A17	-0.09336	0.043723	0.263202	0.018701	-0.09651	0.030051	-0.27027	-0.33851
SLC22A18	0.324644	0.238483	0.253718	-0.03649	0.063388	-0.17881	0.392536	0.357564
SLC22A18A	0.116535	0.153289	0.155704	-0.25803	-0.18736	-0.46965	0.253781	0.035563
SLC22A2	0.145308	0.174476	-0.19742	-0.00762	-0.26989	-0.29184	0.184896	0.373485
SLC22A23	NA	NA	-0.17978	-0.02518	-0.07903	-0.19602	NA	NA
SLC22A24	NA	NA	0.056992	-0.00518	0.062824	0.132362	NA	NA

SLC22A25	NA	NA	-0.14153	-0.21392	-0.18027	0.20731	NA	NA
SLC22A3	0.37124	-0.29178	0.158758	0.06698	0.483618	0.073463	0.238276	0.144762
SLC22A4	0.071927	-0.0939	0.323383	-0.26525	-0.12164	-0.27388	-0.07209	0.317177
SLC22A5	0.634165	-0.02968	0.334547	-0.15903	0.214032	-0.14262	0.563439	0.185812
SLC22A6	0.146327	0.187405	0.247501	-0.08711	-0.1495	0.230901	-0.02978	0.069972
SLC22A7	0.09566	0.672439	-0.16871	-0.31283	0.264518	0.072773	0.221657	0.101202
SLC22A8	-0.13008	0.231988	0.068619	-0.33621	-0.05339	-0.26283	-0.37285	-0.15053
SLC22A9	0.36093	0.33752	0.070434	0.073282	-0.11738	0.123012	0.481822	0.364814
SLC23A1	0.17624	0.12368	0.21555	-0.15779	0.004433	-0.01837	-0.32376	0.280835
SLC23A2	-0.2309	0.142421	-0.06515	-0.12025	0.054472	0.533221	0.311108	0.400623
SLC23A3	0.059988	0.29658	-0.16089	-0.06724	0.257195	-0.03498	0.183865	0.346751
SLC24A1	0.207705	-0.34016	-0.1229	-0.08982	-0.06932	0.03315	-0.03526	-0.01661
SLC24A2	0.168306	0.493014	-0.01809	-0.15825	-0.02546	-0.17245	0.159248	0.158723
SLC24A3	-0.28434	-0.10331	0.053713	0.123594	-0.04336	-0.0204	-0.39303	-0.46342
SLC24A4	-0.16587	0.025615	-0.16605	0.252639	-0.26945	0.300965	-0.40039	-0.2803
SLC24A5	-0.23984	0.367915	NA	NA	NA	NA	-0.15194	-0.01041
SLC24A6	-0.37221	0.13821	-0.1416	-0.12161	0.17407	0.33173	-0.63502	-0.12676
SLC25A1	-0.04943	0.487895	0.49122	0.084836	0.182388	-0.14318	0.671109	0.420551
SLC25A10	0.068234	0.470326	0.464601	0.07626	0.275345	-0.11142	0.105855	-0.29719
SLC25A11	0.110183	0.66635	0.242996	0.052645	0.003015	-0.01439	-0.47299	-0.50208
SLC25A12	0.168313	-0.20078	0.130571	-0.19396	-0.12369	-0.25953	-0.14646	-0.18748
SLC25A13	0.300011	0.33454	0.164886	-0.26628	0.094893	-0.31222	-0.10707	-0.36525
SLC25A14	0.203334	0.533424	-0.32579	0.043726	0.000316	-0.03781	-0.10184	-0.14226
SLC25A15	0.318096	-0.00935	0.173733	0.098809	0.30984	0.041627	0.26729	0.453988
SLC25A16	-0.06667	0.437081	-0.15306	-0.16849	-0.16372	-0.09334	0.011585	0.191018
SLC25A17	-0.332	-0.61809	-0.11949	-0.05235	-0.06658	0.022109	0.19465	0.459066
SLC25A18	0.296949	-0.3122	-0.28648	-0.08852	-0.24281	0.187973	0.06026	0.070973
SLC25A19	-0.16714	0.52965	0.16753	0.177546	0.050594	0.118712	-0.44364	-0.38251
SLC25A2	-0.29929	0.286648	-0.16898	-0.37755	0.185343	0.059315	0.033149	-0.1046
SLC25A20	-0.22167	-0.41306	0.217019	-0.18071	0.012146	-0.30221	-0.18968	0.115059
SLC25A21	0.134977	-0.26641	-0.14617	-0.15289	-0.23231	-0.10375	-0.01939	0.21129
SLC25A22	0.2291	0.172308	-0.20225	0.379864	0.085557	0.224243	0.099746	0.305811
SLC25A23	0.171096	0.36961	0.014868	-0.39563	0.03019	-0.16861	-0.09504	-0.24228
SLC25A24	-0.23596	0.54827	-0.43544	-0.08764	0.090418	-0.32999	0.331105	0.282263
SLC25A25	-0.16198	0.302164	0.048731	-0.09284	0.114084	-0.04286	-0.39333	0.060499
SLC25A26	0.163998	-0.28657	-0.04329	-0.02381	-0.00049	-0.25649	0.206274	0.016132
SLC25A27	0.334516	-0.3221	0.110045	0.034161	0.37228	0.248743	0.412617	0.120902
SLC25A28	-0.13678	0.319827	0.354611	-0.07148	0.262255	0.363728	-0.64956	-0.45362
SLC25A29	-0.24222	-0.57874	0.074096	0.072264	0.251795	0.385554	0.038718	0.228494
SLC25A3	-0.13313	-0.20339	-0.12502	-0.10564	0.043967	-0.13268	-0.28506	-0.42873
SLC25A30	0.020125	0.389285	0.134418	-0.01209	-0.06167	-0.32404	-0.19754	-0.32418
SLC25A31	0.014886	0.703064	0.065138	0.132191	-0.00449	-0.04856	-0.38424	0.050334
SLC25A32	0.029124	-0.43707	-0.11536	-0.12454	0.068587	-0.04385	-0.57397	-0.27852
SLC25A33	-0.02721	-0.50425	-0.12499	-0.0626	0.290007	-0.22571	NA	NA
SLC25A34	-0.242	0.577021	NA	NA	NA	NA	-0.37921	0.054629
SLC25A35	-0.05629	0.229365	0.208606	-0.02153	0.13351	-0.21698	0.141573	0.476323
SLC25A36	0.047423	-0.60504	-0.25588	-0.25561	-0.02039	0.205272	0.486788	0.359609
SLC25A37	-0.2653	0.466681	0.183058	0.093896	0.178891	0.300813	0.057344	0.594124
SLC25A38	0.029311	0.334565	-0.16063	0.168273	0.131729	-0.05398	NA	NA
SLC25A39	0.322009	0.422851	0.393362	0.337336	0.231705	0.109537	NA	NA
SLC25A4	-0.23252	-0.3623	0.14222	-0.11456	0.042177	-0.03822	0.099104	-0.13486
SLC25A40	-0.16146	-0.51096	-0.14636	0.020538	-0.02046	-0.48816	0.421913	0.338394

SLC25A41	-0.01565	-0.0631	-0.18553	-0.2539	0.056367	-0.01571	NA	NA
SLC25A42	-0.22335	0.005709	0.33764	0.103518	0.301405	0.073489	-0.26555	-0.01411
SLC25A43	0.28219	-0.36125	-0.07918	-0.05377	-0.14441	-0.48719	0.02725	0.093381
SLC25A44	-0.30781	0.148338	-0.29963	0.125889	-0.25142	0.071225	NA	NA
SLC25A45	-0.27461	0.292025	0.223436	0.220625	0.275054	0.449963	0.218302	0.488072
SLC25A46	0.312579	-0.16257	-0.03738	-0.25589	-0.0399	-0.36788	NA	NA
SLC25A5	0.156622	-0.18153	0.08637	0.002297	0.210606	-0.06787	0.106712	0.215193
SLC25A6	0.280712	0.446092	0.43556	0.159729	0.436136	0.234505	NA	NA
SLC26A1	0.208637	0.525552	0.03844	-0.18477	0.026362	0.08847	-0.09805	0.462652
SLC26A10	0.169384	-0.48576	0.028473	-0.04047	0.111538	0.145864	-0.32019	-0.28482
SLC26A11	0.079581	0.380759	-0.00755	0.123513	-0.12645	0.179475	0.018877	0.518688
SLC26A2	0.238034	-0.00869	-0.11014	-0.24393	-0.15256	-0.0381	-0.36269	-0.58814
SLC26A3	0.2245	0.173962	0.182836	-0.10502	-0.14713	-0.0218	-0.0718	-0.07191
SLC26A4	-0.14417	-0.18867	0.047886	0.044667	0.021036	0.215477	-0.51518	-0.60921
SLC26A5	0.342699	0.653466	NA	NA	NA	NA	0.468377	0.297151
SLC26A6	0.046966	0.303323	-0.16251	0.074539	0.420379	0.339491	0.024299	-0.27779
SLC26A7	0.133964	0.457346	-0.1635	-0.2467	0.12422	-0.13283	0.350953	0.120504
SLC26A8	0.310216	0.803308	-0.15742	-0.29048	0.240346	-0.47677	0.268266	0.480442
SLC26A9	-0.36359	0.265912	-0.28672	0.365119	-0.29677	-0.12054	-0.0374	-0.22942
SLC27A1	0.067079	0.268374	-0.06807	0.034919	-0.01806	-0.17292	0.066531	-0.04218
SLC27A2	0.011962	0.034872	-0.08184	0.064354	0.041221	-0.3606	0.241556	0.184488
SLC27A3	-0.60506	0.113894	-0.17084	0.214993	-0.13483	-0.04586	-0.29576	-0.43863
SLC27A4	-0.2078	0.710089	0.335248	0.12713	0.270478	-0.12473	-0.34331	-0.28957
SLC27A5	0.222791	-0.14304	-0.18829	0.029919	-0.11194	-0.22161	-0.19121	-0.30827
SLC27A6	0.308639	0.361954	0.106932	-0.01198	0.012513	0.335597	-0.072	-0.2443
SLC28A1	0.189923	0.672242	0.017925	-0.27936	0.048876	-0.0334	-0.24742	0.431554
SLC28A2	-0.0067	-0.16522	-0.09516	0.010679	-0.15857	0.032032	NA	NA
SLC28A3	-0.2211	0.402693	-0.06913	0.265197	-0.14024	0.090497	0.220403	0.303853
SLC29A1	0.3758	-0.13423	-0.17639	0.036587	0.24211	0.313245	0.369469	-0.02293
SLC29A2	0.148531	0.469782	0.312899	0.072743	0.24759	0.14617	0.274618	-0.02631
SLC29A3	0.429983	0.237863	0.093073	0.138724	0.120416	0.125043	0.092161	-0.20643
SLC29A4	-0.07778	0.108251	-0.17028	0.058739	0.049977	-0.11632	0.499239	0.177475
SLC2A1	-0.13916	0.100081	-0.03029	-0.05195	0.04845	0.050677	0.3908	-0.3177
SLC2A10	-0.41219	-0.6044	-0.15807	-0.04934	-0.14271	-0.36178	0.305117	-0.00257
SLC2A11	-0.63616	-0.14968	0.116325	0.115499	0.24442	0.310388	0.287148	0.116317
SLC2A12	0.56137	-0.2447	0.081991	-0.15948	0.07724	-0.55305	0.100708	-0.24867
SLC2A13	-0.13071	0.148463	-0.2427	-0.27504	0.02472	-0.09826	0.373559	0.377189
SLC2A14	-0.57876	-0.0672	0.042478	0.320829	-0.02901	0.2845	-0.28615	-0.13779
SLC2A2	0.351607	0.027786	-0.2301	-0.2977	0.075334	0.192934	0.246399	0.522211
SLC2A3	-0.47376	-0.08987	0.072835	0.417997	-0.04103	0.266416	-0.2671	-0.11483
SLC2A4	0.350255	0.129592	-0.10506	-0.19183	0.332773	-0.04778	-0.37604	-0.05255
SLC2A4RG	0.03086	0.113071	0.220011	-0.07321	0.494513	-0.17513	-0.07602	-0.4603
SLC2A5	-0.70865	-0.09611	0.160497	0.250874	-0.20086	-0.14505	-0.21132	0.449733
SLC2A6	-0.20679	0.302787	0.242368	0.59663	-0.03966	0.490139	-0.58878	-0.10337
SLC2A7	0.483914	0.136257	NA	NA	NA	NA	0.151894	0.298146
SLC2A8	0.331799	0.295976	0.241824	0.031393	0.346057	0.044167	0.146201	-0.11329
SLC2A9	0.454385	0.110764	0.168728	-0.0075	0.266627	-0.04548	0.37457	0.524278
SLC30A1	0.134309	-0.18779	0.011566	-0.32432	-0.08253	0.083808	-0.08651	0.124396
SLC30A10	0.184022	-0.00705	-0.213	-0.30596	-0.13823	-0.21025	0.363374	0.098381
SLC30A2	-0.00549	-0.10999	0.29932	0.188534	0.131184	-0.15669	0.273313	0.108712
SLC30A3	0.387643	0.755983	-0.0638	0.255698	0.084528	-0.19868	0.037411	-0.28797
SLC30A4	0.227135	0.567792	0.342485	-0.29813	-0.15626	-0.24656	-0.67715	-0.40363

SLC30A5	-0.04398	-0.50974	0.033121	0.192418	-0.15491	-0.2973	0.002192	0.070296
SLC30A6	-0.13011	-0.20168	-0.17889	-0.05675	-0.17754	-0.18074	0.394044	0.294629
SLC30A7	-0.2941	0.265827	0.049462	-0.18057	0.160462	-0.03887	-0.27535	0.008803
SLC30A8	0.09005	0.39385	0.047404	0.045782	0.337274	-0.32969	0.110117	-0.06583
SLC30A9	0.221849	0.410397	-0.29109	-0.21368	-0.27545	-0.37749	-0.25234	-0.30486
SLC31A1	-0.43015	-0.28989	0.130002	-0.10575	-0.02324	-0.05441	NA	NA
SLC31A2	-0.04687	-0.3677	0.056221	-0.13095	-0.37788	-0.38445	-0.1032	0.333619
SLC32A1	-0.08597	0.157488	0.017293	-0.08014	-0.017	0.190789	0.070687	0.063955
SLC33A1	-0.14893	-0.61519	-0.1347	0.295961	0.173199	-0.15807	-0.04799	-0.08234
SLC34A1	0.16156	0.446223	0.009608	-0.20583	0.390084	0.139957	-0.64143	-0.41058
SLC34A2	0.313191	0.787782	-0.2477	-0.06755	0.119909	-0.1161	-0.11406	0.175188
SLC34A3	0.118057	0.198981	0.042468	-0.06048	-0.14823	0.22068	0.295742	0.407539
SLC35A1	-0.17325	0.085019	-0.29147	0.005517	-0.24901	-0.08242	0.229194	-0.03606
SLC35A2	0.20011	0.140019	0.02754	0.271486	0.026645	-0.38623	0.458121	0.39891
SLC35A3	-0.23844	0.004163	-0.00473	-0.1973	0.145218	-0.18272	0.206679	0.063954
SLC35A4	0.254097	0.571391	0.463496	0.050901	0.237661	0.18711	0.402936	0.335158
SLC35A5	0.065443	-0.32313	-0.32514	-0.28292	-0.31882	-0.40128	-0.64356	-0.24413
SLC35B1	-0.09634	0.011484	0.140206	0.262619	-0.08991	-0.19316	0.598117	0.683316
SLC35B2	0.248231	0.112598	0.191918	0.375262	0.348951	0.066555	0.12417	-0.09873
SLC35B3	-0.05022	-0.13938	-0.1376	-0.26907	-0.06766	-0.28712	0.400469	0.065811
SLC35B4	0.196307	0.659705	-0.07067	0.141433	0.072976	-0.23624	-0.43772	0.009018
SLC35C1	-0.09472	0.285903	-0.18568	0.220982	0.072924	0.015515	0.288735	0.404876
SLC35C2	0.411704	0.678496	0.33971	0.262686	0.322694	0.037573	0.398821	-0.00394
SLC35D1	0.011573	0.195847	0.089811	-0.07166	0.115878	-0.55497	0.294881	0.239565
SLC35D2	0.114314	0.165382	0.258373	0.028944	0.204838	-0.0748	0.34936	0.248026
SLC35D3	0.140787	-0.13745	-0.07874	-0.08112	-0.03639	0.033466	0.007773	-0.3001
SLC35E1	0.095093	0.067633	0.221257	0.063677	0.296906	0.325359	-0.47391	-0.44998
SLC35E2	0.006767	-0.23247	0.330818	0.092596	0.149641	0.068629	-0.00782	0.373837
SLC35E3	-0.24628	-0.04793	0.063617	-0.24105	0.086538	-0.07586	-0.13215	-0.19319
SLC35E4	-0.09037	-0.39615	0.06836	-0.07923	0.283809	0.389707	0.214419	0.010821
SLC35F1	0.135566	0.315137	-0.27314	-0.035	-0.36903	0.263893	-0.32533	-0.07505
SLC35F2	0.207797	-0.30253	-0.2211	0.081816	-0.15757	-0.2399	-0.0505	-0.01844
SLC35F3	0.382837	0.670895	-0.03085	-0.17288	-0.25699	-0.16177	-0.10167	0.201208
SLC35F4	NA	NA	-0.40991	-0.07849	0.065845	0.048997	0.30949	0.261948
SLC35F5	NA	NA	0.073608	-0.18977	-0.05372	-0.36976	0.355097	3.67E-05
SLC36A1	0.031741	0.036228	0.077371	-0.17792	-0.03967	0.030876	-0.55867	-0.12414
SLC36A2	-0.38651	-0.04291	-0.15944	0.06024	-0.29985	0.390873	0.227112	0.064579
SLC36A3	0.048645	0.515209	0.053241	-0.04512	-0.05503	-0.17015	0.079439	-0.25753
SLC36A4	-0.27752	-0.13559	-0.24915	0.026028	-0.4121	-0.33169	-0.21927	-0.38844
SLC37A1	-0.2131	0.275665	0.100474	0.19362	0.172104	-0.11296	0.135068	0.117754
SLC37A2	NA	NA	0.0931	-0.33638	-0.27245	-0.23701	0.191697	0.053663
SLC37A3	-0.04055	-0.18648	-0.15373	-0.08151	0.02105	-0.16195	0.552751	0.451761
SLC37A4	0.176411	0.11813	0.26169	0.005064	0.051973	-0.25257	0.033338	0.174894
SLC38A1	0.307371	-0.26448	-0.32732	-0.18487	0.056532	0.208666	0.047255	0.04749
SLC38A10	NA	NA	0.29164	0.190181	0.355691	0.0186	NA	NA
SLC38A11	NA	NA	0.424666	-0.1157	-0.11749	0.04682	NA	NA
SLC38A2	-0.23283	-0.22286	-0.2804	-0.06898	-0.14647	-0.27232	-0.54592	-0.38263
SLC38A3	-0.04011	0.445155	0.182359	-0.23911	0.136011	-0.17318	0.216588	-0.17979
SLC38A4	-0.11163	0.19356	-0.10658	-0.0298	-0.10107	-0.10969	NA	NA
SLC38A5	0.39819	0.561467	0.120785	0.291978	-0.09878	0.032245	0.092208	-0.01482
SLC38A6	-0.10053	-0.02664	-0.14861	-0.41031	-0.34497	-0.10898	-0.50016	-0.26607
SLC38A7	NA	NA	0.223892	-0.31113	0.215093	0.229241	NA	NA

SLC38A9	NA	NA	-0.21683	-0.20972	-0.08163	-0.27026	NA	NA
SLC39A1	0.256352	-0.06618	0.340297	0.122082	0.241114	-0.03296	0.195427	0.352791
SLC39A10	0.205788	-0.41167	-0.31357	0.116545	-0.20599	0.140288	0.386073	-0.03109
SLC39A11	-0.11496	0.088331	0.374232	0.272309	0.143096	-0.2421	0.473874	0.105699
SLC39A12	0.092772	-0.09958	0.168645	-0.23749	-0.16225	-0.02723	0.310301	0.119845
SLC39A13	0.230572	0.555106	0.065201	0.104139	-0.02466	-0.04963	-0.1543	-0.18908
SLC39A14	0.092674	-0.33843	0.405697	-0.35204	0.271618	-0.14313	0.101789	0.213086
SLC39A2	0.022949	-0.12108	-0.04784	-0.13933	-0.19189	-0.26343	0.023682	-0.01476
SLC39A3	0.468639	-0.07522	0.152533	0.082039	0.165059	-0.16556	0.260091	-0.00387
SLC39A4	0.107352	0.607016	0.132615	-0.05816	-0.00718	-0.17994	0.655527	0.295169
SLC39A5	0.3859	0.293772	0.268669	0.018424	0.278589	-0.08276	0.07823	-0.13626
SLC39A6	-0.01157	-0.57358	0.283092	0.063531	-0.23884	-0.1154	-0.115	-0.363
SLC39A7	-0.13187	0.482998	0.241283	0.30622	-0.07155	-0.02013	-0.33872	-0.26651
SLC39A8	-0.30106	-0.26023	-0.07414	0.081311	-0.11035	-0.39408	0.141276	-0.22289
SLC39A9	-0.00899	0.057249	-0.46828	0.019873	-0.20475	-0.39414	0.170588	0.186926
SLC3A1	-0.38072	0.510604	0.396584	-0.2802	0.389516	-0.3052	0.063213	0.317916
SLC3A2	0.000956	0.30995	0.262227	0.143921	0.366307	0.108898	0.525083	0.470487
SLC40A1	0.025274	-0.23532	0.357608	-0.06758	-0.15676	-0.25724	0.119213	0.214163
SLC41A1	-0.2519	0.027556	0.045183	0.182891	-0.02014	0.451493	-0.20301	-0.15639
SLC41A2	-0.2686	0.124509	0.05104	-0.47303	-0.28691	-0.38361	-0.17549	0.368966
SLC41A3	0.268321	0.495106	0.00297	0.110983	-0.21473	0.053585	-0.16873	-0.47248
SLC43A1	0.176158	0.255528	-0.0431	0.470001	0.078054	0.05453	-0.25178	-0.3037
SLC43A2	0.019007	-0.01796	-0.24829	0.314907	-0.09996	-0.03563	-0.45334	0.306577
SLC43A3	-0.27744	-0.14591	0.022268	0.15223	-0.03339	0.497954	-0.37801	-0.00198
SLC44A1	0.057846	0.219483	-0.18022	-0.20561	0.058175	-0.07195	0.214745	0.023176
SLC44A2	-0.13541	0.402688	0.155105	0.301875	0.142316	0.228037	-0.28535	-0.40199
SLC44A3	-0.32848	-0.17926	0.209924	-0.06555	0.137368	-0.30267	0.470927	0.26649
SLC44A4	-0.18263	0.031314	0.080216	0.181732	0.154858	0.087373	0.098764	0.119798
SLC44A5	0.429125	0.753968	0.048064	-0.07256	-0.02847	-0.00856	-0.07303	0.041046
SLC45A1	NA	NA	0.160914	-0.05044	0.225	-0.10356	-0.30338	0.197066
SLC45A2	0.311292	0.017451	-0.05273	-0.22255	0.205078	0.021492	-0.12225	-0.26183
SLC45A3	-0.14041	-0.07979	-0.3231	0.181558	-0.2283	-0.14549	0.379763	0.222555
SLC45A4	NA	NA	0.350809	0.069915	-0.07378	-0.17713	0.356645	0.423161
SLC46A1	NA	NA	0.117012	-0.16388	-0.17307	0.004379	NA	NA
SLC46A2	NA	NA	0.065981	-0.14371	0.116581	-0.2288	NA	NA
SLC46A3	NA	NA	0.338166	-0.29241	-0.03061	-0.43612	NA	NA
SLC47A1	NA	NA	0.227343	-0.04683	0.062215	0.00353	NA	NA
SLC47A2	NA	NA	-0.01056	-0.44548	0.094894	-0.27512	NA	NA
SLC48A1	NA	NA	0.096442	0.031138	0.171745	-0.10782	NA	NA
SLC4A1	0.509522	0.404447	0.153004	-0.22592	0.045349	-0.05026	0.054794	-0.11229
SLC4A10	-0.17399	0.572665	-0.05162	-0.14507	-0.251	0.087412	-0.58209	-0.05005
SLC4A11	-0.08695	-0.10761	-0.42578	0.032104	-0.08836	-0.1595	0.280448	0.139116
SLC4A1AP	0.002747	-0.29457	0.082416	-0.01249	0.050053	0.218877	-0.48975	-0.54955
SLC4A2	0.211678	0.315979	0.204669	0.091434	0.214341	0.002607	0.356229	0.274789
SLC4A3	-0.20294	-0.45596	0.435125	-0.08482	0.08939	-0.01213	-0.49179	-0.13397
SLC4A4	-0.58493	-0.22209	-0.01639	-0.40544	-0.0641	-0.05844	-0.15504	-0.47215
SLC4A5	0.130798	-0.22453	-0.00117	-0.36812	-0.08081	0.135353	-0.58361	-0.51375
SLC4A7	-0.01592	-0.36581	0.254411	-0.17614	0.202944	0.282088	-0.51023	0.125009
SLC4A8	0.032856	0.461498	-0.04768	0.3736	0.124247	0.215315	-0.09724	0.193892
SLC4A9	0.089386	0.037776	0.303008	-0.31213	-0.11296	0.060799	-0.36462	-0.0539
SLC5A1	-0.01206	0.351135	0.247796	0.494768	0.222775	0.05901	0.241482	0.449669
SLC5A10	0.350651	0.491516	-0.40654	-0.558	0.058899	-0.02879	0.07964	-0.05749

SLC5A11	0.246067	-0.36743	-0.42431	-0.50455	0.068368	-0.07585	0.442794	0.451845
SLC5A12	-0.05928	0.19516	-0.22607	-0.57578	0.086809	-0.49041	-0.00964	0.497405
SLC5A2	0.212958	0.544876	0.262273	-0.18947	-0.02835	-0.20603	-0.07681	-0.10944
SLC5A3	0.020942	0.290515	-0.19448	-0.17023	0.064353	-0.34894	-0.53473	-0.55251
SLC5A4	0.027683	-0.2537	0.081802	0.077991	-0.08382	0.15591	-0.24487	0.471593
SLC5A5	0.324121	0.417701	-0.01136	-0.19695	-0.08758	0.010995	0.203187	0.079693
SLC5A6	0.447903	0.491456	0.355042	0.425321	0.526619	0.200088	0.631323	0.593937
SLC5A7	-0.30431	-0.24867	-0.04045	-0.00976	0.150682	-0.24253	0.190504	-0.25442
SLC5A8	-0.03285	0.178057	0.006301	-0.27806	-0.25046	0.032251	-0.65396	-0.58833
SLC5A9	0.363204	-0.04793	0.35518	-0.00568	0.130267	-0.01789	0.400717	0.41126
SLC6A1	-0.26467	-0.45341	0.219369	-0.06897	0.004333	0.097448	0.192973	0.14465
SLC6A10P	-0.06991	-0.02374	0.080846	0.01168	0.026291	0.066106	NA	NA
SLC6A11	0.190461	0.389286	0.075373	0.172717	0.015401	-0.21292	-0.41649	-0.16912
SLC6A12	-0.18675	-0.22454	0.099154	-0.2118	-0.28537	-0.27899	-0.03505	0.09642
SLC6A13	0.272505	0.728432	-0.15962	0.103671	0.009134	-0.17339	-0.35843	-0.4232
SLC6A14	-0.20691	0.166545	-0.00341	0.090566	0.033823	-0.01992	0.193764	0.315767
SLC6A15	-0.14219	-0.06694	-0.17035	-0.21647	0.034788	0.077719	-0.04331	-0.01244
SLC6A16	-0.12356	0.418537	0.041374	0.328433	-0.07378	0.281101	-0.23154	0.133644
SLC6A17	-0.00242	0.612537	-0.09053	-0.06462	-0.10229	-0.41618	-0.01283	0.132932
SLC6A18	0.224532	0.449417	0.357139	-0.2536	0.150018	-0.0801	-0.35267	-0.19654
SLC6A19	0.232629	-0.2701	0.094976	-0.02753	-0.01801	-0.14813	-0.17548	0.335926
SLC6A2	0.213916	0.711218	0.171857	-0.1806	-0.25665	0.255471	-0.5245	-0.23157
SLC6A20	0.243575	0.619389	0.144853	0.040116	0.143843	0.112714	-0.03689	0.470013
SLC6A3	-0.00107	0.413895	-0.194	-0.09563	0.184711	-0.07568	0.234878	0.249665
SLC6A4	0.26569	0.151545	0.159865	-0.31604	0.324466	0.063931	0.340725	0.514434
SLC6A5	0.181646	0.47159	0.045611	-0.01783	-0.08234	-0.32674	-0.45139	-0.26107
SLC6A6	0.230742	-0.40906	0.120339	0.219699	0.142125	0.379885	0.227029	0.550585
SLC6A7	-0.15383	0.325712	0.1373	0.026095	-0.13407	0.063275	-0.1892	-0.19381
SLC6A8	-0.32159	0.261446	0.00466	-0.05162	-0.02723	-0.04804	-0.32687	0.138281
SLC6A9	0.132211	0.461682	0.038178	-0.08575	0.12404	-0.11532	-0.2823	0.005568
SLC7A1	0.077839	0.66458	0.013613	0.228205	0.571371	0.2541	0.20287	-0.09819
SLC7A10	0.452463	-0.07252	-0.13528	0.56517	-0.00511	0.257389	0.308117	0.233992
SLC7A11	0.058753	0.323088	-0.17619	-0.07027	0.089042	0.021871	0.016011	0.29218
SLC7A13	0.009403	0.136668	-0.01735	0.146412	0.219727	0.109214	0.017838	0.024917
SLC7A14	-0.28933	-0.30848	0.117604	0.044482	-0.25086	0.224954	-0.35246	-0.20505
SLC7A2	0.072624	-0.43692	0.098643	0.105277	0.06622	-0.28666	0.01287	0.228812
SLC7A3	0.062254	-0.07581	0.089266	-0.14038	-0.07566	0.085134	0.604649	0.43019
SLC7A4	0.415531	0.789646	0.294746	-0.00515	0.236489	-0.24018	0.417058	0.144815
SLC7A5	-0.0496	0.307954	0.264405	0.31627	0.411492	0.322561	0.249665	0.258229
SLC7A6	0.257633	0.124067	0.158543	0.101969	0.048838	0.29384	0.191077	0.350173
SLC7A6OS	0.056489	-0.22838	-0.26763	0.037362	0.248974	-0.11453	0.681904	0.052088
SLC7A7	-0.35256	-0.24947	-0.05469	-0.1157	-0.25445	0.174888	-0.1547	0.576508
SLC7A8	-0.03972	0.302778	0.233056	0.450955	-0.12199	0.061442	0.154825	0.354798
SLC7A9	0.102667	-0.24767	0.559681	-0.15179	-0.09916	0.061449	0.326057	0.484632
SLC8A1	-0.19393	0.137081	0.080445	-0.23054	-0.42233	0.131892	-0.36008	-0.26298
SLC8A2	0.243896	-0.00912	-0.22061	-0.22629	0.094887	0.16289	-0.34256	-0.34855
SLC8A3	0.359464	0.477211	-0.10992	0.209375	-0.00902	0.198467	0.077427	0.269969
SLC9A1	-0.45849	0.196642	0.055407	0.056489	0.223118	0.149078	-0.30247	-0.01927
SLC9A10	-0.163	0.129434	0.086575	0.207385	0.098857	0.142783	0.121386	0.028651
SLC9A11	0.075045	0.567408	0.090277	-0.22114	-0.2191	0.088628	0.296389	-0.00428
SLC9A2	-0.27807	0.183756	-0.14678	-0.0737	-0.09732	-0.01688	-0.02524	-0.13094
SLC9A3	0.156006	0.155303	-0.20706	-0.24307	0.253176	0.001794	-0.0738	0.073668

SLC9A3R1	0.366696	0.240865	0.089217	-0.10103	0.198115	-0.28154	0.073198	0.14238
SLC9A3R2	-7.2E-05	-0.00707	0.105634	0.136325	-0.12932	0.121701	-0.05575	0.089433
SLC9A4	-0.19605	0.55861	NA	NA	NA	NA	0.63545	0.225697
SLC9A5	-0.0316	0.734471	-0.25692	0.086677	-0.06778	-0.21779	0.081712	0.103969
SLC9A6	-0.0066	-0.43898	-0.02604	-0.14312	-0.01906	0.015958	0.167259	0.387999
SLC9A7	0.306582	0.156294	0.054465	0.44419	-0.12446	0.193348	0.126787	0.086226
SLC9A8	0.127473	-0.35073	0.111939	-0.00948	0.452023	0.191582	0.285144	0.338051
SLC9A9	-0.36906	-0.19705	0.011342	0.13214	-0.36883	0.385092	-0.32703	0.17248
SLCO1A2	0.082441	0.55599	-0.0757	0.216302	-0.33923	0.071875	0.06831	-0.41097
SLCO1B1	-0.01215	0.724093	0.031985	-0.16564	-0.2183	0.216739	0.044967	0.3013
SLCO1B3	0.149568	0.106695	-0.02105	0.018704	-0.10154	-0.1513	0.134995	-0.04676
SLCO1C1	0.143974	0.037252	0.067934	0.005761	-0.11795	-0.05635	-0.3262	-0.25787
SLCO2A1	-0.21465	-0.10506	0.142415	-0.08911	-0.13435	0.139551	-0.13988	-0.14186
SLCO2B1	-0.06211	-0.11289	-0.0914	0.315134	-0.23629	0.058969	-0.34443	0.435508
SLCO3A1	0.15759	0.235182	0.104781	0.131365	0.456684	0.4236	-0.28685	-0.03774
SLCO4A1	0.21366	0.14806	0.191142	0.267093	0.310594	0.119631	0.376521	0.235856
SLCO4C1	0.185241	0.271507	-0.02659	-0.23235	-0.37385	-0.12606	-0.11539	-0.03342
SLCO5A1	-0.05616	0.163518	0.055816	0.234123	-0.01461	0.061422	0.092374	-0.19441
SLCO6A1	0.424824	-0.08731	-0.22241	-0.08396	-0.0136	-0.1283	-0.31187	-0.31142
SLFN11	-0.22632	-0.28126	0.010012	0.303362	-0.40308	0.491795	-0.64248	-0.12683
SLFN12	-0.48965	-0.44931	0.066328	-0.0665	-0.35796	0.044023	NA	NA
SLFN13	-0.44334	0.493334	0.115791	0.029298	-0.16749	0.307387	-0.34389	0.036479
SLFN5	-0.03273	0.142055	0.08323	0.244227	-0.26943	0.491844	NA	NA
SLFNL1	-0.1783	0.122599	0.054398	-0.03573	0.366859	0.332667	NA	NA
SLIT1	-0.15937	0.382939	0.012485	-0.01973	0.175592	0.104924	0.554743	0.407841
SLIT2	0.137375	-0.09637	0.200497	0.073163	-0.21143	0.205092	-0.29601	-0.38138
SLIT3	-0.0902	-0.28863	0.341835	0.041419	-0.00149	0.124298	-0.26583	-0.00075
SLITRK1	0.150538	0.390292	0.322353	-0.27262	0.041575	0.200936	0.257884	-0.15352
SLITRK2	-0.11921	0.269609	0.170882	-0.12453	0.310674	0.257467	-0.08287	0.153154
SLITRK3	0.199163	0.187498	0.006714	-0.12816	0.134055	-0.04404	-0.19928	0.03777
SLITRK4	-0.54554	-0.20188	0.161647	0.09913	-0.12153	-0.06074	-0.32234	0.032418
SLITRK5	-0.14935	-0.2735	-0.04661	-0.00258	0.021262	-0.2257	0.159564	-0.26345
SLITRK6	-0.00768	-0.20798	-0.08791	-0.11637	-0.13053	-0.04706	0.058176	-0.05598
SLK	-0.34886	-0.63095	-0.32558	-0.27647	0.16966	0.040276	-0.4241	-0.18841
SLMAP	0.055535	-0.1157	-0.46653	-0.27436	-0.03733	-0.31592	-0.41585	-0.28686
SLMO1	0.265949	0.36937	-0.31272	0.222301	0.062645	-0.1166	-0.44184	-0.05735
SLMO2	0.116703	-0.2551	0.029989	-0.22775	0.134166	-0.3208	NA	NA
SLN	-0.60234	-0.09993	-0.17414	-0.18344	-0.26548	-0.37523	-0.09955	-0.11873
SLPI	-0.04992	-0.01368	-0.18763	-0.02207	-0.269	-0.2111	0.327886	-0.15835
SLTM	-0.26597	-0.53634	-0.19964	0.222681	0.06875	0.275355	-0.40248	0.065856
SLU7	0.402299	-0.4168	0.092585	-0.18031	0.056734	-0.32541	0.333857	0.409366
SLURP1	-0.0796	0.690878	-0.09932	-0.21899	0.060959	-0.10993	-0.16359	-0.16444
SMA4	0.05725	-0.50736	0.043032	-0.12682	0.18153	0.319535	-0.23385	-0.27659
SMA5	-0.17094	0.051828	NA	NA	NA	NA	0.027691	-0.15046
SMAD1	-0.01837	0.778371	-0.26448	-0.19158	-0.19895	-0.19319	0.088151	0.27608
SMAD2	0.086395	0.344415	0.110762	-0.33185	-0.25327	-0.42107	-0.15601	0.132773
SMAD3	0.014883	-0.29176	-0.11068	-0.19188	0.186146	0.165062	-0.06143	0.31473
SMAD4	-0.08282	-0.69594	-0.08603	-0.34634	-0.30373	-0.07518	0.001876	0.175542
SMAD5	0.220495	-0.37531	-0.24442	-0.1924	-0.16436	-0.31186	-0.05898	-0.4037
SMAD6	0.021111	-0.32638	0.272919	0.286819	0.386488	0.07555	-0.1467	-0.37001
SMAD7	0.057882	-0.39687	0.142988	-0.02644	-0.05617	0.007081	-0.07502	-0.2466
SMAD9	-0.43144	-0.2925	-0.02752	0.016952	0.166673	-0.17857	-0.19639	-0.58039

SMAP1	0.31656	0.148854	-0.27577	-0.09181	-0.00964	-0.0111	0.442957	0.267046
SMAP2	NA	NA	0.197361	0.667745	0.172247	0.288444	NA	NA
SMARCA1	-0.21568	-0.50005	-0.02577	-0.22913	-0.185	0.459869	-0.08498	-0.18752
SMARCA2	-0.18307	-0.24352	-0.29785	0.182942	0.057003	0.104576	-0.51019	-0.19842
SMARCA4	0.290121	0.342629	0.026756	0.443202	0.328976	0.371293	-0.05879	-0.41457
SMARCA5	-0.14362	-0.59018	-0.15361	0.041508	-0.30992	-0.28859	-0.44385	-0.42419
SMARCAD1	0.017911	-0.72826	-0.14478	-0.10692	0.023981	0.358581	0.456322	0.225424
SMARCAL1	0.179375	-0.14254	0.511919	0.253501	-0.03167	-0.14975	0.050413	-0.06383
SMARCB1	-0.09295	-0.26231	0.211213	0.374658	0.052206	-0.01615	0.634244	0.227044
SMARCC1	0.222074	-0.38576	0.007767	0.436214	0.407969	0.368852	0.437312	-0.01527
SMARCC2	0.361408	0.062455	0.016494	0.093454	-0.01584	0.330863	0.569347	0.011393
SMARCD1	0.445067	0.521772	0.030401	0.262009	0.128214	-0.01278	0.185044	0.227313
SMARCD2	0.195201	0.304075	0.377734	0.363829	0.455906	0.205753	0.234298	-0.06711
SMARCD3	0.008713	0.213407	-0.01725	-0.04011	-0.19275	-0.25708	-0.23971	-0.19771
SMARCE1	0.307981	0.034247	-0.02137	0.158086	-0.1238	-0.1894	-0.34211	-0.30972
SMC1A	-0.20847	0.194709	-0.20208	0.361998	0.290082	0.335641	NA	NA
SMC1B	-0.04235	0.215314	-0.25511	0.028656	-0.04468	0.143392	NA	NA
SMC2	-0.1238	-0.23669	-0.37995	0.075625	-0.10626	0.01108	0.06055	-0.05044
SMC3	-0.19104	-0.76589	-0.17244	0.095769	0.323956	0.259752	-0.2986	-0.36143
SMC4	-0.04632	-0.63478	-0.33187	0.343826	0.068897	0.019936	-0.10092	-0.07544
SMC5	0.455066	0.600852	-0.38682	-0.0985	-0.02226	-0.05293	NA	NA
SMC6	0.236461	-0.29863	-0.05445	0.107829	0.073286	0.369157	NA	NA
SMCHD1	NA	NA	-0.18277	-0.19587	-0.24083	0.056276	NA	NA
SMCP	0.233273	0.289773	0.222479	0.017903	-0.03969	-0.02634	0.085892	-0.04543
SMCR5	NA	NA	0.285983	-0.14118	-0.02738	-0.03461	NA	NA
SMCR7	-0.0999	0.382103	0.269144	-0.24255	0.053028	-0.04482	-0.28764	0.0816
SMCR7L	0.23269	0.612073	-0.08541	0.157292	0.331615	0.247096	NA	NA
SMCR8	0.347964	0.295392	-0.1637	0.080813	-0.2323	-0.36652	0.454072	0.193549
SMEK1	-0.05466	0.662597	-0.37033	0.143687	-0.07748	0.40437	NA	NA
SMEK2	0.332459	-0.46062	-0.30661	-0.35287	-0.0998	-0.14944	0.132928	-0.19987
SMEK3P	NA	NA	0.213531	0.149631	0.088619	-0.04291	0.593879	0.331185
SMG1	-0.11189	-0.73839	-0.07045	-0.14624	0.303012	0.276638	0.66795	0.517683
SMG5	0.297739	0.548773	0.278332	-0.05139	0.327323	0.127458	-0.28999	-0.26232
SMG6	0.078371	0.260754	0.095767	-0.21783	-0.08389	0.314187	-0.19534	0.245363
SMG7	0.199563	-0.06742	0.281733	0.125306	0.155311	0.194379	0.582024	0.307552
SMN1	0.100044	-0.33146	0.055549	0.261338	0.142734	0.274377	-0.02225	0.382647
SMN2	NA	NA	0.170522	0.176298	-0.15931	0.097809	-0.25389	-0.234
SMNDC1	-0.16348	-0.50002	-0.20264	-0.26463	0.26318	-0.07597	-0.02162	0.019874
SMO	0.033127	-0.49167	-0.0006	0.001811	0.003375	0.188334	0.006736	-0.03994
SMOC1	-0.00609	-0.02235	-0.16376	-0.09424	0.176902	0.004368	0.211249	0.398125
SMOC2	0.02537	-0.28819	0.001397	0.003849	0.185377	0.067831	0.060221	-0.26631
SMOX	0.259361	0.266453	-0.19161	0.136598	0.068328	0.148552	0.624292	0.43872
SMPD1	0.046813	0.611388	0.213033	-0.13064	0.207541	0.103355	-0.31507	0.046336
SMPD2	0.40931	0.480917	0.348726	0.129079	0.482782	0.016127	0.465917	0.196097
SMPD3	0.134413	0.243352	0.015454	0.201795	0.210772	-0.19425	-0.0662	0.039637
SMPD4	NA	NA	0.106512	0.349127	0.44658	0.37704	NA	NA
SMPDL3A	-0.1909	-0.2378	-0.00753	-0.43913	-0.25165	-0.50046	-0.2015	-0.20146
SMPDL3B	0.21678	0.142983	0.10293	0.217554	0.062495	0.025504	0.331963	-0.12194
SMPX	0.04291	-0.25987	0.305423	-0.18755	0.103928	-0.20445	-0.19712	-0.36986
SMR3A	0.36244	0.186233	0.297635	0.122949	-0.10324	-0.11536	0.570923	0.292508
SMR3B	0.147762	-0.05079	0.223779	0.121215	-0.12008	0.135815	-0.20747	-0.29557
SMS	NA	NA	-0.16194	0.303861	0.092123	-0.11725	0.384108	0.009771

SMTN	0.11698	-0.16068	0.166105	-0.01384	0.341513	0.261082	-0.11402	-0.27671
SMTNL2	0.320677	-0.17006	0.100075	-0.11422	-0.21547	-0.12481	NA	NA
SMU1	0.330235	-0.51222	-0.1838	-0.173	-0.01945	-0.27491	-0.26933	-0.24876
SMUG1	0.272091	0.427817	0.168403	-0.03442	0.089618	-0.53078	-0.16219	-0.25435
SMURF1	0.126688	0.134006	-0.07563	0.041493	0.110374	0.084839	0.36138	0.266955
SMURF2	0.146309	0.785499	0.287453	-0.28123	0.208778	0.06887	0.085387	0.362736
SMYD1	0.063301	-0.05255	-0.32666	-0.09535	0.250171	0.170688	-0.25499	-0.2126
SMYD2	-0.06741	-0.42097	-0.12545	0.052824	-0.22219	-0.03493	0.618086	0.217587
SMYD3	0.070826	-0.38595	0.195223	0.050446	0.056338	-0.27867	0.34831	-0.39074
SMYD4	NA	NA	0.045372	-0.05759	-0.05237	0.17663	0.041092	0.308467
SMYD5	0.150725	0.546249	0.155163	0.194181	0.286478	0.054526	0.281078	-0.1906
SNAI1	-0.01719	0.031715	0.033855	0.061854	0.049846	0.193522	-0.18507	0.214783
SNAI2	-0.37422	-0.30553	0.001839	0.071852	-0.18734	0.000814	0.117925	0.174516
SNAI3	-0.48818	0.460188	0.291644	0.27348	-0.19208	0.241778	0.224825	0.372241
SNAP23	-0.19479	-0.33381	0.036828	0.123505	-0.1479	-0.11009	-0.34555	-0.23833
SNAP25	0.1221	-0.0387	-0.1527	0.062242	-0.15824	-0.15455	-0.17554	-0.30945
SNAP29	-0.2839	-0.07942	-0.14568	0.094474	-0.17648	-0.53369	0.054011	0.136125
SNAP47	NA	NA	-0.0145	0.155911	-0.2106	0.14565	NA	NA
SNAP91	0.16178	0.202384	0.148009	0.39457	-0.02555	0.233223	-0.2807	-0.15196
SNAPC1	-0.10101	-0.35191	-0.22663	-0.14601	-0.22008	-0.28732	-0.67174	-0.62815
SNAPC2	0.142479	0.43234	0.18158	-0.01199	0.134491	0.092033	-0.23654	-0.31537
SNAPC3	-0.15852	0.134188	-0.2317	-0.15008	-0.00599	-0.36579	0.040554	0.016747
SNAPC4	0.041262	0.506606	-0.06377	0.035747	0.28097	0.340908	-0.14573	0.068767
SNAPC5	0.34614	-0.3551	-0.23019	-0.00587	-0.08023	-0.08298	-0.25837	-0.37149
SNAPIN	NA	NA	-0.11843	-0.16467	-0.51318	-0.48736	NA	NA
SNCA	-0.28667	-0.31547	0.083493	-0.10778	-0.08305	-0.07668	-0.21741	-0.2354
SNCAIP	0.099089	0.041524	-0.27536	0.199385	-0.10472	-0.31584	-0.14168	-0.2819
SNCB	-0.10019	0.054271	0.183531	-0.28167	-0.1442	-0.22805	-0.00949	-0.33903
SNCG	-0.21807	0.025666	0.302591	0.014437	-0.20996	-0.27047	-0.57714	0.168381
SND1	0.347144	0.377952	0.385924	0.425515	0.371874	0.220412	0.63694	0.333987
SNED1	NA	NA	0.302347	0.225387	-0.00655	0.371227	NA	NA
SNF8	0.212599	0.766894	0.410552	0.293709	-0.02742	-0.18622	-0.50819	-0.54729
SNHG1	NA	NA	-0.12954	0.080185	0.048263	0.249764	NA	NA
SNHG10	NA	NA	-0.4786	-0.00775	-0.16958	-0.26259	0.530535	0.434166
SNHG11	NA	NA	0.248735	-0.12982	0.191485	-0.26609	NA	NA
SNHG12	NA	NA	-0.12915	0.313856	0.116286	0.205415	NA	NA
SNHG3-RCC	-0.29321	-0.31085	-0.03226	0.498263	0.242973	-0.04618	-0.36349	-0.49762
SNHG5	NA	NA	0.065909	-0.08199	0.172328	-0.13355	0.001867	-0.167
SNHG7	NA	NA	0.078849	-0.11231	0.317151	0.326412	NA	NA
SNHG8	NA	NA	-0.05258	-0.03168	-0.0271	-0.10211	-0.46852	-0.28659
SNIP	0.193123	0.59064	0.267932	-0.26557	-0.16461	0.1281	-0.54415	-0.4682
SNIP1	-0.44229	-0.32517	-0.29353	-0.15274	0.143871	-0.09125	-0.23182	-0.07538
SNN	0.056392	0.261856	0.002986	0.406978	-0.05987	0.552829	-0.54618	-0.38971
SNPH	0.442497	0.751909	0.2883	0.192588	-0.0229	-0.04805	-0.28937	0.170369
SNRK	-0.39806	-0.53553	-0.01975	-0.27935	0.05905	0.297058	-0.6945	-0.08004
SNRNP200	NA	NA	0.090427	0.365168	0.296649	0.555991	NA	NA
SNRNP25	NA	NA	-0.24308	0.349305	-0.14484	-0.10539	NA	NA
SNRNP27	NA	NA	-0.17036	-0.1313	-0.24754	-0.44588	NA	NA
SNRNP35	NA	NA	0.207106	0.197493	0.175736	0.14354	NA	NA
SNRNP40	NA	NA	-0.05426	0.284457	0.158112	-0.1355	NA	NA
SNRNP48	NA	NA	-0.02343	-0.20919	-0.03298	-0.06009	NA	NA
SNRNP70	NA	NA	0.186923	0.429189	0.307417	0.387066	NA	NA

SNRPA	0.290985	0.270886	0.151469	0.266485	0.08983	0.124279	0.370966	0.071537
SNRPA1	-0.12505	-0.53241	-0.31032	0.307131	-0.19078	0.032077	0.416695	0.527029
SNRPB	0.451553	0.52206	-0.19791	0.344306	-0.01792	-0.11651	0.467804	0.041294
SNRPB2	0.28093	-0.62972	-0.41243	-0.07204	-0.09125	-0.1989	0.524971	0.107192
SNRPC	0.448088	-0.51991	-0.15112	0.429434	-0.09587	-0.05752	0.717248	0.018247
SNRPD1	-0.18767	-0.47053	-0.16644	0.140901	-0.1853	-0.42918	0.061517	0.053369
SNRPD2	0.096326	-0.54549	-0.44409	0.122075	-0.11065	-0.26869	0.234618	-0.04963
SNRPD3	-0.26373	-0.31051	-0.06706	-0.00471	0.002907	-0.22188	0.269783	-0.24195
SNRPE	0.007208	-0.02092	-0.11669	-0.17281	-0.28971	-0.12268	0.092084	0.111158
SNRPF	-0.01052	-0.59289	-0.17886	0.053432	0.037904	-0.21809	0.310373	0.204017
SNRPG	0.364045	0.493137	-0.25092	-0.01203	-0.29742	-0.44719	0.195442	0.132356
SNRPN	-0.01252	-0.5579	0.189799	-0.11021	-0.10705	0.335276	-0.50298	-0.36985
SNTA1	-0.18624	0.148605	-0.03651	-0.0697	0.343707	-0.11187	-0.27408	-0.38201
SNTB1	0.086313	-0.40664	0.440658	-0.38583	0.077577	0.205011	0.57442	0.137252
SNTB2	0.08099	-0.51329	0.075585	-0.23792	0.276662	0.061888	-0.03151	-0.12625
SNTG1	0.157206	0.659292	0.150796	-0.18532	-0.03175	0.101707	0.412058	0.515753
SNTG2	0.170468	0.072635	0.029145	0.195388	-0.21171	-0.14549	0.681224	0.530494
SNUPN	0.257353	-0.30975	-0.11191	0.203483	-0.32582	-0.30206	0.082649	-0.18131
SNURF	NA	NA	0.139359	-0.08357	-0.20573	0.085978	-0.38058	-0.13763
SNW1	0.27322	0.39936	-0.2964	-0.2143	-0.18532	0.16651	-0.3117	-0.5488
SNX1	0.040604	-0.4371	0.183997	0.322194	-0.10934	0.082169	-0.23538	-0.00545
SNX10	-0.20898	-0.20265	0.261365	0.318958	-0.24608	0.329547	-0.2826	-0.06609
SNX11	-0.01813	0.593658	0.115904	-0.1566	-0.15573	-0.15405	-0.35017	0.216082
SNX12	-0.09232	0.424673	0.034391	0.242212	0.075981	-0.15851	-0.19709	-0.10618
SNX13	0.002327	-0.34172	-0.31467	-0.40891	-0.20369	-0.18342	-0.00102	0.024628
SNX14	-0.03262	-0.50629	-0.23487	-0.37754	0.250853	-0.01659	-0.25365	-0.42867
SNX15	0.174337	-0.02308	0.206795	-0.0202	0.383816	-0.13486	0.385391	-0.07594
SNX16	0.235031	-0.03533	-0.28643	-0.21801	-0.23902	-0.49942	-0.10089	-0.52865
SNX17	0.12804	0.234301	0.339485	0.149	0.257458	-0.11815	0.635009	0.41436
SNX18	NA	NA	-0.12521	0.169633	-0.17133	0.169139	NA	NA
SNX19	-0.03493	-0.55787	-0.09201	-0.08453	-0.20578	-0.16569	0.411249	0.40376
SNX2	0.330404	-0.38943	0.259425	-0.05998	-0.17671	-0.07558	-0.12364	0.119524
SNX20	NA	NA	0.034188	0.53762	0.121934	0.477161	NA	NA
SNX21	0.254631	0.086534	0.500299	-0.19343	0.408621	-0.139	0.305782	0.133151
SNX22	-0.24851	-0.03466	-0.16088	0.282419	0.018794	0.201788	-0.46263	-0.27412
SNX24	-0.34306	-0.38748	0.224298	-0.24632	-0.31507	-0.22452	-0.34781	0.073417
SNX25	0.062073	-0.52823	-0.0682	0.036239	0.044407	0.05547	0.478539	0.207222
SNX26	-0.43727	0.378181	-0.01766	0.030169	0.042208	0.328053	-0.09887	-0.01168
SNX27	-0.04125	-0.18443	0.202814	-0.16116	-0.04604	0.48127	0.05712	-0.08748
SNX29	NA	NA	-0.33692	0.183941	-0.13116	-0.0095	NA	NA
SNX3	0.341543	-0.39767	-0.14742	-0.10643	-0.20427	-0.12609	-0.13823	-0.19448
SNX31	NA	NA	0.29506	-0.13605	0.129524	0.164836	NA	NA
SNX32	NA	NA	-0.34988	0.013419	-0.44177	-0.12	NA	NA
SNX33	NA	NA	0.342312	0.065208	0.271274	-0.05918	NA	NA
SNX4	-0.06928	-0.54493	-0.0791	-0.48212	0.075698	-0.09061	0.271382	0.184443
SNX5	0.024589	0.150929	-0.37313	-0.02015	-0.06251	-0.30192	0.445383	0.190775
SNX6	0.00472	0.510833	-0.21355	-0.4132	-0.21473	-0.30918	-0.29258	-0.05255
SNX7	0.217213	-0.30482	0.4475	-0.38555	0.272463	-0.28629	-0.10317	-0.39083
SNX8	0.082084	0.481639	0.34966	0.033678	0.251104	-0.05116	0.167772	0.284387
SNX9	0.267741	0.326562	-0.00328	-0.20002	0.330471	0.104544	0.102178	-0.06311
SOAT1	-0.09621	-0.48677	-0.46782	-0.09105	-0.41516	-0.55125	-0.40091	-0.20914
SOAT2	0.362578	0.163298	0.129307	0.160766	-0.02789	0.064952	0.247457	0.449472

SOBP	NA	NA	0.07698	-0.12975	-0.1164	0.039478	NA	NA
SOCS1	0.194312	-0.10045	0.413792	0.332	0.116737	0.429228	0.105964	0.378381
SOCS2	0.057674	-0.49327	-0.27344	-0.30071	-0.05406	-0.46035	-0.44575	-0.15402
SOCS3	-0.53625	-0.16147	0.13145	0.163374	0.078752	0.250631	-0.35938	-0.17957
SOCS4	0.196742	-0.41296	-0.40149	-0.19714	-0.16332	-0.05619	0.317388	0.349288
SOCS5	0.269187	-0.30478	-0.2641	-0.45944	-0.0972	-0.3584	-0.04822	-0.18623
SOCS6	0.004288	0.199868	-0.26639	-0.39585	-0.21436	-0.60811	0.047884	-0.04699
SOCS7	-0.28624	-0.04759	0.153451	0.102558	0.075411	-0.37495	-0.01553	-0.35529
SOD1	-0.39605	-0.51518	0.092225	-0.11095	-0.03949	-0.1014	-0.40256	-0.3007
SOD2	-0.35274	-0.12732	0.019535	0.022933	0.084032	0.4094	-0.30868	0.38498
SOD3	0.172911	0.289686	0.015103	0.270539	-0.02399	0.347691	-0.12645	-0.0814
SOHLH1	-0.01372	0.482599	-0.30493	-0.06887	-0.15985	-0.12714	NA	NA
SOHLH2	0.234183	-0.07161	-0.11285	-0.18343	0.017789	-0.08173	-0.26076	0.044698
SOLH	0.282117	0.381686	-0.36291	0.237899	0.115367	0.125772	0.483346	0.50191
SON	-0.22241	-0.48844	-0.22845	0.168122	0.002177	0.364642	0.195897	0.359595
SORBS1	-0.07556	-0.33187	0.174864	-0.16446	0.16491	0.271943	-0.14848	-0.17436
SORBS2	-0.17051	-0.26206	-0.27393	-0.06067	-0.15007	0.178409	0.156227	-0.44037
SORBS3	0.176282	-0.09838	0.119412	-0.17975	-0.02906	-0.04222	-0.05691	-0.31669
SORCS1	-0.02341	0.082861	-0.09438	0.10391	-0.12556	0.53121	-0.309	-0.23904
SORCS2	0.1012	-0.05521	0.098925	0.00743	0.084182	0.087739	-0.13692	-0.01715
SORCS3	-0.00896	-0.0794	-0.21516	0.324537	-0.33213	-0.03224	-0.52644	-0.41172
SORD	0.058897	-0.04983	-0.17314	0.418327	0.049044	0.299596	0.323259	0.153697
SORL1	-0.34817	-0.24966	0.2317	-0.04958	0.071865	0.26577	0.313935	-0.12524
SORT1	-0.28459	-0.18566	0.367507	-0.24656	0.19044	-0.46287	0.533798	0.327647
SOS1	0.095142	-0.27827	0.000337	-0.05222	-0.01913	-0.32226	0.330669	0.519093
SOS2	-0.12776	-0.16884	-0.34771	-0.30077	-0.1692	-0.09701	0.191957	0.343141
SOST	-0.05663	0.616113	0.168887	0.460357	-0.00533	0.299826	-0.2394	-0.32668
SOSTDC1	0.149928	-0.16255	-0.108	-0.00393	-0.38092	-0.3022	0.249011	0.004947
SOX1	-0.28375	-0.16919	-0.05342	-0.04147	-0.08762	0.261755	0.408548	0.246274
SOX10	0.15786	0.022923	0.360059	-0.05427	-0.25707	0.111031	-0.4583	-0.32445
SOX11	-0.1469	0.088034	-0.17936	-0.14935	-0.00325	0.060949	-0.30439	-0.2836
SOX12	-0.01316	-0.11883	0.048267	0.269679	0.18202	-0.10968	0.243677	0.079064
SOX13	-0.19094	0.480465	0.085039	0.077636	0.107636	0.071927	0.343187	-0.08375
SOX14	0.098131	0.365397	0.196235	-0.05266	-0.26027	0.188154	-0.50389	-0.33039
SOX15	-0.12589	0.023131	-0.25232	-0.085	0.060219	-0.34943	-0.3469	-0.40215
SOX17	-0.24284	-0.26266	-0.28903	0.114946	-0.17237	0.252243	-0.16969	0.105648
SOX18	-0.3788	-0.20156	0.160612	-0.0753	0.072405	0.156163	-0.36904	-0.26068
SOX2	0.303081	0.712991	0.038621	-0.10373	-0.15865	0.141455	0.039367	-0.176
SOX21	-0.14221	0.137719	0.134732	-0.24193	0.081155	-0.16757	0.131051	0.130753
SOX3	-0.20281	0.685654	0.057081	-0.01797	0.008529	0.019486	-0.21065	0.391613
SOX30	0.503187	0.732493	-0.12691	-0.07377	-0.07877	0.095713	-0.16188	0.071948
SOX4	0.195307	0.092677	-0.13211	0.296518	0.292767	0.203413	-0.06949	-0.6155
SOX5	0.189268	0.720811	0.314212	-0.09763	0.180605	0.250364	0.094684	0.644227
SOX6	0.430748	0.607205	-0.34815	-0.26255	-0.27442	0.026818	0.152102	0.337093
SOX7	-0.16847	-0.24529	0.094366	-0.03249	0.15234	0.439566	-0.24412	0.092457
SOX8	0.165074	0.338336	0.137583	0.325326	-0.30374	0.499922	0.176979	-0.11491
SOX9	0.223448	0.15659	0.137474	0.170623	0.189801	0.101602	0.628454	0.159827
SP1	-0.1675	0.230613	-0.06486	-0.22903	0.251227	-0.12039	-0.12248	-0.2391
SP100	0.390468	-0.34188	0.1366	0.044459	-0.24228	0.147934	0.013355	0.290623
SP110	-0.04782	0.069626	0.244631	0.049511	-0.35853	-0.05209	-0.17487	0.625713
SP140	-0.50799	0.383487	0.200354	0.726446	-0.45774	0.618397	-0.47546	0.146692
SP140L	NA	NA	0.198281	0.3709	-0.24541	0.129264	NA	NA

SP2	0.282824	0.177916	0.025222	0.068756	-0.05085	0.052931	0.558264	0.510717
SP3	0.014949	0.335822	-0.11753	-0.38981	0.073758	-0.11312	-0.13505	-0.28401
SP4	0.081883	-0.53536	-0.03073	-0.10361	-0.13269	0.515331	-0.11886	-0.41011
SP5	-0.09301	-0.04154	0.320089	0.095886	0.038385	0.005884	0.592728	-0.29865
SP6	0.024178	0.207718	NA	NA	NA	NA	0.262247	0.115347
SP7	0.238966	0.450278	-0.11615	-0.08427	-0.00313	-0.06131	-0.00068	-0.30082
SP8	-0.23865	0.132485	0.057509	-0.11459	-0.10279	0.064373	0.060552	0.542696
SPA17	-0.08823	0.222518	-0.12823	-0.40607	-0.11056	-0.66754	0.020111	-0.17412
SPACA1	-0.30502	-0.05554	0.035366	-0.12863	-0.12759	0.051662	0.503721	0.269902
SPACA3	0.51046	0.063044	0.23118	-0.30417	0.20644	-0.35189	-0.18888	-0.02918
SPACA4	0.194641	0.31714	-0.30153	-0.31157	-0.10889	-0.06792	-0.33772	-0.14444
SPACA5	NA	NA	-0.19594	-0.06073	0.179049	-0.30086	-0.09359	0.084773
SPAG1	-0.00529	-0.12907	-0.23385	0.071472	-0.13255	-0.30353	-0.14574	-0.37895
SPAG11B	NA	NA	0.414801	-0.20573	0.197764	-0.03518	NA	NA
SPAG16	0.477591	0.030959	0.01548	-0.07835	-0.02326	-0.18037	-0.36598	-0.49346
SPAG17	-0.15618	-0.09099	0.111864	-0.11016	0.058157	0.012427	0.300208	0.337764
SPAG4	0.137435	-0.20374	0.11412	0.200028	0.016601	-0.25731	0.129794	-0.19416
SPAG4L	0.034664	0.319745	0.178675	-0.06456	0.235119	0.122085	0.289125	0.166113
SPAG5	-0.04472	0.343223	-0.20553	0.190018	-0.10928	-0.17994	0.205442	0.17096
SPAG6	-0.05319	0.238128	0.174701	0.027052	-0.08913	0.184304	0.048797	0.109974
SPAG7	-0.12718	0.623767	-0.03219	-0.09399	-0.35427	-0.17552	0.061638	-0.07135
SPAG8	0.378343	-0.56198	-0.28674	0.232408	0.003574	0.061864	-0.26428	0.0362
SPAG9	-0.21024	-0.42707	0.10791	-0.32671	-0.03498	0.043279	-0.26516	-0.09485
SPAM1	-0.01717	-0.03611	0.114244	0.061018	-0.24018	-0.05316	0.577484	0.569586
SPANXA1	NA	NA	-0.07531	0.060995	0.058285	-0.418	0.546523	0.22532
SPANXB2	0.196711	0.485579	-0.13743	-0.26531	0.132844	-0.14948	NA	NA
SPANXC	-0.01207	-0.14408	NA	NA	NA	NA	-0.11684	-0.45119
SPANXD	0.130298	0.366393	-0.05091	-0.09508	0.070669	-0.36436	NA	NA
SPANXE	NA	NA	NA	NA	NA	NA	0.289068	0.166117
SPANXN1	-0.00762	0.426185	NA	NA	NA	NA	NA	NA
SPANXN2	0.124254	0.222308	NA	NA	NA	NA	NA	NA
SPANXN3	-0.24277	0.329612	0.364612	-0.18352	0.130578	0.199202	NA	NA
SPANXN4	-0.10439	-0.09589	NA	NA	NA	NA	NA	NA
SPANXN5	-0.13912	0.406072	NA	NA	NA	NA	NA	NA
SPARC	-0.20585	-0.37461	0.036493	0.150486	-0.11549	0.036122	-0.04573	-0.11052
SPARCL1	-0.42102	-0.48881	-0.03683	-0.03109	-0.0795	0.169598	-0.31172	-0.30565
SPAST	0.128495	-0.53332	-0.16825	-0.35088	-0.0147	-0.2416	-0.1159	-0.1328
SPATA1	0.119043	-0.61642	0.033332	-0.04376	-0.10107	-0.14479	-0.29118	-0.10135
SPATA12	-0.04253	0.172376	-0.00246	0.126529	-0.03012	-0.09225	0.150475	-0.08363
SPATA13	0.181657	-0.28646	0.364585	-0.1165	0.324116	0.373703	-0.07862	-0.23776
SPATA16	-0.02061	0.179876	0.245097	0.06156	0.218903	0.352363	0.13789	-0.48633
SPATA17	0.124755	-0.04298	-0.09342	-0.10167	-0.23276	-0.27679	NA	NA
SPATA18	-0.15371	-0.1103	0.199051	0.191594	-0.34919	-0.48976	-0.12832	0.099271
SPATA19	0.326673	0.101507	-0.07779	-0.1908	-0.06962	0.216164	-0.07408	-0.21137
SPATA2	0.50257	-0.22109	0.027433	-0.21756	0.430089	-0.25039	0.39829	0.278109
SPATA20	-0.06698	0.678768	0.284711	0.28504	-0.02468	0.096994	0.139359	0.016792
SPATA21	-0.14554	0.189296	NA	NA	NA	NA	0.001356	0.35675
SPATA22	0.107987	-0.0082	0.107065	-0.30851	-0.1146	0.179968	-0.0582	0.124347
SPATA2L	0.139239	0.543943	0.092412	-0.0003	-0.11933	0.132901	NA	NA
SPATA3	-0.09607	0.68162	-0.1111	-0.14568	-0.07013	-0.32572	0.151356	-0.00092
SPATA4	0.119613	-0.42729	-0.07632	-0.08996	0.146437	-0.1518	0.42628	0.260783
SPATA5	0.223694	0.16353	-0.26967	0.274977	-0.28414	-0.1415	-0.23486	-0.131

SPATA5L1	0.385296	-0.64912	-0.34923	-0.14685	0.006942	0.296162	0.521035	0.604431
SPATA6	0.044291	-0.30807	0.106939	-0.3437	0.128886	-0.25115	0.13569	-0.11403
SPATA7	0.446959	0.063638	-0.25332	-0.24666	-0.25464	-0.06901	0.028125	-0.44553
SPATA8	-0.06242	0.305448	-0.1025	0.015567	0.001348	-0.08763	0.207278	0.600282
SPATA9	0.153494	0.731024	0.076598	0.009294	-0.15193	-0.3747	0.313149	0.060392
SPATC1	-0.33804	0.610091	NA	NA	NA	NA	-0.16304	-0.0441
SPATS1	0.055658	0.143454	-0.16427	0.128153	-0.20942	-0.06646	-0.32708	-0.0728
SPATS2	-0.01074	-0.22803	-0.04609	0.150755	0.173109	-0.28706	-0.45148	-0.26734
SPC24	NA	NA	-0.09633	0.247838	0.207115	0.010664	0.022387	-0.38228
SPC25	0.188163	-0.07947	-0.08889	0.095043	0.184504	-0.08733	0.257771	0.037092
SPCS1	-0.11041	-0.52545	0.001022	-0.07508	-0.07073	-0.16012	0.431007	0.228899
SPCS2	-0.39725	-0.40332	-0.05642	0.141383	0.14464	-0.09671	-0.57144	-0.57236
SPCS3	-0.09464	-0.3736	-0.12265	0.126827	-0.28295	-0.34465	0.485202	0.141985
SPDEF	-0.17601	0.247529	-0.07698	0.116273	0.006612	0.041531	0.131679	0.170103
SPDYA	0.086212	0.524215	-0.13882	-0.01741	0.055831	-0.26443	NA	NA
SPDYC	-0.19054	0.5424	NA	NA	NA	NA	NA	NA
SPECC1	0.024658	0.054756	NA	NA	NA	NA	-0.20383	-0.08067
SPEF1	NA	NA	0.108463	-0.18943	-0.09344	-0.1008	NA	NA
SPEF2	NA	NA	-0.26674	-0.04885	-0.26491	-0.22807	NA	NA
SPEG	NA	NA	0.021157	0.04842	0.038738	0.085611	NA	NA
SPEM1	NA	NA	0.227337	0.097656	-0.22372	-0.16204	NA	NA
SPEN	-0.20081	0.072583	0.038071	-0.10332	0.355631	0.18021	0.084714	0.378015
SPERT	0.313689	0.777456	0.05519	-0.09537	0.314042	-0.34132	0.498971	0.296557
SPESP1	0.068145	-0.09834	-0.28719	-0.04189	-0.0926	-0.01953	-0.11454	-0.08303
SPG11	NA	NA	0.085419	0.017524	0.031251	0.069548	NA	NA
SPG20	-0.20269	0.006232	-0.05242	-0.07372	-0.50215	-0.02294	-0.5235	-0.3708
SPG21	0.189451	0.601774	-0.04932	0.206809	0.064618	0.174515	0.426052	0.325775
SPG7	-0.1438	-0.54833	0.199321	0.181782	0.513487	0.262862	0.341829	0.538196
SPHAR	0.135504	-0.35971	-0.25466	-0.44244	0.143088	-0.48831	-0.14002	-0.47223
SPHK1	-0.39338	-0.19893	-0.00912	0.141663	-0.05022	0.192772	-0.13098	0.155822
SPHK2	0.392781	0.36817	-0.14486	0.003693	0.022213	-0.07276	0.318275	0.129795
SPHKAP	NA	NA	-0.24971	0.383479	-0.15775	-0.12329	NA	NA
SPI1	-0.54804	0.209198	0.302958	0.413651	-0.11778	0.559381	-0.56714	-0.04172
SPIB	-0.02012	0.118527	0.13694	0.570418	-0.10275	0.601289	-0.25094	-0.06343
SPIC	0.20985	-0.26479	0.20467	0.261897	-0.28302	0.399772	-0.13828	-0.1816
SPIN1	0.22683	0.471563	-0.24274	-0.32066	-0.13092	-0.13855	-0.33755	-0.48218
SPIN2B	0.298849	0.244236	-0.12734	0.088808	0.187423	0.110217	0.509823	0.110921
SPIN3	0.526989	-0.14659	-0.07865	-0.1444	0.265181	0.071969	NA	NA
SPIN4	NA	NA	-0.40843	-0.00072	0.097545	0.033135	NA	NA
SPINK1	0.453407	0.107167	0.497957	0.073665	-0.01018	-0.17646	0.120261	-0.06464
SPINK2	-0.06223	0.012805	-0.25536	-0.11175	-0.25908	-0.28268	0.122895	0.152419
SPINK4	-0.35977	0.317481	-0.14552	0.027682	0.063805	-0.1669	0.115931	0.311994
SPINK5	0.058191	-0.07557	-0.1163	0.026918	-0.2764	-0.33995	-0.0333	-0.20563
SPINK6	0.143084	0.151535	0.082627	-0.10308	0.229953	-0.04696	-0.38404	-0.07595
SPINK7	0.011298	-0.14793	0.273468	-0.06576	-0.0155	-0.18757	0.105076	-0.18681
SPINK9	NA	NA	NA	NA	NA	NA	0.218748	0.221869
SPINLW1	0.256992	0.653575	-0.199	-0.18164	0.025644	0.164669	0.374065	0.439051
SPINT1	-0.01597	0.220687	0.274829	0.011219	0.019629	0.04498	0.426411	0.016137
SPINT2	-0.12225	0.013348	-0.15993	0.030424	0.131195	-0.12551	0.571405	0.175688
SPINT4	NA	NA	NA	NA	NA	NA	0.214679	-0.05135
SPIRE1	0.086041	0.173574	-0.14519	0.078584	0.04111	-0.04094	-0.4734	-0.25153
SPIRE2	0.470994	0.340804	0.134082	-0.08636	0.501392	0.040682	0.383413	0.16454

SPN	-0.10205	0.391988	0.345623	0.012173	-0.26482	0.496572	-0.6517	-0.41054
SPNS1	NA	NA	0.217626	-0.0052	0.347656	-0.08994	NA	NA
SPNS2	NA	NA	0.369694	0.066683	0.166026	-0.01454	NA	NA
SPNS3	NA	NA	0.157078	0.479391	-0.13418	0.103031	NA	NA
SPO11	-0.27308	0.49989	0.185408	-0.17149	0.181273	-0.0332	0.335977	0.257947
SPOCD1	-0.3493	-0.1325	-0.20258	0.111454	0.003426	-0.12613	-0.34006	-0.26208
SPOCK1	-0.12103	-0.204	0.05443	-0.2259	-0.13292	-0.03705	NA	NA
SPOCK2	0.071999	0.471761	0.343954	0.858824	0.065337	0.757983	-0.63122	0.003042
SPOCK3	NA	NA	0.076383	-0.21636	0.315285	-0.00477	-0.27372	-0.3853
SPON1	-0.05089	-0.43692	0.011712	-0.09904	-0.15939	-0.25337	-0.24438	-0.04913
SPON2	-0.18688	-0.15018	0.013717	0.0718	-0.00303	0.009398	-0.10319	-0.02837
SPOP	0.145923	-0.12355	-0.0636	0.147062	-0.14349	0.052861	-0.2267	-0.16335
SPOPL	NA	NA	0.17695	-0.32521	0.022249	-0.07381	NA	NA
SPP1	-0.41919	-0.44145	-0.02446	0.09598	-0.20899	-0.07698	-0.05023	-0.08211
SPP2	-0.21522	0.474112	-0.15625	-0.0561	0.101057	0.207065	0.125346	0.235804
SPPL2A	-0.39185	-0.25615	0.119165	-0.30942	-0.06174	-0.20036	0.002338	0.297906
SPPL2B	-0.04508	0.32024	-0.00997	0.025376	0.026458	0.280638	0.545191	0.171106
SPPL3	-0.06732	-0.42422	NA	NA	NA	NA	-0.26719	-0.41742
SPR	0.180527	0.283377	0.104815	0.070766	0.029029	-0.24063	0.213228	-0.36227
SPRED1	0.112714	0.449242	-0.27668	-0.25612	-0.41181	0.098768	-0.20064	0.517566
SPRED2	0.10049	0.491003	0.106126	-0.04115	0.16089	0.027722	0.200864	-0.0516
SPRED3	NA	NA	NA	NA	NA	NA	0.107516	0.44915
SPRN	0.024332	0.065757	0.047982	-0.24184	-0.00644	-0.09769	-0.38544	-0.42616
SPRR1A	-0.05594	0.652077	-0.19436	-0.22462	-0.30218	0.009761	-0.19063	0.167985
SPRR1B	-0.07743	0.3333	-0.17431	-0.33914	-0.35164	0.008969	0.355943	0.327578
SPRR2A	-0.0307	0.402537	NA	NA	NA	NA	0.270152	0.180865
SPRR2B	-0.00484	0.233928	NA	NA	NA	NA	0.34757	0.317807
SPRR2C	NA	NA	-0.04275	-0.30277	-0.33481	-0.24638	0.355571	-0.29022
SPRR2D	-0.33977	0.044099	-0.08599	-0.37703	-0.31163	0.034266	0.205784	-0.1863
SPRR2E	-0.02851	0.465523	-0.0008	-0.09587	-0.08363	-0.02152	0.083158	0.201392
SPRR2F	-0.04762	0.462274	NA	NA	NA	NA	0.114843	-0.34916
SPRR2G	-0.06461	0.072244	0.00617	0.097611	-0.17416	0.288777	-0.54227	-0.59586
SPRR3	-0.08758	0.045977	-0.15364	-0.4611	-0.28311	-0.28916	0.045608	-0.08138
SPRR4	0.17865	0.107614	0.000553	-0.02863	0.16787	0.035438	-0.20303	-0.21823
SPRY1	-0.24933	-0.39924	0.189823	-0.37221	-0.09433	-0.139	-0.04859	0.062298
SPRY2	-0.23824	-0.40221	-0.00064	-0.17081	-0.18886	-0.11717	0.000293	-0.51777
SPRY3	-0.03919	-0.14964	-0.40847	-0.43307	0.265614	-0.22726	-0.02085	0.066899
SPRY4	0.252042	0.700007	0.390934	-0.1732	0.075735	0.223555	0.273504	0.294738
SPRYD3	-0.1665	0.254618	0.385764	0.203225	0.320229	0.148075	-0.54555	-0.40884
SPRYD4	0.222517	0.705091	-0.28719	-0.07776	-0.20635	-0.38867	NA	NA
SPRYD5	0.198963	0.282738	0.154613	-0.16986	0.284116	-0.29842	NA	NA
SPSB1	-0.07574	0.219105	0.062868	-0.15156	0.091358	-0.17581	-0.05622	0.115957
SPSB2	0.244116	0.49625	0.046862	-0.38381	0.043239	-0.17458	-0.08532	-0.03211
SPSB3	-0.04435	0.414528	0.225931	0.060976	0.306917	0.186438	-0.08956	-0.02531
SPSB4	0.337601	-0.39122	-0.05354	-0.05416	-0.32077	0.161508	0.01369	-0.34426
SPTA1	-0.29231	0.189432	-0.04757	0.019854	0.009319	0.074213	-0.35448	-0.34937
SPTAN1	0.247585	-0.12159	-0.1129	0.179454	0.146823	0.204668	0.331326	0.120273
SPTB	-0.09146	0.182102	-0.21334	-0.17953	-0.14793	-0.08286	-0.53333	-0.07122
SPTBN1	0.109312	-0.17919	-0.16447	0.108179	0.038211	0.114777	-0.0665	-0.10311
SPTBN2	0.167109	0.570535	0.30927	-0.02902	0.146123	-0.0784	0.377207	0.289458
SPTBN4	0.253075	0.035133	0.03064	-0.06104	0.140895	-0.0749	-0.11211	-0.06218
SPTBN5	0.16085	-0.31264	-0.04432	0.020874	0.061456	0.157127	0.228268	-0.06316

SPTLC1	-0.36469	-0.41682	-0.13801	-0.31489	-0.06293	-0.53111	-0.53035	-0.36363
SPTLC2	-0.33726	-0.30719	-0.20628	0.073149	-0.40316	-0.17678	0.321844	0.19347
SPTLC3	NA	NA	-0.08856	-0.30118	-0.55384	-0.3913	0.058075	0.582081
SPTY2D1	-0.30977	-0.29358	-0.48714	-0.02909	-0.09021	-0.2521	-0.02816	0.179664
SPZ1	-0.14109	-0.00072	-0.22696	-0.23563	-0.28041	0.329636	0.091682	0.172842
SQLE	-0.0381	-0.14871	-0.15684	0.054814	-0.1557	0.008906	0.425156	0.258
SQRDL	-0.15576	-0.01345	-0.24391	-0.07294	-0.2308	0.01865	-0.40186	-0.43451
SQSTM1	0.12415	0.240451	0.413832	-0.09588	0.371511	0.185883	-0.27898	-0.54415
SR140	NA	NA	-0.18908	-0.1812	0.090812	0.372554	0.671596	0.439134
SRA1	NA	NA	0.210935	0.152695	-0.22249	-0.46877	-0.25863	-0.06951
SRBD1	0.220825	-0.00143	-0.14852	-0.16981	-0.06373	-0.28682	NA	NA
SRC	0.252879	0.334365	0.332279	0.083043	0.36962	0.142517	0.254833	0.173362
SRCAP	-0.28959	0.157137	0.172718	0.176753	0.352938	0.155304	-0.24199	-0.1909
SRCRB4D	0.270789	0.392736	-0.07507	-0.1935	-0.11027	-0.3505	-0.11696	-0.05683
SRD5A1	0.034092	-0.27098	-0.11644	-0.06355	0.033638	-0.32452	0.597534	0.450993
SRD5A2	-0.28694	0.366397	-0.13333	-0.07345	0.102829	-0.12952	-0.35995	-0.3249
SRD5A2L2	0.39849	0.217033	0.059695	-0.13905	-0.10745	0.050523	0.538612	-0.02085
SRD5A3	NA	NA	-0.00636	0.255591	-0.44527	-0.40245	NA	NA
SREBF1	-0.08705	0.226282	0.049552	0.080485	0.056514	0.298569	-0.01134	0.248345
SREBF2	-0.06004	0.398738	0.310818	0.133568	0.174684	0.158615	-0.30875	-0.24197
SRF	-0.17521	-0.1749	-0.12962	0.02507	0.27225	0.123032	-0.4002	-0.36778
SRFBP1	0.594545	0.447102	0.126153	-0.18236	0.178599	0.275831	0.281268	0.25483
SRGAP1	-0.20284	-0.04227	0.022438	-0.24399	-0.03197	0.224823	-0.17743	-0.22572
SRGAP2	-0.07063	0.375168	0.009322	0.110785	0.024902	0.264374	-0.17948	-0.38012
SRGAP3	0.093071	0.329388	0.200177	-0.07886	0.19272	0.106118	0.024303	-0.10964
SRGN	-0.50516	-0.33905	0.06309	0.128725	-0.03612	0.177946	-0.57712	-0.09172
SRI	-0.13076	-0.13146	-0.02336	-0.27913	-0.00428	-0.43959	0.037802	-0.35864
SRM	0.039757	0.357812	0.275456	0.327486	0.328203	0.310133	0.566246	0.176438
SRMS	-0.23665	0.284815	0.170558	-0.18229	0.096095	-0.0461	-0.31142	-0.22697
SRP14	NA	NA	-0.14332	-0.18611	-0.2521	0.023277	-0.05924	-0.09321
SRP19	-0.03416	-0.11176	0.02318	0.061106	-0.10582	-0.32022	-0.5545	-0.27276
SRP54	0.316185	0.121287	-0.33192	0.109131	-0.24794	-0.01698	0.306679	0.607605
SRP68	-0.17664	-0.28002	0.238041	0.219513	0.099867	0.180186	0.466579	0.233247
SRP72	-0.02829	0.196205	0.088516	0.112126	-0.31332	0.206006	-0.47217	-0.49219
SRP9	-0.37764	-0.60482	-0.4098	-0.02299	-0.21693	-0.38741	-0.14196	-0.46721
SRPK1	0.615904	0.503969	-0.25197	-0.04151	0.184062	0.156134	0.199021	-0.20214
SRPK2	-0.13239	-0.55564	-0.07805	-0.11181	0.126618	-0.06818	-0.50036	-0.48073
SRPK3	0.229316	0.012319	-0.29687	-0.44443	-0.19104	-0.5259	NA	NA
SRPR	-0.28704	0.098974	-0.18785	0.395465	0.016564	-0.12682	0.669267	0.436876
SRPRB	0.119545	-0.29045	-0.20319	0.325484	0.021823	0.062396	0.270216	0.167483
SRPX	-0.14077	-0.39768	-0.13829	-0.18519	-0.20507	-0.17574	-0.10308	-0.08369
SRPX2	0.253898	-0.2054	0.282505	0.160244	0.161937	-0.37043	-0.16927	0.112713
SRR	-0.32348	-0.26457	-0.16237	0.110533	-0.11999	-0.24647	-0.08001	0.077159
SRRD	NA	NA	0.175159	-0.08963	-0.22166	-0.04683	NA	NA
SRRM1	-0.02404	0.231109	0.020736	0.14138	0.238082	0.413835	0.08192	0.064792
SRRM2	-0.05257	-0.27381	-0.07673	0.359571	0.17058	0.203457	0.511855	0.47114
SRXN1	0.142208	-0.609	0.001563	-0.09932	0.075899	0.063601	0.461418	0.350081
SRY	0.279791	0.401237	0.130859	-0.10899	-0.17943	0.117552	0.329933	-0.12966
SS18	-0.21437	-0.44583	0.237803	0.016293	0.000143	0.227808	0.261725	0.335538
SS18L1	0.178051	0.245207	-0.35773	-0.05483	0.191999	0.083677	-0.08531	-0.19309
SS18L2	0.401861	0.463979	-0.18609	-0.00724	-0.01658	-0.20937	0.200088	0.190995
SSB	0.273793	-0.56233	-0.15812	0.575496	0.158536	0.409379	-0.43097	-0.23513

SSBP1	0.344397	-0.64428	-0.2288	-0.0471	-0.24473	-0.26933	-0.32836	-0.33211
SSBP2	0.078533	0.102398	-0.22236	-0.0032	-0.23598	-0.36802	-0.2784	-0.39938
SSBP3	-0.09393	0.377458	-0.00294	0.203913	0.301411	-0.07129	0.429325	0.184455
SSBP4	0.276517	-0.23917	-0.02375	0.089136	0.055844	-0.10929	0.138928	0.188083
SSFA2	-0.3282	-0.19077	-0.0834	-0.27149	-0.2442	-0.09442	0.116407	-0.04439
SSH1	-0.49314	-0.51603	0.144526	-0.41648	-0.0361	-0.02323	-0.39549	-0.24717
SSH2	0.09078	0.076884	NA	NA	NA	NA	-0.00127	0.176698
SSH3	-0.01274	0.015746	-0.00759	0.179106	0.440862	0.056515	-0.07536	-0.36735
SSNA1	0.197302	0.576488	0.175486	0.090619	0.112828	-0.10511	0.13223	0.055604
SSPN	0.008693	-0.07644	-0.02077	-0.33413	-0.19867	-0.0447	-0.37318	-0.51221
SSPO	NA	NA	0.148255	0.034646	0.124617	0.23102	NA	NA
SSR1	-0.26174	-0.4601	-0.06671	0.121143	0.109658	-0.16322	0.090358	0.052937
SSR2	-0.35569	-0.62183	-0.18672	0.536188	-0.09836	-0.29912	0.343445	-0.04122
SSR3	-0.32341	-0.25103	-0.01068	0.158258	-0.25342	-0.6005	-0.20717	-0.29395
SSR4	0.455612	0.271732	0.228001	0.127233	0.050016	-0.2509	0.022126	-0.18873
SSRP1	0.035505	-0.48756	-0.13554	0.547544	0.148746	0.37508	0.401917	-0.11099
SSSCA1	0.592794	0.53349	-0.16614	0.008541	-0.09272	-0.20109	0.057197	-0.16708
SST	0.120768	0.056381	0.085315	0.076809	-0.21332	0.035095	0.087946	0.231995
SSTR1	-0.18073	-0.04034	-0.25887	-0.09201	-0.14568	-0.2627	0.318825	0.058272
SSTR2	0.044111	0.622143	0.043388	-0.0679	-0.39869	-0.01781	-0.60165	-0.61818
SSTR3	-0.11239	0.641329	0.229365	0.018515	-0.24164	-0.2269	0.44828	0.317083
SSTR4	0.11152	0.399263	0.058239	-0.11111	0.199509	0.250818	-0.00554	0.331937
SSTR5	0.217733	0.534824	0.292613	-0.16666	-0.04254	0.106379	0.310878	-0.18157
SSU72	-0.15603	0.557882	0.176567	0.100326	0.090957	0.119058	-0.03222	0.286445
SSX1	-0.19527	-0.04336	-0.1939	-0.02797	0.002553	-0.26544	-0.26225	0.132793
SSX2	-0.30443	0.432715	0.144302	-0.06755	0.064721	-0.18297	-0.05403	-0.32271
SSX2IP	0.525126	-0.17875	0.267173	-0.41019	0.527931	0.037154	0.140105	0.01777
SSX3	-0.00444	-0.19972	0.297198	-0.02469	0.205578	-0.14742	0.329849	0.114825
SSX4B	NA	NA	-0.25331	-0.15436	0.099171	0.140835	-0.06478	-0.15735
SSX5	-0.08594	0.253501	-0.09087	-0.30151	-0.04712	-0.00594	0.144568	0.313497
SSX6	0.177953	0.712628	NA	NA	NA	NA	0.121677	-0.1504
SSX7	-0.1271	0.43112	0.069223	-0.17807	0.204026	-0.1112	-0.16593	0.172628
SSX8	0.244188	0.492455	0.077115	-0.02955	0.349853	0.072048	NA	NA
SSX9	0.253916	0.610397	0.113213	-0.21253	-0.06287	-0.34559	-0.39185	-0.18588
ST13	0.39894	-0.0784	0.107098	0.259832	0.072248	-0.06333	0.019409	-0.33595
ST14	0.003352	0.062266	0.253896	-0.00305	0.307176	0.232182	0.540831	0.113127
ST18	-0.0364	0.237012	-0.03026	-0.14794	-0.48814	-0.0976	-0.04621	0.262659
ST3GAL1	-0.07503	-0.01518	0.165305	0.462562	0.14859	0.71695	-0.31905	0.424023
ST3GAL2	0.17438	0.487514	0.467012	0.291702	0.343489	0.519766	0.072906	0.503214
ST3GAL3	0.060796	0.298184	0.209823	-0.2052	0.097909	0.240883	-0.18527	-0.2507
ST3GAL4	-0.26277	-0.5104	-0.1003	0.124807	-0.33669	-0.15953	-0.13031	-0.23386
ST3GAL5	-0.0265	-0.44321	0.093993	0.662067	-0.35654	0.306134	-0.37872	0.417581
ST3GAL6	-0.59879	-0.45974	-0.11102	-0.31396	-0.34595	0.045932	-0.51457	-0.03236
ST5	0.07777	0.043169	0.142112	0.070388	0.039829	0.391794	-0.2227	-0.30436
ST6GAL1	0.187467	-0.03654	-0.24664	0.326361	-0.01546	-0.15456	0.044065	-0.30428
ST6GAL2	0.092887	0.321944	0.039587	0.347562	-0.22009	0.132874	-0.04324	-0.04833
ST6GALNAC	-0.39086	-0.05526	-0.24183	0.103707	-0.01502	-0.208	0.190971	-0.00523
ST6GALNAC	-0.63732	-0.02593	0.188655	0.049338	-0.13774	-0.17886	-0.30741	-0.54018
ST6GALNAC	-0.09348	-0.22866	-0.17546	-0.35239	-0.29758	0.117951	-0.24358	-0.57039
ST6GALNAC	-0.05534	0.403236	0.294553	0.231363	0.225163	-0.07709	-0.00046	-0.10836
ST6GALNAC	-0.25655	-0.14013	0.111836	0.047442	-0.17277	-0.06717	-0.50873	-0.32236
ST6GALNAC	-0.42853	-0.16592	0.072904	0.124752	-0.05332	0.11761	-0.16143	-0.23305

ST7	0.206635	-0.12491	-0.03189	0.048196	-0.0678	-0.1892	0.201912	0.284444
ST7L	-0.20439	-0.54352	0.161696	-0.24246	0.05752	-0.16414	0.102067	-0.18133
ST8SIA1	-0.15152	-0.34491	-0.03039	0.213029	-0.46206	0.431072	-0.39757	-0.27489
ST8SIA2	-0.25398	-0.40491	-0.07292	0.016531	0.141884	-0.10147	0.10806	-0.09824
ST8SIA3	-0.34051	0.254176	0.305363	-0.04379	-0.11152	0.180008	0.560652	0.288636
ST8SIA4	-0.01043	0.018786	-0.03504	0.405298	-0.10759	0.175176	-0.61147	0.131732
ST8SIA5	0.238883	0.552972	-0.03316	0.134308	-0.2005	0.006656	-0.22064	-0.10334
ST8SIA6	-0.18295	0.602977	NA	NA	NA	NA	0.592847	-0.14929
STAB1	-0.37114	-0.12114	0.338738	-0.40828	-0.14013	0.276523	-0.34754	0.18779
STAB2	-0.25683	-0.37287	-0.04728	-0.03494	-0.20954	-0.30302	0.41317	0.20121
STAC	0.016339	-0.09362	0.105412	0.103277	-0.02686	0.060655	-0.58487	-0.0624
STAC2	0.123505	0.024056	0.336331	-0.01535	-0.0998	-0.01602	-0.53994	-0.40564
STAC3	-0.16179	0.250683	0.222257	0.197702	-0.01902	0.15884	-0.19466	-0.006
STAG1	0.076122	-0.18434	-0.42893	-0.12448	-0.12138	-0.32634	-0.01384	0.075643
STAG2	0.189265	-0.64159	-0.07751	-0.12002	0.026987	0.236004	-0.09394	-0.37527
STAG3	0.044262	-0.19481	-0.44496	0.483579	-0.03222	-0.35925	-0.15803	-0.18894
STAG3L1	NA	NA	0.215083	-0.23272	0.305049	0.297247	NA	NA
STAG3L2	NA	NA	0.168578	-0.06629	0.132953	0.428609	NA	NA
STAG3L4	NA	NA	-0.08682	-0.27268	-0.36117	-0.22312	NA	NA
STAM	-0.17997	-0.62509	-0.17952	0.101444	-0.15833	-0.33577	0.237183	0.125524
STAM2	0.119606	0.13396	-0.20545	-0.24166	-0.30967	-0.4256	0.241316	0.17098
STAMBP	0.29008	0.326886	-0.05773	0.086414	0.118169	-0.06715	0.468247	-0.1642
STAMBPL1	0.267885	-0.02896	-0.09467	0.195089	0.239048	-0.0967	0.25112	0.022677
STAP1	NA	NA	-0.07234	0.603083	-0.25283	0.369465	NA	NA
STAP2	0.222699	0.260127	-0.19409	-0.09796	0.114858	-0.22006	0.406839	0.139303
STAR	-0.06201	0.028455	0.030725	0.645046	-0.21606	0.31578	-0.58159	-0.5166
STARD10	-0.28858	0.008099	0.205813	0.317883	0.1997	-0.14795	0.516227	-0.12575
STARD13	0.220573	0.064096	0.184148	-0.21746	0.194411	-0.07299	0.002848	-0.17071
STARD3	0.183598	0.591264	0.438465	0.264375	0.256968	-0.03062	-0.22249	-0.05372
STARD3NL	0.322195	-0.07878	0.065824	-0.40178	-0.04987	-0.24936	0.315504	0.278022
STARD4	0.182368	-0.02362	-0.24555	-0.11032	-0.40026	0.092728	-0.27322	0.007952
STARD5	0.070842	0.358658	0.116049	0.303217	-0.08693	-0.31059	0.157307	0.06589
STARD6	-0.09568	0.503874	-0.16163	-0.0547	0.153347	0.198302	-0.42778	0.152644
STARD7	0.125985	0.030538	0.265327	0.065635	0.278826	0.311174	0.460159	0.446238
STARD8	-0.27278	0.221465	0.0856	-0.13413	0.017332	-0.16597	-0.51421	-0.23974
STARD9	NA	NA	-0.14821	-0.03616	0.190175	0.107726	NA	NA
STAT1	0.042596	-0.00048	0.265052	-0.19755	-0.27419	0.21288	-0.36566	-0.08691
STAT2	-0.22116	-0.03755	0.074447	0.083517	-0.11395	0.298962	-0.32983	0.347824
STAT3	-0.21298	-0.40069	0.464537	0.221165	-0.00132	0.382988	-0.19388	0.253488
STAT4	-0.64325	-0.14091	0.176165	-0.00484	-0.49559	0.456508	-0.28875	0.760155
STAT5A	0.168508	-0.07253	0.26865	0.479455	0.097327	0.510515	-0.56031	-0.11657
STAT5B	0.059947	-0.17934	0.529963	0.178248	0.207208	0.507788	0.321901	0.428126
STAT6	0.284979	0.34069	-0.18401	-0.03284	0.274551	0.143978	0.141972	0.416476
STATH	0.054112	0.534444	-0.26289	-0.31065	0.199976	0.015572	0.644154	0.505599
STAU1	0.328708	-0.73837	0.146343	-0.1464	0.348287	0.217161	0.392276	0.390628
STAU2	0.09012	-0.51734	0.232852	-0.50759	-0.10565	-0.53574	0.31285	-0.0581
STBD1	NA	NA	0.031426	-0.24926	0.118885	-0.09556	NA	NA
STC1	0.338923	0.207901	0.014649	-0.04981	0.234692	0.363341	-0.11012	0.054386
STC2	-0.11991	-0.14669	-0.04965	-0.04573	-0.07348	-0.02355	-0.26113	-0.09405
STEAP1	0.146199	-0.29995	-0.15187	-0.0636	-0.05737	-0.23978	-0.03615	-0.29852
STEAP2	0.224322	-0.16687	0.048326	-0.20658	0.038851	-0.18424	-0.34621	-0.01559
STEAP3	0.247297	0.504595	0.150718	-0.05191	0.154702	-0.05361	0.288767	0.254655

STEAP4	-0.42735	-0.38514	0.09241	0.027334	-0.03191	0.059831	-0.10546	0.437295
STGC3	-0.13032	-0.20868	0.003264	-0.23951	-0.02112	0.190823	-0.35577	-0.41066
STH	-0.0738	0.106518	0.043284	-0.25025	-0.08937	-0.08067	0.276837	-0.11643
STIL	-0.20028	0.059753	-0.09635	0.028517	0.208519	0.064798	0.2327	0.24518
STIM1	-0.21299	-0.05616	0.409679	-0.03893	0.032171	-0.00365	-0.1435	-0.40056
STIM2	-0.3398	-0.25193	0.007621	0.286468	0.190946	0.017738	-0.17542	-0.14318
STIP1	0.010534	0.429709	0.173135	0.2638	0.219345	0.112518	0.366321	0.194021
STK10	-0.35905	0.140777	-0.08512	0.45363	-0.15493	0.333382	-0.05906	0.544015
STK11	-0.06814	-0.01246	0.315802	0.003852	0.301467	0.052231	-0.43786	-0.21881
STK11IP	0.300252	0.669567	0.173204	0.050281	0.358144	0.066905	0.24481	0.468892
STK16	0.334136	0.749254	0.314251	-0.00818	0.063077	-0.16981	-0.28565	-0.39351
STK17A	0.087184	0.472983	-0.24496	0.247605	-0.30386	0.055509	-0.16598	0.244553
STK17B	-0.67712	-0.187	0.080783	0.204486	-0.21709	0.295748	-0.0924	0.17208
STK19	-0.09403	-0.17198	0.126608	0.167143	0.116271	0.243098	-0.07125	0.228416
STK24	0.381959	0.140524	0.242553	0.218385	0.5347	-0.15706	0.251274	0.10134
STK25	0.071616	-0.21291	0.269883	0.132336	0.364016	0.27573	0.300414	0.339541
STK3	-0.01767	0.077922	0.113218	-0.1609	-0.20558	-0.12032	-0.24611	-0.04869
STK31	0.03999	0.640918	-0.3021	-0.13792	0.133771	-0.16541	-0.22941	0.010456
STK32A	0.494008	0.329745	0.067676	-0.17905	-0.10022	0.070849	-0.17416	0.024401
STK32B	0.320526	0.604237	0.072935	0.130672	-0.37807	-0.1915	-0.331	-0.0031
STK32C	0.023317	-0.04027	-0.13402	0.212755	0.039433	0.223132	-0.4173	-0.27074
STK33	0.432741	-0.25048	-0.06367	-0.13528	-0.31737	-0.03048	-0.11171	-0.39401
STK35	0.148272	-0.50879	-0.22201	0.117617	0.121577	-0.02496	0.285275	-0.43594
STK36	0.342941	0.399163	0.202563	0.177392	0.294125	0.149845	0.242985	0.289931
STK38	0.083374	0.027409	0.176686	0.003361	0.446371	0.049738	0.380969	0.380166
STK38L	0.094508	0.229168	0.067589	-0.42083	-0.16991	-0.40941	-0.14254	-0.22958
STK39	0.011062	-0.18971	0.101133	-0.05179	0.198228	-0.27274	0.377811	0.099349
STK4	0.430948	0.558305	0.123492	0.201218	0.034992	0.436433	0.194693	-0.27705
STK40	-0.26521	0.820974	0.135474	-0.0723	0.043102	0.253455	0.049655	0.089225
STMN1	-0.29008	0.136851	-0.25228	0.191601	0.195517	0.073793	0.362284	0.124289
STMN2	-0.21128	-0.30798	0.11403	-0.36228	-0.10138	-0.03043	-0.29019	-0.62971
STMN3	0.144005	-0.19325	0.362273	0.330487	0.089059	0.519611	NA	NA
STMN4	-0.05927	0.064385	-0.15954	0.071484	-0.24865	-0.05802	-0.35744	-0.22807
STOM	-0.48284	-0.28471	-0.03782	0.047366	0.020906	0.35259	-0.52666	-0.2042
STOML1	0.061718	0.415122	0.221933	0.003543	0.34586	-0.22946	0.340042	0.371182
STOML2	0.471512	0.620578	0.13086	0.00262	0.001606	-0.05268	0.429058	0.156536
STOML3	-0.00514	0.426636	-0.24291	-0.3742	0.248893	-0.04494	0.243723	0.427995
STON1	-0.04074	-0.10394	0.085876	-0.10532	-0.17622	0.166805	-0.31697	-0.36911
STON2	-0.20514	-0.33997	-0.33583	-0.14358	-0.12453	-0.43665	-0.02638	-0.02711
STOX1	0.299251	-0.249	0.097297	0.07927	0.353129	-0.20929	0.036856	-0.00135
STOX2	-0.06711	0.542909	-0.02765	-0.16024	-0.15617	0.156572	-0.35832	-0.13202
STRA13	-0.37548	0.561912	0.147873	0.119078	0.167466	-0.30849	0.385235	-0.00801
STRA6	0.208608	-0.11004	0.295453	0.098404	0.113197	-0.05452	0.283162	0.01549
STRA8	0.280615	0.107542	-0.15971	0.10549	0.198452	0.151441	NA	NA
STRADA	NA	NA	0.379448	-0.05976	-0.01124	0.195424	NA	NA
STRADB	NA	NA	0.03561	-0.24604	-0.1777	-0.45051	NA	NA
STRAP	0.475862	-0.47568	-0.17498	0.010253	0.271926	-0.17069	-0.37498	-0.68447
STRBP	0.373517	0.046067	-0.15271	0.085899	0.150798	-0.12989	0.407464	0.0632
STRC	0.262645	0.572252	-0.01766	0.024574	0.138473	-0.00686	0.191346	0.431899
STRN	-0.34598	-0.4407	-0.07179	-0.00403	0.033696	-0.20236	-2E-05	-0.03814
STRN3	-0.46551	-0.68834	-0.27125	-0.35812	-0.0225	-0.27696	0.197298	0.231214
STRN4	0.374465	0.580193	0.140592	0.116035	0.119221	0.108926	0.632654	0.346304

STS	-0.5338	0.470111	-0.06136	0.120514	0.110623	-0.07639	-0.0621	0.36567
STT3A	-0.37131	-0.66318	0.073384	0.400929	-0.11219	-0.11614	0.564004	0.503108
STT3B	-0.27911	-0.41181	0.097454	0.069	0.119213	0.009312	0.623991	0.194395
STUB1	0.431741	0.630121	0.25233	0.338937	0.307918	0.250027	0.223603	-0.13381
STX10	-0.06277	0.710809	0.194769	0.203264	0.152602	-0.10042	0.365956	0.412254
STX11	-0.54654	-0.17953	0.242666	0.191931	-0.07634	0.153264	-0.2439	0.308735
STX12	-0.3921	0.05113	-0.05264	-0.55466	-0.13032	-0.39879	-0.65377	-0.56702
STX16	0.287704	-0.36012	-0.05654	0.069467	0.265402	0.147882	0.230021	0.283805
STX17	0.209517	0.562689	0.052274	-0.31226	-0.08908	-0.37607	-0.0619	0.457733
STX18	-0.12427	-0.1426	-0.00696	0.148633	0.039874	-0.05127	0.453935	0.121685
STX19	0.095635	0.229895	NA	NA	NA	NA	0.113434	-0.1513
STX1A	0.107992	0.004399	-0.27445	-0.06955	-0.03824	-0.23283	0.37135	0.325167
STX1B	NA	NA	0.148409	-0.0887	-0.16415	0.036933	NA	NA
STX2	-0.36152	-0.26252	-0.12549	-0.06471	0.014418	-0.25159	-0.17606	-0.15396
STX3	0.123374	0.050872	-0.13507	-0.0588	0.099462	-0.40191	NA	NA
STX4	NA	NA	0.201193	0.198406	0.368675	0.102945	NA	NA
STX5	NA	NA	0.194593	-0.04481	-0.12491	-0.22678	NA	NA
STX6	-0.3406	-0.67241	-0.07653	-0.15706	-0.13095	-0.23622	-0.02426	0.032486
STX7	-0.15819	-0.25708	-0.1801	0.073978	-0.18355	-0.19601	-0.43245	-0.35044
STX8	-0.28551	-0.19453	-0.09158	-0.22439	-0.23994	-0.47333	-0.56689	-0.51667
STXBP1	-0.42163	0.355876	0.067011	0.166505	0.324061	0.290636	0.386982	0.070133
STXBP2	0.281052	0.214607	0.088853	0.13239	0.057196	-0.10286	0.530926	0.29525
STXBP3	-0.27037	-0.62355	-0.10862	-0.23485	0.122079	-0.19776	0.600134	0.159189
STXBP4	-0.06492	-0.27704	0.359598	-0.42702	-0.08354	0.182996	NA	NA
STXBP5	-0.07178	-0.27368	-0.35489	-0.15748	-0.26927	-0.08464	0.121004	0.258571
STXBP5L	NA	NA	-0.14835	-0.34831	-0.32535	-0.34554	NA	NA
STXBP6	-0.23106	-0.30402	-0.14549	0.286101	-0.04107	-0.50232	-0.03096	-0.25211
STYK1	-0.22285	-0.09931	-0.17885	-0.03874	-0.27411	-0.40099	-0.33982	-0.12622
STYX	NA	NA	-0.38749	0.047149	-0.3772	-0.2605	-0.08534	0.320761
STYXL1	0.479468	0.467668	-0.07293	-0.04041	-0.33161	-0.0277	0.314255	-0.0621
SUB1	0.046356	-0.06193	-0.20676	-0.2048	0.022566	-0.2003	0.184061	-0.2476
SUCLA2	0.026931	-0.62019	-0.31276	-0.15038	-0.25142	-0.37867	0.398566	0.085148
SUCLG1	0.307858	0.096155	0.017141	-0.10163	0.068386	-0.06618	0.448159	0.231578
SUCLG2	-0.27629	0.260895	-0.26794	-0.06387	-0.06128	-0.10454	-0.28002	-0.42004
SUCNR1	-0.21326	-0.2668	0.316115	-0.43694	-0.14737	0.021974	NA	NA
SUDS3	0.301226	0.417671	-0.18918	0.187546	0.121537	-0.04862	-0.62421	-0.48832
SUFU	-0.14949	0.319246	0.30617	0.17094	0.155204	0.109568	-0.48408	-0.30846
SUGT1	0.384412	-0.36594	0.039278	-0.02957	-0.02032	-0.25286	0.624598	-0.03043
SULF1	-0.20892	-0.39136	0.000659	-0.109	-0.0439	0.223641	-0.07198	0.018546
SULF2	0.027395	-0.47652	0.088984	0.248299	0.062121	-0.09338	-0.34594	-0.36152
SULT1A1	0.095449	-0.22696	-0.05918	-0.22609	0.037844	-0.20428	-0.40014	-0.23643
SULT1A2	-0.20643	-0.07326	-0.3202	-0.2915	-0.00693	-0.34731	-0.11196	0.198774
SULT1A4	-0.18474	-0.28643	-0.10516	-0.25879	0.01655	-0.21752	0.307742	0.450851
SULT1B1	0.068824	-0.12006	-0.15785	-0.29658	-0.20453	-0.44486	NA	NA
SULT1C2	0.191328	0.573007	-0.32568	0.184834	-0.19816	-0.28845	-0.18294	-0.25489
SULT1C3	-0.01908	0.412459	NA	NA	NA	NA	0.144061	-0.22306
SULT1C4	NA	NA	0.169814	0.103058	-0.33423	0.135763	NA	NA
SULT1E1	-0.30862	0.67228	0.113595	-0.08961	0.032448	-0.1757	-0.03389	-0.07692
SULT2A1	0.371379	0.034622	0.258862	-0.23986	0.123336	-0.4399	0.158952	-0.11847
SULT2B1	0.111664	0.134879	0.386245	0.066368	0.332056	0.045098	0.08382	0.101068
SULT4A1	0.138082	-0.1564	0.244183	-0.11392	0.283861	-0.06038	-0.13946	-0.01481
SULT6B1	-0.19829	0.538575	NA	NA	NA	NA	0.559284	0.549435

SUMF1	0.288664	-0.13702	0.018013	-0.14657	-0.16875	-0.04762	-0.24342	-0.41304
SUMF2	-0.17459	-0.3662	0.285445	0.257409	0.358058	0.07515	-0.1637	-0.14878
SUMO1	-0.1079	0.264844	-0.17792	0.098354	-0.1631	-0.33033	-0.46873	-0.55618
SUMO1P3	NA	NA	-0.25306	0.144006	-0.15241	-0.27379	-0.66989	-0.33987
SUMO2	0.001228	-0.41109	0.026088	0.01346	0.015316	-0.36262	-0.37699	-0.49295
SUMO3	-0.12806	-0.45095	-0.27609	0.090021	-0.18394	-0.41261	0.294814	0.306246
SUMO4	-0.06448	-0.48041	NA	NA	NA	NA	-0.44011	-0.39577
SUNC1	0.552032	0.416554	-0.22951	-0.11945	-0.14142	0.194118	-0.42781	-0.50537
SUOX	-0.24707	0.352416	0.235807	0.225445	0.390682	0.006094	0.012728	0.097292
SUPT16H	-0.05433	-0.23638	-0.39534	0.039841	0.110736	0.30063	0.403832	0.427394
SUPT3H	0.450717	0.362323	-0.01943	-0.14449	-0.00152	-0.30201	-0.08639	-0.40936
SUPT4H1	0.153018	-0.25851	0.149669	-0.37199	-0.17487	-0.19071	0.284507	0.192002
SUPT5H	0.041482	-0.00966	0.034887	0.32661	0.207806	0.386092	0.385892	0.28902
SUPT6H	0.22975	0.371141	0.34982	0.055842	0.103976	0.187688	-0.27846	-0.25864
SUPT7L	0.155943	-0.628	-0.10636	-0.14799	0.18968	-0.20062	0.318036	0.246525
SUPV3L1	0.339706	0.063317	-0.38036	-0.05122	0.09995	-0.1573	0.440287	0.41396
SURF1	0.300429	0.288459	0.124207	-0.17795	0.083587	-0.2597	-0.01196	-0.1059
SURF2	0.327861	0.797964	-0.07125	0.242724	0.168541	0.265438	-0.01116	0.148942
SURF4	-0.55963	-0.43515	-0.18567	0.353169	0.247679	-0.18087	0.346566	0.55093
SURF6	0.181355	-0.43137	0.087498	0.191804	0.29745	0.051257	0.14051	0.337029
SUSD1	0.363485	0.121135	-0.11422	0.189762	0.06041	-0.22654	0.283211	0.242417
SUSD2	-0.147	0.326229	0.079912	-0.01854	-0.09163	-0.23649	-0.0875	0.353237
SUSD3	0.366934	0.085054	0.061405	0.684296	-0.20532	0.283699	-0.01688	0.125769
SUSD4	-0.23605	0.052891	0.08198	-0.00653	-0.36655	0.169962	-0.14727	-0.22107
SUSD5	NA	NA	0.042614	0.228136	-0.29281	-0.24535	NA	NA
SUV39H1	0.264818	0.596996	0.009987	0.35093	0.234468	0.022047	0.192336	0.034503
SUV39H2	0.036446	0.737621	-0.1674	-0.08398	-0.07196	-0.00534	0.27871	0.263844
SUV420H1	-0.24291	-0.25638	-0.51115	-0.0166	-0.18402	-0.37083	0.143689	0.339211
SUV420H2	-0.25839	-0.10375	0.211449	0.109838	0.262184	-0.20603	0.073666	-0.08062
SUZ12	0.144151	0.355984	0.041772	0.020865	-0.0694	0.059601	0.333332	0.140866
SV2A	-0.28644	0.26113	0.184787	0.060152	0.031017	-0.11716	-0.04746	-0.135
SV2B	-0.2198	-0.27517	-0.12459	0.469537	0.15501	0.628311	-0.49521	-0.0759
SV2C	0.443764	0.60587	-0.02092	-0.07911	-0.15652	-0.15767	0.398064	0.278673
SVEP1	NA	NA	-0.16012	0.016615	0.091913	0.218342	-0.1977	0.029093
SVIL	-0.33016	-0.2094	0.022614	-0.22895	-0.09872	0.195144	-0.41233	-0.42055
SVIP	NA	NA	-0.30188	-0.01438	-0.1633	-0.26763	NA	NA
SVOP	-0.12034	0.063283	-0.39105	0.100038	-0.30634	-0.26173	0.261597	-0.13774
SVOPL	-0.36352	-0.15789	-0.03428	-0.26685	-0.13994	-0.23836	NA	NA
SWAP70	-0.42003	-0.62241	-0.19767	0.437688	-0.22811	0.183914	-0.32201	0.108212
SYAP1	0.054826	-0.61308	-0.11098	0.056012	-0.0383	-0.40407	-0.65013	-0.63745
SYCE1	0.133747	0.32565	-0.28537	-0.34172	-0.19438	-0.03768	0.414336	0.497698
SYCE2	NA	NA	-0.37107	-0.01491	0.040756	0.104674	NA	NA
SYCN	NA	NA	-0.33213	-0.19005	-0.0521	0.146494	0.471813	0.30769
SYCP1	0.668802	0.204231	0.25714	-0.10841	0.18633	0.123693	0.424111	0.420026
SYCP2	-0.21103	-0.4379	0.098714	0.420133	-0.01816	0.098993	-0.03804	0.363493
SYCP2L	NA	NA	-0.18161	0.159618	-0.01265	-0.07917	NA	NA
SYCP3	-0.11952	0.754793	-0.33894	0.075585	0.140019	-0.09055	0.262786	0.032481
SYDE1	-0.20247	-0.08545	0.149147	0.0086	0.07654	0.012319	-0.28779	-0.30414
SYDE2	NA	NA	0.374192	-0.21621	0.29295	-0.09336	NA	NA
SYF2	0.177447	0.256793	-0.25752	-0.17896	0.15267	0.050889	-0.16927	-0.22576
SYK	0.15932	0.067523	0.43872	0.339331	0.179437	0.350376	0.441257	0.473122
SYMPK	-0.14255	-0.08953	0.011386	0.255126	0.384739	0.109305	-0.1382	0.034243

SYN1	0.377557	0.406209	0.014611	-0.03234	-0.0044	0.254997	-0.02424	-0.02787
SYN2	-0.66448	-0.44294	-0.021	-0.1095	-0.30177	0.275003	-0.50007	-0.08304
SYN3	0.146506	0.25881	0.024461	0.037986	-0.09062	0.373183	-0.17384	0.066238
SYNC	NA	NA	-0.1114	-0.40104	-0.14748	-0.33321	NA	NA
SYNCRIP	0.09361	-0.35933	-0.2174	0.152097	0.317818	0.240991	-0.06172	-0.03906
SYNE1	-0.35609	0.059302	0.129671	-0.03682	-0.32129	0.498784	-0.73335	-0.62271
SYNE2	0.30625	0.359516	0.076724	0.089519	0.109749	0.28538	0.048132	0.079173
SYNGAP1	-0.01113	0.262159	0.11647	-0.12526	0.279441	0.122983	-0.24412	-0.13565
SYNGR1	-0.4063	0.029759	0.213675	0.056429	-0.00091	0.137892	-0.38823	-0.23811
SYNGR2	0.170021	0.292197	0.199642	0.303568	-0.20339	0.141527	0.140181	-0.0437
SYNGR3	0.002943	-0.13818	-0.20628	0.192837	0.292616	-0.05468	0.0523	-0.01458
SYNGR4	0.046881	0.115729	-0.1282	-0.06481	-0.08904	0.115881	0.158761	-0.07608
SYNJ1	-0.24689	-0.17809	-0.1555	-0.2527	-0.26552	-0.32628	-0.50273	-0.13526
SYNJ2	0.094073	9.02E-05	0.08444	-0.14868	0.271235	-0.07288	0.475324	-0.00096
SYNJ2BP	-0.09043	0.484935	-0.06782	-0.25897	-0.50307	-0.12027	-0.53943	-0.47161
SYNM	NA	NA	-0.13123	-0.00708	0.171426	0.062163	NA	NA
SYNPO	0.384347	0.382517	-0.0704	0.082582	-0.11732	0.025267	-0.42528	-0.1761
SYNPO2	-0.1404	0.063596	0.071767	-0.05047	0.145945	0.257618	-0.30456	-0.23685
SYNPO2L	-0.51482	-0.05254	0.017207	-0.21842	0.099832	-0.21354	-0.49839	-0.22658
SYNPR	-0.12295	-0.07097	-0.07002	-0.18759	0.122165	0.063083	0.023793	-0.15618
SYP	0.25137	0.179122	0.037561	0.411968	0.018414	-0.23753	-0.01437	0.217209
SYPL1	-0.14823	0.206134	-0.05457	-0.04027	0.137294	-0.13675	0.360029	0.229503
SYPL2	-0.18042	-0.22995	NA	NA	NA	NA	-0.32231	-0.12979
SYS1	-0.0704	0.248202	0.365127	0.110266	0.131009	-0.12217	NA	NA
SYT1	0.1615	-0.39636	-0.18207	-0.02565	-0.09736	0.153352	0.190046	-0.20942
SYT10	0.390918	0.41481	0.153344	-0.3044	-0.08613	-0.18874	-0.43943	-0.30687
SYT11	-0.20446	-0.07623	0.1159	0.112442	-0.1162	0.321704	-0.35088	-0.51104
SYT12	-0.32715	0.434412	-0.12495	0.036495	0.030368	-0.0763	0.416984	0.323015
SYT13	-0.22814	0.024335	-0.13649	0.105223	0.164437	0.247949	0.137876	-0.07503
SYT14	0.172948	0.220915	0.248957	0.296086	-0.03791	-0.03796	0.219366	0.087035
SYT15	-0.22626	-0.055	-0.13001	-0.45197	0.052344	0.240488	0.057501	-0.27851
SYT16	-0.05183	0.667462	0.194494	-0.29391	0.0287	-0.35661	-0.09084	0.117961
SYT17	-0.0321	0.388162	0.006382	0.036969	-0.13022	-0.41985	0.105143	-0.29505
SYT2	0.036522	0.305609	-0.069	-0.08617	-0.25934	-0.21684	0.420609	-0.03147
SYT3	0.139984	-0.10358	0.126421	-0.11777	0.054231	0.206068	0.317766	0.062098
SYT4	0.216689	0.083906	-0.03688	-0.2376	-0.24634	-0.18438	-0.39123	-0.39548
SYT5	0.17947	0.189082	-0.00962	-0.17104	-0.22099	-0.04209	-0.22679	-0.13615
SYT6	0.170048	0.497526	-0.28446	0.022274	0.006974	-0.38811	0.144508	0.212172
SYT7	0.102357	0.645264	0.242468	0.219783	0.36794	-0.25107	0.278146	-0.10104
SYT8	-0.39972	0.557385	0.023819	-0.08873	-0.37662	-0.45291	0.171021	0.390672
SYT9	0.134695	0.41908	0.02815	0.115038	0.174646	-0.13963	0.016954	0.393203
SYTL1	-0.40893	0.098909	0.214298	0.580557	-0.05607	-0.02678	0.011526	0.107736
SYTL2	0.028172	-0.12937	-0.05067	-0.19525	-0.01614	-0.07668	-0.08719	-0.09951
SYTL3	-0.34828	0.144185	0.268772	-0.00449	-0.0604	0.230194	-0.1816	0.534517
SYTL4	-0.47781	0.403056	-0.39936	-0.15978	0.082626	-0.3348	-0.04571	-0.1933
SYTL5	0.038795	-0.03688	-0.11154	-0.04565	-0.28809	-0.15801	0.118266	0.093269
SYVN1	-0.11552	-0.10623	0.153311	0.360629	0.235283	0.049869	-0.31023	-0.20963
T	0.212139	0.033419	0.183527	-0.2082	0.237098	-0.12499	0.031192	0.214177
TAAR1	-0.00766	0.53059	0.128559	0.161006	-0.10292	0.038373	-0.10861	-0.24094
TAAR2	0.097094	0.568195	0.170007	-0.38325	0.229474	0.05871	-0.14106	-0.08553
TAAR5	0.171992	-0.06654	0.31262	-0.2413	0.036219	-0.13059	-0.3291	-0.17227
TAAR6	0.521768	0.524893	-0.09632	0.176558	-0.18402	-0.11702	NA	NA

TAAR8	0.418574	0.461455	0.096375	-0.22424	0.263084	-0.24982	0.178992	0.093525
TAAR9	0.286133	0.471903	0.060503	0.033357	0.178487	-0.04417	0.173248	0.028016
TAB3	NA	NA	NA	NA	NA	NA	0.028182	0.324484
TAC1	0.211028	-0.0978	0.237055	-0.09137	-0.09676	-0.0677	-0.21889	-0.03929
TAC3	-0.02606	-0.12462	-0.18813	0.023435	-0.18629	0.167206	-0.16706	-0.03025
TAC4	0.47316	0.34914	0.228039	-0.20623	-0.05492	0.096905	0.024266	-0.27899
TACC1	-0.2989	-0.22436	0.136379	-0.15522	-0.06608	0.109844	-0.13526	-0.18666
TACC2	0.345641	0.212341	-0.12062	-0.36187	0.250031	0.115927	-0.00308	0.063193
TACC3	-0.08122	0.140681	0.100901	0.515295	0.228491	0.095792	0.044483	0.035626
TACR1	0.293904	0.497619	0.041683	-0.1045	-0.01063	-0.24246	0.491586	0.200214
TACR2	0.295235	0.685355	0.067721	-0.15851	0.084004	-0.16734	-0.31481	-0.30051
TACR3	0.095482	0.220044	0.260313	0.251503	0.264623	-0.02531	0.467584	0.21897
TACSTD2	-0.11289	0.03785	0.213352	-0.00598	-0.00683	0.129552	-0.07731	-0.07097
TADA1L	0.010939	-0.14783	-0.39416	0.091147	-0.2577	-0.09222	-0.05849	-0.20642
TADA2B	NA	NA	0.121848	0.162358	0.146237	0.036235	NA	NA
TADA2L	0.076967	-0.57379	-0.10881	0.148017	-0.10561	-0.09628	-0.00496	0.271991
TADA3L	0.439906	0.362023	-0.05869	0.033553	-0.00905	-0.35744	-0.032	-0.29298
TAF1	0.054438	-0.19909	-0.22619	0.135937	0.228342	0.38266	-0.30417	-0.3654
TAF10	0.224054	0.484977	0.243274	0.116917	0.077041	-0.03065	0.602512	0.29103
TAF11	-0.25122	0.32666	-0.47168	0.060612	0.028071	-0.21353	-0.1549	0.435681
TAF12	-0.05476	-0.51337	0.168259	-0.16945	-0.04544	-0.48252	NA	NA
TAF13	-0.26457	0.418757	-0.13448	-0.22876	-0.24956	-0.30006	-0.25669	0.145483
TAF15	0.040445	-0.4415	0.057482	0.239271	0.024103	0.505708	0.174817	0.150468
TAF1A	-0.03263	-0.41906	-0.34774	-0.12759	-0.12252	0.199046	-0.2499	-0.24292
TAF1B	0.100256	-0.28609	0.102538	-0.0353	-0.01384	0.025462	-0.22135	-0.13117
TAF1C	-0.04834	0.17974	-0.02575	0.254805	0.455876	0.21163	0.190679	0.37096
TAF1D	NA	NA	-0.18781	0.201517	-0.0492	-0.09164	NA	NA
TAF1L	-0.10404	-0.54572	-0.01693	-0.0993	0.252538	0.029706	0.395631	0.456973
TAF2	-0.1247	-0.66435	-0.11419	-0.33499	-0.21038	0.076862	0.600773	0.293869
TAF3	NA	NA	-0.32033	0.050039	0.136167	-0.03873	NA	NA
TAF4	0.404405	-0.49578	-0.08755	-0.08476	0.14168	-0.10518	0.426445	0.113869
TAF4B	NA	NA	-0.1379	0.307375	0.051924	0.40394	NA	NA
TAF5	0.006423	0.126901	-0.06751	0.01489	0.001525	-0.34718	0.268014	0.11576
TAF5L	-0.20399	0.2108	0.018914	0.083186	-0.21278	0.13661	-0.1441	-0.05242
TAF6	0.437619	-0.19819	-0.2771	0.279437	0.258934	0.330666	-0.12291	-0.54117
TAF6L	-0.06386	0.626993	0.289214	-0.1611	0.136888	-0.15961	0.297227	0.493726
TAF7	-0.25864	-0.3251	0.089736	-0.00073	0.089952	0.233207	0.045976	-0.36867
TAF7L	-0.2096	0.179083	-0.34843	0.115726	0.123715	-0.0018	-0.01354	-0.32543
TAF8	NA	NA	-0.12179	0.008592	-0.19297	-0.05656	NA	NA
TAF9	0.047001	-0.60846	-0.00419	-0.29086	-0.28276	-0.23856	-0.32537	-0.52765
TAF9B	NA	NA	-0.15671	-0.11575	0.039909	-0.4276	0.346903	0.297823
TAGAP	-0.46191	-0.12035	0.16104	0.652309	-0.01847	0.573377	-0.11826	0.186551
TAGLN	-0.10954	0.016345	0.24154	0.005326	0.016427	0.088445	-0.17802	-0.30142
TAGLN2	-0.346	-0.54299	0.071888	0.171895	0.215316	-0.2083	0.651544	0.294735
TAGLN3	-0.19558	0.036524	-0.14112	0.059336	-0.09939	-0.35569	-0.07664	-0.36624
TAL1	0.114775	0.582335	-0.00908	-0.24117	-0.12936	-0.46274	-0.02239	-0.08814
TAL2	-0.10005	-0.46983	-0.04588	0.102076	0.12558	-0.21199	-0.15683	-0.02985
TALDO1	0.281318	-0.48918	-0.16525	0.148602	0.392853	-0.23783	0.401399	0.148202
TANC1	-0.1871	-0.66286	-0.11127	-0.31802	0.077074	0.118289	-0.19156	0.016427
TANC2	NA	NA	-0.07283	-0.33482	-0.46378	-0.07112	NA	NA
TANK	-0.04048	-0.29122	-0.2895	-0.09765	-0.30992	-0.34154	-0.68001	-0.18968
TAOK1	-0.15926	-0.41518	0.169268	-0.42992	0.106828	0.395552	-0.18653	-0.25042

TAOK2	-0.05748	0.570758	0.192438	0.25946	0.221659	0.071903	0.028017	0.109554
TAOK3	-0.244	0.419641	-0.1159	0.078166	0.188802	-0.18398	-0.25178	0.104104
TAP1	0.59225	0.546038	0.159554	-0.08321	-0.07904	0.153489	-0.06155	0.220663
TAP2	0.256779	-0.26282	0.052264	0.113213	-0.17938	0.035719	-0.00371	0.493937
TAPBP	0.234937	-0.17977	0.323894	0.169709	0.178129	0.351694	0.233408	0.33735
TAPBPL	0.422807	0.426328	0.352198	0.220608	0.145627	-0.06556	-0.13884	0.091398
TAPT1	-0.36354	-0.59289	-0.00843	-0.04521	0.234255	-0.18594	NA	NA
TARBP1	0.142309	-0.19819	0.054876	-0.24658	0.083874	0.190767	0.421276	0.248641
TARBP2	0.218541	0.693357	-0.0071	0.264352	-0.16412	-0.2926	0.002579	-0.04403
TARDBP	0.386311	0.011543	-0.14127	0.017127	0.316052	0.244051	-0.44528	-0.26089
TARP	-0.21672	0.05292	0.104277	0.261601	-0.21681	0.053719	-0.60597	0.273368
TARS	0.151749	-0.46957	-0.10861	-0.10249	0.128965	0.069558	0.264803	-0.09668
TARS2	0.066125	-0.20328	0.021445	0.332831	0.158403	0.418811	NA	NA
TARSL2	0.05529	-0.30394	-0.0749	0.080826	-0.02351	0.063897	NA	NA
TAS1R1	-0.08603	-0.06352	0.041183	-0.18088	0.244871	-0.24825	0.150183	-0.01381
TAS1R2	0.329243	0.398904	0.225239	-0.2628	0.125745	-0.23325	0.343526	0.059327
TAS1R3	NA	NA	NA	NA	NA	NA	-0.15722	0.02948
TAS2R1	0.110507	0.613681	-0.23673	0.007203	0.000737	0.15779	0.418635	0.285674
TAS2R10	0.042508	0.672199	0.033364	-0.2285	-0.01713	0.118062	0.345366	-0.01503
TAS2R13	0.006843	0.159125	-0.26171	0.120525	-0.10149	0.145562	-0.35181	-0.20018
TAS2R14	0.115914	-0.55433	-0.22908	-0.30391	-0.08797	0.165262	0.173139	0.498728
TAS2R16	-0.25776	-0.22971	0.112753	-0.07426	-0.10748	-0.02231	-0.01238	-0.52283
TAS2R3	0.078944	0.614922	-0.03726	-0.0472	0.255342	0.032649	-0.40918	-0.55314
TAS2R38	0.031707	0.008527	-0.40979	-0.52678	-0.20918	-0.2571	-0.25474	-0.3533
TAS2R39	-0.18551	0.464963	0.181692	-0.28544	-0.08516	-0.54488	0.461168	0.516278
TAS2R4	0.199375	0.288988	0.110801	-0.02288	0.044662	0.107075	-0.02641	0.018666
TAS2R40	0.038444	0.510696	-0.09838	0.018735	-0.34621	0.174717	-0.07443	-0.33004
TAS2R41	-0.02041	0.460045	-0.06001	-0.10822	-0.11318	0.026288	NA	NA
TAS2R42	-0.23951	0.017815	-0.08719	0.257323	0.098985	-0.28117	NA	NA
TAS2R43	0.264145	0.178569	-0.00668	-0.3014	-0.0247	0.178071	0.452846	0.437696
TAS2R44	-0.04034	0.497022	-0.06804	-0.35866	0.105199	0.111138	-0.26142	0.303308
TAS2R45	0.158307	-0.00749	-0.29264	-0.305	-0.02265	0.461228	0.309255	0.04813
TAS2R46	0.01469	0.605614	-0.23271	-0.41152	-0.09665	0.018518	0.143997	0.301
TAS2R48	0.146137	0.600803	-0.07389	-0.26289	0.140131	0.366884	0.112491	0.263016
TAS2R49	0.109115	-0.12015	-0.00817	0.032362	-0.15125	0.065936	0.180241	0.450604
TAS2R5	-0.0065	0.130889	-0.00452	0.011032	0.323843	0.003732	0.40338	-0.0328
TAS2R50	0.057775	0.512726	0.300413	-0.18423	0.089665	-0.27459	-0.26659	-0.05329
TAS2R60	0.114829	0.51405	0.224671	-0.05256	-0.05488	-0.23146	-0.26521	0.106288
TAS2R7	0.362503	0.678144	-0.34534	-0.26906	0.159194	0.020735	-0.23099	-0.14203
TAS2R8	0.190253	0.582221	0.022298	-0.00992	-0.28574	-0.0846	0.128435	0.050478
TAS2R9	-0.26733	0.522515	0.0111	0.068672	-0.16157	0.032557	-0.06862	0.340661
TASP1	0.23803	-0.51552	-0.32402	-0.15853	-0.27379	0.06025	0.636799	0.426406
TAT	-0.17653	0.193606	0.009776	-0.26371	0.006035	0.019035	0.259026	0.244686
TATDN1	0.256821	-0.60061	0.0131	-0.24656	-0.19468	-0.28992	-0.1499	-0.45811
TATDN2	-0.06076	-0.10518	-0.09798	0.304942	0.070401	0.154813	-0.25399	-0.42251
TATDN3	NA	NA	-0.04904	-0.07572	-0.46166	-0.27738	0.020535	0.000528
TAX1BP1	0.134351	-0.39958	0.030907	-0.23482	0.107109	0.188995	0.171069	0.221507
TAX1BP3	-0.02897	-0.00332	0.034524	0.127694	0.004504	-0.2214	0.208245	-0.02556
TAZ	0.378482	0.641738	0.308064	0.134412	0.405229	0.059094	0.053377	0.392407
TBC1D1	0.017465	0.640959	0.131308	-0.13738	-0.05663	-0.20807	-0.60383	-0.29024
TBC1D10A	-0.47695	0.42251	0.344075	-0.0891	0.081402	0.021171	-0.32507	-0.44681
TBC1D10B	0.058744	0.486535	0.081217	0.057912	0.105502	0.356617	0.253461	-0.01636

TBC1D10C	-0.40338	0.324191	0.218813	0.767386	-0.18717	0.723475	-0.51035	0.422961
TBC1D12	NA	NA	-0.31204	-0.25826	-0.18024	-0.26054	NA	NA
TBC1D13	0.146851	0.449728	-0.0162	-0.08994	0.300077	0.10815	0.411862	0.585544
TBC1D14	-0.09456	-0.42201	0.085316	0.192256	0.299636	0.277362	0.189167	0.452357
TBC1D15	-0.36288	-0.24541	-0.12677	-0.26554	-0.23497	0.173989	-0.18443	0.36193
TBC1D16	-0.25171	0.491694	0.278802	0.298996	0.365146	0.095672	0.55511	0.391264
TBC1D17	0.113225	0.272097	-0.06949	-0.00576	0.299516	0.006101	0.289374	0.125921
TBC1D19	0.157946	-0.39449	0.042753	-0.29725	-0.07356	-0.15158	0.060427	-0.07813
TBC1D2	-0.39296	0.195434	0.269576	-0.19814	0.298684	0.169878	-0.08233	-0.2111
TBC1D20	0.052327	-0.17457	-0.15414	-0.24208	-0.08153	-0.33339	0.209736	0.270837
TBC1D21	-0.33659	0.389811	-0.26917	-0.47347	0.168387	0.173072	-0.22459	0.320291
TBC1D22A	-0.04248	0.405741	0.252169	0.191732	0.176107	0.264268	0.037018	-0.25217
TBC1D22B	0.000247	0.285065	-0.11377	-0.33034	-0.14934	-0.21991	0.511458	0.496807
TBC1D23	-0.16155	-0.40009	-0.15974	-0.28675	-0.33522	-0.17345	0.114901	0.204778
TBC1D24	NA	NA	-0.16095	0.001507	0.323009	-0.23832	0.527729	0.107185
TBC1D25	0.330503	0.565846	-0.01565	-0.05382	0.342196	0.08162	NA	NA
TBC1D26	NA	NA	0.173918	-0.01431	-0.24149	0.228414	NA	NA
TBC1D29	NA	NA	-0.08063	-0.0197	-0.06178	-0.19033	NA	NA
TBC1D2B	-0.18468	-0.23267	0.179131	-0.01239	0.053246	0.191003	-0.02467	0.315655
TBC1D3	-0.40113	-0.48225	-0.22636	0.044644	-0.05257	0.179436	-0.24661	0.134956
TBC1D30	NA	NA	-0.31587	0.116784	0.251023	-0.00723	NA	NA
TBC1D3B	NA	NA	0.044065	0.052436	-0.22782	0.081362	0.178118	0.452337
TBC1D3C	-0.38511	-0.17232	NA	NA	NA	NA	-0.44357	0.331368
TBC1D4	0.476581	-0.01654	0.056658	0.425178	0.144735	0.467082	0.515783	-0.09563
TBC1D5	0.117648	-0.16181	0.159632	0.240381	0.208153	0.307332	0.534203	0.146201
TBC1D7	0.074562	0.387055	0.179954	-0.10176	0.018977	-0.35258	0.276121	-0.43049
TBC1D8	-0.33934	-0.30056	0.03251	-0.19688	0.024838	-0.05871	NA	NA
TBC1D8B	-0.06186	-0.14102	0.04688	-0.08676	-0.08058	-0.26473	0.013526	-0.10212
TBC1D9	-0.28646	-0.33569	-0.01729	0.528228	-0.0142	0.220402	-0.46326	0.10981
TBC1D9B	0.3669	0.295619	0.192819	0.412863	0.118351	0.044396	-0.44312	-0.59789
TBCA	0.095656	-0.5772	0.275872	-0.15822	-0.06097	-0.19743	0.333174	-0.03474
TBCB	0.353849	0.282697	0.207855	0.188213	0.198376	0.186958	NA	NA
TBCC	0.451123	0.182338	-0.08359	0.015137	0.083553	-0.02863	0.594814	0.28668
TBCCD1	-0.55297	-0.29931	-0.09549	-0.0022	0.048806	0.068141	0.471417	0.087419
TBCD	-0.46166	-0.42005	0.069029	0.381526	0.413329	0.281869	0.188801	0.433073
TBCE	-0.23523	-0.5647	-0.0792	-0.08654	0.169791	-0.35017	0.204843	-0.04757
TBCEL	-0.01651	-0.07168	-0.27123	-0.22821	-0.13022	0.223113	NA	NA
TBK1	0.010658	-0.55037	-0.11965	-0.41394	-0.01063	0.242968	-0.03632	0.507735
TBKBP1	0.283347	-0.18092	0.287832	-0.30168	0.077245	-0.11711	-0.0615	-0.18315
TBL1X	-0.06092	0.475395	0.107163	-0.16952	0.0047	0.038095	-0.1649	-0.53165
TBL1XR1	-0.11694	-0.54632	0.085794	-0.00033	0.092416	0.020715	0.005986	-0.3218
TBL1Y	-0.09194	-0.08663	0.173554	-0.12182	-0.00035	-0.12856	0.381401	0.11226
TBL2	-0.02892	-0.66296	0.045008	0.440117	0.087379	0.108523	0.516234	0.333837
TBL3	0.336879	0.688204	0.191625	0.118159	0.218181	0.02905	0.569155	0.258478
TBP	0.382083	-0.18693	-0.48987	-0.01392	0.133353	-0.28919	0.685216	0.685086
TBPL1	0.310326	0.082717	-0.16347	-0.23009	-0.04516	-0.54807	0.117326	-0.41621
TBPL2	0.303116	0.1267	NA	NA	NA	NA	-0.26216	-0.25104
TBR1	0.077562	0.48817	-0.13483	-0.06159	0.026984	-0.01439	0.445557	0.036557
TBRG1	-0.35814	-0.48243	-0.25455	-0.02465	-0.07151	0.341799	-0.02645	0.177482
TBRG4	0.394794	0.063368	-0.08994	0.23341	0.18382	0.005444	0.465594	0.228153
TBX1	-0.40096	0.381148	0.140786	0.340021	-0.234	0.216342	0.262041	-0.12819
TBX10	0.336964	0.31175	0.191445	0.115815	-0.00645	-0.44206	0.113244	0.114963

TBX15	-0.23541	0.592836	-0.33768	-0.00497	0.157343	-0.55952	0.34403	0.081009
TBX18	NA	NA	0.247806	0.008991	-0.11011	-0.13948	-0.237	-0.20808
TBX19	0.242199	-0.1736	-0.326	-0.10524	-0.15762	-0.12604	0.206145	-0.41036
TBX2	-0.21295	0.273758	0.204857	0.031239	-0.04984	0.237958	-0.28882	-0.10683
TBX20	NA	NA	-0.20226	-0.0303	0.001038	0.138507	0.103995	-0.00676
TBX21	-0.32963	-0.27316	0.02407	0.308528	-0.30627	0.186079	-0.28381	-0.19501
TBX22	0.099757	0.717644	0.072127	-0.02443	-0.1894	-0.18984	0.134354	0.433193
TBX3	0.164384	-0.16919	0.003465	0.173473	0.208509	-0.1051	0.379307	0.561181
TBX4	0.222029	0.190456	-0.09899	-0.11469	-0.22069	0.047182	0.512894	-0.3134
TBX5	0.018391	0.495057	-0.0156	-0.18933	0.333889	-0.20611	0.394905	0.323805
TBX6	0.157824	0.777617	0.377385	-0.02746	0.276124	0.053238	0.300028	-0.24181
TBXA2R	-0.39402	-0.51383	-0.077	0.102701	-0.11214	0.024269	-0.66569	-0.08024
TBXAS1	0.173873	0.013048	-0.27431	0.284009	0.141731	0.100744	-0.20223	0.295537
TC2N	NA	NA	-0.2599	-0.16348	-0.1274	0.039704	NA	NA
TCAP	0.150417	-0.24916	-0.06488	0.152268	-0.13343	0.025152	-0.40961	-0.62067
TCEA1	-0.12926	-0.46043	0.147048	-0.24022	0.098538	-0.04309	0.374716	0.170695
TCEA2	0.082961	-0.06805	0.348185	0.078393	0.022885	0.493259	0.044989	-0.03147
TCEA3	-0.36143	-0.22267	-0.07857	0.138241	-0.09067	-0.22528	-0.21511	-0.25843
TCEAL1	0.320789	-0.37568	0.066896	-0.10271	-0.0046	-0.01492	-0.243	-0.38416
TCEAL2	0.204578	-0.1399	-0.06999	-0.02099	0.031269	0.107135	-0.19563	-0.27573
TCEAL3	-0.28347	-0.17437	0.133648	0.16638	-0.15299	0.100984	-0.23652	-0.18041
TCEAL4	0.356561	-0.2521	-0.26205	0.038832	0.060353	-0.02518	-0.14393	-0.09826
TCEAL5	0.166695	0.584453	0.047567	0.132002	-0.07787	-0.08703	-0.34729	-0.33895
TCEAL6	0.128844	0.329535	0.068382	0.16347	-0.18736	0.094844	0.042213	-0.14641
TCEAL7	0.480334	0.122542	-0.20729	-0.01367	-0.24335	-0.24655	-0.21162	0.010217
TCEAL8	0.188505	0.067921	-0.23886	-0.29116	-0.17364	-0.36157	-0.47337	-0.4439
TCEB1	-0.05751	-0.52153	0.014062	-0.4855	-0.10232	-0.19266	-0.32914	-0.41117
TCEB2	0.456635	0.560488	-0.12445	0.096409	-0.12582	-0.38687	0.066408	-0.31475
TCEB3	0.49646	-0.33253	-0.01295	0.169221	0.237574	0.278592	0.00275	-0.09456
TCEB3B	0.013758	0.441608	-0.22333	-0.02186	0.176547	0.080804	0.253476	-0.2151
TCEB3C	0.286003	0.789037	-0.05161	-0.17394	-0.15499	0.282235	0.290729	0.02569
TCERG1	0.005382	-0.58721	-0.0921	-0.02835	0.050429	0.184698	0.189645	0.181015
TCERG1L	0.135395	-0.46466	-0.29092	0.008913	0.134624	-0.29645	0.182272	0.228975
TCF12	0.264595	-0.58705	0.209941	-0.32328	-0.12435	0.011401	0.041253	0.116876
TCF15	-0.1278	-0.17925	-0.10573	-0.05565	-0.01874	0.006406	0.329903	-0.01378
TCF19	-0.15227	0.096947	-0.14134	0.354084	0.376578	-0.36554	-0.22926	0.05598
TCF20	0.062214	0.257288	0.110793	0.172336	0.362109	0.274788	0.363963	-0.16916
TCF21	0.193115	-0.15789	-0.01627	0.167621	-0.05607	0.042697	0.019348	0.084345
TCF23	0.11783	0.005939	-0.13144	0.067974	0.166621	-0.40718	0.160876	-0.04577
TCF25	-0.37378	0.086672	0.424821	0.187547	0.362797	-0.36349	NA	NA
TCF3	0.03173	-0.51571	-0.20428	0.511461	0.434804	0.150192	0.451951	0.386183
TCF4	-0.21545	-0.24989	0.159397	0.280721	-0.02455	0.59987	-0.38763	-0.37919
TCF7	1	1	1	1	1	1	1	1
TCF7L1	-0.122	-0.24801	-0.16783	0.031885	-0.05441	0.185103	-0.05687	-0.12763
TCF7L2	-0.13414	-0.08868	-0.30403	0.057794	-0.02347	-0.01604	0.606601	0.288939
TCFL5	0.309886	-0.52844	0.015409	-0.16377	0.371549	-0.13124	0.42291	-0.04833
TCHH	NA	NA	0.09181	0.077939	-0.15832	0.107489	NA	NA
TCHHL1	0.354867	0.164717	NA	NA	NA	NA	-0.32238	-0.28516
TCHP	-0.08902	0.085659	-0.04891	0.303679	0.40288	0.221198	-0.07726	0.336681
TCIRG1	-0.21938	-0.02949	0.334399	0.295142	0.089873	0.130854	-0.01214	0.467138
TCL1A	-0.09597	0.457869	0.106501	0.718451	-0.08814	0.387948	-0.3827	0.003692
TCL1B	0.363942	0.651083	0.037836	0.256136	-0.1569	0.081643	-0.34104	-0.15288

TCL6	0.217667	0.315679	0.247198	0.152232	0.080512	0.101232	0.344566	0.10858
TCN1	-0.11301	0.145359	-0.00417	0.056342	0.060739	-0.05911	0.012576	0.233619
TCN2	-0.18765	-0.04929	0.358614	-0.34969	-0.18149	-0.08457	-0.09299	0.195226
TCOF1	0.066714	0.067985	-0.06993	0.333386	0.130078	0.176097	-0.07904	-0.27044
TCP1	0.067744	-0.65044	-0.31417	0.180536	0.153696	-0.05294	-0.1244	-0.3972
TCP10	-0.27216	-0.16312	-0.36624	0.2003	0.101809	-0.03071	0.212052	-0.22509
TCP10L	-0.28829	0.685486	-0.48014	-0.09989	0.013945	-0.14341	0.212469	-0.05726
TCP11	-0.00407	-0.20431	-0.34563	-0.21598	0.241061	0.023142	0.010249	-0.37529
TCP11L1	0.056197	0.7183	0.102747	-0.45551	-0.28885	-0.1866	0.149425	0.432855
TCP11L2	-0.16916	0.073253	-0.1122	-0.35296	-0.17817	-0.50699	0.122133	-0.03785
TCTA	-0.05264	0.457032	0.041712	-0.14363	-0.13855	-0.46084	0.104587	0.378259
TCTE1	NA	NA	0.112064	-0.20794	0.190772	0.124578	NA	NA
TCTE3	0.102592	0.078126	-0.40072	-0.27859	0.013269	-0.23309	NA	NA
TCTEX1D1	-0.06237	0.196953	0.04989	-0.05452	-0.09525	0.018634	-0.39542	0.44836
TCTEX1D2	NA	NA	-0.4025	-0.16722	-0.19014	-0.21288	NA	NA
TCTN1	NA	NA	-0.04628	0.036818	0.348716	0.18642	NA	NA
TCTN2	NA	NA	0.22295	-0.06145	0.336085	0.163995	NA	NA
TCTN3	NA	NA	-0.00991	0.152661	-0.12022	-0.32805	NA	NA
TDG	-0.22053	0.193209	-0.1935	-0.23838	0.045532	-0.03402	NA	NA
TDGF1	0.042575	0.000218	-0.04903	0.129741	0.221127	0.05208	-0.16628	-0.12862
TDGF3	NA	NA	0.018071	0.101648	0.180886	0.130568	0.125257	-0.13026
TDH	NA	NA	-0.09972	0.128673	-0.2086	0.035376	-0.07014	0.109035
TDO2	-0.05246	0.020672	0.09698	0.460742	-0.1671	0.674424	-0.63965	-0.32261
TDP1	0.053656	0.63432	-0.34835	0.302039	-0.10796	-0.10679	-0.59169	-0.41098
TDRD1	-0.06601	0.102427	0.429936	0.119804	0.023694	-0.28238	-0.28911	-0.18093
TDRD10	0.014384	0.126133	-0.01935	0.036154	-0.0691	0.082827	-0.26881	0.54152
TDRD12	NA	NA	-0.63376	-0.02071	0.02487	-0.11567	NA	NA
TDRD3	0.399706	-0.3962	-0.12302	-0.21001	-0.03259	-0.38647	0.070098	-0.18274
TDRD5	0.199805	-0.02846	-0.51351	0.057215	-0.10361	-0.3379	-0.17691	-0.08745
TDRD6	0.277907	0.27608	-0.14237	-0.05	-0.0452	0.098105	-0.07731	0.075026
TDRD7	0.074501	-0.2235	0.085168	-0.45402	-0.03432	-0.24164	-0.24088	-0.04488
TDRD9	-0.429	-0.33124	-0.02774	-0.29687	-0.01252	-0.01862	-0.5533	-0.63351
TDRG1	NA	NA	-0.0313	0.067926	-0.04835	0.0074	NA	NA
TDRKH	-0.09191	-0.13549	-0.00331	0.245338	-0.0339	-0.19922	0.426465	0.181086
TEAD1	-0.07545	0.332301	0.196081	-0.32896	0.086773	0.142389	0.055928	0.142093
TEAD2	-0.02675	-0.12697	0.034517	-0.00197	0.176815	-0.07655	0.025125	-0.10848
TEAD3	-0.05828	-0.00676	0.096019	-0.16275	0.191915	0.112882	-0.66881	-0.5815
TEAD4	0.34792	0.00537	0.218935	0.141202	0.339804	0.190332	0.058136	-0.14431
TEC	-0.14298	0.133531	-0.02297	0.034905	-0.04541	-0.0512	-0.31093	0.211721
TECTA	-0.13447	0.026793	-0.35676	0.29373	-0.08386	0.252492	-0.39566	-0.43589
TECTB	0.139795	0.529098	0.138102	-0.09811	-0.12513	-0.07257	-0.11855	-0.45034
TEDDM1	-0.15552	0.170636	-0.10127	-0.07071	-0.13911	-0.2031	-0.08265	-0.20506
TEF	-0.17862	0.158095	0.405037	-0.40894	0.086561	-0.16178	-0.0678	-0.03341
TEK	0.062712	0.432529	-0.19585	0.143766	-0.10708	-0.02458	-0.12966	0.147911
TEKT1	-0.10517	0.417069	-0.12489	-0.17535	0.076948	0.055689	0.248013	-0.04559
TEKT2	0.303364	0.663395	-0.12494	-0.23267	-0.4591	-0.117	0.525397	0.506318
TEKT3	0.049918	0.277291	-0.42946	-0.41333	-0.08508	0.022275	-0.12238	0.433823
TEKT4	-0.20893	0.038009	0.065501	-0.09775	0.316966	-0.15913	-0.26699	-0.3285
TEKT5	-0.05294	0.194749	0.07399	-0.353	0.005734	-0.11324	NA	NA
TELO2	0.18698	0.730717	0.05695	0.106893	0.418471	0.416209	NA	NA
TENC1	-0.23626	-0.22021	0.322787	0.038732	0.132315	0.389411	-0.35907	-0.15079
TEP1	-0.39075	-0.1759	0.001874	-0.08259	-0.00079	0.128327	-0.46704	0.328284

TEPP	-0.11856	-0.06119	0.334757	-0.24232	0.117135	-0.06024	-0.13761	-0.07825
TERF1	0.048548	0.157873	-0.00833	-0.06357	-0.39879	-0.19981	-0.16253	-0.4774
TERF2	0.151935	-0.27102	-0.01257	0.477406	0.135368	-0.07909	0.551981	0.405316
TERF2IP	-0.3233	-0.35535	0.177809	0.345347	0.409656	0.245653	0.465406	0.275792
TERT	0.091257	0.178013	-0.4898	0.011408	0.340355	-0.11453	0.058218	0.100761
TES	0.035484	-0.23996	0.205771	-0.13681	-0.05205	0.035363	0.273387	-0.15933
TESC	0.114903	-0.00376	0.473435	0.489108	0.126062	0.221944	0.328342	-0.04913
TESK1	-0.09962	0.143101	0.295194	-0.15582	0.130562	0.021068	0.170671	0.228342
TESK2	0.001054	-0.13879	0.033214	-0.17633	-0.06573	-0.26209	0.0824	0.179603
TESSP2	-0.08973	0.378346	-0.05141	-0.08667	0.031738	-0.10082	-0.07154	0.042676
TET1	NA	NA	-0.31064	0.090511	-0.24977	0.098563	NA	NA
TET2	NA	NA	-0.08384	-0.35784	-0.33415	0.145525	NA	NA
TET3	NA	NA	-0.20063	0.079272	0.026769	0.141593	NA	NA
TEX10	0.318014	-0.40327	0.043438	-0.03954	-0.02847	0.202751	0.465385	0.391447
TEX101	0.438058	-0.16581	0.015228	-0.11207	0.212844	-0.12068	-0.10572	-0.11604
TEX11	-0.06417	0.031387	-0.04814	-0.30051	-0.24896	-0.3144	-0.24622	-0.41314
TEX12	-0.08585	0.267207	0.194643	-0.1245	0.078253	-0.10621	-0.06318	0.416527
TEX13A	0.35006	0.716259	-0.14185	-0.34541	-0.10347	-0.08003	-0.11733	-0.18956
TEX13B	-0.14572	-0.2009	0.178777	-0.27502	0.020086	-0.13483	0.474087	0.130661
TEX14	0.109569	0.201795	-0.25984	0.19983	0.013457	-0.56038	-0.23248	-0.4027
TEX15	-0.16014	-0.12478	-0.08951	-0.18457	-0.33794	-0.28569	-0.42705	-0.2284
TEX2	0.047475	-0.14378	0.288452	-0.05547	0.123168	-0.11783	0.126104	-0.13827
TEX261	0.058432	0.474231	0.255375	0.072011	0.036438	-0.16269	0.575894	0.561333
TEX264	0.194626	0.682756	0.285885	-0.01943	0.109027	-0.28563	-0.23723	-0.18098
TEX28	NA	NA	0.117595	0.079129	-0.25199	-0.38428	NA	NA
TEX9	0.122412	-0.17666	NA	NA	NA	NA	0.279983	0.20011
TF	0.115515	0.239035	0.077145	0.247976	-0.2936	0.228467	0.364883	-0.13331
TFAM	0.490797	0.311305	-0.46044	0.037078	-0.09327	-0.09164	0.406469	0.216257
TFAP2A	-0.43655	-0.28849	0.137089	-0.17809	-0.09987	-0.00494	-0.28404	-0.67632
TFAP2B	0.182824	-0.04138	-0.18141	-0.12711	0.151792	-0.16971	0.093719	0.273808
TFAP2C	-0.46818	-0.35833	0.036235	-0.04545	-0.21291	-0.12177	-0.37582	-0.251
TFAP2D	-0.09311	-0.1066	-0.12651	-0.07111	0.38929	-0.2268	0.501162	0.285539
TFAP2E	-0.20469	0.060861	0.031458	0.135907	-0.12275	0.480968	-0.31025	-0.3695
TFAP4	0.552659	0.48226	-0.08032	0.334117	0.420663	-0.14239	0.534992	0.05502
TFB1M	0.596928	0.164475	0.287332	-0.15942	0.006384	-0.10173	0.595025	0.145108
TFB2M	0.017218	-0.56286	-0.28832	-0.08721	-0.17955	0.085559	0.404652	-0.05031
TFCP2	0.143204	-0.53184	-0.38058	0.256432	0.123454	-0.21565	0.495914	0.38427
TFCP2L1	0.201133	0.108071	-0.34722	-0.11871	0.258162	-0.23046	0.32608	-0.27341
TFDP1	0.02475	-0.63404	-0.03718	0.252739	0.306951	-0.077	0.450464	0.088262
TFDP2	0.042573	-0.28836	0.097785	-0.2946	-0.01951	-0.63127	-0.00914	0.303006
TFDP3	0.267657	0.281743	-0.31253	0.101588	0.175401	-0.01379	0.176877	0.052562
TFE3	0.190834	0.375162	0.192894	0.032292	0.066829	-0.10303	-0.21123	0.129826
TFEB	0.011915	-0.14178	0.521342	0.083954	0.365199	0.379392	0.44396	0.261338
TFEC	-0.1419	0.485813	0.127069	0.240207	-0.43654	0.39692	0.101176	-0.12382
TFF1	-0.50869	0.083452	-0.36869	0.058561	-0.03302	-0.34602	-0.02927	-0.09544
TFF2	-0.61178	0.051116	-0.12275	0.136001	-0.03307	-0.45486	-0.05599	-0.22846
TFF3	-0.28559	0.201362	-0.03053	0.248074	-0.08033	-0.26155	0.213949	0.111735
TFG	-0.19519	-0.34181	-0.20195	0.178593	-0.11498	0.01256	-0.17669	-0.26381
TFIP11	0.168451	-0.39223	0.25542	0.153398	0.217881	0.292323	0.526031	0.308662
TFPI	-0.38714	-0.33863	0.406453	-0.22117	-0.15204	-0.13088	-0.09281	0.213458
TFPI2	0.367486	0.030596	-0.05266	-0.01822	0.173001	-0.11436	-0.11795	0.158988
TFPT	0.406644	0.599573	-0.06069	0.158173	-0.06923	0.227292	0.263148	0.118672

TFR2	0.223943	0.171777	0.084395	-0.2087	0.210243	-0.36248	0.260422	0.080594
TFRC	-0.01566	0.079572	-0.09083	0.102062	0.090555	0.093603	0.21167	-0.10298
TG	0.31547	0.031009	0.385012	0.388754	-0.03379	-0.35105	0.444847	0.525578
TGDS	0.316931	-0.6368	-0.06909	-0.05791	0.166724	-0.15055	0.451456	0.479267
TGFA	-0.18645	-0.07363	0.228325	-0.11542	0.166556	-0.2005	0.039591	-0.03113
TGFB1	-0.24979	0.64313	0.24485	0.407964	-0.14168	0.505226	-0.06572	0.169913
TGFB1I1	-0.19986	-0.01609	0.278898	-0.04295	0.140934	0.0836	-0.3208	-0.33133
TGFB2	-0.47072	-0.13647	0.022783	-0.1567	-0.14418	-0.0487	0.057515	-0.06737
TGFB3	-0.22919	-0.13871	-0.02841	0.020786	-0.23975	-0.0556	-0.06918	0.079156
TGFBI	0.097682	-0.24475	0.073231	-0.34714	0.276284	0.12416	0.474746	0.299723
TGFBR1	0.043647	0.332508	-0.00226	-0.41878	-0.09828	-0.05469	-0.50585	-0.14333
TGFBR2	-0.17539	-0.57116	0.116922	0.086615	-0.00978	0.522561	-0.14108	0.016415
TGFBR3	0.054261	-0.14179	0.357647	-0.03888	0.244005	0.377437	-0.13212	-0.20482
TGFBRAP1	0.455589	0.260008	0.315393	-0.02422	0.287524	0.544298	0.048977	-0.27981
TGIF1	0.16889	0.088592	-0.11525	0.048246	0.129651	-0.11118	0.467277	0.111407
TGIF2	0.366093	0.096238	0.096129	0.43682	0.417512	0.565712	0.063379	-0.31397
TGIF2LY	-0.30657	0.133432	0.099317	-0.34422	-0.02514	-0.02929	NA	NA
TGM1	-0.01275	-0.22621	-0.47142	0.056437	-0.06248	-0.18233	-0.37935	0.039496
TGM2	0.475533	0.091912	0.415363	0.178114	0.343394	0.355376	-0.16336	0.180864
TGM3	-0.17144	0.023781	0.191597	-0.09211	0.013545	-0.64879	-0.11139	-0.08697
TGM4	0.003438	-0.1344	0.032945	-0.09038	-0.27891	0.005625	-0.08188	0.040544
TGM5	0.015066	0.29907	0.1046	-0.13673	0.028155	0.02398	0.490727	0.460953
TGM6	-0.00389	0.487595	NA	NA	NA	NA	0.13799	-0.04142
TGM7	0.447912	0.178017	-0.13299	-0.042	-0.35022	-0.25116	-0.31768	-0.12802
TGOLN2	0.282802	0.151799	0.361667	-0.29434	0.363425	0.125041	-0.04231	-0.13732
TGS1	-0.07349	-0.09416	0.174471	0.009363	-0.04998	0.332055	NA	NA
TH	0.308955	0.165178	-0.20155	-0.37814	0.13406	-0.14842	0.244745	-0.11195
TH1L	0.440345	0.188932	0.154969	0.336961	0.326743	0.162374	0.467503	0.062934
THADA	0.523842	-0.17366	0.025887	0.418486	0.156064	0.092457	0.46612	0.20694
THAP1	-0.09679	-0.30518	0.179161	-0.3556	-0.0115	-0.36404	0.308305	0.0439
THAP10	0.625727	-0.2719	-0.44626	-0.01314	-0.05385	-0.45491	0.121537	-0.15165
THAP11	0.222566	-0.18479	0.102408	0.295112	0.359015	0.092672	-0.02337	-0.25522
THAP2	-0.01602	-0.10279	-0.30634	-0.05524	-0.42974	-0.4632	0.403274	0.053766
THAP3	0.280701	0.112166	0.140848	0.033841	-0.01112	-0.13817	-0.15807	-0.39597
THAP4	NA	NA	0.405937	-0.25522	0.183636	0.274199	0.437601	0.235512
THAP5	-0.3604	0.376485	-0.30024	-0.20956	-0.21846	-0.30868	-0.36281	-0.25326
THAP6	0.412248	-0.43712	0.001101	-0.28876	-0.1061	-0.39521	0.412046	0.253589
THAP7	0.119909	0.293442	0.00747	0.264822	0.090355	-0.08736	0.282518	0.116768
THAP8	0.006902	-0.19857	0.27738	0.048602	-0.18944	-0.28991	0.036276	-0.09128
THAP9	-0.23169	-0.58238	-0.33384	-0.14492	-0.14812	-0.12486	-0.23817	-0.09241
THBD	-0.41696	-0.2027	0.001473	-0.02701	-0.07123	0.286752	-0.35044	0.285382
THBS1	-0.25908	-0.36956	-0.04155	-0.33377	-0.09147	0.127766	-0.13532	0.112269
THBS2	-0.22908	-0.33473	0.038331	-0.12166	0.00372	0.019983	-0.05036	-0.04517
THBS3	-0.06691	0.007079	0.210653	-0.13531	-0.10186	0.270241	-0.60179	-0.56108
THBS4	0.091803	-0.14276	0.202676	-0.17068	0.149539	-0.06747	-0.17059	-0.3345
THEG	-0.21426	0.522432	-0.28399	0.198763	0.191085	-0.3699	0.566656	0.503044
THEM2	-0.0103	-0.22468	-0.43778	-0.21379	-0.2224	-0.49372	0.364201	-0.18529
THEM4	-0.31354	-0.02687	-0.01212	0.110962	-0.26367	0.024487	0.153483	-0.0621
THEM5	0.024299	0.187776	0.154047	-0.18232	-0.25473	0.14905	-0.095	0.377471
THG1L	NA	NA	0.425578	0.281164	-0.09871	-0.29942	0.170316	-0.08731
THNSL1	-0.07474	-0.2352	-0.0385	0.194357	0.21019	0.115997	0.435555	0.390252
THNSL2	NA	NA	0.150132	-0.25343	0.250606	0.232318	NA	NA

THOC1	0.061373	-0.42754	-0.37049	0.080517	-0.18731	-0.03124	-0.50998	-0.04159
THOC2	0.277628	0.454527	-0.26433	-0.05073	0.183353	0.136116	-0.55591	-0.47721
THOC3	NA	NA	0.049065	0.199302	0.186181	0.110141	0.466938	0.099203
THOC4	-0.26105	-0.50212	0.135857	0.361666	0.393279	0.027285	0.080854	-0.1382
THOC5	0.118027	0.146969	0.319197	0.083897	0.274033	-0.03642	0.527724	0.250302
THOC6	0.376355	0.245464	-0.1007	0.245205	0.090989	0.043656	NA	NA
THOC7	0.378137	-0.32101	-0.18459	-0.15743	0.140862	-0.29496	NA	NA
THOP1	-0.22327	0.331692	-0.23671	0.104817	0.175252	-0.34635	0.370795	0.37968
THPO	0.037168	-0.00108	0.058996	-0.20811	-0.25398	-0.38447	-0.50242	0.086322
THRA	0.176795	-0.0799	0.621505	-0.04284	0.35209	-0.05195	-0.10716	0.015838
THRAP3	-0.36034	-0.6567	-0.07014	0.16348	0.03105	0.349478	-0.22123	-0.42085
THRB	-0.00641	0.680507	0.12929	-0.27807	-0.13307	-0.17307	0.307015	0.361797
THRSP	-0.00228	-0.1641	0.087295	-0.15271	0.249036	-0.32528	0.234018	0.154003
THSD1	-0.178	-0.14969	-0.01052	-0.2668	0.332772	0.206183	0.005631	0.111694
THSD4	0.313043	0.270584	0.064554	-0.08799	0.012121	0.009823	0.351036	0.069671
THSD7A	NA	NA	0.025812	-0.10835	0.049201	0.128561	NA	NA
THSD7B	NA	NA	0.24813	0.010917	-0.25542	-0.20337	-0.17464	-0.15112
THTPA	0.316786	-0.21704	0.149339	0.245884	-0.00079	-0.2949	0.044017	0.346738
THUMPD1	-0.11264	-0.61246	-0.47412	-0.21345	-0.09813	-0.33078	0.017852	-0.27902
THUMPD2	0.317232	-0.55582	-0.01715	-0.31977	0.072007	0.045498	0.03	0.016313
THUMPD3	0.163749	-0.4908	-0.18786	-0.09458	0.011312	0.042051	-0.1076	-0.20982
THY1	-0.19572	-0.11009	0.190426	0.173393	-0.05067	0.238347	-0.11453	0.058685
THYN1	0.18054	-0.01183	-0.17854	-0.04693	-0.13687	-0.25448	0.021173	0.022925
TIA1	-0.35097	-0.66887	-0.31976	0.089133	-0.03576	-0.29045	0.514094	0.414729
TIAF1	-0.11314	0.323991	NA	NA	NA	NA	-0.4381	0.056369
TIAL1	-0.31283	-0.55829	-0.3267	0.177968	-0.03064	0.332693	-0.1238	-0.10253
TIAM1	-0.08429	-0.32198	0.319764	-0.19788	0.017743	0.029436	0.009192	0.168856
TIAM2	0.061431	0.230811	-0.04998	0.347586	-0.03177	0.394966	0.285151	0.453176
TICAM1	0.112658	0.150161	0.339415	-0.27668	0.125172	0.058526	-0.32703	-0.06715
TICAM2	-0.17234	0.313356	0.096575	0.082322	-0.35956	-0.28286	-0.46769	0.097808
TIE1	-0.25896	0.216951	0.150975	0.267493	0.013885	0.233457	-0.43826	-0.35193
TIFA	-0.14443	0.102271	0.016724	0.303347	0.001591	0.062698	-0.05823	0.092963
TIGD1	0.374042	-0.29545	0.143455	-0.17362	0.002896	-0.09626	0.104492	0.10754
TIGD2	0.220833	-0.24779	-0.33531	-0.15215	-0.05599	-0.25286	0.371497	0.560144
TIGD3	-0.00321	0.523998	0.108474	0.459368	0.218953	-0.21475	-0.01554	0.106222
TIGD4	0.257726	0.44647	-0.06441	0.249167	-0.14972	-0.38018	-0.15942	-0.44196
TIGD5	0.086836	0.680427	0.383539	-0.07184	0.129416	0.215835	0.279681	-0.16615
TIGD6	0.549063	0.788986	0.549478	-0.2168	-0.0483	-0.23654	0.578012	0.496168
TIGD7	0.102058	0.050446	-0.21714	0.055236	-0.21061	-0.06167	-0.29027	0.109675
TIGIT	NA	NA	0.287673	0.700804	-0.37009	0.459815	NA	NA
TIMD4	0.077228	-0.13948	-0.09626	0.689606	-0.39167	0.145857	-0.20478	0.200316
TIMELESS	-0.33098	-0.03203	-0.45433	0.393802	0.25	-0.09563	0.110313	0.138148
TIMM10	0.065017	0.278405	-0.04338	-0.1822	-0.28946	-0.06587	0.182597	0.211644
TIMM13	-0.05175	0.718917	-0.21581	0.16553	0.060738	-0.16453	0.137363	0.084427
TIMM17A	0.034229	-0.07249	-0.23533	-0.09495	-0.11737	-0.12517	0.036769	-0.02643
TIMM17B	0.420747	0.731359	0.033944	-0.27965	0.018607	-0.16359	0.24343	-0.00866
TIMM22	-0.22496	0.333795	-0.07685	0.025134	-0.37235	-0.13249	-0.20033	-0.14643
TIMM23	0.167929	0.031379	-0.26079	0.020112	-0.12025	0.021631	0.083023	-0.14746
TIMM44	0.197438	0.05529	0.011383	0.053561	0.242795	0.10051	0.085428	0.100185
TIMM50	-0.32631	-0.34384	-0.0742	0.108556	-0.2002	-0.14461	-0.61412	-0.27355
TIMM8A	0.45537	0.376433	-0.02688	-0.09122	-0.16057	-0.00367	-0.05437	-0.45245
TIMM8B	-0.0403	0.643471	-0.17689	-0.2688	-0.34505	-0.2195	-0.52335	-0.453

TIMM9	0.227634	-0.25675	-0.27502	-0.30443	-0.33227	0.01986	-0.36897	-0.59565
TIMP1	-0.10061	-0.06601	0.239518	0.211719	0.020781	-0.02916	-0.07405	0.085526
TIMP2	-0.32104	-0.40917	0.147236	-0.36133	-0.18052	-0.14146	-0.16965	-0.0361
TIMP3	-0.00883	-0.26667	0.029998	0.074621	0.097629	0.172499	0.20883	-0.07506
TIMP4	0.00964	-0.02292	0.056902	-0.13258	-0.1045	-0.37207	0.094224	0.164651
TINAG	0.324107	0.049412	-0.26146	-0.21291	-0.0148	-0.25779	0.112484	-0.14328
TINAGL1	-0.01363	-0.10187	0.31646	-0.17395	0.445802	-0.29503	-0.04377	0.098827
TINF2	0.060776	0.224156	-0.11066	0.336609	-0.1697	0.102266	-0.24212	-0.09762
TINP1	0.309763	-0.58164	-0.05562	0.189459	-0.02558	-0.03101	0.455068	0.114779
TIPARP	-0.27411	0.056456	-0.12756	-0.28741	0.076713	-0.21707	-0.00203	0.084453
TIPIN	-0.06361	-0.45651	-0.3126	-0.03349	0.076168	-0.09857	NA	NA
TIPRL	-0.26023	-0.60102	-0.34314	-0.23111	-0.35925	-0.41426	0.300624	-0.03737
TIRAP	-0.14137	0.481514	0.368368	-0.14856	0.020686	0.073362	-0.32009	0.037018
TJAP1	0.144693	0.241517	0.100714	-0.0405	0.169132	0.321786	-0.25788	-0.23685
TJP1	-0.12259	-0.68896	-0.27936	-0.56691	-0.1504	0.065291	0.441948	0.170914
TJP2	-0.07958	-0.40416	-0.09226	-0.00842	0.298638	0.224206	0.545214	0.367376
TJP3	0.247396	0.192307	0.251373	-0.0156	0.351119	-0.01019	0.395595	0.114746
TK1	-0.1179	0.273946	-0.14856	0.297207	0.204068	-0.04709	0.498037	0.194369
TK2	-0.05435	-0.14641	0.248458	-0.20857	0.220244	-0.15816	0.109296	0.631751
TKT	-0.04611	-0.27149	-0.04374	0.41718	0.256828	0.390759	0.524841	0.264054
TKTL1	-0.01086	0.20862	-0.04682	-0.11469	0.032109	0.069687	-0.20504	0.00285
TKTL2	0.346279	0.165044	-0.30647	-0.41267	0.266312	-0.28861	0.247848	-0.04341
TLCD1	0.363945	0.537558	0.069667	0.194214	-0.07215	-0.13403	0.388375	0.076295
TLE1	-0.36595	-0.36758	-0.38761	-0.11134	-0.14585	0.342026	-0.3866	-0.59877
TLE2	0.289772	0.789204	0.013257	-0.1046	0.035591	0.300372	0.370575	-0.0549
TLE3	-0.48235	-0.44029	0.089724	0.156426	0.193601	0.327463	0.38103	0.310517
TLE4	-0.30909	-0.61319	0.043554	-0.00472	-0.37492	0.077067	0.16415	0.254693
TLE6	-0.2542	0.677829	-0.14192	-0.21036	-0.10185	-0.38436	-0.40557	-0.62047
TLK1	0.430568	0.573193	-0.32092	0.116593	-0.05669	-0.15127	-0.34173	-0.51017
TLK2	0.365598	0.507847	-0.05871	-0.35275	-0.35307	-0.30773	0.475715	0.156671
TLL1	-0.15321	-0.33157	0.203565	-0.00835	0.106752	-0.19591	-0.07242	0.307863
TLL2	0.304165	0.02099	-0.13899	0.342913	-0.14236	-0.25008	0.021966	0.73613
TLN1	0.062853	0.200619	0.106369	0.061611	0.108349	0.338877	-0.57486	-0.58944
TLN2	0.033314	0.240343	-0.19846	-0.04438	-0.41116	0.275755	0.283164	0.255773
TLR1	-0.50688	-0.05638	0.14473	0.018655	-0.27102	0.183817	-0.53017	0.006069
TLR10	-0.2834	0.215502	-0.01113	0.697088	-0.13547	0.734761	-0.51052	0.051851
TLR2	-0.4702	-0.22903	0.07484	-0.08065	-0.1518	0.322135	-0.17477	0.114151
TLR3	-0.08116	-0.33002	0.103517	-0.37892	-0.30246	-0.31581	0.270301	0.406623
TLR4	-0.39662	0.345103	0.259334	-0.23119	-0.08484	-0.04475	-0.39388	0.184273
TLR5	-0.292	-0.42009	0.139063	-0.25216	-0.2717	-0.02335	-0.19554	-0.01401
TLR6	-0.0998	0.473086	0.176137	0.229872	-0.22809	0.46718	-0.0043	0.399571
TLR7	-0.3593	-0.04952	0.147006	-0.16361	-0.22552	-0.00769	-0.60001	0.136459
TLR8	-0.09581	0.126242	0.173716	-0.15058	-0.27589	0.104869	-0.32711	0.271469
TLR9	0.013705	0.766853	0.37437	0.830434	0.018618	0.443442	0.111478	0.050047
TLX1	0.24764	-0.00466	-0.1935	-0.19384	0.103656	-0.32285	-0.0656	0.022202
TLX2	0.262541	0.475013	-0.00081	-0.10178	-0.0741	-0.08833	0.274924	-0.25054
TLX3	0.157035	0.767617	0.086456	-0.08689	-0.05309	-0.11696	-0.54497	-0.04021
TM2D1	-0.23035	-0.65646	0.34141	-0.28178	0.097547	-0.26556	-0.25077	-0.27459
TM2D2	0.124259	-0.46726	-0.04404	-0.03694	-0.35748	-0.15066	0.425346	0.440436
TM2D3	-0.22947	-0.70806	0.040888	0.253997	-0.02843	-0.08532	0.024763	0.136672
TM4SF1	0.073713	0.039517	-0.16865	-0.12119	-0.03104	0.16536	0.223913	-0.08526
TM4SF18	-0.34496	-0.46737	-0.05756	0.130334	-0.07332	0.13908	-0.16121	0.102346

TM4SF19	-0.06248	0.559484	-0.05724	0.200696	-0.28248	0.015159	0.069875	0.128127
TM4SF20	0.060515	-0.03062	0.467942	-0.31802	0.054643	0.01764	0.041951	0.498607
TM4SF4	0.097704	0.030017	-0.14062	-0.24626	-0.00242	-0.2663	-0.00038	0.237368
TM4SF5	0.21019	0.108396	0.045834	0.213171	5.56E-05	-0.39284	0.315454	0.30035
TM6SF1	0.251985	0.291743	0.208915	-0.24302	-0.25502	-0.37361	-0.01089	0.305655
TM6SF2	0.151354	0.32057	0.047379	-0.47542	0.015829	-0.38041	0.316781	0.418783
TM7SF2	0.145753	0.399403	0.069136	0.094596	0.022118	-0.31195	0.472554	0.338299
TM7SF3	0.2971	-0.02426	0.1055	0.003941	-0.2612	-0.01388	0.000768	-0.34839
TM7SF4	-0.01644	0.105576	0.260914	-0.12531	-0.15852	0.191087	-0.00604	0.005083
TM9SF1	-0.1278	-0.35885	0.03616	0.139932	-0.02895	-0.22369	0.618376	0.534105
TM9SF2	0.067253	-0.2054	0.082307	0.057165	0.222433	-0.1611	0.372619	0.194114
TM9SF3	-0.20921	0.223146	0.065822	-0.12631	0.166444	-0.13629	NA	NA
TM9SF4	0.488354	-0.22405	0.202172	0.562599	0.08839	-0.21398	0.444931	0.371861
TMBIM1	-0.19233	0.251026	0.272017	0.025436	0.149315	0.185588	-0.48802	-0.38384
TMBIM4	-0.2554	-0.39516	0.04598	-0.29823	-0.2038	-0.14831	-0.19051	0.21938
TMBIM6	NA	NA	0.013659	0.081952	0.149799	0.000297	NA	NA
TMC1	0.15385	0.333068	0.015448	0.14169	-0.01981	-0.05795	NA	NA
TMC2	-0.25376	-0.25895	-0.00951	-0.1224	-0.16115	-0.09888	0.119556	0.283343
TMC3	NA	NA	-0.01619	-0.11375	0.168791	-0.15106	NA	NA
TMC4	0.383953	0.246462	0.249851	0.17928	0.1514	0.108399	0.412814	-0.00936
TMC5	0.126537	-0.01101	0.068974	0.018619	0.375307	-0.10561	0.118639	0.256928
TMC6	0.117275	0.310544	0.314246	0.499533	0.03546	0.30349	0.275819	0.158161
TMC7	0.024293	-0.00591	-0.16478	0.082638	0.266856	0.032562	0.530894	0.383375
TMC8	-0.26074	-0.19977	0.020324	0.753798	-0.15832	0.779522	0.073855	0.37399
TMCC1	0.079076	0.079806	-0.11144	-0.18497	-0.10506	-0.39293	-0.32954	-0.45885
TMCC2	-0.21435	0.215014	0.287159	0.023277	0.144102	0.286396	-0.36843	-0.47714
TMCC3	-0.04917	0.25227	-0.19417	-0.43264	-0.37547	-0.32677	-0.50024	-0.40625
TMCO1	-0.14495	-0.25469	-0.08761	-0.34924	-0.04181	-0.18207	0.236302	-0.07211
TMCO2	-0.15165	0.314882	-0.05172	-0.34226	0.411427	-0.22925	-0.24533	-0.23961
TMCO3	-0.41872	-0.78072	0.048385	-0.08158	0.176257	-0.30414	0.556742	0.407822
TMCO4	-0.14421	-0.54164	0.15609	0.111052	0.200006	0.278912	0.192564	-0.36976
TMCO5A	NA	NA	0.019045	0.159303	0.119061	0.12633	NA	NA
TMCO6	NA	NA	0.334875	-0.00385	0.093144	0.327876	0.154948	0.181838
TMCO7	NA	NA	0.147051	0.384544	0.086819	0.004067	NA	NA
TMED1	0.071993	0.295548	0.172672	0.218585	0.055607	-0.02394	-0.34155	-0.49659
TMED10	-0.30273	-0.49355	-0.16422	0.145107	-0.32995	-0.3445	0.170166	0.223568
TMED2	-0.49162	-0.75028	0.089487	0.17076	-0.04268	-0.0711	-0.04806	-0.24625
TMED3	-0.20237	0.328652	0.179327	0.447289	-0.04767	-0.19393	0.043932	-0.31653
TMED4	0.415504	0.644802	0.065673	-0.151	0.048965	-0.02787	0.556802	0.561253
TMED5	-0.07953	-0.435	0.078572	-0.09863	0.092236	-0.20874	0.147842	0.007168
TMED6	-0.1561	0.046996	0.198925	-0.15501	0.007257	-0.15479	-0.00935	0.105387
TMED7	-0.1728	-0.66978	0.18226	0.067516	-0.3517	-0.20655	-0.11445	0.048139
TMED8	0.013397	0.62737	-0.25795	0.066922	-0.14185	0.235261	0.123573	0.515813
TMED9	-0.4984	-0.34607	0.405387	0.31994	0.14313	-0.10219	0.387491	0.080704
TMEFF1	0.138791	-0.0699	-0.37792	-0.02122	-0.31647	-0.10098	-0.31113	-0.50175
TMEFF2	-0.01943	-0.08626	-0.03346	-0.14094	0.109738	0.050861	0.207865	0.020166
TMEM100	0.077259	-0.43144	0.081534	-0.26308	-0.25141	-0.4972	-0.24482	-0.12305
TMEM101	0.009967	0.297933	0.294829	0.314253	0.105391	-0.34822	0.627087	0.13241
TMEM102	0.216061	0.539925	0.215603	-0.05419	-0.02618	0.060834	-0.28369	-0.16682
TMEM104	0.140722	-0.33277	0.282145	0.107287	0.148332	0.09717	0.518724	0.504056
TMEM105	-0.12507	0.040931	-0.29751	-0.06509	-0.08478	0.12297	0.347493	0.4188
TMEM106A	-0.2699	-0.04072	0.09445	0.23583	-0.27809	0.110533	NA	NA

TMEM106E	0.125818	-0.2248	0.057532	-0.47881	-0.16204	-0.46574	-0.25526	-0.41701
TMEM106C	-0.09689	0.451665	-0.22316	-0.08872	0.15651	-0.40133	-0.33384	-0.33059
TMEM107	-0.31451	-0.44649	-0.24409	-0.04534	-0.1975	-0.3396	0.116945	-0.13704
TMEM108	0.044465	-0.01413	0.058379	0.306426	-0.07752	-0.3234	-0.43231	-0.6089
TMEM109	-0.22749	-0.1814	-0.11938	0.27473	0.15802	0.182744	0.145115	-0.03042
TMEM11	0.011601	0.584682	-0.10033	-0.05571	-0.18961	-0.28081	-0.06131	-0.28239
TMEM110	0.145018	-0.1188	-0.02316	0.183234	-0.05938	-0.12109	-0.1518	0.086779
TMEM111	-0.27621	-0.47504	-0.08336	-0.23921	-0.15065	-0.40492	NA	NA
TMEM114	NA	NA	0.182779	-0.24043	-0.00012	-0.15245	NA	NA
TMEM115	0.137672	0.670287	0.307639	-0.07715	0.252058	-0.1872	-0.1993	-0.13626
TMEM116	-0.23577	-0.45818	-0.01289	-0.15272	-0.08526	-0.23219	0.382079	0.422322
TMEM117	0.201669	-0.01718	-0.11674	-0.27773	-0.1174	-0.24499	-0.4313	-0.3486
TMEM119	-0.09003	-0.17274	0.16737	0.24948	0.115011	0.167895	0.120489	0.449084
TMEM120A	NA	NA	0.387553	-0.33225	-0.0399	-0.4346	NA	NA
TMEM120E	NA	NA	-0.1369	0.010762	0.189834	0.222837	NA	NA
TMEM121	0.020946	-0.13184	0.007998	0.268658	-0.11812	0.034066	0.353637	0.026249
TMEM123	-0.1076	-0.64405	-0.16544	0.016138	0.051778	0.22527	0.726325	0.103734
TMEM125	-0.31384	0.234201	0.072441	0.294813	0.042242	-0.36241	0.354308	0.113943
TMEM126A	0.101919	-0.01792	0.182371	-0.36383	-0.10545	-0.27683	0.315092	0.171912
TMEM126E	0.05151	-0.62376	-0.16582	-0.40464	-0.1823	-0.04286	-0.13279	-0.14254
TMEM127	0.127696	0.653447	0.168129	-0.33765	0.184075	-0.09426	-0.31358	0.013099
TMEM128	-0.00251	0.10461	0.06435	-0.39129	-0.17445	-0.29909	-0.22501	-0.27202
TMEM129	0.000644	-0.24814	-0.21775	0.281617	-0.01022	-0.19	-0.33274	0.004014
TMEM130	0.36114	0.150327	-0.07426	0.063567	-0.19362	0.059851	-0.16855	-0.14074
TMEM131	NA	NA	0.042897	0.020607	0.097408	-0.11274	NA	NA
TMEM132A	NA	NA	-0.14565	0.307244	0.093879	0.213464	0.089055	0.009598
TMEM132E	NA	NA	-0.04701	-0.48297	0.146472	-0.319	NA	NA
TMEM132C	NA	NA	-0.01358	0.422494	0.070773	0.350086	NA	NA
TMEM132D	0.19119	0.502026	0.175566	0.231974	0.325215	-0.11181	NA	NA
TMEM132E	0.110458	0.35391	NA	NA	NA	NA	NA	NA
TMEM133	-0.24617	-0.51751	-0.21744	-0.39086	-0.23903	-0.21126	NA	NA
TMEM134	-0.45095	0.632015	0.377397	0.150727	0.185806	0.087484	-0.09392	-0.02853
TMEM135	0.055867	-0.1934	-0.21744	-0.26903	-0.27946	-0.47177	NA	NA
TMEM136	-0.17865	-0.38562	0.308835	0.059321	-0.12319	-0.30916	NA	NA
TMEM138	-0.1581	0.584587	-0.05335	-0.01496	-0.12186	0.297587	NA	NA
TMEM139	0.369143	0.497303	0.111147	-0.01569	-0.145	-0.22885	NA	NA
TMEM140	-0.41394	-0.14567	0.337994	-0.38993	-0.20039	-0.13984	NA	NA
TMEM141	-0.50963	0.343567	-0.16265	-0.07728	-0.07021	-0.28804	NA	NA
TMEM143	-0.09577	0.621279	-0.13471	0.049376	0.112297	-0.06793	NA	NA
TMEM144	0.047249	0.117445	0.190747	-0.20278	0.131873	-0.43357	NA	NA
TMEM145	0.136899	0.672855	-0.22777	0.165698	0.154277	-0.02196	NA	NA
TMEM146	0.171587	-0.17723	-0.14366	-0.04249	-0.16446	0.026147	NA	NA
TMEM147	0.146981	-0.21978	0.238118	0.301617	-0.10087	0.188133	NA	NA
TMEM149	-0.18368	0.317964	0.093669	0.52406	-0.05521	0.185161	NA	NA
TMEM14A	0.00799	-0.71208	-0.13889	-0.28978	-0.03934	-0.32959	0.478743	0.263953
TMEM14B	-0.07588	-0.5743	-0.00519	-0.18069	-0.3358	-0.1265	-0.19394	-0.29755
TMEM14C	-0.09067	-0.667	0.073583	-0.21	-0.05789	-0.25269	-0.04076	-0.22761
TMEM14E	NA	NA	-0.19052	-0.16183	-0.2018	0.119722	NA	NA
TMEM150	0.360965	0.500614	0.344262	0.079236	0.248926	0.092222	-0.02212	0.043615
TMEM151A	NA	NA	0.133298	0.129866	0.083725	-0.30253	NA	NA
TMEM154	-0.05151	0.215001	0.105351	0.135001	0.045218	-0.03585	-0.39654	-0.27965
TMEM155	0.161838	0.502706	-0.11182	0.019907	0.070537	-0.03966	NA	NA

TMEM156	-0.09253	0.096082	-0.21204	0.631015	-0.19522	0.164789	-0.74306	-0.47262
TMEM158	-0.12822	-0.05786	0.025985	0.043279	0.061763	0.341137	NA	NA
TMEM159	-0.33506	0.124337	-0.0093	-0.17625	0.283742	-0.39699	0.459	0.405706
TMEM160	0.284987	-0.26851	0.213882	-0.08062	0.085396	-0.20129	NA	NA
TMEM161A	0.322696	-0.48172	0.371816	0.121883	0.283875	0.040448	NA	NA
TMEM161E	0.064857	0.40782	0.18516	-0.33965	-0.06607	-0.10773	NA	NA
TMEM163	0.167855	-0.21991	-0.15634	0.662027	-0.1169	0.632518	NA	NA
TMEM164	NA	NA	NA	NA	NA	NA	-0.3821	0.276244
TMEM165	-0.21592	-0.51102	-0.15367	0.158814	-0.28474	-0.08139	NA	NA
TMEM167A	NA	NA	-0.00794	-0.03047	-0.43676	-0.40676	NA	NA
TMEM167E	NA	NA	0.024273	-0.20267	0.180901	-0.48159	NA	NA
TMEM168	0.232725	-0.35386	-0.31744	-0.13932	-0.0324	-0.10716	NA	NA
TMEM169	-0.08242	0.229679	0.114695	0.188417	-0.11635	-0.01532	NA	NA
TMEM17	-0.01969	-0.4036	0.182666	-0.23588	0.095527	-0.2018	-0.37204	0.00069
TMEM170A	NA	NA	-0.21421	-0.01035	0.129946	-0.16178	NA	NA
TMEM170E	NA	NA	-0.15643	-0.44465	-0.19325	0.097632	NA	NA
TMEM171	-0.26838	0.080323	-0.34598	-0.12795	-0.20948	-0.11921	NA	NA
TMEM173	-0.14261	0.186593	0.327295	0.35614	-0.07554	0.469803	-0.32261	0.042488
TMEM174	0.020256	0.134472	-0.09598	-0.07161	-0.1259	0.009293	NA	NA
TMEM175	-0.14391	0.600449	0.12465	0.017762	-0.09902	0.309284	NA	NA
TMEM176A	0.341594	0.160421	0.398424	0.093478	0.107397	0.067021	NA	NA
TMEM176E	NA	NA	0.451877	0.142272	0.12587	0.208606	-0.21726	-0.38911
TMEM177	0.307835	0.169519	0.185642	0.224208	0.13403	-0.22633	NA	NA
TMEM178	-0.02059	-0.26144	-0.15432	-0.0561	0.064295	-0.03947	0.225319	0.127115
TMEM179	-0.12927	0.250253	0.13776	-0.13265	0.372823	0.291838	NA	NA
TMEM179E	NA	NA	0.070017	0.081627	-0.14041	-0.09902	NA	NA
TMEM18	-0.30366	-0.50571	0.06531	-0.17735	0.029336	-0.05429	0.453177	0.515462
TMEM180	0.065341	0.696391	0.173127	-0.00701	0.011767	-0.08754	NA	NA
TMEM181	NA	NA	-0.22392	0.034207	0.244693	-0.21789	NA	NA
TMEM182	0.060794	-0.15042	-0.14326	0.059895	-0.0904	-0.26012	NA	NA
TMEM183A	-0.02933	-0.32208	NA	NA	NA	NA	NA	NA
TMEM183E	NA	NA	-0.03584	0.011385	-0.21042	-0.30882	0.188448	-0.05772
TMEM184A	NA	NA	0.299592	-0.20485	0.416196	-0.021	NA	NA
TMEM184E	NA	NA	0.103039	0.145909	0.378176	0.140001	NA	NA
TMEM184C	NA	NA	-0.18547	-0.11553	-0.21633	-0.40673	NA	NA
TMEM185A	0.255717	0.528513	-0.06987	-0.22387	0.268605	-0.08448	0.214506	0.203674
TMEM185E	NA	NA	-0.13324	0.134407	0.033897	0.073776	0.264416	-0.02353
TMEM186	0.278858	0.534255	-0.18916	0.185886	0.100349	0.067307	0.597681	0.278017
TMEM187	NA	NA	-0.22351	-0.00173	0.063965	-0.42984	NA	NA
TMEM19	0.182351	-0.31562	0.411624	-0.03446	0.089169	-0.00611	-0.43867	-0.54477
TMEM190	NA	NA	-0.01387	-0.08766	0.027575	-0.47247	NA	NA
TMEM192	NA	NA	0.051465	-0.19829	0.095691	-0.08031	NA	NA
TMEM194A	NA	NA	-0.45378	0.201851	0.053638	0.017963	NA	NA
TMEM196	NA	NA	0.163664	-0.0936	0.094183	0.087225	NA	NA
TMEM198	NA	NA	0.575549	0.169639	0.386618	-0.14514	NA	NA
TMEM199	NA	NA	0.028126	0.046981	-0.20714	-0.53288	NA	NA
TMEM2	-0.43539	0.113265	0.404899	-0.17415	-0.06209	0.192439	0.296452	0.350823
TMEM20	0.284733	-0.10045	-0.2147	-0.49559	0.03161	-0.27321	0.291533	0.222898
TMEM200A	NA	NA	-0.02216	-0.27027	-0.25661	-0.2111	NA	NA
TMEM200E	NA	NA	0.138237	0.083318	0.040833	0.162378	NA	NA
TMEM201	NA	NA	-0.27633	0.233538	-0.08417	0.047103	NA	NA
TMEM202	NA	NA	0.000858	-0.16591	-0.21342	0.152666	NA	NA

TMEM203	NA	NA	0.164319	0.243828	0.270096	0.289229	NA	NA
TMEM204	NA	NA	0.029682	0.252066	0.030576	0.220455	NA	NA
TMEM205	NA	NA	0.149115	0.006146	0.007316	-0.31064	NA	NA
TMEM206	NA	NA	-0.10665	0.117475	-0.07449	0.024412	NA	NA
TMEM208	NA	NA	-0.11303	0.087595	0.009333	-0.29828	NA	NA
TMEM209	NA	NA	-0.07081	0.035982	0.076869	-0.01712	NA	NA
TMEM213	NA	NA	0.046089	-0.13348	1.12E-05	-0.01333	NA	NA
TMEM214	NA	NA	0.472936	0.206881	0.185864	-0.07239	NA	NA
TMEM216	NA	NA	-0.10048	-0.14663	-0.2262	-0.16847	NA	NA
TMEM217	NA	NA	-0.34644	-0.28155	-0.15476	0.060461	NA	NA
TMEM218	NA	NA	0.029925	0.362947	-0.39136	0.065641	NA	NA
TMEM219	NA	NA	0.36362	-0.12754	0.157895	-0.20557	NA	NA
TMEM22	0.132424	-0.46917	-0.03275	-0.06094	-0.02573	0.102893	-0.24724	0.295167
TMEM220	NA	NA	0.199173	-0.20555	-0.18188	-0.52748	NA	NA
TMEM222	NA	NA	0.193663	0.035593	0.206331	-0.28177	NA	NA
TMEM25	-0.22246	0.268838	0.019277	0.060101	-0.06959	0.174035	-0.20802	0.201147
TMEM26	-0.08932	0.405793	-0.06355	-0.25359	-0.00381	0.089027	0.096114	0.584393
TMEM27	-0.25237	0.427652	-0.0959	-0.15318	-0.25598	-0.26816	0.224132	0.462843
TMEM30A	-0.15539	-0.35351	-0.34787	-0.15136	-0.23426	-0.19253	-0.37164	-0.25875
TMEM30B	0.204741	-0.25454	-0.30324	0.124646	0.076658	-0.32155	0.315699	0.112352
TMEM31	0.034944	-0.1838	-0.4032	0.050678	-0.36907	-0.18632	0.020325	-0.15297
TMEM33	0.028286	-0.05758	-0.16743	0.044312	-0.19744	-0.43373	NA	NA
TMEM35	0.069995	-0.33471	-0.32742	-0.05606	-0.04823	-0.37235	-0.24044	-0.28329
TMEM37	0.179728	-0.14762	0.3675	-0.13655	-0.01909	-0.30113	-0.06932	0.283778
TMEM38A	-0.24587	-0.20637	-0.01443	-0.19974	0.190641	0.123595	0.16956	-0.19221
TMEM38B	0.087362	0.081026	-0.03521	-0.08378	0.077744	-0.22363	-0.01635	-0.35116
TMEM39A	-0.25032	0.065903	-0.30322	0.252388	-0.12013	-0.11557	0.503649	0.510132
TMEM39B	0.050663	0.409705	0.227714	0.233563	-0.00384	0.08834	0.511806	0.527289
TMEM40	-0.05368	0.321037	0.118964	-0.19733	-0.00538	-0.15518	0.456563	0.454694
TMEM41A	-0.20187	-0.33264	-0.06436	-0.14314	0.226226	0.014301	0.395523	0.583852
TMEM41B	-0.15816	0.099091	-0.31104	-0.29502	-0.08069	0.013295	-0.2701	-0.08177
TMEM42	0.078162	-0.25948	0.09781	0.174918	0.050323	-0.04342	0.097353	0.013767
TMEM43	0.057308	-0.38213	-0.18391	0.015091	0.052208	0.388074	0.249049	-0.00427
TMEM44	-0.1152	0.279705	0.098073	-0.1486	0.326224	0.416836	-0.46709	-0.51127
TMEM45A	-0.44501	-0.35011	-0.17759	0.090536	-0.27012	-0.41156	-0.34343	-0.05558
TMEM45B	-0.00894	0.119672	0.092277	-0.11937	0.043954	-0.3421	-0.0883	-0.14125
TMEM47	-0.10393	-0.25701	0.26997	-0.05258	-0.05772	0.199443	-0.32185	-0.42634
TMEM48	0.27516	0.262064	-0.13617	0.145583	0.151502	-0.12414	0.36327	0.231827
TMEM49	-0.05206	0.002817	0.13218	-0.03364	-0.07838	-0.18188	0.142495	0.582688
TMEM5	0.174894	0.004878	-0.10782	0.312004	-0.12097	-0.18135	0.060334	-0.08169
TMEM50A	-0.22991	-0.29013	-0.01253	-0.2673	0.134697	-0.09304	0.359807	0.375349
TMEM50B	0.025473	0.549558	-0.06946	-0.07277	-0.24875	-0.10699	0.019951	0.047136
TMEM51	-0.46005	-0.3964	-0.0896	-0.04449	0.27421	0.247716	0.041721	0.141932
TMEM52	-0.01281	0.568689	0.130037	0.034316	0.333789	-0.4168	-0.036	0.373853
TMEM53	0.385799	0.698737	0.333418	-0.12505	0.159186	-0.26288	-0.04012	-0.30311
TMEM54	-0.3193	0.290468	0.251936	0.079298	-0.03604	-0.29986	0.303302	0.076078
TMEM55A	0.221828	0.204285	0.20109	-0.23554	-0.10008	-0.11506	-0.13062	-0.34514
TMEM55B	-0.26228	0.27388	0.086077	0.02343	0.054694	0.025436	0.260918	0.511324
TMEM56	-0.18524	-0.07219	-0.24816	-0.26341	0.102532	-0.4124	0.055488	0.03424
TMEM57	-0.38318	-0.2446	-0.06554	0.130681	0.149818	-0.03803	0.52763	0.337416
TMEM59	-0.44126	-0.11989	0.177277	-0.31925	-0.07531	-0.23814	0.478255	0.259721
TMEM59L	NA	NA	-0.03648	-0.26959	-0.0966	0.176468	NA	NA

TMEM60	-0.05951	0.0569	-0.14248	-0.28195	-0.32118	-0.28742	-0.07577	-0.17885
TMEM61	-0.0053	0.586701	-0.05534	0.138386	-0.17807	-0.46207	0.055271	0.07091
TMEM62	-0.02225	0.172788	0.458581	-0.07499	0.13354	-0.01057	0.524232	0.428309
TMEM63A	0.315163	-0.05081	0.153158	0.036703	0.370436	0.209941	0.564089	0.235658
TMEM63B	-0.26973	-0.21272	-0.00709	0.050061	0.125053	-0.26897	-0.51296	-0.56342
TMEM63C	-0.03255	0.102982	NA	NA	NA	NA	0.021002	-0.22897
TMEM64	0.164809	0.220585	-0.07983	0.049087	-0.04859	-0.14996	0.363881	0.098261
TMEM65	0.030821	0.680369	-0.1666	-0.25967	-0.22791	-0.6405	0.398549	0.14673
TMEM66	-0.22205	-0.49119	0.271569	0.268441	-0.08864	-0.0459	-0.14151	-0.01293
TMEM67	0.093089	-0.47826	-0.08268	-0.13193	-0.23888	-0.24109	0.000246	-0.20281
TMEM68	0.013491	0.06766	0.091513	-0.15581	-0.14702	-0.20322	-0.00409	-0.14183
TMEM69	-0.25639	-0.35379	0.298968	-0.20836	0.09222	0.022868	0.580843	0.700492
TMEM70	-0.05191	0.234523	0.097408	-0.11982	-0.47049	-0.15656	-0.47184	-0.43046
TMEM71	-0.05814	0.078598	-0.22037	0.164372	0.096844	0.269638	-0.49683	-0.08125
TMEM72	NA	NA	-0.01383	-0.28914	-0.1063	-0.17585	NA	NA
TMEM74	-0.21876	-0.01523	-0.18505	-0.14602	-0.25725	-0.44695	-0.43024	-0.37524
TMEM77	-0.13217	-0.39223	0.06363	-0.09896	-0.02624	-0.37499	0.305501	0.438611
TMEM79	-0.10785	-0.41533	0.06487	0.230768	0.14486	-0.12756	0.461811	0.102389
TMEM8	-0.10127	0.161388	0.284974	0.227216	0.196919	-0.32274	0.462594	0.25447
TMEM80	0.059044	0.261889	0.147862	-0.14374	0.016484	-0.2716	0.087072	-0.11406
TMEM81	-0.06885	0.48335	0.040002	0.199442	-0.05754	-0.15164	0.141814	0.030668
TMEM84	-0.08942	0.012765	0.284156	-0.07821	-0.24565	0.084504	NA	NA
TMEM85	0.098699	-0.59858	-0.07931	0.076135	-0.3296	-0.36654	0.315401	0.161103
TMEM86A	-0.09979	0.645255	0.206486	-0.11269	-0.00404	-0.02218	-0.59345	0.017895
TMEM86B	-0.00878	0.43335	0.035956	-0.06371	-0.02962	-0.11019	-0.06893	0.644019
TMEM87A	-0.37924	-0.23016	0.069234	-0.21326	-0.13457	0.19626	-0.12693	-0.06071
TMEM87B	-0.09494	-0.20474	-0.08357	-0.09501	0.140986	-0.23432	0.109574	-0.20699
TMEM88	0.413042	0.794449	-0.02408	0.086742	-0.11719	-0.01556	-0.47779	0.118012
TMEM89	0.132228	0.094142	-0.15179	-0.34678	0.045099	-0.35348	0.333889	0.118916
TMEM9	0.314706	0.003147	0.135804	-0.0038	0.235885	-0.12014	0.673254	0.260836
TMEM91	NA	NA	NA	NA	NA	NA	-0.39993	-0.29957
TMEM92	0.031482	0.803071	0.206261	0.258085	-0.18062	-0.16712	0.090796	0.622346
TMEM93	-0.2385	-0.25455	-0.12665	0.309314	-0.27363	-0.02384	-0.27971	-0.14565
TMEM95	-0.19859	0.574622	0.128395	-0.25753	0.125104	-0.2321	-0.34338	-0.21056
TMEM97	0.198594	0.580286	0.162389	0.154832	-0.01413	-0.02997	0.304083	-0.07271
TMEM98	0.064643	-0.02567	0.302175	-0.02263	0.042177	-0.18825	0.390095	0.261303
TMEM99	0.178316	0.593146	0.497086	-0.24953	-0.27422	-0.05516	0.322918	0.009782
TMEM9B	-0.05202	0.061557	-0.14335	-0.13573	-0.12556	-0.49916	0.089468	0.112806
TMF1	-0.4129	-0.45263	-0.29274	-0.05485	-0.14604	-0.27036	0.28934	0.321684
TMIE	-0.03025	-0.17905	-0.04394	-0.1408	-0.27878	-0.09419	-0.05524	-0.10273
TMIGD1	-0.03005	0.052079	NA	NA	NA	NA	NA	NA
TMIGD2	0.342404	0.601409	0.172242	0.182269	0.029909	0.05682	0.044195	0.208077
TMLHE	0.431708	-0.39165	0.107064	-0.48525	0.133064	-0.51777	0.492709	0.254346
TMOD1	-0.25422	-0.09832	0.007857	-0.05179	0.179684	-0.0547	-0.32937	-0.29384
TMOD2	0.120464	0.360039	-0.00946	0.058647	-0.1447	-0.15503	0.50552	0.397211
TMOD3	-0.14916	0.334093	0.029901	-0.11604	-0.11579	-0.09991	-0.10925	0.011089
TMOD4	-0.09742	0.387921	-0.39509	0.106509	-0.15527	-0.121	0.636447	0.375096
TMPO	-0.19207	-0.23694	-0.38895	0.12524	-0.04515	-0.07991	0.187398	0.156234
TMPRSS11/	0.093438	0.547579	-0.12133	0.164688	-0.17509	-0.19525	0.471779	0.39597
TMPRSS11I	0.068223	0.258256	0.06865	-0.14296	-0.0735	0.268071	0.200094	0.108566
TMPRSS11I	-0.02126	0.383916	-0.23226	-0.06325	-0.02444	-0.07563	-0.33576	-0.3004
TMPRSS11I	-0.1755	0.087201	-0.20875	0.025284	-0.20945	-0.25514	NA	NA

TMPRSS11I	-0.11179	0.184477	-0.27237	-0.0635	0.227016	0.292734	NA	NA
TMPRSS12	-0.17277	0.314861	-0.03141	-0.27831	-0.06809	-0.17648	0.240175	-0.02191
TMPRSS13	-0.13068	-0.18749	0.073708	0.213373	-0.05258	-0.33273	0.031264	-0.23257
TMPRSS2	0.193076	0.091358	-0.06223	-0.18754	0.05016	-0.02911	0.29488	0.164679
TMPRSS3	0.032716	0.355384	0.050117	0.204215	0.041919	-0.21246	0.122075	0.393328
TMPRSS4	0.022663	0.091309	-0.0292	-0.12502	-0.01401	-0.37713	0.224519	-0.152
TMPRSS5	0.077924	0.70383	-0.16381	0.195583	-0.21427	0.138455	0.372673	0.343956
TMPRSS6	0.301839	0.086865	0.03605	-0.21363	-0.0508	-0.22642	-0.15181	-0.2808
TMPRSS7	NA	NA	NA	NA	NA	NA	0.257939	0.340567
TMPRSS9	0.223903	0.707998	-0.13168	-0.11861	-0.02978	0.009323	0.23076	0.003464
TMSB10	0.037877	-0.2063	-0.34862	0.410883	-0.26642	-0.27412	0.359083	-0.1145
TMSB15A	NA	NA	-0.15172	0.180891	-0.21855	-0.4314	NA	NA
TMSB15B	NA	NA	-0.03597	-0.26799	-0.23579	-0.44629	NA	NA
TMSB4X	NA	NA	0.273773	-0.16358	-0.26664	0.221748	-0.72228	-0.56929
TMSB4Y	0.221374	0.063376	0.170438	-0.02741	-0.07105	-0.28145	0.243693	-0.09853
TMTC1	0.198038	0.64183	0.022013	0.03662	-0.18774	-0.02462	NA	NA
TMTC2	-0.05958	-0.47026	-0.24147	0.209548	-0.06115	-0.16614	-0.46601	-0.171
TMTC3	-0.30776	-0.55903	-0.2816	-0.31687	0.206101	-0.24098	0.221555	0.227064
TMTC4	0.275668	-0.28575	0.088398	-0.00334	0.307579	-0.16057	0.516513	0.280051
TMUB1	0.311296	0.370342	0.444432	-0.00261	0.21055	0.034882	NA	NA
TMUB2	-0.00389	-0.37753	0.376133	-0.13318	0.214794	0.166938	-0.49372	-0.12936
TNC	-0.32236	-0.17402	0.073915	0.01413	-0.00046	0.262141	-0.47862	-0.42069
TNF	-0.17412	0.166492	0.139178	0.57854	0.087191	0.401107	-0.21097	0.30487
TNFAIP1	-0.12673	-0.54251	-0.00867	-0.15604	0.246661	0.082421	-0.24171	0.096471
TNFAIP2	-0.29583	-0.10277	0.030992	0.362928	-0.04678	0.563132	-0.30586	0.574246
TNFAIP3	-0.05518	-0.21324	0.066313	0.466425	0.118927	0.366939	-0.36156	0.063573
TNFAIP6	-0.37177	-0.20727	0.062539	-0.24094	-0.0743	0.055663	0.39984	0.458535
TNFAIP8	0.111373	0.409088	0.030503	0.456194	-0.29153	0.531943	-0.37206	0.185436
TNFAIP8L1	-0.08566	0.066008	-0.00026	0.070713	0.019346	0.146821	-0.55226	-0.60032
TNFAIP8L2	-0.10813	0.168773	0.287146	0.377303	-0.29643	0.274146	-0.51526	0.380789
TNFAIP8L3	0.112082	0.127135	0.165285	-0.10529	-0.04658	0.205171	-0.43853	-0.48516
TNFRSF10A	-0.18591	0.005152	0.204581	0.171006	0.009828	0.184619	0.307711	0.37035
TNFRSF10B	0.049812	0.350647	0.333072	-0.03261	-0.02593	0.235961	-0.12575	0.208979
TNFRSF10C	-0.01103	0.764851	0.231361	0.165264	-0.0273	-0.20764	0.16265	0.207245
TNFRSF10D	-0.57796	0.035495	0.093849	0.156593	0.015396	0.057856	0.128295	0.528897
TNFRSF11A	-0.14622	-0.09972	-0.41676	0.132337	-0.12141	-0.02835	-0.29967	-0.32007
TNFRSF11B	-0.15239	-0.25087	-0.14846	0.093825	-0.16705	0.163067	0.094347	0.316919
TNFRSF12A	-0.07696	-0.14938	0.135438	-0.15764	0.303328	0.263272	-0.00458	0.172834
TNFRSF13B	0.109852	-0.15108	-0.04672	0.702536	-0.21274	0.395336	-0.13276	0.226236
TNFRSF13C	-0.46178	0.328594	0.121798	0.844279	-0.15997	0.445333	-0.53146	-0.40551
TNFRSF14	-0.28115	0.209981	0.265136	0.168549	-0.08503	-0.01595	-0.23789	0.291861
TNFRSF17	-0.22756	-0.06059	0.081139	-0.08817	-0.0846	-0.10021	-0.18162	-0.15167
TNFRSF18	-0.4389	-0.27896	0.392035	0.35561	-0.09714	0.039345	-0.16524	0.201341
TNFRSF19	0.144315	-0.6071	0.3254	-0.22868	-0.23149	0.062098	0.536218	-0.09224
TNFRSF1A	0.068282	0.049977	0.066599	-0.40847	-0.00736	0.360078	-0.08042	0.431001
TNFRSF1B	-0.26172	-0.01306	0.57904	0.225789	0.377394	0.386929	-0.66077	-0.35503
TNFRSF21	0.081787	0.309966	-0.13854	-0.01078	0.075206	0.209344	0.203475	0.29145
TNFRSF25	0.285195	0.114712	-0.04587	-0.01259	0.093175	4.9E-05	0.161316	0.294529
TNFRSF4	-0.1433	0.669344	0.29683	0.196907	0.013719	0.178433	-0.36709	0.310553
TNFRSF6B	0.082606	0.559082	0.517796	0.356113	0.182377	0.61845	-0.16226	0.226908
TNFRSF8	-0.01246	0.064749	0.026392	0.602512	0.057099	0.397338	0.146795	0.086568
TNFRSF9	-0.26315	0.342553	0.223198	0.512766	-0.11185	0.66568	-0.29715	0.020175

TNFSF10	-0.1458	-0.18238	0.077093	-0.22539	-0.22174	-0.23849	-0.28864	0.211907
TNFSF11	-0.17521	-0.19822	0.176677	0.572527	-0.04664	0.585608	-0.08906	0.175513
TNFSF12	-0.24815	-0.24891	-0.1174	0.07448	-0.33305	0.05832	-0.39317	-0.26772
TNFSF13	-0.24366	-0.28103	0.198052	0.399939	-0.14752	-0.19992	0.233251	0.226977
TNFSF13B	-0.00735	-0.36814	0.233274	0.38502	-0.43243	0.49549	-0.60182	-0.17685
TNFSF14	-0.63229	-0.04777	0.369931	0.423176	0.00415	0.593919	-0.6564	-0.15311
TNFSF15	0.284676	0.677963	0.39917	0.175859	-0.14279	-0.04403	-0.41387	-0.40665
TNFSF18	0.312023	0.200938	-0.06996	-0.06023	0.046055	-0.02892	-0.11168	0.638156
TNFSF4	-0.07332	-0.2635	0.209759	-0.13483	-0.15898	-0.32822	-0.17324	-0.32678
TNFSF8	-0.31256	0.038276	0.232142	0.172504	-0.15691	0.036217	-0.45151	-0.03037
TNFSF9	0.179315	-0.13008	0.27249	0.035342	0.019577	0.171638	0.037904	-0.01838
TNIK	-0.09682	-0.32033	-0.0877	-0.39524	-0.2014	0.28415	0.143464	0.224808
TNIP1	-0.16007	0.130506	0.416003	0.160037	0.122271	-0.01129	-0.46538	-0.16622
TNIP2	-0.25842	0.224385	0.321023	0.373361	0.240211	-0.15975	-0.29642	-0.42666
TNIP3	-0.29891	0.53792	0.010029	0.107156	0.137416	0.037284	-0.70111	-0.22725
TNK1	-0.26438	0.621466	0.099181	-0.14511	0.092112	0.01353	0.544124	0.429816
TNK2	-0.162	0.58946	0.099044	0.158101	0.284346	0.481878	-0.02116	0.157092
TNKS	0.046785	-0.15186	0.236587	-0.0392	-0.06601	0.142797	0.434539	0.292794
TNKS1BP1	-0.07188	0.086865	0.108069	0.219666	0.091359	0.047335	0.509652	0.396683
TNKS2	0.478284	0.762645	-0.1003	-0.1577	0.001184	0.140826	0.130663	-0.00535
TNMD	0.375193	0.082177	0.181408	-0.29315	-0.33271	-0.09717	-0.13561	0.320942
TNN	0.268773	-0.08078	-0.10607	-0.23377	-0.17395	-0.42916	-0.0913	-0.61986
TNNC1	0.205624	0.19675	-0.05694	0.189513	-0.37068	-0.38309	0.339175	-0.10942
TNNC2	0.352465	-0.175	0.195995	-0.4344	0.337424	-0.396	-0.0399	0.058142
TNNI1	0.406695	0.412879	-0.02448	-0.29006	-0.08986	0.065427	-0.283	0.089284
TNNI2	-0.60447	-0.25072	0.086628	0.384346	-0.2798	-0.00516	-0.11943	0.009244
TNNI3	0.194923	0.43981	-0.17528	-0.18003	-0.05783	0.050896	-0.23012	-0.02487
TNNI3K	-0.07555	-0.12502	0.004402	-0.43084	-0.38502	0.043039	0.23213	0.244426
TNNT1	-0.28647	-0.01442	-0.24038	-0.02691	-0.07907	-0.21864	-0.0726	-0.07528
TNNT2	-0.45153	0.660676	-0.19725	0.118783	-0.21341	0.216141	-0.38102	-0.43865
TNNT3	0.183931	-0.00036	0.39073	-0.10929	0.029245	0.087104	-0.09316	0.237542
TNP1	-0.27677	0.238347	-0.05434	-0.11865	0.325093	0.00817	0.411555	0.267945
TNP2	0.327575	0.253883	0.050092	-0.11112	0.218608	0.093275	-0.0868	-0.329
TNPO1	-0.21677	-0.03116	0.146496	-0.19575	-0.03346	0.404004	0.436544	0.161005
TNPO2	0.056486	0.38572	-0.07747	-0.04448	0.230089	0.351083	-0.44374	-0.22441
TNPO3	0.242407	0.282122	0.217207	0.065781	0.194345	0.228618	0.494623	0.044857
TNR	0.054605	-0.00882	-0.12311	-0.16134	0.023078	-0.02731	-0.00777	0.146598
TNRC18	NA	NA	0.013733	-0.13169	0.280112	0.24963	NA	NA
TNRC4	-0.0366	-0.16807	-0.01873	0.42526	0.277568	-0.23454	0.52404	0.211499
TNRC6A	0.022652	-0.15315	-0.02276	-0.0718	0.208839	0.35204	0.124959	0.194295
TNRC6B	0.090323	0.743462	-0.04293	0.083102	-0.0064	0.015368	0.014772	-0.30119
TNRC6C	0.119847	-0.24968	-0.00651	0.411574	0.347149	0.303562	0.641419	0.242119
TNS1	-0.11863	-0.02818	0.117123	-0.12885	0.090892	0.144809	-0.12514	-0.14891
TNS3	0.021135	-0.5779	0.270838	0.127811	0.080295	0.164445	0.102187	0.1487
TNS4	-0.01269	0.779684	0.239212	-0.16445	0.070026	0.246638	0.181049	-0.1541
TNXB	0.164312	0.39188	-0.08868	-0.10057	-0.16396	0.003125	0.284201	0.296468
TOB1	0.079168	0.188736	0.182344	0.108159	-0.15872	-0.19184	0.536958	-0.12102
TOB2	-0.12454	0.386082	0.070565	0.081869	0.188436	-0.2089	-0.20172	-0.467
TOE1	-0.26745	-0.41932	0.055298	0.07978	0.30995	0.299407	0.165725	0.401527
TOLLIP	0.096696	0.250511	0.168019	-0.09479	0.063246	-0.17799	0.492286	0.296837
TOM1	-0.01749	0.266042	0.449546	-0.17103	0.211489	-0.00887	-0.354	-0.04542
TOM1L1	-0.11899	-0.45295	0.221415	-0.1853	-0.19597	-0.14412	0.474037	0.447175

TOM1L2	0.00428	0.595905	0.231836	-0.21077	-0.0006	0.086117	0.180058	0.258259
TOMM20	-0.08232	-0.3654	-0.1812	0.018142	0.114212	-0.04053	0.496952	0.136947
TOMM20L	NA	NA	-0.13079	-0.0827	-0.25168	-0.23348	NA	NA
TOMM22	0.203131	0.650735	0.008671	0.294813	0.033135	-0.22522	-0.08548	-0.50843
TOMM34	0.472764	0.365455	0.181002	0.180978	0.371202	-0.08447	0.399871	-0.1559
TOMM40	0.180767	0.399785	0.017561	0.207152	0.132839	0.047839	0.282927	-0.13842
TOMM40L	-0.19672	0.543534	-0.08041	-0.14681	-0.33977	-0.26248	-0.32803	-0.34991
TOMM5	NA	NA	-0.32571	-0.1059	-0.09523	-0.18911	NA	NA
TOMM6	NA	NA	-0.14922	-0.216	-0.20583	-0.11391	NA	NA
TOMM7	0.360172	-0.08354	0.07172	-0.23425	-0.23671	-0.19908	-0.54853	-0.20498
TOMM70A	0.172259	-0.1027	-0.11444	0.026249	0.085203	-0.01948	-0.19486	-0.12925
TOP1	-0.1085	-0.20377	-0.15262	0.038229	0.211757	0.126041	0.050893	-0.04304
TOP1MT	0.190913	-0.2852	0.261828	0.42563	0.22815	0.489091	0.561552	0.02066
TOP1P2	NA	NA	-0.13185	-0.05972	0.269926	0.153841	0.000787	-0.16297
TOP2A	0.059064	0.106531	-0.1455	0.09655	-0.02738	0.044042	0.490409	0.144883
TOP2B	0.00456	-0.52847	-0.13171	-0.21315	0.094195	0.509417	0.486917	0.278027
TOP3A	-0.03592	-0.48286	-0.09433	0.374133	-0.11493	-0.23046	-0.54153	-0.23276
TOP3B	0.303842	0.651146	0.400422	0.020819	0.437008	0.016205	0.561607	0.325149
TOPBP1	0.038922	-0.16383	-0.34787	0.032082	-0.15127	0.157229	-0.46942	-0.31216
TOPORS	-0.03972	-0.51258	-0.15354	-0.38023	-0.07986	0.140158	-0.30804	0.122931
TOR1A	0.22151	-0.0722	-0.14815	-0.03444	-0.01387	-0.2372	-0.19853	-0.12905
TOR1AIP1	-0.21585	-0.50802	-0.29598	-0.09163	-0.28258	-0.21937	0.2061	-0.02889
TOR1AIP2	-0.08858	-0.24647	-0.60434	-0.46247	-0.39447	-0.50047	0.22439	0.199579
TOR1B	-0.13679	-0.28116	-0.00889	-0.03813	0.000177	-0.19374	0.10536	0.229455
TOR2A	-0.00908	0.453791	0.208102	-0.02402	0.174075	-0.11213	0.232151	0.302127
TOR3A	-0.38374	-0.15376	0.070314	0.280577	0.241799	0.109607	0.373199	0.231612
TOX	-0.36559	0.063314	-0.10379	-0.05912	-0.0825	-0.22173	-0.31208	-0.46538
TOX2	NA	NA	-0.0348	0.451765	-0.09845	0.512661	NA	NA
TOX3	NA	NA	-0.03109	-0.01597	0.392093	-0.16305	NA	NA
TOX4	NA	NA	-0.23204	-0.23816	-0.12241	-0.05661	NA	NA
TP53	0.198027	-0.45726	-0.00966	0.570942	-0.114	-0.03589	0.396216	0.226066
TP53BP1	0.333429	-0.11573	0.111389	0.104772	-0.03435	0.32241	0.169722	-0.08557
TP53BP2	-0.15597	-0.57097	-0.22261	-0.01951	-0.0038	0.244471	0.498833	0.231696
TP53I11	0.10583	0.682568	0.042946	0.276572	0.278465	0.200512	0.050962	0.18848
TP53I13	-0.12519	0.363587	0.371932	0.008256	0.286762	-0.02798	-0.49912	-0.23021
TP53I3	-0.13621	0.267634	0.013842	0.135806	0.016752	-0.12331	0.11199	0.352525
TP53INP1	-0.47651	-0.43185	0.259255	-0.05199	-0.27344	-0.02249	-0.39775	-0.40428
TP53INP2	0.019044	0.177447	-0.13876	-0.09269	0.311158	-0.02067	-0.04575	0.045604
TP53RK	0.406381	-0.27072	0.125727	0.094504	0.225711	-0.2854	0.629144	0.407241
TP53TG1	NA	NA	0.266237	-0.50029	-0.25478	-0.33018	NA	NA
TP53TG3	0.045774	0.298292	0.009581	0.162261	-0.07118	-0.26388	0.443649	-0.09397
TP53TG5	NA	NA	0.040744	-0.12231	-0.13437	0.073825	NA	NA
TP63	NA	NA	-0.34824	0.182598	-0.10653	0.242303	NA	NA
TP73	-0.02183	0.400837	0.388569	0.115731	0.403419	-0.1583	0.375898	-0.12684
TPBG	0.068658	0.12823	-0.22249	0.185724	-0.03858	-0.0197	NA	NA
TPCN1	0.113601	0.062104	0.113364	0.082946	0.439563	-0.10475	0.300443	0.40252
TPCN2	-0.10316	0.475261	0.180305	-0.08215	0.061911	-0.03321	-0.34507	0.213639
TPD52	-0.34324	-0.06759	-0.04478	-0.00075	-0.346	-0.18429	-0.55702	-0.11838
TPD52L1	0.310851	-0.2281	0.164696	0.016777	0.041844	0.068449	-0.13767	-0.28985
TPD52L2	0.193057	0.154898	-0.12541	0.188018	0.078882	-0.30766	-0.01083	-0.24012
TPD52L3	0.309402	0.62655	-0.17541	-0.14723	-0.1825	0.111227	0.451364	0.383206
TPH1	0.259531	-0.02121	0.087949	0.134625	0.056461	-0.20623	-0.06366	-0.02603

TPH2	-0.08363	0.334972	-0.04984	-0.04144	-0.06006	0.066467	-0.10561	-0.06404
TPI1	0.098282	-0.65565	-0.37553	0.159335	-0.23852	0.101098	0.083593	0.144301
TPK1	0.055404	0.137925	0.12704	-0.18149	-0.02225	-0.27602	-0.26103	-0.03697
TPM1	-0.23216	-0.45599	-0.16329	-0.27788	-0.04408	-0.23937	-0.34232	-0.27549
TPM2	-0.01705	0.058442	0.032429	-0.01092	0.054095	0.073319	-0.16376	-0.28797
TPM3	-0.27366	0.287219	-0.36665	0.237444	-0.2101	-0.08535	0.438204	0.30031
TPM4	0.020239	-0.08527	-0.24435	0.087288	-0.07643	0.04205	-0.26417	-0.19221
TPMT	0.212992	-0.09248	0.096821	-0.12897	0.008726	-0.07452	0.102617	0.063711
TPO	-0.29384	-0.16067	-0.29467	0.050781	-0.08784	0.171661	-0.29083	0.253383
TPP1	-0.03189	-0.01106	0.05859	-0.06721	-0.00218	0.103801	-0.03769	0.437402
TPP2	0.224117	-0.82575	-0.05169	0.217771	0.16395	0.176989	-0.18304	0.101805
TPPP	-0.30931	-0.30914	-0.01269	-0.09032	-0.01734	-0.07198	-0.26546	-0.05285
TPPP2	NA	NA	-0.14333	-0.18206	-0.07941	-0.27474	NA	NA
TPPP3	NA	NA	0.435835	-0.12409	0.129689	-0.33964	NA	NA
TPR	0.030094	-0.52116	-0.02617	0.158129	-0.04381	0.548049	0.559628	0.372693
TPRG1	NA	NA	-0.34788	0.284907	-0.2822	-0.26811	NA	NA
TPRG1L	NA	NA	0.240397	-0.14556	0.215503	-0.0274	NA	NA
TPRKB	0.313044	-0.41624	-0.14907	-0.10856	-0.12525	-0.37996	-0.50741	-0.47537
TPRX1	-0.01298	0.644018	-0.20653	0.209515	-0.25786	-0.60412	-0.3104	-0.20404
TPRXL	NA	NA	-0.39169	0.390586	-0.02996	0.537178	0.054389	0.485762
TPSAB1	NA	NA	0.300597	0.347578	-0.01989	0.135857	0.001804	0.074491
TPSD1	0.051961	0.632455	0.000885	0.135983	0.046934	-0.06326	-0.15623	-0.12879
TPSG1	-0.37923	0.262274	0.001793	0.021903	-0.1753	0.195942	0.133871	-0.01654
TPST1	-0.12687	-0.30854	-0.02485	0.042517	-0.17088	-0.1841	-0.12516	0.165902
TPST2	0.166615	-0.16834	0.378084	0.26707	-0.06974	-0.13543	0.20744	0.360668
TPT1	0.046017	-0.65993	-0.25906	0.461176	0.023048	-0.03797	0.774046	0.589844
TPTE	0.046699	0.407866	0.122619	-0.34933	0.098804	-0.14213	0.233975	0.315855
TPTE2	0.356104	0.430844	0.111198	0.072697	-0.09495	-0.04136	-0.03985	0.436278
TPX2	0.336153	0.242522	-0.06019	0.307036	0.206909	0.025857	0.473012	0.122764
TRA2A	-0.02289	-0.0443	-0.24377	0.044389	0.149342	0.075975	0.135783	0.18772
TRABD	0.175834	0.2221	0.151787	0.172866	0.080304	0.121301	NA	NA
TRADD	-0.39349	-0.27111	0.029354	-0.0299	0.204988	0.174618	0.216608	0.359871
TRAF1	0.083008	0.757659	0.11441	0.681304	0.040964	0.730734	0.375927	0.512837
TRAF2	0.054535	0.255359	0.261516	0.319918	0.443976	0.411597	0.160825	0.461454
TRAF3	0.29566	0.255345	-0.19838	0.459175	-0.10834	0.179611	0.239892	0.66865
TRAF3IP1	0.084549	-0.48619	0.171011	-0.19508	0.264914	-0.044	0.071569	-0.01377
TRAF3IP2	-0.04682	-0.02964	0.221339	-0.02906	0.066049	-0.1321	0.026978	-0.08762
TRAF3IP3	-0.48503	0.258001	0.078664	0.649072	-0.34078	0.668571	-0.39508	0.49895
TRAF4	0.393798	0.215715	0.322277	0.091582	0.30996	0.009172	0.177734	-0.06387
TRAF5	0.143417	0.467929	-0.05339	0.17619	0.19702	0.518397	-0.11514	-0.14828
TRAF6	0.395733	0.147729	-0.3116	-0.2319	-0.21575	-0.26257	0.484297	0.473275
TRAF7	-0.07714	0.150727	-0.05316	0.302806	0.341632	-0.36847	0.538089	0.11313
TRAFD1	-0.04554	0.331898	0.215776	0.02449	0.243391	-0.11839	-0.30375	0.066753
TRAIP	0.168898	-0.0018	-0.36923	0.093137	0.304936	-0.09146	-0.40521	-0.44578
TRAK1	0.315887	0.361625	0.03311	-0.01698	0.2262	0.054562	0.205343	0.266261
TRAK2	-0.01485	0.09738	0.260682	-0.49934	-0.22435	-0.23022	-0.19166	-0.20409
TRAM1	-0.14466	-0.50099	0.368003	-0.06115	0.033419	0.134323	0.435288	0.216935
TRAM1L1	-0.18065	-0.43113	-0.05935	-0.07986	-0.14213	-0.07919	-0.58397	-0.3905
TRAM2	-0.45146	-0.3872	-0.28711	0.099672	-0.02951	0.038664	-0.25983	0.144847
TRAP1	0.513923	0.220222	0.229161	0.341149	0.291214	0.269855	0.532493	-0.02312
TRAPPC1	0.043435	0.539402	0.080657	0.315508	-0.20057	-0.25215	-0.25657	-0.32716
TRAPPC10	NA	NA	-0.23737	-0.01784	0.15623	0.104251	NA	NA

TRAPPC2	0.021644	-0.59191	-0.02424	-0.02569	0.101518	-0.12743	0.077096	0.111803
TRAPPC2L	-0.00784	0.423915	-0.00688	-0.17325	0.037691	-0.08012	NA	NA
TRAPPC3	-0.35747	-0.65061	0.454214	-0.19765	-0.12243	-0.07552	-0.29301	-0.27227
TRAPPC4	0.023975	-0.32894	-0.09616	-0.01659	-0.43357	-0.35236	-0.02301	-0.1531
TRAPPC5	NA	NA	0.308384	-0.14351	-0.03932	-0.17987	0.079353	-0.21967
TRAPPC6A	0.196013	0.644031	0.222424	0.251861	-0.01336	0.361184	0.096154	-0.35293
TRAPPC6B	-0.4997	-0.59798	-0.2412	-0.41984	-0.2452	-0.23242	-0.67077	-0.28575
TRAPPC9	NA	NA	0.219447	0.281696	0.052723	-0.01975	NA	NA
TRAT1	-0.37301	0.573177	0.107021	0.63355	-0.32422	0.594892	0.528591	0.647663
TRDMT1	-0.07364	-0.38567	-0.17367	-0.3751	-0.22619	-0.51554	-0.00768	0.348531
TRDN	-0.23959	0.302209	-0.07546	-0.09436	0.06247	0.165189	0.144274	0.033491
TREH	0.002015	0.542784	0.222822	-0.11921	0.129059	-0.27753	-0.14653	0.356636
TREM1	-0.50996	-0.22923	-0.00062	0.170378	-0.06048	-0.06692	-0.36347	-0.1532
TREM2	-0.21283	-0.01231	0.207834	-0.31482	-0.38075	0.189298	-0.19345	0.017772
TREML1	0.547072	0.704627	0.172343	0.412167	0.215025	0.145747	-0.39802	-0.66346
TREML2	0.102955	-0.12462	-0.0854	0.556238	0.009798	0.370237	0.474072	0.566942
TREML4	-0.31514	0.760068	0.035265	-0.04655	-0.10452	-0.18454	0.214198	0.16742
TRERF1	-0.20712	-0.01535	-0.16412	0.189724	-0.0849	0.149994	0.033763	0.386484
TREX1	0.199533	0.665383	0.056653	-0.05808	-0.05725	-0.29715	0.548594	0.302356
TREX2	-0.05773	-0.09209	-0.13359	-0.15823	-0.05468	0.071658	0.080626	-0.07056
TRH	0.15851	-0.13045	0.047501	0.078611	-0.00641	-0.03208	-0.37489	-0.39441
TRHDE	0.17048	-0.19263	0.201579	-0.29748	-0.31805	-0.22905	0.537995	0.323215
TRHR	0.362134	0.448748	-0.18679	0.126877	-0.00115	-0.31303	-0.02912	-0.33795
TRIAP1	0.169428	0.510666	-0.28958	-0.11036	-0.07849	-0.24421	-0.46158	-0.24571
TRIB1	0.001794	0.036506	0.021926	0.073564	-0.01533	-0.00025	0.413535	0.414766
TRIB2	-0.61972	-0.20488	0.183412	0.481451	-0.12181	0.638994	-0.285	-0.03916
TRIB3	0.348002	0.040113	-0.06359	0.170609	0.221452	-0.06792	-0.01056	-0.02301
TRIM10	0.198414	0.287281	-0.31712	-0.3197	-0.0199	-0.35012	0.101425	0.202679
TRIM11	-0.10511	0.368267	0.015634	0.055428	0.176115	0.272162	0.180398	0.004573
TRIM13	0.035323	0.167025	0.045242	-0.36358	0.133573	-0.12311	-0.28957	-0.51686
TRIM14	-0.25081	0.04879	0.216553	0.046519	0.06441	0.090115	-0.14759	0.488768
TRIM15	0.290928	0.309971	-0.06771	-0.1869	0.047153	-0.04373	0.049646	0.158894
TRIM16	-0.28222	0.654457	NA	NA	NA	NA	0.190068	0.204168
TRIM16L	NA	NA	0.182111	-0.05496	0.161056	0.263223	0.4557	0.347383
TRIM17	-0.05091	-0.24471	-0.15025	-0.1512	0.036737	0.149051	-0.09636	0.304868
TRIM2	0.103224	-0.04739	-0.17685	-0.12277	-0.0258	0.022898	0.294615	0.047352
TRIM21	0.346335	0.524901	0.295485	0.011455	-0.0993	0.114152	0.124418	0.500778
TRIM22	-0.18336	-0.54647	0.131663	0.20578	-0.54311	0.048309	-0.14454	0.355849
TRIM23	0.171329	-0.20973	-0.04977	-0.09191	-0.17472	-0.06798	0.044866	-0.11526
TRIM24	0.285061	0.309929	-0.42059	-0.17001	0.093059	-0.18349	0.658697	0.411186
TRIM25	0.019312	-0.03165	0.147459	-0.00173	0.057055	0.187089	0.474926	0.370497
TRIM26	0.35785	0.542726	0.122336	0.148847	0.2402	0.23147	0.539548	0.368356
TRIM27	0.268863	-0.31294	-0.12998	0.160283	0.08334	0.034191	NA	NA
TRIM28	0.36406	0.150673	0.093321	0.428445	0.311901	0.354813	0.703735	0.199056
TRIM29	-0.1984	0.284454	0.351281	0.04747	0.06031	-0.01774	0.343074	-0.09351
TRIM3	-0.15534	0.444844	0.089735	0.13552	0.04478	-0.33579	0.027214	0.290397
TRIM31	0.343784	0.225762	-0.10272	-0.20216	0.133625	0.002962	-0.31381	-0.25722
TRIM32	0.154352	-0.36944	-0.06082	0.094194	0.010823	0.179726	0.501849	0.429649
TRIM33	0.034779	-0.56463	0.096076	-0.12762	0.240787	0.446684	0.45267	0.379797
TRIM34	0.331735	0.556841	0.227923	0.000464	-0.18499	-0.43445	0.206373	-0.08128
TRIM35	0.105206	-0.40344	-0.12696	-0.04112	-0.22644	0.11136	0.256015	0.337
TRIM36	-0.35511	0.115256	-0.0214	-0.24066	0.08891	-0.38147	-0.13134	0.27358

TRIM37	-0.1593	-0.45309	0.106712	-0.25297	0.017903	-0.4091	0.388386	0.209048
TRIM38	-0.13103	-0.21812	0.018237	0.062909	-0.34349	0.059894	-0.45512	-0.00653
TRIM39	0.13415	0.366515	-0.39672	0.203666	0.100703	0.102633	0.109898	0.43305
TRIM4	-0.02185	0.579285	0.045587	0.06343	0.469296	0.071615	-0.26932	-0.30468
TRIM40	0.315441	0.559672	-0.10129	-0.16586	-0.0452	0.070598	-0.27488	-0.01387
TRIM41	0.170616	0.08227	0.289722	0.08712	0.023689	0.297687	-0.02969	0.37509
TRIM42	-0.22991	0.207218	-0.16211	-0.18214	-0.16379	-0.08993	0.060302	-0.09626
TRIM43	0.160466	0.726292	0.20472	0.037123	-0.21073	0.049173	-0.43338	0.101672
TRIM44	0.410589	0.369447	0.076402	-0.06869	0.259263	0.489163	0.599241	0.274618
TRIM45	-0.21103	0.003654	0.067752	-0.09136	0.268697	-0.17374	0.487797	0.253906
TRIM46	-0.12806	0.068571	0.142509	-0.19232	-0.02604	0.012855	0.127802	0.219188
TRIM47	0.111397	0.270085	0.2918	0.171463	0.092932	0.177197	-0.04006	0.083033
TRIM48	0.070466	0.477839	0.264525	-0.16847	-0.11015	0.025907	0.593593	0.501622
TRIM49	NA	NA	0.155764	-0.1254	-0.11468	-0.08068	-0.65886	-0.63745
TRIM5	-0.28419	-0.59925	0.114614	0.123556	-0.30936	-0.12419	-0.03521	0.61269
TRIM50	-0.13448	0.467719	-0.11617	0.233894	0.23506	0.173041	NA	NA
TRIM52	0.237578	0.253398	0.224996	0.043292	0.110996	0.433016	0.010574	0.247542
TRIM54	0.295742	0.620431	0.089537	-0.02694	-0.21962	0.11069	0.303671	0.502423
TRIM55	0.49955	0.635354	0.236415	-0.01116	-0.24011	-0.02615	-0.35231	-0.12231
TRIM56	0.325045	-0.553	0.231641	0.062056	0.441631	0.306888	-0.40325	-0.39261
TRIM58	-0.27773	0.459797	-0.13368	-0.0172	0.040204	0.102881	0.345271	0.174724
TRIM59	-0.27322	-0.02163	-0.14619	0.141229	-0.16574	0.026035	0.391782	0.371171
TRIM6	0.004176	0.364867	0.239171	-0.20646	-0.41955	-0.15492	-0.33531	-0.22363
TRIM6-TRIM	0.052362	0.507927	NA	NA	NA	NA	-0.33175	-0.2104
TRIM60	0.236435	0.001754	0.206086	0.141561	0.015604	0.279808	0.233664	0.278468
TRIM61	-0.25972	0.355955	NA	NA	NA	NA	NA	NA
TRIM62	-0.20371	-0.03729	0.075703	0.118426	0.214264	-0.17263	-0.37259	-0.1019
TRIM63	-0.17214	-0.12233	0.121084	0.030069	-0.04131	0.024181	0.168864	-0.40798
TRIM64	NA	NA	-0.22512	-0.17219	0.181521	0.199465	NA	NA
TRIM65	-0.32006	-0.25932	-0.04504	0.131193	0.127058	-0.10219	NA	NA
TRIM66	NA	NA	-0.23501	0.178961	0.090061	-0.11463	NA	NA
TRIM67	-0.06194	0.369317	NA	NA	NA	NA	-0.06782	0.001706
TRIM68	0.242953	0.349102	0.278911	-0.1456	0.284811	0.24833	-0.38591	-0.2831
TRIM69	-0.51675	-0.19843	0.250722	-0.25989	-0.37136	-0.16981	-0.49517	0.100544
TRIM7	0.124895	0.727325	-0.11839	-0.09614	-0.17563	-0.33304	0.01164	-0.2876
TRIM71	NA	NA	NA	NA	NA	NA	0.218329	0.091505
TRIM72	0.209501	0.314951	-0.42605	0.008339	-0.07361	-0.38524	NA	NA
TRIM73	-0.10345	0.483336	-0.07297	0.399499	-0.19981	-0.0851	NA	NA
TRIM74	-0.15801	0.221249	0.273284	-0.11116	0.071001	-0.07101	-0.08509	0.280378
TRIM8	-0.34258	0.002042	0.138359	0.307116	0.188947	0.390872	-0.26049	0.241345
TRIM9	0.166881	-0.00144	-0.05636	-0.07424	-0.06836	-0.18022	-0.46683	-0.47374
TRIML1	-0.09737	0.028458	-0.35251	-0.11611	0.037753	-0.17822	NA	NA
TRIML2	NA	NA	-0.35164	-0.15124	0.097285	0.096287	NA	NA
TRIO	0.032161	0.217614	0.018513	0.016133	0.09327	0.065761	0.500405	0.099836
TRIOBP	-0.11564	0.586878	0.365756	0.037709	0.301679	0.155607	-0.4219	-0.47459
TRIP10	0.219593	0.314401	0.047379	-0.01196	0.166703	0.150054	0.411699	0.248126
TRIP11	-0.27874	-0.06476	-0.20423	0.076348	-0.20693	-0.14811	-0.29562	-0.1527
TRIP12	-0.04708	-0.7054	0.001207	0.006486	0.077156	0.056332	-0.03597	-0.06159
TRIP13	-0.05753	-0.23615	-0.15367	0.313414	0.094347	-0.20354	0.592219	0.143998
TRIP4	-0.0141	-0.58267	-0.38262	-0.26288	-0.21216	-0.47167	0.558921	0.444328
TRIP6	0.287371	-0.06625	-0.06342	-0.01402	-0.01939	0.437584	0.185216	-0.08164
TRIT1	-0.2036	-0.62714	-0.0155	0.033603	0.087861	-0.19148	-0.02556	-0.21491

TRMT1	0.288525	0.504269	-0.03762	0.561533	0.262104	0.453882	-0.2637	0.269052
TRMT11	0.275811	-0.53398	0.00316	-0.06843	0.09267	0.141245	NA	NA
TRMT12	0.433155	0.203086	-0.04856	0.03075	-0.2525	-0.1233	-0.44795	-0.56173
TRMT2A	NA	NA	0.324855	0.109607	0.416254	0.090655	NA	NA
TRMT2B	NA	NA	-0.0119	0.02944	0.651671	0.100922	NA	NA
TRMT5	0.071836	0.042772	-0.33101	0.174727	0.192027	0.133297	0.05988	-0.2793
TRMT6	NA	NA	-0.47202	0.168978	-0.01962	0.086941	NA	NA
TRMT61A	NA	NA	-0.01752	-0.0561	0.207184	0.262993	NA	NA
TRMT61B	NA	NA	-0.20204	0.090619	-0.10032	-0.32512	NA	NA
TRMU	0.051299	-0.32135	-0.24687	0.048285	-0.04476	0.138067	0.141799	-0.03315
TRNAU1AP	NA	NA	-0.06624	-0.04063	0.006552	-0.06248	NA	NA
TRNT1	0.137423	-0.24667	-0.48114	0.059961	0.089783	0.092709	-0.17986	-0.11732
TRO	-0.344	-0.38207	-0.06966	0.05531	-0.13405	0.42109	-0.25406	0.170676
TROAP	-0.05603	0.452534	-0.29638	0.177978	0.163969	0.040175	0.431304	0.406536
TROVE2	0.129713	0.044581	0.156377	0.041543	0.078002	0.158374	0.04881	-0.15134
TRPA1	0.15312	0.418773	0.077059	-0.37929	0.077689	-0.03584	0.139227	0.427853
TRPC1	-0.01229	-0.23117	-0.139	-0.2471	-0.01137	0.01965	-0.24548	-0.07998
TRPC2	NA	NA	0.074277	-0.23847	0.042683	-0.01756	-0.37177	-0.17545
TRPC3	-0.20926	-0.10832	0.145702	-0.10392	-0.21996	-0.11621	0.288646	0.453617
TRPC4	0.156595	0.659301	0.042445	-0.03347	-0.05298	-0.22244	-0.4592	-0.42103
TRPC4AP	0.335731	0.5203	0.286682	0.133133	0.444723	-0.00023	0.391979	0.242953
TRPC5	0.231783	0.584973	0.015726	0.048824	-0.06298	-0.00455	-0.4402	-0.26972
TRPC6	-0.10256	-0.22649	0.156153	-0.0078	-0.27805	-0.14596	-0.34227	-0.11067
TRPC7	0.11473	0.258012	-0.32089	-0.17074	0.20164	0.253767	-0.06522	0.499392
TRPM1	0.139138	0.200856	0.047226	0.425587	-0.0371	0.146235	0.444496	0.065755
TRPM2	0.290936	0.362208	0.050002	-0.20291	0.101635	0.217033	-0.23446	-0.32039
TRPM3	0.342364	0.602896	0.445326	-0.45199	-0.03676	0.248712	-0.26814	-0.40082
TRPM4	-0.47693	0.24483	-0.28677	0.062982	0.086576	-0.04865	-0.33374	-0.32109
TRPM5	-0.23639	0.12071	-0.07123	0.006825	-0.045	0.337543	0.169116	0.302914
TRPM6	0.062826	-0.17097	0.004412	-0.35596	-0.0814	-0.19254	0.080947	-0.09612
TRPM7	-0.21213	0.340447	-0.01378	-0.07775	-0.00898	-0.0922	NA	NA
TRPM8	0.169297	0.025117	0.122691	-0.21301	0.058704	0.010365	-0.41058	-0.34606
TRPS1	-0.30774	-0.24695	-0.11951	-0.04043	-0.20373	0.062655	-0.43447	-0.34571
TRPT1	-0.01789	0.393234	0.085232	0.153787	-0.03285	-0.39193	-0.20746	-0.07972
TRPV1	-0.06171	0.332508	-0.26116	-0.17906	0.052467	0.01337	0.109308	0.197278
TRPV2	-0.3319	-0.3914	0.165348	0.326288	-0.15448	0.507533	-0.07171	0.515887
TRPV3	0.112281	0.537881	-0.0145	0.187379	0.029143	0.079287	-0.44112	-0.11381
TRPV4	-0.15627	0.115827	0.091326	0.041177	0.264027	0.178634	0.234863	0.24034
TRPV5	0.098773	0.669778	0.0781	-0.08061	0.028931	-0.07983	-0.47446	-0.20745
TRPV6	0.196489	0.454301	0.088424	-0.09275	-0.11477	-0.25328	0.325212	0.497306
TRRAP	0.486502	-0.34505	0.075988	0.150565	0.282336	0.357745	0.371458	0.205258
TRUB1	0.014064	0.103973	-0.34459	0.019762	-0.05687	-0.26586	0.329643	0.338033
TRUB2	0.318662	0.546049	-0.28512	0.299411	-0.13012	-0.12928	-0.07367	-0.21347
TSC1	-0.25642	-0.27948	-0.41136	0.067298	-0.04014	-0.24998	-0.57806	-0.28639
TSC2	0.471025	0.309691	0.285057	0.050239	0.425246	0.291587	0.352486	0.077428
TSC22D1	-0.00554	-0.58272	0.396612	-0.01688	0.141672	0.08529	-0.13591	-0.30432
TSC22D2	-0.12267	-0.49217	-0.39893	-0.00479	-0.01064	0.138694	-0.05961	-0.20505
TSC22D3	-0.17801	-0.21957	0.094502	0.176043	-0.09885	-0.05171	0.04461	-0.1861
TSC22D4	0.235477	0.057786	0.266736	0.227596	0.454079	0.073335	-0.1124	-0.11591
TSEN15	NA	NA	-0.11152	-0.16194	-0.16916	-0.34475	NA	NA
TSEN2	0.324516	-0.30616	-0.51131	-0.2373	0.149519	0.308439	0.30545	-0.06019
TSEN34	0.255798	0.087708	0.029486	-0.07522	0.313681	0.181146	0.497555	-0.10475

TSEN54	0.270825	0.508225	0.275817	0.306574	0.338309	0.035808	0.597626	0.407969
TSFM	0.046516	0.263945	-0.61143	0.191687	-0.18273	0.00585	0.450577	0.271884
TSG101	0.05124	-0.44016	-0.01983	-0.35799	0.017696	-0.13808	0.58665	0.501956
TSGA10	0.361079	0.378614	-0.01254	-0.11769	-0.11914	-0.1452	0.349781	0.319822
TSGA10IP	-0.06621	0.584965	0.104658	-0.04237	0.169753	0.028216	-0.48118	-0.34498
TSGA13	0.025524	-0.31518	0.132256	0.008757	-0.08607	-0.17652	-0.14108	-0.0827
TSGA14	0.320188	-0.27015	0.084274	0.054062	-0.06446	-0.10427	0.241854	0.411454
TSHB	0.025115	0.534506	-0.03383	0.210006	-0.00038	-0.07357	-0.19763	-0.2969
TSHR	0.225024	0.034641	0.146321	-0.16081	-0.20194	-0.38197	-0.05679	0.060923
TSHZ1	0.085569	-0.49334	0.323081	0.011871	0.254984	0.121521	NA	NA
TSHZ2	0.011704	-0.12064	0.336063	-0.13922	-0.0574	0.332227	NA	NA
TSHZ3	-0.42113	-0.05144	-0.1689	-0.18497	-0.03431	0.147392	NA	NA
TSKS	-0.12869	-0.03778	0.181945	0.172378	-0.16503	0.007692	-0.00352	0.471538
TSKU	-0.12304	0.601167	0.386255	0.017455	0.065447	-0.10984	NA	NA
TSLP	0.213875	0.421064	-0.04767	-0.02121	0.021087	0.292334	-0.72891	-0.42361
TSN	0.096486	-0.10043	0.014838	0.075403	-0.07241	-0.16556	-0.34592	-0.20199
TSNARE1	0.517298	0.734266	0.080418	-0.05589	0.249866	0.033211	0.004122	-0.26633
TSNAX	-0.45776	-0.38007	-0.11748	-0.15191	-0.10817	-0.17319	-0.30292	-0.1724
TSNAXIP1	0.368094	-0.02881	-0.03976	0.142031	0.322055	0.03475	-0.32145	-0.31687
TSP50	0.159016	0.145147	-0.14057	-0.04533	0.341987	0.041656	-0.30142	-0.4253
TSPAN1	-0.35716	0.095026	0.109919	-0.10952	-0.04852	-0.07464	-0.05171	-0.36776
TSPAN10	0.190198	0.676691	0.033759	-0.00783	-0.13933	0.188347	-0.35912	-0.1201
TSPAN11	NA	NA	0.086043	0.370966	-0.06026	0.454704	-0.06403	0.035822
TSPAN12	0.218294	-0.53714	-0.01837	-0.18942	-0.06719	-0.29296	-0.14092	-0.22479
TSPAN13	-0.52596	-0.39991	-0.14549	0.228886	-0.32744	-0.13888	-0.03479	-0.14263
TSPAN14	-0.38757	-0.07564	0.009316	0.281274	0.295304	0.143069	0.060778	-0.25709
TSPAN15	-0.41701	-0.01248	-0.19146	0.000332	0.084208	-0.06638	-0.14012	-0.08806
TSPAN16	-0.07509	0.636232	0.056189	-0.03105	-0.15421	0.162671	0.480477	0.488892
TSPAN17	-0.1281	0.502426	0.491175	0.121913	0.297312	-0.03485	-0.21594	0.022756
TSPAN18	-0.0995	-0.11492	0.234791	-0.07001	0.046244	0.374452	-0.24874	-0.42774
TSPAN19	NA	NA	-0.03233	-0.10496	-0.26458	-0.07332	NA	NA
TSPAN2	0.065348	-0.20311	0.158735	-0.19731	-0.36612	-0.37364	-0.49327	-0.48881
TSPAN3	-0.30423	-0.42055	-0.13053	-0.02743	-0.09427	-0.32968	0.053751	-0.01285
TSPAN31	0.337465	0.592192	0.284807	-0.07729	-0.07626	-0.45031	-0.19717	0.004177
TSPAN32	-0.01069	0.407464	0.048116	0.218979	-0.24689	0.510757	0.155569	0.280304
TSPAN33	0.494784	0.433681	0.135153	0.558158	0.438586	0.38627	-0.44046	-0.34409
TSPAN4	-0.39312	0.288968	0.254402	0.001546	-0.06504	0.412297	-0.41862	-0.06246
TSPAN5	0.163899	-0.37952	0.141157	0.080304	0.007472	0.011782	0.030878	-0.1572
TSPAN6	0.265233	-0.07193	0.06203	-0.07308	-0.09776	-0.10842	0.139904	-0.149
TSPAN7	0.445709	-0.46046	0.240253	-0.1633	-0.08658	-0.19748	-0.03581	-0.31393
TSPAN8	-0.27491	-0.00016	0.137907	0.045981	-0.25227	-0.22764	0.202868	-0.10597
TSPAN9	-0.36239	-0.18457	0.018302	0.025735	-0.06738	0.273757	-0.15386	-0.02483
TSPO	-0.524	0.415611	0.166734	-0.14903	-0.08124	0.011489	0.060214	-0.07023
TSPY1	0.182826	0.200184	NA	NA	NA	NA	0.120073	-0.1108
TSPY2	-0.27023	0.56701	NA	NA	NA	NA	0.388514	0.169626
TSPYL1	0.041211	-0.56404	-0.35617	0.068595	0.142238	0.130048	0.019193	-0.43069
TSPYL2	0.027249	-0.1427	0.060467	0.236024	0.054598	0.471878	-0.2188	0.017387
TSPYL4	0.131868	-0.1313	-0.08453	0.117114	0.353349	0.324745	0.275227	0.138936
TSPYL5	-0.37008	-0.34807	-0.06316	0.095289	-0.1161	-0.00435	-0.28675	-0.07653
TSPYL6	0.114255	0.563908	-0.21042	0.000682	-0.13293	0.004787	-0.43095	-0.12296
TSR1	-0.43745	-0.38885	-0.47212	0.228955	-0.21191	0.127737	-0.60462	-0.4647
TSR2	0.343821	0.070109	0.066276	0.367578	0.243177	0.009758	NA	NA

TSSC1	0.032735	-0.00448	0.277911	0.407982	-0.0638	-0.3172	0.504883	0.544236
TSSC4	0.209829	0.625844	0.175072	0.135509	0.165569	0.020092	-0.42819	-0.32894
TSSK1B	-0.20918	-0.37123	-0.44	-0.19385	0.008244	0.018142	NA	NA
TSSK2	-0.02775	-0.12878	-0.05576	-0.22225	0.038869	-0.07973	0.213005	0.031516
TSSK3	-0.03758	-0.17506	0.145328	-0.1155	-0.27024	-0.20315	-0.06436	-0.22514
TSSK4	0.106291	0.492383	-0.13923	-0.26807	-0.17169	0.023579	0.306078	0.203298
TSSK6	-0.18752	-0.34536	-0.30295	-0.17112	0.271094	-0.09135	NA	NA
TST	0.016055	0.171479	0.075676	-0.02541	0.073772	-0.17748	0.371999	0.043461
TSTA3	-0.14463	0.468791	0.130415	0.243924	-0.07905	-0.05459	0.070218	-0.23158
TTBK1	0.002012	0.093067	0.061452	-0.11762	-0.04066	-0.4795	-0.2026	0.121239
TTBK2	0.079111	0.682917	0.005587	-0.13231	-0.137	-0.42219	0.025384	0.272357
TTC1	0.35418	0.421573	0.238569	0.184324	-0.19353	-0.29195	0.182153	0.009621
TTC12	0.222666	0.492617	-0.15038	-0.08542	0.074084	-0.25368	0.288383	0.004092
TTC13	0.153124	0.486752	-0.30109	-0.0956	-0.09742	-0.3135	0.347771	0.171105
TTC14	0.103147	-0.4474	0.184273	0.154928	-0.02576	0.442935	-0.3359	0.360704
TTC15	-0.0355	-0.25899	0.409856	0.330952	0.3488	0.184077	0.405201	0.344067
TTC16	0.251219	0.754121	-0.12243	0.457874	0.124892	0.382262	-0.59216	-0.58876
TTC17	0.150084	-0.43579	-0.07157	-0.0176	0.20491	0.147184	0.380566	0.575968
TTC18	0.108447	0.096909	0.03891	-0.08442	0.134608	-0.0249	0.090961	0.308084
TTC19	-0.13204	0.3448	-0.13023	0.278632	0.028727	-0.15443	0.474835	0.439922
TTC21A	-0.05748	-0.3201	-0.40502	0.266071	0.038178	0.001684	-0.0362	0.174265
TTC21B	-0.09969	0.224209	-0.07321	-0.29877	-0.01103	0.164673	-0.28419	0.42922
TTC22	NA	NA	0.21332	-0.15933	-0.10284	-0.36577	0.724079	0.580915
TTC23	0.110032	0.33952	0.252797	0.200596	-0.10952	-0.37503	-0.33472	-0.2257
TTC23L	NA	NA	0.219003	0.170254	-0.04551	0.388656	NA	NA
TTC25	0.394697	-0.10759	0.045323	-0.15263	0.050652	-0.15964	-0.11915	-0.31781
TTC26	0.435861	0.007275	-0.09405	0.039694	-0.18808	0.119671	NA	NA
TTC27	-0.18071	-0.20422	0.064122	0.046413	0.132734	0.307187	NA	NA
TTC28	NA	NA	-0.12474	0.038979	-0.0126	0.245804	NA	NA
TTC29	-0.20937	0.597156	-0.00653	0.034742	-0.19377	-0.02973	NA	NA
TTC3	0.017567	-0.47324	-0.0898	-0.04778	-0.18825	0.027131	0.247669	0.267046
TTC30A	0.228317	0.154245	-0.13382	-0.49012	-0.1857	-0.25172	NA	NA
TTC30B	0.069491	-0.29309	0.010637	-0.21081	-0.24136	-0.22688	NA	NA
TTC31	0.121091	-0.36515	0.268781	0.137161	0.334453	0.144718	NA	NA
TTC32	0.166846	-0.06432	-0.07254	-0.31185	0.105088	-0.28086	-0.25152	0.047257
TTC33	-0.19733	-0.54538	-0.07966	-0.33029	-0.11843	-0.53757	NA	NA
TTC35	0.262625	0.564336	0.144141	-0.26059	-0.18923	-0.2778	NA	NA
TTC37	NA	NA	0.242535	-0.30814	0.082678	-0.07817	NA	NA
TTC38	NA	NA	0.363441	0.040825	0.218741	-0.12149	NA	NA
TTC39A	NA	NA	0.103536	0.105338	0.334364	-0.16478	NA	NA
TTC39B	NA	NA	-0.28416	-0.29005	-0.03633	-0.2749	NA	NA
TTC39C	NA	NA	0.355154	0.477576	0.042868	0.702914	NA	NA
TTC4	-0.01991	0.046299	0.248226	0.048715	0.280559	0.092893	0.105948	0.028717
TTC5	0.056626	0.120159	-0.08579	0.182045	-0.11506	0.11233	0.314901	0.264687
TTC6	0.121381	0.19195	NA	NA	NA	NA	0.335957	0.113275
TTC7A	-0.18109	0.504276	-0.08164	0.296217	-0.02745	-0.28905	0.191526	0.470904
TTC7B	0.109599	0.27175	0.343924	-0.13772	0.150767	0.110614	-0.21544	-0.31043
TTC8	0.130762	0.48186	-0.19288	-0.26759	-0.17147	-0.13475	-0.29767	-0.43432
TTC9	NA	NA	0.295258	0.749786	-0.14523	0.300259	NA	NA
TTC9B	0.24495	0.551197	-0.15465	-0.20166	-0.15659	-0.61256	0.275662	-0.14011
TTC9C	0.031408	-0.25342	0.014566	0.016597	-0.17508	-0.34829	0.200497	-0.10071
TTF1	-0.0073	-0.37395	-0.26343	-0.04085	0.138331	0.266712	-0.45275	-0.44836

TTF2	-0.14654	-0.10199	-0.06157	0.05068	0.3212	-0.06865	0.384919	0.138376
TTK	0.287598	-0.13997	-0.56667	0.070479	-0.01918	-0.1109	0.430434	0.032001
TTL	-0.15911	-0.39231	0.070678	-0.10724	0.099768	0.038632	-0.37998	-0.33763
TTLL1	0.010274	-0.21946	-0.03645	-0.10743	-0.01028	-0.3553	0.087511	-0.1654
TTLL10	0.191185	0.282356	-0.11014	-0.04985	0.008634	-0.15252	0.055578	-0.12482
TTLL11	0.305573	0.010701	-0.00178	-0.32611	0.277998	0.058896	NA	NA
TTLL12	-0.16912	0.428006	-0.13128	0.166853	0.168829	0.035383	0.411105	0.053314
TTLL13	-0.16844	0.566554	NA	NA	NA	NA	-0.19314	-0.04378
TTLL2	0.093025	0.595297	-0.19987	-0.1623	-0.03388	0.40039	0.113681	0.085947
TTLL3	0.196774	0.007593	0.08906	0.23851	0.042345	0.207803	-0.03948	0.325355
TTLL4	0.380931	-0.1858	0.178363	0.11384	0.201941	0.25464	0.624791	0.396118
TTLL5	0.122908	-0.14781	-0.29561	-0.07064	0.050981	-0.20723	-0.01822	0.09325
TTLL6	0.419458	0.100279	0.153158	-0.0642	-0.15933	-0.15314	-0.06172	-0.26428
TTLL7	-0.03569	0.051231	-0.12282	-0.29112	-0.23725	-0.02866	0.078467	0.002979
TTLL9	0.020427	0.517427	NA	NA	NA	NA	0.072476	-0.1256
TTN	0.129876	0.279673	-0.09441	0.401758	-0.23474	0.508018	-0.39693	-0.31989
TTPA	-0.25729	-0.08649	0.04639	0.005027	0.091647	-0.16059	0.299397	0.36264
TTPAL	NA	NA	0.167467	0.097288	0.418744	0.030776	NA	NA
TTR	0.055525	0.030098	-0.13532	0.145816	-0.20278	-0.28839	0.535666	0.382215
TTRAP	-0.14341	-0.29436	-0.5117	0.013871	-0.17707	-0.25825	-0.33119	-0.29621
TTTY1	NA	NA	0.188398	-0.07231	0.279928	-0.04571	-0.26383	-0.41017
TTTY10	NA	NA	0.134692	-0.33706	0.294156	0.021353	-0.20975	-0.20494
TTTY11	NA	NA	0.16819	0.014188	-0.1721	0.207511	0.354614	0.38545
TTTY12	NA	NA	-0.08213	0.009184	-0.24152	-0.21125	-0.08325	-0.20714
TTTY13	NA	NA	0.264629	-0.12643	-0.18512	-0.24003	-0.02397	-0.04165
TTTY14	NA	NA	0.237279	-0.07784	-0.2687	-0.33509	-0.20525	-0.2711
TTTY2	NA	NA	0.112012	-0.06505	-0.09578	-0.2175	0.127445	-0.08178
TTTY5	NA	NA	0.065808	-0.05863	0.38523	-0.00344	0.355605	0.218804
TTTY6	NA	NA	-0.16012	-0.19006	0.036693	-0.22351	0.065208	0.039736
TTTY6B	NA	NA	NA	NA	NA	NA	0.358922	0.10002
TTTY7	NA	NA	0.220363	-0.23558	0.099291	-0.17397	-0.05294	-0.06613
TTTY8	NA	NA	0.008561	-0.13787	-0.06831	0.115456	-0.11379	-0.26564
TTYH1	0.258198	-0.17588	-0.10547	-0.05923	-0.20419	0.042166	0.103104	-0.19039
TTYH2	0.317003	-0.11947	0.145227	0.320538	-0.03728	0.344712	-0.13608	0.150037
TTYH3	-0.26973	0.170685	0.388251	0.098789	0.422613	0.206432	0.494159	0.594574
TUB	-0.51221	0.429221	-0.07862	-0.04182	-0.36281	0.210894	-0.37525	-0.3507
TUBA1A	-0.19096	-0.6358	0.20854	0.063372	0.274047	0.097188	-0.33552	-0.37138
TUBA1B	-0.24418	-0.05828	-0.56254	0.319383	0.099499	0.051795	NA	NA
TUBA1C	0.010017	0.084961	-0.06864	0.218098	0.272415	0.097352	NA	NA
TUBA3C	0.153151	0.12214	-0.00683	0.231887	0.420287	0.129736	NA	NA
TUBA3D	NA	NA	0.147748	-0.11296	0.118252	0.085226	-0.45571	-0.42353
TUBA3E	0.127228	0.643437	NA	NA	NA	NA	NA	NA
TUBA4A	0.217522	0.083647	0.012076	0.274134	0.327132	0.048403	NA	NA
TUBA8	-0.00357	0.472049	-0.39138	-0.217	-0.07693	-0.38471	0.546363	0.196819
TUBAL3	0.232178	0.110409	0.303204	-0.27803	0.126328	-0.29732	0.027692	0.148521
TUBB	-0.2282	-0.36755	0.183892	0.315749	0.151811	0.091541	0.421147	0.114143
TUBB1	-0.04628	0.39066	-0.13732	-0.11359	-0.06401	-0.06772	NA	NA
TUBB2A	NA	NA	0.058075	0.141052	0.153013	-0.00859	-0.38908	-0.15066
TUBB2B	0.09296	-0.03913	-0.11748	0.252859	0.126481	0.266203	0.068894	-0.23439
TUBB2C	-0.19416	-0.30994	0.048158	0.058262	0.186006	0.300986	0.351169	0.122363
TUBB3	0.067478	-0.09595	0.190411	0.337694	0.239986	0.084209	0.213111	-0.28633
TUBB4	0.013915	-0.147	0.299595	0.10556	0.162974	0.142329	-0.54851	-0.39959

TUBB4Q	-0.16196	0.291951	NA	NA	NA	NA	-0.1815	-0.35754
TUBB6	-0.39325	-0.38671	0.019722	0.115066	0.084334	0.299699	-0.22572	-0.06774
TUBB8	-0.07704	-0.39871	NA	NA	NA	NA	-0.11774	-0.19709
TUBD1	0.158918	0.198866	0.171528	-0.28448	0.008578	0.022457	-0.5939	-0.43769
TUBE1	0.121473	-0.60615	-0.18913	0.013692	0.08782	0.072651	-0.34328	0.020862
TUBG1	-0.18221	0.050715	0.423825	0.156786	0.186427	-0.06151	0.037379	-0.19137
TUBG2	0.154508	0.137588	0.396295	0.160045	0.262439	0.014065	-0.12671	-0.24148
TUBGCP2	-0.23662	-0.13431	0.204087	0.15016	0.287012	0.289234	0.207426	0.35124
TUBGCP3	0.096322	-0.70341	-0.09144	0.16812	0.256813	-0.12111	0.445944	0.237294
TUBGCP4	NA	NA	-0.12328	-0.02517	-0.05558	-0.01488	NA	NA
TUBGCP5	-0.11714	-0.64796	-0.21023	0.167932	-0.14253	0.008835	-0.41768	-0.40907
TUBGCP6	0.20765	0.373484	0.236639	0.226473	0.324547	0.517317	-0.2006	0.171528
TUFM	0.656429	0.275357	0.204422	0.191767	0.334574	0.120384	0.473644	-0.04118
TUFT1	0.20738	-0.07774	-0.11839	-0.4878	0.133623	-0.16258	0.15462	-0.23184
TULP1	-0.05133	0.322511	-0.17059	0.125955	0.301357	-0.03358	-0.13616	-0.07025
TULP2	0.184128	0.662118	0.029512	-0.03922	-0.21758	-0.4323	0.137393	0.114046
TULP3	0.489618	0.298488	0.189904	-0.13362	0.035864	0.0093	0.303283	0.16078
TULP4	0.409165	-0.46445	0.011921	-0.09204	0.473099	0.111078	0.702806	0.552603
TUSC1	0.003795	-0.51773	-0.25484	0.044703	-0.14184	-0.31392	0.332746	0.395256
TUSC2	0.086986	-0.47996	0.0748	0.089069	-0.06313	-0.12509	-0.16426	-0.14707
TUSC3	-0.13428	-0.48651	0.225565	0.182948	-0.08913	-0.37101	-0.2768	-0.09465
TUSC4	-0.21044	0.326962	-0.0838	0.219924	0.21171	-0.24073	0.259965	0.094972
TUSC5	0.13321	-0.08406	-0.02129	-0.03217	0.06221	0.075762	0.108252	0.198404
TUT1	0.155118	0.42956	0.3285	0.328637	0.322267	0.159845	NA	NA
TWF1	-0.19976	-0.3059	-0.32238	-0.16455	-0.09695	-0.33747	NA	NA
TWF2	-0.15373	-0.05492	0.363573	-0.02904	0.27473	-0.0125	NA	NA
TWIST1	-0.14548	-0.1924	0.051156	0.068869	-0.04461	0.311215	-0.10805	-0.2908
TWIST2	NA	NA	0.139127	0.098207	-0.09653	0.119419	NA	NA
TWISTNB	0.497189	-0.11438	-0.1963	-0.40069	0.008644	0.193126	0.201849	0.044808
TWSG1	-0.25227	-0.3865	-0.39237	-0.05982	-0.33384	-0.514	-0.24476	-0.2458
TXK	-0.0185	0.37196	0.149993	0.496348	-0.09871	0.678177	-0.17695	0.159648
TXLNA	-0.42493	0.324458	0.377077	0.061096	0.234011	0.243662	-0.47361	-0.24963
TXLNB	0.306095	0.369593	-0.01072	0.343587	-0.11789	0.138277	0.220103	0.108891
TXN	-0.15865	-0.53098	-0.15819	-0.29742	-0.2008	-0.38709	-0.16122	-0.31585
TXN2	0.228934	0.634691	0.184587	0.248963	0.126422	-0.1038	0.15036	-0.23011
TXNDC10	-0.06677	-0.58978	-0.11572	-0.08205	-0.11102	-0.18517	0.226708	0.234478
TXNDC11	-0.209	-0.00529	0.339104	0.193282	0.191628	0.104558	0.238931	0.382603
TXNDC12	-0.30587	0.247794	0.053995	0.174208	0.088706	-0.49781	0.561486	0.126072
TXNDC13	0.06436	-0.16885	-0.07509	0.065403	-0.16508	-0.00396	-0.32279	-0.40891
TXNDC14	-0.10396	-0.16869	0.008247	0.456521	-0.19333	-0.48028	0.155224	0.411371
TXNDC15	NA	NA	0.36297	0.334526	-0.16626	-0.4964	NA	NA
TXNDC16	NA	NA	-0.29292	-0.36745	0.061856	0.04544	NA	NA
TXNDC17	NA	NA	-0.34471	0.001597	-0.41272	-0.44217	NA	NA
TXNDC2	0.160151	0.516179	-0.10668	0.183708	-0.27345	-0.27438	NA	NA
TXNDC3	0.05222	0.199704	-0.15745	-0.03733	-0.34106	-0.18106	-0.45751	0.524094
TXNDC4	-0.15739	0.483767	0.110948	0.136605	-0.13314	-0.13997	-0.56086	-0.33211
TXNDC5	-0.15251	0.026627	0.066209	0.186848	0.138694	0.062089	0.477891	0.2518
TXNDC6	-0.22823	0.15946	-0.10536	0.116094	0.029238	0.310576	-0.39836	-0.34577
TXNDC8	0.172789	-0.23476	NA	NA	NA	NA	0.208936	0.082616
TXNDC9	0.349307	-0.19354	-0.03781	-0.19178	-0.16423	-0.42496	-0.10573	-0.30039
TXNIP	-0.36192	-0.06742	-0.03647	-0.31106	-0.17727	-0.24085	-0.57665	-0.73981
TXNL1	-0.06826	-0.29443	0.123225	0.180322	-0.03024	-0.0738	-0.43059	-0.47086

TXNL4A	-0.02469	0.384418	-0.216	0.318118	-0.31377	-0.45026	0.374912	0.222893
TXNL4B	-0.05132	0.266018	0.006089	0.062507	0.30617	0.055076	0.560954	0.439642
TXNRD1	-0.15478	-0.20265	-0.24741	-0.11841	0.090036	-0.53626	0.012025	0.07583
TXNRD2	0.078351	0.564199	0.163194	0.075869	0.41755	0.136606	-0.1333	-0.49629
TXNRD3	NA	NA	0.030789	0.063101	0.001701	-0.0949	NA	NA
TYK2	0.083693	0.630246	0.063907	0.404309	0.254544	0.104548	-0.3447	0.181728
TYMP	NA	NA	0.293609	0.081879	-0.09036	0.491282	NA	NA
TYMS	-0.15913	-0.23814	-0.4666	0.335961	-0.23112	-0.01286	0.209771	0.15828
TYR	0.134429	0.464137	-0.05208	0.028055	-0.20838	0.040846	0.302468	0.15347
TYRO3	-0.23686	-0.16633	0.15819	0.178812	0.007818	0.030565	0.28153	0.171892
TYROBP	-0.41153	-0.07434	0.303351	-0.30267	-0.25247	0.120504	-0.48463	0.124907
TYRP1	0.111693	-0.37462	-0.07655	0.321784	-0.24077	0.019797	0.027048	0.253615
TYSND1	-0.17798	0.312097	0.252005	0.058172	0.245351	0.070736	0.175175	0.330338
TYW1	0.337608	-0.48218	-0.14083	-0.19754	-0.08986	0.106876	NA	NA
TYW3	-0.11619	0.101468	-0.03478	-0.32919	-0.15129	-0.29133	NA	NA
U2AF1	0.091472	-0.24097	-0.32506	0.301131	0.030445	0.351745	-0.22634	-0.22211
U2AF1L4	0.341183	0.627977	-0.09987	0.336401	-0.19018	-0.18304	-0.07506	0.224381
U2AF2	0.379172	0.222128	0.197763	0.373288	0.004553	0.054584	-0.16119	-0.17439
UACA	0.01704	-0.42787	-0.05261	-0.11496	-0.05775	-0.08901	0.019948	0.371634
UAP1	-0.27598	-0.25565	-0.2234	-0.06238	-0.0589	-0.26902	-0.41342	-0.54245
UAP1L1	-0.3893	-0.2774	0.32102	0.138043	0.329371	0.375431	-0.14845	0.29261
UBA1	NA	NA	0.140872	0.259319	0.167349	0.170333	NA	NA
UBA2	NA	NA	-0.27774	0.06442	-0.03766	-0.04518	-0.37555	-0.19848
UBA3	NA	NA	-0.20406	-0.07002	-0.06137	-0.22648	NA	NA
UBA5	NA	NA	-0.4828	0.122891	-0.05046	-0.31755	NA	NA
UBA52	0.4774	0.547915	0.061779	-0.06103	-0.11684	-0.10546	0.025768	-0.09786
UBA6	NA	NA	0.065977	-0.29842	0.010086	0.265494	NA	NA
UBA7	NA	NA	0.290503	0.036096	0.168432	0.293755	NA	NA
UBAC1	NA	NA	0.336487	0.092991	0.29709	-0.12625	NA	NA
UBAC2	NA	NA	0.329232	0.126834	0.394019	-0.10864	NA	NA
UBAP1	-0.00351	-0.26816	0.018677	-0.02428	-0.153	-0.09487	0.235288	0.243095
UBAP2	0.167242	0.700741	-0.10127	0.175496	0.053289	0.261753	-0.07882	-0.30063
UBAP2L	0.180751	-0.06797	-0.19053	0.28381	-0.01431	0.118477	0.431268	0.099803
UBASH3A	0.228159	0.426076	0.122446	0.61957	-0.10507	0.807815	-0.57385	0.31009
UBASH3B	NA	NA	0.040108	0.1207	-0.29608	0.294233	NA	NA
UBB	-0.28899	-0.6971	0.116671	-0.05547	-0.31895	-0.16649	0.229883	0.199195
UBC	NA	NA	0.226696	-0.23326	0.019916	-0.04388	0.426032	0.302061
UBD	0.308427	-0.13939	-0.03056	0.500629	-0.25884	0.638732	-0.30037	0.005631
UBE2A	-0.24177	-0.47855	0.145818	-0.40156	0.176703	0.061012	0.297173	0.255492
UBE2B	0.10656	-0.12367	0.157706	-0.37648	-0.00178	-0.22097	-0.20668	-0.13907
UBE2C	0.156549	0.224331	-0.06764	0.297492	0.174008	-0.10619	0.538703	0.069995
UBE2D1	0.440579	-0.21813	-0.40102	-0.42063	-0.07495	-0.36792	0.09462	0.026179
UBE2D2	0.196328	0.657653	0.277353	-0.04174	0.02056	-0.09465	-0.28027	-0.59406
UBE2D3	-0.36835	0.233047	-0.22305	-0.15097	-0.22858	-0.25287	0.141095	0.50888
UBE2D4	0.684431	0.130448	0.17593	-0.10657	-0.18318	-0.32784	-0.50387	-0.44059
UBE2E1	0.55194	0.016495	0.045664	-0.11489	-0.17343	0.168949	-0.36989	-0.47535
UBE2E2	-0.07947	-0.01819	0.07881	0.110011	-0.30182	0.002909	-0.45345	-0.22992
UBE2E3	0.183876	-0.40893	-0.18548	-0.07302	-0.12384	-0.00052	0.610485	0.396986
UBE2F	0.14032	-0.11878	0.084482	0.193691	-0.28696	-0.51126	0.272496	0.274718
UBE2G1	-0.3288	-0.51907	-0.16789	-0.26653	-0.48003	-0.25484	0.140014	-0.03651
UBE2G2	0.069172	0.187809	0.10107	0.111438	0.158385	0.371981	-0.46546	-0.23186
UBE2H	-0.2003	-0.67315	-0.15213	-0.20584	-0.07709	-0.22974	-0.54609	-0.42161

UBE2I	0.294751	0.525478	-0.11936	0.362891	0.215191	0.210305	0.627613	0.110921
UBE2J1	0.01267	0.396048	-0.07404	0.021451	-0.0613	-0.1783	-0.00371	-0.0049
UBE2J2	-0.04716	0.585527	0.239179	0.101443	0.217805	0.052542	-0.23123	-0.15305
UBE2K	NA	NA	-0.20125	-0.1413	-0.0391	-0.00726	NA	NA
UBE2L3	-0.03197	0.361672	-0.09516	-0.23513	-0.12149	-0.35428	0.231802	0.087575
UBE2L6	0.050559	-0.05614	0.388484	-0.17501	-0.18293	0.052314	-0.3227	0.095197
UBE2M	0.124703	0.526193	0.126561	0.07225	0.213856	-0.05527	0.154831	-0.223
UBE2MP1	NA	NA	0.239677	-0.12044	0.194497	0.038037	-0.18628	0.083538
UBE2N	0.063224	-0.05407	-0.23577	-0.04262	0.19351	-0.24569	-0.20618	-0.05239
UBE2NL	0.263448	-0.3074	-0.30189	0.024066	0.00985	-0.39277	-0.57139	-0.61171
UBE2O	-0.04861	0.264856	0.255741	0.153463	0.067164	0.20667	0.469345	0.2114
UBE2Q1	0.123227	-0.38344	0.001929	0.091531	0.145604	-0.14092	0.024781	-0.03392
UBE2Q2	-0.11805	-0.21284	-0.33379	0.028642	-0.29681	-0.29103	-0.40854	-0.47486
UBE2R2	-0.02039	-0.54117	0.044348	0.015922	0.137698	0.256999	-0.32244	-0.39378
UBE2S	NA	NA	-0.18281	0.331629	0.097006	0.086273	0.137062	0.224588
UBE2T	-0.30831	-0.41287	-0.20853	0.011734	0.024859	-0.20715	0.250801	-0.06833
UBE2U	0.008811	0.104375	0.142928	0.135245	-0.21811	-0.15961	0.462022	0.323067
UBE2V1	0.397449	-0.4352	-0.01088	0.107553	0.070628	-0.09386	-0.03006	-0.38389
UBE2V2	-0.21045	-0.65239	-0.32253	-0.12231	-0.29562	-0.33362	-0.0142	-0.22538
UBE2W	-0.10637	0.079043	0.167988	-0.30237	-0.27843	0.024197	-0.13719	-0.13365
UBE2Z	-0.25554	-0.42843	0.352778	0.378839	0.104784	0.146301	0.299834	0.236133
UBE3A	0.037636	0.075304	0.01236	-0.1504	-0.03709	0.382746	-0.29727	-0.20953
UBE3B	0.001467	-0.17697	-0.31013	0.26423	0.021718	-0.17082	-0.66508	-0.54071
UBE3C	0.025947	-0.38418	0.020852	0.080002	0.268592	-0.34145	-0.07083	-0.12013
UBE4A	-0.18719	-0.5248	-0.12314	-0.21204	-0.16064	-0.0303	-0.32131	-0.24449
UBE4B	-0.4327	-0.19635	-0.02455	-0.05668	0.164026	0.11074	-0.44994	-0.58035
UBFD1	0.482873	-0.25029	-0.03299	0.195082	0.090583	-0.24627	NA	NA
UBIAD1	0.097391	0.787454	-0.17145	0.365917	0.066032	-0.01183	-0.28074	-0.41867
UBL3	0.194679	-0.29706	-0.10922	-0.48547	0.224337	-0.49993	0.215732	-0.21244
UBL4A	0.183954	0.346357	0.116745	0.01446	0.301029	-0.02069	0.412483	0.065525
UBL4B	0.111424	-0.13915	0.080006	-0.16584	0.31555	0.020336	0.160796	0.324163
UBL5	0.593022	0.017669	-0.42214	-0.17764	-0.09433	-0.60158	-0.48865	-0.37253
UBL7	0.325492	0.481633	0.268807	0.225754	0.03936	-0.15995	0.362441	0.182542
UBLCP1	0.098928	-0.66614	0.396505	0.084444	-0.43894	-0.09554	-0.56316	-0.23795
UBN1	-0.35716	0.309955	0.272943	-0.01394	0.407155	0.290501	0.022362	-0.03546
UBN2	NA	NA	-0.27537	-0.00899	-0.04426	0.026613	NA	NA
UBOX5	0.147648	0.373165	-0.2901	-0.10002	0.113449	0.203073	0.221589	-0.15196
UBP1	0.192432	-0.60513	-0.29443	0.009331	0.016703	0.09388	0.504338	0.416211
UBQLN1	-0.1071	-0.32558	-0.24874	-0.04771	-0.07897	0.001645	0.373538	0.334808
UBQLN2	0.147043	-0.41867	-0.07768	-0.03454	0.264008	0.252571	-0.30026	-0.13272
UBQLN3	-0.12578	0.100373	0.009259	-0.08008	-0.00677	-0.43803	-0.1971	-0.02955
UBQLN4	-0.2257	0.568458	-0.06081	0.418435	0.188914	-0.04725	0.261506	0.000873
UBQLNL	0.274622	0.511493	-0.21145	-0.13349	-0.33301	0.038773	NA	NA
UBR1	-0.32553	0.354986	0.015537	-0.24287	-0.29728	-0.06278	0.441187	0.446402
UBR2	-0.05873	-0.13349	-0.19056	-0.3932	-0.05046	-0.22143	-0.30925	-0.28168
UBR3	NA	NA	-0.00704	-0.38293	-0.09056	-0.17206	NA	NA
UBR4	NA	NA	0.092005	0.137351	0.364977	0.624649	NA	NA
UBR5	NA	NA	-0.14253	0.174476	-0.09219	0.132869	NA	NA
UBR7	NA	NA	-0.31167	-0.04491	-0.02534	-0.4228	NA	NA
UBTD1	-0.10322	-0.2151	0.409389	-0.09395	0.111441	0.154929	0.11027	0.169388
UBTD2	-0.00369	-0.50072	-0.01503	0.110567	-0.20987	-0.3644	NA	NA
UBTF	0.208887	0.170898	0.194043	0.30585	0.400494	0.247612	0.089614	-0.14197

UBXN1	NA	NA	0.252378	0.352438	0.20422	0.285683	NA	NA
UBXN10	NA	NA	0.170569	-0.35356	0.36617	-0.42502	NA	NA
UBXN11	NA	NA	0.181009	0.217624	0.219867	-0.0524	NA	NA
UBXN2A	NA	NA	-0.23905	-0.21721	-0.21057	-0.24789	NA	NA
UBXN2B	NA	NA	-0.01195	-0.20165	-0.38414	-0.42678	NA	NA
UBXN4	NA	NA	0.119535	0.135851	-0.0862	0.004973	NA	NA
UBXN6	NA	NA	0.356997	0.006714	0.091757	-0.08037	NA	NA
UBXN7	NA	NA	0.141095	0.091222	0.150173	0.175409	NA	NA
UBXN8	NA	NA	0.224546	-0.05032	-0.21643	-0.4578	NA	NA
UCA1	NA	NA	0.223757	-0.16797	-0.37215	-0.11226	NA	NA
UCHL1	0.113493	0.316099	0.018741	0.258233	-0.21181	-0.13524	-0.36914	-0.44491
UCHL3	0.488369	-0.40908	0.138578	-0.0103	0.18802	-0.29661	0.485622	-0.14206
UCHL5	0.109959	-0.38031	-0.19387	-0.06072	-0.28909	-0.26903	0.477763	0.034803
UCHL5IP	0.248479	-0.02794	-0.38492	0.365376	0.235489	-0.17883	0.000598	0.256085
UCK1	0.175343	-0.3486	-0.00949	0.018252	0.276745	-0.13473	-0.02983	-0.20438
UCK2	0.122356	-0.046	-0.22861	-0.02663	-0.37074	-0.31769	0.736251	0.27389
UCKL1	0.482043	0.286285	0.361732	0.082339	0.469757	0.212381	0.552766	0.501974
UCN	-0.0507	0.049105	0.237295	-0.12966	0.086557	0.115805	-0.39199	0.028907
UCN2	-0.16399	-0.16977	-0.17894	0.014174	-0.15514	0.156112	-0.17084	-0.35011
UCN3	0.306332	0.383279	0.382123	0.010081	-0.211	-0.19809	-0.62579	-0.21632
UCP1	0.296977	0.462648	0.326642	-0.1086	-0.06507	-0.20714	NA	NA
UCP2	0.041057	0.178667	0.108227	0.597432	-0.10219	-0.10911	-0.05374	0.197235
UCP3	-0.21184	0.44076	-0.20258	-0.11831	-0.2605	0.026255	-0.58244	-0.18307
UCRC	-0.30588	0.03887	0.068568	-0.33598	-0.27203	-0.33376	0.080461	-0.11805
UEVLD	-0.08266	-0.22712	-0.28024	-0.28133	-0.07171	-0.26921	-0.33164	-0.11178
UFC1	0.116039	0.633464	0.170772	0.102331	-0.15897	-0.39584	0.638187	0.229504
UFD1L	-0.24015	0.554473	-0.18099	-0.05026	-0.10214	-0.40652	0.310938	0.312962
UFM1	-0.1494	0.6373	-0.23017	0.011766	-0.05286	-0.3222	-0.24483	-0.30138
UFSP2	NA	NA	-0.05495	-0.05417	-0.12883	-0.41643	NA	NA
UGCG	-0.03888	0.214902	0.393463	0.06271	0.096331	0.385072	-0.53575	-0.41879
UGCGL1	0.183379	-0.27496	0.058635	0.253561	0.083694	-0.32159	-0.07203	-0.01383
UGCGL2	0.258027	-0.04653	0.091729	-0.44521	0.321979	-0.04436	-0.09984	-0.07606
UGDH	-0.25259	0.136845	0.010135	-0.06887	-0.17902	0.096598	0.208	-0.14937
UGP2	-0.13961	-0.24291	-0.3832	-0.22813	-0.22164	-0.36345	-0.09064	-0.42132
UGT1A1	0.076052	0.002857	NA	NA	NA	NA	0.013102	0.132965
UGT1A10	0.166966	-0.16789	NA	NA	NA	NA	0.158984	0.202817
UGT1A3	0.416913	0.513373	NA	NA	NA	NA	-0.02876	0.187942
UGT1A4	-0.53247	0.400819	NA	NA	NA	NA	-0.2925	0.225674
UGT1A5	0.128138	0.411783	NA	NA	NA	NA	-0.14259	-0.12582
UGT1A6	0.253468	0.06626	-0.2964	-0.35698	-0.11744	0.052857	0.211851	0.242561
UGT1A7	0.128488	-0.05821	NA	NA	NA	NA	0.136775	0.097018
UGT1A8	0.245166	0.081643	-0.27451	-0.31988	-0.12815	-0.05356	0.07335	0.091311
UGT1A9	0.423151	-0.13707	NA	NA	NA	NA	0.11071	0.421342
UGT2A1	0.093509	-0.16734	0.253451	-0.20623	0.098239	0.056879	0.336666	0.420021
UGT2A3	0.356662	-0.15092	-0.3361	-0.13787	-0.29535	-0.12162	0.638821	-0.05466
UGT2B10	-0.12089	-0.05271	-0.29747	-0.12054	-0.11619	-0.32568	0.08204	-0.38308
UGT2B11	-0.1044	0.033449	-0.28817	-0.05677	-0.07888	-0.31859	0.218991	-0.08312
UGT2B15	-0.0193	0.284657	-0.23989	-0.0373	-0.13587	-0.24908	0.037895	-0.25446
UGT2B17	0.029612	0.312093	-0.25594	-0.04436	-0.09946	-0.22495	-0.02088	-0.2888
UGT2B28	0.238917	0.204516	-0.23825	-0.38636	-0.01596	-0.30319	0.298226	-0.13063
UGT2B4	0.451438	0.01783	-0.16661	-0.19521	-0.09487	0.129716	0.540922	0.270945
UGT2B7	-0.52191	-0.27919	-0.25232	-0.41271	0.039452	-0.09663	0.23316	-0.20499

UGT3A1	-0.12477	0.426196	0.380111	-0.00462	0.134629	0.170955	0.414857	0.356527
UGT3A2	0.092186	0.539168	0.206861	0.075069	-0.14739	0.047451	0.366717	0.19218
UGT8	0.278411	0.586347	-0.03328	0.021693	-0.17135	0.037096	-0.15217	-0.59501
UHMK1	0.033801	-0.00993	0.024395	0.053028	0.192794	0.018927	-0.18152	-0.253
UHRF1	-0.10111	-0.60801	-0.44322	0.309349	0.298201	-0.21535	0.140902	0.360279
UHRF1BP1	NA	NA	0.007577	-0.36226	0.109274	0.009566	NA	NA
UHRF1BP1l	NA	NA	0.133454	-0.0062	-0.0979	0.111465	NA	NA
UHRF2	-0.16996	-0.63907	-0.28757	0.164852	-0.01943	-0.13805	-0.56294	-0.28182
UIMC1	0.16712	0.316381	0.181967	0.162711	0.209517	0.253714	NA	NA
ULBP1	0.29357	-0.25915	0.059292	0.107168	0.309136	-0.36596	0.079045	0.282432
ULBP2	-0.25213	0.204459	-0.19234	0.033923	0.052305	0.024267	NA	NA
ULBP3	0.314772	0.800513	-0.06762	0.169443	-0.26784	-0.0907	0.18251	0.089807
ULK1	-0.44472	0.11908	-0.02026	-0.02839	0.241672	0.148911	-0.25298	-0.17412
ULK2	-0.23909	-0.20151	-0.11487	-0.08764	0.094659	-0.03031	-0.73599	-0.65001
ULK3	0.022593	0.058103	0.16785	-0.01195	0.127391	-0.22054	-0.07831	0.397705
ULK4	NA	NA	-0.24473	0.091234	0.033979	-0.13299	NA	NA
UMOD	-0.27198	0.62699	0.011884	-0.31528	0.060548	-0.15025	0.134129	-0.10973
UMODL1	0.453362	0.171958	-0.08813	-0.14205	0.222707	-0.16212	-0.13488	0.007035
UMPS	0.085061	0.683189	-0.33241	-0.10198	-0.16339	-0.14419	0.313775	0.665053
UNC119	-0.09708	0.345915	0.443701	0.100824	0.133155	-0.01182	-0.34	-0.29547
UNC119B	NA	NA	0.013261	-0.00615	0.231204	0.24595	NA	NA
UNC13A	NA	NA	0.156018	0.403452	0.272055	0.228852	-0.37101	-0.25798
UNC13B	-0.15128	0.256715	-0.00647	0.129009	0.16109	0.009881	-0.13252	-0.16187
UNC13C	NA	NA	0.194833	0.235523	-0.02206	0.12778	-0.04206	-0.13881
UNC13D	-0.18276	-0.218	0.056386	-0.0041	0.014412	0.084335	0.007917	0.503751
UNC45A	-0.05758	-0.03942	0.320266	0.047441	0.422045	0.298598	-0.02199	-0.38443
UNC45B	-0.03152	0.522848	-0.047	0.020385	0.054737	-0.18178	-0.34991	-0.15033
UNC50	0.093578	-0.69856	0.15811	-0.02126	-0.00165	-0.08913	0.419825	0.30421
UNC5A	-0.17199	-0.18245	0.421315	-0.46124	0.180363	0.04571	-0.12234	-0.08035
UNC5B	-0.22595	-0.02432	-0.05122	-0.01699	-0.0807	-0.14476	0.287529	0.193574
UNC5C	0.096544	0.506367	-0.05929	-0.43177	-0.01684	-0.29674	0.43225	0.150036
UNC5CL	0.031161	-0.27739	-0.15402	0.237627	0.151261	0.160651	0.14676	0.474931
UNC5D	0.455467	0.187395	-0.01108	0.432958	-0.16762	0.159554	0.070241	0.635072
UNC84A	-0.08891	-0.7208	-0.36617	-0.40614	0.230302	0.084202	0.576837	0.405366
UNC84B	-0.63497	-0.08385	0.321875	0.234297	0.383952	0.117892	-0.46325	-0.07962
UNC93A	0.442585	0.533891	-0.24411	0.053535	0.105027	-0.20176	0.217927	0.406701
UNC93B1	-0.12369	-0.02825	0.216854	0.056872	0.19305	-0.1939	0.072267	0.338366
UNG	-0.45116	-0.02329	-0.14585	0.354067	0.092392	-0.03554	0.227754	-0.04422
UNK	NA	NA	-0.19571	0.202683	-0.10415	0.027764	0.410685	0.283341
UNKL	NA	NA	-0.00966	0.037948	0.468107	0.22365	0.586725	0.455337
UNQ6975	NA	NA	0.141401	-0.13406	-0.1713	-0.1215	NA	NA
UPB1	0.007133	0.227738	-0.20856	0.262348	-0.08037	0.408198	-0.52901	0.205478
UPF1	0.131719	0.468914	-0.21263	0.212386	0.356384	0.34085	NA	NA
UPF2	-0.41745	-0.62846	-0.27021	-0.03576	0.014051	0.171568	-0.40041	-0.11584
UPF3A	0.073799	-0.58944	0.090395	0.184964	0.489009	0.120972	0.225825	0.147248
UPF3B	0.261803	0.296009	-0.2638	0.134572	0.089094	-0.13652	0.148695	0.271747
UPK1A	-0.14154	-0.04145	-0.0171	0.276742	0.061372	-0.2257	0.386692	0.094937
UPK1B	-0.15009	0.406935	-0.02133	0.045668	-0.12407	-0.25407	-0.02328	-0.38691
UPK2	-0.02551	0.471793	0.254844	-0.02216	-0.12173	-0.31342	-0.30185	-0.03132
UPK3A	-0.16073	-0.17542	-0.3075	-0.12187	0.003604	-0.39525	-0.13446	0.120851
UPK3B	0.241841	0.717835	0.179584	-0.09652	0.163294	0.033072	-0.17186	0.082696
UPP1	-0.38249	0.54456	-0.02803	-0.13964	-0.1202	-0.05319	-0.43302	-0.0226

UPP2	0.242355	0.634365	-0.29078	-0.17003	-0.06658	-0.3773	0.347463	0.042304
UPRT	0.219631	0.476282	-0.1853	0.129007	-0.18429	-0.42744	0.116659	0.1864
UQCC	NA	NA	0.062288	-0.01415	0.342681	-0.2978	NA	NA
UQCR	0.324204	0.184931	-0.13404	-0.32208	-0.29357	-0.46108	-0.17441	-0.38453
UQCRB	-0.0284	-0.62171	0.260998	-0.30637	-0.35916	-0.37307	-0.50715	-0.6657
UQCRC1	0.052763	0.24098	0.097982	0.210995	0.096154	-0.34834	0.262619	-0.03659
UQCRC2	0.075152	-0.48844	-0.06351	-0.08303	0.070596	-0.27855	-0.15355	-0.44638
UQCRFS1	0.063833	-0.32795	-0.41223	-0.11947	-0.39836	-0.45772	0.373634	0.256145
UQCRH	-0.02745	-0.29486	0.113259	-0.08019	0.1875	-0.55326	-0.08867	-0.24177
UQCRQ	0.204623	-0.41527	0.432034	-0.24027	-0.00509	-0.38175	NA	NA
URB1	NA	NA	0.141087	0.098347	0.259657	0.400822	NA	NA
URB2	NA	NA	-0.33544	0.027927	0.046387	0.111155	NA	NA
URG4	0.225222	0.68151	0.288679	-0.30481	0.296344	0.330339	0.282403	0.319988
URM1	0.179174	0.452162	0.117006	0.063415	-0.01012	-0.42014	NA	NA
UROC1	-0.18432	0.417879	-0.13364	-0.21519	0.363249	0.068068	0.013764	-0.23903
UROD	-0.44119	-0.31426	0.152596	-0.30384	-0.0779	-0.27548	-0.4304	-0.35012
UROS	-0.01268	0.384815	-0.18311	0.051307	-0.03065	-0.35804	-0.17718	-0.08566
USE1	NA	NA	0.127272	-0.04281	0.061707	-0.02575	NA	NA
USF1	-0.24887	0.427663	-0.08283	0.030687	-0.12847	-0.06045	-0.17197	0.31358
USF2	0.546834	0.2396	0.189524	0.31782	-0.02667	0.001001	-0.30674	-0.28146
USH1C	0.579458	0.0811	0.350537	-0.06018	0.304553	0.18612	0.52334	0.113655
USH1G	-0.13705	0.615213	0.274971	-0.26123	0.293838	0.143071	-0.39347	-0.26916
USH2A	0.261696	0.63215	-0.06684	-0.20836	0.035864	-0.25882	-0.42057	-0.17207
USHBP1	-0.05632	0.568458	0.143121	0.070865	-0.12573	0.096604	-0.16319	0.027693
USMG5	0.127719	-0.33388	-0.02673	-0.35434	-0.07086	-0.2316	-0.04117	0.166511
USO1	NA	NA	-0.02091	-0.05371	-0.15777	-0.23054	NA	NA
USP1	-0.04152	0.221983	-0.16757	-0.10438	0.186038	0.096782	-0.09948	-0.28023
USP10	0.046954	-0.69125	-0.07278	0.158476	0.046832	0.081201	0.081038	-0.11324
USP11	0.282944	0.269653	-0.05552	0.438268	0.192852	0.636424	0.048515	-0.09068
USP12	-0.04245	-0.11694	-0.08374	-0.1379	0.068869	-0.35572	-0.31059	-0.3521
USP13	-0.02219	-0.26704	-0.17299	-0.05506	-0.10168	-0.05391	-0.0709	-0.16002
USP14	-0.20752	-0.21994	-0.19479	0.160842	-0.21086	0.426572	0.037361	0.120582
USP15	-0.0169	-0.56781	0.059576	-0.29712	-0.07074	0.030298	-0.43845	-0.08602
USP16	-0.05586	-0.58336	-0.38004	-0.04002	-0.13891	-0.09792	0.421787	0.433791
USP18	0.027491	0.51421	0.105637	-0.09736	-0.38895	-0.13729	NA	NA
USP19	0.169047	0.271329	0.226792	0.004311	0.4332	0.187086	0.475835	0.274961
USP2	-0.01953	0.163442	-0.05512	-0.45176	0.293242	-0.33049	0.238221	0.009979
USP20	-0.25803	-0.06275	0.215978	0.050561	0.376172	0.373739	-0.47911	-0.16101
USP21	0.06921	0.337278	-0.07402	0.323038	-0.02836	-0.10421	0.566087	0.585269
USP22	NA	NA	0.07109	0.276282	0.361043	0.111528	NA	NA
USP24	NA	NA	0.113828	0.048761	0.152059	0.173383	NA	NA
USP25	-0.06614	-0.49594	0.032253	-0.4598	-0.13039	0.012033	-0.53063	-0.1952
USP26	0.044812	0.38749	0.049005	-0.37273	0.15234	0.179823	-0.29764	-0.27642
USP27X	NA	NA	0.033924	0.076276	0.107831	-0.05469	NA	NA
USP28	0.037481	0.630804	-0.37857	0.069692	-0.10529	0.073955	-0.29643	-0.10922
USP29	0.103206	-0.01677	-0.21241	-0.14819	-0.28315	0.016663	-0.11837	-0.06817
USP3	0.100281	-0.07689	-0.24769	-0.19389	-0.33272	-0.12617	0.221412	0.296664
USP30	-0.0658	-0.10997	0.042524	-0.25048	0.062287	-0.44608	0.337408	0.154774
USP31	0.126237	-0.19542	-0.16374	-0.46757	0.260323	-0.16611	0.131844	0.272411
USP32	-0.51137	0.079053	0.16	-0.38722	-0.10446	0.341237	-0.31156	-0.02694
USP33	-0.06383	-0.34169	0.100137	-0.14163	0.187628	-0.12993	0.195039	0.201497
USP34	-0.2674	-0.72912	-0.11465	-0.13522	0.078459	0.215278	0.562615	0.468033

USP35	NA	NA	-0.13204	-0.14782	0.260973	0.192777	NA	NA
USP36	-0.10684	-0.03408	0.06157	-0.0611	-0.04126	0.182017	NA	NA
USP37	-0.12509	0.001462	0.014458	-0.30391	0.156029	0.106336	-0.10669	0.146989
USP38	-0.06767	-0.24704	-0.11055	-0.16202	-0.33789	-0.47549	-0.30374	-0.15003
USP39	0.045454	-0.45467	-0.06575	0.081252	0.290543	-0.07494	0.69941	0.447786
USP4	0.004709	0.068914	-0.05061	0.122161	0.223454	0.388201	-0.07686	0.507941
USP40	0.327976	0.774001	0.35123	-0.00901	0.253631	-0.04264	0.156946	-0.00759
USP42	NA	NA	0.068595	-0.15754	0.172919	0.296802	0.152946	0.186833
USP43	NA	NA	-0.28836	-0.09701	0.088782	-0.10795	-0.47557	-0.28227
USP44	0.041039	-0.09491	0.079839	0.151446	-0.21281	0.22119	0.403401	-0.01824
USP45	NA	NA	-0.17882	0.112204	0.036205	0.050302	0.121689	0.010289
USP46	-0.07548	0.195518	-0.13539	-0.32987	-0.36929	-0.41953	-0.36471	-0.23554
USP47	0.13489	-0.05265	-0.09111	-0.1504	0.110007	0.037667	-0.51488	-0.36487
USP48	-0.06842	-0.31453	-0.02175	0.217124	-0.11396	0.257708	-0.21176	-0.24737
USP49	-0.18528	0.158877	-0.32131	-0.0823	0.199111	0.278947	-0.5803	-0.58037
USP5	0.480554	0.069017	0.093271	0.353458	0.079393	-0.03354	0.295346	0.334674
USP50	0.123362	-0.03985	NA	NA	NA	NA	-0.33386	0.110858
USP51	0.139347	0.335878	-0.05304	-0.00943	-0.28891	0.153613	-0.06175	-0.0262
USP53	-0.22572	-0.02311	-0.07693	-0.30148	0.137497	0.169901	0.326955	0.231493
USP54	0.043152	-0.26403	-0.11921	-0.13959	0.29831	-0.24947	-0.16257	-0.02729
USP6	-0.20476	-0.62885	0.08578	-0.19189	-0.15474	-0.1273	-0.40034	-0.09352
USP6NL	NA	NA	-0.11665	0.19536	-0.14611	-0.41199	0.361679	0.035405
USP7	0.267853	0.650448	-0.14245	-0.00247	0.35955	0.07798	0.421389	-0.07622
USP8	0.014476	-0.68796	-0.0365	-0.14229	0.047941	0.011366	-0.57804	-0.31657
USP9X	0.188724	-0.37316	-0.12962	-0.19342	-0.0723	-0.02836	-0.3103	-0.37231
USP9Y	0.128019	0.009334	0.032864	-0.01586	-0.20992	-0.13734	0.345869	-0.21926
USPL1	-0.00964	-0.76194	0.04115	-0.28485	0.164254	-0.0121	0.083198	-0.09291
UST	-0.12187	-0.37911	0.030935	-0.02112	-0.37776	-0.03525	-0.14267	0.079757
UTF1	0.288954	0.167199	0.050915	-0.23362	-0.04414	-0.31207	-0.28089	-0.60694
UTP11L	-0.15062	-0.54063	-0.175	0.029754	0.012692	-0.10129	-0.28921	0.057728
UTP14A	0.39785	-0.32423	0.108281	-0.05503	0.201876	0.101672	0.471008	0.33392
UTP14C	0.383075	-0.10284	-0.01829	-0.41762	0.180196	-0.26847	0.376996	0.021646
UTP15	0.440922	-0.21888	-0.22719	-0.06389	-0.10387	-0.07169	0.001245	-0.09438
UTP18	0.022412	-0.4973	-0.05347	0.315922	-0.10958	0.215044	NA	NA
UTP20	0.308133	0.354359	-0.21852	0.199934	0.117937	0.102092	-0.11067	0.014969
UTP23	NA	NA	-0.07443	-0.18009	-0.04583	0.029199	NA	NA
UTP3	NA	NA	-0.38702	0.238089	-0.09793	0.162911	NA	NA
UTP6	0.287911	-0.64757	0.061268	0.240925	0.070613	0.317778	NA	NA
UTRN	0.160142	-0.00963	-0.2629	0.07774	-0.12986	0.313847	-0.06701	0.115478
UTS2	-0.0675	0.410402	-0.1305	-0.12491	-0.34739	-0.3672	-0.08133	0.009198
UTS2D	0.232947	0.039191	-0.08731	0.509768	-0.02875	0.362845	0.420592	-0.03549
UTS2R	-0.29408	0.68334	-0.01149	0.104829	-0.11511	0.17132	-0.26833	-0.59655
UTX	-0.21923	-0.58394	-0.36713	-0.11622	0.0427	0.045439	0.153105	0.493756
UTY	0.239746	0.470586	0.117233	-0.05656	-0.2541	-0.13054	0.140397	-0.01431
UVRAG	-0.21563	-0.68645	-0.1016	0.41655	0.088057	0.184191	-0.30219	-0.2442
UXS1	0.208259	0.741219	0.037018	0.240691	0.09869	0.024262	-0.04132	-0.14096
UXT	0.56734	0.632755	-0.26046	0.344093	0.043926	-0.31104	0.309289	0.122673
VAC14	0.255678	0.723337	0.028701	0.301703	0.361467	0.204775	0.667583	0.420659
VAMP1	-0.34934	0.019699	-0.32104	-0.14516	0.029195	-0.19383	-0.44747	0.035756
VAMP2	-0.16229	0.446454	-0.04584	0.025294	-0.26728	-0.19679	-0.14921	-0.24589
VAMP3	0.13247	-0.12868	0.116572	-0.53729	-0.04836	-0.3329	0.111129	0.162953
VAMP4	-0.52136	-0.578	-0.14015	-0.08564	-0.20846	-0.42849	-0.58682	-0.21792

VAMP5	-0.33519	-0.27919	0.204899	-0.04468	-0.15485	0.12976	-0.24431	0.18959
VAMP7	NA	NA	-0.28692	-0.28562	-0.00733	-0.49408	NA	NA
VAMP8	-0.02547	0.165818	0.074732	-0.11502	-0.30658	-0.46986	-0.04508	0.093705
VANGL1	0.203546	0.422022	0.27329	-0.08325	0.186393	-0.15194	0.210356	0.197724
VANGL2	0.305731	-0.64587	NA	NA	NA	NA	0.539472	0.347231
VAPA	-0.27799	-0.49254	-0.2696	-0.40664	-0.24164	-0.34565	0.046622	0.063446
VAPB	0.20221	0.070889	-0.04537	-0.13455	0.229414	-0.21098	-0.41727	-0.28935
VARS	0.557889	0.557013	0.140452	0.293987	0.395862	-0.00262	0.407098	0.098855
VARS2	0.367378	0.725248	0.076136	0.041112	0.211632	-0.04787	NA	NA
VASH1	-0.21749	0.004283	0.02758	0.088838	-0.06268	0.204933	-0.37139	0.281586
VASH2	0.10726	-0.39714	-0.01153	0.223255	-0.0155	0.266586	NA	NA
VASN	-0.32185	-0.21547	-0.03831	0.331134	0.035968	0.239554	NA	NA
VASP	-0.0265	0.049787	0.010607	-0.09048	-0.06257	0.086854	-0.434	-0.60775
VAT1	-0.24795	0.07259	0.459977	-0.13895	0.073383	0.057681	-0.30627	-0.47764
VAT1L	NA	NA	-0.03134	0.631879	0.23231	0.284838	NA	NA
VAV1	-0.25314	-0.09234	0.277972	0.66092	0.10921	0.640191	-0.60796	0.086069
VAV2	0.393234	0.218937	-0.20017	0.187059	0.423393	-0.01635	0.056606	0.17324
VAV3	0.261783	-0.02814	0.405547	-0.07879	0.279127	-0.24658	0.173355	-0.52524
VAX1	0.347588	0.602245	NA	NA	NA	NA	-0.10452	0.114452
VAX2	-0.07362	0.026198	-0.06324	-0.02482	-0.05119	-0.21623	-0.0989	-0.41499
VBP1	0.070509	-0.6879	-0.34692	-0.06996	-0.0717	-0.37145	0.104828	-0.31778
VCAM1	-0.26771	-0.29069	-0.1346	0.450494	-0.09443	0.549818	-0.486	-0.03051
VCAN	-0.28216	-0.45578	0.038437	-0.17047	-0.22785	0.09771	NA	NA
VCL	-0.11043	-0.18119	-0.32109	-0.09828	0.099162	0.230345	-0.29636	-0.30879
VCP	-0.17852	0.007851	0.159633	0.228496	0.217915	0.192517	-0.37163	-0.07691
VCPIP1	0.313369	0.270939	0.113117	-0.3774	-0.26397	-0.31911	-0.03435	-0.18448
VCX	0.066481	0.21879	-0.26193	-0.04776	0.219289	-0.08595	-0.14867	0.068635
VCX2	NA	NA	-0.27206	0.101153	0.174573	-0.05653	0.04177	-0.02627
VCX3A	NA	NA	-0.26771	-0.1305	0.223867	-0.08063	-0.14985	0.02023
VCY	NA	NA	-0.07075	-0.15962	0.133714	-0.25626	-0.01765	0.277864
VCY1B	0.0343	0.503286	NA	NA	NA	NA	0.005648	0.271251
VDAC1	0.134006	-0.3476	0.294976	0.179355	0.204134	-0.32111	0.373591	0.320896
VDAC2	0.006267	0.408666	-0.08795	-0.01049	0.217806	-0.03219	-0.64207	-0.64675
VDAC3	-0.18226	-0.1714	0.241503	-0.13942	0.036973	-0.25602	0.328626	0.012073
VDR	-0.09602	-0.15415	0.020192	0.031227	0.058727	-0.13085	0.125734	0.054937
VEGFA	-0.13454	0.096103	-0.26403	0.338502	0.156115	0.262611	0.388192	0.43057
VEGFB	-0.07238	0.078041	0.312521	0.003291	0.219937	0.332636	-0.33597	-0.33252
VEGFC	-0.4426	-0.40528	0.045584	0.224122	-0.2424	0.268314	-0.03843	0.389605
VENTX	-0.17184	0.280373	-0.09515	-0.01334	-0.14919	0.026902	-0.06491	-0.02723
VEPH1	-0.20116	0.235909	-0.03853	-0.23463	-0.06522	-0.44541	-0.02342	0.327487
VEZF1	0.012528	-0.16618	0.047365	-0.25253	0.152743	0.001278	NA	NA
VEZT	-0.36433	-0.02013	-0.20342	-0.3276	0.049806	-0.08252	-0.31978	-0.03212
VGF	0.29122	0.749136	-0.1211	-0.12035	0.255972	-0.19452	0.302276	-0.06464
VGLL1	0.279485	-0.01133	-0.29269	-0.29299	-0.18615	0.089468	0.420383	0.402579
VGLL2	-0.04644	0.354621	0.030266	-0.27643	0.456881	-0.05781	-0.17954	0.054845
VGLL3	-0.41587	-0.47057	-0.13166	-0.0198	-0.31071	-0.05535	-0.25233	-0.1069
VGLL4	-0.06356	0.111156	0.182609	0.027247	0.236669	0.14485	0.019803	0.170205
VHL	-0.02408	-0.18025	-0.28595	-0.04413	-0.02718	0.077903	0.242478	0.321484
VHLL	0.458067	0.310884	-0.26229	0.195045	0.129506	-0.06216	0.055934	0.143938
VIL1	0.557394	0.065507	0.214205	0.025136	0.408347	-0.0326	0.494721	0.199305
VILL	0.072844	0.350078	-0.33816	-0.06632	-0.15384	-0.20999	0.292887	0.218086
VIM	-0.49934	-0.66617	0.171497	0.215518	-0.1481	0.387418	-0.38678	-0.26583

VIP	0.006881	-0.07515	-0.1001	0.278503	-0.08352	-0.02462	-0.13181	-0.44512
VIPR1	0.085656	0.120419	0.14532	0.021283	0.148218	-0.1242	0.320065	0.094258
VIPR2	0.194845	-0.05377	0.060313	-0.09849	0.018127	0.181987	-0.2768	-0.08269
VIT	0.157402	-0.26146	-0.13832	0.163231	-0.17627	-0.14841	0.151305	-0.29607
VKORC1	-0.259	-0.06613	0.409628	0.142054	0.098103	-0.00678	0.117142	0.17857
VKORC1L1	0.238608	0.606387	-0.18854	0.060414	-0.17159	-0.24445	0.152666	-0.24156
VLDLR	0.202242	0.650664	-0.49322	-0.57133	-0.00809	-0.07533	-0.23306	-0.04997
VMA21	NA	NA	-0.21652	-0.15901	-0.01532	-0.36939	NA	NA
VMAC	0.338221	0.741026	0.369358	0.104169	0.331524	0.117871	-0.10865	0.083116
VMO1	-0.2148	0.489056	0.244568	-0.26731	-0.20254	-0.15626	-0.27027	0.247539
VN1R1	0.250533	-0.34437	0.143518	-0.09376	-0.24623	0.512505	0.063711	-0.0607
VN1R2	-0.31842	0.352255	0.005656	0.124588	0.223741	0.064821	-0.07538	-0.10619
VN1R4	0.171968	0.76035	-0.17848	-0.09607	-0.11365	0.177059	0.481228	0.518376
VN1R5	0.317264	0.787432	0.071422	-0.14771	0.032534	0.237791	-0.33255	-0.2695
VNN1	-0.3331	-0.27853	-0.10626	-0.03171	-0.0796	-0.31745	0.116002	0.569216
VNN2	-0.29847	0.155998	0.048854	0.381332	0.073898	0.313385	-0.6028	0.16489
VNN3	-0.40745	0.417901	-0.1471	0.291317	0.084186	-0.3023	-0.3365	0.120424
VPRBP	-0.24619	0.217738	-0.12859	0.289089	0.205427	0.159865	0.370233	0.101569
VPREB1	0.154426	0.172396	0.170883	-0.02715	0.318708	0.089889	0.546714	0.296954
VPREB3	-0.34517	0.483312	0.13441	0.73071	-0.16188	0.558316	-0.03183	0.219699
VPS11	0.319314	0.745976	0.381748	0.081623	0.215545	0.282986	-0.49813	-0.34604
VPS13A	-0.27946	-0.30746	-0.04887	-0.14962	-0.17037	0.002695	-0.15305	0.199802
VPS13B	-0.2437	0.701347	0.148862	-0.17591	-0.11483	0.293614	-0.38741	-0.07273
VPS13C	0.207484	-0.07246	0.091608	-0.09521	0.050816	0.2412	-0.32541	-0.0985
VPS13D	0.09921	0.105272	-0.20762	-0.18687	0.094662	-0.14865	-0.0431	0.09611
VPS16	0.039376	0.270842	-0.12881	0.246277	0.272619	0.448247	0.018429	0.149197
VPS18	-0.21801	0.107137	-0.00493	0.292882	-0.25381	0.083731	-0.2107	0.119183
VPS24	0.02592	-0.27638	-0.14681	-0.33568	0.035853	-0.48634	0.290545	-0.16052
VPS25	0.322096	-0.16755	0.302262	-0.05517	-0.04862	-0.289	0.478164	0.12689
VPS26A	-0.10483	-0.59367	-0.12635	-0.14203	0.096063	-0.16396	0.179267	0.070234
VPS26B	0.021029	0.194632	0.380766	-0.0668	0.327393	0.071233	0.007508	0.086691
VPS28	0.017911	0.634204	0.26536	0.222008	-0.04813	-0.08998	0.531201	0.146385
VPS29	-0.21935	-0.70688	0.015041	-0.21018	0.151382	-0.03142	-0.44268	-0.04957
VPS33A	0.123269	0.765305	-0.22257	0.081308	-0.10449	-0.28961	0.123345	0.427691
VPS33B	0.198459	-0.49416	0.078428	-0.03828	0.348674	-0.08665	0.319126	0.392182
VPS35	0.40231	0.045805	-0.07271	-0.44545	0.388015	-0.48574	0.22592	-0.22848
VPS36	0.451947	0.039477	-0.03923	-0.03507	0.069591	-0.34593	-0.03222	-0.23138
VPS37A	0.007191	-0.55609	-0.00672	-0.4451	-0.14774	-0.48659	-0.49139	-0.24674
VPS37B	-0.50133	0.268073	0.029146	-0.32807	-0.02747	-0.34171	0.154831	0.014508
VPS37C	-0.36921	0.214672	-0.10007	-0.1014	0.250869	-0.17667	-0.43441	-0.08617
VPS37D	NA	NA	-0.01087	-0.2058	-0.04177	-0.16883	-0.30749	-0.43983
VPS39	0.139454	0.287981	0.273199	-0.03662	-0.01555	0.180448	0.209854	0.305822
VPS41	0.345392	-0.17345	-0.12702	-0.52892	-0.10406	-0.23521	-0.36386	-0.29296
VPS45	-0.36693	-0.48892	-0.12175	0.062735	-0.26088	-0.15435	NA	NA
VPS4A	-0.01603	0.421516	0.031955	0.088484	0.212052	-0.15815	0.329272	0.226648
VPS4B	-0.1733	-0.37301	-0.10598	-0.1587	-0.10326	-0.13713	0.437622	0.231317
VPS52	0.502675	0.491832	0.05112	0.165202	0.032185	-0.09874	0.612815	0.50945
VPS53	-0.31439	-0.51607	0.115228	0.049831	0.031629	0.291978	0.051719	0.420156
VPS54	-0.15099	-0.66341	-0.13134	-0.14374	0.077514	-0.44054	0.380766	0.124481
VPS72	0.487029	0.480249	-0.13689	0.213809	-0.09542	-0.01023	0.683451	0.28675
VPS8	-0.06657	-0.59196	-0.00512	-0.21525	-0.07663	-0.27767	-0.0712	0.023982
VRK1	0.058675	-0.02687	-0.54294	0.006704	0.130174	-0.0106	-0.13509	-0.35587

VRK2	0.318889	0.391305	-0.09292	-0.17944	-0.13964	0.062126	-0.27778	-0.05619
VRK3	0.113931	0.365357	0.084041	0.095896	0.22758	-0.18404	0.338682	0.190284
VSIG1	-0.15101	0.530582	-0.11968	0.179808	-0.0817	0.15324	-0.35128	-0.43093
VSIG2	-0.20825	-0.03152	-0.14736	0.149989	-0.13351	-0.02254	0.075857	-0.28294
VSIG4	-0.38261	-0.1002	0.370604	-0.66754	-0.30704	-0.18208	-0.18603	0.157823
VSIG8	-0.3762	0.119433	NA	NA	NA	NA	0.092324	0.392074
VSNL1	0.038172	0.19096	-0.1009	-0.189	-0.31267	-0.14866	0.154076	-0.35893
VSTM1	0.546645	0.639095	NA	NA	NA	NA	NA	NA
VSTM2A	NA	NA	-0.28761	-0.4326	-0.29196	-0.26389	NA	NA
VSTM2B	NA	NA	-0.11435	-0.22007	-0.16016	0.405942	NA	NA
VSTM2L	NA	NA	-0.13805	-0.04154	-0.16375	-0.18757	NA	NA
VSX1	-0.34697	0.576796	-0.0307	-0.24282	0.001908	0.007311	-0.01896	0.1483
VTA1	NA	NA	-0.44204	-0.21911	-0.09048	-0.33623	NA	NA
VTCN1	-0.00378	0.068432	-0.15356	-0.11081	-0.24834	-0.09561	0.295032	-0.06352
VTI1A	0.275654	0.599513	-0.17749	-0.17531	0.24851	-0.43976	NA	NA
VTI1B	0.007664	-0.56302	-0.27637	0.120459	-0.31807	-0.42953	-0.63022	-0.57224
VTN	0.111717	0.075903	-0.01633	-0.25741	0.015988	-0.15033	0.021726	-0.13349
VWA1	NA	NA	0.369928	-0.01555	0.222503	0.250708	0.109289	-0.20741
VWA2	0.355201	-0.03124	NA	NA	NA	NA	0.277136	0.366858
VWA3A	NA	NA	-0.14835	0.052772	-0.00911	0.233493	NA	NA
VWA3B	NA	NA	-0.22177	-0.05495	0.229682	-0.16305	NA	NA
VWA5A	NA	NA	0.187853	0.074217	0.208261	-0.24552	NA	NA
VWA5B1	NA	NA	-0.12166	-0.11228	0.277567	0.100209	NA	NA
VWA5B2	NA	NA	0.165405	-0.17755	0.15975	0.028444	NA	NA
VWC2	-0.17567	0.403003	-0.16245	-0.23753	-0.01351	-0.21646	NA	NA
VWC2L	NA	NA	-0.16305	-0.11767	-0.34291	-0.11429	NA	NA
VWCE	0.225346	-0.11077	-0.26251	-0.02528	-0.12242	-0.05909	-0.1292	-0.07719
VWDE	NA	NA	0.170143	0.084174	-0.13994	-0.10348	NA	NA
VWF	0.29886	-0.32605	0.022903	0.178941	0.097079	0.142259	-0.33985	-0.06052
WAC	-0.26882	-0.42619	-0.08349	-0.26489	0.064635	-0.09815	-0.26767	-0.26583
WAPAL	0.068973	0.76572	-0.31989	-0.15104	0.08227	-0.10099	NA	NA
WARS	0.066295	0.015133	-0.02972	0.227751	-0.22917	0.301395	-0.25615	0.226303
WARS2	NA	NA	-0.0655	0.196369	-0.4084	-0.21989	0.338302	0.247815
WAS	-0.50406	0.25025	0.281292	0.612109	0.053299	0.298661	-0.66785	-0.02962
WASF1	0.174667	-0.31306	-0.33054	-0.00791	-0.26842	0.188208	0.299656	0.082961
WASF2	-0.44978	-0.54419	0.19957	-0.00963	0.190053	0.527429	-0.18849	-0.14591
WASF3	-0.02602	-0.39136	-0.16002	-0.22854	-0.14845	0.183691	-0.12275	-0.10353
WASH2P	NA	NA	-0.05767	0.144158	0.149919	0.2534	NA	NA
WASL	0.281198	-0.15961	-0.08992	-0.38299	-0.15704	-0.154	-0.10039	0.026408
WBP1	NA	NA	0.322712	-0.04874	-0.15297	-0.19354	-0.53048	-0.47687
WBP11	0.118255	-0.19581	-0.30646	0.054598	-0.1486	0.020774	0.479707	0.519213
WBP2	-0.19576	0.044574	0.501522	0.138503	-0.09606	-0.0193	0.135494	0.192576
WBP2NL	-0.1861	0.161015	-0.22011	0.020576	-0.1457	-0.18574	NA	NA
WBP4	0.470591	-0.29153	-0.14633	-0.24109	0.20494	-0.1508	0.463213	0.183565
WBP5	-0.06834	0.015079	-0.4162	-0.19893	-0.16643	-0.29096	-0.54028	-0.61606
WBSCR16	-0.06814	0.802443	0.181096	0.088173	0.246386	0.227392	0.545527	0.457942
WBSCR17	-0.47067	0.547221	-0.1346	-0.278	-0.0819	0.057813	0.370268	0.152811
WBSCR22	0.179568	0.146481	0.08897	0.405847	0.120393	0.178147	0.506749	0.244799
WBSCR23	0.350226	0.820266	0.151546	-0.25559	0.175982	0.24644	0.005018	0.02023
WBSCR27	0.128199	0.272486	0.250968	0.004927	-0.10856	-0.02259	0.165346	-0.20305
WBSCR28	0.473581	0.146511	0.082232	-0.1253	0.07892	0.11855	0.360845	0.383465
WDFY1	0.298954	0.172306	-0.01675	-0.13114	-0.2255	0.008958	0.047066	0.069587

WDFY2	-0.17411	-0.18936	0.047933	0.104026	0.037675	0.523609	0.473715	0.536238
WDFY3	-0.18197	0.268575	0.017268	-0.40957	-0.05559	0.104255	-0.03512	0.160055
WDFY4	NA	NA	0.037242	0.784714	-0.34832	0.689088	NA	NA
WDHD1	-0.33585	-0.15687	-0.59454	0.138128	-0.11752	-0.03692	0.173145	0.341703
WDR1	-0.15053	-0.41155	-0.02335	0.109964	0.272476	0.09226	0.310477	0.253058
WDR12	0.190032	-0.5483	0.2731	-0.22912	0.027805	-0.08273	0.268947	-0.06187
WDR13	0.311692	0.234671	0.386116	0.066575	0.295308	0.141735	-0.13232	-0.21243
WDR16	0.086792	0.256839	-0.04338	-0.14959	-0.02169	0.274984	-0.18643	0.03262
WDR17	0.4091	-0.19073	0.236833	0.055283	-0.1399	0.096073	-0.67788	-0.62573
WDR18	0.130186	0.44943	0.217196	0.212068	0.218483	-0.34477	0.039144	-0.13088
WDR19	0.188983	-0.06273	0.301396	0.00888	-0.1276	0.489232	0.05466	0.110813
WDR20	-0.19371	0.131435	-0.52237	-0.09125	-0.11476	-0.1441	0.303472	0.433868
WDR21A	0.160068	-0.49349	-0.26864	0.108958	0.124595	0.279118	0.351669	0.32802
WDR21C	0.203293	0.23748	-0.27256	-0.42534	0.220098	-0.15173	-0.61372	-0.48255
WDR22	0.032731	0.175097	-3.4E-05	-0.26913	-0.14728	0.464973	-0.3261	-0.31866
WDR23	-0.54706	-0.3778	0.058119	0.189625	0.121683	-0.28411	-0.6316	-0.0023
WDR24	-0.23919	-0.24559	0.037744	0.167097	0.241936	-0.07622	0.490303	0.461048
WDR25	0.015373	0.569994	0.08982	0.312893	-0.0036	-0.22783	-0.39846	-0.24486
WDR26	-0.33557	-0.77062	-0.34543	-0.33113	-0.33663	-0.17419	0.077849	0.032523
WDR27	0.2177	0.010639	0.152293	-0.17495	0.231964	0.434745	0.322309	0.405352
WDR3	0.238213	-0.27091	-0.03793	-0.12275	0.161441	0.205762	0.373687	0.202913
WDR31	-0.07416	-0.28271	0.085543	0.177548	0.076172	-0.03167	0.186455	0.068531
WDR33	0.086679	-0.27509	-0.09945	0.171425	0.255337	0.017847	0.066869	0.136498
WDR34	-0.40072	-0.04447	0.320545	0.251472	0.266915	0.002158	0.310768	0.200874
WDR35	0.367896	-0.43799	-0.05013	-0.07052	0.263941	0.399606	0.334379	0.309279
WDR36	0.574887	-0.23115	-0.0051	-0.22016	0.041376	0.336253	0.338427	0.115446
WDR37	-0.49162	-0.68028	-0.07562	-0.13532	-0.08529	0.256341	0.15963	0.163787
WDR38	NA	NA	0.393559	-0.403	-0.3877	0.054446	NA	NA
WDR4	0.395914	0.191637	0.209787	0.214208	0.305593	-0.06882	0.226051	-0.03368
WDR40A	0.11471	-0.37041	0.000362	-0.10237	0.058492	-0.03666	0.046762	-0.0374
WDR40B	0.426257	0.402826	0.002441	-0.0773	-0.14509	-0.05262	0.395095	-0.14747
WDR41	0.069241	0.166337	-0.04968	-0.04676	-0.37762	-0.53299	-0.15193	-0.48532
WDR43	NA	NA	-0.05527	-0.2305	0.211961	0.092738	NA	NA
WDR44	-0.13399	-0.16629	-0.11245	-0.22559	-0.16839	0.252989	-0.52958	-0.31558
WDR45	-0.25114	0.152616	0.14874	-0.0532	-0.00832	0.178803	0.118859	0.164384
WDR45L	-0.58326	-0.61546	-0.06214	-0.06049	0.217626	-0.21041	0.412728	0.408839
WDR46	0.432147	-0.22142	0.139496	0.390521	0.377899	0.256462	0.519338	0.392919
WDR47	-0.50934	-0.60118	0.430297	-0.13778	-0.01291	-0.2492	0.19437	0.191325
WDR48	0.336221	0.328947	-0.25875	-0.42633	-0.078	-0.06015	0.651294	-0.02146
WDR49	0.212729	0.460288	-0.10485	-0.15565	-0.07756	-0.10224	-0.14318	0.172263
WDR5	-0.08501	0.417973	-0.19467	0.178542	0.205449	-0.10894	0.482478	0.441573
WDR51A	-0.06987	-0.58358	-0.21699	0.288252	0.229296	-0.18559	0.267234	0.090441
WDR51B	0.116557	-0.5313	-0.33807	0.057702	0.199424	-0.10005	-0.20335	-0.39789
WDR52	0.093827	0.099172	-0.18806	0.049861	0.032543	-0.13778	-0.57697	-0.53019
WDR53	-0.07132	0.006408	-0.11996	0.099994	-0.02972	-0.09193	0.191226	0.384702
WDR54	0.016432	0.326593	-0.1056	0.43026	-0.00786	0.28967	0.267068	-0.09529
WDR55	0.118944	-0.17817	0.424049	-0.21774	0.233186	-0.17051	0.093228	0.263534
WDR59	0.383389	-0.43312	0.090721	0.148912	0.268624	0.599813	0.444521	0.491238
WDR5B	0.196571	0.491982	-0.00052	0.143334	-0.10513	-0.25225	0.403624	0.58525
WDR6	-0.02418	-0.01098	-0.14071	0.267423	0.197346	0.434846	0.518045	0.606103
WDR60	0.145829	-0.07517	-0.14091	0.101577	-0.03412	-0.19586	-0.41408	0.219737
WDR61	0.300556	-0.59136	0.205564	-0.0865	-0.08486	0.288941	0.611327	0.339589

WDR62	0.018482	0.172812	-0.14911	-0.01611	0.196924	0.188394	0.439281	0.325684
WDR63	0.201557	0.774473	0.127064	-0.15773	-0.20332	-0.54195	-0.35705	-0.15853
WDR64	0.409691	0.255234	-0.05956	-0.24305	-0.14378	-0.10288	-0.28795	-0.0247
WDR65	0.247226	0.552727	-0.19729	-0.15952	-0.15702	0.262847	-0.20883	-0.1561
WDR66	-0.0238	-0.34084	-0.01961	0.53329	0.078014	-0.04546	0.264556	-0.17332
WDR67	-0.02385	0.016447	0.077906	-0.02654	0.00683	0.145266	-0.08744	-0.03117
WDR68	0.359938	0.546442	0.377572	0.121651	0.140298	0.356221	0.637313	0.356451
WDR69	0.038988	0.180674	-0.09342	0.165858	-0.16604	0.121298	0.047522	0.430308
WDR7	-0.32511	-0.62784	-0.00051	-0.01475	-0.3663	0.006755	-0.30537	0.061075
WDR70	0.038515	0.297157	-0.28023	0.005071	-0.05666	0.038578	0.59274	0.072577
WDR72	-0.12681	0.064637	-0.36955	-0.18497	0.05268	0.011363	-0.12396	-0.14586
WDR73	0.388838	0.494107	-0.19017	-0.00956	0.164498	0.34412	0.260375	0.433095
WDR74	0.131199	-0.66196	0.150833	0.21184	0.229947	0.359624	-0.37111	-0.48935
WDR75	0.308453	0.420092	-0.40367	-0.04631	-0.04612	-0.03507	-0.12457	-0.14116
WDR76	-0.00905	0.123635	-0.16814	0.416775	-0.00169	-0.02782	NA	NA
WDR77	0.227667	-0.53252	0.178943	0.28107	0.362623	0.222168	0.601217	0.397005
WDR78	-0.04228	-0.05236	-0.11792	-0.47586	-0.02674	-0.4397	-0.33839	-0.38388
WDR79	-0.00366	0.663868	-0.05928	0.175594	0.120979	-0.17305	-0.095	-0.50365
WDR8	0.020407	-0.39807	0.209386	0.224561	0.27473	0.093689	0.47947	0.602918
WDR81	0.139211	0.279485	NA	NA	NA	NA	-0.28171	-0.00261
WDR82	NA	NA	0.100902	0.254014	0.252905	0.431911	NA	NA
WDR85	0.10647	-0.38949	-0.0172	0.155815	0.280882	0.150613	NA	NA
WDR86	-0.34188	0.124389	NA	NA	NA	NA	NA	NA
WDR87	NA	NA	-0.14661	-0.06466	0.198234	0.181594	NA	NA
WDR88	NA	NA	-0.07206	-0.05168	0.010208	-0.25211	NA	NA
WDR89	0.181485	0.748131	-0.20518	0.1439	-0.26789	-0.29692	NA	NA
WDR90	0.048539	0.339968	-0.09225	-0.02176	0.37258	0.174418	NA	NA
WDR91	NA	NA	-0.29974	0.300328	-0.22347	0.279291	NA	NA
WDR92	NA	NA	0.133164	0.01686	0.003592	0.204136	NA	NA
WDR93	NA	NA	0.055328	-0.0757	-0.02627	-0.11323	NA	NA
WDSOF1	0.158807	-0.60217	0.031038	-0.04348	-0.01934	0.332454	-0.19448	-0.37572
WDSUB1	0.288169	-0.59265	0.049803	-0.077	-0.08659	-0.38543	-0.09489	-0.19821
WDTC1	0.252326	0.699851	0.189225	0.042251	-0.00439	-0.0753	-0.33852	-0.10299
WDYHV1	NA	NA	0.021496	-0.14963	-0.20882	0.010641	NA	NA
WEE1	-0.04953	-0.51287	-0.388	-0.08354	0.050704	0.13401	0.454914	-0.03057
WFDC1	-0.02876	-0.05536	0.021825	-0.19319	-0.04584	-0.11626	-0.14508	-0.21406
WFDC10A	0.156364	-0.07038	-0.10633	-0.0587	-0.14254	-0.3015	0.476588	-0.10369
WFDC10B	0.166521	0.589655	-0.19658	-0.18064	-0.0021	-0.14607	0.046365	-0.06644
WFDC11	0.185795	0.367864	-0.50055	-0.25439	-0.14551	-0.0718	-0.0243	-0.00704
WFDC12	0.21527	0.593034	-0.21082	-0.09759	-0.31468	-0.365	0.108611	0.456485
WFDC13	0.179997	0.454704	-0.06743	-0.236	0.331903	-0.00209	-0.69302	-0.67204
WFDC2	0.225304	0.063716	-0.30201	-0.17523	-0.28268	-0.23467	0.086757	-0.22833
WFDC3	0.100125	0.244005	0.050847	-0.03906	0.193404	-0.12692	0.118133	0.002676
WFDC5	0.013345	-0.02185	-0.14894	0.054395	-0.19566	-0.15734	0.386436	-0.01449
WFDC6	0.191819	0.561718	-0.15114	-0.2947	0.087966	-0.25262	-0.33112	-0.15441
WFDC8	0.032033	0.687035	0.013042	0.059891	0.186359	-0.03309	0.091741	0.176371
WFDC9	0.191546	0.752013	0.403019	-0.27413	0.040178	0.354633	0.425181	0.474214
WFIKKN1	0.009568	-0.12954	-0.09694	0.080945	0.154884	-0.0993	-0.12942	-0.09761
WFIKKN2	0.262272	-0.00686	0.151159	-0.13933	-0.09511	-0.44008	0.347844	-0.22945
WFS1	0.163114	0.270351	0.629587	-0.11994	0.250576	0.275999	0.205678	-0.3072
WHSC1	0.04677	0.65143	-0.21752	-0.06496	0.23035	-0.12511	0.023887	-0.10315
WHSC1L1	-0.00866	-0.46658	0.091489	-0.03837	0.10486	-0.20091	0.159353	0.241588

WHSC2	0.30379	0.51168	-0.14628	0.229302	0.336949	0.12176	0.199901	0.06313
WIBG	-0.02507	0.601254	-0.12412	-0.11222	-0.0944	-0.29815	0.105747	0.10529
WIF1	0.546025	-0.01251	0.241384	-0.38811	0.096355	-0.03951	0.170391	-0.33245
WIPF1	-0.29582	-0.00216	0.346519	0.688482	-0.04849	0.682413	-0.34504	-0.01507
WIPF2	0.044766	0.189065	0.145045	-0.22925	0.10495	0.062632	NA	NA
WIPF3	NA	NA	NA	NA	NA	NA	0.259014	0.344824
WIPI1	0.032214	-0.05499	0.021376	0.385251	-0.23157	-0.22111	0.020288	0.059848
WIPI2	0.551062	0.481725	0.110354	0.113687	0.085894	0.090013	-0.1856	0.080734
WISP1	-0.25455	0.199121	-0.0361	-0.02212	-0.04933	0.132715	-0.02253	-0.06117
WISP2	-0.31743	0.063719	0.20315	-0.03452	-0.21809	-0.26375	-0.10324	0.026269
WISP3	0.320985	0.544616	-0.25257	-0.26633	-0.03225	0.050587	-0.23844	-0.33093
WIT1	0.564861	0.063026	-0.05873	-0.19779	0.185645	-0.06936	NA	NA
WIZ	NA	NA	-0.25472	0.196931	0.355269	0.342348	NA	NA
WNK1	0.130311	0.496437	-0.02365	0.274548	0.157467	0.10198	0.265795	0.1117
WNK2	0.086634	0.005192	-0.0863	0.015148	0.170423	0.080148	0.172996	-0.27694
WNK3	-0.4114	0.239008	0.070817	-0.0338	-0.00799	0.087199	-0.32444	-0.05421
WNK4	0.190035	0.204695	0.016766	0.221162	0.099924	0.273698	0.424608	-0.06707
WNT1	-0.08534	0.250225	-0.14447	0.456098	-0.15977	0.471451	0.740969	0.642006
WNT10A	0.301106	0.512615	0.351526	0.168159	-0.19736	-0.1858	0.098885	-0.27902
WNT10B	0.227198	-0.28327	0.041187	0.580659	-0.33132	0.170538	-0.51657	-0.12853
WNT11	0.131482	0.128602	0.157702	-0.123	0.280421	-0.17483	0.534733	0.355035
WNT16	-0.15973	0.14829	0.309585	0.295383	-0.41496	0.238839	-0.30143	-0.20648
WNT2	-0.13769	0.036098	-0.14109	0.438133	-0.03494	-0.17214	-0.14686	0.067982
WNT2B	-0.22231	0.250527	-0.27738	0.314425	-0.13733	-0.15197	-0.29306	-0.11663
WNT3	-0.04976	-0.22471	0.050491	0.185195	-0.02531	0.104374	0.15813	0.491623
WNT3A	-0.19645	0.203594	-0.37291	-0.03184	-0.02054	-0.19645	-0.0472	-0.27601
WNT4	-0.26825	0.573189	-0.11043	0.185837	0.094042	-0.26125	0.097894	0.076387
WNT5A	-0.28985	-0.41087	-0.1735	-0.02319	-0.01883	-0.14072	0.081203	0.20929
WNT5B	0.393003	0.306932	0.158106	0.138766	0.171581	-0.02351	0.090599	-0.31392
WNT6	0.182755	0.564187	0.140384	-0.01673	-0.15296	0.265422	0.069016	0.007712
WNT7A	0.032153	0.773702	0.145004	0.230029	-0.26625	0.193385	-0.1088	-0.18316
WNT7B	0.045055	0.460914	0.112164	-0.27858	-0.04315	0.173301	0.353702	0.50967
WNT8A	0.369651	0.3996	0.325888	-0.10057	0.032873	-0.01189	-0.44229	0.096382
WNT8B	0.32246	0.635125	-0.38469	0.006486	0.154344	-0.01477	0.084022	0.09361
WNT9A	-0.0289	0.339491	0.038017	-0.06566	0.142713	0.338577	-0.01489	-0.03901
WNT9B	0.22191	0.341175	0.14266	0.223556	-0.10469	-0.02317	-0.19961	-0.3342
WRB	-0.12897	-0.56416	-0.07787	-0.18942	-0.31107	-0.57442	-0.56073	-0.23726
WRN	-0.1805	-0.62179	0.022009	-0.08874	-0.11988	0.191911	0.275226	0.161009
WRNIP1	-0.25694	-0.6454	-0.07279	0.295102	0.238167	0.172245	0.433951	-0.09199
WSB1	-0.4805	-0.31047	-0.19973	-0.06394	-0.09211	0.361568	-0.64551	-0.33378
WSB2	-0.31429	-0.03885	-0.06773	0.046078	0.170147	-0.36872	-0.57043	-0.41545
WSCD1	NA	NA	-0.11117	-0.1298	-0.12136	-0.11127	NA	NA
WSCD2	NA	NA	0.03471	-0.18561	-0.1142	0.266686	NA	NA
WT1	0.432041	0.127615	-0.33686	-0.19746	0.105219	-0.16897	0.114064	0.432858
WTAP	-0.04435	-0.2792	-0.47487	0.062149	0.153679	-0.04724	0.197978	0.281066
WTIP	NA	NA	NA	NA	NA	NA	-0.2126	-0.20119
WWC1	0.003834	-0.01285	0.113456	0.052404	-0.01533	0.041274	0.135393	0.206157
WWC2	-0.12295	-0.3593	0.092361	0.011165	0.018151	0.149442	-0.02642	-0.2541
WWC3	-0.21604	-0.23621	0.192501	0.079951	0.236116	0.600759	0.091403	0.372359
WWOX	0.242525	0.534649	0.040254	0.027508	0.04103	-0.16954	0.382804	0.15458
WWP1	0.296383	0.169959	0.522609	-0.28468	-0.15455	0.229369	-0.46891	-0.26974
WWP2	0.217051	0.661627	0.196629	0.074584	0.394384	0.125474	-0.04564	-0.34893

WWTR1	-0.09702	0.126348	-0.09336	-0.16689	-0.23562	0.138258	-0.17076	-0.23688
XAB2	0.414512	0.458213	0.078462	0.13193	0.286356	0.106363	0.195062	-0.13487
XAF1	-0.03772	0.256773	0.022485	-0.1075	-0.28975	0.317032	-0.51378	-0.11885
XAGE2	-0.34722	0.396889	NA	NA	NA	NA	-0.51212	-0.08942
XAGE3	-0.08898	0.060377	-0.20812	-0.22351	0.150851	-0.20689	-0.04623	0.201543
XAGE5	0.187051	0.302354	0.101154	0.166037	-0.26814	-0.49708	-0.13467	0.059764
XBP1	-0.43887	0.223918	0.147745	0.356119	0.089722	-0.27002	0.288164	0.385813
XCL1	0.095913	0.466421	-0.05862	-0.08173	-0.24987	-0.28497	-0.21971	0.327356
XCL2	0.409375	0.596566	-0.11083	-0.08822	-0.19457	-0.40105	NA	NA
XCR1	-0.07025	0.195716	0.281018	-0.58934	0.045737	-0.04819	0.331561	0.186666
XDH	0.073567	0.096839	0.223207	-0.11255	0.156488	0.100191	-0.04839	0.252069
XG	-0.08523	0.114356	-0.13273	-0.35624	-0.10673	0.019097	-0.02581	-0.06204
XIAP	NA	NA	-0.08362	-0.30851	-0.11476	-0.10036	NA	NA
XIRP1	NA	NA	0.013729	0.061968	-0.0767	0.084028	NA	NA
XIRP2	NA	NA	0.013091	0.021187	-0.17938	-0.00759	NA	NA
XIST	NA	NA	-0.05143	-0.04313	0.115892	0.256475	NA	NA
XK	-0.08326	-0.23513	-0.29823	-0.35675	-0.09946	-0.39997	0.081207	0.016798
XKR3	-0.0097	0.29744	0.073892	0.131961	-0.28433	0.038265	0.221287	-0.28847
XKR4	-0.15749	-0.34206	0.543868	-0.04345	-0.18751	0.105966	-0.33488	-0.3024
XKR5	0.016255	0.72075	0.089148	-0.35314	-0.21118	-0.32861	0.267581	0.469669
XKR6	0.307638	0.38024	0.148196	-0.07992	-0.00898	-0.12405	-0.2107	-0.11181
XKR7	0.04733	0.446258	NA	NA	NA	NA	-0.16196	-0.16069
XKR8	-0.05615	0.748646	0.080832	0.212343	-0.07844	-0.33246	0.061225	0.133142
XKR9	0.267169	0.162219	NA	NA	NA	NA	0.417816	0.206219
XKRX	0.104887	0.253762	-0.25923	0.235088	0.085907	-0.19331	0.071163	0.401581
XPA	-0.05922	0.053167	-0.21725	-0.02777	0.015259	-0.12759	-0.50806	-0.47865
XPC	0.201433	-0.42441	0.111217	0.187454	0.313062	0.150427	0.202831	0.425732
XPNPEP1	-0.32054	-0.08608	-0.01262	0.168313	0.094099	-0.40509	-0.09285	0.342959
XPNPEP2	0.570358	0.363133	0.366819	-0.40563	0.234649	-0.11939	-0.04228	0.460079
XPNPEP3	0.029374	-0.08504	0.040541	-0.06282	0.221924	0.066249	-0.56977	-0.54195
XPO1	0.303384	-0.74961	-0.30634	0.152467	-0.03245	0.253998	0.281551	0.055561
XPO4	0.531638	0.036951	-0.0868	-0.24244	0.129987	0.173378	-0.11868	-0.31638
XPO5	0.0837	0.74198	-0.25562	-0.1694	0.042996	0.318346	0.016635	-0.29846
XPO6	-0.23702	0.444357	-0.11154	0.222764	0.085098	-0.13321	0.208591	0.130355
XPO7	0.310399	0.090911	0.175213	-0.06056	-0.20733	0.112035	0.367718	0.523246
XPOT	NA	NA	-0.0884	0.114784	0.126645	0.036721	0.252349	0.335827
XPR1	0.058694	-0.12029	-0.05826	-0.44242	-0.15335	-0.60702	0.133394	-0.12778
XRCC1	0.159934	0.208816	0.159975	0.487508	0.387759	0.344878	0.603397	-0.05931
XRCC2	-0.07479	-0.08031	-0.32843	0.073425	-0.02161	-0.12532	-0.47679	-0.33018
XRCC3	-0.01861	0.423841	-0.17742	-0.16867	0.135408	-0.20052	0.272408	0.469762
XRCC4	-0.02627	-0.37297	0.359352	-0.26936	-0.22238	-0.60564	0.303609	0.357422
XRCC5	0.043998	0.346746	0.099075	0.231065	-0.1178	-0.05184	0.506549	-0.15744
XRCC6	-0.12568	-0.65911	-0.00375	0.193526	0.175163	0.04563	0.661532	0.161011
XRCC6BP1	0.092854	-0.19643	-0.20758	-0.3189	-0.13704	-0.38991	NA	NA
XRN1	0.160648	0.71647	-0.31361	-0.27775	-0.23503	-0.26762	-0.14761	0.330778
XRN2	0.20489	-0.51148	-0.30146	0.170467	0.218383	0.149205	-0.38808	-0.13247
XRRA1	NA	NA	0.085498	0.066924	-0.25934	-0.10199	NA	NA
XYLB	0.371438	-0.39242	0.268949	-0.10625	0.33252	-0.10807	NA	NA
XYLT1	-0.25475	0.715784	-0.05853	-0.1134	0.124732	-0.19191	0.497734	0.562743
XYLT2	-0.18476	-0.28567	0.278035	0.103439	-0.05289	0.064672	-0.29545	-0.43417
YAF2	0.047323	0.164693	-0.34399	-0.28222	-0.01016	-0.22304	-0.71857	-0.65108
YAP1	0.042742	-0.40199	0.102448	-0.00425	0.083884	-0.0118	0.189131	-0.15795

YARS	-0.03906	0.404599	0.333064	0.33774	0.404671	-0.05837	-0.36565	-0.0821
YARS2	0.497369	-0.29168	-0.34282	0.122495	-0.29247	0.014459	-0.21393	-0.60815
YBX1	NA	NA	0.22471	0.487555	0.418657	-0.15458	-0.26152	-0.61729
YBX2	0.491758	0.324338	-0.31152	-0.23706	-0.02356	-0.10464	0.275767	-0.07115
YDJC	NA	NA	-0.19478	0.092043	-0.00513	-0.11946	NA	NA
YEATS2	-0.24861	-0.60903	-0.21759	0.090846	0.028998	0.16494	0.186616	0.014434
YEATS4	-0.01617	-0.33727	-0.31772	0.317353	0.015793	-0.28785	0.207555	0.009953
YES1	-0.0209	-0.36753	-0.31321	-0.07243	-0.18747	-0.14017	-0.48993	-0.46337
YIF1A	0.047051	0.448733	0.145434	0.129271	0.092814	-0.20595	-0.36558	-0.22721
YIF1B	-0.08808	0.040874	0.287633	-0.03086	-0.03046	-0.35485	-0.11706	-0.23624
YIPF1	-0.38526	-0.30613	0.196491	-0.15775	0.148275	-0.41541	-0.30896	-0.12758
YIPF2	-0.17409	0.465611	0.347521	0.203926	0.096027	-0.22842	-0.46941	-0.46249
YIPF3	-0.13781	0.201016	-0.01863	0.177361	-0.0306	-0.25653	-0.11561	-0.30263
YIPF4	-0.01272	-0.35742	-0.02722	-0.49785	-0.14214	-0.57213	-0.27608	-0.455
YIPF5	-0.01212	0.190359	0.325048	-0.01977	-0.13493	-0.05034	-0.47418	-0.2829
YIPF6	0.283831	-0.49863	-0.08046	-0.37804	0.034885	-0.18239	0.252501	0.011893
YIPF7	0.318518	0.553723	0.326357	0.071055	0.165148	0.200363	NA	NA
YKT6	0.26445	0.236564	0.127739	0.168693	0.042588	-0.20494	-0.00651	0.038064
YLPM1	NA	NA	-0.27055	0.339003	0.081519	0.382377	NA	NA
YME1L1	-0.33718	-0.62309	-0.34616	-0.07097	-0.26428	-0.51446	0.333538	0.336511
YOD1	-0.42709	-0.33708	-0.35588	-0.20415	-0.30745	-0.12249	-0.19421	0.240319
YPEL1	-0.2621	-0.38217	0.044092	-0.05849	0.142698	0.242081	0.460173	0.186265
YPEL2	-0.17649	-0.0415	0.140338	-0.02725	-0.16118	-0.21239	0.073388	0.17902
YPEL3	-0.1614	0.180883	0.339385	0.026724	-0.02363	-0.02811	0.047946	0.164624
YPEL4	-0.13438	-0.25318	0.329223	-0.34761	-0.20962	0.065744	-0.35062	0.191345
YPEL5	-0.14898	-0.27311	0.109792	-0.2702	-0.10707	-0.05739	0.018194	-0.05295
YRDC	-0.21601	-0.13392	0.257699	0.168353	0.269069	-0.16581	-0.03696	0.08247
YSK4	0.307901	0.083966	0.170679	-0.26602	0.302521	0.091199	0.260582	0.241343
YTHDC1	-0.47123	-0.32031	-0.2047	0.029371	-0.13049	-0.02579	-0.62501	-0.34411
YTHDC2	-0.08831	-0.67316	0.005398	-0.13231	-0.19802	0.259414	0.281776	0.479032
YTHDF1	0.25481	0.081102	-0.11814	0.131006	0.341228	0.240681	0.343224	0.214733
YTHDF2	-0.26105	-0.61235	-0.04173	0.004269	0.255831	0.284103	0.428045	0.062469
YTHDF3	-0.16655	-0.7135	-0.15707	-0.20812	-0.49126	-0.06731	0.053354	-0.27933
YWHAB	0.441179	-0.40677	0.042869	-0.0789	0.393007	0.031196	0.233441	0.006752
YWHAE	-0.10364	-0.01422	-0.27008	0.245563	-0.29735	-0.47332	0.569448	0.166056
YWHAG	0.257507	0.643582	-0.09716	-0.28589	-0.00097	0.196894	-0.45927	-0.50934
YWHAH	-0.19414	-0.03776	-0.14372	0.046836	-0.08603	-0.00239	0.312741	-0.05064
YWHAQ	-0.06163	-0.51604	-0.0204	-0.01398	0.275048	-0.1357	0.148314	-0.24213
YWHAZ	-0.07168	-0.3331	0.120501	-0.13566	-0.09686	-0.2809	0.564105	0.459467
YY1	0.076281	-0.14323	-0.09442	-0.03894	0.160038	0.108256	-0.35951	-0.39397
YY1AP1	-0.50117	-0.01862	-0.24305	0.013861	-0.06095	-0.12699	-0.2663	-0.39563
YY2	-0.15855	-0.43122	NA	NA	NA	NA	0.250914	0.229584
ZACN	NA	NA	-0.09889	0.01245	0.110418	0.058202	NA	NA
ZADH2	0.05516	0.037478	-0.20049	0.000577	-0.02644	-0.3292	-0.11734	-0.14526
ZAK	-0.05105	-0.44055	-0.09377	-0.13054	0.169238	-0.08974	0.104692	-0.26193
ZAN	0.22837	0.222878	-0.1844	-0.32357	0.109771	-0.18972	-0.32597	0.019168
ZAP70	-0.0304	0.683344	0.243255	0.681149	-0.15689	0.760072	-0.45004	0.330391
ZAR1	0.336556	0.522505	-0.36106	-0.04482	0.001507	-0.0508	0.460801	0.269634
ZBBX	NA	NA	0.11423	-0.29452	0.110668	-0.26461	NA	NA
ZBED1	-0.05074	-0.00047	0.204055	0.25598	0.329754	0.177863	0.289921	0.195056
ZBED2	-0.04467	0.464697	0.062328	0.440832	-0.12926	0.38079	-0.00864	-0.14993
ZBED3	0.36809	0.499343	0.109742	-0.01238	-0.02248	0.3616	-0.29635	-0.3246

ZBED4	0.014904	-0.2647	-0.3478	0.083206	0.182924	-0.15502	0.593844	0.334054
ZBED5	-0.25865	-0.66883	-0.43144	-0.03865	0.021723	0.010055	-0.46528	-0.42088
ZBP1	0.049034	0.295769	0.392311	0.137327	-0.00994	0.270388	-0.43558	-0.17879
ZBTB1	0.331767	0.552798	-0.48816	0.089362	-0.08007	-0.09779	0.140978	0.244667
ZBTB10	0.114117	-0.15314	-0.16972	-0.03678	-0.16078	-0.24965	0.253052	0.20334
ZBTB11	-0.0101	-0.60171	-0.24509	-0.21967	-0.26302	0.103334	-0.52355	-0.38761
ZBTB12	-0.30187	0.153569	0.2103	-0.22521	-0.01619	0.084707	0.226568	-0.39703
ZBTB16	-0.62811	0.192999	0.32844	-0.11802	0.010763	0.03446	0.171978	-0.1388
ZBTB17	-0.41049	0.056988	0.186316	-0.07877	0.156664	0.075708	-0.02766	0.410432
ZBTB2	0.469186	-0.50037	-0.31375	0.15694	0.252332	0.09003	0.037322	-0.01846
ZBTB20	0.242856	-0.12885	0.139209	-0.26549	0.000312	0.295332	0.126287	-0.15323
ZBTB22	0.178014	0.646658	0.089368	0.038388	0.028719	-0.15863	NA	NA
ZBTB24	0.460034	0.482885	-0.32897	-0.06366	0.168503	0.049808	-0.0197	0.062469
ZBTB25	-0.18238	-0.02461	-0.06659	0.211769	-0.24214	0.196484	-0.30693	0.14309
ZBTB26	0.23878	-0.32715	-0.03628	-0.38744	0.10711	-0.07578	0.309464	0.107003
ZBTB3	0.104687	0.514033	-0.1931	0.095163	0.033803	-0.11812	-0.32475	-0.28165
ZBTB32	0.047084	0.676348	0.101702	0.79065	-0.06796	0.531144	-0.13823	0.257901
ZBTB33	0.466216	0.156206	-0.22526	-0.02581	0.164183	0.041581	0.244599	0.04859
ZBTB34	NA	NA	-0.01034	-0.29251	-0.27748	0.184446	NA	NA
ZBTB37	0.034634	0.568645	0.255652	-0.10912	-0.26694	0.008786	-0.00178	-0.0778
ZBTB38	NA	NA	0.060153	-0.19901	-0.01771	0.192215	-0.07409	0.104878
ZBTB39	0.394536	0.755608	-0.26689	0.284675	-0.04642	-0.37931	0.387904	0.261972
ZBTB4	-0.23035	-0.43644	0.124667	0.066705	-0.11073	0.295047	-0.24748	-0.21112
ZBTB40	-0.40219	-0.03221	0.040808	0.039432	0.03758	0.188096	-0.52131	-0.30953
ZBTB41	-0.02742	-0.58876	-0.19473	-0.17301	-0.10452	0.003249	0.451148	0.124088
ZBTB43	-0.296	-0.21649	0.222917	-0.16411	0.178903	0.277666	NA	NA
ZBTB44	NA	NA	-0.2595	-0.15945	-0.23912	0.113973	-0.41467	-0.37626
ZBTB45	0.410959	0.555566	-0.2343	-0.00831	0.079332	0.216373	NA	NA
ZBTB46	-0.2558	0.058018	0.040458	0.107762	0.080934	0.369793	NA	NA
ZBTB47	-0.23001	0.000797	0.219061	-0.10542	-0.09171	0.076876	NA	NA
ZBTB48	0.067835	0.545603	0.253307	-0.09816	0.265934	0.218806	NA	NA
ZBTB5	0.115367	-0.11832	-0.00999	0.057881	0.084898	0.011286	0.479604	0.313611
ZBTB6	-0.56473	-0.50811	-0.27995	0.10231	0.206357	-0.32763	NA	NA
ZBTB7A	0.197462	0.667571	0.098344	-0.08889	0.22921	0.178561	-0.19508	0.040066
ZBTB7B	0.24704	0.645415	-0.26265	0.041773	0.02423	-0.17045	0.361516	0.363784
ZBTB7C	NA	NA	0.314326	-0.15033	-0.02327	-0.03262	NA	NA
ZBTB8OS	-0.28565	-0.27093	0.278529	-0.21985	-0.1776	-0.41368	-0.56088	-0.47496
ZBTB9	0.411858	0.39203	-0.22304	0.249825	0.135166	0.245144	0.548489	0.277791
ZC3H10	0.123204	0.045311	-0.03484	0.044552	-0.09585	-0.17301	-0.00296	0.055275
ZC3H11A	0.03444	-0.28148	-0.25831	-0.11142	-0.11534	0.168168	0.006198	0.027684
ZC3H12A	-0.0269	0.36813	0.172785	0.346548	0.210944	0.100911	-0.24377	0.180919
ZC3H12B	0.450129	0.153569	NA	NA	NA	NA	0.323566	0.066712
ZC3H12C	NA	NA	-0.16427	-0.08865	-0.05146	-0.06124	NA	NA
ZC3H12D	NA	NA	-0.22503	0.187048	0.016043	0.196725	NA	NA
ZC3H13	0.367313	0.840331	-0.1232	0.324298	0.100053	0.079596	NA	NA
ZC3H14	0.143865	-0.25882	-0.13727	-0.23532	-0.08502	-0.02359	0.417729	0.282029
ZC3H15	NA	NA	-0.23571	-0.24723	-0.08442	-0.03213	0.093622	-0.128
ZC3H18	NA	NA	0.045704	0.075797	0.465713	0.422462	NA	NA
ZC3H3	0.328519	0.481957	0.17386	-0.08468	0.028826	-0.00344	0.43705	0.271766
ZC3H4	NA	NA	-0.18214	0.226136	0.228265	0.134318	NA	NA
ZC3H6	0.02696	-0.35642	-0.10971	-0.07304	-0.14847	-0.14524	-0.41895	-0.31132
ZC3H7A	-0.32898	-0.63786	-0.22688	-0.20772	-0.08003	0.160063	-0.39671	-0.25753

ZC3H7B	-0.13022	-0.28346	-0.25943	0.119041	-0.03148	-0.01147	-0.23818	-0.32459
ZC3H8	0.247284	-0.47537	-0.28609	0.065196	0.013088	0.038383	0.138835	-0.17306
ZC3HAV1	-0.11188	-0.10541	0.083141	0.084494	-0.03672	0.102975	-0.23682	0.254165
ZC3HAV1L	0.12563	0.293944	-0.13552	-0.11293	0.042443	0.148674	-0.44763	-0.35584
ZC3HC1	0.317995	-0.0054	0.109253	0.355046	-0.12652	0.415831	0.292492	0.018835
ZC4H2	NA	NA	-0.32157	-0.16325	-0.06408	-0.20981	NA	NA
ZCCHC10	-0.08588	0.252241	0.099307	-0.23839	-0.22672	-0.512	0.089131	-0.38467
ZCCHC11	-0.11925	-0.67163	-0.09726	0.235637	0.027747	0.304983	0.423819	0.210448
ZCCHC12	-0.22347	-0.36682	-0.19826	-0.0396	-0.08425	0.120321	0.026542	-0.16292
ZCCHC13	0.068323	0.13908	0.035948	0.071356	-0.11009	0.163605	-0.38957	0.31004
ZCCHC14	-0.00547	-0.61353	0.188401	-0.22602	0.296876	-0.04291	0.31993	0.176574
ZCCHC16	0.109828	0.351273	NA	NA	NA	NA	0.291966	0.225321
ZCCHC17	0.218991	-0.10726	-0.10246	-0.27064	-0.18662	-0.21234	0.455751	0.320493
ZCCHC18	NA	NA	-0.12536	0.378488	-0.26848	0.227653	NA	NA
ZCCHC2	0.26405	0.296794	0.186435	-0.18308	-0.09172	0.085912	-0.6138	-0.3261
ZCCHC24	NA	NA	0.07143	-0.04498	0.044915	0.227469	NA	NA
ZCCHC3	0.281493	0.060322	-0.27293	0.36649	0.2095	0.145575	0.659403	0.363786
ZCCHC4	NA	NA	-0.26468	-0.15039	0.134917	-0.44248	NA	NA
ZCCHC5	0.285975	0.167176	0.223648	-0.06399	0.121531	0.098115	-0.23543	-0.41142
ZCCHC6	-0.03295	0.415247	-0.21925	-0.20995	-0.10652	-0.21604	-0.65773	-0.45624
ZCCHC7	0.248039	0.174072	-0.27223	-0.03507	-0.23649	0.061987	0.146911	0.06086
ZCCHC8	0.020328	0.0722	-0.28286	0.145232	0.03606	-0.44478	0.194787	0.491271
ZCCHC9	0.288206	-0.60079	0.133895	-0.0272	-0.21062	-0.26544	0.432723	0.212954
ZCRB1	0.164337	0.136721	-0.21336	-0.31404	-0.28327	-0.44949	-0.22012	-0.20653
ZCWPW1	-0.51452	0.15815	-0.07018	0.24014	0.230463	0.118799	-0.4346	-0.22905
ZCWPW2	NA	NA	NA	NA	NA	NA	-0.28844	-0.18172
ZDBF2	NA	NA	-0.1225	-0.18685	-0.17898	0.011681	NA	NA
ZDHHC1	0.133185	0.397445	0.464919	-0.12738	0.285721	-0.19303	-0.19951	-0.53502
ZDHHC11	-0.32344	-0.10462	0.039659	0.074381	0.122405	0.015088	-0.09529	-0.11487
ZDHHC12	-0.01528	0.332118	0.232573	-0.00585	0.118943	-0.42146	0.361018	0.016834
ZDHHC13	-0.00495	0.021071	-0.17561	-0.16689	-0.10949	-0.12721	0.432938	0.208712
ZDHHC14	0.14407	0.301633	-0.1891	-0.12122	-0.00523	0.144095	-0.31409	-0.08548
ZDHHC15	0.195109	-0.02875	-0.07101	0.043479	-0.1703	-0.26911	-0.30845	-0.07745
ZDHHC16	0.010246	0.59676	0.138549	0.231143	-0.04469	-0.44447	0.067605	-0.08603
ZDHHC17	0.350333	-0.36246	-0.06225	-0.10015	-0.01325	0.232713	-0.14969	0.221945
ZDHHC18	-0.35915	0.196713	0.43279	0.116495	0.170559	0.115604	-0.29995	0.056858
ZDHHC19	0.145757	0.410782	0.152492	-0.05815	-0.02453	-0.33978	-0.47003	0.360204
ZDHHC2	0.247044	0.052291	0.243835	0.175111	-0.07931	-0.10527	-0.05392	-0.16235
ZDHHC20	-0.16005	-0.51008	-0.03783	-0.4939	0.142463	-0.25485	0.377851	-0.05563
ZDHHC21	-0.00933	-0.27176	0.000488	-0.16092	0.118421	0.089737	-0.23412	0.132511
ZDHHC22	0.053752	0.096736	-0.16218	-0.12923	0.301043	0.124998	-0.13837	-0.03961
ZDHHC23	0.290536	0.085862	-0.11839	-0.1596	0.199691	0.100001	-0.09063	-0.33838
ZDHHC24	0.266033	0.700784	0.113813	0.259921	-0.05861	-0.32452	0.221748	0.202113
ZDHHC3	0.250612	-0.15304	-0.0089	0.112183	-0.03442	-0.35283	0.39839	0.429903
ZDHHC4	0.2029	0.193933	0.059071	-0.26958	-0.03544	-0.24233	-0.45387	-0.05331
ZDHHC5	0.162318	0.63869	-0.09527	0.118219	-0.05229	-0.15224	-0.1695	-0.2127
ZDHHC6	-0.03768	-0.54248	-0.1106	0.037857	0.234614	-0.24597	0.52274	0.632275
ZDHHC7	-0.43423	-0.41995	-0.21622	0.163659	0.101887	-0.08552	0.015847	0.049545
ZDHHC8	0.16982	-0.25237	0.207747	-0.11141	0.286022	-0.02836	0.191332	-0.11704
ZDHHC9	0.499316	0.228615	0.173576	0.318901	0.288931	-0.06818	0.291005	0.29283
ZEB1	-0.31018	-0.29717	-0.0483	-0.09835	-0.06579	-0.00935	NA	NA
ZEB2	-0.53634	-0.67416	0.066213	0.126747	-0.23079	0.295999	NA	NA

ZER1	0.136879	0.465671	0.336185	-0.39258	0.213204	-0.00604	NA	NA
ZFAND1	0.131396	-0.07028	0.053977	-0.2454	-0.13909	-0.05415	0.164059	-0.30765
ZFAND2A	0.150652	0.229408	-0.23704	-0.32914	-0.03086	-0.06734	-0.06529	-0.32575
ZFAND2B	0.068947	0.436401	0.19724	-0.28106	0.085891	-0.30057	0.49306	0.465227
ZFAND3	0.167308	-0.14371	0.101442	-0.25192	0.113164	-0.06927	0.238191	0.179979
ZFAND5	-0.21899	-0.67013	0.059324	-0.295	-0.12659	0.163326	NA	NA
ZFAND6	-0.20684	-0.5519	-0.16042	-0.35527	-0.07369	0.071379	-0.38782	-0.22341
ZFAT	NA	NA	-0.35529	0.30089	-0.20381	-0.27152	NA	NA
ZFC3H1	NA	NA	0.015157	-0.03644	-0.08291	0.179793	NA	NA
ZFHX2	0.152645	0.007428	-0.32377	0.020228	-0.26234	0.108325	NA	NA
ZFHX3	NA	NA	-0.011	-0.14467	-0.03398	0.32196	NA	NA
ZFHX4	-0.34356	-0.32906	0.1121	-0.15969	-0.04572	-0.22921	-0.22813	-0.29279
ZFP1	0.095559	-0.35887	-0.14241	-0.29089	0.143161	-0.24817	-0.33152	-0.48803
ZFP106	-0.12141	-0.52535	-0.04598	-0.15302	-0.32267	0.074937	0.110327	0.105247
ZFP112	NA	NA	-0.06172	-0.48525	-0.09459	-0.09169	NA	NA
ZFP14	-0.05226	-0.14853	0.065972	-0.09068	0.106506	0.034416	NA	NA
ZFP161	0.037161	-0.11392	-0.52853	0.306014	-0.03095	-0.26158	0.071081	0.298105
ZFP2	0.217514	0.093036	-0.07157	-0.19981	-0.02011	-0.13755	-0.07321	-0.03225
ZFP28	0.492927	0.224115	0.264132	-0.1476	-0.09223	0.468451	0.32951	0.126254
ZFP3	0.143804	-0.02069	-0.15871	-0.29265	-0.27721	-0.23684	0.274504	0.280826
ZFP30	0.367842	-0.39766	-0.00751	-0.05849	-0.23306	0.21751	NA	NA
ZFP36	-0.45389	-0.30917	0.011001	0.070657	-0.03583	-0.0975	-0.51121	-0.33018
ZFP36L1	0.068164	-0.46913	0.257438	0.04786	-0.02818	0.284222	-0.58307	-0.47058
ZFP36L2	NA	NA	0.186454	0.127452	0.27143	0.121242	0.181077	0.023695
ZFP37	0.16784	-0.09312	-0.18795	0.147308	-0.15514	-0.0232	-0.29516	-0.08966
ZFP41	0.120759	-0.11355	-0.12738	-0.21902	-0.00984	0.079632	-0.30581	-0.24257
ZFP42	-0.2598	0.501768	-0.26953	0.13198	-0.15677	-0.0006	-0.57413	-0.44313
ZFP57	NA	NA	0.301484	0.003457	0.163528	-0.18889	NA	NA
ZFP62	NA	NA	0.039424	-0.14427	0.105533	0.204907	NA	NA
ZFP64	0.205653	0.334707	-0.47768	0.217599	0.052922	-0.18525	0.623384	0.359864
ZFP82	NA	NA	-0.15269	-0.09213	0.111511	0.207513	NA	NA
ZFP90	0.15286	0.071329	0.062422	-0.03258	0.108244	0.098173	-0.20082	-0.41539
ZFP91	-0.33164	-0.09264	0.072303	-0.1119	0.016904	0.42177	-0.6658	-0.39676
ZFPL1	0.350055	0.753783	0.14189	0.157311	0.284635	-0.10745	0.615496	0.289837
ZFPM1	-0.23521	0.569831	0.155133	-0.05708	0.008155	0.267479	0.395026	0.097503
ZFPM2	-0.14172	-0.39234	-0.03464	0.00828	-0.3486	0.114508	-0.21331	-0.04827
ZFR	0.27667	-0.48072	-0.19536	-0.47277	-0.00363	-0.00272	-0.28131	-0.44949
ZFR2	NA	NA	-0.01379	0.124555	-0.16944	0.421346	NA	NA
ZFX	-0.30236	-0.65019	-0.12372	-0.30184	-0.06781	0.050892	0.063042	0.21108
ZFY	0.299006	0.429456	-0.07838	-0.04712	-0.28298	-0.31316	0.426676	-0.06292
ZFYVE1	-0.09643	0.417658	-0.04532	-0.22825	-0.29489	-0.248	-0.21185	-0.31898
ZFYVE16	0.135895	-0.66775	0.016426	-0.34304	-0.0259	0.153771	0.37755	0.255591
ZFYVE19	-0.25358	0.177405	-0.00255	0.06344	-0.29444	-0.31278	0.414225	0.449003
ZFYVE20	-0.34021	-0.32383	-0.31971	-0.38179	-0.16604	0.164413	-0.50215	-0.15621
ZFYVE21	-0.41843	-0.13597	-0.09631	-0.07026	-0.09298	0.129988	0.078465	0.080965
ZFYVE26	-0.25735	-0.43544	-0.15931	-0.03764	-0.2103	0.067936	-0.3126	0.188109
ZFYVE27	0.566257	0.113594	-0.05048	-0.02129	0.43619	0.276087	0.158793	0.327511
ZFYVE28	0.218759	0.357762	0.050735	-0.39153	0.016381	-0.06557	-0.3092	0.04886
ZFYVE9	0.425448	0.700344	0.275258	-0.22127	-0.04668	0.015209	0.4177	0.223935
ZG16	-0.01241	0.16071	NA	NA	NA	NA	NA	NA
ZGPAT	0.288038	0.571737	0.342601	0.228474	0.323569	0.288489	0.355492	0.332345
ZHX1	-0.05544	0.105338	0.050827	-0.47115	0.134057	-0.1241	-0.26092	-0.19789

ZHX2	0.245332	-0.52185	0.242484	0.502757	0.081467	0.374947	-0.02481	-0.01539
ZHX3	-0.05618	0.069612	0.17619	-0.0922	0.499031	0.236805	-0.20695	-0.2802
ZIC1	0.006791	-0.21236	-0.17191	-0.04881	-0.05795	-0.22577	0.16897	-0.01192
ZIC2	-0.51705	-0.25172	0.071817	0.158305	-0.14565	-0.22037	-0.01075	0.139807
ZIC3	0.184926	0.744211	-0.23538	-0.22669	0.043159	0.106307	-0.08566	-0.22739
ZIC4	-0.07246	-0.18144	0.142193	-0.14922	0.169987	-0.0396	0.17951	0.032761
ZIC5	-0.40097	0.350574	0.065887	-0.12218	-0.25013	-0.02676	0.097296	-0.11161
ZIK1	-0.27007	-0.07259	0.160766	0.266531	-0.25519	0.602484	-0.39011	0.234079
ZIM2	0.018405	0.029581	0.208546	-0.08333	-0.0377	-0.04444	-0.23719	0.017612
ZIM3	0.224613	0.73957	-0.03569	0.080281	-0.03182	0.29574	0.588315	0.334232
ZKSCAN1	0.144801	0.165516	0.086703	-0.16741	0.142552	0.273866	-0.21067	-0.18324
ZKSCAN2	0.362175	0.301666	0.028326	-0.06978	0.057018	0.11411	NA	NA
ZKSCAN3	0.241234	-0.19995	-0.26227	0.031587	-0.35172	-0.2692	NA	NA
ZKSCAN4	0.496769	-0.22924	-0.11853	-0.11748	0.002884	0.005054	NA	NA
ZKSCAN5	0.387053	0.662664	-0.19432	-0.0812	0.160835	-0.52946	NA	NA
ZMAT1	0.194043	0.24644	-0.23006	0.251552	-0.1471	0.452374	-0.1439	0.379545
ZMAT2	0.229424	0.385417	-0.00399	0.176001	-0.16491	-0.06854	0.096651	-0.05221
ZMAT3	-0.26016	-0.39984	0.097805	-0.37044	0.025339	-0.38522	-0.54353	-0.50056
ZMAT4	-0.05153	0.447258	-0.0625	-0.13491	-0.36485	-0.09159	NA	NA
ZMAT5	0.092614	-0.64425	0.298604	-0.17228	-0.09949	-0.32286	0.19239	-0.03901
ZMIZ1	-0.51301	-0.69487	-0.18064	-0.45995	0.010474	-0.13822	NA	NA
ZMIZ2	0.363958	0.241291	-0.06369	0.459956	0.182742	0.454354	-0.40631	-0.40412
ZMPSTE24	-0.04619	-0.12406	-0.08582	-0.25533	0.397604	-0.10604	-0.19049	-0.12396
ZMYM1	-0.10848	0.378888	0.087286	-0.01986	-0.11503	0.046869	-0.06958	-0.14878
ZMYM2	0.136801	-0.67117	-0.00558	-0.28646	0.1284	0.08765	NA	NA
ZMYM3	0.342475	0.300503	0.133708	0.095638	0.330535	0.264403	0.028436	-0.03828
ZMYM4	0.352354	-0.14051	-0.22181	-0.06174	-0.18383	-0.10965	0.337138	0.069215
ZMYM5	0.204937	-0.34356	0.034617	-0.37378	0.136271	0.019705	0.615494	0.361408
ZMYM6	0.224017	-0.3334	0.355871	-0.28804	-0.13238	0.088856	-0.21231	-0.20242
ZMYND10	-0.41728	0.650714	-0.12318	-0.22599	-0.00602	-0.09512	0.222755	-0.01969
ZMYND11	-0.51883	-0.4992	-0.38469	-0.14037	-0.13343	-0.14503	-0.0492	0.110432
ZMYND12	-0.04466	0.563267	-0.00913	-0.46467	-0.23676	-0.06282	0.043267	-0.17026
ZMYND15	-0.27071	0.194084	0.060219	-0.00231	-0.33482	-0.06297	-0.30325	0.455478
ZMYND17	0.186631	0.245664	-0.11744	0.09301	0.147184	0.063665	-0.15713	-0.12798
ZMYND19	0.435795	0.454454	0.134495	0.399465	0.351794	0.241399	0.270533	0.309857
ZMYND8	0.24842	-0.45041	0.159635	-0.09337	0.426524	0.190677	0.376008	0.08953
ZNF10	0.148046	-0.2596	-0.24155	-0.08407	-0.10394	0.265184	0.368831	0.420007
ZNF100	-0.07387	0.413343	-0.50325	0.052016	-0.10257	0.058125	-0.09353	-0.01579
ZNF101	0.023649	0.154813	-0.1614	0.058481	-0.32393	-0.04984	0.012177	0.266213
ZNF107	NA	NA	-0.30882	0.348996	-0.22908	0.067895	NA	NA
ZNF114	-0.32962	-0.0457	-0.10201	0.165732	0.202367	0.170926	-0.48439	-0.44197
ZNF117	0.372146	0.257563	-0.42253	0.087712	-0.32534	-0.09187	0.451313	0.228755
ZNF12	0.385447	-0.72232	-0.01749	-0.21351	0.117	0.200317	-0.36084	-0.40486
ZNF121	0.199517	-0.11331	-0.26041	-0.18816	-0.24832	-0.03802	0.340307	-0.09129
ZNF124	-0.01856	-0.32128	-0.20112	-0.19647	-0.18511	-0.16012	-0.13965	-0.29411
ZNF131	NA	NA	-0.12057	-0.09849	0.208371	0.030419	-0.26631	-0.5044
ZNF132	-0.24071	-0.33942	-0.09343	-0.05531	-0.32982	-0.04801	-0.30064	0.331426
ZNF133	-0.0472	0.142099	-0.29628	-0.07532	0.050104	0.080004	0.565172	0.367228
ZNF134	-0.36309	0.192552	-0.19871	0.004301	-0.35865	0.136371	0.467852	0.412832
ZNF135	-0.09644	-0.00618	0.017164	-0.05609	-0.1392	0.303151	-0.32736	0.119651
ZNF136	0.165417	0.217678	0.027456	-0.14842	0.13914	0.161724	-0.34713	-0.00411
ZNF137	0.064158	0.141457	-0.44431	-0.0601	-0.08741	-0.31622	-0.055	0.083568

ZNF138	0.130539	-0.03751	-0.24492	-0.21498	-0.24858	-0.49796	NA	NA
ZNF14	-0.14573	-0.15201	-0.22169	-0.11714	-0.15943	-0.13141	-0.41957	-0.39856
ZNF140	0.144804	0.534487	-0.36137	-0.2015	-0.21278	-0.21525	-0.55295	-0.51012
ZNF141	0.319427	0.078588	-0.33116	0.043477	-0.28477	-0.05382	-0.13739	-0.1505
ZNF142	0.378746	0.507739	0.057243	0.116126	0.320137	0.342163	-0.34033	-0.18921
ZNF143	-0.01024	-0.62306	-0.26825	-0.20109	-0.40845	-0.47756	0.244337	0.019045
ZNF146	0.280607	0.639711	-0.5194	0.10674	-0.21963	-0.27546	0.051434	-0.21863
ZNF148	0.296614	0.320757	0.070494	-0.36337	0.013928	0.013078	0.160837	0.11891
ZNF154	0.157169	0.03799	-0.16489	0.094401	0.006137	0.465689	NA	NA
ZNF155	-0.08075	0.654696	-0.29671	-0.05743	-0.36152	-0.30999	0.100453	0.08852
ZNF157	0.308264	0.288996	0.129863	-0.14298	-0.01557	-0.42018	0.031739	0.396933
ZNF16	0.23108	0.241466	0.0812	-0.18174	-0.068	-0.05154	0.434234	0.263271
ZNF160	0.120914	0.590423	-0.33097	-0.18788	-0.04343	0.112981	-0.44796	-0.41056
ZNF165	0.065973	-0.05084	-0.42353	-0.16214	-0.24911	-0.01859	0.151404	0.039215
ZNF167	0.107174	0.007375	0.287217	0.148705	-0.13967	0.048809	-0.07001	0.332619
ZNF169	-0.06122	-0.10111	-0.09087	-0.05304	0.062637	0.271184	0.248357	0.413738
ZNF17	0.393026	0.19366	-0.369	-0.43215	-0.17269	-0.37346	0.364394	0.193229
ZNF174	0.309821	-0.04294	-0.03358	-0.01948	0.233932	-0.2582	0.116187	0.004096
ZNF175	0.509566	0.465174	-0.17542	-0.21859	-0.06894	-0.21386	-0.39955	0.058125
ZNF177	0.486881	0.121755	-0.25981	-0.09364	0.083478	-0.01803	0.25772	0.301791
ZNF18	-0.11972	-0.18467	-0.08804	-0.08696	-0.16835	-0.24336	0.389876	0.573684
ZNF180	0.133818	0.718931	-0.36124	-0.40993	-0.05607	-0.2249	NA	NA
ZNF181	0.004803	-0.63432	-0.21001	0.127026	-0.17742	-0.35957	-0.12968	-0.11751
ZNF182	0.196526	0.735685	-0.22157	0.150052	0.068511	-0.07297	0.290039	0.456438
ZNF184	-0.02013	-0.35724	-0.47281	-0.39465	-0.11852	-0.25726	0.106358	-0.0214
ZNF185	-0.15829	-0.32176	-0.16496	-0.11203	-0.17942	0.023315	NA	NA
ZNF187	0.326931	0.463701	-0.29286	-0.32655	-0.28429	-0.14977	-0.28374	-0.14904
ZNF189	0.132696	0.014723	-0.0516	-0.26163	0.084842	0.002295	0.007122	-0.0651
ZNF19	0.167532	0.196127	0.015544	-0.10783	0.004951	-0.19482	0.415122	0.116652
ZNF192	0.189718	0.694586	-0.16186	-0.12827	0.179959	0.062026	-0.2184	-0.5154
ZNF193	0.260903	-0.39282	-0.29214	-0.09039	0.014552	0.028935	0.567829	0.383618
ZNF195	0.100271	-0.1782	-0.27703	-0.04009	-0.21168	-0.11359	0.401421	0.336473
ZNF197	0.313912	-0.17825	-0.28981	0.088424	-0.28139	-0.19017	-0.13982	-0.19543
ZNF2	0.202405	0.320997	0.054076	0.03662	-0.0879	-0.26377	-0.08961	-0.17654
ZNF20	0.280111	0.267749	-0.07266	-0.35624	-0.10836	-0.41211	-0.00291	-0.24284
ZNF200	0.131587	-0.03097	-0.42634	-0.05935	-0.07743	-0.34671	0.351246	0.345113
ZNF202	-0.05035	-0.5104	-0.12964	-0.04814	0.117523	0.281501	0.022686	0.247674
ZNF205	0.133622	0.368082	0.273121	-0.04206	0.223837	0.134605	0.524293	0.180504
ZNF207	0.673396	0.376973	0.042471	-0.01694	-0.03791	0.186864	-0.15587	-0.13308
ZNF208	0.360862	0.593286	-0.25532	-0.02824	-0.20145	0.204309	0.311667	-0.0021
ZNF211	-0.30042	-0.07365	-0.19674	0.003473	-0.27145	0.117249	0.062782	0.35142
ZNF212	0.139021	0.461945	-0.1373	0.165458	0.270269	0.231197	-0.32311	-0.0059
ZNF213	-0.07067	-0.15873	0.137315	0.015883	0.155311	-0.1675	0.525693	0.116918
ZNF214	0.072305	-0.48172	-0.53969	-0.31334	-0.16724	-0.61315	-0.40953	-0.37873
ZNF215	0.041555	0.532211	0.037329	-0.04167	0.23451	0.072505	-0.03087	0.190468
ZNF217	0.099932	-0.3667	0.006536	-0.12625	0.55569	0.082996	0.323195	0.002285
ZNF219	0.264367	-0.04825	-0.01811	0.066062	0.055306	-0.01598	-0.07695	-0.0024
ZNF22	0.012065	0.037012	-0.60705	-0.01664	-0.14415	0.053056	-0.00415	-0.26717
ZNF221	0.49385	0.246751	0.017297	-0.32562	0.08077	-0.34931	-0.15362	0.268093
ZNF222	-0.1518	-0.52359	-0.1327	-0.07468	-0.42158	-0.08845	-0.18357	-0.30005
ZNF223	-0.18867	0.424234	-0.14052	-0.23596	-0.37104	-0.62061	-0.60287	-0.61222
ZNF224	-0.05035	-0.29785	-0.10351	-0.1046	-0.15155	-0.05432	0.541722	0.47814

ZNF225	-0.55865	0.242425	-0.34891	-0.00888	-0.17737	-0.12754	0.491414	0.558211
ZNF226	-0.02234	0.283525	-0.24765	-0.32262	-0.43686	-0.1578	-0.13199	-0.10569
ZNF227	0.323062	0.182415	-0.36331	-0.15316	-0.31474	-0.52373	-0.33335	-0.42963
ZNF229	-0.04065	-0.33701	-0.29065	0.004932	-0.01707	0.048872	-0.30863	0.135784
ZNF23	0.18383	-0.59306	-0.43231	-0.05684	-0.00431	-0.00292	-0.6081	-0.38952
ZNF230	-0.15822	0.167311	-0.26939	0.095883	-0.30566	-0.48879	0.640747	0.487356
ZNF232	-0.16924	-0.19356	-0.32437	0.001006	-0.31935	-0.08193	-0.24663	-0.30016
ZNF233	0.392042	-0.30179	-0.00827	-0.15246	-0.08112	-0.12679	0.035749	-0.15083
ZNF234	-0.12389	-0.06384	0.073094	-0.26303	-0.0693	-0.04075	-0.06452	-0.19659
ZNF235	-0.05983	-0.54781	0.075918	-0.50287	-0.33291	0.00358	0.647169	0.52985
ZNF236	-0.18353	-0.46629	0.134468	-0.0509	0.050163	0.524013	0.297104	0.506025
ZNF238	0.192898	-0.40798	-0.02815	0.342094	0.188167	-0.00178	0.176073	-0.14448
ZNF239	0.120914	-0.30476	-0.41827	-0.18312	0.096767	0.02932	0.188112	0.242267
ZNF24	-0.08951	-0.35825	0.210378	-0.12288	0.006177	0.228568	0.198229	0.482869
ZNF248	-0.13422	-0.07067	-0.12423	-0.10894	-0.03644	0.045026	-0.67869	-0.25025
ZNF25	-0.41732	-0.41678	0.109575	-0.11173	-0.2185	-0.16214	-0.37745	-0.26394
ZNF250	-0.20526	-0.39526	-0.06315	-0.23178	-0.09487	-0.21763	0.325534	0.095571
ZNF251	NA	NA	0.112721	0.116678	0.121495	-0.11452	NA	NA
ZNF253	0.264553	0.20892	-0.30271	-0.11353	-0.28326	-0.51366	0.015427	0.249353
ZNF254	0.154268	-0.11444	-0.29686	-0.02496	-0.23852	-0.07006	0.081286	0.071355
ZNF256	0.298817	0.667625	-0.01656	0.008029	-0.34504	0.238864	-0.20391	0.029925
ZNF257	0.257883	0.394762	0.078752	0.172116	-0.26869	-0.12147	0.216778	0.196694
ZNF259	0.461641	0.054439	-0.00914	0.172076	0.03901	0.153845	-0.23913	-0.45875
ZNF26	-0.00103	-0.30553	-0.23083	-0.23902	0.091185	0.448475	0.349788	0.462152
ZNF260	0.388233	0.262292	NA	NA	NA	NA	NA	NA
ZNF263	0.228271	-0.34731	-0.02959	0.088634	0.30355	0.126785	-0.29592	-0.22532
ZNF264	-0.36755	0.010001	0.003352	0.005494	0.042599	0.272914	-0.42186	-0.39975
ZNF266	0.274859	0.141104	-0.17328	0.060976	-0.07063	0.264237	-0.31008	0.227514
ZNF267	0.04571	-0.38033	-0.34742	0.130558	-0.20332	-0.07434	-0.06212	-0.24527
ZNF268	0.206068	0.332881	-0.11222	-0.20323	-0.01295	0.192049	-0.40822	-0.47001
ZNF271	-0.17519	-0.29718	-0.15802	-0.34357	-0.06699	-0.14098	-0.36602	-0.23632
ZNF273	0.294638	-0.48082	-0.19679	0.025659	-0.25862	0.052921	0.039359	0.112917
ZNF274	0.061111	-0.29155	-0.29112	0.175664	-0.18581	0.139909	-0.11868	0.348906
ZNF275	NA	NA	0.147643	-0.03408	0.296058	0.367335	0.329633	0.085328
ZNF276	-0.14157	0.54259	0.083919	0.276036	0.03215	0.353438	-0.70681	0.289177
ZNF277	NA	NA	-0.03634	-0.23219	-0.17032	-0.48293	0.45903	0.05051
ZNF28	-0.03279	0.29931	-0.24353	-0.16659	0.13547	0.090012	-0.40986	-0.47346
ZNF280A	NA	NA	-0.02322	0.148452	-0.02901	0.412791	NA	NA
ZNF280B	NA	NA	0.155185	0.160206	-0.18942	-0.17437	NA	NA
ZNF280C	NA	NA	-0.14518	-0.20453	0.140029	0.096678	NA	NA
ZNF280D	NA	NA	-0.08554	-0.01686	-0.24995	0.304456	NA	NA
ZNF281	-0.14468	-0.17858	0.021402	-0.11456	-0.16367	0.248737	NA	NA
ZNF282	0.466803	0.612764	0.313227	-0.00928	0.416452	0.161101	NA	NA
ZNF283	NA	NA	-0.29218	-0.19595	-0.37699	-0.02933	NA	NA
ZNF284	NA	NA	-0.32538	-0.03749	-0.08348	-0.34445	-0.12093	-0.14745
ZNF285A	0.505819	-0.10409	0.149452	-0.14291	-0.05339	0.029127	0.056347	0.019789
ZNF286A	-0.23691	-0.15395	-0.1435	0.025543	-0.22079	-0.2579	NA	NA
ZNF287	-0.15765	-0.3113	-0.14059	-0.22629	-0.3672	-0.13215	0.09883	-0.06202
ZNF292	NA	NA	-0.09145	-0.02538	0.113762	-0.01157	NA	NA
ZNF295	-0.01242	-0.45405	-0.1858	-0.28056	0.066318	0.103915	0.326486	0.272154
ZNF296	NA	NA	0.129802	0.137761	0.119226	0.198033	NA	NA
ZNF3	0.276842	0.433506	0.115208	-0.13752	0.314533	0.175149	0.285069	0.193123

ZNF30	0.192396	-0.53533	-0.26486	0.052599	-0.2347	-0.29544	0.108687	0.373042
ZNF300	0.007141	-0.24131	-0.37194	0.198953	0.024233	0.287194	-0.19125	0.427055
ZNF302	-0.14745	-0.64274	-0.17333	-0.008	-0.18178	-0.0886	0.040144	0.185028
ZNF304	-0.25749	0.242995	-0.01533	0.091224	-0.2337	0.438182	-0.36126	0.048181
ZNF311	0.318595	-0.18241	-0.15842	0.010706	-0.10503	-0.29507	0.142148	0.222347
ZNF317	0.376189	0.808361	-0.18731	0.223431	0.072813	-0.02702	-0.35201	-0.27145
ZNF318	0.362689	0.040293	-0.3058	0.228099	0.216773	-0.09946	-0.0296	-0.08323
ZNF319	0.107515	-0.11588	-0.32697	0.161961	0.104238	-0.16856	-0.11642	-0.38203
ZNF32	0.119678	-0.20251	-0.17016	-0.10308	-0.00262	-0.19347	-0.4499	-0.44117
ZNF320	0.001295	-0.27191	-0.02191	-0.02035	0.029668	-0.3172	-0.54189	-0.48709
ZNF322A	-0.25524	-0.5601	-0.27546	-0.27823	-0.08897	-0.08467	-0.40547	-0.23882
ZNF322B	0.081154	0.577795	-0.01819	-0.49517	-0.02947	-0.01228	-0.32482	-0.36748
ZNF323	0.158302	-0.0996	-0.30187	-0.45473	-0.03291	-0.15628	-0.22561	-0.21492
ZNF324	0.119121	0.4377	0.240982	-0.07735	0.484228	0.271421	0.334811	0.009534
ZNF326	-0.13157	0.167409	-0.25045	-0.363	0.266785	0.062261	0.031728	0.059055
ZNF329	0.05685	-0.43294	-0.11968	-0.21414	-0.25116	-0.14157	-0.10811	0.116735
ZNF330	-0.3576	-0.5225	0.0694	0.038446	-0.05097	-0.21351	-0.21105	-0.40578
ZNF331	0.027361	-0.1724	0.029204	0.02359	-0.41011	0.244456	-0.34187	-0.13627
ZNF333	-0.09865	0.078367	-0.12269	-0.12285	0.018436	0.103906	-0.03095	0.136024
ZNF334	0.324602	-0.01673	0.028772	-0.08751	0.072749	0.319214	0.276781	0.320908
ZNF335	0.328206	-0.2837	0.181958	0.090304	0.306585	0.50137	-0.11139	0.180655
ZNF337	0.0966	0.003696	-0.08363	0.027477	0.131479	0.28427	0.296012	0.403894
ZNF33A	-0.20875	-0.46038	-0.19498	-0.06223	-0.28133	-0.1868	-0.32704	-0.29991
ZNF33B	-0.23894	-0.49406	-0.31667	-0.12786	0.159626	-0.36025	NA	NA
ZNF34	0.155417	0.304702	0.212569	-0.17544	0.052545	0.273991	0.392985	0.309236
ZNF341	0.327626	0.56834	0.179292	0.17531	0.226192	-0.2318	0.565414	0.297283
ZNF343	0.329679	0.045148	-0.14097	-0.15897	-0.07557	0.22587	-0.25067	-0.45055
ZNF345	0.273817	0.652071	-0.09121	-0.17455	-0.08016	-0.05605	-0.60823	-0.29112
ZNF346	0.07771	-0.28888	0.2826	-0.06016	0.387992	0.331964	0.0539	0.070703
ZNF347	0.208115	0.285434	-0.09123	-0.24214	0.18725	0.072638	-0.23847	0.314448
ZNF35	0.448796	0.309409	-0.18821	-0.14973	-0.27468	-0.19295	0.399422	0.26766
ZNF350	0.009395	-0.22586	-0.34061	-0.16954	-0.04486	-0.12924	0.174688	0.390685
ZNF354A	0.542473	0.10263	-0.14143	-0.14871	-0.17467	-0.15429	-0.44021	-0.03553
ZNF354B	0.279634	-0.02276	-0.03507	-0.2383	0.075092	-0.07525	0.182843	-0.00562
ZNF354C	-0.10516	0.191632	-0.31863	-0.10136	-0.00835	-0.25271	-0.00138	0.040049
ZNF358	0.483277	0.322812	0.39465	-0.11023	0.23143	-0.04697	-0.2768	-0.63504
ZNF362	NA	NA	-0.0466	0.176769	-0.02802	0.296988	NA	NA
ZNF365	0.148266	0.305046	0.046287	-0.19511	-0.18526	0.203882	-0.05357	0.194899
ZNF366	-0.2345	-0.39961	0.355122	0.265673	0.246611	0.532566	-0.40264	-0.0716
ZNF367	0.437546	0.44996	-0.28189	0.158391	0.025576	-0.08199	0.28295	0.479291
ZNF37A	0.163012	0.500019	0.15901	0.056212	-0.14178	-0.00665	-0.0751	-0.01867
ZNF382	0.202474	0.237625	-0.12596	0.04878	-0.36696	0.433481	-0.02888	-0.32102
ZNF383	0.051486	-0.09669	-0.16859	-0.26535	-0.47707	-0.11691	0.158477	0.189939
ZNF384	0.552432	0.250731	0.013879	0.160022	0.252777	0.382327	-0.29846	-0.067
ZNF385A	NA	NA	0.321714	0.13131	-0.07195	0.202825	NA	NA
ZNF385B	NA	NA	0.270677	-0.12742	-0.08306	-0.02792	NA	NA
ZNF385D	NA	NA	0.125566	-0.01123	-0.122	-0.19728	NA	NA
ZNF394	-0.27884	-0.44289	0.210348	-0.26918	0.322888	0.197991	-0.45908	-0.3187
ZNF395	-0.10562	-0.29032	0.170558	0.237976	0.251243	0.308587	0.539736	0.079348
ZNF396	0.179494	0.337202	0.004053	-0.30226	-0.37872	-0.46369	-0.56634	-0.54549
ZNF397	-0.18551	-0.56837	0.093752	0.181018	-0.03752	0.140063	-0.24099	-0.05908
ZNF398	-0.13487	0.457384	0.111729	-0.34089	0.154086	0.25441	-0.00543	-0.1086

ZNF404	-0.03823	-0.52485	0.184553	-0.15005	-0.21032	0.316051	-0.22961	-0.10667
ZNF407	-0.25724	-0.63058	-0.01536	-0.27072	0.070173	0.147043	0.527631	0.356079
ZNF408	0.34989	0.655022	0.244845	-0.07793	0.305223	-0.0686	0.552916	0.431104
ZNF41	0.202875	0.471257	-0.51885	0.041325	-0.02659	-0.41328	0.128439	0.509249
ZNF410	-0.17902	-0.44758	-0.094	-0.0824	-0.1649	-0.14705	-0.12456	0.105982
ZNF414	0.010861	0.520717	0.16311	0.103364	0.177719	0.067482	0.285436	0.078252
ZNF415	0.119952	-0.33814	-0.2637	-0.37625	-0.15442	-0.36016	-0.12939	-0.42307
ZNF416	0.287523	0.278076	-0.27195	-0.13738	-0.40847	-0.05635	0.447917	0.322165
ZNF417	-0.0841	0.178809	0.089746	0.074973	-0.25004	-0.01158	0.559117	0.33367
ZNF418	-0.24764	-0.0628	0.046809	0.091824	-0.06752	0.16018	-0.36472	0.200994
ZNF419	0.156863	0.075463	-0.20349	0.028226	-0.04952	-0.08023	0.012481	0.377017
ZNF420	0.237225	-0.55742	-0.03922	-0.25145	-0.11081	0.011037	0.062932	-0.21075
ZNF423	0.483425	-0.20055	0.044886	-0.00298	0.059994	-0.12066	0.144934	0.108959
ZNF425	-0.07375	-0.33898	-0.05942	-0.18976	-0.21544	-0.17391	-0.28	-0.34589
ZNF426	0.172113	-0.0695	-0.04777	-0.2253	0.041775	0.118298	-0.07662	0.154785
ZNF428	-0.35217	-0.10824	0.02774	0.253034	-0.09182	0.147651	NA	NA
ZNF429	0.220296	-0.15996	-0.28734	0.012997	-0.15546	0.101689	-0.42676	-0.1416
ZNF43	0.15469	-0.14975	-0.10932	0.11022	-0.2671	-0.0778	-0.50325	0.010206
ZNF430	-0.48103	-0.40638	-0.34018	0.160349	-0.13587	0.086519	-0.56591	-0.63733
ZNF431	0.462915	-0.46882	-0.52567	0.089594	-0.27631	0.134976	-0.29275	-0.22734
ZNF432	-0.19198	0.53838	-0.05318	-0.32812	-0.05022	-0.20183	0.296464	-0.06158
ZNF433	0.299944	0.134476	0.108072	-0.11014	-0.06223	-0.44444	-0.15072	-0.19252
ZNF434	0.181601	0.138334	-0.34329	-0.17072	0.044332	0.014118	0.347051	0.152538
ZNF436	-0.15354	0.468373	-0.23423	-0.0268	-0.14893	-0.0339	-0.28834	-0.19552
ZNF438	-0.39158	0.114772	-0.14344	-0.23972	-0.26882	-0.06399	-0.42289	-0.02556
ZNF439	0.178557	-0.25106	0.005386	-0.00219	0.157825	0.038159	-0.20199	0.069948
ZNF44	0.063411	0.36739	-0.20661	-0.16395	0.271465	-0.17495	-0.14686	-0.04529
ZNF440	-0.24275	-0.3283	-0.26272	-0.11895	0.038083	-0.11173	NA	NA
ZNF441	0.261942	0.684594	-0.15637	-0.091	-0.02216	-0.20646	-0.18054	-0.00566
ZNF442	0.435533	0.150852	-0.28138	-0.12878	0.037977	-0.43352	0.134907	0.240975
ZNF443	-0.1493	-0.20261	-0.08033	-0.23449	-0.01411	-0.35832	0.390217	0.370859
ZNF444	0.319626	0.150761	-0.09989	0.126693	0.084818	0.00939	0.354158	-0.09067
ZNF445	-0.23355	-0.23342	-0.16031	0.125302	-0.06083	0.082256	-0.32155	-0.14479
ZNF446	0.101354	0.539724	0.068907	-0.17837	0.118631	-0.12138	0.473314	0.397001
ZNF449	-0.02922	-0.10623	-0.11477	-0.31671	-0.062	-0.53424	0.108475	0.318398
ZNF45	-0.13737	-0.67051	-0.43551	-0.01587	-0.22014	-0.16458	0.303504	0.225769
ZNF451	-0.12108	-0.56614	0.0201	-0.09537	0.041895	0.295083	-0.12767	-0.32617
ZNF454	-0.31489	-0.27749	0.040067	0.080136	0.077218	0.014631	-0.49825	-0.34088
ZNF460	0.114282	-0.08477	NA	NA	NA	NA	-0.5024	-0.44083
ZNF461	NA	NA	-0.10827	-0.29281	-0.21785	-0.28939	NA	NA
ZNF462	0.505115	0.042242	0.082822	0.050886	0.308838	0.210023	0.251053	-0.10545
ZNF467	-0.39084	0.076265	0.157116	-0.06228	0.047239	0.10906	-0.64677	-0.31576
ZNF468	0.320639	0.539778	-0.66572	-0.0435	-0.02643	-0.09618	-0.24289	-0.13798
ZNF469	NA	NA	-0.10997	0.212226	-0.12216	0.391256	NA	NA
ZNF470	0.451401	0.289155	NA	NA	NA	NA	NA	NA
ZNF471	0.210588	0.255545	-0.18358	-0.17638	-0.42001	-0.03121	-0.33136	0.038581
ZNF473	0.28464	-0.00564	-0.27152	-0.3068	0.089819	0.030674	0.564045	0.373597
ZNF474	-0.3754	-0.41668	NA	NA	NA	NA	0.322143	0.189495
ZNF479	NA	NA	-0.14593	-0.0741	-0.14444	0.141692	NA	NA
ZNF48	NA	NA	0.039725	0.150555	0.4625	0.005541	NA	NA
ZNF480	0.413705	0.363024	-0.28108	-0.0287	-0.34595	-0.06375	-0.02994	0.102569
ZNF483	-0.11505	-0.05643	-0.04526	0.016887	0.042124	0.194801	-0.52788	-0.57326

ZNF484	-0.05865	-0.45722	-0.19921	-0.35664	-0.07432	-0.27086	0.176411	0.389366
ZNF485	0.230724	-0.55257	-0.37493	0.052429	-0.06317	0.184003	-0.34684	-0.63996
ZNF486	NA	NA	-0.2421	-0.02608	-0.20787	-0.11683	NA	NA
ZNF488	-0.08164	-0.07659	0.009796	0.339031	-0.08496	-0.33189	NA	NA
ZNF490	0.290235	0.052721	0.165153	0.18966	0.289132	-0.08813	-0.4701	-0.36434
ZNF491	-0.22236	0.389401	0.236613	-0.06321	0.13686	-0.13592	0.318762	0.279265
ZNF493	0.20145	-0.25836	-0.19359	-0.17441	-0.13032	-0.07035	-0.21542	-0.43849
ZNF496	0.226474	0.336814	0.251049	-0.22309	0.198257	0.009237	0.165629	0.007617
ZNF497	0.063918	0.794053	0.138421	-0.037	0.196174	-0.09325	-0.00947	-0.25097
ZNF498	0.34875	0.628477	-0.26117	-0.03956	0.260986	-0.16028	-0.26385	0.165817
ZNF500	0.440719	-0.15772	-0.12244	-0.14101	0.185979	0.157616	0.273627	0.067934
ZNF501	0.033773	-0.40973	-0.02036	-0.12688	-0.18023	-0.21271	-0.10336	-0.31582
ZNF502	0.077907	-0.50341	0.02241	-0.23401	0.095387	0.043128	0.189534	0.265204
ZNF503	0.322122	0.057305	-0.01287	0.072601	0.401199	0.133026	0.161169	-0.07217
ZNF506	NA	NA	-0.14849	-0.12342	0.129303	0.070444	NA	NA
ZNF507	0.469418	-0.45629	-0.45964	-0.32475	-0.01342	-0.11397	0.214285	0.373282
ZNF509	-0.03376	0.76562	-0.20775	0.185803	-0.06973	-0.02287	0.471778	0.570862
ZNF510	0.252288	0.693416	-0.32061	0.106899	0.05708	-0.06694	0.136874	0.060044
ZNF511	-0.27725	0.371158	-0.04798	0.173147	0.166379	-0.19084	-0.3564	-0.42872
ZNF512	0.02471	-0.1579	-0.01888	-0.03146	-0.24521	0.326591	0.38038	0.41505
ZNF512B	NA	NA	0.086906	0.174038	-0.20163	0.500823	NA	NA
ZNF513	0.202817	0.460706	0.253817	-0.03867	0.184227	0.183942	-0.17704	0.030813
ZNF514	0.291988	0.044676	0.54768	-0.07501	0.372132	0.406883	0.060189	0.003097
ZNF516	NA	NA	0.051458	-0.31286	-0.16621	-0.29096	NA	NA
ZNF517	-0.07059	-0.11617	0.137006	-0.07941	0.150715	-0.11027	-0.01533	-0.06317
ZNF518A	NA	NA	-0.12135	-0.33176	0.176591	-0.20407	NA	NA
ZNF518B	NA	NA	-0.29548	-0.48366	-0.20442	0.164306	NA	NA
ZNF519	0.335001	-0.23109	-0.28655	0.000654	-0.27829	-0.20685	-0.16128	-0.0617
ZNF521	-0.15014	-0.29096	-0.07942	-0.02197	-0.20146	-0.22562	-0.47602	-0.09313
ZNF524	0.320348	0.690527	0.137693	0.017157	0.207135	-0.2987	0.359013	-0.04601
ZNF526	-0.09898	0.062595	0.224486	0.19694	0.129451	0.201226	-0.31856	-0.40189
ZNF527	NA	NA	-0.22197	-0.30887	-0.2887	0.232544	NA	NA
ZNF528	-0.02196	0.461445	-0.14978	-0.09174	-0.37255	-0.2895	0.313239	0.255847
ZNF529	0.50439	0.07277	-0.16921	-0.09513	-0.19564	0.30026	-0.04334	0.31349
ZNF530	-0.00714	-0.06853	-0.09808	0.054959	-0.10239	0.499973	0.065137	-0.02327
ZNF532	-0.07824	-0.13889	-0.32361	-0.36676	-0.31589	0.011187	-0.47515	-0.39963
ZNF536	0.397489	0.371235	-0.08158	0.214041	0.010914	0.314103	-0.24979	-0.21268
ZNF540	0.208987	0.297292	0.152835	-0.07772	-0.41181	0.358739	-0.31551	0.241982
ZNF541	-0.01057	-0.03863	0.231963	-0.10217	0.182261	-0.15039	NA	NA
ZNF542	-0.01078	0.334187	0.016879	-0.13185	-0.11273	0.142695	-0.37509	-0.66324
ZNF543	0.070216	-0.26982	0.103768	-0.37647	0.286328	0.302498	-0.23413	-0.0499
ZNF544	-0.10027	0.396868	-0.32487	-0.08376	-0.26509	0.138459	-0.36554	-0.3355
ZNF546	0.099321	0.222373	0.409874	-0.29779	0.059352	0.443752	-0.4539	0.273683
ZNF547	0.060694	0.349126	-0.01189	0.063062	-0.09774	-0.0812	0.103065	-0.06992
ZNF548	-0.00998	-0.62287	-0.02802	0.129211	-0.08113	0.18898	0.277002	0.46204
ZNF549	-0.08679	-0.38828	-0.03796	0.177737	-0.25203	0.067236	-0.37814	-0.03465
ZNF550	0.110659	-0.29825	-0.20859	-0.03952	0.142976	0.397408	-0.01409	0.074629
ZNF551	-0.08386	-0.44145	-0.38784	-0.29841	-0.03008	0.406771	0.349328	0.499432
ZNF552	0.260716	0.36291	-0.07397	-0.06688	-0.35129	-0.1885	0.35159	0.213293
ZNF554	0.097386	0.547166	-0.59023	-0.053	0.111062	0.011125	0.431329	-0.02647
ZNF555	-0.02819	0.14049	-0.15683	-0.27652	-0.01413	-0.43721	-0.21284	-0.38189
ZNF556	-0.01727	0.469114	-0.15265	0.019281	-0.22276	-0.4244	-0.2214	-0.22708

ZNF557	0.242427	0.738026	-0.2572	-0.19902	-0.14571	-0.20693	-0.61228	-0.59976
ZNF558	0.393321	-0.18546	-0.15129	-0.03751	0.123411	0.037849	-0.05266	0.210071
ZNF559	0.189033	-0.317	-0.0691	0.196336	-0.02433	-0.05334	-0.23117	-0.30402
ZNF560	0.172602	0.578642	-0.13948	-0.06474	-0.00219	-0.1668	0.466244	0.212669
ZNF561	0.260463	0.05417	-0.30262	-0.29742	-0.22211	-0.29355	-0.50258	-0.12682
ZNF562	0.130009	0.736336	-0.48649	-0.17496	-0.19193	0.10093	-0.37135	-0.39496
ZNF563	0.267666	-0.2529	0.240891	-0.08717	0.376874	-0.14123	-0.49378	-0.33798
ZNF564	0.641689	-0.36632	-0.01016	-0.16832	-0.01362	-0.1782	0.60892	0.518459
ZNF565	0.221084	0.518992	-0.11464	-0.5136	-0.11072	0.07605	0.00948	-0.31127
ZNF566	0.357351	-0.33302	-0.18704	-0.051	0.129975	0.284004	0.453519	0.358026
ZNF567	0.091445	-0.14131	-0.13429	-0.5124	-0.30412	-0.02932	-0.27872	-0.38293
ZNF568	-0.1606	0.101797	-0.14164	-0.29939	-0.37439	-0.27741	-0.29668	-0.07332
ZNF569	0.095547	0.088853	-0.21257	0.157258	0.00763	-0.0432	-0.00018	-0.06793
ZNF57	0.171067	-0.30771	-0.27429	0.00484	0.090491	-0.36334	NA	NA
ZNF570	0.24902	-0.32051	-0.08303	-0.16203	-0.21656	-0.40778	-0.01048	-0.40616
ZNF571	0.299383	0.678767	-0.20708	-0.44571	-0.20072	-0.11088	0.390513	0.361512
ZNF572	0.336953	-0.38115	0.058135	-0.26261	-0.03429	0.021945	0.495246	0.256242
ZNF573	0.555937	0.057261	-0.12082	0.056428	0.089267	0.320828	0.028944	-0.19307
ZNF574	0.405198	0.390879	0.149589	0.101904	0.215095	0.110381	0.694197	0.278395
ZNF575	-0.0934	0.507846	-0.11438	-0.15097	-0.15736	0.196037	0.345872	0.200012
ZNF576	0.1986	0.541602	0.135142	0.022848	-0.06859	-0.22127	0.14226	-0.09423
ZNF577	0.171312	-0.42614	0.037501	-0.145	-0.08274	-0.05902	-0.47973	-0.47358
ZNF578	0.423735	0.033277	-0.4797	-0.12571	-0.1373	-0.04878	NA	NA
ZNF579	0.468966	0.418154	0.065435	-0.00747	-0.04607	0.233664	0.3417	-0.27029
ZNF580	0.028432	0.318638	-0.06712	0.265038	0.057095	0.260025	0.256676	0.092252
ZNF581	0.511302	0.468193	0.253751	0.090044	0.17175	0.206137	0.617651	0.047119
ZNF582	-0.33444	-0.36364	-0.0451	-0.11434	-0.09791	-0.4025	-0.50461	-0.21887
ZNF583	-0.0008	0.444357	-0.06672	-0.06466	-0.32247	0.233643	NA	NA
ZNF584	0.117767	-0.46797	0.276881	0.115413	0.089183	0.149958	0.302298	0.236573
ZNF585A	0.23823	-0.02843	-0.30454	-0.19681	-0.05643	-0.1174	0.319853	0.151192
ZNF585B	0.106106	-0.62307	-0.14622	-0.14175	-0.11	-0.20829	-0.26148	-0.36245
ZNF586	-0.09965	0.717088	-0.0266	0.088344	-0.26904	0.051502	0.256414	0.44555
ZNF587	0.086777	0.520905	-0.20837	-0.0375	0.003689	0.11103	0.574379	0.325614
ZNF589	0.060389	0.529142	0.250911	0.307357	0.384511	0.564014	0.328452	0.602145
ZNF592	-0.30291	-0.23295	-0.03	0.057047	0.166231	0.173183	-0.39255	-0.40204
ZNF593	0.181378	0.710379	0.180379	0.196231	0.114149	-0.00872	-0.03797	-0.19701
ZNF594	NA	NA	-0.38521	-0.13227	-0.17835	0.11071	NA	NA
ZNF595	0.106119	-0.41668	-0.47508	-0.06845	-0.28905	-0.21003	-0.30662	-0.1207
ZNF596	0.04488	0.197838	-0.3147	-0.09357	-0.2237	-0.3868	-0.03817	-0.13563
ZNF597	0.226158	-0.09408	-0.25177	-0.13049	-0.14079	-0.04365	0.349014	0.505328
ZNF598	0.311477	0.044125	0.040963	-0.03454	0.516873	0.277077	0.155408	0.094045
ZNF599	0.067525	-0.01624	0.066477	-0.09643	-0.00229	-0.18135	0.367978	0.372068
ZNF600	-0.16303	0.291171	-0.48221	-0.24736	-0.01879	0.047484	-0.63414	-0.59126
ZNF605	0.045863	-0.56843	0.128342	-0.2788	-0.01998	0.044832	NA	NA
ZNF606	-0.02566	-0.28476	-0.35791	-0.10358	-0.23835	-0.11408	0.077693	-0.09249
ZNF607	0.593314	0.609396	0.19716	-0.04729	0.111425	-0.08725	0.261035	-0.00427
ZNF608	0.430119	-0.38397	0.142075	-0.17421	-0.11903	0.03805	0.140903	-0.07431
ZNF609	0.252463	0.213829	0.095371	0.077352	0.094605	0.185814	0.318746	0.364871
ZNF610	0.243303	-0.28073	-0.348	-0.13756	-0.15816	-0.23259	0.049817	-0.18483
ZNF611	-0.26698	-0.0797	-0.15545	-0.09393	0.193086	0.304795	-0.26083	0.065221
ZNF613	0.046519	0.326746	-0.40283	-0.32034	-0.25361	-0.09946	0.082684	-0.0266
ZNF614	0.338897	-0.20359	-0.49233	-0.39168	-0.38189	-0.31454	0.399268	0.197772

ZNF615	0.141497	0.031651	-0.22628	-0.39541	-0.15573	-0.39743	0.162148	0.025617
ZNF616	0.275845	-0.44201	-0.2371	-0.23296	-0.10554	-0.39811	0.68513	0.47364
ZNF618	-0.06898	0.05089	-0.25777	0.053623	-0.27703	0.066825	-0.07181	-0.10949
ZNF619	-0.30306	0.061528	-0.18461	-0.1807	-0.14882	-0.30938	0.158191	0.158987
ZNF620	0.173539	0.128425	-0.02197	-0.02816	0.113795	0.172909	-0.35033	-0.34744
ZNF621	0.154342	0.2128	0.015682	-0.08788	-0.23696	-0.16271	-0.20288	0.270527
ZNF622	0.235505	0.171613	0.220669	0.118826	0.341175	-0.24029	0.075517	-0.39386
ZNF623	-0.02912	-0.53832	-0.01732	0.183945	0.038735	0.135824	-0.20268	-0.3616
ZNF624	0.266883	0.703529	-0.15325	-0.07436	-0.26209	-0.22486	-0.31554	0.021663
ZNF625	-0.26231	0.508284	-0.23729	-0.08044	-0.18993	-0.24006	0.372975	0.52584
ZNF626	0.046641	-0.46975	-0.23205	0.06281	-0.31843	-0.15458	-0.64143	-0.40111
ZNF627	0.059873	-0.48674	-0.29096	-0.25713	-0.12425	-0.42125	0.003407	-0.10076
ZNF628	0.194171	0.490645	0.011414	0.062778	0.207632	0.115618	0.298841	0.049159
ZNF629	NA	NA	0.287602	0.125461	0.509326	0.559521	0.279722	-0.10809
ZNF630	NA	NA	-0.24444	-0.15051	-0.26466	-0.40772	0.090592	0.261993
ZNF638	-0.13127	-0.43712	-0.20553	-0.03375	-0.06003	0.012968	NA	NA
ZNF639	0.084894	-0.14568	-0.15586	-0.21956	-0.13733	0.084773	0.461816	0.432405
ZNF641	0.221003	0.163528	-0.05651	-0.08739	0.041824	0.131676	-0.39281	0.066528
ZNF642	0.485965	-0.29101	0.06709	-0.04933	0.089295	-0.30498	-0.02388	-0.11129
ZNF643	0.047305	0.108379	-0.18416	0.09747	0.153306	-0.1594	-0.0047	0.054366
ZNF644	-0.19451	-0.63405	0.109223	-0.214	0.165544	0.085991	0.074705	-0.29663
ZNF645	0.441496	0.194358	-0.27595	-0.12008	0.092045	-0.30339	0.030834	-0.04408
ZNF646	0.025092	0.508283	0.25822	0.241293	0.277733	0.116794	0.296491	0.11247
ZNF648	-0.10558	0.155346	NA	NA	NA	NA	-0.31213	-0.15328
ZNF649	0.094891	-0.42821	-0.39469	-0.32591	-0.0044	0.01789	0.276199	0.067559
ZNF652	-0.0434	-0.32343	0.22324	-0.12512	0.370037	0.140567	-0.48711	-0.52827
ZNF653	0.000771	0.617969	-0.11533	0.019987	0.189812	0.341051	-0.27678	0.042898
ZNF654	-0.45332	0.491573	-0.24617	-0.26775	-0.30821	-0.19799	-0.38185	-0.22553
ZNF655	0.102229	0.133578	0.236439	-0.27096	-0.0795	0.089036	-0.26999	-0.12544
ZNF658	-0.15861	-0.22657	NA	NA	NA	NA	-0.54848	-0.4403
ZNF658B	NA	NA	-0.03049	-0.44251	-0.19006	-0.19835	0.363039	0.185858
ZNF660	-0.22146	0.121064	0.256106	0.029072	-0.27453	0.215333	0.151426	0.242455
ZNF662	0.306656	-0.37562	NA	NA	NA	NA	0.026773	0.077983
ZNF663	0.252339	0.710241	0.421201	-0.09147	0.116537	0.115825	NA	NA
ZNF664	-0.00578	-0.58892	-0.14742	-0.00209	0.19793	0.148636	0.283342	0.259641
ZNF665	0.109028	0.554669	-0.44811	0.132758	-0.07818	0.078492	-0.19015	-0.22373
ZNF667	-0.11352	-0.09895	0.037522	0.10417	0.198814	0.269609	-0.61854	-0.1948
ZNF668	-0.03601	0.428175	0.306527	0.007186	0.165147	-0.01857	-0.10663	0.069617
ZNF669	-0.22062	-0.27117	-0.03655	-0.08983	0.033117	-0.15031	-0.19431	-0.43143
ZNF670	-0.1041	0.47005	-0.29879	-0.27261	-0.14923	-0.29466	0.329845	0.133232
ZNF671	-0.58036	0.033352	-0.01762	0.313529	-0.17707	0.193809	-0.46138	0.094987
ZNF672	-0.17002	0.803434	0.027069	0.195031	0.014932	-0.06248	0.033946	0.170633
ZNF673	0.405514	-0.07159	-0.32544	-0.21983	0.02148	0.077788	-0.35511	-0.37378
ZNF674	NA	NA	0.05546	-0.26009	-0.23165	-0.16828	NA	NA
ZNF675	-0.12565	0.160635	-0.33517	0.104527	-0.27892	-0.07891	0.401254	0.338898
ZNF676	0.325079	0.284772	-0.06618	0.0078	-0.3427	0.077323	NA	NA
ZNF677	-0.27344	-0.27086	NA	NA	NA	NA	-0.11822	-0.30428
ZNF678	0.095815	0.809636	-0.41218	0.053983	-0.3605	0.03085	-0.36753	-0.40664
ZNF679	0.053107	0.183757	NA	NA	NA	NA	NA	NA
ZNF680	0.346453	-0.3236	-0.33477	-0.01485	-0.28974	-0.1819	-0.32731	-0.42697
ZNF681	0.038034	0.155779	-0.40912	-0.01809	-0.02803	-0.07315	-0.25636	-0.40012
ZNF682	0.010427	0.603003	0.065917	-0.08336	0.005092	-0.42725	-0.3959	-0.46855

ZNF683	-0.04871	0.030918	0.078451	-0.20678	-0.19176	-0.10149	-0.40003	0.663248
ZNF684	0.336225	-0.2859	-0.22486	-0.33034	-0.34527	-0.46934	-0.01043	-0.19779
ZNF687	0.265414	-0.00509	0.252942	0.011115	0.1511	0.035184	0.23382	-0.10817
ZNF688	0.00103	0.654805	0.079593	0.135	-0.09278	-0.23193	0.070799	0.205105
ZNF689	0.111643	0.203299	0.215676	0.347774	0.145996	-0.07462	-0.29347	-0.20594
ZNF69	0.057309	0.410475	0.015639	-0.05	0.132674	0.01342	-0.51515	-0.44984
ZNF691	0.197165	0.553685	0.51548	0.209992	0.179906	-0.0232	0.425481	0.124645
ZNF692	-0.16362	0.402461	-0.00417	0.293581	0.044034	0.206558	0.58495	0.551975
ZNF695	0.524173	0.437067	-0.01315	0.086475	-0.12708	-0.20014	0.291234	0.012016
ZNF696	-0.09067	0.124559	0.322693	-0.0816	0.108078	0.41805	0.410362	-0.01588
ZNF697	NA	NA	0.166988	0.07617	0.338051	-0.02501	NA	NA
ZNF699	NA	NA	NA	NA	NA	NA	-0.30903	-0.26798
ZNF7	0.133637	-0.15041	0.084624	-0.48853	-0.09762	0.073625	0.543717	0.464945
ZNF70	0.01628	-0.36839	0.041127	0.123146	0.088893	0.075791	-0.63686	-0.28253
ZNF700	-0.12928	-0.55058	-0.1524	-0.13317	-0.01453	0.015445	-0.01547	0.389525
ZNF701	0.035196	-0.60951	-0.42171	-0.27289	0.060515	-0.49476	-0.02375	-0.01979
ZNF703	-0.0102	-0.54189	0.177737	-0.08115	0.387364	0.023872	0.167773	-0.48277
ZNF704	0.105009	0.126677	0.318187	-0.16624	-0.15833	0.095408	0.270526	0.095546
ZNF705A	-0.10217	0.608144	NA	NA	NA	NA	NA	NA
ZNF706	0.343843	0.44364	0.172563	-0.38555	0.118577	-0.3731	0.132614	-0.03829
ZNF707	0.039191	-0.37992	-0.14603	0.188678	-0.02496	-0.04602	0.592428	0.660906
ZNF708	0.067926	-0.32412	-0.28181	0.061879	-0.19888	-0.10622	0.110184	-0.0889
ZNF709	0.001601	0.416451	-0.14788	-0.18754	0.011341	-0.16631	0.014648	-0.01542
ZNF71	0.397995	0.534738	0.066784	0.009426	-0.02892	0.039843	-0.30753	-0.22187
ZNF710	0.25912	0.37982	-0.25623	-0.11547	0.16094	0.34593	0.473324	0.361916
ZNF711	-0.03828	-0.63876	-0.298	-0.1017	-0.28593	0.250462	NA	NA
ZNF713	0.329585	0.795058	0.016662	0.16011	0.003389	-0.17373	0.345135	-0.19934
ZNF714	0.212679	-0.06619	-0.35866	0.052357	-0.28423	-0.01638	-0.4544	-0.34416
ZNF717	NA	NA	0.095144	-0.24941	-0.22747	-0.22207	NA	NA
ZNF718	NA	NA	-0.09543	0.519369	-0.07384	-0.40592	0.605423	0.457909
ZNF720	-0.04004	-0.12893	-0.24231	-0.0879	0.031202	-0.39506	NA	NA
ZNF721	-0.19273	-0.23799	-0.3859	0.186349	-0.0982	0.081201	-0.27894	-0.26639
ZNF737	NA	NA	-0.24123	-0.0615	-0.31267	0.046439	NA	NA
ZNF738	NA	NA	-0.36576	0.148212	-0.23143	-0.08867	NA	NA
ZNF74	0.193114	-0.27014	0.100284	0.21226	0.370292	0.323246	0.11289	-0.53114
ZNF740	0.127953	0.364184	-0.39961	0.103095	0.225886	-0.43171	0.000414	0.321631
ZNF746	0.318428	0.389597	-0.10937	0.009176	0.286006	0.393659	NA	NA
ZNF747	-0.07463	0.656146	0.065563	-0.01092	0.170323	-0.16198	NA	NA
ZNF749	0.498024	0.638487	NA	NA	NA	NA	0.140208	-0.18742
ZNF750	0.012717	0.464828	-0.11749	-0.1271	-0.04907	0.205782	NA	NA
ZNF75A	-0.02093	-0.05859	-0.22419	-0.02086	-0.1462	0.0705	-0.52557	-0.19548
ZNF75D	NA	NA	-0.28845	0.008582	0.0965	0.281404	NA	NA
ZNF76	0.32465	0.480504	0.23215	0.059105	0.252892	0.06	0.183754	0.22069
ZNF761	-0.08498	-0.2166	-0.44229	-0.07579	-0.09539	0.016852	NA	NA
ZNF763	0.149978	0.540864	NA	NA	NA	NA	NA	NA
ZNF764	-0.0646	-0.42406	-0.09172	0.160842	0.377549	0.103391	NA	NA
ZNF765	NA	NA	-0.45868	-0.23209	-0.03892	0.225808	0.150963	0.157298
ZNF766	0.103098	-0.02955	-0.04245	-0.27816	-0.02904	0.083221	NA	NA
ZNF767	0.230882	0.002107	0.095565	-0.07921	0.252626	0.33644	-0.12787	0.338754
ZNF768	-0.02136	-0.00596	0.402385	0.099365	0.421426	0.07729	NA	NA
ZNF77	-0.09194	-0.57089	-0.15995	-0.27545	-0.13396	-0.33143	0.434194	0.168369
ZNF770	0.123263	-0.31529	-0.19623	-0.21588	-0.29253	-0.28058	NA	NA

ZNF771	0.107877	0.483829	0.453141	-0.07671	0.284644	-0.11447	NA	NA
ZNF772	-0.00933	-0.3244	-0.0002	-0.29243	-0.3147	0.244078	NA	NA
ZNF773	-0.18011	0.153051	0.129828	-0.07482	0.051562	0.359442	NA	NA
ZNF774	0.1015	0.54632	NA	NA	NA	NA	NA	NA
ZNF775	0.406617	-0.00247	0.029215	-0.12424	-0.12855	0.197765	NA	NA
ZNF776	-0.50553	-0.53818	-0.40444	-0.31012	-0.31628	-0.00511	NA	NA
ZNF777	0.255258	0.352437	-0.13848	0.001213	0.27513	0.173562	NA	NA
ZNF778	NA	NA	-0.24244	-0.23643	-0.00642	-0.26182	NA	NA
ZNF780A	-0.2734	0.253483	-0.47554	-0.20259	-0.23895	-0.31945	NA	NA
ZNF780B	0.213967	0.121977	-0.00491	-0.07652	-0.44625	-0.25439	NA	NA
ZNF781	0.178729	0.163348	-0.17014	0.150531	-0.14219	0.09062	0.079349	0.057525
ZNF782	0.104419	-0.44726	NA	NA	NA	NA	NA	NA
ZNF783	NA	NA	-0.13398	0.053539	0.262851	0.299368	NA	NA
ZNF784	0.274627	0.798622	-0.20769	-0.05316	0.046612	0.057464	NA	NA
ZNF785	0.201865	-0.15247	-0.08917	0.004014	0.314849	0.024323	NA	NA
ZNF786	-0.13873	0.508413	-0.04403	0.239648	0.008307	0.395803	NA	NA
ZNF787	0.223914	0.683353	0.169733	-0.00685	0.363431	-0.02433	NA	NA
ZNF789	-0.01595	0.044155	-0.1708	-0.04903	0.182127	-0.0687	NA	NA
ZNF79	0.114716	0.319765	0.020172	-0.06843	-0.016	-0.00621	-0.58846	-0.37666
ZNF790	0.164546	0.596086	-0.09522	-0.02676	-0.3767	-0.10262	NA	NA
ZNF791	0.297832	-0.36216	-0.16692	-0.21337	0.041599	-0.11309	NA	NA
ZNF792	0.150754	0.124067	0.065385	0.095274	0.147742	0.205372	NA	NA
ZNF793	0.300636	0.639896	NA	NA	NA	NA	NA	NA
ZNF8	0.086393	0.716374	-0.39841	-0.29969	-0.50088	-0.16089	-0.07642	0.43979
ZNF80	-0.10564	0.139656	0.002329	0.404472	-0.32156	0.224341	0.254896	0.576049
ZNF800	0.42671	-0.087	0.064959	-0.15945	0.031131	-0.01985	NA	NA
ZNF804A	0.241293	-0.27152	0.159267	0.030566	0.292325	0.450061	NA	NA
ZNF804B	0.12295	0.152684	0.228526	-0.32418	-0.10334	0.162667	NA	NA
ZNF805	-0.04908	-0.55946	NA	NA	NA	NA	-0.16192	-0.15951
ZNF808	NA	NA	-0.47386	-0.2168	-0.17295	-0.02493	0.105396	0.416438
ZNF81	0.363608	-0.14989	-0.34716	-0.05307	-0.05614	-0.10171	0.31609	0.417908
ZNF812	NA	NA	-0.10987	0.095305	0.176243	-0.03492	NA	NA
ZNF813	0.232675	0.076642	-0.48084	-0.11569	-0.10759	-0.13565	NA	NA
ZNF821	NA	NA	0.103989	-0.13791	-0.02691	-0.31762	NA	NA
ZNF823	NA	NA	-0.36757	-0.18473	-0.05344	-0.45716	NA	NA
ZNF827	NA	NA	0.245867	0.243368	-0.04939	0.109785	NA	NA
ZNF829	NA	NA	-0.10503	0.193116	-0.34229	0.157305	NA	NA
ZNF83	-0.13693	-0.23384	-0.31559	-0.01833	0.132042	-0.2662	-0.26393	0.255588
ZNF830	NA	NA	-0.10422	-0.14652	-0.10879	-0.01077	NA	NA
ZNF831	NA	NA	-0.04568	0.624621	-0.23784	0.674816	NA	NA
ZNF835	NA	NA	0.078859	0.003488	-0.01883	0.157467	NA	NA
ZNF839	NA	NA	-0.15594	0.183549	-0.05954	0.300373	NA	NA
ZNF84	-0.05503	0.326308	-0.16987	-0.11018	0.03862	0.149259	NA	NA
ZNF841	NA	NA	-0.28921	-0.18266	0.067437	-0.25386	NA	NA
ZNF843	NA	NA	-0.00544	-0.10402	-0.11351	0.137579	NA	NA
ZNF844	NA	NA	0.033207	0.152111	0.0699	-0.07659	NA	NA
ZNF845	NA	NA	-0.44772	-0.29206	-0.14038	0.064234	NA	NA
ZNF85	0.120526	-0.39607	-0.37854	0.033349	-0.44467	-0.02109	-0.08326	-0.00488
ZNF853	NA	NA	0.301901	0.051577	0.352524	0.288978	NA	NA
ZNF862	NA	NA	-0.11523	-0.16528	0.185152	0.202513	NA	NA
ZNF90	NA	NA	-0.02252	-0.02201	-0.04592	0.147591	NA	NA
ZNF91	-0.06491	-0.42206	-0.09257	-0.10658	-0.12047	-0.04469	-0.58301	-0.32057

ZNF92	0.232567	0.534521	-0.4712	0.285878	-0.13318	-0.12572	-0.32585	-0.32945
ZNF93	0.178608	0.162657	-0.21567	-0.34615	-0.14628	-0.22851	-0.29947	-0.3265
ZNF99	NA	NA	-0.42869	0.016712	-0.20188	0.062007	-0.03321	-0.21535
ZNFX1	0.13973	-0.26516	0.371869	-0.26034	0.18707	0.134153	-0.02181	0.284692
ZNHIT1	0.407801	-0.09656	0.275724	0.073014	0.051517	-0.1554	0.238701	-0.15864
ZNHIT2	0.20862	0.093314	0.134538	-0.07076	0.087522	-0.12302	-0.08052	-0.09286
ZNHIT3	0.486566	0.478288	-0.14165	-0.12957	-0.10408	-0.13471	0.209783	-0.08229
ZNHIT6	NA	NA	-0.07538	-0.21116	0.137081	0.39455	NA	NA
ZNRD1	0.429193	-0.25322	-0.08263	-0.15095	-0.0135	0.091784	0.167701	0.052715
ZNRF1	0.378791	0.666494	-0.18278	-0.13452	0.02444	-0.28368	0.285915	0.357713
ZNRF2	0.28705	0.055944	-0.24369	-0.14271	-0.26001	-0.3761	0.030053	0.001616
ZNRF3	NA	NA	0.375599	0.0483	0.429909	-0.13902	NA	NA
ZNRF4	-0.44247	0.119032	-0.01174	0.149275	-0.00671	-0.43164	-0.33291	-0.21634
ZP1	0.144206	0.331785	-0.09185	0.159452	-0.17518	-0.66796	0.411174	0.089341
ZP2	0.375037	0.657221	-0.00667	-0.25378	0.077024	-0.40363	0.139563	0.520798
ZP3	0.495781	0.535449	0.05814	-0.18483	0.123042	0.050205	0.022411	-0.51395
ZP4	0.364861	0.138833	0.353688	0.046283	0.174725	0.154424	0.071905	0.349972
ZPBP	0.521185	0.710098	-0.19435	-0.52668	-0.00788	0.070953	-0.21299	0.125377
ZPBP2	0.082219	0.48155	-0.19153	-0.03009	-0.05217	-0.01757	0.179781	-0.14892
ZPLD1	0.229262	0.471874	-0.01497	-0.10345	0.213781	0.122229	0.029561	0.110363
ZRANB1	0.063093	0.185906	-0.50278	-0.01698	0.127312	-0.1793	-0.22616	-0.05421
ZRANB2	-0.09815	-0.11164	-0.04966	-0.31656	0.168392	0.122005	NA	NA
ZRANB3	-0.01944	0.508225	-0.24904	-0.07063	0.022666	0.104316	0.38811	0.104718
ZRSR2	0.080883	0.100041	0.35488	0.26973	0.237351	0.418855	NA	NA
ZSCAN1	-0.09888	0.210385	0.192441	-0.12652	0.201265	0.0487	-0.26732	0.000187
ZSCAN10	0.160772	0.604737	-0.20581	-0.02596	-0.10146	0.151658	NA	NA
ZSCAN12	0.30434	-0.1066	NA	NA	NA	NA	NA	NA
ZSCAN16	0.243058	0.114356	-0.18737	-0.13279	-0.16375	-0.42502	NA	NA
ZSCAN18	-0.01114	-0.33319	0.143859	-0.0619	-0.1708	0.521856	NA	NA
ZSCAN2	0.386871	-0.07016	-0.05479	-0.13145	0.198482	-0.49736	0.28236	-0.13607
ZSCAN20	0.285968	0.09225	-0.30058	-0.26643	-0.12926	-0.30652	NA	NA
ZSCAN21	0.23705	-0.08145	0.11283	-0.13729	0.171898	-0.1859	NA	NA
ZSCAN22	0.173738	0.240317	0.200727	0.001131	0.376069	0.204152	NA	NA
ZSCAN29	0.116094	0.058381	-0.18868	0.06941	0.029186	-0.01886	NA	NA
ZSCAN4	-0.13769	-0.05052	-0.05186	-0.19232	0.179771	0.07134	-0.06705	0.009656
ZSCAN5A	NA	NA	-0.43551	0.242796	0.061342	-0.10838	NA	NA
ZSWIM1	0.385561	0.66303	0.101234	-0.03473	0.125698	-0.22026	0.239511	-0.35119
ZSWIM2	0.258678	0.685252	0.054077	0.017411	0.063912	0.282857	0.255081	0.429335
ZSWIM3	0.056439	0.238372	0.308031	-0.00311	0.384507	-0.205	0.53124	0.379442
ZSWIM4	0.241651	0.712439	NA	NA	NA	NA	-0.43647	-0.38881
ZSWIM5	NA	NA	0.22496	-0.02504	0.494798	0.035705	NA	NA
ZSWIM6	NA	NA	0.048253	-0.31624	-0.09839	-0.13204	NA	NA
ZSWIM7	NA	NA	-0.02819	-0.1626	-0.28158	-0.05418	NA	NA
ZUFSP	NA	NA	-0.388	-0.17143	0.190292	-0.56432	NA	NA
ZW10	0.304653	0.78853	-0.30732	0.07794	0.07292	0.206729	-0.00895	-0.14863
ZWILCH	-0.0963	-0.10535	-0.21205	0.055638	0.184125	-0.20432	0.188935	0.078063
ZWINT	-0.51627	0.296183	-0.39722	0.205268	0.159818	-0.19441	0.059694	-0.05576
ZXDA	0.329885	-0.20145	-0.27742	-0.23555	0.085473	-0.19865	-0.06479	0.152776
ZXDB	0.538534	-0.58913	NA	NA	NA	NA	0.550779	0.314755
ZXDC	-0.00108	-0.34796	0.233558	-0.22195	0.348714	0.293423	0.339945	0.245513
ZYG11A	0.493378	0.545577	-0.40362	-0.08887	-0.01175	-0.52459	0.049035	0.050429
ZYG11B	-0.10836	0.212664	0.219992	-0.66946	0.30272	-0.23382	-0.21325	-0.38011

ZYX	0.266267	0.045904	0.288975	0.079937	0.218134	0.334698	-0.17544	-0.06294
ZZEF1	-0.30782	-0.09967	-0.13899	-0.13551	-0.08247	0.134183	-0.33599	0.064994
ZZZ3	-0.1395	-0.58102	-0.15738	-0.32294	-0.09246	-0.30074	-0.21977	-0.24248

GSE44076_	GSE44076_	GSE50760_	GSE50760_	META NOR	sd normal	META TUM	sd tumor	META ALL
-0.24937	0.247619	-0.26054	0.118584	0.178525	0.10464	-0.05056	0.244212	0.063981
0.195973	0.190823	-0.10211	0.26308	0.004156	0.259482	0.102259	0.146285	0.053207
-0.03297	0.242498	NA	NA	0.079443	0.137566	0.015674	0.25639	0.047558
-0.11024	0.147757	0.249892	-0.12856	-0.06037	0.310503	0.007268	0.164336	-0.02655
-0.19815	-0.11374	-0.52681	-0.32284	-0.12998	0.367442	-0.26156	0.274433	-0.19577
0.105351	0.077283	0.254745	-0.22359	-0.05714	0.229306	-0.02237	0.199459	-0.03976
0.015999	0.00053	NA	NA	-0.17321	0.165299	0.126713	0.085635	-0.02325
-0.07354	0.070813	-0.20944	-0.42525	-0.0105	0.219342	-0.07153	0.246258	-0.04101
-0.05856	-0.08035	0.31138	-0.31134	0.004145	0.186029	0.08599	0.225129	0.045067
-0.16806	0.135808	NA	NA	0.333512	0.174362	0.166258	0.296614	0.249885
0.069655	0.168187	0.433714	-0.13975	0.18738	0.264153	0.234046	0.135213	0.210713
-0.01969	0.20231	-0.09832	0.010274	0.230288	0.15468	0.043178	0.252812	0.136733
0.112625	0.007479	0.277708	-0.08238	0.050326	0.268293	0.029112	0.17937	0.039719
NA	NA	NA	NA	-0.4016	0.124796	-0.247	0.155152	-0.3243
0.012638	-0.1361	-0.38186	-0.10126	-0.16086	0.274813	-0.0574	0.204682	-0.10913
0.054916	-0.05393	NA	NA	0.328956	0.541478	0.113241	0.082483	0.221098
-0.15439	-0.2089	0.322109	-0.63248	-0.03223	0.373189	-0.03226	0.31231	-0.03225
0.106037	0.004211	-0.4193	0.302704	0.014466	0.2954	-0.03549	0.255445	-0.01051
0.348447	0.405727	0.241841	0.392343	0.075372	0.306137	0.203962	0.142558	0.139667
-0.09376	0.172198	-0.14938	0.377396	0.146549	0.195816	0.036421	0.130096	0.091485
0.381105	-0.02172	0.667373	-0.27778	0.046697	0.191184	0.321433	0.245752	0.184065
-0.3092	0.188291	0.092364	0.242795	0.263105	0.102951	-0.02173	0.282026	0.120686
-0.07683	-0.41504	0.06409	-0.40266	-0.16694	0.192834	0.023459	0.115948	-0.07174
-0.1555	0.068671	-0.52556	0.268016	-0.07105	0.308894	-0.19643	0.23259	-0.13374
0.007465	-0.1867	0.356336	-0.33452	-0.21069	0.21944	0.026373	0.195225	-0.09216
0.043461	-0.23757	-0.33596	-0.59488	-0.17612	0.284577	-0.0443	0.226954	-0.11021
0.303228	0.276201	0.568134	0.520492	0.372577	0.135699	0.399062	0.139298	0.385819
0.234244	-0.10043	-0.08229	-0.2999	-0.02314	0.253098	0.141022	0.209986	0.05894
0.563862	0.12338	0.212493	0.336626	-0.11528	0.326858	0.266051	0.210252	0.075386
-0.20719	-0.17004	-0.47522	0.078227	-0.08114	0.225547	-0.2684	0.226275	-0.17477
-0.24374	-0.07447	-0.72065	0.165522	0.102385	0.223917	-0.28505	0.283588	-0.09133
0.051767	0.206982	-0.05592	-0.23264	0.057027	0.262485	-0.10488	0.126635	-0.02392
-0.15408	0.181266	0.279888	-0.22341	0.000577	0.326875	-0.03598	0.232054	-0.0177
-0.05653	0.054021	0.335121	0.097012	0.07086	0.083924	0.026451	0.229139	0.048656
0.071926	0.046275	0.241232	0.118203	0.14117	0.178308	0.06669	0.258006	0.10393
-0.14273	-0.23869	-0.37816	-0.56645	-0.20521	0.315759	-0.20399	0.194171	-0.2046
-0.07235	0.117883	-0.28087	-0.5984	0.021501	0.367526	-0.11477	0.306307	-0.04663
-0.12335	0.093073	-0.71466	0.282374	0.049188	0.195267	-0.11634	0.341904	-0.03358
-0.21372	-0.26976	-0.64752	-0.45762	-0.17228	0.278179	-0.22167	0.231396	-0.19698
-0.10375	0.157626	0.322176	0.614755	0.355239	0.15389	0.051977	0.224885	0.203608
-0.24017	-0.18994	-0.73985	-0.1974	-0.1828	0.237777	-0.21888	0.319797	-0.20084
-0.10149	-0.28344	-0.56191	-0.41903	-0.19288	0.296922	-0.20298	0.190982	-0.19793
0.282451	0.257131	-0.37527	0.500802	0.092965	0.29576	0.137872	0.293954	0.115418
0.022572	-0.01051	0.312646	0.08435	-0.16807	0.216976	-0.01896	0.227177	-0.09351
-0.14168	0.058651	-0.48585	0.486146	0.062175	0.240072	-0.02718	0.394468	0.017496
-0.09335	0.475968	0.234279	0.114537	0.217346	0.222063	0.047038	0.187806	0.132192
0.104911	0.109707	-0.51944	-0.20816	-0.10659	0.233699	-0.09009	0.25899	-0.09834
NA	NA	0.609352	0.055701	0.062985	0.251405	0.1712	0.31348	0.117092
0.048985	-0.01734	0.048225	-0.11981	-0.09994	0.366229	-0.06929	0.270922	-0.08462
0.194789	0.149234	0.618202	0.097313	0.151169	0.294645	0.275701	0.179462	0.213435
0.161058	0.075874	0.152965	0.475158	0.007314	0.276329	0.215613	0.072611	0.111464

0.253085	-0.10885	0.714332	0.168387	0.053103	0.229355	0.276395	0.220114	0.164749
0.128136	0.206072	0.570054	0.385009	0.313073	0.161286	0.331915	0.146151	0.322494
0.060348	-0.03923	0.055185	0.447443	0.211119	0.311156	0.069528	0.126088	0.140324
0.159696	0.121416	NA	NA	-0.00334	0.202604	0.011475	0.253422	0.00407
-0.16561	0.13773	-0.67292	-0.18345	-0.07283	0.293907	-0.14396	0.304529	-0.10839
0.416453	0.111166	0.480157	0.123882	0.15737	0.223694	0.273215	0.212673	0.215293
0.128382	0.265361	-0.21922	0.316517	0.208563	0.127453	0.089082	0.243347	0.148823
-0.00815	-0.15415	-0.06599	-0.31328	-0.25759	0.251877	-0.02358	0.236755	-0.14058
-0.01922	-0.04878	0.197598	0.357305	0.009274	0.211678	0.097282	0.228431	0.053278
NA	NA	0.040408	0.356406	0.081929	0.320651	0.201946	0.099412	0.141937
0.129978	0.008383	-0.62875	-0.63192	-0.06144	0.41581	-0.12965	0.343172	-0.09554
-0.17341	-0.41548	-0.20304	-0.57814	-0.27267	0.279397	-0.20989	0.182756	-0.24128
0.17515	-0.19217	0.114952	-0.16196	0.029732	0.242039	0.146505	0.128757	0.088118
-0.12529	-0.07836	-0.41808	0.005539	0.108951	0.117898	-0.03317	0.222802	0.037892
-0.13661	0.094598	-0.52801	0.005988	-0.18461	0.253796	-0.21584	0.188957	-0.20023
-0.33106	0.042889	-0.81566	0.121734	0.120114	0.345072	-0.19514	0.493229	-0.03751
-0.09558	0.046227	0.254949	-0.33577	-0.08273	0.316253	0.011201	0.29819	-0.03577
0.05474	0.31026	0.392529	0.206086	0.34813	0.139793	0.189807	0.157246	0.268969
0.443947	0.134381	0.700336	0.214318	0.046266	0.230933	0.332449	0.26259	0.189358
0.187347	0.319133	0.417843	0.403758	0.330818	0.0434	0.242271	0.18241	0.286544
0.11729	0.139116	-0.01833	0.363144	0.087891	0.260977	0.079022	0.242146	0.083457
0.066563	-0.07786	-0.69654	0.049578	-0.18669	0.29935	-0.02269	0.375116	-0.10469
-0.10223	-0.0996	-0.15217	-0.47258	-0.23239	0.274716	-0.23246	0.165295	-0.23243
-0.1873	-0.01881	-0.10008	-0.24787	-0.0048	0.230591	-0.09147	0.07522	-0.04814
-0.17145	0.097748	-0.48586	-0.03388	0.196588	0.315942	-0.07931	0.315228	0.058639
0.09363	0.206542	0.561754	-0.11665	-0.03063	0.231574	0.149257	0.207531	0.059316
-0.22951	0.088349	-0.01346	-0.32387	-0.06718	0.22919	-0.05971	0.36244	-0.06344
0.292581	0.290447	0.369579	0.339514	0.119606	0.228825	0.165791	0.204277	0.142699
0.352368	0.193936	0.540273	0.678755	0.146772	0.352125	0.185141	0.336682	0.165957
-0.28286	0.253114	0.280662	0.534604	0.182266	0.349039	-0.01473	0.287541	0.083766
0.124134	-0.021	0.360012	0.46942	-0.02714	0.457941	0.114726	0.231206	0.043793
-0.33034	-0.0699	-0.38245	-0.64331	-0.03813	0.394512	-0.12291	0.214419	-0.08052
0.198084	0.168598	-0.12187	0.164499	0.207251	0.164786	0.204668	0.21389	0.205959
-0.06659	0.140105	0.231042	-0.06964	0.100741	0.209983	0.138473	0.168325	0.119607
-0.39816	0.186093	-0.67337	0.334818	0.013749	0.23467	-0.30113	0.279296	-0.14369
-0.20381	-0.10054	-0.15729	0.011524	0.064021	0.304394	0.023112	0.264629	0.043567
-0.19435	-0.08132	-0.735	0.404071	-0.05579	0.258154	-0.33942	0.238227	-0.1976
0.08361	0.109222	-0.29287	-0.20602	-0.01719	0.174773	0.016708	0.180409	-0.00024
NA	NA	NA	NA	-0.15333	0.155415	0.196909	0.243671	0.02179
0.187386	-0.23044	-0.16679	-0.39242	-0.16648	0.232442	-0.01728	0.22943	-0.09188
NA	NA	NA	NA	-0.09072	0.316985	-0.07453	0.2708	-0.08263
0.087201	0.232346	0.131522	0.516536	0.082086	0.292687	-0.07099	0.171069	0.005546
0.000293	-0.35876	0.225376	-0.48826	-0.1802	0.213426	0.180316	0.111557	5.78E-05
-0.22661	0.596745	-0.14068	0.631191	0.274607	0.417023	-0.14917	0.342087	0.062718
-0.31701	-0.19604	-0.71355	-0.59541	-0.11104	0.371896	-0.25389	0.264071	-0.18247
-0.05258	-0.20347	-0.13326	-0.22581	-0.05088	0.212887	-0.01124	0.139557	-0.03106
-0.07779	-0.09216	0.308291	-0.7496	-0.23074	0.307088	-0.11124	0.231805	-0.17099
-0.00586	0.098868	-0.26938	0.268662	0.160246	0.165769	-0.03508	0.20227	0.062581
0.431835	0.164548	0.522296	0.324523	-0.01591	0.279543	0.366609	0.120606	0.175351
0.056733	-0.26196	-0.20379	-0.53514	-0.1577	0.255094	-0.12132	0.346835	-0.13951
-0.15578	0.109763	-0.01188	0.234344	0.009881	0.196889	-0.05239	0.096558	-0.02125
0.241895	0.283272	-0.24369	0.13918	0.089707	0.181874	0.142073	0.190632	0.11589

-0.10862	0.221301	-0.59046	0.266352	0.149484	0.123928	-0.00277	0.337867	0.073355
0.03368	0.145925	-0.30531	-0.40083	-0.01444	0.27518	-0.01266	0.189528	-0.01355
0.082682	0.119794	0.468385	0.342765	0.045019	0.180858	-0.00028	0.291049	0.022368
-0.08704	-0.05934	-0.1928	0.108754	0.063235	0.230989	-0.11792	0.292701	-0.02734
0.100574	0.007167	-0.11761	-0.00611	-0.00831	0.24955	0.014331	0.263523	0.00301
0.148243	0.181372	-0.51901	0.140492	-0.02932	0.225366	-0.05033	0.259257	-0.03983
0.076081	0.214885	-0.38327	0.568578	0.035912	0.311574	-0.15266	0.229551	-0.05837
-0.03946	0.052101	0.284966	-0.45717	-0.09498	0.241306	0.054166	0.246737	-0.02041
-0.00457	-0.236	-0.73045	-0.33374	-0.16525	0.125722	-0.05085	0.398117	-0.10805
0.121695	0.250365	-0.33192	0.479305	0.119922	0.219048	0.016679	0.300512	0.0683
NA	NA	-0.01019	-0.32685	0.001175	0.271655	-0.16354	0.183328	-0.08118
-0.1316	0.123997	-0.62941	-0.02894	0.020036	0.340171	-0.06769	0.365088	-0.02383
0.288608	0.176423	0.384744	0.160891	0.135098	0.216233	0.124574	0.278145	0.129836
-0.04205	-0.00324	-0.38059	-0.58479	-0.07238	0.26452	-0.13586	0.192265	-0.10412
-0.26257	-0.08949	-0.6855	0.301025	-0.10079	0.304253	-0.27003	0.233234	-0.18541
-0.14141	0.290896	-0.63373	0.266741	0.061543	0.238789	-0.14148	0.301135	-0.03997
-0.11514	-0.02707	-0.5376	-0.06996	-0.00886	0.122275	-0.06962	0.298284	-0.03924
-0.14661	0.133876	-0.67255	0.0406	0.236645	0.169698	-0.04856	0.352576	0.094044
0.123573	0.135163	0.660396	-0.10709	0.081038	0.222769	0.167655	0.302505	0.124347
-0.08646	0.636323	-0.37998	0.752824	0.649044	0.070895	-0.05987	0.277815	0.294586
-0.14452	0.071878	-0.54308	0.260879	-0.09597	0.312736	-0.20538	0.22834	-0.15068
0.23692	-0.06573	-0.07651	-0.26095	-0.05243	0.151175	0.111526	0.149407	0.029545
-0.08212	0.047448	-0.63221	0.285585	-0.11647	0.244428	-0.19639	0.216493	-0.15643
0.077519	0.231267	0.368729	0.207118	-0.02853	0.228687	0.036573	0.191553	0.004023
-0.30747	-0.00993	0.157913	0.060174	-0.07071	0.188544	-0.10542	0.243421	-0.08806
0.097184	-0.08396	-0.50652	-0.11127	-0.0297	0.12703	-0.06155	0.317369	-0.04563
0.076704	0.160773	-0.04383	0.175724	-0.02145	0.281452	-0.12519	0.207114	-0.07332
0.156677	-0.08614	0.766041	-0.25882	0.012199	0.156162	0.204852	0.372618	0.108526
0.133906	0.019432	0.457169	-0.00233	-0.00518	0.143034	0.157899	0.228489	0.076362
0.027082	0.119707	NA	NA	0.185085	0.377145	0.159601	0.222711	0.172343
0.268124	-0.24969	NA	NA	-0.21353	0.265697	0.107173	0.220672	-0.05318
-0.08906	0.083257	NA	NA	0.119839	0.142888	0.031121	0.110282	0.07548
0.214804	0.077937	NA	NA	0.080058	0.15468	-0.01535	0.134304	0.032356
0.214265	-0.02301	NA	NA	0.050854	0.400617	0.186542	0.135317	0.118698
0.0356	0.13864	-0.1748	-0.37242	0.040778	0.293622	0.096212	0.212879	0.068495
0.379642	0.140205	0.741765	0.130936	0.19342	0.115824	0.3854	0.285901	0.28941
0.169712	0.174583	-0.26314	-0.03476	0.225125	0.270494	0.039068	0.294827	0.132097
0.146537	0.085953	0.30834	0.564122	0.152105	0.301829	0.17005	0.191425	0.161077
-0.19076	-0.01088	-0.18107	0.182581	-0.05497	0.18591	-0.09196	0.133128	-0.07346
-0.35556	0.020145	-0.44685	-0.10875	-0.16347	0.149618	-0.32928	0.11359	-0.24638
0.089285	0.180222	-0.39122	0.14199	0.197402	0.241117	0.005135	0.216202	0.101269
-0.21664	0.168532	-0.01729	0.101145	0.238275	0.16085	0.09209	0.215776	0.165182
-0.0539	0.129604	0.557966	0.010704	0.018154	0.39799	0.090678	0.258707	0.054416
0.230559	0.192943	0.320472	0.070918	0.022437	0.292896	0.197502	0.184381	0.109969
-0.18727	0.000524	0.188225	-0.21936	-0.26792	0.173748	-0.14089	0.184005	-0.2044
-0.23114	-0.24047	-0.30662	-0.37049	0.029557	0.29657	-0.19859	0.195048	-0.08452
0.159502	0.237623	-0.41299	0.138095	0.049426	0.239394	-0.0079	0.241405	0.020764
NA	NA	-0.44126	-0.5068	-0.20965	0.201931	-0.30162	0.139158	-0.25564
0.250135	0.295227	-0.23036	0.281034	0.204638	0.127948	0.250097	0.241988	0.227368
0.020165	0.222786	-0.10861	0.405127	0.192885	0.287066	-0.06664	0.209756	0.063125
NA	NA	-0.4861	-0.38257	-0.32223	0.172518	-0.31463	0.182581	-0.31843
-0.04671	0.068119	-0.36015	0.301529	0.10681	0.186979	0.033867	0.271433	0.070338

-0.18208	0.135176	-0.35688	0.158073	0.116167	0.053982	-0.22731	0.113907	-0.05557
0.150686	0.058816	0.360344	-0.04211	0.066095	0.167447	0.115653	0.167709	0.090874
0.552997	0.164998	0.429696	0.536216	-0.11252	0.382369	0.41559	0.121911	0.151537
0.127119	-0.34473	0.522104	-0.2059	-0.17441	0.121157	0.033259	0.288136	-0.07058
0.105071	0.175465	-0.45164	0.433533	-0.0394	0.328458	0.009734	0.249281	-0.01483
0.220038	-0.35464	0.298623	-0.25274	-0.2024	0.215646	0.144298	0.21072	-0.02905
0.174265	0.160272	-0.25378	0.152666	0.047631	0.209449	0.044367	0.237179	0.045999
-0.30988	0.020412	-0.46976	0.164009	0.173735	0.195278	-0.14603	0.221892	0.013854
0.078549	0.078032	0.511247	0.146299	-0.15499	0.316653	0.122051	0.245609	-0.01647
-0.21811	0.236486	-0.54808	-0.10752	0.102325	0.204892	-0.03161	0.332517	0.035355
-0.15988	0.297417	0.076446	0.516829	0.209808	0.215822	-0.05877	0.215649	0.075517
0.005693	0.127967	0.153994	-0.40836	-0.05754	0.246825	0.111027	0.132401	0.026746
-0.11662	0.368356	0.522301	0.005683	-0.00264	0.315809	0.046251	0.34018	0.021805
-0.05713	-0.02043	-0.24486	-0.01101	-0.12126	0.262525	-0.0879	0.1446	-0.10458
0.36742	0.297641	NA	NA	0.162525	0.126873	0.166182	0.187459	0.164353
NA	NA	0.339605	0.257471	0.083466	0.44181	0.018545	0.299668	0.051005
-0.25276	0.036756	-0.11523	0.241135	0.056661	0.353031	-0.0681	0.167477	-0.00572
-0.05251	0.04744	-0.01232	0.045822	-0.07011	0.29043	-0.13343	0.222144	-0.10177
0.096711	0.13512	-0.04679	-0.04798	0.096953	0.240492	-0.05274	0.149703	0.022107
0.181943	0.15948	-0.22907	-0.4713	-0.12488	0.243052	-0.10342	0.212096	-0.11415
0.053214	0.077765	0.245076	-0.40767	-0.17124	0.187357	0.065491	0.190462	-0.05287
0.025376	0.237179	-0.3974	0.521095	0.133137	0.318784	0.050959	0.324187	0.092048
0.211392	0.401589	0.236503	0.650268	0.351573	0.239227	0.287037	0.106642	0.319305
-0.32522	-0.32766	0.195362	-0.40312	-0.2576	0.165285	-0.21334	0.288685	-0.23547
-0.11055	0.06776	0.026668	-0.2881	-0.13818	0.273318	-0.04699	0.236271	-0.09259
0.157828	-0.21072	0.48459	-0.29655	-0.12804	0.266859	0.095205	0.31986	-0.01642
0.470801	0.261295	0.235242	0.49195	0.140296	0.31255	0.313886	0.103879	0.227091
0.472262	0.282554	0.593678	0.410941	0.295248	0.111313	0.373932	0.172624	0.33459
0.027994	0.16631	-0.66281	-0.02778	0.002189	0.13524	-0.07922	0.299183	-0.03852
-0.02522	0.004763	-0.21887	-0.42736	-0.10832	0.235117	0.025575	0.191982	-0.04137
-0.1384	0.308429	-0.18754	0.323909	0.073831	0.189007	-0.14145	0.132042	-0.03381
-0.0222	-0.00829	-0.47022	-0.18228	-0.15732	0.103685	-0.16892	0.268956	-0.16312
0.373102	0.006196	0.412457	0.234323	0.120673	0.255494	0.248981	0.261084	0.184827
0.252522	0.323716	-0.14864	0.504597	0.161765	0.230802	0.098798	0.132562	0.130281
-0.10619	-0.34076	-0.15839	-0.38591	-0.11477	0.328992	-0.22369	0.119894	-0.16923
-0.23203	-0.24913	-0.18776	-0.34589	-0.11977	0.195534	-0.22143	0.081409	-0.1706
-0.07497	-0.2987	-0.09508	-0.52862	-0.12325	0.264613	0.010491	0.154439	-0.05638
0.029704	-0.24137	0.381817	-0.17838	-0.15458	0.240216	0.026989	0.248805	-0.0638
0.04612	-0.06393	NA	NA	-0.02935	0.040935	0.241973	0.188463	0.10631
0.045883	-0.40235	0.09971	-0.44019	-0.1935	0.212351	0.017549	0.247075	-0.08798
-0.13807	0.272055	0.136304	0.479266	0.215167	0.377288	0.100274	0.406382	0.157721
-0.07042	-0.34877	-0.22953	-0.52637	-0.17529	0.24463	-0.02511	0.139436	-0.1002
0.1835	0.080263	0.557389	0.369258	0.010158	0.378786	0.18134	0.324042	0.095749
0.080647	-0.01667	-0.307	-0.55181	-0.05916	0.38597	-0.0354	0.252255	-0.04728
0.043423	0.083856	NA	NA	0.171035	0.221607	0.210247	0.140822	0.190641
0.010503	0.132759	0.207532	0.112603	0.074327	0.29008	0.123795	0.194472	0.099061
-0.01797	0.093923	-0.1679	-0.08546	-0.04	0.208075	-0.18071	0.150716	-0.11035
-0.0406	-0.36089	0.277384	-0.48808	-0.10361	0.363921	0.053935	0.25363	-0.02484
-0.00116	-0.0042	-0.28987	-0.29945	0.00026	0.377327	-0.22975	0.263534	-0.11474
0.077263	0.03095	-0.24787	-0.5868	-0.12049	0.307985	0.061237	0.210509	-0.02962
0.325553	0.218891	0.239536	0.46297	0.322236	0.174208	0.255302	0.173123	0.288769
-0.31499	-0.30697	-0.55952	-0.62082	-0.34108	0.190315	-0.36848	0.116588	-0.35478

0.012094	-0.24725	-0.41397	-0.25633	-0.16212	0.413699	-0.17367	0.339911	-0.1679
0.100286	0.067548	0.352823	0.094339	0.114436	0.136017	0.234941	0.135908	0.174688
-0.05479	0.104213	0.252388	0.297887	0.003121	0.37106	0.03535	0.311663	0.019236
-0.04379	0.13141	0.372127	0.109395	-0.05237	0.27415	0.003402	0.182748	-0.02448
NA	NA	0.629513	0.249219	0.12217	0.352612	0.262266	0.291958	0.192218
0.481681	0.199723	0.664506	0.228555	0.194199	0.091202	0.3632	0.197849	0.278699
-0.09239	-0.34932	0.075989	-0.18978	-0.25961	0.098689	-0.16873	0.220625	-0.21417
0.093957	0.081824	-0.00827	-0.19983	-0.1318	0.284039	0.093686	0.244531	-0.01906
0.189091	0.099443	NA	NA	0.03869	0.4771	0.103455	0.146117	0.071072
0.146214	0.131485	NA	NA	-0.00373	0.139126	0.025848	0.250646	0.011062
0.080997	-0.42031	0.221904	-0.29542	-0.27713	0.125827	0.017098	0.186494	-0.13001
0.15751	0.13866	0.0018	0.243068	0.126962	0.141864	0.140427	0.144175	0.133695
-0.23178	0.152044	-0.52628	0.331634	-0.03487	0.222694	-0.15957	0.245422	-0.09722
-0.09221	-0.0115	-0.60494	-0.09183	-0.19917	0.287634	-0.04158	0.321875	-0.12038
0.196564	0.146168	0.037939	-0.3736	-0.03651	0.396394	0.124763	0.17575	0.044126
-0.2	0.303787	-0.51211	0.341074	0.237603	0.229398	-0.08076	0.249412	0.078421
0.363753	0.327409	0.316913	0.165518	0.092134	0.288571	0.225649	0.175122	0.158891
-0.03241	0.300396	-0.0018	0.144747	0.233009	0.330067	0.141826	0.146183	0.187418
-0.04448	0.017242	0.496711	0.128068	-0.01226	0.202264	0.036315	0.254464	0.012027
0.048715	-0.20617	-0.00767	0.15825	0.034612	0.419495	0.017489	0.216652	0.026051
0.045687	0.454532	0.262274	0.494264	0.377935	0.202991	0.200658	0.157724	0.289297
-0.02629	0.177607	NA	NA	0.050196	0.294459	-0.09023	0.057793	-0.02002
0.134272	0.010803	-0.19349	0.165075	-0.0366	0.186333	-0.06227	0.140779	-0.04944
0.227428	-0.25661	-0.22871	-0.11909	-0.30589	0.135786	-0.03215	0.214633	-0.16902
-0.34128	-0.02817	-0.44069	0.188778	-0.10033	0.24233	-0.32337	0.166896	-0.21185
-0.05526	-0.11048	-0.18929	-0.4823	-0.11358	0.26904	-0.06247	0.07536	-0.08802
-0.16759	0.450597	0.340842	0.331611	0.21683	0.391158	0.009132	0.190682	0.112981
0.03852	0.187464	-0.03229	-0.19315	0.096989	0.339768	0.07826	0.180773	0.087625
0.060789	0.052187	0.506834	0.235666	-0.02747	0.307767	-0.02464	0.378349	-0.02605
0.196956	0.047565	NA	NA	0.130332	0.232869	0.227409	0.187468	0.178871
0.03358	0.158122	0.347131	0.1254	0.23253	0.305461	0.085569	0.163411	0.15905
0.019588	-0.03853	NA	NA	-0.00908	0.124201	0.065173	0.251294	0.028049
-0.15861	0.150259	-0.31759	-0.12019	0.018592	0.253036	-0.00734	0.253676	0.005627
-0.02343	0.095594	-0.2462	-0.12575	-0.02266	0.117608	-0.03955	0.136969	-0.03111
0.063733	0.113641	0.049865	0.093486	0.170679	0.202076	-0.01207	0.205729	0.079302
0.112876	-0.08099	-0.04096	-0.43289	0.133879	0.327286	-0.02241	0.159807	0.055737
-0.34454	0.387072	-0.67419	0.379807	0.234271	0.235024	-0.31691	0.291858	-0.04132
-0.09593	0.157222	0.125972	0.412967	0.185992	0.154763	0.016091	0.119116	0.101042
-0.03473	0.187887	NA	NA	0.089227	0.205264	0.03067	0.049377	0.059948
0.037147	0.060526	0.227733	0.122749	0.007675	0.139336	0.028521	0.14472	0.018098
0.021026	-0.21463	-0.49664	-0.66804	-0.08558	0.324394	-0.01723	0.280452	-0.0514
-0.01771	-0.14067	NA	NA	0.043685	0.260721	0.28786	0.432139	0.165773
NA	NA	NA	NA	0.019261	0.384515	0.247988	0.37016	0.133624
0.084373	0.052736	NA	NA	-0.03326	0.247806	-0.08452	0.180408	-0.05889
0.022985	0.236529	0.088166	0.649093	0.462285	0.253676	-0.09358	0.242042	0.184352
-0.25173	-0.30315	-0.22109	-0.55913	-0.10648	0.326393	-0.16501	0.164765	-0.13574
-0.24268	0.280066	-0.68806	0.19099	0.128444	0.164116	-0.2488	0.22995	-0.06018
-0.20348	-0.28607	-0.34306	-0.17156	-0.17261	0.24031	-0.25928	0.157433	-0.21594
-0.22953	0.183649	-0.20838	-0.33365	-0.07287	0.300657	0.04534	0.210183	-0.01377
-0.11167	-0.1632	0.475466	-0.17349	-0.01845	0.251208	0.117834	0.224665	0.049691
0.140497	0.302252	0.029269	0.216958	0.22814	0.305434	0.092036	0.101394	0.160088
-0.0297	0.233109	0.238657	0.288163	0.25639	0.238965	-0.02322	0.167654	0.116585

0.03774	0.07123	-0.33806	-0.64635	-0.0173	0.377188	0.03042	0.215311	0.006561
-0.02443	-0.13998	0.001906	-0.3381	0.117257	0.433083	0.16702	0.183888	0.142138
0.015948	0.064107	-0.62889	0.210505	0.095169	0.324219	-0.03202	0.363612	0.031576
-0.07204	0.116753	-0.0929	-0.07354	0.019706	0.171284	-0.10142	0.129221	-0.04086
-0.11736	-0.15882	-0.12092	-0.3656	0.010512	0.271174	-0.0464	0.218965	-0.01795
-0.0998	-0.19748	0.335459	-0.28874	-0.10401	0.213319	0.012086	0.231459	-0.04596
-0.01681	0.12237	NA	NA	0.029874	0.343191	0.009241	0.122699	0.019558
0.11663	-0.08043	-0.02838	0.018078	-0.05248	0.191217	-0.05469	0.236458	-0.05359
-0.10382	-0.15567	0.349221	0.118751	0.012683	0.313814	0.057026	0.29074	0.034854
-0.23604	-0.28077	0.110085	-0.09695	-0.15299	0.214308	-0.08003	0.190998	-0.11651
-0.03344	-0.00385	0.068338	-0.42158	-0.06052	0.203126	-0.06078	0.08231	-0.06065
-0.02351	-0.02711	0.474769	-0.02284	0.110297	0.172931	0.040246	0.277824	0.075271
0.051089	-0.41146	-0.19747	-0.52062	-0.15693	0.284561	0.014584	0.282923	-0.07117
-0.00121	-0.18178	0.185201	-0.47096	-0.04532	0.282649	-0.0606	0.169836	-0.05296
0.030736	-0.02989	-0.50256	0.041412	-0.01801	0.239635	-0.22766	0.194732	-0.12283
0.203455	-0.05031	0.67137	0.360219	0.155614	0.179773	0.247867	0.223521	0.20174
0.013849	-0.39692	-0.4028	-0.5091	-0.21168	0.266728	-0.08813	0.262411	-0.1499
-0.16568	-0.05796	-0.39329	-0.57692	-0.01867	0.364953	-0.16557	0.153197	-0.09212
0.200317	0.248473	0.387622	-0.19495	0.062579	0.314389	0.252868	0.173333	0.157723
0.124766	0.204812	0.349323	0.242972	0.041179	0.214643	0.267545	0.106489	0.154362
-0.26696	0.067471	-0.09975	0.361462	0.164386	0.179311	-0.13282	0.274328	0.015783
-0.24732	0.246665	0.067665	0.420911	0.262079	0.140655	-0.0487	0.13896	0.106687
-0.11686	-0.24481	-0.17991	-0.47577	-0.0669	0.368376	-0.06226	0.129523	-0.06458
0.020933	0.167689	0.248433	-0.42583	-0.04729	0.230254	0.047018	0.138526	-0.00014
0.279401	0.146473	0.221004	0.201083	0.161256	0.201039	0.158552	0.185031	0.159904
0.181959	0.174951	0.419522	-0.10107	0.063298	0.148087	0.108294	0.275317	0.085796
0.200889	0.077824	0.252327	0.242276	0.015906	0.186792	0.226309	0.034481	0.121108
-0.17822	0.147587	-0.26722	0.383653	0.099116	0.262431	-0.11775	0.126232	-0.00932
0.089068	0.180164	0.249296	-0.12853	0.195049	0.232921	0.242682	0.180632	0.218866
0.284427	0.22489	0.49595	0.383013	0.152916	0.156895	0.284313	0.117101	0.218614
0.01619	0.19983	-0.00179	-0.01961	0.00621	0.2006	0.054479	0.137054	0.030344
0.215069	0.355338	0.324934	0.183904	0.182012	0.140073	0.266731	0.089863	0.224371
-0.18971	0.15706	0.021265	-0.26637	0.231183	0.321528	-0.05505	0.126115	0.088068
0.123302	-0.07119	-0.31627	-0.07148	-0.05051	0.223796	-0.08944	0.188869	-0.06998
0.00013	-0.36402	-0.25239	-0.45808	-0.24204	0.275517	-0.07075	0.230824	-0.1564
0.354762	0.081634	0.634807	0.107645	0.129631	0.236435	0.337072	0.216014	0.233352
-0.16904	-0.17357	-0.36264	-0.47552	-0.07604	0.24499	-0.2323	0.118971	-0.15417
0.084408	-0.25496	-0.40755	-0.49151	-0.10218	0.288984	-0.04481	0.207896	-0.07349
-0.0087	0.291473	-0.48926	0.43129	0.205151	0.176028	-0.03935	0.320151	0.082901
-0.29838	0.326835	-0.11812	0.295548	0.174513	0.296533	-0.30088	0.098208	-0.06318
0.211139	-0.12148	-0.27247	-0.43155	0.030242	0.306287	0.083701	0.193021	0.056971
0.073513	0.009219	-0.71898	-0.43783	-0.1923	0.224742	-0.20525	0.382945	-0.19878
-0.19018	-0.19479	-0.3508	-0.32702	-0.10779	0.162116	-0.0978	0.212358	-0.1028
0.040528	-0.19794	-0.30707	-0.42717	-0.0887	0.291274	-0.02235	0.315343	-0.05553
3.02E-05	-0.34306	-0.49585	-0.33769	-0.25142	0.374271	-0.14462	0.32612	-0.19802
-0.03256	0.132509	-0.35168	-0.3307	-0.01963	0.21517	-0.07772	0.221994	-0.04868
0.12695	0.084147	-0.16115	0.070176	-0.05995	0.147205	0.090946	0.15157	0.015497
NA	NA	NA	NA	0.035496	0.350319	0.004486	0.12734	0.019991
-0.38637	0.081635	-0.42316	-0.39513	-0.0897	0.167457	-0.17178	0.194723	-0.13074
-0.19374	-0.20013	-0.83906	-0.66946	-0.21504	0.318559	-0.35603	0.343043	-0.28553
-0.28712	0.311268	-0.6255	0.394222	0.082868	0.229716	-0.18417	0.252244	-0.05065
0.08262	0.080087	-0.4194	0.105175	-0.03602	0.213165	0.01498	0.317922	-0.01052

-0.22355	-0.40644	-0.47981	-0.54749	-0.34575	0.270926	-0.10576	0.248408	-0.22576
-0.09962	0.226003	-0.08189	0.19078	0.143296	0.153455	-0.00751	0.14145	0.067894
0.022352	-0.02934	NA	NA	-0.00363	0.299552	0.116406	0.149432	0.056386
-0.19279	-0.26894	-0.69725	-0.19628	-0.03845	0.183979	-0.33228	0.21603	-0.18536
0.00655	-0.03836	-0.3113	0.021416	-0.07456	0.366052	-0.2214	0.191944	-0.14798
-0.12922	0.136368	NA	NA	-0.11646	0.228479	0.056763	0.201616	-0.02985
-0.1299	-0.07512	-0.27346	-0.13663	0.010214	0.198329	-0.00089	0.197542	0.004661
0.018	0.052742	0.475628	0.580576	0.074304	0.434741	0.110547	0.356499	0.092426
-0.07744	0.167593	-0.12146	0.214455	0.177833	0.316852	0.021947	0.219615	0.09989
0.254641	-0.07488	0.320742	-0.21984	-0.1976	0.178768	0.120954	0.158675	-0.03832
-0.17407	0.041434	-0.34709	-0.06568	-0.08514	0.148978	-0.2683	0.19138	-0.17672
0.228475	0.308028	0.125256	0.37776	0.209782	0.304506	0.045491	0.212687	0.127636
0.364398	0.067838	0.644667	-0.10038	-0.20593	0.304199	0.257355	0.258499	0.025711
-0.10204	0.099782	-0.37606	-0.00136	0.00287	0.096149	-0.26949	0.157814	-0.13331
0.104718	-0.16403	0.146584	-0.15262	-0.20354	0.052615	0.038367	0.189215	-0.08259
-0.10694	0.2994	0.136184	-0.23196	0.000827	0.290794	0.041623	0.268379	0.021225
NA	NA	-0.37589	0.346818	0.433521	0.155708	-0.10803	0.322784	0.162747
-0.20874	0.253865	-0.06787	0.271739	0.13814	0.161909	-0.09482	0.142517	0.021661
-0.15047	0.075026	-0.26813	0.25487	-0.09778	0.285754	-0.14867	0.154891	-0.12323
-0.15162	0.35598	0.099263	0.430038	0.256445	0.316078	-0.02666	0.238544	0.114892
-0.15391	0.037585	0.135314	-0.05747	-0.07752	0.177693	-0.07961	0.191317	-0.07856
0.134451	0.12931	0.090112	0.292903	0.069161	0.353008	0.107486	0.125309	0.088323
-0.00344	0.085955	-0.09377	-0.10683	0.007094	0.151933	-0.06217	0.266573	-0.02754
0.067781	0.123901	-0.46162	-0.20498	0.051004	0.296875	0.085938	0.314707	0.068471
-0.05569	-0.0598	0.042826	-0.25406	0.113638	0.34294	0.053231	0.291275	0.083434
0.053083	-0.20962	-0.07403	-0.21031	-0.09181	0.338487	-0.00147	0.247922	-0.04664
-0.21543	0.136518	-0.00689	0.479662	0.244741	0.19758	0.01527	0.191353	0.130005
-0.21784	0.015617	-0.24124	-0.19679	0.010713	0.413089	-0.17418	0.223368	-0.08173
-0.02044	-0.26972	0.366457	-0.05075	0.119421	0.357427	0.220257	0.274969	0.169839
-0.18705	-0.10387	-0.67033	-0.46072	0.007809	0.408214	-0.19759	0.29443	-0.09489
-0.41162	-0.0243	-0.53745	-0.111	0.141178	0.190931	-0.30483	0.213631	-0.08183
-0.02689	0.152538	-0.40788	-0.49888	-0.15829	0.24775	-0.16433	0.293663	-0.16131
0.203361	0.420568	0.334373	0.384548	0.203175	0.271795	0.223291	0.115588	0.213233
0.117583	-0.06106	0.463954	0.118141	0.161773	0.226481	0.135941	0.29	0.148857
0.569493	0.207345	0.879478	0.270824	0.07348	0.157813	0.391549	0.284143	0.232514
0.092619	0.098552	0.17332	-0.41378	-0.02835	0.248507	0.232277	0.194469	0.101962
-0.17739	0.010571	0.432214	-0.05181	-0.021	0.222272	-0.0004	0.359567	-0.0107
-0.29968	-0.07552	-0.63851	-0.42788	-0.06651	0.342035	-0.2462	0.272776	-0.15636
-0.14992	-0.07536	0.111954	-0.39769	-0.03922	0.300275	-0.02732	0.154107	-0.03327
0.003378	-0.24909	0.250531	-0.13105	-0.05974	0.321761	0.057509	0.304633	-0.00111
0.160222	0.187072	0.397278	0.160816	0.143959	0.064052	0.187615	0.214319	0.165787
0.262744	-0.11611	0.007313	-0.19458	-0.01887	0.260668	0.147026	0.136231	0.064079
-0.02917	-0.26982	0.168961	-0.58066	-0.10841	0.339385	-0.04722	0.175435	-0.07782
-0.10528	-0.23161	0.077906	-0.58143	-0.17031	0.317999	-0.10001	0.168065	-0.13516
-0.3742	-0.11106	-0.21964	-0.39554	-0.05517	0.216407	-0.18311	0.158939	-0.11914
-0.29467	-0.1236	-0.72521	-0.32226	-0.10034	0.396538	-0.20701	0.365041	-0.15367
-0.17791	-0.03103	-0.16285	0.562278	0.165869	0.247448	0.012787	0.169808	0.089328
-0.11216	-0.07261	-0.71763	-0.37722	-0.10339	0.285346	-0.29727	0.255563	-0.20033
0.048763	-0.11745	-0.31394	-0.14077	-0.19372	0.252216	-0.12876	0.157646	-0.16124
NA	NA	NA	NA	0.223579	0.475138	0.133815	0.255568	0.178697
-0.13196	0.359742	-0.68936	0.145654	0.208276	0.105846	-0.11219	0.293574	0.048042
0.104727	0.215183	0.122162	0.047284	-0.01026	0.15641	0.127445	0.155432	0.058593

-0.00306	0.237016	-0.09186	-0.10939	0.049407	0.139468	0.052677	0.165749	0.051042
-0.0218	-0.10768	0.455577	-0.10469	-0.04041	0.132421	0.1238	0.221262	0.041695
0.036162	0.254201	-0.01644	0.414706	-0.0222	0.308651	-0.03351	0.113663	-0.02785
-0.00854	-0.06416	-0.53691	-0.17014	-0.12655	0.31199	-0.13508	0.253998	-0.13082
0.153841	-0.4361	-0.15688	0.070365	-0.04068	0.268595	0.07448	0.244765	0.0169
-0.20161	0.251514	-0.41342	0.625074	0.401858	0.22573	-0.27205	0.117421	0.064905
0.360533	-0.00151	0.701138	-0.13117	0.065353	0.169165	0.288911	0.314882	0.177132
-0.14664	0.188391	-0.32882	-0.21456	-0.09895	0.165196	-0.19002	0.121392	-0.14449
-0.00608	0.107597	-0.58332	0.251036	0.139645	0.17641	0.015016	0.359244	0.07733
0.1361	-0.05043	0.190496	-0.16121	-0.02799	0.09405	0.103321	0.153828	0.037667
-0.05053	0.155864	-0.0649	-0.37761	0.057543	0.373076	-0.06313	0.197628	-0.00279
0.374479	-0.36379	0.348267	-0.09805	-0.1594	0.140657	0.411478	0.166595	0.126037
0.002854	0.128032	-0.0737	-0.12562	0.243397	0.205643	0.086661	0.191156	0.165029
0.195499	-0.0689	0.541784	-0.12766	-0.16411	0.184407	0.199878	0.243541	0.017882
0.261455	0.280388	-0.19711	0.511102	0.116497	0.374335	0.115057	0.242644	0.115777
0.033126	-0.03526	-0.14055	-0.01584	-0.12419	0.263577	0.099844	0.265786	-0.01217
0.260347	-0.0941	0.501185	-0.25691	-0.09504	0.157437	0.242314	0.284763	0.073635
-0.17261	-0.30006	-0.66089	-0.3149	-0.29899	0.133741	-0.20045	0.292355	-0.24972
0.287206	0.337513	0.259718	0.161872	0.204846	0.238937	0.296152	0.119586	0.250499
0.095226	-0.09168	0.102643	-0.02797	0.103065	0.195308	0.027391	0.152113	0.065228
0.118748	0.257602	0.399568	0.489309	0.163104	0.227263	0.236387	0.130261	0.199745
0.204407	0.136964	0.31509	0.640053	0.118342	0.401837	0.185764	0.179046	0.152053
-0.47625	-0.25645	-0.2307	-0.46777	-0.13089	0.218414	-0.30656	0.117549	-0.21872
-0.15773	0.349819	0.098449	0.274926	0.167732	0.143948	0.017309	0.228719	0.09252
0.367916	0.150744	0.199279	0.542456	0.094468	0.350837	0.044454	0.328847	0.069461
-0.14974	0.01913	-0.61244	0.280046	0.070656	0.144098	-0.22177	0.26452	-0.07555
-0.11581	-0.08502	-0.35683	-0.43712	-0.04315	0.498796	0.055168	0.351601	0.006007
-0.30362	0.205708	-0.15944	-0.13763	-0.01036	0.184476	-0.24685	0.126685	-0.12861
-0.47053	0.198133	-0.38419	0.218738	-0.10413	0.279478	-0.31975	0.112623	-0.21194
0.173533	0.178674	0.485836	0.051848	0.158702	0.086929	0.183492	0.191941	0.171097
-0.03833	0.050743	0.226188	0.522437	0.119429	0.233604	-0.04226	0.177298	0.038584
0.35654	0.331795	0.353635	0.121925	0.182118	0.200929	0.22676	0.143113	0.204439
-0.11415	-0.14402	0.139096	-0.38083	-0.24125	0.188822	-0.14835	0.221358	-0.1948
-0.09466	-0.44363	-0.17541	-0.46395	-0.33887	0.104125	-0.06395	0.110248	-0.20141
0.060956	-0.0237	-0.18488	-0.43537	-0.03894	0.306573	0.084592	0.256787	0.022826
0.094388	0.048879	0.510251	0.033915	-0.00203	0.217019	0.101627	0.225246	0.049799
0.190109	0.111392	0.027077	-0.22821	-0.08252	0.257087	0.072476	0.169167	-0.00502
0.111465	0.207446	0.206845	-0.0359	0.079036	0.220805	0.04733	0.19388	0.063183
0.097216	0.076287	-0.16111	-0.05774	-0.1921	0.294833	-0.00815	0.097597	-0.10012
-0.00527	0.229763	-0.7136	0.454225	0.179739	0.180438	-0.14779	0.378378	0.015974
-0.01274	-0.19779	0.300617	-0.24506	-0.08266	0.288239	0.028298	0.208871	-0.02718
0.400123	0.168556	0.713854	0.455392	0.135489	0.422178	0.505464	0.158451	0.320477
-0.12904	-0.30136	-0.31349	-0.39423	-0.10748	0.320143	-0.05945	0.289429	-0.08346
-0.09792	0.242221	-0.56245	0.373453	0.027457	0.329927	-0.22462	0.226125	-0.09858
-0.22586	-0.09901	-0.22299	-0.4465	-0.10853	0.196079	-0.18502	0.05405	-0.14678
-0.07649	-0.08993	0.105942	-0.34306	-0.03756	0.209097	-0.00792	0.171227	-0.02274
-0.22283	-0.54002	-0.71419	-0.65625	-0.3565	0.244253	-0.37713	0.229814	-0.36681
-0.02999	-0.41802	-0.19363	-0.52644	-0.23278	0.310608	-0.05198	0.103261	-0.14238
-0.16455	-0.13838	-0.04124	0.067249	0.147502	0.260684	-0.11052	0.144348	0.018492
0.036527	0.25495	-0.21529	0.051162	0.019205	0.166704	-0.04012	0.155544	-0.01046
0.044123	0.113286	0.378133	-0.00959	-0.0265	0.267776	0.036478	0.182436	0.004989
0.110216	0.06136	0.036765	-0.07003	0.006439	0.247418	-0.03225	0.23046	-0.0129

0.24831	0.199396	-0.24194	0.001505	-0.00781	0.279074	-0.0471	0.263591	-0.02746
0.153437	0.582648	-0.01358	0.691465	0.451774	0.371843	-0.01642	0.094271	0.217678
-0.23501	-0.40171	0.187529	-0.37231	-0.29499	0.210768	-0.13447	0.214729	-0.21473
-0.25648	0.042111	-0.28254	0.431231	0.045279	0.235342	-0.29726	0.314424	-0.12599
-0.13354	-0.07449	-0.60195	-0.65112	-0.21276	0.29358	-0.34456	0.194433	-0.27866
0.273439	0.289871	0.452709	0.342772	0.109883	0.222056	0.137693	0.262654	0.123788
0.11741	0.05895	-0.00052	-0.02525	-0.05431	0.108012	0.107758	0.188541	0.026725
0.100463	0.269394	-0.06801	0.412971	0.246071	0.124597	0.101602	0.176529	0.173837
0.359785	-0.06526	0.676551	-0.32555	-0.11052	0.370057	0.325715	0.237332	0.1076
0.026106	0.341609	0.03501	0.527303	0.045673	0.314801	0.120856	0.195823	0.083264
0.320081	0.083156	0.296782	0.532564	0.099	0.353666	0.260482	0.064446	0.179741
-0.3025	0.673221	0.080883	0.636546	0.434496	0.263386	-0.15409	0.271298	0.140203
0.254581	0.069458	0.370684	0.189743	0.134854	0.194196	0.273177	0.110608	0.204016
0.063641	0.089176	NA	NA	0.146751	0.455146	0.088626	0.330759	0.117688
-0.07411	0.135418	0.161598	0.501531	0.252867	0.395315	0.022835	0.30771	0.137851
-0.00537	-0.09988	-0.07473	-0.27553	-0.17338	0.195222	-0.04538	0.092554	-0.10938
-0.02323	-0.10096	-0.17532	-0.01118	-0.10536	0.118398	-0.11436	0.174706	-0.10986
0.042896	0.362815	-0.01051	0.363537	0.062883	0.298598	-0.02971	0.157468	0.016587
-0.00221	-0.13074	-0.27648	-0.3032	-0.06785	0.374545	-0.09104	0.2509	-0.07944
0.042492	-0.43815	NA	NA	-0.19051	0.230341	0.220381	0.198466	0.014936
0.174631	-0.23603	-0.40141	-0.50733	-0.25974	0.155706	-0.08909	0.258753	-0.17441
-0.23472	0.064391	-0.1736	0.075891	-0.08475	0.226195	-0.1866	0.21548	-0.13568
0.192986	0.229299	0.089197	0.415413	0.002923	0.313322	0.109289	0.123901	0.056106
-0.04126	0.022276	-0.65952	-0.28939	-0.12881	0.246368	-0.15109	0.268097	-0.13995
0.325511	-0.28855	0.365683	-0.04285	-0.10721	0.355954	0.294819	0.145557	0.093805
-0.15036	-0.34904	-0.2575	-0.52605	-0.20029	0.306164	-0.18787	0.159356	-0.19408
-0.32214	0.101851	-0.72982	0.598456	0.139225	0.305491	-0.2649	0.293604	-0.06284
0.062644	0.081665	-0.0511	0.062582	0.092862	0.336971	0.020775	0.184208	0.056819
NA	NA	-0.19819	-0.31158	0.109157	0.276841	0.118496	0.206252	0.113826
-0.26423	0.058132	-0.27382	0.020333	0.142165	0.254525	-0.13152	0.338613	0.005323
0.01643	0.042588	0.256141	0.180418	0.088741	0.319585	0.059299	0.124398	0.07402
-0.43334	0.168662	-0.62079	-0.13761	-0.00425	0.234111	-0.28875	0.263454	-0.1465
0.076159	-0.39266	-0.21168	-0.52592	-0.22059	0.189822	-0.03379	0.215476	-0.12719
-0.285	0.023938	-0.51356	-0.03311	0.005003	0.125247	-0.12346	0.3082	-0.05923
0.008878	0.136037	0.324886	0.161372	0.207671	0.251559	0.177172	0.292905	0.192421
0.21931	0.108119	0.497959	0.140525	0.114365	0.200224	0.147374	0.277144	0.130869
0.049143	0.115061	-0.60624	0.33491	-0.00948	0.281573	-0.17237	0.246252	-0.09092
-0.03528	-0.32818	-0.27348	-0.32807	-0.25131	0.090664	-0.01293	0.195775	-0.13212
0.27333	0.231049	0.425904	0.370909	0.181868	0.153996	0.179287	0.207629	0.180578
-0.13428	0.751852	-0.44961	0.65786	0.419404	0.270959	-0.12009	0.313223	0.149656
-0.21232	0.23169	-0.22995	0.207724	0.058317	0.169452	-0.1216	0.144307	-0.03164
-0.05326	0.049843	-0.14907	-0.02877	-0.10502	0.157687	-0.11417	0.135525	-0.10959
-0.13437	0.272406	-0.47733	0.228042	0.041291	0.250127	-0.12621	0.196347	-0.04246
0.093053	-0.28722	-0.51492	-0.13791	-0.15056	0.301468	-0.05862	0.23017	-0.10459
0.057426	-0.10177	-0.63523	-0.05343	-0.01296	0.114538	-0.18838	0.387628	-0.10067
0.244284	0.268698	-0.15437	0.48092	0.072029	0.342882	0.110957	0.180221	0.091493
0.39386	0.18371	0.400537	0.566154	0.197007	0.338768	0.300312	0.095773	0.24866
0.05667	0.170876	0.225937	0.390465	0.289711	0.219988	0.266327	0.164252	0.278019
NA	NA	NA	NA	0.08965	0.261211	0.07948	0.190962	0.084565
0.067861	0.124947	-0.06439	0.064061	-0.03608	0.210104	-0.09335	0.128585	-0.06472
0.190813	0.18844	-0.38348	-0.16655	0.018361	0.327819	-0.09348	0.215963	-0.03756
0.049552	0.241818	-0.14211	0.175927	0.041103	0.203882	0.007398	0.21078	0.02425

-0.08304	0.016293	-0.47262	0.1441	-0.0603	0.175365	-0.10552	0.254721	-0.08291
-0.04951	-0.13034	0.027157	-0.41557	-0.02077	0.252465	0.085356	0.146464	0.032295
0.023182	0.13551	0.027882	-0.08221	0.205083	0.207872	0.003555	0.068339	0.104319
0.289893	-0.08333	0.281036	0.043174	0.058549	0.095544	0.240725	0.122039	0.149637
-0.10509	-0.45179	-0.16379	-0.59693	-0.19206	0.311395	-0.19419	0.196015	-0.19313
0.272669	-0.41178	-0.20979	-0.2601	-0.3055	0.223367	0.058507	0.180495	-0.1235
0.043189	0.034423	-0.44155	0.07482	-0.06895	0.137269	-0.07828	0.186707	-0.07361
-0.00571	0.222135	-0.33711	0.281737	0.151103	0.093276	-0.00328	0.182475	0.073909
-0.13588	0.109273	-0.32965	-0.24419	0.076936	0.30896	0.034773	0.270933	0.055854
-0.04587	0.030023	-0.15002	-0.05339	0.082204	0.189315	0.111697	0.218112	0.096951
-0.13523	-0.37791	0.215316	-0.58765	-0.32126	0.171255	-0.04279	0.138534	-0.18203
-0.07586	0.298317	-0.31756	0.195037	0.345504	0.136583	-0.02774	0.172959	0.158882
0.210251	0.183836	0.118709	0.628928	0.196869	0.250322	0.1349	0.084911	0.165884
-0.12459	-0.38779	-0.49191	-0.51998	-0.29082	0.219591	-0.2271	0.200204	-0.25896
-0.27229	-0.1645	-0.33339	-0.59106	-0.14769	0.290149	-0.27691	0.203196	-0.2123
-0.04189	-0.21178	0.092537	-0.46299	-0.15333	0.280786	-0.0163	0.182382	-0.08481
-0.0023	-0.14762	0.251074	-0.08175	-0.08091	0.184306	-0.00849	0.234725	-0.0447
-0.29824	0.067433	-0.58482	0.09281	0.039253	0.142926	-0.25461	0.227041	-0.10768
0.031755	0.250251	-0.197	-0.36615	-0.11828	0.270676	-0.17225	0.151093	-0.14527
0.15272	-0.03693	-0.01936	-0.11561	-0.02107	0.12685	0.143094	0.266887	0.061012
0.099107	0.178493	-0.09793	0.106742	0.159771	0.262662	-0.02432	0.1064	0.067727
0.13396	-0.10469	-0.55763	-0.22895	-0.18488	0.118736	-0.10749	0.234039	-0.14618
0.133091	-0.02332	-0.12662	-0.0745	0.120057	0.309233	0.062692	0.171576	0.091374
-0.07912	0.262876	0.314468	0.50497	0.25457	0.189404	0.097793	0.158542	0.176182
0.541804	-0.1205	0.624379	-0.26208	-0.12716	0.123985	0.42843	0.157626	0.150636
-0.06225	0.347821	-0.06514	0.288429	0.212421	0.349163	0.085833	0.237438	0.149127
-0.22446	0.129344	-0.70863	-0.05895	-0.08333	0.226903	-0.20322	0.270653	-0.14328
0.05637	0.059572	0.019687	0.191235	0.06633	0.333447	0.080054	0.132538	0.073192
-0.15414	-0.19165	-0.03259	-0.24054	-0.08576	0.331956	-0.01687	0.308611	-0.05131
-0.16422	0.044955	-0.25805	-0.0443	0.003561	0.157575	-0.20583	0.112284	-0.10114
0.053572	0.149753	0.398437	0.199523	0.027297	0.331112	0.064014	0.293741	0.045655
0.087586	0.013893	0.061726	0.166902	0.181752	0.195672	0.120855	0.132428	0.151303
0.059234	-0.11357	0.136315	-0.14475	-0.2584	0.188971	0.101038	0.101987	-0.07868
0.270775	0.051868	0.292791	0.13114	0.015792	0.146199	0.114043	0.304613	0.064918
NA	NA	0.341542	0.199452	0.082185	0.187782	0.01546	0.231477	0.048822
0.124368	0.022495	0.094242	-0.0905	-0.02631	0.079576	-0.0333	0.155422	-0.02981
NA	NA	0.062491	0.173105	-0.15567	0.209851	-0.1348	0.206736	-0.14524
-0.1015	0.218446	-0.43084	0.008444	0.201005	0.289715	0.084263	0.329476	0.142634
-0.06125	-0.02912	-0.18242	0.029976	-0.08864	0.098319	-0.12607	0.177959	-0.10736
-0.11288	0.176806	-0.23776	0.309634	-0.10233	0.328532	-0.06817	0.119256	-0.08525
-0.14323	0.228949	0.127612	0.355278	0.103314	0.265694	0.008104	0.221347	0.055709
0.279143	0.27784	0.745762	0.231904	0.163747	0.303723	0.272846	0.264497	0.218297
0.312931	0.160933	0.439837	0.303	0.017055	0.243951	0.185403	0.213035	0.101229
-0.18997	0.29967	-0.23452	0.082464	-0.02256	0.380811	-0.01828	0.249014	-0.02042
0.073735	0.204227	0.573173	0.091588	0.01394	0.145029	0.158958	0.30006	0.086449
0.117485	-0.14629	-0.00186	-0.4757	-0.16361	0.256132	-0.05565	0.104303	-0.10963
0.008534	-0.15719	-0.3614	-0.49343	-0.06818	0.272649	-0.06905	0.2024	-0.06862
-0.30945	-0.0627	-0.23625	-0.49764	-0.08349	0.334627	-0.1394	0.232512	-0.11144
0.062086	0.005463	0.673891	-0.12088	0.139758	0.162965	0.229826	0.2596	0.184792
0.182953	-0.20177	0.246398	0.111529	0.038104	0.306318	0.203775	0.163049	0.12094
0.157553	0.154609	0.042071	-0.27993	0.142731	0.287189	0.233881	0.116147	0.188306
-0.13364	-0.09636	-0.49875	-0.49361	-0.06983	0.25827	-0.1702	0.242314	-0.12001

0.311945	-0.00095	0.295597	0.073586	0.07861	0.189952	0.113093	0.206832	0.095851
0.176033	0.13884	-0.12832	0.333009	0.159368	0.325136	0.193884	0.192107	0.176626
-0.13577	-0.02823	-0.21592	-0.47194	-0.13072	0.20931	-0.16375	0.112581	-0.14724
0.018752	0.2659	NA	NA	0.297025	0.193879	-0.00317	0.139059	0.146929
0.127291	0.127008	-0.11436	-0.21685	0.064332	0.210623	0.076447	0.209211	0.07039
-0.03559	0.100153	-0.00856	-0.26352	0.055984	0.245476	-0.01743	0.108798	0.019279
-0.11281	0.296209	-0.57306	-0.22462	0.150529	0.243825	-0.07351	0.273862	0.038509
NA	NA	0.214445	-0.20541	0.010007	0.16077	-0.0476	0.244409	-0.0188
0.215136	0.156075	-0.22472	-0.12295	-0.14469	0.237189	-0.0129	0.163274	-0.07879
-0.29386	0.207476	0.240103	0.552453	0.131503	0.315285	-0.00755	0.250768	0.061974
-0.32177	0.432059	-0.12118	0.414792	0.444884	0.191817	-0.21106	0.146661	0.116912
-0.11668	0.319652	0.113443	0.371707	0.197969	0.164206	-0.10798	0.340774	0.044997
-0.00701	0.244716	0.118196	-0.19898	-0.01882	0.229012	-0.01358	0.228835	-0.0162
-0.02766	0.056478	-0.65557	-0.0773	-0.08841	0.303392	-0.19595	0.28556	-0.14218
-0.11154	-0.02801	-0.50914	0.202546	0.0786	0.124179	-0.1762	0.225331	-0.0488
-0.05301	0.366893	0.045291	0.479968	0.476667	0.152805	-0.03847	0.104878	0.219099
0.147902	-0.14577	0.43885	0.056818	-0.04201	0.087706	0.059827	0.221339	0.00891
-0.20733	0.337068	-0.29201	-0.18728	0.05082	0.17425	-0.15746	0.278309	-0.05332
-5.5E-05	0.229506	0.08767	-0.22086	-0.14855	0.286274	-0.1242	0.248185	-0.13638
-0.13823	0.023083	0.102211	-0.15297	-0.03335	0.365616	-0.12128	0.155401	-0.07732
-0.14099	-0.37471	-0.13843	-0.44947	-0.27423	0.275662	-0.14754	0.095725	-0.21088
0.018768	0.2141	-0.16741	0.153402	0.266087	0.17399	0.115416	0.211292	0.190752
-0.14937	-0.07118	0.355579	-0.01878	0.005807	0.23381	0.112582	0.295053	0.059194
0.102298	0.051479	0.026928	-0.16255	-0.15835	0.195864	0.049221	0.184081	-0.05457
-0.18501	-0.3415	NA	NA	-0.1921	0.248695	-0.0089	0.331541	-0.1005
0.095404	-0.21298	-0.01873	-0.25001	-0.10408	0.15978	0.106267	0.189975	0.001092
0.034874	0.037228	-0.00383	-0.26404	-0.08016	0.134058	-0.09782	0.136366	-0.08899
0.240217	0.016777	0.054756	0.107043	0.082922	0.335781	0.116543	0.113918	0.099733
0.315707	0.083186	-0.21597	-0.1012	-0.0488	0.116062	0.125921	0.301445	0.038563
-0.0779	0.042096	NA	NA	-0.07462	0.235341	-0.10118	0.187164	-0.0879
-0.04301	0.213282	NA	NA	0.011831	0.284645	-0.11814	0.1479	-0.05315
0.254563	0.093316	0.307219	0.441253	-0.01687	0.261344	0.281893	0.141693	0.132513
0.196147	0.02903	NA	NA	-0.09855	0.202283	0.171942	0.186513	0.036694
0.078504	0.146676	0.122705	0.060611	-0.19888	0.274098	-0.00056	0.167728	-0.09972
0.234375	0.320872	0.404272	0.613527	0.40664	0.165246	0.265626	0.116926	0.336133
0.135541	-0.05378	0.242371	-0.01349	-0.0348	0.21767	0.064823	0.26562	0.01501
0.100955	-0.27802	0.345074	-0.32796	-0.10406	0.259432	0.136498	0.163876	0.016219
0.26931	0.08952	0.046684	0.281072	0.045041	0.262914	0.168095	0.113675	0.106568
0.255725	0.151561	0.549162	0.429253	0.23274	0.121889	0.269904	0.22302	0.251322
-0.09612	0.161222	0.38027	0.320461	0.119604	0.194942	0.095375	0.276104	0.107489
0.336869	0.097354	0.189318	0.143127	-0.00812	0.304542	0.18427	0.351021	0.088074
-0.22465	0.183854	0.271244	0.072064	0.077084	0.374858	-0.10824	0.233864	-0.01558
0.017735	0.027083	-0.49199	-0.13666	0.131895	0.320516	-0.10801	0.255783	0.011944
-0.18291	0.300272	-0.51792	0.391669	0.23908	0.239484	-0.27069	0.241204	-0.0158
-0.01092	-0.22535	-0.3744	-0.48843	-0.1947	0.212448	-0.00025	0.324697	-0.09747
0.088621	-0.41101	0.403188	-0.38451	-0.13875	0.347998	-0.00612	0.27113	-0.07243
-0.0291	0.266654	-0.00305	0.171406	0.045923	0.318591	0.037341	0.121563	0.041632
0.010366	0.251975	0.391515	0.315574	0.08358	0.18303	0.085785	0.277249	0.084683
0.206982	0.086968	0.508649	0.187654	0.062662	0.129785	0.312761	0.16756	0.187711
-0.36682	0.198759	-0.40632	0.228686	0.05132	0.175434	-0.15303	0.210943	-0.05085
-0.24954	-0.2875	0.3443	-0.55586	-0.13332	0.493586	-0.07741	0.355066	-0.10537
0.337321	-0.0969	0.512625	0.353429	-0.00216	0.208166	0.276643	0.211749	0.137241

-0.14992	-0.43268	-0.20559	-0.50125	-0.19225	0.270798	-0.1244	0.151172	-0.15833
0.358918	-0.28537	0.440768	0.16506	0.083156	0.30033	0.121016	0.373712	0.102086
-0.15191	0.154629	-0.62026	0.152885	0.025611	0.307082	-0.11119	0.312927	-0.04279
0.129443	0.206672	0.761169	0.259538	0.218702	0.310185	0.347036	0.343355	0.282869
-0.2327	0.482894	0.34905	0.700973	0.439384	0.260583	-0.10134	0.294258	0.169024
-0.1693	-0.30758	-0.32234	-0.42122	-0.13774	0.216998	-0.15569	0.126241	-0.14671
0.019828	0.26001	-0.47509	-0.19061	0.081072	0.239145	-0.08877	0.212946	-0.00385
0.048468	0.166312	NA	NA	0.157163	0.012938	-0.03391	0.1165	0.061627
NA	NA	-0.32557	-0.14472	-0.02442	0.529523	-0.29603	0.255337	-0.16023
NA	NA	0.14353	-0.09572	-0.10301	0.238248	0.046576	0.171527	-0.02822
NA	NA	-0.48963	-0.10532	-0.14542	0.035358	0.053757	0.558349	-0.04583
-0.09041	0.068197	-0.51097	-0.09876	-0.0352	0.144338	-0.12103	0.260937	-0.07812
0.003119	0.143545	0.508421	0.103991	0.132637	0.274821	-0.01928	0.305566	0.056678
0.138618	-0.21767	0.316158	-0.05784	-0.10945	0.253869	0.190808	0.124391	0.040681
0.228365	0.344591	0.411315	0.145416	0.110476	0.264732	0.295987	0.235494	0.203231
-0.06285	-0.2608	0.132352	-0.48491	-0.31239	0.216739	-0.05798	0.189026	-0.18518
0.084216	-0.02435	0.333692	-0.00042	-0.0895	0.140418	0.163159	0.09675	0.036829
0.022202	-0.37332	0.395828	0.281884	-0.1072	0.23337	-0.04625	0.241257	-0.07673
-0.03441	0.139209	-0.05541	0.138294	0.266432	0.265009	0.133543	0.151382	0.199988
-0.01455	-0.07496	-0.17026	-0.67024	-0.10296	0.299351	-0.07734	0.133894	-0.09015
0.223361	0.347218	0.06974	0.093056	0.143819	0.3538	0.069836	0.19543	0.106827
0.082411	-0.09154	0.618725	-0.413	-0.08988	0.262989	0.147414	0.303717	0.028765
-0.44555	0.079984	-0.54595	-0.31393	-0.04442	0.201222	-0.29066	0.199193	-0.16754
0.029002	0.217853	0.134235	-0.17819	0.126753	0.231847	0.17343	0.209342	0.150092
-0.14187	0.033751	0.252067	0.02149	0.043188	0.115734	0.051384	0.188556	0.047286
-0.16586	-0.36569	-0.20482	-0.38696	-0.19995	0.257659	-0.11638	0.183994	-0.15817
-0.07747	-0.30633	0.373534	-0.09475	-0.01991	0.223868	-0.00134	0.189106	-0.01062
-0.00245	0.185231	-0.23025	-0.19358	0.025364	0.213012	-0.0258	0.110622	-0.00022
-0.15829	-0.39709	-0.49759	-0.57071	-0.23997	0.220115	-0.13855	0.342504	-0.18926
-0.16755	-0.37314	0.09647	-0.55642	-0.20783	0.245349	-0.1111	0.222439	-0.15946
-0.07903	0.173172	-0.3037	-0.49391	-0.07163	0.259325	0.043047	0.220765	-0.01429
-0.0651	-0.16521	0.049719	-0.35001	-0.03543	0.211334	-0.13329	0.246276	-0.08436
-0.00042	-0.26236	-0.34419	-0.09982	-0.11423	0.167505	-0.07327	0.235519	-0.09375
-0.14163	0.204674	0.26714	0.303049	0.385829	0.198111	0.140834	0.183422	0.263331
0.121227	-0.31599	-0.50715	-0.53421	-0.13492	0.270576	-0.083	0.26835	-0.10896
-0.20527	0.227209	0.049812	-0.05679	0.193097	0.305248	-0.14353	0.185342	0.024785
-0.13267	-0.36426	-0.59619	-0.57142	-0.26243	0.230328	-0.27046	0.212534	-0.26644
-0.013	0.05556	-0.78712	0.263634	-0.09847	0.212493	-0.0416	0.385971	-0.07003
0.120838	-0.01038	-0.61249	-0.30648	-0.10542	0.187436	-0.14186	0.290527	-0.12364
-0.00631	-0.14764	-0.38185	-0.47586	-0.10537	0.212508	-0.18666	0.21292	-0.14602
0.031998	-0.05743	0.021676	-0.31405	-0.10489	0.299057	-0.03123	0.128346	-0.06806
-0.16077	-0.06633	-0.77558	-0.57	-0.19433	0.313719	-0.21653	0.298553	-0.20543
0.299829	0.245056	0.65423	0.449703	0.150236	0.310187	0.283985	0.19403	0.21711
-0.03325	0.162812	0.337425	0.197577	0.07465	0.338606	0.242546	0.139557	0.158598
0.040133	-0.24159	-0.07955	-0.09439	-0.15096	0.081439	0.059342	0.103648	-0.04581
0.218236	0.160276	0.361785	0.550765	0.298586	0.267387	0.232464	0.163465	0.265525
0.075113	0.220997	0.21137	0.549635	0.238023	0.21752	0.105282	0.095255	0.171652
0.26762	0.127163	0.466049	-0.03149	0.083064	0.076893	0.296835	0.146126	0.18995
0.124565	0.200556	0.05165	0.469399	0.292789	0.259192	0.162867	0.084416	0.227828
0.011875	-0.13178	-0.36074	0.043489	-0.25657	0.200559	-0.12055	0.1715	-0.18856
-0.04289	-0.15119	-0.06652	-0.00213	-0.13049	0.243639	0.06489	0.170642	-0.0328
0.180153	-0.08549	-0.34968	-0.27958	-0.1494	0.096203	-0.07936	0.266534	-0.11438

0.069499	0.03408	0.485467	0.262187	-0.00499	0.370582	0.151204	0.226487	0.073108
0.26021	-0.00659	0.208684	0.186316	0.087203	0.1744	0.152683	0.265202	0.119943
-0.35532	-0.16402	-0.90024	0.023977	-0.11081	0.214579	-0.3765	0.280754	-0.24366
-0.10771	0.117727	-0.64807	0.142292	-0.05331	0.172217	-0.25504	0.249009	-0.15418
0.15675	-0.04082	0.468991	0.0684	-0.0206	0.079555	0.234502	0.151094	0.106949
-0.32335	-0.11103	-0.47679	-0.21916	-0.23101	0.325384	-0.15593	0.278463	-0.19347
0.188544	0.243439	0.166246	0.070021	0.202419	0.15981	0.189933	0.080543	0.196176
0.232293	0.039094	0.549322	0.351513	0.130012	0.207909	0.285291	0.158581	0.207652
-0.24336	-0.05492	-0.09036	-0.07196	-0.05764	0.249805	-0.02152	0.150611	-0.03958
0.227594	0.250714	0.108359	0.313601	0.195793	0.135963	0.160128	0.072961	0.17796
-0.03124	0.22928	NA	NA	0.184763	0.087843	0.059205	0.126868	0.121984
0.265888	-0.00659	0.292367	-0.28857	-0.10496	0.127558	0.12474	0.127474	0.009889
NA	NA	-0.59038	0.444016	-0.02289	0.311422	-0.19187	0.306089	-0.10738
0.059899	0.291496	0.298558	0.140725	0.078235	0.18335	0.04331	0.212954	0.060773
NA	NA	0.39872	-0.59079	0.039616	0.361653	0.096225	0.207931	0.06792
-0.00841	-0.01324	-0.36637	0.07716	-0.10806	0.169756	-0.23141	0.165068	-0.16973
0.261847	0.023934	-0.0485	-0.10948	-0.0819	0.293604	0.035345	0.155914	-0.02328
0.238966	-0.29731	0.030391	-0.35314	-0.3191	0.170923	0.189805	0.12458	-0.06465
-0.04303	-0.03027	-0.27526	-0.00107	-0.14877	0.155575	-0.09062	0.153939	-0.1197
-0.16186	-0.13343	0.056566	0.082006	0.118157	0.330501	-0.06301	0.1864	0.027572
0.10143	0.145792	-0.17092	-0.18163	0.08021	0.183613	0.037544	0.178413	0.058877
-0.28404	-0.32825	-0.46586	-0.12814	0.020083	0.305158	-0.25427	0.167351	-0.1171
-0.12957	0.184629	0.163749	0.089552	-0.14867	0.283127	-0.07659	0.157595	-0.11263
0.105701	0.146932	0.246744	-0.10104	-0.00434	0.23495	0.024201	0.31386	0.009932
0.061545	-0.10685	-0.28269	-0.43761	-0.09377	0.256877	-0.04652	0.173998	-0.07014
0.028349	0.244995	-0.25746	0.093478	0.125124	0.245944	-0.03298	0.223507	0.046073
-0.05605	-0.29006	-0.37366	-0.49661	-0.12521	0.231208	-0.12799	0.167618	-0.1266
-0.12147	0.026632	-0.30679	-0.03186	-0.15255	0.256233	-0.15884	0.17124	-0.15569
0.003587	-0.05227	-0.40317	-0.01069	0.059817	0.107804	-0.13433	0.189952	-0.03726
-0.1385	-0.20348	-0.03402	0.025765	-0.00407	0.21271	-0.03888	0.262724	-0.02148
0.42823	-0.17787	0.680455	-0.0384	0.013449	0.271849	0.393551	0.24274	0.2035
-0.08412	-0.25438	-0.12824	-0.48185	-0.23819	0.298665	-0.13861	0.224066	-0.1884
-0.23441	-0.08257	-0.38459	0.149506	-0.05593	0.27003	-0.08869	0.259862	-0.07231
0.507913	0.222465	NA	NA	0.030906	0.236173	0.349778	0.206915	0.190342
-0.00052	0.195306	-0.39731	0.256112	0.089296	0.579631	-0.13521	0.332432	-0.02296
-0.1173	0.05998	-0.30725	-0.08579	0.043196	0.2452	-0.1333	0.099335	-0.04505
0.030784	-0.28319	0.464965	-0.28193	-0.16766	0.205612	0.117417	0.305094	-0.02512
-0.12802	0.024204	-0.158	-0.04731	-0.04141	0.326116	0.026217	0.306257	-0.00759
0.044512	0.349186	0.449401	0.618543	0.258064	0.278173	0.136559	0.233297	0.197311
-0.26263	-0.31826	-0.13334	-0.56517	-0.33801	0.209984	-0.17373	0.101647	-0.25587
-0.18241	0.23327	-0.02603	0.106306	0.03054	0.170025	-0.05895	0.094136	-0.0142
0.13322	-0.07202	-0.12241	-0.48946	-0.18079	0.196947	0.051506	0.227352	-0.06464
0.205309	0.062409	0.088788	-0.3028	-0.047	0.203982	0.139678	0.19735	0.046338
0.217356	0.307613	-0.20056	0.725736	0.352201	0.326471	0.007868	0.187206	0.180034
0.092409	-0.02328	NA	NA	-0.01796	0.283804	0.053711	0.051326	0.017874
-0.12563	-0.34639	-0.23323	-0.38545	-0.00579	0.365872	-0.11672	0.205444	-0.06125
-0.11771	0.139041	0.10205	-0.08235	0.108564	0.203496	-0.06139	0.131122	0.023587
-0.01043	0.258562	-0.36376	0.323772	0.014228	0.246174	-0.03745	0.278741	-0.01161
0.106664	-0.38467	0.091391	-0.22731	-0.2299	0.184562	0.064362	0.172312	-0.08277
0.038366	-0.36787	-0.4705	-0.49931	-0.2461	0.200731	-0.23338	0.200988	-0.23974
0.056583	0.164278	0.114453	-0.24203	0.121893	0.221269	0.164551	0.151851	0.143222
0.191213	0.309563	-0.00111	0.290374	0.096702	0.263507	0.202863	0.228859	0.149782

-0.00723	0.221185	0.126843	-0.27468	-0.01441	0.31361	-0.00631	0.268117	-0.01036
0.312762	0.156043	0.435791	0.306986	0.211041	0.147193	0.273842	0.145266	0.242442
-0.04675	0.166529	0.624145	0.306194	0.047413	0.181291	0.01859	0.352552	0.033002
-0.36834	-0.28502	-0.04322	-0.54795	-0.18445	0.295511	-0.21447	0.139898	-0.19946
0.039176	0.231061	-0.56192	0.118782	0.073821	0.127218	-0.0598	0.345566	0.007009
-0.00519	-0.0699	-0.30564	-0.27676	-0.12726	0.119072	-0.12061	0.130876	-0.12393
-0.04227	0.032989	-0.48937	-0.4579	-0.11884	0.228994	-0.19437	0.229204	-0.15661
0.085646	-0.03714	-0.08532	-0.36579	-0.22264	0.139738	-0.05499	0.115199	-0.13881
-0.04214	-0.14638	-0.79421	-0.64694	-0.37252	0.235083	-0.3218	0.337826	-0.34716
0.141916	-0.3611	0.313187	-0.43762	-0.28801	0.142814	0.16878	0.171774	-0.05962
-0.20604	0.117181	-0.70176	-0.17514	0.03216	0.197487	-0.29367	0.281738	-0.13076
0.138237	0.079647	0.320271	0.051844	0.123473	0.161318	0.000405	0.329983	0.061939
0.357146	0.318811	0.536837	0.418667	0.265785	0.152179	0.4148	0.13992	0.340293
-0.06815	0.175552	0.265713	0.000873	-0.00583	0.295203	-0.10445	0.247183	-0.05514
-0.10134	0.328647	0.62665	0.21923	0.103968	0.277038	0.010806	0.345964	0.057387
-0.111	-0.09538	NA	NA	-0.07858	0.22502	-0.14741	0.271151	-0.113
0.023276	0.009037	NA	NA	0.025986	0.242456	-0.05521	0.220503	-0.01461
-0.26426	0.228591	0.229453	0.277946	0.051689	0.2162	-0.09051	0.230741	-0.01941
-0.14938	0.191479	-0.58959	0.171071	0.067134	0.199895	-0.12888	0.281511	-0.03087
-0.10481	-0.2311	0.301784	-0.49484	-0.15561	0.336801	-0.04695	0.198355	-0.10128
-0.34306	-0.47515	-0.55745	-0.19531	-0.31175	0.108759	-0.37461	0.173403	-0.34318
-0.16213	0.10602	NA	NA	-0.08311	0.125829	0.014479	0.127159	-0.03431
-0.3932	-0.35495	0.048599	-0.37732	-0.17588	0.229635	-0.18516	0.22465	-0.18052
-0.12872	0.136179	-0.00502	0.287667	-0.03719	0.326739	-0.07739	0.140181	-0.05729
0.129899	0.235295	-0.3487	0.224102	0.09097	0.163837	-0.15842	0.31112	-0.03373
-0.04287	0.135276	0.062411	0.072139	0.083398	0.213488	0.044403	0.101044	0.063901
0.022353	0.183677	0.035738	0.101754	0.095146	0.252189	-0.07412	0.239857	0.010514
0.116985	0.016744	-0.02105	-0.18861	0.007554	0.169062	-0.06281	0.31307	-0.02763
-0.03525	0.051047	NA	NA	-0.00702	0.090715	-0.01136	0.187642	-0.00919
-0.00916	-0.11261	0.424116	-0.24488	-0.23301	0.212215	-0.03256	0.234508	-0.13279
0.214607	0.225828	0.305766	0.460348	0.004819	0.32458	0.17365	0.11669	0.089234
-0.08285	-0.29898	0.156564	-0.56116	-0.12399	0.306711	-0.02545	0.236232	-0.07472
0.283897	-0.16496	0.188834	-0.30278	0.008903	0.222485	0.152504	0.250778	0.080703
0.076092	0.027437	-0.4809	-0.43795	-0.22064	0.161924	-0.11174	0.239663	-0.16619
NA	NA	-0.11718	-0.38587	0.109916	0.477846	0.042604	0.250852	0.07626
0.012717	0.125536	-0.08611	-0.3941	-0.09419	0.268939	0.060032	0.174678	-0.01708
0.430462	-0.06264	0.299613	0.237189	-0.04734	0.164328	0.350873	0.212635	0.151767
0.019639	0.280274	0.534371	0.524111	0.231274	0.179601	0.211943	0.314439	0.221608
0.09107	-0.08636	-0.03207	-0.20145	-0.03339	0.224742	0.083884	0.13269	0.025246
-0.18328	-0.39792	-0.4036	-0.56253	-0.22922	0.303393	-0.24056	0.159597	-0.23489
NA	NA	NA	NA	-0.05189	0.334086	0.168891	0.251524	0.058499
NA	NA	NA	NA	0.164428	0.052217	0.190115	0.189093	0.177272
-0.05879	-0.2504	-0.46702	-0.49703	-0.18264	0.229407	-0.09002	0.228344	-0.13633
0.052451	0.373243	-0.01473	0.451009	0.181243	0.209454	0.015248	0.23771	0.098245
-0.03934	0.181927	-0.15645	0.152479	-0.03175	0.317668	-0.11746	0.252635	-0.07461
-0.23212	0.461168	0.052909	0.423142	0.291684	0.317108	0.044758	0.241095	0.168221
NA	NA	NA	NA	0.211961	0.099856	-0.03803	0.111003	0.086964
0.150582	-0.20113	0.449196	-0.06921	-0.05476	0.245792	0.07352	0.411042	0.009378
0.172893	0.362635	0.23421	0.425354	0.189267	0.236035	0.270246	0.176747	0.229756
0.474642	0.191754	0.593253	0.507045	0.105026	0.261601	0.34472	0.178991	0.224873
-0.0139	-0.3446	-0.27524	-0.54272	-0.21491	0.25213	-0.15795	0.137897	-0.18643
-0.05152	0.172911	0.14637	0.19797	0.034387	0.20776	-0.0453	0.128137	-0.00546

0.099782	0.111231	0.241961	0.06895	0.1066	0.281772	0.227758	0.084633	0.167179
0.112769	0.075239	-0.44222	-0.31993	0.024342	0.254569	0.036156	0.277977	0.030249
-0.28676	0.042115	-0.21384	-0.17274	0.061882	0.18155	-0.0326	0.210512	0.014639
0.114653	-0.18543	0.096095	-0.12951	0.026765	0.224574	0.081056	0.144448	0.053911
0.149397	0.209805	0.171919	0.044504	-0.00118	0.18342	0.035215	0.175782	0.017015
-0.18658	-0.03331	-0.13591	0.08761	-0.06851	0.264155	0.027353	0.261609	-0.02058
0.101461	-0.02533	-0.3708	-0.59337	-0.07479	0.32307	-0.06832	0.262796	-0.07156
0.14385	0.176043	0.56827	0.236389	0.196722	0.159077	0.297823	0.206123	0.247272
0.100812	-0.24342	-0.20338	-0.58938	-0.11906	0.336351	0.064309	0.203354	-0.02737
-0.00692	-0.34485	0.410121	-0.40151	0.002978	0.315711	0.055097	0.215833	0.029038
0.048607	-0.34261	0.126143	-0.45207	-0.34435	0.249294	0.020762	0.226357	-0.16179
-0.00103	0.172387	-0.14038	0.246065	0.056875	0.318847	-0.0756	0.155444	-0.00936
-0.16663	0.313313	-0.05664	-0.40344	-0.01085	0.254657	-0.02887	0.169261	-0.01986
0.04995	0.220517	-0.45237	-0.451	-0.08435	0.362755	-0.06862	0.257581	-0.07649
0.325767	-0.24336	0.805976	-0.23723	-0.12745	0.266239	0.391175	0.227774	0.131863
-0.17377	-0.31092	-0.53104	-0.34024	-0.23089	0.087391	-0.31721	0.142393	-0.27405
0.043497	0.081384	-0.37917	0.302173	0.002678	0.198985	-0.16647	0.246418	-0.08189
-0.17243	-0.07054	-0.42781	-0.1319	-0.00896	0.311722	-0.13237	0.244075	-0.07067
-0.10115	0.431478	-0.12492	0.368994	0.247598	0.331937	-0.00639	0.157957	0.120605
0.044112	0.317618	0.162481	0.252764	0.299461	0.374893	0.040109	0.197194	0.169785
0.082688	-0.24415	-0.21562	-0.39752	-0.06923	0.204792	0.090667	0.250515	0.010717
-0.28424	0.537432	-0.43926	0.571402	0.468904	0.338969	-0.25972	0.358479	0.10459
-0.01458	-0.36621	0.17965	-0.50626	-0.36678	0.170232	-0.01797	0.219202	-0.19238
0.142881	0.118679	-0.40741	-0.3187	0.092007	0.220374	0.009937	0.250759	0.050972
-0.05079	-0.28288	-0.71816	-0.28079	-0.1984	0.141672	-0.16855	0.270872	-0.18348
0.065961	-0.01095	-0.14216	-0.48533	-0.0844	0.270003	-0.02123	0.175982	-0.05282
0.487014	-0.20488	0.694033	0.082769	-0.0365	0.278645	0.464046	0.193172	0.213771
-0.07377	-0.04559	0.169809	-0.64575	-0.12171	0.302397	-0.0306	0.170795	-0.07615
0.053555	-0.13734	-0.45392	-0.10508	0.016332	0.278623	0.118353	0.342624	0.067342
0.172361	0.350751	0.145158	0.052113	0.094405	0.172803	0.198109	0.138429	0.146257
-0.17491	0.177436	0.263891	-0.32518	0.128078	0.287656	0.108855	0.260171	0.118466
0.471507	0.318043	0.609353	0.544989	0.26213	0.192776	0.334356	0.241058	0.298243
-0.0985	0.349735	0.124166	0.570913	0.131063	0.290384	0.110122	0.228695	0.120593
0.108229	-0.19076	-0.06474	-0.34891	-0.07733	0.257366	0.031813	0.201359	-0.02276
-0.05826	-0.14369	0.154754	-0.58059	-0.16026	0.281248	0.051039	0.294268	-0.05461
-0.18075	0.056695	0.273897	0.234288	-0.16797	0.299551	-0.12781	0.204872	-0.14789
0.052131	0.072535	0.136368	0.063677	-0.02871	0.314453	0.038008	0.179834	0.004647
0.183581	-0.28805	0.659776	-0.29559	-0.08998	0.299969	0.215303	0.34702	0.062664
0.201249	-0.00175	0.63215	0.18669	-0.10367	0.17109	0.012365	0.378533	-0.04565
-0.08131	0.0192	-0.05228	-0.43546	-0.10907	0.22385	0.039063	0.168316	-0.035
0.024685	-0.27295	-0.35359	-0.53176	-0.31114	0.122604	-0.14603	0.190105	-0.22858
0.018724	0.07756	-0.59134	0.065617	-0.11227	0.311398	-0.11507	0.34012	-0.11367
0.341709	0.206298	-0.25422	0.058877	0.047373	0.270224	0.037998	0.277093	0.042685
0.144561	0.111397	0.473696	0.094652	0.163071	0.23118	0.23913	0.251573	0.201101
0.257017	0.137886	0.004452	0.052368	0.092248	0.200177	0.114101	0.128981	0.103174
0.101461	0.036994	-0.37549	-0.26676	0.05414	0.262174	0.011806	0.286535	0.032973
0.176568	0.118535	-0.23686	0.064207	0.122606	0.316167	0.125419	0.217987	0.124013
0.242834	-0.22919	-0.25813	-0.31101	-0.12581	0.315362	0.09917	0.216745	-0.01332
-0.18949	0.316998	NA	NA	0.202924	0.148056	0.021352	0.151065	0.112138
-0.0237	0.042417	0.636948	0.360042	0.079262	0.266361	0.139207	0.267831	0.109235
0.249187	0.062635	0.005998	-0.36606	-0.03014	0.194937	0.060557	0.117124	0.015208
0.15205	0.010409	-0.02674	-0.09504	-0.00817	0.156424	-0.09298	0.202379	-0.05058

0.092051	0.165227	-0.16919	0.297699	0.022803	0.280496	0.063508	0.212885	0.043156
0.095945	-0.00198	-0.32887	0.025173	0.11451	0.176265	-0.01454	0.200392	0.049986
-0.22374	0.410101	-0.55976	0.273155	0.246479	0.262614	0.019433	0.362093	0.132956
-0.28328	0.325999	-0.34855	-0.38437	-0.12873	0.25358	-0.12064	0.163533	-0.12468
-0.35018	0.33814	-0.29477	0.513934	0.366718	0.226597	-0.17433	0.243367	0.096193
0.009224	0.151553	-0.19867	0.208727	0.200249	0.032462	-0.06093	0.139995	0.069658
-0.0483	0.077632	-0.04625	0.086136	0.029443	0.062155	-0.10969	0.072162	-0.04012
-0.35372	0.492554	-0.01901	0.456968	0.198174	0.27552	-0.12361	0.193496	0.037281
0.12475	0.376244	0.444698	0.375836	0.177317	0.314266	0.122771	0.321388	0.150044
0.317301	0.063506	-0.23891	-0.04626	0.094401	0.223286	-0.01306	0.28382	0.040669
0.082245	0.092598	-0.36468	-0.16178	0.122421	0.266615	-0.06722	0.162717	0.027602
-0.05302	-0.21103	-0.26392	-0.4905	-0.17057	0.217353	-0.08668	0.135523	-0.12862
-0.30975	0.281333	-0.26392	0.398303	0.21008	0.133855	-0.15231	0.229665	0.028884
0.143652	-0.07936	-0.11638	-0.26845	-0.04987	0.370671	-0.03595	0.322062	-0.04291
0.03017	0.070778	-0.23487	-0.05776	-0.1351	0.228513	-0.03848	0.171213	-0.08679
-0.20912	0.109104	-0.00099	0.058935	0.047027	0.167072	-0.11691	0.207437	-0.03494
-0.17373	0.085821	-0.24078	-0.08672	0.014377	0.355247	-0.07632	0.286717	-0.03097
-0.27177	0.206022	-0.35306	0.16884	0.209055	0.079622	-0.25117	0.216687	-0.02106
-0.39537	-0.02376	0.242227	0.697253	0.188175	0.302996	0.002053	0.296181	0.095114
-0.06562	0.052143	NA	NA	-0.15205	0.357728	-0.0315	0.272408	-0.09177
-0.32942	0.226498	-0.51985	0.119072	0.212394	0.263748	-0.05488	0.307053	0.078758
-0.06076	-0.21971	0.429358	-0.04469	-0.13033	0.127321	0.126684	0.207029	-0.00182
0.25783	0.069977	0.160034	0.491666	0.046965	0.450179	0.172002	0.138678	0.109483
0.289813	0.090546	0.281118	0.010623	-0.00938	0.212115	0.15914	0.135503	0.074882
0.27062	-0.29115	-0.1699	-0.03742	-0.28564	0.175964	-0.03415	0.243833	-0.1599
0.006339	-0.04208	0.010034	-0.00944	0.07422	0.140602	0.021508	0.240459	0.047864
-0.25338	-0.41251	-0.68904	-0.51689	-0.30127	0.177073	-0.20643	0.298635	-0.25385
-0.179	-0.22099	-0.4042	-0.17144	-0.01839	0.199563	-0.11319	0.204226	-0.06579
-0.0208	0.095523	-0.63981	0.460185	0.007752	0.319006	-0.1638	0.280973	-0.07803
0.264081	0.398756	0.507447	0.190224	0.120651	0.163653	0.307788	0.12907	0.21422
0.012803	0.209127	0.197684	0.084143	0.017864	0.290356	0.02249	0.113281	0.020177
-0.16403	-0.36145	-0.44988	-0.61543	-0.12364	0.38725	-0.15734	0.164624	-0.14049
0.18313	0.187074	-0.22389	0.153304	0.171829	0.322979	0.093576	0.266882	0.132702
0.092189	0.070264	-0.12787	-0.22915	-0.09028	0.295678	0.081962	0.194715	-0.00416
NA	NA	0.101516	-0.37624	-0.08758	0.214126	0.020564	0.175524	-0.03351
-0.13245	0.197541	0.237402	0.390465	0.207725	0.251633	0.107197	0.256707	0.157461
-0.22037	0.198455	-0.60261	0.125587	0.048057	0.338281	-0.12197	0.294929	-0.03696
0.120377	0.007403	-0.53554	-0.30843	-0.10245	0.139759	-0.07248	0.264821	-0.08746
-0.34141	0.032069	0.442345	0.313745	-0.04745	0.261722	-0.07143	0.307616	-0.05944
-0.00327	-0.02462	0.491338	-0.348	-0.08519	0.247929	0.006037	0.265197	-0.03957
-0.18983	0.05061	-0.56094	0.303905	0.187157	0.193307	0.032111	0.398636	0.109634
-0.17543	0.082858	-0.48673	0.543069	0.014863	0.324449	-0.16887	0.187759	-0.077
-0.21051	-0.14821	0.214202	-0.09708	-0.04332	0.103691	-0.11524	0.273234	-0.07928
0.090159	0.256064	-0.13304	-0.18066	-0.00159	0.201398	-0.02547	0.275979	-0.01353
-0.18767	-0.28685	-0.35415	-0.48313	-0.0497	0.296297	-0.13262	0.144999	-0.09116
0.267015	0.27466	-0.20252	0.313079	0.127699	0.275931	0.10038	0.329197	0.11404
0.097222	0.268699	-0.02927	0.351401	0.31673	0.064219	0.145073	0.143506	0.230901
-0.05301	0.321215	-0.20325	0.13761	0.140505	0.208946	-0.03991	0.221316	0.050296
0.235299	0.056032	0.195983	-0.1775	0.118406	0.228472	0.131614	0.107438	0.12501
-0.15441	-0.10728	-0.20134	-0.13221	-0.03566	0.145702	-0.16986	0.119059	-0.10276
-0.18339	-0.08606	-0.15271	-0.23929	-0.05056	0.408697	-0.07797	0.303817	-0.06427
NA	NA	NA	NA	0.111709	0.331793	0.147766	0.214533	0.129738

0.126971	0.104752	0.580227	0.499978	0.221049	0.252316	0.286675	0.15528	0.253862
0.173496	0.282744	0.459077	0.203884	0.060373	0.211723	0.121106	0.284314	0.09074
0.045807	0.299182	0.049904	0.436885	0.004344	0.320959	-0.0076	0.128056	-0.00163
-0.27887	-0.05836	-0.33911	-0.3938	-0.1829	0.229893	-0.22	0.151184	-0.20145
0.460705	0.321321	0.518209	0.222893	0.178626	0.110751	0.31643	0.208754	0.247528
-0.49271	0.326957	-0.41219	0.094235	0.068768	0.272212	-0.2389	0.359776	-0.08507
0.490997	0.139632	0.653163	0.106695	0.157453	0.073188	0.408775	0.157031	0.283114
0.18588	0.188051	0.143163	0.0363	0.132229	0.111462	0.169813	0.050768	0.151021
-0.24256	-0.09286	-0.31696	-0.24525	-0.22729	0.243433	-0.28309	0.301457	-0.25519
0.096589	0.211149	-0.26157	-0.08143	-0.10925	0.230458	-0.04773	0.156935	-0.07849
0.506086	0.234971	0.326817	0.223728	0.11759	0.133773	0.274443	0.175529	0.196017
-0.23502	-0.04995	-0.38244	-0.00456	-0.09443	0.240476	-0.15111	0.27965	-0.12277
-0.02521	0.164955	-0.13521	-0.04471	-0.09892	0.236436	-0.03667	0.188996	-0.0678
0.503419	0.083819	0.599515	0.219478	0.089307	0.206102	0.349025	0.17997	0.219166
0.199004	-0.02615	0.057319	-0.21215	-0.03169	0.379218	0.033093	0.09532	0.000703
0.157978	-0.19458	-0.1358	-0.08686	0.195411	0.33181	0.093402	0.185063	0.144407
-0.10772	-0.10905	NA	NA	0.181388	0.325205	0.037182	0.240538	0.109285
0.125911	0.01183	0.557369	0.171104	0.003064	0.305503	0.095956	0.35218	0.04951
0.023222	-0.1167	-0.02215	-0.20686	-0.00671	0.185373	-0.00995	0.124497	-0.00833
-0.13776	-0.29259	-0.24288	-0.55352	-0.16124	0.281902	-0.12208	0.211395	-0.14166
-0.00761	0.226045	0.380269	0.369102	0.110875	0.163169	-0.09263	0.273112	0.009123
-0.0368	-0.30883	-0.24143	0.125524	-0.19314	0.224644	-0.13304	0.136814	-0.16309
-0.25489	0.180757	-0.53971	0.381685	0.22444	0.364793	-0.35752	0.298899	-0.06654
0.217081	0.276009	-0.34183	0.728071	0.231998	0.276484	0.026395	0.207533	0.129197
0.207067	0.211677	0.477188	0.564937	-0.02313	0.358905	0.029307	0.290653	0.003087
0.016103	0.316516	-0.17731	0.295718	0.055662	0.383308	-0.06077	0.199883	-0.00255
-0.19445	-0.01065	-0.7658	-0.30023	-0.08885	0.321963	-0.27786	0.24057	-0.18335
-0.1004	-0.17275	-0.35509	-0.00629	-0.0849	0.254092	-0.27849	0.181119	-0.18169
-0.19634	0.259531	-0.10383	-0.08389	0.025452	0.451727	-0.25396	0.23016	-0.11426
0.091946	-0.31893	0.16728	-0.47024	-0.15911	0.27896	0.006671	0.16458	-0.07622
-0.34446	-0.13677	-0.26774	-0.57315	-0.01218	0.427547	-0.27801	0.148621	-0.14509
-0.29696	0.715203	-0.30705	0.631057	0.497233	0.355297	-0.1945	0.319412	0.151365
-0.13538	0.138353	-0.18464	-0.03982	0.08129	0.217383	-0.01853	0.131569	0.031381
0.170083	0.295946	-0.09529	0.40781	0.12993	0.224984	0.074979	0.194311	0.102455
-0.10084	-0.13196	-0.41219	-0.49278	-0.23448	0.194845	-0.21841	0.165646	-0.22645
-0.29769	-0.31278	-0.1754	-0.62524	-0.18652	0.305615	-0.24728	0.151042	-0.2169
-0.21826	0.640897	-0.40592	0.679892	0.504912	0.265784	-0.15154	0.32638	0.176687
0.086113	0.349763	0.226277	0.777782	0.513887	0.213279	0.089641	0.099723	0.301764
-0.35517	-0.51076	-0.21276	-0.45095	-0.15519	0.421632	-0.16108	0.203218	-0.15813
0.044436	-0.37695	0.251582	-0.36574	-0.12763	0.319602	0.111626	0.210644	-0.008
0.061741	0.055459	0.128231	0.313918	0.249227	0.270825	0.154155	0.218713	0.201691
-0.33437	0.610086	-0.43955	0.784615	0.566941	0.243045	-0.28003	0.337559	0.143453
0.152934	0.334185	0.039078	0.214169	0.226702	0.091806	0.150018	0.115343	0.18836
-0.41273	0.639218	0.410326	0.867825	0.509524	0.323133	-0.2118	0.354574	0.148864
0.188286	-0.03922	0.226907	-0.15946	0.141713	0.371937	0.149667	0.161738	0.14569
0.046374	0.516259	0.11033	0.677393	0.313281	0.396689	0.091442	0.045333	0.202361
-0.02633	-0.20407	0.083171	-0.57346	-0.12799	0.272946	-0.08718	0.196732	-0.10759
0.202156	0.333922	-0.4064	0.035813	0.154024	0.205807	0.022114	0.236799	0.088069
0.234664	0.199148	-0.36703	0.615502	0.155849	0.258845	0.031365	0.230693	0.093607
0.263298	-0.18381	-0.54189	-0.00917	-0.05932	0.327948	-0.09541	0.32894	-0.07736
-0.13267	-0.13568	-0.11799	-0.31854	-0.02359	0.18042	-0.15895	0.147682	-0.09127
0.237697	0.330431	-0.15785	0.263949	0.120675	0.210672	0.04728	0.293443	0.083977

-0.00664	-0.35183	-0.0057	-0.50558	-0.11828	0.261693	-0.1267	0.158855	-0.12249
0.136875	0.248026	0.046346	0.651689	0.192693	0.257597	0.066108	0.088519	0.1294
0.360544	0.289382	0.573419	0.309452	0.32788	0.184165	0.287478	0.209472	0.307679
-0.16977	0.253345	-0.13112	0.196597	0.222009	0.182619	-0.01392	0.17069	0.104046
-0.08246	-0.14918	-0.02483	-0.46789	-0.01706	0.418292	-0.03604	0.103037	-0.02655
0.017556	-0.40174	-0.13109	-0.45915	-0.22433	0.170137	-0.09911	0.281003	-0.16172
0.439983	0.28375	0.123521	0.610094	0.22207	0.213776	0.309125	0.137593	0.265598
-0.32998	0.020694	-0.36472	0.014092	0.171388	0.185455	-0.27833	0.255189	-0.05347
0.324641	-0.05375	0.344689	0.707818	0.066027	0.379306	0.218625	0.168143	0.142326
0.058679	-0.17362	-0.70244	-0.6663	-0.02596	0.450654	-0.15379	0.330156	-0.08988
0.076976	0.370485	-0.21559	0.06485	0.134103	0.340635	-0.03294	0.197045	0.05058
0.08254	-0.08059	0.25355	0.215767	0.22496	0.246886	0.228601	0.221087	0.226781
-0.06051	-0.03866	0.021187	-0.28322	-0.23997	0.2676	-0.06796	0.187052	-0.15397
0.353269	0.152796	0.724484	0.641626	0.340825	0.166342	0.338378	0.219415	0.339602
0.132375	0.282042	-0.20693	0.360322	0.183112	0.237992	-0.08904	0.159503	0.047038
0.010988	-0.00618	0.512557	-0.35726	0.092572	0.355901	0.052892	0.318236	0.072732
-0.35395	-0.22976	-0.42732	-0.05624	-0.13715	0.258171	-0.26851	0.200985	-0.20283
-0.18559	-0.10044	0.035702	0.232596	-0.1117	0.244362	-0.2056	0.135969	-0.15865
-0.00299	0.062014	0.297868	0.344981	0.249398	0.218568	0.147665	0.140705	0.198532
0.018959	-0.11188	-0.05391	-0.10701	0.038532	0.270794	0.010572	0.097907	0.024552
-0.00189	-0.36595	-0.11096	-0.40229	-0.31182	0.183379	-0.03742	0.243229	-0.17462
0.19052	-0.17779	-0.17308	-0.50665	-0.05133	0.304323	0.067913	0.194233	0.008291
-0.16927	-0.19128	-0.26857	-0.64598	-0.25185	0.293811	-0.1862	0.174536	-0.21902
0.06985	0.182598	0.059977	-0.32348	0.067562	0.280525	0.097421	0.110678	0.082491
0.251533	0.144369	0.343621	0.726402	0.255732	0.300004	0.171078	0.110774	0.213405
0.154095	-0.00586	-0.34837	0.078222	0.137341	0.278222	0.052908	0.283123	0.095125
-0.05079	-0.23534	0.201955	-0.2656	-0.14302	0.259638	-0.17196	0.243879	-0.15749
-0.33156	0.180319	-0.458	0.637846	0.110868	0.319707	-0.21947	0.152912	-0.0543
-0.22018	-0.13093	0.2505	0.35342	-0.11372	0.235739	-0.0892	0.203851	-0.10146
0.201573	-0.1949	0.165032	-0.16263	-0.14591	0.286516	0.094447	0.34813	-0.02573
0.149913	-0.07686	0.122043	-0.14124	-0.01227	0.254866	0.054131	0.162125	0.020929
0.225292	-0.33332	0.297743	-0.37373	-0.10237	0.197566	0.172541	0.226043	0.035085
0.255417	-0.35936	0.181844	-0.42226	-0.14975	0.218613	0.091446	0.256939	-0.02915
0.246781	-0.02865	0.012537	0.283584	-0.20596	0.287889	-0.07579	0.178127	-0.14087
-0.18652	0.259674	0.249531	0.502746	0.304553	0.213963	-0.02465	0.373824	0.139952
-0.1099	-0.40956	-0.37032	-0.40506	-0.194	0.175438	-0.11619	0.203783	-0.15509
0.067645	-0.22162	0.325796	-0.0422	-0.05425	0.219911	0.066178	0.243565	0.005965
0.074765	0.095894	0.310235	0.127333	-0.00111	0.177651	-0.03435	0.22223	-0.01773
-0.2559	-0.27753	-0.08173	-0.61235	-0.17822	0.236783	-0.07204	0.206877	-0.12513
0.040682	0.264238	0.301749	0.470565	-0.07534	0.422944	-0.14714	0.299017	-0.11124
0.266649	0.031598	0.127507	-0.14171	-0.03152	0.203407	0.092791	0.233803	0.030633
-0.21236	-0.32544	-0.2188	-0.41628	-0.31938	0.196796	-0.27833	0.158865	-0.29886
-0.0436	0.207303	0.349044	0.334094	-0.04155	0.328144	-0.04898	0.208192	-0.04526
0.282415	0.051011	0.800245	-0.05932	0.099407	0.287483	0.241592	0.319553	0.170499
-0.17464	-0.35997	0.119469	-0.53738	-0.31689	0.176965	-0.18104	0.278812	-0.24897
-0.13907	-0.41766	0.084115	-0.3739	-0.27679	0.138728	-0.15126	0.23083	-0.21402
-0.08293	-0.33903	0.001174	-0.51232	-0.25753	0.318732	-0.08967	0.191412	-0.1736
0.326216	-0.10997	0.776585	0.176436	-0.02989	0.258266	0.259595	0.339793	0.114854
0.248644	-0.134	0.630674	0.114002	-0.21083	0.245187	0.151829	0.449169	-0.0295
0.025035	0.09102	NA	NA	-0.01134	0.091038	-0.07496	0.136365	-0.04315
-0.10047	-0.35075	-0.13093	-0.43504	-0.16248	0.190477	-0.07454	0.139073	-0.11851
0.120895	0.077704	0.370825	0.228127	0.19689	0.138056	0.08847	0.290252	0.14268

0.126018	0.296407	0.515009	0.215387	0.243999	0.310902	0.249442	0.193099	0.24672
-0.21533	0.215211	-0.57399	-0.05115	0.177551	0.141789	-0.106	0.350939	0.035774
-0.35863	-0.39988	-0.37229	-0.54535	-0.27983	0.324852	-0.20597	0.283621	-0.2429
-0.09755	-0.34434	0.042897	-0.5482	-0.20942	0.258199	-0.12301	0.229465	-0.16621
-0.21204	-0.364	-0.37684	-0.52537	-0.33642	0.192249	-0.25214	0.117519	-0.29428
-0.18055	-0.34946	-0.16534	-0.55434	-0.2322	0.240243	-0.12722	0.139199	-0.17971
-0.05057	-0.34421	0.178496	-0.23487	-0.22715	0.204195	-0.00985	0.114196	-0.1185
0.137486	0.001019	-0.48458	-0.43608	-0.05072	0.257567	-0.00648	0.277233	-0.0286
0.206723	0.092312	0.223969	0.073552	-0.05705	0.303642	0.125655	0.198328	0.034301
NA	NA	0.283135	-0.1151	-0.14677	0.197729	-0.03392	0.202361	-0.09035
NA	NA	NA	NA	0.183893	0.16795	0.117855	0.284207	0.150874
NA	NA	NA	NA	0.113079	0.448765	-0.02278	0.070922	0.045151
-0.30219	-0.0735	-0.63899	-0.12345	-0.07403	0.157337	-0.35883	0.224842	-0.21643
-0.34247	-0.17772	-0.46026	-0.39351	-0.09813	0.254213	-0.28478	0.138788	-0.19145
0.139642	0.057231	-0.13345	0.090257	0.102631	0.24676	-0.01542	0.202978	0.043603
-0.10697	-0.11158	0.305442	-0.44216	0.041325	0.397761	-0.00597	0.23396	0.01768
NA	NA	0.443292	0.10111	-0.02019	0.399164	0.238989	0.153882	0.109397
NA	NA	0.749513	0.348073	0.315648	0.125957	0.447473	0.217664	0.381561
0.236389	0.268952	0.3185	0.306998	0.121238	0.199368	0.132803	0.204421	0.12702
0.185218	0.261992	0.013639	0.226048	0.024867	0.281925	-0.07487	0.217352	-0.025
0.127819	0.436633	0.198904	0.255554	0.20482	0.281018	0.073109	0.246663	0.138965
-0.20806	0.16638	0.30943	0.376351	-0.00806	0.27291	-0.08873	0.225073	-0.04839
0.166342	0.252185	0.638967	0.20161	0.186111	0.163505	0.102104	0.343391	0.144107
0.061437	0.121675	NA	NA	-0.03688	0.370705	0.086852	0.162939	0.024986
NA	NA	NA	NA	-0.28589	0.289268	-0.23197	0.329921	-0.25893
NA	NA	NA	NA	-0.14428	0.048076	-0.09998	0.14421	-0.12213
0.069678	0.053398	-0.25122	-0.09741	0.041593	0.341456	-0.05876	0.197748	-0.00858
0.098725	0.016353	-0.40557	0.365947	0.242688	0.215865	0.016412	0.259906	0.12955
0.13324	0.107816	0.082591	0.366676	0.215505	0.1316	0.01411	0.236874	0.114807
0.183377	0.423909	0.537446	0.413172	0.227673	0.204071	0.321996	0.207107	0.274835
-0.08508	0.308753	0.022716	0.535785	0.365061	0.183174	0.006085	0.107051	0.185573
-0.13273	-0.30093	-0.08879	-0.41889	-0.1885	0.297894	-0.05066	0.163031	-0.11958
-0.15564	-0.19196	-0.57522	-0.02494	-0.17821	0.169517	-0.3029	0.146612	-0.24056
0.118277	0.150756	-0.47135	0.019102	0.192815	0.142141	-0.21182	0.329475	-0.0095
NA	NA	NA	NA	0.180684	0.247034	0.191003	0.28019	0.185843
-0.09716	0.287214	-0.56663	0.184751	-0.02161	0.205159	-0.09498	0.315591	-0.05829
-0.26387	-0.16638	-0.31687	-0.34981	-0.2304	0.115178	-0.20919	0.234455	-0.2198
0.141482	0.125498	-0.13254	0.371008	0.13946	0.151835	0.145042	0.206762	0.142251
0.169843	0.215763	0.40215	0.316208	0.157682	0.233979	0.282449	0.092864	0.220066
0.047326	0.001818	-0.16161	-0.26324	-0.04569	0.323484	0.108396	0.166148	0.031353
0.00789	0.25787	-0.19933	-0.10485	0.097999	0.238003	0.004813	0.224957	0.051406
-0.06022	-0.03877	0.430407	-0.09507	0.008904	0.247383	0.129484	0.211086	0.069194
-0.06499	-0.04537	-0.03959	-0.4095	-0.06264	0.257603	-0.02807	0.084743	-0.04535
-0.50123	-0.28765	-0.52534	-0.56134	-0.23487	0.20203	-0.28536	0.279805	-0.26011
-0.13411	-0.31551	0.031881	-0.59762	-0.21494	0.259774	0.003071	0.214335	-0.10593
-0.24549	0.01977	-0.35402	0.136186	0.050003	0.115005	-0.05744	0.192258	-0.00372
-0.23073	-0.24295	0.066215	-0.48602	-0.17005	0.215363	-0.00061	0.192662	-0.08533
0.016242	0.043302	-0.38508	-0.46666	0.019353	0.437395	-0.09345	0.221567	-0.03705
0.084146	0.059115	-0.28432	0.12182	-0.13489	0.199279	-0.0029	0.148314	-0.0689
0.115638	0.237323	0.079059	-0.01533	0.025188	0.157062	0.131325	0.122855	0.078256
-0.04014	-0.00256	-0.21274	0.105974	-0.1467	0.17668	-0.02989	0.21702	-0.0883
0.314161	-0.30169	0.185218	-0.40429	-0.11854	0.280363	0.096159	0.232385	-0.01119

0.141696	0.065932	NA	NA	0.149209	0.359243	0.405249	0.247684	0.277229
-0.23643	0.078184	-0.29341	-0.23724	-0.0721	0.281541	-0.22419	0.095627	-0.14815
0.190053	-0.26587	0.348996	-0.44414	-0.24087	0.17914	0.180463	0.164552	-0.0302
-0.11536	0.098275	-0.63871	-0.18118	-0.19002	0.186431	-0.26984	0.272881	-0.22993
NA	NA	-0.21254	-0.22908	0.107996	0.292109	-0.19265	0.260335	-0.04233
-0.13286	-0.42736	NA	NA	-0.05506	0.247896	-0.0653	0.114505	-0.06018
0.268644	-0.14413	0.305799	-0.06023	0.036943	0.203593	0.156048	0.15648	0.096495
-0.09857	-0.18737	-0.68146	0.046343	-0.16604	0.152064	-0.29249	0.29665	-0.22927
-0.35857	0.268467	-0.73607	-0.18091	-0.0013	0.192773	-0.3044	0.324923	-0.15285
0.077181	-0.25145	0.075433	-0.08841	-0.06728	0.26406	-0.04918	0.294575	-0.05823
0.031153	0.106983	NA	NA	-0.04545	0.132257	-0.01766	0.201486	-0.03155
0.107556	0.064325	-0.36692	0.166114	0.171146	0.272841	-0.00784	0.288515	0.081655
0.119003	0.147924	-0.40508	-0.12829	-0.00212	0.270426	-0.01486	0.262766	-0.00849
0.455891	0.25438	-0.05142	0.531891	0.197903	0.250638	0.312633	0.225146	0.255268
-0.11483	0.067765	-0.23283	0.136536	0.148532	0.205608	-0.05984	0.16742	0.044347
-0.06333	0.1264	-0.18171	0.463318	0.215177	0.175601	0.057448	0.216794	0.136312
-0.01638	0.083313	0.140641	-0.62111	-0.08472	0.481314	0.017619	0.283893	-0.03355
0.053424	-0.04179	NA	NA	0.222666	0.454347	0.023233	0.19319	0.122949
0.069805	-0.04232	0.203136	0.180418	-0.02548	0.198019	0.083333	0.113644	0.028926
-0.03933	-0.29745	-0.40328	-0.43913	0.019953	0.439736	-0.04063	0.279412	-0.01034
NA	NA	0.326419	-0.06207	-0.13257	0.396904	-0.04998	0.248735	-0.09127
0.141644	0.157969	0.098267	0.328755	0.015499	0.284808	-0.04771	0.149356	-0.01611
-0.01724	-0.37787	-0.25188	-0.4241	-0.26985	0.114897	-0.04527	0.257827	-0.15756
0.001206	0.183856	0.524724	0.360458	0.235394	0.238369	0.221657	0.193811	0.228525
-0.05917	0.079289	-0.48397	0.2473	0.032235	0.236893	-0.25998	0.160825	-0.11387
-0.01823	-0.25696	-0.42003	-0.48335	-0.35065	0.120673	-0.26623	0.213673	-0.30844
0.040615	0.098777	0.516571	0.211357	-0.0884	0.254516	0.139059	0.322354	0.025328
0.174701	-0.11243	0.143708	-0.29015	-0.01044	0.242266	0.096289	0.187232	0.042924
0.245124	0.298427	-0.39013	0.291862	0.192118	0.267543	0.0053	0.283921	0.098709
0.035197	0.139992	0.268684	0.146047	-0.07446	0.236665	0.012263	0.290778	-0.0311
-0.12965	0.135571	-0.39307	-0.38476	0.084278	0.338691	-0.15522	0.159946	-0.03547
0.498572	0.391044	0.683463	0.348532	0.19005	0.157767	0.436477	0.170797	0.313263
0.156528	0.223407	NA	NA	0.216338	0.269649	0.150314	0.205702	0.183326
-0.03876	0.101438	-0.18227	-0.31272	0.081624	0.449703	-0.01543	0.140261	0.033098
0.138718	-0.01032	-0.06837	-0.30521	-0.15742	0.329072	0.078633	0.427254	-0.03939
0.229398	0.42541	0.658086	0.481584	0.338832	0.176425	0.283859	0.259554	0.311346
0.499292	0.132151	0.682301	0.285926	0.034481	0.181332	0.306324	0.288333	0.170402
-0.07722	0.031399	-0.08739	0.064176	0.032043	0.206486	-0.11308	0.055574	-0.04052
-0.09845	-0.31108	-0.44024	-0.3425	-0.08434	0.267535	-0.13811	0.253965	-0.11122
0.024021	0.215757	-0.23312	-0.39141	-0.0854	0.255565	0.069668	0.236096	-0.00787
0.112733	-0.02649	0.007574	-0.21172	0.046994	0.353358	0.001906	0.236409	0.02445
0.351403	0.41118	0.228412	0.42849	0.318886	0.204579	0.218854	0.136557	0.26887
0.108001	0.154349	0.091114	0.386507	0.157606	0.226267	0.028825	0.225053	0.093216
0.264942	-0.02287	0.426189	-0.16527	0.006364	0.143226	0.217306	0.184947	0.111835
0.125527	0.105835	0.522064	0.098527	0.174369	0.312898	0.096985	0.337704	0.135677
0.185569	-0.18922	0.550679	-0.22871	-0.12939	0.284957	0.107222	0.309389	-0.01108
0.136737	-0.13584	0.431008	-0.47542	-0.13744	0.315455	0.228199	0.252113	0.04538
-0.24489	-0.19473	-0.80877	-0.03025	-0.09665	0.283348	-0.24094	0.330231	-0.1688
-0.1439	0.057314	-0.58346	0.003436	-0.00748	0.049081	-0.2568	0.235199	-0.13214
-0.19961	-0.41634	-0.09079	-0.56279	-0.12312	0.310433	-0.16856	0.173353	-0.14584
0.302359	0.185426	0.583499	-0.00414	0.173176	0.226453	0.393543	0.129131	0.28336
-0.408	0.262283	-0.17576	-0.26604	0.077453	0.212874	0.003448	0.285155	0.04045

-0.13088	0.328885	0.536456	0.372036	0.096323	0.216217	-0.06824	0.354846	0.01404
-0.13463	-0.43852	0.193389	-0.56092	-0.13585	0.331579	0.002769	0.14391	-0.06654
0.089685	-0.06362	-0.32422	-0.09809	-0.17306	0.129493	-0.12604	0.264539	-0.14955
0.449688	0.284578	0.387345	0.217003	0.167811	0.283767	0.400712	0.087907	0.284261
-0.32946	0.224324	-0.40952	-0.02027	0.069944	0.23775	-0.25537	0.143131	-0.09271
-0.15967	0.269111	-0.23335	0.24166	0.104558	0.184769	-0.12082	0.139948	-0.00813
0.038308	0.279311	-0.09835	0.382234	0.055247	0.220612	-0.02245	0.187802	0.016397
0.040294	0.097382	-0.07565	-0.02325	0.115577	0.329986	0.055561	0.193416	0.085569
0.054627	0.045814	-0.40823	-0.49138	0.017166	0.364118	-0.07869	0.246352	-0.03076
-0.12366	-0.2933	-0.39489	-0.45474	-0.0937	0.267277	-0.10833	0.183381	-0.10101
0.514596	0.15302	0.685362	-0.05844	0.158467	0.176568	0.41354	0.202839	0.286004
0.102684	0.105206	-0.0547	0.003202	-0.09141	0.319705	0.122769	0.182789	0.015678
-0.00992	-0.31664	-0.80725	-0.12138	-0.12374	0.184057	-0.17942	0.450735	-0.15158
-0.01528	0.245933	NA	NA	-0.04898	0.188667	0.155878	0.223836	0.053447
-0.20061	-0.32012	-0.11918	-0.3592	-0.38809	0.10612	-0.21964	0.161372	-0.30386
0.130413	0.377808	0.550164	0.416287	0.177296	0.284967	0.080414	0.379748	0.128855
-0.05105	0.108003	-0.35906	-0.00731	0.042114	0.121786	-0.09872	0.250764	-0.0283
0.305641	0.412354	0.483421	0.051845	0.254363	0.250401	0.302362	0.18567	0.278362
NA	NA	0.278221	-0.01633	0.223341	0.193064	0.190556	0.292049	0.206949
0.176285	0.022348	0.288922	0.483256	0.119877	0.244601	0.266213	0.142771	0.193045
NA	NA	NA	NA	-0.01548	0.193951	-0.04433	0.224277	-0.02991
-0.0618	0.277364	0.379746	0.086464	0.127274	0.15067	0.043819	0.195528	0.085547
0.021069	-0.30195	-0.45979	-0.5163	-0.22361	0.202805	-0.13356	0.218817	-0.17858
-0.02374	0.018716	-0.32067	0.068251	-0.03025	0.192034	-0.19252	0.158981	-0.11139
-0.04035	0.040911	0.023377	-0.03819	-0.20147	0.238725	-0.14137	0.195739	-0.17142
-0.0727	-0.19227	-0.29309	-0.37494	-0.13213	0.305231	-0.03572	0.324544	-0.08393
-0.19549	-0.1701	-0.35994	0.451939	-0.03003	0.24003	-0.11429	0.172078	-0.07216
0.03835	-0.40247	-0.06466	-0.23383	-0.14226	0.191518	-0.01839	0.272001	-0.08032
-0.09731	0.145094	-0.17729	0.025174	0.187757	0.314926	-0.13948	0.204771	0.024139
-0.08867	-0.06286	-0.04355	-0.02953	-0.01877	0.35173	-0.18671	0.144027	-0.10274
0.21411	0.144095	0.360991	0.409316	0.217594	0.169514	0.304502	0.070942	0.261048
0.035221	0.071634	0.092813	-0.51492	-0.22405	0.273232	-0.15611	0.18935	-0.19008
-0.07992	-0.1389	0.085661	0.080257	0.029916	0.106528	-0.07821	0.134394	-0.02415
-0.04163	0.088342	0.352725	0.387504	0.249179	0.185386	0.098773	0.154998	0.173976
0.074739	-0.05755	-0.01785	-0.11836	-0.02131	0.209455	0.031212	0.118465	0.004953
0.140906	-0.21292	0.305456	-0.71792	-0.11075	0.462811	-0.02602	0.226133	-0.06839
0.192935	-0.09268	0.106551	-0.09501	0.035138	0.35734	0.211865	0.105551	0.123501
-0.18198	0.153009	-0.65228	0.137383	0.199122	0.130122	-0.32045	0.251982	-0.06067
0.103194	0.180027	-0.05649	0.514633	0.163735	0.298774	0.101849	0.112843	0.132792
-0.19122	0.062163	-0.47354	-0.31652	-0.08637	0.176442	-0.23228	0.21461	-0.15932
0.076188	0.038392	0.218599	0.02719	-0.17989	0.245965	-0.11543	0.269497	-0.14766
0.049949	0.166666	-0.68464	0.093835	0.031027	0.310873	-0.25837	0.239956	-0.11367
0.410326	0.238558	-0.19286	0.224933	0.287529	0.241861	0.064808	0.33009	0.176169
-0.33488	-0.18533	-0.48628	-0.13765	-0.26164	0.209033	-0.32973	0.171309	-0.29569
0.085764	0.24618	-0.11872	0.426346	0.101135	0.206278	0.010581	0.186824	0.055858
0.090255	0.028281	-0.17922	0.213767	-0.1268	0.281031	0.033557	0.302794	-0.04662
-0.16648	0.226593	0.05565	0.024091	0.098538	0.332531	-0.04205	0.156884	0.028244
0.471561	0.103001	-0.24524	0.287279	0.210616	0.232346	0.322728	0.299855	0.266672
0.351603	0.181826	0.577914	0.197123	0.272693	0.155182	0.267611	0.237915	0.270152
0.057999	0.308496	0.602022	0.034949	0.346864	0.228935	0.217355	0.23233	0.28211
0.372445	0.2754	0.677923	-0.021	0.077447	0.184478	0.441487	0.189915	0.259467
-0.14524	-0.43948	-0.38183	-0.17161	-0.29222	0.181032	-0.13513	0.197041	-0.21368

-0.00517	-0.43554	-0.06564	-0.50069	-0.38002	0.169918	-0.08167	0.094444	-0.23084
-0.30887	-0.39634	-0.37753	-0.52738	-0.41811	0.167492	-0.23513	0.157192	-0.32662
-0.1899	0.189272	-0.46235	0.215365	-0.02874	0.321387	-0.13943	0.263777	-0.08408
0.19275	-0.01712	-0.21926	-0.02826	-0.12177	0.150139	-0.09501	0.228663	-0.10839
0.123985	-0.12479	0.204436	-0.22017	0.041976	0.192918	0.131164	0.159669	0.08657
-0.29882	0.046121	-0.58236	-0.03067	0.0223	0.130574	-0.20756	0.252304	-0.09263
-0.06179	0.01121	0.262098	-0.31044	0.005714	0.274311	0.070501	0.193204	0.038108
-0.05924	-0.0528	-0.45588	-0.43442	-0.07626	0.234085	-0.09654	0.21924	-0.0864
0.223008	0.266542	0.544	0.087613	0.081588	0.266906	0.204331	0.281531	0.14296
0.021616	-0.18572	0.172229	-0.31101	0.097725	0.356508	0.065935	0.178194	0.08183
0.314718	0.175645	-0.00423	0.390208	0.12043	0.237888	0.166961	0.139647	0.143696
-0.36533	-0.1112	-0.46114	-0.22589	-0.05494	0.169696	-0.13922	0.303017	-0.09708
0.573394	-0.12745	0.751274	0.310699	0.116424	0.239699	0.273913	0.390266	0.195168
-0.19085	0.189161	-0.47786	0.161152	-0.06959	0.200967	-0.25441	0.192972	-0.162
0.042484	-0.17486	-0.0181	-0.1127	-0.18032	0.208524	-0.08395	0.234321	-0.13213
-0.10353	0.102476	-0.37794	0.063644	0.053122	0.153609	-0.02522	0.267096	0.013953
0.268038	0.495703	0.609074	0.577054	0.341613	0.238949	0.252734	0.264392	0.297173
0.40594	0.384138	0.190074	0.325533	0.26531	0.191641	0.185551	0.27401	0.225431
0.235769	0.175789	0.322608	0.043677	0.088251	0.170311	0.080682	0.249162	0.084466
-0.30256	0.074898	-0.71975	0.107861	-0.08349	0.146325	-0.19556	0.295648	-0.13953
0.108296	0.209164	-0.46661	0.015609	0.117289	0.286677	-0.15368	0.238403	-0.0182
0.129832	0.273546	0.103257	-0.09287	0.00922	0.183072	0.204901	0.197311	0.10706
-0.13611	-0.42101	-0.48084	-0.57369	-0.27478	0.232976	-0.15703	0.20042	-0.2159
0.014693	0.188054	-0.24995	-0.43362	0.106501	0.375005	0.140176	0.348705	0.123339
0.085352	0.356055	0.097932	0.247656	0.1599	0.283472	0.120027	0.269398	0.139963
-0.19888	0.215029	0.109102	0.297028	0.096083	0.214934	-0.08779	0.259549	0.004147
0.038636	-0.17689	-0.34151	-0.46697	-0.05488	0.263426	0.003259	0.22429	-0.02581
0.231645	0.107082	0.005623	-0.00283	-0.18337	0.206089	-0.09622	0.318534	-0.13979
0.206743	0.042683	0.244286	0.282198	0.025137	0.225115	0.01518	0.194309	0.020158
0.061998	0.112534	0.080467	-0.16208	-0.04768	0.107334	-0.00609	0.075915	-0.02688
-0.06818	-0.04924	0.114647	-0.02003	-0.02107	0.301577	-0.06189	0.204798	-0.04148
-0.22065	0.313012	-0.52384	0.134402	0.227948	0.314314	-0.23866	0.25826	-0.00536
-0.29711	0.116565	-0.67986	-0.10044	-0.13931	0.211596	-0.2875	0.273111	-0.21341
0.184479	-0.13458	-0.36561	-0.45863	-0.01261	0.349798	0.061974	0.225062	0.02468
0.055287	0.010478	-0.26158	-0.45821	-0.15552	0.371078	-0.06397	0.225596	-0.10974
-0.19329	-0.43447	-0.07159	-0.41433	-0.16082	0.297133	-0.13772	0.095398	-0.14927
-0.1591	0.130067	0.174749	0.12874	0.041062	0.153166	0.002038	0.205709	0.02155
0.141728	0.25678	-0.17751	-0.04464	0.125867	0.150231	0.054197	0.192652	0.090032
-0.06825	0.051457	-0.11442	-0.13577	0.180953	0.239159	-0.12112	0.135047	0.029914
0.160167	-0.06579	-0.11364	-0.18693	-0.00834	0.228136	0.143963	0.297987	0.06781
-0.09126	-0.06086	-0.61197	-0.40883	-0.02751	0.2637	-0.09312	0.303065	-0.06032
-0.12949	0.223344	-0.15034	0.182876	0.216081	0.093513	-0.09329	0.068125	0.061397
0.142279	-0.16087	-0.07134	-0.2288	-0.30061	0.168557	-0.09723	0.192885	-0.19892
0.083506	0.287417	0.050445	-0.02499	0.138223	0.211477	0.121951	0.075483	0.130087
0.450449	-0.13672	0.522711	0.060636	-0.00539	0.176571	0.150134	0.351465	0.072373
-0.17746	-0.08761	-0.34968	-0.02606	-0.27642	0.210611	-0.04324	0.243033	-0.15983
-0.04323	0.264521	-0.06856	0.158745	0.035236	0.239131	0.100524	0.194883	0.06788
0.091182	0.114771	0.142632	-0.09574	0.011428	0.184228	0.104149	0.111118	0.057789
0.020523	0.09733	-0.17774	-0.08894	-0.08297	0.149244	-0.00452	0.091358	-0.04375
0.014721	-0.11827	-0.21489	-0.16063	-0.28461	0.211678	-0.02229	0.17902	-0.15345
-0.12596	0.055911	-0.2256	-0.02866	-0.06676	0.197544	-0.14687	0.145363	-0.10682
0.024212	-0.31541	0.028137	-0.2494	-0.27037	0.17847	-0.0053	0.153389	-0.13784

0.364496	0.283991	0.647483	0.286104	-0.03955	0.421808	0.155363	0.306027	0.057908
-0.02555	-0.17806	-0.08135	-0.1952	-0.24855	0.183831	-0.16423	0.185741	-0.20639
-0.04251	-0.03671	-0.06197	-0.07524	-0.08728	0.318905	-0.00084	0.165227	-0.04406
-0.08719	-0.10262	-0.28213	-0.19085	-0.23778	0.134178	-0.11027	0.224425	-0.17403
0.145635	-0.2683	-0.13461	0.054119	-0.14693	0.280509	-0.00356	0.136143	-0.07524
0.31311	0.235927	0.419179	0.157216	0.183257	0.307484	0.193577	0.318586	0.188417
0.222969	-0.32032	0.508939	-0.2143	-0.31619	0.212945	0.053493	0.286634	-0.13135
0.246929	0.132848	-0.17522	0.003777	0.026277	0.157226	-0.09967	0.264336	-0.0367
-0.12821	0.185421	-0.21019	0.097594	0.064222	0.209559	-0.023	0.159419	0.02061
0.106248	0.170646	0.13379	0.512191	0.070734	0.433982	0.1104	0.154236	0.090567
0.000415	0.280893	-0.04877	-0.14068	0.018942	0.224123	-0.00902	0.254859	0.00496
0.411199	0.280245	0.599357	0.190437	0.060203	0.169134	0.24437	0.261733	0.152287
0.167606	0.149259	-0.10795	0.168909	0.127375	0.105855	0.076634	0.148085	0.102005
-0.24733	0.186768	-0.412	0.362628	0.140587	0.299532	-0.18579	0.165032	-0.0226
0.134097	-0.16549	0.170002	0.099676	-0.23278	0.242441	-0.10678	0.236191	-0.16978
0.326833	-0.19356	-0.02082	-0.25699	-0.12022	0.11644	0.217272	0.240474	0.048527
0.040227	0.107489	-0.13003	-0.04235	-0.0609	0.149168	-0.05874	0.172566	-0.05982
-0.18595	-0.07995	-0.42876	-0.16398	-0.14301	0.219903	-0.20326	0.228789	-0.17313
-0.21067	-0.04958	-0.21677	-0.26022	-0.03301	0.354229	-0.12922	0.217605	-0.08111
0.047519	-0.24009	0.27679	-0.07507	-0.0791	0.200582	-0.01816	0.183738	-0.04863
0.079466	0.044402	0.5513	0.155095	0.033452	0.18578	0.203084	0.190875	0.118268
-0.23033	-0.24299	-0.57043	-0.04579	-0.16889	0.146946	-0.3634	0.202908	-0.26615
0.182944	-0.17745	0.047082	-0.33299	-0.10523	0.163077	0.065027	0.20343	-0.0201
0.126985	-0.02696	0.641739	0.091531	-0.07338	0.289724	0.193576	0.233783	0.060096
0.443967	0.066017	0.792952	0.288983	0.068426	0.182092	0.287314	0.3218	0.17787
0.052583	-0.14759	0.61659	0.088572	-0.19509	0.214888	0.019168	0.333889	-0.08796
-0.13172	-0.32829	-0.51543	-0.39934	-0.17818	0.185601	-0.24356	0.18723	-0.21087
0.063815	-0.04633	0.019006	-0.16054	0.1171	0.204811	0.140445	0.088408	0.128772
0.206277	-0.29093	0.342314	-0.36828	-0.38817	0.101175	0.147391	0.14933	-0.12039
0.017857	0.056735	-0.29846	0.13486	-0.0521	0.268833	0.027001	0.177602	-0.01255
0.190001	0.310971	0.586308	0.479614	0.217607	0.287542	0.37467	0.182348	0.296139
-0.36206	0.111601	-0.83744	0.15722	-0.05926	0.337354	-0.31094	0.36889	-0.1851
0.128483	-0.21631	0.219438	-0.34354	0.029697	0.275028	0.103279	0.209493	0.066488
-0.20027	0.282419	-0.63249	0.161598	-0.02605	0.198884	-0.14332	0.262744	-0.08469
-0.20677	-0.16293	-0.25794	-0.23338	-0.03474	0.360132	-0.26861	0.214063	-0.15167
-0.02351	0.153677	-0.06635	-0.0768	0.107989	0.11838	-0.1394	0.178095	-0.01571
-0.40431	-0.10816	-0.37019	-0.002	-0.00738	0.169026	-0.33246	0.129803	-0.16992
0.539408	0.003544	0.445988	0.68948	0.01016	0.3689	0.375238	0.17914	0.192699
-0.06348	0.020099	-0.38402	-0.51132	0.009859	0.403771	-0.2006	0.170698	-0.09537
0.059372	-0.11488	-0.29135	-0.32003	-0.1518	0.142885	-0.00267	0.160266	-0.07724
0.031435	0.259223	-0.56875	0.136609	0.2061	0.194095	-0.08028	0.302282	0.062912
NA	NA	NA	NA	0.009762	0.50905	0.148653	0.121574	0.079207
0.313521	-0.15282	0.343751	0.028312	-0.05471	0.318885	0.097	0.363011	0.021145
-0.07177	0.306607	-0.13796	0.142035	-0.08068	0.275082	-0.03841	0.152059	-0.05954
0.157237	0.173218	0.415707	0.081062	0.108555	0.35623	-0.00491	0.300434	0.051823
0.097518	0.116201	0.336437	-0.20104	0.170942	0.279259	0.217654	0.125703	0.194298
-0.13379	-0.05777	-0.22766	-0.05401	-0.10964	0.288137	-0.07238	0.192023	-0.09101
-0.00438	-0.20796	0.012771	-0.47019	-0.12869	0.239339	-0.00424	0.125922	-0.06647
0.15393	-0.28261	0.061377	-0.16721	-0.14618	0.16617	-0.09783	0.209555	-0.12201
-0.19874	-0.38828	-0.20964	-0.08341	-0.2987	0.160569	-0.1574	0.214909	-0.22805
-0.04226	-0.1487	0.14037	-0.29946	-0.11248	0.246635	-0.15688	0.268803	-0.13468
0.197451	-0.13609	-0.00743	0.268688	-0.03058	0.211294	0.073159	0.158444	0.021289

0.090437	-0.11694	0.111558	-0.54487	-0.08067	0.390091	0.021817	0.228496	-0.02943
0.459847	0.464711	0.680605	0.265032	0.322411	0.096927	0.393182	0.224682	0.357796
-0.25411	-0.29625	-0.54322	-0.38988	-0.05491	0.338949	-0.22186	0.20613	-0.13838
-0.00981	0.025364	NA	NA	0.086871	0.07723	-0.01056	0.131341	0.038155
-0.13132	0.092179	-0.3043	0.138712	0.169622	0.375403	0.024883	0.352433	0.097253
-0.0374	0.07419	-0.24112	-0.26209	-0.10376	0.147041	-0.11717	0.094837	-0.11046
0.125189	0.01916	0.336969	0.184634	0.202178	0.22465	0.293731	0.132545	0.247955
0.086466	0.207428	0.134569	0.500031	0.147616	0.273603	0.178637	0.08575	0.163127
-0.05171	0.028869	NA	NA	-0.11239	0.199771	0.022735	0.105288	-0.04483
0.012928	0.094715	-0.44819	0.350194	-0.03695	0.336759	0.003094	0.268109	-0.01693
-0.13202	0.223002	-0.13391	0.382339	0.030231	0.337576	0.004922	0.196471	0.017576
0.300555	0.351787	0.64642	0.438493	0.188122	0.2079	0.292899	0.240245	0.240511
0.177992	0.251809	-0.00824	0.003087	0.048003	0.217479	0.140627	0.143455	0.094315
0.091607	0.463336	0.529132	0.496559	0.318156	0.175705	0.159167	0.336289	0.238662
-0.04864	0.093675	-0.00755	-0.03353	0.110089	0.276234	-0.00323	0.110489	0.053427
0.12602	0.087354	0.636087	0.433575	0.193891	0.240058	0.123491	0.316602	0.158691
0.248363	-0.18107	0.571001	-0.24145	-0.17398	0.230639	0.115431	0.350968	-0.02928
-0.12965	0.104077	-0.18458	0.353103	0.012091	0.224666	-0.09207	0.112469	-0.03999
0.13967	0.177295	0.364794	0.303302	0.062747	0.212761	0.082125	0.216861	0.072436
-0.19823	0.18917	NA	NA	-0.08255	0.173093	0.030881	0.152452	-0.02583
-0.13898	0.190908	-0.46049	0.530525	0.183626	0.315289	-0.14078	0.192193	0.021422
-0.02864	0.108435	-0.22231	-0.13975	0.016663	0.103137	0.017266	0.141889	0.016964
0.052934	0.07091	-0.71024	-0.32953	-0.0663	0.150694	-0.09526	0.354891	-0.08078
0.100472	0.115442	-0.28917	-0.16865	0.02933	0.201994	-0.08613	0.141389	-0.0284
0.465091	0.262555	0.533208	0.41043	0.256437	0.142219	0.431356	0.150146	0.343897
0.342347	0.069532	0.655058	0.440644	0.168741	0.189527	0.432656	0.127611	0.300699
-0.17151	-0.38163	0.001398	-0.47622	-0.10426	0.307652	-0.10877	0.116261	-0.10651
NA	NA	NA	NA	0.064184	0.138468	-0.14485	0.328698	-0.04033
0.464086	0.224237	0.724708	0.40349	0.274335	0.174141	0.469052	0.170264	0.371693
0.269122	0.148647	0.465636	-0.0298	0.175674	0.218668	0.228332	0.205327	0.202003
-0.17785	-0.28634	-0.54995	-0.49835	-0.46223	0.12436	-0.32665	0.233378	-0.39444
-0.04654	-0.09466	0.443589	-0.29865	-0.19961	0.191361	0.027323	0.230992	-0.08615
0.009153	-0.07829	0.007514	0.327595	0.026365	0.204316	-0.00689	0.13696	0.009736
-0.10126	0.158087	-0.6303	0.474543	-0.10841	0.412189	-0.27725	0.350411	-0.19283
-0.3333	-0.32955	-0.06405	-0.58079	-0.20199	0.265926	-0.16767	0.16912	-0.18483
-0.00167	-0.33406	0.325733	-0.48656	-0.23731	0.243765	-0.00116	0.165304	-0.11924
-0.20295	0.232634	-0.67204	0.276807	0.186284	0.18508	-0.03083	0.419903	0.077728
-0.08535	-0.19671	-0.49003	-0.61253	-0.20885	0.205461	-0.09978	0.225187	-0.15432
-0.22776	0.154871	-0.62236	0.267241	0.067191	0.248441	-0.18255	0.249215	-0.05768
-0.34859	0.075705	-0.60275	0.134893	0.011684	0.157917	-0.18695	0.246233	-0.08763
0.207776	0.117522	0.403341	-0.2926	0.05545	0.184152	0.149443	0.202948	0.102446
0.155526	0.06195	0.047445	-0.09097	0.063188	0.100058	0.135276	0.134587	0.099232
0.109477	0.175745	-0.10003	-0.26158	0.153166	0.393034	-0.09886	0.121379	0.027155
0.035425	0.136005	0.077462	-0.05814	0.024001	0.248801	0.017437	0.188554	0.020719
0.232716	0.253142	0.208758	0.071851	0.264833	0.279727	0.240868	0.158179	0.252851
0.068696	-0.37201	-0.16479	-0.55381	-0.13653	0.356069	0.023084	0.207502	-0.05673
-0.11129	0.015633	0.234952	0.185991	-0.1124	0.245107	-0.09072	0.229462	-0.10156
0.280402	0.230971	0.202275	0.192325	0.044351	0.215055	0.14852	0.316866	0.096436
-0.0751	0.056448	0.015491	0.2093	0.126722	0.293139	-0.07088	0.152971	0.027923
-0.06691	0.077572	0.058298	0.56917	0.022185	0.311534	-0.03946	0.138069	-0.00864
-0.42154	0.124779	-0.68796	-0.12501	0.152854	0.216911	-0.20713	0.408529	-0.02714
-0.11725	0.012511	-0.69338	-0.23196	-0.04853	0.110587	-0.0264	0.366024	-0.03746

0.176932	0.204612	-0.15118	0.515564	0.129451	0.359116	0.13496	0.210737	0.132206
-0.00598	-0.21501	0.016568	-0.28375	-0.10162	0.199114	0.068337	0.079282	-0.01664
0.179702	0.112646	0.36775	0.46158	0.132561	0.283948	0.222666	0.135212	0.177613
0.169806	-0.00737	-0.09438	-0.348	-0.06758	0.242827	0.060479	0.17314	-0.00355
-0.11855	0.112447	-0.77219	0.39143	0.075629	0.277105	-0.14429	0.375979	-0.03433
0.031471	0.222922	-0.23387	0.026324	0.029187	0.160374	-0.01821	0.244185	0.005486
0.127961	0.062955	0.6266	-0.33522	0.028058	0.233375	0.153722	0.24586	0.09089
-0.08619	0.185076	0.20633	0.229249	0.137937	0.091714	0.019088	0.220956	0.078512
-0.05019	-0.29247	0.081574	-0.62647	-0.02158	0.443117	0.075806	0.147116	0.027113
0.031587	0.375512	0.215632	0.537802	0.312145	0.208543	0.092002	0.150975	0.202073
0.040478	0.162983	-0.34618	0.130441	0.028121	0.25349	-0.18433	0.279325	-0.07811
0.335399	0.126977	0.454307	0.242173	-0.08072	0.288788	0.176832	0.206758	0.048055
0.180466	-0.15137	0.004284	-0.64001	-0.24943	0.258003	0.036982	0.084211	-0.10622
0.228331	0.309919	0.214851	0.171529	0.163808	0.226984	0.116196	0.203071	0.140002
0.089673	-0.16397	0.402912	-0.28695	-0.18421	0.195367	0.155437	0.165427	-0.01439
-0.11746	0.039985	-0.10377	0.460676	0.06963	0.350076	-0.20777	0.164405	-0.06907
-0.08271	-0.30432	-0.60343	-0.58112	-0.15173	0.343596	-0.16408	0.29712	-0.15791
-0.03839	0.049335	0.329697	-0.25792	0.087955	0.341771	0.066009	0.220799	0.076982
-0.08129	-0.39089	0.221742	-0.56077	-0.0552	0.455668	-0.05134	0.267695	-0.05327
0.132278	0.164497	0.324864	0.144061	0.081075	0.377949	0.030589	0.268939	0.055832
-0.15759	-0.08044	0.091556	-0.16657	-0.1217	0.220205	-0.22259	0.202023	-0.17215
-0.16408	0.600263	-0.24558	0.532648	0.36886	0.387405	-0.15205	0.170682	0.108404
0.037086	0.101313	-0.58653	-0.01613	-0.13811	0.152391	-0.11772	0.245893	-0.12791
-0.28353	0.002516	-0.46254	-0.23849	0.072301	0.202041	-0.16574	0.196788	-0.04672
-0.23048	-0.39707	-0.24316	-0.50204	-0.3108	0.180753	-0.05985	0.21331	-0.18533
-0.04465	-0.42244	0.001864	-0.0015	-0.10025	0.204523	-0.02297	0.223551	-0.06161
-0.06614	0.092158	0.029558	-0.30579	-0.08082	0.369297	-0.08273	0.165751	-0.08177
-0.2745	-0.11041	-0.05709	-0.22401	-0.07075	0.113672	-0.14455	0.170175	-0.10765
0.179392	0.181977	NA	NA	0.068957	0.086913	0.025352	0.262733	0.047154
NA	NA	-0.23069	-0.15875	-0.12133	0.091125	-0.04633	0.211106	-0.08383
-0.05315	-0.13355	-0.29324	-0.04114	-0.04898	0.074784	-0.13589	0.107797	-0.09244
0.332872	0.207676	-0.38007	0.49803	0.182057	0.223349	0.031607	0.245699	0.106832
0.000522	0.144526	-0.47375	-0.30258	0.11728	0.301191	0.040728	0.274343	0.079004
-0.11555	-0.01177	-0.01492	-0.40117	0.060305	0.388467	0.019738	0.2913	0.040021
-0.1086	-0.44012	-0.47643	-0.54733	-0.23619	0.222337	-0.14769	0.256395	-0.19194
0.037112	-0.25611	0.035261	-0.36457	-0.13672	0.213	0.070412	0.167631	-0.03316
0.283185	0.358643	0.233195	0.50181	0.247394	0.171077	0.290403	0.08512	0.268899
0.109855	0.348226	-0.0202	0.307367	0.162151	0.203134	0.098764	0.185195	0.130457
0.038901	0.290283	-0.55424	0.036472	0.138919	0.135274	0.011384	0.324686	0.075151
-0.06221	0.311551	-0.21657	0.272198	0.028826	0.28506	-0.08274	0.314087	-0.02696
0.212098	0.011466	0.399545	-0.06866	-0.11872	0.101944	0.211604	0.264833	0.046443
0.112995	0.094815	0.631409	0.005894	0.095581	0.35384	0.169719	0.350408	0.13265
0.036265	0.00224	NA	NA	-0.03583	0.121643	-0.02119	0.127734	-0.02851
-0.34911	0.702682	-0.2142	0.787861	0.52785	0.338892	-0.16383	0.155982	0.182008
0.203116	0.323126	0.467154	0.110697	0.188353	0.220571	0.255721	0.14644	0.222037
0.322252	0.263534	-0.05446	0.109913	0.269057	0.226337	0.267663	0.186053	0.26836
0.058462	0.10469	0.38789	-0.11901	0.165175	0.301316	0.211397	0.190701	0.188286
0.141889	0.125489	-0.3962	0.520015	0.119981	0.274979	-0.00785	0.203739	0.056063
0.031733	0.199896	0.006559	-0.26556	0.024091	0.317013	-0.10992	0.200904	-0.04291
-0.09284	0.053293	-0.02846	0.001758	-0.03987	0.171769	-0.02989	0.132266	-0.03488
-0.42726	0.120548	-0.64236	0.276435	0.139202	0.174381	-0.21936	0.322088	-0.04008
-0.15904	0.44639	-0.13425	0.500865	0.396355	0.300819	-0.1368	0.214565	0.129777

0.173357	0.15037	NA	NA	0.159501	0.135214	0.083137	0.306995	0.121319
0.212241	0.380183	NA	NA	0.297436	0.087429	0.359954	0.138641	0.328695
NA	NA	NA	NA	0.057157	0.519595	-0.00098	0.395102	0.028088
0.318435	0.365436	NA	NA	0.389012	0.218811	0.31319	0.159302	0.351101
0.073559	0.084498	NA	NA	-0.18521	0.238804	-0.00979	0.18849	-0.0975
0.107399	0.131395	NA	NA	0.056228	0.211667	0.334572	0.233978	0.1954
0.06434	0.684946	0.241853	0.654395	0.460332	0.237414	0.010338	0.166957	0.235335
-0.19269	0.199816	-0.03648	0.348263	0.079266	0.23306	0.039862	0.247387	0.059564
-0.09323	0.040043	0.166997	0.361403	0.214056	0.16962	0.060348	0.166076	0.137202
0.013405	0.437528	0.164591	0.318556	0.201611	0.217643	0.050609	0.122669	0.12611
-0.34948	0.330564	0.11331	0.367365	0.113798	0.235742	-0.09705	0.197361	0.008373
0.214339	0.072635	0.445607	-0.25082	0.154258	0.283747	0.320726	0.177543	0.237492
0.117347	0.283313	-0.17383	0.233953	0.304778	0.157732	0.047011	0.159312	0.175895
0.0001	-0.17332	-0.35985	-0.17429	-0.17694	0.105046	-0.05297	0.254496	-0.11495
0.148858	0.3283	0.328426	0.337095	0.21581	0.294735	0.115789	0.285658	0.165799
-0.01597	-0.06511	0.062659	-0.25434	-0.19312	0.147646	-0.11607	0.131491	-0.1546
0.006752	-0.20443	-0.38137	-0.38285	-0.07369	0.220354	0.025198	0.312602	-0.02425
0.0357	-0.27854	0.293831	-0.34552	-0.29991	0.129183	0.17132	0.1431	-0.06429
-0.19797	-0.29069	-0.62518	-0.27448	-0.28541	0.232558	-0.23474	0.29958	-0.26008
0.165429	-0.17462	0.365724	-0.31085	-0.22133	0.254007	0.219571	0.193932	-0.00088
-0.3075	-0.39628	-0.62282	-0.56098	-0.09495	0.345124	-0.17459	0.267816	-0.13477
0.305715	-0.13891	0.24577	-0.30464	-0.12313	0.212091	0.206025	0.130803	0.04145
-0.34391	-0.21092	-0.3659	-0.33077	-0.3202	0.125232	-0.23388	0.125244	-0.27704
-0.02629	-0.21952	-0.14621	-0.34918	-0.19866	0.149588	-0.04699	0.189361	-0.12283
-0.12332	-0.26374	-0.25397	-0.38459	-0.18578	0.330315	-0.09289	0.186161	-0.13933
0.205512	0.177391	-0.21531	-0.58489	0.062199	0.374916	0.141584	0.241603	0.101892
0.088153	0.111724	0.626779	0.525729	0.145236	0.437377	0.128385	0.298758	0.13681
-0.02311	-0.37522	-0.456	-0.59865	-0.26111	0.245947	-0.23738	0.152722	-0.24925
0.479934	0.376143	0.70602	0.304362	0.224222	0.16208	0.334371	0.230439	0.279296
0.236622	0.24782	-0.05217	0.296718	0.116009	0.173577	0.088103	0.220766	0.102056
-0.05356	0.210157	-0.65128	-0.01585	-0.09122	0.264534	-0.12419	0.308405	-0.10771
-0.36572	0.147781	-0.53417	-0.07181	-0.00985	0.130735	-0.21813	0.212033	-0.11399
-0.05529	-0.27288	-0.23757	-0.49308	-0.14109	0.30177	-0.17346	0.277017	-0.15727
-0.04621	-0.22206	-0.17059	0.18612	-0.0126	0.184372	0.163551	0.301958	0.075476
0.205486	0.444678	0.545861	0.625213	0.324129	0.253325	0.258298	0.163529	0.291213
-0.22527	-0.20018	0.094099	-0.33467	-0.15069	0.183394	-0.13696	0.208595	-0.14382
-0.23548	0.200337	-0.64212	-0.15831	0.012373	0.272846	-0.11363	0.47602	-0.05063
0.126017	-0.06305	0.190322	-0.16743	-0.16317	0.209008	0.023321	0.187668	-0.06993
-0.15954	0.102239	0.078614	-0.36988	-0.15189	0.217916	-0.11117	0.150959	-0.13153
0.052046	-0.37386	-0.43403	-0.55204	-0.2751	0.202766	-0.15817	0.206546	-0.21663
-0.01628	0.076218	0.001202	0.27749	0.101418	0.129602	0.018441	0.131535	0.05993
0.138898	0.100625	-0.36085	0.295441	-0.06034	0.270064	-0.01932	0.286042	-0.03983
0.098363	0.130314	0.16894	-0.05105	0.102587	0.22077	0.096679	0.163668	0.099633
-0.23705	0.121266	-0.25144	0.245612	0.002474	0.200817	-0.14004	0.17689	-0.06878
0.19179	0.292275	0.641203	-0.09455	0.160832	0.309201	0.285955	0.222607	0.223393
0.32623	0.639087	0.520042	0.707763	0.381853	0.289139	0.208694	0.224175	0.295273
-0.35674	0.256401	-0.72286	0.012929	0.09019	0.273755	-0.21759	0.30357	-0.0637
-0.2356	0.503796	0.115349	0.633634	0.132082	0.476549	-0.20994	0.257106	-0.03893
0.577932	0.242953	0.808408	0.418308	0.086597	0.330039	0.449056	0.244913	0.267826
0.153106	0.153193	-0.00087	0.438369	0.004836	0.344472	0.049381	0.192596	0.027108
-0.19867	0.128569	-0.65291	0.224435	0.00467	0.154773	-0.14692	0.290599	-0.07112
0.079116	0.399113	0.138567	0.251009	0.159025	0.201407	0.064458	0.185601	0.111741

0.039773	0.162235	-0.32591	0.081335	-0.02866	0.210778	-0.01386	0.284904	-0.02126
-0.16985	0.299991	-0.24534	0.397621	0.081413	0.300764	-0.09389	0.24922	-0.00624
-0.17172	0.288785	-0.15035	0.359212	0.087449	0.289824	0.008622	0.213773	0.048035
-0.24096	-0.44425	-0.42592	-0.43527	-0.32035	0.111528	-0.25875	0.282159	-0.28955
0.142719	0.24782	-0.06121	0.27445	0.255813	0.130686	0.044087	0.23734	0.14995
-0.15548	-0.06904	0.004859	-0.09405	0.074991	0.316101	-0.19018	0.278324	-0.05759
-0.123	-0.03562	0.105092	-0.19833	-0.12657	0.27863	-0.17743	0.25743	-0.152
-0.0123	0.494626	0.531472	0.0062	0.247235	0.338055	0.123049	0.263175	0.185142
0.303971	-0.19994	-0.04034	-0.14147	-0.01061	0.318035	0.034142	0.242007	0.011765
0.378194	0.04695	0.404014	0.080259	-0.03708	0.140686	0.326233	0.138418	0.144578
0.171154	-0.04797	0.306973	-0.17456	-0.01973	0.275798	0.136213	0.223584	0.058242
-0.03088	-0.14183	0.157554	-0.29257	-0.03555	0.217612	0.088378	0.103029	0.026412
-0.09029	-0.16581	0.216942	-0.147	-0.132	0.2384	0.041936	0.258745	-0.04503
-0.21529	0.049678	-0.2053	0.598941	0.033187	0.366204	-0.07817	0.213631	-0.02249
-0.04021	-0.06662	-0.21255	-0.54033	-0.18712	0.237655	-0.09717	0.098979	-0.14215
0.375078	0.033912	0.385592	-0.21228	-0.04392	0.303728	0.224741	0.202041	0.090411
0.256013	0.029539	-0.02582	-0.22482	0.045149	0.305699	0.116793	0.17778	0.080971
0.068065	0.197001	NA	NA	0.107403	0.32608	0.144012	0.045164	0.125707
0.068498	0.166571	-0.03505	0.248602	0.303598	0.175951	0.208255	0.219053	0.255927
0.351411	0.03554	0.391532	0.149371	0.262762	0.162031	0.35064	0.235214	0.306701
0.220792	0.380597	-0.20668	0.464679	0.24766	0.195595	0.13587	0.233172	0.191765
0.140779	-0.02108	-0.45258	-0.03189	-0.10729	0.153097	-0.08398	0.263708	-0.09563
-0.12853	0.03692	-0.44441	0.233797	0.031692	0.199616	-0.16304	0.194864	-0.06567
0.000638	-0.04207	-0.50107	0.373752	0.008954	0.218342	-0.10522	0.24087	-0.04813
-0.02534	-0.05819	0.123574	-0.20132	0.078485	0.327591	0.036032	0.104403	0.057259
0.061615	-0.14314	-0.15597	-0.1361	0.040339	0.287714	0.032064	0.201442	0.036202
0.102514	-0.27156	-0.09297	0.071537	-0.29744	0.252834	0.061042	0.231363	-0.1182
-0.1257	-0.23125	-0.3491	0.507233	0.128521	0.34163	-0.23739	0.154815	-0.05444
0.059005	0.082022	NA	NA	0.076175	0.188518	0.180464	0.108815	0.12832
0.123941	0.364672	0.010808	0.119165	0.131387	0.199041	-0.05664	0.278218	0.037373
0.023095	0.348127	-0.6766	0.501514	0.243889	0.245114	-0.16504	0.347347	0.039423
0.028974	-0.2697	-0.38665	-0.28299	-0.06705	0.249977	-0.172	0.211985	-0.11953
-0.24653	-0.30491	-0.20835	-0.65302	-0.18317	0.377768	-0.18765	0.163372	-0.18541
0.412632	-0.00715	0.009681	-0.4602	-0.1932	0.197311	0.282575	0.185167	0.044686
-0.04685	0.027071	-0.25359	-0.48315	0.035906	0.358243	-0.17887	0.208729	-0.07148
-0.23446	0.164072	-0.73336	-0.00673	-0.08789	0.304532	-0.26353	0.267797	-0.17571
0.025056	-0.16069	0.25621	0.497633	0.152062	0.379893	0.166191	0.11014	0.159127
-0.00678	0.037064	-0.61218	-0.07947	-0.1049	0.160541	-0.02831	0.340895	-0.0666
0.241608	-0.11366	0.382696	-0.0593	-0.19612	0.293649	0.124495	0.191534	-0.03581
0.039989	0.065303	-0.13118	-0.19444	-0.07044	0.175126	0.00046	0.166041	-0.03499
0.080603	0.115169	-0.32414	0.133265	0.034947	0.287198	-0.0369	0.260489	-0.00098
-0.09605	-0.35165	-0.41668	-0.55107	-0.28372	0.248034	-0.15619	0.167562	-0.21995
0.049174	0.058906	-0.05453	0.121061	0.231156	0.156715	-0.08147	0.112647	0.074844
-0.05316	-0.3063	-0.39824	-0.2894	-0.08262	0.248657	-0.22324	0.168771	-0.15293
-0.00024	-0.113	-0.59807	-0.31326	-0.03894	0.22057	-0.20068	0.273233	-0.11981
0.236865	-0.10043	0.27724	-0.28913	-0.20165	0.07177	0.125762	0.114484	-0.03794
0.199956	-0.05026	0.568356	-0.2362	-0.23645	0.145024	0.11137	0.285823	-0.06254
-0.17793	0.541609	0.115562	0.530458	0.27536	0.414518	-0.07498	0.153058	0.100192
-0.05943	-0.31658	-0.40599	-0.55673	-0.09249	0.288712	-0.07143	0.219929	-0.08196
-0.06239	-0.24385	0.442666	-0.07332	-0.1185	0.129212	0.040395	0.230914	-0.03905
-0.02245	-0.03673	-0.18245	0.225269	0.012052	0.144283	-0.00025	0.153283	0.005903
0.093407	-0.31251	0.216069	-0.55507	-0.24834	0.166807	0.038788	0.199958	-0.10478

-0.05795	-0.1931	-0.10781	-0.17364	0.006739	0.235218	-0.0588	0.071604	-0.02603
0.05014	0.004395	NA	NA	0.065606	0.07412	-0.03983	0.095413	0.012888
0.074032	-0.20321	0.592418	0.038671	0.052843	0.2464	0.228401	0.244735	0.140622
-0.29238	-0.0355	-0.37915	0.426613	0.118144	0.229544	-0.19576	0.205019	-0.03881
0.032846	-0.14835	-0.28411	-0.4037	-0.17164	0.175313	-0.00286	0.193116	-0.08725
-0.07537	-0.37001	-0.2086	-0.47212	-0.24322	0.165337	-0.09223	0.125977	-0.16773
-0.09433	-0.15305	0.011981	-0.52816	-0.13603	0.302996	-0.09217	0.089079	-0.1141
-0.02367	-0.19953	0.489183	0.029101	0.08534	0.239149	0.154786	0.184479	0.120063
-0.12051	-0.35815	0.30267	-0.18141	-0.03696	0.221777	-0.00846	0.163029	-0.02271
0.270457	0.377535	0.657683	0.44715	0.088947	0.287013	0.128723	0.322949	0.108835
-0.25092	0.379466	0.102677	0.511153	0.143906	0.246938	-0.05808	0.205714	0.042913
0.487944	0.145075	0.626148	-0.07306	0.165525	0.173321	0.419525	0.142716	0.292525
-0.24509	0.456541	-0.56579	0.559518	0.189032	0.289112	-0.29342	0.238984	-0.05219
-0.04714	0.242379	-0.17772	0.339265	0.150881	0.185065	-0.0647	0.202192	0.043089
-0.43235	0.072487	-0.6877	0.316195	-0.04073	0.20068	-0.46416	0.156055	-0.25245
-0.44981	0.187209	-0.09592	0.293631	0.189199	0.141216	-0.24188	0.197333	-0.02634
0.012149	0.298043	0.618794	0.378454	0.15357	0.216805	0.15081	0.277009	0.15219
0.053082	0.114307	-0.25476	0.013226	0.05401	0.116764	0.001675	0.26333	0.027843
0.095416	0.019538	0.597164	-0.20675	-0.01348	0.213378	0.21576	0.243557	0.101139
-0.16766	0.232096	NA	NA	0.286885	0.274896	0.2348	0.31886	0.260842
0.349372	-0.18256	0.542259	0.126354	-0.0782	0.379966	0.416358	0.122472	0.169079
0.33344	0.037403	0.395163	-0.08261	0.034992	0.095552	0.30432	0.123896	0.169656
-0.11492	0.077295	NA	NA	-0.09464	0.379239	0.04924	0.145044	-0.0227
-0.04339	0.719408	-0.44253	0.835113	0.581966	0.292447	-0.14926	0.207886	0.216352
0.167597	0.539505	0.407788	0.38631	0.274864	0.196902	0.188803	0.2671	0.231834
0.088852	0.003685	0.045125	-0.13803	0.061298	0.322338	0.013092	0.26978	0.037195
-0.44872	0.498297	-0.49192	0.428571	0.109428	0.390305	-0.29054	0.195013	-0.09056
-0.01178	0.132243	-0.3359	0.286119	-0.05961	0.353453	-0.14527	0.200471	-0.10244
-0.06167	0.221124	-0.44149	0.124808	0.093468	0.075234	-0.13107	0.165911	-0.0188
0.049879	0.147473	0.312714	0.005424	0.173491	0.133814	0.200177	0.119765	0.186834
-0.30107	-0.26836	-0.09551	-0.14853	-0.12973	0.271733	-0.23686	0.334596	-0.18329
-0.11243	0.027932	-0.22879	0.185701	-0.08864	0.295314	-0.18868	0.2114	-0.13866
-0.22221	-0.42747	-0.01933	-0.18525	-0.3798	0.154743	-0.2678	0.170992	-0.3238
0.148117	0.246885	-0.00593	0.2222	0.253636	0.116268	0.071558	0.148677	0.162597
0.099383	-0.33984	0.242465	-0.33822	-0.18926	0.176608	0.018681	0.209435	-0.08529
0.012205	0.077518	0.036225	-0.08467	-0.16393	0.26581	-0.01471	0.203219	-0.08932
0.0628	0.22398	-0.09679	0.520015	0.08787	0.276728	0.015365	0.211316	0.051618
0.032868	0.004588	NA	NA	0.192661	0.309614	0.084813	0.232789	0.138737
-0.37295	0.222075	-0.5999	0.521113	0.173169	0.195967	-0.26641	0.195667	-0.04662
-0.21802	0.222854	-0.48814	0.039418	0.013205	0.224727	-0.26233	0.230048	-0.12456
-0.11837	-0.25942	-0.78765	-0.01122	-0.29025	0.165518	-0.22807	0.357601	-0.25916
0.310656	0.295557	0.569142	-0.20476	0.08305	0.171906	0.305005	0.255292	0.194027
0.067129	0.223207	-0.3673	0.334881	0.032436	0.23609	-0.054	0.191732	-0.01078
-0.18204	-0.05724	-0.62725	-0.02214	-0.0439	0.108401	-0.32614	0.179959	-0.18502
0.152276	0.220579	0.402593	0.273447	0.219847	0.289603	0.17955	0.189047	0.199698
-0.16288	-0.1995	0.399308	0.293852	0.024807	0.284297	0.095137	0.210368	0.059972
-0.17865	0.057126	-0.17315	-0.07201	-0.17398	0.227839	-0.18711	0.323759	-0.18055
0.03404	0.042021	0.107664	-0.56245	0.023676	0.409395	0.070124	0.176931	0.0469
-0.04677	-0.43288	-0.23574	-0.47454	-0.36027	0.150407	-0.07464	0.108857	-0.21746
-0.25596	-0.21588	-0.16997	-0.66706	-0.14418	0.36525	-0.1024	0.131048	-0.12329
0.082042	-0.39515	-0.32462	-0.24666	-0.40894	0.198474	-0.08725	0.144036	-0.2481
-0.04309	0.193893	0.163936	0.224922	0.221826	0.020362	0.074053	0.148695	0.14794

-0.20298	0.100625	-0.59146	-0.18425	-0.00093	0.135713	-0.3132	0.168794	-0.15706
0.079586	-0.08599	-0.14947	-0.28531	0.115334	0.353495	-0.03647	0.166893	0.03943
-0.21729	-0.40822	-0.27321	-0.53128	-0.24629	0.228752	-0.17867	0.180883	-0.21248
-0.08941	0.078724	-0.23432	-0.09506	-0.03171	0.211049	-0.02726	0.145853	-0.02948
-0.10961	-0.2439	-0.15859	-0.16952	-0.12368	0.289772	-0.08059	0.148877	-0.10214
0.078403	-0.34026	0.214601	-0.26039	-0.12467	0.164578	-0.03158	0.221262	-0.07812
-0.26671	-0.33088	-0.45988	0.079576	-0.25821	0.238683	-0.34324	0.178041	-0.30073
-0.08903	0.197576	-0.02417	0.385121	0.16165	0.183984	-0.12955	0.095329	0.016048
-0.15047	-0.37015	-0.18241	-0.49916	-0.15591	0.229899	-0.06928	0.205542	-0.1126
-0.00386	-0.2649	0.157163	-0.47554	-0.11573	0.244176	0.032693	0.255205	-0.04152
-0.10677	-0.20154	-0.13776	-0.11903	-0.19515	0.321895	-0.11543	0.172793	-0.15529
NA	NA	0.436789	0.468571	0.032528	0.423404	0.125855	0.325342	0.079191
-0.15584	0.107613	-0.0459	-0.17202	-0.07719	0.297794	-0.05947	0.21845	-0.06833
0.032996	0.127937	0.429487	0.068831	0.158097	0.355529	0.178101	0.323334	0.168099
NA	NA	0.748763	0.024094	0.153254	0.28596	0.328463	0.252234	0.240859
-0.02091	0.191956	NA	NA	0.319111	0.257721	0.027936	0.195855	0.173524
-0.14626	-0.37037	-0.03806	-0.28333	-0.29953	0.267177	-0.20008	0.181029	-0.24981
0.09984	-0.02548	0.292133	0.179835	0.047615	0.253476	0.15639	0.20005	0.102002
0.152104	0.110703	-0.13161	-0.22429	0.112531	0.291959	0.082197	0.252899	0.097364
0.095048	0.081175	NA	NA	0.043294	0.199036	0.091881	0.088426	0.067587
-0.03363	0.152407	NA	NA	0.028032	0.254191	-0.03609	0.239741	-0.00403
0.023467	0.133139	NA	NA	-0.05397	0.381134	0.102707	0.14636	0.024369
-0.09409	0.291774	0.005227	0.299113	0.159721	0.181709	0.00868	0.106646	0.0842
-0.09137	-0.08282	-0.40433	-0.00038	0.003102	0.297523	-0.11705	0.259571	-0.05698
-0.01895	0.051554	0.248566	0.03969	-0.09816	0.21804	0.082283	0.155936	-0.00794
0.031024	0.039983	-0.12606	-0.16821	-0.04133	0.223452	0.074814	0.202258	0.01674
0.126637	0.260188	0.547784	0.236384	0.151917	0.308365	0.166843	0.233521	0.15938
0.227515	0.104013	0.508909	0.520476	0.064439	0.252509	0.201668	0.16526	0.133053
0.262779	0.023364	0.302657	0.3068	0.003183	0.190286	0.040513	0.296147	0.021848
0.014151	0.132421	-0.24556	-0.34719	-0.07347	0.365637	-0.01726	0.360686	-0.04536
-0.17577	0.296383	-0.00223	0.079279	0.211799	0.197941	-0.08517	0.303372	0.063313
0.002759	-0.20627	0.344527	-0.30146	-0.04649	0.287348	0.133961	0.148223	0.043736
-0.04365	0.290707	0.575488	0.355454	0.185145	0.161502	0.185564	0.340298	0.185354
0.322227	-0.00204	0.597721	-0.29514	-0.21529	0.290981	0.158137	0.26421	-0.02858
-0.21077	0.141417	0.129039	0.198783	0.142799	0.044548	0.081419	0.226627	0.112109
0.269809	0.06144	-0.1661	-0.10263	-0.12441	0.299955	-0.01434	0.16908	-0.06938
0.189399	0.09208	0.700928	-0.27964	0.196135	0.276758	0.254529	0.284184	0.225332
-0.22074	-0.04629	NA	NA	0.167494	0.327861	-0.03653	0.199943	0.065483
0.426827	0.044194	0.456349	0.129871	0.0331	0.226092	0.351688	0.174983	0.192394
-0.10479	0.23915	-0.1089	-0.17795	0.112349	0.219986	0.031853	0.170475	0.072101
0.021375	-0.05792	0.057516	-0.54944	-0.07461	0.29713	0.214399	0.238142	0.069895
0.29155	-0.11715	0.505432	0.107961	-0.15507	0.156041	0.205353	0.199453	0.025143
0.02843	0.35213	0.108655	0.266556	0.134302	0.169721	0.025472	0.128986	0.079887
-0.04487	0.204485	0.420326	0.290505	0.036731	0.25889	0.075234	0.217369	0.055982
0.139723	0.406706	0.323588	0.507321	0.226827	0.217998	0.206691	0.077743	0.216759
-0.29608	-0.33454	-0.01295	-0.61071	-0.34187	0.212212	-0.17293	0.236902	-0.2574
-0.0416	0.203187	0.076833	0.249057	-0.07606	0.284539	0.003289	0.185283	-0.03639
-0.08584	0.090864	-0.5373	-0.08189	-0.20202	0.231324	-0.20551	0.195957	-0.20377
0.0952	0.308697	0.313434	0.212285	0.239367	0.315578	0.159351	0.204479	0.199359
-0.114	0.15501	-0.60375	0.305914	0.028887	0.228543	-0.18899	0.302133	-0.08005
-0.12324	0.297228	NA	NA	0.064693	0.314837	-0.11557	0.207941	-0.02544
0.411062	-0.06234	-0.15284	-0.47058	0.086094	0.422235	0.073688	0.229744	0.079891

0.156902	0.048561	0.693929	0.156827	-0.09248	0.176207	0.166268	0.30297	0.036892
NA	NA	NA	NA	0.187746	0.23118	0.135309	0.229027	0.161528
NA	NA	NA	NA	0.163265	0.179288	-0.07621	0.284906	0.043526
-0.16826	-0.01411	0.244175	-0.00188	-0.14208	0.181691	-0.05194	0.148533	-0.09701
NA	NA	NA	NA	-0.23553	0.305773	-0.20062	0.177193	-0.21808
0.093098	0.000257	-0.04068	0.054711	0.068404	0.194429	-0.04795	0.1459	0.010228
0.088487	0.117908	-0.06599	-0.11014	-0.02029	0.409665	0.025182	0.151441	0.002448
0.109293	0.188588	-0.49014	0.405334	0.07038	0.208137	-0.10373	0.341918	-0.01668
0.015836	0.178866	0.030977	0.41119	0.163628	0.255822	0.146583	0.138089	0.155106
-0.14332	-0.03052	-0.35624	-0.55812	-0.04892	0.300136	0.01663	0.289264	-0.01614
0.234694	0.04581	NA	NA	0.137411	0.164382	-0.0128	0.212393	0.062307
0.044	0.311205	0.229752	0.160898	0.074464	0.312412	0.076249	0.134499	0.075356
-0.25966	-0.07932	-0.35149	-0.18832	-0.05431	0.230093	-0.20245	0.300263	-0.12838
-0.23649	0.059256	0.03906	0.307974	0.142766	0.223059	-0.21317	0.193781	-0.0352
-0.2157	0.112275	-0.3707	-0.22587	-0.00223	0.345328	-0.19417	0.159405	-0.0982
-0.01057	-0.06976	-0.25122	-0.44909	-0.21778	0.198598	-0.0353	0.143466	-0.12654
-0.20284	-0.25733	-0.44425	-0.4734	-0.43108	0.15814	-0.2345	0.124837	-0.33279
0.235796	0.138489	0.222353	-0.22117	0.342609	0.325136	0.197911	0.067254	0.27026
0.190708	0.188705	NA	NA	0.213319	0.347563	-0.00879	0.272831	0.102264
0.289196	0.0877	0.542401	0.376621	-0.11711	0.345358	0.232382	0.275595	0.057636
0.089655	0.13205	0.37515	0.181882	0.195379	0.188414	0.156512	0.13304	0.175945
-0.13401	0.10629	-0.56198	0.128587	-0.23139	0.279684	-0.2945	0.20098	-0.26295
-0.2038	0.009361	-0.69552	-0.38024	-0.20755	0.168868	-0.21492	0.299044	-0.21123
-0.30929	-0.2232	-0.74742	-0.13241	-0.09998	0.225671	-0.3023	0.245892	-0.20114
0.051923	-0.01133	-0.50915	0.323389	0.023463	0.159248	-0.12626	0.339492	-0.0514
0.228596	0.040133	-0.55704	0.532941	0.170595	0.33705	0.042955	0.386297	0.106775
-0.15657	-0.47955	-0.5079	-0.42924	-0.34412	0.112814	-0.14363	0.197347	-0.24387
-0.19629	-0.0509	-0.81116	0.098194	0.072666	0.267277	-0.02652	0.435732	0.023075
0.199208	-0.16447	0.262302	-0.38444	-0.30359	0.227686	0.138074	0.157679	-0.08276
0.019367	-0.26036	0.283572	-0.60588	-0.29075	0.222229	0.168556	0.124743	-0.0611
-0.16883	0.256379	-0.62228	0.33831	0.01444	0.301001	-0.27184	0.19184	-0.1287
-0.13606	-0.2263	-0.46009	0.35178	0.046818	0.27246	-0.01368	0.265722	0.016568
-0.1075	-0.11943	0.32765	-0.35102	-0.34832	0.195285	-0.15358	0.268099	-0.25095
0.114406	0.004513	NA	NA	0.113752	0.230344	0.061218	0.140932	0.087485
-0.28142	0.601695	-0.38552	0.594739	0.355149	0.371894	-0.32963	0.30499	0.012761
-0.29961	0.518065	-0.48076	0.229019	0.312291	0.300443	-0.21573	0.267832	0.04828
0.092767	0.081312	0.13354	0.249898	0.235469	0.124128	0.180347	0.178216	0.207908
0.089361	-0.06981	0.133098	0.075612	-0.09052	0.269286	-0.09746	0.192769	-0.09399
-0.18191	0.306274	0.309197	0.293553	0.254278	0.094276	0.034839	0.221527	0.144559
-0.00992	-0.00488	NA	NA	0.110309	0.419831	0.178535	0.169157	0.144422
-0.33791	0.223978	-0.54669	0.354318	0.270625	0.219538	-0.11498	0.326566	0.077824
-0.13477	0.331221	-0.51558	0.443424	0.079815	0.264333	-0.10077	0.243397	-0.01048
-0.38475	0.206173	-0.59916	0.327484	-0.033	0.29299	-0.30363	0.259823	-0.16832
0.041957	0.148878	-0.64668	-0.31729	-0.0488	0.356676	-0.08141	0.301993	-0.0651
0.022147	0.171606	-0.48622	-0.28687	-0.06515	0.258696	-0.05244	0.216503	-0.05879
-0.04023	-0.03525	-0.63108	-0.08041	-0.0914	0.148467	-0.10798	0.271527	-0.09969
-0.14863	-0.13849	-0.28013	-0.49991	0.01313	0.364027	-0.04964	0.219321	-0.01825
0.114466	-0.08127	-0.3304	-0.29199	-0.11625	0.097562	0.10349	0.227624	-0.00638
0.019651	0.35084	0.556689	0.36759	0.134775	0.223033	0.056245	0.373999	0.09551
0.130501	0.385572	0.525057	0.272197	0.03072	0.291175	0.004708	0.286924	0.017714
-0.12455	0.172338	0.295666	0.479958	-0.0285	0.391232	-0.09192	0.234174	-0.06021
-0.04262	0.425788	0.125856	-0.08522	0.061727	0.232095	-0.01097	0.09119	0.025379

0.280727	0.024563	0.757272	0.107309	-0.09625	0.216338	0.212557	0.39088	0.058153
-0.06865	-0.4188	-0.19311	-0.50659	-0.17746	0.275313	-0.04604	0.13484	-0.11175
NA	NA	NA	NA	-0.07018	0.237555	0.176331	0.260864	0.053075
NA	NA	NA	NA	-0.12815	0.323337	0.03918	0.229597	-0.04449
NA	NA	NA	NA	-0.31351	0.15059	-0.01923	0.082909	-0.16637
0.087494	0.243353	0.652719	0.271046	0.206892	0.044009	0.291954	0.360631	0.249423
-0.001	-0.08138	0.006902	-0.25036	0.120806	0.244126	0.051667	0.052344	0.086236
-0.16075	0.028562	0.16609	-0.1966	-0.01349	0.382176	-0.09358	0.382333	-0.05354
0.243765	0.036202	0.47642	0.323682	-0.07401	0.235896	0.176667	0.327126	0.05133
-0.22064	-0.21664	-0.18331	-0.18255	-0.08689	0.13009	-0.07716	0.131046	-0.08203
0.031457	-0.37874	-0.07778	-0.39484	-0.31408	0.165786	-0.11083	0.140688	-0.21245
0.293462	-0.03519	-0.09159	0.304715	-0.00829	0.273571	0.022655	0.171218	0.00718
-0.05332	0.050009	0.251071	0.209041	0.145344	0.216178	0.025688	0.284469	0.085516
0.173018	0.09969	NA	NA	0.07271	0.038155	0.306475	0.188737	0.189593
-0.08465	0.052961	-0.02736	-0.24644	-0.09655	0.152591	-0.07135	0.077785	-0.08395
0.039453	-0.07083	-0.25378	0.232574	0.040265	0.248583	-0.024	0.133749	0.008133
-0.13704	0.048924	-0.31262	0.085297	-0.01969	0.154264	-0.15009	0.169343	-0.08489
0.094762	-0.00735	NA	NA	0.012479	0.0911	-0.02329	0.184329	-0.00541
-0.13781	0.119087	-0.19779	0.046081	-0.00428	0.123888	-0.16497	0.235051	-0.08462
0.149533	-0.0449	NA	NA	-0.14256	0.138114	0.017289	0.187021	-0.06264
0.094738	0.068738	NA	NA	-0.05953	0.146257	0.126713	0.028244	0.033592
0.037817	0.212711	0.26133	0.182324	0.067172	0.356284	0.042986	0.211294	0.055079
-0.10359	0.086451	-0.51855	0.216047	0.186348	0.068178	-0.19268	0.30258	-0.00317
-0.02965	0.134507	-0.01766	0.213182	-0.01444	0.328485	-0.159	0.234501	-0.08672
-0.17582	0.127147	-0.11672	-0.24105	-0.01575	0.207069	-0.16931	0.231104	-0.09253
0.324244	0.390329	0.438129	0.066094	0.235604	0.145728	0.223364	0.284998	0.229484
0.005752	0.18195	-0.43594	-0.54752	-0.06323	0.329226	-0.19143	0.207507	-0.12733
-0.00254	-0.09827	NA	NA	0.078158	0.216605	0.049148	0.187364	0.063653
-0.00202	0.092104	NA	NA	0.027806	0.138918	0.224858	0.265691	0.126332
-0.07086	0.051126	NA	NA	-0.08141	0.191159	-0.03137	0.209539	-0.05639
-0.07327	-0.24158	-0.49915	-0.2834	-0.13274	0.314391	-0.10964	0.25356	-0.12119
0.19301	-0.21872	0.531574	0.036441	-0.04944	0.161561	0.257665	0.153924	0.104111
-0.07847	-0.25368	NA	NA	-0.15424	0.222417	0.014915	0.232166	-0.06966
-0.13032	-0.04388	NA	NA	-0.0145	0.251308	-0.01339	0.256902	-0.01395
0.318572	0.204072	0.220668	0.072604	0.020645	0.12304	0.208456	0.136985	0.114551
0.0626	0.087764	NA	NA	-0.15217	0.199884	0.087559	0.095104	-0.03231
-0.19875	0.036192	-0.64487	0.221497	0.077733	0.106565	-0.20311	0.477299	-0.06269
0.129237	0.175762	0.372491	0.358891	-0.01366	0.495221	0.17438	0.17984	0.08036
-0.14696	0.11376	-0.7441	-0.0152	-0.15634	0.215658	-0.25954	0.276351	-0.20794
0.10303	-0.0302	NA	NA	-0.25187	0.201222	0.04304	0.363725	-0.10442
0.068692	0.120842	0.246218	-0.42156	0.089021	0.339748	0.011814	0.171872	0.050418
0.192202	0.082891	0.482525	0.555095	0.188313	0.227265	0.204954	0.246821	0.196633
-0.09972	-0.04371	0.281113	-0.48429	-0.03876	0.31439	-0.08329	0.278631	-0.06103
-0.05545	0.099844	-0.3767	-0.06848	0.103061	0.173176	0.028371	0.452838	0.065716
-0.10115	-0.21876	NA	NA	0.07771	0.221277	-0.04229	0.062761	0.017712
0.065687	-0.07904	-0.68078	-0.30557	-0.07078	0.291883	-0.01147	0.398003	-0.04112
-0.06921	-0.04317	NA	NA	-0.1672	0.291869	0.002477	0.228511	-0.08236
0.070814	0.098749	NA	NA	0.103927	0.010158	-0.01145	0.131134	0.046236
-0.04661	0.08129	NA	NA	0.078412	0.118421	0.081498	0.161115	0.079955
0.364504	-0.10445	0.382814	-0.08	-0.08096	0.049932	0.084142	0.291874	0.001593
0.117053	-0.38504	NA	NA	-0.2886	0.111563	0.006282	0.16841	-0.14116
-0.15595	-0.17907	-0.14527	-0.44069	-0.22617	0.264057	-0.19392	0.175626	-0.21004

0.202822	-0.00514	-0.51556	-0.53766	-0.09327	0.310599	0.029298	0.310389	-0.03199
0.118229	0.2325	0.136175	0.237905	0.06947	0.1843	0.063549	0.060598	0.066509
-0.07374	0.049419	NA	NA	0.205108	0.230519	0.08073	0.294724	0.142919
-0.20747	0.010269	NA	NA	0.094366	0.258805	0.001775	0.216485	0.04807
0.101517	0.191625	0.21446	-0.2002	-0.04146	0.229432	0.030242	0.204074	-0.00561
0.014996	0.306304	NA	NA	-0.00763	0.238881	-0.0414	0.08542	-0.02451
-0.02113	0.321545	-0.17974	0.345225	0.18943	0.249618	-0.08888	0.081791	0.050274
0.085763	0.036956	0.439878	0.02225	-0.10708	0.193863	0.211234	0.202632	0.052077
0.104997	0.081685	0.331568	0.122586	-0.04383	0.163742	0.16329	0.189126	0.059728
-0.1173	0.086158	0.207913	0.251047	-0.00934	0.203933	-0.01088	0.209289	-0.01011
0.182644	-0.2634	NA	NA	-0.16035	0.138297	0.000981	0.28185	-0.07969
0.106616	0.322848	0.16145	0.29878	0.163035	0.160635	0.188361	0.098735	0.175698
0.017873	0.056212	0.067568	0.020836	-0.00764	0.148027	-0.1363	0.208011	-0.07197
-0.09477	0.092389	-0.28409	0.31056	0.314952	0.159308	-0.07694	0.158923	0.119004
0.085848	0.02457	-0.30138	-0.18423	-0.01742	0.142918	-0.1512	0.253791	-0.08431
-0.23167	-0.16501	-0.22144	0.001955	0.028409	0.20823	0.068066	0.321349	0.048238
0.218202	0.337399	0.015041	0.342518	0.304547	0.266693	0.018253	0.229332	0.1614
0.205972	0.232623	-0.28319	0.157032	0.113626	0.241986	0.145326	0.251945	0.129476
NA	NA	NA	NA	0.18968	0.127539	0.111727	0.389116	0.150703
0.097299	0.087661	NA	NA	0.07268	0.073431	-0.11625	0.334072	-0.02178
-0.23749	-0.17165	0.052594	-0.51549	-0.13016	0.293991	-0.14076	0.138614	-0.13546
0.098898	0.085695	-0.27101	0.246324	0.020476	0.145974	0.035989	0.17551	0.028233
-0.10444	-0.11144	-0.21549	-0.1719	0.041573	0.171411	-0.10548	0.16999	-0.03195
-0.29104	-0.19557	0.008932	-0.35139	-0.17347	0.121289	-0.17328	0.115798	-0.17338
0.072523	0.314445	0.145662	0.256823	0.111362	0.279036	-0.02251	0.146745	0.044426
0.099062	0.071518	0.488416	0.399045	0.116302	0.160944	0.221316	0.271314	0.168809
0.099016	0.137097	NA	NA	-0.13781	0.277189	-0.10373	0.141564	-0.12077
0.140293	0.234933	-0.25157	0.511197	0.115293	0.398143	0.068398	0.204359	0.091846
-0.22402	0.170545	-0.13282	-0.04022	0.096628	0.118644	-0.09845	0.14583	-0.00091
0.04264	-0.08284	-0.50929	0.502863	0.041579	0.31814	0.012449	0.335328	0.027014
-0.18545	-0.12777	-0.01538	-0.47562	-0.19504	0.189174	-0.14633	0.090547	-0.17069
-0.07349	-0.27132	0.407658	-0.43042	-0.23006	0.216682	0.07197	0.260175	-0.07905
-0.12594	-0.01793	NA	NA	-0.08832	0.057337	-0.23901	0.113245	-0.16367
0.005428	-0.37036	-0.32619	0.158945	-0.16973	0.308102	-0.25606	0.262226	-0.2129
0.181004	0.032288	-0.024	0.094313	-0.16269	0.211583	-0.0391	0.1336	-0.1009
-0.08335	-0.22554	-0.26221	0.285123	-0.1111	0.308965	-0.11937	0.113691	-0.11524
0.035339	-0.12978	-0.0464	-0.1945	-0.0774	0.124377	0.15441	0.210217	0.038504
-0.18924	-0.37861	-0.17996	-0.20664	-0.24766	0.080953	-0.12359	0.137072	-0.18562
-0.13171	0.022409	-0.12154	0.331619	0.071176	0.41621	0.003542	0.196484	0.037359
0.019439	-0.24956	0.636791	-0.3956	-0.23305	0.121897	0.158569	0.321689	-0.03724
-0.11159	-0.41914	0.096689	-0.17168	-0.35003	0.122695	-0.05517	0.137604	-0.2026
-0.29876	0.242126	-0.51658	0.165943	0.128706	0.129741	-0.25212	0.207017	-0.06171
0.023667	0.24102	0.143175	0.146198	-0.03002	0.261619	0.053485	0.062874	0.011731
-0.01299	0.240394	0.528159	0.364398	0.197614	0.135921	0.020233	0.374247	0.108924
-0.05089	0.32325	0.133911	0.023618	0.028027	0.257394	-0.14688	0.230406	-0.05943
0.419498	0.137623	0.813076	0.041116	0.13406	0.19064	0.377937	0.318867	0.255998
-0.05309	0.163517	-0.46476	0.336615	0.247679	0.083941	-0.09081	0.279987	0.078434
-0.13116	0.104059	-0.56782	0.191066	-0.02505	0.232875	-0.18369	0.267083	-0.10437
0.021389	0.10995	-0.30589	-0.35104	-0.03617	0.218816	-0.08582	0.248872	-0.06099
0.142683	0.086918	NA	NA	-0.03591	0.141668	0.135206	0.271618	0.04965
-0.0091	0.208375	-0.06091	0.165848	0.316195	0.177392	0.110612	0.194756	0.213403
0.114679	0.146082	0.446448	-0.14442	-0.00622	0.235208	0.19409	0.170393	0.093936

-0.06897	0.050951	NA	NA	-0.08917	0.194275	-0.17658	0.136155	-0.13287
0.120464	-0.13312	NA	NA	-0.0579	0.23533	-0.09952	0.161115	-0.07871
-0.47513	-0.28836	-0.32717	-0.63123	-0.26652	0.278377	-0.3142	0.170942	-0.29036
-0.0096	-0.13856	NA	NA	-0.00478	0.22564	-0.06249	0.247694	-0.03364
-0.34783	-0.0349	-0.6489	-0.38569	-0.13191	0.221124	-0.39414	0.174105	-0.26302
-0.09141	-0.02132	NA	NA	0.08656	0.314955	0.065894	0.224106	0.076227
-0.04301	-0.24839	0.390456	-0.52882	-0.23638	0.217838	0.031309	0.215408	-0.10253
-0.05225	0.395256	0.352794	-0.00657	0.181318	0.265774	0.119745	0.170193	0.150531
0.282965	0.180951	NA	NA	0.223147	0.390576	0.240788	0.157944	0.231968
-0.13345	0.32717	-0.09607	0.391964	0.160486	0.249386	-0.21827	0.234138	-0.02889
-0.32874	0.102298	-0.1446	0.549901	0.072018	0.288281	-0.08963	0.178209	-0.0088
0.235362	0.007361	-0.30719	-0.38942	-0.03167	0.336723	0.003943	0.201953	-0.01386
-0.20676	-0.18528	-0.36819	-0.20088	-0.11921	0.241094	-0.25464	0.189061	-0.18693
0.052043	0.116539	0.244286	-0.06923	0.058751	0.126506	0.02239	0.163354	0.04057
-0.15262	0.106196	NA	NA	0.059703	0.1632	-0.07396	0.136575	-0.00713
0.086606	0.048659	0.181276	0.601077	0.396134	0.271468	0.093825	0.194866	0.24498
-0.23041	-0.16223	-0.21012	-0.47574	-0.05943	0.288014	-0.20583	0.115592	-0.13263
0.046714	0.170134	0.273049	0.397181	0.224183	0.108692	0.103411	0.099279	0.163797
0.221578	0.187652	0.648755	0.46832	0.087772	0.27393	0.174922	0.294717	0.131347
0.03563	0.236366	NA	NA	-0.00644	0.17986	-0.11478	0.223717	-0.06061
0.112599	0.25025	-0.28403	0.402905	0.097261	0.28784	0.014666	0.241375	0.055964
-0.15364	-0.00147	0.450788	0.025006	-0.02063	0.110092	-0.11547	0.327261	-0.06805
-0.01702	-0.01204	NA	NA	0.084698	0.195268	-0.02702	0.429166	0.02884
0.013735	0.008224	-0.66368	-0.14138	-0.02722	0.194206	-0.05134	0.446375	-0.03928
0.277347	0.245483	0.046474	0.207376	0.039947	0.243927	0.069567	0.18721	0.054757
0.056794	0.021312	-0.4245	-0.58374	-0.04662	0.311099	-0.04681	0.215855	-0.04671
0.140432	0.0014	-0.08759	-0.48711	-0.30169	0.190137	-0.19516	0.267564	-0.24842
-0.13391	0.008902	-0.14511	0.292013	0.007997	0.214837	-0.04175	0.174866	-0.01688
-0.26228	-0.06528	-0.60634	-0.38728	-0.218	0.196966	-0.30072	0.249319	-0.25936
-0.23177	0.04938	0.483397	0.403934	-0.1017	0.330733	0.04279	0.2749	-0.02946
0.126509	0.339214	0.448757	0.096931	0.052975	0.208267	0.1001	0.288021	0.076537
0.13778	0.103183	-0.05938	-0.26017	0.003224	0.264944	0.004566	0.127042	0.003895
-0.0599	-0.12228	NA	NA	-0.31956	0.136483	-0.22314	0.271317	-0.27135
0.156932	0.321991	0.078984	-0.53178	-0.0587	0.353282	0.055453	0.092684	-0.00162
-0.0451	0.126003	0.370727	0.136647	0.119412	0.070822	0.083475	0.209811	0.101443
-0.15309	0.023092	-0.07162	-0.31989	-0.06038	0.215829	-0.07833	0.05189	-0.06935
0.024062	0.069577	-0.16952	-0.25518	0.039917	0.218961	-0.05949	0.119509	-0.00979
0.119	0.012889	0.293807	0.078179	-0.03992	0.109597	0.117067	0.125942	0.038576
0.384879	-0.09568	NA	NA	-0.36387	0.22264	0.128115	0.237592	-0.11788
0.186612	0.111039	NA	NA	0.114421	0.074857	0.067134	0.100686	0.090777
0.356244	0.46142	NA	NA	0.18236	0.274833	0.215929	0.142574	0.199145
0.460995	0.251248	NA	NA	0.040398	0.275217	0.299644	0.220918	0.170021
-0.18606	-0.09007	NA	NA	-0.05843	0.042135	-0.09587	0.20028	-0.07715
-0.14177	-0.33602	-0.12939	-0.30309	-0.20241	0.168342	-0.03509	0.11622	-0.11875
-0.07303	0.180422	0.283648	-0.10195	-0.12217	0.240175	-0.0551	0.281921	-0.08863
-0.08854	0.129199	0.068127	0.131468	0.141944	0.111508	0.043461	0.183024	0.092703
-0.16756	0.111848	-0.18727	-0.39687	-0.01856	0.266159	-0.083	0.173502	-0.05078
-0.33118	-0.03504	NA	NA	-0.2009	0.169275	-0.25883	0.166569	-0.22987
-0.07985	0.165058	0.293616	0.22407	0.139211	0.223394	0.065428	0.210677	0.10232
-0.30729	0.243705	NA	NA	0.113539	0.141899	-0.24389	0.053712	-0.06518
-0.36214	0.009155	-0.46114	0.186197	-0.13913	0.289872	-0.35696	0.075414	-0.24805
0.110639	0.301847	NA	NA	0.354581	0.084503	0.028895	0.186438	0.191738

-0.26464	-0.00781	-0.14351	-0.24325	0.007025	0.357967	-0.05243	0.284404	-0.0227
-0.38474	0.187387	-0.14805	-0.35138	-0.14281	0.199322	-0.30556	0.095157	-0.22418
-0.34375	-0.0137	0.121069	-0.14914	0.090599	0.272261	-0.01672	0.261576	0.036938
-0.32458	0.207835	-0.4405	-0.06864	-0.08422	0.213744	-0.27021	0.12873	-0.17721
NA	NA	-0.51043	-0.45193	-0.02698	0.396636	-0.00498	0.474944	-0.01598
-0.42918	-0.05727	-0.40242	-0.24292	0.039457	0.207303	-0.30756	0.177511	-0.13405
-0.40915	-0.00047	NA	NA	0.146511	0.227994	-0.30742	0.317579	-0.08045
-0.23604	-0.03632	NA	NA	0.129642	0.130607	-0.28508	0.191515	-0.07772
-0.27569	0.191795	-0.45277	0.431773	-0.06525	0.398643	-0.37039	0.116897	-0.21782
-0.04928	0.015595	NA	NA	-0.20636	0.172914	-0.05826	0.04511	-0.13231
-0.31633	-0.02195	NA	NA	-0.04249	0.01495	-0.29399	0.079868	-0.16824
-0.38333	-0.07529	NA	NA	-0.2156	0.123432	-0.15806	0.156494	-0.18683
-0.05392	0.214365	NA	NA	0.086835	0.231676	0.01035	0.204303	0.048592
-0.26484	-0.34271	-0.06451	-0.37626	-0.14596	0.208499	-0.23609	0.123215	-0.19103
-0.2471	-0.37388	NA	NA	-0.13469	0.190444	-0.23878	0.084266	-0.18673
-0.29872	-0.18674	NA	NA	-0.15036	0.059101	-0.27294	0.084387	-0.21165
-0.30574	0.190653	-0.75376	-0.31947	0.122506	0.25891	-0.26691	0.293338	-0.0722
-0.02184	0.283005	NA	NA	-0.03561	0.248129	-0.02294	0.165895	-0.02927
-0.28278	-0.1233	-0.05328	-0.45722	-0.32637	0.258267	-0.35856	0.235011	-0.34247
0.121176	0.160134	0.189216	-0.16017	0.053862	0.185357	0.087915	0.121397	0.070889
-0.21211	0.137572	0.352982	-0.09244	-0.03958	0.211603	-0.08538	0.311379	-0.06248
0.108137	0.063177	NA	NA	-0.05113	0.183317	0.065121	0.194022	0.006997
-0.10556	0.112824	0.114085	-0.11822	0.067121	0.147686	-0.01524	0.115783	0.02594
-0.23591	0.141021	-0.15988	-0.21125	-0.16687	0.193236	-0.12951	0.130563	-0.14819
-0.17204	0.18219	NA	NA	0.09306	0.06305	-0.092	0.20096	0.00053
-0.46018	-0.34513	-0.52176	-0.49295	-0.28331	0.184137	-0.34498	0.168818	-0.31414
-0.23855	-0.34691	0.057269	-0.56587	-0.20972	0.257811	-0.14429	0.142066	-0.17701
-0.011	-0.06166	-0.04214	-0.42156	-0.12898	0.228471	0.087457	0.191249	-0.02076
0.00968	-0.32891	NA	NA	-0.26566	0.299681	0.028438	0.355375	-0.11861
-0.34948	-0.01965	-0.76461	0.207418	0.041223	0.161472	-0.26243	0.309721	-0.1106
-0.27054	-0.45623	-0.64597	-0.38154	-0.33839	0.179778	-0.35376	0.203188	-0.34608
-0.13027	0.130965	NA	NA	0.280142	0.101213	-0.10769	0.06006	0.086227
-0.2215	0.168979	NA	NA	-0.24637	0.294516	-0.09991	0.158615	-0.17314
-0.00063	0.22896	NA	NA	-0.09977	0.221207	0.066335	0.183035	-0.01672
0.003953	0.316816	NA	NA	0.296857	0.187617	0.058212	0.07675	0.177535
0.042021	-0.07647	0.151849	-0.26737	0.019956	0.202486	0.100885	0.044079	0.060421
-0.25003	0.206187	0.125573	0.27395	0.063178	0.209932	-0.07549	0.193973	-0.00616
0.091562	0.186149	0.026093	-0.0174	-0.10619	0.244239	0.077388	0.033149	-0.0144
-0.12991	-0.261	NA	NA	-0.01954	0.165745	0.002412	0.169616	-0.00857
0.038557	-0.00331	NA	NA	0.003863	0.101975	-0.14068	0.121446	-0.06841
-0.09398	-0.07648	NA	NA	0.152983	0.223272	-0.0915	0.251374	0.030741
0.104974	0.126896	0.45449	0.076222	-0.09219	0.233548	0.022538	0.311107	-0.03482
0.119383	-0.00183	NA	NA	0.078222	0.176541	0.124607	0.142711	0.101415
0.122091	0.041221	-0.29608	-0.22077	-0.11023	0.158786	-0.18361	0.276887	-0.14692
-0.14557	0.270886	-0.09984	0.335018	0.322509	0.231042	-0.11625	0.119677	0.103128
-0.1167	-0.04023	-0.36078	0.04762	-0.1492	0.154489	-0.263	0.181046	-0.2061
-0.10507	0.129592	NA	NA	0.012034	0.199021	0.059072	0.151798	0.035553
-0.19398	0.041146	-0.05365	-0.34144	-0.11896	0.178544	0.017927	0.225045	-0.05052
0.062193	-0.18801	-0.37279	-0.06412	-0.19179	0.097041	-0.03916	0.222951	-0.11548
-0.09245	0.24313	-0.82877	-0.48719	-0.13572	0.325513	-0.09526	0.435646	-0.11549
0.276429	0.305434	0.015699	0.037161	0.053726	0.154323	0.029577	0.153588	0.041651
0.077147	0.084301	-0.37168	-0.1062	-0.0466	0.30877	-0.03709	0.234293	-0.04185

0.196741	-0.35633	0.404983	-0.4345	-0.08981	0.287719	0.144099	0.290648	0.027143
0.16163	0.354661	0.583655	0.264005	0.207819	0.217969	0.184811	0.355302	0.196315
-0.0799	0.084588	NA	NA	0.038827	0.319878	0.080366	0.230116	0.059596
0.086988	0.465499	0.037658	0.105457	0.117638	0.218428	0.098202	0.188764	0.10792
-0.49642	0.266108	-0.37815	0.239833	-0.06473	0.295289	-0.3252	0.16353	-0.19497
-0.18957	0.05618	NA	NA	-0.24896	0.215946	-0.26733	0.172084	-0.25815
0.188642	-0.33553	-0.46709	-0.43641	-0.21607	0.20047	-0.08411	0.277547	-0.15009
-0.09811	0.233184	-0.75669	0.018714	0.12382	0.093766	-0.34089	0.30011	-0.10854
0.169228	0.103927	0.540471	-0.10034	0.104518	0.157639	0.191514	0.275037	0.148016
0.092413	0.099985	-0.5411	0.325576	0.069481	0.191019	-0.18036	0.342751	-0.05544
-0.07945	-0.27933	-0.1432	-0.56739	-0.23197	0.25209	-0.08535	0.108763	-0.15866
0.124873	0.003302	NA	NA	0.051777	0.160714	-0.02527	0.235117	0.013255
0.036466	0.005707	-0.20146	-0.17689	-0.18478	0.260585	-0.07778	0.102103	-0.13128
0.291926	0.313564	0.317274	-0.03963	0.139549	0.28303	0.183518	0.167954	0.161533
0.004163	0.047478	NA	NA	0.09041	0.155376	-0.05063	0.069043	0.019891
0.459255	0.170865	0.762379	0.026215	0.07842	0.123851	0.323255	0.362152	0.200838
0.139268	0.163146	0.139969	0.060023	0.070441	0.110726	0.071563	0.133239	0.071002
-0.0461	-0.41148	-0.16458	-0.60047	-0.23632	0.313395	-0.13732	0.140325	-0.18682
0.318299	0.043454	0.293725	0.056419	0.033591	0.066131	0.15123	0.195947	0.09241
0.211394	-0.35148	0.424208	-0.10671	-0.23505	0.223806	0.090484	0.297233	-0.07228
0.195915	-0.09322	-0.32214	0.00853	-0.04184	0.131925	0.047205	0.227904	0.002684
0.200934	0.404352	0.33816	0.255374	0.141	0.369075	0.108009	0.326176	0.124505
-0.29648	0.482747	-0.44203	0.647534	0.353408	0.286611	-0.11863	0.267731	0.117389
0.336961	0.406094	0.909991	0.47195	0.123374	0.337933	0.390053	0.326858	0.256713
0.262081	0.095437	0.36096	0.185274	-0.11399	0.306226	0.034924	0.33974	-0.03953
0.194539	0.26602	0.300119	-0.08358	0.100875	0.328281	0.171352	0.329527	0.136114
0.33859	0.331982	0.676185	0.241809	0.144058	0.223889	0.185614	0.439554	0.164836
0.267688	0.006779	NA	NA	-0.06122	0.142505	0.058185	0.155597	-0.00152
-0.09666	0.065025	-0.65812	-0.52522	-0.10569	0.281147	-0.1946	0.311598	-0.15014
0.163272	-0.05583	NA	NA	-0.17518	0.109557	0.05254	0.269767	-0.06132
0.221702	0.14659	-0.08918	0.080379	0.129313	0.26203	0.061637	0.240683	0.095475
0.108313	-0.00972	0.466128	0.246302	-0.06665	0.241502	0.147658	0.21753	0.040502
0.114927	0.136959	-0.13174	-0.26084	-0.01614	0.182026	-0.05594	0.151964	-0.03604
0.027686	-0.14767	0.087248	-0.07049	-0.17889	0.15661	-0.0138	0.245211	-0.09635
0.088238	-0.086	-0.63175	-0.09308	-0.12575	0.352254	-0.20193	0.306396	-0.16384
-0.05114	0.159974	-0.1477	0.320293	0.074014	0.219084	-0.04362	0.097718	0.0152
-0.00393	-0.11987	NA	NA	-0.04674	0.17964	0.156601	0.201956	0.05493
0.157024	0.312215	0.28173	-0.1292	0.093615	0.183013	0.165193	0.146464	0.129404
0.405013	-0.02825	0.728121	0.011422	-0.07375	0.156405	0.257439	0.323943	0.091843
0.195662	0.042633	-0.2382	-0.20703	-0.05396	0.112406	0.049053	0.214215	-0.00245
-0.12059	-0.03521	-0.03132	-0.23983	-0.06239	0.120461	0.05887	0.163198	-0.00176
-0.00373	0.002526	0.431786	0.392303	0.034925	0.247222	0.119122	0.280587	0.077023
0.21184	0.290975	0.499384	0.2536	0.147905	0.149111	0.043316	0.433816	0.095611
-0.03055	-0.04247	-0.65086	-0.20541	0.05895	0.221548	-0.2436	0.300251	-0.09233
0.017289	0.06578	-0.00897	0.181886	0.001604	0.252466	-0.09299	0.121156	-0.04569
0.05532	0.156448	-0.4199	-0.23014	0.099439	0.275993	-0.04609	0.255067	0.026676
-0.18067	0.046729	-0.26917	-0.1689	0.071782	0.243184	0.04042	0.26005	0.056101
0.06663	0.270252	NA	NA	-0.04327	0.215291	0.061876	0.281678	0.009301
0.023618	-0.33297	-0.27063	-0.46524	-0.31448	0.340254	-0.18545	0.270542	-0.24996
-0.13128	0.133588	NA	NA	-0.0385	0.284861	0.028888	0.252361	-0.00481
0.058471	0.037832	NA	NA	0.088198	0.254983	0.078767	0.212091	0.083482
0.00323	0.020995	-0.25483	0.166358	-0.08522	0.194104	-0.05117	0.158444	-0.06819

-0.2354	0.071877	-0.75174	-0.41881	-0.15884	0.209167	-0.2927	0.292541	-0.22577
0.023444	0.485736	0.316768	0.35881	0.361056	0.135735	0.099158	0.242654	0.230107
-0.09011	0.160955	-0.45046	-0.2531	-0.00413	0.19168	-0.11221	0.262344	-0.05817
-0.03423	-0.02025	0.241107	-0.5393	-0.162	0.283034	0.058793	0.141285	-0.0516
0.003134	0.404074	0.567615	0.516275	0.247176	0.215364	0.204956	0.237052	0.226066
-0.09257	0.089972	NA	NA	0.094315	0.326694	0.014554	0.268258	0.054434
0.085299	0.364175	-0.20741	0.34867	0.29266	0.115665	0.086196	0.196116	0.189428
0.016673	0.232195	0.027966	-0.28437	0.005309	0.196226	-0.02035	0.116726	-0.00752
0.046504	-0.1943	-0.26549	-0.19809	-0.12657	0.309196	0.040871	0.216163	-0.04285
-0.16951	0.101721	-0.60997	-0.05143	0.109426	0.123944	-0.12877	0.293737	-0.00967
-0.17671	0.040892	-0.4727	-0.07717	0.015077	0.170209	-0.13125	0.271304	-0.05808
0.13187	0.390572	-0.21166	0.617895	0.367085	0.194586	0.121357	0.230646	0.244221
0.135539	0.2704	NA	NA	0.12501	0.313321	0.236106	0.251839	0.180558
0.084656	0.187378	-0.18096	-0.04344	0.040336	0.378217	-0.02394	0.122596	0.008197
0.029952	0.161967	0.059153	0.019157	0.16273	0.293191	0.171468	0.140768	0.167099
-0.07922	0.057356	-0.1595	0.193688	0.122728	0.244842	0.054074	0.163844	0.088401
0.114827	0.045918	-0.06772	0.132979	0.222	0.157133	-0.09877	0.160967	0.061617
0.012614	0.231571	NA	NA	0.275523	0.145564	-0.04541	0.118952	0.115057
0.069868	0.359681	0.121433	-0.09511	0.164055	0.183708	0.147001	0.272825	0.155528
0.073462	0.110847	NA	NA	-0.14584	0.218172	0.031208	0.225849	-0.05732
0.056997	0.117796	-0.15114	-0.04583	0.090848	0.168977	-0.1183	0.180421	-0.01373
-0.01453	0.188078	NA	NA	0.136388	0.11571	0.022023	0.289699	0.079205
0.122762	-0.05576	0.148984	-0.58802	-0.2235	0.246341	0.066021	0.088689	-0.07874
0.20916	0.267871	NA	NA	-0.06143	0.224957	0.00551	0.143763	-0.02796
0.046046	0.072227	0.031164	-0.16601	-0.05704	0.109779	0.078715	0.166381	0.010835
0.217094	0.1357	0.277497	0.261535	0.097887	0.327939	0.210438	0.117585	0.154162
0.137182	-0.30447	0.173691	-0.3104	-0.25962	0.271896	0.010685	0.342341	-0.12447
0.114413	-0.33264	-0.29893	-0.54092	-0.15138	0.284346	0.109244	0.264345	-0.02107
-0.29034	-0.01214	-0.76872	-0.42614	-0.15416	0.175867	-0.17343	0.387695	-0.1638
-0.33676	-0.17758	-0.71619	-0.25514	-0.0068	0.274604	-0.33289	0.292512	-0.16985
0.001461	-0.09191	0.288297	-0.15697	-0.16502	0.113871	0.096949	0.147787	-0.03403
0.110634	0.098174	0.371468	0.272247	0.085624	0.152812	0.2304	0.146318	0.158012
0.162189	0.285198	0.467821	0.652264	0.281737	0.281986	0.104	0.278412	0.192869
-0.1156	-0.03537	-0.06799	-0.09038	-0.04569	0.046048	-0.06901	0.064854	-0.05735
-0.01232	0.143746	NA	NA	0.037735	0.118952	-0.07628	0.214156	-0.01927
-0.10168	0.132954	0.033404	0.338576	0.108961	0.169144	-0.13598	0.140064	-0.01351
-0.05545	-0.15371	-0.10626	-0.20907	-0.05137	0.158756	0.00312	0.143177	-0.02413
0.068489	0.144688	NA	NA	0.011251	0.291016	0.069378	0.089241	0.040314
-0.0947	0.045718	NA	NA	-0.06335	0.095398	-0.15599	0.094262	-0.10967
0.017164	-0.18114	-0.33785	-0.12825	-0.21993	0.095388	-0.23078	0.231159	-0.22536
-0.09885	0.068823	NA	NA	0.123289	0.16481	-0.01741	0.074276	0.052941
-0.29961	0.010415	-0.64841	-0.31298	-0.1139	0.227914	-0.22301	0.268662	-0.16846
0.269376	0.161834	NA	NA	0.039189	0.129201	0.012978	0.198526	0.026084
-0.30021	-0.21538	-0.73444	-0.23283	-0.22636	0.122303	-0.3736	0.278348	-0.29998
0.055205	0.035721	-0.33944	-0.18854	-0.03152	0.123755	-0.18466	0.214778	-0.10809
0.029726	0.267155	NA	NA	0.048448	0.258682	0.026629	0.180999	0.037539
-0.1063	0.132867	0.627749	0.248704	0.066141	0.170222	0.090063	0.30342	0.078102
-0.08771	-0.22744	NA	NA	-0.24542	0.134681	-0.18877	0.11727	-0.21709
-0.08494	0.160549	-0.26007	-0.01308	-0.06359	0.262883	-0.21579	0.127043	-0.13969
-0.09222	0.202185	-0.58679	0.268453	0.149743	0.341381	-0.09941	0.334724	0.025165
-0.02804	0.211914	0.313171	0.085986	0.108696	0.093945	0.144213	0.170628	0.126454
0.036566	-0.16831	-0.08153	0.050034	-0.07409	0.20087	-0.01844	0.056217	-0.04626

0.008211	-0.09008	-0.17299	-0.10982	-0.02551	0.156986	-0.12911	0.101492	-0.07731
0.151639	-0.20251	NA	NA	-0.01512	0.157356	0.066153	0.241501	0.025515
-0.1339	0.036158	NA	NA	-0.05871	0.1266	0.015331	0.122161	-0.02169
0.155148	0.29989	0.101993	0.538825	0.222608	0.227636	0.157956	0.12234	0.190282
0.252527	0.340779	NA	NA	0.16184	0.164627	0.259407	0.043214	0.210623
0.276768	0.381368	0.584534	0.246678	0.163118	0.202745	0.324578	0.203839	0.243848
0.156731	0.229832	0.119428	0.227092	0.160177	0.315067	0.140884	0.149165	0.15053
0.115799	0.120182	0.158306	-0.68458	-0.11559	0.332539	0.063712	0.089855	-0.02594
0.086797	0.34676	NA	NA	0.251891	0.139993	0.13979	0.102967	0.19584
0.066865	0.185481	NA	NA	0.201352	0.065135	-0.05158	0.176282	0.074885
0.187907	-0.2018	NA	NA	-0.20418	0.202651	-0.09347	0.389639	-0.14882
-0.25557	0.276303	-0.21066	0.378171	0.037053	0.29222	-0.09261	0.259003	-0.02778
0.107498	-0.06811	NA	NA	-0.11917	0.314227	0.037451	0.218233	-0.04086
-0.00226	0.160525	-0.15207	-0.06477	0.127971	0.1787	0.093095	0.304262	0.110533
0.031527	0.181309	NA	NA	0.100936	0.122734	-0.04063	0.062191	0.030154
-0.15445	0.182145	-0.0497	0.450855	0.108254	0.306477	-0.05482	0.14011	0.026718
0.21222	0.123834	0.536827	0.324159	0.035823	0.193868	0.054706	0.369102	0.045264
-0.18403	-0.05211	-0.03238	-0.14508	-0.13491	0.079905	-0.1803	0.129431	-0.1576
0.047491	0.373871	0.676247	0.246567	0.23457	0.201993	0.229241	0.302565	0.231905
0.158954	-0.25328	0.084695	-0.38022	-0.13319	0.239075	0.165264	0.082135	0.016038
0.080943	0.155867	0.004475	0.69404	0.130164	0.440934	0.058018	0.061926	0.094091
-0.07474	0.325625	0.385122	0.09761	0.179226	0.088517	0.261039	0.209929	0.220133
0.035183	0.00769	0.76206	-0.02867	0.029112	0.080556	0.25617	0.342109	0.142641
0.205005	-0.02338	0.818126	0.104765	0.031986	0.206973	0.226619	0.4271	0.129302
0.083596	0.153028	-0.20809	-0.09396	0.085852	0.171344	0.093414	0.329791	0.089633
-0.17949	0.119719	0.310497	-0.0255	-0.07761	0.157885	-0.01432	0.245792	-0.04596
-0.06892	-0.06891	0.265615	-0.00945	-0.02503	0.128022	0.059179	0.155185	0.017077
0.095431	0.252761	0.097213	0.068638	0.171485	0.142715	0.040046	0.138739	0.105766
-0.00899	0.245464	0.442986	0.156466	0.214992	0.076603	0.282958	0.21263	0.248975
-0.1432	0.045	0.207409	0.092781	0.067982	0.043881	0.173164	0.255178	0.120573
0.210832	0.203489	0.380225	0.133272	-0.05175	0.274566	0.227808	0.161874	0.088027
0.04448	-0.00687	0.317656	-0.00256	-0.0725	0.102339	0.14509	0.169553	0.036294
0.087197	0.182411	0.234048	0.046189	-0.02228	0.170446	0.051194	0.140357	0.014457
0.019381	-0.15083	0.10026	0.080374	-0.19637	0.195132	-0.18617	0.28513	-0.19127
-0.02241	0.112439	0.371907	-0.06278	-0.10853	0.253441	0.003543	0.19808	-0.05249
0.252034	-0.0176	0.364928	0.354337	0.02105	0.27443	0.16186	0.143617	0.091455
0.166569	0.045996	0.256724	0.426707	0.087447	0.238921	0.127727	0.221221	0.107587
-0.1936	-0.05229	0.043631	0.079469	0.090458	0.225386	-0.1435	0.237473	-0.02652
-0.04956	0.283686	NA	NA	-0.0582	0.26407	-0.06381	0.128187	-0.06101
-0.16044	0.09638	NA	NA	0.095979	0.152288	0.063041	0.344428	0.07951
0.1248	0.068546	0.358681	0.632571	0.131631	0.297402	0.010416	0.300416	0.071024
0.075553	0.234803	-0.00679	0.626374	0.199475	0.286602	0.105256	0.227802	0.152365
0.0053	0.107369	NA	NA	0.0913	0.173668	0.016514	0.359002	0.053907
0.137417	-0.18303	0.238421	-0.40031	-0.00398	0.325581	0.217248	0.244653	0.106632
-0.29627	0.134716	-0.06401	-0.37711	-0.09584	0.212425	-0.12763	0.158725	-0.11173
0.148541	0.134997	-0.43324	-0.13607	-0.01534	0.116642	-0.00089	0.252553	-0.00812
0.071445	0.266962	0.616459	0.447248	0.152551	0.242121	0.163428	0.379648	0.15799
0.285927	0.105941	NA	NA	-0.0993	0.273284	-0.10036	0.306851	-0.09983
0.124995	-0.2048	-0.56741	-0.57199	-0.27019	0.171625	-0.16589	0.298774	-0.21804
-0.07896	0.107176	-0.60455	0.34237	0.09596	0.28158	-0.01489	0.371294	0.040533
0.042356	0.199174	0.342318	0.295075	0.153441	0.238467	0.168449	0.314483	0.160945
0.10021	-0.26737	0.09197	-0.22225	-0.14513	0.181371	0.117873	0.058267	-0.01363

-0.0244	-0.29599	-0.34262	-0.38284	-0.18601	0.220306	-0.04852	0.204734	-0.11726
-0.08734	-0.04397	NA	NA	-0.05268	0.331685	-0.08976	0.386412	-0.07122
-0.26855	0.165208	NA	NA	-0.04772	0.193332	-0.25887	0.16409	-0.15329
-0.00469	0.110926	-0.16657	0.015426	0.00273	0.243916	-0.04019	0.074781	-0.01873
0.007786	-0.09743	NA	NA	0.127518	0.370247	0.117357	0.165338	0.122437
-0.07709	0.142976	NA	NA	0.045309	0.254188	-0.03149	0.278211	0.00691
-0.03948	0.258261	0.467123	0.244838	-0.013	0.39185	0.087274	0.382665	0.037138
0.14794	0.288963	0.678565	0.049387	0.087949	0.224375	0.207071	0.335565	0.14751
0.125383	0.206787	NA	NA	0.102234	0.214069	0.004223	0.083663	0.053229
0.102269	0.016226	-0.07699	-0.05237	0.09817	0.204237	0.123295	0.21158	0.110733
-0.01054	0.013306	-0.26093	-0.19961	-0.07409	0.248432	-0.15521	0.231332	-0.11465
0.179142	0.015319	NA	NA	0.107562	0.130451	0.179156	2.06E-05	0.143359
0.080376	0.219151	-0.64204	-0.03068	-0.01834	0.217529	-0.23128	0.291454	-0.12481
0.080657	0.133281	NA	NA	-0.12233	0.184783	0.042077	0.052476	-0.04013
0.03772	-0.00778	NA	NA	-0.06165	0.214199	-0.26428	0.219976	-0.16297
-0.15598	0.190338	NA	NA	-0.16421	0.281671	-0.21539	0.082131	-0.1898
0.165902	0.100435	NA	NA	0.04482	0.107655	0.094104	0.389234	0.069462
0.133916	0.063641	NA	NA	-0.04357	0.07315	-0.03773	0.148895	-0.04065
0.180231	0.248392	0.037898	0.059077	0.138557	0.230555	0.203938	0.117437	0.171248
-0.26285	0.108098	-0.17794	0.409544	0.192066	0.14953	-0.19868	0.258801	-0.00331
0.15043	-0.02042	NA	NA	-0.09038	0.054832	0.198784	0.064097	0.0542
0.073643	0.102664	-0.43215	-0.22498	-0.08013	0.322124	-0.06868	0.260297	-0.07441
0.012828	0.086404	0.41645	0.122451	0.021175	0.118157	0.047457	0.267077	0.034316
0.149302	0.365095	-0.06891	0.41951	0.100827	0.33799	0.096244	0.272486	0.098536
-0.00307	0.008487	-0.72439	-0.44438	0.032351	0.301257	-0.18412	0.331145	-0.07588
0.148457	-0.05718	-0.09068	-0.58928	-0.15191	0.318306	0.055027	0.135187	-0.04844
0.221249	0.043563	NA	NA	-0.02647	0.12368	0.119698	0.17467	0.046615
0.031728	0.082385	-0.51176	0.062242	0.011181	0.173079	-0.0308	0.275832	-0.00981
-0.02301	0.140049	-0.01844	0.603972	0.019342	0.4008	-0.04643	0.152061	-0.01355
-0.10539	-0.26424	NA	NA	-0.13322	0.185265	0.003502	0.149337	-0.06486
-0.20295	0.051445	-0.68772	-0.08029	0.071171	0.159976	-0.14015	0.328833	-0.03449
0.215279	0.074959	0.139077	-0.38403	-0.08297	0.228453	0.084046	0.094313	0.000537
0.064802	-0.0512	NA	NA	0.160837	0.299862	0.015875	0.069194	0.088356
0.035128	-0.1573	NA	NA	-0.12082	0.170351	-0.09866	0.148126	-0.10974
0.079711	0.108509	-0.4342	0.386416	0.012969	0.330211	-0.11856	0.251462	-0.0528
0.189676	-0.03827	0.226299	-0.52151	-0.23622	0.204938	0.116203	0.191581	-0.06001
-0.16527	-0.34388	0.396103	-0.42671	-0.0763	0.291342	0.093435	0.265207	0.008566
0.139038	0.082126	-0.28836	0.327544	-0.02803	0.23528	0.002601	0.224567	-0.01271
-0.01714	-0.2641	0.271253	-0.21397	0.007355	0.285253	-0.00464	0.204323	0.001356
0.099235	-0.23395	-0.4989	-0.03599	-0.19761	0.233566	-0.21788	0.2132	-0.20775
-0.04722	0.206224	-0.28849	0.375676	0.044166	0.299492	-0.02245	0.167116	0.010859
-0.2195	0.188372	-0.58645	-0.29875	0.043294	0.24532	-0.23793	0.326284	-0.09732
0.04634	-0.38143	-0.35144	-0.2314	-0.14572	0.195991	-0.07082	0.187826	-0.10827
-0.04207	-0.30004	0.116175	-0.41901	-0.10695	0.298434	0.123167	0.132097	0.00811
-0.18577	0.123067	-0.22992	-0.13674	-0.08389	0.176551	-0.21714	0.197911	-0.15051
-0.01557	-0.14489	NA	NA	-0.28644	0.181933	-0.1488	0.132271	-0.21762
0.040686	-0.09675	NA	NA	-0.21032	0.080614	-0.0451	0.195667	-0.12771
-0.10382	0.063047	-0.03099	0.054972	0.024469	0.035849	-0.21478	0.157679	-0.09516
-0.05221	0.074222	0.470889	0.035031	-0.11553	0.213088	-0.04482	0.334849	-0.08017
0.128097	0.296678	0.55692	0.053462	0.209828	0.135125	0.306419	0.250694	0.258124
-0.22485	0.097982	0.147774	0.429522	0.256838	0.118519	-0.0614	0.334978	0.097721
0.047796	0.025364	0.197195	0.060018	-0.09224	0.221556	0.07966	0.116519	-0.00629

0.141393	0.134183	-0.08725	-0.13839	-0.17973	0.277633	-0.00943	0.210381	-0.09458
0.068914	-0.10871	-0.42361	-0.2675	-0.19392	0.140542	-0.03204	0.23411	-0.11298
0.121486	-0.34599	-0.21301	-0.39354	-0.18261	0.208438	-0.11843	0.161863	-0.15052
-0.29035	-0.08829	-0.26985	-0.03418	-0.06148	0.209749	-0.11538	0.158655	-0.08843
-0.14176	0.228216	0.095691	0.180916	-0.01821	0.223324	-0.09449	0.242099	-0.05635
-0.28046	-0.25447	-0.07382	-0.01096	-0.12052	0.138469	-0.26815	0.168824	-0.19433
-0.06966	0.030733	0.408342	0.115888	0.007677	0.211522	0.065496	0.304451	0.036587
0.080147	-0.08751	0.347759	-0.08476	0.104323	0.240161	0.122751	0.279565	0.113537
-0.28101	-0.12717	NA	NA	-0.08773	0.056809	-0.1114	0.151858	-0.09957
-0.07847	-0.06734	NA	NA	-0.09799	0.245191	-0.1855	0.09408	-0.14174
0.124809	0.274073	NA	NA	0.104933	0.321875	0.215135	0.163812	0.160034
-0.1693	-0.00065	0.083595	0.442174	0.00532	0.251845	-0.05107	0.214513	-0.02288
-0.10597	0.059452	-0.04705	0.214174	-0.01362	0.218154	-0.16326	0.254278	-0.08844
0.05082	0.137564	NA	NA	0.009806	0.138396	-0.01988	0.154201	-0.00504
0.127508	0.009171	-0.19471	-0.07883	-0.0407	0.107811	-0.0732	0.179068	-0.05695
0.096998	0.173649	NA	NA	0.211641	0.264217	0.29588	0.237214	0.25376
-0.15207	0.053533	NA	NA	0.108274	0.260147	0.135957	0.293424	0.122115
0.016	0.182477	0.747844	0.251865	0.052356	0.175969	0.185022	0.319856	0.118689
-0.04328	0.144864	NA	NA	-0.05892	0.19169	-0.02269	0.021236	-0.0408
0.161069	0.290519	NA	NA	0.066327	0.167347	0.193157	0.20632	0.129742
0.107669	0.19871	NA	NA	0.184983	0.193193	0.223512	0.083723	0.204247
-0.04317	-0.06217	-0.21625	-0.08519	-0.1204	0.106472	-0.05937	0.176121	-0.08988
0.332978	0.103103	0.113609	-0.31643	-0.04808	0.206314	0.163393	0.095206	0.057658
-0.00504	0.178403	0.113138	0.007984	-0.06478	0.204986	0.037969	0.093595	-0.0134
-0.0926	-0.449	-0.18946	-0.41787	-0.25029	0.198093	-0.03579	0.284869	-0.14304
0.094156	0.27842	NA	NA	0.042369	0.200453	0.176824	0.16918	0.109596
-0.00902	-0.01577	0.316919	-0.04241	-0.01455	0.120894	0.034643	0.259394	0.010048
0.244835	0.156056	NA	NA	-0.03636	0.136741	0.217751	0.14323	0.090698
0.120981	0.193795	NA	NA	-0.18044	0.32968	0.028788	0.295978	-0.07583
-0.11681	0.00147	-0.32034	-0.2288	-0.14844	0.274198	-0.13196	0.137511	-0.1402
0.067431	0.012988	-0.29357	-0.45375	-0.24724	0.191891	-0.12893	0.146465	-0.18808
-0.05645	0.099456	NA	NA	0.022618	0.142916	-0.06633	0.099381	-0.02186
-0.26794	0.107232	-0.28881	0.377005	0.129232	0.145575	-0.2148	0.260808	-0.04279
-0.27803	0.044433	-0.20568	0.365996	0.052811	0.19104	-0.18394	0.285177	-0.06556
-0.04208	0.177547	0.059394	-0.27773	-0.00597	0.272031	-0.01	0.199045	-0.00799
-0.2308	0.085251	-0.14696	0.371599	0.122456	0.138746	-0.17127	0.257308	-0.02441
0.091196	-0.05627	-0.33874	-0.5102	-0.05244	0.280357	-0.12511	0.170068	-0.08878
0.182019	0.142748	NA	NA	0.090998	0.133708	0.042216	0.141817	0.066607
-0.00081	0.170082	0.32465	0.201696	0.171788	0.228016	0.053429	0.281986	0.112608
-0.09368	0.043685	0.274298	-0.34753	-0.20195	0.172723	0.029246	0.178592	-0.08635
-0.07716	0.018059	0.043506	-0.50572	0.014134	0.272213	0.081617	0.173363	0.047876
-0.05838	-0.30016	-0.52542	-0.42633	-0.06573	0.334717	-0.1239	0.306164	-0.09482
-0.13132	-0.3671	-0.34378	-0.46198	-0.17915	0.20243	-0.14331	0.204439	-0.16123
0.0628	-0.25631	-0.11186	-0.34291	-0.18985	0.158673	-0.03509	0.096116	-0.11247
NA	NA	0.192844	0.058485	-0.0269	0.117619	0.052661	0.156654	0.012882
-0.22774	0.086492	0.156616	-0.11293	-0.06746	0.128071	-0.0835	0.169821	-0.07548
-0.18695	-0.30903	-0.52377	-0.46831	-0.22876	0.191112	-0.19347	0.206192	-0.21112
0.068929	0.177778	0.195139	-0.35307	0.015936	0.350088	0.031021	0.211564	0.023478
NA	NA	-0.39604	-0.61802	-0.17438	0.301916	0.010696	0.312304	-0.08184
-0.15284	-0.06307	-0.16859	-0.19835	0.052085	0.335112	-0.1371	0.139785	-0.04251
-0.39095	-0.04141	-0.34372	-0.44819	-0.18785	0.222097	-0.18993	0.223022	-0.18889
-0.18671	-0.05759	-0.2517	-0.38328	0.0311	0.36736	-0.12732	0.133624	-0.04811

0.115267	0.311636	0.67727	0.734572	0.34098	0.230763	0.325364	0.209944	0.333172
-0.03596	-0.24156	NA	NA	-0.13816	0.149171	-0.04796	0.115393	-0.09306
-0.10853	0.086719	NA	NA	-0.02269	0.121554	-0.12992	0.19069	-0.0763
0.345026	-0.25559	NA	NA	-0.28507	0.166046	0.175767	0.164208	-0.05465
0.443295	0.04112	0.646252	0.198324	0.077331	0.182548	0.375218	0.25375	0.226275
0.551757	-0.16094	0.82988	0.38416	0.009072	0.266061	0.420624	0.269254	0.214848
0.508518	0.186386	0.452782	0.316519	0.18878	0.20378	0.283328	0.216539	0.236054
-0.23468	0.163978	NA	NA	-0.03733	0.211745	-0.01549	0.183382	-0.02641
0.27589	-0.04414	NA	NA	-0.04719	0.119936	0.238877	0.102198	0.095844
0.502892	0.236618	0.359974	0.265431	0.264624	0.02761	0.442993	0.074211	0.353808
0.047362	-0.07431	NA	NA	-0.07388	0.069309	0.059646	0.339096	-0.00712
0.15601	-0.02974	-0.03349	-0.5212	-0.22687	0.195029	-0.08726	0.165417	-0.15707
0.083782	0.086842	NA	NA	-0.12564	0.20352	0.032866	0.148165	-0.04639
0.296023	0.166894	NA	NA	0.02765	0.3572	0.229866	0.164465	0.128758
0.0669	0.156236	0.510789	-0.29714	-0.00054	0.189066	0.104309	0.232475	0.051885
0.257292	0.15895	0.568993	0.344466	-0.04089	0.299108	0.218837	0.249855	0.088972
0.112394	0.121892	0.788156	0.130405	0.038923	0.228612	0.205651	0.354873	0.122287
-0.07437	0.049564	-0.34276	-0.57223	0.016012	0.348152	-0.13088	0.146443	-0.05744
0.082786	0.104905	NA	NA	0.020375	0.235528	0.17725	0.137625	0.098813
0.151995	0.004932	-0.21373	-0.44168	-0.27578	0.25345	0.024392	0.227906	-0.1257
0.514325	-0.13999	NA	NA	0.014786	0.162368	0.334927	0.129879	0.174857
0.135507	0.163511	NA	NA	0.030108	0.176442	-0.08113	0.388325	-0.02551
-0.04103	0.027088	NA	NA	0.09876	0.220749	-0.05766	0.087437	0.02055
-0.20899	-0.40763	-0.30809	-0.48578	-0.1359	0.386322	-0.11978	0.182827	-0.12784
0.219326	0.112739	0.493246	0.314397	0.223115	0.243859	0.23177	0.172977	0.227443
0.12858	0.152232	0.577493	0.501635	0.125342	0.323522	0.203291	0.299869	0.164316
-0.09504	0.15472	-0.33888	0.209285	-0.00625	0.30432	-0.06051	0.242002	-0.03338
0.461725	0.122003	0.82893	0.140599	0.126503	0.246279	0.419107	0.250617	0.272805
0.081183	0.082755	-0.10908	0.605897	0.222832	0.255691	-0.10486	0.140453	0.058985
0.580198	0.237882	0.825975	0.326055	0.13487	0.201655	0.460237	0.252354	0.297554
0.005587	-0.05763	0.078146	-0.42476	-0.12465	0.191113	-0.10028	0.224803	-0.11247
0.066284	-0.12542	0.462043	-0.05264	-0.09214	0.234608	0.053192	0.240194	-0.01948
0.07248	0.32371	NA	NA	0.067041	0.322434	0.108284	0.345054	0.087663
0.351909	0.292307	0.624017	0.510531	0.313437	0.12895	0.333302	0.217805	0.323369
-0.14487	-0.16712	NA	NA	0.002463	0.157104	0.05774	0.373914	0.030102
0.557325	-0.17642	0.715087	-0.06531	0.05563	0.249742	0.420701	0.306619	0.238165
0.510805	-0.23249	0.789565	0.123488	-0.01282	0.222475	0.395407	0.265821	0.191291
0.1884	0.077571	NA	NA	0.143819	0.173659	0.252383	0.124742	0.198101
-0.00295	0.209358	NA	NA	0.107026	0.232143	0.187429	0.186507	0.147227
-0.1293	-0.29246	NA	NA	-0.29584	0.136477	-0.22438	0.098475	-0.26011
-0.09529	0.13589	NA	NA	0.022567	0.136188	0.090784	0.162416	0.056676
0.026858	0.362015	0.462086	0.247819	0.119967	0.26823	0.183633	0.389802	0.1518
0.011934	-0.10309	NA	NA	0.043966	0.193035	0.09641	0.282779	0.070188
0.212057	-0.08637	0.152248	0.336615	0.086381	0.200243	0.104301	0.156221	0.095341
0.013467	0.149397	0.059015	0.30373	-0.01625	0.278249	-0.0761	0.14518	-0.04618
-0.09529	0.091118	0.141258	-0.07742	-0.03355	0.242586	-0.05711	0.111615	-0.04533
NA	NA	0.0712	0.302879	0.02773	0.284743	0.003461	0.17424	0.015595
0.259319	0.320506	0.091072	0.364455	0.241599	0.162037	0.020935	0.197559	0.131267
0.248342	0.137643	NA	NA	0.345313	0.295249	0.130729	0.098308	0.238021
-0.14698	0.226866	-0.34914	0.113877	0.137298	0.059341	-0.05959	0.243474	0.038856
0.065455	-0.17592	NA	NA	-0.23916	0.043366	-0.04757	0.243943	-0.14337
0.005465	0.094535	NA	NA	0.066845	0.110793	-0.07343	0.094589	-0.00329

0.034444	0.16354	NA	NA	-0.06473	0.333764	-0.13244	0.152042	-0.09859
NA	NA	0.000327	0.21994	-0.06245	0.21812	0.009317	0.094177	-0.02657
0.147282	0.082474	0.703912	-0.05612	0.042256	0.064963	0.296974	0.285216	0.169615
-0.09495	0.056731	NA	NA	0.067214	0.128642	-0.08555	0.101181	-0.00917
-0.00358	0.219414	0.448021	0.160052	0.04448	0.137838	0.15438	0.215924	0.09943
0.189392	0.039782	0.65955	0.138661	0.059456	0.205225	0.277513	0.285035	0.168485
-0.07152	0.217693	-0.20485	0.082643	0.13928	0.186445	0.003452	0.263364	0.071366
0.075773	-0.17704	NA	NA	-0.03579	0.098969	0.032909	0.057238	-0.00144
0.160477	0.147708	-0.05957	-0.32603	-0.08343	0.213541	-0.00103	0.159653	-0.04223
-0.13537	0.161333	0.271455	0.293007	0.166199	0.222622	-0.10882	0.228348	0.02869
-0.00202	0.088893	NA	NA	-0.04154	0.19841	-0.21281	0.321117	-0.12718
-0.14128	0.003599	-0.57563	-0.20214	-0.12467	0.184013	-0.12194	0.268746	-0.1233
0.075367	0.109062	0.067523	-0.28722	0.031187	0.214669	-0.08077	0.152679	-0.02479
-0.19362	0.291377	-0.05681	0.039947	0.113597	0.274161	-0.07966	0.269971	0.016967
-0.04222	0.182979	NA	NA	0.110711	0.14194	-0.02178	0.189334	0.044465
0.259522	0.139369	-0.36959	-0.2707	-0.11465	0.281211	0.176711	0.324993	0.03103
-0.05003	0.117993	-0.32488	-0.20461	0.043262	0.200348	0.017046	0.281166	0.030154
-0.07381	0.186399	-0.21402	-0.4376	-0.27067	0.328854	-0.1674	0.256109	-0.21903
-0.14634	0.174478	0.220082	0.014869	0.026397	0.133264	-0.00049	0.192408	0.012953
0.280188	0.108947	-0.14724	0.374036	0.123871	0.323131	0.218649	0.213597	0.17126
-0.07603	0.090322	-0.17046	0.084986	0.128308	0.056132	0.003369	0.273415	0.065838
-0.03702	0.007296	-0.43426	0.180945	-0.01054	0.158553	-0.16884	0.194387	-0.08969
0.152442	-0.05406	0.396282	0.17614	0.188031	0.181582	0.400373	0.176307	0.294202
0.213019	0.126115	-0.53076	-0.01194	-0.01759	0.127971	-0.13591	0.338068	-0.07675
0.228371	0.173676	-0.51213	-0.30904	-0.01642	0.205831	-0.02257	0.332595	-0.01949
-0.0095	-0.27234	-0.28716	-0.43923	-0.33357	0.11629	-0.069	0.161727	-0.20128
0.06303	-0.25339	-0.29702	-0.34077	-0.18547	0.159949	-0.16946	0.176015	-0.17747
0.116671	-0.07043	NA	NA	0.135781	0.150613	0.210396	0.101498	0.173089
0.009887	-0.17056	0.478442	-0.04442	0.1125	0.259621	0.206476	0.197908	0.159488
-0.01762	0.122121	-0.22221	0.227334	0.020068	0.232623	-0.03398	0.137807	-0.00695
-0.17142	0.272826	-0.08607	0.246489	0.181065	0.174028	-0.06504	0.148852	0.058014
0.304619	0.119094	0.430463	0.163176	0.03947	0.126087	0.179877	0.232124	0.109673
0.012011	0.099606	0.524038	-0.47215	-0.10287	0.236989	0.103912	0.33083	0.00052
0.380789	0.244128	0.461319	0.092308	0.192728	0.139297	0.45283	0.09736	0.322779
0.308518	0.176242	NA	NA	0.062828	0.100901	0.340991	0.112567	0.201909
0.139079	-0.2005	0.002346	-0.20645	-0.20281	0.03187	0.125801	0.129918	-0.0385
0.215082	0.244945	0.409443	0.385501	0.218897	0.16829	0.239409	0.198181	0.229153
-0.08167	-0.2257	NA	NA	-0.15017	0.294063	-0.0908	0.051942	-0.12049
0.133777	0.102916	NA	NA	0.018909	0.179701	0.097929	0.423514	0.058419
-0.07867	-0.28664	-0.53755	-0.61418	-0.19235	0.347107	-0.2194	0.216734	-0.20588
-0.00496	0.29211	-0.00248	0.189119	0.082332	0.291564	-0.18743	0.219258	-0.05255
-0.02032	-0.11465	0.253789	-0.17325	-0.14149	0.034057	0.051703	0.154615	-0.0449
0.051398	-0.08495	0.21247	-0.51958	-0.16186	0.240785	0.104614	0.110818	-0.02862
0.121853	0.109653	0.266248	0.334759	0.00355	0.31602	0.019101	0.219354	0.011325
0.090738	-0.05648	0.211413	0.350758	0.076217	0.210145	0.194117	0.141471	0.135167
0.191463	-0.32031	0.217231	0.148467	-0.17152	0.215396	0.078703	0.149606	-0.04641
0.122473	0.154789	-0.12198	0.017802	0.088213	0.174024	0.037071	0.119719	0.062642
0.04949	0.098765	NA	NA	-0.06356	0.142291	0.000752	0.128781	-0.0314
-0.09381	-0.04776	NA	NA	-0.01588	0.059407	-0.0897	0.037503	-0.05279
0.311608	0.086313	0.573769	-0.30576	-0.19267	0.291669	0.231389	0.267007	0.019359
0.054246	0.266554	0.072603	0.395941	0.145964	0.239646	0.077069	0.076254	0.111516
0.167833	-0.31622	0.392594	-0.34852	-0.2001	0.156758	0.163423	0.163432	-0.01834

0.003383	0.01134	-0.12238	0.180418	0.021148	0.136946	-0.04119	0.124785	-0.01002
0.103054	0.094155	0.082117	-0.0958	-0.01004	0.083935	0.039278	0.109691	0.014618
0.054987	-0.12249	0.043199	-0.61766	-0.12637	0.374785	0.049302	0.047834	-0.03854
-0.02244	0.201746	-0.25187	-0.14824	-0.08435	0.201668	-0.00377	0.180269	-0.04406
0.20231	0.166857	0.105704	-0.17672	-0.12989	0.206046	0.005061	0.193795	-0.06241
0.029275	0.148804	-0.24527	0.341395	0.05525	0.247769	-0.0202	0.212061	0.017525
-0.0208	-0.05487	-0.09993	0.010548	-0.22419	0.247157	-0.05511	0.154537	-0.13965
-0.04217	-0.02722	NA	NA	-0.19254	0.141435	-0.0341	0.204803	-0.11332
-0.0664	0.032802	0.350198	-0.18711	-0.06143	0.183831	-0.00173	0.237633	-0.03158
-0.20097	0.327087	-0.39522	-0.01208	0.048363	0.24514	-0.23777	0.114957	-0.0947
0.145611	0.044778	0.047948	-0.26556	-0.22268	0.187549	0.097231	0.056498	-0.06273
-0.24551	-0.28284	-0.25754	-0.35161	-0.18153	0.174329	-0.17416	0.106833	-0.17785
0.087777	-0.39033	0.091713	-0.10512	-0.22506	0.127731	-0.04098	0.198845	-0.13302
0.10213	-0.18043	-0.17619	0.545786	0.053287	0.336665	-0.12962	0.163378	-0.03817
0.091354	0.042627	NA	NA	-0.17801	0.254586	0.127639	0.033005	-0.02518
0.135756	-0.30525	-0.43817	0.041232	-0.11803	0.163501	-0.15608	0.244417	-0.13706
0.022521	0.146222	-0.17092	-0.06848	-0.0245	0.16007	-0.02604	0.117935	-0.02527
-0.192	0.092604	-0.09694	0.116254	0.280431	0.333279	-0.01736	0.233719	0.131536
-0.29064	0.001952	-0.13216	0.13046	-0.0004	0.150699	-0.22891	0.24346	-0.11465
0.128631	-0.12703	NA	NA	-0.21092	0.208973	-0.07671	0.239532	-0.14382
-0.02279	-0.22362	NA	NA	-0.11986	0.175047	-0.14587	0.265529	-0.13286
0.101399	-0.33064	-0.06799	-0.02191	-0.14224	0.17177	-0.00028	0.208434	-0.07126
-0.12524	-0.05751	-0.07096	-0.42359	-0.08591	0.240997	-0.14569	0.081561	-0.1158
-0.01085	-0.06667	0.07183	-0.53815	-0.1466	0.395873	-0.12803	0.184122	-0.13732
0.026304	-0.2509	-0.29389	-0.51279	-0.30249	0.169477	-0.15433	0.205493	-0.22841
-0.0137	0.071334	-0.39699	-0.29856	-0.04828	0.261849	-0.08909	0.238267	-0.06868
-0.03183	0.120425	0.222465	0.02555	-0.03972	0.116015	-0.03642	0.160499	-0.03807
0.070072	-0.09867	NA	NA	-0.13521	0.135458	-0.04641	0.325206	-0.09081
-0.0585	0.079916	NA	NA	-0.14868	0.21169	-0.17358	0.304475	-0.16113
-0.1543	-0.36597	0.182887	-0.59634	-0.3001	0.287488	-0.05579	0.27856	-0.17795
-0.10707	0.15997	0.492197	0.087998	0.101304	0.071265	0.262506	0.2266	0.181905
0.13518	0.088727	-0.07153	-0.09594	0.013007	0.296278	0.107814	0.288088	0.06041
0.21439	0.021479	0.756747	-0.04853	0.005347	0.172376	0.159087	0.400884	0.082217
0.100765	0.033673	NA	NA	-0.09546	0.145347	0.0707	0.192617	-0.01238
0.181931	0.060348	NA	NA	0.038644	0.146335	-0.01828	0.170677	0.010183
0.102228	0.074797	NA	NA	0.104008	0.261759	-0.05371	0.288877	0.025149
0.436848	0.0104	0.581228	0.170608	0.031219	0.299172	0.320562	0.240675	0.17589
-0.26182	-0.22687	-0.33308	-0.31109	-0.23645	0.076112	-0.17331	0.16922	-0.20488
0.151093	-0.06147	NA	NA	-0.05287	0.254295	-0.04928	0.279229	-0.05108
0.086516	0.1509	0.095003	-0.18669	-0.14146	0.166584	-0.02269	0.220301	-0.08208
-0.18968	0.108526	-0.1595	-0.19606	-0.00956	0.309686	-0.18101	0.236909	-0.09529
0.344078	0.634662	0.443107	0.616317	0.4069	0.238622	0.338255	0.15945	0.372578
-0.30642	0.047679	0.020823	0.102603	-0.04721	0.426054	-0.11437	0.404586	-0.08079
0.065069	-0.34334	NA	NA	-0.08858	0.172661	0.150219	0.120538	0.030821
0.002984	0.221266	NA	NA	0.323642	0.241895	0.161688	0.358315	0.242665
0.03916	0.095867	0.35321	-0.36412	-0.10375	0.18383	0.068021	0.272231	-0.01787
-0.02175	0.086309	-0.17249	-0.13099	-0.00022	0.244928	-0.10344	0.329523	-0.05183
-0.00939	0.049588	NA	NA	0.015602	0.190123	-0.08677	0.18738	-0.03558
-0.22035	0.087703	0.056737	-0.00777	0.0519	0.047477	-0.12988	0.147197	-0.03899
-0.0673	0.130279	NA	NA	-0.07249	0.331739	0.078212	0.142681	0.002863
-0.16535	-0.19396	-0.01182	-0.53892	-0.25942	0.19575	-0.16152	0.104316	-0.21047
0.050871	-0.19219	NA	NA	0.029893	0.252073	-0.07167	0.102239	-0.02089

-0.12937	-0.16047	-0.68829	-0.22203	0.046907	0.27018	-0.11498	0.333526	-0.03404
-0.00475	-0.15431	NA	NA	-0.03804	0.146431	0.03045	0.174596	-0.0038
-0.06291	-0.4268	NA	NA	-0.19067	0.193313	-0.18062	0.245397	-0.18564
-0.08757	-0.49695	-0.66624	-0.53399	-0.3616	0.179696	-0.29309	0.294429	-0.32735
-0.06137	-0.18014	0.022041	-0.25376	-0.21472	0.107059	0.049848	0.090121	-0.08244
-0.10971	-0.02827	-0.22082	-0.21278	-0.16608	0.17623	-0.10592	0.105469	-0.136
NA	NA	-0.16643	0.138358	0.228607	0.363014	0.007518	0.26923	0.118062
-0.06179	0.009426	0.377316	0.288101	-0.09157	0.278529	-0.01957	0.220368	-0.05557
-0.18699	0.164419	0.071483	0.096382	0.013081	0.184619	-0.14309	0.113442	-0.06501
-0.07379	0.155439	NA	NA	0.068096	0.080905	0.037425	0.251875	0.05276
0.005902	0.047241	NA	NA	-0.12918	0.145454	0.058304	0.313649	-0.03544
-0.13051	0.294019	-0.47655	0.443272	0.05841	0.391264	-0.23382	0.171213	-0.0877
0.061537	0.016926	0.053754	0.296311	0.081859	0.1438	0.122734	0.089277	0.102297
-0.00986	0.000434	0.166356	-0.178	-0.0571	0.253452	0.028286	0.108337	-0.01441
-0.32	-0.24323	-0.4492	-0.53701	-0.22229	0.229716	-0.2618	0.155695	-0.24204
-0.22431	0.007978	-0.32387	0.152464	-0.05189	0.174837	-0.18486	0.126484	-0.11837
-0.12929	-0.2953	-0.24074	-0.47109	-0.21361	0.269053	-0.19722	0.096678	-0.20541
-0.36875	-0.24744	-0.25017	-0.46257	-0.21543	0.148638	-0.25526	0.135657	-0.23535
-0.00599	0.247592	-0.45246	0.343724	0.01548	0.353733	-0.16928	0.227121	-0.0769
-0.34762	0.198669	-0.74845	0.102591	-0.05285	0.230863	-0.38649	0.263773	-0.21967
0.006969	0.141952	-0.37239	-0.00033	-0.06223	0.246629	-0.09465	0.238119	-0.07844
-0.03138	0.207659	0.290366	0.355109	0.101217	0.222789	0.017718	0.211733	0.059468
-0.03635	0.196946	NA	NA	-0.0613	0.406422	0.085648	0.180109	0.012176
-0.044	0.100549	0.380578	-0.35372	-0.09317	0.252692	0.046987	0.336698	-0.02309
-0.14995	-0.15046	-0.06642	-0.06522	-0.0745	0.05217	-0.21135	0.143267	-0.14292
0.133732	0.204729	0.600166	0.372745	0.091572	0.239067	0.24573	0.250907	0.168651
0.089287	0.047647	0.061323	-0.25242	0.011029	0.178727	0.091972	0.178209	0.0515
-0.18296	0.652863	NA	NA	0.423926	0.317018	-0.10897	0.085755	0.15748
-0.11196	-0.16192	-0.07121	-0.51763	-0.07553	0.23413	-0.10259	0.068291	-0.08906
-0.29393	-0.3001	0.041434	0.206324	-0.0132	0.174545	-0.17285	0.257887	-0.09303
-0.17533	-0.31498	NA	NA	-0.16505	0.109687	-0.11892	0.183066	-0.14199
-0.27446	0.153483	-0.16148	0.11472	0.067284	0.241454	-0.06099	0.175069	0.003145
0.074238	0.318238	-0.27402	0.132795	0.25028	0.151628	-0.2289	0.34114	0.010691
0.071007	-0.21496	0.102681	-0.19543	-0.15134	0.08292	-0.0434	0.150951	-0.09737
0.160452	-0.3907	-0.00736	-0.59307	-0.31506	0.232816	0.042148	0.179864	-0.13646
0.00291	0.03419	0.225821	-0.32189	-0.14317	0.179429	0.050425	0.242987	-0.04637
-0.09354	-0.07194	-0.20339	-0.27842	-0.12034	0.126902	0.050225	0.234579	-0.03506
-0.09226	-0.10988	0.456755	-0.04942	-0.18492	0.260147	-0.04966	0.375376	-0.11729
0.027832	0.244403	-0.30648	0.024676	-0.07574	0.289718	-0.12088	0.15598	-0.09831
0.018417	0.272813	0.725358	0.248944	0.08532	0.255481	0.14252	0.411321	0.11392
-0.08547	-0.17345	NA	NA	-0.14658	0.26227	-0.0758	0.046602	-0.11119
0.012733	0.189541	0.212097	-0.35647	-0.09417	0.259197	0.059732	0.170695	-0.01722
0.101711	-0.50398	-0.10267	-0.39201	-0.2786	0.194065	0.039998	0.121958	-0.1193
-0.31564	-0.36451	-0.45751	-0.37825	-0.18572	0.349838	-0.22688	0.227369	-0.2063
-0.05988	0.11538	-0.02099	0.143039	-0.05682	0.245285	-0.16769	0.152539	-0.11225
-0.0345	-0.29771	0.007505	-0.43319	-0.27402	0.209805	-0.04705	0.042487	-0.16054
0.169573	0.062873	-0.4413	-0.37437	0.055713	0.314409	0.003518	0.298822	0.029615
-0.1113	-0.2359	0.325184	0.180418	-0.09689	0.208932	0.092916	0.191738	-0.00199
-0.0077	0.213462	NA	NA	0.146779	0.280148	-0.11927	0.295433	0.013754
-0.16199	-0.19141	0.200698	-0.43667	-0.22654	0.307538	0.017466	0.164589	-0.10454
-0.20185	-0.1696	-0.67805	0.101528	-0.0597	0.312474	-0.21237	0.331367	-0.13603
0.363452	-0.21552	0.578464	-0.02617	-0.07571	0.175039	0.274098	0.212999	0.099193

0.219932	0.155529	NA	NA	-0.14826	0.405564	-0.03205	0.302634	-0.09015
-0.22948	0.280057	NA	NA	0.073492	0.302148	-0.15735	0.150854	-0.04193
0.038908	-0.18076	0.134077	0.048816	0.057894	0.183787	0.117481	0.093156	0.087688
0.000746	0.328311	0.466575	0.253903	0.168294	0.205009	0.20104	0.182147	0.184667
-0.1312	-0.0365	NA	NA	-0.06591	0.181641	-0.11288	0.184486	-0.08939
0.065126	0.077197	-0.18011	-0.19575	0.117323	0.241673	-0.02307	0.102414	0.047125
-0.02306	-0.18762	-0.40771	-0.15255	-0.16709	0.098821	-0.17786	0.165179	-0.17247
0.027099	0.076722	NA	NA	-0.03076	0.150431	0.08326	0.240572	0.026248
0.129211	0.12519	NA	NA	-0.01256	0.241128	0.161002	0.240471	0.074223
-0.04047	0.051668	-0.17414	-0.07666	-0.00746	0.254691	0.054284	0.251598	0.023413
0.169754	0.174529	NA	NA	0.289833	0.27503	0.220137	0.357536	0.254985
0.079462	0.355618	-0.39958	0.233848	0.101217	0.217441	-0.10183	0.235843	-0.00031
-0.0604	-0.00388	0.052452	0.18929	0.007983	0.194399	-0.11332	0.164972	-0.05267
-0.02499	-0.02073	NA	NA	-0.08507	0.381464	-0.01772	0.094018	-0.0514
-0.09521	-0.29741	NA	NA	-0.08182	0.192556	-0.09311	0.401277	-0.08747
0.000858	0.140269	NA	NA	-0.0235	0.355858	0.093481	0.165363	0.034989
0.070829	-0.02789	0.500405	0.520015	-0.01256	0.324752	0.198756	0.232082	0.0931
-0.10901	0.490602	NA	NA	0.348717	0.11944	-0.08478	0.06192	0.131969
-0.14509	0.082998	NA	NA	-0.1792	0.236877	-0.17743	0.197936	-0.17831
-0.08919	0.258812	NA	NA	0.050215	0.211647	-0.09522	0.149857	-0.0225
0.054561	-0.31651	-0.38652	-0.23257	-0.18843	0.121067	-0.02744	0.250261	-0.10794
0.019827	0.110949	-0.16536	-0.01546	0.016997	0.133896	-0.1255	0.148636	-0.05425
0.081052	0.369708	NA	NA	0.035513	0.234506	-0.08438	0.316142	-0.02443
0.134078	-0.4597	NA	NA	-0.2092	0.219828	-0.06395	0.215669	-0.13657
0.04329	0.121979	-0.00119	-0.04827	-0.0606	0.311809	0.022183	0.375914	-0.01921
-0.19429	-0.06984	NA	NA	-0.16065	0.218733	-0.15971	0.051205	-0.16018
-0.31192	0.179839	NA	NA	0.037241	0.301104	-0.07072	0.185847	-0.01674
-0.01693	0.040522	0.299001	-0.40046	-0.01401	0.298482	0.186157	0.184503	0.086073
-0.09948	-0.0288	-0.56298	-0.05924	-0.00775	0.112107	-0.23356	0.264368	-0.12066
0.097288	-0.08814	-0.14248	0.190151	-0.16394	0.278145	-0.12752	0.188269	-0.14573
-0.47482	-0.03365	NA	NA	0.108952	0.425898	-0.447	0.212384	-0.16902
0.014651	-0.21088	0.240384	-0.09569	-0.19791	0.173637	0.008779	0.159029	-0.09456
0.220408	0.233795	0.641751	0.335581	0.114736	0.20514	0.242089	0.27808	0.178412
-0.17363	-0.42825	-0.32494	-0.5354	-0.33826	0.177344	-0.14377	0.202598	-0.24102
0.24259	-0.06943	0.122685	0.19059	0.108562	0.297945	0.166725	0.279947	0.137643
0.028805	0.198682	NA	NA	0.15997	0.130631	0.02973	0.181106	0.09485
-0.0341	0.142316	NA	NA	0.011092	0.092008	-0.01844	0.180973	-0.00367
-0.22026	-0.37854	NA	NA	-0.40171	0.169172	-0.37293	0.190823	-0.38732
0.061227	0.20458	-0.05902	0.257973	0.057673	0.255394	0.009972	0.316689	0.033822
0.211498	0.037571	0.565916	0.121133	0.108081	0.114923	0.218468	0.203974	0.163275
0.034725	0.062358	-0.30182	0.350505	-0.05454	0.331371	-0.17283	0.150072	-0.11369
0.272194	-0.10807	0.342951	0.172714	-0.17683	0.217039	0.118814	0.21414	-0.02901
-0.0867	0.049579	0.336008	-0.02496	-0.07755	0.1845	-0.07658	0.306344	-0.07706
-0.1063	0.159877	0.070235	0.25781	0.058	0.250875	-0.19932	0.277154	-0.07066
0.025645	0.260063	NA	NA	0.001228	0.283855	0.074359	0.196182	0.037794
0.142337	0.134497	0.354405	0.270594	0.148214	0.288347	0.095027	0.249428	0.121621
0.010947	-0.38461	-0.61734	-0.45172	-0.43043	0.12501	-0.19242	0.36993	-0.31142
0.089829	0.038591	NA	NA	0.033643	0.302799	-0.09462	0.149874	-0.03049
0.088014	-0.23882	-0.54791	-0.26504	0.001141	0.251696	0.11377	0.510377	0.057456
-0.11672	-0.32586	-0.68676	-0.50059	-0.25105	0.267363	-0.24999	0.251738	-0.25052
0.01782	0.242955	-0.44506	0.31045	0.01739	0.263766	-0.17744	0.180912	-0.08002
0.198829	0.331362	NA	NA	-0.1744	0.375799	-0.10321	0.242727	-0.1388

0.341942	0.20839	0.48372	0.425847	0.223641	0.182117	0.199388	0.295845	0.211515
0.027778	0.122355	NA	NA	0.297815	0.156423	0.044833	0.319778	0.171324
0.405311	0.383105	0.601603	0.706992	0.484128	0.156218	0.363118	0.183677	0.423623
NA	NA	0.507633	-0.49626	-0.33245	0.175021	0.008128	0.347682	-0.16216
0.287039	0.083661	0.273776	0.177637	0.167963	0.166506	0.266555	0.128682	0.217259
0.458505	0.299258	NA	NA	0.090656	0.339732	0.218618	0.366672	0.154637
0.293674	0.181022	0.439271	0.380865	0.221316	0.09642	0.218301	0.240559	0.219809
0.283631	-0.18631	NA	NA	-0.17541	0.170674	0.004807	0.20302	-0.0853
-0.08026	0.210323	-0.01573	0.50857	0.147154	0.254606	-0.07098	0.221346	0.038089
0.184099	0.010198	NA	NA	-0.05409	0.192072	-0.10679	0.227675	-0.08044
-0.00158	0.17924	-0.01222	0.001479	-0.00279	0.221554	-0.02737	0.151863	-0.01508
0.220019	-0.10044	NA	NA	-0.20699	0.17585	0.009941	0.201416	-0.09853
0.132228	-0.38413	-0.10188	-0.5194	-0.22511	0.341305	0.139416	0.192102	-0.04285
0.162427	0.2592	-0.12916	0.067802	0.039142	0.198278	0.0607	0.131146	0.049921
0.099543	0.211415	NA	NA	0.140929	0.094905	0.082216	0.021122	0.111572
0.142641	-0.04813	-0.22714	0.204656	0.030786	0.12336	-0.13981	0.253184	-0.05451
0.226078	0.109324	0.18507	0.495228	0.140218	0.263374	0.06348	0.165084	0.101849
0.390893	-0.08703	0.636395	-0.2669	-0.08096	0.18198	0.368338	0.197289	0.143686
0.262632	-0.04394	0.284911	0.374023	0.046727	0.243425	0.054997	0.263776	0.050862
-0.09916	-0.05027	-0.05459	-0.39564	-0.0885	0.213312	-0.02442	0.18976	-0.05646
-0.12148	-0.29992	0.147767	-0.15932	-0.14313	0.117792	-0.10188	0.173105	-0.12251
-0.10691	0.069148	-0.17562	-0.07598	0.067665	0.103658	-0.03189	0.132406	0.017886
0.156008	0.13183	NA	NA	-0.04883	0.165922	0.147513	0.047433	0.049343
-0.10733	-0.19584	0.231742	-0.34294	-0.24686	0.078628	0.01151	0.191342	-0.11768
-0.10635	0.336334	-0.24457	0.031023	0.132555	0.211505	-0.05751	0.160136	0.037521
0.126763	0.188313	-0.17838	-0.11419	0.055706	0.294536	0.03734	0.110597	0.046523
0.219614	0.004342	-0.22435	-0.17858	-0.12324	0.262276	-0.14019	0.242859	-0.13172
-0.12584	0.045191	NA	NA	-0.10715	0.137154	0.229891	0.272374	0.061369
-0.07533	0.113074	0.148482	-0.24106	0.094318	0.215013	-0.02049	0.183237	0.036913
0.301535	-0.04806	0.504038	0.194472	0.05897	0.090948	0.252201	0.17473	0.155585
-0.00358	0.095437	0.007306	-0.05117	0.021273	0.317463	0.029551	0.290361	0.025412
-0.09263	0.083347	-0.07319	-0.50169	-0.18403	0.350196	-0.2	0.224915	-0.19202
0.134187	0.14583	NA	NA	-0.16142	0.226415	0.074175	0.054696	-0.04362
-0.15008	-0.16469	-0.38033	0.086086	-0.03832	0.12856	-0.2059	0.144125	-0.12211
0.042726	-0.12884	-0.01802	-0.10941	-0.22511	0.101207	0.025676	0.047727	-0.09972
-0.10515	0.357248	NA	NA	-0.01857	0.455561	-0.07405	0.058966	-0.04631
-0.07722	-0.22993	0.251535	-0.13977	-0.03855	0.272403	-0.08343	0.213695	-0.06099
0.151215	-0.15531	-0.19399	-0.19295	-0.05887	0.174997	0.093242	0.176724	0.017185
0.214848	0.130235	NA	NA	-0.13052	0.332604	-0.0123	0.358321	-0.07141
0.070594	-0.2424	-0.20463	-0.47359	-0.15728	0.217496	-0.06197	0.151031	-0.10962
-0.08573	0.112162	-0.54087	-0.30636	-0.0302	0.299933	-0.106	0.260714	-0.0681
-0.08051	-0.04712	-0.15573	-0.47808	-0.25646	0.163116	-0.14867	0.121008	-0.20257
0.127624	0.210874	NA	NA	0.000551	0.19954	0.13235	0.128866	0.066451
-0.0314	0.093074	-0.17923	-0.29299	0.061963	0.263951	-0.01592	0.254246	0.023023
0.116588	-0.12135	0.466793	-0.12036	-0.03739	0.18398	0.209611	0.171739	0.086112
0.188618	0.094345	0.468123	0.180033	0.002882	0.188193	0.195954	0.191253	0.099418
-0.02607	0.087985	NA	NA	0.124707	0.051931	0.215364	0.341434	0.170035
0.050717	0.255213	0.264826	0.028952	0.021038	0.249918	0.098713	0.184569	0.059875
-0.05322	0.144986	-0.07772	0.050964	-0.01524	0.136321	0.028217	0.109375	0.006489
0.081087	0.038214	-0.2432	-0.09157	-0.11565	0.176511	-0.12586	0.200665	-0.12075
-0.00891	0.340246	0.453969	-0.14647	0.112091	0.196984	0.139336	0.244051	0.125713
0.020155	-0.19167	NA	NA	-0.2406	0.345335	-0.09644	0.126241	-0.16852

-0.04138	-0.27921	NA	NA	-0.19973	0.340696	0.028837	0.101431	-0.08545
0.224559	0.091319	-0.17092	-0.17503	0.047558	0.204252	-0.05011	0.23844	-0.00127
0.113559	0.211639	-0.54393	-0.43743	-0.15091	0.294252	-0.1389	0.245668	-0.14491
0.303339	0.238176	0.473328	0.241908	0.22272	0.05773	0.351208	0.111453	0.286964
-0.18471	-0.0192	0.387273	0.292288	-0.10982	0.269625	-0.08121	0.269961	-0.09551
0.060262	0.054444	0.127953	0.290858	0.132897	0.136434	0.133307	0.135165	0.133102
-0.1642	-0.32768	0.326735	0.036888	-0.0726	0.182538	0.00039	0.219416	-0.0361
-0.37925	0.254005	NA	NA	-0.00069	0.296862	-0.19509	0.274723	-0.09789
0.0283	-0.01326	NA	NA	-0.09074	0.179029	-0.07652	0.198622	-0.08363
0.101558	0.24577	-0.26803	-0.05779	0.0363	0.149662	-0.05207	0.183674	-0.00788
0.0335	0.108187	-0.00703	0.016078	0.154798	0.122588	0.062528	0.074131	0.108663
-0.02377	0.179685	-0.49361	0.031535	-0.07637	0.237003	-0.10985	0.274093	-0.09311
0.18592	0.224914	NA	NA	0.214017	0.134661	0.324745	0.176381	0.269381
0.049161	0.399276	0.693913	0.114387	0.224885	0.154724	0.329576	0.240051	0.27723
-0.19476	-0.20365	NA	NA	-0.20565	0.101984	-0.09661	0.147592	-0.15113
-0.19523	0.285143	-0.12391	0.080744	0.103888	0.170864	-0.04074	0.208888	0.031574
0.059406	0.077385	NA	NA	0.035824	0.19011	-0.02627	0.107582	0.004776
-0.21136	0.287801	-0.18901	0.297067	0.078799	0.306542	-0.23067	0.030567	-0.07594
0.430251	0.277951	0.674109	0.265819	0.070722	0.256037	0.406996	0.182309	0.238859
-0.07089	0.204145	0.177921	-0.25445	-0.06257	0.196005	0.076865	0.113693	0.007149
-0.02067	-0.35271	NA	NA	-0.05306	0.214373	-0.08348	0.135026	-0.06827
-0.07474	0.259142	NA	NA	0.052975	0.235236	0.059036	0.183511	0.056006
-0.05554	-0.22774	-0.30205	-0.05413	-0.07943	0.210489	-0.11741	0.130491	-0.09842
0.196262	-0.14393	NA	NA	-0.07669	0.102318	0.086118	0.197651	0.004713
-0.11554	-0.36628	-0.01559	-0.22783	-0.11921	0.176068	-0.07756	0.122675	-0.09838
-0.1319	-0.35307	NA	NA	-0.16798	0.232131	-0.17858	0.206654	-0.17328
-0.03698	0.025069	-0.03682	-0.21093	-0.02665	0.257993	-0.04572	0.194579	-0.03618
0.191345	-0.23798	NA	NA	-0.15443	0.16265	-0.05904	0.18656	-0.10674
-0.01824	0.121499	-0.01943	-0.29062	-0.0806	0.200819	-0.11176	0.157554	-0.09618
-0.00546	0.116183	0.062182	0.047936	0.063246	0.240056	-0.03377	0.184085	0.01474
0.009495	0.011538	0.473802	0.100238	0.072471	0.065297	0.081603	0.22256	0.077037
0.04982	0.143323	NA	NA	0.159335	0.123366	-0.06909	0.103762	0.045123
-0.15606	0.188692	NA	NA	-0.10724	0.327114	-0.04027	0.134265	-0.07375
0.142626	0.157474	-0.21223	-0.29971	0.017875	0.214448	0.050262	0.209258	0.034068
-0.06511	-0.40169	NA	NA	-0.20708	0.289874	-0.0283	0.248092	-0.11769
-0.01112	0.217281	0.284483	-0.41781	-0.14458	0.295718	0.056787	0.29735	-0.0439
0.113558	0.200312	NA	NA	0.000374	0.177078	-0.06459	0.212649	-0.03211
0.291511	0.034091	0.505196	0.520015	0.331999	0.210102	0.241005	0.269875	0.286502
-0.00835	-0.08711	NA	NA	-0.06907	0.448836	0.045561	0.301506	-0.01175
-0.23758	0.105892	NA	NA	0.06352	0.169759	-0.11401	0.174061	-0.02525
-0.14853	-0.10997	0.436196	-0.22904	-0.00107	0.189324	0.003579	0.33412	0.001254
-0.41637	0.005106	-0.5508	0.209599	-0.05317	0.18033	-0.30665	0.176756	-0.17991
0.081902	0.115709	NA	NA	0.008495	0.192356	0.114636	0.185797	0.061565
-0.00035	0.277596	0.422399	0.278246	0.234164	0.0518	0.238601	0.176097	0.236383
0.151033	0.235843	NA	NA	0.014628	0.204671	0.178953	0.057329	0.09679
-0.01578	0.075002	0.335512	0.075074	0.048158	0.19937	0.14606	0.139438	0.097109
-0.34091	-0.25456	-0.09833	-0.23506	-0.1619	0.210166	-0.26962	0.127385	-0.21576
0.01048	0.123881	0.20246	0.336813	0.11737	0.252101	-0.04799	0.285291	0.034689
0.36899	0.25796	NA	NA	0.168346	0.232763	0.188566	0.289803	0.178456
-0.15208	0.209377	NA	NA	-0.22284	0.335063	-0.25589	0.225898	-0.23937
-0.22259	0.224184	NA	NA	0.016906	0.17178	-0.21293	0.096843	-0.09801
0.137743	0.041076	0.014726	0.103439	0.015116	0.18944	0.143868	0.305781	0.079492

-0.02925	0.026125	0.077982	0.321211	-0.11415	0.300847	-0.16516	0.23977	-0.13965
0.136771	0.245176	0.490727	0.328834	0.171093	0.128326	0.280239	0.173866	0.225666
0.08158	-0.18072	0.250647	0.057806	-0.06185	0.16204	0.058422	0.143955	-0.00171
-0.20152	-0.40633	-0.05547	-0.35614	-0.23504	0.142603	-0.03289	0.13537	-0.13396
0.000155	-0.14744	0.087599	-0.22389	0.047955	0.290429	0.033911	0.093695	0.040933
-0.08096	0.211299	NA	NA	0.033461	0.190106	-0.13694	0.119833	-0.05174
0.006337	0.069837	NA	NA	-0.07289	0.130815	-0.11557	0.202324	-0.09423
0.077453	0.208068	-0.05365	-0.41726	-0.10721	0.312697	0.131852	0.217859	0.012318
0.077955	0.029877	NA	NA	0.179546	0.310402	-0.11513	0.165677	0.032207
-0.09514	0.134368	-0.76812	-0.10355	-0.15791	0.205424	-0.14856	0.307835	-0.15324
0.04576	0.041094	-0.43061	-0.37302	0.048245	0.327621	-0.00443	0.275319	0.021908
-0.06476	-0.26318	-0.33168	-0.35065	-0.02362	0.259663	0.029556	0.23331	0.002966
-0.40032	0.175732	-0.62785	0.309873	0.028569	0.234677	-0.24226	0.230317	-0.10685
-0.00169	0.164072	-0.04892	0.14913	0.074914	0.222045	0.061132	0.140153	0.068023
-0.03121	-0.36804	-0.35649	-0.33495	-0.22758	0.12974	-0.18604	0.239656	-0.20681
-0.396	0.264662	-0.72663	0.27301	-0.04312	0.277925	-0.34238	0.222706	-0.19275
0.009156	-0.04762	0.066853	0.316375	0.082402	0.390988	0.080694	0.123548	0.081548
0.00731	0.143893	-0.65103	0.550546	0.017585	0.339712	-0.09918	0.272194	-0.0408
0.070086	0.209787	0.140673	0.080305	0.162009	0.196026	-0.03558	0.195514	0.063216
-0.07612	-0.04317	0.022979	0.077303	0.023102	0.214461	-0.08063	0.211581	-0.02876
0.347405	0.14207	0.030315	-0.5047	-0.16783	0.21074	-0.00703	0.201614	-0.08743
-0.13159	0.093483	-0.60602	-0.14204	-0.10633	0.197338	-0.22616	0.250567	-0.16625
-0.31751	-0.14538	-0.45202	-0.50289	-0.22787	0.274338	-0.24416	0.229485	-0.23602
-0.08217	0.135452	0.395827	0.380193	0.123485	0.285928	0.066626	0.246469	0.095055
-0.10255	-0.01967	-0.58937	0.21579	-0.10088	0.257103	-0.22101	0.18487	-0.16095
0.334484	-0.46931	0.36896	-0.28236	-0.25904	0.139458	0.264106	0.205578	0.002532
0.117313	0.08309	NA	NA	0.040286	0.275863	0.12646	0.09763	0.083373
0.054592	0.09602	0.113491	-0.06739	0.17737	0.2581	0.179379	0.151263	0.178375
0.22209	-0.01997	-0.08003	-0.16972	-0.19996	0.191518	0.026513	0.166694	-0.08672
0.40095	0.312657	0.643573	0.335744	0.178086	0.12403	0.270582	0.310306	0.224334
0.208441	-0.05759	0.211064	-0.36356	-0.08496	0.28901	0.106809	0.1868	0.010925
0.069317	0.098104	NA	NA	0.142749	0.158533	0.0664	0.188107	0.104575
-0.03874	0.082387	0.237595	0.35453	0.11313	0.214106	0.076618	0.119129	0.094874
-0.02611	0.186364	NA	NA	-0.06857	0.22721	-0.06644	0.150438	-0.06751
0.104451	0.047419	0.120776	-0.3508	0.027868	0.238918	0.003509	0.154562	0.015688
0.168101	-0.17881	0.317009	-0.21492	-0.07035	0.174152	0.044158	0.194659	-0.01309
0.177495	-0.17125	0.133146	0.087407	-0.11545	0.312003	0.14194	0.257096	0.013243
-0.33032	0.21784	-0.59466	-0.22283	0.061453	0.259516	-0.08656	0.351331	-0.01255
0.126125	-0.12199	-0.52718	-0.48305	-0.26888	0.21354	-0.22141	0.300296	-0.24514
-0.16833	-0.45006	0.015302	-0.3594	-0.1799	0.26095	-0.03662	0.160295	-0.10826
0.305249	0.150459	0.418474	-0.43664	0.172159	0.416595	0.275071	0.228313	0.223615
0.06372	0.12773	0.107403	-0.47744	-0.06771	0.247142	-0.09756	0.181167	-0.08263
0.244652	0.116553	-0.59126	-0.16503	0.080847	0.262011	-0.09272	0.301047	-0.00594
0.039478	0.153652	-0.29994	-0.39493	-0.04638	0.196979	-0.10193	0.191343	-0.07416
-0.05675	-0.43733	-0.09729	-0.51078	-0.23066	0.248087	-0.0457	0.14344	-0.13818
-0.02164	0.143329	-0.26529	0.26476	0.295133	0.248386	-0.0447	0.278267	0.125215
0.093634	0.032288	-0.5331	-0.44089	-0.056	0.254504	-0.06252	0.247194	-0.05926
0.07384	0.171202	-0.33857	-0.50741	0.006472	0.292787	0.009677	0.177142	0.008075
-0.17693	-0.14921	-0.62321	-0.41519	0.010905	0.362025	-0.13546	0.256899	-0.06228
-0.14715	-0.2065	-0.29361	-0.40213	-0.12106	0.185505	-0.2432	0.2087	-0.18213
0.114256	0.245275	-0.4889	0.410282	0.002373	0.309995	-0.0276	0.252608	-0.01261
0.073206	-0.03421	-0.5755	-0.49157	-0.26494	0.151654	-0.14092	0.263569	-0.20293

-0.23072	-0.42875	-0.64488	-0.65643	-0.23938	0.331899	-0.16447	0.252623	-0.20192
0.018316	0.210581	0.106812	-0.20166	-0.02103	0.154438	-0.0216	0.258837	-0.02132
-0.03457	-0.0979	0.101012	-0.14101	-0.09768	0.169845	0.077148	0.123548	-0.01027
0.005341	-0.06121	-0.27078	-0.02864	-0.06109	0.146036	0.008779	0.197039	-0.02615
0.053061	0.271172	-0.21288	-0.42563	0.035659	0.291781	-0.02523	0.171502	0.005212
0.17503	0.066591	NA	NA	0.018472	0.152366	0.100152	0.18724	0.059312
0.140326	-0.11391	-0.10294	-0.45352	-0.06979	0.310885	0.147587	0.196343	0.038898
0.144284	0.123922	0.175313	-0.10754	0.105946	0.304333	0.235802	0.085272	0.170874
-0.13655	0.178964	-0.14661	-0.511	-0.1081	0.259598	-0.11148	0.165049	-0.10979
-0.0272	0.046234	-0.32141	-0.31246	0.003532	0.208357	0.032277	0.207523	0.017905
0.083141	0.25346	0.527442	-0.42908	0.080884	0.349913	0.195382	0.283817	0.138133
0.073903	0.238539	0.676942	0.287076	-0.09077	0.43468	-0.10392	0.428779	-0.09734
0.192245	0.416633	0.664373	0.049785	0.37311	0.188221	0.38788	0.21168	0.380495
-0.11885	-0.26185	-0.3361	-0.43359	-0.04818	0.289086	-0.14632	0.255597	-0.09725
0.102891	-0.43071	-0.70234	-0.53617	-0.30008	0.203395	0.039993	0.507834	-0.13004
-0.03584	-0.2877	-0.67095	-0.43927	-0.1005	0.247135	-0.09999	0.343774	-0.10024
0.170116	-0.28826	0.159819	-0.39469	-0.28335	0.174985	0.129151	0.085613	-0.0771
0.159713	-0.33005	0.176118	-0.4179	0.007942	0.336645	0.226148	0.088952	0.117045
-0.1829	0.026793	-0.28365	-0.28551	-0.03432	0.342831	-0.05161	0.238928	-0.04296
0.077335	0.142212	-0.21706	0.461597	0.402542	0.205796	0.030439	0.199907	0.216491
-0.18341	0.000206	-0.01262	-0.3308	0.13806	0.308646	-0.11645	0.108378	0.010806
-0.34085	-0.13031	-0.43177	-0.68949	-0.14842	0.32851	-0.1982	0.202219	-0.17331
-0.01714	-0.28103	0.463703	0.160025	0.073334	0.230075	0.09844	0.2007	0.085887
0.096235	-0.38937	0.011823	-0.1377	-0.04375	0.280041	0.12229	0.100661	0.039269
0.007839	-0.24213	-0.24892	-0.05736	0.039098	0.178278	0.071107	0.178624	0.055103
-0.23465	0.073989	-0.71915	0.293825	-0.01113	0.266251	-0.18791	0.309539	-0.09952
0.004294	0.204767	0.072669	-0.11106	0.034052	0.381139	0.034974	0.135579	0.034513
-0.21726	-0.19821	-0.22269	-0.62579	-0.25771	0.185678	-0.16698	0.116762	-0.21234
-0.13033	-0.41985	0.090033	-0.5486	-0.26794	0.270202	-0.13943	0.180746	-0.20368
0.177415	0.120782	-0.21572	0.443551	0.213441	0.171428	-0.02461	0.186826	0.094417
-0.27122	-0.11346	-0.08936	-0.30844	0.050754	0.313679	-0.0776	0.189872	-0.01342
-0.06207	-0.05778	0.213119	0.493843	0.099736	0.308986	-0.03694	0.172716	0.031395
-0.37141	-0.48029	-0.53869	-0.43518	-0.28028	0.234393	-0.29323	0.274575	-0.28675
-0.02151	-0.46001	-0.4979	-0.46356	-0.31499	0.168002	-0.12503	0.258614	-0.22001
-0.18536	0.128338	-0.27749	-0.19341	-0.01663	0.172776	-0.11171	0.160545	-0.06417
0.038485	0.138468	0.356475	0.645012	0.159556	0.454994	0.041075	0.186764	0.100316
-0.08249	0.275293	-0.32163	0.26987	0.016051	0.257284	-0.05429	0.154416	-0.01912
0.069741	0.012679	NA	NA	-0.05148	0.246445	-0.12633	0.172427	-0.08891
0.108118	-0.01766	-0.37312	-0.31154	-0.14219	0.15852	-0.12611	0.223577	-0.13415
-0.05391	0.217047	-0.42358	-0.2351	0.113705	0.33046	-0.04221	0.234414	0.035745
-0.03738	0.169399	0.600601	0.131466	0.016471	0.359314	0.083839	0.329527	0.050155
-0.22033	0.110036	-0.29713	0.44453	0.14728	0.238097	-0.02148	0.344058	0.0629
-0.01848	-0.40595	0.475936	-0.48951	-0.16002	0.250311	0.029273	0.242744	-0.06537
0.019956	-0.31108	-0.51726	-0.58566	-0.30062	0.214015	-0.08961	0.321008	-0.19511
0.052285	-0.22962	0.066495	-0.21297	0.001279	0.188996	0.110777	0.15008	0.056028
-0.19677	0.158473	-0.34759	0.344288	0.014123	0.291039	-0.13404	0.16224	-0.05996
-0.02422	0.202404	0.409385	-0.33866	-0.0098	0.318232	-0.04235	0.300363	-0.02608
-0.13655	-0.06438	-0.61213	-0.5183	-0.10223	0.294495	-0.23989	0.339473	-0.17106
0.014794	-0.06973	-0.60983	-0.4546	0.110037	0.358934	-0.19384	0.264324	-0.0419
-0.40293	-0.0427	-0.69466	-0.41284	-0.16252	0.231276	-0.28481	0.252869	-0.22367
0.31832	-0.46115	-0.40182	-0.3876	-0.31935	0.121604	-0.05257	0.302917	-0.18596
0.318959	0.189907	-0.2544	0.427671	0.050301	0.221577	0.226478	0.253735	0.138389

-0.06651	-0.13199	0.062687	-0.51499	-0.05223	0.261572	-0.00247	0.177669	-0.02735
-0.02009	0.116367	-0.73744	0.058974	0.037141	0.301131	-0.21524	0.293253	-0.08905
-0.05175	-0.08532	0.138682	-0.39406	0.040082	0.277474	0.063935	0.225279	0.052009
0.085084	-0.07511	-0.6499	-0.16399	-0.1673	0.118985	-0.11639	0.274444	-0.14185
0.1419	0.149561	0.332413	0.300016	0.075188	0.1922	0.151069	0.156025	0.113128
0.452795	-0.34254	0.486579	-0.2069	-0.15191	0.194133	0.421355	0.07711	0.134721
-0.0523	0.246074	-0.22454	-0.15548	0.147166	0.29265	-0.08647	0.12719	0.030347
0.031929	-0.31417	0.452365	-0.34618	-0.15243	0.298173	0.138339	0.229746	-0.00705
-0.15639	-0.35004	NA	NA	-0.13101	0.22636	-0.13111	0.067042	-0.13106
0.224131	-0.38583	0.064206	-0.24119	-0.16224	0.176289	0.294698	0.141464	0.066228
-0.07676	-0.04297	-0.71752	-0.19187	0.052487	0.206471	-0.18774	0.316819	-0.06763
-0.22195	-0.00143	0.047765	-0.37832	-0.24971	0.192184	-0.2049	0.193739	-0.22731
0.021169	-0.32366	-0.3161	-0.55107	-0.24186	0.335462	-0.22744	0.191005	-0.23465
-0.24173	0.266702	-0.47907	0.162437	0.143521	0.109193	-0.0107	0.386598	0.066408
0.033706	-0.03574	0.417149	-0.34903	-0.07829	0.317856	0.059021	0.221964	-0.00963
-0.09313	0.140984	-0.27843	-0.04261	0.048618	0.287673	-0.11624	0.295797	-0.03381
-0.11661	-0.45624	-0.12398	-0.5686	-0.32936	0.156063	-0.1438	0.160176	-0.23658
-0.07274	0.344845	0.36851	0.265049	0.094942	0.217558	-0.05465	0.220534	0.020145
0.352967	0.308982	0.407177	0.203532	0.075518	0.262181	0.133494	0.294782	0.104506
0.345947	0.109282	0.701591	0.227002	0.074374	0.381429	0.422719	0.156292	0.248547
0.087023	0.152623	-0.13131	-0.28056	-0.03933	0.214368	-0.14656	0.216869	-0.09295
0.049254	0.206752	0.286996	0.290167	0.218841	0.144181	0.072258	0.179301	0.145549
0.081798	0.115575	-0.32259	-0.07726	-0.03669	0.082107	0.095678	0.249053	0.029495
-0.01606	0.10938	0.190264	0.665738	0.242772	0.338725	-0.0008	0.13759	0.120986
-0.28105	0.10532	-0.36307	0.454417	0.02346	0.300266	-0.22446	0.218033	-0.1005
-5.3E-05	0.178619	-0.1923	0.170724	0.257725	0.140949	-0.00246	0.165403	0.127632
-0.21622	0.32234	-0.43431	0.341885	0.122112	0.224829	-0.14153	0.265817	-0.00971
0.008125	-0.18407	-0.00011	-0.32897	0.05283	0.355657	0.047451	0.170305	0.05014
0.070179	-0.40387	-0.15693	-0.3758	-0.37608	0.217281	-0.19539	0.24187	-0.28574
-0.1711	0.094945	-0.08512	0.123783	0.131137	0.040242	-0.11465	0.170185	0.008245
-0.24656	-0.06618	-0.69136	-0.09657	-0.03293	0.087488	-0.22979	0.295471	-0.13136
0.170777	0.218227	-0.04205	0.178816	0.18284	0.042761	0.187241	0.21257	0.185041
0.04535	0.179293	0.141036	-0.14088	-0.16404	0.267579	0.094804	0.140798	-0.03462
-0.05829	-0.17161	0.386892	-0.16773	-0.12914	0.083364	0.000624	0.289942	-0.06426
-0.33235	-0.1829	-0.17388	-0.30085	-0.04761	0.23085	-0.17553	0.131545	-0.11157
0.097595	0.030015	-0.18656	-0.11801	-0.09657	0.180321	0.025893	0.150405	-0.03534
0.142337	-0.19639	0.015464	-0.33909	-0.15793	0.157918	0.119636	0.120114	-0.01915
0.060348	-0.00711	0.640644	0.119089	-0.02227	0.141107	0.095663	0.28788	0.036697
-0.24512	0.084076	-0.01604	-0.0872	-0.06469	0.154635	-0.08622	0.128241	-0.07546
0.035619	-0.51738	0.260367	-0.34799	-0.22492	0.246343	0.027216	0.284928	-0.09885
0.051247	-0.04492	NA	NA	-0.057	0.147048	0.079184	0.119224	0.011094
0.007391	0.042157	-0.15637	-0.20874	0.067401	0.246555	-0.14342	0.322431	-0.03801
0.235455	0.193495	-0.09153	0.185026	0.109032	0.222734	0.205346	0.256526	0.157189
-0.03484	0.567658	0.286453	0.938188	0.584873	0.394937	-0.0208	0.289622	0.282037
0.040517	0.173546	0.480791	0.226224	0.066693	0.247285	0.191626	0.241109	0.129159
-0.26787	0.262671	-0.20742	0.408605	0.093918	0.298537	-0.17002	0.23737	-0.03805
-0.07574	0.194092	0.18746	0.184142	0.09057	0.120487	0.029039	0.284846	0.059804
-0.02329	0.033826	NA	NA	0.172584	0.251547	-0.08504	0.102706	0.04377
-0.14432	-0.14555	0.234824	0.176514	-0.03983	0.228977	0.053808	0.250302	0.006991
-0.47717	0.170137	-0.29841	0.288935	0.296476	0.156043	-0.2049	0.215553	0.04579
-0.04202	0.299912	0.189906	0.509657	0.195029	0.211115	-0.13351	0.225173	0.030757
0.338075	0.545849	0.477694	0.62238	0.220299	0.331358	0.127685	0.244336	0.173992

0.276693	-0.24194	0.406372	-0.17371	-0.06576	0.167773	0.282028	0.145231	0.108132
0.23394	0.186213	0.663084	0.207769	0.044511	0.445999	0.300025	0.310794	0.172268
0.095975	-0.04789	0.500828	-0.33877	-0.19756	0.234801	0.054947	0.321623	-0.0713
0.363604	0.17137	0.721383	-0.00945	0.043511	0.267214	0.391366	0.258003	0.217439
0.048219	-0.37122	-0.12554	-0.44893	-0.2773	0.160303	-0.04774	0.16035	-0.16252
0.176015	-0.23868	-0.38982	-0.43841	-0.12271	0.335496	-0.15151	0.228301	-0.13711
-0.00217	0.131957	0.022434	-0.43504	-0.11641	0.376061	0.006833	0.335141	-0.05479
0.183481	-0.23596	0.066543	-0.30698	-0.02072	0.238569	0.180938	0.157675	0.080107
-0.08826	0.044918	-0.69003	-0.17027	-0.11799	0.335768	-0.17794	0.371392	-0.14796
0.014339	0.369815	0.401968	0.316708	0.2757	0.239052	0.00316	0.245629	0.13943
-0.41377	-0.07059	-0.7163	-0.25626	-0.22265	0.248563	-0.19772	0.379613	-0.21019
0.259496	0.268139	0.347254	0.416202	0.052094	0.338273	0.213029	0.206762	0.132561
0.256415	0.137197	-0.1406	-0.46528	-0.0124	0.241422	0.060154	0.21424	0.023875
0.259116	-0.17678	-0.06004	-0.20696	0.060556	0.357482	0.054209	0.279429	0.057382
-0.21506	0.316414	-0.16024	0.44801	0.26522	0.191494	-0.05845	0.303301	0.103384
-0.0772	0.142793	-0.49656	0.156598	0.190306	0.311222	-0.07883	0.258952	0.055738
0.05979	-0.07208	-0.51158	-0.07801	-0.10828	0.105429	-0.15172	0.225783	-0.13
0.072375	0.082955	NA	NA	0.075871	0.317237	0.125279	0.058568	0.100575
0.32999	0.474294	0.617223	0.501661	0.280452	0.227902	0.283711	0.222167	0.282082
-0.20818	0.152839	-0.34858	-0.05111	0.012743	0.236687	-0.09961	0.293389	-0.04344
0.215121	0.378376	0.195145	0.505022	0.198486	0.24859	-0.02396	0.335508	0.087265
-0.06467	0.159448	-0.36266	0.115539	0.124291	0.146954	-0.09195	0.273615	0.016168
0.103928	0.207105	-0.01428	0.199153	-0.02887	0.205562	-0.00025	0.128672	-0.01456
-0.23498	0.203599	-0.59355	0.465851	-0.04341	0.352687	-0.17634	0.261203	-0.10987
-0.02093	0.31563	0.040085	0.294753	0.162731	0.146366	-0.01653	0.074411	0.073098
0.04481	-0.07285	-0.27867	-0.08917	-0.09312	0.212454	-0.07508	0.190088	-0.0841
0.051601	0.119986	-0.20209	0.134939	0.221874	0.173951	-0.05052	0.145863	0.085679
0.032035	-0.41156	-0.27117	-0.42964	-0.12256	0.264069	-0.05351	0.234463	-0.08803
0.005378	-0.46471	-0.34366	-0.55206	-0.24369	0.244656	-0.08886	0.168635	-0.16628
0.206593	0.112385	-0.58554	-0.0781	-0.05517	0.164959	-0.07174	0.270185	-0.06345
0.226927	0.233578	-0.20644	0.424001	0.168751	0.277912	0.006379	0.178844	0.087565
-0.06082	0.032014	-0.4399	0.196165	-0.17191	0.251104	-0.20116	0.220508	-0.18653
0.10446	-0.17026	-0.70643	-0.40511	-0.15405	0.184555	-0.08148	0.365897	-0.11776
-0.02891	-0.01757	-0.27899	0.029311	-0.11634	0.247854	-0.1093	0.19504	-0.11282
0.099968	-0.02652	0.345894	0.441246	0.121276	0.263115	0.057816	0.171604	0.089546
0.07389	0.222593	-0.03104	0.041419	0.139329	0.317616	0.101975	0.149949	0.120652
-0.02413	-0.09215	0.041159	0.007594	0.037592	0.272399	-0.04011	0.132354	-0.00126
0.133246	0.114901	0.021939	-0.00127	0.160932	0.263334	0.123436	0.05602	0.142184
-0.36557	0.105096	-0.3201	-0.18872	-0.08875	0.131921	-0.23437	0.181643	-0.16156
-0.14858	-0.41006	-0.22208	-0.54523	-0.2093	0.250734	-0.21263	0.215483	-0.21097
-0.18516	-0.39187	-0.14009	-0.48825	-0.26007	0.202668	-0.24382	0.117053	-0.25194
-0.08916	0.046545	-0.56027	-0.21071	0.027105	0.184378	-0.11366	0.24519	-0.04328
-0.11435	-0.03822	NA	NA	-0.05652	0.328461	-0.00475	0.289182	-0.03064
0.492675	0.136138	0.388488	0.311129	0.03018	0.297833	0.360959	0.094818	0.195569
-0.20795	0.413787	-0.7392	0.263969	0.282353	0.341001	-0.29023	0.267625	-0.00394
0.307905	0.377168	0.819496	0.799465	0.394343	0.261142	0.38505	0.293617	0.389697
0.152451	-0.05411	0.209263	0.317524	-0.09579	0.319119	0.042178	0.206674	-0.02681
-0.27543	0.118439	-0.02682	0.311368	0.168709	0.15987	-0.18023	0.132043	-0.00576
0.133337	0.317777	0.117079	0.429647	0.168554	0.226487	0.239017	0.118972	0.203785
0.3701	-0.27891	0.529753	-0.11712	-0.16533	0.098859	0.230193	0.224978	0.032434
0.110588	0.258594	0.344557	0.190811	0.074681	0.222335	0.19621	0.157882	0.135446
0.099055	-0.18055	-0.54522	-0.39963	-0.10942	0.278186	-0.13426	0.275913	-0.12184

-0.08262	0.043237	-0.22372	-0.28239	-0.14189	0.170049	-0.0921	0.305284	-0.11699
0.13712	0.197598	-0.31696	-0.05848	0.022842	0.17999	-0.08287	0.170406	-0.03001
-0.02878	0.05099	0.251166	0.013267	-0.13916	0.263573	-0.00757	0.215873	-0.07336
-0.33407	-0.30923	0.252582	-0.37208	-0.11625	0.197485	-0.13004	0.215876	-0.12315
-0.14305	0.118502	-0.45514	-0.37181	-0.16241	0.300846	-0.03238	0.310149	-0.0974
0.118475	-0.03501	-0.23409	-0.01436	-0.02726	0.095814	-0.01336	0.211068	-0.02031
NA	NA	0.259797	-0.23149	-0.12968	0.378312	-0.18408	0.384769	-0.15688
NA	NA	0.259819	-0.31503	-0.04651	0.242496	0.042136	0.309764	-0.00219
0.072497	-0.0717	0.4523	0.144035	0.083012	0.169291	0.031856	0.296835	0.057434
0.045061	-0.22792	0.443699	-0.24625	-0.21622	0.134148	-0.04633	0.310429	-0.13128
0.229726	0.310008	0.554333	0.347525	0.112678	0.271949	0.283263	0.201592	0.19797
0.215454	0.193425	0.716626	0.097132	0.108579	0.267065	0.091937	0.36475	0.100258
0.330441	0.142414	0.738991	0.495695	0.222829	0.190625	0.391567	0.20732	0.307198
0.117167	0.269193	0.278464	-0.28361	0.068842	0.334162	-0.09097	0.31227	-0.01106
0.060258	-0.24383	-0.13799	-0.46864	-0.21051	0.211543	0.042156	0.225998	-0.08418
0.067171	-0.24057	-0.54452	-0.26047	-0.16094	0.123471	-0.09236	0.333806	-0.12665
0.151799	-0.00039	0.707533	0.477789	-0.0103	0.360167	0.31535	0.235808	0.152526
0.002315	-0.31502	-0.31578	-0.47037	-0.26054	0.21889	-0.11265	0.255607	-0.18659
0.12671	0.329729	0.287087	0.255436	0.245555	0.232991	0.304985	0.104358	0.27527
0.172169	0.227043	-0.51646	0.007432	0.073768	0.087353	-0.03014	0.462449	0.021816
-0.29203	-0.42027	-0.45146	-0.56562	-0.29869	0.233141	-0.30366	0.175187	-0.30117
-0.04207	0.190849	-0.36993	-0.30523	-0.1308	0.222743	-0.17173	0.154212	-0.15126
-0.09809	0.13787	0.246847	0.025945	0.004065	0.167021	-0.04213	0.219696	-0.01903
-0.16674	-0.41049	-0.27508	-0.43489	-0.23825	0.292872	-0.2198	0.219951	-0.22903
0.237574	-0.08735	0.371018	-0.15458	0.203252	0.275765	0.300791	0.17041	0.252021
-0.16927	-0.18555	-0.20252	-0.34861	-0.0998	0.300627	-0.01552	0.178553	-0.05766
-0.13288	0.205196	-0.62363	0.404566	0.099509	0.284665	-0.15459	0.252384	-0.02754
0.211776	0.007749	0.205021	-0.05128	0.055336	0.188792	0.178104	0.160626	0.11672
-0.00505	-0.14215	0.117619	-0.38892	0.009515	0.335885	0.133453	0.111286	0.071484
-0.07958	-0.19943	0.12837	-0.31821	-0.11295	0.262072	-0.00626	0.107756	-0.05961
-0.14215	-0.17215	0.07963	-0.35878	-0.09254	0.176743	-0.01982	0.173271	-0.05618
0.041069	-0.3421	0.314732	-0.44764	-0.35446	0.155583	-0.0754	0.306709	-0.21493
0.136067	0.094205	NA	NA	0.028798	0.199785	0.090791	0.139221	0.059794
0.042455	-0.16468	-0.42586	-0.39451	-0.06574	0.21591	-0.01687	0.238109	-0.04131
-0.2327	-0.42632	-0.45075	-0.39963	-0.26648	0.15234	-0.20846	0.284144	-0.23747
-0.06906	0.098109	-0.01105	0.061796	-0.00197	0.195891	-0.05131	0.102152	-0.02664
-0.43853	0.110939	NA	NA	-0.06396	0.167162	-0.25854	0.22309	-0.16125
-0.30068	0.351013	0.362192	-0.02631	0.169637	0.208563	-0.05596	0.27089	0.056839
-0.07055	-0.17798	0.358536	0.142465	-0.0228	0.204395	-0.01042	0.291613	-0.01661
-0.33477	-0.34188	-0.52549	-0.4616	-0.10981	0.320147	-0.18059	0.242777	-0.1452
-0.3121	-0.22231	-0.72666	-0.58433	-0.38901	0.165435	-0.32813	0.245484	-0.35857
-0.2458	0.053804	-0.22016	0.279696	-0.09992	0.318046	-0.19015	0.061575	-0.14504
0.099582	0.01819	0.703703	-0.19893	-0.04369	0.174968	0.273384	0.263388	0.114846
-0.01399	0.177155	-0.13634	0.291887	0.070708	0.267873	0.019754	0.1114	0.045231
0.305012	0.144268	-0.09745	0.201837	-0.0166	0.260757	0.144807	0.150075	0.064104
0.042015	0.123906	0.304709	0.328021	0.070616	0.303792	0.056057	0.314844	0.063336
-0.08708	0.030466	0.101247	0.018909	-0.05532	0.406085	-0.00732	0.186254	-0.03132
0.072957	0.401756	-0.06394	0.029631	0.182683	0.277647	0.022855	0.106491	0.102769
0.391099	0.176208	0.332985	0.462198	0.183654	0.380573	0.28579	0.100339	0.234722
-0.08511	-0.20285	-0.25141	-0.56909	-0.40096	0.194615	-0.14648	0.097226	-0.27372
0.263688	0.008631	0.346161	-0.16113	0.019775	0.344209	0.151104	0.250965	0.085439
0.141349	-0.22312	NA	NA	-0.04266	0.163722	0.107767	0.202015	0.032556

0.153541	-0.07725	0.647963	0.042476	0.085613	0.165775	0.261925	0.242484	0.173769
-0.14008	0.235982	-0.24103	-0.04474	0.068601	0.402321	-0.13392	0.18543	-0.03266
0.040623	-0.22924	0.21732	-0.37985	-0.26762	0.141614	-0.05955	0.201061	-0.16358
-0.00236	-0.20148	0.347142	-0.55744	-0.11631	0.263647	0.094332	0.156812	-0.01099
-0.11831	0.303559	-0.16021	0.095343	0.065696	0.183645	-0.02227	0.145151	0.021711
-0.03416	-0.06035	-0.41607	-0.21226	-0.03197	0.250706	-0.11621	0.290413	-0.07409
0.092117	0.139019	0.284098	0.073645	0.116801	0.1279	0.102295	0.166556	0.109548
0.082757	-0.25552	-0.25165	-0.17802	-0.33257	0.121135	-0.04715	0.177255	-0.18986
0.263664	0.155332	-0.31556	0.115258	0.093065	0.246561	0.071578	0.243643	0.082321
0.096077	0.22656	-0.1537	0.02521	0.105307	0.132743	-0.00207	0.125366	0.051616
0.03815	-0.37818	-0.08819	-0.37342	-0.2019	0.214688	0.073451	0.127392	-0.06422
0.165529	0.278541	0.389744	0.187168	0.247209	0.091377	0.185074	0.141436	0.216141
0.05914	0.270367	0.145094	0.108994	0.078567	0.176186	-0.00104	0.127903	0.038762
-0.14479	0.046076	-0.00322	0.080433	0.026576	0.070966	-0.03751	0.144758	-0.00547
-0.03348	-0.01912	NA	NA	-0.06219	0.041073	-0.04998	0.108307	-0.05609
0.113252	0.186581	-0.27395	0.171656	0.136923	0.073727	-0.04966	0.163388	0.043631
0.132313	0.091177	-0.13778	-0.01131	-0.0032	0.138134	0.147648	0.205905	0.072225
0.067276	0.034318	-0.30234	-0.23869	-0.08598	0.117739	-0.08325	0.158744	-0.08462
-0.03219	-0.45026	0.035801	-0.42998	-0.22872	0.257649	0.032703	0.045207	-0.09801
-0.08846	0.031567	0.217739	-0.23608	-0.09682	0.13741	-0.04163	0.200141	-0.06923
-0.10253	-0.01562	0.023304	-0.4759	-0.06974	0.289011	-0.00509	0.114222	-0.03741
-0.00146	0.184808	0.30861	-0.01879	-0.08127	0.242935	0.031522	0.16255	-0.02488
0.036785	0.102626	0.513381	0.186741	0.082689	0.084355	-0.00406	0.403819	0.039313
0.034847	-0.03797	-0.3623	-0.20027	-0.12898	0.148397	-0.15204	0.186589	-0.14051
-0.01673	0.183709	-0.15229	0.299721	-0.06124	0.358896	-0.01642	0.219669	-0.03883
-0.18487	0.044082	0.081855	0.017514	0.078588	0.091059	0.089677	0.214872	0.084132
-0.02631	0.133103	-0.56529	-0.18608	0.098829	0.39074	-0.03242	0.355603	0.033206
-0.04309	0.189973	0.276619	0.156071	0.19031	0.080726	0.038673	0.271119	0.114492
0.030929	-0.04047	0.555125	0.31818	0.126548	0.286761	0.01242	0.35107	0.069484
0.007554	0.258559	NA	NA	-0.12165	0.335144	-0.00372	0.277306	-0.06269
0.347407	0.205929	0.665781	0.189882	0.083843	0.296967	0.286535	0.356436	0.185189
-0.10902	-0.02844	-0.38516	-0.22306	-0.07402	0.16012	-0.06972	0.218621	-0.07187
0.106238	0.157162	-0.11246	-0.27561	-0.04933	0.289827	0.143913	0.194671	0.047294
-0.12707	-0.04429	-0.53557	-0.18415	-0.26028	0.165059	-0.17082	0.219886	-0.21555
0.122335	0.141677	0.203136	-0.42156	-0.09085	0.257684	0.109283	0.102058	0.009215
0.134769	0.020674	-0.08666	0.631685	0.027254	0.331895	0.004646	0.252253	0.01595
-0.22677	-0.06957	0.079417	-0.36103	0.079607	0.389743	-0.11745	0.127553	-0.01892
-0.05919	-0.21504	0.449728	-0.5484	-0.04695	0.329398	0.041457	0.210478	-0.00275
0.179671	0.077721	0.332596	0.091337	0.332193	0.309294	0.322536	0.144746	0.327365
0.117543	0.32925	0.539024	0.303936	0.065119	0.360246	0.198576	0.229545	0.131848
0.240503	0.052724	-0.22722	-0.47285	-0.05198	0.308604	-0.05853	0.198784	-0.05526
0.031025	0.059578	-0.15056	-0.4023	-0.01724	0.286723	0.029086	0.225943	0.005922
0.141629	-0.0091	NA	NA	-0.01062	0.182825	0.090973	0.17417	0.040176
0.060129	0.014516	-0.10324	-0.35243	0.005708	0.261858	0.127785	0.20724	0.066747
0.094948	0.032621	-0.05304	-0.448	-0.05621	0.274225	0.048736	0.068715	-0.00374
-0.0816	-0.03109	0.109713	-0.71381	-0.19429	0.265737	-0.01391	0.259524	-0.1041
-0.17385	0.097215	-0.47678	0.168569	0.098412	0.303477	-0.04638	0.400904	0.026015
-0.04494	0.022595	0.286503	-0.29259	-0.12618	0.25314	0.042197	0.246059	-0.04199
NA	NA	NA	NA	-0.00832	0.399112	0.091096	0.213597	0.041389
0.093737	-0.19079	NA	NA	-0.01615	0.226144	0.000586	0.164653	-0.00778
0.059869	-0.42531	NA	NA	-0.19115	0.142167	-0.03964	0.224028	-0.1154
-0.09084	0.228102	-0.31033	0.062588	0.078733	0.182621	-0.00311	0.228183	0.037814

-0.05866	-0.02769	-0.3488	-0.40902	0.008748	0.425721	-0.07265	0.248331	-0.03195
NA	NA	NA	NA	-0.02454	0.335828	-0.1103	0.065759	-0.06742
NA	NA	NA	NA	-0.23532	0.282624	-0.10992	0.135278	-0.17262
0.028565	-0.34658	-0.18369	-0.27769	-0.185	0.241304	-0.12834	0.099725	-0.15667
0.1966	0.133242	0.14975	-0.39883	-0.0634	0.332457	0.171383	0.114235	0.053991
-0.04638	0.105479	NA	NA	-0.19123	0.176375	-0.10284	0.174303	-0.14703
0.426883	-0.34609	0.230484	0.089991	-0.22124	0.162479	0.18471	0.288196	-0.01826
0.070365	0.162591	-0.33427	-0.56473	0.076247	0.423139	-0.10373	0.15887	-0.01374
-0.07562	-0.39134	NA	NA	-0.26771	0.259356	-0.17754	0.305897	-0.22263
0.094931	0.236209	0.158979	0.259925	-0.03297	0.320628	0.084416	0.137107	0.025724
0.147591	0.382529	-0.19818	-0.41693	0.044541	0.474758	0.195848	0.264207	0.120194
-0.00129	-0.02524	0.525627	0.307871	-0.01673	0.180272	0.052601	0.270312	0.017934
0.205196	-0.10738	0.480453	0.304532	0.014587	0.203393	0.188714	0.263435	0.101651
-0.07373	-0.19592	-0.25891	-0.36986	-0.23584	0.167386	0.062341	0.222734	-0.08675
-0.24118	-0.10948	-0.27199	-0.05549	-0.07933	0.228275	-0.15358	0.104403	-0.11645
0.078792	0.225757	-0.34979	0.293171	0.168294	0.145714	-0.06469	0.196429	0.051804
-0.19333	0.070591	-0.43836	0.263196	0.139154	0.21708	-0.16133	0.217697	-0.01109
0.081446	0.002641	NA	NA	-0.06757	0.326956	-0.05927	0.233048	-0.06342
0.132427	0.28722	0.448024	0.785332	0.354332	0.293369	0.288401	0.290293	0.321366
0.330748	0.250125	0.03698	0.490327	0.122436	0.317477	0.186247	0.160601	0.154341
0.002127	0.174796	-0.16911	0.274508	0.118899	0.139104	-0.09517	0.130255	0.011864
-0.17297	-0.16444	-0.24693	-0.27009	-0.16239	0.244483	-0.1562	0.148151	-0.1593
-0.00219	-0.11975	0.125633	-0.19642	0.006817	0.159484	-0.08918	0.138887	-0.04118
-0.28271	0.19659	-0.63419	0.314067	-0.09045	0.35056	-0.3655	0.149376	-0.22798
-0.02343	-0.34271	-0.43407	-0.31234	-0.16339	0.248276	-0.07482	0.304763	-0.1191
0.23345	-0.07861	-0.12721	-0.19805	-0.02457	0.389707	0.069922	0.235281	0.022676
-0.03374	0.18747	NA	NA	0.037014	0.391991	-0.02399	0.314405	0.006511
-0.07102	0.343253	-0.42296	0.054584	0.07202	0.239116	-0.02176	0.240661	0.025128
-0.00037	0.051014	NA	NA	-0.19571	0.225977	-0.12554	0.155845	-0.16062
-0.1074	0.174075	0.459815	-0.62419	-0.00946	0.347368	0.067861	0.242795	0.029199
-0.08483	-0.19306	0.659828	-0.42965	-0.16249	0.215848	0.209766	0.365409	0.023639
-0.0146	0.055213	-0.15774	-0.24486	-0.06433	0.141786	-0.05777	0.144422	-0.06105
0.110411	0.021178	-0.27845	0.219569	0.115182	0.296666	-0.01548	0.170079	0.049854
-0.16104	-0.01454	NA	NA	0.015759	0.303585	0.165752	0.266754	0.090756
-0.10565	0.197833	0.236283	-0.45737	0.04403	0.256956	-0.04168	0.186185	0.001173
0.18607	0.255657	0.458141	0.546188	0.354117	0.198211	0.107232	0.22227	0.230674
-0.01738	0.032316	-0.16868	0.050263	0.149478	0.125148	0.02332	0.170576	0.086399
-0.16578	-0.15059	-0.02524	-0.36996	-0.13023	0.241257	-0.02651	0.143333	-0.07837
-0.17506	-0.26004	-0.44434	-0.47634	-0.15421	0.267151	-0.12935	0.268337	-0.14178
-0.08904	-0.16183	-0.17639	-0.43696	-0.17307	0.194086	-0.05503	0.16243	-0.11405
-0.13094	-0.31559	-0.33905	-0.30653	-0.24556	0.169365	-0.17785	0.104276	-0.2117
0.003792	0.244039	0.090217	-0.2969	0.036005	0.212985	-0.07747	0.179567	-0.02073
0.066311	-0.00994	-0.01209	-0.18302	-0.02119	0.188192	-0.04021	0.08467	-0.0307
-0.1581	-0.18906	-0.4043	-0.47316	0.006607	0.326192	-0.05097	0.273973	-0.02218
0.125526	-0.01183	0.605571	-0.20445	0.108109	0.302088	0.267055	0.24087	0.187582
0.238251	0.181533	0.651735	-0.0506	0.234002	0.281773	0.256317	0.256815	0.24516
-0.13336	-0.25723	0.312887	-0.40459	-0.24359	0.099987	0.008393	0.264321	-0.1176
-0.33733	-0.27628	-0.29701	-0.30539	0.030066	0.370994	-0.21203	0.176322	-0.09098
-0.07347	0.502893	0.385033	0.59225	0.523039	0.047577	0.111358	0.201823	0.317198
-0.30892	0.389272	-0.27391	0.532927	0.411181	0.10949	-0.16482	0.160019	0.123181
-0.05806	0.008103	-0.43234	-0.57218	-0.3239	0.255803	-0.27311	0.159014	-0.2985
0.194013	0.204831	-0.30189	0.055856	0.237041	0.164992	0.156485	0.315619	0.196763

0.441994	-0.09503	0.009643	0.000527	-0.10309	0.246119	0.223894	0.196766	0.060404
-0.03401	-0.31431	0.063415	-0.2846	-0.20429	0.173799	-0.0695	0.144216	-0.1369
-0.15846	-0.47229	-0.40876	-0.4867	-0.32197	0.146504	-0.20691	0.287507	-0.26444
0.366805	-0.25404	0.276884	-0.21972	-0.02029	0.204687	0.278172	0.095044	0.128941
0.13983	0.083418	-0.15352	0.074556	-0.00339	0.09917	0.102786	0.176921	0.049699
0.041589	0.290726	0.297301	0.483835	0.297909	0.292975	0.182062	0.153428	0.239985
0.037353	0.042564	-0.40827	0.14514	-0.08338	0.200583	-0.03791	0.215156	-0.06065
-0.17048	0.352168	0.073481	0.610895	0.285288	0.226821	0.071573	0.252055	0.178431
-0.00443	0.170566	0.531407	0.093991	0.001972	0.235713	0.033656	0.355115	0.017814
0.065814	0.368396	0.200865	0.408954	0.144914	0.243432	0.108676	0.088022	0.126795
-0.10149	0.032	-0.17787	-0.47476	-0.11553	0.248486	-0.12085	0.209345	-0.11819
-0.24999	-0.26736	-0.02647	-0.41017	-0.08215	0.251252	-0.12072	0.087572	-0.10143
-0.01295	0.282503	-0.38492	-0.42156	0.124315	0.288264	-0.01903	0.258973	0.052645
0.202868	-0.04888	0.324965	-0.22103	0.070558	0.344518	0.260851	0.211584	0.165705
-0.04727	0.232037	0.18631	-0.17042	-0.03356	0.156644	-0.04105	0.129747	-0.03731
-0.14314	-0.20347	-0.69014	-0.44537	-0.13817	0.202745	-0.17247	0.278133	-0.15532
-0.28996	0.018746	-0.81452	0.052772	-0.06014	0.128652	-0.3165	0.297722	-0.18832
0.129694	0.264947	-0.35374	-0.0699	-0.09106	0.314174	-0.08124	0.269363	-0.08615
0.240916	0.146023	-0.5367	-0.17952	0.018868	0.350486	-0.1352	0.41794	-0.05816
0.103316	0.380368	0.419666	0.533754	0.369184	0.292951	0.121368	0.187911	0.245276
-0.20366	0.267906	-0.07241	0.150664	0.176544	0.208707	-0.15506	0.198092	0.010741
-0.1067	0.739402	-0.50239	0.834016	0.491697	0.374303	-0.21842	0.230584	0.136637
-0.15792	-0.20513	-0.306	-0.23893	-0.05195	0.146675	-0.2339	0.160061	-0.14293
0.124614	0.375564	0.452932	0.416816	0.162137	0.202265	0.155116	0.240233	0.158626
-0.11149	0.426598	-0.34504	0.470795	0.41291	0.206811	-0.06014	0.194398	0.176383
-0.00319	0.642255	0.174786	0.593158	0.392949	0.236999	-0.0166	0.243583	0.188177
-0.09757	0.412093	-0.47674	0.846625	0.312788	0.329616	-0.23558	0.241622	0.038603
0.29292	0.038098	0.675311	0.229296	0.112428	0.211698	0.198886	0.322016	0.155657
0.015712	0.050525	0.253658	-0.13853	0.050753	0.245577	0.197528	0.172851	0.124141
-0.17114	0.13254	0.21831	-0.15655	-0.04356	0.259401	-0.07264	0.186324	-0.0581
0.10576	0.009761	NA	NA	0.103489	0.298831	-0.15354	0.22747	-0.02503
-0.29491	0.2256	-0.46636	0.608615	0.03849	0.383538	-0.21562	0.153973	-0.08856
-0.17702	0.1113	0.187118	0.405308	0.169279	0.156424	-0.07308	0.210352	0.048098
NA	NA	0.122961	0.120577	0.170496	0.157276	0.009441	0.199647	0.089968
NA	NA	0.178298	0.400661	0.189935	0.182268	0.000985	0.25691	0.09546
-0.35642	0.388384	-0.55129	0.511154	0.211612	0.233335	-0.30761	0.367807	-0.048
-0.16258	0.091859	-0.10346	-0.00446	-0.01103	0.354509	-0.17811	0.127233	-0.09457
-0.2784	0.075851	-0.51396	0.090795	-0.01216	0.169174	-0.29049	0.160948	-0.15132
0.264012	0.328369	0.409444	0.311848	0.086728	0.247186	0.059392	0.401834	0.07306
-0.02711	-0.15599	-0.07207	-0.4653	-0.07683	0.287903	0.03473	0.272087	-0.02105
-0.00767	0.359567	0.503195	0.262665	0.24187	0.148406	0.073623	0.277337	0.157747
0.064072	0.306829	0.605073	0.420689	0.152174	0.268586	0.054613	0.288115	0.103394
0.035511	0.112822	0.437764	0.100751	-0.00755	0.200762	0.057957	0.210438	0.025203
0.083985	0.380171	0.482716	0.340107	0.133084	0.193286	0.063591	0.343102	0.098337
-0.09151	-0.01935	-0.48193	-0.16363	0.01404	0.278045	-0.02714	0.281755	-0.00655
0.023738	-0.06973	0.235562	-0.00072	-0.20013	0.202759	0.019881	0.271554	-0.09013
0.202964	-0.12851	0.665994	-0.6068	-0.07071	0.30038	0.295274	0.263151	0.112279
0.049813	-0.1921	0.04347	-0.52867	-0.05586	0.365409	0.105864	0.249735	0.025003
0.181353	0.483737	0.272901	0.391438	0.205806	0.336721	0.114273	0.208031	0.160039
-0.10084	0.145781	-0.77313	0.1956	-0.01219	0.277794	-0.17444	0.304686	-0.09331
0.264322	0.460374	0.630941	0.448594	0.256894	0.186803	0.259367	0.215012	0.258131
0.070303	0.238252	0.537488	0.443581	0.123378	0.267262	0.071614	0.337648	0.097496

0.255199	0.374319	0.666444	0.484833	0.262719	0.179076	0.265857	0.280446	0.264288
-0.03389	-0.16573	-0.35624	-0.33481	-0.29928	0.171288	-0.14416	0.220239	-0.22172
-0.2527	0.100312	-0.69849	0.125971	-0.10363	0.186219	-0.35631	0.173539	-0.22997
0.206224	0.022904	0.455616	0.229279	-0.18782	0.266455	0.154347	0.337667	-0.01674
-0.06718	-0.19813	0.135393	-0.51888	-0.04649	0.331151	0.103542	0.218931	0.028528
0.139572	0.040666	0.305259	-0.23338	-0.17427	0.106063	0.038029	0.215461	-0.06812
-0.16433	0.082191	-0.54557	0.35056	-0.04138	0.283525	-0.258	0.180792	-0.14969
-0.34929	0.396701	0.083884	0.347943	0.139784	0.218878	-0.04604	0.201581	0.046872
-0.05118	-0.08744	0.498406	0.543774	0.090746	0.410771	0.003341	0.28272	0.047043
0.232383	0.078547	-0.02833	-0.46288	0.126052	0.386728	0.197803	0.157004	0.161928
-0.10265	0.140866	0.458154	0.593051	0.2392	0.276042	0.213205	0.23273	0.226203
-0.08507	0.118033	0.210102	-0.07354	-0.08515	0.306485	-0.03504	0.177557	-0.06009
-0.10997	-0.13308	-0.24136	-0.13779	-0.12362	0.144504	-0.15405	0.199071	-0.13883
0.236053	-0.34548	0.230664	0.137978	0.07759	0.224485	0.179101	0.144298	0.128345
-0.15452	0.022624	-0.6372	-0.29505	-0.12895	0.127891	-0.19915	0.230957	-0.16405
-0.18051	-0.28834	-0.81645	-0.18872	-0.18612	0.148916	-0.33362	0.25469	-0.25987
-0.26021	-0.08547	-0.00877	0.107397	0.16332	0.198933	-0.15615	0.238852	0.003584
0.007697	0.161347	-0.49679	-0.05861	0.052921	0.332513	-0.04911	0.317945	0.001906
-0.18616	0.25091	-0.56025	-0.00962	0.131853	0.150842	-0.20741	0.234544	-0.03778
0.059188	-0.00556	-0.28214	0.246087	0.149448	0.30407	-0.14854	0.132378	0.000456
-0.21445	0.34428	0.077852	0.680187	0.365411	0.26424	0.062025	0.230057	0.213718
-0.26065	0.492165	-0.36251	0.398353	0.259969	0.271214	-0.23391	0.240612	0.013032
0.00909	0.699899	0.334529	0.782147	0.504696	0.249483	0.079647	0.307683	0.292171
-0.09817	0.803107	-0.06119	0.837519	0.590567	0.392223	-0.13924	0.243148	0.225662
-0.25674	0.171741	0.335151	0.270306	0.055319	0.22379	-0.04903	0.21856	0.003142
-0.22025	0.092944	-0.31647	0.596523	0.415235	0.21574	0.011662	0.268247	0.213449
NA	NA	NA	NA	-0.04315	0.223842	-0.04134	0.201586	-0.04224
-0.27528	-0.00287	-0.46685	-0.10231	-0.08662	0.15049	-0.16294	0.212792	-0.12478
0.109097	0.325598	-0.17453	0.433811	0.203041	0.280528	0.126632	0.208077	0.164836
-0.01315	0.01648	0.09439	0.022208	0.032825	0.212701	-0.08993	0.12197	-0.02855
-0.03403	0.017854	-0.20211	0.082819	0.061458	0.066313	0.014648	0.202485	0.038053
0.091199	-0.0288	0.494685	-0.31025	-0.03454	0.198945	0.219032	0.321555	0.092244
0.18662	0.13052	0.646772	0.064419	0.039357	0.222115	0.232263	0.227623	0.13581
0.269788	-0.01061	0.749481	-0.08717	0.121255	0.197927	0.341347	0.3071	0.231301
0.305222	0.179251	0.676314	0.106241	-0.05055	0.290543	0.262659	0.297874	0.106055
0.36309	0.013417	0.652744	-0.03821	-0.10834	0.261874	0.21728	0.271408	0.054471
0.124789	-0.01843	0.239495	0.282376	-0.14606	0.239683	-0.00554	0.200473	-0.0758
0.310166	0.092711	0.692039	0.063286	-0.00393	0.361706	0.302547	0.274044	0.149307
0.110434	0.230309	0.564801	0.302443	0.032751	0.387178	0.130324	0.279783	0.081538
-0.25123	-0.43599	-0.07206	-0.59201	-0.28136	0.219727	-0.15943	0.09317	-0.2204
-0.24464	-0.13695	-0.37294	-0.1423	-0.14911	0.153202	-0.21181	0.304835	-0.18046
0.199745	-0.19556	-0.03765	-0.204	-0.12502	0.127127	0.067207	0.101553	-0.0289
-0.12963	0.211703	-0.78794	0.373587	0.081235	0.244449	-0.2501	0.345019	-0.08443
-0.26016	-0.12761	-0.24556	0.045922	-0.0201	0.232731	-0.25997	0.262377	-0.14004
-0.03461	0.122124	-0.62309	0.403161	0.078493	0.292039	-0.10231	0.300491	-0.01191
-0.2518	0.093614	-0.49008	0.083934	-0.155	0.193679	-0.22508	0.191633	-0.19004
-0.05943	0.245236	-0.20524	-0.40721	-0.06127	0.268269	-0.21094	0.115977	-0.13611
-0.18604	0.062751	-0.675	0.103572	-0.07902	0.204109	-0.172	0.368696	-0.12551
-0.2651	0.663897	-0.23982	0.546296	0.34871	0.30145	-0.16406	0.146796	0.092326
-0.19051	0.76246	-0.34329	0.879164	0.611346	0.244273	-0.16525	0.102386	0.223048
0.072061	0.357336	0.180315	0.44099	0.330109	0.256554	-0.00962	0.161457	0.160247
-0.19304	0.187918	0.222625	0.218719	0.181511	0.163551	-0.06664	0.172697	0.057437

-0.19499	0.594177	-0.35944	0.59822	0.3787	0.321291	-0.21235	0.231459	0.083177
-0.23182	0.473372	-0.53336	0.009884	0.14233	0.268596	-0.21925	0.208074	-0.03846
-0.09101	0.397654	-0.02842	0.222802	0.174369	0.263626	-0.11493	0.128318	0.029717
-0.26792	0.648977	-0.30361	0.578361	0.491337	0.232475	-0.15366	0.205747	0.168839
-0.3801	0.00733	-0.2156	-0.47344	0.118362	0.323185	-0.21325	0.208165	-0.04745
[truncated: 583,427 more chars]
